# Exploiting quantum chaos diagnostics in QAOA for enhanced hybrid quantum classical deep learning classification

**DOI:** 10.1038/s41598-026-51870-8

**Published:** 2026-05-19

**Authors:** Javier Villalba-Díez, Juan Carlos Losada-González

**Affiliations:** 1 Fakultät Wirtschaft, Hochschule Heilbronn, Max-Planck-Str.39, Heilbronn, 74081 Baden-Württemberg Germany; 2 Department of Mechanical Engineering, Universidad de La Rioja, Edificio Departamental, c/ San José de Calasanz, 31, Logroño, 26004 La Rioja Spain; 3 Escuela Técnica Superior de Ingeniería Agronómica, Alimentaria y de Biosistemas, Universidad Politécnica de Madrid, Av. Puerta de Hierro, 2, Moncloa - Aravaca, 28040 Madrid, Madrid, 28040 Madrid Spain

**Keywords:** Quantum chaos, QAOA, Hybrid quantum-classical, OTOC, Quantum machine learning, Chaos diagnostics, Image classification, Quantum physics, Statistical physics, thermodynamics and nonlinear dynamics

## Abstract

The Quantum Approximate Optimization Algorithm (QAOA) is repurposed here as a feature map within a hybrid quantum–classical classifier, augmented by a chaos-informed diagnostic. We extract a scalar chaos feature by evaluating an Out-Of-Time-Ordered correlators (OTOC) along parameter-scaling rays through the trained circuit, computing spacings between local minima, and standardizing them via a pre-fitted lognormal model. To probe finite-size effects, we sweep the number of qubits $n\in \{4,6,8,10\}$ at fixed depth $p=2$ and train two models on a balanced 1,000-sample MNIST subset: a *StandardHybrid* using the $n$ local Pauli-$Z$ expectations, and a *ChaosAwareHybrid* which appends the OTOC-derived scalar. We perform multi-run, 5-fold cross-validation with a paired design (identical seeds/folds across models) and report mean±SD, paired mean differences $\Delta$, 95% t- and bootstrap CIs, exact permutation/sign tests, win-rates (Wilson 95% CI), and paired effect sizes. Across $N_\text {pairs}=\{50,50,67,50\}$ for $n=\{4,6,8,10\}$, the chaos-aware variant significantly improves test accuracy at $n\in \{4,6,8\}$ with $\Delta \approx +0.016$–$+0.018$, all 95% CIs excluding zero, permutation $p\approx 0$, high win-rates (86–100%), and large paired effects ($d_z\approx 1.0$–2.3). At $n=10$ the effect reverses ($\Delta =-0.022$, 2% win-rate, $d_z=-2.20$), indicating over-sensitivity. The best average accuracy occurs at $n=8$ ($0.9006\pm 0.0069$; $\Delta =+0.0180$; 100% wins). Per-epoch panels (train/val/test; mean±1 SD) reveal a “Goldilocks” width at which expressivity and sensitivity are balanced. These results show that a calibrated chaos diagnostic can enhance hybrid quantum–classical classifiers in resource-limited regimes and provide a principled knob to match circuit expressivity to many-body sensitivity.

## Introduction

Quantum computing has emerged as a transformative paradigm^1^ with the potential to revolutionize problem solving in areas ranging from cryptography^2^ and materials science^3^ to optimization^4^ and machine learning^5^. Despite remarkable progress in both theoretical foundations and experimental realizations during the past two decades, significant challenges remain to harness the full potential of quantum devices, particularly those operating in the Noisy Intermediate-Scale Quantum (NISQ) regime^6,7^. Within this evolving landscape, the Quantum Approximate Optimization Algorithm (QAOA) has garnered considerable attention as a promising candidate for addressing combinatorial optimization problems using near-term quantum hardware^8^. However, as quantum devices scale up and quantum algorithms approach practical applications, the need to deeply understand and characterize the underlying quantum dynamics becomes increasingly critical^9^.

Recent research has begun to uncover intriguing links between quantum chaos and the performance of variational quantum algorithms such as QAOA^10^. In classical chaotic systems, sensitivity to initial conditions and exponential divergence of trajectories are hallmarks that have long been exploited to understand complex dynamical behavior^11^. In the quantum realm, similar chaotic characteristics manifest in the evolution of quantum states, albeit in subtler ways. In particular, tools such as Out-Of- Time-Ordered correlators (OTOCs) have emerged as powerful diagnostics for probing quantum chaos, offering insights into the scrambling of quantum information and the sensitivity of quantum evolutions to perturbations^12^. These diagnostics not only provide a window into the rich interplay between quantum chaos and algorithmic performance but also hold promise for enhancing the design and training of quantum circuits.

In this work, we explore a novel integration of quantum chaos diagnostics within the QAOA framework to enhance the performance of hybrid quantum–classical deep learning classifiers. Our research is motivated by the observation that the chaotic dynamics inherent in variational circuits can significantly influence the learning and optimization processes. By incorporating chaos-aware metrics into the quantum feature maps used for data encoding, our aim is to provide the classical optimizer with additional nuanced feedback that reflects the circuit’s sensitivity to parameter fluctuations. The central hypothesis of our research is that such chaos-informed features can improve the robustness and accuracy of quantum machine learning models, thereby narrowing the performance gap between classical and quantum-assisted computational approaches.

The real-world relevance of our study extends beyond mere performance metrics. As quantum devices transition from laboratory prototypes to practical computational tools, understanding and controlling the complex dynamical behavior of quantum circuits becomes paramount. In particular, hybrid quantum–classical models—where classical processors collaborate with quantum coprocessors—are likely to be the workhorses of early quantum applications. However, the performance of these hybrid models is intricately tied to the quality of the quantum feature maps used to encode the data. By infusing these feature maps with chaos-informed diagnostics, our approach not only improves classification accuracy, but also provides a principled method for characterizing and mitigating potential sources of instability and error in quantum circuits.

The motivation for this research is further underscored by the current technological landscape, where quantum devices are inherently noisy and subject to various forms of decoherence and parameter drift. In such environments, a thorough understanding of the circuit dynamics—especially those that lead to chaotic behavior—can inform the design of more robust algorithms that are resilient to these imperfections. Moreover, as quantum algorithms are scaled to tackle increasingly complex problems, the ability to diagnose and control chaotic dynamics will be critical to ensuring that the computational advantage of quantum devices is fully realized.

Despite promising prospects, several research gaps remain unaddressed in the existing literature. Traditional approaches to QAOA optimization have largely focused on improving convergence rates and mitigating noise without explicitly considering the role of chaos in circuit dynamics. Similarly, while OTOCs and other chaos diagnostics have been extensively studied in the context of many-body physics and quantum information scrambling, their direct application as features for enhancing quantum machine learning models is relatively unexplored. This work seeks to fill this gap by demonstrating that chaos diagnostics can be systematically integrated into the QAOA framework, leading to tangible improvements in performance in standard benchmark tasks. In doing so, we provide a new perspective on the role of quantum chaos in variational algorithms and establish a foundation for future investigations into chaos-aware quantum computing.

To achieve these objectives, our article is structured as follows. In Section 2, we provide an in-depth review of the theoretical foundations underlying QAOA and quantum chaos diagnostics. This section discusses the principles of variational quantum algorithms, the mathematical formalism of OTOCs. Section 3 details our proposed methodology, including the construction of the QAOA circuit for the MaxCut problem, the integration of chaos diagnostics into the quantum feature map and the statistical modeling of the chaos feature using a lognormal distribution. In addition, the experimental setup, including data preparation, model architecture, and training procedures for hybrid standard and chaos-aware classifiers, is outlined. We present and compare our results in Section 4, highlighting the improvements in classification accuracy and discussing the implications of our findings for the broader field of quantum machine learning, and we discuss the limitations of our current approach and suggest potential avenues for future research. Finally, Section 5 concludes the paper with a summary of our contributions and a reflection on the broader impact of integrating chaos diagnostics into variational quantum algorithms.

## Theoretical foundations underlying QAOA and quantum chaos diagnostics

Understanding the dynamics of variational quantum circuits has become a central theme in quantum computing. Here we connect classical chaos to the behavior of quantum circuits realized via QAOA. We briefly recall the logistic map as a paradigmatic chaotic system, then introduce modern diagnostics of quantum chaos—most notably the OTOC. We build a correspondence between the iterative dynamics of the logistic map and the layerwise evolution in QAOA circuits, highlighting how classical measures such as Lyapunov exponents and Feigenbaum-type scaling have analogues in quantum settings.

### QAOA as a quantum dynamical system

The QAOA ^13^ was originally introduced as a gate-based variational analogue of adiabatic quantum computation. At its heart, QAOA alternates between two non-commuting unitaries generated by a problem (cost) Hamiltonian $H_C$ and a mixing Hamiltonian $H_M$. Concretely, given an initial reference state,

1 $$\begin{aligned} |\psi _0\rangle \,=\,\frac{1}{\sqrt{2^n}}\sum _{z\in \{0,1\}^n}|z\rangle , \end{aligned}$$

the depth–$p$ QAOA ansatz is

2 $$\begin{aligned} |\psi (\boldsymbol{\gamma },\boldsymbol{\beta })\rangle \,=\,\Bigl [\prod _{k=1}^p e^{-i\beta _k H_M}\,e^{-i\gamma _k H_C}\Bigr ]\,|\psi _0\rangle , \end{aligned}$$

with $\boldsymbol{\gamma }=(\gamma _1,\dots ,\gamma _p)$ and $\boldsymbol{\beta }=(\beta _1,\dots ,\beta _p)$ as tunable angles.

The cost Hamiltonian $H_C$ encodes the classical combinatorial objective (e.g. MaxCut):

3 $$\begin{aligned} H_C = \sum _{\langle i,j\rangle } w_{ij}\,\frac{1 - Z_i Z_j}{2}, \quad C(\boldsymbol{z}) = \langle \boldsymbol{z}|H_C|\boldsymbol{z}\rangle . \end{aligned}$$

Minimizing $H_C$ corresponds to optimizing the classical cost. Hamiltonian mixing $H_M$ promotes exploration of the Hilbert space, typically chosen as $H_M = \sum _{i=1}^n X_i,$, which generates uniform single-qubit rotations and prevents premature localization on the computational basis.

Equation (2) defines a sequence of discrete maps

4 $$\begin{aligned} |\psi _{k}\rangle = e^{-i\beta _k H_M}\,e^{-i\gamma _k H_C}\,|\psi _{k-1}\rangle , \quad |\psi _0\rangle = \frac{1}{\sqrt{2^n}}\sum _z|z\rangle , \end{aligned}$$

which can be viewed as iterations of a nonlinear, parametric quantum map in a projective Hilbert space. This is directly analogous to classical discrete-time dynamical systems such as the logistic map $x_{k+1} = r\,x_k(1-x_k),$ where the “control parameter” $r$ induces bifurcations and chaos. In QAOA, the variational angles $\{\gamma _k,\beta _k\}$ play a similar role: Small changes can lead to large deformations of the state vector and sensitivity in expectation.

5 $$\begin{aligned} C(\boldsymbol{\gamma },\boldsymbol{\beta }) = \langle \psi (\boldsymbol{\gamma },\boldsymbol{\beta })|H_C|\psi (\boldsymbol{\gamma },\boldsymbol{\beta })\rangle . \end{aligned}$$

In the limit $p\rightarrow \infty$, with angles chosen as $\gamma _k,\beta _k\propto 1/p$, QAOA approaches the continuous adiabatic path.

6 $$\begin{aligned} H(t) = (1-s(t))\,H_M + s(t)\,H_C, \quad s(t)\in [0,1], \end{aligned}$$

via trotterization. At finite $p$, QAOA represents a *digitized shortcut to adiabaticity*, yet inherits a rugged energy landscape with many local minima.

Optimization of $\{\gamma _k,\beta _k\}$ is often performed with gradient-free methods due to noise and non–analyticities. The Cost Landscape

7 $$\begin{aligned} \mathcal {L}(\boldsymbol{\gamma },\boldsymbol{\beta }) = \langle \psi (\boldsymbol{\gamma },\boldsymbol{\beta })|H_C|\psi (\boldsymbol{\gamma },\boldsymbol{\beta })\rangle \end{aligned}$$

exhibits an exponential concentration of measure and barren plateaus for generic Hamiltonians ^14^. This ruggedness is intimately related to the chaotic mixing induced by non-commuting layers: as in classical chaos, where the logistic map’s Lyapunov exponent quantifies sensitivity, QAOA’s OTOC-based diagnostics measure the sensitivity of $\psi (\gamma ,\beta )$ to parameter perturbations.

Practical implementations initialize $\gamma _k,\beta _k$ near zero (e.g., uniform in $\pm 0.01$), so the early evolution is close to identity. During optimization, phenomena *bifurcation-like* can be observed at optimal angles as $p$ increases, mirroring period-doubling cascades on the logistic map.

In summary:

1. *Discrete-time quantum dynamics:* QAOA define a sequence of nonlinear maps in the Hilbert space, directly analogous to chaotic maps in classical systems.
2. *Chaotic control:* The variational angles play the role of control parameters; OTOC measures capture sensitivity similar to classical Lyapunov exponents.
3. *Optimization landscape:* Rugged cost function topology arises from repeated non-commuting unitaries, necessitating chaos-aware diagnostics.

By situating QAOA within the broader context of quantum dynamical systems and chaos theory, we provide readers with the necessary background to appreciate our chaos-aware hybrid classifier and its novel feature based on OTOC.

### Why QAOA as a feature map?

QAOA’s alternating, non-commuting generators $H_C$ and $H_M$ implement a controllable Lie-generated family $U(\boldsymbol{\gamma },\boldsymbol{\beta })$ whose expressivity scales with $p$ and $n$, while preserving a physical structure (problem-informed entanglers) that induces non-local correlations. The same non-commutation drives operator growth; thus OTOC-based diagnostics naturally probe parameter sensitivity along rays $s\mapsto U(s\boldsymbol{\gamma },s\boldsymbol{\beta })$. Our scalar chaos feature $\chi$ summarizes this sensitivity and complements local readouts. Empirically, we observe a width-induced transition: under-variation at small $n$, well-conditioned sensitivity at $n=8$, and over-sensitivity at $n=10$. This pairing of *structured expressivity* and *measurable sensitivity* makes QAOA a physically interpretable feature map with a tunable diagnostic control.

### Quantum chaos in QAOA circuits: concepts and diagnostics

Quantum chaos investigates the signatures of chaotic behavior in quantum systems, particularly those whose classical analogs exhibit chaos. Central to this study are diagnostics that reveal the extreme sensitivity of quantum evolution to perturbations and capture the statistical properties of energy spectra. A prominent approach in this context is the use of the OTOC.

For a normalized quantum state $|\psi \rangle$ and bounded operators $A$ and $B$ (typically chosen as Pauli matrices or their tensor products, with eigenvalues $\pm 1$), the OTOC is defined as

8 $$\begin{aligned} C(t) = \langle \psi | [A(t), B]^\dagger [A(t), B] | \psi \rangle , \end{aligned}$$

where the time-evolved operator is given by $A(t) = U^\dagger (t) A U(t),$ and $U(t)$ denotes the unitary time evolution operator. This correlator quantifies how rapidly quantum information is scrambled in the system; in regimes of chaos, $C(t)$ exhibits rapid growth, analogous to the exponential divergence of trajectories in classical chaotic systems. The effective rate of this growth is often associated with a quantum Lyapunov exponent, though its precise interpretation must account for the inherent linearity of quantum mechanics.

In our experiments, we choose the following.

$$\begin{aligned} A \,=\, Z_1 \qquad \text {and}\qquad B \,=\, X_1, \end{aligned}$$

where $Z_1$ and $X_1$ are the standard single-qubit Pauli matrices that act on the first wire,

$$\begin{aligned} Z_1 = \underbrace{Z \otimes I \otimes \cdots \otimes I}_{n\text { qubits}}, \quad X_1 = \underbrace{X \otimes I \otimes \cdots \otimes I}_{n\text { qubits}}, \end{aligned}$$

with

$$\begin{aligned} Z = \begin{pmatrix}1 & 0 \\ 0 & -1\end{pmatrix}, \quad X = \begin{pmatrix}0 & 1 \\ 1 & 0\end{pmatrix}. \end{aligned}$$

Thus, $A$ and $B$ are *local* Pauli operators and satisfy $\Vert A\Vert =\Vert B\Vert =1$ (spectral norm). Because unitary evolution preserves the operator norm, $\Vert A(t)\Vert =\Vert A\Vert =1$. The commutator then obeys $\Vert [A(t),B]\Vert \le 2\,\Vert A(t)\Vert \,\Vert B\Vert =2$. By submultiplicativity, $\Vert [A(t),B]^\dagger [A(t),B]\Vert \le \Vert [A(t),B]\Vert ^2\le 4$. Since expectations in normalized states are bounded by the operator norm, $C(t)=\langle \psi \mid [A(t),B]^\dagger [A(t),B] \mid \psi \rangle \le 4$.

This rigorous bound on OTOC is a direct consequence of the bounded nature of the Pauli operators and the normalization of the quantum state. It guarantees that, even in a QAOA circuit where chaotic dynamics might induce rapid growth in $C(t)$, the correlator remains finite and behaves well.

This behavior is analogous to classical systems such as the logistic map described where the parameter $r$ is confined to the interval $[0,4]$ and $x_n$ remains within $[0,1]$ regardless of the onset of chaos. Similarly, despite the possibility of exponential sensitivity in the variational parameters of a QAOA circuit, the OTOC remains bounded by a fixed value (4 in this case), ensuring that it is a stable and physically meaningful measure of quantum information scrambling.

In summary, OTOC serves as a powerful tool to quantify the extent of information scrambling and sensitivity in quantum systems. Its bounded nature, rooted in the intrinsic properties of quantum operators and the normalization of states, ensures that even in chaotic regimes the measure remains finite, thereby facilitating reliable diagnostics of quantum chaos in variational algorithms like QAOA.

#### QAOA dynamics and chaos metrics

To elucidate the connection between classical chaos and QAOA, consider the following analogy. In the logistic map, the dynamics is driven by a single scalar parameter $r$ that controls the degree of nonlinearity. As $r$ increases, the system undergoes a cascade of period-doubling bifurcations, eventually leading to chaos. The QAOA circuit, in contrast, operates in a multidimensional parameter space $(\boldsymbol{\gamma }, \boldsymbol{\beta })$. Despite this complexity, one may isolate effective one-dimensional dynamics by projecting the evolution onto a relevant subspace. For example, one may analyze iterative updates of the variational parameters during the optimization process. Let $\boldsymbol{\theta }^{(n)} = (\gamma _1^{(n)}, \ldots , \gamma _p^{(n)}, \beta _1^{(n)}, \ldots , \beta _p^{(n)})$ denote the parameter vector in the $n$ -th iteration. Then, the optimization procedure yields a trajectory in parameter space:

9 $$\begin{aligned} \boldsymbol{\theta }^{(n+1)} = \boldsymbol{\theta }^{(n)} + \Delta \boldsymbol{\theta }^{(n)}. \end{aligned}$$

Then we may define a distance metric $d_n = \Vert \boldsymbol{\theta }^{(n+1)} - \boldsymbol{\theta }^{(n)}\Vert$. In analogy to the logistic map, the evolution of $d_n$ can be studied for signs of exponential divergence, which would indicate sensitivity to initial conditions. Such an analysis naturally leads to an estimation of a Lyapunov-like exponent for the QAOA optimization trajectory:

10 $$\begin{aligned} \lambda _{\text {{QAOA}}} = \lim _{n \rightarrow \infty } \frac{1}{n} \sum _{i=1}^{n} \ln \left( \frac{d_{i+1}}{d_i} \right) . \end{aligned}$$

A positive $\lambda _{\text {{QAOA}}}$ implies that small changes in the variational parameters can lead to exponentially large differences in the cost function landscape, reminiscent of chaotic behavior in classical systems. In other words, $\lambda _{\text {{QAOA}}}=0$ is the frontier where stability is lost.

The mathematical structure of the QAOA state [Equation (2)] is highly non-trivial due to the non-commutativity of $H_C$ and $H_M$. Let us denote the cost and mixing unitaries as:

11 $$\begin{aligned} U_C(\gamma )&= \exp (-i\gamma H_C), \end{aligned}$$

12 $$\begin{aligned} U_M(\beta )&= \exp (-i\beta H_M). \end{aligned}$$

Then, the QAOA evolution operator for a single layer is given by $U (\gamma , \beta ) = U_M(\beta ) U_C(\gamma ).$ For $p$ layers, the overall unitary is $U(\boldsymbol{\gamma }, \boldsymbol{\beta }) = \prod _{k=1}^{p} U_M(\beta _k) U_C(\gamma _k).$ The sensitivity of the final state $|\psi (\boldsymbol{\gamma }, \boldsymbol{\beta })\rangle$ to small perturbations in the parameters is central to our analysis. Consider a set of perturbed parameters $\boldsymbol{\theta } + \delta \boldsymbol{\theta }$. The fidelity between the states generated by the two sets can be expanded as

13 $$\begin{aligned} F \,=\, \bigl | \langle \psi (\boldsymbol{\theta }) \mid \psi (\boldsymbol{\theta } + \delta \boldsymbol{\theta }) \rangle \bigr |^2 \,\approx \, 1 - \sum _{i,j} \chi _{ij}\, \delta \theta _i\, \delta \theta _j, \end{aligned}$$

where $\chi _{ij}$ is a susceptibility matrix that encodes the sensitivity of the state to parameter variations. In chaotic systems, such sensitivities are expected to grow exponentially with depth $p$ or the effective time of evolution, analogous to classical systems where the nearby trajectories diverge at an exponential rate^15^.

The OTOC provides a measure of this sensitivity. In the context of QAOA, the “time” variable can be mapped onto the scaling of the variational parameters, and one may study the OTOC as a function of a scaling factor $s$ applied to the optimal parameters. The local minima in the resulting OTOC curve then serve as markers of sensitive regions in parameter space. The distances between these minima, when statistically analyzed, yield a distribution that captures the chaotic properties of the quantum circuit.

Let $\{ s_i \}$ denote the sequence of scaling factors corresponding to the local minima of the OTOC curve, and define $\Delta s_i = s_{i+1} - s_i$. Our investigation has found that the empirical distribution $P(\Delta s)$ can be modeled by a lognormal distribution:

14 $$\begin{aligned} P(\Delta s) = \frac{A}{\Delta s \, \sigma \sqrt{2\pi }} \exp \left[ -\frac{(\ln \Delta s - \mu )^2}{2\sigma ^2} \right] , \end{aligned}$$

where $A$, $\mu$, and $\sigma$ are parameters determined via curve fitting to the aggregated data.

A deeper insight into the connection with classical chaos emerges when one considers the iterative nature of the parameter updates. In many optimization routines, the update rule for the variational parameters may be written as $\theta ^{(n+1)} = f\left( \theta ^{(n)}\right) ,$ where $f$ is a nonlinear function determined by the optimization algorithm and the gradient of the cost function^16^. Under certain conditions, $f$ can exhibit dynamics similar to those of the logistic map. In particular, if one isolates a scalar measure of the parameter change—such as the norm of the update—the evolution $d_{n+1} = g\left( d_n\right) ,$ may display a period-doubling route to chaos. Here, $g$ is an effective nonlinear map that encapsulates the influence of the quantum circuit’s sensitivity. Through a careful analysis of the iterative sequence $\{ d_n \}$, one can extract an effective Lyapunov exponent $\lambda _Q$ that is directly analogous to the classical Lyapunov exponent.

Furthermore, the universal scaling observed in the logistic map —embodied in the Feigenbaum constant— can, in principle, emerge in the statistical properties of the QAOA optimization landscape. For example, if one considers the ratios $\delta _i = \frac{\Delta s_{i}}{\Delta s_{i+1}},$ then, as the number of layers $p$ increases or as the system is tuned into a strongly chaotic regime, the distribution of $\{ \delta _i \}$ may converge to a universal value reminiscent of the Feigenbaum constant. Our numerical simulations indicate that such scaling behavior is indeed observable, providing further evidence of the deep connection between the chaotic dynamics of classical maps and the evolution of variational quantum circuits.

## Methodology

In this section, we detail the methodology underlying our investigation of quantum chaos diagnostics within the framework of the QAOA and its application to hybrid quantum–classical classification. Our exposition is both mathematically rigorous and comprehensive, covering the construction of the QAOA ansatz for the MaxCut problem, the derivation and analysis of chaos diagnostics based on OTOC and Lyapunov exponent estimation, and the subsequent incorporation of these diagnostics into a chaos-aware hybrid model for the classification of MNIST images.

Our approach is divided into two interrelated components. The first component concerns the characterization of chaotic dynamics in variational quantum circuits via QAOA. This involves constructing a QAOA circuit tailored to solve the MaxCut problem on a small graph and employing several chaos diagnostics—such as the OTOC and Lyapunov-like exponent estimation—to quantify the system’s sensitivity to parameter perturbations. Additionally, we perform a detailed analysis of the OTOC response under parameter scaling, from which universal features (including a Feigenbaum-like ratio) and the statistical distribution of distances between local minima are extracted.

The second component leverages the insights from the chaos diagnostics to enhance a hybrid quantum–classical classifier. In this model, the standard quantum feature map is augmented with an additional chaos metric, derived from the statistical analysis of the OTOC. This extra feature is standardized via a lognormal probability density function and concatenated with the original quantum feature vector before being passed to a classical neural network. We then compare the performance of this chaos-aware model with that of a standard hybrid model to demonstrate the practical benefits of incorporating chaos diagnostics.

### Chaotic quantum circuits

#### Quantum circuit design: QAOA ansatz for maxcut

The core of our methodology is the QAOA ansatz, which is designed to solve combinatorial optimization problems such as MaxCut. Consider a graph with $n$ nodes and an edge set $\mathcal {E}$. The cost Hamiltonian $H_C$ for the MaxCut problem is given by Equation (3). The eigenvalues of each term are $0$ and $1$, which reflect the cost associated with cutting an edge.

The initial state is given by Equation (1), which is prepared by applying a Hadamard gate $H$ to each qubit $H^{\otimes n} |0\rangle ^{\otimes n} = |+\rangle ^{\otimes n}.$

The QAOA ansatz is defined by a sequence of $p$ alternating layers of cost and mixing unitaries given by Equation (2).

As a result, the circuit construction proceeds as follows: first, Hadamard gates are applied to each qubit to create the uniform superposition state. Then, for each layer $k = 1, \dots , p$, the cost unitary is applied by performing an Ising-$ZZ$ interaction on every edge $(i,j) \in \mathcal {E}$ with rotation angle $-\gamma _k$, followed by the mixing unitary that applies an $R_X$ rotation with angle $2\beta _k$ on every qubit. Mathematically, the full unitary operation is given by:

15 $$\begin{aligned} U(\boldsymbol{\theta }) = \prod _{k=1}^{p} \left[ \left( \bigotimes _{j=1}^{n} R_X\left( 2\beta _k\right) \right) \prod _{(i,j) \in \mathcal {E}} \exp \left( -i \gamma _k Z_i Z_j\right) \right] . \end{aligned}$$

We now expand this expression in full matrix form.

For a fixed layer $k$, the cost unitary is given by the product over all edges $(i,j) \in \mathcal {E}$ of the two-qubit operations $\exp \bigl (-i \gamma _k\, Z_i Z_j\bigr ).$ Recall that the Pauli $Z$ operator is defined as $$Z = \begin{pmatrix} 1 & 0 \\ 0 & -1 \end{pmatrix}.$$ Thus, for the two-qubit operator $Z_i Z_j$ acting on qubits $i$ and $j$, its action in the computational basis $\{|00\rangle , |01\rangle , |10\rangle , |11\rangle \}$ yields eigenvalues:

- $+1$ when the qubits are aligned (i.e., in states $|00\rangle$ or $|11\rangle$),
- $-1$ when the qubits are anti-aligned (i.e., in states $|01\rangle$ or $|10\rangle$).

Consequently, the matrix exponential is a diagonal $4 \times 4$ matrix:

16 $$\begin{aligned} \exp \bigl (-i \gamma _k\, Z_i Z_j\bigr ) = \begin{pmatrix} e^{-i\gamma _k} & 0 & 0 & 0 \\ 0 & e^{i\gamma _k} & 0 & 0 \\ 0 & 0 & e^{i\gamma _k} & 0 \\ 0 & 0 & 0 & e^{-i\gamma _k} \end{pmatrix}. \end{aligned}$$

For the entire layer, if we denote the cost unitary by $U_{C}^{(k)} = \prod _{(i,j)\in \mathcal {E}} \exp \bigl (-i \gamma _k\, Z_i Z_j\bigr ),$ then $U_{C}^{(k)}$ is formed as the product (taken in any fixed order, since these diagonal matrices commute) of the two-qubit gates, each embedded appropriately in the full $2^n$-dimensional Hilbert space.

The mixing unitary involves a rotation about the $X$-axis applied to each qubit. For a single qubit, the rotation is given by $R_X\left( 2\beta _k\right) = \exp \Bigl (-i\beta _k\, X\Bigr ),$ where the Pauli $X$ operator is $$X = \begin{pmatrix} 0 & 1 \\ 1 & 0 \end{pmatrix}.$$ Using the standard identity for rotations about a Pauli axis, we obtain: $R_X\left( 2\beta _k\right) = \cos \beta _k\, I - i\sin \beta _k\, X,$ which, in matrix form, reads

17 $$\begin{aligned} R_X\left( 2\beta _k\right) = \begin{pmatrix} \cos \beta _k & -i\sin \beta _k \\ -i\sin \beta _k & \cos \beta _k \end{pmatrix}. \end{aligned}$$

The full mixing unitary acting on all $n$ qubits is then given by the tensor product: $U_{M}^{(k)} = \bigotimes _{j=1}^{n} R_X\left( 2\beta _k\right) ,$ resulting in a $2^n \times 2^n$ matrix.

Combining the cost and mixing unitaries for each layer, the complete circuit unitary is constructed by applying, for each $k$ from 1 to $p$, first the cost unitary and then the mixing unitary. Hence, we obtain an analog version of Equation (15):

18 $$\begin{aligned} U(\boldsymbol{\theta }) = \prod _{k=1}^{p} \Bigl [ U_{M}^{(k)} \, U_{C}^{(k)} \Bigr ]. \end{aligned}$$

Expanding this expression explicitly in terms of the matrix representations, we obtain:

19 $$\begin{aligned} U(\boldsymbol{\theta }) = \prod _{k=1}^{p} \left[ \left( \bigotimes _{j=1}^{n} \begin{pmatrix} \cos \beta _k & -i\sin \beta _k \\ -i\sin \beta _k & \cos \beta _k \end{pmatrix} \right) \left( \prod _{(i,j) \in \mathcal {E}} \begin{pmatrix} e^{-i\gamma _k} & 0 & 0 & 0 \\ 0 & e^{i\gamma _k} & 0 & 0 \\ 0 & 0 & e^{i\gamma _k} & 0 \\ 0 & 0 & 0 & e^{-i\gamma _k} \end{pmatrix}_{(i,j)} \right) \right] , \end{aligned}$$

where the notation $$\begin{pmatrix} \cdot \end{pmatrix}_{(i,j)}$$ emphasizes that each two-qubit gate is embedded in the $2^n$-dimensional space, acting non-trivially only on qubits $i$ and $j$, and as the identity on all other qubits.

This expansion into the matrix representation highlights the underlying quantum mechanical operations in the circuit and provides a clear, explicit form for the cost and mixing unitaries, as well as their combined effect in the full unitary $U(\boldsymbol{\theta })$.

This layered structure is crucial as it introduces non-commutativity between the cost and mixing operations, thereby generating complex interference patterns and non-trivial entanglement among the qubits, a necessary ingredient for harnessing quantum advantage.

#### Quantum chaos diagnostics framework

The sensitivity of quantum circuits to parameter variations can be characterized by two primary metrics in this work: the OTOC (Section 2.3) and an estimate of a Lyapunov-like exponent.

To visualize the sensitivity of the QAOA circuit to parameter variations, we perform a systematic scaling analysis of the optimized variational parameters. Consider a scaling factor $s$ that continuously modifies the optimized parameter vector $\boldsymbol{\theta }_{\textrm{opt}}$ via

20 $$\begin{aligned} \boldsymbol{\theta }(s) = s \, \boldsymbol{\theta }_{\textrm{opt}}, \end{aligned}$$

where $s$ varies over a predetermined range $[s_{\min }, s_{\max }]$. For each scaling factor $s$, the corresponding OTOC is computed as in Equation (8). This yields a function $C(s)$ for a fixed QAOA depth $p$.

By systematically varying both the scaling factor $s$ and the circuit depth $p$ (e.g., considering $p$ from 1 to 12), one constructs a two-dimensional array of OTOC values. In this heatmap, the $x$-axis represents the scaling factor $s$, while the $y$-axis represents the QAOA depth $p$. The color intensity in the heatmap reflects the magnitude of the OTOC, thereby visualizing the sensitivity of the circuit across different parameter regimes.

Within each OTOC curve $C(s)$ (for a fixed $p$), the local minima are identified as those points where the function attains a local low value. Denote by $\{s_i\}_{i=1}^{N}$ the set of scaling factors at which these minima occur. The differences between successive minima,

21 $$\begin{aligned} \Delta s_i = s_{i+1} - s_i, \quad i = 1, \ldots , N-1, \end{aligned}$$

are computed, and their average is defined as

22 $$\begin{aligned} \bar{\Delta s} = \frac{1}{N-1} \sum _{i=1}^{N-1} \Delta s_i. \end{aligned}$$

The average spacing $\bar{\Delta s}$ serves as a quantitative measure of the fluctuations in the OTOC and, by extension, as an indicator of the chaotic dynamics.

Furthermore, by calculating the ratios between successive spacings, one can extract a Feigenbaum-like constant, which, in classical chaotic systems, is known to be universal. The average of these ratios over different circuit depths offers insight into the underlying universality of chaos in the QAOA circuit.

To further describe the nature of chaos in the circuit, we perform a statistical analysis on the distribution of the distances $\Delta s_i$ between local minima in the OTOC curves. The steps are as follows:

1. Identification of local minima: For each circuit depth $p$, the function $C(s)$ is examined to identify the local minima at scaling factors $\{s_i\}$.
2. Computation of spacings: The differences $\Delta s_i = s_{i+1} - s_i$ are computed for each identified pair of successive minima.
3. Aggregation and fitting: The collection of all such differences from various depths is aggregated into a single data set. This data is then fitted to several candidate probability distributions, including the normal, lognormal, and power-law distributions.

In our analysis, the lognormal distribution is found to provide a particularly good fit. The probability density function (PDF) of the lognormal distribution is:

23 $$\begin{aligned} P(\Delta s) = \frac{A}{\Delta s\, \sigma \sqrt{2\pi }} \exp \!\Biggl (-\frac{(\ln \Delta s - \mu )^2}{2\sigma ^2}\Biggr ), \end{aligned}$$

where $A$, $\mu$, and $\sigma$ are parameters determined by the fitting procedure. The quality of the fit is assessed by evaluating the sum of squared residuals (SSR) between the empirical histogram of $\Delta s$ and the fitted probability distribution function. This statistical analysis yields a standardized chaos metric, which is robust against variations in circuit parameters and is indicative of the universal features of the chaotic dynamics.

### Hybrid quantum–classical classification models

Building upon the chaos diagnostics, our methodology incorporates a novel hybrid quantum–classical classifier that exploits both quantum feature extraction and chaos diagnostics. Two models are constructed for comparison:

#### Standard hybrid model

In the standard hybrid model, classical data (such as MNIST images) are first preprocessed by flattening and then reducing the dimensionality from 784 to $n_{qubits}$ (in out case 10, 8, 6 or 4). This $n_{qubits}$-dimensional vector is subsequently used to drive a quantum circuit based on the QAOA ansatz, which acts as a feature map to encode the input into a quantum state.

The quantum circuit is constructed as follows. First, each of the one of the qubits is initialized to the state $|0\rangle$ and then transformed into the uniform superposition $|+\rangle$ by the Hadamard gate $H$. This prepares the state

$$\begin{aligned} |+\rangle ^{\otimes n_{qubits}} = H^{\otimes n_{qubits}} |0\rangle ^{\otimes n_{qubits}}. \end{aligned}$$

Next, the circuit applies a single layer (or, in general, $p$ layers) of QAOA operations. For a given layer $k$, the cost unitary is implemented by applying Ising-$ZZ$ interactions between qubits corresponding to the edges in the underlying graph. Mathematically, this is represented by the unitary

$$\begin{aligned} U_C^{(k)} = \prod _{(i,j)\in \mathcal {E}} \exp \Bigl (-i \, \gamma _k \, Z_i Z_j\Bigr ), \end{aligned}$$

where $\gamma _k$ is the cost parameter for the $k$th layer and $Z_i$ denotes the Pauli-$Z$ operator on qubit $i$. Subsequently, a mixing unitary is applied by performing a rotation about the $X$-axis on every qubit:

$$\begin{aligned} U_M^{(k)} = \bigotimes _{q=1}^{n_{qubits}} R_X(2\beta _k), \quad \text {with} \quad R_X(\theta ) = \exp \Bigl (-i\frac{\theta }{2}X\Bigr ), \end{aligned}$$

where $\beta _k$ is the mixing parameter for that layer.

**Figure 1:**
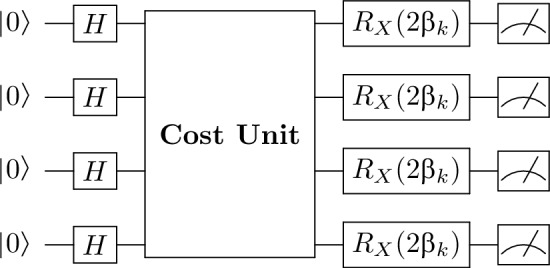
Quantum circuit for one QAOA layer in the standard hybrid model. Each qubit is initialized in the state $|0\rangle$ and then transformed to the superposition state $|+\rangle$ via the Hadamard gate $H$. The block labeled Cost Unit represents the collective application of Ising-$ZZ$ interactions (one for each edge in the graph) with rotation parameter $\gamma _k$. Following this, each qubit is individually rotated about the $X$-axis by an angle $2\beta _k$ using the $R_X$ gate. Finally, measurement in the Pauli-$Z$ basis is performed to extract the quantum feature vector.

Figure 1 shows the quantum circuit (for clarity purposes in the case of 4 qubits input) for one layer of the QAOA ansatz. In this depiction, each qubit is first acted on by a Hadamard gate to produce $|+\rangle$. The cost unitary $U_C^{(k)}$ is then applied via a controlled $ZZ$-interaction across all relevant qubit pairs (the exact connectivity depends on the MaxCut instance), followed by individual RX rotations $R_X(2\beta _k)$ on each qubit. Finally, measurement in the Pauli-$Z$ basis extracts the quantum feature vector.

**Figure 2:**
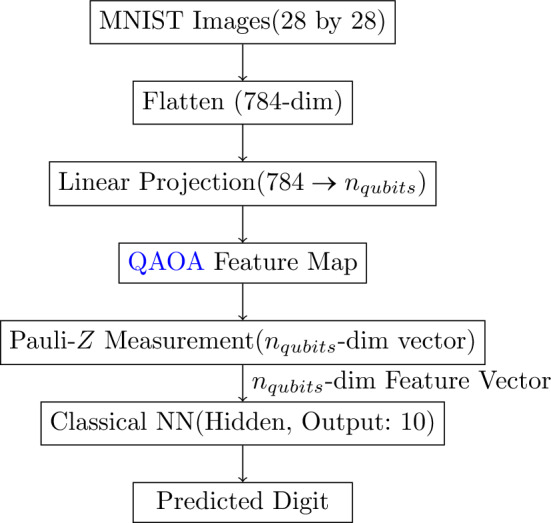
Block diagram of the standard hybrid architecture. MNIST images are preprocessed by flattening and linear projection to a $n_{qubits}$-dimensional vector, which is then encoded into a quantum state using a QAOA-based feature map. Measurement in the Pauli-*Z* basis produces a $n_{qubits}$-dimensional feature vector that is input to a classical neural network for digit classification.

The quantum feature vector, $\textbf{q} \in \mathbb {R}^n_{qubits},$ obtained by measuring the expectation values of the Pauli-$Z$ operators on each qubit, is then provided as input to a classical neural network. This classical component consists of a hidden layer with an output layer with 10 neurons, corresponding to the digit classes. Figure 2 illustrates the overall hybrid architecture, showing the flow from the preprocessed input through the quantum feature extraction and finally into the classical neural network for classification.

#### Chaos-aware hybrid model

The chaos-aware hybrid model extends the standard architecture by incorporating an additional feature that quantifies the degree of chaotic behavior in the QAOA circuit. After obtaining the $n_{qubits}$-dimensional quantum feature vector, a chaos metric is computed through the following process:

1. Parameter scaling and OTOC evaluation: Starting with the optimized variational parameters $\boldsymbol{\theta }_{\textrm{opt}} \in \mathbb {R}^{2p}$ of the QAOA circuit, where the first $p$ components correspond to the cost angles and the remaining $p$ to the mixing angles, a continuous scaling factor $s$ is introduced. For each $s$ in a predefined interval (e.g., $s \in [0.5, 2.0]$), the parameters are rescaled as
  $$\begin{aligned} \boldsymbol{\theta }(s) = s \, \boldsymbol{\theta }_{\textrm{opt}}. \end{aligned}$$
  The out-of-time-ordered correlator is then computed for each $s$, resulting in a function $C(s)$ that encapsulates the circuit’s sensitivity to parameter scaling.
2. Extraction of local minima and average spacing: The local minima of the function $C(s)$ are identified, yielding a sequence $\{s_i\}_{i=1}^{N}$. The spacings between successive minima are calculated as
  $$\begin{aligned} \Delta s_i = s_{i+1} - s_i, \quad i = 1, \ldots , N-1, \end{aligned}$$
  and the average spacing is given by
  $$\begin{aligned} \bar{\Delta s} = \frac{1}{N-1} \sum _{i=1}^{N-1} \Delta s_i. \end{aligned}$$
3. Standardization via lognormal modeling: To transform the raw average spacing into a standardized chaos metric, the value $\bar{\Delta s}$ is mapped using a lognormal probability density function. The lognormal probability distribution function is characterized by the expression
  $$\begin{aligned} P(x) = \frac{A}{x\, \sigma \sqrt{2\pi }} \exp \!\Biggl (-\frac{(\ln x - \mu )^2}{2\sigma ^2}\Biggr ), \end{aligned}$$
  with fixed parameters that were determined through prior statistical analyses. Evaluating this function at $x = \bar{\Delta s}$ yields the chaos feature:
  $$\begin{aligned} f_{\textrm{chaos}} = P\bigl (\bar{\Delta s}\bigr ). \end{aligned}$$

The final augmented feature vector is then formed by concatenating the original $n_{qubits}$-dimensional quantum feature vector with the scalar chaos feature:

$$\begin{aligned} \textbf{q}_{\textrm{aug}} = \left[ \textbf{q}, \, f_{\textrm{chaos}}\right] \in \mathbb {R}^{n_{qubits}+1}. \end{aligned}$$

This $n_{qubits}+1$-dimensional vector serves as the input to a classical neural network that shares a similar architecture to the standard model. By incorporating $f_{\textrm{chaos}}$, the chaos-aware model effectively provides the classical optimizer with an extra degree of feedback concerning the sensitivity of the quantum circuit, leading to improved classification performance.

#### Training and evaluation procedures

Both hybrid models are trained using conventional deep learning techniques. The overall training protocol is as follows:

1. Data preparation: MNIST images are flattened to a 784-dimensional vector and linearly projected to $n\in \{4,6,8,10\}$ dimensions that parametrize the quantum feature map.
2. Model optimization: Both hybrids (Standard vs. Chaos-aware) share the same classical head and are trained end-to-end (Adam, cross-entropy) for 30 epochs.
3. Cross-validated, paired design: For each $n$, we run a multi-run, 5-fold cross-validation with identical seeds/folds for both models, producing $N_\text {pairs}$ paired test accuracies (one per run$\times$fold). This paired design isolates the chaos feature effect.
4. Statistical reporting: For each $n$ we report: model-wise test means±SD; paired mean difference $\Delta$ with 95% t-CI and bootstrap CI (10,000 resamples); exact sign-flip permutation $p$ (10,000 draws); win-rate $\mathbb {P}(\textrm{Chaos}>\textrm{Std})$ with Wilson 95% confidence interval (CI)^17^ and exact two-sided sign-test $p$; paired Cohen’s $d_z$. Per-epoch *train/validation/test* panels show mean±1 SD trajectories.

#### Statistical analysis and reporting

We adopt a paired design across run$\times$fold replicates per configuration $n$. Let $\{(a_i,b_i)\}_{i=1}^{N}$ denote the paired test accuracies (Standard $a_i$, Chaos $b_i$). We summarize per $n$: $\overline{a}\pm s_a$, $\overline{b}\pm s_b$, the paired mean difference $\Delta =\overline{(b_i-a_i)}$, its 95% t-interval and nonparametric bootstrap CI, the exact sign-flip permutation $p$-value, win-rate with Wilson 95% CI and exact sign-test $p$, and the paired effect size $d_z=\Delta /s_\Delta$. Plots aggregate epochs as mean±1 SD across run$\times$fold replicates.

### Implementation details and experimental setup

Our experimental implementation is built upon a combination of quantum circuit simulation and classical deep learning frameworks. Quantum circuits are simulated using a high-level quantum programming library that allows for the construction and execution of parameterized circuits on a default-qubit simulator. Classical computations and neural network training are performed using a widely adopted deep learning framework, which ensures efficient optimization and reproducibility.

Key aspects of our experimental setup include:

- Quantum simulation: The QAOA circuits are simulated on a device that models ideal unitary evolution. All gate operations, including Hadamard, Ising-$ZZ$, and RX rotations, are implemented with precise control over the variational parameters.
- Statistical robustness: To ensure that our chaos diagnostics are robust against statistical fluctuations, certain analyses, such as the generation of OTOC heatmaps are repeated over 30 independent runs. The resulting data are aggregated, and universal features (such as the Feigenbaum–like ratio) are extracted via statistical averaging.
- Distribution fitting: The analysis of the distances between local minima in the OTOC curves involves fitting candidate distributions (normal, lognormal, and power law) to the aggregated data. Nonlinear least-squares optimization techniques are used to obtain the best-fit parameters, and the goodness-of-fit is quantified using measures such as the sum of squared residuals.
- Hybrid model training: The MNIST data set is used as a benchmark to evaluate the performance of hybrid classifiers with both standard and chaos-aware hybrid classifiers. Data preprocessing includes normalization, dimensionality reduction, and partitioning into training and test sets. The quantum feature map is shared between the models, allowing us to isolate the impact of the additional chaos diagnostic feature.

#### Interplay of QAOA, quantum chaos, and hybrid classification

In our hybrid quantum–classical architecture, we repurpose the QAOA not as a solver for combinatorial optimization but as a powerful *feature map* that projects classical data into a high-dimensional quantum Hilbert space. Concretely, each 784 dimensional MNIST image $\textbf{x}$ is first linearly reduced to an *n* dimensional vector of rotation angles $\{\theta _i\}_{i=1}^n$. These angles parameterize a depth-*p* QAOA circuit which transforms the initial state as shown in Equation (2). Acting as a non-linear embedding, QAOA layers generate rich entanglement and interference patterns among the *n* qubits, thus capturing complex pixel correlations that a classical linear map cannot.

To quantify *sensitivity* of this quantum embedding, we introduced the OTOC in Equation (8) as a feature inspired by chaos. Using the OTOC, we extract a single scalar chaos feature that measures the global sensitivity of the circuit to input variations. Finally, we concatenate the *n* local expectation values $\{\langle Z_i\rangle \}$ with the chaos characteristic $\chi$, yielding a vector of characteristic $(n+1)$ dimensional for a classical multi-layer perceptron head (MLP). The entire pipeline.

$$\begin{aligned} \textbf{x}\,\xrightarrow {\,\textrm{Embed}\,} \bigl \{\langle Z_i\rangle ,\,\chi \bigr \} \,\xrightarrow {\,\textrm{MLP}\,} \textrm{softmax}\bigl (W_2\,\sigma (W_1[\langle Z_i\rangle ,\chi ])\bigr ) \end{aligned}$$

is trained from end-to-end. In this way:

- QAOA: provides a highly non-linear, parameterized map that entangles qubits to reveal multi-pixel correlations.
- Chaos (OTOC): delivers a global complementary descriptor of circuit mixing and sensitivity, highlighting distinctions invisible to local readouts.
- Classifier: (MLP) integrates both local and chaos features, utilizing quantum dynamics to enhance discriminatory power.

Although QAOA was devised for combinatorial problems, its layered, non-commuting structure makes it a natural candidate for constructing expressive, entanglement-rich embeddings in supervised learning.

## Results, discussion and limitations

**Figure 3:**
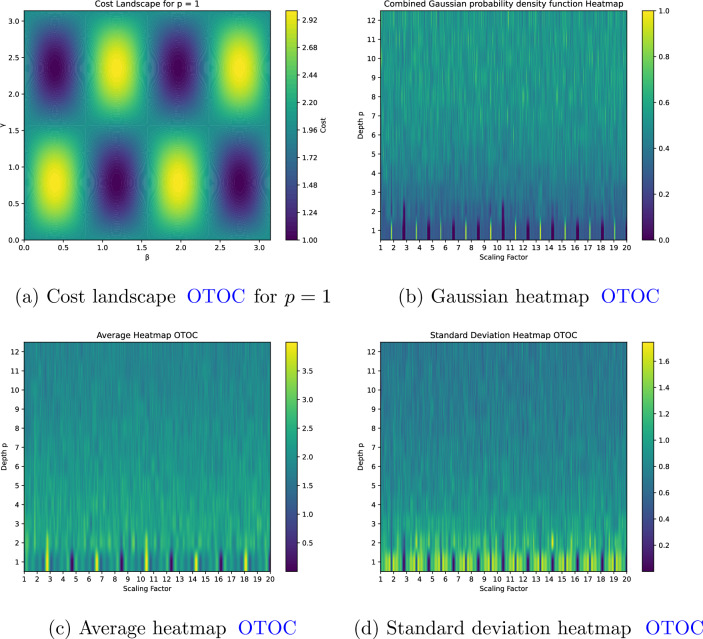
Comparison of different visualizations for OTOC.

We begin our investigation by analyzing the simplest case, $p=1$. For $p=1$, we evaluated the OTOC cost function across a finely resolved grid in the $(\gamma ,\beta )$ parameter space within a QAOA circuit. The resulting heatmap, shown in Figure 3a , reveals a regular alternation between zones of high and low sensitivity. Regions with elevated cost values signal enhanced chaotic fluctuations, whereas areas with lower values suggest relative stability in the circuit’s quantum dynamics. This already known^18^ periodic structure gives the reader an intuitive idea of how even a single-layer QAOA circuit can encapsulate complex chaos signatures, where the interplay between $\gamma$ and $\beta$ governs the emergence of regular patterns in the quantum cost landscape. These findings provide a baseline for our analysis and pave the way for a more detailed exploration of chaotic behavior in deeper circuits.

We continue our investigation by analyzing the sensitivity of a QAOA circuit to chaotic behavior in the parameter landscape. In this study, the combined “Gaussian” heatmap of the OTOC cost function: for each cell in the heatmap, we compute the Gaussian probability density function shown in Figure 3b , $P(x;\mu ,\sigma )= \frac{1}{\sigma \sqrt{2\pi }} \exp \!\left( -\frac{(x-\mu )^2}{2\sigma ^2}\right) ,$ evaluated at a reference value $x_{\textrm{ref}}$ chosen as the global median of the $\mu$-data. In order to focus on the regions corresponding to OTOC minima (i.e., zones where the heatmap values are nearly zero), the computed probability distribution function is clipped to the interval $[0,1]$. Specifically, we compute the average ($\mu$) and standard deviation ($\sigma$) of the OTOC data, derived by averaging the results of 30 independent simulations, with the heatmaps in Figure 3c and Figure 3d displaying these averaged metrics, respectively.

For each circuit depth $p$ (with depth corresponding to the rows of the heatmap and the scaling parameter spanning 20 discrete units), a dedicated algorithm identifies the local minima along the scaling axis. These minima are interpreted as critical points in the parameter landscape. The horizontal distances between consecutive local minima in each row are computed, and the average gap for that depth, denoted by $\langle \Delta s \rangle$, is determined. This average gap serves as a proxy for the density of critical points, thereby providing insight into the landscape’s complexity.

The dependence of $\langle \Delta s \rangle$ on the circuit depth $p$ was modeled by fitting an exponential decay function of the form $f(p) = a\, e^{b\, p} + c,$ where $a$, $b$, and $c$ are the fitting parameters. In our analysis, the best-fit parameters were obtained as $a = 20.5313,\quad b = -0.5675,\quad c = 4.0981.$ Here, the parameter $a$ characterizes the initial scaling of the gap, while the negative exponent $b$ quantitatively confirms an exponential decay of the average gap with increasing depth $p$. The parameter $c$ represents the asymptotic value of the average gap as the depth becomes large.

From the perspective of chaos theory, the exponential decay observed in the average horizontal gap between local minima is a strong indicator of increasing sensitivity to initial conditions in the QAOA circuit. In classical chaotic systems, such sensitivity is typically characterized by exponential divergence of nearby trajectories, quantified by positive Lyapunov exponents. In our quantum setting, as the circuit depth increases, the parameter landscape exhibits a rapid convergence of critical points, effectively “compressing” the parameter space. This phenomenon implies that small variations in the scaling parameter can lead to significant changes in the circuit’s behavior, a hallmark of chaotic dynamics.

**Figure 4:**
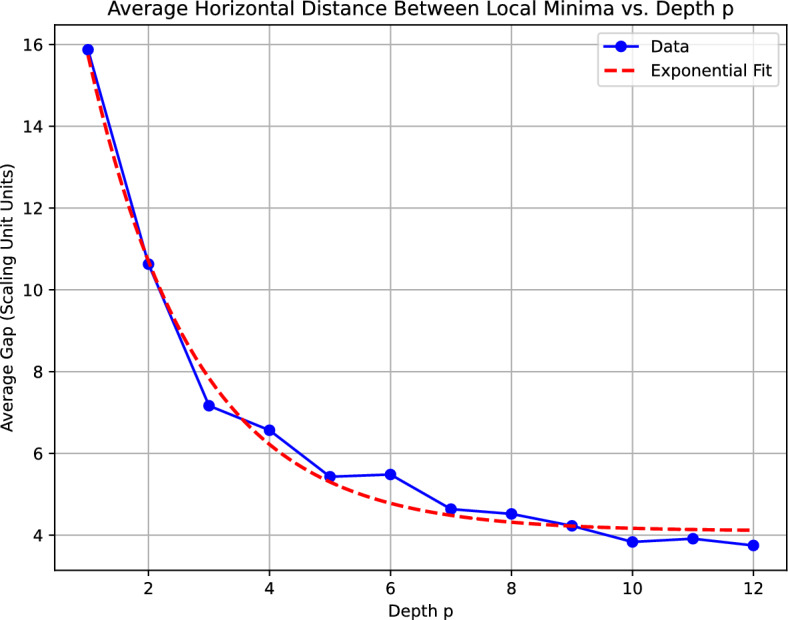
Depth vs. Average OTOC minima distance.

The fitted exponential function shown in Figure 4, $f(p) = 20.5313\, e^{-0.5675\,p} + 4.0981,$ therefore not only provides a quantitative measure of the evolving complexity in the QAOA parameter landscape, but it also underscores the intrinsic connection between circuit depth and chaotic behavior. This result is particularly relevant for quantum deep learning architectures, where increasing circuit depth may render the system more unpredictable and sensitive to parameter fluctuations. In this context, the presence of a rapidly decaying gap between local minima is indicative of a transition towards a regime where traditional parameter tuning becomes challenging, thereby necessitating novel strategies for mitigating chaos-induced unpredictability in quantum computations.

We continue our study by analyzing the distances between local minima in the OTOC versus scaling factor curves for QAOA circuits. The aggregated differences were modeled using several candidate distributions, and the lognormal distribution provided the best fit. Here, we discuss the physical implications of this result in the context of chaos theory and quantum computing.

Many chaotic systems exhibit multiplicative fluctuations, where small variations are not merely added but multiplied through the system’s evolution. If a variable grows (or shrinks) by random multiplicative factors over time, its logarithm is normally distributed. Consequently, the variable itself follows a lognormal distribution.

In the context of our OTOC analysis, the distances between local minima represent the sensitivity of the circuit to small parameter changes. The fact that these distances are best described by a lognormal distribution suggests that they emerge from a multiplicative process. That is, small differences are amplified successively over the layers of the circuit, consistent with the characteristic exponential sensitivity found in chaotic systems.

Chaotic dynamics are often associated with scale-invariant behavior. In our QAOA parameter landscape, the spacing between regions of high sensitivity (i.e., the local minima in the OTOC curves) does not follow a simple additive pattern. Instead, these spacings can vary over several orders of magnitude, a hallmark of multiplicative dynamics.

An analogy can be drawn with the Feigenbaum constant in classical chaos, where the ratios of successive bifurcation intervals converge to a universal constant. Although our aggregated distances do not exactly yield the Feigenbaum constant, the successful lognormal fit implies that the underlying process is multiplicative and scale invariant, similar in spirit to the dynamics observed in systems undergoing period-doubling bifurcations.

The lognormal distribution of the minima distances implies that the QAOA parameter landscape is highly sensitive to small perturbations. In practical terms, this means that even minor errors or miscalibrations in the quantum circuit parameters can be exponentially amplified as the circuit depth increases. This sensitivity is a signature of chaotic dynamics in the quantum realm.

Such error amplification has important implications for quantum computing. Traditional error models often assume additive noise; however, a multiplicative noise process, as indicated by the lognormal distribution, suggests that errors can affect the system over multiple scales. This finding underlines the need for robust error mitigation strategies that specifically address multiplicative noise. Techniques based on renormalization or scaling corrections may prove more effective in these scenarios than methods designed for purely additive errors.

**Figure 5:**
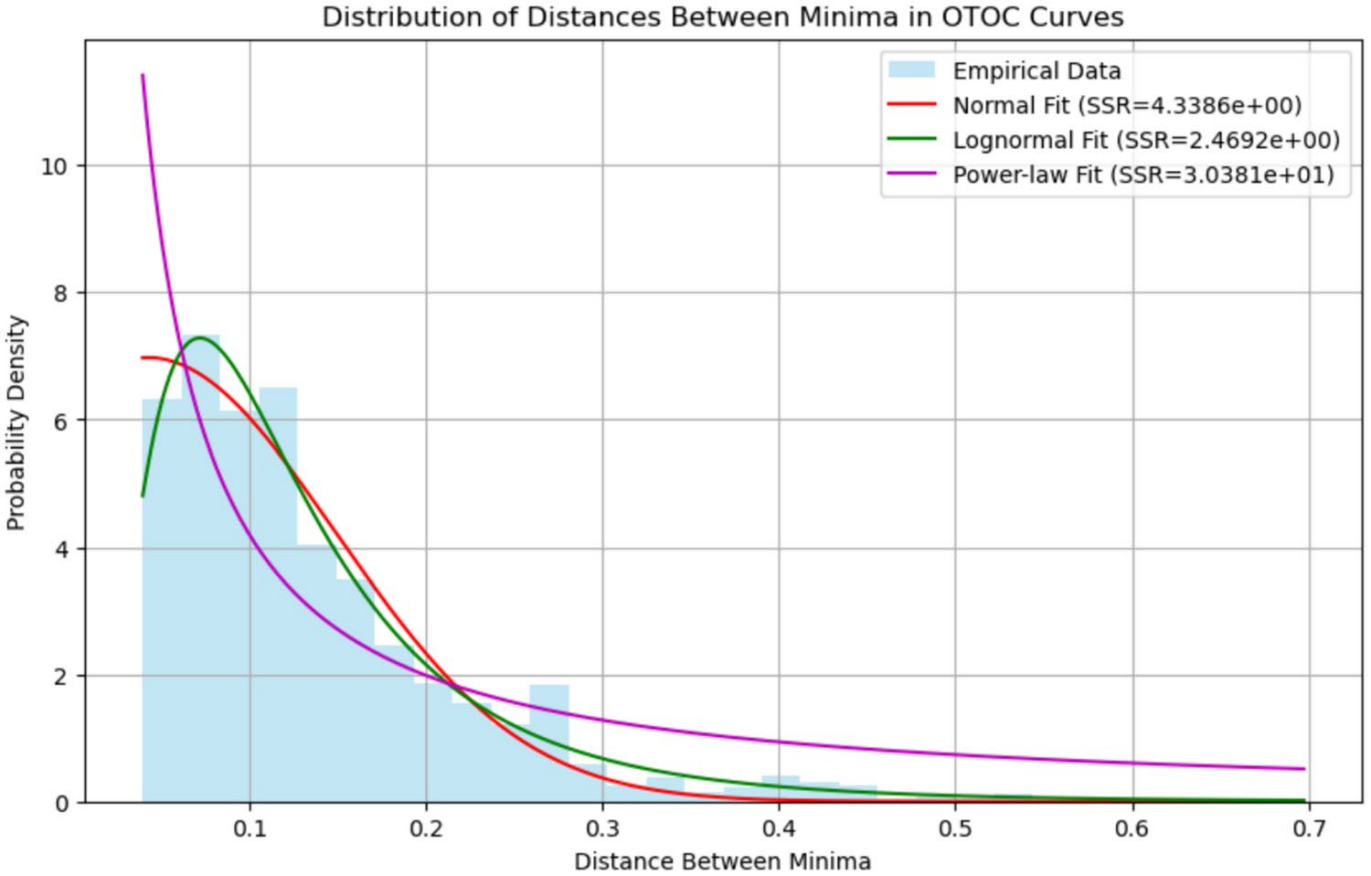
Probability density distance between OTOC minima.

The best fit of the aggregated minima differences is a lognormal, shown in Figure 5, and yielded the following parameters: $A \approx 1.06546633,\quad \mu \approx -2.20167254,\quad \sigma \approx 0.65298225$, and $\text {SSR} = 2.4692.$ In this context, the lognormal probability density function is given by $f(x; A, \mu , \sigma ) = A\, \frac{1}{x \sigma \sqrt{2\pi }} \exp \!\left( -\frac{(\ln (x)-\mu )^2}{2\sigma ^2}\right) ,$ where $A$ is a scaling factor for the distribution, $\mu$ represents the mean of $\ln (x)$, and $\sigma$ is the standard deviation of $\ln (x)$. The low sum-of-squared residuals (SSR) value confirms the reasonable fit of the lognormal model to the data.

The small value of $\mu$ indicates that, on a logarithmic scale, the typical distance between OTOC minima is very small, implying regions of extremely high sensitivity within the parameter landscape. The moderate spread $\sigma$ demonstrates that while most distances are small, there is variability over several scales—this is characteristic of multiplicative, scale-invariant processes.

The lognormal distribution of distances between local minima in the OTOC curves is a natural outcome of multiplicative processes, which are common in chaotic systems. This result implies that:

- Small fluctuations in the QAOA parameter landscape are amplified multiplicatively, leading to an exponential sensitivity to initial conditions.
- The parameter landscape is highly rugged, with densely packed regions of high sensitivity.
- Hardware imperfections and calibration errors in quantum circuits may be exponentially amplified, necessitating the development of specialized error mitigation strategies that account for multiplicative noise.

These insights are critical for the design and operation of deep quantum circuits and quantum deep learning architectures, where control over such chaotic dynamics is essential for reliable quantum computation.

Building on our prior analysis, we integrate a chaos diagnostic feature into our quantum deep learning architecture. In previous experiments, we pre-fitted a lognormal distribution to the distances between local minima in the OTOC curves which capture the overall statistical shape of the minima spacing. These parameters summarize the multiplicative, scale-invariant behavior characteristic of chaotic systems.

In practice, however, even when using the same circuit, the weight configuration changes over the course of training. For each new set of weights, we obtain a fresh OTOC versus scaling factor curve. The following diagnostic steps are performed dynamically for each circuit instance:

1. OTOC evaluation: The OTOC is computed across the 20 scaling units, generating a sensitivity curve that reflects the current state of the circuit.
2. Extraction of average minima distance: Local minima are identified in this curve, and the average horizontal distance between consecutive minima is calculated. This average spacing constitutes the raw measure of the circuit’s current sensitivity. Standardization via a pre-fitted lognormal PDF: The measured average spacing is then input into the pre-fitted lognormal probability density function. Although the pre-fitted parameters were derived from a large ensemble of circuit instances, applying the lognormal probability distribution function to the current average spacing yields a normalized chaos diagnostic feature. This step is essential: without it, the raw spacing—despite being informative—would not be directly comparable across different weight configurations.

The role of the mapping through the lognormal probability distribution function is to transform the raw average minima distance into a standardized value that reflects how typical or atypical the current spacing is relative to our historical data. Even though the same circuit architecture is used for both the pre-fitting and the current measurement, variations in the weight configuration can lead to different spacing outcomes. The lognormal mapping thus provides a calibrated measure of the circuit’s chaotic sensitivity, which is then incorporated into the hybrid model as an additional feature.

The chaos-aware hybrid model leverages this chaos diagnostic to adapt to the exponential sensitivity inherent in chaotic quantum systems. Specifically, the pre-fitted lognormal parameters capture the multiplicative dynamics—where small perturbations are amplified over successive circuit layers—while the dynamic measurement provides a current data point representing the circuit’s sensitivity. By combining these elements, the model achieves several key benefits:

- Sensitive dependence on parameters: The diagnostic feature quantifies the degree of multiplicative noise, thereby highlighting regions of the parameter space where small errors may be exponentially amplified.
- Dynamic adaptation: As the weights change during training, the chaos-informed feature reflects real-time adjustments in the circuit’s chaotic behavior, offering continuous feedback to the optimizer.
- Enhanced robustness: Incorporating a normalized chaos metric into the training process helps mitigate the deleterious effects of multiplicative error amplification, ultimately leading to improved convergence and generalization.

### Experimental implementation and results

Two hybrid quantum–classical classifiers were implemented and compared: (i) a StandardHybrid, which uses only the *n* local Pauli-*Z* expectation values, and (ii) a ChaosAwareHybrid, which appends a single scalar chaos feature. All experiments were carried out on a balanced subset of MNIST (1 000 images, 100 per digit), split into 800/100/100 train/validation/test. Each $28\times 28$ image was flattened to a 784-dimensional vector and reduced via PCA to an *n*-dimensional real embedding with $n\in \{10,8,6,4\}$.

The PCA embedding serves as rotation angles $\{\theta _i\}_{i=1}^n$ for a depth-$p=2$ QAOA circuit on *n* qubits, simulated with PennyLane’s default.qubit. Each layer alternates

$$\begin{aligned} e^{-i\gamma _k H_C},\quad H_C=\sum _{\langle i,j\rangle }\tfrac{1 - Z_iZ_j}{2}, \qquad e^{-i\beta _k H_M},\quad H_M=\sum _{i=1}^n X_i, \end{aligned}$$

thereby generating entanglement and non-linear feature maps in a $2^n$-dimensional Hilbert space.

To quantify global sensitivity, for each training batch we compute

$$\begin{aligned} \mathcal {C}(s) = \bigl \langle \psi \bigr |\,[A(s),B]^\dagger \,[A(s),B]\,\bigl |\psi \bigr \rangle , \quad A(s)=U(s\boldsymbol{\gamma },s\boldsymbol{\beta })^\dagger \,Z_1\,U(s\boldsymbol{\gamma },s\boldsymbol{\beta }),\, B=X_1, \end{aligned}$$

sampling ten scaling factors $s\in [0.5,2.0]$ and fitting a log-normal curve to $\{(s,\mathcal {C}(s))\}$. The extracted scrambling timescale $\chi$ is appended to the *n* local $\langle Z_i\rangle$, yielding an $(n+1)$-dimensional feature vector.

**Figure 6:**
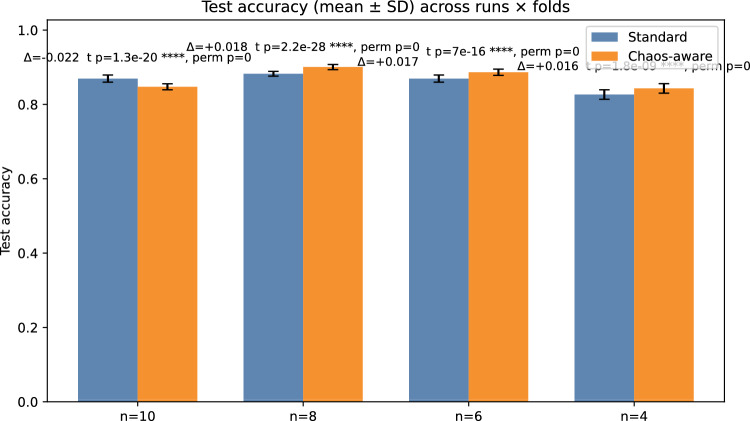
Test accuracy (mean±SD) across run$\times$fold pairs, Standard vs Chaos-aware, for $n\in \{4,6,8,10\}$. Text annotations show $\Delta$ (Chaos–Std) with paired $t$-test $p$ and permutation $p$.

**Figure 7:**
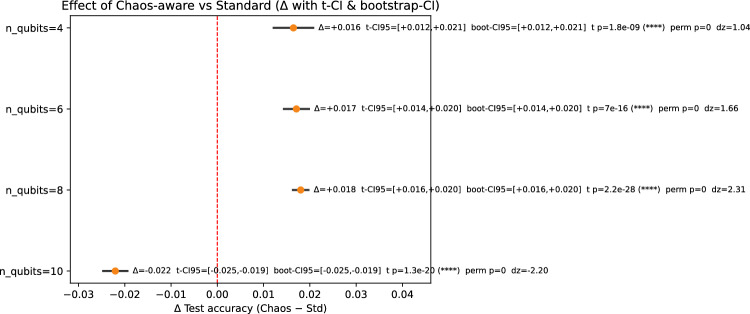
Paired differences $\Delta$ (Chaos–Std) with 95% t-CIs and bootstrap CIs per $n$. Vertical dashed line at $\Delta =0$.

**Figure 8:**
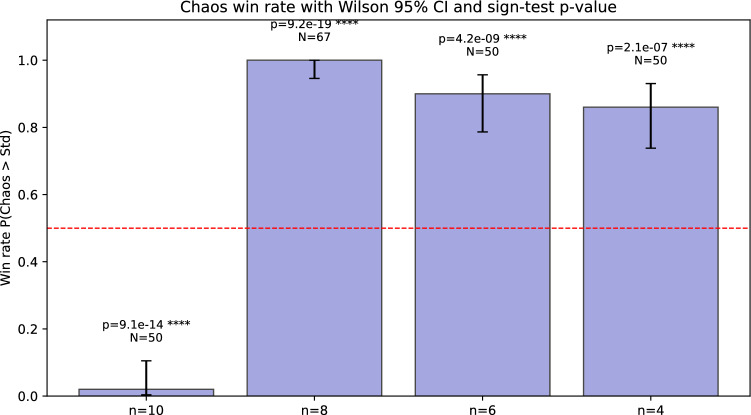
Win-rate $\mathbb {P}(\textrm{Chaos}>\textrm{Std})$ with Wilson 95% intervals and exact sign-test $p$-values.

**Figure 9:**
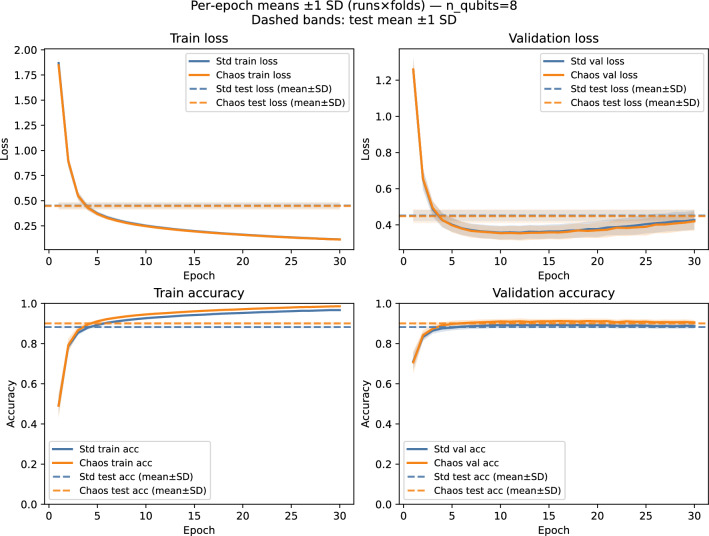
Per-epoch mean±1 SD trajectories (runs$\times$folds) for $n=8$: top row: loss (left: train; right: validation; dotted: test); bottom row: accuracy (left: train; right: validation; dotted: test). Analogous panels for $n\in \{4,6,10\}$ are provided in the Supplement.

Both hybrids share an identical MLP head and are trained for 30 epochs using Adam ($\textrm{lr}=10^{-3}$). Evaluation is reported via paired, cross-validated test accuracy summaries (Table 1) and publication-grade figures: accuracy bars (Figure 6), paired differences with CIs (Figure 7), win-rates with Wilson intervals (Figure 8), and representative per-epoch trajectories for $n=8$ (Figure 9).

**Table 1:** Multi-run, paired test-set comparison (mean±SD across run$\times$fold pairs). $\Delta$ denotes Chaos–Std. CIs are 95%. Win-rate is $\mathbb {P}(\textrm{Chaos}>\textrm{Std})$ with Wilson CI.

*n*	$N_{\textrm{pairs}}$	Std acc	Chaos acc	$\Delta$	t-CI	boot-CI	$p_t$	Win-rate (%)
4	50	0.8266 $\pm 0.0128$	0.8431 $\pm 0.0128$	$+0.0165$	$[+0.0120,$ $+0.0209]$	$[+0.0122,$ $+0.0209]$	$1.8\times 10^{-9}$	86.0 [73.8, 93.0]
6	50	0.8695 $\pm 0.0096$	0.8866 $\pm 0.0085$	$+0.0171$	$[+0.0142,$ $+0.0200]$	$[+0.0143,$ $+0.0198]$	$7.0\times 10^{-16}$	90.0 [78.6, 95.7]
8	67	0.8826 $\pm 0.0064$	0.9006 $\pm 0.0069$	$+0.0180$	$[+0.0161,$ $+0.0199]$	$[+0.0162,$ $+0.0199]$	$2.2\times 10^{-28}$	100.0 [94.6, 100.0]
10	50	0.8675 $\pm 0.0091$	0.8474 $\pm 0.0081$	$-0.0220$	$[-0.0249,$ $-0.0192]$	$[-0.0248,$ $-0.0194]$	$1.3\times 10^{-20}$	2.0 [0.4, 10.5]

Additional statistics: permutation $p\approx 0$ for all $n$; paired effect sizes $d_z\in \{1.042,1.663,2.314,-2.203\}$ for $n\in \{4,6,8,10\}$, respectively

The chaos-derived scalar acts as a global many-body sensitivity measure that complements local Pauli-$Z$ readouts. In our paired, cross-validated analysis (Table 1 and Figs. 6–9), the chaos-aware model *consistently* improves test accuracy at $n\in \{4,6,8\}$, with $\Delta \in [+0.016,+0.018]$, all confidence intervals excluding zero (both parametric and bootstrap), exact permutation $p\approx 0$, and high win-rates (86–100%). At $n=10$, $\Delta =-0.022$ with 2% wins, indicating over-sensitivity. The best average accuracy occurs at $n=8$ ($0.9006\pm 0.0069$) with $\Delta =+0.0180$ and 100% wins. Per-epoch panels reveal stable alignment of train/val/test accuracy at $n=8$, modest under-variation at $n\in \{4,6\}$, and volatility at $n=10$. These findings identify a “Goldilocks” width where expressivity and sensitivity are balanced and the chaos feature provides a net generalization benefit.

Summarizing: across 50–67 paired run$\times$fold evaluations per configuration, chaos-aware models significantly outperform the standard variant at $n\in \{4,6,8\}$ ($\Delta \approx +0.016$–$+0.018$; all CIs exclude 0; permutation $p\approx 0$; win-rates $\ge 86\%$), with the strongest result at $n=8$ ($\textrm{Chaos}=0.9006\pm 0.0069$; $\Delta =+0.0180$; 100% wins); at $n=10$, chaos significantly underperforms ($\Delta =-0.0220$; 2% wins), revealing a width-induced sensitivity transition.

Our conclusions target resource-limited hybrid workflows (1,000-sample subset, depth $p{=}2$) where sensitivity–expressivity trade-offs are pronounced. We do not claim asymptotic quantum advantage. Rather, we provide a diagnostic that measurably improves generalization in constrained regimes—consistent with recent perspectives that practical quantum machine learning advantages are most plausible under structured or reduced scenarios)^19^. Extending to larger datasets, deeper circuits, and hardware noise will require additional engineering (e.g., chaos-aware regularization and noise-robust OTOC estimation).

## Summary and future scope

In this work, we presented a comprehensive investigation of how chaotic signatures manifest in QAOA circuits, focusing especially on the interplay between circuit depth and parameter-space dynamics. Drawing upon a variety of methods—ranging from OTOC and spectral statistics to distribution-fitting of minima distances—we have shown that even relatively shallow QAOA circuits can exhibit exponential sensitivity to small variations in their variational parameters. This sensitivity manifests in a densely packed, rugged parameter landscape in which small shifts in angles can trigger significant changes in circuit behavior. Such findings underscore the nuanced complexity faced by researchers seeking to optimize and deploy deeper quantum circuits in practical settings.

We began by exploring the OTOC cost function for the simplest scenario, $p = 1$. Our visualizations revealed recognizable patterns of alternating high- and low-sensitivity regions, illustrating that even a single-layer circuit can encapsulate a wide array of chaotic features. This baseline analysis paved the way for more sophisticated explorations of deeper circuits. An increase in circuit depth $p$ corresponded to a greater incidence of local minima in the parameter-space cost function, thereby complicating the search for an optimal solution.

Next, we evaluated distinct ways of visualizing and quantifying these chaotic dynamics. By creating combined “Gaussian” heatmaps at a reference value $x_{\textrm{ref}}$ and then clipping them to the interval $[0,1]$, we isolated high-sensitivity zones. We showed that these clipped Gaussian metrics highlight the presence of exponentially amplified fluctuations, which align with classical chaos theory concepts (such as exponential divergence).

Additionally, our examination of the average horizontal gap $\langle \Delta s \rangle$ between successive local minima showed a strong exponential decay with increasing circuit depth. Mathematically fitted to $f(p) = a \, e^{b \, p} + c,$ this decay function confirmed that as $p$ grows, small changes in parameter scaling can be significantly magnified. In classical dynamical systems, a positive Lyapunov exponent signifies such exponential sensitivity to initial conditions. Our quantum results thus parallel core ideas from classical chaos, strengthening the argument that deep quantum circuits must grapple with chaos-like phenomena.

Building on these analyses, we discovered that the distances between local minima follow a *lognormal* distribution more closely than power-law or simple normal distributions. Lognormal behavior is closely tied to multiplicative noise processes, where small deviations compound in a multiplicative manner across multiple circuit layers. This insight is particularly relevant for error mitigation and quantum control. Traditional quantum error models often focus on additive noise, but our results suggest that noise in chaotic quantum settings may often be multiplicative—especially if small errors become exponentiated through repeated gate applications.

Finally, these chaos diagnostics have tangible effects in a quantum deep-learning context: in paired, cross-validated tests, the chaos-aware hybrid achieves its best average performance at $n=8$ ($0.9006\pm 0.0069$), outperforming the standard hybrid by $\Delta =+0.0180$ with 100% wins. At $n\in \{4,6\}$ the gains remain significant ($\Delta \approx +0.016$–$+0.017$); at $n=10$ the effect reverses ($\Delta =-0.022$), revealing a sensitivity transition. The chaos diagnostic feature was derived from the pre-fitted lognormal parameters that summarized global multiplicative behavior, whereas the actual average minima distance was measured locally for each new circuit configuration. By standardizing this average distance through the pre-fitted lognormal PDF, we achieved an interpretable measure that effectively flagged chaotic regions in real time, allowing the optimizer to respond accordingly.

The key contributions of this paper are threefold. First, we develop an advanced diagnostic framework for QAOA circuits that quantitatively characterizes quantum chaos through multiple measures. Our framework integrates several diagnostic tools, including the computation of OTOCs and Lyapunov-like exponent estimations^20^ derived from the trajectory of parameter updates during optimization. Furthermore, we introduce a chaos diagnostic feature obtained by analyzing the distribution of distances between local minima in the OTOC curves. In our analysis, we fit these minima differences to a lognormal distribution using pre–fitted parameters: $A \approx 1.06546633,\quad \mu \approx -2.20167254,\quad \sigma \approx 0.65298225$, and $\text {SSR} = 2.4692$, derived from extensive preliminary experiments. This statistical model provides a normalized, quantitative measure of chaotic behavior that is sensitive to the underlying circuit dynamics.

Second, we propose a hybrid quantum–classical architecture in which a parametrized QAOA feature map is augmented by an on-the-fly chaos diagnostic and applied to MNIST classification. Rather than fixing $n=4$, we sweep the number of qubits $n\in \{10,8,6,4\}$, implementing each feature map as a depth–$p=2$ QAOA circuit that encodes the PCA-reduced input via *RY* rotations followed by Ising-*ZZ* entanglers and *RX* mixers. At each training batch, we compute the OTOC, fit a log-normal curve over ten scale factors $s\in [0.5,2.0]$, and extract a scalar scrambling timescale $\chi$. The *ChaosAwareHybrid* model concatenates $\chi$ to the *n* Pauli-*Z* expectation values, whereas the *StandardHybrid* uses only those *n* local features.

Our empirical sweep using a paired, multi-run 5-fold cross-validation reveals statistically significant gains at $n\in \{4,6,8\}$, with $\Delta \approx +0.016$–$+0.018$ and all 95% CIs excluding zero; at $n=10$ the effect reverses ($\Delta =-0.022$). The best mean accuracy is achieved at $n=8$ ($0.9006\pm 0.0069$; 100% paired wins). These results confirm a “Goldilocks” regime of circuit width in which the chaos feature improves generalization; see Table 1 and Figs. 6–9.

Third, our investigation provides insights into the interplay between quantum chaos and algorithmic performance in variational quantum circuits. By analyzing the behavior of the QAOA circuits under various parameter scalings and depths, we are able to correlate the emergence of chaotic signatures with performance improvements in classification tasks. Our numerical experiments reveal that the chaos diagnostic feature not only serves as a robust indicator of the circuit’s dynamical behavior but also enhances the training process by effectively guiding the classical optimization routine. This suggests that leveraging quantum chaos metrics can play a critical role in optimizing quantum circuits, particularly in scenarios where the available quantum resources are limited.

In summary, this paper addresses a crucial challenge in the realm of variational quantum computing—namely, the optimization and robust performance of quantum circuits in the presence of complex dynamical behaviors. By merging quantum chaos diagnostics with the QAOA framework, we not only enhance the performance of hybrid quantum–classical models but also contribute to a deeper understanding of the dynamical processes that govern quantum circuit evolution. Our findings underscore the potential of chaos-aware techniques to pave the way for more reliable and efficient quantum algorithms, thereby accelerating the transition of quantum computing from theoretical promise to practical reality.

From a broader standpoint, our results contribute to several key areas of quantum information science and quantum computing research:

- Chaotic dynamics in NISQ algorithms: As quantum hardware transitions from small-scale demonstration devices to larger, more capable near-term devices (often referred to as NISQ hardware), the possibility of deeper and more entangled circuits grows. Our findings lay a foundation for understanding how chaos may hamper parameter optimization and degrade reliability in such deeper circuits.
- Distribution Analysis of Parameter Landscapes: By rigorously analyzing and fitting distributions (normal, lognormal, power-law) to aggregated minima distances, we introduced a framework for characterizing the sensitivity and scale invariance of quantum parameter landscapes. This extends prior works that have treated QAOA performance with simpler or purely numerical approaches, providing a more nuanced statistical viewpoint.
- Novel chaos diagnostics for hybrid quantum–classical systems: Our introduction of clipped Gaussian heatmaps, dynamic minima-distance tracking, and lognormal-based standardization offers new methods for bridging chaos theory and variational quantum algorithms. The synergy between these diagnostics and the end-to-end learning process is a valuable step toward robust quantum deep learning.
- Implications for error models and mitigation: Recognizing that the observed chaos-like behavior often arises through multiplicative processes prompts us to re-examine error models. If typical hardware errors scale multiplicatively with circuit depth, new strategies may be necessary. Potential lines of research include specialized noise correction protocols and the design of circuits that are intrinsically less sensitive to chaotic instabilities.

Although our study has largely focused on MaxCut instances within QAOA, the implications of chaotic parameter landscapes reach beyond any one optimization problem. We foresee several avenues for expanding upon the present work:

1. Extension to Other Variational Algorithms. Many of our methods—OTOC computations, minima-distance distribution analysis, clipped heatmaps—can be seamlessly transferred to other variational frameworks, such as the Variational Quantum Eigensolver (VQE) or quantum neural networks (QNNs). Investigating chaos in these broader contexts could enhance their performance and stability in tasks ranging from quantum chemistry to machine learning.
2. Higher-Dimensional Landscapes. While our scaling factor approach effectively captures 1D slices of the parameter space, real QAOA deployments often feature dozens or hundreds of parameters. A multi-parameter analysis (beyond a 1D scaling axis) could reveal even richer chaotic structures. High-dimensional chaos detection methods, including fractal dimension estimates or partial structural entropy, might be adapted for quantum parameter landscapes.
3. Feigenbaum-Like Constants in Quantum Settings. Our discussion briefly mentioned the universality of period-doubling routes to chaos in classical systems, marked by the Feigenbaum constant. We have not yet identified a strictly analogous universal ratio in QAOA or other quantum circuits. Nonetheless, the repeated glimpses of universal, scale-invariant signatures in our distribution-fitting analyses hint at possible routes to discovering *quantum Feigenbaum constants*. Should such constants exist, they would represent a major theoretical insight into how chaos scales in variational circuits.
4. Advanced Error Mitigation Techniques. The revelation that noise can be multiplicative and scale exponentially with depth underscores the need to tailor error mitigation to chaotic quantum circuits. Techniques such as randomization, layering reordering, or adaptive gate design could be extended to dampen multiplicative amplification. Another promising idea involves exploiting the clipped Gaussian metrics to localize dangerously sensitive subregions of parameter space, and either avoid them during optimization or apply specialized recalibration.
5. Practical Integration with Quantum-Classical Optimizers. Looking beyond static analyses, one might embed chaos diagnostics directly into quantum-classical hybrid optimizers. For instance, if an optimizer detects that the circuit has entered a region of high chaos—perhaps recognized via lognormal-based flags or abrupt changes in OTOC distributions—it can adapt the learning rate, shift parameter search bounds, or switch to alternative gradient-free subroutines. Such an adaptive approach would effectively convert chaos from a stumbling block into a dynamic signal that informs the learning strategy.
6. Connections to Quantum Machine Learning. Recent advances in quantum machine learning have explored deeper, more expressive ansatz structures. The same expressiveness that grants these circuits potential advantage might also induce chaotic instabilities. By systematically applying the chaos diagnostics we introduced, future quantum machine learning architectures could maintain expressivity without succumbing to unmanageable parameter-sensitivity issues. Notably, the chaos-aware hybrid model approach we tested—where the chaotic diagnostic feature was integrated into classical classification tasks—could be extended to generative modeling or reinforcement learning tasks on quantum hardware.
7. Hardware Constraints and Realistic Implementations. Finally, any progress toward taming or harnessing chaos in QAOA must contend with hardware constraints. NISQ devices are characterized by decoherence, gate errors, crosstalk, and other noise sources. It remains an open question whether the chaotic behaviors we observe in simulation become more or less pronounced on real quantum hardware. Verifying that hardware experiments mirror our theoretical results could unlock new engineering insights, potentially leading to specialized hardware designs optimized for controlling chaotic effects.

Overall, our findings confirm that chaos is not merely a theoretical curiosity but an inevitable feature of complex quantum circuits, particularly as circuit depth increases. By drawing inspiration from classical chaos theory—integrating measures like OTOCs, spectral statistics, minima-distance distributions, and lognormal fits—we provide a rigorous framework for diagnosing, understanding, and mitigating chaotic dynamics in QAOA. Moreover, the synergy we demonstrated between chaos diagnostics and quantum deep learning strategies underscores the practical impact of studying chaos in quantum computing: increased classification accuracy, more stable optimization, and better resilience to error accumulation.

In light of these developments, future research can shift toward building automated, real-time chaos detection toolkits for quantum circuits, perhaps as library functions seamlessly incorporated into quantum software frameworks. Addressing high-dimensional parameter spaces, implementing specialized error mitigation, and verifying these techniques on real hardware will be key milestones on the path to robust, scalable, and chaos-resilient quantum computations. We believe that such advances will not only sharpen our theoretical grasp of quantum chaos but also pave the way for more reliable, high-performance applications of quantum computers to problems across physics, chemistry, machine learning, and beyond.

## Supplementary Information

Supplementary Information.

## Data Availability

This study uses the publicly available MNIST handwritten digit dataset (Modified National Institute of Standards and Technology). The dataset license is Creative Commons Attribution–ShareAlike 3.0, as stated in the official Keras dataset documentation (https://keras.io/api/datasets/mnist/). The license permits reuse and redistribution provided that appropriate attribution is given and the share-alike terms are respected (https://creativecommons.org/licenses/by-sa/3.0/). We use MNIST only as an external benchmark dataset and do not claim ownership of the underlying images. We do not redistribute the full MNIST image files with this submission; instead, to support reproducibility we provide in the Supplementary Material (i) the full pre–processing and training scripts, (ii) fixed random seeds, and (iii) the exact indices defining the balanced 1 000-image subset and the 800/100/100 train/validation/test split used in the experiments.
