# Supplementary material for: Exploiting quantum chaos diagnostics in QAOA for enhanced hybrid quantum classical deep learning classification
Source: Sci Rep. 2026 May 19;16:15744. doi: 10.1038/s41598-026-51870-8 (PMC13190741; doi:10.1038/s41598-026-51870-8)
Supplement: Supplementary file 1 — Supplementary Information. [file 41598_2026_51870_MOESM1_ESM.pdf]

In [2]:
```
#!/usr/bin/env python3
"""
Quantum Chaos Diagnostics in QAOA with OTOC, Heatmap, and Distribution Analysis
-------------------------------------------------------------------------------------
This module implements a research framework for diagnosing quantum chaos in QAOA.
It builds a QAOA circuit for the MaxCut problem on a small graph and computes several diagnostics,
including:
  - The out-of-time-ordered correlator (OTOC), which measures the sensitivity of the evolved operator
    to small perturbations.
  - Spectral statistics (eigenphase spacings) of the QAOA unitary.
  - A Lyapunov-like exponent computed from the parameter-update trajectory.
  - A heatmap of OTOC values versus (i) QAOA depth and (ii) a scaling factor on the optimized parameters.

Additionally, the code analyzes the distribution of the distances between local minima in the OTOC curves.
It fits several candidate distributions (normal, lognormal, and power-law) to the aggregated minima differences,
plots the empirical histogram overlaid with the fitted curves, and prints the fitted parameters.

For robust results, the code repeats the heatmap generation 30 times and averages:
  - The OTOC heatmap,
  - The estimated Feigenbaum-like ratio from the heatmap,
  - And the distribution fitting results.

Author: Villalba-Diez
Date: 20250203
"""

import pennylane as qml
from pennylane import numpy as np
import matplotlib.pyplot as plt
from scipy.optimize import minimize, curve_fit
import time, copy
from scipy.signal import find_peaks
import pandas as pd
import numpy as np


# ===============================================================================
# Helper Functions for Cost Hamiltonian and Initial State
# ===============================================================================
```



```
def cost_hamiltonian_maxcut(n_qubits, edges):
    """
    Construct the cost Hamiltonian for the MaxCut problem.
    For each edge (i, j), the cost term is: 0.5*(I - Z_i Z_j)
    """
    dim = 2 ** n_qubits
    H = np.zeros((dim, dim), dtype=complex)
    I2 = np.eye(2)
    Z = np.array([[1, 0], [0, -1]])
    for (i, j) in edges:
        ops = []
        for qubit in range(n_qubits):
            ops.append(Z if (qubit == i or qubit == j) else I2)
        ZZ = ops[0]
        for op in ops[1:]:
            ZZ = np.kron(ZZ, op)
        H += 0.5 * (np.eye(dim) - ZZ)
    return H

def all_plus_state(n_qubits):
    """
    Generate the all-plus state (uniform superposition) as a state vector.
    """
    dim = 2 ** n_qubits
    state = np.ones(dim) / np.sqrt(dim)
    return state

# ===============================================================================
# QAOA Experiment Class (Using PennyLane)
# ===============================================================================

class QAOAExperiment:
    def _ansatz(self, params):
        """
        QAOA ansatz for MaxCut.
        Parameters:
            params (array[float]): Array of length 2*p (first p are gamma angles, next p are beta angles).
        Returns:
            state (array[complex]): The statevector produced by the QAOA circuit.
        """
```



```
        # Prepare uniform superposition
        for q in range(self.n_qubits):
            qml.Hadamard(wires=q)
        # Apply p layers of cost and mixing unitaries.
        for layer in range(self.p):
            gamma = params[layer]
            beta = params[self.p + layer]
            for (i, j) in self.edges:
                qml.IsingZZ(-gamma, wires=[i, j])
            for q in range(self.n_qubits):
                qml.RX(2 * beta, wires=q)
        return qml.state()

    def __init__(self, n_qubits, p, edges):
        """
        Initialize a QAOA experiment.
        Parameters:
            n_qubits (int): Number of qubits (graph nodes).
            p (int): QAOA depth (number of layers).
            edges (list[tuple]): List of edges defining the MaxCut instance.
        """
        self.n_qubits = n_qubits
        self.p = p
        self.edges = edges
        self.dev = qml.device("default.qubit", wires=n_qubits)
        self.qaoa_circuit = qml.QNode(self._ansatz, self.dev)
        self.H_cost = cost_hamiltonian_maxcut(n_qubits, edges)
        self.psi0 = all_plus_state(n_qubits)
        self.parameter_trajectory = []
        self.cost_history = []

    def simulate_circuit(self, params):
        """Simulate the QAOA circuit and return the statevector."""
        return np.array(self.qaoa_circuit(params))

    def cost_function(self, params):
        """
        Evaluate the expectation value of the cost Hamiltonian for the QAOA state.
        """
        psi = self.simulate_circuit(params)
        cost = np.real(np.vdot(psi, self.H_cost @ psi))
```



```
        self.parameter_trajectory.append(params.copy())
        self.cost_history.append(cost)
        return cost

    def optimize_parameters(self, initial_params):
        """
        Optimize QAOA parameters using COBYLA.
        """
        self.parameter_trajectory = []
        self.cost_history = []
        def callback(xk):
            cost = self.cost_function(xk)
            print(f"Current parameters: {xk}, Cost: {cost:.4f}")
        result = minimize(self.cost_function, initial_params, method="COBYLA",
                          callback=callback, options={"maxiter": 100, "disp": True})
        return result

    def get_unitary(self, params):
        """
        Obtain the unitary matrix representation of the QAOA circuit.
        """
        U = qml.matrix(self.qaoa_circuit)(params)
        return np.array(U)

    def compute_otoc(self, params, A_op, B_op):
        """
        Compute the out-of-time-ordered correlator (OTOC).
        The OTOC is defined as:
            C = <psi0| [A(t), B]† [A(t), B] |psi0>
        where A(t) = U† A U.
        Parameters:
            params (array[float]): QAOA parameters.
            A_op (ndarray): Operator A.
            B_op (ndarray): Operator B.
        Returns:
            otoc (float): The computed OTOC value.
        """
        U = self.get_unitary(params)
        A_t = U.conj().T @ A_op @ U
        commutator = A_t @ B_op - B_op @ A_t
        psi0 = self.psi0
```

```
        otoc = np.real(np.vdot(psi0, (commutator.conj().T @ commutator) @ psi0))
        return otoc

    def compute_spectral_statistics(self, params, plot=True):
        """
        Compute the eigenphase spacing distribution of the QAOA unitary and check its fit to
        a Wigner-Dyson distribution versus a Poisson distribution.

        Parameters:
            params (array[float]): QAOA parameters.
            plot (bool): Whether to display the plot.

        Returns:
            spacings_norm (ndarray): Normalized eigenphase spacings (mean = 1).
            ssr_results (dict): A dictionary with SSR values for the Wigner-Dyson and Poisson fits.
        """
        # Obtain the unitary matrix and compute its eigenvalues and eigenphases.
        U = self.get_unitary(params)
        eigenvals = np.linalg.eigvals(U)
        eigenphases = np.sort(np.angle(eigenvals))

        # Compute spacings between consecutive (unwrapped) eigenphases and add the wrap-around spacing.
        spacings = np.diff(np.unwrap(eigenphases))
        wrap_spacing = (2 * np.pi - eigenphases[-1] + eigenphases[0])
        spacings = np.append(spacings, wrap_spacing)

        # Normalize spacings to have a mean of 1.
        spacings_norm = spacings / np.mean(spacings)

        # Compute the empirical histogram.
        hist, bin_edges = np.histogram(spacings_norm, bins=30, density=True)
        bin_centers = (bin_edges[:-1] + bin_edges[1:]) / 2

        # Define the theoretical distributions.
        # Wigner-Dyson distribution for the GOE is given by:
        def wigner_dyson(s):
            return (np.pi / 2) * s * np.exp(-np.pi * s**2 / 4)

        # Poisson distribution (for uncorrelated levels) is given by:
        def poisson(s):
            return np.exp(-s)
```



```
        # Compute the sum of squared residuals (SSR) for each theoretical distribution.
        ssr_wd = np.sum((hist - wigner_dyson(bin_centers))**2)
        ssr_poisson = np.sum((hist - poisson(bin_centers))**2)

        ssr_results = {'Wigner-Dyson': ssr_wd, 'Poisson': ssr_poisson}
        print("SSR for Wigner-Dyson fit: {:.4e}".format(ssr_wd))
        print("SSR for Poisson fit: {:.4e}".format(ssr_poisson))

        if plot:
            plt.figure(figsize=(8, 5))
            plt.hist(spacings_norm, bins=30, density=True, alpha=0.75, color="teal", label="Empirical Data")
            s_fit = np.linspace(0, np.max(spacings_norm), 300)
            plt.plot(s_fit, wigner_dyson(s_fit), 'r-', label=f"Wigner-Dyson (SSR={ssr_wd:.4e})")
            plt.plot(s_fit, poisson(s_fit), 'b--', label=f"Poisson (SSR={ssr_poisson:.4e})")
            plt.xlabel("Normalized Eigenphase Spacing")
            plt.ylabel("Probability Density")
            plt.title("Spectral Statistics of the QAOA Unitary")
            plt.legend()
            plt.grid(True)
            plt.show(block=True)

        return spacings_norm, ssr_results

    def compute_lyapunov_exponent(self):
        """
        Estimate a Lyapunov-like exponent from the logged parameter trajectory.
        """
        traj = np.array(self.parameter_trajectory)
        if traj.shape[0] < 2:
            print("Not enough data to compute Lyapunov exponent.")
            return None, None, None
        dists = np.linalg.norm(np.diff(traj, axis=0), axis=1)
        epsilon = 1e-12  # to avoid log(0)
        log_dists = np.log(dists + epsilon)
        iterations = np.arange(1, len(log_dists)+1)
        coeffs = np.polyfit(iterations, log_dists, 1)
        lambda_est = coeffs[0]
        plt.figure(figsize=(8, 5))
        plt.plot(iterations, log_dists, "o", label="log(distances)")
        plt.plot(iterations, np.polyval(coeffs, iterations), "r-", label=f"Fit (λ = {lambda_est:.4f})")
```



```
        plt.xlabel("Iteration")
        plt.ylabel("Log Distance")
        plt.title("Lyapunov Exponent Estimation")
        plt.legend()
        plt.grid(True)
        plt.show()
        return lambda_est, iterations, log_dists

# ==============================================================================
# Heatmap Generation and Feigenbaum-like Constant Estimation
# ==============================================================================

def otoc_heatmap():
    """
    Vary the QAOA depth (p) and, for each depth, scan over a range of scaling factors
    applied to the optimized parameters. Compute the OTOC for each combination and display
    the results as a heatmap.
    """
    n_qubits = 4
    edges = [(0, 1), (1, 2), (2, 3), (3, 0)]
    depth_range = range(1, 13)  # p = 1 to 12.
    scaling_factors = np.linspace(0.1, 20, 1000)
    heatmap_data = np.zeros((len(depth_range), len(scaling_factors)))

    def single_qubit_operator(n_qubits, op, target):
        op_list = []
        I2 = np.eye(2)
        for q in range(n_qubits):
            op_list.append(op if q == target else I2)
        M = op_list[0]
        for m in op_list[1:]:
            M = np.kron(M, m)
        return M

    Z = np.array([[1, 0], [0, -1]])
    X = np.array([[0, 1], [1, 0]])
    A_op = single_qubit_operator(n_qubits, Z, 0)
    B_op = single_qubit_operator(n_qubits, X, 0)

    for i, p in enumerate(depth_range):
        print(f"Simulating for QAOA depth p = {p}")
```



```
        experiment = QAOAExperiment(n_qubits, p, edges)
        initial_params = np.random.uniform(0, np.pi, 2*p)
        opt_result = experiment.optimize_parameters(initial_params)
        optimal_params = opt_result.x

        for j, scale in enumerate(scaling_factors):
            scaled_params = optimal_params * scale
            otoc_val = experiment.compute_otoc(scaled_params, A_op, B_op)
            heatmap_data[i, j] = otoc_val

    plt.figure(figsize=(10, 6))
    extent = [scaling_factors[0], scaling_factors[-1], depth_range[0]-0.5, depth_range[-1]+0.5]
    plt.imshow(heatmap_data, aspect='auto', extent=extent, origin='lower', cmap='viridis')
    plt.colorbar(label='OTOC Value')
    plt.xlabel('Scaling Factor on Parameters')
    plt.ylabel('QAOA Depth (p)')
    plt.title('OTOC Heatmap across QAOA Depths and Parameter Scaling')
    plt.show()

    return heatmap_data, scaling_factors, depth_range

def estimate_feigenbaum_from_heatmap(heatmap_data, scaling_factors):
    """
    For each QAOA depth (each row in heatmap_data), identify local minima in the OTOC vs. scaling factor curve.
    Compute the differences between the positions of successive minima, then compute the ratio between consecutive
    Average these ratios over all depths to estimate a Feigenbaum-like constant.
    Returns (avg_ratio, std_ratio).
    """
    feigenbaum_ratios = []
    for row in heatmap_data:
        minima_indices, _ = find_peaks(-row)
        if len(minima_indices) < 3:
            continue
        minima_positions = scaling_factors[minima_indices]
        differences = np.diff(minima_positions)
        ratios = differences[:-1] / differences[1:]
        feigenbaum_ratios.extend(ratios)
    if feigenbaum_ratios:
        avg_ratio = np.mean(feigenbaum_ratios)
        std_ratio = np.std(feigenbaum_ratios)
        return avg_ratio, std_ratio
```

```python
    else:
        return None, None

def analyze_minima_differences(heatmap_data, scaling_factors):
    """
    For each row in the heatmap (each QAOA depth), identify local minima in the OTOC vs. scaling factor curve.
    Compute the differences between successive minima positions, aggregate these differences across all depths,
    and fit the resulting distribution to candidate functions (normal, lognormal, and power-law).
    Returns the aggregated differences and a dictionary of fitted parameters and SSR for each candidate.
    """
    from scipy.stats import norm, lognorm
    aggregated_differences = []
    for row in heatmap_data:
        minima_indices, _ = find_peaks(-row)
        if len(minima_indices) < 2:
            continue
        minima_positions = scaling_factors[minima_indices]
        differences = np.diff(minima_positions)
        aggregated_differences.extend(differences)
    aggregated_differences = np.array(aggregated_differences)

    # Candidate probability density functions.
    def normal_pdf(x, A, mu, sigma):
        return A * norm.pdf(x, mu, sigma)

    def lognormal_pdf(x, A, mu, sigma):
        return A * lognorm.pdf(x, s=sigma, scale=np.exp(mu))

    def power_law(x, A, alpha):
        return A * x ** (-alpha)

    hist, bin_edges = np.histogram(aggregated_differences, bins=30, density=True)
    bin_centers = (bin_edges[:-1] + bin_edges[1:]) / 2

    fit_results = {}

    try:
        popt_norm, _ = curve_fit(normal_pdf, bin_centers, hist, p0=[1, np.mean(aggregated_differences), np.std(aggr
        residuals_norm = hist - normal_pdf(bin_centers, *popt_norm)
        ssr_norm = np.sum(residuals_norm**2)
        fit_results['normal'] = {'params': popt_norm, 'SSR': ssr_norm}
```



```python
    except Exception as e:
        fit_results['normal'] = {'error': str(e)}

    try:
        popt_lognorm, _ = curve_fit(lognormal_pdf, bin_centers, hist, p0=[1, np.log(np.mean(aggregated_differences)
        residuals_lognorm = hist - lognormal_pdf(bin_centers, *popt_lognorm)
        ssr_lognorm = np.sum(residuals_lognorm**2)
        fit_results['lognormal'] = {'params': popt_lognorm, 'SSR': ssr_lognorm}
    except Exception as e:
        fit_results['lognormal'] = {'error': str(e)}

    try:
        popt_power, _ = curve_fit(power_law, bin_centers, hist, p0=[1, 1])
        residuals_power = hist - power_law(bin_centers, *popt_power)
        ssr_power = np.sum(residuals_power**2)
        fit_results['powerlaw'] = {'params': popt_power, 'SSR': ssr_power}
    except Exception as e:
        fit_results['powerlaw'] = {'error': str(e)}

    plt.figure(figsize=(10, 6))
    plt.hist(aggregated_differences, bins=30, density=True, alpha=0.5, label="Empirical Data", color="skyblue")
    x_fit = np.linspace(np.min(aggregated_differences), np.max(aggregated_differences), 300)
    if 'params' in fit_results.get('normal', {}):
        plt.plot(x_fit, normal_pdf(x_fit, *fit_results['normal']['params']), 'r-',
                 label=f"Normal Fit (SSR={fit_results['normal']['SSR']:.4e})")
    if 'params' in fit_results.get('lognormal', {}):
        plt.plot(x_fit, lognormal_pdf(x_fit, *fit_results['lognormal']['params']), 'g-',
                 label=f"Lognormal Fit (SSR={fit_results['lognormal']['SSR']:.4e})")
    if 'params' in fit_results.get('powerlaw', {}):
        plt.plot(x_fit, power_law(x_fit, *fit_results['powerlaw']['params']), 'm-',
                 label=f"Power-law Fit (SSR={fit_results['powerlaw']['SSR']:.4e})")
    plt.xlabel("Distance Between Minima")
    plt.ylabel("Probability Density")
    plt.title("Distribution of Distances Between Minima in OTOC Curves")
    plt.legend()
    plt.grid(True)
    plt.show()

    return aggregated_differences, fit_results

# ==============================================================================
```



```python
# Main Execution
# ==============================================================================

def main():
    print("=== Quantum Chaos Diagnostics in QAOA ===")

    # Define experiment settings.
    n_qubits = 4
    p = 2  # QAOA depth for each experiment.
    edges = [(0, 1), (1, 2), (2, 3), (3, 0)]
    num_runs = 30  # Number of independent runs for heatmap averaging.

    # Run a single QAOA experiment and optimize parameters.
    experiment = QAOAExperiment(n_qubits, p, edges)
    initial_params = np.random.uniform(0, np.pi, 2 * p)
    print("Initial parameters:", initial_params)
    opt_result = experiment.optimize_parameters(initial_params)
    optimal_params = opt_result.x
    print("\nOptimal parameters found:", optimal_params)
    print("Final cost value: {:.4f}".format(experiment.cost_history[-1]))

    # Plot cost function history.
    plt.figure(figsize=(8, 5))
    plt.plot(experiment.cost_history, "o-", color="navy")
    plt.xlabel("Iteration")
    plt.ylabel("Cost")
    plt.title("Cost Function History during Optimization")
    plt.grid(True)
    plt.show()

    # Compute and plot the Lyapunov-like exponent.
    lambda_est, iterations, log_dists = experiment.compute_lyapunov_exponent()
    if lambda_est is not None:
        print(f"Estimated Lyapunov exponent: {lambda_est:.4f}")

    # Compute and plot spectral statistics of the QAOA unitary.
    # This is the added functionality.
    print("Computing spectral statistics...")
    experiment.compute_spectral_statistics(optimal_params)

    # Generate OTOC heatmaps over multiple runs.
```



```python
    all_heatmaps = []
    for run in range(num_runs):
        print(f"Run {run+1}/{num_runs} for heatmap generation...")
        heatmap_data, scaling_factors, depth_range = otoc_heatmap()
        all_heatmaps.append(heatmap_data)
    avg_heatmap = np.mean(np.array(all_heatmaps), axis=0)
    std_heatmap = np.std(np.array(all_heatmaps), axis=0)

    # ✅ Convert NumPy arrays to Pandas DataFrames before saving
    df_avg_heatmap = pd.DataFrame(avg_heatmap)
    df_std_heatmap = pd.DataFrame(std_heatmap)

    # ✅ Define correct filenames
    avg_heatmap_filename = "avg_heatmap.csv"
    std_heatmap_filename = "std_heatmap.csv"

    # ✅ Save to CSV correctly
    df_avg_heatmap.to_csv(avg_heatmap_filename, index=False, header=False)
    df_std_heatmap.to_csv(std_heatmap_filename, index=False, header=False)


    # Plot the averaged OTOC heatmap.
    plt.figure(figsize=(10, 6))
    extent = [scaling_factors[0], scaling_factors[-1], depth_range[0]-0.5, depth_range[-1]+0.5]
    plt.imshow(avg_heatmap, aspect='auto', extent=extent, origin='lower', cmap='viridis')
    plt.colorbar(label='OTOC Value')
    plt.xlabel('Scaling Factor on Parameters')
    plt.ylabel('QAOA Depth (p)')
    plt.title('Averaged OTOC Heatmap over 30 Runs')
    plt.show()

    # Plot the standard deviation heatmap.
    plt.figure(figsize=(10, 6))
    extent = [scaling_factors[0], scaling_factors[-1], depth_range[0]-0.5, depth_range[-1]+0.5]
    plt.imshow(std_heatmap, aspect='auto', extent=extent, origin='lower', cmap='viridis')
    plt.colorbar(label='OTOC Value')
    plt.xlabel('Scaling Factor on Parameters')
    plt.ylabel('QAOA Depth (p)')
    plt.title('Std OTOC Heatmap over 30 Runs')
    plt.show()
```

```python
    # Estimate the Feigenbaum-like ratio from the averaged heatmap.
    avg_ratio, std_ratio = estimate_feigenbaum_from_heatmap(avg_heatmap, scaling_factors)
    if avg_ratio is not None:
        print(f"\nEstimated Feigenbaum-like ratio from averaged heatmap: {avg_ratio:.4f} ± {std_ratio:.4f}")
    else:
        print("\nNot enough data to estimate a Feigenbaum-like ratio from the averaged heatmap.")

    # Analyze the distribution of minima differences.
    aggregated_differences, fit_results = analyze_minima_differences(avg_heatmap, scaling_factors)
    print("\nFitting results for the distribution of minima differences (averaged over 30 runs):")
    for dist_name, result in fit_results.items():
        if "params" in result:
            print(f"{dist_name.capitalize()} Fit: Params = {result['params']}, SSR = {result['SSR']:.4e}")
        else:
            print(f"{dist_name.capitalize()} Fit: Error = {result['error']}")

if __name__ == "__main__":
    main()
```



```
=== Quantum Chaos Diagnostics in QAOA ===
Initial parameters: [2.23082129 0.36817736 2.35505024 2.44105184]
Current parameters: [3.23082129 0.36817736 2.35505024 2.44105184], Cost: 1.8197
Current parameters: [2.23082129 1.36817736 2.35505024 2.44105184], Cost: 2.1633
Current parameters: [2.23082129 0.36817736 3.35505024 2.44105184], Cost: 3.2833
Current parameters: [2.23082129 0.36817736 2.35505024 3.44105184], Cost: 3.2931
Current parameters: [2.12312551 0.12614865 1.67512061 1.75729793], Cost: 1.1497
Current parameters: [ 2.00551455 -0.13816281  0.9325922   2.36141602], Cost: 1.8333
Current parameters: [ 2.00685999 -0.13513916  1.89182266  1.40908646], Cost: 1.4858
Current parameters: [1.89471729 0.2277837  1.67512061 1.75729793], Cost: 1.3083
Current parameters: [2.43016064 0.45405537 1.56765255 1.56583717], Cost: 2.0222
Current parameters: [ 2.10642433 -0.04242579  1.5518576   1.89371627], Cost: 1.2223
Current parameters: [2.15538719 0.1262615  1.86895758 1.91185019], Cost: 1.6318
Current parameters: [2.10188827 0.04616386 1.69942694 1.66682354], Cost: 1.3067
Current parameters: [2.1781722  0.18722596 1.58354786 1.73541517], Cost: 1.3536
Current parameters: [2.06466925 0.14550095 1.66555927 1.76211308], Cost: 1.1852
Current parameters: [2.12576937 0.1468244  1.69381626 1.74342139], Cost: 1.1469
Current parameters: [2.13517238 0.14435683 1.73352876 1.79069361], Cost: 1.0985
Current parameters: [2.17432147 0.17325776 1.73166399 1.82987087], Cost: 1.1044
Current parameters: [2.14791165 0.10970824 1.7834349  1.79796092], Cost: 1.1258
Current parameters: [2.11040715 0.1552303  1.73810554 1.80566246], Cost: 1.1203
Current parameters: [2.13621316 0.15473309 1.74167668 1.78238687], Cost: 1.1020
Current parameters: [2.14830883 0.1383719  1.71012052 1.77585341], Cost: 1.0937
Current parameters: [2.16503282 0.11472957 1.72135804 1.77244325], Cost: 1.0825
Current parameters: [2.18977362 0.12301705 1.71967627 1.78955888], Cost: 1.0677
Current parameters: [2.21663498 0.11360813 1.71783877 1.80233094], Cost: 1.0554
Current parameters: [2.24058924 0.1238535  1.7301141  1.79020228], Cost: 1.0552
Current parameters: [2.23771471 0.12179114 1.74387276 1.79670796], Cost: 1.0608
Current parameters: [2.25678415 0.12985703 1.70804544 1.80403048], Cost: 1.0525
Current parameters: [2.26752705 0.10448063 1.69810939 1.7931463 ], Cost: 1.0484
Current parameters: [2.26701301 0.10917123 1.6756808  1.77190354], Cost: 1.0846
Current parameters: [2.27255736 0.09731403 1.70519031 1.80397852], Cost: 1.0401
Current parameters: [2.27892009 0.08892289 1.71045232 1.81425258], Cost: 1.0358
Current parameters: [2.28702591 0.07608665 1.70973155 1.81787856], Cost: 1.0305
Current parameters: [2.29578218 0.06429845 1.70843635 1.82305828], Cost: 1.0257
Current parameters: [2.30450355 0.06109408 1.70711765 1.83555113], Cost: 1.0258
Current parameters: [2.30165326 0.06695757 1.71159703 1.8199753 ], Cost: 1.0267
Current parameters: [2.29390817 0.04976454 1.71348524 1.82108276], Cost: 1.0216
Current parameters: [2.2964462  0.03900472 1.70398047 1.81546275], Cost: 1.0178
Current parameters: [2.29454773 0.02397052 1.70156544 1.81251709], Cost: 1.0154
Current parameters: [2.30617753 0.02075564 1.70678808 1.80407452], Cost: 1.0140
```



```
Current parameters: [2.30182552 0.02242511 1.70742406 1.79783724], Cost: 1.0172
Current parameters: [2.31650676 0.0140982  1.70008558 1.81101732], Cost: 1.0107
Current parameters: [2.319565   0.00665027 1.70998013 1.82004028], Cost: 1.0047
Current parameters: [2.32821014 0.00903813 1.71738587 1.83047366], Cost: 1.0062
Current parameters: [ 2.31759951 -0.00871825  1.7119411   1.82053083], Cost: 1.0007
Current parameters: [ 2.32443617 -0.01619333  1.70013959  1.82203029], Cost: 0.9994
Current parameters: [ 2.33167998 -0.02305222  1.70466422  1.81088797], Cost: 0.9985
Current parameters: [ 2.32661689 -0.02301153  1.70110008  1.806124  ], Cost: 1.0028
Current parameters: [ 2.33946034 -0.02944034  1.71166162  1.82057483], Cost: 0.9919
Current parameters: [ 2.35299864 -0.02587402  1.71524536  1.82651565], Cost: 0.9911
Current parameters: [ 2.35527579 -0.02917102  1.70858732  1.82732193], Cost: 0.9901
Current parameters: [ 2.35612068 -0.04238778  1.71187704  1.83493256], Cost: 0.9862
Current parameters: [ 2.3630437  -0.05569895  1.71392812  1.8310831 ], Cost: 0.9811
Current parameters: [ 2.36961795 -0.06970624  1.71547652  1.82956062], Cost: 0.9762
Current parameters: [ 2.3754536  -0.08410799  1.71700324  1.83014773], Cost: 0.9714
Current parameters: [ 2.37509575 -0.09514751  1.71830221  1.8411229 ], Cost: 0.9696
Current parameters: [ 2.36759903 -0.10638873  1.71675608  1.83342978], Cost: 0.9679
Current parameters: [ 2.37406673 -0.11515665  1.72722519  1.82945098], Cost: 0.9628
Current parameters: [ 2.38307178 -0.12597426  1.73259209  1.82530064], Cost: 0.9573
Current parameters: [ 2.39197774 -0.13679151  1.73813127  1.82116192], Cost: 0.9518
Current parameters: [ 2.40111172 -0.1476405   1.74320559  1.81700722], Cost: 0.9463
Current parameters: [ 2.41006218 -0.1584548   1.74867372  1.81286228], Cost: 0.9408
Current parameters: [ 2.41891051 -0.16924316  1.75435924  1.80872299], Cost: 0.9354
Current parameters: [ 2.42711062 -0.17981412  1.76131926  1.80463498], Cost: 0.9299
Current parameters: [ 2.43455796 -0.1900546   1.76955491  1.80063692], Cost: 0.9244
Current parameters: [ 2.44260106 -0.20055855  1.77680271  1.79656438], Cost: 0.9191
Current parameters: [ 2.45348055 -0.21102597  1.7758005   1.79266488], Cost: 0.9153
Current parameters: [ 2.45549328 -0.22261103  1.78421501  1.78674274], Cost: 0.9100
Current parameters: [ 2.46218294 -0.23055348  1.7955986   1.78415034], Cost: 0.9050
Current parameters: [ 2.46436971 -0.24410757  1.80287353  1.78579976], Cost: 0.8988
Current parameters: [ 2.46629481 -0.25772989  1.80985579  1.78827266], Cost: 0.8928
Current parameters: [ 2.46932181 -0.27057036  1.81804364  1.79002175], Cost: 0.8873
Current parameters: [ 2.47824561 -0.27263577  1.83069441  1.78957621], Cost: 0.8878
Current parameters: [ 2.46315748 -0.26913533  1.8226187   1.7898079 ], Cost: 0.8885
Current parameters: [ 2.46884984 -0.27978145  1.81584742  1.777602  ], Cost: 0.8836
Current parameters: [ 2.46882281 -0.29474942  1.81224193  1.78026664], Cost: 0.8785
Current parameters: [ 2.47060368 -0.30913395  1.80669303  1.77845994], Cost: 0.8760
Current parameters: [ 2.47278606 -0.30898648  1.80388316  1.76324611], Cost: 0.8835
Current parameters: [ 2.46496972 -0.30782826  1.80144342  1.77863399], Cost: 0.8793
Current parameters: [ 2.46417889 -0.31664498  1.81861178  1.7805553 ], Cost: 0.8697
Current parameters: [ 2.46868261 -0.32427936  1.82648064  1.79073638], Cost: 0.8641
```



```
Current parameters: [ 2.4723436  -0.33325838  1.83859776  1.78892242], Cost: 0.8590
Current parameters: [ 2.47256433 -0.32676557  1.8520066   1.79362755], Cost: 0.8646
Current parameters: [ 2.46494844 -0.3354087   1.83939477  1.7899653 ], Cost: 0.8588
Current parameters: [ 2.46869523 -0.35029773  1.83680779  1.79127931], Cost: 0.8522
Current parameters: [ 2.46972602 -0.36114138  1.83643289  1.78008321], Cost: 0.8486
Current parameters: [ 2.47394101 -0.37551426  1.83941506  1.78338494], Cost: 0.8415
Current parameters: [ 2.47813427 -0.38996002  1.84206775  1.78667746], Cost: 0.8344
Current parameters: [ 2.48234247 -0.40435701  1.84485409  1.79005304], Cost: 0.8271
Current parameters: [ 2.48663191 -0.4184216   1.8481436   1.79418919], Cost: 0.8195
Current parameters: [ 2.49100871 -0.43186238  1.85184468  1.79972378], Cost: 0.8119
Current parameters: [ 2.49539873 -0.44511812  1.85556738  1.80566475], Cost: 0.8043
Current parameters: [ 2.49978582 -0.45843001  1.85930807  1.81146949], Cost: 0.7966
Current parameters: [ 2.50417919 -0.47153112  1.86290736  1.81781339], Cost: 0.7890
Current parameters: [ 2.50856282 -0.48493386  1.86679653  1.82330568], Cost: 0.7810
Current parameters: [ 2.51279091 -0.49625777  1.86867826  1.83302638], Cost: 0.7753
Current parameters: [ 2.51577253 -0.50319417  1.88234718  1.83248101], Cost: 0.7671
Current parameters: [ 2.51946813 -0.51488045  1.89199044  1.83152106], Cost: 0.7574
Current parameters: [ 2.52259385 -0.52577883  1.90193525  1.82743491], Cost: 0.7478
Current parameters: [ 2.5259389  -0.53677499  1.91214672  1.82464893], Cost: 0.7386
Current parameters: [ 2.52960204 -0.54726194  1.92313512  1.82468114], Cost: 0.7302
Current parameters: [ 2.5259389  -0.53677499  1.91214672  1.82464893], Cost: 0.7386

Optimal parameters found: [ 2.5259389  -0.53677499  1.91214672  1.82464893]
Final cost value: 0.7386

   Return from subroutine COBYLA because the MAXFUN limit has been reached.

   NFVALS =  100   F = 7.386185E-01    MAXCV = 0.000000E+00
   X = 2.525939E+00  -5.367750E-01   1.912147E+00   1.824649E+00
```

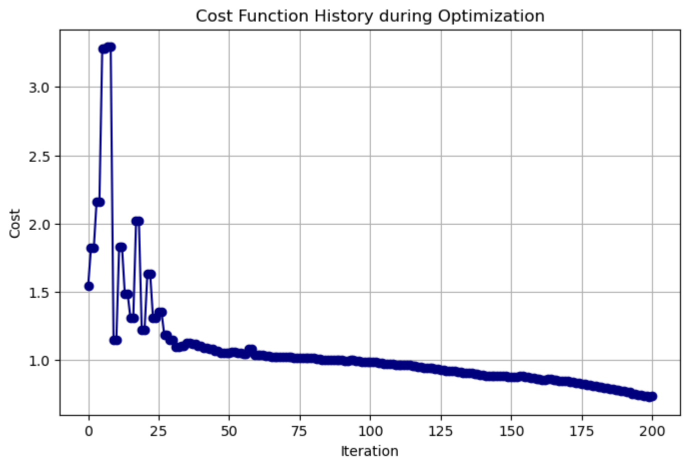

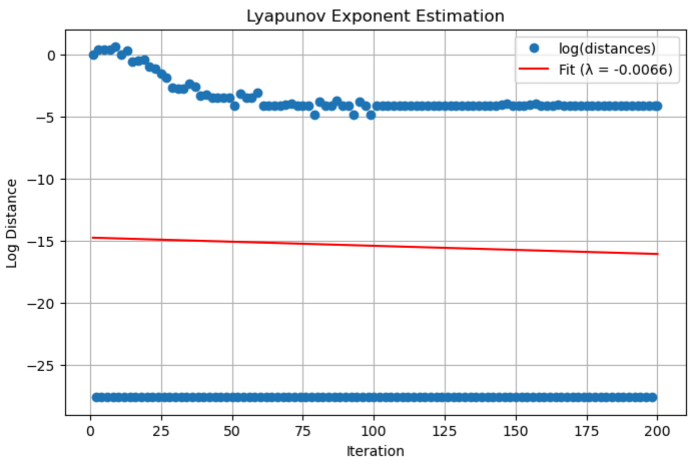


```
Estimated Lyapunov exponent: -0.0066
Computing spectral statistics...
SSR for Wigner-Dyson fit: 1.0483e+01
SSR for Poisson fit: 8.6568e+00
```


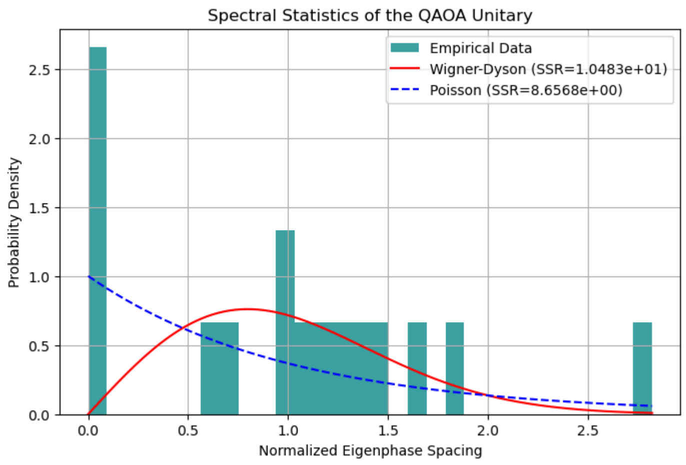


```
Run 1/30 for heatmap generation...
Simulating for QAOA depth p = 1
Current parameters: [3.54626007 2.46510652], Cost: 1.6945
Current parameters: [3.54626007 3.46510652], Cost: 2.6963
Current parameters: [4.11758104 1.6443798 ], Cost: 2.2693
Current parameters: [3.83192055 2.05474316], Cost: 2.9173
Current parameters: [3.30000856 2.42197652], Cost: 1.9190
Current parameters: [3.57304538 2.71366747], Cost: 1.2478
Current parameters: [3.65585977 2.94955256], Cost: 1.4049
Current parameters: [3.61445258 2.83161002], Cost: 1.2331
Current parameters: [3.67342385 2.81090642], Cost: 1.1526
Current parameters: [3.79465015 2.78042392], Cost: 1.0425
Current parameters: [3.91957115 2.78486734], Cost: 1.0104
Current parameters: [3.93793452 2.66122355], Cost: 1.0611
Current parameters: [3.97900624 2.8948331 ], Cost: 1.1701
Current parameters: [3.9492887  2.83985022], Cost: 1.0664
Current parameters: [3.88835896 2.78640403], Cost: 1.0142
Current parameters: [3.92158141 2.75368206], Cost: 1.0002
Current parameters: [3.93123531 2.72396061], Cost: 1.0050
Current parameters: [3.92640836 2.73882133], Cost: 1.0008
Current parameters: [3.92901177 2.75609554], Cost: 1.0004
Current parameters: [3.90953809 2.76363691], Cost: 1.0023
Current parameters: [3.92867694 2.75695141], Cost: 1.0005
Current parameters: [3.922426   2.74986821], Cost: 1.0000
Current parameters: [3.92137243 2.74610672], Cost: 1.0001
Current parameters: [3.92629619 2.74933866], Cost: 1.0000
Current parameters: [3.92758823 2.75080336], Cost: 1.0000
Current parameters: [3.92689933 2.747481  ], Cost: 1.0000
Current parameters: [3.9253197  2.74935059], Cost: 1.0000
Current parameters: [3.92653415 2.74976504], Cost: 1.0000
Current parameters: [3.9264753  2.74888442], Cost: 1.0000
Current parameters: [3.92617389 2.74850026], Cost: 1.0000
Current parameters: [3.92671699 2.74884994], Cost: 1.0000
Current parameters: [3.92677177 2.74861202], Cost: 1.0000
Current parameters: [3.92685452 2.74905166], Cost: 1.0000
Current parameters: [3.92677332 2.74893257], Cost: 1.0000
Current parameters: [3.92687133 2.7489127 ], Cost: 1.0000
Current parameters: [3.92696937 2.74893242], Cost: 1.0000
Current parameters: [3.92700092 2.74883752], Cost: 1.0000
Current parameters: [3.9270338  2.74900889], Cost: 1.0000
Current parameters: [3.9270338  2.74900889], Cost: 1.0000
```

```
   Normal return from subroutine COBYLA

   NFVALS =   39   F = 1.000000E+00    MAXCV = 0.000000E+00
   X = 3.927034E+00   2.749009E+00
Simulating for QAOA depth p = 2
Current parameters: [1.20748619 2.43805711 1.03120109 0.54976545], Cost: 2.5607
Current parameters: [0.20748619 3.43805711 1.03120109 0.54976545], Cost: 2.4140
Current parameters: [0.20748619 2.43805711 2.03120109 0.54976545], Cost: 1.1781
Current parameters: [0.20748619 2.43805711 2.03120109 1.54976545], Cost: 2.4693
Current parameters: [-0.39910386  1.89769639  2.04874362 -0.03311625], Cost: 1.4223
Current parameters: [0.62677132 2.25291276 2.0372117  0.3500521 ], Cost: 1.6683
Current parameters: [0.09768799 2.87401833 2.03778376 0.33104454], Cost: 1.5130
Current parameters: [0.02249634 2.29483413 2.0338521  0.46168078], Cost: 1.0654
Current parameters: [-0.11916481  2.22324359  2.04056331  0.65471413], Cost: 1.4192
Current parameters: [0.02664618 2.29300168 2.15875412 0.45970414], Cost: 1.0644
Current parameters: [0.14693839 2.15299622 2.15085373 0.29128253], Cost: 1.3980
Current parameters: [-0.09096566  2.4758878   2.16381025  0.33643793], Cost: 1.0155
Current parameters: [-0.18695988  2.48855234  2.16901645  0.41532149], Cost: 1.0150
Current parameters: [-0.2192228   2.44595634  2.16895041  0.38290343], Cost: 0.9289
Current parameters: [-0.29002037  2.37445036  2.16973495  0.3087483 ], Cost: 0.8110
Current parameters: [-0.38249558  2.31167233  2.17194034  0.25282522], Cost: 0.7380
Current parameters: [-0.49408551  2.30524128  2.17719648  0.30853611], Cost: 0.5708
Current parameters: [-0.6010177   2.29753432  2.18232964  0.3726045 ], Cost: 0.4579
Current parameters: [-0.70810682  2.23347316  2.18584771  0.37898516], Cost: 0.2927
Current parameters: [-0.79782215  2.14648389  2.18825181  0.38078674], Cost: 0.1470
Current parameters: [-0.90324974  2.08304715  2.19191989  0.40252034], Cost: 0.0760
Current parameters: [-0.92796947  2.04120024  2.19058749  0.28736394], Cost: 0.2490
Current parameters: [-0.90077812  2.08222314  2.25435828  0.40156678], Cost: 0.2193
Current parameters: [-0.9613295   2.14994024  2.11450297  0.44475395], Cost: 0.0498
Current parameters: [-0.94154735  2.10211624  2.09073141  0.4704968 ], Cost: 0.0286
Current parameters: [-0.93611309  2.1402094   1.99334769  0.53876678], Cost: 0.2078
Current parameters: [-0.95270072  2.08192853  2.06114284  0.42050855], Cost: 0.0910
Current parameters: [-0.97043015  2.09418174  2.09217929  0.47928846], Cost: 0.0239
Current parameters: [-0.96060596  2.06632915  2.14544683  0.49330766], Cost: 0.0239
Current parameters: [-0.9678539   2.1022446   2.08804749  0.50908517], Cost: 0.0321
Current parameters: [-0.97416647  2.10696623  2.1000899   0.47724901], Cost: 0.0239
Current parameters: [-0.98966303  2.08302478  2.09548695  0.45758038], Cost: 0.0269
Current parameters: [-0.98004659  2.08860326  2.09383312  0.46843442], Cost: 0.0239
Current parameters: [-0.97210634  2.09762127  2.08549338  0.47798703], Cost: 0.0302
Current parameters: [-0.96944613  2.08714697  2.10524235  0.48408854], Cost: 0.0155
```



```
Current parameters: [-0.96979912  2.08015698  2.11788937  0.49002226], Cost: 0.0133
Current parameters: [-0.97155748  2.07618242  2.11190474  0.50378583], Cost: 0.0169
Current parameters: [-0.96270364  2.08269614  2.11858048  0.49196249], Cost: 0.0147
Current parameters: [-0.96771128  2.06764629  2.11940212  0.48102361], Cost: 0.0087
Current parameters: [-0.97112046  2.0597907   2.1272193   0.47054985], Cost: 0.0074
Current parameters: [-0.97228271  2.04545541  2.12248239  0.47440354], Cost: 0.0065
Current parameters: [-0.97171212  2.04504637  2.11113524  0.46368486], Cost: 0.0092
Current parameters: [-0.96467992  2.0442671   2.12360899  0.47366095], Cost: 0.0053
Current parameters: [-0.95805717  2.0370325   2.13506338  0.47775205], Cost: 0.0073
Current parameters: [-0.96204926  2.0463733   2.11990102  0.47965509], Cost: 0.0059
Current parameters: [-0.96490332  2.04091884  2.12160985  0.47369883], Cost: 0.0053
Current parameters: [-0.96113927  2.04585301  2.12067076  0.46754943], Cost: 0.0048
Current parameters: [-0.95574146  2.04342802  2.12450863  0.46418954], Cost: 0.0038
Current parameters: [-0.9510806   2.04137201  2.12793122  0.45935525], Cost: 0.0033
Current parameters: [-0.95039829  2.04328539  2.12495151  0.45242489], Cost: 0.0041
Current parameters: [-0.95076577  2.04478572  2.12979582  0.45952704], Cost: 0.0035
Current parameters: [-0.94446564  2.04053783  2.12574973  0.46279368], Cost: 0.0024
Current parameters: [-0.94036932  2.03536414  2.1282612   0.46613745], Cost: 0.0021
Current parameters: [-0.9346542   2.0338502   2.12365202  0.46393836], Cost: 0.0016
Current parameters: [-0.93299288  2.03308968  2.11778744  0.46876572], Cost: 0.0026
Current parameters: [-0.9325629   2.03675544  2.12476864  0.46503288], Cost: 0.0015
Current parameters: [-0.92875417  2.0355782   2.12794145  0.45911037], Cost: 0.0010
Current parameters: [-0.92219494  2.03372249  2.12751726  0.4553172 ], Cost: 0.0006
Current parameters: [-0.91926852  2.02972421  2.13347896  0.45628827], Cost: 0.0007
Current parameters: [-0.92038871  2.03614002  2.12821938  0.44814537], Cost: 0.0011
Current parameters: [-0.91972066  2.03583111  2.12721655  0.45746197], Cost: 0.0006
Current parameters: [-0.92123669  2.0311527   2.12487105  0.4561741 ], Cost: 0.0006
Current parameters: [-0.92163841  2.03268742  2.12898897  0.45583458], Cost: 0.0005
Current parameters: [-0.92043723  2.03211059  2.13007619  0.4549088 ], Cost: 0.0005
Current parameters: [-0.9204101   2.03126099  2.13171474  0.45554701], Cost: 0.0006
Current parameters: [-0.92081281  2.0327893   2.13056518  0.45457286], Cost: 0.0006
Current parameters: [-0.91991168  2.03090225  2.12886146  0.45413236], Cost: 0.0004
Current parameters: [-0.91822033  2.03118571  2.12811193  0.45469082], Cost: 0.0004
Current parameters: [-0.91740014  2.02965296  2.1279869   0.45557232], Cost: 0.0003
Current parameters: [-0.91598198  2.02852082  2.12726461  0.45558016], Cost: 0.0002
Current parameters: [-0.91485065  2.02849217  2.12884302  0.45578651], Cost: 0.0002
Current parameters: [-0.91388707  2.02829879  2.12927221  0.45741888], Cost: 0.0002
Current parameters: [-0.91532312  2.02770279  2.12915378  0.45589018], Cost: 0.0002
Current parameters: [-0.91394081  2.02812711  2.12958333  0.45426811], Cost: 0.0002
Current parameters: [-0.91458724  2.02910547  2.1310704   0.45379034], Cost: 0.0002
Current parameters: [-0.91335636  2.02721522  2.12821371  0.45339305], Cost: 0.0002
```



```
Current parameters: [-0.91257819  2.02691824  2.12843336  0.45385315], Cost: 0.0001
Current parameters: [-0.91173385  2.02665426  2.12881402  0.45401495], Cost: 0.0001
Current parameters: [-0.91105652  2.02621642  2.12844331  0.4544221 ], Cost: 0.0001
Current parameters: [-0.91034489  2.02606815  2.12903535  0.45414868], Cost: 0.0001
Current parameters: [-0.90966384  2.02576718  2.12924736  0.45355344], Cost: 0.0000
Current parameters: [-0.90886185  2.02549934  2.12944653  0.45399963], Cost: 0.0000
Current parameters: [-0.90838006  2.02633483  2.12937446  0.4538643 ], Cost: 0.0000
Current parameters: [-0.90895397  2.02559515  2.12991618  0.45401306], Cost: 0.0000
Current parameters: [-0.90830503  2.02479902  2.12920764  0.45368962], Cost: 0.0000
Current parameters: [-0.90787941  2.02416812  2.12874376  0.45408875], Cost: 0.0000
Current parameters: [-0.90859593  2.02461068  2.12890499  0.45282824], Cost: 0.0000
Current parameters: [-0.90792273  2.02507959  2.129169    0.45357985], Cost: 0.0000
Current parameters: [-0.90783636  2.0249607   2.12935192  0.45351241], Cost: 0.0000
Current parameters: [-0.90771525  2.02501496  2.12981673  0.45358142], Cost: 0.0000
Current parameters: [-0.90783266  2.02510845  2.12986412  0.45339478], Cost: 0.0000
Current parameters: [-0.90746911  2.02469747  2.12983628  0.45330455], Cost: 0.0000
Current parameters: [-0.90767986  2.02427272  2.12994895  0.45327463], Cost: 0.0000
Current parameters: [-0.9073175   2.02409337  2.12992478  0.45300193], Cost: 0.0000
Current parameters: [-0.90688877  2.02388     2.12998861  0.45307271], Cost: 0.0000
Current parameters: [-0.90676567  2.0236994   2.12955402  0.45303052], Cost: 0.0000
Current parameters: [-0.90687617  2.02348981  2.12961119  0.45301646], Cost: 0.0000
Current parameters: [-0.90642706  2.02367309  2.12964888  0.45269278], Cost: 0.0000
Current parameters: [-0.90596298  2.02362314  2.12974169  0.45258351], Cost: 0.0000
Current parameters: [-0.90554923  2.0236224   2.1298044   0.45233193], Cost: 0.0000
Current parameters: [-0.90596298  2.02362314  2.12974169  0.45258351], Cost: 0.0000

   Return from subroutine COBYLA because the MAXFUN limit has been reached.

   NFVALS =  100   F = 3.588586E-06    MAXCV = 0.000000E+00
   X =-9.059630E-01   2.023623E+00   2.129742E+00   4.525835E-01
Simulating for QAOA depth p = 3
Current parameters: [1.53647108 2.69353072 0.11087849 1.17817242 2.4639261  0.55392196], Cost: 2.2738
Current parameters: [0.53647108 3.69353072 0.11087849 1.17817242 2.4639261  0.55392196], Cost: 1.1799
Current parameters: [0.53647108 3.69353072 1.11087849 1.17817242 2.4639261  0.55392196], Cost: 3.6453
Current parameters: [0.53647108 3.69353072 0.11087849 2.17817242 2.4639261  0.55392196], Cost: 2.2824
Current parameters: [0.53647108 3.69353072 0.11087849 1.17817242 3.4639261  0.55392196], Cost: 1.6934
Current parameters: [0.53647108 3.69353072 0.11087849 1.17817242 2.4639261  1.55392196], Cost: 1.6532
Current parameters: [ 0.29054576  3.82424028 -0.73796105  0.79858322  2.28710501  0.39096941], Cost: 1.1729
Current parameters: [ 0.09263732  3.92942882 -0.47526746  0.49310876  2.1448082   0.25983332], Cost: 1.4028
Current parameters: [ 0.50036042  3.82424028 -0.73796105  0.66265012  2.28710501  0.39096941], Cost: 1.7348
Current parameters: [ 0.02246545  3.89804453 -0.78634806  1.18834026  2.1872642   0.29895938], Cost: 1.4071
```



```
Current parameters: [ 0.29054576  3.82424028 -0.73796105  0.79858322  2.45652513  0.20713029], Cost: 1.0486
Current parameters: [ 0.04989534  3.85216624 -0.75626967  0.73117948  2.79768957  0.47177322], Cost: 1.6919
Current parameters: [ 0.23544391  3.58494361 -0.7542443   0.77630924  2.42120973  0.22100255], Cost: 1.1816
Current parameters: [ 0.32578411  3.84417439 -0.61545747  0.87624244  2.56701546 -0.2566743 ], Cost: 2.3562
Current parameters: [ 0.19434883  3.86737058 -0.8031008   0.97514216  2.33819846  0.25149787], Cost: 1.2160
Current parameters: [ 0.30719279  3.80773618 -0.63814249  0.8527882   2.47835261  0.2483265 ], Cost: 0.7975
Current parameters: [ 0.19901151  3.83473743 -0.6147458   0.84215644  2.65093088  0.38841235], Cost: 0.8966
Current parameters: [ 0.37882902  3.78286383 -0.69989954  0.90870459  2.5317937   0.25716332], Cost: 0.6657
Current parameters: [ 0.44122603  3.76292382 -0.57994511  1.03172608  2.63563166  0.12335996], Cost: 0.5889
Current parameters: [ 0.53855349  3.76192046 -0.53621735  1.04209424  2.620631    0.18586375], Cost: 0.3567
Current parameters: [ 0.55459125  3.77479663 -0.52795619  1.14309783  2.57400135  0.23837752], Cost: 0.2021
Current parameters: [ 0.57915958  3.66489379 -0.50438183  1.16540288  2.55641632  0.27813006], Cost: 0.2412
Current parameters: [ 0.53093501  3.77956683 -0.51334138  1.1456012   2.61618329  0.27477398], Cost: 0.1342
Current parameters: [ 0.59436107  3.84134494 -0.49419597  1.17992954  2.65277538  0.34478568], Cost: 0.2256
Current parameters: [ 0.53858986  3.76754943 -0.56079185  1.16824507  2.645264    0.26511707], Cost: 0.1673
Current parameters: [ 0.45356965  3.78681202 -0.45891952  1.21988408  2.62525185  0.2427555 ], Cost: 0.2790
Current parameters: [ 0.46919557  3.7908066  -0.56960517  1.12436904  2.56592074  0.34923315], Cost: 0.1574
Current parameters: [ 0.5415465   3.72528587 -0.49835919  1.1471975   2.61377588  0.29956134], Cost: 0.1382
Current parameters: [ 0.52222162  3.77614602 -0.5078493   1.09175304  2.64576937  0.27109967], Cost: 0.1389
Current parameters: [ 0.51535764  3.78194904 -0.52721344  1.13913787  2.60421678  0.29350437], Cost: 0.1287
Current parameters: [ 0.50314995  3.77673157 -0.52537094  1.14184564  2.60366799  0.28596361], Cost: 0.1459
Current parameters: [ 0.52189569  3.79759695 -0.51630007  1.14221901  2.61148667  0.31603241], Cost: 0.1184
Current parameters: [ 0.54246642  3.80589338 -0.51782389  1.13013693  2.59415166  0.32201366], Cost: 0.0857
Current parameters: [ 0.56603017  3.8131225  -0.53094066  1.12234048  2.58728363  0.33145019], Cost: 0.0643
Current parameters: [ 0.57970317  3.82336494 -0.52332378  1.11059076  2.56608737  0.3377231 ], Cost: 0.0473
Current parameters: [ 0.59672341  3.83882481 -0.5229279   1.10785668  2.56500602  0.35867698], Cost: 0.0372
Current parameters: [ 0.59415071  3.84006377 -0.54279319  1.09733155  2.54641227  0.36950625], Cost: 0.0458
Current parameters: [ 0.59842888  3.83991091 -0.52443554  1.12168349  2.55821613  0.35797257], Cost: 0.0370
Current parameters: [ 0.61686064  3.85669561 -0.52557717  1.11759539  2.55876274  0.33962068], Cost: 0.0252
Current parameters: [ 0.64275216  3.84144588 -0.51785584  1.11530094  2.5559216   0.33879457], Cost: 0.0157
Current parameters: [ 0.65619912  3.85360523 -0.49387753  1.11204702  2.5482974   0.33674122], Cost: 0.0086
Current parameters: [ 0.68151196  3.86207401 -0.4916835   1.11677745  2.56367111  0.33599238], Cost: 0.0039
Current parameters: [ 0.69308598  3.85617506 -0.48325293  1.11508167  2.5615563   0.30898526], Cost: 0.0105
Current parameters: [ 0.68315377  3.86318857 -0.49366569  1.13033601  2.55644323  0.33554842], Cost: 0.0074
Current parameters: [ 0.69627294  3.87021794 -0.50224165  1.09929831  2.55019185  0.34567054], Cost: 0.0023
Current parameters: [ 0.70121385  3.85740878 -0.49839078  1.09973957  2.54872957  0.35187468], Cost: 0.0021
Current parameters: [ 0.70271147  3.86945209 -0.48182852  1.09139152  2.56068407  0.37037791], Cost: 0.0107
Current parameters: [ 0.69993587  3.85344089 -0.50626798  1.09818476  2.56144255  0.35269657], Cost: 0.0022
Current parameters: [ 0.71036194  3.85411396 -0.52349216  1.10047623  2.53287764  0.35153786], Cost: 0.0113
Current parameters: [ 0.70587965  3.85417766 -0.49367534  1.09344079  2.55107391  0.33985283], Cost: 0.0048
Current parameters: [ 0.69627316  3.85577296 -0.49887281  1.09434775  2.54671478  0.35263985], Cost: 0.0045
```

```
Current parameters: [ 0.69461406  3.85424078 -0.4952511   1.11262641  2.55074345  0.34862457], Cost: 0.0010
Current parameters: [ 0.69825514  3.8544536  -0.49993623  1.11730905  2.54878075  0.34867602], Cost: 0.0014
Current parameters: [ 0.69378401  3.8556211  -0.49517406  1.11335698  2.55100342  0.35209693], Cost: 0.0012
Current parameters: [ 0.6985116   3.85588078 -0.4905759   1.11442246  2.55477909  0.34728851], Cost: 0.0009
Current parameters: [ 0.69966421  3.85265312 -0.4896217   1.11444329  2.55438031  0.34885138], Cost: 0.0008
Current parameters: [ 0.69607438  3.84870334 -0.4853598   1.11593369  2.55552338  0.34555656], Cost: 0.0014
Current parameters: [ 0.70006009  3.85368099 -0.4876907   1.11468191  2.55118159  0.34868386], Cost: 0.0006
Current parameters: [ 0.70434795  3.8546357  -0.48663133  1.10849137  2.549683    0.34890084], Cost: 0.0003
Current parameters: [ 0.71045109  3.85481123 -0.48899976  1.11121239  2.54653616  0.34798428], Cost: 0.0003
Current parameters: [ 0.71097062  3.85387958 -0.48616297  1.11016919  2.54488381  0.34648261], Cost: 0.0007
Current parameters: [ 0.71119185  3.85549594 -0.48787985  1.11179379  2.54754459  0.34841785], Cost: 0.0001
Current parameters: [ 0.71140753  3.8569821  -0.4878396   1.11185103  2.5470236   0.35198538], Cost: 0.0000
Current parameters: [ 0.71096656  3.85866308 -0.48786985  1.1118914   2.5465368   0.35124038], Cost: 0.0000
Current parameters: [ 0.71285015  3.85826072 -0.48902967  1.11121117  2.55011497  0.35238607], Cost: 0.0001
Current parameters: [ 0.71293173  3.85702552 -0.48843246  1.11253465  2.54623735  0.35175604], Cost: 0.0001
Current parameters: [ 0.71018365  3.85695263 -0.48767956  1.11541263  2.54789927  0.35251701], Cost: 0.0002
Current parameters: [ 0.71203806  3.85712251 -0.48607852  1.11137929  2.54683814  0.35218305], Cost: 0.0000
Current parameters: [ 0.71214508  3.85706774 -0.48637637  1.11051421  2.54651882  0.35217   ], Cost: 0.0000
Current parameters: [ 0.71122054  3.85695512 -0.48530599  1.11250837  2.54649603  0.35324592], Cost: 0.0000
Current parameters: [ 0.71230706  3.85760358 -0.48626856  1.11128378  2.54725497  0.35283947], Cost: 0.0000
Current parameters: [ 0.71196409  3.85667259 -0.48530142  1.11199938  2.54803446  0.35109345], Cost: 0.0000
Current parameters: [ 0.71335409  3.85649431 -0.48535717  1.11311932  2.54736789  0.35070731], Cost: 0.0000
Current parameters: [ 0.71174227  3.85747092 -0.48519162  1.11209886  2.54775191  0.3506868 ], Cost: 0.0000
Current parameters: [ 0.71087778  3.85656466 -0.48474006  1.11254886  2.5494357   0.35088738], Cost: 0.0000
Current parameters: [ 0.71182099  3.85629158 -0.48503581  1.11143543  2.54751304  0.3507363 ], Cost: 0.0000
Current parameters: [ 0.71221121  3.85679893 -0.48496914  1.11183592  2.54817624  0.3511579 ], Cost: 0.0000
Current parameters: [ 0.71226369  3.85677375 -0.48585156  1.11160688  2.54843757  0.35060923], Cost: 0.0000
Current parameters: [ 0.71238785  3.85691788 -0.48604369  1.11142465  2.54820569  0.35088912], Cost: 0.0000
Current parameters: [ 0.7117293   3.85701736 -0.48609185  1.11168782  2.54909142  0.3509512 ], Cost: 0.0000
Current parameters: [ 0.71289412  3.8567308  -0.48603036  1.11229697  2.54830528  0.35043983], Cost: 0.0000
Current parameters: [ 0.71215378  3.85655343 -0.485777    1.11134453  2.54821217  0.35037987], Cost: 0.0000
Current parameters: [ 0.71234047  3.85658879 -0.48584867  1.11156771  2.54851981  0.35071503], Cost: 0.0000
Current parameters: [ 0.71256248  3.85678078 -0.48551451  1.1114137   2.54864899  0.35072911], Cost: 0.0000
Current parameters: [ 0.71266571  3.85656456 -0.48592757  1.11190618  2.54845634  0.35063   ], Cost: 0.0000
Current parameters: [ 0.7122363   3.85665175 -0.48591531  1.11162071  2.54869287  0.35080216], Cost: 0.0000
Current parameters: [ 0.71238834  3.85661752 -0.48593217  1.11150259  2.54853517  0.35069675], Cost: 0.0000
Current parameters: [ 0.7124043   3.85667426 -0.48595595  1.11148162  2.54839855  0.3508877 ], Cost: 0.0000
Current parameters: [ 0.71229793  3.85649831 -0.48592914  1.11136373  2.54843259  0.3506107 ], Cost: 0.0000
Current parameters: [ 0.71242947  3.85665423 -0.48586294  1.11146335  2.54856012  0.35069909], Cost: 0.0000
Current parameters: [ 0.71242303  3.85660768 -0.48592887  1.111518    2.54850912  0.35068023], Cost: 0.0000
Current parameters: [ 0.71240676  3.8566427  -0.485959    1.11156308  2.54860075  0.35071535], Cost: 0.0000
```



```
Current parameters: [ 0.7124025   3.85657802 -0.48593146  1.1114925   2.5485502   0.35071703], Cost: 0.0000
Current parameters: [ 0.71232702  3.85658721 -0.48594213  1.11144879  2.54853909  0.35064867], Cost: 0.0000
Current parameters: [ 0.71237709  3.85663931 -0.48594834  1.11148038  2.54848323  0.35077386], Cost: 0.0000
Current parameters: [ 0.71237709  3.85663931 -0.48594834  1.11148038  2.54848323  0.35077386], Cost: 0.0000

   Normal return from subroutine COBYLA

   NFVALS =   96   F = 4.968996E-08    MAXCV = 0.000000E+00
   X = 7.123771E-01   3.856639E+00  -4.859483E-01   1.111480E+00   2.548483E+00
       3.507739E-01
Simulating for QAOA depth p = 4
Current parameters: [1.1042017  1.50595807 0.16257835 0.38780238 1.37665457 1.95783789
 1.05099752 1.48041978], Cost: 1.7471
Current parameters: [1.1042017  2.50595807 0.16257835 0.38780238 1.37665457 1.95783789
 1.05099752 1.48041978], Cost: 1.2425
Current parameters: [1.1042017  2.50595807 1.16257835 0.38780238 1.37665457 1.95783789
 1.05099752 1.48041978], Cost: 2.4409
Current parameters: [1.1042017  2.50595807 0.16257835 1.38780238 1.37665457 1.95783789
 1.05099752 1.48041978], Cost: 1.1161
Current parameters: [1.1042017  2.50595807 0.16257835 1.38780238 2.37665457 1.95783789
 1.05099752 1.48041978], Cost: 2.3510
Current parameters: [1.1042017  2.50595807 0.16257835 1.38780238 1.37665457 2.95783789
 1.05099752 1.48041978], Cost: 2.4209
Current parameters: [1.1042017  2.50595807 0.16257835 1.38780238 1.37665457 1.95783789
 2.05099752 1.48041978], Cost: 2.8973
Current parameters: [1.1042017  2.50595807 0.16257835 1.38780238 1.37665457 1.95783789
 1.05099752 2.48041978], Cost: 0.3705
Current parameters: [ 1.38054469  2.67073762 -0.22872723  1.42907688  0.97342346  1.53178124
  0.46938949  2.72387636], Cost: 2.2110
Current parameters: [0.61161934 2.52066999 0.12764163 1.39148747 1.34065311 1.91979851
 0.99907013 2.50215618], Cost: 1.0430
Current parameters: [1.1042017  2.27555329 0.06555444 1.38780238 1.37665457 1.95783789
 1.05099752 2.48041978], Cost: 0.9149
Current parameters: [1.14913437 2.4142761  0.63691101 1.3935836  1.32017491 1.89816111
 0.96953301 2.51452019], Cost: 0.4063
Current parameters: [1.1042017  2.50595807 0.16257835 1.6365029  1.40211141 1.95783789
 1.05099752 2.48041978], Cost: 0.6037
Current parameters: [1.15238637 2.60441915 0.20394662 1.29440734 1.83977993 1.893842
 0.96363701 2.51698821], Cost: 2.5524
Current parameters: [1.1042017  2.50595807 0.16257835 1.38780238 1.37665457 2.1595145
 0.90325943 2.48041978], Cost: 0.5543
```



```
Current parameters: [1.17052553 2.6414849  0.21951973 1.3534652  1.09367618 2.13713538
 1.3797137  2.53075442], Cost: 1.7736
Current parameters: [1.16538988 2.5644977  0.11059882 1.38677523 1.24458559 1.83820999
 0.90698582 2.49198565], Cost: 0.7414
Current parameters: [1.10068223 2.51189022 0.18018451 1.38357478 1.36621024 1.95405498
 1.05285844 2.23142638], Cost: 0.5850
Current parameters: [1.11999871 2.5386958  0.17686044 1.37941285 1.30836633 2.0037823
 1.13411373 2.4990439 ], Cost: 0.4844
Current parameters: [1.10590195 2.52894452 0.10799193 1.38622572 1.39205772 1.96482112
 1.0605303  2.47642944], Cost: 0.5423
Current parameters: [1.1268712  2.52050694 0.25616995 1.35966132 1.44236202 1.9369215
 1.05084762 2.50477405], Cost: 0.4024
Current parameters: [0.98467418 2.51821886 0.18685792 1.38019603 1.3637856  1.94462341
 1.04479381 2.49313872], Cost: 0.3577
Current parameters: [0.98041993 2.4578377  0.19142184 1.37136261 1.35916432 1.9401399
 1.0510165  2.50109034], Cost: 0.4103
Current parameters: [0.98858208 2.52195761 0.18353815 1.35430072 1.35270684 1.93757051
 1.03516593 2.4938774 ], Cost: 0.3290
Current parameters: [0.99907809 2.52988284 0.19701663 1.37842869 1.32036732 1.90714486
 1.0052862  2.50412844], Cost: 0.2343
Current parameters: [0.99939718 2.53101147 0.21645305 1.37811371 1.30650645 1.89664236
 0.99929225 2.44766033], Cost: 0.4029
Current parameters: [0.99670706 2.52496912 0.19487298 1.3796879  1.33061692 1.91900276
 0.9790729  2.50133308], Cost: 0.2727
Current parameters: [1.00634619 2.54127153 0.21614318 1.37633179 1.28455321 1.94229322
 1.00943457 2.53273323], Cost: 0.1258
Current parameters: [1.01937236 2.55787884 0.25202707 1.36481575 1.30011848 1.92609229
 1.00954906 2.57192164], Cost: 0.0435
Current parameters: [1.02407773 2.57862396 0.23087594 1.36720199 1.27648455 1.91571432
 1.01130247 2.62020566], Cost: 0.0164
Current parameters: [1.02553434 2.52450319 0.24273957 1.36078479 1.26159656 1.90721227
 1.0149488  2.64224907], Cost: 0.0537
Current parameters: [0.99411049 2.58162479 0.23707175 1.36505666 1.273401   1.91257108
 1.00996638 2.62252045], Cost: 0.0252
Current parameters: [1.04440175 2.60962209 0.26867964 1.36161668 1.24450076 1.91137099
 1.01486156 2.61602345], Cost: 0.0555
Current parameters: [1.02342014 2.5774085  0.23763871 1.39751162 1.27763301 1.91372003
 1.01180868 2.62237162], Cost: 0.0056
Current parameters: [1.022567   2.58628427 0.24223475 1.40020424 1.30217694 1.9480207
 1.05042852 2.64534848], Cost: 0.0994
Current parameters: [1.02358973 2.5766285  0.24165896 1.39796456 1.27554625 1.93489213
```



```
 0.99021333 2.62874237], Cost: 0.0068
Current parameters: [1.03152018 2.58757659 0.23216555 1.40202998 1.30691235 1.8764613
 0.975162   2.6322916 ], Cost: 0.0434
Current parameters: [1.02176512 2.57701681 0.21623346 1.40712152 1.2613942  1.91491928
 1.0077791  2.6104653 ], Cost: 0.0214
Current parameters: [1.02148132 2.56352485 0.23355127 1.39732294 1.27957721 1.91281958
 1.01197127 2.62749663], Cost: 0.0078
Current parameters: [1.03237299 2.57074436 0.25621948 1.39622931 1.25709412 1.90518147
 1.01049388 2.62530887], Cost: 0.0309
Current parameters: [1.02208473 2.56723964 0.2472668  1.3978835  1.28850292 1.91731872
 1.0091806  2.59706306], Cost: 0.0147
Current parameters: [1.02520791 2.58078379 0.23547454 1.39881513 1.28513456 1.90479041
 1.0027314  2.62455357], Cost: 0.0085
Current parameters: [1.03069993 2.57688089 0.23621284 1.39800276 1.27893179 1.91526205
 1.01300013 2.62231762], Cost: 0.0037
Current parameters: [1.02943365 2.5818465  0.23562111 1.40185462 1.2872506  1.92180809
 1.02068352 2.62793949], Cost: 0.0042
Current parameters: [1.03050099 2.57808811 0.23004455 1.40113579 1.2766225  1.91652744
 1.01291437 2.62014203], Cost: 0.0042
Current parameters: [1.03065991 2.57681565 0.23585797 1.39793305 1.27935155 1.91269015
 1.01563776 2.62114537], Cost: 0.0038
Current parameters: [1.03048808 2.57500507 0.23782737 1.39899899 1.2824925  1.9175373
 1.01218849 2.61637135], Cost: 0.0042
Current parameters: [1.0310442  2.57638116 0.23783318 1.40132024 1.27809311 1.91463189
 1.01299481 2.62271616], Cost: 0.0034
Current parameters: [1.03657554 2.5771691  0.2408052  1.40078172 1.27453572 1.91617315
 1.0153679  2.62253246], Cost: 0.0035
Current parameters: [1.03040017 2.57315457 0.23734729 1.40109158 1.27737315 1.91576698
 1.01436067 2.6234035 ], Cost: 0.0036
Current parameters: [1.03566869 2.57449117 0.23677129 1.40272097 1.28119115 1.91149878
 1.01045662 2.62538654], Cost: 0.0029
Current parameters: [1.03589386 2.57704681 0.23685938 1.40505987 1.28546434 1.91420772
 1.01398441 2.62869277], Cost: 0.0006
Current parameters: [1.03595062 2.57985062 0.23816362 1.40810121 1.28953382 1.91522263
 1.01643504 2.63300826], Cost: 0.0013
Current parameters: [1.03592224 2.57844872 0.2375115  1.40658054 1.28749908 1.91471517
 1.01520973 2.63085052], Cost: 0.0006
Current parameters: [1.03575958 2.57795772 0.23891715 1.40576118 1.2879006  1.91406616
 1.01486336 2.63129497], Cost: 0.0006
Current parameters: [1.03807637 2.57692129 0.23799484 1.40653214 1.2888148  1.91229772
 1.01324474 2.63218322], Cost: 0.0004
```

```
Current parameters: [1.0382865  2.57812993 0.23900646 1.40817402 1.29073092 1.91194595
 1.01379428 2.63461989], Cost: 0.0006
Current parameters: [1.03771305 2.57741871 0.2376011  1.40630542 1.28781156 1.91169086
 1.01336335 2.63353969], Cost: 0.0004
Current parameters: [1.03846042 2.57791668 0.23920796 1.40830764 1.29123333 1.91222476
 1.01374777 2.63402408], Cost: 0.0006
Current parameters: [1.03658517 2.57669619 0.23747393 1.40700332 1.289513   1.91187273
 1.01262929 2.63217396], Cost: 0.0004
Current parameters: [1.03888055 2.57798474 0.23936006 1.40818427 1.29104948 1.9123436
 1.01392287 2.63403578], Cost: 0.0006
Current parameters: [1.03808548 2.57695719 0.23817902 1.40656395 1.28859216 1.91358653
 1.01192681 2.63275844], Cost: 0.0004
Current parameters: [1.03887898 2.57798019 0.23933991 1.40817969 1.29107081 1.91220999
 1.01405894 2.63397472], Cost: 0.0006
Current parameters: [1.03799266 2.57675501 0.23893254 1.40806582 1.2881171  1.9120569
 1.01326456 2.6321248 ], Cost: 0.0002
Current parameters: [1.03875193 2.57774488 0.24043409 1.4099869  1.29015304 1.91192078
 1.01405707 2.63384688], Cost: 0.0004
Current parameters: [1.03846569 2.57819244 0.23841532 1.40845489 1.28805713 1.91163852
 1.01255906 2.63146945], Cost: 0.0001
Current parameters: [1.03980742 2.57694471 0.23568738 1.4104306  1.28765479 1.91213575
 1.01289717 2.63165159], Cost: 0.0002
Current parameters: [1.03730723 2.57836101 0.23848158 1.40928864 1.28726247 1.91183548
 1.01181622 2.63219237], Cost: 0.0002
Current parameters: [1.03948143 2.5776862  0.23959289 1.40882929 1.28657145 1.90957262
 1.01095722 2.62962477], Cost: 0.0004
Current parameters: [1.0379814  2.57901449 0.23838499 1.4086541  1.28761397 1.91249429
 1.01358395 2.63052888], Cost: 0.0001
Current parameters: [1.03805343 2.57937843 0.23850149 1.40898085 1.28819632 1.91265898
 1.013848   2.6310385 ], Cost: 0.0002
Current parameters: [1.0385141  2.57929299 0.23921733 1.40923841 1.28701447 1.91162991
 1.01315667 2.62946586], Cost: 0.0001
Current parameters: [1.03885151 2.57999483 0.23898376 1.40871736 1.2858864  1.91063154
 1.01373877 2.6299894 ], Cost: 0.0001
Current parameters: [1.03904814 2.57938486 0.23947274 1.40909021 1.28668374 1.91223203
 1.01291381 2.62967058], Cost: 0.0001
Current parameters: [1.03834201 2.57967362 0.23906407 1.40900244 1.28724891 1.91154015
 1.01189818 2.6280823 ], Cost: 0.0001
Current parameters: [1.0386104  2.57895874 0.2384611  1.4093845  1.28666271 1.91187335
 1.01325963 2.62925172], Cost: 0.0001
Current parameters: [1.03945297 2.57918694 0.23953399 1.41078066 1.28739167 1.91109867
```



```
 1.01312087 2.6295977 ], Cost: 0.0000
Current parameters: [1.03926439 2.5779289  0.24079209 1.41105213 1.28708933 1.91099381
 1.01368688 2.62925537], Cost: 0.0000
Current parameters: [1.04000639 2.57912653 0.23947639 1.41036487 1.28783313 1.91105562
 1.01353851 2.62928725], Cost: 0.0000
Current parameters: [1.03966999 2.57996028 0.23975309 1.41120875 1.28853208 1.91186222
 1.01457066 2.62940214], Cost: 0.0001
Current parameters: [1.03957505 2.57890082 0.23952254 1.41007842 1.2884326  1.91085294
 1.01350106 2.6297675 ], Cost: 0.0000
Current parameters: [1.04135978 2.57831792 0.23912178 1.41078372 1.28774461 1.91102487
 1.01277307 2.62994553], Cost: 0.0000
Current parameters: [1.04001444 2.57925325 0.23968147 1.4103692  1.28806175 1.91073661
 1.01292771 2.62868032], Cost: 0.0000
Current parameters: [1.04015125 2.57936125 0.23976702 1.41027924 1.2877527  1.91089336
 1.01354789 2.62948132], Cost: 0.0000
Current parameters: [1.04011698 2.57933803 0.23937674 1.41022433 1.28737691 1.91013706
 1.01382134 2.62938826], Cost: 0.0000
Current parameters: [1.04017957 2.57966841 0.23946541 1.41014575 1.2877957  1.91096452
 1.01340487 2.62956568], Cost: 0.0000
Current parameters: [1.04013571 2.57915409 0.23994951 1.40956474 1.28728313 1.91122805
 1.01340571 2.62936307], Cost: 0.0000
Current parameters: [1.04048842 2.57918483 0.23969582 1.41043258 1.2877715  1.91087186
 1.01336441 2.6296561 ], Cost: 0.0000
Current parameters: [1.04028438 2.57935643 0.23975611 1.41018642 1.28787068 1.91089251
 1.01366403 2.62940574], Cost: 0.0000
Current parameters: [1.04017051 2.57959909 0.2398511  1.41063749 1.28784516 1.91101166
 1.01370036 2.62946483], Cost: 0.0000
Current parameters: [1.04025273 2.57975337 0.24005763 1.41071824 1.28768597 1.91136495
 1.0137752  2.62941079], Cost: 0.0000
Current parameters: [1.04013368 2.57955178 0.2398079  1.41061327 1.28784144 1.91106824
 1.01383713 2.6296427 ], Cost: 0.0000
Current parameters: [1.04007428 2.57970605 0.24001778 1.41074894 1.2881703  1.91087631
 1.01349464 2.62935922], Cost: 0.0000
Current parameters: [1.04010952 2.57986251 0.23996877 1.41094844 1.28810096 1.9105439
 1.01350446 2.6291245 ], Cost: 0.0000
Current parameters: [1.03998688 2.579696   0.23973135 1.41111706 1.28821653 1.9107038
 1.01345306 2.62886127], Cost: 0.0000
Current parameters: [1.0401804  2.57968647 0.24008138 1.41104384 1.28808704 1.91050789
 1.01352096 2.62913117], Cost: 0.0000
Current parameters: [1.04042539 2.57993179 0.2398043  1.41107707 1.28816704 1.91055821
 1.01324362 2.62925733], Cost: 0.0000
```



```
Current parameters: [1.04024329 2.57986891 0.23996184 1.41087269 1.28822306 1.91054863
 1.01362754 2.6290483 ], Cost: 0.0000
Current parameters: [1.04025313 2.57981833 0.2400626  1.41061775 1.28823617 1.91038104
 1.013368   2.62879335], Cost: 0.0000
Current parameters: [1.04030855 2.57994489 0.24005073 1.4109053  1.28811641 1.91068867
 1.01361774 2.62895069], Cost: 0.0000
Current parameters: [1.04024329 2.57986891 0.23996184 1.41087269 1.28822306 1.91054863
 1.01362754 2.6290483 ], Cost: 0.0000

   Return from subroutine COBYLA because the MAXFUN limit has been reached.

   NFVALS =  100   F = 9.838887E-07    MAXCV = 0.000000E+00
   X = 1.040243E+00   2.579869E+00   2.399618E-01   1.410873E+00   1.288223E+00
       1.910549E+00   1.013628E+00   2.629048E+00
Simulating for QAOA depth p = 5
Current parameters: [2.858452   2.54987011 0.92681259 1.13920849 2.0841385  2.62101529
 0.1970313  3.10742796 0.44987217 2.10747152], Cost: 0.5817
Current parameters: [2.858452   3.54987011 0.92681259 1.13920849 2.0841385  2.62101529
 0.1970313  3.10742796 0.44987217 2.10747152], Cost: 0.8745
Current parameters: [2.858452   2.54987011 1.92681259 1.13920849 2.0841385  2.62101529
 0.1970313  3.10742796 0.44987217 2.10747152], Cost: 0.3046
Current parameters: [2.858452   2.54987011 1.92681259 2.13920849 2.0841385  2.62101529
 0.1970313  3.10742796 0.44987217 2.10747152], Cost: 1.6590
Current parameters: [2.858452   2.54987011 1.92681259 1.13920849 3.0841385  2.62101529
 0.1970313  3.10742796 0.44987217 2.10747152], Cost: 2.7479
Current parameters: [2.858452   2.54987011 1.92681259 1.13920849 2.0841385  3.62101529
 0.1970313  3.10742796 0.44987217 2.10747152], Cost: 0.9365
Current parameters: [2.858452   2.54987011 1.92681259 1.13920849 2.0841385  2.62101529
 1.1970313  3.10742796 0.44987217 2.10747152], Cost: 2.0797
Current parameters: [2.858452   2.54987011 1.92681259 1.13920849 2.0841385  2.62101529
 0.1970313  4.10742796 0.44987217 2.10747152], Cost: 2.8018
Current parameters: [2.858452   2.54987011 1.92681259 1.13920849 2.0841385  2.62101529
 0.1970313  3.10742796 1.44987217 2.10747152], Cost: 2.3217
Current parameters: [2.858452   2.54987011 1.92681259 1.13920849 2.0841385  2.62101529
 0.1970313  3.10742796 0.44987217 3.10747152], Cost: 1.4756
Current parameters: [ 3.0383339   2.4900528   1.98339815  0.86256534  1.58507916  2.49194542
 -0.16555736  2.59734107  0.03786976  1.86829245], Cost: 2.5853
Current parameters: [2.94839295 2.51996145 1.95510537 1.00088692 1.83460883 2.55648036
 0.01573697 2.85238451 0.24387097 1.98788198], Cost: 2.6212
Current parameters: [3.0680408  2.54987011 1.92681259 1.27548958 2.0841385  2.62101529
 0.1970313  3.10742796 0.44987217 2.10747152], Cost: 0.4912
```



```
Current parameters: [2.57851067 2.54457653 1.93182017 1.54498445 2.03997388 2.60959316
 0.16494376 3.06228745 0.41341172 2.08630519], Cost: 1.3020
Current parameters: [2.858452   2.30164679 1.92681259 1.13920849 2.11389057 2.62101529
 0.1970313  3.10742796 0.44987217 2.10747152], Cost: 0.1259
Current parameters: [2.85839591 2.33801851 1.93509419 1.09836262 2.59693578 2.60212521
 0.14396447 3.03277395 0.38957336 2.07246635], Cost: 0.8112
Current parameters: [2.83848291 2.33472935 1.93500478 1.10980681 2.11455179 2.86556861
 0.18883911 3.09923577 0.44167998 2.09927932], Cost: 0.0310
Current parameters: [3.03989901 1.97142948 1.86377174 1.3501116  2.1096952  2.97921991
 0.19826982 3.07887679 0.44113077 2.13363462], Cost: 0.3177
Current parameters: [2.82315022 2.36013084 1.6889239  1.08723158 2.11505949 2.86647432
 0.20968277 3.09294563 0.43538984 2.09298919], Cost: 0.2217
Current parameters: [2.83269965 2.32817611 2.00046115 1.07064666 2.11573083 2.87960095
 0.67606739 3.04075695 0.39399315 2.07060781], Cost: 1.8419
Current parameters: [2.84109052 2.33040935 1.93507231 1.11364616 2.11446545 2.86541458
 0.18840639 3.25327794 0.24488202 2.10034908], Cost: 0.4358
Current parameters: [2.83565525 2.30398875 1.95085227 1.0383577  2.11681372 2.89579558
 0.06527382 3.41782696 0.79172826 2.04037613], Cost: 2.0192
Current parameters: [2.8058427  2.27239055 1.92965294 1.06174868 2.07527457 2.76112593
 0.20350106 3.11838533 0.45784533 2.30401401], Cost: 0.3908
Current parameters: [2.90057815 2.54663386 1.95764931 1.16679322 2.2290179  3.18147672
 0.08860841 2.98935059 0.40617786 2.34666295], Cost: 1.1106
Current parameters: [2.86393023 2.2935529  1.95065253 1.02276544 2.01848987 2.83228858
 0.07476945 2.96092932 0.376334   2.02451457], Cost: 0.7996
Current parameters: [2.79485507 2.35415844 1.93483305 1.16204191 2.0151603  2.89019773
 0.19401853 3.10682897 0.44766805 2.10243917], Cost: 0.0294
Current parameters: [2.63913148 2.33738248 1.9384025  1.29129874 2.1234406  2.82039381
 0.15313823 3.06042632 0.43546613 2.07639767], Cost: 0.1394
Current parameters: [2.85298544 2.26620649 1.92418453 1.22167009 2.02771403 2.91399736
 0.19508873 3.09846906 0.454129   2.10458572], Cost: 0.0759
Current parameters: [2.74512113 2.37141368 1.84463418 1.24888995 1.82668372 2.88686283
 0.17245683 3.12721776 0.38664069 2.16497621], Cost: 0.2019
Current parameters: [2.80588372 2.40545622 1.94179782 1.18003087 2.04938868 2.96705651
 0.16620492 3.07552944 0.43831562 2.16227407], Cost: 0.1162
Current parameters: [2.79338616 2.35508551 1.93970106 1.16356061 2.01393051 2.88987257
 0.24230342 3.07636495 0.42294556 2.10465787], Cost: 0.0690
Current parameters: [2.76943548 2.36582378 1.9455628  1.16816865 2.01065387 2.92598224
 0.24566132 3.16142935 0.53055413 2.07292681], Cost: 0.4180
Current parameters: [2.76326261 2.32213401 1.93399724 1.13463827 2.00965594 2.92055446
 0.18632142 3.09992549 0.439604   2.09975903], Cost: 0.0945
Current parameters: [2.83765019 2.3693781  1.95632341 1.14495239 1.99013306 2.85280783
```

```
 0.13358094 3.03824106 0.4443988  2.05328643], Cost: 0.1253
Current parameters: [2.78916994 2.34269267 1.93495123 1.15153028 2.00962529 2.87015786
 0.1966515  3.0849329  0.483047   2.14067114], Cost: 0.1172
Current parameters: [2.81824303 2.34235644 1.97049184 1.14186987 2.04258534 2.8450384
 0.17692739 3.16683964 0.37522043 2.13886325], Cost: 0.0490
Current parameters: [2.8133489  2.3641967  1.82656919 1.13191068 2.05938944 2.87395078
 0.19323393 3.10779138 0.4323771  2.09255366], Cost: 0.0641
Current parameters: [2.78646551 2.36046993 1.9127135  1.18598249 1.97012662 2.88665937
 0.18478342 3.11508392 0.42791244 2.11465952], Cost: 0.0304
Current parameters: [2.75803234 2.35240719 1.93560272 1.19519364 2.04202609 2.87093336
 0.18132695 3.09760219 0.44135496 2.09415133], Cost: 0.0085
Current parameters: [2.75624648 2.36281433 1.93821432 1.20569212 2.04344349 2.87141348
 0.1878562  3.11781336 0.44810357 2.07836777], Cost: 0.0228
Current parameters: [2.75922857 2.34749091 1.93639432 1.19178929 2.03683812 2.86153398
 0.18047249 3.09547048 0.44155489 2.08489143], Cost: 0.0122
Current parameters: [2.76815035 2.36729905 1.94160332 1.19967899 2.04414985 2.87585967
 0.16340026 3.08518581 0.43432263 2.08762984], Cost: 0.0196
Current parameters: [2.76292701 2.34335935 1.94254622 1.20372733 2.04022364 2.87437544
 0.18119802 3.09738348 0.44272324 2.09465126], Cost: 0.0069
Current parameters: [2.77551103 2.33426818 1.91924792 1.20953093 2.05196837 2.87481893
 0.18012807 3.09827237 0.43980345 2.09141588], Cost: 0.0118
Current parameters: [2.76488559 2.34159254 1.94678194 1.20109578 2.04355277 2.87136509
 0.17978806 3.10486053 0.43128783 2.09652433], Cost: 0.0101
Current parameters: [2.76532618 2.34413336 1.94464381 1.20823446 2.04357535 2.85702241
 0.1760091  3.09797747 0.4564854  2.11505853], Cost: 0.0024
Current parameters: [2.76153637 2.33977564 1.9434954  1.20435056 2.04312308 2.86103493
 0.16469529 3.10358812 0.46071887 2.11423647], Cost: 0.0020
Current parameters: [2.74136385 2.32706909 1.94407334 1.20593055 2.04602058 2.86544365
 0.1695187  3.09128849 0.46213819 2.12841224], Cost: 0.0062
Current parameters: [2.76404232 2.33821881 1.94901137 1.19800154 2.05407108 2.86213347
 0.16714335 3.10162925 0.4657234  2.11127674], Cost: 0.0052
Current parameters: [2.7751733  2.33842509 1.94143941 1.19159693 2.02682456 2.87018935
 0.17103895 3.10607835 0.46678876 2.12798996], Cost: 0.0126
Current parameters: [2.75170743 2.34756871 1.94474563 1.22151937 2.04338577 2.86013372
 0.17138401 3.12158975 0.47224832 2.1097327 ], Cost: 0.0161
Current parameters: [2.76394514 2.34594263 1.94609703 1.2086733  2.04342701 2.86252134
 0.15490395 3.09515898 0.45856478 2.11523122], Cost: 0.0025
Current parameters: [2.76377469 2.33928831 1.93844998 1.20642396 2.04695808 2.86366066
 0.16470789 3.10435111 0.46005657 2.116083  ], Cost: 0.0027
Current parameters: [2.7640269  2.33242528 1.93774565 1.20503404 2.0409649  2.85182305
 0.16180277 3.09900965 0.46102766 2.10862914], Cost: 0.0019
```



```
Current parameters: [2.7666548  2.32797818 1.94179637 1.20827634 2.04089441 2.85269911
 0.16143264 3.10056791 0.45910784 2.10926815], Cost: 0.0020
Current parameters: [2.76208947 2.33375877 1.93811279 1.2000527  2.04387705 2.84511118
 0.15864069 3.10334671 0.45014158 2.11220893], Cost: 0.0054
Current parameters: [2.76356694 2.33131824 1.93703398 1.20289276 2.03996898 2.85599428
 0.16253987 3.09719617 0.45687846 2.10474744], Cost: 0.0034
Current parameters: [2.75913217 2.33607506 1.93791996 1.21536193 2.03917121 2.84972584
 0.16198808 3.10434212 0.46574609 2.10216255], Cost: 0.0041
Current parameters: [2.76981037 2.33514051 1.93754802 1.20342038 2.03915401 2.85165159
 0.16118905 3.10203877 0.46136397 2.10648503], Cost: 0.0019
Current parameters: [2.77199758 2.33273665 1.93660256 1.20216903 2.02931827 2.8523326
 0.16226243 3.09934685 0.46801252 2.11550229], Cost: 0.0024
Current parameters: [2.76872944 2.33458425 1.93766157 1.20135912 2.0435976  2.84950058
 0.16146959 3.0986992  0.46576419 2.10705946], Cost: 0.0019
Current parameters: [2.77852789 2.33351665 1.94747384 1.20106246 2.04075939 2.85560107
 0.16224508 3.10550494 0.46490819 2.10229456], Cost: 0.0029
Current parameters: [2.77305299 2.33426353 1.93298852 1.20577593 2.04250033 2.85430675
 0.16125467 3.10322641 0.46174311 2.1083844 ], Cost: 0.0022
Current parameters: [2.76938237 2.33513202 1.93768479 1.20324572 2.03910528 2.85267943
 0.15749366 3.10198007 0.46191848 2.10645943], Cost: 0.0021
Current parameters: [2.77243808 2.33880699 1.93912766 1.20652583 2.03923557 2.85107775
 0.16066466 3.0970844  0.45966177 2.10718778], Cost: 0.0011
Current parameters: [2.77339401 2.33842061 1.93939943 1.204239   2.03993023 2.85042864
 0.16015335 3.09708208 0.45754613 2.10899085], Cost: 0.0013
Current parameters: [2.77184805 2.34230307 1.94020122 1.21095453 2.03943424 2.84612401
 0.16090103 3.09851341 0.45998618 2.10814504], Cost: 0.0013
Current parameters: [2.77152669 2.34064713 1.94064733 1.20620221 2.04006165 2.85315712
 0.16144793 3.09798172 0.46010825 2.10860329], Cost: 0.0010
Current parameters: [2.77644705 2.34039422 1.94539287 1.20664544 2.04031676 2.85427137
 0.16271198 3.09801929 0.46176238 2.10570457], Cost: 0.0009
Current parameters: [2.77731395 2.33982716 1.94539569 1.20660413 2.03803374 2.8541326
 0.16286618 3.09771037 0.4635696  2.10806409], Cost: 0.0008
Current parameters: [2.77631558 2.34208555 1.94536635 1.20762415 2.03547933 2.85688868
 0.16616944 3.09239597 0.46339538 2.10729523], Cost: 0.0015
Current parameters: [2.77869206 2.34224167 1.94352271 1.20511845 2.03772142 2.85399736
 0.16278732 3.09832294 0.46438976 2.10726129], Cost: 0.0008
Current parameters: [2.78055018 2.33870768 1.94202927 1.21034114 2.04144918 2.85675049
 0.16344615 3.09830311 0.46391261 2.11022898], Cost: 0.0016
Current parameters: [2.77730196 2.33949929 1.94499961 1.20742771 2.03743848 2.85491373
 0.15970151 3.09660023 0.46441709 2.10688692], Cost: 0.0007
Current parameters: [2.7761317  2.3427651  1.9494151  1.20564097 2.0377987  2.85559392
```



```
 0.15594728 3.09854821 0.46461415 2.10967816], Cost: 0.0008
Current parameters: [2.77674858 2.33775962 1.94596768 1.20758221 2.03540413 2.85471144
 0.16093025 3.09867413 0.46404474 2.10780129], Cost: 0.0005
Current parameters: [2.77650605 2.33876777 1.9441599  1.20813691 2.03243365 2.85745303
 0.15868364 3.10211874 0.46297164 2.10311301], Cost: 0.0010
Current parameters: [2.77391586 2.33516174 1.94650515 1.20509919 2.03661843 2.85317896
 0.16142407 3.09946864 0.46973646 2.10615392], Cost: 0.0014
Current parameters: [2.77670632 2.33896642 1.94633616 1.20997879 2.03485427 2.85199795
 0.16082859 3.0989822  0.46430404 2.10751247], Cost: 0.0005
Current parameters: [2.7780507  2.33878626 1.94747385 1.2099888  2.03491208 2.85213814
 0.16108148 3.09894483 0.46458972 2.10679799], Cost: 0.0005
Current parameters: [2.77772556 2.33794398 1.94610296 1.20871254 2.0339037  2.8499362
 0.15968448 3.09821616 0.46224114 2.10797184], Cost: 0.0005
Current parameters: [2.77742439 2.3384188  1.94577718 1.21035685 2.03579911 2.85191329
 0.16025886 3.10002382 0.46444305 2.10797618], Cost: 0.0006
Current parameters: [2.77701246 2.33941067 1.94668676 1.21183018 2.03349959 2.85290877
 0.16208875 3.09745242 0.46338867 2.10950902], Cost: 0.0005
Current parameters: [2.77688687 2.3380144  1.94527855 1.21032891 2.0347658  2.8517683
 0.1616705  3.09845661 0.46461171 2.10680936], Cost: 0.0005
Current parameters: [2.77479998 2.33848105 1.94782733 1.20913892 2.03399977 2.8505102
 0.16114032 3.09827655 0.46645625 2.10805042], Cost: 0.0005
Current parameters: [2.77569706 2.3397357  1.94616677 1.2103274  2.03212777 2.85258344
 0.15996049 3.09988354 0.46398911 2.10549841], Cost: 0.0004
Current parameters: [2.77371158 2.33951515 1.94748627 1.21079643 2.03312312 2.85219699
 0.16110815 3.10021846 0.46180622 2.10409395], Cost: 0.0008
Current parameters: [2.77585488 2.34093502 1.94575478 1.20954793 2.03214694 2.85218659
 0.16098272 3.10044879 0.46388192 2.10569911], Cost: 0.0003
Current parameters: [2.77759292 2.34064491 1.9449371  1.2090175  2.02934306 2.85215823
 0.16106619 3.10051512 0.46570576 2.10563944], Cost: 0.0002
Current parameters: [2.7779864  2.34288141 1.94391895 1.20876102 2.02951663 2.85213533
 0.15955961 3.09828176 0.46678812 2.10490474], Cost: 0.0002
Current parameters: [2.77775245 2.3426031  1.94374894 1.207449   2.02947684 2.85347928
 0.15952297 3.09802058 0.46670915 2.10512525], Cost: 0.0002
Current parameters: [2.77798686 2.34263591 1.94386057 1.2083461  2.02978559 2.8518468
 0.15938313 3.09856133 0.46698766 2.10426199], Cost: 0.0002
Current parameters: [2.77827566 2.34337574 1.94395547 1.20947369 2.02830472 2.85128115
 0.15997568 3.09807534 0.46700195 2.10418526], Cost: 0.0001
Current parameters: [2.77858771 2.34391353 1.9433313  1.21019246 2.02788972 2.85080021
 0.15928434 3.09903432 0.4677404  2.10460416], Cost: 0.0001
Current parameters: [2.77815683 2.34524005 1.9431323  1.210674   2.02844384 2.85043851
 0.15870481 3.09828435 0.46763933 2.10410268], Cost: 0.0001
```



```
Current parameters: [2.77872217 2.34410645 1.9436811  1.21045858 2.0281319  2.85128427
 0.15957374 3.09919886 0.46822964 2.10435505], Cost: 0.0001
Current parameters: [2.77997324 2.34425228 1.94423345 1.20991393 2.02777573 2.85035934
 0.15869596 3.09906419 0.46719101 2.10478455], Cost: 0.0001
Current parameters: [2.77814296 2.34399107 1.94388301 1.20992865 2.02775961 2.850509
 0.15919255 3.0989729  0.46815873 2.10489905], Cost: 0.0001
Current parameters: [2.77919076 2.34347104 1.94269721 1.21037032 2.02814827 2.84970081
 0.15984493 3.09826055 0.46834068 2.10512638], Cost: 0.0002
Current parameters: [2.77861515 2.34342302 1.94344256 1.2105347  2.02775135 2.85099159
 0.15871511 3.09865187 0.4679353  2.10446715], Cost: 0.0001
Current parameters: [2.77858771 2.34391353 1.9433313  1.21019246 2.02788972 2.85080021
 0.15928434 3.09903432 0.4677404  2.10460416], Cost: 0.0001

   Return from subroutine COBYLA because the MAXFUN limit has been reached.

   NFVALS =  100   F = 8.054545E-05    MAXCV = 0.000000E+00
   X = 2.778588E+00   2.343914E+00   1.943331E+00   1.210192E+00   2.027890E+00
       2.850800E+00   1.592843E-01   3.099034E+00   4.677404E-01   2.104604E+00
Simulating for QAOA depth p = 6
Current parameters: [1.7597714  1.67504176 2.72985166 2.79561434 2.04470893 1.1647367
 1.68599699 1.18728894 0.17177411 1.39695098 2.46463536 2.60745963], Cost: 1.1781
Current parameters: [0.7597714  2.67504176 2.72985166 2.79561434 2.04470893 1.1647367
 1.68599699 1.18728894 0.17177411 1.39695098 2.46463536 2.60745963], Cost: 1.2880
Current parameters: [0.7597714  1.67504176 3.72985166 2.79561434 2.04470893 1.1647367
 1.68599699 1.18728894 0.17177411 1.39695098 2.46463536 2.60745963], Cost: 2.2415
Current parameters: [0.7597714  1.67504176 2.72985166 3.79561434 2.04470893 1.1647367
 1.68599699 1.18728894 0.17177411 1.39695098 2.46463536 2.60745963], Cost: 2.4734
Current parameters: [0.7597714  1.67504176 2.72985166 2.79561434 3.04470893 1.1647367
 1.68599699 1.18728894 0.17177411 1.39695098 2.46463536 2.60745963], Cost: 1.8899
Current parameters: [0.7597714  1.67504176 2.72985166 2.79561434 2.04470893 2.1647367
 1.68599699 1.18728894 0.17177411 1.39695098 2.46463536 2.60745963], Cost: 2.5830
Current parameters: [0.7597714  1.67504176 2.72985166 2.79561434 2.04470893 1.1647367
 2.68599699 1.18728894 0.17177411 1.39695098 2.46463536 2.60745963], Cost: 2.0922
Current parameters: [0.7597714  1.67504176 2.72985166 2.79561434 2.04470893 1.1647367
 1.68599699 2.18728894 0.17177411 1.39695098 2.46463536 2.60745963], Cost: 2.2122
Current parameters: [0.7597714  1.67504176 2.72985166 2.79561434 2.04470893 1.1647367
 1.68599699 1.18728894 1.17177411 1.39695098 2.46463536 2.60745963], Cost: 2.1280
Current parameters: [0.7597714  1.67504176 2.72985166 2.79561434 2.04470893 1.1647367
 1.68599699 1.18728894 0.17177411 2.39695098 2.46463536 2.60745963], Cost: 2.3395
Current parameters: [0.7597714  1.67504176 2.72985166 2.79561434 2.04470893 1.1647367
 1.68599699 1.18728894 0.17177411 1.39695098 3.46463536 2.60745963], Cost: 2.4082
```

```
Current parameters: [0.7597714  1.67504176 2.72985166 2.79561434 2.04470893 1.1647367
 1.68599699 1.18728894 0.17177411 1.39695098 2.46463536 3.60745963], Cost: 1.0493
Current parameters: [ 0.61533339  1.51270685  2.41228186  2.4402782   1.78437053  0.79155477
  1.39272788  0.87448896 -0.12732787  1.06342172  2.11991731  2.48398916], Cost: 1.1997
Current parameters: [0.68755239 1.59387431 2.57106676 2.61794627 1.91453973 0.97814574
 1.53936244 1.03088895 0.02222312 1.23018635 2.29227634 2.54572439], Cost: 1.0822
Current parameters: [0.75017833 1.66426004 2.97178907 2.77201414 2.02741815 1.13995125
 1.66651907 1.16651385 0.1519088  1.37479912 2.44174039 2.59925915], Cost: 0.8221
Current parameters: [0.85315747 1.59195121 2.72985166 2.79561434 2.04470893 1.1647367
 1.68599699 1.18728894 0.17177411 1.39695098 2.46463536 2.60745963], Cost: 0.2691
Current parameters: [1.01780559 1.7750856  2.71802684 2.77968086 2.0330352  1.14800301
 1.67284664 1.17326281 0.15836221 1.38199534 2.44917801 2.60192314], Cost: 0.5174
Current parameters: [0.85315747 1.59195121 2.72985166 2.86949024 1.94387564 1.1647367
 1.68599699 1.18728894 0.17177411 1.39695098 2.46463536 2.60745963], Cost: 0.2851
Current parameters: [0.84789209 1.58172414 2.70324004 2.98347549 2.18495172 1.12707772
 1.65640227 1.1557233  0.14159078 1.36329348 2.42984877 2.59499983], Cost: 1.0504
Current parameters: [0.85315747 1.59195121 2.72985166 2.79561434 2.04470893 1.24197331
 1.5877142  1.18728894 0.17177411 1.39695098 2.46463536 2.60745963], Cost: 0.3694
Current parameters: [0.84713886 1.58026113 2.69943317 2.76126456 2.02252076 1.33770393
 1.84109605 1.15120773 0.13727296 1.35847867 2.42487244 2.59321742], Cost: 0.3169
Current parameters: [0.85315747 1.59195121 2.72985166 2.79561434 2.04470893 1.1647367
 1.68599699 1.27367699 0.08142975 1.39695098 2.46463536 2.60745963], Cost: 0.3232
Current parameters: [0.84594886 1.57794976 2.69341883 2.75447292 2.01813372 1.16357402
 1.70804462 1.34735921 0.33830776 1.35087192 2.41701051 2.59040145], Cost: 0.2771
Current parameters: [0.85315747 1.59195121 2.72985166 2.79561434 2.04470893 1.1647367
 1.68599699 1.18728894 0.17177411 1.48678521 2.37771695 2.60745963], Cost: 0.0946
Current parameters: [0.84115333 1.56863531 2.66918191 2.72710362 2.00045457 1.16280055
 1.7227118  1.19422616 0.20084362 1.67301045 2.49496734 2.5790535 ], Cost: 0.8071
Current parameters: [0.86060914 1.60032619 2.74623532 2.8139464  2.05813998 1.18398944
 1.70112697 1.20342652 0.18720501 1.50399224 2.39550122 2.72149458], Cost: 0.2205
Current parameters: [0.81901074 1.54872173 2.63784725 2.69235937 1.97199236 1.09087277
 1.64683305 1.12960378 0.12769502 1.41909403 2.2706069  2.68953408], Cost: 0.3589
Current parameters: [0.86959546 1.54716364 2.79818718 2.75266959 2.06537593 1.12007675
 1.70438394 1.14413875 0.18869651 1.46504069 2.41571927 2.61059783], Cost: 0.2175
Current parameters: [0.81378976 1.63610833 2.89845456 2.79640679 1.97087892 1.21568355
 1.67300473 1.24483287 0.15521251 1.50242023 2.24799741 2.56622483], Cost: 0.4883
Current parameters: [0.94051441 1.67481937 2.72280034 2.77369887 2.04103275 1.14955643
 1.69032057 1.17422517 0.1748466  1.47692934 2.37281936 2.60173704], Cost: 0.2852
Current parameters: [0.85038051 1.59794217 2.73613366 2.8096946  2.04751624 1.12433895
 1.64654647 1.20210068 0.17755683 1.49908348 2.38171706 2.61040092], Cost: 0.0838
Current parameters: [0.84422727 1.57261545 2.70783693 2.8863162  2.11781696 1.10994398
```



```
 1.66479407 1.18611787 0.18547254 1.48148701 2.34156692 2.58690708], Cost: 0.2661
Current parameters: [0.84669486 1.6061972  2.74681896 2.82370106 2.04756221 1.1272951
 1.63838857 1.16785254 0.13340649 1.51562709 2.38696427 2.6143328 ], Cost: 0.2053
Current parameters: [0.82356948 1.60581476 2.81275629 2.80844835 2.0145129  1.14730846
 1.64318803 1.24895661 0.19220695 1.49909419 2.31508617 2.58818086], Cost: 0.2656
Current parameters: [0.83905771 1.58443587 2.70843456 2.78549732 2.03018551 1.10698276
 1.63697835 1.19402529 0.17208035 1.48133639 2.35447629 2.63033862], Cost: 0.1136
Current parameters: [0.87113774 1.57926159 2.73575575 2.81340657 2.04159163 1.12875116
 1.64114533 1.20703276 0.17262514 1.50502063 2.37782533 2.61022398], Cost: 0.0828
Current parameters: [0.8589217  1.56910335 2.70957964 2.79849781 2.03255848 1.1269049
 1.64970094 1.21940395 0.19053115 1.54295948 2.39861869 2.59464284], Cost: 0.0344
Current parameters: [0.85518638 1.56546957 2.70227524 2.78575033 2.04690815 1.15966382
 1.60827884 1.21090246 0.21062823 1.53581921 2.40943791 2.58972523], Cost: 0.0637
Current parameters: [0.8589217  1.56910335 2.70957964 2.78442414 2.05176764 1.1269049
 1.64970094 1.23311746 0.1761896  1.54581271 2.39585806 2.59464284], Cost: 0.0356
Current parameters: [0.85286647 1.56321273 2.69773867 2.80696914 2.0160527  1.12602478
 1.65124771 1.24981623 0.17689225 1.51278795 2.43415048 2.58667103], Cost: 0.0247
Current parameters: [0.86308407 1.57237211 2.69370454 2.80005864 2.0128731  1.11981747
 1.64877707 1.24454293 0.17418562 1.50781589 2.42998267 2.56233444], Cost: 0.0381
Current parameters: [0.88498161 1.59301965 2.68117061 2.80099417 2.01419275 1.12413178
 1.66178285 1.26730023 0.19683496 1.51870247 2.44591734 2.61277352], Cost: 0.0517
Current parameters: [0.85686902 1.55782732 2.71484782 2.78860103 2.01087236 1.11893417
 1.65219291 1.24357901 0.1791953  1.51092915 2.44687583 2.59038945], Cost: 0.0291
Current parameters: [0.85294587 1.53292817 2.68982693 2.8200867  2.04738628 1.1041956
 1.67158231 1.24859884 0.20268787 1.50377281 2.44143328 2.57764813], Cost: 0.0509
Current parameters: [0.84900032 1.56234835 2.70875715 2.80281016 2.00999633 1.12968066
 1.65313522 1.2676509  0.19162088 1.5081755  2.4205226  2.58151789], Cost: 0.0623
Current parameters: [0.83612998 1.56029971 2.69190496 2.82260691 2.0073385  1.12498407
 1.6543476  1.23911385 0.14496206 1.55129612 2.45935196 2.587288  ], Cost: 0.0572
Current parameters: [0.84631426 1.55522035 2.67996561 2.79571883 2.0073676  1.11648584
 1.64776303 1.24553769 0.17312732 1.50731905 2.42531431 2.59707629], Cost: 0.0284
Current parameters: [0.86319011 1.55375751 2.69725732 2.80812896 2.01301122 1.13009348
 1.65045294 1.25154074 0.17419826 1.51512435 2.43205109 2.58644566], Cost: 0.0264
Current parameters: [0.84983706 1.56340009 2.69319169 2.79959291 2.01577025 1.14461154
 1.67210112 1.24148305 0.17461531 1.5068248  2.43465225 2.58548078], Cost: 0.0276
Current parameters: [0.8551933  1.56525793 2.70257828 2.81152097 2.01419556 1.11940919
 1.65882557 1.25002822 0.17042448 1.50947993 2.42925714 2.58967089], Cost: 0.0192
Current parameters: [0.85865692 1.56809637 2.70675826 2.81943139 2.02325345 1.12566609
 1.64647281 1.23927601 0.16155055 1.49205279 2.43070155 2.59979657], Cost: 0.0115
Current parameters: [0.85855073 1.56810591 2.70683655 2.82648105 2.01354834 1.12567403
 1.64649206 1.23242943 0.16862676 1.49087895 2.43208039 2.59970504], Cost: 0.0105
```



```
Current parameters: [0.85105658 1.56919217 2.7069446  2.83806806 2.01140235 1.12371829
 1.64674574 1.22411456 0.14857435 1.50351632 2.44334266 2.60402679], Cost: 0.0335
Current parameters: [0.86386888 1.57828997 2.70522035 2.82346386 2.00796755 1.1291557
 1.64481582 1.23568665 0.16758609 1.49342377 2.43273753 2.60568416], Cost: 0.0089
Current parameters: [0.87756897 1.58029007 2.69454754 2.82839628 2.02070271 1.11940236
 1.65427487 1.23512569 0.17875748 1.48982711 2.44174157 2.61466877], Cost: 0.0164
Current parameters: [0.86211979 1.57677406 2.70299069 2.82529768 2.003487   1.12917179
 1.64532029 1.24437001 0.16514843 1.4856475  2.44067381 2.60337739], Cost: 0.0125
Current parameters: [0.86063371 1.58090373 2.71920708 2.82652267 2.00812014 1.13418967
 1.64371406 1.24409581 0.1782081  1.48415794 2.41369347 2.61546315], Cost: 0.0165
Current parameters: [0.86938358 1.58424415 2.70451963 2.82135985 2.00720616 1.12634093
 1.64277473 1.2319449  0.16594709 1.48859257 2.42825152 2.59565729], Cost: 0.0110
Current parameters: [0.86574907 1.57893479 2.71132428 2.82263946 2.00877614 1.12981884
 1.64562574 1.23549327 0.16866696 1.49415823 2.43644967 2.60431595], Cost: 0.0088
Current parameters: [0.86553905 1.57500348 2.71285646 2.81231097 2.00427122 1.12399339
 1.64446158 1.23107044 0.16818378 1.4905006  2.43742088 2.60978533], Cost: 0.0154
Current parameters: [0.86183187 1.5820317  2.71061761 2.82086472 2.00970732 1.13202726
 1.6498388  1.23335417 0.16910729 1.49227059 2.43722458 2.60416314], Cost: 0.0105
Current parameters: [0.87327637 1.57226508 2.71007504 2.82194412 2.00671565 1.13914374
 1.65132404 1.23390365 0.165734   1.49335975 2.43411685 2.60404669], Cost: 0.0091
Current parameters: [0.86579193 1.57892792 2.7107594  2.82296873 2.01129883 1.13318003
 1.64065409 1.23310347 0.16871083 1.49139319 2.43824729 2.60559538], Cost: 0.0111
Current parameters: [0.86487242 1.57743364 2.70757661 2.81830764 2.00749447 1.13346401
 1.64329532 1.23929126 0.17726207 1.50222412 2.43660711 2.59843684], Cost: 0.0028
Current parameters: [0.86388355 1.57839009 2.70698361 2.82290063 2.00506463 1.13511728
 1.64270731 1.24043283 0.17555375 1.51611724 2.43617078 2.59464383], Cost: 0.0056
Current parameters: [0.86179238 1.57726298 2.71147543 2.8179497  2.00517533 1.1359122
 1.64148674 1.24107857 0.17668199 1.50108956 2.43253092 2.59809934], Cost: 0.0029
Current parameters: [0.86927949 1.57845089 2.70804506 2.8210167  2.01121665 1.13294568
 1.64597451 1.24546119 0.18823194 1.50162051 2.43117648 2.60099843], Cost: 0.0055
Current parameters: [0.86582838 1.57723903 2.70944526 2.82059248 2.00546318 1.13113222
 1.64384517 1.24411522 0.17593614 1.50063144 2.43988592 2.59665093], Cost: 0.0043
Current parameters: [0.86069676 1.57561466 2.69737208 2.81465441 2.00452472 1.14099724
 1.64224085 1.24201746 0.17983193 1.49883397 2.43929619 2.59589206], Cost: 0.0057
Current parameters: [0.86554344 1.57501213 2.70869972 2.81310135 2.00460225 1.1302645
 1.64273894 1.23828246 0.17849348 1.50373296 2.43664733 2.60005881], Cost: 0.0041
Current parameters: [0.86542118 1.57803487 2.70752532 2.81966391 2.00527408 1.13314721
 1.6430865  1.2373484  0.17866601 1.50143046 2.4364274  2.59730901], Cost: 0.0026
Current parameters: [0.86798551 1.5804041  2.70689766 2.8168974  2.00697825 1.13208637
 1.64211409 1.23736539 0.17703541 1.50062706 2.43378888 2.59225362], Cost: 0.0036
Current parameters: [0.86729127 1.5757856  2.70793021 2.81979188 2.00493096 1.13470875
```



```
 1.64475127 1.23701718 0.17789013 1.50130986 2.43591529 2.5968552 ], Cost: 0.0025
Current parameters: [0.8637752  1.57141611 2.7083704  2.82113502 2.0077868  1.1323977
 1.64482512 1.23562189 0.17926547 1.50083884 2.43563027 2.59370267], Cost: 0.0028
Current parameters: [0.86869598 1.57454588 2.7076644  2.82054974 2.00531807 1.13495588
 1.64148628 1.23714158 0.17781244 1.50123773 2.43601077 2.59729878], Cost: 0.0022
Current parameters: [0.86914907 1.57583223 2.7118494  2.81993061 2.00767938 1.13833427
 1.64051186 1.23422996 0.17898221 1.50072663 2.43964587 2.59716574], Cost: 0.0011
Current parameters: [0.86911961 1.57648132 2.71234966 2.82254978 2.0092258  1.13886546
 1.63999501 1.22984764 0.17565352 1.4982177  2.43881984 2.60081266], Cost: 0.0030
Current parameters: [0.8686774  1.57536339 2.71042985 2.81861023 2.00754642 1.13914286
 1.64045483 1.23461134 0.17958677 1.49765679 2.44028605 2.59736206], Cost: 0.0014
Current parameters: [0.86762504 1.57562535 2.70922297 2.82041526 2.00659567 1.14328186
 1.63905448 1.2338729  0.17988238 1.50465776 2.44150618 2.59518902], Cost: 0.0011
Current parameters: [0.86810654 1.57587821 2.70869117 2.82014353 2.00796062 1.14398018
 1.63935795 1.23339014 0.18235244 1.50508003 2.43938549 2.5962631 ], Cost: 0.0010
Current parameters: [0.87055126 1.57620157 2.71133353 2.82344866 2.0082875  1.1446283
 1.63952952 1.23763435 0.18594326 1.50497    2.4416334  2.59554258], Cost: 0.0020
Current parameters: [0.86726089 1.57425677 2.70925839 2.81982865 2.0064485  1.14352007
 1.6395925  1.23308668 0.18336437 1.50581396 2.44045593 2.59874099], Cost: 0.0013
Current parameters: [0.87089225 1.57576704 2.71029797 2.81463623 2.0074142  1.14290713
 1.63717408 1.23160845 0.18310835 1.50685795 2.43906748 2.59359417], Cost: 0.0020
Current parameters: [0.86962869 1.57597258 2.70671704 2.82037202 2.00913107 1.14273455
 1.64027349 1.23244295 0.1825649  1.50560454 2.44137239 2.59650511], Cost: 0.0011
Current parameters: [0.86814405 1.5771687  2.70924099 2.82346769 2.00970445 1.14639021
 1.63800383 1.22894054 0.17917524 1.50425142 2.43820779 2.59857959], Cost: 0.0002
Current parameters: [0.86748589 1.57673804 2.70662108 2.82188604 2.01001418 1.14942709
 1.63706941 1.22826469 0.17965949 1.4980636  2.43936111 2.59932047], Cost: 0.0010
Current parameters: [0.867259   1.57603066 2.70936137 2.82488754 2.00918904 1.14533775
 1.6378791  1.22709636 0.18064316 1.50353342 2.43798328 2.5966719 ], Cost: 0.0002
Current parameters: [0.86421045 1.57255737 2.71032916 2.82480484 2.01502537 1.14520268
 1.63760751 1.22768099 0.17903419 1.50475266 2.43803753 2.59627262], Cost: 0.0003
Current parameters: [0.86591024 1.57779094 2.70889446 2.82499831 2.00963439 1.1439274
 1.63529269 1.22744978 0.18135777 1.50362607 2.43849998 2.59723993], Cost: 0.0003
Current parameters: [0.86381285 1.5804611  2.71065949 2.82485631 2.00961634 1.14573882
 1.64255169 1.22493917 0.18030213 1.50440734 2.43804638 2.59631111], Cost: 0.0005
Current parameters: [0.86642299 1.57625464 2.70779281 2.82494845 2.00905642 1.14536113
 1.6381814  1.2285386  0.18076499 1.50341379 2.4349258  2.59598004], Cost: 0.0003
Current parameters: [0.86903777 1.57480057 2.70763325 2.82897807 2.00771049 1.14386848
 1.63746467 1.22474837 0.17651811 1.50592389 2.43741211 2.59855679], Cost: 0.0008
Current parameters: [0.86851081 1.57635832 2.71056612 2.82287663 2.00948141 1.14562195
 1.63665128 1.22554809 0.18057452 1.50435602 2.43657855 2.59550414], Cost: 0.0003
```

```
Current parameters: [0.86689541 1.57598455 2.70872606 2.82502983 2.00892279 1.14658192
 1.63751514 1.22704336 0.18089789 1.50451316 2.43845439 2.59616629], Cost: 0.0001
Current parameters: [0.86814866 1.57665762 2.71039789 2.82717481 2.00972483 1.14787347
 1.63725979 1.2283026  0.18212069 1.50456749 2.43828655 2.59557419], Cost: 0.0001
Current parameters: [0.86705781 1.57573784 2.70844237 2.82499572 2.0093046  1.14670081
 1.63775336 1.22648176 0.18206985 1.50490212 2.43804172 2.59735738], Cost: 0.0000
Current parameters: [0.86546786 1.57444493 2.71044801 2.82461485 2.00719177 1.1472596
 1.6373124  1.22722139 0.18241381 1.50539911 2.43770432 2.59830323], Cost: 0.0001
Current parameters: [0.86738633 1.57562977 2.70849321 2.82479214 2.00916867 1.14566107
 1.63824389 1.22711387 0.18249609 1.50611224 2.43832538 2.59692747], Cost: 0.0000
Current parameters: [0.86788737 1.57450741 2.70709962 2.82340449 2.00893348 1.14690096
 1.63864154 1.2276531  0.18414527 1.50504192 2.44014517 2.59611394], Cost: 0.0003
Current parameters: [0.86818767 1.57642774 2.70815053 2.8250307  2.0076884  1.1455981
 1.63826065 1.2268631  0.18267779 1.50594427 2.43826214 2.59712208], Cost: 0.0000
Current parameters: [0.86738633 1.57562977 2.70849321 2.82479214 2.00916867 1.14566107
 1.63824389 1.22711387 0.18249609 1.50611224 2.43832538 2.59692747], Cost: 0.0000

   Return from subroutine COBYLA because the MAXFUN limit has been reached.

   NFVALS =  100   F = 2.977250E-05    MAXCV = 0.000000E+00
   X = 8.673863E-01   1.575630E+00   2.708493E+00   2.824792E+00   2.009169E+00
       1.145661E+00   1.638244E+00   1.227114E+00   1.824961E-01   1.506112E+00
       2.438325E+00   2.596927E+00
Simulating for QAOA depth p = 7
Current parameters: [2.94651361 1.08108657 1.04641807 0.74058181 1.60826563 1.27680447
 1.61006157 0.12089267 0.29115671 1.69654589 1.7786743  1.3110131
 1.25902085 2.08634296], Cost: 2.6263
Current parameters: [1.94651361 2.08108657 1.04641807 0.74058181 1.60826563 1.27680447
 1.61006157 0.12089267 0.29115671 1.69654589 1.7786743  1.3110131
 1.25902085 2.08634296], Cost: 1.9947
Current parameters: [1.94651361 2.08108657 2.04641807 0.74058181 1.60826563 1.27680447
 1.61006157 0.12089267 0.29115671 1.69654589 1.7786743  1.3110131
 1.25902085 2.08634296], Cost: 2.1283
Current parameters: [1.94651361 2.08108657 1.04641807 1.74058181 1.60826563 1.27680447
 1.61006157 0.12089267 0.29115671 1.69654589 1.7786743  1.3110131
 1.25902085 2.08634296], Cost: 2.1574
Current parameters: [1.94651361 2.08108657 1.04641807 0.74058181 2.60826563 1.27680447
 1.61006157 0.12089267 0.29115671 1.69654589 1.7786743  1.3110131
 1.25902085 2.08634296], Cost: 2.3897
Current parameters: [1.94651361 2.08108657 1.04641807 0.74058181 1.60826563 2.27680447
 1.61006157 0.12089267 0.29115671 1.69654589 1.7786743  1.3110131
```



```
 1.25902085 2.08634296], Cost: 2.4462
Current parameters: [1.94651361 2.08108657 1.04641807 0.74058181 1.60826563 1.27680447
 2.61006157 0.12089267 0.29115671 1.69654589 1.7786743  1.3110131
 1.25902085 2.08634296], Cost: 3.5410
Current parameters: [1.94651361 2.08108657 1.04641807 0.74058181 1.60826563 1.27680447
 1.61006157 1.12089267 0.29115671 1.69654589 1.7786743  1.3110131
 1.25902085 2.08634296], Cost: 3.0171
Current parameters: [1.94651361 2.08108657 1.04641807 0.74058181 1.60826563 1.27680447
 1.61006157 0.12089267 1.29115671 1.69654589 1.7786743  1.3110131
 1.25902085 2.08634296], Cost: 1.9010
Current parameters: [1.94651361 2.08108657 1.04641807 0.74058181 1.60826563 1.27680447
 1.61006157 0.12089267 1.29115671 2.69654589 1.7786743  1.3110131
 1.25902085 2.08634296], Cost: 1.5212
Current parameters: [1.94651361 2.08108657 1.04641807 0.74058181 1.60826563 1.27680447
 1.61006157 0.12089267 1.29115671 2.69654589 2.7786743  1.3110131
 1.25902085 2.08634296], Cost: 0.6190
Current parameters: [1.94651361 2.08108657 1.04641807 0.74058181 1.60826563 1.27680447
 1.61006157 0.12089267 1.29115671 2.69654589 2.7786743  2.3110131
 1.25902085 2.08634296], Cost: 1.4309
Current parameters: [1.94651361 2.08108657 1.04641807 0.74058181 1.60826563 1.27680447
 1.61006157 0.12089267 1.29115671 2.69654589 2.7786743  1.3110131
 2.25902085 2.08634296], Cost: 3.5092
Current parameters: [1.94651361 2.08108657 1.04641807 0.74058181 1.60826563 1.27680447
 1.61006157 0.12089267 1.29115671 2.69654589 2.7786743  1.3110131
 1.25902085 3.08634296], Cost: 0.5866
Current parameters: [ 1.82573867  2.12875977  1.0108064   0.69721001  1.50291919  1.15639228
  1.19764848 -0.15178537  1.31616712  2.79783144  3.0193135   1.09446839
  0.48817778  3.09496678], Cost: 3.6739
Current parameters: [1.46666646 2.08108657 1.04641807 0.74058181 1.60826563 1.27680447
 1.75058455 0.12089267 1.29115671 2.69654589 2.7786743  1.3110131
 1.25902085 3.08634296], Cost: 0.6289
Current parameters: [2.22551313 2.09325154 1.03733089 0.72951445 1.58138394 1.24607839
 2.54230085 0.05131228 1.29753872 2.72239135 2.8400792  1.25575648
 1.06232162 3.08854354], Cost: 1.0516
Current parameters: [1.94651361 2.53661377 1.04641807 0.74058181 1.81440871 1.27680447
 1.61006157 0.12089267 1.29115671 2.69654589 2.7786743  1.3110131
 1.25902085 3.08634296], Cost: 1.0046
Current parameters: [1.94970009 1.65500306 1.04383612 0.73743723 2.51059671 1.26807422
 1.61512719 0.10112269 1.29297004 2.7038894  2.79612136 1.29531295
 1.20313241 3.08696822], Cost: 0.9211
Current parameters: [1.94651361 2.08108657 1.43284748 0.42329281 1.60826563 1.27680447
```



```
 1.61006157 0.12089267 1.29115671 2.69654589 2.7786743  1.3110131
 1.25902085 3.08634296], Cost: 1.5383
Current parameters: [1.94806155 2.07445245 1.66673939 1.5242519  1.60387688 1.27256347
 1.61252236 0.11128877 1.29203759 2.70011323 2.78714976 1.30338625
 1.23187127 3.0866467 ], Cost: 2.1584
Current parameters: [ 1.94651361  2.08108657  1.04641807  0.74058181  1.60826563  1.73419331
  1.61006157 -0.08108615  1.29115671  2.69654589  2.7786743   1.3110131
  1.25902085  3.08634296], Cost: 0.5531
Current parameters: [1.95491072 2.0450983  0.92273751 0.74278969 1.58445791 2.13242263
 1.62341065 0.81226941 1.29593525 2.71589774 2.82465137 1.26963957
 1.11174183 3.08799065], Cost: 1.9568
Current parameters: [ 2.00108431  2.03554352  0.97733848  0.75626557  1.55527055  1.67533654
  1.57102828 -0.05756766  1.29115671  3.0360879   2.43913229  1.3110131
  1.25902085  3.11801363], Cost: 0.6979
Current parameters: [ 1.55098571  2.37558151  1.40822954  0.62665369  1.97314455  2.14738143
  1.91675152 -0.32192504  1.29702689  2.91544755  2.6400256   1.26018801
  1.07809671  2.8528317 ], Cost: 0.6455
Current parameters: [ 1.94009649  2.08012449  1.0298966   0.73869272  1.61108515  1.73873559
  1.61887101 -0.0963407   1.70296729  2.49658363  2.58210918  1.3019938
  1.22691449  3.08191553], Cost: 1.8026
Current parameters: [ 1.94651361  2.08108657  1.04641807  0.74058181  1.60826563  1.73419331
  1.61006157 -0.08108615  1.18798238  2.59337156  2.67549997  1.13618412
  1.25902085  3.08634296], Cost: 0.1668
Current parameters: [ 1.93051705  2.07868831  1.00523344  0.7358727   1.61529412  1.74551627
  1.6320217  -0.11911261  0.92162328  2.4033735   2.49397029  1.45144171
  1.1789863   3.0753063 ], Cost: 2.0305
Current parameters: [ 1.96972951  1.95639019  1.01657485  0.74670017  1.81845463  1.70761609
  1.59345572 -0.0745634   1.18838254  2.5949921   2.67935012  1.13271947
  1.25074021  3.09981655], Cost: 0.1122
Current parameters: [ 1.93200894  1.9479014   0.91747867  0.73558005  1.83990025  1.73409183
  1.64557607 -0.16530721  1.11763175  2.70678074  2.81142858  0.88430956
  1.61006559  3.07373539], Cost: 1.7240
Current parameters: [ 1.87728628  1.97793878  1.04742252  0.74239086  1.8352962   1.73861486
  1.37234684 -0.07015327  1.18699337  2.58936634  2.66598417  1.14474713
  1.29355555  3.08652881], Cost: 0.2174
Current parameters: [ 1.95320557  1.93844739  0.91901312  0.73674162  1.77003485  1.6470445
  1.48994628 -0.19048844  1.16369398  2.67980309  2.81890523  0.94703107
  0.86626229  3.10612825], Cost: 3.0633
Current parameters: [ 2.0049183   1.92305829  1.02502816  0.81470192  1.80329033  1.67387185
  1.58331961 -0.06032744  1.18843359  2.59519886  2.67984136  1.13227741
  1.24916661  2.86791561], Cost: 0.3514
```



```
Current parameters: [ 1.99219785  1.92735575  1.2865201   1.13685621  1.81987709  1.70068917
  1.61094105 -0.05363584  1.17277656  2.60559915  2.67981059  1.10829831
  1.27425232  3.24562746], Cost: 0.2649
Current parameters: [ 1.88097732  2.03111434  1.12169456  0.70872015  1.90884384  1.78291938
  1.66521983 -0.16481597  1.18962513  2.6506805   2.64064954  1.12196089
  1.21244246  3.04300663], Cost: 0.1176
Current parameters: [1.93573212 1.97899385 1.02513593 0.72098234 1.86184718 1.95001588
 1.65324511 0.34653095 1.1720945  2.63348959 2.68472919 1.08425099
 1.20938548 3.08474821], Cost: 3.2303
Current parameters: [ 2.16114294  2.04595316  1.07664941  0.70322786  1.80006689  1.77631323
  1.53877154 -0.10222252  1.17753095  2.57960647  2.66358675  1.07897773
  1.25626659  3.09734462], Cost: 0.3687
Current parameters: [ 2.12269109  1.827778    0.99580227  0.74243197  2.11577741  1.68995211
  1.55639529 -0.16723862  1.22905302  2.67917651  2.77476773  1.43154262
  1.30055823  3.13606821], Cost: 0.5353
Current parameters: [ 1.96692958  1.85806569  1.17248755  0.62234575  1.77887346  1.60572588
  1.59890775 -0.05479489  1.18193134  2.59370902  2.68075341  1.12965155
  1.2749382   3.10634883], Cost: 0.3370
Current parameters: [ 1.95730397  2.0000082   1.032304    0.74312904  1.84556737  1.68763363
  1.60234345 -0.06517643  1.18838254  2.51767972  2.75666249  1.13271947
  1.25074021  3.09260526], Cost: 0.2105
Current parameters: [ 1.9796952   2.09068618  1.00060456  0.75320042  1.87515604  1.54312864
  1.5882804  -0.01673264  1.18472998  2.66613642  2.60827385  1.13098247
  1.26444071  3.11021874], Cost: 0.2228
Current parameters: [ 1.97250142  1.94674408  1.01667678  0.74745997  1.83453694  1.70485772
  1.59084871 -0.07179426  1.30653186  2.58908742  2.67311781  1.09821946
  1.25484076  3.1015293 ], Cost: 0.2826
Current parameters: [ 1.94920497  1.96454212  0.99281518  0.76208794  1.78357552  1.68979488
  1.62471513 -0.16525958  1.17786856  2.45352137  2.53000916  1.21052188
  1.29307655  3.11399666], Cost: 0.8519
Current parameters: [1.96224265 1.95375613 1.02090254 0.73556903 1.83944529 1.76136653
 1.6142306  0.02941615 1.17694335 2.59419328 2.65869055 1.14963831
 1.24963598 3.10055039], Cost: 0.5570
Current parameters: [ 1.97813008  1.95160708  0.98707949  0.70454123  1.83759157  1.70763759
  1.58924093 -0.0823682   1.19265435  2.59858371  2.68455234  1.1548733
  1.25174576  3.08675774], Cost: 0.1080
Current parameters: [ 2.01356594  1.91954857  1.01553102  0.75601106  1.89840768  1.70137718
  1.58123681 -0.105481    1.19387171  2.62478146  2.70917105  1.21348347
  1.26353982  3.11281004], Cost: 0.0667
Current parameters: [ 2.01654518  1.91727793  1.01948342  0.75665225  1.89714629  1.69485382
  1.56907917 -0.10287691  1.19780197  2.61661646  2.70212641  1.22479442
```

```
  1.20516502  3.11599675], Cost: 0.1867
Current parameters: [ 1.9991284   1.90087508  0.97814772  0.76273896  1.90223684  1.68832349
  1.57872418 -0.15764997  1.15580151  2.67891032  2.76067803  1.15594374
  1.27024492  3.13081239], Cost: 0.2274
Current parameters: [ 2.01055926  1.93826845  1.02177224  0.75357541  1.86726859  1.707002
  1.58209238 -0.09867297  1.20945806  2.65434576  2.73757362  1.23627822
  1.2610196   3.11083831], Cost: 0.1082
Current parameters: [ 1.99977887  1.93114589  0.99925591  0.77135068  1.86164652  1.69082675
  1.60095762 -0.17479577  1.19016607  2.58704914  2.66572339  1.2656299
  1.30732868  3.12630896], Cost: 0.2518
Current parameters: [ 2.03977095  1.94091414  1.02422901  0.73616598  1.89804476  1.71926025
  1.5737656  -0.11279994  1.19201204  2.62212021  2.70639338  1.20440063
  1.26476984  3.15478609], Cost: 0.0995
Current parameters: [ 2.09548315  1.93568944  1.03523974  0.76901141  1.88774789  1.7113143
  1.56085049 -0.12450419  1.17972178  2.62510858  2.70517716  1.19299106
  1.2760359   3.03334702], Cost: 0.0562
Current parameters: [ 2.09301622  1.89465462  1.05608703  0.75373268  1.87060037  1.73667228
  1.56260128 -0.13521854  1.1798169   2.60944416  2.72084215  1.19303473
  1.27567447  3.03179008], Cost: 0.0210
Current parameters: [ 2.0970058   1.87631205  1.12109189  0.70313913  1.86554636  1.65224453
  1.56420094 -0.12443088  1.16801969  2.62832763  2.707213    1.18989005
  1.29846982  3.03372486], Cost: 0.1045
Current parameters: [ 2.11399156  1.87627399  1.03023655  0.76316006  1.84824835  1.71826806
  1.54185284 -0.11262854  1.17948888  2.59546831  2.73033347  1.19587484
  1.28578453  3.04580379], Cost: 0.0173
Current parameters: [ 2.06681688  1.886791    1.04786278  0.76150204  1.85878891  1.73500712
  1.43324371 -0.11738194  1.17543596  2.59558284  2.72332413  1.20103874
  1.30995044  3.0363463 ], Cost: 0.0562
Current parameters: [ 2.10009238  1.8545676   1.02872697  0.77283214  1.87944539  1.72588246
  1.5552408  -0.07913166  1.17275129  2.59162521  2.72193016  1.19829242
  1.31053591  3.0377852 ], Cost: 0.0779
Current parameters: [ 2.165597    1.9052654   1.03714164  0.73530536  1.79235494  1.78831484
  1.54062202 -0.09153087  1.13848667  2.62405806  2.73422279  1.20605584
  1.27174454  3.04827441], Cost: 0.1014
Current parameters: [ 2.12842237  1.87319314  1.03077583  0.76064285  1.8451014   1.72792341
  1.53923145 -0.11790925  1.23520652  2.60038179  2.72914489  1.17628905
  1.29125778  3.04515099], Cost: 0.0486
Current parameters: [ 2.11790952  1.87482786  1.01588111  0.7420818   1.85836364  1.7178497
  1.53994407 -0.11631496  1.18161689  2.59559739  2.73458505  1.20691083
  1.28628545  3.03914368], Cost: 0.0139
Current parameters: [ 2.11937986  1.89937022  1.02690639  0.74158372  1.86423347  1.71448439
```



```
  1.54407083 -0.11507274  1.18183932  2.55922895  2.77649475  1.20279833
  1.28858206  3.03536495], Cost: 0.0126
Current parameters: [ 2.11990502  1.90516746  1.03110579  0.74264489  1.85694925  1.71552388
  1.54774549 -0.11669139  1.18826714  2.54495791  2.76172806  1.22137772
  1.29524626  3.03435788], Cost: 0.0275
Current parameters: [ 2.11344565  1.89089396  1.00620775  0.75145741  1.85356782  1.7107369
  1.55225922 -0.16209824  1.16613991  2.559252    2.7878388   1.19472099
  1.30915702  3.03717781], Cost: 0.0159
Current parameters: [ 2.13206357  1.89583304  1.03569988  0.75210602  1.87550169  1.71528754
  1.54073787 -0.12103057  1.18320714  2.5678623   2.78455545  1.21794064
  1.29008467  3.04360995], Cost: 0.0073
Current parameters: [ 2.13313927  1.87694925  1.03042176  0.76242093  1.88157577  1.70754697
  1.53323164 -0.13596897  1.17938478  2.55155484  2.78583652  1.22074791
  1.23799723  3.03771399], Cost: 0.0563
Current parameters: [ 2.14651006  1.89525447  1.04186069  0.74460249  1.88544305  1.7130344
  1.53701133 -0.12604398  1.17533641  2.55800053  2.77237073  1.2056134
  1.2937986   3.05115417], Cost: 0.0148
Current parameters: [ 2.129386    1.88721108  1.06427332  0.72945084  1.85998786  1.67384964
  1.54420586 -0.12223695  1.18065673  2.58548311  2.79572096  1.22150787
  1.29730916  3.03946289], Cost: 0.0047
Current parameters: [ 2.11513427  1.88618924  1.06190819  0.72372933  1.86408448  1.67101994
  1.54701194 -0.12270093  1.186232    2.58271752  2.79647882  1.22309877
  1.29478741  3.06501678], Cost: 0.0172
Current parameters: [ 2.14831768  1.90116343  1.06675371  0.71161464  1.83255756  1.70347321
  1.54609178 -0.11603238  1.15759539  2.60614836  2.81208193  1.22553592
  1.29468631  3.0498165 ], Cost: 0.0153
Current parameters: [ 2.11850131  1.87963326  1.07314486  0.72540731  1.85816723  1.68361035
  1.52096571 -0.1258622   1.17979646  2.58174347  2.79796005  1.22264257
  1.3024845   3.03702064], Cost: 0.0075
Current parameters: [ 2.12600854  1.9297281   1.05078172  0.73952515  1.86824023  1.65306311
  1.51557344 -0.12275011  1.17596947  2.60580957  2.79213906  1.22102983
  1.30219814  3.03610514], Cost: 0.0103
Current parameters: [ 2.12207201  1.87226257  1.06090171  0.73849042  1.88518149  1.67546457
  1.55708386 -0.0939435   1.16299733  2.5971442   2.80818826  1.22124069
  1.32971987  3.02276761], Cost: 0.0427
Current parameters: [ 2.13827296  1.88736157  1.06416192  0.72804877  1.85719732  1.6773023
  1.54298828 -0.12611406  1.20595524  2.59226746  2.80069317  1.21081296
  1.3024048   3.0365907 ], Cost: 0.0320
Current parameters: [ 2.13462259  1.88231672  1.05780093  0.73195353  1.85448801  1.66915146
  1.53889595 -0.11671607  1.18057985  2.5817111   2.79791514  1.22233772
  1.29887967  3.04284055], Cost: 0.0063
```



```
Current parameters: [ 2.12934026  1.88200973  1.06440474  0.73345043  1.86199215  1.66855814
  1.54183354 -0.12828724  1.17088944  2.58394993  2.79874591  1.22566213
  1.27146355  3.03127225], Cost: 0.0036
Current parameters: [ 2.13130385  1.88187361  1.05784143  0.72329683  1.86609003  1.66848112
  1.54130036 -0.1302688   1.17264385  2.58253563  2.79903443  1.23312946
  1.27244464  3.0279257 ], Cost: 0.0065
Current parameters: [ 2.12894263  1.88891057  1.07317151  0.74212532  1.85052929  1.66705638
  1.54631196 -0.13136087  1.16881215  2.573385    2.78616716  1.24353823
  1.27453463  3.0280516 ], Cost: 0.0150
Current parameters: [ 2.13233475  1.88404911  1.07013207  0.73023675  1.86566647  1.66922843
  1.5394408  -0.1173362   1.17388316  2.5828448   2.79472663  1.22631275
  1.26683818  3.03163067], Cost: 0.0080
Current parameters: [ 2.14307584  1.88091055  1.06899581  0.72844463  1.86978786  1.6645988
  1.53918118 -0.13941476  1.15737383  2.57602567  2.78931283  1.21350656
  1.27560097  3.03709642], Cost: 0.0055
Current parameters: [ 2.12894032  1.88831384  1.06661068  0.73269231  1.8627694   1.66767452
  1.54305868 -0.12761969  1.17086216  2.57440358  2.80866078  1.22372841
  1.2719429   3.02983728], Cost: 0.0044
Current parameters: [ 2.11683222  1.88424154  1.05545277  0.72371603  1.84981716  1.66695256
  1.54497043 -0.12336622  1.16638258  2.57869822  2.78869522  1.21104459
  1.26866157  3.02205612], Cost: 0.0038
Current parameters: [ 2.1248123   1.87728035  1.06386799  0.73831895  1.87003317  1.66206749
  1.54233765 -0.12757     1.17471863  2.57999949  2.79594741  1.22467381
  1.2746482   3.02674388], Cost: 0.0021
Current parameters: [ 2.12027688  1.87127512  1.0620483   0.74247994  1.87686689  1.6658345
  1.54936091 -0.11773757  1.15347377  2.58845347  2.80407718  1.22493112
  1.28297811  3.02090855], Cost: 0.0027
Current parameters: [ 2.12694003  1.86686279  1.06727398  0.73594596  1.86761343  1.6673898
  1.5494422  -0.12766692  1.17743739  2.57563087  2.79715678  1.22397255
  1.27386711  3.026358  ], Cost: 0.0036
Current parameters: [ 2.13018602  1.87786159  1.06063707  0.74685002  1.86991815  1.6602392
  1.54105906 -0.13661622  1.1838936   2.59434553  2.80364354  1.20911415
  1.2753323   3.01312349], Cost: 0.0089
Current parameters: [ 2.12339122  1.87508284  1.07084572  0.73238379  1.86639372  1.6516164
  1.5433485  -0.12789449  1.17438015  2.58423327  2.79876323  1.22564492
  1.27631047  3.02706751], Cost: 0.0015
Current parameters: [ 2.1064076   1.87269556  1.06497216  0.73335448  1.87290935  1.64772404
  1.54794949 -0.13300285  1.17468519  2.58642437  2.802413    1.22141703
  1.27051618  3.04880336], Cost: 0.0222
Current parameters: [ 2.11814313  1.87048652  1.0748976   0.732289    1.86381644  1.65710472
  1.53218879 -0.13109342  1.17293157  2.58525291  2.80055813  1.22609361
```



```
  1.27726691  3.0268854 ], Cost: 0.0020
Current parameters: [ 2.12093206  1.8784875   1.07428959  0.72258575  1.87796023  1.64686183
  1.53737065 -0.12171581  1.17690721  2.56787053  2.79660091  1.22278002
  1.28716562  3.01237337], Cost: 0.0041
Current parameters: [ 2.12674287  1.87201149  1.06261282  0.73760839  1.86372723  1.64680256
  1.53659633 -0.12334171  1.17458276  2.57964642  2.79900752  1.22636849
  1.27719927  3.03012374], Cost: 0.0035
Current parameters: [ 2.13508884  1.8638911   1.07788885  0.72121581  1.85212047  1.64804721
  1.54939889 -0.12267052  1.16943548  2.58853504  2.81110027  1.22256417
  1.28085652  3.02119668], Cost: 0.0051
Current parameters: [ 2.12326629  1.87820659  1.07463368  0.73873063  1.86023499  1.65083812
  1.54586735 -0.13138241  1.17159858  2.58226051  2.79340516  1.23286802
  1.27924813  3.02242538], Cost: 0.0024
Current parameters: [ 2.11998003  1.87552649  1.06975639  0.73285801  1.86502389  1.65222087
  1.54416605 -0.12466996  1.17822231  2.58533416  2.80134272  1.22833097
  1.27570201  3.02500119], Cost: 0.0037
Current parameters: [ 2.12820873  1.87718019  1.06930895  0.73757275  1.86812671  1.65143816
  1.54093982 -0.13319201  1.17799806  2.59013613  2.7990265   1.21843223
  1.27644994  3.01997517], Cost: 0.0021
Current parameters: [ 2.12088255  1.87509404  1.06867641  0.73037001  1.86219277  1.65149617
  1.54405578 -0.12718014  1.17270631  2.58401308  2.79639981  1.22185798
  1.27517023  3.02527019], Cost: 0.0012
Current parameters: [ 2.12870939  1.86945756  1.07334713  0.7254591   1.85656645  1.649502
  1.54665722 -0.12608231  1.16927483  2.58580138  2.8021534   1.22067819
  1.27809917  3.02339234], Cost: 0.0014
Current parameters: [ 2.12066798  1.87629768  1.06833117  0.7302262   1.86090805  1.6531498
  1.54455051 -0.12649395  1.17472133  2.58530448  2.79527528  1.22045076
  1.28131256  3.02802681], Cost: 0.0011
Current parameters: [ 2.11671838  1.87504103  1.07057442  0.7294589   1.86390517  1.64846661
  1.54878093 -0.13467748  1.16974043  2.58485003  2.79481327  1.2149022
  1.28181944  3.03504727], Cost: 0.0024
Current parameters: [ 2.12184886  1.87678188  1.06985668  0.72807189  1.85858712  1.65180727
  1.54372696 -0.12898053  1.17988756  2.58278816  2.79428615  1.22035733
  1.27960396  3.0292348 ], Cost: 0.0019
Current parameters: [ 2.11995133  1.87792171  1.07064545  0.7252653   1.86746611  1.65127501
  1.54216837 -0.12422122  1.17329981  2.57777788  2.79344399  1.21807719
  1.28731517  3.0209578 ], Cost: 0.0019
Current parameters: [ 2.12066798  1.87629768  1.06833117  0.7302262   1.86090805  1.6531498
  1.54455051 -0.12649395  1.17472133  2.58530448  2.79527528  1.22045076
  1.28131256  3.02802681], Cost: 0.0011
```

```
   Return from subroutine COBYLA because the MAXFUN limit has been reached.

   NFVALS =  100   F = 1.056068E-03    MAXCV = 0.000000E+00
   X = 2.120668E+00   1.876298E+00   1.068331E+00   7.302262E-01   1.860908E+00
       1.653150E+00   1.544551E+00  -1.264940E-01   1.174721E+00   2.585304E+00
       2.795275E+00   1.220451E+00   1.281313E+00   3.028027E+00
Simulating for QAOA depth p = 8
Current parameters: [3.96690047 1.24263377 0.88214734 2.57544481 1.67029484 3.02212552
 0.06143719 3.02205418 1.27155496 1.75367447 0.24481127 1.28009033
 1.91103614 2.11260862 2.7236932  0.57896744], Cost: 1.7635
Current parameters: [3.96690047 2.24263377 0.88214734 2.57544481 1.67029484 3.02212552
 0.06143719 3.02205418 1.27155496 1.75367447 0.24481127 1.28009033
 1.91103614 2.11260862 2.7236932  0.57896744], Cost: 1.1194
Current parameters: [3.96690047 2.24263377 1.88214734 2.57544481 1.67029484 3.02212552
 0.06143719 3.02205418 1.27155496 1.75367447 0.24481127 1.28009033
 1.91103614 2.11260862 2.7236932  0.57896744], Cost: 1.9551
Current parameters: [3.96690047 2.24263377 0.88214734 3.57544481 1.67029484 3.02212552
 0.06143719 3.02205418 1.27155496 1.75367447 0.24481127 1.28009033
 1.91103614 2.11260862 2.7236932  0.57896744], Cost: 2.0463
Current parameters: [3.96690047 2.24263377 0.88214734 2.57544481 2.67029484 3.02212552
 0.06143719 3.02205418 1.27155496 1.75367447 0.24481127 1.28009033
 1.91103614 2.11260862 2.7236932  0.57896744], Cost: 2.1200
Current parameters: [3.96690047 2.24263377 0.88214734 2.57544481 1.67029484 4.02212552
 0.06143719 3.02205418 1.27155496 1.75367447 0.24481127 1.28009033
 1.91103614 2.11260862 2.7236932  0.57896744], Cost: 2.9918
Current parameters: [3.96690047 2.24263377 0.88214734 2.57544481 1.67029484 3.02212552
 1.06143719 3.02205418 1.27155496 1.75367447 0.24481127 1.28009033
 1.91103614 2.11260862 2.7236932  0.57896744], Cost: 1.5426
Current parameters: [3.96690047 2.24263377 0.88214734 2.57544481 1.67029484 3.02212552
 0.06143719 4.02205418 1.27155496 1.75367447 0.24481127 1.28009033
 1.91103614 2.11260862 2.7236932  0.57896744], Cost: 2.5368
Current parameters: [3.96690047 2.24263377 0.88214734 2.57544481 1.67029484 3.02212552
 0.06143719 3.02205418 2.27155496 1.75367447 0.24481127 1.28009033
 1.91103614 2.11260862 2.7236932  0.57896744], Cost: 0.7242
Current parameters: [3.96690047 2.24263377 0.88214734 2.57544481 1.67029484 3.02212552
 0.06143719 3.02205418 2.27155496 2.75367447 0.24481127 1.28009033
 1.91103614 2.11260862 2.7236932  0.57896744], Cost: 2.9327
Current parameters: [3.96690047 2.24263377 0.88214734 2.57544481 1.67029484 3.02212552
 0.06143719 3.02205418 2.27155496 1.75367447 1.24481127 1.28009033
 1.91103614 2.11260862 2.7236932  0.57896744], Cost: 3.6996
Current parameters: [3.96690047 2.24263377 0.88214734 2.57544481 1.67029484 3.02212552
```



```
 0.06143719 3.02205418 2.27155496 1.75367447 0.24481127 2.28009033
 1.91103614 2.11260862 2.7236932  0.57896744], Cost: 3.4865
Current parameters: [3.96690047 2.24263377 0.88214734 2.57544481 1.67029484 3.02212552
 0.06143719 3.02205418 2.27155496 1.75367447 0.24481127 1.28009033
 2.91103614 2.11260862 2.7236932  0.57896744], Cost: 3.3512
Current parameters: [3.96690047 2.24263377 0.88214734 2.57544481 1.67029484 3.02212552
 0.06143719 3.02205418 2.27155496 1.75367447 0.24481127 1.28009033
 1.91103614 3.11260862 2.7236932  0.57896744], Cost: 3.2973
Current parameters: [3.96690047 2.24263377 0.88214734 2.57544481 1.67029484 3.02212552
 0.06143719 3.02205418 2.27155496 1.75367447 0.24481127 1.28009033
 1.91103614 2.11260862 3.7236932  0.57896744], Cost: 3.2121
Current parameters: [3.96690047 2.24263377 0.88214734 2.57544481 1.67029484 3.02212552
 0.06143719 3.02205418 2.27155496 1.75367447 0.24481127 1.28009033
 1.91103614 2.11260862 2.7236932  1.57896744], Cost: 3.2339
Current parameters: [ 3.96742363  2.3285498   0.7706669   2.45180211  1.5368165   2.77235778
  0.00499227  2.83298022  2.32428192  1.45905994 -0.1521083   0.91159801
  1.56059854  1.76935943  2.39180572  0.24418099], Cost: 2.2476
Current parameters: [3.96716205 2.28559178 0.82640712 2.51362346 1.60355567 2.89724165
 0.03321473 2.9275172  2.29791844 1.60636721 0.04635149 1.09584417
 1.73581734 1.94098402 2.55774946 0.41157422], Cost: 2.5538
Current parameters: [4.21690013 2.24248797 0.88229314 2.57559061 1.67044063 3.02227132
 0.06158298 3.02219998 2.27170076 1.75367447 0.24481127 1.28009033
 1.91103614 2.11260862 2.7236932  0.57896744], Cost: 0.9054
Current parameters: [3.95529848 2.06698877 1.05493758 2.74687691 1.84062859 3.17947334
 0.24037319 3.18617962 2.46268209 1.72077513 0.20048762 1.23894113
 1.87190308 2.07427829 2.68663162 0.54158214], Cost: 1.3193
Current parameters: [3.96690047 2.01305254 0.84200996 2.53530744 1.63015746 3.03336856
 0.02129981 2.9819168  2.23141758 1.75367447 0.24481127 1.28009033
 1.91103614 2.11260862 2.7236932  0.57896744], Cost: 1.2714
Current parameters: [3.96177890e+00 2.34628521e+00 8.20070244e-01 2.51272288e+00
 1.60705143e+00 3.48509811e+00 2.27803313e-03 2.95586313e+00
 2.21818402e+00 1.73805423e+00 2.23766882e-01 1.26055314e+00
 1.89245619e+00 2.09440979e+00 2.70609677e+00 5.61217307e-01], Cost: 1.8830
Current parameters: [3.96690047 2.24263377 0.67051292 2.59685156 1.79630228 3.02212552
 0.08284393 3.04346092 2.29296171 1.75367447 0.24481127 1.28009033
 1.91103614 2.11260862 2.7236932  0.57896744], Cost: 0.5791
Current parameters: [ 3.96511504  2.24704772  0.84114733  2.45956371  2.1784615   3.01701022
 -0.05320189  2.90496371  2.15893371  1.7482291   0.23747499  1.27327948
  1.90455898  2.10626433  2.71755891  0.57277957], Cost: 1.5584
Current parameters: [3.96690047 2.24263377 0.67051292 2.63648903 1.79630228 3.02212552
 0.27030558 2.92991137 2.17941215 1.75367447 0.24481127 1.28009033
```



```
 1.91103614 2.11260862 2.7236932  0.57896744], Cost: 0.9242
Current parameters: [ 3.9651324   2.24700479  0.66891176  3.02419971  1.79471632  3.01705997
 -0.13364347  2.94084489  2.19477142  1.74828206  0.23754634  1.27334571
  1.90462198  2.10632603  2.71761857  0.57283975], Cost: 1.2809
Current parameters: [3.935024   2.27367433 0.70020109 2.59687157 1.78302917 3.00218497
 0.08250381 3.21438499 2.1214831  1.7457002  0.23683699 1.27211606
 1.90306186 2.10463434 2.71571893 0.57099317], Cost: 1.9873
Current parameters: [4.23374524 2.02017192 0.3761557  2.58578659 1.89542326 3.13317813
 0.10551506 3.04921768 2.42548598 1.75057651 0.21528971 1.25791103
 1.89352005 2.09694917 2.71096829 0.56549377], Cost: 0.6116
Current parameters: [3.95998332 2.25619635 0.66858614 2.59486595 1.79123238 3.00759549
 0.08639009 3.03022234 2.33048331 1.99580126 0.22550023 1.26212004
 1.89391738 2.0958289  2.70744934 0.56258686], Cost: 0.2713
Current parameters: [3.96050023 2.25569299 0.66810471 2.59486563 1.79144761 3.00791884
 0.08639561 3.03021633 2.33049831 1.99577565 0.3098328  1.16985929
 1.89404629 2.09595821 2.70757866 0.56271617], Cost: 0.1841
Current parameters: [3.95451157 2.27727579 0.65437269 2.59027807 1.7839019  2.9806318
 0.09468388 2.99955564 2.4173595  2.00939139 0.47685154 1.30842972
 1.85704376 2.05973752 2.67259434 0.52741639], Cost: 0.9938
Current parameters: [3.96064762 2.25554947 0.66796744 2.59486553 1.79150899 3.00801104
 0.08639718 3.03021462 2.33050259 1.99576835 0.30982297 1.16985016
 1.98134422 2.00649277 2.70761553 0.56275304], Cost: 0.1655
Current parameters: [3.95248659 2.27743254 0.65848384 2.59080843 1.78364091 2.98207785
 0.09369979 3.00312048 2.40727101 2.00795683 0.28065201 1.13119159
 2.1426207  2.16110582 2.67594664 0.53080504], Cost: 0.2500
Current parameters: [3.96070991 2.25548881 0.66790942 2.5948655  1.79153492 3.00805001
 0.08639785 3.03021389 2.3305044  1.99576527 0.30981881 1.1698463
 1.98134055 2.00648917 2.61878941 0.65070136], Cost: 0.1913
Current parameters: [3.95042645 2.28088859 0.65862508 2.59038709 1.78231803 2.97862213
 0.09444664 3.00031564 2.41522296 2.00928493 0.27769784 1.12724452
 1.97346732 1.99591632 2.86597988 0.71856869], Cost: 0.3811
Current parameters: [3.96791558 2.23229229 0.67041496 2.60343854 1.80260347 2.89821962
 0.09847006 3.03537975 2.34826149 1.98042388 0.28915012 1.15065789
 1.9630923  1.98861524 2.69032976 0.54531629], Cost: 0.5160
Current parameters: [3.93008807 2.34457272 0.63960831 2.54941444 1.73146622 3.09452625
 0.06174814 2.95240561 2.39119962 1.96721504 0.22528005 1.07966282
 1.92545386 1.94912994 2.65141194 0.50221094], Cost: 1.5518
Current parameters: [3.98003759 2.27701298 0.62553119 2.54273549 1.7944763  2.97336142
 0.03270606 2.97960461 2.27427528 2.00261258 0.31904386 1.17841065
 1.98948528 2.01446683 2.71532565 0.57053051], Cost: 0.1802
Current parameters: [3.96082457 2.23072349 0.77284898 2.56098656 2.00345778 3.04731999
```



```
 0.05634859 2.98638565 2.33317175 2.01117382 0.30689759 1.16112131
 1.98839919 2.012057   2.71261552 0.5658192 ], Cost: 0.1354
Current parameters: [3.93242514 2.25929331 0.80089088 2.65475853 1.99594718 3.03487382
 0.00469176 2.9515362  2.3154733  2.01109433 0.309493   1.16297344
 1.98977927 2.01324913 2.71351058 0.56679006], Cost: 0.2903
Current parameters: [4.08556216 2.11427778 0.64595471 2.65446781 2.05027707 3.11009423
 0.02540539 2.9922621  2.33780264 2.03940398 0.30121119 1.14929388
 2.0004821  2.02255387 2.72316518 0.57338746], Cost: 0.1920
Current parameters: [3.85881868 2.1866352  0.71941591 2.55782679 2.02286321 3.04942251
 0.05318882 2.98322588 2.33001198 2.01117382 0.30689759 1.16112131
 1.98839919 2.012057   2.71261552 0.5658192 ], Cost: 0.0369
Current parameters: [3.85537864 2.01400717 0.8295627  2.51070042 1.91721741 3.06899735
 0.00786863 2.92677725 2.31339846 2.03250842 0.31504195 1.16314407
 2.00401999 2.02610898 2.72579726 0.57735689], Cost: 0.1152
Current parameters: [3.84394396 2.18885429 0.73402895 2.58200676 1.99824744 3.09662846
 0.07195028 3.05191128 2.30074097 2.02412828 0.3260441  1.17854724
 2.02283931 1.97578559 2.74894615 0.53146694], Cost: 0.1712
Current parameters: [3.84679359 2.18415758 0.71744498 2.49744206 2.0827776  2.99378636
 0.00991725 3.08364795 2.22439091 2.07881157 0.30095089 1.12869011
 1.99278584 2.12691057 2.70215142 0.66541347], Cost: 0.4605
Current parameters: [3.85923482 2.1827049  0.71738936 2.57342125 2.02914147 3.05092219
 0.14617292 2.92602353 2.27543093 2.02285977 0.30968859 1.16017972
 1.994613   2.02336461 2.71712034 0.57536766], Cost: 0.0472
Current parameters: [3.86091975 2.18167126 0.72103624 2.55867603 2.024674   3.05509786
 0.05166397 2.98889435 2.31394943 1.9546429  0.29751057 1.15264671
 1.99550219 2.01901508 2.71934455 0.57260668], Cost: 0.1735
Current parameters: [3.85840511 2.18378057 0.70775574 2.54585037 2.03712099 3.04319058
 0.06016436 2.9664904  2.36683276 2.00128163 0.39529898 1.23240693
 1.99069726 2.02837105 2.71111312 0.57776378], Cost: 0.3264
Current parameters: [3.86196556 2.18201713 0.72031252 2.5553498  2.02717515 3.05058288
 0.04839518 2.9857699  2.30967632 2.00701659 0.31321969 1.16684209
 1.98579461 2.01823571 2.66817912 0.52982448], Cost: 0.1507
Current parameters: [3.84188186 2.21348871 0.69734374 2.52301764 2.01148258 3.11109269
 0.05295158 2.94312305 2.36528531 2.05003033 0.27290671 1.11320335
 1.96409743 1.98896325 2.71789026 0.57027833], Cost: 0.0915
Current parameters: [3.86234905 2.18095607 0.7208916  2.55569179 2.02869299 3.0483011
 0.0484489  2.98644409 2.30904225 2.00801376 0.31478527 1.16829277
 1.94398226 1.97905022 2.71463974 0.57834758], Cost: 0.1250
Current parameters: [3.85580796 2.15899304 0.71602351 2.56687641 2.02577642 2.96860628
 0.08676767 2.99162875 2.3775373  2.02936349 0.27939566 1.12351796
 1.99883507 1.97920054 2.72876674 0.53733516], Cost: 0.1107
```

```
Current parameters: [3.86229017 2.18951297 0.71653576 2.54672591 2.03309033 3.04482456
 0.04197672 2.97194803 2.31983426 2.01313814 0.30273416 1.16855145
 1.97421183 2.03015711 2.7508575  0.53111436], Cost: 0.0357
Current parameters: [3.859421   2.18755568 0.7147125  2.5455558  2.0326011  3.04618424
 0.04802    2.97140772 2.32928521 2.02366634 0.21448032 1.2541383
 1.98032226 2.02723459 2.74354569 0.54360392], Cost: 0.0358
Current parameters: [3.87585741 2.18650743 0.70367215 2.53591582 2.05205985 3.04166189
 0.02203468 2.95440064 2.3040072  2.01803586 0.3048279  1.1696389
 2.00924232 2.00137175 2.75413921 0.5340849 ], Cost: 0.0380
Current parameters: [3.91715742 2.13216674 0.65747545 2.60349653 2.04618083 3.07803142
 0.03568061 2.98138971 2.33259937 2.02961384 0.2965175  1.16026874
 1.96231251 2.05229658 2.75576827 0.53493737], Cost: 0.0433
Current parameters: [3.86589434 2.19999817 0.68328884 2.54266159 1.98636978 3.02816407
 0.03660948 2.97047913 2.30792679 2.01257906 0.30525085 1.17210781
 1.97534213 2.03124425 2.75118204 0.53365446], Cost: 0.0123
Current parameters: [3.89400164 2.18929897 0.67394076 2.50449104 1.97800195 3.02963337
 0.0681753  2.94222951 2.27260114 2.00409354 0.27764182 1.16264011
 1.93005149 2.01393288 2.7910469  0.46156026], Cost: 0.3344
Current parameters: [3.86397248 2.15814333 0.71081604 2.53230142 1.95951585 3.03240301
 0.02427774 2.95613721 2.30628761 2.02115608 0.30686997 1.17190281
 1.98135996 2.03618765 2.75382742 0.54015213], Cost: 0.0069
Current parameters: [3.8560197  2.16575468 0.70692933 2.50538274 1.97907837 3.00718879
 0.01058845 2.99705235 2.26802052 2.08314885 0.29094269 1.14792008
 1.99090196 2.09190982 2.76205457 0.58999373], Cost: 0.1417
Current parameters: [3.84161851 2.17077184 0.74385332 2.5526737  1.97276827 3.03150804
 0.01682347 2.94841449 2.3115232  2.0224262  0.3130113  1.16335882
 1.9620897  2.05717198 2.76994547 0.52473368], Cost: 0.0123
Current parameters: [ 3.85648438  2.17774674  0.6904422   2.61232141  1.92792258  3.01657673
 -0.01949519  2.90828036  2.28803618  2.03928283  0.28646557  1.17697991
  2.00699821  2.00916923  2.74403301  0.55816136], Cost: 0.0911
Current parameters: [3.85885907 2.16022769 0.70075434 2.52623426 1.96109542 3.03010906
 0.02866455 2.94154778 2.33896111 2.02634916 0.34141675 1.20095821
 1.98905886 2.0470672  2.76133574 0.55162481], Cost: 0.1165
Current parameters: [3.87001984 2.15744987 0.70757784 2.52446162 1.96457776 3.02146098
 0.00847007 2.94794052 2.31444212 2.01285306 0.30147456 1.16838826
 1.97041663 2.04091976 2.74500768 0.54303438], Cost: 0.0261
Current parameters: [ 3.85990811  2.16039269  0.71199878  2.53052852  1.94918328  3.04292138
 -0.01135617  2.99479746  2.32266625  2.02205466  0.30584116  1.17478086
  1.98571672  2.0202158   2.76347021  0.52425031], Cost: 0.0071
Current parameters: [3.87024108 2.15057202 0.71410837 2.53941588 1.96294453 3.01469064
 0.02299283 2.96461483 2.29506914 2.01337776 0.31424744 1.18458313
```



```
 1.98148413 2.03716539 2.7562136  0.53679899], Cost: 0.0041
Current parameters: [3.88222597 2.14525593 0.71060342 2.52695314 1.95323653 3.01980276
 0.03608449 2.95721275 2.27361291 2.01195715 0.2982797  1.17953074
 1.96437475 2.02205217 2.77824477 0.49844811], Cost: 0.0807
Current parameters: [3.84656112 2.13888994 0.70008387 2.53932421 1.96803482 3.01398469
 0.02047196 2.96331818 2.2902285  2.00903923 0.313425   1.1850261
 1.9795903  2.03552203 2.75545629 0.53451389], Cost: 0.0094
Current parameters: [3.87015984e+00 2.16202226e+00 7.07593523e-01 2.58050790e+00
 1.94544823e+00 3.00847533e+00 2.31620750e-03 2.94448849e+00
 2.28339861e+00 2.02338676e+00 3.02200360e-01 1.18541555e+00
 1.99524273e+00 2.02211153e+00 2.75237107e+00 5.44141054e-01], Cost: 0.0241
Current parameters: [3.87258932 2.1562819  0.71258418 2.53561411 1.96337183 3.03199049
 0.01262893 2.96238487 2.27907962 2.00885728 0.31971194 1.194616
 1.97656084 2.04486384 2.75444454 0.54086905], Cost: 0.0069
Current parameters: [3.86746632 2.15403162 0.71274394 2.52896698 1.96907687 3.00017815
 0.01786371 2.98787639 2.27456374 2.0476006  0.30228461 1.16994019
 1.98825143 2.05966111 2.76421107 0.55667113], Cost: 0.0365
Current parameters: [3.88175924 2.13674078 0.70414741 2.55370792 1.96876687 3.02397963
 0.02325962 2.96791086 2.30052478 2.01907313 0.3146828  1.18082219
 1.97648739 2.04716527 2.75559626 0.5327857 ], Cost: 0.0074
Current parameters: [3.86521706 2.15439243 0.72194234 2.53635718 1.96077431 3.01310361
 0.02488094 2.96759469 2.29027026 2.02945743 0.32090024 1.18782516
 1.99286001 2.045509   2.7089086  0.50513089], Cost: 0.0164
Current parameters: [3.86773949 2.14875611 0.7196316  2.54102097 1.96663845 3.01663212
 0.02719437 2.96718996 2.29922183 2.0221625  0.29887555 1.2034073
 1.96965618 2.0320693  2.75196255 0.54161808], Cost: 0.0062
Current parameters: [3.87120665 2.14815876 0.71706163 2.54177337 1.96340057 3.01314995
 0.02277375 2.9691321  2.28748358 2.03081122 0.34653229 1.16876309
 1.94959012 2.00698372 2.74544344 0.54960409], Cost: 0.0160
Current parameters: [3.86460898 2.14471734 0.73340532 2.55431192 1.95897146 3.02767963
 0.03783084 2.98817666 2.29231707 1.97890672 0.304074   1.16963574
 1.98637019 2.03628502 2.73775502 0.55675003], Cost: 0.0732
Current parameters: [3.86736596 2.1529586  0.71017266 2.53812654 1.95979588 3.01402171
 0.02651524 2.96099412 2.30973947 2.01849589 0.33167817 1.19975235
 1.98901132 2.04164625 2.76244885 0.54111238], Cost: 0.0305
Current parameters: [3.87028388 2.13986312 0.72173283 2.53659308 1.95982894 3.01638797
 0.01930224 2.9603667  2.29447845 2.01570824 0.31461398 1.18440931
 1.98463817 2.03684085 2.75699674 0.53837743], Cost: 0.0069
Current parameters: [3.87379646 2.15259024 0.71692885 2.53890941 1.98862906 3.01872317
 0.02024478 2.96179908 2.29422892 2.01457825 0.31453108 1.18445826
 1.99310513 2.02618809 2.75747437 0.53589415], Cost: 0.0053
```



```
Current parameters: [3.86957039 2.1509155  0.71616019 2.54138195 1.9662533  3.01229092
 0.03130438 2.95494025 2.29135851 2.01339189 0.31501074 1.1833626
 1.97929546 2.04236741 2.75516204 0.53950327], Cost: 0.0042
Current parameters: [3.85896767 2.15714666 0.73011311 2.54898687 1.96795575 3.01564574
 0.01443568 2.96631903 2.30000503 2.01420887 0.31720522 1.18103506
 1.97305627 2.04500557 2.76541422 0.52716584], Cost: 0.0040
Current parameters: [3.86184516 2.15768069 0.72654662 2.54473553 1.96700062 3.01387694
 0.01372702 2.96126393 2.29725552 2.01996729 0.31631854 1.18260482
 1.97011569 2.04304457 2.77169856 0.51829288], Cost: 0.0030
Current parameters: [3.86531515 2.15709584 0.73286079 2.54590092 1.96348264 3.02160946
 0.02805665 2.96761391 2.28919836 2.00523149 0.30780068 1.17688449
 1.9678215  2.03497854 2.77742365 0.50758371], Cost: 0.0323
Current parameters: [3.86151281 2.15578254 0.72774074 2.53486079 1.97141348 3.01336078
 0.01736941 2.96849671 2.29692652 2.02235992 0.31730021 1.18057415
 1.96816241 2.04911223 2.77356266 0.51936248], Cost: 0.0024
Current parameters: [3.86040692 2.16199897 0.72570589 2.54209943 1.96963868 3.00575323
 0.01117692 2.97385572 2.28387904 2.0386136  0.30782126 1.17409545
 1.97596974 2.05382306 2.77776668 0.52858219], Cost: 0.0138
Current parameters: [3.86049784 2.15684265 0.72844748 2.53386649 1.97100802 3.01334964
 0.01761157 2.96810083 2.29756454 2.02178691 0.3124257  1.18400286
 1.97588386 2.05613303 2.76754847 0.51169544], Cost: 0.0029
Current parameters: [3.86006349 2.15716928 0.73188049 2.53502704 1.97087148 3.01236145
 0.01887628 2.97195305 2.29161368 2.03452273 0.33042428 1.17600406
 1.96215234 2.04156192 2.75315588 0.51155146], Cost: 0.0057
Current parameters: [3.86646548 2.16043047 0.72370519 2.53176092 1.96847618 3.00537078
 0.01293193 2.97318731 2.29936643 2.0170392  0.31561677 1.17808418
 1.96648436 2.04724625 2.77080857 0.51848327], Cost: 0.0039
Current parameters: [3.86241889 2.1496992  0.73168079 2.530398   1.98396578 3.01095031
 0.00521805 2.95148012 2.30115355 2.02127961 0.31259063 1.1781159
 1.95671292 2.05700874 2.76676583 0.52333777], Cost: 0.0070
Current parameters: [3.86096791 2.1586353  0.72526511 2.53363732 1.9711161  3.01118617
 0.01801042 2.96653266 2.30476135 2.02500755 0.32618266 1.1876145
 1.97082045 2.05142296 2.77599007 0.52132168], Cost: 0.0086
Current parameters: [3.86579237 2.15626755 0.73140768 2.53452795 1.97074263 3.01644473
 0.01460754 2.97004663 2.29892854 2.02417038 0.3167665  1.18111307
 1.96876151 2.04848593 2.77412954 0.5193047 ], Cost: 0.0028
Current parameters: [3.85398809 2.15032139 0.72329411 2.53338691 1.97224309 3.01693101
 0.00905887 2.97360337 2.2986236  2.0226594  0.31637596 1.18213027
 1.96870258 2.04534217 2.77424052 0.51692067], Cost: 0.0021
Current parameters: [3.85092331 2.1529789  0.72701884 2.52970084 1.97130456 3.01608561
 0.00830498 2.97218429 2.29729122 2.02180048 0.31586758 1.18328519
```



```
 1.96983671 2.04283907 2.77455991 0.51800806], Cost: 0.0020
Current parameters: [3.85064362 2.15022399 0.72971994 2.52939452 1.97215978 3.02126386
 0.01437377 2.97383383 2.29500067 2.01377445 0.3125888  1.18124618
 1.96657379 2.03905318 2.77779906 0.51153174], Cost: 0.0107
Current parameters: [3.85215958 2.15037382 0.72933327 2.53133593 1.97272391 3.01273495
 0.00697462 2.97372376 2.29418111 2.02063829 0.31732597 1.18690887
 1.96993957 2.04307981 2.77538985 0.51748818], Cost: 0.0022
Current parameters: [3.84902612 2.15567299 0.72572087 2.53306237 1.97116699 3.01346491
 0.00512873 2.97435442 2.29079663 2.02999359 0.3102212  1.17917518
 1.97307556 2.04498577 2.77730657 0.52223775], Cost: 0.0056
Current parameters: [3.85009549 2.15024248 0.72931038 2.53040045 1.97230718 3.01243532
 0.00956058 2.9716189  2.30132996 2.02346078 0.31408968 1.1813563
 1.9707279  2.04100239 2.77499076 0.51723835], Cost: 0.0021
Current parameters: [3.85127100e+00 2.15054668e+00 7.28123709e-01 2.52725833e+00
 1.97726482e+00 3.01596644e+00 1.53405211e-03 2.96398341e+00
 2.29785169e+00 2.02080368e+00 3.13583388e-01 1.18223967e+00
 1.96380427e+00 2.04717289e+00 2.77075278e+00 5.20242681e-01], Cost: 0.0030
Current parameters: [3.85122889 2.15200856 0.72763509 2.52920952 1.97106621 3.01608673
 0.00633626 2.97087303 2.29638493 2.02021159 0.31960064 1.1787682
 1.97319025 2.04385368 2.77604746 0.51676161], Cost: 0.0041
Current parameters: [3.85285953 2.14430102 0.72584057 2.52547588 1.9609048  3.01579334
 0.009539   2.97011977 2.29563799 2.02290529 0.31438078 1.18467501
 1.97015018 2.04346376 2.77200933 0.52209854], Cost: 0.0037
Current parameters: [3.85092331 2.1529789  0.72701884 2.52970084 1.97130456 3.01608561
 0.00830498 2.97218429 2.29729122 2.02180048 0.31586758 1.18328519
 1.96983671 2.04283907 2.77455991 0.51800806], Cost: 0.0020

   Return from subroutine COBYLA because the MAXFUN limit has been reached.

   NFVALS =  100   F = 2.034704E-03    MAXCV = 0.000000E+00
   X = 3.850923E+00   2.152979E+00   7.270188E-01   2.529701E+00   1.971305E+00
       3.016086E+00   8.304476E-03   2.972184E+00   2.297291E+00   2.021800E+00
       3.158676E-01   1.183285E+00   1.969837E+00   2.042839E+00   2.774560E+00
       5.180081E-01
Simulating for QAOA depth p = 9
Current parameters: [1.64533456 1.54148185 0.24265341 0.96489461 0.76217528 1.58703478
 1.54515533 0.5402221  2.37119029 0.88085924 0.00841543 1.43786665
 2.67821659 2.65141234 2.68285149 1.96131711 2.69581729 2.23172068], Cost: 1.5547
Current parameters: [1.64533456 2.54148185 0.24265341 0.96489461 0.76217528 1.58703478
 1.54515533 0.5402221  2.37119029 0.88085924 0.00841543 1.43786665
 2.67821659 2.65141234 2.68285149 1.96131711 2.69581729 2.23172068], Cost: 1.4989
```

```
Current parameters: [1.64533456 2.54148185 1.24265341 0.96489461 0.76217528 1.58703478
 1.54515533 0.5402221  2.37119029 0.88085924 0.00841543 1.43786665
 2.67821659 2.65141234 2.68285149 1.96131711 2.69581729 2.23172068], Cost: 1.6566
Current parameters: [1.64533456 2.54148185 0.24265341 1.96489461 0.76217528 1.58703478
 1.54515533 0.5402221  2.37119029 0.88085924 0.00841543 1.43786665
 2.67821659 2.65141234 2.68285149 1.96131711 2.69581729 2.23172068], Cost: 2.0804
Current parameters: [1.64533456 2.54148185 0.24265341 0.96489461 1.76217528 1.58703478
 1.54515533 0.5402221  2.37119029 0.88085924 0.00841543 1.43786665
 2.67821659 2.65141234 2.68285149 1.96131711 2.69581729 2.23172068], Cost: 2.7416
Current parameters: [1.64533456 2.54148185 0.24265341 0.96489461 0.76217528 2.58703478
 1.54515533 0.5402221  2.37119029 0.88085924 0.00841543 1.43786665
 2.67821659 2.65141234 2.68285149 1.96131711 2.69581729 2.23172068], Cost: 1.1515
Current parameters: [1.64533456 2.54148185 0.24265341 0.96489461 0.76217528 2.58703478
 2.54515533 0.5402221  2.37119029 0.88085924 0.00841543 1.43786665
 2.67821659 2.65141234 2.68285149 1.96131711 2.69581729 2.23172068], Cost: 1.9733
Current parameters: [1.64533456 2.54148185 0.24265341 0.96489461 0.76217528 2.58703478
 1.54515533 1.5402221  2.37119029 0.88085924 0.00841543 1.43786665
 2.67821659 2.65141234 2.68285149 1.96131711 2.69581729 2.23172068], Cost: 2.1997
Current parameters: [1.64533456 2.54148185 0.24265341 0.96489461 0.76217528 2.58703478
 1.54515533 0.5402221  3.37119029 0.88085924 0.00841543 1.43786665
 2.67821659 2.65141234 2.68285149 1.96131711 2.69581729 2.23172068], Cost: 1.6981
Current parameters: [1.64533456 2.54148185 0.24265341 0.96489461 0.76217528 2.58703478
 1.54515533 0.5402221  2.37119029 1.88085924 0.00841543 1.43786665
 2.67821659 2.65141234 2.68285149 1.96131711 2.69581729 2.23172068], Cost: 2.7760
Current parameters: [1.64533456 2.54148185 0.24265341 0.96489461 0.76217528 2.58703478
 1.54515533 0.5402221  2.37119029 0.88085924 1.00841543 1.43786665
 2.67821659 2.65141234 2.68285149 1.96131711 2.69581729 2.23172068], Cost: 2.2894
Current parameters: [1.64533456 2.54148185 0.24265341 0.96489461 0.76217528 2.58703478
 1.54515533 0.5402221  2.37119029 0.88085924 0.00841543 2.43786665
 2.67821659 2.65141234 2.68285149 1.96131711 2.69581729 2.23172068], Cost: 2.7020
Current parameters: [1.64533456 2.54148185 0.24265341 0.96489461 0.76217528 2.58703478
 1.54515533 0.5402221  2.37119029 0.88085924 0.00841543 1.43786665
 3.67821659 2.65141234 2.68285149 1.96131711 2.69581729 2.23172068], Cost: 2.7706
Current parameters: [1.64533456 2.54148185 0.24265341 0.96489461 0.76217528 2.58703478
 1.54515533 0.5402221  2.37119029 0.88085924 0.00841543 1.43786665
 2.67821659 3.65141234 2.68285149 1.96131711 2.69581729 2.23172068], Cost: 2.5542
Current parameters: [1.64533456 2.54148185 0.24265341 0.96489461 0.76217528 2.58703478
 1.54515533 0.5402221  2.37119029 0.88085924 0.00841543 1.43786665
 2.67821659 2.65141234 3.68285149 1.96131711 2.69581729 2.23172068], Cost: 2.3429
Current parameters: [1.64533456 2.54148185 0.24265341 0.96489461 0.76217528 2.58703478
 1.54515533 0.5402221  2.37119029 0.88085924 0.00841543 1.43786665
```



```
 2.67821659 2.65141234 2.68285149 2.96131711 2.69581729 2.23172068], Cost: 1.0898
Current parameters: [1.64533456 2.54148185 0.24265341 0.96489461 0.76217528 2.58703478
 1.54515533 0.5402221  2.37119029 0.88085924 0.00841543 1.43786665
 2.67821659 2.65141234 2.68285149 2.96131711 3.69581729 2.23172068], Cost: 3.3448
Current parameters: [1.64533456 2.54148185 0.24265341 0.96489461 0.76217528 2.58703478
 1.54515533 0.5402221  2.37119029 0.88085924 0.00841543 1.43786665
 2.67821659 2.65141234 2.68285149 2.96131711 2.69581729 3.23172068], Cost: 2.8161
Current parameters: [ 1.78979874  2.55263061  0.21114768  0.8486974   0.51385791  2.65646491
  1.38092487  0.33074703  2.26195716  0.55622629 -0.2189757   1.12801528
  2.35467261  2.37110274  2.4447706   2.97364364  2.24519791  1.88675923], Cost: 3.4445
Current parameters: [ 1.66209262  2.54277513  0.2389987   0.95141555  0.73337008  2.59508878
  1.52610434  0.51592267  2.35851908  1.36867249 -0.01796229  1.40192341
  2.64068499  2.61889599  2.65523374  2.962747    2.64354475  2.19170462], Cost: 2.0616
Current parameters: [1.62609846 2.7907407  0.24265341 0.96489461 0.76217528 2.58703478
 1.54515533 0.5402221  2.37119029 0.88085924 0.00841543 1.43786665
 2.67821659 2.65141234 2.68285149 2.96131711 2.69581729 2.23172068], Cost: 0.8558
Current parameters: [ 1.12934117  2.76185768  0.24106568  0.95903884  0.74966131  2.59053371
  1.53687893  0.5296656   2.36568548  0.86747021 -0.00304396  1.42225167
  2.66191158  2.63728612  2.67085339  2.9619383   2.6731083   2.21433633], Cost: 1.3878
Current parameters: [1.62609846e+00 2.79074070e+00 1.36551178e-03 1.03031745e+00
 7.62175275e-01 2.58703478e+00 1.54515533e+00 5.40222105e-01
 2.37119029e+00 8.80859243e-01 8.41543149e-03 1.43786665e+00
 2.67821659e+00 2.65141234e+00 2.68285149e+00 2.96131711e+00
 2.69581729e+00 2.23172068e+00], Cost: 1.0003
Current parameters: [ 1.63237526e+00  2.79903979e+00  3.77686172e-01  1.44452399e+00
  7.51830460e-01  2.58992721e+00  1.53831355e+00  5.31495446e-01
  2.36663967e+00  8.69791044e-01 -1.05760400e-03  1.42495835e+00
  2.66473786e+00  2.63973474e+00  2.67293312e+00  2.96183063e+00
  2.67704464e+00  2.21734970e+00], Cost: 1.6408
Current parameters: [1.62609846 2.7907407  0.24265341 0.96489461 0.82949399 2.82780062
 1.54515533 0.5402221  2.37119029 0.88085924 0.00841543 1.43786665
 2.67821659 2.65141234 2.68285149 2.96131711 2.69581729 2.23172068], Cost: 0.8320
Current parameters: [ 1.63967797  2.80869533  0.24619849  0.93818592  1.30347757  2.69705375
  1.5303535   0.52134245  2.36134528  0.85691379 -0.01207897  1.40994024
  2.64905609  2.62614848  2.66139364  2.96242808  2.65520368  2.20062985], Cost: 0.8395
Current parameters: [1.62609846 2.7907407  0.24265341 0.96489461 0.82949399 2.82780062
 1.74189774 0.38597417 2.37119029 0.88085924 0.00841543 1.43786665
 2.67821659 2.65141234 2.68285149 2.96131711 2.69581729 2.23172068], Cost: 0.7617
Current parameters: [ 1.64101637  2.81046495  0.24654789  0.93555349  0.83625425  2.82786596
  2.04895757  0.76861368  2.36037495  0.85455371 -0.01409891  1.40718779
  2.64618201  2.62365845  2.65927874  2.96253758  2.65120077  2.19756551], Cost: 1.8210
```



```
Current parameters: [ 1.62609846  2.7907407   0.24265341  0.96489461  0.82949399  2.82780062
  1.74189774  0.38597417  2.59653803  0.88085924 -0.0998361   1.43786665
  2.67821659  2.65141234  2.68285149  2.96131711  2.69581729  2.23172068], Cost: 1.3915
Current parameters: [1.63992075 2.80901633 0.24626187 0.9377084  0.83575775 2.82786116
 1.72675352 0.35830615 2.54143559 0.85648568 0.46947081 1.40944095
 2.64853474 2.6256968  2.66101    2.96244794 2.65447756 2.20007399], Cost: 1.6694
Current parameters: [1.62609846 2.7907407  0.24265341 0.96489461 0.82949399 2.82780062
 1.74189774 0.38597417 2.37119029 0.88085924 0.00841543 1.61842268
 2.50530182 2.65141234 2.68285149 2.96131711 2.69581729 2.23172068], Cost: 0.7324
Current parameters: [ 1.65162354  2.82448959  0.24931701  0.9146909   0.84106104  2.82791242
  1.7139315   0.33488071  2.2695186   0.83584953 -0.0062796   1.94304257
  2.83851605  2.60392445  2.64251766  2.96340537  2.61947683  2.17327997], Cost: 2.6804
Current parameters: [1.62609846 2.7907407  0.24265341 0.96489461 0.82949399 2.82780062
 1.74189774 0.38597417 2.37119029 0.88085924 0.00841543 1.61842268
 2.50530182 2.8132525  2.49230554 2.96131711 2.69581729 2.23172068], Cost: 0.5709
Current parameters: [ 1.64480483e+00  2.81547399e+00  2.47536910e-01  9.28102206e-01
  8.37971041e-01  2.82788255e+00  1.72140234e+00  3.48529706e-01
  2.29667895e+00  8.47873326e-01 -2.35400014e-03  1.56033703e+00
  2.44043469e+00  3.18613130e+00  2.78798066e+00  2.96284752e+00
  2.63987025e+00  2.18889169e+00], Cost: 1.2689
Current parameters: [1.62665999 2.79078404 0.24253095 0.96444296 0.82852879 2.82807049
 1.74125938 0.38515995 2.3707657  0.87959741 0.00753157 1.61721831
 2.50404422 2.81216295 2.49138013 3.21129662 2.69581729 2.23172068], Cost: 0.4133
Current parameters: [ 1.7079509   2.83271286  0.23852457  0.86576556  0.75100338  2.85350089
  1.6500254   0.25148762  2.2168806   0.7107797  -0.09183237  1.41536151
  2.2868148   2.68246541  2.34902655  3.22924153  2.61016506  2.16615155], Cost: 2.8350
Current parameters: [1.62665999 2.79078404 0.24253095 0.96444296 0.82852879 2.82807049
 1.74125938 0.38515995 2.3707657  0.87959741 0.00753157 1.61721831
 2.50404422 2.81216295 2.49138013 3.21129662 2.84778269 2.03320993], Cost: 0.4940
Current parameters: [1.61980811 2.81256876 0.25261269 0.94911933 0.87923453 2.8162424
 1.74978693 0.38520783 2.3181098  0.90366541 0.03620887 1.6147037
 2.49738051 2.84300217 2.49742543 3.2267379  3.08084513 2.53612308], Cost: 3.3437
Current parameters: [ 1.62618533e+00  2.79074741e+00  2.42634469e-01  9.64824736e-01
  8.29344666e-01  2.82784237e+00  1.78286833e+00  3.00115970e-01
  2.27237511e+00  1.06874019e+00 -4.86748667e-04  1.54519033e+00
  2.55203907e+00  2.80267976e+00  2.48332558e+00  3.21171365e+00
  2.68057232e+00  2.22005024e+00], Cost: 0.5683
Current parameters: [1.6479855  2.81354064 0.24600472 0.92757832 0.82870267 2.83024461
 1.60711964 0.57574965 2.56584184 1.16529247 0.0144967  1.75189966
 2.30907869 2.81540008 2.47506754 3.22704634 2.58824191 2.15850158], Cost: 1.1700
Current parameters: [1.41093324 2.77413572 0.24269012 0.96502998 0.82978328 2.82771974
```



```
 1.75338929 0.362629   2.34414664 0.87802464 0.00626853 1.63422364
 2.42645971 2.75263539 2.55810311 3.21136959 2.69314973 2.22967859], Cost: 0.9027
Current parameters: [1.41352104 2.78981739 0.24557645 0.93924426 0.83258106 2.82856754
 1.67600322 0.46452897 2.44216652 0.85133244 0.00971934 1.53600074
 2.73963015 3.02467558 2.22689894 3.22295772 2.6128745  2.17492338], Cost: 0.2491
Current parameters: [1.47469833 2.79453863 0.30330817 1.1521663  0.83919872 2.82671723
 1.67744051 0.47624757 2.45214535 0.79423554 0.01640668 1.55036235
 2.74262267 3.03145889 2.23555116 3.27641731 2.56368957 2.20428607], Cost: 0.3252
Current parameters: [ 1.1982643   2.80101442  0.3174297   1.13881022  0.83812197  2.82994627
  1.63804674  0.43777658  2.37631717  1.05404479 -0.00510379  1.45478334
  2.70839058  3.041106    2.13410702  3.01611422  2.6912005   1.96198939], Cost: 1.1229
Current parameters: [1.41863191 2.79575821 0.2456483  0.92645788 0.83455518 2.82859955
 1.84793829 0.62804706 2.38791978 0.84461701 0.00681992 1.50137188
 2.73837962 3.01498912 2.22405287 3.22095974 2.58330094 2.14674706], Cost: 0.6906
Current parameters: [1.40995754 2.78954238 0.24713419 0.94498939 0.71430952 2.86163655
 1.6805254  0.46819912 2.44582456 0.85706829 0.02032356 1.54198885
 2.74691075 3.03034955 2.2324836  3.2226916  2.62260301 2.18237082], Cost: 0.2341
Current parameters: [1.41515714 2.79494294 0.24693978 0.93250845 0.72707757 2.85859294
 1.70591301 0.42615633 2.4933398  0.85006099 0.2528108  1.50159165
 2.74802264 3.01626514 2.23418067 3.22093498 2.59432809 2.15573482], Cost: 0.6436
Current parameters: [1.40215593 2.91070336 0.24646118 0.94250723 0.7148986  2.86147184
 1.68234832 0.46274963 2.44013535 0.85459014 0.01955837 1.56302104
 2.76223135 3.02165611 2.23741995 3.22280658 2.61839985 2.17915318], Cost: 0.1492
Current parameters: [1.41372366 2.86549785 0.24976625 0.91900026 0.71476186 2.86310729
 1.66829331 0.42922432 2.38495602 0.84766916 0.01044455 1.73710856
 2.8897651  2.99491008 2.25440899 3.21681088 2.55692767 2.11695055], Cost: 1.3215
Current parameters: [1.40305752 2.91109569 0.36597459 0.91057097 0.71691863 2.86090704
 1.68340117 0.46393327 2.44069604 0.85502927 0.02095249 1.56456386
 2.76395966 3.02125408 2.23710096 3.22441224 2.63121254 2.19093955], Cost: 0.0834
Current parameters: [1.40978518 2.93682204 0.34399861 0.92492467 0.7431524  2.85530706
 1.66736152 0.49848718 2.44414739 0.87821398 0.04048543 1.57664442
 2.78312739 3.06911019 2.20320792 3.24225409 2.80783691 2.34105731], Cost: 0.7917
Current parameters: [1.3957804  2.91321342 0.36744021 0.90967155 0.72494413 2.85866309
 1.68763637 0.4637158  2.4384318  0.86537476 0.02424619 1.56445073
 2.76769483 3.11476509 2.31757672 3.22052293 2.62689651 2.18167705], Cost: 0.4056
Current parameters: [ 1.46730875  2.95786281  0.37604027  0.86262202  0.67882334  2.87360476
  1.61219462  0.41869251  2.39259201  0.76596154 -0.03713048  1.44983313
  2.6652124   3.0345616   2.16402439  3.24567574  2.58292303  2.1761587 ], Cost: 1.7292
Current parameters: [1.40305752 2.91109569 0.36597459 0.91057097 0.75054005 2.9811543
 1.68581208 0.46204309 2.44483821 0.85502927 0.01896269 1.56345758
 2.76501912 3.02026247 2.23826845 3.22441224 2.63121254 2.19093955], Cost: 0.0633
```

```
Current parameters: [ 1.39623044  2.91704     0.37001087  0.91502336  0.74833655  2.9708596
  1.78135655  0.39312068  2.63034464  0.86971068 -0.06371942  1.52097061
  2.81778888  2.97675569  2.25739392  3.2215594   2.63963737  2.19080068], Cost: 0.7550
Current parameters: [1.41424602 2.91432895 0.36634984 0.90637847 0.75044367 2.98143077
 1.71784745 0.44848599 2.47061027 0.88427014 0.0199868  1.62600662
 2.6985242  3.06124168 2.18919166 3.23238835 2.61538144 2.1886597 ], Cost: 0.0405
Current parameters: [1.44855518 2.92881328 0.36996232 0.8946156  0.75075009 2.98325698
 1.59637188 0.58669464 2.56167749 0.99270834 0.02577691 1.63613467
 2.67325833 3.01561488 2.22155075 3.25800934 2.56612298 2.18078553], Cost: 0.3512
Current parameters: [1.38945008 2.91303084 0.36644647 0.90528659 0.75041215 2.98150438
 1.72251634 0.43605111 2.4555939  0.91253785 0.01832003 1.61210943
 2.7090414  3.06316042 2.18209692 3.34122839 2.63348917 2.15299871], Cost: 0.2403
Current parameters: [1.23810217 2.90317025 0.36872013 0.91248627 0.75154141 2.9821249
 1.7160352  0.45606299 2.47422153 0.82412781 0.02378047 1.67320535
 2.60200559 2.99333752 2.25331427 3.25180443 2.5811634  2.26471812], Cost: 0.1977
Current parameters: [1.39562528 2.91423464 0.36664356 0.90430219 0.75102836 2.98140866
 1.72408315 0.43105701 2.44867468 0.93589113 0.01788426 1.59837136
 2.73267684 3.07696134 2.16395117 3.23351891 2.54720611 2.2543643 ], Cost: 0.0994
Current parameters: [1.50446216 2.91599226 0.3661407  0.91942029 0.74935109 2.9815017
 1.76517998 0.3748783  2.39177354 0.98752805 0.01628365 1.62904049
 2.61827784 2.96063655 2.29608659 3.22288389 2.64229258 2.21740347], Cost: 0.4173
Current parameters: [1.36904297 2.91307976 0.41712253 0.94146074 0.74101305 2.94679016
 1.71364644 0.44554837 2.45748021 0.92663749 0.02083378 1.6182001
 2.69800784 3.03284841 2.1491675  3.18937262 2.6319908  2.14515505], Cost: 0.2447
Current parameters: [1.36039451 2.93693877 0.25683695 0.9845917  0.79210692 3.11167836
 1.69022513 0.44568558 2.45454591 0.92039588 0.00946679 1.58939932
 2.67145057 3.16433991 2.21765468 3.16925772 2.65029547 2.15524416], Cost: 0.1767
Current parameters: [1.41609519 2.91999539 0.35956562 0.9015661  0.75408195 2.98944095
 1.80169267 0.5335949  2.44403455 0.87712967 0.01988726 1.61165916
 2.69738997 3.05797356 2.18502063 3.22776784 2.60562174 2.18089699], Cost: 0.1727
Current parameters: [1.39946443 2.91341622 0.35216696 0.85353365 0.74942876 2.98173854
 1.71865145 0.44369057 2.47032668 0.897967   0.02894618 1.62063683
 2.69783853 3.05893262 2.18714357 3.2191835  2.62616491 2.18023431], Cost: 0.0389
Current parameters: [1.40714447 2.91913304 0.35097582 0.86885749 0.75918337 2.98763226
 1.7301848  0.42817715 2.49488443 0.8854712  0.14531642 1.60472969
 2.6981557  3.0540752  2.18613473 3.21987485 2.61223556 2.17576123], Cost: 0.2468
Current parameters: [1.39305168 2.90036485 0.34515968 0.8580538  0.75721211 2.98661396
 1.72530678 0.44537041 2.46805378 0.909513   0.03573282 1.66465972
 2.73249065 3.05427222 2.19823156 3.2159689  2.62852035 2.17708382], Cost: 0.1936
Current parameters: [ 1.43061235e+00  2.92509034e+00  3.48224032e-01  8.22327493e-01
  7.34545813e-01  3.00298116e+00  1.67543354e+00  4.28985656e-01
```



```
  2.43049661e+00  8.31160957e-01 -1.15762729e-03  1.62428951e+00
  2.67901911e+00  3.06044398e+00  2.14737555e+00  3.20814787e+00
  2.58676041e+00  2.16641892e+00], Cost: 0.3737
Current parameters: [1.40243167 2.91570543 0.35316302 0.8540554  0.7523847  2.97567215
 1.7010451  0.46050714 2.44338559 0.89923937 0.04472917 1.62906313
 2.69303026 3.06683844 2.17153106 3.23326032 2.65822988 2.20906   ], Cost: 0.0766
Current parameters: [ 1.40533998  2.93013839  0.34532625  0.85187084  0.75145589  2.98082984
  1.74465133  0.42721683  2.5492465   0.88497501 -0.01061393  1.59415723
  2.70827618  3.0523874   2.16615816  3.21365161  2.67663967  2.22998568], Cost: 0.0927
Current parameters: [1.39949567 2.91328789 0.35295419 0.85664696 0.69044742 2.99818032
 1.72186712 0.44403564 2.47057325 0.90285428 0.03415764 1.62363768
 2.69997112 3.05989653 2.19199923 3.21890081 2.63156828 2.18446373], Cost: 0.0565
Current parameters: [1.45054892 2.92014275 0.34612009 0.85742734 0.7595903  2.98651468
 1.74304036 0.40259837 2.436238   0.93944587 0.01741866 1.61305549
 2.65177183 3.00946162 2.23474996 3.21043754 2.64042023 2.1981356 ], Cost: 0.0811
Current parameters: [1.41345772 2.85584702 0.3538737  0.86267578 0.7492422  2.98220079
 1.71658425 0.4425219  2.47736669 0.89083764 0.02773309 1.61783122
 2.68685416 3.06596884 2.18491132 3.2189323  2.62785513 2.18424   ], Cost: 0.0337
Current parameters: [1.35723241 2.82361864 0.33486523 0.80741888 0.75142565 2.99311983
 1.72538835 0.46305891 2.45896479 0.93362591 0.02420355 1.550325
 2.69262341 3.04511399 2.17701615 3.21206646 2.63340589 2.17794578], Cost: 0.0958
Current parameters: [1.43987246 2.86133765 0.33938096 0.87279544 0.75454057 2.99880808
 1.71259951 0.44101489 2.47391218 0.91048574 0.02559979 1.60232542
 2.70305506 3.09433401 2.17574534 3.20622389 2.63964706 2.16304304], Cost: 0.0665
Current parameters: [1.35241654 2.86278825 0.31486077 0.90918575 0.76591895 3.03143253
 1.70077012 0.43736126 2.48079001 0.87841025 0.01746472 1.62140627
 2.63716753 3.09563357 2.21839673 3.20224818 2.63230858 2.19583347], Cost: 0.1607
Current parameters: [1.42286183 2.86666672 0.35390382 0.85771894 0.75478168 2.98138576
 1.68643664 0.47345443 2.50450947 0.91095196 0.02414302 1.61896611
 2.67463073 3.0581033  2.19422666 3.22312096 2.61121018 2.17867412], Cost: 0.0494
Current parameters: [1.4541736  2.79789237 0.35569181 0.89002437 0.70381105 3.00433743
 1.70639705 0.46196279 2.47784716 0.9027745  0.0390305  1.62733984
 2.6932906  3.0322685  2.17682452 3.22674836 2.66188072 2.24748037], Cost: 0.0479
Current parameters: [1.41844765 2.8487608  0.38000102 0.85084803 0.73895409 2.95949354
 1.71956201 0.44426466 2.47800062 0.89302161 0.02549099 1.61021282
 2.68513559 3.09843873 2.21697805 3.21596035 2.63464986 2.19714244], Cost: 0.0791
Current parameters: [1.41454534 2.85525883 0.33565298 0.86557183 0.74322913 2.96377791
 1.71632383 0.44503027 2.4768602  0.88920226 0.02889657 1.61786072
 2.68693728 3.06481797 2.1851247  3.2039207  2.62408376 2.18697836], Cost: 0.0580
Current parameters: [1.41299681 2.85339017 0.3348681  0.87233289 0.74129943 2.9630768
 1.71589943 0.4346942  2.47698372 0.8953867  0.02175388 1.61306755
```



```
 2.68279207 3.06922084 2.18434203 3.27067821 2.63540863 2.17358195], Cost: 0.0493
Current parameters: [1.4096921  2.85713616 0.36163882 0.87596419 0.74986626 2.97772199
 1.70519982 0.44242453 2.47427315 0.88384384 0.0246899  1.60628485
 2.69747417 3.05524319 2.19282938 3.2161992  2.6331571  2.17741253], Cost: 0.0380
Current parameters: [1.40230554 2.85237556 0.38111834 0.88026712 0.74148026 2.96745073
 1.72126646 0.43581256 2.48174588 0.91386675 0.02333745 1.62656826
 2.66496244 3.06237854 2.15590013 3.20854143 2.63630446 2.16957615], Cost: 0.0585
Current parameters: [1.41197499 2.85931101 0.35762045 0.87440731 0.75186756 2.98137319
 1.72157485 0.44416082 2.47245199 0.89735217 0.02307457 1.61031469
 2.69308233 3.06981756 2.18008244 3.22252282 2.60853327 2.19718557], Cost: 0.0314
Current parameters: [1.4199451  2.86289344 0.36058866 0.88469307 0.75363835 2.98375787
 1.7576298  0.48579944 2.46637871 0.8813917  0.01768785 1.60603045
 2.68378212 3.06534497 2.18282614 3.22554117 2.61429239 2.18386514], Cost: 0.0443
Current parameters: [1.40090015 2.85402135 0.35898863 0.87459476 0.74631351 2.98109654
 1.7155795  0.45443799 2.48130241 0.88717944 0.02796488 1.61292296
 2.70423638 3.08236259 2.16780619 3.22465211 2.60816912 2.19582967], Cost: 0.0322
Current parameters: [1.41787645 2.86414045 0.36279723 0.88008028 0.75713269 2.98233253
 1.73051368 0.43471242 2.48741356 0.89276818 0.07614765 1.59108349
 2.68588507 3.06400584 2.17930777 3.22744096 2.60151948 2.19664661], Cost: 0.0623
Current parameters: [1.41115012 2.86711618 0.35588119 0.87400921 0.75719937 2.97955644
 1.72978551 0.43995923 2.49286607 0.89648775 0.01366503 1.60372758
 2.6971225  3.06720596 2.17704995 3.2213857  2.6192564  2.20703197], Cost: 0.0187
Current parameters: [ 1.4305685   2.87265905  0.36558871  0.85481099  0.75154204  2.98873312
  1.72015993  0.42751644  2.49169591  0.8640949  -0.00850685  1.59141786
  2.68649012  3.05883424  2.152681    3.22622278  2.60652011  2.21097883], Cost: 0.2015
Current parameters: [1.39619652 2.87803757 0.35149598 0.87634896 0.7657271  2.9816041
 1.72908431 0.43466145 2.49056607 0.88956561 0.00988341 1.60030563
 2.68491314 3.07855569 2.18249353 3.21823438 2.61353406 2.19814929], Cost: 0.0464
Current parameters: [1.40175286 2.84791453 0.34449233 0.90293131 0.74841622 3.00782375
 1.72302042 0.44566915 2.49656257 0.9014248  0.01561659 1.60968055
 2.6828657  3.06675811 2.18837042 3.22143069 2.63691738 2.23545173], Cost: 0.0170
Current parameters: [1.38901408 2.83770666 0.34145121 0.88317377 0.75173944 3.01778179
 1.72858469 0.44767968 2.49357142 0.9066834  0.01710696 1.6010264
 2.68150521 3.06136359 2.18644208 3.2217347  2.64077263 2.23406402], Cost: 0.0286
Current parameters: [1.38914448 2.84946395 0.34044062 0.92259911 0.73782345 2.98307494
 1.71543709 0.46005605 2.49087307 0.9309172  0.00905171 1.57544607
 2.69632395 3.06307495 2.18970845 3.21838463 2.63327585 2.23947051], Cost: 0.0089
Current parameters: [1.38995813e+00 2.83792036e+00 3.39748325e-01 9.26191853e-01
 7.26173599e-01 2.98776431e+00 1.72307439e+00 4.53238648e-01
 2.50335923e+00 9.31520794e-01 3.13782571e-03 1.57421775e+00
 2.69988681e+00 3.06089429e+00 2.19774160e+00 3.21600292e+00
```



```
 2.62052730e+00 2.22834333e+00], Cost: 0.0213
Current parameters: [1.3769538  2.85263987 0.34124899 0.93249344 0.76243066 2.9777216
 1.72559327 0.46320273 2.49205815 0.95081014 0.01444042 1.60782754
 2.72999459 3.05336839 2.20177265 3.22388594 2.6374043  2.24197331], Cost: 0.0415
Current parameters: [1.39469293 2.83007786 0.34031088 0.92711079 0.7576897  2.97509457
 1.71212692 0.4600813  2.49301748 0.92743564 0.0054862  1.57135676
 2.69262723 3.06528788 2.18696088 3.21763648 2.6305568  2.23924089], Cost: 0.0234
Current parameters: [ 1.40620892  2.86370406  0.35035364  0.92239681  0.72003477  2.99057924
  1.7006049   0.45903591  2.48383178  0.91906364 -0.01303179  1.56761398
  2.70721933  3.04103805  2.16607476  3.2282953   2.63148888  2.26624307], Cost: 0.0930
Current parameters: [1.39215671 2.84643412 0.3551554  0.91445368 0.73224698 2.97596635
 1.71805174 0.46093688 2.4914471  0.93255846 0.00889928 1.57404311
 2.69597885 3.07807956 2.20488436 3.21932363 2.63990906 2.24845568], Cost: 0.0280
Current parameters: [1.39505786 2.85105596 0.33307014 0.92118307 0.7401493  2.98812537
 1.71376058 0.46121833 2.48938732 0.92928556 0.00939163 1.5710459
 2.70328891 3.06775092 2.19418241 3.21900596 2.63295519 2.23941063], Cost: 0.0136
Current parameters: [1.39685061 2.8502956  0.34486583 0.92744055 0.7372864  2.99061089
 1.71765601 0.45643354 2.49099067 0.94541715 0.00855608 1.57562688
 2.69552123 3.07325236 2.17638428 3.21157023 2.64501776 2.22843726], Cost: 0.0030

   Return from subroutine COBYLA because the MAXFUN limit has been reached.

   NFVALS =  100   F = 8.947370E-03    MAXCV = 0.000000E+00
   X = 1.389144E+00   2.849464E+00   3.404406E-01   9.225991E-01   7.378234E-01
       2.983075E+00   1.715437E+00   4.600560E-01   2.490873E+00   9.309172E-01
       9.051707E-03   1.575446E+00   2.696324E+00   3.063075E+00   2.189708E+00
       3.218385E+00   2.633276E+00   2.239471E+00
Current parameters: [1.38914448 2.84946395 0.34044062 0.92259911 0.73782345 2.98307494
 1.71543709 0.46005605 2.49087307 0.9309172  0.00905171 1.57544607
 2.69632395 3.06307495 2.18970845 3.21838463 2.63327585 2.23947051], Cost: 0.0089
Simulating for QAOA depth p = 10
Current parameters: [1.57346044 1.46294798 2.58955031 0.39319533 1.16424474 1.61394366
 2.92145119 1.12986281 2.09301532 0.21379899 0.78422812 0.6218607
 2.78391886 3.08657124 2.70011325 2.04642168 2.79543284 1.16432341
 2.04775717 2.59110676], Cost: 1.5054
Current parameters: [1.57346044 2.46294798 2.58955031 0.39319533 1.16424474 1.61394366
 2.92145119 1.12986281 2.09301532 0.21379899 0.78422812 0.6218607
 2.78391886 3.08657124 2.70011325 2.04642168 2.79543284 1.16432341
 2.04775717 2.59110676], Cost: 1.7917
Current parameters: [1.57346044 1.46294798 3.58955031 0.39319533 1.16424474 1.61394366
 2.92145119 1.12986281 2.09301532 0.21379899 0.78422812 0.6218607
```

```
 2.78391886 3.08657124 2.70011325 2.04642168 2.79543284 1.16432341
 2.04775717 2.59110676], Cost: 2.1770
Current parameters: [1.57346044 1.46294798 2.58955031 1.39319533 1.16424474 1.61394366
 2.92145119 1.12986281 2.09301532 0.21379899 0.78422812 0.6218607
 2.78391886 3.08657124 2.70011325 2.04642168 2.79543284 1.16432341
 2.04775717 2.59110676], Cost: 0.6665
Current parameters: [1.57346044 1.46294798 2.58955031 1.39319533 2.16424474 1.61394366
 2.92145119 1.12986281 2.09301532 0.21379899 0.78422812 0.6218607
 2.78391886 3.08657124 2.70011325 2.04642168 2.79543284 1.16432341
 2.04775717 2.59110676], Cost: 1.2006
Current parameters: [1.57346044 1.46294798 2.58955031 1.39319533 1.16424474 2.61394366
 2.92145119 1.12986281 2.09301532 0.21379899 0.78422812 0.6218607
 2.78391886 3.08657124 2.70011325 2.04642168 2.79543284 1.16432341
 2.04775717 2.59110676], Cost: 3.2431
Current parameters: [1.57346044 1.46294798 2.58955031 1.39319533 1.16424474 1.61394366
 3.92145119 1.12986281 2.09301532 0.21379899 0.78422812 0.6218607
 2.78391886 3.08657124 2.70011325 2.04642168 2.79543284 1.16432341
 2.04775717 2.59110676], Cost: 2.4665
Current parameters: [1.57346044 1.46294798 2.58955031 1.39319533 1.16424474 1.61394366
 2.92145119 2.12986281 2.09301532 0.21379899 0.78422812 0.6218607
 2.78391886 3.08657124 2.70011325 2.04642168 2.79543284 1.16432341
 2.04775717 2.59110676], Cost: 3.7405
Current parameters: [1.57346044 1.46294798 2.58955031 1.39319533 1.16424474 1.61394366
 2.92145119 1.12986281 3.09301532 0.21379899 0.78422812 0.6218607
 2.78391886 3.08657124 2.70011325 2.04642168 2.79543284 1.16432341
 2.04775717 2.59110676], Cost: 1.9296
Current parameters: [1.57346044 1.46294798 2.58955031 1.39319533 1.16424474 1.61394366
 2.92145119 1.12986281 2.09301532 1.21379899 0.78422812 0.6218607
 2.78391886 3.08657124 2.70011325 2.04642168 2.79543284 1.16432341
 2.04775717 2.59110676], Cost: 2.5863
Current parameters: [1.57346044 1.46294798 2.58955031 1.39319533 1.16424474 1.61394366
 2.92145119 1.12986281 2.09301532 0.21379899 1.78422812 0.6218607
 2.78391886 3.08657124 2.70011325 2.04642168 2.79543284 1.16432341
 2.04775717 2.59110676], Cost: 3.9218
Current parameters: [1.57346044 1.46294798 2.58955031 1.39319533 1.16424474 1.61394366
 2.92145119 1.12986281 2.09301532 0.21379899 0.78422812 1.6218607
 2.78391886 3.08657124 2.70011325 2.04642168 2.79543284 1.16432341
 2.04775717 2.59110676], Cost: 0.6299
Current parameters: [1.57346044 1.46294798 2.58955031 1.39319533 1.16424474 1.61394366
 2.92145119 1.12986281 2.09301532 0.21379899 0.78422812 1.6218607
 3.78391886 3.08657124 2.70011325 2.04642168 2.79543284 1.16432341
```



```
 2.04775717 2.59110676], Cost: 1.3568
Current parameters: [1.57346044 1.46294798 2.58955031 1.39319533 1.16424474 1.61394366
 2.92145119 1.12986281 2.09301532 0.21379899 0.78422812 1.6218607
 2.78391886 4.08657124 2.70011325 2.04642168 2.79543284 1.16432341
 2.04775717 2.59110676], Cost: 2.8854
Current parameters: [1.57346044 1.46294798 2.58955031 1.39319533 1.16424474 1.61394366
 2.92145119 1.12986281 2.09301532 0.21379899 0.78422812 1.6218607
 2.78391886 3.08657124 3.70011325 2.04642168 2.79543284 1.16432341
 2.04775717 2.59110676], Cost: 1.7579
Current parameters: [1.57346044 1.46294798 2.58955031 1.39319533 1.16424474 1.61394366
 2.92145119 1.12986281 2.09301532 0.21379899 0.78422812 1.6218607
 2.78391886 3.08657124 2.70011325 3.04642168 2.79543284 1.16432341
 2.04775717 2.59110676], Cost: 1.4649
Current parameters: [1.57346044 1.46294798 2.58955031 1.39319533 1.16424474 1.61394366
 2.92145119 1.12986281 2.09301532 0.21379899 0.78422812 1.6218607
 2.78391886 3.08657124 2.70011325 2.04642168 3.79543284 1.16432341
 2.04775717 2.59110676], Cost: 1.8221
Current parameters: [1.57346044 1.46294798 2.58955031 1.39319533 1.16424474 1.61394366
 2.92145119 1.12986281 2.09301532 0.21379899 0.78422812 1.6218607
 2.78391886 3.08657124 2.70011325 2.04642168 2.79543284 2.16432341
 2.04775717 2.59110676], Cost: 2.2288
Current parameters: [1.57346044 1.46294798 2.58955031 1.39319533 1.16424474 1.61394366
 2.92145119 1.12986281 2.09301532 0.21379899 0.78422812 1.6218607
 2.78391886 3.08657124 2.70011325 2.04642168 2.79543284 1.16432341
 3.04775717 2.59110676], Cost: 2.5603
Current parameters: [1.57346044 1.46294798 2.58955031 1.39319533 1.16424474 1.61394366
 2.92145119 1.12986281 2.09301532 0.21379899 0.78422812 1.6218607
 2.78391886 3.08657124 2.70011325 2.04642168 2.79543284 1.16432341
 2.04775717 3.59110676], Cost: 2.3881
Current parameters: [ 1.62743132  1.42443549  2.49921094  1.50603529  1.09239467  1.26736251
  2.6793344   0.71637643  1.9231152  -0.04443266  0.34636231  1.6267722
  2.68614322  2.78319741  2.54839509  1.93410801  2.63507171  0.94925827
  1.78811127  2.35462425], Cost: 2.7049
Current parameters: [1.58123024 1.45740362 2.5765448  1.40944008 1.153901   2.09536261
 2.88659539 1.07033617 2.06855603 0.17662325 0.72119175 1.62256777
 2.76984281 3.04289671 2.67827148 2.0302527  2.77234681 1.13336204
 2.01037783 2.55706209], Cost: 1.5896
Current parameters: [1.78807722 1.46294798 2.71776747 1.39319533 1.16424474 1.61394366
 2.92145119 1.12986281 2.09301532 0.21379899 0.78422812 1.6218607
 2.78391886 3.08657124 2.70011325 2.04642168 2.79543284 1.16432341
 2.04775717 2.59110676], Cost: 0.7735
```



```
Current parameters: [1.31501919 1.46127333 3.01559307 1.398102   1.16112045 1.60666794
 2.91092312 1.11188301 2.08562748 0.2025702  0.76518822 1.62207427
 2.77966724 3.07337951 2.69351602 2.0415379  2.78845979 1.15497164
 2.03646688 2.5808237 ], Cost: 0.6705
Current parameters: [1.57346044 1.69954713 2.58955031 1.47394707 1.16424474 1.61394366
 2.92145119 1.12986281 2.09301532 0.21379899 0.78422812 1.6218607
 2.78391886 3.08657124 2.70011325 2.04642168 2.79543284 1.16432341
 2.04775717 2.59110676], Cost: 0.6515
Current parameters: [1.56975973 1.62318757 2.58823228 0.92190706 1.16066142 1.60559896
 2.90937628 1.10924132 2.08454202 0.2009204  0.76239077 1.62210565
 2.77904257 3.07144131 2.69254672 2.04082035 2.78743527 1.15359763
 2.03480805 2.57931285], Cost: 0.6240
Current parameters: [1.56975973 1.62318757 2.58823228 0.92190706 1.16066142 1.60559896
 2.90937628 0.92747694 2.08454202 0.2009204  0.9340349  1.62210565
 2.77904257 3.07144131 2.69254672 2.04082035 2.78743527 1.15359763
 2.03480805 2.57931285], Cost: 1.4470
Current parameters: [1.54672167 1.62292443 2.58002715 0.91151524 1.13835416 1.55365072
 2.83420639 1.50070876 2.03179321 0.12074734 0.97671337 1.62363052
 2.74868621 2.97725298 2.64544286 2.00595037 2.73764802 1.08682645
 1.95419591 2.50589223], Cost: 2.6149
Current parameters: [1.56975973 1.62318757 2.58823228 0.92190706 1.16066142 1.60559896
 2.72700065 1.10924132 2.08454202 0.37191493 0.76239077 1.62210565
 2.77904257 3.07144131 2.69254672 2.04082035 2.78743527 1.15359763
 2.03480805 2.57931285], Cost: 0.5527
Current parameters: [1.55636438 1.62303457 2.58346145 0.9158648  1.14769098 1.57539395
 3.04681085 1.11939949 2.05387152 0.72313584 0.65672757 1.62299227
 2.76139204 3.01667603 2.66515844 2.0205454  2.75848675 1.1147739
 1.98793657 2.53662284], Cost: 1.2868
Current parameters: [1.56975973 1.62318757 2.58823228 0.92190706 0.93040465 1.60559896
 2.72700065 1.10924132 2.18191667 0.37191493 0.76239077 1.62210565
 2.77904257 3.07144131 2.69254672 2.04082035 2.78743527 1.15359763
 2.03480805 2.57931285], Cost: 1.0807
Current parameters: [1.56259412 1.62310572 2.58568021 0.91867486 1.37770912 1.58944132
 2.71622588 1.11467525 2.52735396 0.36583904 0.70586812 1.62257993
 2.76960073 3.0421456  2.67789585 2.02997462 2.77194977 1.13282957
 2.00973499 2.55647659], Cost: 0.5908
Current parameters: [1.56355743 1.6276134  2.59861401 0.90893957 1.16891837 1.64542781
 2.75482453 1.15675888 2.10406682 0.40159073 0.81270999 1.62210565
 2.77904257 3.30314265 2.69254672 2.04082035 2.78743527 1.15359763
 2.03480805 2.57931285], Cost: 1.5665
Current parameters: [1.56967643 1.60735636 2.54387777 0.95767167 1.21054816 1.41596104
```



```
 2.59609056 0.95857993 1.98707743 0.2488582  0.4123539  1.62355639
 2.75016193 3.07931937 2.64773273 2.00764552 2.74006834 1.09007241
 1.95811472 2.50946145], Cost: 2.0252
Current parameters: [1.56975973 1.62318757 2.58823228 0.92190706 1.16066142 1.60559896
 2.72700065 1.10924132 2.08454202 0.37191493 0.76239077 1.62210565
 2.77904257 3.07144131 2.69254672 2.04082035 2.78743527 1.15359763
 2.20314797 2.39448409], Cost: 0.5286
Current parameters: [1.55548417 1.62550414 2.59009094 0.90985976 1.20431861 1.60308434
 2.72602072 1.14367624 2.08108021 0.37907468 0.70451074 1.62282416
 2.76473885 2.98803543 2.67035167 2.02438984 2.76397583 1.12213547
 2.56179969 2.71857421], Cost: 2.5272
Current parameters: [1.56975973 1.62318757 2.58823228 0.92190706 1.16066142 1.60559896
 2.72700065 1.10924132 2.08454202 0.37191493 0.76239077 1.62210565
 2.77904257 3.07144131 2.69254672 2.04082035 2.58701659 1.30303776
 2.20314797 2.39448409], Cost: 0.0673
Current parameters: [1.55931546 1.62488242 2.58959211 0.91309303 1.19260185 1.60375921
 2.72628371 1.13443456 2.08200929 0.37715314 0.72004464 1.62263132
 2.76857769 3.01041998 2.6763084  2.02879947 2.85831622 1.71132253
 2.17289746 2.36505511], Cost: 2.3936
Current parameters: [1.56975973 1.62318757 2.58823228 0.92190706 1.16066142 1.60559896
 2.72700065 1.10924132 2.08454202 0.37191493 0.76239077 1.62210565
 2.77904257 3.07144131 2.84129362 1.83988663 2.58701659 1.30303776
 2.20314797 2.39448409], Cost: 0.6422
Current parameters: [1.56294883 1.62429281 2.58911905 0.91615927 1.18149035 1.60439923
 2.72653312 1.12567029 2.08289038 0.37533086 0.73477609 1.62244845
 2.77221823 3.03164819 3.07661389 2.35199668 2.55068253 1.283287
 2.18342106 2.37529292], Cost: 1.6168
Current parameters: [1.56975973 1.62318757 2.58823228 0.92190706 1.16066142 1.60559896
 2.72700065 1.10924132 2.08454202 0.37191493 0.76239077 1.87179083
 2.79158484 3.07144131 2.69254672 2.04082035 2.58701659 1.30303776
 2.20314797 2.39448409], Cost: 0.0862
Current parameters: [1.56636337 1.62373872 2.58867448 0.91904083 1.17104809 1.60500069
 2.72676751 1.11743389 2.08371841 0.37361834 0.74862027 1.59675347
 3.27668796 3.05159784 2.67494173 2.04118177 2.56889804 1.29318874
 2.19331084 2.38491411], Cost: 1.3514
Current parameters: [1.57131348 1.62207884 2.58563152 0.92515558 1.19971225 1.81074477
 2.65928366 1.09733756 2.02913939 0.38382527 0.74978516 1.62237944
 2.77359209 2.98950973 2.68408922 2.03455945 2.57807729 1.291049
 2.14992482 2.42384613], Cost: 0.2658
Current parameters: [1.56178798 1.61910684 2.57422197 0.92733441 1.04465903 1.79022068
 2.91431193 1.07729967 2.24268276 0.26019748 0.61145554 1.62378365
```

```
 2.70883878 3.19297877 2.58892717 2.03399497 2.48004726 1.23497101
 2.27983985 2.16354621], Cost: 1.1268
Current parameters: [1.55934535 1.6204097  2.57707815 0.92322292 1.18835718 1.49008889
 2.68391915 1.40398559 2.0529151  0.31309635 0.95212664 1.62461669
 2.69059699 2.91004124 2.56086092 2.01636668 2.45049719 1.19873737
 2.09925315 2.27477623], Cost: 1.4001
Current parameters: [1.57496146 1.61623713 2.57045725 0.94010711 1.19198654 1.51310169
 2.63786389 1.01460022 2.01556001 0.33668337 0.62334626 1.62292599
 2.75051233 3.0818488  2.65003856 2.03251909 2.54293325 1.26890987
 2.13949333 2.38768791], Cost: 0.8064
Current parameters: [1.63347416 1.6235707  2.48158363 0.92078449 1.1619942  1.60673533
 2.73439302 1.11271675 2.08497856 0.38134544 0.76607111 1.62206735
 2.77980507 3.07282705 2.69372989 2.04169622 2.58826716 1.30471494
 2.20446073 2.39711011], Cost: 0.0865
Current parameters: [1.55904751 1.62310922 2.60335371 0.92146608 1.17961138 1.58451843
 2.8630084  1.08568484 2.05237392 0.55411144 0.72541812 1.62258537
 2.76571328 3.01169502 2.67240959 2.03309017 2.56599451 1.28258732
 2.16293679 2.39092215], Cost: 0.2706
Current parameters: [1.58571178 1.50620243 2.59776242 0.88197981 1.16066142 1.60559896
 2.72700065 1.10924132 2.08454202 0.37191493 0.76239077 1.62210565
 2.77904257 3.07144131 2.69254672 2.04082035 2.58701659 1.30303776
 2.20314797 2.39448409], Cost: 0.1086
Current parameters: [1.78179968 1.65927425 2.71489493 0.9323853  1.16236306 1.60474032
 2.72816491 1.11187843 2.08824247 0.37008209 0.75933327 1.62213082
 2.77670322 3.06863811 2.68918229 2.04182059 2.58358803 1.30220578
 2.2043291  2.3920856 ], Cost: 0.3705
Current parameters: [1.56887353 1.66323991 2.58794184 0.80413204 1.15795695 1.60370834
 2.72446121 1.10436577 2.0794311  0.36847518 0.75722776 1.62216348
 2.77789121 3.06833249 2.69076016 2.0394978  2.58512825 1.30050525
 2.20042731 2.39132958], Cost: 0.1406
Current parameters: [1.57178236 1.62144811 2.59050484 0.92923948 1.03763397 1.61852457
 2.76087977 1.13054427 1.88162758 0.35564902 0.78084553 1.62188692
 2.78210727 3.11592567 2.69748849 2.0469277  2.59232935 1.31280507
 2.23961331 2.37877659], Cost: 0.2215
Current parameters: [1.55436622 1.62422102 2.5854794  0.92254934 1.17755537 1.60303479
 2.73412503 1.1326646  2.09287234 0.37046302 0.73862474 1.62263029
 2.75333616 3.03546517 2.6548627  2.04190654 2.5482447  1.28230815
 2.38380066 2.5454187 ], Cost: 1.1497
Current parameters: [1.56692517 1.62319501 2.58715687 0.92332715 1.16216374 1.6044596
 2.73014914 1.11586454 2.08642178 0.36891492 0.7562805  1.61574479
 2.90154752 3.06500936 2.68085631 2.03999752 2.57494632 1.2952989
```



```
 2.20180454 2.38596551], Cost: 0.2024
Current parameters: [1.56960836 1.6227582  2.58698098 0.92280594 1.1477655  1.62464298
 2.74284202 1.10206874 2.09934916 0.3630133  0.75479528 1.62239798
 2.77322287 3.08888665 2.69098027 2.03966074 2.56015431 1.26701194
 2.21016794 2.37701132], Cost: 0.0693
Current parameters: [1.55947297 1.62279941 2.58328816 0.92609598 1.14634287 1.63618567
 2.76250306 1.11581324 2.11650284 0.35075866 0.73068108 1.62205908
 2.76781881 3.07892398 2.66254039 2.0416819  2.62121328 1.3737943
 2.20462626 2.33706371], Cost: 0.1550
Current parameters: [1.57069036 1.62330568 2.58886469 0.92208375 1.15900845 1.61214219
 2.7319147  1.05346886 2.08644691 0.37384546 0.77888586 1.62177367
 2.78565141 3.07968057 2.69676448 2.04394267 2.59453269 1.31311784
 2.21350732 2.40085647], Cost: 0.1421
Current parameters: [1.56005026 1.62251701 2.58267317 0.92718923 1.16472603 1.58573876
 2.72607889 1.11939874 2.08638703 0.35334435 0.85410976 1.62236337
 2.76149603 3.03479621 2.65713014 2.03818014 2.56313862 1.29645165
 2.17684659 2.35000439], Cost: 0.2142
Current parameters: [1.56916028 1.62353477 2.58901013 0.92083593 1.21073231 1.6084844
 2.74180068 1.11306693 2.06520312 0.36217824 0.76547631 1.622113
 2.77889617 3.08752322 2.70106153 2.04712368 2.58653503 1.30239193
 2.2144661  2.38118101], Cost: 0.0565
Current parameters: [1.56167122 1.62352197 2.58613533 0.92375867 1.19653124 1.60517333
 2.74298668 1.12315251 2.07806252 0.35913226 0.76204891 1.62177279
 2.77610827 3.06389807 2.78608087 2.1282176  2.57648443 1.30850055
 2.20134942 2.36197076], Cost: 0.3012
Current parameters: [1.56905501 1.62360989 2.58918633 0.92061584 1.21087245 1.60916038
 2.70859713 1.11387341 2.06553449 0.39425615 0.76633033 1.622113
 2.77889617 3.09145569 2.70106153 2.04712368 2.58653503 1.30239193
 2.24269309 2.3501892 ], Cost: 0.0766
Current parameters: [1.55994338 1.62529411 2.59050391 0.9194504  1.21287599 1.62375077
 2.72816934 1.14182458 2.08056184 0.39804661 0.79234415 1.62165639
 2.77765098 3.18012119 2.69010745 2.05864114 2.58148765 1.31679707
 2.1668175  2.40774898], Cost: 0.2921
Current parameters: [1.56321566 1.62539194 2.59454452 0.92475484 1.21200025 1.5994762
 2.77066922 1.11372771 2.0593809  0.40912803 0.75501814 1.62072417
 2.773391   3.07013136 2.69657769 2.04587381 2.58316174 1.3006504
 2.20109505 2.38396671], Cost: 0.0867
Current parameters: [1.5898386  1.60635354 2.56918862 0.88926215 1.24268432 1.64434638
 2.80099926 1.06505151 2.05245864 0.35103949 0.78338747 1.63574369
 2.79619349 3.107839   2.68789691 2.04514881 2.56983196 1.28335397
 2.22836569 2.3275286 ], Cost: 0.1487
```



```
Current parameters: [1.56197931 1.6228342  2.58559836 0.9205614  1.21629013 1.61188351
 2.75246045 1.12646652 2.07289934 0.3571827  0.76642923 1.62285825
 2.77139937 3.07871323 2.68388506 2.05200956 2.57433374 1.30473293
 2.25947865 2.40925237], Cost: 0.1159
Current parameters: [1.56832776 1.6239411  2.589323   0.92203387 1.21025266 1.60648911
 2.73988872 1.11375431 2.06500123 0.36255418 0.76357727 1.65306154
 2.77952559 3.08679793 2.70055924 2.04727771 2.58634526 1.30284448
 2.21334146 2.38241697], Cost: 0.0497
Current parameters: [1.56416002 1.62136056 2.58129117 0.92850411 1.22117711 1.58358396
 2.72267568 1.09578678 2.05166331 0.34843542 0.72917133 1.64979375
 2.76867472 3.08772456 2.67972125 2.04883142 2.57018353 1.29928075
 2.2032869  2.37912801], Cost: 0.0152
Current parameters: [1.56430585 1.62128309 2.58121379 0.92833099 1.22130281 1.58379158
 2.72286424 1.0955375  2.05159674 0.34832326 0.72928878 1.64978659
 2.76878731 3.08785692 2.6911533  2.03338158 2.55036448 1.31391096
 2.20338136 2.37891398], Cost: 0.0450
Current parameters: [1.55545464 1.62008013 2.57501379 0.93418285 1.22729352 1.57278754
 2.72146296 1.09781129 2.05227733 0.33822977 0.71673134 1.6513379
 2.76022045 3.08578596 2.6924886  2.01097454 2.60130499 1.27309257
 2.20605796 2.37753605], Cost: 0.0472
Current parameters: [1.57768845 1.62357274 2.58939681 0.92844671 1.21733644 1.5618465
 2.73044245 1.09671799 2.05773096 0.34680322 0.73058209 1.64975092
 2.76934906 3.09665577 2.68035732 2.04964085 2.57072254 1.30036889
 2.20929583 2.37536608], Cost: 0.0183
Current parameters: [1.60701628 1.62947629 2.6073164  0.93632099 1.22943056 1.6042826
 2.71079284 1.09829821 2.04564873 0.3449033  0.71834881 1.65079977
 2.76138293 3.07302914 2.67139846 2.04490732 2.57243135 1.29388368
 2.19668033 2.3839068 ], Cost: 0.0434
Current parameters: [1.56539912 1.6212265  2.58206567 0.92904514 1.2072976  1.5873585
 2.7267932  1.09769931 2.02914225 0.34845161 0.72092217 1.64976623
 2.77038157 3.0967603  2.68399583 2.04979498 2.57334418 1.30114521
 2.21016528 2.38174192], Cost: 0.0203
Current parameters: [1.55836203 1.62294867 2.58137269 0.93753349 1.20831975 1.56988286
 2.7187366  1.10687694 2.02745413 0.33635119 0.76493675 1.65075535
 2.75613855 3.06989307 2.6591687  2.0448287  2.56507724 1.29479462
 2.19390358 2.36373192], Cost: 0.0108
Current parameters: [1.56054792 1.62268169 2.58177294 0.93665527 1.21246756 1.56410161
 2.71266967 1.10473558 2.02091332 0.34001886 0.77117643 1.64999518
 2.77331506 3.06866445 2.66433932 2.04477613 2.55596093 1.27838219
 2.19420306 2.37408427], Cost: 0.0116
Current parameters: [1.55140767 1.62561655 2.5825317  0.94694998 1.20245655 1.568933
```



```
 2.72074598 1.12217698 2.03334854 0.32690349 0.74922805 1.64879757
 2.80176907 3.05906648 2.63836027 2.04058722 2.57303017 1.30775149
 2.18927182 2.34924659], Cost: 0.0777
Current parameters: [1.557142   1.63442078 2.5830541  0.91026728 1.20604376 1.56766286
 2.71560041 1.10940188 2.02577736 0.33660398 0.76293742 1.65078966
 2.75590556 3.06746448 2.65397393 2.04012856 2.56634733 1.29557603
 2.19191813 2.3654062 ], Cost: 0.0107
Current parameters: [1.55263541 1.63892381 2.57922389 0.90297135 1.20379036 1.55810902
 2.71125536 1.1131727  2.02468342 0.32202178 0.75375152 1.65243811
 2.74454591 3.04702085 2.68614984 2.07897533 2.56439923 1.28632508
 2.18543829 2.35337849], Cost: 0.0092
Current parameters: [1.54981959 1.66704871 2.57501109 0.9125092  1.20643047 1.56052301
 2.71371482 1.10932502 2.02480806 0.32169392 0.75525355 1.65242544
 2.74459938 3.05190149 2.68678478 2.07982311 2.56289268 1.28471215
 2.18668549 2.35215263], Cost: 0.0069
Current parameters: [1.54318084 1.66431465 2.57196955 0.91102377 1.21329616 1.55855157
 2.69882041 1.12539149 2.02582876 0.32477244 0.76107166 1.65471681
 2.72919877 3.09261563 2.67926525 2.09758564 2.56265503 1.27649605
 2.15751799 2.36095322], Cost: 0.0573
Current parameters: [1.56523322 1.66702947 2.54926848 0.91204063 1.20408901 1.55873923
 2.71301548 1.11454187 2.02504614 0.32526497 0.75530136 1.65250452
 2.74484564 3.04900717 2.68608902 2.07935843 2.56468652 1.28714058
 2.18479529 2.35440394], Cost: 0.0065
Current parameters: [1.57330489 1.65882688 2.53877727 0.89659673 1.22219335 1.57240302
 2.7389935  1.08881561 2.01590277 0.31580133 0.76175852 1.66050345
 2.74663842 3.05814031 2.67813634 2.0843881  2.55769831 1.27118731
 2.18843058 2.3260578 ], Cost: 0.0234
Current parameters: [1.56514991 1.66731038 2.55002801 0.9113656  1.22499754 1.55283184
 2.71206444 1.11534472 2.01332426 0.32560646 0.76009807 1.65237758
 2.74737275 3.0480494  2.68966107 2.08200274 2.57511815 1.30113075
 2.18636745 2.35709019], Cost: 0.0159
Current parameters: [1.56498386 1.66716355 2.54625692 0.91215075 1.1824147  1.55224544
 2.70027808 1.06576919 2.02918634 0.32559407 0.76642763 1.65419965
 2.74271541 3.04170397 2.68325837 2.0874027  2.58029383 1.2961551
 2.1802983  2.36938861], Cost: 0.0311
Current parameters: [1.56625504 1.66606234 2.54609452 0.91026209 1.20475843 1.56343821
 2.70247337 1.11720877 2.02929329 0.30383772 0.76009813 1.65327691
 2.74560622 3.05471394 2.68427094 2.08098166 2.56347748 1.28841881
 2.20077099 2.35913611], Cost: 0.0006
Current parameters: [1.55496515 1.66647038 2.54174855 0.9117137  1.21030332 1.55665294
 2.72077821 1.12298143 2.03088741 0.31481815 0.75293157 1.65560948
```

```
 2.72537113 3.03657805 2.66305286 2.09450511 2.55237001 1.2786606
 2.23096302 2.38425428], Cost: 0.0860
Current parameters: [1.56553637 1.66641976 2.54640294 0.91119198 1.2044007  1.56197618
 2.69157618 1.11800401 2.02921914 0.31314767 0.75876573 1.67889544
 2.74612723 3.05521673 2.68385516 2.08110916 2.56332039 1.28879342
 2.20775849 2.35146515], Cost: 0.0004
Current parameters: [1.56610803 1.66605352 2.54492988 0.90951828 1.20483871 1.56309534
 2.66360528 1.11531458 2.02876837 0.33636946 0.76084124 1.64435574
 2.74226081 3.05933015 2.68390704 2.08393476 2.56447772 1.28467896
 2.23004021 2.32285717], Cost: 0.0043
Current parameters: [1.56978618 1.66777555 2.55132804 0.90704994 1.19846042 1.57375293
 2.6982568  1.12398365 2.03392667 0.32154285 0.77528676 1.67961039
 2.75275355 3.05547371 2.68648634 2.09378584 2.56122532 1.297035
 2.21056973 2.35319075], Cost: 0.0234
Current parameters: [1.56092421 1.66734157 2.54413947 0.9105433  1.20074416 1.56628927
 2.69625479 1.12667458 2.03735485 0.30898669 0.77257296 1.68199907
 2.73956768 3.05320411 2.71124579 2.05285324 2.59861623 1.26453279
 2.2125462  2.34789466], Cost: 0.0215
Current parameters: [1.57783353 1.66879263 2.55363928 0.9133679  1.21475383 1.57720959
 2.68317902 1.11714223 2.03464361 0.3133727  0.75377861 1.6792335
 2.74349707 3.04484512 2.67735825 2.08378946 2.5691195  1.28181521
 2.2015674  2.35508678], Cost: 0.0126
Current parameters: [1.60223282 1.67358301 2.56691601 0.91736203 1.18709786 1.54975025
 2.69000826 1.11988483 2.00235173 0.30053464 0.7556707  1.67939366
 2.73381435 3.06067188 2.69334392 2.06764377 2.55038537 1.29364768
 2.21054764 2.34422323], Cost: 0.0187
Current parameters: [1.56972209 1.66747161 2.53513129 0.91324362 1.19620697 1.56387796
 2.69366665 1.12660769 2.03657484 0.32654047 0.75244923 1.67757493
 2.74328074 3.04895611 2.68844637 2.07295336 2.55139607 1.28702763
 2.19672092 2.3508454 ], Cost: 0.0050
Current parameters: [1.5496415  1.66335387 2.54701686 0.90575461 1.22635247 1.57250051
 2.67628705 1.09982029 2.0371864  0.29707382 0.76492523 1.67979398
 2.74216841 3.05252144 2.70594954 2.0610018  2.53646858 1.31020373
 2.20724872 2.35022523], Cost: 0.0415
Current parameters: [1.56145852 1.66802098 2.54488121 0.91547085 1.19837458 1.55737935
 2.6896761  1.12272085 2.0306816  0.30349075 0.74918295 1.67754784
 2.76663878 3.05190553 2.67717215 2.07988795 2.56829205 1.29165204
 2.20364788 2.33977015], Cost: 0.0118
Current parameters: [1.56853519 1.66712724 2.54847452 0.91160865 1.20764004 1.55311246
 2.69171573 1.11876917 2.03762487 0.31191919 0.76499727 1.67901364
 2.74445193 3.05434819 2.68066974 2.08100457 2.56194424 1.28792836
```



```
 2.20561305 2.34854236], Cost: 0.0004
Current parameters: [1.56139427 1.66440909 2.54320264 0.9106963  1.20432415 1.56056087
 2.68255718 1.12339242 2.02685263 0.31176942 0.75831255 1.67945266
 2.74017329 3.0776222  2.6811489  2.08736146 2.56483902 1.28338921
 2.1934158  2.35330331], Cost: 0.0133
Current parameters: [1.56173348 1.6662901  2.54403949 0.91328033 1.200487   1.56313871
 2.68832463 1.11698025 2.02322448 0.31166185 0.76664642 1.67915691
 2.74317611 3.04888045 2.67902211 2.08033235 2.56314515 1.28815988
 2.20374153 2.35015338], Cost: 0.0007
Current parameters: [1.56978935 1.66667519 2.54251655 0.91095629 1.19152524 1.55594128
 2.68729776 1.09532561 2.0356818  0.3173222  0.75673942 1.67888825
 2.74703761 3.05521666 2.6857206  2.08357462 2.57120231 1.29203825
 2.20445202 2.35861813], Cost: 0.0042
Current parameters: [1.56480155 1.66631918 2.54572438 0.91123849 1.20261229 1.56521905
 2.69538485 1.11839242 2.03229403 0.31131175 0.75576464 1.67947962
 2.73763049 3.05602677 2.68101116 2.08117749 2.56759862 1.29641722
 2.20754107 2.34553502], Cost: 0.0013
Current parameters: [1.57176995 1.66239072 2.53758122 0.90420429 1.2089717  1.56687774
 2.7042587  1.10548376 2.02510142 0.31202061 0.76042464 1.6823125
 2.7488554  3.05901986 2.6804172  2.0819931  2.55814167 1.2768322
 2.20626182 2.33757245], Cost: 0.0050
Current parameters: [1.56553637 1.66641976 2.54640294 0.91119198 1.2044007  1.56197618
 2.69157618 1.11800401 2.02921914 0.31314767 0.75876573 1.67889544
 2.74612723 3.05521673 2.68385516 2.08110916 2.56332039 1.28879342
 2.20775849 2.35146515], Cost: 0.0004

   Return from subroutine COBYLA because the MAXFUN limit has been reached.

   NFVALS =  100   F = 3.820940E-04    MAXCV = 0.000000E+00
   X = 1.565536E+00   1.666420E+00   2.546403E+00   9.111920E-01   1.204401E+00
       1.561976E+00   2.691576E+00   1.118004E+00   2.029219E+00   3.131477E-01
       7.587657E-01   1.678895E+00   2.746127E+00   3.055217E+00   2.683855E+00
       2.081109E+00   2.563320E+00   1.288793E+00   2.207758E+00   2.351465E+00
Simulating for QAOA depth p = 11
Current parameters: [2.69638074 1.99188054 2.01014367 1.02166077 2.943378   3.01647325
 2.8308811  1.88912793 0.29390742 2.59282582 3.04087624 0.79746787
 1.00645955 0.2908839  0.01279819 2.67668866 1.34304021 0.19712194
 1.75268318 1.1084472  0.66899197 0.91281542], Cost: 1.9825
Current parameters: [1.69638074 2.99188054 2.01014367 1.02166077 2.943378   3.01647325
 2.8308811  1.88912793 0.29390742 2.59282582 3.04087624 0.79746787
 1.00645955 0.2908839  0.01279819 2.67668866 1.34304021 0.19712194
```



```
 1.75268318 1.1084472  0.66899197 0.91281542], Cost: 2.1063
Current parameters: [1.69638074 1.99188054 3.01014367 1.02166077 2.943378   3.01647325
 2.8308811  1.88912793 0.29390742 2.59282582 3.04087624 0.79746787
 1.00645955 0.2908839  0.01279819 2.67668866 1.34304021 0.19712194
 1.75268318 1.1084472  0.66899197 0.91281542], Cost: 2.9761
Current parameters: [1.69638074 1.99188054 2.01014367 2.02166077 2.943378   3.01647325
 2.8308811  1.88912793 0.29390742 2.59282582 3.04087624 0.79746787
 1.00645955 0.2908839  0.01279819 2.67668866 1.34304021 0.19712194
 1.75268318 1.1084472  0.66899197 0.91281542], Cost: 2.4096
Current parameters: [1.69638074 1.99188054 2.01014367 1.02166077 3.943378   3.01647325
 2.8308811  1.88912793 0.29390742 2.59282582 3.04087624 0.79746787
 1.00645955 0.2908839  0.01279819 2.67668866 1.34304021 0.19712194
 1.75268318 1.1084472  0.66899197 0.91281542], Cost: 2.3947
Current parameters: [1.69638074 1.99188054 2.01014367 1.02166077 2.943378   4.01647325
 2.8308811  1.88912793 0.29390742 2.59282582 3.04087624 0.79746787
 1.00645955 0.2908839  0.01279819 2.67668866 1.34304021 0.19712194
 1.75268318 1.1084472  0.66899197 0.91281542], Cost: 0.9035
Current parameters: [1.69638074 1.99188054 2.01014367 1.02166077 2.943378   4.01647325
 3.8308811  1.88912793 0.29390742 2.59282582 3.04087624 0.79746787
 1.00645955 0.2908839  0.01279819 2.67668866 1.34304021 0.19712194
 1.75268318 1.1084472  0.66899197 0.91281542], Cost: 0.9995
Current parameters: [1.69638074 1.99188054 2.01014367 1.02166077 2.943378   4.01647325
 2.8308811  2.88912793 0.29390742 2.59282582 3.04087624 0.79746787
 1.00645955 0.2908839  0.01279819 2.67668866 1.34304021 0.19712194
 1.75268318 1.1084472  0.66899197 0.91281542], Cost: 1.6018
Current parameters: [1.69638074 1.99188054 2.01014367 1.02166077 2.943378   4.01647325
 2.8308811  1.88912793 1.29390742 2.59282582 3.04087624 0.79746787
 1.00645955 0.2908839  0.01279819 2.67668866 1.34304021 0.19712194
 1.75268318 1.1084472  0.66899197 0.91281542], Cost: 1.3765
Current parameters: [1.69638074 1.99188054 2.01014367 1.02166077 2.943378   4.01647325
 2.8308811  1.88912793 0.29390742 3.59282582 3.04087624 0.79746787
 1.00645955 0.2908839  0.01279819 2.67668866 1.34304021 0.19712194
 1.75268318 1.1084472  0.66899197 0.91281542], Cost: 0.7767
Current parameters: [1.69638074 1.99188054 2.01014367 1.02166077 2.943378   4.01647325
 2.8308811  1.88912793 0.29390742 3.59282582 4.04087624 0.79746787
 1.00645955 0.2908839  0.01279819 2.67668866 1.34304021 0.19712194
 1.75268318 1.1084472  0.66899197 0.91281542], Cost: 2.2572
Current parameters: [1.69638074 1.99188054 2.01014367 1.02166077 2.943378   4.01647325
 2.8308811  1.88912793 0.29390742 3.59282582 3.04087624 1.79746787
 1.00645955 0.2908839  0.01279819 2.67668866 1.34304021 0.19712194
 1.75268318 1.1084472  0.66899197 0.91281542], Cost: 2.3472
```



```
Current parameters: [1.69638074 1.99188054 2.01014367 1.02166077 2.943378   4.01647325
 2.8308811  1.88912793 0.29390742 3.59282582 3.04087624 0.79746787
 2.00645955 0.2908839  0.01279819 2.67668866 1.34304021 0.19712194
 1.75268318 1.1084472  0.66899197 0.91281542], Cost: 0.3519
Current parameters: [1.69638074 1.99188054 2.01014367 1.02166077 2.943378   4.01647325
 2.8308811  1.88912793 0.29390742 3.59282582 3.04087624 0.79746787
 2.00645955 1.2908839  0.01279819 2.67668866 1.34304021 0.19712194
 1.75268318 1.1084472  0.66899197 0.91281542], Cost: 3.3969
Current parameters: [1.69638074 1.99188054 2.01014367 1.02166077 2.943378   4.01647325
 2.8308811  1.88912793 0.29390742 3.59282582 3.04087624 0.79746787
 2.00645955 0.2908839  1.01279819 2.67668866 1.34304021 0.19712194
 1.75268318 1.1084472  0.66899197 0.91281542], Cost: 1.8661
Current parameters: [1.69638074 1.99188054 2.01014367 1.02166077 2.943378   4.01647325
 2.8308811  1.88912793 0.29390742 3.59282582 3.04087624 0.79746787
 2.00645955 0.2908839  0.01279819 3.67668866 1.34304021 0.19712194
 1.75268318 1.1084472  0.66899197 0.91281542], Cost: 2.0098
Current parameters: [1.69638074 1.99188054 2.01014367 1.02166077 2.943378   4.01647325
 2.8308811  1.88912793 0.29390742 3.59282582 3.04087624 0.79746787
 2.00645955 0.2908839  0.01279819 2.67668866 2.34304021 0.19712194
 1.75268318 1.1084472  0.66899197 0.91281542], Cost: 3.0433
Current parameters: [1.69638074 1.99188054 2.01014367 1.02166077 2.943378   4.01647325
 2.8308811  1.88912793 0.29390742 3.59282582 3.04087624 0.79746787
 2.00645955 0.2908839  0.01279819 2.67668866 1.34304021 1.19712194
 1.75268318 1.1084472  0.66899197 0.91281542], Cost: 2.5020
Current parameters: [1.69638074 1.99188054 2.01014367 1.02166077 2.943378   4.01647325
 2.8308811  1.88912793 0.29390742 3.59282582 3.04087624 0.79746787
 2.00645955 0.2908839  0.01279819 2.67668866 1.34304021 0.19712194
 2.75268318 1.1084472  0.66899197 0.91281542], Cost: 2.6249
Current parameters: [1.69638074 1.99188054 2.01014367 1.02166077 2.943378   4.01647325
 2.8308811  1.88912793 0.29390742 3.59282582 3.04087624 0.79746787
 2.00645955 0.2908839  0.01279819 2.67668866 1.34304021 0.19712194
 1.75268318 2.1084472  0.66899197 0.91281542], Cost: 2.5776
Current parameters: [1.69638074 1.99188054 2.01014367 1.02166077 2.943378   4.01647325
 2.8308811  1.88912793 0.29390742 3.59282582 3.04087624 0.79746787
 2.00645955 0.2908839  0.01279819 2.67668866 1.34304021 0.19712194
 1.75268318 1.1084472  1.66899197 0.91281542], Cost: 2.0825
Current parameters: [1.69638074 1.99188054 2.01014367 1.02166077 2.943378   4.01647325
 2.8308811  1.88912793 0.29390742 3.59282582 3.04087624 0.79746787
 2.00645955 0.2908839  0.01279819 2.67668866 1.34304021 0.19712194
 1.75268318 1.1084472  0.66899197 1.91281542], Cost: 1.9769
Current parameters: [ 1.63697207  1.91528268  1.81279155  0.90296205  2.8267488   4.10687249
```

```
  2.81755277  1.79217354  0.22823256  3.61042947  2.83532827  0.5794202
  2.06543453 -0.13187258 -0.19742834  2.44651268  0.96938328 -0.10138327
  1.43711662  0.79944254  0.42872647  0.68720441], Cost: 1.3194
Current parameters: [ 1.66667641  1.95358161  1.91146761  0.96231141  2.8850634   4.06167287
  2.82421694  1.84065074  0.26106999  3.60162764  2.93810226  0.68844403
  2.03594704  0.07950566 -0.09231508  2.56160067  1.15621175  0.04786933
  1.5948999   0.95394487  0.54885922  0.80000991], Cost: 2.1238
Current parameters: [1.44881325 1.99188054 2.01014367 1.02166077 2.943378   4.01647325
 2.8308811  1.88912793 0.29390742 3.59282582 3.04087624 0.79746787
 2.00645955 0.3256738  0.01279819 2.67668866 1.34304021 0.19712194
 1.75268318 1.1084472  0.66899197 0.91281542], Cost: 0.2220
Current parameters: [ 1.50437268  1.97990947  1.97930055  1.00310998  2.92515064  4.03060126
  2.82879809  1.87397544  0.28364344  3.595577    3.00875224  0.76339035
  2.01567644  0.80205573 -0.020057    2.64071567  1.28464335  0.15047014
  1.70336496  1.0601545   0.63144215  0.87755587], Cost: 1.8676
Current parameters: [1.44881325 1.75881952 2.10060116 1.02166077 2.943378   4.01647325
 2.8308811  1.88912793 0.29390742 3.59282582 3.04087624 0.79746787
 2.00645955 0.3256738  0.01279819 2.67668866 1.34304021 0.19712194
 1.75268318 1.1084472  0.66899197 0.91281542], Cost: 0.5046
Current parameters: [ 1.44046617e+00  2.18203486e+00  2.46723883e+00  1.01267512e+00
  2.93454902e+00  4.02331659e+00  2.82987213e+00  1.88178835e+00
  2.88935742e-01  3.59415844e+00  3.02531599e+00  7.80961374e-01
  2.01092404e+00  3.05518435e-01 -3.11623502e-03  2.65926403e+00
  1.31475389e+00  1.74524702e-01  1.72879438e+00  1.08505515e+00
  6.50803558e-01  8.95736371e-01], Cost: 1.2546
Current parameters: [1.44881325 1.99188054 2.01014367 1.19687608 2.76505359 4.01647325
 2.8308811  1.88912793 0.29390742 3.59282582 3.04087624 0.79746787
 2.00645955 0.3256738  0.01279819 2.67668866 1.34304021 0.19712194
 1.75268318 1.1084472  0.66899197 0.91281542], Cost: 0.2198
Current parameters: [1.44230101e+00 1.99611870e+00 1.99544840e+00 1.55134031e+00
 3.11323743e+00 4.02181229e+00 2.83009392e+00 1.88340173e+00
 2.90028607e-01 3.59386550e+00 3.02873642e+00 7.84589805e-01
 2.00994266e+00 3.09948956e-01 3.82048273e-04 2.66309429e+00
 1.32097174e+00 1.79491990e-01 1.73404558e+00 1.09019715e+00
 6.54801706e-01 8.99490660e-01], Cost: 2.3071
Current parameters: [1.44881325 1.99188054 2.01014367 1.19687608 2.76505359 3.98000784
 2.58355485 1.88912793 0.29390742 3.59282582 3.04087624 0.79746787
 2.00645955 0.3256738  0.01279819 2.67668866 1.34304021 0.19712194
 1.75268318 1.1084472  0.66899197 0.91281542], Cost: 0.4821
Current parameters: [1.44541007 1.99409533 2.00246418 1.18560644 2.75392803 3.52358173
 2.90809654 1.88613552 0.29188042 3.59336914 3.03453218 0.79073802
```



```
 2.00827977 0.31745627 0.00630973 2.66958448 1.33150761 0.18790883
 1.74294349 1.09891004 0.66157639 0.90585213], Cost: 0.3268
Current parameters: [1.44881325 1.99188054 2.01014367 1.19687608 2.76505359 4.01647325
 2.8308811  2.02933422 0.08692385 3.59282582 3.04087624 0.79746787
 2.00645955 0.3256738  0.01279819 2.67668866 1.34304021 0.19712194
 1.75268318 1.1084472  0.66899197 0.91281542], Cost: 0.2096
Current parameters: [1.44442037 1.99473943 2.00023086 1.18232905 2.75069254 4.01774552
 2.83655932 2.44190388 0.36611639 3.59352714 3.03268723 0.78878087
 2.00880911 0.31506648 0.00442278 2.66751846 1.32815374 0.18522951
 1.74011103 1.09613648 0.65941981 0.90382709], Cost: 1.6053
Current parameters: [1.44881325 1.99188054 2.01014367 1.19687608 2.76505359 4.01647325
 2.8308811  2.02933422 0.08692385 3.841914   3.06220879 0.79746787
 2.00645955 0.3256738  0.01279819 2.67668866 1.34304021 0.19712194
 1.75268318 1.1084472  0.66899197 0.91281542], Cost: 0.2238
Current parameters: [ 1.44113867e+00  1.99687515e+00  1.99282549e+00  1.17146169e+00
  2.73996412e+00  4.01869597e+00  2.84080124e+00  2.00874714e+00
  7.25008900e-02  3.55013001e+00  3.53295558e+00  7.82291241e-01
  2.01056435e+00  3.07142278e-01 -1.83406868e-03  2.66066787e+00
  1.31703281e+00  1.76345278e-01  1.73071901e+00  1.08693976e+00
  6.52268931e-01  8.97112367e-01], Cost: 1.6269
Current parameters: [ 1.44881325  1.99188054  2.01014367  1.19687608  2.76505359  4.01647325
  2.8308811   2.02933422  0.08692385  3.59282582  3.04087624  0.97098734
  2.00645955  0.3256738  -0.16717679  2.67668866  1.34304021  0.19712194
  1.75268318  1.1084472   0.66899197  0.91281542], Cost: 0.0341
Current parameters: [1.43757862 1.99919202 1.98479202 1.1596726  2.72832576 4.01972703
 2.84540294 1.99919731 0.06581045 3.59512834 3.00454054 1.32804451
 2.01246846 0.29854597 0.16328411 2.65323624 1.30496864 0.16670751
 1.72053039 1.076963   0.64451154 0.88982812], Cost: 2.6986
Current parameters: [ 1.44881325  1.99188054  2.01014367  1.19687608  2.76505359  4.01647325
  2.8308811   2.02933422  0.08692385  3.59282582  3.04087624  0.97098734
  2.00645955  0.3256738  -0.16717679  2.88954391  1.21191949  0.19712194
  1.75268318  1.1084472   0.66899197  0.91281542], Cost: 0.3137
Current parameters: [ 1.43332457  2.00196055  1.9751925   1.14558532  2.71441859  4.02095909
  2.8509017   1.98778582  0.05781575  3.5960002   2.99078185  0.91125151
  2.01474376  0.28827389 -0.24378025  2.90334161  1.75255481  0.15519095
  1.70835558  1.06504135  0.6352419   0.88112387], Cost: 2.2010
Current parameters: [ 1.44881325  1.99188054  2.01014367  1.19687608  2.76505359  4.01647325
  2.8308811   2.02933422  0.08692385  3.59282582  3.04087624  0.97098734
  2.00645955  0.3256738  -0.16717679  2.67668866  1.34304021  0.37874011
  1.58088434  1.1084472   0.66899197  0.91281542], Cost: 0.7960
Current parameters: [ 1.43431572  2.00131551  1.97742909  1.14886751  2.71765882  4.02067203
```



```
  2.84962054  1.99044458  0.05967843  3.59579707  2.99398748  0.91507412
  2.01421364  0.29066718 -0.23887825  2.61771523  1.28622172  0.47636196
  2.12882926  1.06781898  0.63740163  0.88315187], Cost: 0.1756
Current parameters: [ 1.44881325  1.99188054  2.01014367  1.19687608  2.76505359  4.01647325
  2.8308811   2.02933422  0.08692385  3.59282582  3.04087624  0.97098734
  2.00645955  0.3256738  -0.16717679  2.67668866  1.34304021  0.19712194
  1.75268318  1.26190392  0.4716318   0.91281542], Cost: 0.0983
Current parameters: [ 1.43477979  2.0010135   1.97847629  1.15040427  2.71917593  4.02053763
  2.84902069  1.99168944  0.06055056  3.59570196  2.99548839  0.9168639
  2.01396543  0.29178774 -0.23658308  2.61960297  1.28804048  0.14571558
  1.7766934   1.47647281  0.96089005  0.8841014 ], Cost: 1.7250
Current parameters: [ 1.44881325  1.99188054  2.01014367  1.19687608  2.76505359  4.01647325
  2.8308811   2.02933422  0.08692385  3.59282582  3.04087624  0.97098734
  2.24833244  0.3256738  -0.16717679  2.67668866  1.34304021  0.19712194
  1.75268318  1.1084472   0.66899197  0.97604127], Cost: 0.4298
Current parameters: [ 1.44096303  1.99698946  1.99242915  1.17088007  2.73938994  4.01874683
  2.84102826  2.00827599  0.07217081  3.59443471  3.01548659  0.94071106
  1.86759387  0.30671818 -0.20600219  2.64475532  1.31227374  0.16836558
  1.76611433  1.07641713  0.6472986   1.38218774], Cost: 1.2623
Current parameters: [ 1.32259484  2.07015097  1.8960275   1.1044104   2.83438254  4.02590687
  2.82949022  2.01921653  0.08007034  3.59466285  3.01942626  0.94823294
  2.0126139   0.24855001 -0.18911501  2.65266861  1.30404719  0.16597139
  1.71975219  1.07620098  0.64391903  0.88927175], Cost: 0.2470
Current parameters: [ 1.58230469  1.86017997  2.04954787  1.2261124   2.5737118   4.04687638
  2.84496329  1.96374996  0.04163306  3.6008116   2.93593562  0.85374513
  2.00984703  0.21405436 -0.29727787  2.55297294  1.1795265   0.05909584
  1.68439503  0.9615597   0.56046783  0.79029308], Cost: 2.7589
Current parameters: [ 1.41213264  2.03415     1.96077384  1.19358088  2.68800253  4.08996364
  2.93115389  1.96451203  0.17577859  3.59340254  3.03414212  0.9638437
  2.00839168  0.47618612 -0.1740642   2.66914767  1.33079853  0.18734236
  1.74234465  1.09832365  0.66112044  0.90542399], Cost: 0.2884
Current parameters: [ 1.49363188  1.99632168  1.98732619  1.09726576  2.92883335  3.89554534
  2.67139249  2.11017976 -0.08749555  3.59446915  3.01290786  0.93687372
  1.9944228   0.67601038 -0.21287778  2.64071342  1.31265819  0.16729564
  1.77943069  1.07456389  0.64691618  0.87660755], Cost: 2.1011
Current parameters: [ 1.40420232  2.10066075  2.16757286  1.17842225  2.75720016  4.04109197
  2.86476796  2.02055941  0.02108266  3.51036805  2.98756113  0.90745748
  2.00736532  0.32761571 -0.1079856   2.67315352  1.33730143  0.19253737
  1.74783658  1.10370138  0.66530188  0.90935039], Cost: 0.1320
Current parameters: [ 1.41116005  2.13218176  2.26361761  1.13447258  2.8250155   3.97969582
  2.7875619   2.01127844  0.27703569  3.80862922  3.15638601  1.10865784
```



```
  1.99724037  0.29979521 -0.35612184  2.65691113  1.32976759  0.18278494
  1.77670003  1.09158408  0.65875033  0.89279418], Cost: 1.0358
Current parameters: [ 1.43233176  2.00698781  1.99783688  1.38677214  2.90719901  4.04027108
  2.87756091  1.9987983   0.10332672  3.58156691  3.0289327   0.95516706
  1.99800374  0.3130948  -0.17227605  2.66870074  1.34391793  0.19507827
  1.7793202   1.10484533  0.66830134  0.90451842], Cost: 0.3236
Current parameters: [ 1.43923547  2.00001402  2.00523027  1.20581756  2.75013369  3.90374433
  2.87405494  2.01515645  0.09789098  3.58313248  3.0457986   0.96284209
  2.00692838  0.32478    -0.1621976   2.67485886  1.3400698   0.19474895
  1.75017456  1.10599075  0.66708196  0.91102191], Cost: 0.0430
Current parameters: [ 1.44169302  1.99694255  2.00068281  1.19704308  2.75188681  4.04160041
  2.84457493  2.01895127  0.04477342  3.53164775  3.27186186  0.93423494
  2.00081253  0.31209608 -0.15046268  2.66792093  1.3392127   0.19200125
  1.7689135   1.10202931  0.66558759  0.90387021], Cost: 0.1784
Current parameters: [ 1.44109409  1.9988138   2.00213127  1.20459043  2.75260223  4.020925
  2.85364322  2.24134868  0.19439536  3.5451926   3.03991003  0.93193708
  1.99946244  0.31444796 -0.14444873  2.67008982  1.34378078  0.19544296
  1.77473012  1.10547966  0.66842999  0.90596073], Cost: 0.2443
Current parameters: [ 1.43892377  2.02744163  2.0732531   1.18424956  2.78192622  4.00819303
  2.82119634  2.02714848  0.13569996  3.64521755  3.07296622  1.00419285
  2.00383926  0.31864253 -0.21406902  2.67139228  1.33968544  0.19340241
  1.75965677  1.10403397  0.66635954  0.90744704], Cost: 0.0894
Current parameters: [ 1.49727676  2.02006795  1.99896475  1.18323064  2.77815063  4.01815006
  2.84217406  2.02472593  0.09303171  3.58909709  3.04016973  0.96804772
  2.00652324  0.31855914 -0.16476661  2.67846661  1.34592645  0.1967996
  1.75234242  1.10811353  0.66873253  0.9125718 ], Cost: 0.0454
Current parameters: [ 1.43626337  1.991938    2.00590972  1.19055578  2.75342209  4.01795807
  2.83571508  2.02277489  0.08411316  3.59480296  3.03616951  0.95924058
  2.0010593   0.3102523  -0.18600176  2.73369053  1.44633753  0.18959414
  1.76711251  1.09949915  0.66367477  0.90187653], Cost: 0.2385
Current parameters: [ 1.44829308  2.0270535   1.99544965  1.22134205  2.73669982  4.01329975
  2.80900744  2.03611369  0.07387794  3.60044164  3.03983698  0.97582828
  2.00673777  0.32441776 -0.17292671  2.67523922  1.34068727  0.20722888
  1.76336779  1.10698946  0.66785851  0.91175109], Cost: 0.0600
Current parameters: [ 1.44409347  1.97522385  2.01009777  1.17105283  2.76834409  4.01936371
  2.84698193  2.0144015   0.08992003  3.58998659  3.03074442  0.96481564
  2.00594369  0.3139543  -0.16983461  2.66616121  1.32966817  0.27251183
  1.84011494  1.09828584  0.66165763  0.90387459], Cost: 0.1060
Current parameters: [ 1.43881251  2.00144886  2.00466665  1.19047644  2.77304014  4.01473701
  2.83144633  2.02975003  0.08760546  3.5926334   3.04341832  0.97553109
  2.00497315  0.33007484 -0.16283102  2.67995886  1.3483489   0.20260255
```

```
  1.75847704  1.15429969  0.70464442  0.91850172], Cost: 0.0902
Current parameters: [ 1.47348258  1.96135364  2.02121299  1.20419611  2.72160121  4.02589456
  2.84152899  2.01521801  0.08210795  3.59553609  3.02394111  0.93287398
  2.00211056  0.28155429 -0.21508754  2.64730232  1.31439162  0.15978697
  1.75285044  1.13149528  0.68981706  0.87350984], Cost: 0.1244
Current parameters: [ 1.45203523  1.99199519  2.01005576  1.19295165  2.77822921  4.00875891
  2.82001265  2.03657108  0.07747033  3.59274875  3.04168149  0.97125009
  2.01976148  0.35048618 -0.16691225  2.67745476  1.34428387  0.19771852
  1.75331386  1.10947989  0.66979493  0.86192841], Cost: 0.0683
Current parameters: [ 1.46445955  1.98147154  2.01097009  1.17795695  2.78959995  3.99350792
  2.80282833  2.04519466  0.04923249  3.59472403  3.03497898  0.94031698
  1.97616659  0.38307899 -0.20927167  2.65796039  1.33407163  0.17253015
  1.77126864  1.09665976  0.66309327  0.96577769], Cost: 0.2507
Current parameters: [ 1.44858237  1.9920308   2.00962267  1.19611151  2.76429879  4.02220346
  2.86959109  2.05048992  0.05434391  3.61232562  3.04179547  0.99172312
  2.00658304  0.3251163  -0.17428177  2.67620668  1.3422578   0.19649689
  1.75202241  1.10780017  0.66848887  0.91234301], Cost: 0.0585
Current parameters: [ 1.45977922  1.97501357  2.01500988  1.1902824   2.75022131  4.01152689
  2.81161482  2.01355017  0.09228011  3.58392497  3.03238583  1.04461151
  1.98356869  0.30890442 -0.09858394  2.65248477  1.33553783  0.15312032
  1.77228798  1.09588982  0.66407273  0.90706097], Cost: 0.1193
Current parameters: [ 1.44764041  1.99294902  2.00911528  1.19754061  2.76374983  4.0171052
  2.83353085  2.05124459  0.09828116  3.54797002  3.03716271  0.99456577
  2.00660448  0.32470286 -0.19360885  2.67646846  1.34268274  0.19810128
  1.7537185   1.10813817  0.66875168  0.91226101], Cost: 0.0543
Current parameters: [ 1.44801785  1.99047558  2.00877414  1.19978012  2.75655657  4.01791395
  2.83872026  2.12373842  0.13669819  3.60910087  3.04240655  0.92991322
  1.99907269  0.31741605 -0.12619568  2.66900927  1.34121867  0.18847528
  1.76568355  1.1047547   0.66774316  0.90982378], Cost: 0.0483
Current parameters: [ 1.44116679  2.00022683  1.99875668  1.19606692  2.74410731  4.03476154
  2.85512038  2.01331018  0.11030096  3.59347067  3.03902643  0.96999006
  2.00362821  0.36246912 -0.16698609  2.67195617  1.33978166  0.18929746
  1.75361333  1.10486761  0.66688289  0.91065928], Cost: 0.0707
Current parameters: [ 1.44896887  1.99157655  2.00984384  1.18537783  2.75506851  4.0177273
  2.82956065  2.03004595  0.08136293  3.58687361  3.06654589  0.96794408
  2.00657029  0.32493073 -0.16505813  2.67633991  1.34247407  0.19697527
  1.75252813  1.10797578  0.66862542  0.91239179], Cost: 0.0240
Current parameters: [ 1.44596883  1.99264253  2.00717888  1.22686418  2.78223228  4.02639725
  2.84054624  2.02200445  0.081434    3.57697188  3.09358964  0.96062162
  2.00131038  0.31860575 -0.16057536  2.67015014  1.34027532  0.19066026
  1.76217901  1.10453752  0.66714828  0.90946993], Cost: 0.0294
```



```
Current parameters: [ 1.4543821   1.97837681  1.99074087  1.18761708  2.75602147  4.01473999
  2.82544871  2.03111071  0.08935231  3.5968793   3.07301532  0.975653
  2.01371989  0.32469509 -0.17224058  2.67676888  1.34317044  0.19753157
  1.75311623  1.10855165  0.66907319  0.91470989], Cost: 0.0259
Current parameters: [ 1.44838773  1.99499137  2.021448    1.18724179  2.75346323  4.02003144
  2.83135989  2.02905028  0.0791136   3.5814441   3.06548724  0.96450024
  2.06328898  0.32336397 -0.15751293  2.67069001  1.34155002  0.18688381
  1.7590152   1.1055579   0.66800237  0.92779186], Cost: 0.0406
Current parameters: [ 1.44896887  1.99157655  2.00984384  1.18537783  2.75506851  4.0177273
  2.82956065  2.03004595  0.08136293  3.58687361  3.06654589  0.96794408
  2.00657029  0.32493073 -0.16505813  2.70131835  1.32708713  0.1891549
  1.75992569  1.10797578  0.66862542  0.91239179], Cost: 0.0108
Current parameters: [ 1.45527285  1.98262581  2.01830298  1.19474915  2.75134756  4.02062338
  2.82913621  2.02845316  0.08619411  3.58452719  3.07290952  0.96851261
  2.0095774   0.31953929 -0.15164974  2.71612126  1.30526223  0.22158179
  1.72110292  1.1010738   0.66729883  0.9157    ], Cost: 0.0212
Current parameters: [ 1.44441834  1.99439841  2.00572963  1.18204418  2.75756801  4.01806741
  2.82951051  2.02968117  0.08111584  3.58693984  3.06577256  0.96712372
  2.00679217  0.3221502  -0.16584906  2.70045236  1.32568133  0.18803183
  1.75873843  1.0884463   0.6913431   0.91154298], Cost: 0.0188
Current parameters: [ 1.42001077  2.00737305  1.9848452   1.16620092  2.77086382  4.02133496
  2.82902984  2.02682534  0.08171463  3.58632865  3.06389311  0.96248014
  2.00940297  0.30327431 -0.16481375  2.69634239  1.31121368  0.1829257
  1.7466736   1.10940519  0.64572178  0.90790419], Cost: 0.0084
Current parameters: [ 1.42430055  2.00666648  1.98545438  1.16415885  2.77736158  4.01711913
  2.82333969  2.03074053  0.07616945  3.58642472  3.06404994  0.96025746
  2.00574083  0.31587076 -0.1677731   2.68375069  1.29211771  0.18116634
  1.7476109   1.10981328  0.64624404  0.91796583], Cost: 0.0234
Current parameters: [ 1.41796079  1.99714089  1.9843007   1.16073645  2.77743326  4.01672692
  2.81726112  2.02700989  0.06990288  3.58378181  3.06805981  0.94115496
  1.99895208  0.30521935 -0.1711612   2.72421109  1.33891641  0.17305482
  1.7404274   1.09344413  0.62534538  0.92893595], Cost: 0.0402
Current parameters: [ 1.42262903  2.00423748  1.98603144  1.16876718  2.7652247   4.02398738
  2.83371448  2.02642532  0.08057146  3.587789    3.06213536  0.94335106
  2.01206507  0.29764317 -0.1841978   2.69502447  1.30731103  0.18297888
  1.74306235  1.11505131  0.64984318  0.90289654], Cost: 0.0439
Current parameters: [ 1.42930401  1.98938737  1.99032034  1.16498589  2.75457379  4.02152233
  2.81977966  2.01464162  0.08130803  3.58255771  3.06093728  0.98214002
  1.9970795   0.27698121 -0.13945133  2.68988293  1.30266955  0.15414175
  1.74940408  1.10675407  0.64016619  0.89891159], Cost: 0.0355
Current parameters: [ 1.42129065  1.99789237  1.97231175  1.1648202   2.76798443  4.0234219
```



```
  2.83335068  2.02500627  0.07249334  3.57553902  3.05600302  0.95497931
  2.00984401  0.30288352 -0.15594258  2.69579527  1.30985073  0.19509927
  1.75855543  1.10873715  0.64512999  0.90761078], Cost: 0.0008
Current parameters: [ 1.41463713  1.99823491  1.99164127  1.14981861  2.77634185  4.02205818
  2.83346145  2.0164152   0.08863794  3.5881584   3.06463494  0.96442103
  2.00707925  0.29178169 -0.16067847  2.69744856  1.30540917  0.2242857
  1.79283808  1.09621104  0.63192925  0.90749933], Cost: 0.0093
Current parameters: [ 1.420514    1.99859023  1.97239095  1.16349032  2.77076718  4.02306468
  2.83138031  2.0014431   0.06067685  3.57161981  3.05584245  0.96509131
  2.01139428  0.30503849 -0.16541305  2.69794246  1.3107217   0.19756716
  1.75572585  1.1132617   0.64824956  0.90935618], Cost: 0.0088
Current parameters: [ 1.40736244  2.00269258  1.96741782  1.15597965  2.77900365  4.0217101
  2.83034758  2.03347436  0.07999135  3.57476199  3.06345917  0.95827845
  2.00479963  0.30024325 -0.13189122  2.70898104  1.31786156  0.20223166
  1.7647431   1.14792065  0.67010555  0.92031978], Cost: 0.0918
Current parameters: [ 1.42324458  1.99639492  1.97285575  1.1625641   2.77047036  4.05246032
  2.82404146  2.02763932  0.07108821  3.57797424  3.05467212  0.95697697
  2.00957813  0.30525672 -0.15719283  2.69597536  1.31039915  0.19524418
  1.75924123  1.10913892  0.64548141  0.90787619], Cost: 0.0006
Current parameters: [ 1.4140651   2.00227126  1.96178006  1.16153386  2.74971181  4.06417199
  2.84868518  2.00947185  0.09544088  3.57652666  3.05550423  0.95748395
  2.00556512  0.33885337 -0.14898797  2.69688681  1.30801316  0.18823604
  1.75892043  1.09693602  0.63343494  0.90837471], Cost: 0.0089
Current parameters: [ 1.41945437  2.00228615  1.97359666  1.16049713  2.77926927  4.04962021
  2.81926436  2.03051555  0.07173019  3.5801998   3.05816136  0.9636719
  2.01737853  0.3136704  -0.15352478  2.70066868  1.315617    0.19546938
  1.75448507  1.10818343  0.64296509  0.88508439], Cost: 0.0103
Current parameters: [ 1.44255615  1.96625708  1.97083531  1.16017698  2.76582367  4.04767402
  2.8158082   2.02731487  0.0506186   3.56611181  3.0473262   0.93358109
  2.00495754  0.32191185 -0.15474464  2.69030579  1.29424024  0.19687675
  1.77718195  1.09789846  0.6373216   0.8928315 ], Cost: 0.0254
Current parameters: [ 1.42249749  2.01046082  1.96236785  1.17516439  2.75602037  4.04440012
  2.81788847  2.03537537  0.06615136  3.57851609  3.05294608  0.95547109
  2.01026699  0.30254331 -0.15313228  2.69615304  1.31014923  0.2000724
  1.76322563  1.10419621  0.64113924  0.90682142], Cost: 0.0026
Current parameters: [ 1.42601081  2.01016058  1.98376428  1.16544184  2.76365277  4.08121386
  2.79390124  2.00527578  0.06033746  3.5901572   3.06236366  0.97388579
  2.00287442  0.31130231 -0.17583076  2.69977752  1.30427419  0.20003915
  1.76913811  1.11143171  0.64430119  0.91696062], Cost: 0.0102
Current parameters: [ 1.42341565  1.99486202  1.97338961  1.16182512  2.76976007  4.05708062
  2.8418693   2.03637671  0.05379766  3.58775563  3.05605413  0.96814131
```



```
  2.00845329  0.30473374 -0.1591428   2.69751591  1.30942116  0.19534113
  1.75956706  1.10583381  0.64175639  0.90947299], Cost: 0.0035
Current parameters: [ 1.41251558  1.99036756  1.97517264  1.16562561  2.76745091  4.05217088
  2.8218968   2.03131001  0.07113493  3.5733348   3.05437493  0.96048597
  2.00958184  0.3066928  -0.16093315  2.69556021  1.30972522  0.19543367
  1.75944155  1.10917423  0.64550886  0.907862  ], Cost: 0.0011
Current parameters: [ 1.4351801   2.00237381  1.97036504  1.1590751   2.77284521  4.05493525
  2.82684266  2.03462334  0.07648035  3.55714542  3.05374878  0.96772109
  2.00849095  0.30278972 -0.16688387  2.69807517  1.31038085  0.19600007
  1.76026034  1.10597491  0.64188331  0.90936923], Cost: 0.0060
Current parameters: [ 1.42391071  1.99640841  1.97440698  1.15284827  2.76386655  4.0506032
  2.82158102  2.02946361  0.07086289  3.57984763  3.04835038  0.95841646
  2.01539636  0.30662026 -0.15763599  2.69698061  1.31084128  0.19592235
  1.75748348  1.10975397  0.64577832  0.90980642], Cost: 0.0054
Current parameters: [ 1.42301147  1.99711058  1.97826903  1.1705334   2.77448846  4.05638117
  2.82595193  2.02465402  0.06941731  3.57361427  3.05889269  0.95513018
  2.0342633   0.30320337 -0.15201721  2.69364769  1.30916893  0.18976473
  1.76444661  1.1051444   0.64228653  0.91596948], Cost: 0.0053
Current parameters: [ 1.42461292  1.99440272  1.97442699  1.16152905  2.76711967  4.05336779
  2.82359092  2.02750051  0.07068416  3.57599802  3.06267039  0.95634449
  2.01026337  0.30395055 -0.15391347  2.69807175  1.30628857  0.20217185
  1.75093621  1.10759557  0.64511496  0.90846068], Cost: 0.0016
Current parameters: [ 1.42543942  1.99291117  1.97570952  1.17509575  2.77845081  4.05403209
  2.82404541  2.02501389  0.07621012  3.5822236   3.03488222  0.96104819
  2.00848207  0.30414953 -0.15442929  2.70079888  1.30542373  0.20437225
  1.74996774  1.10469661  0.64185937  0.91081002], Cost: 0.0045
Current parameters: [ 1.42056596  2.00292651  1.98230843  1.16145606  2.76999881  4.05393853
  2.82607616  2.02711245  0.06713485  3.57302315  3.05147087  0.95316239
  2.00604032  0.30537332 -0.15363876  2.6939713   1.31115834  0.19552989
  1.75841956  1.10885396  0.64525984  0.90672913], Cost: 0.0018

   Return from subroutine COBYLA because the MAXFUN limit has been reached.

   NFVALS =  100   F = 5.726002E-04    MAXCV = 0.000000E+00
   X = 1.423245E+00   1.996395E+00   1.972856E+00   1.162564E+00   2.770470E+00
       4.052460E+00   2.824041E+00   2.027639E+00   7.108821E-02   3.577974E+00
       3.054672E+00   9.569770E-01   2.009578E+00   3.052567E-01  -1.571928E-01
       2.695975E+00   1.310399E+00   1.952442E-01   1.759241E+00   1.109139E+00
       6.454814E-01   9.078762E-01
Current parameters: [ 1.42324458  1.99639492  1.97285575  1.1625641   2.77047036  4.05246032
  2.82404146  2.02763932  0.07108821  3.57797424  3.05467212  0.95697697
```

```
 2.00957813  0.30525672 -0.15719283  2.69597536  1.31039915  0.19524418
 1.75924123  1.10913892  0.64548141  0.90787619], Cost: 0.0006
Simulating for QAOA depth p = 12
Current parameters: [1.24734355 2.10497448 3.03200371 0.75146828 1.20844916 2.4158666
 1.3751451  0.91268868 1.68774655 1.0654666  0.09190733 2.81669502
 1.07787038 1.77104234 1.12395613 0.12856494 0.07546961 0.53837944
 0.25271041 2.83968829 0.21098196 3.08364905 2.29073656 1.91597357], Cost: 3.2924
Current parameters: [0.24734355 3.10497448 3.03200371 0.75146828 1.20844916 2.4158666
 1.3751451  0.91268868 1.68774655 1.0654666  0.09190733 2.81669502
 1.07787038 1.77104234 1.12395613 0.12856494 0.07546961 0.53837944
 0.25271041 2.83968829 0.21098196 3.08364905 2.29073656 1.91597357], Cost: 1.0825
Current parameters: [0.24734355 3.10497448 4.03200371 0.75146828 1.20844916 2.4158666
 1.3751451  0.91268868 1.68774655 1.0654666  0.09190733 2.81669502
 1.07787038 1.77104234 1.12395613 0.12856494 0.07546961 0.53837944
 0.25271041 2.83968829 0.21098196 3.08364905 2.29073656 1.91597357], Cost: 1.8960
Current parameters: [0.24734355 3.10497448 3.03200371 1.75146828 1.20844916 2.4158666
 1.3751451  0.91268868 1.68774655 1.0654666  0.09190733 2.81669502
 1.07787038 1.77104234 1.12395613 0.12856494 0.07546961 0.53837944
 0.25271041 2.83968829 0.21098196 3.08364905 2.29073656 1.91597357], Cost: 1.8254
Current parameters: [0.24734355 3.10497448 3.03200371 0.75146828 2.20844916 2.4158666
 1.3751451  0.91268868 1.68774655 1.0654666  0.09190733 2.81669502
 1.07787038 1.77104234 1.12395613 0.12856494 0.07546961 0.53837944
 0.25271041 2.83968829 0.21098196 3.08364905 2.29073656 1.91597357], Cost: 1.4112
Current parameters: [0.24734355 3.10497448 3.03200371 0.75146828 1.20844916 3.4158666
 1.3751451  0.91268868 1.68774655 1.0654666  0.09190733 2.81669502
 1.07787038 1.77104234 1.12395613 0.12856494 0.07546961 0.53837944
 0.25271041 2.83968829 0.21098196 3.08364905 2.29073656 1.91597357], Cost: 1.4482
Current parameters: [0.24734355 3.10497448 3.03200371 0.75146828 1.20844916 2.4158666
 2.3751451  0.91268868 1.68774655 1.0654666  0.09190733 2.81669502
 1.07787038 1.77104234 1.12395613 0.12856494 0.07546961 0.53837944
 0.25271041 2.83968829 0.21098196 3.08364905 2.29073656 1.91597357], Cost: 2.5138
Current parameters: [0.24734355 3.10497448 3.03200371 0.75146828 1.20844916 2.4158666
 1.3751451  1.91268868 1.68774655 1.0654666  0.09190733 2.81669502
 1.07787038 1.77104234 1.12395613 0.12856494 0.07546961 0.53837944
 0.25271041 2.83968829 0.21098196 3.08364905 2.29073656 1.91597357], Cost: 1.8547
Current parameters: [0.24734355 3.10497448 3.03200371 0.75146828 1.20844916 2.4158666
 1.3751451  0.91268868 2.68774655 1.0654666  0.09190733 2.81669502
 1.07787038 1.77104234 1.12395613 0.12856494 0.07546961 0.53837944
 0.25271041 2.83968829 0.21098196 3.08364905 2.29073656 1.91597357], Cost: 2.4000
Current parameters: [0.24734355 3.10497448 3.03200371 0.75146828 1.20844916 2.4158666
 1.3751451  0.91268868 1.68774655 2.0654666  0.09190733 2.81669502
```



```
 1.07787038 1.77104234 1.12395613 0.12856494 0.07546961 0.53837944
 0.25271041 2.83968829 0.21098196 3.08364905 2.29073656 1.91597357], Cost: 2.4964
Current parameters: [0.24734355 3.10497448 3.03200371 0.75146828 1.20844916 2.4158666
 1.3751451  0.91268868 1.68774655 1.0654666  1.09190733 2.81669502
 1.07787038 1.77104234 1.12395613 0.12856494 0.07546961 0.53837944
 0.25271041 2.83968829 0.21098196 3.08364905 2.29073656 1.91597357], Cost: 2.6371
Current parameters: [0.24734355 3.10497448 3.03200371 0.75146828 1.20844916 2.4158666
 1.3751451  0.91268868 1.68774655 1.0654666  0.09190733 3.81669502
 1.07787038 1.77104234 1.12395613 0.12856494 0.07546961 0.53837944
 0.25271041 2.83968829 0.21098196 3.08364905 2.29073656 1.91597357], Cost: 1.6460
Current parameters: [0.24734355 3.10497448 3.03200371 0.75146828 1.20844916 2.4158666
 1.3751451  0.91268868 1.68774655 1.0654666  0.09190733 2.81669502
 2.07787038 1.77104234 1.12395613 0.12856494 0.07546961 0.53837944
 0.25271041 2.83968829 0.21098196 3.08364905 2.29073656 1.91597357], Cost: 0.8344
Current parameters: [0.24734355 3.10497448 3.03200371 0.75146828 1.20844916 2.4158666
 1.3751451  0.91268868 1.68774655 1.0654666  0.09190733 2.81669502
 2.07787038 2.77104234 1.12395613 0.12856494 0.07546961 0.53837944
 0.25271041 2.83968829 0.21098196 3.08364905 2.29073656 1.91597357], Cost: 1.0365
Current parameters: [0.24734355 3.10497448 3.03200371 0.75146828 1.20844916 2.4158666
 1.3751451  0.91268868 1.68774655 1.0654666  0.09190733 2.81669502
 2.07787038 1.77104234 2.12395613 0.12856494 0.07546961 0.53837944
 0.25271041 2.83968829 0.21098196 3.08364905 2.29073656 1.91597357], Cost: 1.2443
Current parameters: [0.24734355 3.10497448 3.03200371 0.75146828 1.20844916 2.4158666
 1.3751451  0.91268868 1.68774655 1.0654666  0.09190733 2.81669502
 2.07787038 1.77104234 1.12395613 1.12856494 0.07546961 0.53837944
 0.25271041 2.83968829 0.21098196 3.08364905 2.29073656 1.91597357], Cost: 2.4236
Current parameters: [0.24734355 3.10497448 3.03200371 0.75146828 1.20844916 2.4158666
 1.3751451  0.91268868 1.68774655 1.0654666  0.09190733 2.81669502
 2.07787038 1.77104234 1.12395613 0.12856494 1.07546961 0.53837944
 0.25271041 2.83968829 0.21098196 3.08364905 2.29073656 1.91597357], Cost: 1.4906
Current parameters: [0.24734355 3.10497448 3.03200371 0.75146828 1.20844916 2.4158666
 1.3751451  0.91268868 1.68774655 1.0654666  0.09190733 2.81669502
 2.07787038 1.77104234 1.12395613 0.12856494 0.07546961 1.53837944
 0.25271041 2.83968829 0.21098196 3.08364905 2.29073656 1.91597357], Cost: 2.5128
Current parameters: [0.24734355 3.10497448 3.03200371 0.75146828 1.20844916 2.4158666
 1.3751451  0.91268868 1.68774655 1.0654666  0.09190733 2.81669502
 2.07787038 1.77104234 1.12395613 0.12856494 0.07546961 0.53837944
 1.25271041 2.83968829 0.21098196 3.08364905 2.29073656 1.91597357], Cost: 1.9457
Current parameters: [0.24734355 3.10497448 3.03200371 0.75146828 1.20844916 2.4158666
 1.3751451  0.91268868 1.68774655 1.0654666  0.09190733 2.81669502
 2.07787038 1.77104234 1.12395613 0.12856494 0.07546961 0.53837944
```



```
 0.25271041 3.83968829 0.21098196 3.08364905 2.29073656 1.91597357], Cost: 2.1758
Current parameters: [0.24734355 3.10497448 3.03200371 0.75146828 1.20844916 2.4158666
 1.3751451  0.91268868 1.68774655 1.0654666  0.09190733 2.81669502
 2.07787038 1.77104234 1.12395613 0.12856494 0.07546961 0.53837944
 0.25271041 2.83968829 1.21098196 3.08364905 2.29073656 1.91597357], Cost: 1.9857
Current parameters: [0.24734355 3.10497448 3.03200371 0.75146828 1.20844916 2.4158666
 1.3751451  0.91268868 1.68774655 1.0654666  0.09190733 2.81669502
 2.07787038 1.77104234 1.12395613 0.12856494 0.07546961 0.53837944
 0.25271041 2.83968829 0.21098196 4.08364905 2.29073656 1.91597357], Cost: 1.8362
Current parameters: [0.24734355 3.10497448 3.03200371 0.75146828 1.20844916 2.4158666
 1.3751451  0.91268868 1.68774655 1.0654666  0.09190733 2.81669502
 2.07787038 1.77104234 1.12395613 0.12856494 0.07546961 0.53837944
 0.25271041 2.83968829 0.21098196 3.08364905 3.29073656 1.91597357], Cost: 2.0177
Current parameters: [0.24734355 3.10497448 3.03200371 0.75146828 1.20844916 2.4158666
 1.3751451  0.91268868 1.68774655 1.0654666  0.09190733 2.81669502
 2.07787038 1.77104234 1.12395613 0.12856494 0.07546961 0.53837944
 0.25271041 2.83968829 0.21098196 3.08364905 2.29073656 2.91597357], Cost: 2.3895
Current parameters: [ 2.10770074e-01  3.47272441e+00  2.88317884e+00  6.15551915e-01
  1.14831536e+00  2.34896736e+00  1.11327250e+00  7.71417361e-01
  1.44670049e+00  8.06786646e-01 -1.92507208e-01  2.71359817e+00
  2.12326035e+00  1.73406940e+00  1.04896171e+00 -1.62191018e-01
 -4.45784417e-02  2.31301992e-01  4.94014120e-02  2.59426673e+00
  3.51861883e-04  2.90036647e+00  2.07425196e+00  1.63146208e+00], Cost: 1.2380
Current parameters: [0.24272357 3.15142885 3.01320407 0.73429925 1.20085303 2.40741585
 1.86005478 0.89484321 1.65729748 1.03279001 0.05597993 2.80367177
 2.08360407 1.76637189 1.11448279 0.09183649 0.06030507 0.49958925
 0.22702831 2.8086865  0.18437505 3.06049669 2.26339011 1.88003392], Cost: 0.8649
Current parameters: [-1.42920983e-03  3.08023353e+00  3.03200371e+00  7.51468280e-01
  1.20844916e+00  2.41586660e+00  1.37514510e+00  9.12688683e-01
  1.68774655e+00  1.06546660e+00  9.19073276e-02  2.81669502e+00
  2.07787038e+00  1.77104234e+00  1.12395613e+00  1.28564941e-01
  7.54696082e-02  5.38379440e-01  2.52710414e-01  2.83968829e+00
  2.10981963e-01  3.08364905e+00  2.29073656e+00  1.91597357e+00], Cost: 0.9094
Current parameters: [0.30445851 2.64109009 3.00235445 0.72439069 1.19646916 2.40253877
 1.37658853 0.88454427 1.63972477 1.01393175 0.03524557 2.79615582
 2.08691308 1.7636765  1.10901555 0.07063983 0.05155334 0.47720272
 0.2122067  2.79079481 0.16901972 3.04713505 2.24760797 1.8592925 ], Cost: 0.7929
Current parameters: [0.30445851 2.64109009 3.00235445 0.72439069 1.19646916 2.40253877
 1.37658853 0.88454427 1.63972477 0.82898599 0.20345695 2.79615582
 2.08691308 1.7636765  1.10901555 0.07063983 0.05155334 0.47720272
 0.2122067  2.79079481 0.16901972 3.04713505 2.24760797 1.8592925 ], Cost: 0.7771
```



```
Current parameters: [ 3.19062308e-01  2.63264950e+00  2.96518904e+00  6.90448862e-01
  1.18145219e+00  2.38583230e+00  1.37839786e+00  8.49265166e-01
  1.57952933e+00  1.13470933e+00  5.43889342e-01  2.77040987e+00
  2.09824813e+00  1.75444340e+00  1.09028751e+00 -1.96944673e-03
  2.15742427e-02  4.00517548e-01  1.61435187e-01  2.72950670e+00
  1.16419941e-01  3.00136465e+00  2.19354617e+00  1.78824263e+00], Cost: 1.9397
Current parameters: [0.30445851 2.64109009 2.78963266 0.72439069 1.19646916 2.40253877
 1.37658853 0.88454427 1.77106188 0.82898599 0.20345695 2.79615582
 2.08691308 1.7636765  1.10901555 0.07063983 0.05155334 0.47720272
 0.2122067  2.79079481 0.16901972 3.04713505 2.24760797 1.8592925 ], Cost: 0.9926
Current parameters: [0.31481182 2.63510617 3.27654616 0.70032775 1.18582293 2.39069478
 1.37787125 0.85953327 2.03066075 0.78452527 0.15761759 2.77790332
 2.09494902 1.75713072 1.09573837 0.01916374 0.03029977 0.42283704
 0.17621241 2.74734482 0.13172929 3.01468629 2.20928105 1.80892194], Cost: 1.5541
Current parameters: [0.30445851 2.64109009 3.00235445 0.54423248 1.19646916 2.40253877
 1.37658853 1.05787349 1.63972477 0.82898599 0.20345695 2.79615582
 2.08691308 1.7636765  1.10901555 0.07063983 0.05155334 0.47720272
 0.2122067  2.79079481 0.16901972 3.04713505 2.24760797 1.8592925 ], Cost: 1.1634
Current parameters: [0.31147312 2.63703584 3.00222159 1.08706489 1.18925609 2.39451419
 1.3774576  1.21259023 1.60349104 0.79886281 0.1723997  2.78378932
 2.09235762 1.75924158 1.10001995 0.03576357 0.03715354 0.44036869
 0.18781972 2.76135642 0.14375458 3.02515025 2.22164058 1.82516526], Cost: 0.6890
Current parameters: [0.31147312 2.63703584 3.00222159 1.08706489 1.18925609 2.18479765
 1.3774576  1.21259023 1.60349104 0.79886281 0.1723997  2.91987376
 2.09235762 1.75924158 1.10001995 0.03576357 0.03715354 0.44036869
 0.18781972 2.76135642 0.14375458 3.02515025 2.22164058 1.82516526], Cost: 0.7655
Current parameters: [0.31604698 2.63439228 3.00213496 1.10423886 1.18455283 2.66759782
 1.37802428 1.19854453 1.57986494 0.77922108 0.15214892 3.19659807
 2.09590772 1.75634981 1.0941544  0.01302261 0.0277642  0.41635118
 0.17191827 2.74216121 0.12728052 3.01081513 2.20470862 1.8029127 ], Cost: 1.7988
Current parameters: [0.31147312 2.63703584 3.00222159 1.08706489 1.43459621 2.39451419
 1.3774576  1.21259023 1.60349104 0.79886281 0.1723997  2.78378932
 2.09235762 1.75924158 1.10001995 0.03576357 0.03715354 0.39232468
 0.18781972 2.76135642 0.14375458 3.02515025 2.22164058 1.82516526], Cost: 1.0466
Current parameters: [ 0.32302615  2.63035851  3.00200276  1.13044433  1.22711959  2.35975318
  1.37888896  1.17711243  1.54381428  0.74925009  0.12124859  2.70992701
  2.10132475  1.7519373   1.08520424 -0.02167745  0.01343715  0.9026807
  0.14765451  2.71287155  0.10214302  2.9889414   2.17887243  1.76895788], Cost: 0.9745
Current parameters: [0.31147312 2.63703584 3.00222159 1.08706489 1.18925609 2.39451419
 1.3774576  1.21259023 1.60349104 0.79886281 0.1723997  2.78378932
 2.09235762 1.75924158 1.10001995 0.21061107 0.03715354 0.44036869
```

```
 0.18781972 2.76135642 0.14375458 3.02515025 2.22164058 1.6464802 ], Cost: 1.5990
Current parameters: [0.32683922 2.62815466 3.00193054 1.14476171 1.11234037 2.34828031
 1.37936138 1.16540298 1.52411797 0.73287542 0.10436618 2.68554877
 2.10428435 1.74952652 1.08031432 0.2010932  0.00560956 0.4053226
 0.13439798 2.69686913 0.08840914 2.97699068 2.1647568  2.23176722], Cost: 1.1106
Current parameters: [0.31147312 2.63703584 3.00222159 1.08706489 1.18925609 2.39451419
 1.3774576  1.21259023 1.60349104 0.79886281 0.1723997  2.78378932
 2.09235762 1.75924158 1.10001995 0.03576357 0.03715354 0.44036869
 0.18781972 2.92673441 0.14375458 3.02515025 2.03415693 1.82516526], Cost: 0.7122
Current parameters: [ 0.32324658  2.63023111  3.00199859  1.131272    1.13032352  2.35908995
  1.37891627  1.17643552  1.54267567  0.74830349  0.12027265  2.70851774
  2.10149584  1.75179794  1.08492156 -0.12809527  0.01298465  0.41351649
  0.14688817  3.08862792  0.10134909  2.98825055  2.51486398  1.85240816], Cost: 1.4368
Current parameters: [0.31147312 2.63703584 3.00222159 1.08706489 1.18925609 2.39451419
 1.3774576  1.21259023 1.60349104 0.79886281 0.1723997  2.78378932
 2.09235762 1.75924158 1.10001995 0.03576357 0.03715354 0.44036869
 0.00794461 2.76135642 0.31737757 3.02515025 2.22164058 1.82516526], Cost: 0.7605
Current parameters: [ 0.32110196  2.63147064  3.00203921  1.12321935  1.14105853  2.36554273
  1.37865056  1.18302137  1.55375363  0.75751325  0.12976797  2.72222901
  2.09983125  1.75315385  1.08767185 -0.09824718  0.01738719  0.41840782
  0.50929735  2.69212505  0.46441854  2.9949721   2.16428472  1.84744566], Cost: 3.2499
Current parameters: [ 0.31147312  2.63703584  3.00222159  1.08706489  1.18925609  2.39451419
  1.3774576   1.21259023  1.60349104  0.79886281  0.1723997   2.78378932
  2.09235762  1.75924158  1.10001995  0.03576357 -0.17197933  0.44036869
  0.18781972  2.76135642  0.14375458  3.16212996  2.22164058  1.82516526], Cost: 0.8815
Current parameters: [ 0.31693047  2.63388165  3.00211822  1.1075562   1.16193909  2.378094
  1.37813374  1.19583145  1.5753013   0.77542706  0.14823726  2.7488987
  2.09659346  1.75579123  1.0930214  -0.04018988  0.30725132  0.4279219
  0.11360329  2.72211806  0.05984335  3.41352843  2.18913292  1.83779315], Cost: 1.0652
Current parameters: [0.3176202  2.57522635 3.0272353  1.109909   1.19936307 2.40575826
 1.42147178 1.23633438 1.6440048  0.84234038 0.22020261 2.8011173
 2.09235762 1.75924158 1.28577938 0.08463231 0.05733059 0.49198066
 0.22199084 2.80260559 0.17915618 3.05595543 2.25802618 1.87298446], Cost: 1.8417
Current parameters: [ 0.30133249  2.7624782   2.95082992  1.04947522  1.15610984  2.36398024
  1.28741817  1.15624414  1.50753368  0.69897972  0.06330829  2.73238286
  2.0942804   1.75767536  1.40485672 -0.09902518 -0.01093539  0.32877664
  0.08398876  2.6588742   0.03299706  2.94084016  2.13219692  1.73274066], Cost: 0.8894
Current parameters: [0.31147312 2.63703584 3.00222159 1.08706489 1.18925609 2.39451419
 1.3774576  1.21259023 1.60349104 0.79886281 0.1723997  2.78378932
 1.93446886 1.56540883 1.10001995 0.03576357 0.03715354 0.44036869
 0.18781972 2.76135642 0.14375458 3.02515025 2.22164058 1.82516526], Cost: 0.4996
```



```
Current parameters: [0.31333987 2.63045209 3.00456084 1.09405177 1.18377454 2.39170765
 1.38177593 1.21088508 1.60067706 0.79744906 0.17122343 2.77720556
 1.5459045  1.87798701 1.08344735 0.02248472 0.03559672 0.44231286
 0.17356134 2.7560086  0.12732812 3.01712029 2.21741911 1.83265928], Cost: 1.3304
Current parameters: [0.06461857 2.60822883 3.00392741 1.09139181 1.18083706 2.39981832
 1.376902   1.21708764 1.60625389 0.80182779 0.17565965 2.77695543
 1.94009265 1.57136456 1.09040241 0.02704681 0.04091752 0.43988631
 0.19036468 2.75266737 0.13462804 3.01598699 2.22486952 1.8280656 ], Cost: 0.5065
Current parameters: [ 0.26778184  2.65092262  2.99097931  1.06482823  1.32451932  2.24540141
  1.35902205  1.0949543   1.51568337  0.72156096  0.09139336  2.87217129
  1.85086827  1.35196752  1.14598079  0.10022363 -0.06262983  0.47798256
 -0.01640965  2.90788013  0.1827747   3.15296755  2.1078859   1.84374737], Cost: 0.3880
Current parameters: [ 0.27014832  2.62867214  2.99995987  1.07302985  1.32814799  2.24943832
  1.11670202  1.10347907  1.53022885  0.74201655  0.11388396  2.87839248
  1.8481293   1.35419858  1.15050619  0.11776877 -0.05538575  0.49651259
 -0.00414134  2.92268964  0.19548479  3.16402741  2.12094927  1.8609157 ], Cost: 0.5660
Current parameters: [ 0.283398    2.67644239  2.97639996  1.07915733  1.30424113  2.18930323
  1.37629465  1.03781109  1.4390278   0.97009742  0.36769291  2.8289374
  1.87056615  1.26125874  1.03886958 -0.02238005 -0.1118184   0.44110724
 -0.17741702  2.85691615  0.05077266  3.09341756  2.0223743   1.84162728], Cost: 2.0265
Current parameters: [ 0.36210908  2.66400301  3.0938378   1.07031027  1.29876731  2.25675573
  1.35690027  1.10065233  1.68227941  0.69920101  0.06680895  2.84874498
  1.8476107   1.27169519  1.11626999  0.06294154 -0.05794539  0.46239567
 -0.01891885  2.87278052  0.14809907  3.11946278  2.10662948  1.83843335], Cost: 1.0562
Current parameters: [-0.04933781  2.62870461  3.08810607  1.05942844  1.3947969   2.14809134
  1.36597176  1.01629363  1.6192094   0.61525791 -0.0224049   2.9107743
  1.88254477  1.54374581  1.14214868  0.10660073 -0.12955483  0.49449475
 -0.17161217  2.97936406  0.17072197  3.21035175  2.02475565  1.85925939], Cost: 0.5551
Current parameters: [ 0.26761673  2.65222221  3.09930275  1.10353135  1.31672942  2.24404983
  1.35843015  0.95787528  1.45713381  0.63730792  0.18146007  2.86260533
  1.85175643  1.35457451  1.14376177  0.07850895 -0.05662897  0.45939862
  0.05620271  2.88888733  0.25213664  3.15469517  2.09656405  1.82819807], Cost: 1.5484
Current parameters: [ 0.20156577  2.68137639  2.75550975  1.01210385  1.32520924  2.16747155
  1.35694264  1.25283971  1.46986829  0.76228404 -0.17594832  2.84421463
  1.85598614  1.29904085  1.06354398 -0.00461706 -0.08052035  0.46742778
  0.09480696  2.89113655  0.37142787  3.18142975  2.01424131  1.86784771], Cost: 1.9456
Current parameters: [ 0.26645966  2.65758075  2.97351457  1.07105852  1.31040068  2.37865996
  1.35746046  1.09199388  1.48312845  0.69469912  0.05771722  3.06064496
  1.86260592  1.34876675  1.11247635  0.08111493 -0.06014401  0.49054729
 -0.04699967  2.8997355   0.13802752  3.13465129  2.11117033  1.86727384], Cost: 0.5838
Current parameters: [ 0.28201138  2.53556218  2.98753925  1.04912264  1.32143532  2.24223406
```



```
  1.35936428  1.09365578  1.50144866  0.70548777  0.08048507  2.86703344
  1.85316992  1.35020694  1.14215215  0.08585924 -0.06843082  0.46296106
 -0.02627745  2.89573265  0.17235035  3.14385589  2.09735704  1.82988701], Cost: 0.5692
Current parameters: [ 0.25929975  2.6818442   3.0633036   0.8927284   1.3258492   2.23976259
  1.35848355  1.21486409  1.46061335  0.65154898  0.14465345  2.866754
  1.85178276  1.34911303  1.1377844   0.10711267 -0.06040323  0.49631063
 -0.03013037  2.91934287  0.17122718  3.15660468  2.11461082  1.86458541], Cost: 0.5806
Current parameters: [ 0.26873685  2.6528804   2.932689    0.99258855  1.31974735  2.2522269
  1.35824594  1.02976317  1.55821003  0.71331918  0.10945168  2.8708731
  1.85326422  1.3552751   1.14083529  0.10233874 -0.05578108  0.48708516
 -0.00681139  2.90861715  0.18345668  3.15168428  2.11711321  1.85481659], Cost: 0.3399
Current parameters: [ 0.26304954  2.65592151  2.81851155  1.07945657  1.32723223  2.2374948
  1.35901576  1.10467566  1.60870454  0.56756356  0.21475055  2.86782556
  1.85083272  1.34800796  1.14146029  0.10231991 -0.06493479  0.48555158
 -0.03279298  2.91444405  0.17202378  3.15493055  2.10721255  1.85252115], Cost: 0.1103
Current parameters: [ 0.25924167  2.65578993  2.81887766  1.07363266  1.33424473  2.23883242
  1.35937522  1.10979193  1.61752191  0.57502545  0.22248377  2.88086883
  1.84633937  1.34678042  1.1499873   0.03828842 -0.06530472  0.4902552
 -0.03156028  2.97468496  0.1819084   3.16527589  2.15237945  1.78150116], Cost: 0.1208
Current parameters: [ 0.27300435  2.66888844  2.79721589  1.10810943  1.28747119  2.22451648
  1.35642208  1.07894656  1.55684315  0.52504739  0.15918112  2.79762582
  1.875302    1.34939309  1.08498135  0.12169135 -0.06061586  0.45735271
 -0.06229891  3.01666912  0.10227013  3.10692383  2.23223488  1.93574376], Cost: 0.7240
Current parameters: [ 0.2634903   2.65486641  2.81928865  1.07586441  1.33829336  2.23413518
  1.36022119  1.10902805  1.61591709  0.57385551  0.22132747  2.88029422
  1.82316703  1.35640993  1.15290024  0.11732768 -0.13497396  0.49205353
 -0.02146723  2.93021822  0.19758825  3.06552946  2.10831847  1.85376755], Cost: 0.1906
Current parameters: [ 0.30991884  2.66598081  2.80663477  1.08113451  1.31604147  2.2404455
  1.36213622  1.11085595  1.60913636  0.56946336  0.2088615   2.8613044
  1.64277718  1.45953585  1.11967615  0.08426646 -0.03329     0.48254387
 -0.04851382  2.90244985  0.1471735   3.18894753  2.10355937  1.86145374], Cost: 0.2652
Current parameters: [ 0.25552993  2.65739599  2.8184171   1.0687883   1.31639438  2.23877477
  1.35835034  1.11270417  1.6226278   0.57908145  0.22660815  2.89017149
  1.84477527  1.34448677  1.15147108  0.12388497 -0.06855118  0.37249973
 -0.03190558  2.9333258   0.18891166  3.1696495   2.10908003  1.85810691], Cost: 0.1684
Current parameters: [ 0.25012003  2.61545356  2.80270011  1.09321147  1.32666397  2.25128623
  1.40726971  1.14116468  1.65318412  0.61863313  0.24443462  2.89161731
  1.84631929  1.34554507  1.32259782  0.12972748 -0.03147126  0.55013484
 -0.03200995  2.95470001  0.1751914   3.19772685  2.13725163  1.93694595], Cost: 0.9572
Current parameters: [ 0.26079731  2.62488584  2.83101561  1.08859355  1.3436832   2.23599373
  1.38086415  1.11794248  1.63173271  0.59160118  0.24102055  2.88605621
```



```
  1.84645054  1.3430514   1.06720332  0.14072498 -0.0612755   0.51136905
 -0.0155743   2.94676408  0.20596467  3.18092698  2.12081008  1.8696544 ], Cost: 0.1311
Current parameters: [ 0.25337126  2.67829507  2.78311169  1.07168496  1.30555259  2.24970447
  1.35955474  1.11302905  1.60776366  0.56963608  0.1952957   2.86206781
  1.85246319  1.35268683  1.14173106  0.26887373 -0.04425925  0.50599053
 -0.05363209  2.89937636  0.13147601  3.15317012  2.10876814  1.68350855], Cost: 0.6949
Current parameters: [ 0.25640961  2.65527103  2.8184818   1.07942226  1.38276366  2.22424354
  1.35888068  1.10402077  1.60820042  0.56713347  0.21430377  2.87628208
  1.8453316   1.34050415  1.14151384  0.10251784  0.02510169  0.47504216
 -0.04470634  2.92492811  0.18231557  3.09619444  2.09549966  1.85271753], Cost: 0.2026
Current parameters: [ 0.28472008  2.6670105   2.80427013  1.07657698  1.52710518  2.2938355
  1.36023802  1.11095366  1.61077046  0.57061006  0.20901398  2.83308793
  1.87271915  1.37889887  1.13974585  0.10179054 -0.12704387  0.45232225
  0.00511596  2.86831266  0.11614266  3.20018205  2.1535208   1.85324174], Cost: 0.3580
Current parameters: [ 0.29690748  2.66121167  2.81746336  1.07724071  1.33803232  2.2972536
  1.35779862  1.09570568  1.60209794  0.5616677   0.20854926  2.82896501
  1.85658529  1.34739659  1.14614113  0.10817073 -0.07253586  0.4882767
 -0.08537956  2.96045111  0.21262896  3.16531683  2.05973931  1.85339775], Cost: 0.0881
Current parameters: [ 0.44580403  2.68444869  2.80953652  1.07048237  1.36411793  2.16425035
  1.35601451  1.07896579  1.58897319  0.55025696  0.19315093  2.9168027
  1.92167888  1.41391556  1.15763499  0.12215535 -0.09146112  0.49560112
 -0.12223626  2.98737028  0.22286735  3.19213041  2.03822195  1.8545362 ], Cost: 0.0824
Current parameters: [ 0.44371507  2.68511264  2.808999    1.06941919  1.37058512  2.15712099
  1.35513307  1.0733414   1.58477495  0.54656102  0.18927786  2.92102841
  1.91013282  1.39444303  1.15983246  0.1252373  -0.09623195  0.49739951
 -0.18972163  2.93340007  0.2804475   3.19824159  2.10190939  1.85542465], Cost: 0.0518
Current parameters: [ 0.45399393  2.68269768  2.80680652  1.07744417  1.31741864  2.21488178
  1.36323614  1.12489838  1.62194172  0.57991684  0.21990353  2.88761421
  2.00847354  1.5624356   1.13947001  0.09794113 -0.05866488  0.4842758
 -0.17885579  2.88198835  0.32484385  3.14978563  2.1353548   1.84837823], Cost: 0.4465
Current parameters: [ 0.43969993  2.68480346  2.82357903  1.07459685  1.37085567  2.15183838
  1.35754181  1.0530028   1.57751894  0.59584379  0.26476083  2.9192509
  1.91453158  1.39300432  1.15020027  0.11792183 -0.1067998   0.49458305
 -0.24712967  2.93134989  0.22602775  3.18642647  2.09481033  1.85444385], Cost: 0.3180
Current parameters: [ 0.40113883  2.73623484  2.68253414  1.05409943  1.37103967  2.10088892
  1.34784684  1.13173662  1.55994732  0.6000713   0.13387317  2.91564214
  1.91027887  1.34214955  1.08885857  0.03963833 -0.12849658  0.49830224
 -0.18851294  2.90592276  0.33957471  3.22467646  2.0153828   1.86335299], Cost: 0.2717
Current parameters: [ 0.5146787   2.69015889  2.78524389  1.0735825   1.3544818   2.18024332
  1.35918512  1.07997024  1.53868035  0.56703814  0.2274723   2.91242133
  1.90264121  1.35370302  1.16308828  0.12454423 -0.07953611  0.49253023
```

```
 -0.14444075  2.91654432  0.29244797  3.18672505  2.12148236  1.85021046], Cost: 0.1298
Current parameters: [ 0.38993046  2.71728641  2.8691765   1.10581893  1.37615942  2.13640372
  1.35981078  0.94937431  1.52095564  0.43490799  0.24523826  2.93354278
  1.90652796  1.41117691  1.14998901  0.09547079 -0.10794362  0.49609648
 -0.12787793  2.91574981  0.37291577  3.23851118  2.05718305  1.84042945], Cost: 0.6949
Current parameters: [ 0.46757931  2.69836968  2.83686234  1.07877051  1.3301889   2.14612092
  1.34736145  1.0747151   1.63374636  0.51436648  0.18142809  2.91264839
  1.93006257  1.36007802  1.1549049   0.09690323 -0.09897498  0.51464172
 -0.182859    2.86955947  0.2425867   3.20639042  2.0877036   1.86541615], Cost: 0.1237
Current parameters: [ 0.51654027  2.70916193  2.8645192   1.05738529  1.43133573  2.19091541
  1.37697108  1.08857693  1.7257054   0.56047717  0.14928565  2.92052359
  1.84962736  1.34079084  1.11437576  0.12019052 -0.09373964  0.44472898
 -0.20186533  3.0212551   0.34381203  3.15749716  2.10577094  1.82040027], Cost: 0.2084
Current parameters: [ 0.43983884  2.70195229  2.84273773  0.9826437   1.374018    2.15380816
  1.35503684  1.13473566  1.56023256  0.50957107  0.21299227  2.91905677
  1.90780475  1.39096998  1.15355343  0.12743435 -0.09619316  0.50578059
 -0.19625969  2.94148873  0.27967156  3.20170827  2.10272627  1.86413132], Cost: 0.1154
Current parameters: [ 0.44306079  2.69030185  2.80444097  1.06895425  1.3675661   2.1785969
  1.39929764  1.071285    1.57668095  0.53833263  0.17954036  2.95130898
  1.91259179  1.39350039  1.15341717  0.11883505 -0.0971456   0.49609708
 -0.19707057  2.9293272   0.27065737  3.1931604   2.10006202  1.85619933], Cost: 0.0275
Current parameters: [ 0.44344495  2.69038517  2.79782634  1.07330377  1.36726285  2.22959177
  1.31730932  1.07515959  1.57364328  0.5327956   0.1708894   3.02554934
  1.91229869  1.39034997  1.13948943  0.11627801 -0.0950454   0.5064625
 -0.20377923  2.93475416  0.26504434  3.19244749  2.1025888   1.86896297], Cost: 0.0903
Current parameters: [ 0.44534461  2.67647505  2.77582742  1.03202793  1.36486477  2.1814902
  1.39896635  1.03961037  1.59541216  0.53229087  0.18687143  2.95002358
  1.91404455  1.39487356  1.15043994  0.11801783 -0.09457831  0.49857293
 -0.19369518  2.92812782  0.26965205  3.19137333  2.10317346  1.85977438], Cost: 0.0355
Current parameters: [ 0.45729316  2.58077016  2.81215052  1.07173753  1.37122526  2.17410242
  1.40030015  1.09087648  1.5567464   0.52061923  0.15796606  2.95015908
  1.90876627  1.3867847   1.14639787  0.10164643 -0.10674892  0.4776484
 -0.20898303  2.92195406  0.26977917  3.1877519   2.08231578  1.83661705], Cost: 0.1347
Current parameters: [ 0.43939942  2.68217173  2.80691735  1.06470996  1.37605655  2.18338948
  1.40646257  1.08114942  1.59299935  0.55447127  0.19431074  2.96858567
  1.90701946  1.39281859  1.18881953  0.11938648 -0.09410444  0.50909327
 -0.19118963  2.916283    0.28574852  3.20702549  2.08005555  1.85098108], Cost: 0.0318
Current parameters: [ 0.43934695  2.6821702   2.78097269  1.084059    1.35181094  2.17306181
  1.4183641   1.08661084  1.58442836  0.5298669   0.14692869  2.9444502
  1.91564213  1.37648767  1.20671863  0.12378554 -0.09075266  0.51886384
 -0.20241525  2.97680682  0.26152454  3.21007244  2.14160444  1.92361653], Cost: 0.2902
```



```
Current parameters: [ 0.43793294  2.68909711  2.80555482  1.06817906  1.32146555  2.16790371
  1.39957405  1.07066471  1.57691174  0.53962453  0.17964641  2.9595464
  1.9055672   1.38731051  1.15278106  0.13340387 -0.08110158  0.5036023
 -0.20822863  2.94275229  0.28308843  3.18114106  2.0897117   1.84063999], Cost: 0.0385
Current parameters: [ 0.4401035   2.706456    2.77550983  1.06699571  1.38942891  2.18857925
  1.40028293  1.08214904  1.57889015  0.52706104  0.14874762  2.94309945
  1.92019905  1.38984857  1.15619302  0.19324422 -0.10552089  0.50696721
 -0.19635784  2.90864466  0.24058635  3.21310049  2.10742818  1.7849115 ], Cost: 0.0665
Current parameters: [ 0.43408502  2.6874062   2.82186686  1.05721478  1.37022607  2.18053134
  1.39884409  1.06059578  1.57070467  0.55733608  0.16783039  2.95354079
  1.95642168  1.37067359  1.15731637  0.12287573 -0.10218601  0.49568199
 -0.19093264  2.93368438  0.27927129  3.18465738  2.10211993  1.85397128], Cost: 0.0503
Current parameters: [ 0.45592193  2.69591299  2.84366897  1.03414011  1.35526728  2.18103288
  1.39845857  1.04220028  1.55279571  0.59218202  0.12011356  2.94774315
  1.8637122   1.42218661  1.1515606   0.11528223 -0.08878449  0.5059772
 -0.18628496  2.90692287  0.25661411  3.21335292  2.10297145  1.86181173], Cost: 0.0699
Current parameters: [ 0.44106948  2.69012414  2.80431329  1.07016977  1.38152775  2.17655355
  1.39910354  1.0696777   1.57436039  0.53635646  0.17748864  2.94948546
  1.92125551  1.38772366  1.14828562  0.11319197 -0.04159727  0.48902199
 -0.20572977  2.92906681  0.26573915  3.20939421  2.09607917  1.8550633 ], Cost: 0.0666
Current parameters: [ 0.43358302  2.69762791  2.79868281  1.06918877  1.40498879  2.15096419
  1.39473598  1.07001779  1.57008999  0.52899605  0.16372294  2.95943093
  1.89451823  1.39403286  1.1737105   0.12828721 -0.07404579  0.5164698
 -0.18911537  2.90076162  0.2628151   3.09406506  2.08920182  1.86883753], Cost: 0.0608
Current parameters: [ 0.44645572  2.70460147  2.79894514  1.0620832   1.36075905  2.1871532
  1.3896467   1.06652331  1.56953205  0.53023446  0.17053618  2.9442224
  1.91355008  1.39477345  1.18890626  0.09883914 -0.10037644  0.4655517
 -0.21079156  2.93213823  0.26540287  3.18720212  2.09424348  1.8409992 ], Cost: 0.0848
Current parameters: [ 0.42400675  2.68723673  2.80397676  1.0644683   1.3545704   2.15263588
  1.4016132   1.08178869  1.5896242   0.54784163  0.18566659  2.98231402
  1.90463643  1.38994265  1.14875929  0.16636764 -0.09777252  0.40704719
 -0.1719362   2.90167087  0.26601823  3.19845719  2.10371081  1.89183779], Cost: 0.0318
Current parameters: [ 0.47837871  2.69583339  2.80253515  1.06712087  1.37387531  2.14306131
  1.39892319  1.06774102  1.57409203  0.53607955  0.17632272  2.97535427
  1.92830125  1.40998065  1.156436    0.11927869 -0.10152878  0.49800036
 -0.20388572  2.93662379  0.27186752  3.19981252  2.09873477  1.85336791], Cost: 0.0548
Current parameters: [ 0.40720029  2.6851949   2.80520135  1.06672139  1.36411374  2.1456952
  1.40087455  1.08291997  1.58888538  0.54812751  0.18827801  2.98266782
  1.90025354  1.38833578  1.15487887  0.07007313 -0.09154551  0.49737714
 -0.15649073  2.9379723   0.24899616  3.19175095  2.16654339  1.80554093], Cost: 0.0464
```



```
   Return from subroutine COBYLA because the MAXFUN limit has been reached.

   NFVALS =  100   F = 2.754596E-02    MAXCV = 0.000000E+00
   X = 4.430608E-01   2.690302E+00   2.804441E+00   1.068954E+00   1.367566E+00
       2.178597E+00   1.399298E+00   1.071285E+00   1.576681E+00   5.383326E-01
       1.795404E-01   2.951309E+00   1.912592E+00   1.393500E+00   1.153417E+00
       1.188350E-01  -9.714560E-02   4.960971E-01  -1.970706E-01   2.929327E+00
       2.706574E-01   3.193160E+00   2.100062E+00   1.856199E+00
Current parameters: [ 0.44306079  2.69030185  2.80444097  1.06895425  1.3675661   2.1785969
  1.39929764  1.071285    1.57668095  0.53833263  0.17954036  2.95130898
  1.91259179  1.39350039  1.15341717  0.11883505 -0.0971456   0.49609708
 -0.19707057  2.9293272   0.27065737  3.1931604   2.10006202  1.85619933], Cost: 0.0275
```


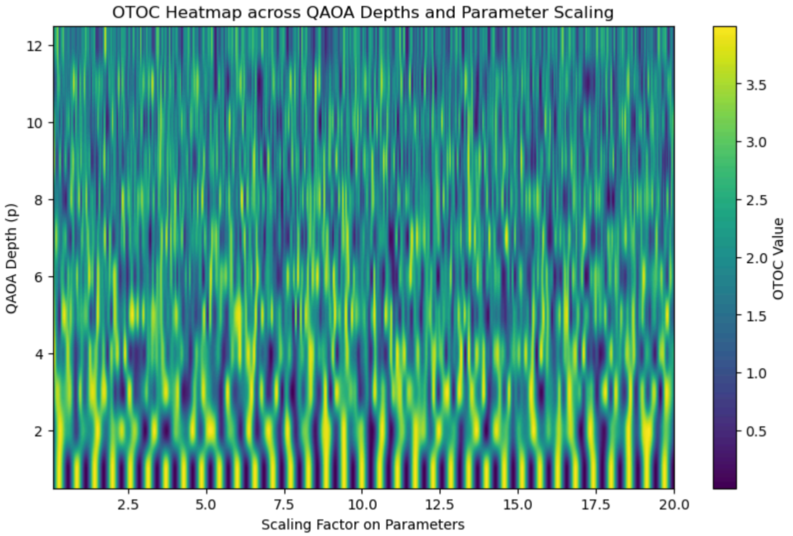

```
Run 2/30 for heatmap generation...
Simulating for QAOA depth p = 1
Current parameters: [3.24382089 0.05807052], Cost: 2.0467
Current parameters: [2.24382089 1.05807052], Cost: 2.8646
Current parameters: [ 2.0022198  -0.91230513], Cost: 1.6307
Current parameters: [ 1.04483444 -1.2011187 ], Cost: 2.8647
Current parameters: [ 2.4737806 -1.0785291], Cost: 1.1036
Current parameters: [ 2.93778444 -1.26480964], Cost: 1.6272
Current parameters: [ 2.39066862 -1.3143095 ], Cost: 1.1468
Current parameters: [ 2.96601588 -1.16630399], Cost: 1.6564
Current parameters: [ 2.71989824 -1.12241654], Cost: 1.2715
Current parameters: [ 2.3690808  -1.01024329], Cost: 1.2173
Current parameters: [ 2.53544106 -1.08873883], Cost: 1.1228
Current parameters: [ 2.45728251 -1.13881228], Cost: 1.0324
Current parameters: [ 2.43110557 -1.1955663 ], Cost: 1.0136
Current parameters: [ 2.36998296 -1.1825173 ], Cost: 1.0005
Current parameters: [ 2.32160705 -1.2220903 ], Cost: 1.0178
Current parameters: [ 2.36464975 -1.15172575], Cost: 1.0057
Current parameters: [ 2.38526079 -1.18579275], Cost: 1.0022
Current parameters: [ 2.36049074 -1.19492852], Cost: 1.0023
Current parameters: [ 2.36747223 -1.17511923], Cost: 1.0003
Current parameters: [ 2.35968189 -1.17570729], Cost: 1.0001
Current parameters: [ 2.35269098 -1.17221984], Cost: 1.0003
Current parameters: [ 2.358877   -1.17952972], Cost: 1.0000
Current parameters: [ 2.36053936 -1.18055502], Cost: 1.0001
Current parameters: [ 2.35693045 -1.1796899 ], Cost: 1.0000
Current parameters: [ 2.35654457 -1.17777528], Cost: 1.0000
Current parameters: [ 2.35554717 -1.17609603], Cost: 1.0000
Current parameters: [ 2.35746848 -1.17745894], Cost: 1.0000
Current parameters: [ 2.35619766 -1.17743166], Cost: 1.0000
Current parameters: [ 2.3564215  -1.17824779], Cost: 1.0000
Current parameters: [ 2.35683175 -1.1785126 ], Cost: 1.0000
Current parameters: [ 2.35617815 -1.17826736], Cost: 1.0000
Current parameters: [ 2.35617316 -1.17836723], Cost: 1.0000
Current parameters: [ 2.35612821 -1.17826487], Cost: 1.0000
Current parameters: [ 2.356184   -1.17816753], Cost: 1.0000
Current parameters: [ 2.35619056 -1.17806775], Cost: 1.0000
Current parameters: [ 2.3562905  -1.17806431], Cost: 1.0000
Current parameters: [ 2.3561377  -1.17798286], Cost: 1.0000
Current parameters: [ 2.3561377  -1.17798286], Cost: 1.0000
```



```
   Normal return from subroutine COBYLA

   NFVALS =   38   F = 1.000000E+00    MAXCV = 0.000000E+00
   X = 2.356138E+00  -1.177983E+00
Simulating for QAOA depth p = 2
Current parameters: [1.9008436  2.10806572 2.24334316 1.24970009], Cost: 2.5564
Current parameters: [1.9008436  3.10806572 2.24334316 1.24970009], Cost: 1.4359
Current parameters: [1.9008436  3.10806572 3.24334316 1.24970009], Cost: 2.4071
Current parameters: [1.9008436  3.10806572 2.24334316 2.24970009], Cost: 2.3953
Current parameters: [1.9070078  3.74249289 1.69346114 0.7064746 ], Cost: 2.6023
Current parameters: [1.9039257  3.42527931 1.96840215 0.97808735], Cost: 3.1305
Current parameters: [2.1508318  3.1056368  2.24334316 1.24970009], Cost: 1.1457
Current parameters: [2.22638763 2.6204773  2.17618622 1.18335612], Cost: 1.8440
Current parameters: [2.18704825 3.12613718 2.48805099 1.21993135], Cost: 1.3122
Current parameters: [2.15064664 3.08657975 2.25986062 1.37212954], Cost: 1.2215
Current parameters: [2.27419585 3.2933369  2.13557764 1.22880569], Cost: 1.1234
Current parameters: [2.34678471 3.21800808 2.08286187 1.18518834], Cost: 1.4725
Current parameters: [2.23745732 3.28942963 2.08553561 1.23489331], Cost: 1.2997
Current parameters: [2.26412472 3.36012098 2.23850378 1.20713382], Cost: 0.8484
Current parameters: [2.29557877 3.38914566 2.30135983 1.30634233], Cost: 0.7657
Current parameters: [2.3548396  3.40518478 2.39814353 1.2564543 ], Cost: 0.7444
Current parameters: [2.38450494 3.451963   2.36963514 1.25142556], Cost: 0.6758
Current parameters: [2.32743247 3.56002966 2.39584336 1.25299385], Cost: 0.6855
Current parameters: [2.48551386 3.50372297 2.34113703 1.29536826], Cost: 0.7425
Current parameters: [2.38554569 3.45796153 2.34327954 1.19508227], Cost: 0.6135
Current parameters: [2.35885123 3.45606062 2.28887609 1.21025818], Cost: 0.6655
Current parameters: [2.40780534 3.43837035 2.33381026 1.19783711], Cost: 0.6314
Current parameters: [2.40466954 3.50062201 2.35511078 1.15532509], Cost: 0.5996
Current parameters: [2.3777298  3.47396218 2.3808478  1.11281204], Cost: 0.6007
Current parameters: [2.40354105 3.52253059 2.40962934 1.17660225], Cost: 0.5744
Current parameters: [2.37254253 3.57014543 2.43456926 1.18409685], Cost: 0.5892
Current parameters: [2.42801253 3.5176826  2.42844884 1.17642188], Cost: 0.5887
Current parameters: [2.40984487 3.52831431 2.40303042 1.18788966], Cost: 0.5857
Current parameters: [2.38655469 3.50154137 2.41774714 1.1900774 ], Cost: 0.5923
Current parameters: [2.39655966 3.53250607 2.41359599 1.16764925], Cost: 0.5644
Current parameters: [2.39457528 3.5324242  2.40726494 1.15350309], Cost: 0.5560
Current parameters: [2.38253838 3.53209832 2.40928435 1.14375273], Cost: 0.5492
Current parameters: [2.38608932 3.53420849 2.41745066 1.13108821], Cost: 0.5435
Current parameters: [2.38217395 3.54417615 2.41455074 1.12008609], Cost: 0.5454
Current parameters: [2.38813327 3.52976891 2.4124056  1.12766843], Cost: 0.5475
Current parameters: [2.37968873 3.53144551 2.43140016 1.13011327], Cost: 0.5349
```



```
Current parameters: [2.38212908 3.53390867 2.44659719 1.13119415], Cost: 0.5325
Current parameters: [2.36842141 3.53312694 2.45117689 1.13708107], Cost: 0.5328
Current parameters: [2.38450579 3.52803444 2.44684311 1.13575697], Cost: 0.5368
Current parameters: [2.37798534 3.53050221 2.45075849 1.11712114], Cost: 0.5231
Current parameters: [2.37391158 3.53487547 2.45523054 1.1033945 ], Cost: 0.5156
Current parameters: [2.37147132 3.52886407 2.46143257 1.09060452], Cost: 0.5105
Current parameters: [2.37753501 3.53542496 2.47005221 1.08111617], Cost: 0.5073
Current parameters: [2.37467079 3.53813484 2.46397278 1.06727298], Cost: 0.5143
Current parameters: [2.37117612 3.53899103 2.47273228 1.08195294], Cost: 0.5029
Current parameters: [2.365523   3.53687558 2.48692019 1.08448492], Cost: 0.4954
Current parameters: [2.36344926 3.54682428 2.49597867 1.09215353], Cost: 0.4972
Current parameters: [2.35444197 3.53426517 2.49204399 1.07508898], Cost: 0.4864
Current parameters: [2.34206788 3.52614393 2.49145483 1.08006097], Cost: 0.4844
Current parameters: [2.34515178 3.52142567 2.49683037 1.08066623], Cost: 0.4855
Current parameters: [2.33262587 3.52672121 2.49378067 1.06784432], Cost: 0.4765
Current parameters: [2.32397916 3.52490144 2.49277736 1.05499688], Cost: 0.4741
Current parameters: [2.31618161 3.5339174  2.50282903 1.05600445], Cost: 0.4629
Current parameters: [2.30416784 3.54325263 2.50385009 1.05941338], Cost: 0.4575
Current parameters: [2.30781353 3.55632041 2.50775772 1.05271912], Cost: 0.4546
Current parameters: [2.30158731 3.55747121 2.52203219 1.05218064], Cost: 0.4453
Current parameters: [2.29207723 3.55969814 2.53335426 1.04764736], Cost: 0.4360
Current parameters: [2.28399741 3.56313273 2.5462347  1.04657268], Cost: 0.4304
Current parameters: [2.27735815 3.56769348 2.54583825 1.03318973], Cost: 0.4192
Current parameters: [2.27369506 3.57793728 2.5482744  1.02224204], Cost: 0.4125
Current parameters: [2.26156032 3.57394656 2.55165004 1.0139013 ], Cost: 0.4034
Current parameters: [2.25110105 3.57558599 2.56065069 1.00675673], Cost: 0.3924
Current parameters: [2.24013864 3.5756798  2.56979243 1.00040172], Cost: 0.3816
Current parameters: [2.22928308 3.57623672 2.57878664 0.99368671], Cost: 0.3709
Current parameters: [2.21860857 3.57759005 2.58725704 0.98616234], Cost: 0.3603
Current parameters: [2.20823883 3.57992358 2.59477632 0.97752381], Cost: 0.3498
Current parameters: [2.19768384 3.57816939 2.60572942 0.97441171], Cost: 0.3412
Current parameters: [2.18860016 3.58248849 2.61303711 0.96494754], Cost: 0.3306
Current parameters: [2.1799264  3.58710532 2.62131386 0.95605446], Cost: 0.3204
Current parameters: [2.17389915 3.59129183 2.6346688  0.95260025], Cost: 0.3140
Current parameters: [2.1603432  3.59531525 2.63482656 0.94595464], Cost: 0.3029
Current parameters: [2.14882971 3.59648583 2.63655038 0.93559905], Cost: 0.2931
Current parameters: [2.13370734 3.59717139 2.64036693 0.9349521 ], Cost: 0.2847
Current parameters: [2.12372541 3.60738059 2.64096724 0.92863439], Cost: 0.2749
Current parameters: [2.11157407 3.61116535 2.63400096 0.92283505], Cost: 0.2706
Current parameters: [2.10446129 3.61695112 2.64426682 0.91544005], Cost: 0.2598
Current parameters: [2.09608415 3.62662477 2.65309147 0.91385571], Cost: 0.2494
```



```
Current parameters: [2.09120098 3.63695218 2.66060456 0.90629308], Cost: 0.2409
Current parameters: [2.07992771 3.63867388 2.67101392 0.90389831], Cost: 0.2323
Current parameters: [2.06839526 3.64857091 2.67462871 0.90425213], Cost: 0.2242
Current parameters: [2.06494352 3.65686559 2.68712548 0.90694565], Cost: 0.2251
Current parameters: [2.07059315 3.65126238 2.67495584 0.89726261], Cost: 0.2223
Current parameters: [2.05786607 3.65086268 2.67244781 0.88856134], Cost: 0.2144
Current parameters: [2.05077683 3.6607913  2.6634466  0.88478192], Cost: 0.2130
Current parameters: [2.04231359 3.65552994 2.66529797 0.87289055], Cost: 0.2112
Current parameters: [2.03858637 3.66481546 2.65722753 0.86400823], Cost: 0.2198
Current parameters: [2.0435057  3.65088673 2.65913405 0.87313684], Cost: 0.2183
Current parameters: [2.03044468 3.65649549 2.67174565 0.88068571], Cost: 0.1993
Current parameters: [2.02643282 3.65735473 2.68659333 0.8833037 ], Cost: 0.1935
Current parameters: [2.01718037 3.66989586 2.68761755 0.88285213], Cost: 0.1854
Current parameters: [2.00330902 3.67231278 2.69041882 0.8890196 ], Cost: 0.1812
Current parameters: [1.99512169 3.67683993 2.69688773 0.87830669], Cost: 0.1719
Current parameters: [1.98812748 3.68616209 2.70504766 0.87184647], Cost: 0.1647
Current parameters: [1.97782756 3.69076991 2.69998598 0.86229662], Cost: 0.1553
Current parameters: [1.96634048 3.69243088 2.70064176 0.85185638], Cost: 0.1483
Current parameters: [1.9562419  3.70341348 2.69600152 0.85175565], Cost: 0.1424
Current parameters: [1.95846097 3.71240738 2.69226516 0.83974043], Cost: 0.1466
Current parameters: [1.95576517 3.70001241 2.68899922 0.85129926], Cost: 0.1467
Current parameters: [1.9445433  3.70586805 2.70573255 0.85431798], Cost: 0.1342
Current parameters: [1.94119908 3.71503021 2.71171677 0.86495756], Cost: 0.1349
Current parameters: [1.9445433  3.70586805 2.70573255 0.85431798], Cost: 0.1342

   Return from subroutine COBYLA because the MAXFUN limit has been reached.

   NFVALS =  100   F = 1.342151E-01    MAXCV = 0.000000E+00
   X = 1.944543E+00   3.705868E+00   2.705733E+00   8.543180E-01
Simulating for QAOA depth p = 3
Current parameters: [3.96507609 2.10054709 2.11411036 0.24496546 2.22893504 2.91811553], Cost: 1.6546
Current parameters: [3.96507609 3.10054709 2.11411036 0.24496546 2.22893504 2.91811553], Cost: 1.9227
Current parameters: [3.96507609 2.10054709 3.11411036 0.24496546 2.22893504 2.91811553], Cost: 1.7262
Current parameters: [3.96507609 2.10054709 2.11411036 1.24496546 2.22893504 2.91811553], Cost: 3.2027
Current parameters: [3.96507609 2.10054709 2.11411036 0.24496546 3.22893504 2.91811553], Cost: 2.6403
Current parameters: [3.96507609 2.10054709 2.11411036 0.24496546 2.22893504 3.91811553], Cost: 0.4913
Current parameters: [ 4.26268588  1.98367991  2.08288641 -0.42974297  1.79935721  4.42509381], Cost: 1.7266
Current parameters: [3.47440984 2.08877583 2.11096538 0.17700651 2.18566648 3.96918012], Cost: 0.8804
Current parameters: [3.96507609 2.1650773  1.8725822  0.24496546 2.22893504 3.91811553], Cost: 0.6055
Current parameters: [3.97397421 2.58000179 2.25048229 0.21786597 2.21168115 3.93847819], Cost: 1.5540
Current parameters: [3.96507609 2.10054709 2.11411036 0.37923281 2.01805052 3.91811553], Cost: 0.8072
```

```
Current parameters: [4.03028613 1.83542433 2.10391082 0.45828932 2.55692506 4.06734359], Cost: 1.7140
Current parameters: [4.03198229 2.01515295 2.10626198 0.03922125 2.14537476 3.95495034], Cost: 1.6651
Current parameters: [3.94487724 2.08253767 2.11586585 0.26703676 2.26380085 3.67304041], Cost: 0.2114
Current parameters: [3.94136555 2.04873884 2.11574109 0.35371117 2.34720935 3.67396727], Cost: 0.0578
Current parameters: [3.94208298 2.15474317 2.15195718 0.38294362 2.3908411  3.65614318], Cost: 0.1049
Current parameters: [3.87909077 2.05044711 2.11619749 0.3525283  2.34645623 3.67876396], Cost: 0.0592
Current parameters: [3.93589898 1.97588957 2.12475694 0.34868595 2.43426965 3.62295681], Cost: 0.2300
Current parameters: [3.94136555 2.03356659 2.17252886 0.36511934 2.32929132 3.67396727], Cost: 0.1287
Current parameters: [3.93830388 2.0506449  2.0521261  0.44516681 2.29730769 3.64730613], Cost: 0.3028
Current parameters: [3.95050047 2.05004762 2.1049097  0.35139248 2.38310597 3.79283097], Cost: 0.0843
Current parameters: [3.93954988 2.02733785 2.11025633 0.32757673 2.39590634 3.65498142], Cost: 0.0683
Current parameters: [3.94056911 2.04321968 2.09356127 0.37439063 2.34850466 3.66904741], Cost: 0.0787
Current parameters: [3.94132447 2.05967643 2.11987839 0.36009327 2.35512365 3.6719614 ], Cost: 0.0611
Current parameters: [3.94242601 2.06682296 2.10466464 0.33254396 2.33868992 3.67171264], Cost: 0.0429
Current parameters: [3.9449065  2.06994565 2.09946884 0.3266169  2.34606577 3.70076654], Cost: 0.0161
Current parameters: [3.94800729 2.07679309 2.10318728 0.30867428 2.34297285 3.72474006], Cost: 0.0231
Current parameters: [3.94529431 2.07438427 2.0981178  0.33532591 2.33461351 3.70469878], Cost: 0.0213
Current parameters: [3.94506371 2.0692674  2.07043596 0.32796172 2.3569609  3.70432427], Cost: 0.0037
Current parameters: [3.94546152 2.09353807 2.0747688  0.3364255  2.37418795 3.70392582], Cost: 0.0054
Current parameters: [3.96059766 2.06841605 2.07058197 0.32875419 2.35734907 3.70317878], Cost: 0.0039
Current parameters: [3.94378406 2.05186555 2.0732589  0.34312324 2.3723027  3.71842706], Cost: 0.0112
Current parameters: [3.94360918 2.06797723 2.05001808 0.31557688 2.36472269 3.68582392], Cost: 0.0070
Current parameters: [3.94646702 2.07240527 2.06820899 0.31747951 2.35768868 3.71513905], Cost: 0.0044
Current parameters: [3.9453356  2.07409024 2.06172089 0.3348979  2.34763406 3.70744741], Cost: 0.0041
Current parameters: [3.94468786 2.06901815 2.06514606 0.3251605  2.35867387 3.69962665], Cost: 0.0037
Current parameters: [3.94496343 2.06746888 2.06895569 0.32954392 2.35912947 3.70594191], Cost: 0.0030
Current parameters: [3.94392146 2.06138955 2.067665   0.33280992 2.36206851 3.70736342], Cost: 0.0035
Current parameters: [3.94540962 2.06963991 2.06757234 0.33058375 2.36180605 3.7063747 ], Cost: 0.0026
Current parameters: [3.94355547 2.07376025 2.07227612 0.3334929  2.364721   3.70513594], Cost: 0.0039
Current parameters: [3.94227357 2.07044341 2.06615587 0.33133859 2.36052943 3.7071313 ], Cost: 0.0024
Current parameters: [3.94632173 2.07219987 2.06274839 0.33540767 2.3570171  3.70815968], Cost: 0.0020
Current parameters: [3.9475049  2.07388343 2.06014127 0.33199425 2.35753096 3.71433126], Cost: 0.0008
Current parameters: [3.94831397 2.07556494 2.05356547 0.3283526  2.35843718 3.71385267], Cost: 0.0005
Current parameters: [3.94894465 2.08062174 2.05292928 0.32833614 2.35956691 3.71963064], Cost: 0.0010
Current parameters: [3.9480681  2.07392475 2.05233955 0.33028368 2.3610034  3.71468375], Cost: 0.0003
Current parameters: [3.94845102 2.06828407 2.05023616 0.32883111 2.35796069 3.71832777], Cost: 0.0003
Current parameters: [3.95190484 2.07381404 2.0524376  0.33093499 2.36095398 3.71438416], Cost: 0.0001
Current parameters: [3.95300033 2.07181393 2.05510266 0.32582257 2.36570548 3.7145202 ], Cost: 0.0010
Current parameters: [3.95229283 2.07289866 2.04764094 0.33563127 2.35999722 3.71063339], Cost: 0.0004
Current parameters: [3.95165901 2.07289829 2.05518056 0.33341186 2.36032452 3.71493629], Cost: 0.0003
Current parameters: [3.9518291  2.07531352 2.05282906 0.33140763 2.36190668 3.71385854], Cost: 0.0002
```



```
Current parameters: [3.95246433 2.07518368 2.05108937 0.3316308  2.36071383 3.71765682], Cost: 0.0002
Current parameters: [3.95203584 2.07363134 2.05104807 0.33198065 2.36070822 3.7135598 ], Cost: 0.0001
Current parameters: [3.95274856 2.07281389 2.05010388 0.33201229 2.36201181 3.71334383], Cost: 0.0001
Current parameters: [3.95287584 2.07319215 2.0496718  0.33128081 2.36184868 3.71312931], Cost: 0.0001
Current parameters: [3.95404848 2.07311406 2.04901173 0.33192762 2.36096927 3.71223148], Cost: 0.0001
Current parameters: [3.95258595 2.07285039 2.04999239 0.33108122 2.36190079 3.71234989], Cost: 0.0001
Current parameters: [3.95192392 2.07345154 2.0482176  0.33166346 2.36186503 3.71389081], Cost: 0.0000
Current parameters: [3.9523614  2.07255405 2.04797288 0.3309166  2.36138327 3.71529369], Cost: 0.0001
Current parameters: [3.95235101 2.07411665 2.04836656 0.33129908 2.36220529 3.71413198], Cost: 0.0000
Current parameters: [3.9514037  2.0737618  2.0475254  0.33322582 2.36246953 3.71429362], Cost: 0.0001
Current parameters: [3.95135542 2.07333465 2.04807563 0.3311068  2.36216372 3.71344631], Cost: 0.0001
Current parameters: [3.9516151  2.07360329 2.04846959 0.33165576 2.36170989 3.71407079], Cost: 0.0000
Current parameters: [3.95220296 2.0737898  2.0480145  0.33180915 2.36107275 3.71362396], Cost: 0.0000
Current parameters: [3.95266633 2.07360368 2.04867981 0.33220802 2.36086091 3.71338377], Cost: 0.0000
Current parameters: [3.9520804  2.0740227  2.04796894 0.33213182 2.3612573  3.71345394], Cost: 0.0000
Current parameters: [3.95220137 2.0740916  2.04746865 0.33269701 2.36102767 3.71401235], Cost: 0.0000
Current parameters: [3.95205614 2.07436355 2.04674104 0.3330389  2.36057877 3.71390835], Cost: 0.0000
Current parameters: [3.95245236 2.07446095 2.04747755 0.33260851 2.36111798 3.71416381], Cost: 0.0000
Current parameters: [3.95216796 2.07464316 2.04770824 0.33262796 2.36094026 3.7143617 ], Cost: 0.0000
Current parameters: [3.95204438 2.07464342 2.047597   0.33253194 2.36106964 3.71443915], Cost: 0.0000
Current parameters: [3.9523172  2.07470213 2.04781242 0.33289965 2.36113905 3.71465917], Cost: 0.0000
Current parameters: [3.95251705 2.07458358 2.04779216 0.33269434 2.36093836 3.71497789], Cost: 0.0000
Current parameters: [3.95253425 2.07452658 2.04797115 0.33258253 2.36104216 3.71495063], Cost: 0.0000
Current parameters: [3.95267054 2.07499004 2.0476514  0.33277141 2.36084017 3.71509728], Cost: 0.0000
Current parameters: [3.95250382 2.07504103 2.04776426 0.33279032 2.360743   3.71517895], Cost: 0.0000
Current parameters: [3.95276794 2.07480349 2.04739515 0.33310005 2.36075609 3.71521302], Cost: 0.0000
Current parameters: [3.95313602 2.07470092 2.04734975 0.33332874 2.36086495 3.7153749 ], Cost: 0.0000
Current parameters: [3.95287228 2.07476383 2.04745435 0.33306692 2.36056138 3.71514498], Cost: 0.0000
Current parameters: [3.9526949  2.07472624 2.04704636 0.33286235 2.36076597 3.71543407], Cost: 0.0000
Current parameters: [3.95262929 2.07490624 2.04722126 0.3333688  2.36088843 3.71491526], Cost: 0.0000
Current parameters: [3.95270437 2.07459041 2.04745848 0.33309851 2.36082526 3.71517613], Cost: 0.0000
Current parameters: [3.95266339 2.07461265 2.04754074 0.33316947 2.36080421 3.71520676], Cost: 0.0000
Current parameters: [3.95257974 2.07454766 2.04733702 0.33306716 2.36082361 3.71533894], Cost: 0.0000
Current parameters: [3.9527319  2.07460029 2.04752166 0.33301934 2.36085117 3.71523195], Cost: 0.0000
Current parameters: [3.95263799 2.07465932 2.0474255  0.33310923 2.36101644 3.71506347], Cost: 0.0000
Current parameters: [3.95286517 2.07453647 2.04741338 0.33321695 2.36091316 3.7152601 ], Cost: 0.0000
Current parameters: [3.95272161 2.07455203 2.04744727 0.33307861 2.3607535  3.71512554], Cost: 0.0000
Current parameters: [3.95274691 2.07458559 2.04746743 0.33311782 2.36083725 3.7151677 ], Cost: 0.0000
Current parameters: [3.95270938 2.0745477  2.04739603 0.33312101 2.36082278 3.71523728], Cost: 0.0000
Current parameters: [3.95268875 2.07455216 2.04747186 0.33309859 2.36084752 3.71516524], Cost: 0.0000
Current parameters: [3.95269752 2.07464801 2.04738981 0.33310157 2.36085292 3.7151423 ], Cost: 0.0000
```



```
Current parameters: [3.95269752 2.07464801 2.04738981 0.33310157 2.36085292 3.7151423 ], Cost: 0.0000

   Normal return from subroutine COBYLA

   NFVALS =   94   F = 3.178358E-08    MAXCV = 0.000000E+00
   X = 3.952698E+00   2.074648E+00   2.047390E+00   3.331016E-01   2.360853E+00
       3.715142E+00
Simulating for QAOA depth p = 4
Current parameters: [3.87204451 1.11116365 0.47850422 2.10256461 2.57815615 1.43619981
 0.16402959 2.82476367], Cost: 1.2157
Current parameters: [2.87204451 2.11116365 0.47850422 2.10256461 2.57815615 1.43619981
 0.16402959 2.82476367], Cost: 2.4594
Current parameters: [2.87204451 1.11116365 1.47850422 2.10256461 2.57815615 1.43619981
 0.16402959 2.82476367], Cost: 1.9767
Current parameters: [2.87204451 1.11116365 0.47850422 3.10256461 2.57815615 1.43619981
 0.16402959 2.82476367], Cost: 2.1746
Current parameters: [2.87204451 1.11116365 0.47850422 2.10256461 3.57815615 1.43619981
 0.16402959 2.82476367], Cost: 0.8713
Current parameters: [2.87204451 1.11116365 0.47850422 2.10256461 2.57815615 2.43619981
 0.16402959 2.82476367], Cost: 3.1386
Current parameters: [2.87204451 1.11116365 0.47850422 2.10256461 2.57815615 1.43619981
 1.16402959 2.82476367], Cost: 3.1706
Current parameters: [2.87204451 1.11116365 0.47850422 2.10256461 2.57815615 1.43619981
 0.16402959 3.82476367], Cost: 1.9233
Current parameters: [ 2.7678386   0.72540264  0.20201801  1.78127006  2.55192661  0.89666669
 -0.38274686  2.56036569], Cost: 2.0031
Current parameters: [ 2.81994155  0.91828314  0.34026111  1.94191733  2.56504138  1.16643325
 -0.10935864  2.69256468], Cost: 2.6872
Current parameters: [2.86634158 1.35601178 0.46337282 2.08498096 2.57672067 1.40667252
 0.13410588 2.81029383], Cost: 0.9731
Current parameters: [2.98901267 1.11116365 0.43441965 2.10256461 2.57815615 1.43619981
 0.16402959 2.82476367], Cost: 0.8777
Current parameters: [2.94986631 1.1109274  0.71288629 2.08828557 2.57699045 1.41222177
 0.13972963 2.81301324], Cost: 0.7579
Current parameters: [2.87204451 1.11116365 0.47850422 2.1127354  2.45357062 1.43619981
 0.16402959 2.82476367], Cost: 0.6123
Current parameters: [2.86368206 1.11088234 0.48953783 2.35854087 2.45987558 1.40764837
 0.13509484 2.81077205], Cost: 0.8184
Current parameters: [2.87204451 1.11116365 0.47850422 2.1127354  2.45357062 1.52517554
 0.07623255 2.82476367], Cost: 0.7931
Current parameters: [2.84209578 1.11015619 0.51801927 2.10980525 2.40404554 1.55098832
```



```
 0.36870498 2.77465498], Cost: 0.6186
Current parameters: [2.85554476 1.11060861 0.50027437 2.11112108 2.42628561 1.43412694
 0.21060153 3.06731921], Cost: 1.1233
Current parameters: [2.83826256 1.06725899 0.47675944 2.07462321 2.41405201 1.3730554
 0.16529809 2.75026513], Cost: 0.7660
Current parameters: [2.85026708 1.11461676 0.42072286 2.11605174 2.45384135 1.44176877
 0.1696733  2.82749273], Cost: 0.6192
Current parameters: [2.8652576  1.23140878 0.48585029 2.10156306 2.44215591 1.41768544
 0.16409281 2.80312622], Cost: 0.6379
Current parameters: [2.87043227 1.1115172  0.48694165 2.11705941 2.44865641 1.47221006
 0.20996484 2.80520087], Cost: 0.4499
Current parameters: [2.86030808 1.11317278 0.49986249 2.11364592 2.43189775 1.4888215
 0.25633999 2.83544922], Cost: 0.4561
Current parameters: [2.85045313 1.11149173 0.49518543 2.13921809 2.45046538 1.46962449
 0.20734456 2.80393381], Cost: 0.4269
Current parameters: [2.88391476 1.11253772 0.48750486 2.18353679 2.44391538 1.47141923
 0.2273898  2.78620243], Cost: 0.3962
Current parameters: [2.88889697 1.11901284 0.48776218 2.18959306 2.44440979 1.49490054
 0.21322172 2.79718957], Cost: 0.4436
Current parameters: [2.86471234 1.09884398 0.4955911  2.17957638 2.42733529 1.48464989
 0.24184698 2.7351146 ], Cost: 0.2788
Current parameters: [2.85564096 1.09619935 0.50249369 2.1793604  2.47486493 1.49200714
 0.26870832 2.70801208], Cost: 0.2451
Current parameters: [2.85821055 1.06612262 0.50248134 2.18184116 2.47789682 1.49493855
 0.26660739 2.71409451], Cost: 0.2662
Current parameters: [2.85330216 1.09889129 0.54452146 2.18129105 2.45877344 1.47331745
 0.279123   2.67050519], Cost: 0.2000
Current parameters: [2.82341905 1.10094954 0.51697453 2.18827213 2.44195172 1.46262864
 0.29971297 2.63335311], Cost: 0.1592
Current parameters: [2.85871369 1.10007607 0.49967196 2.15355486 2.44140144 1.46049946
 0.29354852 2.60000238], Cost: 0.2016
Current parameters: [2.83805938 1.10188881 0.51604817 2.19411403 2.43324949 1.46996236
 0.32359892 2.63848189], Cost: 0.1520
Current parameters: [2.80772048 1.10187782 0.53005955 2.15027103 2.42287509 1.48034145
 0.34638447 2.65000314], Cost: 0.1629
Current parameters: [2.83542534 1.10280044 0.51940623 2.19837209 2.42733993 1.4974743
 0.31563604 2.62930865], Cost: 0.1434
Current parameters: [2.81265866 1.12863327 0.53691123 2.2363095  2.42764761 1.49904434
 0.33748924 2.60706458], Cost: 0.1900
Current parameters: [2.83534196 1.10655391 0.51701804 2.20414611 2.37045131 1.48597576
 0.29707143 2.64113811], Cost: 0.1724
```

```
Current parameters: [2.83223385 1.07291916 0.52233458 2.20420299 2.4264536  1.49821849
 0.31940432 2.62538796], Cost: 0.1295
Current parameters: [2.82554472 1.07264841 0.51487752 2.20943753 2.42864628 1.50154717
 0.31957084 2.63540789], Cost: 0.1330
Current parameters: [2.8410517  1.06254998 0.50145111 2.19483    2.42890605 1.5055508
 0.32268248 2.61135971], Cost: 0.1145
Current parameters: [2.86074079 1.04717036 0.50522014 2.18846823 2.4389487  1.51864059
 0.31924175 2.61505504], Cost: 0.1319
Current parameters: [2.84712878 1.06386541 0.49894685 2.20715086 2.43153896 1.50258898
 0.31846495 2.60762977], Cost: 0.1121
Current parameters: [2.83575402 1.06171335 0.49544903 2.21498127 2.43256531 1.5044868
 0.33431799 2.58497979], Cost: 0.0935
Current parameters: [2.83327411 1.05498029 0.48556034 2.21993357 2.40725173 1.50182282
 0.33486194 2.57254608], Cost: 0.0868
Current parameters: [2.81757182 1.04952736 0.48171944 2.21562503 2.41537044 1.49645533
 0.31603774 2.55778545], Cost: 0.0851
Current parameters: [2.81785372 1.0410635  0.48105215 2.21535068 2.41769686 1.48471147
 0.32051242 2.56070957], Cost: 0.0930
Current parameters: [2.81844772 1.07102976 0.46791739 2.2122203  2.41589386 1.49693415
 0.32321359 2.54168087], Cost: 0.0849
Current parameters: [2.81079207 1.07139184 0.45827962 2.21699176 2.41716081 1.4994489
 0.32406997 2.54949653], Cost: 0.0724
Current parameters: [2.80176567 1.06322438 0.44710593 2.22975026 2.4199089  1.52210991
 0.32783933 2.54715842], Cost: 0.0461
Current parameters: [2.78057171 1.06964094 0.45037211 2.2487935  2.41543035 1.53108278
 0.33084801 2.54896716], Cost: 0.0547
Current parameters: [2.80922145 1.06479953 0.44505549 2.2410093  2.42239568 1.51893137
 0.32167125 2.54628195], Cost: 0.0494
Current parameters: [2.80421075 1.05672487 0.42627022 2.22152437 2.41712615 1.53933177
 0.31993758 2.55488983], Cost: 0.0420
Current parameters: [2.80469614 1.06291156 0.43096746 2.21918701 2.42822723 1.54490354
 0.31729719 2.55899499], Cost: 0.0486
Current parameters: [2.80164343 1.0413278  0.41358188 2.22407892 2.4322654  1.53411773
 0.33735002 2.55248043], Cost: 0.0473
Current parameters: [2.79689626 1.05389675 0.4250662  2.21906105 2.42108846 1.5364796
 0.31045866 2.54705157], Cost: 0.0339
Current parameters: [2.8035769  1.05213855 0.42719062 2.21633573 2.41708417 1.55084893
 0.31278575 2.52080329], Cost: 0.0234
Current parameters: [2.80522643 1.02763275 0.44302613 2.2185987  2.41486608 1.55721691
 0.30540605 2.52498562], Cost: 0.0233
Current parameters: [2.81518262 1.02608506 0.44318233 2.20902746 2.41634357 1.55046698
```



```
 0.30365793 2.52455417], Cost: 0.0299
Current parameters: [2.78537489 1.02498155 0.43739622 2.21943638 2.40044713 1.56178469
 0.29701612 2.50936862], Cost: 0.0184
Current parameters: [2.77796277 1.00945021 0.42522394 2.22390473 2.41551314 1.56039431
 0.31113042 2.50019592], Cost: 0.0070
Current parameters: [2.78772348 1.0074804  0.41536712 2.24694814 2.41926869 1.55839098
 0.2982322  2.49214769], Cost: 0.0128
Current parameters: [2.77665128 1.01071937 0.43378048 2.22852577 2.41355031 1.55051852
 0.31568653 2.49528682], Cost: 0.0108
Current parameters: [2.76460174 1.01338007 0.43156993 2.22033401 2.43641698 1.57536054
 0.30939993 2.49209485], Cost: 0.0052
Current parameters: [2.77199147 1.01424204 0.43325731 2.22053607 2.43223681 1.58364895
 0.31606687 2.48465968], Cost: 0.0078
Current parameters: [2.74620484 1.00433171 0.41855866 2.22345137 2.43540332 1.58269674
 0.32188866 2.5049951 ], Cost: 0.0237
Current parameters: [2.7654828  1.00111862 0.43948845 2.22141135 2.4353163  1.57850631
 0.30570025 2.49418355], Cost: 0.0056
Current parameters: [2.7695983  1.00488232 0.42415847 2.19894006 2.4425295  1.56573597
 0.30144719 2.47892243], Cost: 0.0058
Current parameters: [2.7737873  1.01253611 0.4310116  2.21837463 2.44395577 1.57465569
 0.31453918 2.50053055], Cost: 0.0122
Current parameters: [2.75709638 1.0227701  0.4482902  2.22897702 2.44669641 1.56765698
 0.30013752 2.47702878], Cost: 0.0138
Current parameters: [2.76547293 1.01949624 0.43328659 2.21440504 2.42956697 1.57951366
 0.30033123 2.4967276 ], Cost: 0.0055
Current parameters: [2.76597324 1.01291855 0.42773626 2.22344348 2.43915556 1.57866742
 0.30547341 2.49124481], Cost: 0.0057
Current parameters: [2.76974371 1.01154649 0.42814666 2.22636881 2.42737629 1.56658533
 0.3067448  2.49145209], Cost: 0.0041
Current parameters: [2.76415877 1.00940003 0.42460173 2.22599995 2.42393363 1.56701647
 0.30744888 2.49143577], Cost: 0.0031
Current parameters: [2.75942413 1.0108139  0.42695226 2.22807349 2.42327155 1.56447504
 0.30494628 2.48774328], Cost: 0.0049
Current parameters: [2.76353717 1.01047733 0.42544351 2.22862874 2.42342957 1.56799123
 0.30840256 2.4934305 ], Cost: 0.0035
Current parameters: [2.76457779 1.006876   0.42042439 2.22295126 2.4237859  1.56439435
 0.30877347 2.49580524], Cost: 0.0032
Current parameters: [2.76425642 1.0124573  0.42272289 2.22574316 2.42402677 1.56659448
 0.30864419 2.4906046 ], Cost: 0.0031
Current parameters: [2.76672912 1.01033312 0.42174654 2.22449614 2.41976295 1.57000417
 0.31190427 2.48787215], Cost: 0.0024
```



```
Current parameters: [2.76798095 1.00892307 0.41675163 2.22704135 2.42128385 1.57342042
 0.30858304 2.48685325], Cost: 0.0018
Current parameters: [2.76853933 1.01027379 0.41693372 2.22602035 2.41901958 1.57382755
 0.30621627 2.4879269 ], Cost: 0.0021
Current parameters: [2.76539357 1.00916791 0.41680574 2.22301436 2.42410663 1.57881064
 0.30950251 2.48729271], Cost: 0.0022
Current parameters: [2.7662345  1.00556928 0.41359897 2.22849789 2.41964134 1.57239783
 0.30906975 2.48131286], Cost: 0.0011
Current parameters: [2.77206163 1.00330444 0.41235876 2.22929194 2.42146286 1.57224574
 0.31023    2.47742774], Cost: 0.0011
Current parameters: [2.7723624  1.0019296  0.41023753 2.22741388 2.42147528 1.57084113
 0.31070269 2.47917557], Cost: 0.0015
Current parameters: [2.77120234 1.00251684 0.41108598 2.23537887 2.41937155 1.57476261
 0.31340388 2.47790112], Cost: 0.0006
Current parameters: [2.77007082 1.00811088 0.40748298 2.23721542 2.42107315 1.57322822
 0.31453831 2.47554094], Cost: 0.0006
Current parameters: [2.76975608 1.00727769 0.40644279 2.23866336 2.42295351 1.57361859
 0.31187543 2.47625365], Cost: 0.0007
Current parameters: [2.76707535 1.0062709  0.41103085 2.24118692 2.4209363  1.57109958
 0.31623739 2.4719521 ], Cost: 0.0011
Current parameters: [2.76873585 1.00751371 0.40741101 2.23611195 2.42338271 1.57519983
 0.31613042 2.47516536], Cost: 0.0007
Current parameters: [2.7701633  1.00905527 0.40566452 2.23691337 2.41684826 1.57843163
 0.31265981 2.47266296], Cost: 0.0005
Current parameters: [2.77317069 1.00798884 0.40511399 2.23728013 2.41781621 1.57838028
 0.31323742 2.4708282 ], Cost: 0.0003
Current parameters: [2.77747759 1.01143824 0.40744132 2.23957282 2.41808456 1.58186324
 0.3144451  2.47332879], Cost: 0.0013
Current parameters: [2.77296452 1.00459472 0.39909399 2.23647811 2.41542757 1.5769355
 0.31534998 2.47140531], Cost: 0.0002
Current parameters: [2.77315613 1.00461107 0.39872029 2.23360028 2.41554974 1.57574471
 0.31382743 2.46965911], Cost: 0.0005
Current parameters: [2.77264475 1.00596556 0.3982353  2.23698897 2.41585591 1.57652784
 0.31564601 2.47077475], Cost: 0.0002
Current parameters: [2.77124754 1.00456692 0.39901833 2.23916761 2.4151641  1.57599616
 0.3162792  2.46857734], Cost: 0.0001
Current parameters: [2.7715042  1.00507684 0.39965029 2.23852964 2.41433487 1.57580602
 0.31766598 2.46838894], Cost: 0.0002
Current parameters: [2.77264226 1.00475145 0.39780047 2.24219302 2.41513762 1.57737551
 0.31682832 2.46923946], Cost: 0.0001
Current parameters: [2.77008832 1.00574341 0.39731671 2.24254468 2.4132137  1.57885691
```



```
 0.31606352 2.47019524], Cost: 0.0001
Current parameters: [2.77227667 1.00445947 0.39756486 2.24167821 2.4159494  1.5787438
 0.31746831 2.46866116], Cost: 0.0001

   Return from subroutine COBYLA because the MAXFUN limit has been reached.

   NFVALS =  100   F = 8.143434E-05    MAXCV = 0.000000E+00
   X = 2.772642E+00   1.004751E+00   3.978005E-01   2.242193E+00   2.415138E+00
       1.577376E+00   3.168283E-01   2.469239E+00
Current parameters: [2.77264226 1.00475145 0.39780047 2.24219302 2.41513762 1.57737551
 0.31682832 2.46923946], Cost: 0.0001
Simulating for QAOA depth p = 5
Current parameters: [1.82069554 1.18366667 1.94535231 0.84103796 0.18794478 2.9114611
 3.14057377 1.78150306 1.86889299 2.08149914], Cost: 2.2692
Current parameters: [1.82069554 2.18366667 1.94535231 0.84103796 0.18794478 2.9114611
 3.14057377 1.78150306 1.86889299 2.08149914], Cost: 2.9237
Current parameters: [1.82069554 1.18366667 2.94535231 0.84103796 0.18794478 2.9114611
 3.14057377 1.78150306 1.86889299 2.08149914], Cost: 2.9247
Current parameters: [1.82069554 1.18366667 1.94535231 1.84103796 0.18794478 2.9114611
 3.14057377 1.78150306 1.86889299 2.08149914], Cost: 2.3942
Current parameters: [1.82069554 1.18366667 1.94535231 0.84103796 1.18794478 2.9114611
 3.14057377 1.78150306 1.86889299 2.08149914], Cost: 2.3388
Current parameters: [1.82069554 1.18366667 1.94535231 0.84103796 0.18794478 3.9114611
 3.14057377 1.78150306 1.86889299 2.08149914], Cost: 0.6707
Current parameters: [1.82069554 1.18366667 1.94535231 0.84103796 0.18794478 3.9114611
 4.14057377 1.78150306 1.86889299 2.08149914], Cost: 1.0715
Current parameters: [1.82069554 1.18366667 1.94535231 0.84103796 0.18794478 3.9114611
 3.14057377 2.78150306 1.86889299 2.08149914], Cost: 3.5253
Current parameters: [1.82069554 1.18366667 1.94535231 0.84103796 0.18794478 3.9114611
 3.14057377 1.78150306 2.86889299 2.08149914], Cost: 2.3849
Current parameters: [1.82069554 1.18366667 1.94535231 0.84103796 0.18794478 3.9114611
 3.14057377 1.78150306 1.86889299 3.08149914], Cost: 2.5640
Current parameters: [1.82200597 1.03052977 1.79198175 0.81179523 0.17167175 4.28548578
 3.04679126 1.11359728 1.46781205 1.63849663], Cost: 2.6454
Current parameters: [1.82135076 1.10709822 1.86866703 0.82641659 0.17980826 4.09847344
 3.09368252 1.44755017 1.66835252 1.85999789], Cost: 2.9685
Current parameters: [2.07069506 1.18366667 1.94535231 0.84103796 0.18794478 3.9114611
 3.14057377 1.78199356 1.86889299 2.08149914], Cost: 1.0769
Current parameters: [1.74603693 1.1539709  1.91561122 0.83536731 0.18478917 3.98399067
 3.12238779 2.25436397 1.79111677 1.99559363], Cost: 1.5116
Current parameters: [1.82069554 1.36057808 1.76871043 0.84103796 0.18794478 3.9114611
```

```
 3.14057377 1.78150306 1.86889299 2.08149914], Cost: 0.6702
Current parameters: [1.79510383 1.71178632 2.12041647 0.83906941 0.18684932 3.93663945
 3.13426058 1.77963446 1.84189328 2.05167738], Cost: 1.5470
Current parameters: [1.82069554 1.36057808 1.76871043 0.59269177 0.16764489 3.89116121
 3.14057377 1.78150306 1.86889299 2.08149914], Cost: 0.5202
Current parameters: [ 1.79061325  1.34515757  1.75322163  0.63857998 -0.19689509  3.55750531
  3.1331528   1.77930657  1.83715564  2.04644455], Cost: 3.3868
Current parameters: [1.82069554 1.36057808 1.76871043 0.59269177 0.22452183 3.83428426
 2.90386715 1.78150306 1.86889299 2.08149914], Cost: 0.6341
Current parameters: [1.7907664  1.34523608 1.75330049 0.57122311 0.55553951 3.62980236
 3.30544239 1.77931775 1.83731721 2.04662302], Cost: 2.4455
Current parameters: [1.82069554 1.36057808 1.76871043 0.59269177 0.16764489 3.89116121
 3.14057377 1.78150306 2.05422091 1.91370891], Cost: 0.4671
Current parameters: [1.76240346 1.33069693 1.73869695 0.55087791 0.19421921 4.11103679
 3.11138873 1.7772468  2.35478425 2.23433372], Cost: 2.3877
Current parameters: [1.80471296 1.33997634 1.74805409 0.57911508 0.17340031 3.98070727
 3.12505008 1.99220771 1.99379357 1.84393356], Cost: 0.2819
Current parameters: [1.8047702  1.25537403 1.66332269 0.57825644 0.17292249 3.99168957
 3.12229639 1.96303603 1.98201683 1.8309259 ], Cost: 0.3447
Current parameters: [1.77813087 1.39020127 1.79831579 0.55627517 0.18359872 4.12808849
 3.1000211  1.88341514 1.89701462 1.73182077], Cost: 0.7317
Current parameters: [1.80471271 1.34089881 1.74897797 0.58840972 0.05280515 3.98759308
 3.09441837 1.99233539 1.99568939 1.84602753], Cost: 0.2132
Current parameters: [1.73223273e+00 1.34258197e+00 1.75055609e+00 5.81199668e-01
 1.24800781e-04 3.79956541e+00 3.14835825e+00 2.06584066e+00
 1.93340997e+00 1.76315139e+00], Cost: 0.6203
Current parameters: [1.68847316 1.33812428 1.74619921 0.58651544 0.05577588 4.00779671
 3.09027757 1.9917233  2.02303313 1.87622929], Cost: 0.2830
Current parameters: [1.91670178 1.33394434 1.74184846 0.5447016  0.01665007 4.05589724
 3.10204439 2.10376732 2.12126788 1.96320695], Cost: 1.6838
Current parameters: [1.79100804 1.36725523 1.77534524 0.57651459 0.0490371  4.04713451
 3.08505035 1.94198054 1.93864512 1.77917698], Cost: 0.4332
Current parameters: [1.80471271 1.38512635 1.70481781 0.58840972 0.05285846 3.98753977
 3.09419649 1.99233539 1.99568939 1.84602753], Cost: 0.2202
Current parameters: [1.80441795 1.34093463 1.75012945 0.58705824 0.07921557 3.95671777
 2.97637714 1.99668077 1.99329046 1.84247851], Cost: 0.1963
Current parameters: [1.80441795 1.34093463 1.75012945 0.60777744 0.08090917 3.95841136
 2.97637714 1.99668077 2.03696642 1.80293563], Cost: 0.1842
Current parameters: [1.80399395 1.34145767 1.75131508 0.72520957 0.08822906 3.96418442
 2.974831   2.00293162 2.0026027  1.8258179 ], Cost: 0.2248
Current parameters: [1.80450664 1.34674738 1.75596048 0.61250999 0.07902085 3.93117516
```



```
 2.98059423 1.94338127 2.0461111  1.81426846], Cost: 0.1608
Current parameters: [1.86745611 1.34814662 1.7639732  0.60748973 0.06232939 3.94173991
 2.9647058  1.9481454  2.12356915 1.88431054], Cost: 0.1228
Current parameters: [1.87839796 1.34532726 1.7682653  0.60344665 0.02689533 3.83299322
 2.98006368 1.98993272 2.14354627 1.88442489], Cost: 0.2052
Current parameters: [1.87011372 1.30442565 1.72018938 0.6087229  0.06172902 3.93904857
 2.96444019 1.94087804 2.12527648 1.88670149], Cost: 0.0774
Current parameters: [1.92184187 1.26354578 1.69170498 0.59255447 0.02620122 3.98398297
 2.93144709 1.86912729 2.13097869 1.86111439], Cost: 0.1978
Current parameters: [1.82934416 1.30378124 1.71954399 0.61137063 0.01979713 3.94858846
 2.95207232 1.94070885 2.13554251 1.89804053], Cost: 0.0832
Current parameters: [1.81203902 1.28155798 1.70347584 0.59732217 0.1448935  3.94277188
 2.9741003  1.93185728 2.17538339 1.92798696], Cost: 0.2019
Current parameters: [1.8766352  1.30115439 1.71806553 0.66751368 0.06237579 3.94080815
 2.96223813 1.94244523 2.11311552 1.90198036], Cost: 0.0515
Current parameters: [1.87658783 1.31813523 1.70130137 0.66729648 0.06664086 3.93582549
 2.94318169 1.94314361 2.11272997 1.90140997], Cost: 0.0523
Current parameters: [1.88063366 1.32747909 1.68774734 0.67045121 0.05065768 3.95211834
 3.00664672 1.93999529 2.11641677 1.90587824], Cost: 0.0781
Current parameters: [1.87665562 1.30249304 1.71940838 0.65940825 0.06118929 3.93378418
 2.96320931 1.93017064 2.09583781 1.92213965], Cost: 0.0458
Current parameters: [1.88601426 1.29083643 1.71184911 0.65057894 0.05860768 3.95576641
 2.95537802 1.97655624 2.07137842 1.93784292], Cost: 0.1116
Current parameters: [1.89149206 1.30251451 1.72158876 0.65790586 0.05398021 3.92514168
 2.96128871 1.93541689 2.11474946 1.93743361], Cost: 0.0751
Current parameters: [1.864469   1.29178683 1.71430766 0.67093979 0.0517007  3.88483068
 2.97053323 1.93092315 2.07657526 1.89821499], Cost: 0.0647
Current parameters: [1.85922046 1.3021488  1.71823907 0.66002342 0.07969878 3.92767717
 2.96954767 1.93886442 2.10343872 1.93296952], Cost: 0.0530
Current parameters: [1.86858147 1.2848333  1.71397209 0.67335691 0.0798358  3.95248415
 2.95106354 1.88118513 2.09454803 1.91446316], Cost: 0.0507
Current parameters: [1.87316168 1.32222678 1.74107463 0.66511112 0.06379463 3.93597328
 2.96213104 1.92653776 2.09037046 1.91799811], Cost: 0.0619
Current parameters: [1.88680661 1.30397254 1.71806794 0.65867228 0.0708692  3.93200245
 2.96844486 1.93008441 2.09353717 1.91949691], Cost: 0.0499
Current parameters: [1.87177615 1.31400955 1.7040242  0.66297293 0.05150727 3.94105566
 2.98287336 1.92412947 2.09628081 1.92301157], Cost: 0.0475
Current parameters: [1.87054466 1.30329557 1.71949358 0.63129928 0.06081362 3.93389957
 2.96429843 1.92447052 2.09991754 1.91224129], Cost: 0.0533
Current parameters: [1.87509188 1.29788935 1.71569505 0.65865471 0.06038794 3.94176579
 2.96085951 1.9365219  2.08607475 1.92292648], Cost: 0.0454
```



```
Current parameters: [1.87459122 1.29851325 1.7157111  0.65840099 0.05791528 3.93649349
 2.96247526 1.94035599 2.0837201  1.92103854], Cost: 0.0423
Current parameters: [1.87040799 1.29138082 1.71190824 0.66359066 0.05709226 3.93040565
 2.9611506  1.9336062  2.07907036 1.91577531], Cost: 0.0307
Current parameters: [1.87036871 1.28304138 1.70710733 0.66766013 0.05021092 3.92194309
 2.95921873 1.93128629 2.07644454 1.91585804], Cost: 0.0269
Current parameters: [1.86456788 1.28579956 1.71260088 0.67368305 0.04876404 3.91898479
 2.95792678 1.92715535 2.06788252 1.91006652], Cost: 0.0351
Current parameters: [1.87023863 1.27883145 1.71128509 0.66702431 0.04918615 3.92317537
 2.96398583 1.93099347 2.07665524 1.91574547], Cost: 0.0261
Current parameters: [1.86658795 1.27601895 1.70764222 0.65429997 0.0486609  3.92134793
 2.96356601 1.92791258 2.07693956 1.9098838 ], Cost: 0.0317
Current parameters: [1.86755151 1.27904157 1.7113687  0.66552523 0.05180977 3.92085141
 2.96515516 1.92992142 2.07443058 1.92138163], Cost: 0.0252
Current parameters: [1.86012009 1.27446779 1.70762242 0.67074022 0.0579543  3.91909403
 2.96741746 1.93650302 2.08028397 1.91958635], Cost: 0.0161
Current parameters: [1.86856829 1.27181075 1.70331382 0.67286577 0.06696822 3.91579037
 2.97291298 1.93676579 2.07668183 1.91690001], Cost: 0.0166
Current parameters: [1.86136184 1.27143641 1.71133989 0.66987912 0.06067549 3.91821017
 2.96228307 1.937594   2.08029447 1.91965295], Cost: 0.0157
Current parameters: [1.85908869 1.26424295 1.70599282 0.67392897 0.06318677 3.92791442
 2.95974739 1.9328397  2.08022215 1.92321915], Cost: 0.0128
Current parameters: [1.85944524 1.26505451 1.70712298 0.67506644 0.0645266  3.92590372
 2.95989586 1.92743094 2.08496744 1.92300531], Cost: 0.0132
Current parameters: [1.85837743 1.25816696 1.70318354 0.67855736 0.0625384  3.92615394
 2.960795   1.94045541 2.08778403 1.93080894], Cost: 0.0132
Current parameters: [1.85792017 1.26125337 1.70504731 0.67685103 0.05998067 3.92373752
 2.95867143 1.93103542 2.07740825 1.92210423], Cost: 0.0113
Current parameters: [1.84819906 1.26096833 1.70883271 0.68580743 0.06538675 3.92471892
 2.95954084 1.9323914  2.07403628 1.91880887], Cost: 0.0083
Current parameters: [1.83921698 1.25403791 1.70385191 0.6790467  0.06818067 3.92257311
 2.95970694 1.93196629 2.07508488 1.91322337], Cost: 0.0070
Current parameters: [1.83754682 1.25734634 1.70076098 0.67853909 0.07067866 3.92011601
 2.95688663 1.93148138 2.0734624  1.91701671], Cost: 0.0064
Current parameters: [1.83876851 1.26046073 1.6940262  0.68374022 0.06980017 3.92161568
 2.94893884 1.93459984 2.07690064 1.90848411], Cost: 0.0063
Current parameters: [1.83477387 1.26189566 1.69625821 0.68255211 0.06517438 3.92329828
 2.94615697 1.93429955 2.07853449 1.90986177], Cost: 0.0055
Current parameters: [1.82598673 1.25652294 1.69530075 0.68801571 0.06393605 3.92478786
 2.94028592 1.93028616 2.0713741  1.91116287], Cost: 0.0058
Current parameters: [1.83541091 1.26050734 1.70056938 0.6814257  0.06939223 3.92397404
```



```
 2.94201609 1.93604234 2.07917569 1.90979373], Cost: 0.0050
Current parameters: [1.82745472 1.26659054 1.70126741 0.68321167 0.07608669 3.93180594
 2.946662   1.93885804 2.08123366 1.90928864], Cost: 0.0072
Current parameters: [1.83588275 1.26386967 1.70221248 0.67885732 0.069466   3.92430889
 2.94166575 1.93297526 2.07525019 1.9059009 ], Cost: 0.0089
Current parameters: [1.83146744 1.25781797 1.70239679 0.68474415 0.06733184 3.91385059
 2.94141895 1.94477623 2.08336098 1.91226802], Cost: 0.0025
Current parameters: [1.83309647 1.25512323 1.69846763 0.67992358 0.06416264 3.9186148
 2.94016077 1.95560136 2.07756341 1.91549749], Cost: 0.0045
Current parameters: [1.83385383 1.25430723 1.70180463 0.68378711 0.06908053 3.91630844
 2.93827448 1.94293858 2.08674464 1.91508505], Cost: 0.0028
Current parameters: [1.82553282 1.25759431 1.69835252 0.6878695  0.06329087 3.90581614
 2.94421972 1.94508664 2.09241003 1.91573776], Cost: 0.0060
Current parameters: [1.83224573 1.25853584 1.70582747 0.68128477 0.06782404 3.9106958
 2.93735712 1.94357663 2.08201543 1.90977116], Cost: 0.0036
Current parameters: [1.83222136 1.2601925  1.70197062 0.68560392 0.06786892 3.91322807
 2.94029307 1.94465363 2.08256417 1.91462125], Cost: 0.0030
Current parameters: [1.83428611 1.25678062 1.70697171 0.68929323 0.06473446 3.91538967
 2.94197203 1.94556041 2.08355267 1.91170454], Cost: 0.0027
Current parameters: [1.83132791 1.25815354 1.70063767 0.68639547 0.06721341 3.91424494
 2.93923247 1.94526802 2.08364816 1.91026033], Cost: 0.0022
Current parameters: [1.82756885 1.25434012 1.70082822 0.68894895 0.06799894 3.91392646
 2.93800112 1.94324639 2.07942778 1.9115536 ], Cost: 0.0013
Current parameters: [1.82518557 1.2556078  1.702138   0.68848357 0.06738919 3.91591879
 2.93709142 1.94335101 2.08025971 1.91196335], Cost: 0.0012
Current parameters: [1.82380311 1.25542796 1.70317274 0.6903601  0.07395511 3.9174709
 2.93848571 1.94523778 2.08045858 1.91010979], Cost: 0.0010
Current parameters: [1.82493326 1.25622352 1.70279803 0.68978627 0.07413893 3.91972899
 2.93898605 1.94333369 2.07871799 1.90919297], Cost: 0.0013
Current parameters: [1.81907793 1.25340221 1.7006778  0.6936406  0.07306819 3.91680904
 2.94029118 1.94350097 2.08321872 1.90852914], Cost: 0.0003
Current parameters: [1.81638506 1.24995636 1.69805925 0.6938253  0.07166323 3.91880585
 2.94024057 1.94864346 2.08161213 1.90835937], Cost: 0.0008
Current parameters: [1.81801487 1.25517486 1.70134763 0.69371429 0.07228044 3.91527084
 2.94135174 1.94425233 2.08143699 1.90689153], Cost: 0.0004
Current parameters: [1.81675649 1.25066077 1.7029635  0.69303421 0.07433202 3.91425496
 2.93617973 1.94136279 2.08458098 1.90519952], Cost: 0.0005
Current parameters: [1.82027044 1.25240868 1.70295838 0.69563986 0.07171168 3.91767648
 2.94079158 1.94390224 2.08347719 1.90776706], Cost: 0.0004
Current parameters: [1.81741657 1.25590127 1.69983771 0.69924419 0.07426279 3.91528427
 2.93812864 1.94269389 2.08329507 1.91182125], Cost: 0.0007
```

```
Current parameters: [1.81794517 1.25188621 1.70209326 0.69321323 0.07341838 3.91640076
 2.94156617 1.94245593 2.08160567 1.91049949], Cost: 0.0003
Current parameters: [1.81641066 1.25239525 1.70308105 0.69140432 0.0741061  3.91502536
 2.94622784 1.94242718 2.08704559 1.91119516], Cost: 0.0008
Current parameters: [1.82006001 1.25070142 1.69994806 0.69356921 0.07424481 3.91472005
 2.94242049 1.94207626 2.08162413 1.9099782 ], Cost: 0.0003
Current parameters: [1.81930881 1.24821586 1.70397385 0.6961773  0.07391089 3.91114819
 2.94371386 1.94311889 2.07766985 1.9098447 ], Cost: 0.0008
Current parameters: [1.82006001 1.25070142 1.69994806 0.69356921 0.07424481 3.91472005
 2.94242049 1.94207626 2.08162413 1.9099782 ], Cost: 0.0003

   Return from subroutine COBYLA because the MAXFUN limit has been reached.

   NFVALS =  100   F = 2.667130E-04    MAXCV = 0.000000E+00
   X = 1.820060E+00   1.250701E+00   1.699948E+00   6.935692E-01   7.424481E-02
       3.914720E+00   2.942420E+00   1.942076E+00   2.081624E+00   1.909978E+00
Simulating for QAOA depth p = 6
Current parameters: [1.65084321 0.16042886 1.36377595 1.15314184 1.64964703 1.18627959
 2.08318973 2.02909384 0.91380265 1.8700372  1.62101843 1.74850249], Cost: 0.3698
Current parameters: [1.65084321 1.16042886 1.36377595 1.15314184 1.64964703 1.18627959
 2.08318973 2.02909384 0.91380265 1.8700372  1.62101843 1.74850249], Cost: 2.1045
Current parameters: [1.65084321 0.16042886 2.36377595 1.15314184 1.64964703 1.18627959
 2.08318973 2.02909384 0.91380265 1.8700372  1.62101843 1.74850249], Cost: 2.3674
Current parameters: [1.65084321 0.16042886 1.36377595 2.15314184 1.64964703 1.18627959
 2.08318973 2.02909384 0.91380265 1.8700372  1.62101843 1.74850249], Cost: 2.6674
Current parameters: [1.65084321 0.16042886 1.36377595 1.15314184 2.64964703 1.18627959
 2.08318973 2.02909384 0.91380265 1.8700372  1.62101843 1.74850249], Cost: 0.5153
Current parameters: [1.65084321 0.16042886 1.36377595 1.15314184 1.64964703 2.18627959
 2.08318973 2.02909384 0.91380265 1.8700372  1.62101843 1.74850249], Cost: 0.7895
Current parameters: [1.65084321 0.16042886 1.36377595 1.15314184 1.64964703 1.18627959
 3.08318973 2.02909384 0.91380265 1.8700372  1.62101843 1.74850249], Cost: 3.7112
Current parameters: [1.65084321 0.16042886 1.36377595 1.15314184 1.64964703 1.18627959
 2.08318973 3.02909384 0.91380265 1.8700372  1.62101843 1.74850249], Cost: 3.4799
Current parameters: [1.65084321 0.16042886 1.36377595 1.15314184 1.64964703 1.18627959
 2.08318973 2.02909384 1.91380265 1.8700372  1.62101843 1.74850249], Cost: 2.6099
Current parameters: [1.65084321 0.16042886 1.36377595 1.15314184 1.64964703 1.18627959
 2.08318973 2.02909384 0.91380265 2.8700372  1.62101843 1.74850249], Cost: 1.8290
Current parameters: [1.65084321 0.16042886 1.36377595 1.15314184 1.64964703 1.18627959
 2.08318973 2.02909384 0.91380265 1.8700372  2.62101843 1.74850249], Cost: 1.4519
Current parameters: [1.65084321 0.16042886 1.36377595 1.15314184 1.64964703 1.18627959
 2.08318973 2.02909384 0.91380265 1.8700372  1.62101843 2.74850249], Cost: 2.3749
```



```
Current parameters: [ 1.80732593 -0.09314952  1.07177354  0.81728233  1.62838415  1.12493198
  1.59474477  1.57445922  0.58634254  1.65673814  1.46283807  1.45539884], Cost: 1.8539
Current parameters: [1.72908457 0.03363967 1.21777475 0.98521209 1.63901559 1.15560578
 1.83896725 1.80177653 0.7500726  1.76338767 1.54192825 1.60195067], Cost: 2.6355
Current parameters: [1.65697879 0.40753508 1.35232673 1.13997302 1.64881333 1.18387419
 2.06403812 2.01126792 0.90096316 1.86167388 1.61481628 1.73701009], Cost: 0.6098
Current parameters: [1.54066651 0.16042886 1.30473278 1.15314184 1.64964703 1.18627959
 2.08318973 2.02909384 0.91380265 1.8700372  1.62101843 1.74850249], Cost: 0.3102
Current parameters: [1.4215828  0.15850572 1.51894602 1.13493178 1.64849417 1.18295337
 2.05670659 2.00444387 0.896048   1.85847227 1.612442   1.73261062], Cost: 0.5625
Current parameters: [1.54066651 0.16042886 1.30473278 1.16103964 1.52489678 1.18627959
 2.08318973 2.02909384 0.91380265 1.8700372  1.62101843 1.74850249], Cost: 0.3614
Current parameters: [1.53709869 0.15831893 1.30261207 1.39726156 1.66867378 1.1826303
 2.0541344  2.00204973 0.89432357 1.85734902 1.61160901 1.73106711], Cost: 0.8521
Current parameters: [1.54066651 0.16042886 1.30473278 1.15314184 1.64964703 1.062254
 2.09876707 2.02909384 0.91380265 1.8700372  1.62101843 1.74850249], Cost: 0.3008
Current parameters: [1.53472971 0.15691797 1.30120396 1.1379227  1.65462695 1.09107406
 2.33697844 1.98409283 0.88138974 1.84892426 1.60536129 1.71949027], Cost: 0.2994
Current parameters: [1.53472971 0.15691797 1.30120396 1.1379227  1.65462695 1.09107406
 2.33697844 2.05714902 0.77996098 1.84892426 1.60536129 1.71949027], Cost: 0.8427
Current parameters: [1.52524845 0.15131098 1.29556831 1.11361727 1.66258004 1.09402537
 2.37444791 2.09995136 1.08862896 1.81520625 1.58035637 1.67315686], Cost: 1.2952
Current parameters: [1.53472971 0.15691797 1.30120396 1.1379227  1.65462695 1.09107406
 2.33697844 1.98409283 0.88138974 1.74785403 1.60536129 1.79304167], Cost: 0.3128
Current parameters: [1.52880676 0.15341528 1.29768336 1.12273908 1.65959525 1.09291775
 2.36038565 1.91374474 0.90805157 1.98908785 1.58974069 1.90944866], Cost: 0.2602
Current parameters: [1.52441772 0.16052766 1.30587346 1.13215928 1.66019164 1.09463843
 2.37408558 1.92649635 0.91723619 1.99507047 1.71163405 1.91766964], Cost: 0.2211
Current parameters: [1.54300939 0.10854334 1.24686773 1.0457153  1.66461158 1.08655168
 2.32426075 1.72640631 0.90213369 1.96646246 1.76581634 1.87394266], Cost: 1.4311
Current parameters: [1.52294233 0.16291852 1.30862661 1.23951495 1.6669882  1.0961537
 2.38615007 1.88137385 0.92510837 2.00593478 1.70789117 1.8769242 ], Cost: 0.3861
Current parameters: [1.51705135 0.15049679 1.29518263 1.21970301 1.67567689 1.08676979
 2.32607146 2.0145037  0.90454169 1.93869928 1.70000008 2.11951203], Cost: 0.5746
Current parameters: [1.52514348 0.28438371 1.30155592 1.12388929 1.66087378 1.0938722
 2.36975965 1.91625445 0.91661451 1.99110332 1.7076797  1.92404881], Cost: 0.3200
Current parameters: [1.46225308 0.16032347 1.30207963 1.13022582 1.66006923 1.09428526
 2.37127372 1.92387913 0.91535108 1.99384256 1.71072345 1.91598232], Cost: 0.2205
Current parameters: [1.45581051 0.15937414 1.42535509 1.12020831 1.66082535 1.0933138
 2.36558547 1.91141758 0.91416493 1.98896233 1.70593696 1.92291813], Cost: 0.3754
Current parameters: [1.46246866 0.16045096 1.30220777 1.13468498 1.59818284 1.09420022
```



```
 2.37059665 1.92054183 0.90981217 1.99337362 1.711292   1.91966136], Cost: 0.2718
Current parameters: [1.45668509 0.15811371 1.29690626 1.11755048 1.65332875 1.0972104
 2.40041311 1.97331326 1.02278921 2.00245725 1.69928846 1.89846273], Cost: 0.2315
Current parameters: [1.46225961 0.16032733 1.30208351 1.13024257 1.66006375 1.03230168
 2.37914998 1.92460672 0.91440648 1.99426097 1.71074068 1.91517453], Cost: 0.2197
Current parameters: [1.45942246 0.1601593  1.2977989  1.12379913 1.66862611 1.03947332
 2.41570569 2.00624498 0.85425941 2.04200016 1.70778822 1.87611358], Cost: 0.1439
Current parameters: [1.46105517 0.16110728 1.29874984 1.12794722 1.66726214 1.03280673
 2.36023259 2.01969532 0.86042998 2.05880317 1.71209337 1.86058478], Cost: 0.1606
Current parameters: [1.46576975 0.12042841 1.23963728 1.0399142  1.69824547 1.04131883
 2.43017111 1.99197634 0.83794577 2.05722212 1.73669899 1.84513808], Cost: 0.2024
Current parameters: [1.45789428 0.15909676 1.29671389 1.12379015 1.67022731 1.03965897
 2.41954149 1.99290796 0.85982476 2.09375124 1.70375073 1.90743405], Cost: 0.1984
Current parameters: [1.45749915 0.13825296 1.26448548 1.14356864 1.69564536 1.0379711
 2.40714881 2.08128241 0.83618861 1.99201043 1.71122947 1.94219908], Cost: 0.1052
Current parameters: [1.45630533 0.14254387 1.26933434 1.1763075  1.69665152 1.04284399
 2.44233976 2.07551298 0.82864982 1.97658042 1.7083079  1.90768579], Cost: 0.2725
Current parameters: [1.46020124 0.10551483 1.21663871 1.14029847 1.72620561 1.02701612
 2.32845295 2.09849407 0.85250006 2.02860651 1.72853817 1.99509292], Cost: 0.0795
Current parameters: [1.45818562 0.05358813 1.25132533 1.1410853  1.72612314 1.02707818
 2.32875232 2.09945464 0.85245078 2.02896523 1.72900985 1.99428787], Cost: 0.1040
Current parameters: [1.45773801 0.15432979 1.27361782 1.06278693 1.7434993  1.02338822
 2.30303639 2.13377302 0.84413608 2.01598245 1.73365861 2.03411846], Cost: 0.0376
Current parameters: [1.45576369 0.15769876 1.27749064 1.06877511 1.7217652  1.02416205
 2.30919754 2.13854479 0.84645789 2.01861133 1.79082169 2.03926876], Cost: 0.1262
Current parameters: [1.46171256 0.14098013 1.24480896 1.02771246 1.64800566 1.02097736
 2.28432196 2.15173172 0.82607379 2.00843987 1.68070134 2.05375559], Cost: 0.1227
Current parameters: [1.45949504 0.15765689 1.27946766 1.07211643 1.74532807 1.02284673
 2.29616344 2.10714919 0.7920218  2.02305089 1.73621501 2.04956946], Cost: 0.1578
Current parameters: [1.45006376 0.16673384 1.28798952 1.06854771 1.74263887 1.03025548
 2.36165393 2.13903727 0.87454927 2.10107715 1.70592952 2.08632872], Cost: 0.1929
Current parameters: [1.39567898 0.15423119 1.27003296 1.06101036 1.74319249 1.01988786
 2.30062105 2.13010376 0.8428751  2.01420123 1.73269342 2.0328649 ], Cost: 0.0411
Current parameters: [1.46546585 0.15587909 1.27619706 1.0605757  1.74300962 0.90912701
 2.31499007 2.10248631 0.86822221 1.99397926 1.73085984 2.05148534], Cost: 0.1602
Current parameters: [1.45632951 0.16748333 1.29389691 1.09420629 1.73171549 1.03705135
 2.2832366  2.11948493 0.86587896 1.99720761 1.72478237 2.05663685], Cost: 0.1284
Current parameters: [1.46249488 0.13321116 1.24013361 0.99447494 1.76578679 1.06724305
 2.32293168 2.06791833 0.87708493 1.98553697 1.74193851 2.0457333 ], Cost: 0.1023
Current parameters: [1.45937209 0.16446595 1.28901327 1.0513219  1.73025964 1.02074875
 2.27981414 2.10758272 0.85524607 2.04604013 1.73397931 2.00252871], Cost: 0.1372
```



```
Current parameters: [1.45846593 0.14175626 1.23262408 1.0563593  1.80878662 1.02875011
 2.27931672 2.21859118 0.85268302 1.9946725  1.71734464 2.00406286], Cost: 0.0754
Current parameters: [1.45632946 0.16964005 1.29246578 1.0615543  1.73758197 1.03015264
 2.34196579 2.13992941 0.83624028 1.99132748 1.72156867 2.00474929], Cost: 0.0235
Current parameters: [1.45582639 0.1445634  1.30910674 1.05960974 1.73724198 1.02964939
 2.33770995 2.14115522 0.83447024 1.98560908 1.72294543 2.00173997], Cost: 0.0258
Current parameters: [1.45204494 0.16011119 1.30361648 1.05962982 1.7460967  1.03632581
 2.37620751 2.15512232 0.85012142 2.026797   1.70507319 2.02553991], Cost: 0.0747
Current parameters: [1.4569073  0.17261466 1.29861601 1.06719272 1.75706037 1.01212833
 2.34687218 2.13106844 0.84269132 1.98909149 1.72817734 2.00397496], Cost: 0.0241
Current parameters: [1.46049711 0.1634735  1.27739525 1.04551204 1.71153146 0.98649176
 2.34758597 2.14665082 0.84164288 1.97448694 1.70367249 2.01321384], Cost: 0.0411
Current parameters: [1.456062   0.17987988 1.30384866 1.03869929 1.74293411 1.03285826
 2.33713822 2.14256346 0.83612495 1.98590222 1.72415582 2.01558285], Cost: 0.0342
Current parameters: [1.4589756  0.15085761 1.26388832 1.04309879 1.74784928 1.0512701
 2.35879361 2.10880284 0.85196275 1.97301705 1.72312696 2.00380533], Cost: 0.0158
Current parameters: [1.45861334 0.16231532 1.25167293 1.05660097 1.74097464 1.06199534
 2.35637888 2.11908611 0.84459113 1.97453313 1.71990913 1.99066127], Cost: 0.0322
Current parameters: [1.46027329 0.13350486 1.25224916 1.02897746 1.78841439 1.04474192
 2.34788679 2.13873334 0.86198139 1.95380645 1.71993647 1.9945302 ], Cost: 0.0879
Current parameters: [1.46045185 0.14796308 1.26216279 1.0433984  1.75347574 1.04876093
 2.3623865  2.09796544 0.82507305 1.97191084 1.71900829 2.01111173], Cost: 0.0094
Current parameters: [1.4609873  0.14724042 1.26100962 1.03566286 1.75342262 1.04579222
 2.36213279 2.09619358 0.82292763 1.98044215 1.7207021  2.00163078], Cost: 0.0136
Current parameters: [1.46215001 0.15150166 1.27258486 1.0470335  1.7504089  1.04993275
 2.35286759 2.07638573 0.83027033 1.9693564  1.7037175  2.0088968 ], Cost: 0.0058
Current parameters: [1.47763402 0.15155891 1.2735179  1.04753528 1.75027514 1.05081468
 2.35353061 2.07734855 0.83060782 1.96982682 1.70451198 2.00925981], Cost: 0.0040
Current parameters: [1.47854369 0.15144725 1.27986815 1.05123424 1.73833395 1.04964151
 2.35847261 2.06255421 0.82908063 1.96334448 1.72621252 2.01363974], Cost: 0.0204
Current parameters: [1.47642436 0.15147788 1.27885605 1.04994244 1.75821709 1.06075574
 2.3541683  2.07612363 0.830759   1.97539736 1.70818895 2.00927423], Cost: 0.0012
Current parameters: [1.47639093 0.14793484 1.28149276 1.0527164  1.75343877 1.06091927
 2.37357782 2.07565674 0.83498321 1.98995619 1.6943361  2.02058127], Cost: 0.0030
Current parameters: [1.47614934 0.15356161 1.28502443 1.04753027 1.75999658 1.06140094
 2.36295758 2.07783536 0.82969397 1.96767743 1.70599255 2.00245254], Cost: 0.0031
Current parameters: [1.48068822 0.13761204 1.27700284 1.0485403  1.78112486 1.05858719
 2.34762731 2.07529805 0.83447136 1.9698023  1.69906587 2.01698583], Cost: 0.0057
Current parameters: [1.47688965 0.16255553 1.27312691 1.04827279 1.76413497 1.0583209
 2.35747906 2.0720324  0.83390139 1.9755027  1.70956257 2.0115496 ], Cost: 0.0019
Current parameters: [1.47700732 0.16096425 1.28754528 1.03119749 1.75435031 1.07019264
```

```
 2.34564088 2.08289067 0.82564402 1.97489176 1.70323729 2.02290879], Cost: 0.0054
Current parameters: [1.47582717 0.15556967 1.28545763 1.05703046 1.76092267 1.0532714
 2.35289555 2.08195092 0.82674971 1.97606251 1.70955276 2.01326556], Cost: 0.0019
Current parameters: [1.47849481 0.15395807 1.29014321 1.04308186 1.75960373 1.05126006
 2.34512645 2.06973974 0.8396975  1.99117752 1.70374598 1.99416109], Cost: 0.0091
Current parameters: [1.47789882 0.15534606 1.27360738 1.05787059 1.75776782 1.06793334
 2.35139879 2.07820612 0.82632775 1.97634397 1.7021098  2.00535187], Cost: 0.0018
Current parameters: [1.47607897 0.15246685 1.28011716 1.05212671 1.75689904 1.06211948
 2.35294651 2.07756333 0.83714147 1.97323181 1.70770264 2.01014126], Cost: 0.0008
Current parameters: [1.47609037 0.15075066 1.27480331 1.04999598 1.75857395 1.06336125
 2.35553077 2.0892372  0.83970339 1.97715593 1.71356519 2.00795456], Cost: 0.0018
Current parameters: [1.47826876 0.15020153 1.28048047 1.05800165 1.75354594 1.06734159
 2.35465192 2.07198324 0.83658052 1.97151865 1.71808319 2.01414152], Cost: 0.0047
Current parameters: [1.47637184 0.15488697 1.28190924 1.04793669 1.75572679 1.06523867
 2.35083442 2.07941533 0.83576015 1.97299592 1.70678335 2.01367982], Cost: 0.0014
Current parameters: [1.47598948 0.15404864 1.28114266 1.0518699  1.75489938 1.06185329
 2.35276836 2.07715523 0.83741486 1.97510119 1.70848477 2.00835737], Cost: 0.0011
Current parameters: [1.47775053 0.1503451  1.28157896 1.05133965 1.76121473 1.06150568
 2.34941604 2.07653494 0.83963742 1.97540633 1.7054059  2.00864932], Cost: 0.0018
Current parameters: [1.47807994 0.15210283 1.28044827 1.05245301 1.75628163 1.06212729
 2.35506376 2.07765309 0.83762693 1.97475363 1.70630342 2.01137609], Cost: 0.0007
Current parameters: [1.47164311 0.15113226 1.28026255 1.05299158 1.75618272 1.06255119
 2.35690884 2.07718991 0.83835668 1.97703747 1.70385155 2.01303961], Cost: 0.0008
Current parameters: [1.47833239 0.15316411 1.27973749 1.05444797 1.75683036 1.06441005
 2.35467576 2.07810349 0.83657861 1.97519032 1.70511716 2.01036551], Cost: 0.0004
Current parameters: [1.47894358 0.15325126 1.27556391 1.05547598 1.75331496 1.06177876
 2.35324648 2.07922764 0.83614762 1.97330062 1.70114561 2.01011995], Cost: 0.0007
Current parameters: [1.47819949 0.15074454 1.28161391 1.05463167 1.75559526 1.06503703
 2.3546571  2.07921114 0.83574939 1.97449965 1.70460538 2.00924314], Cost: 0.0001
Current parameters: [1.47799307 0.15291797 1.28313408 1.05361024 1.75727941 1.06526488
 2.35925821 2.0797117  0.83602102 1.97060796 1.70242816 2.00630272], Cost: 0.0007
Current parameters: [1.47807282 0.15182863 1.28371188 1.05639672 1.75591    1.06333889
 2.35403024 2.07880627 0.83461859 1.97407888 1.70407254 2.01037068], Cost: 0.0003
Current parameters: [1.47793897 0.15076454 1.2809989  1.05592595 1.75704494 1.06361382
 2.35546602 2.08609532 0.83570213 1.97615208 1.70660674 2.00928611], Cost: 0.0004
Current parameters: [1.47909604 0.14783036 1.28071197 1.05865898 1.75371445 1.06863724
 2.35606547 2.07777495 0.8354245  1.97302633 1.70765405 2.01085263], Cost: 0.0003
Current parameters: [1.47916831 0.1488494  1.28206408 1.05501925 1.75727558 1.06559063
 2.35312437 2.07881952 0.83690917 1.97550069 1.70329339 2.00842174], Cost: 0.0004
Current parameters: [1.47816811 0.15127747 1.28211755 1.05451219 1.75516417 1.06574227
 2.35411806 2.07975973 0.8368772  1.97402119 1.70438013 2.00990302], Cost: 0.0002
```



```
Current parameters: [1.47856903 0.15065008 1.28168438 1.05275629 1.75545083 1.06649584
 2.3542984  2.07959989 0.8330555  1.97494566 1.70416123 2.01047166], Cost: 0.0001
Current parameters: [1.476691   0.15054564 1.28199202 1.05468514 1.75594025 1.06544876
 2.35520683 2.07899834 0.83593693 1.97522118 1.70444607 2.00961274], Cost: 0.0002
Current parameters: [1.47723865 0.15011686 1.27961631 1.05515911 1.75368662 1.06419568
 2.35434714 2.07959135 0.83532431 1.97416582 1.70242017 2.0093628 ], Cost: 0.0003
Current parameters: [1.4783555  0.15102566 1.28184338 1.05437156 1.75599344 1.06535824
 2.35586964 2.07929284 0.83553001 1.97349076 1.70411316 2.00850551], Cost: 0.0000
Current parameters: [1.47856296 0.15163313 1.28236027 1.05425214 1.75500266 1.0656289
 2.35616403 2.07912719 0.8353148  1.97418549 1.70434746 2.00733575], Cost: 0.0000
Current parameters: [1.47849707 0.1517967  1.28236987 1.05411248 1.75525056 1.0653119
 2.35616116 2.07992484 0.83531454 1.97446228 1.704281   2.00718164], Cost: 0.0000
Current parameters: [1.47856296 0.15163313 1.28236027 1.05425214 1.75500266 1.0656289
 2.35616403 2.07912719 0.8353148  1.97418549 1.70434746 2.00733575], Cost: 0.0000

   Return from subroutine COBYLA because the MAXFUN limit has been reached.

   NFVALS =  100   F = 5.961791E-06    MAXCV = 0.000000E+00
   X = 1.478563E+00   1.516331E-01   1.282360E+00   1.054252E+00   1.755003E+00
       1.065629E+00   2.356164E+00   2.079127E+00   8.353148E-01   1.974185E+00
       1.704347E+00   2.007336E+00
Simulating for QAOA depth p = 7
Current parameters: [1.89990499 2.03499786 2.1525111  2.24207385 2.70513829 2.87259537
 1.30940788 1.78527132 1.10869963 0.75193155 1.88254399 1.57140877
 2.63270855 2.51106008], Cost: 0.5839
Current parameters: [1.89990499 3.03499786 2.1525111  2.24207385 2.70513829 2.87259537
 1.30940788 1.78527132 1.10869963 0.75193155 1.88254399 1.57140877
 2.63270855 2.51106008], Cost: 1.5573
Current parameters: [1.89990499 2.03499786 3.1525111  2.24207385 2.70513829 2.87259537
 1.30940788 1.78527132 1.10869963 0.75193155 1.88254399 1.57140877
 2.63270855 2.51106008], Cost: 0.6678
Current parameters: [1.89990499 2.03499786 2.1525111  3.24207385 2.70513829 2.87259537
 1.30940788 1.78527132 1.10869963 0.75193155 1.88254399 1.57140877
 2.63270855 2.51106008], Cost: 2.1193
Current parameters: [1.89990499 2.03499786 2.1525111  2.24207385 3.70513829 2.87259537
 1.30940788 1.78527132 1.10869963 0.75193155 1.88254399 1.57140877
 2.63270855 2.51106008], Cost: 1.6571
Current parameters: [1.89990499 2.03499786 2.1525111  2.24207385 2.70513829 3.87259537
 1.30940788 1.78527132 1.10869963 0.75193155 1.88254399 1.57140877
 2.63270855 2.51106008], Cost: 1.6562
Current parameters: [1.89990499 2.03499786 2.1525111  2.24207385 2.70513829 2.87259537
```



```
 2.30940788 1.78527132 1.10869963 0.75193155 1.88254399 1.57140877
 2.63270855 2.51106008], Cost: 3.3346
Current parameters: [1.89990499 2.03499786 2.1525111  2.24207385 2.70513829 2.87259537
 1.30940788 2.78527132 1.10869963 0.75193155 1.88254399 1.57140877
 2.63270855 2.51106008], Cost: 3.0003
Current parameters: [1.89990499 2.03499786 2.1525111  2.24207385 2.70513829 2.87259537
 1.30940788 1.78527132 2.10869963 0.75193155 1.88254399 1.57140877
 2.63270855 2.51106008], Cost: 1.2162
Current parameters: [1.89990499 2.03499786 2.1525111  2.24207385 2.70513829 2.87259537
 1.30940788 1.78527132 1.10869963 1.75193155 1.88254399 1.57140877
 2.63270855 2.51106008], Cost: 3.3353
Current parameters: [1.89990499 2.03499786 2.1525111  2.24207385 2.70513829 2.87259537
 1.30940788 1.78527132 1.10869963 0.75193155 2.88254399 1.57140877
 2.63270855 2.51106008], Cost: 3.3181
Current parameters: [1.89990499 2.03499786 2.1525111  2.24207385 2.70513829 2.87259537
 1.30940788 1.78527132 1.10869963 0.75193155 1.88254399 2.57140877
 2.63270855 2.51106008], Cost: 2.4748
Current parameters: [1.89990499 2.03499786 2.1525111  2.24207385 2.70513829 2.87259537
 1.30940788 1.78527132 1.10869963 0.75193155 1.88254399 1.57140877
 3.63270855 2.51106008], Cost: 2.1240
Current parameters: [1.89990499 2.03499786 2.1525111  2.24207385 2.70513829 2.87259537
 1.30940788 1.78527132 1.10869963 0.75193155 1.88254399 1.57140877
 2.63270855 3.51106008], Cost: 2.6575
Current parameters: [2.22419973 1.89728567 2.14063492 2.02486956 2.55331311 2.72089996
 0.92027408 1.44342238 1.01924065 0.36269432 1.49574062 1.30390758
 2.41483714 2.21771538], Cost: 1.8363
Current parameters: [2.06205236 1.96614177 2.14657301 2.13347171 2.6292257  2.79674767
 1.11484098 1.61434685 1.06397014 0.55731294 1.68914231 1.43765818
 2.52377284 2.36438773], Cost: 1.7567
Current parameters: [1.6573742  2.02616797 2.15174962 2.22814705 2.69540349 2.86286889
 1.28445722 1.76335249 1.10296366 0.72697426 1.85774275 1.55425701
 2.61873897 2.49225127], Cost: 1.3959
Current parameters: [1.89990499 2.04573789 2.02797335 2.24207385 2.70513829 2.87259537
 1.30940788 1.78527132 1.10869963 0.75193155 1.88254399 1.57140877
 2.63270855 2.51106008], Cost: 0.5361
Current parameters: [1.90913978 2.29357877 2.04793589 2.23643075 2.70119378 2.86865422
 1.29929794 1.77638986 1.10637543 0.74181892 1.87249459 1.56445892
 2.62704811 2.5034388 ], Cost: 0.4840
Current parameters: [1.90913978 2.29357877 2.04793589 2.23643075 2.70119378 2.86865422
 1.21089784 1.77638986 1.10637543 0.83019552 1.87249459 1.56445892
 2.62704811 2.5034388 ], Cost: 0.1996
```



```
Current parameters: [1.95257388 2.30168963 2.04199887 2.20988946 2.68264149 2.8501178
 1.34183673 1.73461762 1.09544398 1.01678241 1.82522914 1.5317716
 2.6004253  2.46759353], Cost: 0.2948
Current parameters: [1.90913978 2.29357877 2.04793589 2.23643075 2.70119378 2.86865422
 1.21089784 1.68272645 1.10637543 0.83019552 1.9552724  1.56445892
 2.62704811 2.5034388 ], Cost: 0.5098
Current parameters: [1.96390117 2.30380488 2.04045053 2.20296769 2.67780319 2.84528364
 1.18002082 1.95994777 1.09259314 0.869427   1.9985264  1.52324699
 2.59348227 2.45824535], Cost: 0.5757
Current parameters: [1.90913978 2.29357877 2.04793589 2.23643075 2.70119378 2.86865422
 1.21089784 1.77638986 1.10637543 0.83019552 1.87249459 1.47209569
 2.62704811 2.58766488], Cost: 0.2654
Current parameters: [1.95905441 2.3028998  2.04111304 2.2059294  2.67987342 2.84735209
 1.18275365 1.79930323 1.09381297 0.86595474 1.82398438 1.72566361
 2.59645308 2.66470418], Cost: 1.1209
Current parameters: [1.90913978 2.29357877 2.04793589 2.14790697 2.70119378 2.86865422
 1.21089784 1.77638986 1.10637543 0.83019552 1.87249459 1.56445892
 2.71530082 2.5034388 ], Cost: 0.6636
Current parameters: [1.94475049 2.3002287  2.04306825 2.43715881 2.68598314 2.85345659
 1.19081886 1.792737   1.09741296 0.85570731 1.83788584 1.54247054
 2.75389204 2.46825833], Cost: 0.1645
Current parameters: [1.94475049 2.3002287  2.04306825 2.43715881 2.77433369 2.76503046
 1.19081886 1.792737   1.09741296 0.85570731 1.83788584 1.54247054
 2.75389204 2.46825833], Cost: 0.1698
Current parameters: [1.97170504 2.30526218 2.03938382 2.4628063  2.85553063 3.02349986
 1.17562063 1.80511052 1.09062908 0.87501775 1.81168969 1.52582705
 2.72322708 2.44162945], Cost: 0.2978
Current parameters: [1.93442818 2.30461208 2.04344627 2.44407243 2.69081575 2.85828507
 1.20320501 1.80361807 1.21832598 0.86809675 1.8501978  1.55098512
 2.76082689 2.47759551], Cost: 0.4760
Current parameters: [2.04448178 2.27955509 2.0358679  2.42784542 2.64690339 2.81525302
 1.09370065 1.74125845 1.12242213 0.80396118 1.7267629  1.46755888
 2.67037325 2.37508973], Cost: 0.4867
Current parameters: [1.84104501 2.30110878 2.04314414 2.44395945 2.68353756 2.8510131
 1.15929571 1.80573356 1.09597197 0.86769458 1.80612922 1.55576134
 2.71713366 2.43716982], Cost: 0.0240
Current parameters: [1.73443304 2.31753613 2.0263081  2.52482216 2.66162127 2.83195505
 1.22316827 1.79897371 0.9578094  0.89535877 1.82231006 1.42023191
 2.73777291 2.44992284], Cost: 0.3777
Current parameters: [1.89088936 2.3025813  2.04327113 2.46439    2.62851151 2.87022492
 1.16182435 1.80990776 1.03611909 0.87231252 1.80862246 1.60613481
```

```
 2.73553541 2.49021837], Cost: 0.1967
Current parameters: [1.76595702 2.31039293 2.03197896 2.47545464 2.77161828 2.79210599
 1.09871013 1.82885346 1.12076791 0.90907964 1.71367229 1.63974472
 2.58317108 2.50312858], Cost: 0.1656
Current parameters: [1.81446194 2.30035897 2.0414931  2.45800183 2.69758656 2.85597712
 1.09075022 1.8189314  1.09681007 0.78154794 1.83854453 1.55127602
 2.74041822 2.46142253], Cost: 0.1313
Current parameters: [1.84307715 2.35307169 2.07739837 2.44271766 2.68266956 2.85014584
 1.15707098 1.80377917 1.09546052 0.86546926 1.80391782 1.554232
 2.71588806 2.43549273], Cost: 0.0219
Current parameters: [1.83696024 2.28525609 2.1815608  2.44944394 2.68653369 2.84924464
 1.15777809 1.80910385 1.09260452 0.87055392 1.8044453  1.5518776
 2.71288165 2.43516502], Cost: 0.0099
Current parameters: [1.84931473 2.28749758 2.17992514 2.45789385 2.68094116 2.84435067
 1.15351253 1.84759236 1.08905276 0.87677758 1.83679531 1.54841631
 2.68476144 2.42036202], Cost: 0.0961
Current parameters: [1.81861265 2.28459145 2.18343108 2.37564456 2.69849673 2.84311772
 1.12577578 1.85095778 1.07545303 0.90738205 1.77461644 1.56189998
 2.7703859  2.4046021 ], Cost: 0.0127
Current parameters: [1.83704795 2.28530643 2.18152132 2.45092509 2.6866596  2.84901114
 1.12468855 1.77808913 1.09281156 0.90361048 1.83162685 1.54878894
 2.7136787  2.43747865], Cost: 0.0209
Current parameters: [1.80909527 2.2825488  2.18508966 2.41937708 2.70046657 2.8488888
 1.1908835  1.75595918 1.07244621 0.84208782 1.82816386 1.6143145
 2.69906674 2.37238202], Cost: 0.0659
Current parameters: [1.84076123 2.28656574 2.18032554 2.49344354 2.689481   2.84207296
 1.14990056 1.81293415 1.09889091 0.87646358 1.7929608  1.5662693
 2.73606166 2.40575449], Cost: 0.0102
Current parameters: [1.88040834 2.27307887 2.1808036  2.43271671 2.65494954 2.83476599
 1.11768298 1.77442432 1.0846944  0.84566027 1.7429003  1.51011458
 2.67843143 2.39168965], Cost: 0.3746
Current parameters: [1.82013298 2.2849801  2.18204051 2.44657534 2.65254914 2.80205652
 1.16228118 1.80789758 1.10256665 0.8686814  1.81029804 1.55399747
 2.71905885 2.44077762], Cost: 0.0055
Current parameters: [1.74738386 2.29360111 2.17957215 2.47566616 2.67594271 2.80528662
 1.2110692  1.80301514 1.0586468  0.89616206 1.81480751 1.50055477
 2.74615904 2.45298653], Cost: 0.0132
Current parameters: [1.81183562 2.28078312 2.18995419 2.44473113 2.678256   2.81359578
 1.16878406 1.81111384 1.11086252 0.86417611 1.78153778 1.56680515
 2.69754483 2.47770961], Cost: 0.0285
Current parameters: [1.77498249 2.30276594 2.160591   2.46966865 2.664462   2.73857651
```



```
 1.12386984 1.81598317 1.11063479 0.9231571  1.81216147 1.60586483
 2.69723357 2.42176331], Cost: 0.1045
Current parameters: [1.81206681 2.28745968 2.18392817 2.4422441  2.64425544 2.82083374
 1.17991163 1.80778647 1.15013046 0.88008359 1.82290719 1.55755689
 2.741769   2.44165226], Cost: 0.0861
Current parameters: [1.82948722 2.28465782 2.18201272 2.44292508 2.67338211 2.78654347
 1.16674203 1.80538995 1.110455   0.86627012 1.81479886 1.54552515
 2.72228035 2.43869881], Cost: 0.0064
Current parameters: [1.8470662  2.28624349 2.18344119 2.45078369 2.64934128 2.79313258
 1.17509523 1.80586561 1.07351948 0.87535113 1.81925475 1.57812675
 2.74549838 2.46630428], Cost: 0.0931
Current parameters: [1.82088342 2.31093345 2.19913757 2.44609483 2.65225544 2.80167269
 1.16048685 1.80705263 1.10231948 0.86671143 1.80951614 1.55318872
 2.71866851 2.44018135], Cost: 0.0060
Current parameters: [1.79903327 2.28279655 2.18046041 2.45184655 2.6618651  2.80388871
 1.1308232  1.81388116 1.10154653 0.82890022 1.8277477  1.55404695
 2.73682687 2.45110584], Cost: 0.0297
Current parameters: [1.82270551 2.30118387 2.15680492 2.443565   2.65290731 2.80546692
 1.16392254 1.80568561 1.10308257 0.86470718 1.80655102 1.55253056
 2.7188571  2.44391538], Cost: 0.0117
Current parameters: [1.82409288 2.27551071 2.18748858 2.44478056 2.63944852 2.79515783
 1.14787324 1.78497216 1.09679555 0.8591601  1.77443718 1.52100569
 2.71275386 2.424121  ], Cost: 0.1106
Current parameters: [1.822002   2.28722566 2.17930502 2.45659662 2.64883579 2.79289946
 1.14836437 1.82068622 1.1087937  0.88254425 1.80660981 1.54657282
 2.72152308 2.4507523 ], Cost: 0.0020
Current parameters: [1.79424223 2.29387077 2.17105419 2.46053555 2.65875535 2.76650057
 1.13802829 1.81451024 1.10834476 0.89956922 1.81000313 1.57930745
 2.7051215  2.43012497], Cost: 0.0304
Current parameters: [1.82539833 2.28747493 2.17879004 2.47772782 2.643494   2.79001978
 1.15314391 1.80843078 1.11421785 0.87665851 1.81553841 1.54522819
 2.71006697 2.45636226], Cost: 0.0019
Current parameters: [1.83148232 2.288963   2.17778088 2.47683437 2.64732624 2.79856375
 1.16115528 1.84954434 1.10415969 0.87304532 1.83818697 1.53282251
 2.67652483 2.44555226], Cost: 0.0978
Current parameters: [1.84411336 2.28534592 2.17944745 2.47062823 2.63738398 2.78863702
 1.14157482 1.80988368 1.12450135 0.86963958 1.81435618 1.55924081
 2.70397852 2.45393207], Cost: 0.0062
Current parameters: [1.84698667 2.28775163 2.18098515 2.48454655 2.640885   2.77608926
 1.16367095 1.80317766 1.08066858 0.88481999 1.82189511 1.56798232
 2.73731545 2.47995722], Cost: 0.0815
```



```
Current parameters: [1.8192701  2.27663663 2.18448133 2.47545161 2.65026542 2.79731749
 1.16031756 1.80943698 1.11322884 0.87455047 1.80343905 1.55566434
 2.69767721 2.4711321 ], Cost: 0.0046
Current parameters: [1.82913615 2.32131199 2.17425603 2.48388859 2.673641   2.78357951
 1.1374847  1.81002444 1.13648895 0.87553351 1.79786516 1.53241868
 2.71263864 2.47895524], Cost: 0.0174
Current parameters: [1.81308051 2.28204651 2.17921035 2.47980806 2.64621754 2.79190372
 1.13900412 1.81088113 1.11186902 0.85668277 1.82450595 1.54764857
 2.7179433  2.46095556], Cost: 0.0041
Current parameters: [1.82858913 2.28573724 2.17899676 2.48353242 2.65002622 2.79940451
 1.14900893 1.80646428 1.11193552 0.88014023 1.81557198 1.54730651
 2.71123774 2.45162171], Cost: 0.0023
Current parameters: [1.82456265 2.28679431 2.17928088 2.46390029 2.64396274 2.79418302
 1.14310196 1.7955062  1.11165705 0.88704522 1.8291229  1.53940371
 2.70244098 2.46672861], Cost: 0.0089
Current parameters: [1.82743879 2.28634194 2.17876557 2.47820031 2.64335914 2.7857952
 1.1482235  1.80546838 1.10542392 0.87343113 1.80864241 1.54017809
 2.7053878  2.45414506], Cost: 0.0022
Current parameters: [1.8178134  2.27862148 2.18333427 2.4770027  2.63429181 2.79501741
 1.15236981 1.79561495 1.12895391 0.87618199 1.80341208 1.53691578
 2.71742689 2.44879392], Cost: 0.0016
Current parameters: [1.81497637 2.27874325 2.18292183 2.47581035 2.63528142 2.79866162
 1.15183448 1.80238803 1.13311091 0.87433513 1.80555408 1.53104402
 2.70819564 2.44340029], Cost: 0.0066
Current parameters: [1.82863594 2.27119281 2.19167653 2.48426101 2.62437899 2.79527369
 1.16332093 1.80161424 1.11925681 0.87699124 1.80345003 1.53121994
 2.73397388 2.45396982], Cost: 0.0021
Current parameters: [1.81359437 2.27221811 2.18337621 2.47626712 2.62904581 2.79317271
 1.15447667 1.79456927 1.12410023 0.87857135 1.80765413 1.54338776
 2.71502695 2.44148433], Cost: 0.0031
Current parameters: [1.8114541  2.28785931 2.18527583 2.48890143 2.64115022 2.77790278
 1.15005237 1.79396628 1.13240226 0.88578689 1.79424085 1.54397762
 2.72828703 2.44994678], Cost: 0.0056
Current parameters: [1.81640373 2.2845202  2.17322679 2.47746049 2.63021459 2.79447042
 1.16084757 1.79693132 1.13070601 0.87533269 1.80604686 1.5378237
 2.71828427 2.45026598], Cost: 0.0029
Current parameters: [1.82089478 2.27415121 2.17006941 2.47577175 2.6343865  2.80766768
 1.14324578 1.78561565 1.11859685 0.87134252 1.78767762 1.53897375
 2.7240459  2.44897086], Cost: 0.0285
Current parameters: [1.82120679 2.27275721 2.18139517 2.474279   2.64345606 2.78975145
 1.15789689 1.79248214 1.13118522 0.8732341  1.80615603 1.53490842
```



```
 2.71926987 2.44627279], Cost: 0.0034
Current parameters: [1.81779664 2.2821411  2.18653494 2.47675882 2.63498879 2.79626536
 1.15485595 1.79468913 1.12796933 0.87237143 1.80202685 1.53864926
 2.71773454 2.44583521], Cost: 0.0025
Current parameters: [1.81716019 2.28335339 2.18936083 2.47203124 2.63308836 2.79416877
 1.15305334 1.78971781 1.12852999 0.88122273 1.81151446 1.53426198
 2.71689756 2.45372509], Cost: 0.0033
Current parameters: [1.8214511  2.28047015 2.18224497 2.47664723 2.63227255 2.79352505
 1.14892513 1.79543589 1.13014923 0.87564367 1.80554207 1.53629545
 2.71942885 2.44502279], Cost: 0.0015
Current parameters: [1.82814357 2.2786629  2.18525251 2.47464371 2.6291228  2.79099963
 1.1495626  1.79824687 1.13681248 0.87436514 1.80455839 1.54474438
 2.71549813 2.44998092], Cost: 0.0024
Current parameters: [1.81992423 2.28084769 2.18222401 2.4710819  2.63311614 2.79350403
 1.14848541 1.79929955 1.12967883 0.87643072 1.80486556 1.53726178
 2.72251115 2.44487429], Cost: 0.0017
Current parameters: [1.81550333 2.27872735 2.18298691 2.47961825 2.63202715 2.79228332
 1.14454659 1.79705418 1.13103149 0.86612477 1.81227592 1.53788671
 2.72308476 2.44953132], Cost: 0.0038
Current parameters: [1.82047686 2.27999643 2.18263962 2.47469613 2.62896986 2.78920914
 1.14912938 1.79456334 1.12933599 0.8733408  1.80262302 1.53321579
 2.71829704 2.44557994], Cost: 0.0036
Current parameters: [1.82631211 2.27752581 2.18747408 2.48191107 2.62890222 2.79456261
 1.15437306 1.80226748 1.12742327 0.87776835 1.80717107 1.53202806
 2.72531419 2.44576949], Cost: 0.0001
Current parameters: [1.82423097 2.27573213 2.18838841 2.48156763 2.62683816 2.79679225
 1.15526361 1.80001156 1.13257743 0.87829427 1.8059619  1.53127895
 2.72798879 2.44458622], Cost: 0.0005
Current parameters: [1.8329995  2.27507855 2.18352148 2.48302178 2.62926208 2.80319138
 1.15119921 1.79926872 1.12178241 0.88037265 1.80420624 1.53420703
 2.7298671  2.44894027], Cost: 0.0018
Current parameters: [1.82366062 2.27674036 2.18758434 2.48501148 2.63064634 2.79257387
 1.15260042 1.80358324 1.12804714 0.88101528 1.80367958 1.53252897
 2.72578417 2.44865022], Cost: 0.0000
Current parameters: [1.82628789 2.28420363 2.18919978 2.48965001 2.63318933 2.78827919
 1.15345098 1.80336349 1.12930901 0.88596317 1.80644759 1.53669507
 2.73054244 2.44103471], Cost: 0.0037
Current parameters: [1.82397352 2.27698256 2.18612527 2.48718884 2.63223775 2.79563368
 1.15018085 1.80424034 1.13013028 0.88255886 1.80489838 1.53021045
 2.72161438 2.44644624], Cost: 0.0010
Current parameters: [1.8221993  2.27379887 2.19212901 2.47973784 2.62958245 2.79448996
```

```
 1.15675168 1.81540324 1.12693118 0.87973333 1.80206463 1.53051694
 2.72552658 2.45232821], Cost: 0.0024
Current parameters: [1.82145677 2.27602603 2.18756065 2.48753922 2.63095394 2.79293259
 1.14980505 1.80423182 1.12833261 0.87686088 1.80745186 1.53348408
 2.72795031 2.45055937], Cost: 0.0004
Current parameters: [1.82439081 2.27496903 2.18618312 2.48541912 2.63229398 2.79182155
 1.15333303 1.80417137 1.12850153 0.87959742 1.80266692 1.53266429
 2.72623053 2.44729551], Cost: 0.0002
Current parameters: [1.82449447 2.27472311 2.18867636 2.48336968 2.63466505 2.79131485
 1.15409655 1.80098586 1.1287179  0.88285788 1.80686169 1.53057389
 2.72603144 2.45144452], Cost: 0.0003
Current parameters: [1.82341288 2.27476061 2.18973045 2.48473411 2.63064075 2.79237704
 1.15121141 1.80317936 1.12697429 0.88138691 1.80373957 1.53331917
 2.72534063 2.44711718], Cost: 0.0000
Current parameters: [1.82079552 2.2721303  2.186326   2.48541715 2.6283866  2.79213971
 1.1539968  1.8032233  1.12555002 0.88382161 1.80632577 1.53576843
 2.7240798  2.44683779], Cost: 0.0003
Current parameters: [1.82157009 2.27517806 2.18932553 2.48466093 2.6312038  2.79272789
 1.15138296 1.80291811 1.12512603 0.88149278 1.80356709 1.53111014
 2.7265374  2.44601608], Cost: 0.0002
Current parameters: [1.82467773 2.27354526 2.18889787 2.48508551 2.62666553 2.78816561
 1.14978187 1.8008762  1.12649546 0.88000441 1.80242351 1.53048415
 2.72378075 2.44849984], Cost: 0.0009
Current parameters: [1.82365886 2.27467345 2.19125328 2.48717448 2.63058073 2.79309648
 1.15325396 1.80250425 1.12635113 0.8806508  1.80337702 1.53304011
 2.72461099 2.44742607], Cost: 0.0001
Current parameters: [1.82032521 2.27639099 2.18947078 2.4822123  2.63276331 2.79291627
 1.15136781 1.80019673 1.12667078 0.87911708 1.80141036 1.53639616
 2.72255725 2.44802009], Cost: 0.0004
Current parameters: [1.8242087  2.2758336  2.18873607 2.48487934 2.62960166 2.79391243
 1.15104475 1.80260393 1.12602284 0.88257864 1.80241113 1.5337231
 2.72643232 2.44891248], Cost: 0.0002
Current parameters: [1.82637362 2.26977216 2.18957143 2.48436926 2.63310147 2.7949063
 1.14930013 1.80210083 1.1250574  0.88002764 1.80435746 1.53456863
 2.72636484 2.44591566], Cost: 0.0002

   Return from subroutine COBYLA because the MAXFUN limit has been reached.

Current parameters: [1.82341288 2.27476061 2.18973045 2.48473411 2.63064075 2.79237704
 1.15121141 1.80317936 1.12697429 0.88138691 1.80373957 1.53331917
 2.72534063 2.44711718], Cost: 0.0000
```



```
   NFVALS =  100   F = 1.038903E-05    MAXCV = 0.000000E+00
   X = 1.823413E+00   2.274761E+00   2.189730E+00   2.484734E+00   2.630641E+00
       2.792377E+00   1.151211E+00   1.803179E+00   1.126974E+00   8.813869E-01
       1.803740E+00   1.533319E+00   2.725341E+00   2.447117E+00
Simulating for QAOA depth p = 8
Current parameters: [2.16209609 2.17965647 2.41904769 1.67357431 0.62001483 0.66589562
 1.88474008 1.1063947  2.67440916 0.05197199 1.56847613 1.93458545
 0.02467749 1.65838266 1.64689193 2.75715284], Cost: 1.8772
Current parameters: [2.16209609 3.17965647 2.41904769 1.67357431 0.62001483 0.66589562
 1.88474008 1.1063947  2.67440916 0.05197199 1.56847613 1.93458545
 0.02467749 1.65838266 1.64689193 2.75715284], Cost: 1.0741
Current parameters: [2.16209609 3.17965647 3.41904769 1.67357431 0.62001483 0.66589562
 1.88474008 1.1063947  2.67440916 0.05197199 1.56847613 1.93458545
 0.02467749 1.65838266 1.64689193 2.75715284], Cost: 2.7425
Current parameters: [2.16209609 3.17965647 2.41904769 2.67357431 0.62001483 0.66589562
 1.88474008 1.1063947  2.67440916 0.05197199 1.56847613 1.93458545
 0.02467749 1.65838266 1.64689193 2.75715284], Cost: 2.7490
Current parameters: [2.16209609 3.17965647 2.41904769 1.67357431 1.62001483 0.66589562
 1.88474008 1.1063947  2.67440916 0.05197199 1.56847613 1.93458545
 0.02467749 1.65838266 1.64689193 2.75715284], Cost: 2.5868
Current parameters: [2.16209609 3.17965647 2.41904769 1.67357431 0.62001483 1.66589562
 1.88474008 1.1063947  2.67440916 0.05197199 1.56847613 1.93458545
 0.02467749 1.65838266 1.64689193 2.75715284], Cost: 2.5202
Current parameters: [2.16209609 3.17965647 2.41904769 1.67357431 0.62001483 0.66589562
 2.88474008 1.1063947  2.67440916 0.05197199 1.56847613 1.93458545
 0.02467749 1.65838266 1.64689193 2.75715284], Cost: 2.5987
Current parameters: [2.16209609 3.17965647 2.41904769 1.67357431 0.62001483 0.66589562
 1.88474008 2.1063947  2.67440916 0.05197199 1.56847613 1.93458545
 0.02467749 1.65838266 1.64689193 2.75715284], Cost: 2.4902
Current parameters: [2.16209609 3.17965647 2.41904769 1.67357431 0.62001483 0.66589562
 1.88474008 1.1063947  3.67440916 0.05197199 1.56847613 1.93458545
 0.02467749 1.65838266 1.64689193 2.75715284], Cost: 3.6594
Current parameters: [2.16209609 3.17965647 2.41904769 1.67357431 0.62001483 0.66589562
 1.88474008 1.1063947  2.67440916 1.05197199 1.56847613 1.93458545
 0.02467749 1.65838266 1.64689193 2.75715284], Cost: 3.6509
Current parameters: [2.16209609 3.17965647 2.41904769 1.67357431 0.62001483 0.66589562
 1.88474008 1.1063947  2.67440916 0.05197199 2.56847613 1.93458545
 0.02467749 1.65838266 1.64689193 2.75715284], Cost: 1.7737
Current parameters: [2.16209609 3.17965647 2.41904769 1.67357431 0.62001483 0.66589562
 1.88474008 1.1063947  2.67440916 0.05197199 1.56847613 2.93458545
 0.02467749 1.65838266 1.64689193 2.75715284], Cost: 3.5394
```



```
Current parameters: [2.16209609 3.17965647 2.41904769 1.67357431 0.62001483 0.66589562
 1.88474008 1.1063947  2.67440916 0.05197199 1.56847613 1.93458545
 1.02467749 1.65838266 1.64689193 2.75715284], Cost: 2.9384
Current parameters: [2.16209609 3.17965647 2.41904769 1.67357431 0.62001483 0.66589562
 1.88474008 1.1063947  2.67440916 0.05197199 1.56847613 1.93458545
 0.02467749 2.65838266 1.64689193 2.75715284], Cost: 2.5032
Current parameters: [2.16209609 3.17965647 2.41904769 1.67357431 0.62001483 0.66589562
 1.88474008 1.1063947  2.67440916 0.05197199 1.56847613 1.93458545
 0.02467749 1.65838266 2.64689193 2.75715284], Cost: 3.6776
Current parameters: [2.16209609 3.17965647 2.41904769 1.67357431 0.62001483 0.66589562
 1.88474008 1.1063947  2.67440916 0.05197199 1.56847613 1.93458545
 0.02467749 1.65838266 1.64689193 3.75715284], Cost: 2.5560
Current parameters: [ 2.3251912   3.29247519  2.18467559  1.43829112  0.40750982  0.46275455
  1.67056764  0.90746957  2.31122788 -0.3100109   1.47019429  1.58826716
 -0.23721859  1.45762543  1.28115561  2.54898529], Cost: 2.4230
Current parameters: [ 2.24364365  3.23606583  2.30186164  1.55593272  0.51376233  0.56432508
  1.77765386  1.00693214  2.49281852 -0.12901945  1.51933521  1.76142631
 -0.10627055  1.55800405  1.46402377  2.65306907], Cost: 2.6673
Current parameters: [2.39015547 3.17965647 2.41904769 1.67357431 0.62001483 0.66589562
 1.88474008 1.1063947  2.7768246  0.05197199 1.56847613 1.93458545
 0.02467749 1.65838266 1.64689193 2.75715284], Cost: 1.3012
Current parameters: [ 1.94788012e+00  3.19640601e+00  2.38425182e+00  1.63864318e+00
  5.88465440e-01  6.35736440e-01  1.85294313e+00  1.07686144e+00
  3.10517999e+00 -1.76951370e-03  1.55388480e+00  1.88316958e+00
 -1.42047090e-02  1.62857740e+00  1.59259318e+00  2.72624741e+00], Cost: 0.6143
Current parameters: [ 1.93022871  3.20571074  2.36492197  1.61923819  0.57093908  0.61898237
  1.83527925  1.06045508  3.11879524 -0.03162407  1.545779    1.85460696
 -0.0358046   1.61201993  1.83173526  2.70907878], Cost: 0.6097
Current parameters: [ 1.93022871  3.20571074  2.36492197  1.61923819  0.57093908  0.61898237
  1.83527925  1.06045508  3.11879524  0.05478837  1.545779    1.76428593
 -0.0358046   1.61201993  1.83173526  2.70907878], Cost: 0.9761
Current parameters: [ 1.8999112   3.22169225  2.33172163  1.58590879  0.54083636  0.59020611
  1.80494033  1.03227604  3.14218036  0.08686971  1.53185673  2.04869936
 -0.07290389  1.58358136  1.84420835  2.67959049], Cost: 1.2807
Current parameters: [ 1.93022871  3.20571074  2.36492197  1.52625173  0.57093908  0.61898237
  1.83527925  1.06045508  3.11879524 -0.03162407  1.545779    1.85460696
  0.04773293  1.61201993  1.83173526  2.70907878], Cost: 0.6185
Current parameters: [ 1.90803729  3.21740868  2.34062042  1.77719184  0.54890488  0.5979191
  1.81307216  1.03982895  3.13591238 -0.08286717  1.53558836  1.86467042
  0.13848393  1.59120384  1.84086515  2.68749432], Cost: 0.4461
Current parameters: [ 1.90803729  3.21740868  2.42494284  1.77719184  0.54890488  0.5979191
```



```
  1.72079687  1.03982895  3.13591238 -0.08286717  1.53558836  1.86467042
  0.13848393  1.59120384  1.84086515  2.68749432], Cost: 0.4944
Current parameters: [ 1.88357659  3.23030284  2.50928466  1.7883159   0.52461748  0.57470191
  1.97559762  1.0170936   3.1547799  -0.13935036  1.52435563  1.87576297
  0.14917838  1.5682591   1.85092866  2.66370265], Cost: 0.3398
Current parameters: [ 1.88357659  3.23030284  2.50928466  1.7883159   0.61208982  0.57470191
  1.97559762  1.0170936   3.1547799  -0.13935036  1.52435563  1.87576297
  0.14917838  1.5682591   1.85092866  2.5744077 ], Cost: 0.4135
Current parameters: [ 1.85598018  3.24484995  2.51234755  1.800866    0.68310845  0.54850842
  1.98787083  0.99144373  3.17606612 -0.20307434  1.51168294  1.88827751
  0.16124379  1.54237299  1.86228225  2.8339092 ], Cost: 0.9129
Current parameters: [ 1.88357659  3.23030284  2.50928466  1.7883159   0.52461748  0.66256705
  1.97559762  1.0170936   3.1547799  -0.13935036  1.52435563  1.87576297
  0.14917838  1.47935062  1.85092866  2.66370265], Cost: 0.2673
Current parameters: [ 1.85711695  3.24425072  2.51222138  1.80034902  0.49216736  0.835782
  1.98736526  0.99250031  3.17518928 -0.20044938  1.51220496  1.887762
  0.16074678  1.63636654  1.86181456  2.64624956], Cost: 0.7364
Current parameters: [ 1.88357659  3.33903346  2.50928466  1.7883159   0.52461748  0.66256705
  1.97559762  1.07875926  3.1547799  -0.13935036  1.52435563  1.87576297
  0.14917838  1.47935062  1.85092866  2.66370265], Cost: 0.3199
Current parameters: [ 1.86244211  3.10707153  2.51163035  1.79792729  0.49869814  0.65165199
  1.98499696  1.22255134  3.17108178 -0.18815284  1.51465036  1.88534713
  0.15841857  1.45724834  1.85962371  2.6497621 ], Cost: 0.5142
Current parameters: [ 1.87929164  3.22733879  2.51544225  1.79449742  0.53020055  0.66790411
  1.98122451  1.0223199   3.16432164 -0.1298401   1.64659071  1.88486168
  0.15605909  1.48462505  1.86053753  2.66917177], Cost: 0.7279
Current parameters: [ 1.88329773  3.25447569  2.46556051  1.75584709  0.44081481  0.60428274
  1.947066    0.95584976  3.10696918 -0.28942383  1.48930483  1.82078704
  0.1107409   1.4039346   1.79062426  2.59963784], Cost: 0.9356
Current parameters: [ 1.92791138  3.23330299  2.50305209  1.7820591   0.51896641  0.657165
  1.96990222  1.01180366  3.26907927 -0.14897644  1.52174206  1.86655346
  0.14221387  1.47401196  1.84120277  2.65816693], Cost: 0.7815
Current parameters: [ 2.09597845  3.22089741  2.53118543  1.81445754  0.52841327  0.67413423
  1.99976615  1.02690427  3.05011098 -0.13554828  1.49702945  1.91162822
  0.17744174  1.48430195  1.88798794  2.67397806], Cost: 0.3383
Current parameters: [ 1.81457481  3.23470401  2.50564248  1.79433907  0.48390452  0.64213873
  1.98194783  0.99355715  3.11527709 -0.03102594  1.43778937  2.0544642
  0.15393757  1.44406861  1.87105584  2.63909343], Cost: 0.5896
Current parameters: [ 1.86647395  3.22483816  2.52312846  1.80659733  0.52022651  0.66674392
  1.99263163  1.01958411  3.12597628 -0.07174186  1.49164707  1.78876774
  0.16864662  1.47664272  1.85438203  2.66631828], Cost: 0.4124
```

```
Current parameters: [ 1.87912944  3.2326471   2.50441672  1.78342903  0.52055362  0.65834598
  1.97114923  1.01296014  3.15821016 -0.14689328  1.52231343  1.86863818
  0.14373875  1.47517909  1.91072631  2.65972174], Cost: 0.2132
Current parameters: [ 1.85661933  3.23309479  2.50597319  1.78844839  0.59695157  0.65307448
  1.97602802  1.00653662  3.14245769 -0.18264185  1.49237732  1.87497293
  0.14863114  1.46457914  1.92146547  2.74001467], Cost: 0.2917
Current parameters: [ 1.87919492  3.23281115  2.5040763   1.82957251  0.52031236  0.65805059
  1.97083815  1.01267087  3.15796251 -0.1473555   1.52254528  1.86838229
  0.10159679  1.47488716  1.91045135  2.65948541], Cost: 0.2133
Current parameters: [ 1.86572393  3.24326951  2.48530877  1.76751802  0.4834844   0.63249778
  1.95889097  0.98574964  3.09810889 -0.21742931  1.50773465  1.84503269
  0.1288689   1.44152047  1.89398617  2.63161928], Cost: 0.1026
Current parameters: [ 1.8587765   3.24693177  2.47808614  1.80539435  0.47666208  0.64059647
  1.95229083  0.97929224  3.10346773 -0.23347192  1.50454429  1.84818324
  0.17055004  1.44952413  1.89684445  2.62493621], Cost: 0.0729
Current parameters: [ 1.84124499  3.24684308  2.4823935   1.78463687  0.46214472  0.72074507
  1.95962551  0.97390888  3.08254682 -0.2638115   1.47915024  1.85001738
  0.14706946  1.52343221  1.90407477  2.61607812], Cost: 0.1796
Current parameters: [ 1.85770466  3.24752034  2.43494054  1.80542336  0.47599207  0.64004437
  1.99708223  0.97825446  3.10427109 -0.23004445  1.50407603  1.85283148
  0.17054686  1.4489785   1.89724956  2.62427988], Cost: 0.0594
Current parameters: [ 1.81959363  3.25213879  2.43693882  1.80869546  0.44959787  0.62372054
  1.98802985  0.96066554  3.07450539 -0.19125068  1.45744787  1.9369026
  0.17369604  1.4238095   1.91229368  2.60756852], Cost: 0.2844
Current parameters: [ 1.85010208  3.23934677  2.47385447  1.80888081  0.46851769  0.63315686
  2.03525241  0.99266632  3.11013527 -0.24759988  1.50058481  1.85627913
  0.17387078  1.44217182  1.90037738  2.61695806], Cost: 0.0631
Current parameters: [ 1.84920526  3.19027092  2.4007537   1.80780655  0.46898697  0.63665546
  1.96900881  1.07643098  3.09362884 -0.24647529  1.4915019   1.85231108
  0.17301777  1.44298187  1.9007665   2.62009671], Cost: 0.2622
Current parameters: [ 1.90935133  3.23931779  2.44037345  1.81159486  0.47863008  0.6479932
  2.0027643   0.97807062  3.07890804 -0.23149391  1.49797234  1.86810134
  0.17727223  1.44667525  1.89191092  2.62686404], Cost: 0.0795
Current parameters: [ 1.88404329  3.29213481  2.43315685  1.80919211  0.46280996  0.60425977
  2.00256895  0.97790083  3.09297931 -0.23225004  1.50627474  1.80706019
  0.17488084  1.4654167   1.99161187  2.62061277], Cost: 0.1672
Current parameters: [ 1.89481336  3.25086151  2.42277011  1.79678533  0.45981556  0.63814449
  1.99137729  0.9453531   3.18941166 -0.26464203  1.45216281  1.83555678
  0.16169383  1.42540333  1.87410195  2.61933738], Cost: 0.0880
Current parameters: [ 1.85725819  3.2447568   2.43318264  1.80685083  0.46988409  0.64752943
  2.00033338  0.9556664   3.10886652 -0.24764516  1.55538361  1.85217878
```



```
  0.17283738  1.43464227  1.88779643  2.62818226], Cost: 0.0842
Current parameters: [ 1.85908072  3.2498308   2.433232    1.8032754   0.49554166  0.63813029
  1.99508821  0.97862933  3.10735551 -0.23724856  1.50716162  1.86091935
  0.16824291  1.45017247  1.90110821  2.60393631], Cost: 0.0623
Current parameters: [ 1.8514806   3.23987102  2.43982708  1.81412908  0.4920139   0.64859915
  2.0063092   0.96396191  3.09156973 -0.21045212  1.49577076  1.81319559
  0.18024912  1.43661351  1.88021277  2.60510725], Cost: 0.0706
Current parameters: [ 1.85757095  3.22683976  2.43479418  1.80527643  0.47586989  0.62579031
  1.99694849  0.96636139  3.10437422 -0.23027124  1.50401463  1.85261727
  0.17038331  1.46314798  1.89904745  2.62416019], Cost: 0.0729
Current parameters: [ 1.85966187  3.27825535  2.43633498  1.80875315  0.47651851  0.61439685
  2.00116391  0.98665172  3.10152266 -0.23310733  1.50872364  1.84980414
  0.17457315  1.47412134  1.85847927  2.62481926], Cost: 0.0437
Current parameters: [ 1.85961913  3.27817352  2.43650588  1.78568368  0.47667311  0.6145422
  2.00132161  0.98679349  3.10163948 -0.23289211  1.5085944   1.8499349
  0.19564627  1.47426259  1.85862152  2.62497313], Cost: 0.0527
Current parameters: [ 1.84827444  3.28302914  2.43637407  1.81266159  0.51337317  0.61054122
  2.00468619  0.97853275  3.09310556 -0.25525974  1.49615171  1.84867564
  0.17689718  1.46681575  1.85903977  2.66364017], Cost: 0.1056
Current parameters: [ 1.86495787  3.27648717  2.43878501  1.81542801  0.48533257  0.60487156
  2.00191948  0.99218724  3.11549733 -0.2158874   1.51562716  1.85354465
  0.18157899  1.467021    1.8601006   2.63111275], Cost: 0.0175
Current parameters: [ 1.86683511  3.281161    2.44823902  1.81887411  0.49325437  0.64122514
  2.01280357  0.99363115  3.13849237 -0.19781088  1.52145017  1.85616979
  0.18053356  1.50377958  1.85967305  2.63620597], Cost: 0.0766
Current parameters: [ 1.86037293  3.28764325  2.44556801  1.81517567  0.48238057  0.60276225
  2.00541495  0.97148262  3.1125336  -0.20643129  1.51035848  1.86728119
  0.18125581  1.46394729  1.86187992  2.62922192], Cost: 0.0227
Current parameters: [ 1.85154333  3.27053835  2.42420465  1.83186118  0.46777755  0.58888712
  1.99123337  1.01988447  3.11823031 -0.1990908   1.51322238  1.87368123
  0.18957744  1.44490324  1.84392212  2.61538088], Cost: 0.0568
Current parameters: [ 1.8573997   3.27901842  2.44089005  1.81829567  0.48805185  0.60286372
  2.00373114  0.99970895  3.09517711 -0.20814804  1.52794504  1.85427886
  0.18459665  1.47386626  1.87224426  2.63187448], Cost: 0.0173
Current parameters: [ 1.87818066  3.30501696  2.4363128   1.82537967  0.47495281  0.57913753
  2.00493725  0.99897875  3.10867459 -0.20881084  1.52827783  1.82748175
  0.18735207  1.47084563  1.90413632  2.62437767], Cost: 0.0394
Current parameters: [ 1.86481394  3.27493445  2.4428562   1.80200355  0.49346474  0.59630063
  2.00607801  1.00521047  3.09993479 -0.20192361  1.53125214  1.84859571
  0.1667106   1.4663697   1.86726357  2.63655798], Cost: 0.0166
Current parameters: [ 1.84558781  3.29447345  2.44816105  1.79044806  0.47757857  0.59689663
```



```
  2.01305325  1.00220395  3.06632328 -0.17885876  1.53610589  1.86479373
  0.14573802  1.47377738  1.87487447  2.62305095], Cost: 0.0256
Current parameters: [ 1.86104051  3.2802452   2.43468765  1.80149136  0.48276122  0.60697079
  2.0066563   1.00066213  3.10329153 -0.20476996  1.54949019  1.84800774
  0.16638258  1.45467009  1.86716947  2.64788734], Cost: 0.0322
Current parameters: [ 1.87435564  3.26454629  2.46201811  1.8089312   0.50463865  0.57755286
  2.00912591  0.98699026  3.0937566  -0.21258784  1.56689524  1.85079185
  0.16985331  1.4707034   1.85026037  2.60954679], Cost: 0.0721
Current parameters: [ 1.88541471  3.27325144  2.44203688  1.80886986  0.49191589  0.60437294
  2.00851012  1.0081055   3.08393784 -0.20053223  1.53045725  1.85752802
  0.17417888  1.46484694  1.86691164  2.64137346], Cost: 0.0069
Current parameters: [ 1.92159614  3.28088703  2.44153671  1.78695654  0.48258865  0.59673663
  2.01373742  1.0061943   3.09798146 -0.19225478  1.51049355  1.85412868
  0.1449995   1.44826161  1.85801518  2.64058568], Cost: 0.0382
Current parameters: [ 1.88284875  3.25383061  2.4348081   1.80601542  0.48667933  0.59162742
  2.00790338  1.00194818  3.08700258 -0.19541154  1.52640373  1.85643118
  0.17093997  1.47648908  1.87188504  2.65033132], Cost: 0.0151
Current parameters: [ 1.90421974  3.2771704   2.43034307  1.82454072  0.46040022  0.61878865
  2.01371588  0.98784837  3.0592277  -0.19924748  1.54743967  1.87802702
  0.18551167  1.46292587  1.87041916  2.64852617], Cost: 0.0401
Current parameters: [ 1.87955587  3.28068449  2.44180445  1.81377456  0.50909353  0.5986339
  2.01078911  1.00989893  3.07424614 -0.20455183  1.52083613  1.86159968
  0.17485164  1.4599113   1.86899809  2.65804863], Cost: 0.0161
Current parameters: [ 1.88407647  3.27279417  2.43263855  1.81007517  0.49364058  0.60278557
  2.01871089  1.01072679  3.08525229 -0.20006038  1.5300467   1.8593965
  0.17550576  1.4636227   1.86468911  2.63666933], Cost: 0.0054
Current parameters: [ 1.87872423  3.26818516  2.43171081  1.81790501  0.48701264  0.59816324
  2.00550979  1.02822117  3.08712249 -0.19202588  1.52482396  1.86664006
  0.1813841   1.45458598  1.85874114  2.63225394], Cost: 0.0118
Current parameters: [ 1.88547824  3.2751262   2.42409725  1.809885    0.49891438  0.60412683
  2.01075079  1.00653236  3.08311626 -0.19532368  1.53061078  1.85607474
  0.17543266  1.46496001  1.86186865  2.63441568], Cost: 0.0093
Current parameters: [ 1.87980075  3.26569604  2.43954429  1.81528205  0.49144806  0.60846556
  2.02726803  1.00582381  3.07815647 -0.18909352  1.52364339  1.84037594
  0.18031388  1.45441701  1.86051263  2.63644377], Cost: 0.0099
Current parameters: [ 1.8836567   3.26716846  2.43146952  1.80852897  0.49660308  0.60470869
  2.01679805  1.00818539  3.083792   -0.20216175  1.52933402  1.86181509
  0.17383333  1.45567636  1.8738362   2.63182682], Cost: 0.0115
Current parameters: [ 1.88533258  3.27212108  2.43731917  1.81428621  0.50207574  0.62025478
  2.02379069  1.01849711  3.0961912  -0.18924958  1.52879137  1.86367306
  0.17563171  1.47677386  1.87121592  2.63731526], Cost: 0.0137
```



```
Current parameters: [ 1.88619565  3.27731418  2.43150736  1.81946326  0.49170065  0.59826369
  2.01837002  1.01219364  3.08685942 -0.20056666  1.52998002  1.85384188
  0.17232782  1.46492917  1.87202814  2.6346794 ], Cost: 0.0055
Current parameters: [ 1.88678817  3.28126353  2.43258427  1.79573798  0.49151996  0.59628472
  2.01980769  1.01515514  3.08587505 -0.19788344  1.52831926  1.85367131
  0.1968496   1.46647015  1.8754773   2.63482303], Cost: 0.0162
Current parameters: [ 1.8905422   3.26856236  2.43167626  1.81405395  0.49683833  0.6032404
  2.01761679  1.00962302  3.09243869 -0.20511101  1.53197159  1.85723542
  0.1816689   1.46031357  1.86222889  2.64045171], Cost: 0.0055
Current parameters: [ 1.88537409  3.26978301  2.44631194  1.81384763  0.50405137  0.58949263
  2.02250675  1.00926755  3.08215441 -0.20067354  1.54300133  1.86129744
  0.17493707  1.46951278  1.85576663  2.62374811], Cost: 0.0115
Current parameters: [ 1.88845773  3.27420903  2.43105857  1.81534915  0.48651114  0.60560286
  2.0212115   1.00689977  3.07898982 -0.19951092  1.53457041  1.86551099
  0.1785188   1.46418007  1.86418721  2.63910953], Cost: 0.0069
Current parameters: [ 1.88728167  3.27435224  2.43345778  1.80866805  0.49371647  0.60076184
  2.01975685  1.0096769   3.08741633 -0.19576695  1.52997436  1.86077237
  0.17349733  1.46063036  1.86460585  2.63784231], Cost: 0.0059
Current parameters: [ 1.89128315  3.27383878  2.4325175   1.8077624   0.48993107  0.60439426
  2.02048909  1.01379134  3.08431556 -0.20651643  1.52372609  1.85743433
  0.16972343  1.4651594   1.85917779  2.63459132], Cost: 0.0092
Current parameters: [ 1.88537578  3.27146273  2.43155621  1.8086213   0.49414587  0.60321638
  2.01896808  1.01497798  3.08306172 -0.19962893  1.53430685  1.85730551
  0.17391448  1.46289404  1.86537994  2.63882874], Cost: 0.0056
Current parameters: [ 1.87590939  3.27739202  2.43153452  1.80953001  0.4891756   0.60409355
  2.02001131  1.0086083   3.09196147 -0.20327434  1.53625538  1.8577216
  0.17245066  1.46116007  1.8616064   2.63898455], Cost: 0.0097
Current parameters: [ 1.88454678  3.2681279   2.43137816  1.80878368  0.48997175  0.60140997
  2.01823993  1.00933684  3.08734205 -0.19850279  1.53084777  1.85855278
  0.17481761  1.46649619  1.86525019  2.63562773], Cost: 0.0081
Current parameters: [ 1.8807511   3.27104295  2.43098758  1.81246957  0.4979818   0.59649751
  2.02103711  1.0102533   3.08221826 -0.20143849  1.52523317  1.86159591
  0.17405621  1.46606367  1.86479262  2.64717424], Cost: 0.0046
Current parameters: [ 1.88122552  3.27412791  2.43110103  1.80928101  0.49763029  0.59432818
  2.0220833   1.01137308  3.08173177 -0.2019463   1.52464246  1.86054988
  0.17926179  1.46687015  1.866761    2.64778641], Cost: 0.0071
Current parameters: [ 1.88138731  3.2714067   2.43129482  1.81191333  0.4993034   0.5981522
  2.02264775  1.00885975  3.08268004 -0.20154434  1.52567918  1.86109975
  0.17343727  1.46773239  1.8659309   2.64757955], Cost: 0.0040
Current parameters: [ 1.88072545  3.27150488  2.43245925  1.81454784  0.5008322   0.60140597
  2.02277132  1.01292353  3.08477967 -0.19891558  1.52446075  1.86348232
```

```
  0.17390709  1.46964199  1.86599763  2.64827615], Cost: 0.0026
Current parameters: [ 1.88127041  3.27202256  2.43245778  1.81417858  0.50175553  0.6000606
  2.02195707  1.01329178  3.08532512 -0.20013928  1.52590158  1.86565751
  0.17332405  1.47086381  1.86606311  2.64746571], Cost: 0.0033
Current parameters: [ 1.88009497  3.27048269  2.43662525  1.81711838  0.50360381  0.60034394
  2.02535092  1.01256956  3.08304683 -0.19748067  1.52575022  1.86186942
  0.17427169  1.47001818  1.86327772  2.64653204], Cost: 0.0017
Current parameters: [ 1.88145023  3.26880296  2.43676207  1.81706526  0.50464293  0.60038509
  2.02514395  1.01268191  3.08163763 -0.19737771  1.52452137  1.86264898
  0.1746347   1.46933398  1.86515306  2.64543155], Cost: 0.0021
Current parameters: [ 1.87598337  3.27066463  2.43767766  1.81905319  0.50529005  0.60337677
  2.02749945  1.01210625  3.08433619 -0.19866447  1.52653309  1.86244824
  0.1723614   1.4664852   1.86454966  2.6473148 ], Cost: 0.0007
Current parameters: [ 1.87512678  3.27232852  2.43439707  1.82088481  0.50941669  0.60576561
  2.02527302  1.01047612  3.08326266 -0.19617801  1.52645712  1.86103293
  0.17181542  1.4665941   1.86270331  2.64726052], Cost: 0.0007
Current parameters: [ 1.87547026  3.27232216  2.43224267  1.82093369  0.50950226  0.60552462
  2.02776647  1.0113551   3.08351255 -0.1966234   1.52578597  1.86127437
  0.17158341  1.46643155  1.86170492  2.64599757], Cost: 0.0005
Current parameters: [ 1.87512678  3.27232852  2.43439707  1.82088481  0.50941669  0.60576561
  2.02527302  1.01047612  3.08326266 -0.19617801  1.52645712  1.86103293
  0.17181542  1.4665941   1.86270331  2.64726052], Cost: 0.0007

   Return from subroutine COBYLA because the MAXFUN limit has been reached.

   NFVALS =  100   F = 6.922470E-04    MAXCV = 0.000000E+00
   X = 1.875127E+00   3.272329E+00   2.434397E+00   1.820885E+00   5.094167E-01
       6.057656E-01   2.025273E+00   1.010476E+00   3.083263E+00  -1.961780E-01
       1.526457E+00   1.861033E+00   1.718154E-01   1.466594E+00   1.862703E+00
       2.647261E+00
Simulating for QAOA depth p = 9
Current parameters: [3.66859018 0.19581898 2.64912555 2.80142123 1.45359209 0.69007078
 2.96999633 0.63983438 1.77621015 0.75673198 2.29962231 1.45411015
 3.10148046 0.60258351 1.90752757 0.77962192 1.85051227 0.58146651], Cost: 0.9911
Current parameters: [3.66859018 1.19581898 2.64912555 2.80142123 1.45359209 0.69007078
 2.96999633 0.63983438 1.77621015 0.75673198 2.29962231 1.45411015
 3.10148046 0.60258351 1.90752757 0.77962192 1.85051227 0.58146651], Cost: 2.3378
Current parameters: [3.66859018 0.19581898 3.64912555 2.80142123 1.45359209 0.69007078
 2.96999633 0.63983438 1.77621015 0.75673198 2.29962231 1.45411015
 3.10148046 0.60258351 1.90752757 0.77962192 1.85051227 0.58146651], Cost: 0.6833
Current parameters: [3.66859018 0.19581898 3.64912555 3.80142123 1.45359209 0.69007078
```



```
 2.96999633 0.63983438 1.77621015 0.75673198 2.29962231 1.45411015
 3.10148046 0.60258351 1.90752757 0.77962192 1.85051227 0.58146651], Cost: 1.8593
Current parameters: [3.66859018 0.19581898 3.64912555 2.80142123 2.45359209 0.69007078
 2.96999633 0.63983438 1.77621015 0.75673198 2.29962231 1.45411015
 3.10148046 0.60258351 1.90752757 0.77962192 1.85051227 0.58146651], Cost: 2.0926
Current parameters: [3.66859018 0.19581898 3.64912555 2.80142123 1.45359209 1.69007078
 2.96999633 0.63983438 1.77621015 0.75673198 2.29962231 1.45411015
 3.10148046 0.60258351 1.90752757 0.77962192 1.85051227 0.58146651], Cost: 2.3545
Current parameters: [3.66859018 0.19581898 3.64912555 2.80142123 1.45359209 0.69007078
 3.96999633 0.63983438 1.77621015 0.75673198 2.29962231 1.45411015
 3.10148046 0.60258351 1.90752757 0.77962192 1.85051227 0.58146651], Cost: 2.8894
Current parameters: [3.66859018 0.19581898 3.64912555 2.80142123 1.45359209 0.69007078
 2.96999633 1.63983438 1.77621015 0.75673198 2.29962231 1.45411015
 3.10148046 0.60258351 1.90752757 0.77962192 1.85051227 0.58146651], Cost: 2.9737
Current parameters: [3.66859018 0.19581898 3.64912555 2.80142123 1.45359209 0.69007078
 2.96999633 0.63983438 2.77621015 0.75673198 2.29962231 1.45411015
 3.10148046 0.60258351 1.90752757 0.77962192 1.85051227 0.58146651], Cost: 2.7320
Current parameters: [3.66859018 0.19581898 3.64912555 2.80142123 1.45359209 0.69007078
 2.96999633 0.63983438 1.77621015 1.75673198 2.29962231 1.45411015
 3.10148046 0.60258351 1.90752757 0.77962192 1.85051227 0.58146651], Cost: 1.0317
Current parameters: [3.66859018 0.19581898 3.64912555 2.80142123 1.45359209 0.69007078
 2.96999633 0.63983438 1.77621015 0.75673198 3.29962231 1.45411015
 3.10148046 0.60258351 1.90752757 0.77962192 1.85051227 0.58146651], Cost: 0.9042
Current parameters: [3.66859018 0.19581898 3.64912555 2.80142123 1.45359209 0.69007078
 2.96999633 0.63983438 1.77621015 0.75673198 2.29962231 2.45411015
 3.10148046 0.60258351 1.90752757 0.77962192 1.85051227 0.58146651], Cost: 1.2000
Current parameters: [3.66859018 0.19581898 3.64912555 2.80142123 1.45359209 0.69007078
 2.96999633 0.63983438 1.77621015 0.75673198 2.29962231 1.45411015
 4.10148046 0.60258351 1.90752757 0.77962192 1.85051227 0.58146651], Cost: 0.8949
Current parameters: [3.66859018 0.19581898 3.64912555 2.80142123 1.45359209 0.69007078
 2.96999633 0.63983438 1.77621015 0.75673198 2.29962231 1.45411015
 3.10148046 1.60258351 1.90752757 0.77962192 1.85051227 0.58146651], Cost: 2.8271
Current parameters: [3.66859018 0.19581898 3.64912555 2.80142123 1.45359209 0.69007078
 2.96999633 0.63983438 1.77621015 0.75673198 2.29962231 1.45411015
 3.10148046 0.60258351 2.90752757 0.77962192 1.85051227 0.58146651], Cost: 3.1115
Current parameters: [3.66859018 0.19581898 3.64912555 2.80142123 1.45359209 0.69007078
 2.96999633 0.63983438 1.77621015 0.75673198 2.29962231 1.45411015
 3.10148046 0.60258351 1.90752757 1.77962192 1.85051227 0.58146651], Cost: 3.2992
Current parameters: [3.66859018 0.19581898 3.64912555 2.80142123 1.45359209 0.69007078
 2.96999633 0.63983438 1.77621015 0.75673198 2.29962231 1.45411015
 3.10148046 0.60258351 1.90752757 0.77962192 2.85051227 0.58146651], Cost: 2.7135
```



```
Current parameters: [3.66859018 0.19581898 3.64912555 2.80142123 1.45359209 0.69007078
 2.96999633 0.63983438 1.77621015 0.75673198 2.29962231 1.45411015
 3.10148046 0.60258351 1.90752757 0.77962192 1.85051227 1.58146651], Cost: 1.0717
Current parameters: [ 3.75065041 -0.00545358  3.69512666  2.62567002  1.24296925  0.44030996
  2.64028891  0.2975296   1.47003172  0.70466736  2.2666111   1.37688338
  3.06985549  0.28218529  1.54463549  0.38867164  1.54709621  0.52341497], Cost: 2.6640
Current parameters: [3.70962029 0.0951827  3.67212611 2.71354563 1.34828067 0.56519037
 2.80514262 0.46868199 1.62312094 0.73069967 2.28311671 1.41549677
 3.08566798 0.4423844  1.72608153 0.58414678 1.69880424 0.55244074], Cost: 3.4893
Current parameters: [3.91118915 0.19581898 3.64912555 2.80142123 1.45359209 0.69007078
 3.03037631 0.63983438 1.77621015 0.75673198 2.29962231 1.45411015
 3.10148046 0.60258351 1.90752757 0.77962192 1.85051227 0.58146651], Cost: 0.7914
Current parameters: [3.54378812 0.17631149 3.65358401 2.7843873  1.43317836 0.66586377
 3.44551208 0.60665795 1.74653511 0.75168584 2.29642284 1.44662527
 3.09841535 0.57153028 1.8723558  0.74173072 1.82110496 0.57584012], Cost: 1.5525
Current parameters: [3.66619599 0.44562236 3.64912555 2.80142123 1.45359209 0.69007078
 2.97961586 0.63983438 1.77621015 0.75673198 2.29962231 1.45411015
 3.10148046 0.60258351 1.90752757 0.77962192 1.85051227 0.58146651], Cost: 0.6443
Current parameters: [3.68641993 0.46546837 3.67173755 2.71503013 1.35005972 0.56730001
 2.7668856  0.47157332 1.62570712 0.73113944 2.28339554 1.41614907
 3.0859351  0.44509069 1.72914675 0.58744899 1.70136708 0.55293108], Cost: 3.0662
Current parameters: [3.66476016 0.44671002 3.65093687 2.79450093 1.44529869 0.93758269
 2.97485329 0.62635593 1.7641542  0.75468191 2.29832247 1.4510693
 3.10023521 0.58996765 1.89323849 0.76422802 1.83856508 0.5791807 ], Cost: 1.1195
Current parameters: [3.66619599 0.44562236 3.77005196 2.83307251 1.45359209 0.69007078
 2.97961586 0.63983438 1.77621015 0.75673198 2.29962231 1.45411015
 3.10148046 0.60258351 1.90752757 0.77962192 1.85051227 0.58146651], Cost: 0.6989
Current parameters: [3.6651509  0.44641403 3.5843448  3.0415343  1.4475556  0.68554559
 2.97614935 0.63002386 1.76743502 0.7552398  2.2986762  1.45189682
 3.10057409 0.59340084 1.89712702 0.76841721 1.84181631 0.57980275], Cost: 0.7371
Current parameters: [3.66619599 0.44562236 3.64912555 2.80142123 1.56005302 0.69007078
 2.97961586 0.57432812 1.77621015 0.75673198 2.29962231 1.45411015
 3.10148046 0.60258351 1.90752757 0.77962192 1.85051227 0.58146651], Cost: 0.5208
Current parameters: [3.66366193 0.44754193 3.64414578 2.80253461 1.69644751 0.67909849
 2.97121055 0.77640229 1.75493296 0.75311386 2.29732827 1.44874345
 3.09928276 0.58031815 1.88230919 0.75245369 1.82942704 0.57743235], Cost: 1.3334
Current parameters: [3.66619599 0.44562236 3.64912555 2.80142123 1.56005302 0.69007078
 2.97961586 0.57432812 1.79716517 0.63350095 2.29962231 1.45411015
 3.10148046 0.60258351 1.90752757 0.77962192 1.85051227 0.58146651], Cost: 0.2788
Current parameters: [3.6642279  0.4471132  3.645258   2.80228594 1.55964262 0.68154912
 2.97308785 0.55844301 2.04214365 0.65932144 2.29784064 1.44994208
```



```
 3.09977361 0.58529105 1.88794164 0.75852163 1.83413636 0.57833337], Cost: 0.8829
Current parameters: [3.66619599 0.44562236 3.64912555 2.80142123 1.56005302 0.69007078
 2.97961586 0.57432812 1.79716517 0.63350095 2.1846829  1.50324193
 3.10148046 0.60258351 1.90752757 0.77962192 1.85051227 0.58146651], Cost: 0.4534
Current parameters: [3.66567555 0.4460166  3.64810282 2.8016499  1.5599445  0.68781732
 2.9778896  0.57012749 1.79506346 0.62895557 2.40050005 1.68252393
 3.10102911 0.59801072 1.9023483  0.77404218 1.84618185 0.58063799], Cost: 0.8040
Current parameters: [3.66619599 0.44562236 3.64912555 2.80142123 1.56005302 0.69007078
 2.97961586 0.57432812 1.79716517 0.63350095 2.29962231 1.45411015
 3.22587596 0.59030503 1.90752757 0.77962192 1.85051227 0.58146651], Cost: 0.4083
Current parameters: [3.66412529 0.44719094 3.64505634 2.80233103 1.55962122 0.68110479
 2.97274747 0.55761474 1.78880292 0.61541593 2.30441055 1.43514999
 3.11678585 0.84718495 1.8869204  0.75742143 1.8332825  0.57817   ], Cost: 0.2165
Current parameters: [3.66412529 0.44719094 3.64505634 2.80233103 1.55962122 0.68110479
 2.97274747 0.55761474 1.78880292 0.61541593 2.30441055 1.43514999
 3.11678585 0.84718495 1.79530566 0.84246106 1.8332825  0.57817   ], Cost: 0.2292
Current parameters: [3.65977949 0.4504829  3.6365163  2.8042404  1.55871502 0.66228789
 2.9583328  0.52253839 1.77125308 0.57746093 2.31445961 1.39535835
 3.10036924 0.86878247 2.04790481 0.92818554 1.79712242 0.57125162], Cost: 1.6529
Current parameters: [3.66412529 0.44719094 3.64505634 2.80233103 1.55962122 0.68110479
 2.97274747 0.55761474 1.78880292 0.61541593 2.30441055 1.43514999
 3.11678585 0.84718495 1.8869204  0.75742143 1.85677225 0.45539691], Cost: 0.1467
Current parameters: [3.66187802 0.44889326 3.64064017 2.80331839 1.55915261 0.67137431
 2.96529345 0.53947627 1.77972766 0.59578887 2.30960705 1.41457319
 3.10829659 0.85835333 1.8662661  0.73379087 2.09805541 0.49632064], Cost: 0.9988
Current parameters: [3.63420204 0.44227437 3.64426351 2.80536009 1.5632513  0.6854094
 3.09297521 0.56351434 1.79407989 0.61631326 2.30497949 1.43648099
 3.11733091 0.852707   1.89317483 0.76415944 1.86200161 0.45639743], Cost: 0.2053
Current parameters: [3.65167332 0.44891377 3.64554914 2.77154883 1.51972614 0.61710479
 2.99279003 0.46116357 1.71553698 0.57005756 2.30774531 1.38339952
 3.0954792  0.80811217 1.78207599 0.64196126 1.76282146 0.42788829], Cost: 1.2984
Current parameters: [3.66440508 0.44723691 3.64509928 2.80216698 1.55942463 0.78686364
 2.97162331 0.55729523 1.77653474 0.57959025 2.32812488 1.406003
 3.08161547 0.82948875 1.88308075 0.75328491 1.8535619  0.45478269], Cost: 0.1299
Current parameters: [3.66204341 0.44949193 3.64055339 2.79926761 1.55384237 0.91501332
 2.96008155 0.52605215 1.80164242 0.68448958 2.24691453 1.48452225
 3.19887637 0.89994099 1.86097638 0.72771162 1.83066923 0.44371692], Cost: 0.4011
Current parameters: [3.67849316 0.44955167 3.64454246 2.80429436 1.5981993  0.74130923
 2.91501922 0.62031193 1.78381141 0.5966889  2.31713927 1.42008597
 3.0983053  0.83938729 1.88667466 0.75715669 1.84026746 0.39632742], Cost: 0.2420
Current parameters: [3.63851529 0.44624586 3.6391251  2.79997229 1.66685689 0.86487793
```

```
 3.04968327 0.70452509 1.74850246 0.5441131  2.33648136 1.36840841
 3.06458716 0.8323823  1.83497272 0.69928476 1.8357117  0.53854027], Cost: 0.4064
Current parameters: [3.66294198 0.44772557 3.64346891 2.80280542 1.56039731 0.78990215
 2.97170075 0.55276045 1.89590235 0.57898618 2.33991157 1.38559354
 3.06322419 0.82326863 1.87129657 0.74010416 1.84388494 0.45586409], Cost: 0.1737
Current parameters: [3.70418871 0.44721347 3.65695033 2.75688903 1.56056069 0.78771527
 2.98213355 0.55914155 1.7781862  0.57987108 2.32830293 1.40641955
 3.08178604 0.83121692 1.88503812 0.75539361 1.85519846 0.4550958 ], Cost: 0.1194
Current parameters: [3.79553707 0.44739478 3.63576509 2.83582093 1.5600066  0.78859664
 3.00487758 0.55604434 1.77641443 0.57867905 2.32777322 1.40454205
 3.08197013 0.83136225 1.88032248 0.750139   1.85131886 0.45540701], Cost: 0.2420
Current parameters: [3.70479291 0.38514503 3.65695252 2.75688065 1.56059502 0.78775589
 2.97977478 0.55919735 1.77806044 0.58091536 2.32472748 1.40072799
 3.08289448 0.83175121 1.88513148 0.75549419 1.85527652 0.45511074], Cost: 0.1217
Current parameters: [3.70256785 0.43348822 3.65601439 2.75546671 1.56352178 0.79289007
 2.98330523 0.55848639 1.78330206 0.55901176 2.39322905 1.50777968
 3.06269513 0.82935444 1.88015265 0.74969642 1.85127092 0.45696644], Cost: 0.1208
Current parameters: [3.70418871 0.44721347 3.71225856 2.7713654  1.58206784 0.78771527
 2.98213355 0.54590803 1.7781862  0.57987108 2.32830293 1.40641955
 3.08178604 0.83121692 1.88503812 0.75539361 1.85519846 0.4550958 ], Cost: 0.1871
Current parameters: [3.7029178  0.44698378 3.70367053 2.76804923 1.46356187 0.79242909
 2.98392627 0.62083793 1.78052188 0.57779398 2.33021439 1.40786669
 3.08202402 0.83721044 1.88243307 0.75223954 1.85314629 0.45680332], Cost: 0.0428
Current parameters: [3.7028418  0.44704134 3.70352119 2.76808262 1.46354603 0.79210004
 2.9836742  0.62022455 1.77543174 0.60525927 2.37787194 1.38687436
 3.10101375 0.84436838 1.88167679 0.75142478 1.85251396 0.45668234], Cost: 0.0329
Current parameters: [3.70206607 0.44659071 3.70347457 2.76682465 1.46162784 0.79763707
 2.9863326  0.62586992 1.76402263 0.69325177 2.36733585 1.39605209
 3.01854321 0.81842269 1.88270126 0.7522258  1.85348017 0.45869583], Cost: 0.0080
Current parameters: [3.70264345 0.44615334 3.7046092  2.76657097 1.46174824 0.80013707
 2.98824773 0.63053014 1.7663543  0.69829445 2.36600073 1.40133879
 3.04277984 0.76184765 1.88254605 0.75205859 1.85828438 0.459615  ], Cost: 0.0642
Current parameters: [3.69137436 0.44663052 3.69393508 2.7591119  1.44576449 0.82039506
 2.99147694 0.63199547 1.76737442 0.68405987 2.38052926 1.38854685
 3.003502   0.82426283 1.96110299 0.83434005 1.82212025 0.46689014], Cost: 0.2482
Current parameters: [3.70018317 0.44640554 3.70433154 2.76259814 1.45646369 0.79232419
 2.9929096  0.61643532 1.7558585  0.69034607 2.36714731 1.39256192
 3.01681186 0.81105597 1.87252761 0.74126555 1.79653346 0.44943453], Cost: 0.0665
Current parameters: [3.6876926  0.44812949 3.68898569 2.73435508 1.40971678 0.770827
 2.9682164  0.59833269 1.74177044 0.6801973  2.38080382 1.38073825
 2.98808058 0.83221904 1.84620861 0.70843322 1.91725462 0.44706285], Cost: 0.1535
```



```
Current parameters: [3.69841264 0.44606495 3.70327618 2.76701122 1.48113311 0.78702424
 3.00041868 0.65694484 1.75506743 0.66914178 2.38287097 1.37619667
 2.99530347 0.80663624 1.87866402 0.74787641 1.85432414 0.47364227], Cost: 0.0055
Current parameters: [3.68004254 0.44660139 3.69220766 2.75248719 1.50050505 0.86187825
 3.0230656  0.70430402 1.7519816  0.69359883 2.36311535 1.3901455
 3.01848951 0.84418049 1.84809369 0.71265719 1.85775985 0.50352856], Cost: 0.2034
Current parameters: [3.6992082  0.44619566 3.70324473 2.76713135 1.48332272 0.78445177
 2.99722223 0.66050341 1.75547835 0.67010735 2.38225061 1.37699194
 2.99624595 0.80719521 1.92438125 0.70584729 1.8535734  0.47034129], Cost: 0.0049
Current parameters: [3.70969387 0.44864138 3.69879408 2.76233839 1.51154743 0.73863838
 2.93675861 0.72544174 1.7618456  0.68668574 2.37526082 1.39062562
 3.00527871 0.82269486 1.91550182 0.71663562 1.85054297 0.41016167], Cost: 0.0964
Current parameters: [3.71045071 0.44804288 3.70354433 2.76598673 1.48195099 0.78795499
 2.95205124 0.65827407 1.75290436 0.66803671 2.38318485 1.3750818
 2.99433922 0.80426654 1.92184839 0.70311859 1.84377777 0.51006582], Cost: 0.0040
Current parameters: [3.69832842 0.4464841  3.70091633 2.76297092 1.47707584 0.70192318
 2.98977538 0.66177226 1.76421223 0.69758888 2.36619087 1.39930585
 3.01896035 0.82403462 1.92580417 0.70711457 1.84255401 0.5708288 ], Cost: 0.1057
Current parameters: [3.71031188 0.44832722 3.70269356 2.76688184 1.48139456 0.79097269
 2.95016503 0.65479324 1.81293281 0.67250557 2.38726066 1.36775141
 2.98368696 0.80798023 1.91670611 0.69745622 1.84363164 0.51018244], Cost: 0.0140
Current parameters: [3.69410549 0.44790479 3.69428294 2.74157725 1.46950276 0.76762772
 2.96629073 0.67891887 1.75103282 0.60640541 2.41644536 1.33689292
 2.99342984 0.73137583 1.90670074 0.68415534 1.80901232 0.51103756], Cost: 0.3522
Current parameters: [3.74532113 0.4478266  3.69563164 2.79735821 1.48462211 0.7788891
 2.96366796 0.66386265 1.74970912 0.6595712  2.39087646 1.36683852
 2.97765875 0.80449509 1.90049405 0.72251404 1.85030391 0.51515618], Cost: 0.0025
Current parameters: [3.80521469 0.44893394 3.67302864 2.83952703 1.45812865 0.80311097
 2.96451412 0.64778709 1.74834698 0.68046508 2.37589167 1.38618685
 3.0077463  0.81705631 1.94440197 0.65999996 1.84708637 0.49924819], Cost: 0.0315
Current parameters: [3.73625358 0.44896271 3.72813648 2.78979987 1.49268594 0.79441645
 2.9314331  0.65365968 1.71988475 0.66254346 2.38560053 1.3731038
 2.98436561 0.80741356 1.91153991 0.71603303 1.84935835 0.52386883], Cost: 0.0047
Current parameters: [3.76422364 0.44715281 3.78614758 2.84354732 1.50979118 0.76121264
 3.00010518 0.64381019 1.78276951 0.66016982 2.3985153  1.35908058
 2.96232996 0.81357674 1.88945902 0.71922215 1.86844743 0.49860838], Cost: 0.0307
Current parameters: [3.7423794  0.44833707 3.68876484 2.78812178 1.46146179 0.76909053
 2.95315218 0.64157694 1.74264174 0.66168142 2.39385789 1.36353905
 2.95727616 0.82153697 1.88665364 0.71355292 1.88765101 0.50629571], Cost: 0.0602
Current parameters: [3.74578981 0.45127551 3.69588376 2.79760818 1.48389832 0.77762991
 2.96344127 0.6640894  1.74846625 0.66462178 2.37370002 1.34195975
```



```
 2.98171183 0.80459938 1.90176505 0.72392397 1.85121336 0.51470425], Cost: 0.0085
Current parameters: [3.74582981 0.4473134  3.69656898 2.79772594 1.48479494 0.78079163
 2.96631838 0.66714877 1.75163445 0.65200141 2.34767007 1.38957979
 2.94367492 0.78826223 1.90323328 0.72068922 1.84730769 0.51539593], Cost: 0.0345
Current parameters: [3.74089214 0.47818583 3.69439925 2.80206665 1.48448728 0.77878074
 2.96372959 0.66364353 1.74959904 0.65903128 2.39260666 1.3695789
 2.97709891 0.80405422 1.90024502 0.72224576 1.8500957  0.51511634], Cost: 0.0068
Current parameters: [3.70719988 0.43480688 3.68475502 2.84246315 1.4831871  0.77825342
 2.95321017 0.66238292 1.74827075 0.65973954 2.39022954 1.36533963
 2.97768746 0.80345341 1.89899628 0.71991003 1.84908324 0.51454814], Cost: 0.0049
Current parameters: [3.74606021 0.44798488 3.69224216 2.79729689 1.49179932 0.77740245
 2.96171767 0.65681778 1.75368156 0.63675448 2.38262119 1.36976777
 2.98981775 0.81298646 1.8993561  0.7244602  1.85463198 0.51447045], Cost: 0.0118
Current parameters: [3.74787621 0.44719583 3.67508255 2.79476875 1.53153683 0.77805146
 2.96516528 0.63609749 1.74651628 0.67708509 2.39648602 1.36440354
 2.9691361  0.79753886 1.90423307 0.7207092  1.84989949 0.51349445], Cost: 0.0060
Current parameters: [3.74717751 0.44781551 3.69597417 2.79877439 1.47787387 0.77105276
 2.9589574  0.65508158 1.75186924 0.65646333 2.39467604 1.36533062
 2.97349868 0.80144815 1.92137059 0.73806788 1.8476693  0.51042003], Cost: 0.0348
Current parameters: [3.74273843 0.44595194 3.69199741 2.80042498 1.49468365 0.81759083
 2.97779925 0.68284035 1.75209534 0.67103782 2.39280481 1.36929944
 2.97541296 0.83430436 1.9044819  0.74309332 1.85769396 0.52857024], Cost: 0.0661
Current parameters: [3.74504611 0.4480697  3.69507999 2.79609128 1.48791343 0.77177796
 2.96157811 0.66859288 1.74624212 0.65539382 2.3926116  1.36270437
 2.9662451  0.82332001 1.90237861 0.71504999 1.83411878 0.50959462], Cost: 0.0100
Current parameters: [3.75367857 0.44806777 3.69500631 2.79939502 1.50025956 0.76321895
 2.93594254 0.69459952 1.75590978 0.67494697 2.38683424 1.37775982
 2.99055949 0.80286208 1.89249503 0.72984961 1.86099255 0.48657903], Cost: 0.0173
Current parameters: [3.74677286 0.44785703 3.69624046 2.79787657 1.47785699 0.78167371
 2.96278144 0.65071824 1.7507746  0.66830831 2.38514743 1.37292606
 2.98307104 0.80938432 1.88652299 0.73357249 1.83652388 0.50892614], Cost: 0.0014
Current parameters: [3.74607637 0.44431933 3.69876632 2.79303021 1.47987841 0.79107217
 2.96637685 0.65615813 1.74337719 0.6552765  2.40313469 1.36160782
 2.99634951 0.77422097 1.86149471 0.71313135 1.81264076 0.50394482], Cost: 0.1305
Current parameters: [3.74636886 0.4414906  3.69717262 2.79378904 1.47512805 0.76697749
 2.97157337 0.65623464 1.74886431 0.67577646 2.38823741 1.38051538
 2.990067   0.80644951 1.88610853 0.72877943 1.8389331  0.52826683], Cost: 0.0149
Current parameters: [3.73359994 0.46920579 3.69625301 2.80643336 1.49003712 0.76065243
 2.95340122 0.63407707 1.75004262 0.67832461 2.35567574 1.37486138
 2.99513101 0.83822404 1.89248415 0.74119169 1.82255663 0.50583782], Cost: 0.0244
Current parameters: [3.74485068 0.44764975 3.69221557 2.79318821 1.46833117 0.77790182
```



```
 2.95780559 0.64065045 1.74677409 0.67151163 2.38746217 1.37168568
 2.97411299 0.81790113 1.87663097 0.72770698 1.85573596 0.50489481], Cost: 0.0121
Current parameters: [3.73995868 0.44775093 3.69865436 2.79305582 1.48086288 0.77910623
 2.96286772 0.65290774 1.75086936 0.66512449 2.38239878 1.37298819
 2.97627361 0.80635515 1.8818358  0.74063637 1.83650685 0.51072809], Cost: 0.0014
Current parameters: [3.75344533 0.44616882 3.69391154 2.80163538 1.47541722 0.7857867
 2.96499718 0.65186616 1.75110936 0.66836555 2.36519294 1.38607232
 2.97038411 0.80195232 1.88981125 0.72450117 1.83666499 0.50860044], Cost: 0.0062
Current parameters: [3.74985977 0.44774763 3.70032195 2.80314789 1.47970165 0.77900965
 2.97013381 0.65091687 1.74132832 0.66689466 2.38526935 1.37322166
 2.9829444  0.80964305 1.88526197 0.73661544 1.83886664 0.50470673], Cost: 0.0034
Current parameters: [3.74933731 0.44690163 3.71726183 2.80529029 1.4830437  0.77979444
 2.96636617 0.64564438 1.76871618 0.67044813 2.38757099 1.3711724
 2.97863688 0.810808   1.88365435 0.72865946 1.84022477 0.50899045], Cost: 0.0033
Current parameters: [3.75361317 0.44795932 3.69201292 2.80165596 1.48525953 0.78079257
 2.95840027 0.64694918 1.74949919 0.66945879 2.38720253 1.37112853
 2.97921803 0.80786052 1.8837216  0.73553086 1.83611969 0.51499171], Cost: 0.0025
Current parameters: [3.75452576 0.45135374 3.70256333 2.8067987  1.46053    0.78023888
 2.95260375 0.66059431 1.75054348 0.65896965 2.38220048 1.37064043
 2.98310107 0.81494958 1.88058973 0.74003153 1.8337964  0.51697175], Cost: 0.0036
Current parameters: [3.75125345 0.45186166 3.69834196 2.79044425 1.47539827 0.78436624
 2.96850622 0.64431091 1.74958173 0.66493104 2.38447808 1.37039423
 2.98129707 0.81172809 1.88831504 0.73371343 1.83333509 0.51334465], Cost: 0.0033
Current parameters: [3.76353307 0.45301738 3.69997374 2.78209156 1.48363818 0.77672502
 2.95696283 0.66131055 1.75364786 0.67341737 2.38357943 1.37716693
 2.98718012 0.80998097 1.88493016 0.73755691 1.84063376 0.49919918], Cost: 0.0019
Current parameters: [3.74453584 0.44648028 3.70416497 2.79589704 1.48022887 0.78717616
 2.95582168 0.64985083 1.74356483 0.67007011 2.38506478 1.37535784
 2.98436005 0.80956033 1.88913198 0.7317276  1.83782692 0.51221628], Cost: 0.0025
Current parameters: [3.74595192 0.44641816 3.69164712 2.80027119 1.48211903 0.79946563
 2.97105766 0.66085088 1.7556934  0.67529002 2.385257   1.37509486
 2.98102676 0.82331059 1.88714701 0.74367585 1.84283873 0.51372017], Cost: 0.0165
Current parameters: [3.7457861  0.44926432 3.6961752  2.79736193 1.47918648 0.78566459
 2.96185104 0.65200686 1.75055379 0.66581723 2.38670106 1.36985396
 2.98640532 0.80337298 1.87825112 0.7285274  1.82998218 0.50460568], Cost: 0.0107
Current parameters: [3.74656121 0.44773322 3.69649433 2.79790982 1.47812251 0.78126804
 2.96291032 0.65140437 1.7503255  0.67066124 2.38090647 1.36719831
 2.98422229 0.80883822 1.88689313 0.73324288 1.83767455 0.50960653], Cost: 0.0012
Current parameters: [3.74298322 0.45337341 3.69625821 2.79999718 1.48122345 0.77587482
 2.96004929 0.64709231 1.75074794 0.67192507 2.37545714 1.37026782
 2.98735054 0.81576979 1.88785423 0.73491023 1.83272747 0.50795451], Cost: 0.0033
```

```
Current parameters: [3.74628535 0.44952173 3.69719653 2.79822256 1.47600361 0.78098607
 2.96296196 0.65207139 1.74938527 0.67585706 2.38278474 1.36675641
 2.98093062 0.80819206 1.88734456 0.73370073 1.83479346 0.5088436 ], Cost: 0.0007
Current parameters: [3.74656121 0.44773322 3.69649433 2.79790982 1.47812251 0.78126804
 2.96291032 0.65140437 1.7503255  0.67066124 2.38090647 1.36719831
 2.98422229 0.80883822 1.88689313 0.73324288 1.83767455 0.50960653], Cost: 0.0012

   Return from subroutine COBYLA because the MAXFUN limit has been reached.

   NFVALS =  100   F = 1.227493E-03    MAXCV = 0.000000E+00
   X = 3.746561E+00   4.477332E-01   3.696494E+00   2.797910E+00   1.478123E+00
       7.812680E-01   2.962910E+00   6.514044E-01   1.750325E+00   6.706612E-01
       2.380906E+00   1.367198E+00   2.984222E+00   8.088382E-01   1.886893E+00
       7.332429E-01   1.837675E+00   5.096065E-01
Simulating for QAOA depth p = 10
Current parameters: [3.03651029 2.6518115  0.99508753 2.12317693 1.19387717 0.00316432
 1.62552076 0.31358919 2.46568918 0.43419001 0.86846353 2.30888131
 1.65099024 0.0395159  0.42914159 3.08528018 0.39612451 2.65981663
 1.45679964 1.52218242], Cost: 2.8049
Current parameters: [2.03651029e+00 3.65181150e+00 9.95087531e-01 2.12317693e+00
 1.19387717e+00 3.16431667e-03 1.62552076e+00 3.13589194e-01
 2.46568918e+00 4.34190006e-01 8.68463533e-01 2.30888131e+00
 1.65099024e+00 3.95158979e-02 4.29141585e-01 3.08528018e+00
 3.96124514e-01 2.65981663e+00 1.45679964e+00 1.52218242e+00], Cost: 2.1068
Current parameters: [2.03651029e+00 3.65181150e+00 1.99508753e+00 2.12317693e+00
 1.19387717e+00 3.16431667e-03 1.62552076e+00 3.13589194e-01
 2.46568918e+00 4.34190006e-01 8.68463533e-01 2.30888131e+00
 1.65099024e+00 3.95158979e-02 4.29141585e-01 3.08528018e+00
 3.96124514e-01 2.65981663e+00 1.45679964e+00 1.52218242e+00], Cost: 2.7394
Current parameters: [2.03651029e+00 3.65181150e+00 9.95087531e-01 3.12317693e+00
 1.19387717e+00 3.16431667e-03 1.62552076e+00 3.13589194e-01
 2.46568918e+00 4.34190006e-01 8.68463533e-01 2.30888131e+00
 1.65099024e+00 3.95158979e-02 4.29141585e-01 3.08528018e+00
 3.96124514e-01 2.65981663e+00 1.45679964e+00 1.52218242e+00], Cost: 2.6705
Current parameters: [2.03651029e+00 3.65181150e+00 9.95087531e-01 2.12317693e+00
 2.19387717e+00 3.16431667e-03 1.62552076e+00 3.13589194e-01
 2.46568918e+00 4.34190006e-01 8.68463533e-01 2.30888131e+00
 1.65099024e+00 3.95158979e-02 4.29141585e-01 3.08528018e+00
 3.96124514e-01 2.65981663e+00 1.45679964e+00 1.52218242e+00], Cost: 2.6280
Current parameters: [2.03651029 3.6518115  0.99508753 2.12317693 1.19387717 1.00316432
 1.62552076 0.31358919 2.46568918 0.43419001 0.86846353 2.30888131
```



```
 1.65099024 0.0395159  0.42914159 3.08528018 0.39612451 2.65981663
 1.45679964 1.52218242], Cost: 2.8753
Current parameters: [2.03651029e+00 3.65181150e+00 9.95087531e-01 2.12317693e+00
 1.19387717e+00 3.16431667e-03 2.62552076e+00 3.13589194e-01
 2.46568918e+00 4.34190006e-01 8.68463533e-01 2.30888131e+00
 1.65099024e+00 3.95158979e-02 4.29141585e-01 3.08528018e+00
 3.96124514e-01 2.65981663e+00 1.45679964e+00 1.52218242e+00], Cost: 2.9415
Current parameters: [2.03651029e+00 3.65181150e+00 9.95087531e-01 2.12317693e+00
 1.19387717e+00 3.16431667e-03 1.62552076e+00 1.31358919e+00
 2.46568918e+00 4.34190006e-01 8.68463533e-01 2.30888131e+00
 1.65099024e+00 3.95158979e-02 4.29141585e-01 3.08528018e+00
 3.96124514e-01 2.65981663e+00 1.45679964e+00 1.52218242e+00], Cost: 2.0197
Current parameters: [2.03651029e+00 3.65181150e+00 9.95087531e-01 2.12317693e+00
 1.19387717e+00 3.16431667e-03 1.62552076e+00 1.31358919e+00
 3.46568918e+00 4.34190006e-01 8.68463533e-01 2.30888131e+00
 1.65099024e+00 3.95158979e-02 4.29141585e-01 3.08528018e+00
 3.96124514e-01 2.65981663e+00 1.45679964e+00 1.52218242e+00], Cost: 1.9569
Current parameters: [2.03651029e+00 3.65181150e+00 9.95087531e-01 2.12317693e+00
 1.19387717e+00 3.16431667e-03 1.62552076e+00 1.31358919e+00
 3.46568918e+00 1.43419001e+00 8.68463533e-01 2.30888131e+00
 1.65099024e+00 3.95158979e-02 4.29141585e-01 3.08528018e+00
 3.96124514e-01 2.65981663e+00 1.45679964e+00 1.52218242e+00], Cost: 1.9897
Current parameters: [2.03651029e+00 3.65181150e+00 9.95087531e-01 2.12317693e+00
 1.19387717e+00 3.16431667e-03 1.62552076e+00 1.31358919e+00
 3.46568918e+00 4.34190006e-01 1.86846353e+00 2.30888131e+00
 1.65099024e+00 3.95158979e-02 4.29141585e-01 3.08528018e+00
 3.96124514e-01 2.65981663e+00 1.45679964e+00 1.52218242e+00], Cost: 2.0264
Current parameters: [2.03651029e+00 3.65181150e+00 9.95087531e-01 2.12317693e+00
 1.19387717e+00 3.16431667e-03 1.62552076e+00 1.31358919e+00
 3.46568918e+00 4.34190006e-01 8.68463533e-01 3.30888131e+00
 1.65099024e+00 3.95158979e-02 4.29141585e-01 3.08528018e+00
 3.96124514e-01 2.65981663e+00 1.45679964e+00 1.52218242e+00], Cost: 2.0526
Current parameters: [2.03651029e+00 3.65181150e+00 9.95087531e-01 2.12317693e+00
 1.19387717e+00 3.16431667e-03 1.62552076e+00 1.31358919e+00
 3.46568918e+00 4.34190006e-01 8.68463533e-01 2.30888131e+00
 2.65099024e+00 3.95158979e-02 4.29141585e-01 3.08528018e+00
 3.96124514e-01 2.65981663e+00 1.45679964e+00 1.52218242e+00], Cost: 2.2509
Current parameters: [2.03651029e+00 3.65181150e+00 9.95087531e-01 2.12317693e+00
 1.19387717e+00 3.16431667e-03 1.62552076e+00 1.31358919e+00
 3.46568918e+00 4.34190006e-01 8.68463533e-01 2.30888131e+00
 1.65099024e+00 1.03951590e+00 4.29141585e-01 3.08528018e+00
```



```
 3.96124514e-01 2.65981663e+00 1.45679964e+00 1.52218242e+00], Cost: 2.3208
Current parameters: [2.03651029e+00 3.65181150e+00 9.95087531e-01 2.12317693e+00
 1.19387717e+00 3.16431667e-03 1.62552076e+00 1.31358919e+00
 3.46568918e+00 4.34190006e-01 8.68463533e-01 2.30888131e+00
 1.65099024e+00 3.95158979e-02 1.42914159e+00 3.08528018e+00
 3.96124514e-01 2.65981663e+00 1.45679964e+00 1.52218242e+00], Cost: 2.8131
Current parameters: [2.03651029e+00 3.65181150e+00 9.95087531e-01 2.12317693e+00
 1.19387717e+00 3.16431667e-03 1.62552076e+00 1.31358919e+00
 3.46568918e+00 4.34190006e-01 8.68463533e-01 2.30888131e+00
 1.65099024e+00 3.95158979e-02 4.29141585e-01 4.08528018e+00
 3.96124514e-01 2.65981663e+00 1.45679964e+00 1.52218242e+00], Cost: 2.8228
Current parameters: [2.03651029e+00 3.65181150e+00 9.95087531e-01 2.12317693e+00
 1.19387717e+00 3.16431667e-03 1.62552076e+00 1.31358919e+00
 3.46568918e+00 4.34190006e-01 8.68463533e-01 2.30888131e+00
 1.65099024e+00 3.95158979e-02 4.29141585e-01 3.08528018e+00
 1.39612451e+00 2.65981663e+00 1.45679964e+00 1.52218242e+00], Cost: 0.9514
Current parameters: [2.03651029e+00 3.65181150e+00 9.95087531e-01 2.12317693e+00
 1.19387717e+00 3.16431667e-03 1.62552076e+00 1.31358919e+00
 3.46568918e+00 4.34190006e-01 8.68463533e-01 2.30888131e+00
 1.65099024e+00 3.95158979e-02 4.29141585e-01 3.08528018e+00
 1.39612451e+00 3.65981663e+00 1.45679964e+00 1.52218242e+00], Cost: 1.6839
Current parameters: [2.03651029e+00 3.65181150e+00 9.95087531e-01 2.12317693e+00
 1.19387717e+00 3.16431667e-03 1.62552076e+00 1.31358919e+00
 3.46568918e+00 4.34190006e-01 8.68463533e-01 2.30888131e+00
 1.65099024e+00 3.95158979e-02 4.29141585e-01 3.08528018e+00
 1.39612451e+00 2.65981663e+00 2.45679964e+00 1.52218242e+00], Cost: 1.6518
Current parameters: [2.03651029e+00 3.65181150e+00 9.95087531e-01 2.12317693e+00
 1.19387717e+00 3.16431667e-03 1.62552076e+00 1.31358919e+00
 3.46568918e+00 4.34190006e-01 8.68463533e-01 2.30888131e+00
 1.65099024e+00 3.95158979e-02 4.29141585e-01 3.08528018e+00
 1.39612451e+00 2.65981663e+00 1.45679964e+00 2.52218242e+00], Cost: 1.4260
Current parameters: [ 1.85431409  3.74259812  0.7477081   1.90272709  0.99004823 -0.2973782
  1.29909161  1.34762865  3.49024956  0.42135983  0.84126554  2.27144432
  1.53602315 -0.1027844   0.09435224  2.74668982  1.78931195  2.37338904
  1.18293106  1.33661155], Cost: 3.0779
Current parameters: [1.60894242 3.6518115  0.99508753 2.12317693 1.19387717 0.26236639
 1.62552076 1.31358919 3.46568918 0.43419001 0.86846353 2.30888131
 1.65099024 0.0395159  0.42914159 3.08528018 1.39612451 2.65981663
 1.45679964 1.52218242], Cost: 1.1249
Current parameters: [2.56879904 3.67014838 0.94512239 2.07865096 1.15270825 0.82833868
 1.55958933 1.32046441 3.47064983 0.4315986  0.86297014 2.30131987
```



```
 1.62776944 0.0107744  0.36152158 3.01689246 1.47553963 2.60196462
 1.40148428 1.48470123], Cost: 0.6943
Current parameters: [ 2.59487035  3.67975313  0.91895092  2.05532851  1.1311442   0.84365444
  2.04996502  1.32406561  3.47324819  0.43024123  0.86009274  2.29735923
  1.61560652 -0.00428023  0.3261026   2.98107135  1.51713683  2.57166206
  1.3725104   1.46506879], Cost: 1.3381
Current parameters: [2.56879904 3.67014838 1.11144798 1.89200746 1.15270825 0.82833868
 1.55958933 1.32046441 3.47064983 0.4315986  0.86297014 2.30131987
 1.62776944 0.0107744  0.36152158 3.01689246 1.47553963 2.60196462
 1.40148428 1.48470123], Cost: 0.6819
Current parameters: [ 2.59518737  3.67986993  1.47866042  2.21646675  1.13088199  0.84384068
  1.52250514  1.3241094   3.47327978  0.43022472  0.86005775  2.29731107
  1.61545862 -0.0044633   0.3256719   2.98063576  1.51764265  2.57129359
  1.37215808  1.46483006], Cost: 2.0078
Current parameters: [2.56879904 3.8985197  1.11144798 1.89200746 1.25442619 0.82833868
 1.55958933 1.32046441 3.47064983 0.4315986  0.86297014 2.30131987
 1.62776944 0.0107744  0.36152158 3.01689246 1.47553963 2.60196462
 1.40148428 1.48470123], Cost: 0.6681
Current parameters: [2.58432296e+00 3.69882574e+00 1.07212923e+00 1.85533431e+00
 1.70610010e+00 8.37458311e-01 1.53777317e+00 1.32260871e+00
 3.47219700e+00 4.30790365e-01 8.61256818e-01 2.29896154e+00
 1.62052714e+00 1.81025785e-03 3.40431673e-01 2.99556311e+00
 1.50030830e+00 2.58392124e+00 1.38423205e+00 1.47301127e+00], Cost: 1.6344
Current parameters: [2.56879904 3.8985197  1.11144798 1.89200746 1.25442619 0.82833868
 1.55958933 1.56921055 3.47064983 0.4315986  0.86297014 2.30131987
 1.62776944 0.0107744  0.36152158 3.04189962 1.47553963 2.60196462
 1.40148428 1.48470123], Cost: 1.1202
Current parameters: [ 2.59121194e+00  3.92687927e+00  1.05468093e+00  1.83906004e+00
  1.19556611e+00  8.41505287e-01  1.52809192e+00  1.20852766e+00
  3.47288358e+00  4.30431700e-01  8.60496503e-01  2.29791500e+00
  1.61731326e+00 -2.16771856e-03  3.31072704e-01  3.48744211e+00
  1.51129978e+00  2.57591422e+00  1.37657610e+00  1.46782368e+00], Cost: 1.9034
Current parameters: [ 2.56879904  3.8985197   1.11144798  1.89200746  1.25442619  0.82833868
  1.55958933  1.32046441  3.47064983  0.4315986   0.86297014  2.30131987
  1.62776944 -0.21930466  0.45931537  3.01689246  1.47553963  2.60196462
  1.40148428  1.48470123], Cost: 1.2334
Current parameters: [2.58958691 3.92482308 1.05879678 1.84289895 1.19983371 0.84055065
 1.53037562 1.27007843 3.47272162 0.4305163  0.86067585 2.29816187
 1.61807138 0.26288637 0.76400363 2.92180864 1.50870702 2.57780298
 1.37838205 1.46904737], Cost: 0.8217
Current parameters: [2.56879904 3.8985197  1.11144798 1.89200746 1.25442619 0.82833868
```

```
 1.55958933 1.32046441 3.47064983 0.4315986  0.86297014 2.06360558
 1.70517689 0.0107744  0.36152158 3.01689246 1.47553963 2.60196462
 1.40148428 1.48470123], Cost: 1.4821
Current parameters: [2.57555026 3.90706218 1.09434858 1.87605861 1.23669633 0.83230473
 1.55010167 1.30410068 3.47132268 0.4312471  0.86222503 2.48788251
 2.08805131 0.02780826 0.33967665 2.98601233 1.48631132 2.59411771
 1.39398143 1.47961736], Cost: 1.4332
Current parameters: [2.56774857 3.89904314 1.11002169 1.89073644 1.253251   0.82660588
 1.55770728 1.32066066 3.47064983 0.4315986  0.61301352 2.30110403
 1.62710659 0.00995396 0.35959133 3.01494029 1.47553963 2.60196462
 1.40148428 1.48470123], Cost: 1.4476
Current parameters: [2.5557147  3.96126164 0.96965599 1.76229413 1.12040478 0.77289682
 1.43423128 1.25159614 3.473827   0.42993886 1.02772208 2.41611956
 1.44842188 0.05608985 0.17574996 2.78751906 1.526403   2.56491189
 1.3660562  1.46069548], Cost: 1.1413
Current parameters: [2.56351985 3.89860267 1.11122189 1.89180598 1.25423991 0.83673305
 1.55929099 1.32049552 3.7202948  0.4315986  0.86294529 2.30128566
 1.62766437 0.01064435 0.36121561 3.01658301 1.47110658 2.60639767
 1.40591733 1.48913428], Cost: 0.8800
Current parameters: [2.40542914 3.90432976 1.1050036  1.88518438 1.24358397 1.17690908
 1.57095511 1.2929102  3.43090014 0.43106448 0.91218483 2.34419332
 1.58826507 0.04789784 0.35477011 2.99671171 1.32888169 2.75306692
 1.5531094  1.64000214], Cost: 0.7447
Current parameters: [2.57047366 3.89849338 1.11151969 1.89207137 1.25448529 0.82567588
 1.55968397 1.32045454 3.47064271 0.18190373 0.86297803 2.30133073
 1.62780277 0.01081566 0.36161864 3.01699062 1.46385184 2.60055841
 1.40007806 1.48329501], Cost: 0.7055
Current parameters: [2.65610454 3.89900956 1.11550407 1.89500165 1.25459107 0.74051086
 1.57635228 1.30357306 3.46269885 0.45749123 0.89208627 2.32711538
 1.60868764 0.03655593 0.3681422  3.01592002 1.00831391 2.53664468
 1.33646458 1.42179249], Cost: 2.4020
Current parameters: [2.56683856 3.89855051 1.11136402 1.89193264 1.25435702 0.83145601
 1.55947854 1.32047596 3.47065816 0.43159424 0.86296091 2.30130717
 1.62773042 0.01072611 0.36140796 3.01677754 1.47567308 2.77351533
 1.21967668 1.48634748], Cost: 0.9513
Current parameters: [2.70374483 3.8982314  1.11753933 1.89682514 1.25631818 0.66395367
 1.57885777 1.30355084 3.46261594 0.43158027 0.89185745 2.32702587
 1.60996449 0.03735652 0.37087875 3.01880869 1.50074088 2.91450712
 1.70550453 1.38162368], Cost: 2.4964
Current parameters: [2.75437018 3.90068518 1.10554738 1.88674919 1.24956438 0.85139677
 1.5518032  1.32127633 3.47123565 0.43129257 0.86232141 2.30042691
```



```
 1.6250272  0.0073802  0.35353604 3.00881626 1.48491811 2.59513263
 1.39495185 1.64917799], Cost: 0.8329
Current parameters: [2.31993198 3.89237424 1.13205426 1.90992626 1.26912937 0.54755447
 1.59495251 1.30593139 3.46319804 0.4324241  0.8857688  2.32247301
 1.62232991 0.03942109 0.39021921 3.04042405 1.46817104 2.60449225
 1.41498304 1.80605795], Cost: 0.4936
Current parameters: [2.32096056 3.91792682 1.13726778 1.91457224 1.27231144 0.5603443
 1.82555308 1.28233575 3.44053472 0.45575878 0.90879954 2.34130419
 1.60059158 0.0564715  0.36735266 3.02883431 1.50573028 2.56144697
 1.40803813 1.77120542], Cost: 0.0530
Current parameters: [2.2882297  3.77140465 1.01660484 1.80289036 1.15988846 0.64280282
 2.02350801 1.359533   3.56649483 0.27587432 0.92067971 2.35763236
 1.58361154 0.03969232 0.43393239 2.95911151 1.59425196 2.58692783
 1.28184204 2.00128526], Cost: 0.9825
Current parameters: [2.30160977 3.91586624 1.29570164 2.05459553 1.25609159 0.59814121
 1.814988   1.2622586  3.43615669 0.44560513 0.95253593 2.37965372
 1.56450245 0.08760168 0.36339346 3.00602404 1.57524348 2.50938417
 1.37416162 1.78547186], Cost: 0.6294
Current parameters: [2.32817398 3.91759498 1.13765819 1.91492015 1.27243634 0.54842294
 1.82588067 1.2825511  3.44074784 0.45096389 0.90858469 2.34114026
 1.60090304 0.05640683 0.36784551 3.02921461 1.59210937 2.62644863
 1.4687532  1.76539039], Cost: 0.2779
Current parameters: [2.38696371 3.90124648 1.15965538 1.93320621 1.25592634 0.46800938
 1.89757767 1.27081153 3.44647361 0.44448259 0.94725675 2.37594274
 1.57262251 0.08566099 0.38013806 3.01472303 1.33669265 2.63133234
 1.47053881 1.74474336], Cost: 0.2017
Current parameters: [2.32110446 3.96012254 1.13745027 1.91473487 1.17573717 0.56037974
 1.82564654 1.27636041 3.4412568  0.45501445 0.90806267 2.34068075
 1.60109337 0.05575769 0.36758591 3.09575058 1.50547498 2.56215157
 1.40768243 1.77212093], Cost: 0.0419
Current parameters: [2.31940401 3.92312072 1.08326907 1.86534086 1.27737121 0.56583804
 1.83225874 1.22206631 3.42180791 0.46458355 0.96541772 2.39058859
 1.55691168 0.10136697 0.36117481 3.26690385 1.50573904 2.51393262
 1.3971978  1.75342054], Cost: 0.3872
Current parameters: [2.3252128  3.95117358 1.15203775 1.92773435 1.18782395 0.56871961
 1.83964219 1.27739667 3.44417104 0.45189389 0.9037673  2.37401797
 1.71398921 0.05888133 0.38326103 3.11800341 1.50069518 2.56789124
 1.40868914 1.77783769], Cost: 0.4394
Current parameters: [2.29506978 4.01498929 1.06249893 1.84702848 1.11792398 0.53047163
 1.79364382 1.23690643 3.4055106  0.48320371 0.98005826 2.41817031
 1.61074777 0.07549977 0.26834701 2.98084417 1.51154173 2.50408095
```



```
 1.40951564 1.73231211], Cost: 0.2614
Current parameters: [2.32251807 3.97642781 1.12446638 1.9031644  1.16702885 0.56170807
 1.81843035 1.26722464 3.43068289 0.46610827 0.91887398 2.33583085
 1.60046245 0.1111715  0.46893893 3.07314203 1.51545832 2.54701026
 1.40882353 1.75534706], Cost: 0.3367
Current parameters: [2.30345982 3.96502764 1.13515255 1.91173801 1.17867368 0.55590267
 1.85562785 1.24546453 3.42371991 0.46328947 0.95806097 2.11577468
 1.62725063 0.06691301 0.29865009 3.09561559 1.4787293  2.54113631
 1.41695599 1.76969707], Cost: 0.3929
Current parameters: [2.27542157 3.97257583 1.13574701 1.91321702 1.17804498 0.64712558
 1.8283006  1.26452562 3.42263261 0.46720184 0.91043111 2.3508872
 1.59166456 0.06784219 0.36453532 3.0903577  1.47015133 2.59061453
 1.45366091 1.79761776], Cost: 0.0563
Current parameters: [2.30871338 3.97016097 1.13016776 1.90720546 1.17578636 0.53745044
 1.85389197 1.24028265 3.42050688 0.46700736 0.68212447 2.34403625
 1.54821981 0.08172196 0.31682393 3.08814826 1.48577407 2.52799727
 1.41094441 1.75771718], Cost: 0.6968
Current parameters: [2.32080228 3.96070436 1.22056588 1.82147145 1.17600068 0.56047796
 1.82564464 1.27572435 3.44061865 0.45565327 0.90804977 2.34066956
 1.60110418 0.05205552 0.36915844 3.09569238 1.50549658 2.56171807
 1.40815076 1.77148062], Cost: 0.0463
Current parameters: [ 2.30190432  3.98120147  1.12734233  1.91745677  1.18293369  0.55772472
  1.84572656  1.23646856  3.4108293   0.47903139  0.92801155  2.34382899
  1.56603621 -0.16673718  0.43775268  3.09015436  1.48936431  2.5325401
  1.4254835   1.7503816 ], Cost: 0.3882
Current parameters: [2.32765421 3.94931015 1.13744362 1.91472365 1.17088008 0.55705906
 1.82573508 1.33357992 3.45309581 0.443161   0.90830647 2.34089452
 1.60091145 0.05595385 0.36758655 3.10145218 1.50501003 2.48777212
 1.48634366 1.78321309], Cost: 0.0947
Current parameters: [2.28738575 4.00755291 1.13348249 1.91104891 1.19490963 0.56425245
 1.84480089 1.47531462 3.38223215 0.50790865 0.92663803 2.34317852
 1.56786433 0.05206113 0.34556269 3.11442651 1.49106974 2.61489391
 1.33914312 1.72231834], Cost: 0.3078
Current parameters: [2.24426503 3.95025928 1.13990395 1.91691705 1.17373812 0.55100474
 1.82896494 1.27590734 3.5168526  0.44532157 0.90853509 2.34123007
 1.60208549 0.05733486 0.37091457 3.09906284 1.49991334 2.56615082
 1.41167237 1.71202132], Cost: 0.0283
Current parameters: [2.33921298 4.04891486 1.13276504 1.91050586 1.21195332 0.59404736
 1.83253475 1.27706695 3.67980152 0.55173441 0.91677837 2.34048301
 1.58240095 0.05113741 0.3518886  3.09190409 1.49755451 2.56643643
 1.40374858 1.75050025], Cost: 0.1686
```



```
Current parameters: [2.22477166 3.87869976 1.13892212 1.91592639 1.1422427  0.53298748
 1.83344375 1.27660247 3.51903323 0.53414577 0.91023457 2.34293426
 1.60152721 0.05892866 0.37451384 3.09945404 1.50514845 2.56774793
 1.41071511 1.74097043], Cost: 0.1129
Current parameters: [2.24063965 3.88309335 1.0809733  1.86241489 1.12121517 0.58640829
 1.89513617 1.31160187 3.58632602 0.32184973 0.93605996 2.34867622
 1.5510362  0.04178722 0.37063262 3.06773546 1.5060369  2.58413345
 1.34040669 1.82025096], Cost: 0.1467
Current parameters: [2.18800015 3.99900962 1.15486356 1.93007328 1.19836235 0.49136204
 1.83420067 1.27156482 3.51510233 0.44464401 0.91626875 2.34827914
 1.59868069 0.06557777 0.37717979 3.10285148 1.5025641  2.56362627
 1.41247678 1.78424115], Cost: 0.1487
Current parameters: [2.25572156 3.92389681 1.28406191 2.04438121 1.14624697 0.60028908
 1.81175691 1.25367715 3.51948267 0.41929867 0.9732429  2.37668494
 1.5207781  0.07807073 0.32760407 3.0780683  1.52965169 2.51935221
 1.36843141 1.69652365], Cost: 0.8468
Current parameters: [2.26757201 3.94029259 1.12736064 1.93093617 1.15675904 0.52820739
 1.7253745  1.27610435 3.52335336 0.43024081 0.9023722  2.33269929
 1.59192883 0.04560039 0.3634797  3.11543988 1.48057843 2.5985524
 1.3987793  1.73557628], Cost: 0.1100
Current parameters: [2.14788735 3.92330876 1.14278242 1.79269367 1.24970079 0.61095382
 1.76397235 1.33212208 3.55315291 0.43676585 0.91371433 2.3283459
 1.6351375  0.0542831  0.40255577 3.05552723 1.42176918 2.52909897
 1.48527857 1.6693868 ], Cost: 0.0897
Current parameters: [2.24055875 3.95360127 1.12647565 1.90304235 1.17331678 0.5459202
 1.84274739 1.26361085 3.51202089 0.44317511 0.7945037  2.34115217
 1.57469021 0.06536137 0.34674117 3.09354733 1.48625965 2.55461229
 1.40849798 1.70367726], Cost: 0.1941
Current parameters: [2.25733598 3.94328576 1.14633656 1.91687839 1.17199974 0.5334993
 1.84083138 1.28032263 3.52290377 0.44000054 0.91540888 2.34740325
 1.60020552 0.09553286 0.36169486 3.09615607 1.47070222 2.58525153
 1.42699    1.70795885], Cost: 0.0695
Current parameters: [ 2.25948347  3.95363102  1.13357126  1.91753559  1.17054956  0.52358212
  1.85841916  1.25648056  3.50789119  0.44666512  0.92399835  2.35072855
  1.57516096 -0.0291073   0.39950358  3.09354328  1.44855955  2.58245729
  1.43733622  1.69423755], Cost: 0.0271
Current parameters: [ 2.2578473   3.96171341  1.14473791  1.9278299   1.15076563  0.52263282
  1.86171152  1.26306228  3.5093345   0.44603089  0.91894398  2.31098258
  1.59274286 -0.03484815  0.39630823  3.06001565  1.44313763  2.58804185
  1.43929062  1.69777967], Cost: 0.1235
Current parameters: [ 2.26038315  3.94516364  1.10082251  1.89647407  1.19934034  0.5211421
```

```
  1.87285014  1.22266698  3.49425076  0.44698809  0.95728281  2.27530398
  1.56273642 -0.02590145  0.36545931  3.15025382  1.44289761  2.56773144
  1.43309839  1.68646239], Cost: 0.1312
Current parameters: [ 2.25849372  3.95175694  1.14627873  1.92866375  1.1839088   0.53001445
  1.86693465  1.29611392  3.50470627  0.45043     0.91408126  2.33585356
  1.5653641  -0.03164511  0.41725064  3.11666737  1.44421676  2.59986587
  1.42576498  1.69303892], Cost: 0.0498
Current parameters: [ 2.24977967  3.98998296  1.09258816  1.89427673  1.15509966  0.51083233
  1.85629878  1.31126435  3.47114905  0.47080601  0.95189174  2.37944048
  1.56506855 -0.03766359  0.35000715  3.07433901  1.45033152  2.59057287
  1.40477898  1.66384278], Cost: 0.1177
Current parameters: [ 2.23593846  3.92863704  1.14377006  1.9258531   1.16058508  0.51530647
  1.8560677   1.25488027  3.46827482  0.4207359   0.92522637  2.35244641
  1.57461765 -0.02676006  0.40090208  3.09408349  1.44966733  2.57914252
  1.43734943  1.6836729 ], Cost: 0.0242
Current parameters: [ 2.23792783  3.92399249  1.20658402  1.96105772  1.17280793  0.55593949
  1.84497721  1.2413278   3.49130371  0.42196267  0.9587231   2.38128931
  1.51883936 -0.03442161  0.37748345  3.08709827  1.44837539  2.53980079
  1.42597411  1.66429141], Cost: 0.3486
Current parameters: [ 2.24889983  3.91762297  1.13157855  1.93077145  1.1395703   0.51668889
  1.88012408  1.25466617  3.47771233  0.39678288  0.92997277  2.3553286
  1.55839075 -0.02999418  0.3953186   3.09290899  1.46080816  2.58665987
  1.41273334  1.70887758], Cost: 0.0208
Current parameters: [ 2.20976995  3.88424592  1.1056614   1.85924002  1.15734593  0.54781459
  1.87043291  1.28401698  3.50922887  0.35884756  0.9416821   2.36385116
  1.56938856 -0.03855062  0.42341312  3.07474604  1.43907357  2.58046188
  1.42714846  1.72552466], Cost: 0.0642
Current parameters: [ 2.24382961  3.92208571  1.13040799  1.92980548  1.14215158  0.53101863
  1.88125179  1.25602301  3.48325426  0.38839562  0.93491079  2.35072716
  1.56151912 -0.03885596  0.39487029  3.09353559  1.51235257  2.60134579
  1.42844922  1.69190149], Cost: 0.0218
Current parameters: [ 2.26700299  3.89679479  1.13020257  1.91281952  1.1447458   0.46592732
  1.8811619   1.24923944  3.46786426  0.40424034  0.91609994  2.37474355
  1.52032405 -0.02283703  0.38492183  3.0918775   1.45628583  2.65096388
  1.48116023  1.73600879], Cost: 0.1659
Current parameters: [ 2.267257    3.92305436  1.12328486  1.92925046  1.14568379  0.51865344
  1.88305077  1.2286065   3.4986673   0.40176033  0.92735435  2.39222547
  1.58242052 -0.04216265  0.39922879  3.10311712  1.46168684  2.58218222
  1.41707677  1.71366877], Cost: 0.0067
Current parameters: [ 2.26188491  3.89780082  1.13393159  1.94746699  1.14916334  0.51814656
  1.90385368  1.25961224  3.46110998  0.3785838   0.92123472  2.389377
```



```
  1.66961943 -0.05616062  0.3738017   3.14004523  1.45225543  2.6105157
  1.3968092   1.7126065 ], Cost: 0.0820
Current parameters: [ 2.27779095  3.92981134  1.10586766  1.92754776  1.13647269  0.51451354
  1.88403543  1.22162784  3.48089815  0.40135063  0.93174147  2.3924456
  1.58831769 -0.02268653  0.44632233  3.10520875  1.46815566  2.58291869
  1.40448188  1.70275003], Cost: 0.0706
Current parameters: [ 2.25867752  3.92361279  1.12682183  1.92345982  1.15192576  0.52327116
  1.8840056   1.21512345  3.4988922   0.40446237  0.92005152  2.39275069
  1.58028667 -0.04128784  0.39780254  3.09792429  1.45915138  2.59884397
  1.40250332  1.70898826], Cost: 0.0074
Current parameters: [ 2.26602182  3.92564967  1.1097725   1.92403539  1.14339838  0.52187036
  1.89044216  1.23063486  3.49537541  0.39446492  0.87388209  2.3923851
  1.57557732 -0.0445211   0.39138276  3.10798519  1.46678528  2.56098398
  1.41849574  1.70497979], Cost: 0.0516
Current parameters: [ 2.26117148  3.93119199  1.11656821  1.93573872  1.13996277  0.53875291
  1.88371978  1.22549661  3.48975677  0.40434572  0.92745916  2.39430685
  1.58078579 -0.04003048  0.39849494  3.10682581  1.45640914  2.58668926
  1.42593183  1.72370587], Cost: 0.0112
Current parameters: [ 2.23449638  3.95105347  1.12789808  1.93404274  1.16084799  0.49471194
  1.8883436   1.22669053  3.49946226  0.39574786  0.92879291  2.3956473
  1.58522828 -0.04307701  0.4047591   3.10778799  1.47424002  2.57307346
  1.41180151  1.74177391], Cost: 0.0389
Current parameters: [ 2.26965712  3.91579132  1.11011574  1.94362794  1.16451918  0.51965858
  1.88303459  1.22979502  3.49591765  0.40188714  0.92729354  2.39227922
  1.58250032 -0.04181772  0.39890188  3.09020252  1.46283943  2.58131435
  1.41659887  1.71561935], Cost: 0.0050
Current parameters: [ 2.24360315  3.91073159  1.0898532   1.96896841  1.14814725  0.50809024
  1.88357201  1.23004323  3.52496936  0.40002164  0.92947654  2.39334678
  1.58121521 -0.03718855  0.39947293  3.10080425  1.45047437  2.59057624
  1.42149726  1.69336961], Cost: 0.0031
Current parameters: [ 2.23616839  3.90028746  1.09204069  1.96625064  1.1458489   0.50894362
  1.9034682   1.23015867  3.5241589   0.41785586  0.93099768  2.39523823
  1.58289838 -0.03458858  0.40141527  3.09783253  1.45448271  2.58516717
  1.42389253  1.69390473], Cost: 0.0067
Current parameters: [ 2.23655968  3.88793791  1.08171332  1.97142024  1.13556021  0.50704129
  1.84675174  1.2291949   3.52579015  0.42529788  0.92324157  2.39019653
  1.58305483 -0.04945817  0.40097839  3.10931303  1.46641767  2.58925265
  1.40752951  1.71288471], Cost: 0.0408
Current parameters: [ 2.24503363  3.90913802  1.09090031  1.96716602  1.14988098  0.5105998
  1.87923032  1.23122148  3.53148253  0.39971081  0.92818074  2.39558262
  1.58621671 -0.00990926  0.39102842  3.10249669  1.45576645  2.58969356
```



```
  1.42042973  1.69732179], Cost: 0.0135
Current parameters: [ 2.24805241  3.90804653  1.06927327  1.95520175  1.16399965  0.5066241
  1.890512    1.20518613  3.52447838  0.40129495  0.94622464  2.36309055
  1.5768396  -0.03888745  0.3848601   3.12934052  1.44755206  2.58137075
  1.42239303  1.69114846], Cost: 0.0127
Current parameters: [ 2.24342522  3.92103848  1.07520943  1.95987847  1.14519068  0.50375822
  1.88280524  1.23980491  3.51898233  0.40748324  0.93566937  2.4026522
  1.58070518 -0.03847045  0.38758184  3.09702006  1.4515655   2.59251501
  1.41517926  1.68781118], Cost: 0.0123
Current parameters: [ 2.24410184  3.90606987  1.11454741  1.98403752  1.15746044  0.52763559
  1.88217181  1.22682336  3.53107906  0.39367565  0.95260779  2.41428163
  1.55909459 -0.04607345  0.38828634  3.10445008  1.4457531   2.57202968
  1.41096125  1.67516485], Cost: 0.1025
Current parameters: [ 2.25398711  3.91646956  1.09579898  1.98427739  1.14462763  0.49669908
  1.88590757  1.22278442  3.51633595  0.40921884  0.92567971  2.39346714
  1.57526657 -0.03451467  0.39219561  3.10559392  1.45530592  2.59712822
  1.42348927  1.69147969], Cost: 0.0082
Current parameters: [ 2.2462056   3.89207053  1.08629654  1.94920979  1.15620774  0.49060875
  1.88456995  1.23182691  3.52622688  0.39226774  0.9281834   2.40546044
  1.56689942 -0.03853276  0.40091442  3.09978532  1.44137396  2.62033966
  1.45663461  1.70562295], Cost: 0.0264
Current parameters: [ 2.2466938   3.91967101  1.08743285  1.96528611  1.14319181  0.50803229
  1.87931199  1.22053309  3.53679654  0.40637368  0.93071353  2.38848411
  1.56782812 -0.03545113  0.40218882  3.08466989  1.45177469  2.58417814
  1.42720063  1.69538006], Cost: 0.0180
Current parameters: [ 2.24360315  3.91073159  1.0898532   1.96896841  1.14814725  0.50809024
  1.88357201  1.23004323  3.52496936  0.40002164  0.92947654  2.39334678
  1.58121521 -0.03718855  0.39947293  3.10080425  1.45047437  2.59057624
  1.42149726  1.69336961], Cost: 0.0031

   Return from subroutine COBYLA because the MAXFUN limit has been reached.

   NFVALS =  100   F = 3.123005E-03    MAXCV = 0.000000E+00
   X = 2.243603E+00   3.910732E+00   1.089853E+00   1.968968E+00   1.148147E+00
       5.080902E-01   1.883572E+00   1.230043E+00   3.524969E+00   4.000216E-01
       9.294765E-01   2.393347E+00   1.581215E+00  -3.718855E-02   3.994729E-01
       3.100804E+00   1.450474E+00   2.590576E+00   1.421497E+00   1.693370E+00
Simulating for QAOA depth p = 11
Current parameters: [2.20772207 1.45981086 3.03906876 1.76112314 1.98053774 1.78577135
 2.26334224 2.46791678 0.5356113  2.74251006 0.61267395 1.51336989
 2.45622299 3.12478773 2.14430922 0.35892003 2.96527438 2.36582049
```



```
 2.2721135  2.59134036 0.47511248 3.1021357 ], Cost: 2.5702
Current parameters: [2.20772207 2.45981086 3.03906876 1.76112314 1.98053774 1.78577135
 2.26334224 2.46791678 0.5356113  2.74251006 0.61267395 1.51336989
 2.45622299 3.12478773 2.14430922 0.35892003 2.96527438 2.36582049
 2.2721135  2.59134036 0.47511248 3.1021357 ], Cost: 3.3752
Current parameters: [2.20772207 1.45981086 4.03906876 1.76112314 1.98053774 1.78577135
 2.26334224 2.46791678 0.5356113  2.74251006 0.61267395 1.51336989
 2.45622299 3.12478773 2.14430922 0.35892003 2.96527438 2.36582049
 2.2721135  2.59134036 0.47511248 3.1021357 ], Cost: 2.5807
Current parameters: [2.20772207 1.45981086 3.03906876 2.76112314 1.98053774 1.78577135
 2.26334224 2.46791678 0.5356113  2.74251006 0.61267395 1.51336989
 2.45622299 3.12478773 2.14430922 0.35892003 2.96527438 2.36582049
 2.2721135  2.59134036 0.47511248 3.1021357 ], Cost: 2.6307
Current parameters: [2.20772207 1.45981086 3.03906876 1.76112314 2.98053774 1.78577135
 2.26334224 2.46791678 0.5356113  2.74251006 0.61267395 1.51336989
 2.45622299 3.12478773 2.14430922 0.35892003 2.96527438 2.36582049
 2.2721135  2.59134036 0.47511248 3.1021357 ], Cost: 3.2327
Current parameters: [2.20772207 1.45981086 3.03906876 1.76112314 1.98053774 2.78577135
 2.26334224 2.46791678 0.5356113  2.74251006 0.61267395 1.51336989
 2.45622299 3.12478773 2.14430922 0.35892003 2.96527438 2.36582049
 2.2721135  2.59134036 0.47511248 3.1021357 ], Cost: 2.4044
Current parameters: [2.20772207 1.45981086 3.03906876 1.76112314 1.98053774 2.78577135
 3.26334224 2.46791678 0.5356113  2.74251006 0.61267395 1.51336989
 2.45622299 3.12478773 2.14430922 0.35892003 2.96527438 2.36582049
 2.2721135  2.59134036 0.47511248 3.1021357 ], Cost: 1.8473
Current parameters: [2.20772207 1.45981086 3.03906876 1.76112314 1.98053774 2.78577135
 3.26334224 3.46791678 0.5356113  2.74251006 0.61267395 1.51336989
 2.45622299 3.12478773 2.14430922 0.35892003 2.96527438 2.36582049
 2.2721135  2.59134036 0.47511248 3.1021357 ], Cost: 2.0431
Current parameters: [2.20772207 1.45981086 3.03906876 1.76112314 1.98053774 2.78577135
 3.26334224 2.46791678 1.5356113  2.74251006 0.61267395 1.51336989
 2.45622299 3.12478773 2.14430922 0.35892003 2.96527438 2.36582049
 2.2721135  2.59134036 0.47511248 3.1021357 ], Cost: 0.6286
Current parameters: [2.20772207 1.45981086 3.03906876 1.76112314 1.98053774 2.78577135
 3.26334224 2.46791678 1.5356113  3.74251006 0.61267395 1.51336989
 2.45622299 3.12478773 2.14430922 0.35892003 2.96527438 2.36582049
 2.2721135  2.59134036 0.47511248 3.1021357 ], Cost: 1.9954
Current parameters: [2.20772207 1.45981086 3.03906876 1.76112314 1.98053774 2.78577135
 3.26334224 2.46791678 1.5356113  2.74251006 1.61267395 1.51336989
 2.45622299 3.12478773 2.14430922 0.35892003 2.96527438 2.36582049
 2.2721135  2.59134036 0.47511248 3.1021357 ], Cost: 0.7527
```

```
Current parameters: [2.20772207 1.45981086 3.03906876 1.76112314 1.98053774 2.78577135
 3.26334224 2.46791678 1.5356113  2.74251006 0.61267395 2.51336989
 2.45622299 3.12478773 2.14430922 0.35892003 2.96527438 2.36582049
 2.2721135  2.59134036 0.47511248 3.1021357 ], Cost: 2.9883
Current parameters: [2.20772207 1.45981086 3.03906876 1.76112314 1.98053774 2.78577135
 3.26334224 2.46791678 1.5356113  2.74251006 0.61267395 1.51336989
 3.45622299 3.12478773 2.14430922 0.35892003 2.96527438 2.36582049
 2.2721135  2.59134036 0.47511248 3.1021357 ], Cost: 1.3406
Current parameters: [2.20772207 1.45981086 3.03906876 1.76112314 1.98053774 2.78577135
 3.26334224 2.46791678 1.5356113  2.74251006 0.61267395 1.51336989
 2.45622299 4.12478773 2.14430922 0.35892003 2.96527438 2.36582049
 2.2721135  2.59134036 0.47511248 3.1021357 ], Cost: 1.6039
Current parameters: [2.20772207 1.45981086 3.03906876 1.76112314 1.98053774 2.78577135
 3.26334224 2.46791678 1.5356113  2.74251006 0.61267395 1.51336989
 2.45622299 3.12478773 3.14430922 0.35892003 2.96527438 2.36582049
 2.2721135  2.59134036 0.47511248 3.1021357 ], Cost: 2.2597
Current parameters: [2.20772207 1.45981086 3.03906876 1.76112314 1.98053774 2.78577135
 3.26334224 2.46791678 1.5356113  2.74251006 0.61267395 1.51336989
 2.45622299 3.12478773 2.14430922 1.35892003 2.96527438 2.36582049
 2.2721135  2.59134036 0.47511248 3.1021357 ], Cost: 1.6996
Current parameters: [2.20772207 1.45981086 3.03906876 1.76112314 1.98053774 2.78577135
 3.26334224 2.46791678 1.5356113  2.74251006 0.61267395 1.51336989
 2.45622299 3.12478773 2.14430922 0.35892003 3.96527438 2.36582049
 2.2721135  2.59134036 0.47511248 3.1021357 ], Cost: 1.9238
Current parameters: [2.20772207 1.45981086 3.03906876 1.76112314 1.98053774 2.78577135
 3.26334224 2.46791678 1.5356113  2.74251006 0.61267395 1.51336989
 2.45622299 3.12478773 2.14430922 0.35892003 2.96527438 3.36582049
 2.2721135  2.59134036 0.47511248 3.1021357 ], Cost: 1.9507
Current parameters: [2.20772207 1.45981086 3.03906876 1.76112314 1.98053774 2.78577135
 3.26334224 2.46791678 1.5356113  2.74251006 0.61267395 1.51336989
 2.45622299 3.12478773 2.14430922 0.35892003 2.96527438 2.36582049
 3.2721135  2.59134036 0.47511248 3.1021357 ], Cost: 2.7391
Current parameters: [2.20772207 1.45981086 3.03906876 1.76112314 1.98053774 2.78577135
 3.26334224 2.46791678 1.5356113  2.74251006 0.61267395 1.51336989
 2.45622299 3.12478773 2.14430922 0.35892003 2.96527438 2.36582049
 2.2721135  3.59134036 0.47511248 3.1021357 ], Cost: 2.1820
Current parameters: [2.20772207 1.45981086 3.03906876 1.76112314 1.98053774 2.78577135
 3.26334224 2.46791678 1.5356113  2.74251006 0.61267395 1.51336989
 2.45622299 3.12478773 2.14430922 0.35892003 2.96527438 2.36582049
 2.2721135  2.59134036 1.47511248 3.1021357 ], Cost: 1.3473
Current parameters: [2.20772207 1.45981086 3.03906876 1.76112314 1.98053774 2.78577135
```



```
 3.26334224 2.46791678 1.5356113  2.74251006 0.61267395 1.51336989
 2.45622299 3.12478773 2.14430922 0.35892003 2.96527438 2.36582049
 2.2721135  2.59134036 0.47511248 4.1021357 ], Cost: 1.9655
Current parameters: [2.31559745 1.30927732 3.03708953 1.74980723 1.85665543 2.81677628
 3.36751262 2.43129893 1.76349362 2.48692885 0.58946595 1.07211645
 2.32308124 2.94241049 1.83930908 0.15864532 2.72307838 2.11859488
 1.87746969 2.30087367 0.34071594 2.85214548], Cost: 1.8385
Current parameters: [2.26165976 1.38454409 3.03807914 1.75546519 1.91859658 2.80127382
 3.31542743 2.44960785 1.64955246 2.61471946 0.60106995 1.29274317
 2.38965211 3.03359911 1.99180915 0.25878267 2.84417638 2.24220768
 2.07479159 2.44610701 0.40791421 2.97714059], Cost: 2.0071
Current parameters: [2.4380463  1.45981086 3.03906876 1.76112314 1.98053774 2.78577135
 3.26334224 2.46791678 1.5356113  2.83972501 0.61267395 1.51336989
 2.45622299 3.12478773 2.14430922 0.35892003 2.96527438 2.36582049
 2.2721135  2.59134036 0.47511248 3.1021357 ], Cost: 0.9058
Current parameters: [1.99859409 1.44555055 3.03888126 1.76005117 1.96880215 2.7887085
 3.27321048 2.4644479  1.55719899 3.18746176 0.61047542 1.47156917
 2.44361024 3.10751081 2.11541601 0.33994765 2.94233072 2.34240037
 2.23472819 2.56382393 0.46238086 3.07845368], Cost: 0.8401
Current parameters: [2.20772207 1.4630976  2.78909037 1.76112314 1.98053774 2.78577135
 3.26334224 2.46791678 1.5356113  2.74251006 0.61267395 1.51336989
 2.45622299 3.12478773 2.14430922 0.35892003 2.96527438 2.36582049
 2.2721135  2.59134036 0.47511248 3.1021357 ], Cost: 1.0802
Current parameters: [2.1973985  1.95655884 3.06348644 1.76052397 1.9739782  2.78741305
 3.26885803 2.46597787 1.54767761 2.73873117 0.61144509 1.49000563
 2.44917317 3.1151309  2.12815953 0.34831553 2.95245016 2.35272996
 2.25121721 2.57596022 0.46799621 3.08889878], Cost: 1.4899
Current parameters: [2.20772207 1.45981086 3.03906876 1.51215963 2.00327907 2.78577135
 3.26334224 2.46791678 1.5356113  2.74251006 0.61267395 1.51336989
 2.45622299 3.12478773 2.14430922 0.35892003 2.96527438 2.36582049
 2.2721135  2.59134036 0.47511248 3.1021357 ], Cost: 1.0277
Current parameters: [2.1993388  1.44751048 3.05343169 1.81918369 2.47506343 2.7871045
 3.26782135 2.46634228 1.54540976 2.73944141 0.61167606 1.49439692
 2.45049817 3.11694589 2.13119485 0.35030863 2.95486046 2.35519031
 2.25514464 2.5788509  0.46933371 3.09138664], Cost: 0.7749
Current parameters: [2.20772207 1.45981086 3.03906876 1.76112314 1.98053774 3.02538321
 3.19202495 2.46791678 1.5356113  2.74251006 0.61267395 1.51336989
 2.45622299 3.12478773 2.14430922 0.35892003 2.96527438 2.36582049
 2.2721135  2.59134036 0.47511248 3.1021357 ], Cost: 0.9092
Current parameters: [2.20035248 1.4489978  3.05169497 1.77224    1.97820286 2.6360416
 2.78808798 2.46653267 1.54422497 2.73981246 0.61179672 1.49669106
```



```
 2.4511904  3.1178941  2.13278059 0.35134989 2.95611968 2.35647567
 2.25719646 2.58036108 0.47003246 3.09268638], Cost: 1.2187
Current parameters: [2.20701095 1.46080318 3.03908181 1.76119774 1.98135438 2.78556696
 3.26265554 2.71790575 1.5356113  2.74419487 0.61267395 1.51336989
 2.45622299 3.12478773 2.14430922 0.35892003 2.96527438 2.36582049
 2.2721135  2.59134036 0.47511248 3.1021357 ], Cost: 0.9553
Current parameters: [2.17577974 1.28528411 3.1693793  1.87189871 1.90507786 2.74263814
 3.40721984 2.37406537 1.62506801 2.60884069 0.6035635  1.34015294
 2.40395732 3.05319434 2.0245794  0.28030087 2.87019874 2.26877044
 2.11719347 2.47731574 0.42235423 3.00400039], Cost: 2.1487
Current parameters: [2.19385279 1.44701474 3.05835845 1.77834108 1.98000976 2.77680307
 3.27558739 2.45386056 1.5487378  2.74465069 0.61133712 1.75584898
 2.44855375 3.11428242 2.12674057 0.34738378 2.95132338 2.35157978
 2.24938119 2.57460887 0.46737095 3.08773574], Cost: 1.0425
Current parameters: [2.20772207 1.45981086 3.03906876 1.76112314 1.98053774 2.78577135
 3.26334224 2.46791678 1.52294655 2.74251006 0.48831719 1.51336989
 2.45622299 3.12478773 2.14430922 0.35892003 2.96527438 2.36582049
 2.2721135  2.59134036 0.47511248 3.1021357 ], Cost: 0.6351
Current parameters: [2.20037757 1.45303465 3.04928364 1.77024092 1.98025815 2.78102218
 3.26982668 2.46047329 1.28893683 2.74364364 0.63809522 1.50941754
 2.45216173 3.11922462 2.13500571 0.35281099 2.95788661 2.3582793
 2.26007557 2.58248017 0.47101294 3.09451018], Cost: 0.6442
Current parameters: [2.20772207 1.45981086 3.03906876 1.76112314 1.98053774 2.78577135
 3.26334224 2.46791678 1.5356113  2.74251006 0.61267395 1.51336989
 2.55718218 3.05108399 2.14430922 0.35892003 2.96527438 2.36582049
 2.2721135  2.59134036 0.47511248 3.1021357 ], Cost: 0.5679
Current parameters: [2.20020092 1.45287167 3.04952933 1.77046023 1.98025142 2.78090796
 3.26998264 2.46029425 1.53309236 2.7436709  0.61323712 1.50932247
 2.70567245 3.24967733 2.13478194 0.35266406 2.95770892 2.35809792
 2.25978603 2.58226706 0.47091434 3.09432677], Cost: 0.1850
Current parameters: [2.20020092 1.45287167 3.04952933 1.77046023 1.98025142 2.78090796
 3.26998264 2.46029425 1.53309236 2.7436709  0.61323712 1.50932247
 2.70567245 3.24967733 2.03587715 0.35266406 2.95770892 2.35809792
 2.33622452 2.58226706 0.47091434 3.09432677], Cost: 0.2875
Current parameters: [2.18069319 1.4348734  3.07666109 1.79467797 1.9795088  2.76829369
 3.28720597 2.4405236  1.52655895 2.74668179 0.61469782 1.49882461
 2.72940227 3.26971113 2.28959334 0.33643785 2.93808625 2.33806776
 2.43978159 2.55873352 0.46002556 3.07407262], Cost: 0.8386
Current parameters: [2.20020092 1.45287167 3.04952933 1.77046023 1.98025142 2.78090796
 3.26998264 2.46029425 1.53309236 2.7436709  0.61323712 1.50932247
 2.70567245 3.24967733 2.13478194 0.35266406 2.95770892 2.35809792
```



```
 2.25978603 2.66380738 0.47091434 2.99958408], Cost: 0.1935
Current parameters: [2.1848614  1.43871908 3.07086386 1.78950338 1.97966747 2.77098898
 3.28352587 2.44474799 1.52795494 2.74603845 0.61438571 1.50106769
 2.72433193 3.26543051 2.13105543 0.3399049  2.94227902 2.34234759
 2.23898836 2.76561552 0.46235217 3.25319659], Cost: 1.0015
Current parameters: [2.20020092 1.45287167 3.04952933 1.77046023 1.98025142 2.78090796
 3.26998264 2.46029425 1.53309236 2.7436709  0.61323712 1.50932247
 2.70567245 3.24967733 2.13478194 0.35266406 2.86841695 2.44557331
 2.25978603 2.58226706 0.47091434 3.09432677], Cost: 0.1829
Current parameters: [2.18672071 1.44043453 3.06827789 1.78719514 1.97973825 2.77219126
 3.28188429 2.44663236 1.52857765 2.74575148 0.61424649 1.50206825
 2.7220702  3.26352106 2.13150712 0.34145144 3.03893529 2.61988188
 2.24150926 2.56322037 0.46338999 3.07887536], Cost: 1.2758
Current parameters: [2.20020092 1.45287167 3.04952933 1.77046023 1.98025142 2.78090796
 3.26998264 2.46029425 1.53309236 2.7436709  0.61323712 1.50932247
 2.70567245 3.24967733 2.13478194 0.42231703 2.86841695 2.44557331
 2.25978603 2.58226706 0.36711904 3.09432677], Cost: 0.4680
Current parameters: [2.19168432 1.44501406 3.0613744  1.7810331  1.97992721 2.77540088
 3.27750193 2.45166287 1.53024003 2.74498538 0.61387483 1.50473936
 2.71603231 3.2584236  2.13271296 0.54863979 2.85194918 2.42892095
 2.24823903 2.57023364 0.62059523 3.08456479], Cost: 0.5416
Current parameters: [2.20311599 1.33000274 3.04791384 1.77003334 1.97557804 2.7820776
 3.2739124  2.46086405 1.53815505 2.73676444 0.61272153 1.49951947
 2.70271454 3.24562559 2.12800598 0.3482147  2.86303626 2.44008088
 2.25101852 2.57581398 0.46792855 3.08877292], Cost: 0.1332
Current parameters: [2.20038751 1.36104258 3.08602791 1.80095681 1.94782326 2.77138902
 3.32011947 2.43401351 1.56331305 2.68370126 0.61125512 1.41891318
 2.71544982 3.24588847 2.07575777 0.2691336  2.77476348 2.35047165
 2.15542272 2.49429914 0.47219767 3.02049354], Cost: 0.8390
Current parameters: [2.24795974 1.33246353 3.04794619 1.77021707 1.97758938 2.76932746
 3.2310745  2.46156316 1.53380429 2.63051944 0.61316462 1.50794395
 2.7052565  3.24910757 2.13382909 0.35203838 2.8676603  2.44480095
 2.25855312 2.58135961 0.47049447 3.09354577], Cost: 0.0543
Current parameters: [2.2111886  1.32308285 3.05695547 1.77800009 1.97407659 2.69765115
 3.01014599 2.45374291 1.53552418 2.70322065 0.61325485 1.49688917
 2.71089633 3.25267143 2.12702783 0.33672813 2.85092833 2.42784181
 2.24297326 2.56719087 0.47410782 3.08180591], Cost: 0.1258
Current parameters: [2.24798349 1.32976286 3.04791069 1.78136061 2.09958469 2.76959713
 3.23198052 2.46117519 1.53533571 2.63046317 0.61300865 1.50497862
 2.70765378 3.25239135 2.13674603 0.35017751 2.87354622 2.43453306
 2.24370127 2.56723504 0.46924571 3.09641703], Cost: 0.1130
```

```
Current parameters: [2.23981792 1.32467275 3.06391085 1.78798967 2.01607398 2.75908231
 3.23164483 2.44985098 1.5344006  2.62444363 0.6135708  1.4931995
 2.67818413 3.20470243 2.0671607  0.33324725 2.75313167 2.51802772
 2.3777074  2.70156861 0.48231682 3.02244918], Cost: 0.5371
Current parameters: [2.24715767 1.37267498 2.94432698 1.77034671 1.97900864 2.76886483
 3.22952016 2.46138454 1.52857761 2.63241972 0.61369691 1.51806449
 2.71145631 3.25760004 2.14557097 0.31586102 2.83951391 2.45630539
 2.25594572 2.5763888  0.49323516 3.103629  ], Cost: 0.0272
Current parameters: [2.23816723 1.23905433 2.80691111 1.79148742 1.96379194 2.75298926
 3.231479   2.44369242 1.52990536 2.61363499 0.61429954 1.49401764
 2.71670972 3.25322609 2.11250985 0.41325212 2.90525929 2.39832446
 2.26472934 2.58741391 0.43964271 3.05757386], Cost: 0.3955
Current parameters: [2.22599346 1.32429186 2.94697126 1.77038099 1.97938396 2.77452554
 3.24853898 2.46123395 1.53125501 2.68256252 0.61342424 1.61417594
 2.71052115 3.25631906 2.14293666 0.29887427 2.82335127 2.45682944
 2.24897759 2.57064985 0.49880328 3.10156163], Cost: 0.1653
Current parameters: [2.29335358 1.46894347 2.96352901 1.79351572 1.96772369 2.73792012
 3.18494637 2.44231564 1.5283738  2.47991639 0.61451018 1.6487731
 2.72578796 3.26480429 2.12391437 0.34100475 2.84864234 2.40338425
 2.23953151 2.56170091 0.4753653  3.06664611], Cost: 0.1772
Current parameters: [2.25765562 1.34249677 2.97754887 1.6657803  1.94180689 2.77220422
 3.24073984 2.46188637 1.52565462 2.60754769 0.61399459 1.51996404
 2.71427829 3.26146558 2.1511641  0.32675024 2.85378532 2.45359859
 2.25687865 2.57599468 0.49062657 3.10837595], Cost: 0.0109
Current parameters: [2.22563504 1.42218716 2.87996774 1.5332537  2.07629306 2.74299997
 3.19925673 2.44190033 1.52069179 2.6423554  0.61525575 1.51082856
 2.72909817 3.26991545 2.13443194 0.29635211 2.80413031 2.43310624
 2.2555343  2.57410311 0.50575993 3.07608378], Cost: 0.2357
Current parameters: [2.25902477 1.36842684 2.95377471 1.69072594 1.94177475 2.77095123
 3.23653002 2.57139759 1.52149192 2.60430386 0.61441853 1.48695882
 2.71809208 3.2666897  2.15882006 0.31245162 2.84335649 2.46069473
 2.25896718 2.57619163 0.50174051 3.11485272], Cost: 0.0426
Current parameters: [2.24516332 1.25006268 3.05272473 1.5667219  1.94732696 2.75325485
 3.24069857 2.57406458 1.52731356 2.59102328 0.61467461 1.63912243
 2.72471264 3.26244615 2.11968723 0.37759927 2.87818749 2.39614716
 2.25020547 2.57199742 0.45838819 3.06141642], Cost: 0.3828
Current parameters: [2.25628785 1.34181626 2.9756691  1.66329247 1.94263226 2.88993571
 3.20124624 2.46096714 1.5228058  2.60068228 0.61447075 1.52060377
 2.71950769 3.26571184 2.15017504 0.32685798 2.8510844  2.44648607
 2.25702815 2.5752708  0.49213914 3.10308441], Cost: 0.0464
Current parameters: [2.26271938 1.34418575 2.9750028  1.66350769 1.94187658 2.77338796
```



```
 3.23912358 2.46374166 1.58714596 2.60862978 0.60773219 1.52094917
 2.71529056 3.26285219 2.15348301 0.32827292 2.85562673 2.45547824
 2.25987912 2.57820309 0.49164838 3.11027662], Cost: 0.0041
Current parameters: [2.37902487 1.34329982 2.97334111 1.661189   1.94272037 2.77312371
 3.23474721 2.46259139 1.57750963 2.65134854 0.60890546 1.52150176
 2.7205745  3.26708123 2.1522102  0.32821821 2.85264013 2.44793685
 2.25970651 2.57721585 0.49309732 3.1046113 ], Cost: 0.0558
Current parameters: [2.26271938 1.34418575 2.9750028  1.66350769 1.94187658 2.77338796
 3.23912358 2.46374166 1.59344199 2.60862978 0.66955372 1.52094917
 2.7098901  3.26679471 2.15348301 0.32827292 2.85562673 2.45547824
 2.25987912 2.57820309 0.49164838 3.11027662], Cost: 0.0057
Current parameters: [2.26410688 1.34391148 2.97407696 1.66230795 1.94225864 2.77335511
 3.23705164 2.46336125 1.58590134 2.60629243 0.59439123 1.52126702
 2.61684374 3.33848828 2.15308122 0.32835811 2.85442476 2.45224372
 2.26001803 2.57792095 0.49236941 3.10788194], Cost: 0.0196
Current parameters: [2.26558439 1.34794418 2.97078558 1.65961089 1.9417462  2.77554128
 3.23663416 2.46695964 1.58813423 2.60889097 0.60750179 1.52270655
 2.67987637 3.21637653 2.16113991 0.32900432 2.86140619 2.45528153
 2.25950755 2.56163459 0.49457128 3.10430999], Cost: 0.0760
Current parameters: [2.26489194 1.3428011  2.97634093 1.66210604 1.94266269 2.77113372
 3.23283277 2.45774219 1.58607244 2.60001605 0.60761681 1.52077376
 2.70550177 3.24923245 2.16026061 0.31698398 2.85675127 2.41870206
 2.22643419 2.64909479 0.49846613 3.1966411 ], Cost: 0.2560
Current parameters: [2.26492974 1.33934394 2.97336332 1.66286208 1.94343828 2.77296149
 3.23692471 2.46404194 1.58770891 2.60394823 0.60766464 1.52080817
 2.71463403 3.26211316 2.16038617 0.2871335  2.86611381 2.45153597
 2.2724283  2.57967404 0.44903344 3.10862433], Cost: 0.0100
Current parameters: [2.25742127 1.3409104  2.98506227 1.66981021 1.94130842 2.7681224
 3.24033697 2.45282281 1.58399304 2.6053766  0.60810515 1.51783876
 2.73136626 3.28096472 2.2388167  0.33590332 2.84643193 2.41943514
 2.32767598 2.55344611 0.5183377  3.11613852], Cost: 0.1164
Current parameters: [2.26473152 1.34837996 2.96820119 1.65976233 1.9518909  2.77228467
 3.22670769 2.46654669 1.58137324 2.61017576 0.60820389 1.5370305
 2.71558639 3.26507963 2.16303955 0.33765451 2.89092082 2.491248
 2.27592733 2.59241726 0.49501498 3.1195387 ], Cost: 0.0857
Current parameters: [2.2586719  1.34112211 2.99649016 1.67009405 1.91863875 2.77137386
 3.25657976 2.44314913 1.59618146 2.5949031  0.60658634 1.48544943
 2.74017344 3.29199862 2.14480972 0.27849231 2.87828979 2.48442019
 2.20933153 2.53044746 0.52674696 3.08114093], Cost: 0.0623
Current parameters: [2.25599525 1.33705632 2.98594069 1.66749628 1.92793029 2.75613932
 3.18541654 2.46213646 1.58784809 2.62148365 0.60771733 1.51310671
```



```
 2.7151154  3.26172408 2.15055009 0.32587824 2.84924912 2.45573397
 2.25977687 2.5779774  0.49302396 3.10710311], Cost: 0.0069
Current parameters: [2.26883361 1.33318449 2.99820845 1.67232124 1.93611773 2.77459478
 3.25597884 2.453246   1.59007581 2.58191058 0.60732295 1.49943522
 2.70928388 3.25201276 2.1197579  0.31306522 2.80067706 2.49158806
 2.31152083 2.63037361 0.5067602  3.06816487], Cost: 0.1914
Current parameters: [2.26007443 1.35089728 2.97740027 1.6640298  1.9383786  2.77405719
 3.24112465 2.46376342 1.58717633 2.61507563 0.6077258  1.51969687
 2.71503031 3.26254747 2.11537109 0.31880527 2.87410345 2.43811363
 2.28985588 2.56420316 0.50652274 3.12722646], Cost: 0.0237
Current parameters: [2.27238184 1.32495156 2.96937978 1.66061301 1.94963085 2.7717765
 3.23394467 2.46005339 1.58840078 2.58383354 0.60744229 1.52042319
 2.72147948 3.27131973 2.13810236 0.34775321 2.80505032 2.49957523
 2.26378303 2.51342169 0.45717668 3.17170147], Cost: 0.0784
Current parameters: [2.24928483 1.31887248 2.97941134 1.64874383 1.94232227 2.78163656
 3.25372215 2.46793095 1.58741252 2.6499664  0.60753005 1.4964827
 2.71191686 3.26097501 2.15784845 0.3292487  2.856311   2.46605981
 2.26341499 2.58145839 0.48852522 3.11808986], Cost: 0.0109
Current parameters: [2.24607785 1.42432703 2.99968641 1.67309234 1.90178028 2.77690481
 3.253679   2.45511269 1.58987732 2.64278095 0.60717684 1.51188342
 2.72456128 3.27505243 2.14860706 0.36681253 2.85567395 2.45129627
 2.25339728 2.5720063  0.43662437 3.0964126 ], Cost: 0.1214
Current parameters: [2.26122289 1.3011636  2.89753333 1.69050632 1.92714009 2.76537614
 3.23676052 2.45026919 1.58928293 2.59183638 0.60760012 1.48275137
 2.72532288 3.27151416 2.13766313 0.36906232 2.88215851 2.42870218
 2.25765975 2.57688414 0.46897001 3.08206839], Cost: 0.1398
Current parameters: [2.25967583 1.32146298 2.99687896 1.63967201 1.94020204 2.76978882
 3.24141058 2.4899302  1.58900835 2.60374577 0.60757504 1.54726
 2.72276273 3.27011672 2.14547434 0.3378192  2.85975659 2.44042478
 2.25234536 2.57142863 0.48472271 3.09448524], Cost: 0.0354
Current parameters: [2.26118811 1.34605114 2.9599419  1.65153048 1.92238318 2.77349753
 3.24042862 2.46347056 1.58599308 2.61157856 0.6078621  1.53063087
 2.71627438 3.2640038  2.15480995 0.32188681 2.84929286 2.45838118
 2.26129228 2.57911862 0.49589618 3.11107343], Cost: 0.0124
Current parameters: [2.25480668 1.3630161  2.95763181 1.63372499 1.98049455 2.76651697
 3.22974102 2.45801255 1.58727234 2.61634016 0.60779693 1.51509282
 2.72201878 3.26922643 2.14833308 0.32086222 2.84380687 2.44833271
 2.25509912 2.57355011 0.49459015 3.0986209 ], Cost: 0.0228
Current parameters: [2.26131337 1.34330974 2.97381931 1.66975566 1.94373079 2.74574197
 3.24887311 2.46373742 1.58813045 2.61434509 0.60758804 1.52599104
 2.71384554 3.26156096 2.15325478 0.3267518  2.85462218 2.45732033
```



```
 2.25940951 2.57808211 0.49171855 3.11114246], Cost: 0.0101
Current parameters: [2.25152397 1.33775605 2.96713339 1.69742953 1.95462715 2.79401947
 3.23640403 2.46259557 1.58959526 2.63233816 0.60740497 1.55131837
 2.71836864 3.26661036 2.14991188 0.31729075 2.84369607 2.45403972
 2.25309008 2.57224534 0.49501772 3.10355217], Cost: 0.0308
Current parameters: [2.26325311 1.35264659 2.96737866 1.67184102 1.94196916 2.77343518
 3.23806923 2.48809997 1.58609335 2.6083447  0.60782136 1.51033484
 2.71600298 3.26411077 2.15602548 0.32357595 2.85243999 2.45847029
 2.26052423 2.57832908 0.49510974 3.11285944], Cost: 0.0048
Current parameters: [2.26003272 1.32441389 2.99511022 1.64252618 1.94111341 2.77054284
 3.2406173  2.50007101 1.58862974 2.60300048 0.60760891 1.54261966
 2.72318055 3.27072577 2.14628529 0.33659753 2.85900418 2.44110089
 2.25244796 2.57134621 0.48572034 3.09516108], Cost: 0.0384
Current parameters: [2.29159322 1.34394978 2.97512005 1.66293297 1.94213201 2.77347257
 3.23828762 2.46343767 1.58488004 2.61928317 0.60798611 1.52081634
 2.71739897 3.264996   2.15302193 0.3278197  2.85453277 2.45330895
 2.2588806  2.57702857 0.49212645 3.10805804], Cost: 0.0078
Current parameters: [2.26306366 1.34443711 2.97531676 1.66318775 1.94059114 2.7737182
 3.23976123 2.46338192 1.58783088 2.6079051  0.60764444 1.51923106
 2.71478572 3.26244282 2.14521444 0.32412679 2.85925521 2.46155662
 2.2499064  2.57821369 0.49176541 3.10888268], Cost: 0.0028
Current parameters: [2.26183749 1.34384746 2.98254656 1.66472204 1.93704602 2.77314205
 3.24285311 2.45777152 1.58907939 2.60452734 0.60749268 1.51211459
 2.72166171 3.27056077 2.15868368 0.31368323 2.86416992 2.4608859
 2.24643277 2.56409144 0.50402011 3.1053385 ], Cost: 0.0010
Current parameters: [2.26140237 1.34464369 2.98071241 1.66391676 1.93743642 2.77289009
 3.24116552 2.45819826 1.58886974 2.60624843 0.60750656 1.51371787
 2.72166723 3.2706855  2.16198053 0.31408306 2.86846189 2.45424565
 2.23900412 2.56449126 0.5031528  3.11537974], Cost: 0.0065
Current parameters: [2.26355318 1.34124453 2.99265605 1.66613476 1.93690401 2.77412168
 3.24651196 2.45380668 1.59070774 2.59678357 0.60723766 1.50568588
 2.72356893 3.27106428 2.1566456  0.30447926 2.85039049 2.45709817
 2.2454568  2.58293916 0.51146443 3.10980654], Cost: 0.0342
Current parameters: [2.26314609 1.33321089 2.9739812  1.66618143 1.93927886 2.77216254
 3.24147212 2.45737933 1.58907296 2.60012736 0.60752277 1.50964095
 2.72188365 3.27040312 2.15777376 0.3136373  2.86619644 2.45876604
 2.24667247 2.56465183 0.50698606 3.10405881], Cost: 0.0025
Current parameters: [2.25871291 1.35021301 2.97650935 1.67208187 1.92712755 2.773203
 3.24636194 2.4523234  1.59076776 2.60614537 0.60726235 1.50039942
 2.72809716 3.27729473 2.15599562 0.32543705 2.86717136 2.45370137
 2.24374338 2.56248466 0.49250873 3.09450189], Cost: 0.0099
```

```
Current parameters: [2.26187606 1.34350747 2.98251953 1.66479196 1.93718099 2.77311389
 3.24295971 2.45775718 1.58925425 2.60431379 0.60912784 1.51210871
 2.73383624 3.26131768 2.15843629 0.31408611 2.863394   2.46227281
 2.24655793 2.56278331 0.50323847 3.10691859], Cost: 0.0014
Current parameters: [2.26416075 1.3395756  2.98217509 1.6648991  1.93814471 2.77309993
 3.24229101 2.45573403 1.58982532 2.59758827 0.60676415 1.50974956
 2.72067718 3.27791977 2.15498771 0.31851228 2.85074328 2.47060739
 2.24669848 2.54742215 0.49538691 3.11848708], Cost: 0.0045
Current parameters: [2.2616331  1.34383644 2.98221239 1.66483323 1.9387266  2.77255587
 3.24126654 2.45763208 1.58793943 2.60473404 0.60761388 1.51444459
 2.72835027 3.27964263 2.15882856 0.3150976  2.86892317 2.46683826
 2.24915643 2.56954848 0.50402887 3.10798888], Cost: 0.0011
Current parameters: [2.264277   1.34882686 2.98103108 1.6646717  1.93925757 2.77503165
 3.23849689 2.45600279 1.58922369 2.60121032 0.60691556 1.51209294
 2.71190238 3.2597367  2.16808948 0.32047602 2.87872846 2.47761062
 2.25092057 2.55871787 0.50493948 3.10172605], Cost: 0.0241
Current parameters: [2.26359323 1.3421414  2.98706348 1.65497131 1.9344594  2.76868979
 3.24360638 2.46346181 1.5888137  2.59910753 0.60754216 1.5094733
 2.72225211 3.27101783 2.15827442 0.31703456 2.86698687 2.45899945
 2.24665296 2.56420795 0.50248564 3.10438074], Cost: 0.0015
Current parameters: [2.25720633 1.33461047 2.98900527 1.66856818 1.9398511  2.77943735
 3.24282462 2.47102619 1.59107278 2.60872889 0.60708949 1.52914949
 2.72566849 3.27555011 2.15639469 0.31499918 2.86320892 2.45771035
 2.24152025 2.55583015 0.50231794 3.09420674], Cost: 0.0133
Current parameters: [2.2628143  1.34170049 2.98611554 1.67017401 1.9297144  2.77447575
 3.24486469 2.45879004 1.5891388  2.60420944 0.60745426 1.51291387
 2.72042598 3.26937564 2.15298822 0.31344002 2.86949159 2.45912546
 2.25244372 2.56248652 0.50609478 3.11032801], Cost: 0.0003
Current parameters: [2.26183749 1.34384746 2.98254656 1.66472204 1.93704602 2.77314205
 3.24285311 2.45777152 1.58907939 2.60452734 0.60749268 1.51211459
 2.72166171 3.27056077 2.15868368 0.31368323 2.86416992 2.4608859
 2.24643277 2.56409144 0.50402011 3.1053385 ], Cost: 0.0010

   Return from subroutine COBYLA because the MAXFUN limit has been reached.

   NFVALS =  100   F = 9.507447E-04    MAXCV = 0.000000E+00
   X = 2.261837E+00   1.343847E+00   2.982547E+00   1.664722E+00   1.937046E+00
       2.773142E+00   3.242853E+00   2.457772E+00   1.589079E+00   2.604527E+00
       6.074927E-01   1.512115E+00   2.721662E+00   3.270561E+00   2.158684E+00
       3.136832E-01   2.864170E+00   2.460886E+00   2.246433E+00   2.564091E+00
       5.040201E-01   3.105338E+00
```



```
Simulating for QAOA depth p = 12
Current parameters: [1.91321734 0.41740286 0.36774659 1.18313983 0.32439416 0.3617283
 1.51237313 2.51121451 1.13019129 1.3955932  2.28051952 2.91962826
 1.75211687 1.86739697 1.23417653 2.92741006 0.56119416 1.77036577
 0.63188065 0.77978484 0.9094028  2.84951373 1.3122982  0.09731253], Cost: 2.6811
Current parameters: [1.91321734 1.41740286 0.36774659 1.18313983 0.32439416 0.3617283
 1.51237313 2.51121451 1.13019129 1.3955932  2.28051952 2.91962826
 1.75211687 1.86739697 1.23417653 2.92741006 0.56119416 1.77036577
 0.63188065 0.77978484 0.9094028  2.84951373 1.3122982  0.09731253], Cost: 2.1042
Current parameters: [1.91321734 1.41740286 1.36774659 1.18313983 0.32439416 0.3617283
 1.51237313 2.51121451 1.13019129 1.3955932  2.28051952 2.91962826
 1.75211687 1.86739697 1.23417653 2.92741006 0.56119416 1.77036577
 0.63188065 0.77978484 0.9094028  2.84951373 1.3122982  0.09731253], Cost: 2.8901
Current parameters: [1.91321734 1.41740286 0.36774659 2.18313983 0.32439416 0.3617283
 1.51237313 2.51121451 1.13019129 1.3955932  2.28051952 2.91962826
 1.75211687 1.86739697 1.23417653 2.92741006 0.56119416 1.77036577
 0.63188065 0.77978484 0.9094028  2.84951373 1.3122982  0.09731253], Cost: 1.0431
Current parameters: [1.91321734 1.41740286 0.36774659 2.18313983 1.32439416 0.3617283
 1.51237313 2.51121451 1.13019129 1.3955932  2.28051952 2.91962826
 1.75211687 1.86739697 1.23417653 2.92741006 0.56119416 1.77036577
 0.63188065 0.77978484 0.9094028  2.84951373 1.3122982  0.09731253], Cost: 3.3294
Current parameters: [1.91321734 1.41740286 0.36774659 2.18313983 0.32439416 1.3617283
 1.51237313 2.51121451 1.13019129 1.3955932  2.28051952 2.91962826
 1.75211687 1.86739697 1.23417653 2.92741006 0.56119416 1.77036577
 0.63188065 0.77978484 0.9094028  2.84951373 1.3122982  0.09731253], Cost: 0.9955
Current parameters: [1.91321734 1.41740286 0.36774659 2.18313983 0.32439416 1.3617283
 2.51237313 2.51121451 1.13019129 1.3955932  2.28051952 2.91962826
 1.75211687 1.86739697 1.23417653 2.92741006 0.56119416 1.77036577
 0.63188065 0.77978484 0.9094028  2.84951373 1.3122982  0.09731253], Cost: 3.0845
Current parameters: [1.91321734 1.41740286 0.36774659 2.18313983 0.32439416 1.3617283
 1.51237313 3.51121451 1.13019129 1.3955932  2.28051952 2.91962826
 1.75211687 1.86739697 1.23417653 2.92741006 0.56119416 1.77036577
 0.63188065 0.77978484 0.9094028  2.84951373 1.3122982  0.09731253], Cost: 0.1067
Current parameters: [1.91321734 1.41740286 0.36774659 2.18313983 0.32439416 1.3617283
 1.51237313 3.51121451 2.13019129 1.3955932  2.28051952 2.91962826
 1.75211687 1.86739697 1.23417653 2.92741006 0.56119416 1.77036577
 0.63188065 0.77978484 0.9094028  2.84951373 1.3122982  0.09731253], Cost: 1.9252
Current parameters: [1.91321734 1.41740286 0.36774659 2.18313983 0.32439416 1.3617283
 1.51237313 3.51121451 1.13019129 2.3955932  2.28051952 2.91962826
 1.75211687 1.86739697 1.23417653 2.92741006 0.56119416 1.77036577
 0.63188065 0.77978484 0.9094028  2.84951373 1.3122982  0.09731253], Cost: 1.1986
```



```
Current parameters: [1.91321734 1.41740286 0.36774659 2.18313983 0.32439416 1.3617283
 1.51237313 3.51121451 1.13019129 1.3955932  3.28051952 2.91962826
 1.75211687 1.86739697 1.23417653 2.92741006 0.56119416 1.77036577
 0.63188065 0.77978484 0.9094028  2.84951373 1.3122982  0.09731253], Cost: 0.3284
Current parameters: [1.91321734 1.41740286 0.36774659 2.18313983 0.32439416 1.3617283
 1.51237313 3.51121451 1.13019129 1.3955932  2.28051952 3.91962826
 1.75211687 1.86739697 1.23417653 2.92741006 0.56119416 1.77036577
 0.63188065 0.77978484 0.9094028  2.84951373 1.3122982  0.09731253], Cost: 0.3905
Current parameters: [1.91321734 1.41740286 0.36774659 2.18313983 0.32439416 1.3617283
 1.51237313 3.51121451 1.13019129 1.3955932  2.28051952 2.91962826
 2.75211687 1.86739697 1.23417653 2.92741006 0.56119416 1.77036577
 0.63188065 0.77978484 0.9094028  2.84951373 1.3122982  0.09731253], Cost: 3.5214
Current parameters: [1.91321734 1.41740286 0.36774659 2.18313983 0.32439416 1.3617283
 1.51237313 3.51121451 1.13019129 1.3955932  2.28051952 2.91962826
 1.75211687 2.86739697 1.23417653 2.92741006 0.56119416 1.77036577
 0.63188065 0.77978484 0.9094028  2.84951373 1.3122982  0.09731253], Cost: 1.9254
Current parameters: [1.91321734 1.41740286 0.36774659 2.18313983 0.32439416 1.3617283
 1.51237313 3.51121451 1.13019129 1.3955932  2.28051952 2.91962826
 1.75211687 1.86739697 2.23417653 2.92741006 0.56119416 1.77036577
 0.63188065 0.77978484 0.9094028  2.84951373 1.3122982  0.09731253], Cost: 2.3878
Current parameters: [1.91321734 1.41740286 0.36774659 2.18313983 0.32439416 1.3617283
 1.51237313 3.51121451 1.13019129 1.3955932  2.28051952 2.91962826
 1.75211687 1.86739697 1.23417653 3.92741006 0.56119416 1.77036577
 0.63188065 0.77978484 0.9094028  2.84951373 1.3122982  0.09731253], Cost: 2.6861
Current parameters: [1.91321734 1.41740286 0.36774659 2.18313983 0.32439416 1.3617283
 1.51237313 3.51121451 1.13019129 1.3955932  2.28051952 2.91962826
 1.75211687 1.86739697 1.23417653 2.92741006 1.56119416 1.77036577
 0.63188065 0.77978484 0.9094028  2.84951373 1.3122982  0.09731253], Cost: 1.6099
Current parameters: [1.91321734 1.41740286 0.36774659 2.18313983 0.32439416 1.3617283
 1.51237313 3.51121451 1.13019129 1.3955932  2.28051952 2.91962826
 1.75211687 1.86739697 1.23417653 2.92741006 0.56119416 2.77036577
 0.63188065 0.77978484 0.9094028  2.84951373 1.3122982  0.09731253], Cost: 3.1017
Current parameters: [1.91321734 1.41740286 0.36774659 2.18313983 0.32439416 1.3617283
 1.51237313 3.51121451 1.13019129 1.3955932  2.28051952 2.91962826
 1.75211687 1.86739697 1.23417653 2.92741006 0.56119416 1.77036577
 1.63188065 0.77978484 0.9094028  2.84951373 1.3122982  0.09731253], Cost: 0.9464
Current parameters: [1.91321734 1.41740286 0.36774659 2.18313983 0.32439416 1.3617283
 1.51237313 3.51121451 1.13019129 1.3955932  2.28051952 2.91962826
 1.75211687 1.86739697 1.23417653 2.92741006 0.56119416 1.77036577
 0.63188065 1.77978484 0.9094028  2.84951373 1.3122982  0.09731253], Cost: 1.2226
Current parameters: [1.91321734 1.41740286 0.36774659 2.18313983 0.32439416 1.3617283
```



```
 1.51237313 3.51121451 1.13019129 1.3955932  2.28051952 2.91962826
 1.75211687 1.86739697 1.23417653 2.92741006 0.56119416 1.77036577
 0.63188065 0.77978484 1.9094028  2.84951373 1.3122982  0.09731253], Cost: 2.8716
Current parameters: [1.91321734 1.41740286 0.36774659 2.18313983 0.32439416 1.3617283
 1.51237313 3.51121451 1.13019129 1.3955932  2.28051952 2.91962826
 1.75211687 1.86739697 1.23417653 2.92741006 0.56119416 1.77036577
 0.63188065 0.77978484 0.9094028  3.84951373 1.3122982  0.09731253], Cost: 1.5728
Current parameters: [1.91321734 1.41740286 0.36774659 2.18313983 0.32439416 1.3617283
 1.51237313 3.51121451 1.13019129 1.3955932  2.28051952 2.91962826
 1.75211687 1.86739697 1.23417653 2.92741006 0.56119416 1.77036577
 0.63188065 0.77978484 0.9094028  2.84951373 2.3122982  0.09731253], Cost: 1.8403
Current parameters: [1.91321734 1.41740286 0.36774659 2.18313983 0.32439416 1.3617283
 1.51237313 3.51121451 1.13019129 1.3955932  2.28051952 2.91962826
 1.75211687 1.86739697 1.23417653 2.92741006 0.56119416 1.77036577
 0.63188065 0.77978484 0.9094028  2.84951373 1.3122982  1.09731253], Cost: 1.8373
Current parameters: [ 1.98903839  1.48478663  0.27595508  2.30706793  0.05736835  1.36728954
  1.26839136  3.61502095  0.91780529  1.26806215  2.25462827  2.88648095
  1.3532923   1.65498493  0.96775787  2.62614927  0.38562588  1.42057006
  0.53381159  0.64945872  0.58648115  2.67828206  1.10982316 -0.10481035], Cost: 2.1317
Current parameters: [2.39420328 1.41740286 0.36774659 2.18313983 0.46096843 1.3617283
 1.51237313 3.51121451 1.13019129 1.3955932  2.28051952 2.91962826
 1.75211687 1.86739697 1.23417653 2.92741006 0.56119416 1.77036577
 0.63188065 0.77978484 0.9094028  2.84951373 1.3122982  0.09731253], Cost: 0.5568
Current parameters: [1.62350697 1.43311972 0.34633678 2.2120453  1.25490853 1.36302542
 1.45546587 3.53542673 1.08065355 1.36584735 2.27448054 2.91189685
 1.65909346 1.81785315 1.172036   2.85714282 0.52024393 1.68877805
 0.60900664 0.74938707 0.83408329 2.80957498 1.26507213 0.0501686 ], Cost: 2.3909
Current parameters: [1.91151899 1.41995531 0.86635562 2.18783414 0.31579418 1.36193895
 1.50313128 3.51514663 1.12214626 1.39076241 2.27953878 2.91837267
 1.73700968 1.85935095 1.22408479 2.91599852 0.55454376 1.75711576
 0.62816586 0.77484818 0.89717076 2.84302759 1.30462859 0.08965626], Cost: 0.1531
Current parameters: [1.91321734 1.19777019 0.36774659 2.30256131 0.32439416 1.3617283
 1.51237313 3.51121451 1.13019129 1.3955932  2.28051952 2.91962826
 1.75211687 1.86739697 1.23417653 2.92741006 0.56119416 1.77036577
 0.63188065 0.77978484 0.9094028  2.84951373 1.3122982  0.09731253], Cost: 0.1399
Current parameters: [1.91057237 1.180945   0.36817923 1.74634724 0.3110008  1.36205636
 1.49798013 3.51733827 1.1176622  1.38806987 2.27899214 2.91767283
 1.72858937 1.85486634 1.21845994 2.90963806 0.55083702 1.74973059
 0.62609535 0.77209663 0.89035298 2.83941241 1.30035377 0.08538888], Cost: 1.4249
Current parameters: [1.91321734 1.41740286 0.36774659 2.18313983 0.32439416 1.61166338
 1.51807007 3.51121451 1.13019129 1.3955932  2.28051952 2.91962826
```

```
 1.75211687 1.86739697 1.23417653 2.92741006 0.56119416 1.77036577
 0.63188065 0.77978484 0.9094028  2.84951373 1.3122982  0.09731253], Cost: 0.1438
Current parameters: [1.90862596 1.43234242 0.36849761 2.20729363 0.30114474 1.34879281
 2.00197187 3.5218447  1.10844214 1.38253352 2.27786816 2.91623385
 1.71127567 1.84564514 1.20689422 2.89655978 0.54321529 1.73454533
 0.62183799 0.76643894 0.87633437 2.83197893 1.29156397 0.07661436], Cost: 0.0842
Current parameters: [1.90440725 1.44606938 0.36918767 2.22948695 0.27978239 1.34706524
 2.00618273 3.53161207 1.08845828 1.37053385 2.27543199 2.91311495
 1.94651766 1.82565884 1.18182632 2.86821351 0.52669569 1.7016323
 0.61261047 0.75417627 0.84594998 2.81586738 1.27251266 0.05759619], Cost: 1.0674
Current parameters: [1.90862596 1.43234242 0.36849761 2.20729363 0.30114474 1.34879281
 2.00197187 3.5218447  1.10844214 1.38253352 2.27786816 2.91623385
 1.71127567 1.84564514 1.20689422 2.89655978 0.54321529 1.81933506
 0.62183799 0.76643894 0.78448829 2.83197893 1.29156397 0.07661436], Cost: 0.0319
Current parameters: [1.9032733  1.44975905 0.36937315 2.23545229 0.27404042 1.34660088
 2.00731457 3.53423743 1.08308683 1.36730846 2.27477718 2.91227662
 1.66876774 1.82028673 1.17508833 2.86059433 0.5222554  1.99142306
 0.6101302  0.7508802  0.93542064 2.81153676 1.26739186 0.0524843 ], Cost: 0.8657
Current parameters: [1.93489751 1.43196195 0.36901589 2.2065939  0.20862186 1.34876141
 2.00334946 3.5218447  1.10844214 1.38253352 2.27786816 2.91623385
 1.71352756 1.84564514 1.20689422 2.97630453 0.54321529 1.82131011
 0.62183799 0.76643894 0.7863116  2.83197893 1.29156397 0.07661436], Cost: 0.1341
Current parameters: [1.85562835 1.44803843 0.36829681 2.23282627 0.44822504 1.34696346
 2.00403442 3.53251449 1.08661194 1.36942518 2.27520691 2.91282678
 1.67052984 1.82381227 1.17951025 3.07176128 0.52516941 1.79970212
 0.61175792 0.7530433  0.75953233 2.8143788  1.27075247 0.05583906], Cost: 0.0899
Current parameters: [1.90463206 1.42879295 0.37333277 2.20076567 0.31521044 1.34849987
 2.01482371 3.5218447  1.10844214 1.38253352 2.27786816 2.91623385
 1.73228393 1.84564514 1.32456044 2.91242882 0.54321529 1.8377607
 0.62183799 0.76643894 0.80149833 2.83197893 1.29156397 0.07661436], Cost: 0.0675
Current parameters: [1.90202649 1.46968658 0.35181613 2.27059223 0.26051945 1.34683092
 1.96182337 3.53906775 1.0732041  1.3613742  2.27357241 2.91073421
 1.57443326 1.81040279 1.27673322 2.83674086 0.51408586 1.7253098
 0.60556686 0.74481587 0.68665968 2.80356902 1.25797031 0.04307913], Cost: 1.2580
Current parameters: [1.90862596 1.43234242 0.36849761 2.20729363 0.30114474 1.34879281
 2.00197187 3.5218447  1.02004837 1.38253352 2.27786816 2.91623385
 1.71127567 1.93402808 1.20689422 2.89655978 0.54321529 1.81933506
 0.62183799 0.76643894 0.78448829 2.83197893 1.29156397 0.07661436], Cost: 0.0861
Current parameters: [1.89683254 1.43983127 0.37251491 2.21882819 0.31611752 1.34731319
 2.01439276 3.52900744 1.28493391 1.37373375 2.27608163 2.91394665
 1.70196302 2.01721874 1.20167163 2.90763605 0.5311009  1.82197793
```



```
 0.61507113 0.75744631 0.78234215 2.82016378 1.27759299 0.06266769], Cost: 0.1462
Current parameters: [ 1.90862596  1.43234242  0.36849761  2.20729363  0.30114474  1.34879281
  2.00197187  3.5218447   1.10844214  1.38253352  2.27786816  2.91623385
  1.71127567  1.84564514  1.20689422  2.89655978  0.54321529  1.81933506
  0.62183799  0.76643894  0.78448829  2.83197893  1.37987535 -0.01185088], Cost: 0.0273
Current parameters: [1.89598648 1.44036852 0.37280311 2.21965568 0.31719167 1.34720704
 2.01528383 3.5295213  1.11749216 1.37310246 2.27595347 2.91378257
 1.70129493 1.84940208 1.20129696 2.90843066 0.53023182 1.82216753
 0.61458568 0.75680118 0.78218818 2.81931616 1.55439753 0.16191938], Cost: 0.7815
Current parameters: [ 1.90862596  1.43234242  0.36849761  2.20729363  0.30114474  1.34879281
  2.00197187  3.5218447   1.10844214  1.38253352  2.27786816  2.91623385
  1.71127567  1.84564514  1.20689422  2.89655978  0.63049154  1.81933506
  0.62183799  0.76643894  0.78448829  2.74249232  1.37987535 -0.01185088], Cost: 0.1202
Current parameters: [ 1.89757314  1.43936099  0.37226263  2.21810384  0.31517726  1.34740611
  2.01361275  3.52855764  1.11635609  1.37428636  2.27619382  2.91409028
  1.70254783  1.84893046  1.2019996   2.90694048  0.71640397  1.82181196
  0.61549608  0.75801103  0.78247692  3.00872601  1.37278339 -0.0193226 ], Cost: 0.4533
Current parameters: [ 1.90862596  1.43234242  0.36849761  2.20729363  0.30114474  1.34879281
  2.00197187  3.5218447   1.10844214  1.29319232  2.27786816  2.91623385
  1.71127567  1.84564514  1.20689422  2.89655978  0.54321529  1.81933506
  0.62183799  0.85386405  0.78448829  2.83197893  1.37987535 -0.01185088], Cost: 0.0073
Current parameters: [ 1.9001435   1.43772881  0.37138707  2.21558991  0.31191397  1.34772859
  2.01090564  3.52699653  1.11451568  1.46641574  2.27658319  2.91458877
  1.70457751  1.84816645  1.20313785  2.90452643  0.53897434  1.82123596
  0.6169709   1.03221287  0.78294467  2.83385665  1.37443264 -0.01758504], Cost: 0.5126
Current parameters: [ 1.90862596  1.43234242  0.36849761  2.20729363  0.30114474  1.34879281
  2.00197187  3.43600304  1.10844214  1.29319232  2.27786816  2.91623385
  1.71127567  1.84564514  1.20689422  2.89655978  0.54321529  1.81933506
  0.5309743   0.85386405  0.78448829  2.83197893  1.37987535 -0.01185088], Cost: 0.0820
Current parameters: [ 1.90196158  1.43657432  0.37076776  2.21381173  0.30960576  1.34795669
  2.00899083  3.34259351  1.11321391  1.28975912  2.2768586   2.91494137
  1.70601315  1.84762605  1.20394297  2.90281891  0.53988332  1.82082853
  0.794925    0.85139967  0.78327552  2.83345419  1.3755992  -0.01635601], Cost: 0.2603
Current parameters: [ 1.90871324  1.42601808  0.36821638  2.19566228  0.30083738  1.34880143
  2.00159373  3.52200558  1.10811297  1.29299467  2.27786816  2.79194632
  1.71065755  1.84531594  1.20648131  2.89609287  0.54294318  1.81879293
  0.621686    0.85366207  0.78398781  2.83171355  1.37956154 -0.01216415], Cost: 0.0041
Current parameters: [ 1.89506934  1.3109416   0.36789973  1.98110399  0.31427966  1.34704365
  2.01031394  3.53068949  1.11261826  1.28117702  2.27554036  2.81793412
  1.68632245  1.84338532  1.19152606  2.90130878  0.5298894   1.81153552
  0.62211264  0.8439928   0.77131255  2.82987681  1.36350754 -0.02873534], Cost: 0.2471
```



```
Current parameters: [ 1.93746413  1.42601808  0.36821638  2.19566228  0.30900109  1.34880143
  2.00159373  3.52200558  1.10811297  1.29299467  2.15649378  2.79194632
  1.71065755  1.84531594  1.20648131  2.89609287  0.54294318  1.81879293
  0.621686    0.85366207  0.78398781  2.83171355  1.37956154 -0.01216415], Cost: 0.0450
Current parameters: [ 2.13918867  1.4248586   0.36994836  2.19244621  0.37489809  1.34809168
  2.00732052  3.52417377  1.11195173  1.28990404  2.33875585  2.79220287
  1.7056817   1.84676383  1.20364069  2.90110157  0.53988295  1.81966216
  0.62284416  0.85139811  0.78257023  2.83277656  1.37566091 -0.01626052], Cost: 0.1274
Current parameters: [ 1.90909062  1.425519    0.24361799  2.19474441  0.30274834  1.34875396
  2.00367629  3.52113185  1.10990062  1.29406809  2.27808608  2.79222532
  1.71722438  1.8471038   1.20872375  2.89862858  0.54442093  1.82173715
  0.62251145  0.85475902  0.78670584  2.8331548   1.38126577 -0.01046288], Cost: 0.0145
Current parameters: [ 1.89560051  1.43328535  0.37731918  2.20411951  0.31586179  1.34605354
  2.00704826  3.54165333  1.10539479  1.2669228   2.28171451  2.7899704
  1.93981935  1.83176592  1.1683939   2.89026047  0.5124615   1.78961626
  0.61764802  0.83107002  0.74699709  2.82031859  1.34272613 -0.04985283], Cost: 0.8874
Current parameters: [ 1.90883754  1.42648858  0.36796141  2.1965276   0.29960735  1.22392327
  1.99768077  3.52221924  1.10767584  1.29273218  2.27781487  2.7918781
  1.70875526  1.84487875  1.20778131  2.89477844  0.54258183  1.81726673
  0.62148416  0.85339383  0.78257887  2.83136112  1.37914481 -0.01258015], Cost: 0.0373
Current parameters: [ 1.90568136  1.45114795  0.35342468  2.23584231  0.26395311  1.36309839
  1.96289139  3.54866431  1.09967169  1.2578647   2.28197778  2.78872028
  1.57556679  1.82355068  1.27419562  2.83624708  0.50123605  1.72174752
  0.61436528  0.82274525  0.68202959  2.81358751  1.32939336 -0.06337341], Cost: 1.0121
Current parameters: [ 1.90861106  1.5360493   0.36823309  2.13655299  0.30031999  1.34876863
  2.00129922  3.52224215  1.10762896  1.29270404  2.27780915  2.79187078
  1.7098497   1.84483187  1.20587417  2.89540633  0.54254308  1.82214959
  0.62146251  0.85336507  0.78708659  2.83132333  1.37910012 -0.01262476], Cost: 0.0186
Current parameters: [ 1.89573095  1.42633897  0.36981916  2.22028137  0.30344874  1.35056634
  2.006592    3.54369508  1.0972787   1.26461762  2.28028609  2.78886678
  1.6575233   1.82478708  1.16634726  2.87726905  0.50871944  1.986666
  0.61419286  0.82828858  0.92996462  2.81474233  1.33858735 -0.05388801], Cost: 1.1144
Current parameters: [ 1.90753879  1.42979204  0.36843527  2.20175931  0.29496112  1.34555871
  2.12407015  3.52470563  1.10264141  1.28967754  2.27719471  2.79108415
  1.70042388  1.83984371  1.19966657  2.88831335  0.53837658  1.80986832
  0.61913519  0.85027223  0.77574886  2.82725975  1.37572685 -0.01599217], Cost: 0.0104
Current parameters: [1.89881612 1.42501984 0.37309731 2.19346583 0.31961786 1.35120795
 2.00915078 3.53504056 1.11773973 1.27548466 2.28138368 2.79110898
 1.70131034 1.84656449 1.20237806 2.90421859 0.52302345 1.81159434
 0.62060729 0.83890238 0.7695631  2.82646912 1.55370399 0.16096575], Cost: 0.5789
Current parameters: [1.90436587 1.42681955 0.36679785 2.19718775 0.31675828 1.34891578
```



```
 1.99778323 3.52184099 1.12664262 1.29319688 2.27790921 2.79199888
 1.70510929 1.7989015  1.16564817 2.84449678 0.58936043 1.84488
 0.6218415  0.85386871 0.74557317 2.87698428 1.34886101 0.02207465], Cost: 0.0634
Current parameters: [ 1.90307051  1.42729048  0.37492653  2.19680711  0.29837112  1.34967855
  2.01914456  3.53404442  1.09489195  1.27717018  2.2795436   2.79040081
  1.70735174  1.90678058  1.25283373  2.97200745  0.6871125   1.78151683
  0.61808043  0.83965751  0.82164322  2.98212742  1.41206219 -0.06061615], Cost: 0.6088
Current parameters: [ 1.9073311   1.42956335  0.36881548  2.20191168  0.30249115  1.34861906
  2.00324306  3.49748618  1.10942075  1.23874148  2.27765199  2.8434739
  1.70977011  1.84605449  1.20590029  2.89788866  0.54283598  1.81931917
  0.71652528  0.87271398  0.78392571  2.831609    1.37845933 -0.01269559], Cost: 0.1075
Current parameters: [ 1.90364426  1.42098934  0.37057111  2.18612882  0.30961764  1.34985117
  2.00751282  3.32559478  1.11226043  1.38892899  2.27947545  2.69504408
  1.7052731   1.84722132  1.20506601  2.90168761  0.53524904  1.81406262
  0.67930575  0.81461019  0.77760002  2.83105142  1.3780991  -0.01576431], Cost: 0.1335
Current parameters: [ 1.90540284  1.4266439   0.36992998  2.19665528  0.30677641  1.34952784
  2.00565027  3.53739213  1.1109814   1.36519316  2.27898168  2.80785825
  1.70757012  1.84674211  1.20589134  2.89997441  0.53795087  1.81591345
  0.63326208  0.95179753  0.780034    2.83146731  1.37893604 -0.01430716], Cost: 0.1630
Current parameters: [ 1.9220695   1.42214937  0.36860576  2.18931571  0.26296064  1.34920358
  2.00197241  3.51954627  1.11101602  1.29599243  2.27848435  2.79274747
  1.72230281  1.85730901  1.21905117  2.85710984  0.54711595  1.82095613
  0.62402636  0.85674317  0.79473176  2.83578325  1.38887807 -0.01194503], Cost: 0.0108
Current parameters: [ 1.90210577  1.42867871  0.37255885  2.1993704   0.30958901  1.34986121
  2.00347845  3.52822363  1.10684271  1.28792785  2.27977131  2.78631671
  1.82415549  1.83908826  1.1894915   2.89565678  0.52394357  1.7971095
  0.61631716  0.84365736  0.759378    2.82206848  1.3642525  -0.02818295], Cost: 0.2279
Current parameters: [ 1.91153383  1.4167712   0.36751046  2.17899412  0.2949158   1.34902037
  1.99991726  3.52072019  1.06301535  1.29426171  2.278138    2.79431283
  1.71239054  1.80916273  1.21238301  2.89799629  0.54529338  1.81473269
  0.6231247   0.85519006  0.78885199  2.8340057   1.38626243 -0.01441653], Cost: 0.0122
Current parameters: [ 1.90073254  1.37122062  0.36872648  2.09352164  0.31059439  1.34851333
  2.00686244  3.52563867  1.13542185  1.28819044  2.27756146  2.80337418
  1.70008992  1.86458369  1.19538537  2.89888597  0.53472646  1.81450324
  0.61925708  0.8480715   0.77234389  2.82656157  1.3671918  -0.01830773], Cost: 0.0454
Current parameters: [ 1.92097103e+00  1.42601056e+00  3.67975533e-01  2.19572206e+00
  2.58026072e-01  1.34899057e+00  2.00075105e+00  3.52195850e+00
  1.11821740e+00  1.29351990e+00  2.27810884e+00  2.79147316e+00
  1.70997820e+00  1.83549828e+00  1.19332245e+00  2.93007659e+00
  5.42230099e-01  1.82652398e+00  6.21571620e-01  8.53631108e-01
  7.71966210e-01  2.83168641e+00  1.36989116e+00 -2.34084563e-03], Cost: 0.0209
```

```
Current parameters: [ 1.90957215  1.42678738  0.36717371  2.19707527  0.2977907   1.34886358
  1.99882363  3.52201108  1.10172499  1.29298552  2.27786669  2.7919468
  1.70611086  1.85173394  1.18103315  2.89270628  0.54304148  1.80866455
  0.62168254  0.85365451  0.78696146  2.83180942  1.3859506  -0.01858105], Cost: 0.0064
Current parameters: [ 1.90681536  1.42524297  0.37018747  2.1938461   0.30060731  1.3490904
  2.00640726  3.52395102  1.10588097  1.29183114  2.27841071  2.78993298
  1.71446496  1.86101885  1.21744678  2.91624881  0.57836554  1.80847981
  0.61979029  0.85054022  0.79178826  2.86936989  1.38653975 -0.02510716], Cost: 0.0388
Current parameters: [ 1.90867711  1.42617115  0.36811649  2.19590765  0.30060942  1.34885279
  2.00135958  3.52213394  1.12606954  1.29277612  2.27784427  2.79196177
  1.70984624  1.8271796   1.2068894   2.89573248  0.54276044  1.80955259
  0.62160848  0.85348419  0.79275226  2.83153532  1.38823505 -0.02146154], Cost: 0.0122
Current parameters: [ 1.90757477  1.43026239  0.36499045  2.20251578  0.2917969   1.35246674
  1.99251314  3.52732747  1.10382918  1.28814963  2.27903537  2.78815991
  1.6831379   1.84689992  1.22567243  2.88610851  0.53564422  1.78887912
  0.61711325  0.84538291  0.75271093  2.8309046   1.36691964 -0.02489154], Cost: 0.0834
Current parameters: [ 1.90873036  1.42607186  0.36785058  2.19720447  0.29898724  1.34879864
  2.00102442  3.52196629  1.10656407  1.2932993   2.27774777  2.79172112
  1.70879777  1.84410305  1.20477395  2.89427305  0.5428649   1.82850282
  0.62153884  0.85378185  0.79309406  2.8316372   1.35987845 -0.03196079], Cost: 0.0353
Current parameters: [1.89928989 1.42444874 0.36972531 2.19933414 0.30248067 1.35240654
 2.00257854 3.53274469 1.10537274 1.28508044 2.28081241 2.78234086
 1.69115884 1.84896574 1.20499419 2.89758219 0.52838385 1.84667893
 0.61188776 0.8365197  0.80586825 2.83314471 1.40713567 0.00968992], Cost: 0.0603
Current parameters: [ 1.90908092  1.42642265  0.36803689  2.19659478  0.29987591  1.34887252
  2.00097923  3.54173613  1.10764967  1.28376191  2.27789058  2.80115211
  1.7112131   1.84523946  1.20672542  2.89565885  0.55656569  1.81929585
  0.6157329   0.85741289  0.78460792  2.81836688  1.3798951  -0.01199294], Cost: 0.0059
Current parameters: [ 1.90628196  1.42478286  0.3688627   2.19445585  0.30235857  1.34995762
  2.00246094  3.49345834  1.10815245  1.30665144  2.27899352  2.77434868
  1.70600319  1.84737758  1.2073372   2.89778553  0.57563887  1.82044329
  0.6285222   0.84372114  0.78430675  2.79573511  1.38477838 -0.00876195], Cost: 0.0301
Current parameters: [1.89309485e+00 1.42609295e+00 3.68099000e-01 2.19587333e+00
 2.95819134e-01 1.34884947e+00 2.00120593e+00 3.52192298e+00
 1.10785297e+00 1.29321956e+00 2.27383098e+00 2.79185825e+00
 1.71099397e+00 1.84521775e+00 1.20667333e+00 2.89575361e+00
 5.43150141e-01 1.80617255e+00 6.21675326e-01 8.53799685e-01
 7.97690070e-01 2.83164145e+00 1.36674240e+00 1.21866562e-03], Cost: 0.0086
Current parameters: [1.95679119 1.42536921 0.36883473 2.19512553 0.31673126 1.34948151
 2.00278032 3.52302002 1.1085008  1.29212836 2.29137866 2.78975896
 1.70708106 1.8469077  1.20668049 2.89784855 0.54188059 1.80400915
```



```
 0.62032215 0.85039842 0.80244363 2.83135952 1.36525104 0.00664419], Cost: 0.0047
Current parameters: [ 1.90872542  1.42513543  0.36817713  2.19403897  0.30079448  1.34880263
  2.00154096  3.51694451  1.10806703  1.27520751  2.27786816  2.77460028
  1.71057128  1.84526999  1.20642368  2.89602771  0.5429052   1.81871726
  0.61628386  0.87101256  0.78391796  2.83167651  1.37951774 -0.01220787], Cost: 0.0047
Current parameters: [ 1.90919925  1.42759711  0.36850804  2.19926021  0.30144705  1.34945058
  2.00171508  3.48856379  1.10789549  1.28453284  2.27885509  2.82757708
  1.70882838  1.8464362   1.2072234   2.89680645  0.54268288  1.81976933
  0.58464563  0.85967484  0.78526133  2.83134166  1.3821404  -0.00973117], Cost: 0.0119
Current parameters: [ 1.91644039  1.42600618  0.36820103  2.19550239  0.30290406  1.34882389
  2.00153458  3.5212928   1.1081593   1.29336159  2.24774751  2.79209823
  1.71109181  1.84546905  1.20679723  2.89620969  0.54377198  1.81741671
  0.62159375  0.85393007  0.78310377  2.83137841  1.37881439 -0.01260287], Cost: 0.0079
Current parameters: [1.90418436 1.4244439  0.36975457 2.19933376 0.30397287 1.35243158
 2.00264002 3.53295492 1.10540628 1.28499948 2.27462041 2.78284083
 1.69105055 1.84904871 1.2050222  2.89768201 0.52702352 1.84676807
 0.61155201 0.83637248 0.8056537  2.83453744 1.40730429 0.00953785], Cost: 0.0629
Current parameters: [ 1.90843791  1.4263949   0.36976712  2.19708094  0.30475391  1.35002319
  2.00227554  3.52306686  1.10721171  1.29040437  2.27919978  2.78870823
  1.73828481  1.84563976  1.20278573  2.89675762  0.53713556  1.81472431
  0.61719392  0.84671869  0.77926571  2.82828101  1.38086809 -0.01082416], Cost: 0.0153
Current parameters: [ 1.90880266  1.42555933  0.3681707   2.19492416  0.30177516  1.34920605
  1.98631782  3.52166777  1.10903439  1.29340969  2.27795241  2.79205419
  1.71185759  1.84539207  1.206797    2.8963884   0.54411511  1.82024201
  0.62200514  0.85408618  0.78451204  2.83285653  1.37963865 -0.01124256], Cost: 0.0043
Current parameters: [ 1.90792472e+00  1.42619781e+00  3.68107857e-01  2.19642122e+00
  3.05366017e-01  1.34920295e+00  2.00396517e+00  3.52203529e+00
  1.11242538e+00  1.29257407e+00  2.27815021e+00  2.79107977e+00
  1.71024805e+00  1.83396629e+00  1.19595235e+00  2.88315079e+00
  5.53938400e-01  1.82526059e+00  6.20238843e-01  8.51898519e-01
  7.74208546e-01  2.84255078e+00  1.37311662e+00 -2.18731462e-03], Cost: 0.0069
Current parameters: [ 1.90844886  1.42458726  0.38342217  2.19297476  0.30086877  1.3487791
  2.00148573  3.52220507  1.10863759  1.29273575  2.27783366  2.79225869
  1.70956733  1.84562211  1.20590638  2.89585774  0.54253676  1.81831524
  0.62157285  0.85342737  0.78333816  2.83139746  1.37901652 -0.01254132], Cost: 0.0029
Current parameters: [ 1.90675787  1.41108905  0.37689306  2.16800078  0.30397922  1.34889364
  2.00304328  3.52299145  1.11533082  1.29133472  2.27800858  2.79468736
  1.70830981  1.85054022  1.20303071  2.89656475  0.54039096  1.81766602
  0.62035744  0.85118496  0.78049581  2.83011672  1.37702748 -0.01259688], Cost: 0.0018
Current parameters: [ 1.9066785   1.41079517  0.37694605  2.16740513  0.30420622  1.34888188
  2.00314262  3.52656192  1.11538783  1.30179754  2.2781057   2.79042576
```



```
  1.70819275  1.85055922  1.20305184  2.89663872  0.54006631  1.81741809
  0.61132379  0.85580686  0.78023657  2.83011066  1.37709909 -0.01268627], Cost: 0.0025
Current parameters: [ 1.90671701  1.41009827  0.3780193   2.16759675  0.30939394  1.35063914
  2.0052432   3.52249538  1.1167063   1.2981792   2.27946941  2.80515495
  1.71218058  1.85218146  1.20095057  2.89748111  0.53782899  1.8182346
  0.63506514  0.87280469  0.77844299  2.82937382  1.38124553 -0.00625375], Cost: 0.0092
Current parameters: [ 1.90670308  1.41127115  0.37675882  2.16821377  0.30361717  1.36428533
  2.00305457  3.5232323   1.11518859  1.29112401  2.27807451  2.79450641
  1.70714895  1.85060346  1.20382458  2.89621207  0.54005594  1.816316
  0.6201531   0.85080181  0.77910804  2.83010859  1.37646518 -0.0132087 ], Cost: 0.0021
Current parameters: [ 1.90716382  1.41187082  0.37595912  2.17013085  0.30336245  1.34385221
  1.9991334   3.52561665  1.11400807  1.28947517  2.28030283  2.7949763
  1.69798671  1.85226973  1.21154113  2.89071622  0.53392827  1.80136068
  0.62029174  0.84998827  0.76096203  2.82864835  1.37560621 -0.01167733], Cost: 0.0329
Current parameters: [ 1.90662697  1.39737724  0.37692265  2.17542521  0.30399196  1.34898071
  2.0030987   3.523206    1.11533547  1.29119698  2.27788396  2.79447575
  1.70795917  1.85062051  1.20305133  2.89671329  0.54017401  1.81793525
  0.62016095  0.85083412  0.78073443  2.83026458  1.37753152 -0.01230609], Cost: 0.0029
Current parameters: [1.90424208e+00 1.41307788e+00 3.78315900e-01 2.16641961e+00
 3.07296704e-01 1.35101655e+00 2.00425848e+00 3.52870864e+00
 1.11462277e+00 1.28779677e+00 2.27675455e+00 2.79176244e+00
 1.70107603e+00 1.85271464e+00 1.20103812e+00 2.89709320e+00
 5.31269045e-01 1.83209131e+00 6.17384739e-01 8.45114912e-01
 7.90323722e-01 2.83139933e+00 1.39199899e+00 3.74884045e-04], Cost: 0.0157
Current parameters: [ 1.90685802  1.41119311  0.37711109  2.16789295  0.30381912  1.34872892
  2.00369765  3.52849127  1.11489509  1.28865588  2.27789472  2.7975581
  1.70983586  1.85293346  1.20474714  2.89958669  0.54021466  1.81546817
  0.61881063  0.85247567  0.78140791  2.84287589  1.37770087 -0.01473577], Cost: 0.0026
Current parameters: [ 1.90551772  1.41037162  0.37846944  2.1655398   0.30683544  1.34979209
  2.00585501  3.51405959  1.11467373  1.29577755  2.27960111  2.78849698
  1.70926203  1.85842672  1.20688101  2.90484088  0.56399886  1.81508308
  0.62310771  0.84744719  0.78261616  2.83179265  1.38529673 -0.01179401], Cost: 0.0093
Current parameters: [ 1.90744702  1.41317157  0.37702619  2.17176267  0.30068785  1.34886477
  2.00333015  3.52327574  1.12658469  1.29110624  2.27797382  2.79410309
  1.70784733  1.85762903  1.20028181  2.89979959  0.53978487  1.8194158
  0.62002116  0.85083764  0.77810325  2.82959774  1.37447466 -0.01102889], Cost: 0.0005
Current parameters: [ 1.91373865  1.41115616  0.37706469  2.16755655  0.28093502  1.3491805
  2.00296594  3.52274853  1.1215026   1.29182903  2.27858988  2.79444773
  1.70841952  1.84617278  1.19548659  2.91617973  0.54026801  1.82216988
  0.62081124  0.85136994  0.77307656  2.83008518  1.37359429 -0.00471956], Cost: 0.0097
```



```
   Return from subroutine COBYLA because the MAXFUN limit has been reached.

   NFVALS =  100   F = 5.109154E-04    MAXCV = 0.000000E+00
   X = 1.907447E+00   1.413172E+00   3.770262E-01   2.171763E+00   3.006879E-01
       1.348865E+00   2.003330E+00   3.523276E+00   1.126585E+00   1.291106E+00
       2.277974E+00   2.794103E+00   1.707847E+00   1.857629E+00   1.200282E+00
       2.899800E+00   5.397849E-01   1.819416E+00   6.200212E-01   8.508376E-01
       7.781033E-01   2.829598E+00   1.374475E+00  -1.102889E-02
Current parameters: [ 1.90744702  1.41317157  0.37702619  2.17176267  0.30068785  1.34886477
  2.00333015  3.52327574  1.12658469  1.29110624  2.27797382  2.79410309
  1.70784733  1.85762903  1.20028181  2.89979959  0.53978487  1.8194158
  0.62002116  0.85083764  0.77810325  2.82959774  1.37447466 -0.01102889], Cost: 0.0005
```

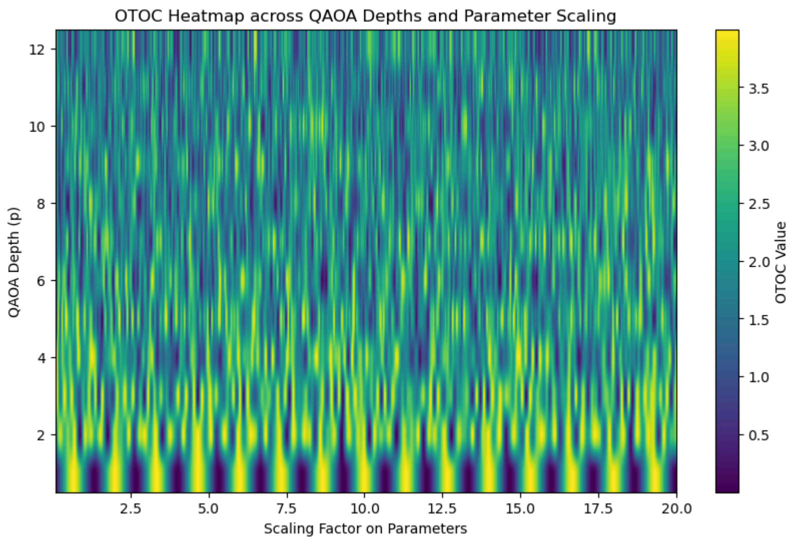


```
Run 3/30 for heatmap generation...
Simulating for QAOA depth p = 1
Current parameters: [3.13940584 2.71927987], Cost: 2.0043
Current parameters: [3.13940584 3.71927987], Cost: 1.9968
Current parameters: [4.13937019 3.7277242 ], Cost: 2.6517
Current parameters: [2.63943925 3.72505974], Cost: 1.3902
Current parameters: [2.13944899 3.72818077], Cost: 1.3520
Current parameters: [2.14100951 3.9781759 ], Cost: 2.1848
Current parameters: [2.12487418 3.22839324], Cost: 1.6955
Current parameters: [2.11346828 3.97682711], Cost: 2.1751
Current parameters: [2.13579677 3.60323414], Cost: 1.1298
Current parameters: [2.12965197 3.47838526], Cost: 1.1233
Current parameters: [2.06722753 3.48145766], Cost: 1.1810
Current parameters: [2.25396148 3.46526481], Cost: 1.0579
Current parameters: [2.37759001 3.44679898], Cost: 1.0615
Current parameters: [2.31577574 3.4560319 ], Cost: 1.0517
Current parameters: [2.31904   3.3936172], Cost: 1.1565
Current parameters: [2.32853645 3.51721535], Cost: 1.0039
Current parameters: [2.3456969  3.57731334], Cost: 1.0150
Current parameters: [2.33711668 3.54726434], Cost: 1.0021
Current parameters: [2.35214118 3.54297423], Cost: 1.0006
Current parameters: [2.38219439 3.55153966], Cost: 1.0037
Current parameters: [2.35197476 3.52735012], Cost: 1.0004
Current parameters: [2.35968793 3.52610828], Cost: 1.0006
Current parameters: [2.34548373 3.5230025 ], Cost: 1.0012
Current parameters: [2.35220128 3.53124979], Cost: 1.0001
Current parameters: [2.35196243 3.53514874], Cost: 1.0000
Current parameters: [2.34818044 3.53612612], Cost: 1.0002
Current parameters: [2.3554086  3.53698796], Cost: 1.0001
Current parameters: [2.35368551 3.53606835], Cost: 1.0000
Current parameters: [2.35332709 3.53697675], Cost: 1.0001
Current parameters: [2.35418984 3.53523208], Cost: 1.0000
Current parameters: [2.35472102 3.53441262], Cost: 1.0000
Current parameters: [2.35562022 3.53403168], Cost: 1.0000
Current parameters: [2.35545018 3.53307004], Cost: 1.0000
Current parameters: [2.35591497 3.53442096], Cost: 1.0000
Current parameters: [2.35640139 3.53437835], Cost: 1.0000
Current parameters: [2.35651403 3.53485346], Cost: 1.0000
Current parameters: [2.35639342 3.53413434], Cost: 1.0000
Current parameters: [2.3564558  3.53446225], Cost: 1.0000
Current parameters: [2.35635944 3.53440556], Cost: 1.0000
```



```
Current parameters: [2.35635658 3.53428895], Cost: 1.0000
Current parameters: [2.35632625 3.53419366], Cost: 1.0000
Current parameters: [2.35632625 3.53419366], Cost: 1.0000

   Normal return from subroutine COBYLA

   NFVALS =   42   F = 1.000000E+00    MAXCV = 0.000000E+00
   X = 2.356326E+00   3.534194E+00
Simulating for QAOA depth p = 2
Current parameters: [3.08501506e+00 2.57937387e+00 1.25140910e-03 2.05991586e+00], Cost: 1.1244
Current parameters: [3.08501506e+00 3.57937387e+00 1.25140910e-03 2.05991586e+00], Cost: 2.6400
Current parameters: [3.08501506 2.57937387 1.00125141 2.05991586], Cost: 1.1988
Current parameters: [3.08501506e+00 2.57937387e+00 1.25140910e-03 3.05991586e+00], Cost: 2.3027
Current parameters: [ 3.53186505  1.8736333  -0.03340287  1.51123161], Cost: 2.1378
Current parameters: [ 3.30844005  2.22650359 -0.01607573  1.78557373], Cost: 1.2272
Current parameters: [ 2.85670529e+00  2.49902153e+00 -2.69416618e-03  1.99744522e+00], Cost: 1.0514
Current parameters: [ 2.85670529e+00  2.57574449e+00 -2.69416618e-03  1.89876094e+00], Cost: 1.0449
Current parameters: [ 2.61835374  2.63086017 -0.05409282  1.89576407], Cost: 1.2742
Current parameters: [ 2.87551111  2.54604317 -0.12040548  1.87566939], Cost: 1.2341
Current parameters: [2.88337587 2.46972697 0.20379245 1.80981016], Cost: 0.9435
Current parameters: [2.77229796 2.58158592 0.38185604 1.88689861], Cost: 0.8291
Current parameters: [2.6421949  2.57683937 0.59284844 1.85476025], Cost: 1.0396
Current parameters: [2.87577391 2.62802542 0.42183164 1.92100215], Cost: 1.0031
Current parameters: [2.77144137 2.62313621 0.37522429 1.84069136], Cost: 0.8620
Current parameters: [2.70052469 2.61397383 0.31155754 1.95385189], Cost: 0.8672
Current parameters: [2.77152185 2.53170655 0.35175409 1.86428074], Cost: 0.7654
Current parameters: [2.73501221 2.4877109  0.36948152 1.84629692], Cost: 0.7142
Current parameters: [2.73245469 2.42722367 0.36171442 1.83285535], Cost: 0.6885
Current parameters: [2.71177536 2.43255293 0.34476843 1.77661437], Cost: 0.7484
Current parameters: [2.71453967 2.42785013 0.33981104 1.84610163], Cost: 0.6830
Current parameters: [2.69031965 2.39948843 0.37208798 1.88448722], Cost: 0.6232
Current parameters: [2.69344436 2.36364478 0.38472778 1.93400443], Cost: 0.6024
Current parameters: [2.64247854 2.34236094 0.41398024 1.93383914], Cost: 0.5338
Current parameters: [2.59721295 2.35148594 0.45129089 1.95338206], Cost: 0.5021
Current parameters: [2.54876142 2.31385237 0.43981601 1.95010464], Cost: 0.4376
Current parameters: [2.51754614 2.26778402 0.4623839  1.93277681], Cost: 0.3536
Current parameters: [2.48698919 2.22193037 0.48558069 1.91455938], Cost: 0.2843
Current parameters: [2.4414503  2.17931977 0.48435341 1.91064915], Cost: 0.2377
Current parameters: [2.40757848 2.17073676 0.53476803 1.89866383], Cost: 0.1965
Current parameters: [2.4181506  2.12238848 0.5646845  1.92236887], Cost: 0.1368
Current parameters: [2.41879699 2.06927249 0.5914095  1.90312573], Cost: 0.1435
```



```
Current parameters: [2.39476893 2.11136345 0.54895173 1.93016559], Cost: 0.1116
Current parameters: [2.36879364 2.10550798 0.56841259 1.98325535], Cost: 0.0660
Current parameters: [2.38907959 2.07791816 0.53618743 2.02442659], Cost: 0.0681
Current parameters: [2.31126108 2.10392246 0.56881135 2.00761889], Cost: 0.0410
Current parameters: [2.30240778 2.14063978 0.5921403  2.05161283], Cost: 0.1075
Current parameters: [2.3180035  2.08963994 0.59575738 2.00660706], Cost: 0.0526
Current parameters: [2.27538073 2.06136119 0.54104504 2.00158393], Cost: 0.0156
Current parameters: [2.26631843 2.06311962 0.52344101 1.94232914], Cost: 0.0765
Current parameters: [2.29593737 2.04756598 0.52510019 2.01204533], Cost: 0.0250
Current parameters: [2.27146889 2.07112566 0.53024209 2.00568142], Cost: 0.0232
Current parameters: [2.26025285 2.04887208 0.55675825 2.02015345], Cost: 0.0040
Current parameters: [2.24397383 2.02924554 0.57082281 2.00881576], Cost: 0.0027
Current parameters: [2.24121015 2.04050796 0.58081528 2.0056835 ], Cost: 0.0084
Current parameters: [2.26037631 2.01030577 0.58010975 2.02501943], Cost: 0.0069
Current parameters: [2.2333006  2.02663974 0.56276923 2.01646903], Cost: 0.0005
Current parameters: [2.23292453 2.0204373  0.55051936 2.00902125], Cost: 0.0036
Current parameters: [2.22980076 2.02148826 0.56688543 2.01416567], Cost: 0.0013
Current parameters: [2.23945509 2.02036953 0.56329329 2.0293792 ], Cost: 0.0007
Current parameters: [2.22937603 2.03197017 0.56377867 2.02049403], Cost: 0.0007
Current parameters: [2.23284166 2.02649091 0.55889294 2.01648804], Cost: 0.0005
Current parameters: [2.2324806  2.02747158 0.55888994 2.01483801], Cost: 0.0007
Current parameters: [2.23580166 2.02623771 0.55836239 2.01896828], Cost: 0.0002
Current parameters: [2.23607031 2.02349161 0.55817535 2.02172702], Cost: 0.0000
Current parameters: [2.23922096 2.02282327 0.55734966 2.02377732], Cost: 0.0001
Current parameters: [2.23658047 2.02221685 0.55781969 2.0203843 ], Cost: 0.0001
Current parameters: [2.23392669 2.02336183 0.55553984 2.02365079], Cost: 0.0002
Current parameters: [2.23584188 2.02311178 0.55990623 2.02251588], Cost: 0.0000
Current parameters: [2.23634049 2.02472737 0.56088207 2.02245487], Cost: 0.0000
Current parameters: [2.23502219 2.02325773 0.56005287 2.02202704], Cost: 0.0000
Current parameters: [2.23524336 2.02287174 0.55953583 2.02432188], Cost: 0.0000
Current parameters: [2.23619862 2.02225759 0.56021728 2.02252081], Cost: 0.0000
Current parameters: [2.23609685 2.02339363 0.56017699 2.02265963], Cost: 0.0000
Current parameters: [2.235963   2.02314624 0.55986521 2.02231078], Cost: 0.0000
Current parameters: [2.23595985 2.02311014 0.55948486 2.02273254], Cost: 0.0000
Current parameters: [2.23588461 2.02312616 0.55903682 2.02291078], Cost: 0.0000
Current parameters: [2.23587424 2.02288696 0.55901345 2.02286914], Cost: 0.0000
Current parameters: [2.23628065 2.02313186 0.55907222 2.02319412], Cost: 0.0000
Current parameters: [2.2365786  2.02322675 0.55889031 2.02286617], Cost: 0.0000
Current parameters: [2.23638372 2.02332654 0.55918771 2.0236143 ], Cost: 0.0000
Current parameters: [2.23633805 2.02290527 0.55907215 2.02326459], Cost: 0.0000
Current parameters: [2.23625086 2.02313569 0.55895966 2.02323059], Cost: 0.0000
```

```
Current parameters: [2.23614298 2.02311088 0.55926523 2.02313972], Cost: 0.0000
Current parameters: [2.23632537 2.02317003 0.5591207  2.02325887], Cost: 0.0000
Current parameters: [2.23640742 2.02319356 0.55916168 2.02322671], Cost: 0.0000
Current parameters: [2.23640686 2.02321227 0.55925968 2.02323346], Cost: 0.0000
Current parameters: [2.23639315 2.02325823 0.55925163 2.02322186], Cost: 0.0000
Current parameters: [2.23648819 2.02324099 0.55925291 2.0232836 ], Cost: 0.0000
Current parameters: [2.23656239 2.02323697 0.55926128 2.02321721], Cost: 0.0000
Current parameters: [2.23664951 2.02323314 0.55927047 2.02316914], Cost: 0.0000
Current parameters: [2.23674582 2.02324752 0.5592716  2.02319186], Cost: 0.0000
Current parameters: [2.23676816 2.02316918 0.55932961 2.02319273], Cost: 0.0000
Current parameters: [2.23675007 2.02321871 0.55923102 2.02319411], Cost: 0.0000
Current parameters: [2.23678922 2.02322246 0.55916329 2.02313194], Cost: 0.0000
Current parameters: [2.23678922 2.02322246 0.55916329 2.02313194], Cost: 0.0000

   Normal return from subroutine COBYLA

   NFVALS =   86   F = 1.821149E-07    MAXCV = 0.000000E+00
   X = 2.236789E+00   2.023222E+00   5.591633E-01   2.023132E+00
Simulating for QAOA depth p = 3
Current parameters: [1.89038361 1.88423529 2.56478626 0.98786373 2.43310414 0.27120321], Cost: 3.9099
Current parameters: [0.89038361 2.88423529 2.56478626 0.98786373 2.43310414 0.27120321], Cost: 2.9285
Current parameters: [0.89038361 1.88423529 3.56478626 0.98786373 2.43310414 0.27120321], Cost: 1.2953
Current parameters: [0.89038361 1.88423529 3.56478626 1.98786373 2.43310414 0.27120321], Cost: 3.0752
Current parameters: [0.89038361 1.88423529 3.56478626 0.98786373 3.43310414 0.27120321], Cost: 1.9350
Current parameters: [0.89038361 1.88423529 3.56478626 0.98786373 2.43310414 1.27120321], Cost: 1.7146
Current parameters: [0.31444976 1.66137915 3.92943925 0.34756954 2.20300839 0.12039594], Cost: 2.7499
Current parameters: [1.35539881 1.83414592 3.64674602 0.84395057 2.38138759 0.23730762], Cost: 1.4608
Current parameters: [0.90028638 2.37774573 3.60172892 0.9229961  2.40979335 0.25592507], Cost: 2.7090
Current parameters: [0.79835215 1.69256469 3.62575292 0.88081236 2.39463422 0.24598961], Cost: 1.2236
Current parameters: [0.79835215 1.69256469 3.73437304 0.94267244 2.39463422 0.24598961], Cost: 1.2029
Current parameters: [0.8235505  1.54421711 3.64717045 1.11611538 2.35566174 0.22044662], Cost: 1.0230
Current parameters: [0.83605545 1.47059808 3.64361156 1.13245039 2.5933247  0.20777064], Cost: 0.4761
Current parameters: [0.84732012 1.40428074 3.64040564 1.14716527 2.64630641 0.44216614], Cost: 0.4713
Current parameters: [0.73246095 1.43098203 3.62118126 1.18092136 2.65843698 0.45011665], Cost: 0.5410
Current parameters: [0.90857696 1.28881121 3.64131371 1.18041719 2.84117767 0.36258559], Cost: 0.7010
Current parameters: [0.8014975  1.31099278 3.66868832 1.09750372 2.60879049 0.42995882], Cost: 0.6180
Current parameters: [0.91673537 1.36123062 3.54696938 1.32479073 2.52162316 0.44324293], Cost: 1.3570
Current parameters: [0.84874044 1.43017691 3.75764651 1.18122876 2.64906817 0.44841133], Cost: 0.5180
Current parameters: [0.85679578 1.47577953 3.63984871 1.05221846 2.67869326 0.46111398], Cost: 0.3450
Current parameters: [0.91767606 1.56320205 3.59838644 1.02811184 2.64714407 0.49242362], Cost: 0.2654
Current parameters: [0.97450574 1.60165703 3.56768795 1.00733702 2.74414487 0.48087131], Cost: 0.4552
```



```
Current parameters: [0.92011416 1.54585605 3.59781819 1.03138073 2.66007847 0.55091391], Cost: 0.4171
Current parameters: [0.90510066 1.57756333 3.60853541 0.95533283 2.56606463 0.43509167], Cost: 0.2778
Current parameters: [0.88557403 1.55992782 3.54511266 1.02342995 2.64934238 0.49204869], Cost: 0.3495
Current parameters: [0.85730721 1.65225723 3.64546815 1.07056006 2.64584741 0.48698705], Cost: 0.1156
Current parameters: [0.89621731 1.66570958 3.64362035 1.16537459 2.59159766 0.44233513], Cost: 0.3228
Current parameters: [0.89461628 1.66420959 3.6485687  1.06281979 2.68488946 0.45910126], Cost: 0.1006
Current parameters: [0.81990413 1.73039107 3.68305667 1.01319691 2.72610947 0.44143298], Cost: 0.0644
Current parameters: [0.84215253 1.74188362 3.71745515 0.99871811 2.73139584 0.48454149], Cost: 0.0672
Current parameters: [0.82266468 1.71929535 3.69931724 0.99941254 2.71255249 0.42702394], Cost: 0.0591
Current parameters: [0.80450871 1.68392867 3.72657782 1.02236765 2.74504054 0.42753243], Cost: 0.0673
Current parameters: [0.82053102 1.70462915 3.68591142 0.97838758 2.72138037 0.43458808], Cost: 0.1035
Current parameters: [0.82664289 1.75569604 3.72812236 1.0291481  2.69897065 0.40119742], Cost: 0.0481
Current parameters: [0.82681212 1.78780046 3.74220352 0.99847348 2.7081034  0.36054143], Cost: 0.0948
Current parameters: [0.79931562 1.76189149 3.73260283 1.02401381 2.69018256 0.40942744], Cost: 0.0581
Current parameters: [0.85047241 1.73485285 3.7252295  1.06100133 2.65637219 0.40934232], Cost: 0.0820
Current parameters: [0.81973662 1.74060776 3.74357672 1.04154765 2.71624439 0.3979972 ], Cost: 0.0475
Current parameters: [0.82546663 1.74195885 3.75397675 1.03627847 2.7157798  0.40656117], Cost: 0.0342
Current parameters: [0.83145553 1.75754683 3.75155444 1.04609703 2.73082326 0.42577401], Cost: 0.0344
Current parameters: [0.82389288 1.74773807 3.75501779 1.02699107 2.7247575  0.40021091], Cost: 0.0308
Current parameters: [0.84230576 1.74947103 3.77679411 1.01446878 2.72456995 0.40207476], Cost: 0.0158
Current parameters: [0.8268326  1.75728722 3.79640517 1.00299124 2.71872313 0.41327914], Cost: 0.0115
Current parameters: [0.82327565 1.7487655  3.80334301 1.00837821 2.72771806 0.41238063], Cost: 0.0101
Current parameters: [0.82744766 1.73301621 3.79741514 0.98855889 2.73458827 0.42774201], Cost: 0.0093
Current parameters: [0.82442143 1.7252277  3.79855644 0.98369287 2.72708333 0.41809748], Cost: 0.0139
Current parameters: [0.84399271 1.74365662 3.8165178  0.98465513 2.74811304 0.43289325], Cost: 0.0058
Current parameters: [0.82231303 1.75570194 3.82627113 0.97934739 2.75430197 0.4470305 ], Cost: 0.0073
Current parameters: [0.8460141  1.73970554 3.82257566 0.98952775 2.73841385 0.44125563], Cost: 0.0059
Current parameters: [0.85631836 1.74482309 3.80864261 1.0017016  2.76548012 0.44589571], Cost: 0.0299
Current parameters: [0.84834604 1.75338893 3.81177931 0.97793264 2.74043277 0.4348528 ], Cost: 0.0053
Current parameters: [0.84926929 1.74916312 3.81034574 0.972805   2.74185595 0.43830998], Cost: 0.0099
Current parameters: [0.84669804 1.75634839 3.82088672 0.97683953 2.7347828  0.42405446], Cost: 0.0029
Current parameters: [0.84876799 1.76586228 3.82160934 0.98850596 2.73469436 0.42048986], Cost: 0.0010
Current parameters: [0.85539216 1.77316744 3.82995139 0.99089151 2.73277493 0.41224784], Cost: 0.0045
Current parameters: [0.85196536 1.76222177 3.81767592 0.99003637 2.73206653 0.41690668], Cost: 0.0016
Current parameters: [0.8355022  1.76862426 3.81794253 0.99136443 2.72866418 0.41889253], Cost: 0.0039
Current parameters: [0.84782358 1.76790248 3.8184203  0.98601379 2.73953072 0.41646374], Cost: 0.0018
Current parameters: [0.85475912 1.77329491 3.81404344 0.98680903 2.73242851 0.42985717], Cost: 0.0008
Current parameters: [0.85529988 1.77102652 3.81400228 0.99154372 2.73693544 0.43344405], Cost: 0.0008
Current parameters: [0.85788172 1.77338233 3.81593001 0.98593217 2.73340637 0.42939211], Cost: 0.0009
Current parameters: [0.85314277 1.77733371 3.81783863 0.99066093 2.72921665 0.43145532], Cost: 0.0006
Current parameters: [0.85315671 1.77499871 3.81891854 0.98967523 2.72741287 0.4335565 ], Cost: 0.0004
```



```
Current parameters: [0.84887386 1.77522229 3.82107059 0.98615863 2.72744775 0.43862043], Cost: 0.0005
Current parameters: [0.85487791 1.7766017  3.81675223 0.98935819 2.72666265 0.43564706], Cost: 0.0004
Current parameters: [0.85492539 1.77258304 3.81607519 0.99207996 2.72065827 0.43662906], Cost: 0.0005
Current parameters: [0.85767874 1.77651876 3.81934798 0.98865892 2.7266249  0.43607001], Cost: 0.0005
Current parameters: [0.85479129 1.77704611 3.8166502  0.98806729 2.72563731 0.43470816], Cost: 0.0007
Current parameters: [0.85554452 1.77395334 3.81539599 0.98919659 2.72891943 0.43656469], Cost: 0.0003
Current parameters: [0.85399256 1.77401035 3.81639865 0.99042411 2.7301405  0.43953854], Cost: 0.0002
Current parameters: [0.85398515 1.77287848 3.81640451 0.99088662 2.72867811 0.43996394], Cost: 0.0001
Current parameters: [0.85514456 1.77260887 3.81576565 0.99446438 2.72916825 0.43933691], Cost: 0.0002
Current parameters: [0.8529913  1.76993562 3.81493949 0.98992563 2.72829273 0.44151065], Cost: 0.0004
Current parameters: [0.85534489 1.77251545 3.81764623 0.99061277 2.7287634  0.44042206], Cost: 0.0002
Current parameters: [0.8544052  1.7733173  3.81588336 0.99080982 2.72845792 0.44047256], Cost: 0.0002
Current parameters: [0.8529866  1.77366427 3.81719906 0.99128104 2.72752542 0.43967307], Cost: 0.0001
Current parameters: [0.85344509 1.77361948 3.81682422 0.99079063 2.72665577 0.43810298], Cost: 0.0001
Current parameters: [0.85266192 1.77355123 3.81725034 0.9904072  2.72740671 0.43990793], Cost: 0.0001
Current parameters: [0.85185891 1.7732433  3.81721557 0.99277896 2.72719581 0.43955866], Cost: 0.0000
Current parameters: [0.85241771 1.77308676 3.81885463 0.9935478  2.72677188 0.43970207], Cost: 0.0001
Current parameters: [0.85183705 1.77401211 3.81739842 0.99288838 2.72771529 0.43934219], Cost: 0.0000
Current parameters: [0.85022933 1.77260851 3.81665509 0.99305573 2.72716035 0.43895515], Cost: 0.0000
Current parameters: [0.8500039  1.77332104 3.81553482 0.99372145 2.72613463 0.43966641], Cost: 0.0000
Current parameters: [0.84993753 1.77249171 3.81676728 0.993101   2.72760574 0.43975632], Cost: 0.0000
Current parameters: [0.85096658 1.77177777 3.81557907 0.99384712 2.72798175 0.43860558], Cost: 0.0000
Current parameters: [0.84991343 1.77224197 3.81585297 0.99137251 2.72691389 0.43874734], Cost: 0.0000
Current parameters: [0.85008647 1.77221537 3.81719088 0.9935698  2.72670952 0.43879985], Cost: 0.0000
Current parameters: [0.84995719 1.77330669 3.81668503 0.99324713 2.72762031 0.43857691], Cost: 0.0000
Current parameters: [0.85013676 1.77254134 3.81645299 0.99263436 2.72711404 0.43888592], Cost: 0.0000
Current parameters: [0.85017141 1.77267026 3.81659428 0.99253727 2.72702488 0.43895259], Cost: 0.0000
Current parameters: [0.8505427  1.77247738 3.8162394  0.99261443 2.72726721 0.43887827], Cost: 0.0000
Current parameters: [0.85051412 1.77262561 3.81589265 0.99275437 2.72703407 0.43902474], Cost: 0.0000
Current parameters: [0.85056047 1.77244466 3.81593274 0.9927589  2.72689526 0.43908654], Cost: 0.0000
Current parameters: [0.85052424 1.77251227 3.81561003 0.9928642  2.72719907 0.43869869], Cost: 0.0000
Current parameters: [0.85042606 1.77238362 3.81543261 0.99286759 2.7275346  0.43895977], Cost: 0.0000
Current parameters: [0.85038584 1.77237084 3.81535518 0.9926442  2.72749763 0.43893614], Cost: 0.0000
Current parameters: [0.8507058  1.77253065 3.81536334 0.99314688 2.72776437 0.43890554], Cost: 0.0000
Current parameters: [0.85000016 1.77236935 3.81548437 0.99307971 2.72754078 0.43886432], Cost: 0.0000
Current parameters: [0.85002356 1.77215475 3.81547798 0.99315679 2.7274595  0.43888477], Cost: 0.0000
Current parameters: [0.84999264 1.77241086 3.81541001 0.9931099  2.72747604 0.43891423], Cost: 0.0000
Current parameters: [0.85000016 1.77236935 3.81548437 0.99307971 2.72754078 0.43886432], Cost: 0.0000

   Return from subroutine COBYLA because the MAXFUN limit has been reached.
```



```
   NFVALS =  100   F = 1.770926E-07    MAXCV = 0.000000E+00
   X = 8.500002E-01   1.772369E+00   3.815484E+00   9.930797E-01   2.727541E+00
       4.388643E-01
Simulating for QAOA depth p = 4
Current parameters: [1.5032158  1.61962018 1.16528447 0.80098051 1.97539513 1.5857156
 1.43418248 2.80442457], Cost: 2.8322
Current parameters: [0.5032158  2.61962018 1.16528447 0.80098051 1.97539513 1.5857156
 1.43418248 2.80442457], Cost: 2.3386
Current parameters: [0.5032158  1.61962018 2.16528447 0.80098051 1.97539513 1.5857156
 1.43418248 2.80442457], Cost: 2.4002
Current parameters: [0.5032158  1.61962018 1.16528447 1.80098051 1.97539513 1.5857156
 1.43418248 2.80442457], Cost: 3.2956
Current parameters: [0.5032158  1.61962018 1.16528447 0.80098051 2.97539513 1.5857156
 1.43418248 2.80442457], Cost: 0.8086
Current parameters: [0.5032158  1.61962018 1.16528447 0.80098051 2.97539513 2.5857156
 1.43418248 2.80442457], Cost: 3.5215
Current parameters: [0.5032158  1.61962018 1.16528447 0.80098051 2.97539513 1.5857156
 2.43418248 2.80442457], Cost: 1.4013
Current parameters: [0.5032158  1.61962018 1.16528447 0.80098051 2.97539513 1.5857156
 1.43418248 3.80442457], Cost: 2.3519
Current parameters: [0.2485424  1.49347481 1.02307727 0.425673   3.24755892 0.87942835
 1.27987721 2.40264693], Cost: 2.4201
Current parameters: [0.99515278 1.60795414 1.15213301 0.76627173 3.00056508 1.5203975
 1.41991219 2.7672678 ], Cost: 1.0953
Current parameters: [0.5032158  1.80664287 0.99938536 0.80098051 2.97539513 1.5857156
 1.43418248 2.80442457], Cost: 0.7947
Current parameters: [0.49831539 2.13621544 1.36937358 0.77452625 2.99457911 1.5359316
 1.42330598 2.77610451], Cost: 0.9652
Current parameters: [0.45541285 1.72884591 1.07535476 1.01415183 3.00254818 1.55856255
 1.40702943 2.77727152], Cost: 0.9099
Current parameters: [0.66085188 2.09602948 0.70522657 0.99431857 2.95804041 1.46838581
 1.48810218 2.78156728], Cost: 1.0715
Current parameters: [0.41930313 1.76519    0.96616787 0.69987683 3.04114667 1.41999928
 1.39556999 2.70882709], Cost: 1.8454
Current parameters: [0.49659252 1.79650531 1.00869432 0.80144782 2.85806809 1.54568834
 1.43074218 2.80098427], Cost: 1.0434
Current parameters: [0.52333293 1.83244738 0.98917939 0.82112417 2.97013954 1.82650477
 1.41809283 2.7529117 ], Cost: 0.4501
Current parameters: [0.53395865 1.84390574 0.99165634 0.84115322 3.0063495  1.92198001
 1.39803837 2.97878697], Cost: 0.5159
Current parameters: [0.43852077 1.85072176 0.97702052 0.82710813 2.96580013 1.83776591
```

```
 1.5059997  2.7593177 ], Cost: 0.5632
Current parameters: [0.69108611 1.76579405 1.04770303 0.82100163 2.99882834 1.85884731
 1.57424467 2.73746207], Cost: 1.0736
Current parameters: [0.54283156 1.79686031 0.8762791  0.85432694 2.96445193 1.82213942
 1.42650969 2.75559786], Cost: 0.5489
Current parameters: [0.5804082  2.0559209  0.96504628 0.90743034 2.98463382 1.80506427
 1.40403227 2.73282793], Cost: 0.4750
Current parameters: [0.43359021 1.8580475  0.97393819 0.74944261 3.07888794 1.89735914
 1.25460355 2.68330543], Cost: 0.5494
Current parameters: [0.51042578 1.84234814 1.00100794 0.81879737 2.85750026 1.84848428
 1.37288161 2.75237273], Cost: 0.8070
Current parameters: [0.54183861 1.87718342 0.96958305 0.78824348 2.96663222 1.82313033
 1.42623713 2.75561397], Cost: 0.5259
Current parameters: [0.53247564 1.90006511 1.00110733 0.91868142 2.99751278 1.8159384
 1.40379465 2.73728545], Cost: 0.2836
Current parameters: [0.56267505 1.8890652  1.02448893 0.93464012 3.03266056 1.83131549
 1.42151582 2.72037368], Cost: 0.3389
Current parameters: [0.5434115  1.89671236 1.0079516  0.92342188 2.98738235 1.79609669
 1.41695472 2.74762499], Cost: 0.3040
Current parameters: [0.50966293 1.89993466 1.00305402 0.91918393 3.03340897 1.77982365
 1.37622999 2.74268449], Cost: 0.1701
Current parameters: [0.48591272 1.90593186 1.01874948 0.9417268  3.03107594 1.76463291
 1.32835309 2.73785914], Cost: 0.1120
Current parameters: [0.43313268 1.91723945 1.02164341 0.95668383 3.04446229 1.75832482
 1.35162452 2.73770609], Cost: 0.1127
Current parameters: [0.48563099 1.9101045  1.02797023 0.94147657 3.03581955 1.77629984
 1.32530945 2.76443177], Cost: 0.0861
Current parameters: [0.48545506 1.89739317 0.98535637 0.97001828 3.05183822 1.77397201
 1.30806106 2.78798059], Cost: 0.0875
Current parameters: [0.49427024 1.93889223 1.02257392 0.94588611 3.03923592 1.77317932
 1.32593029 2.76274828], Cost: 0.0917
Current parameters: [0.46938139 1.91491255 1.03549144 0.90438681 3.05782402 1.76110069
 1.29170477 2.78293526], Cost: 0.1032
Current parameters: [0.50124798 1.90057503 1.05836026 0.96485323 3.07993878 1.78271709
 1.32258545 2.75411579], Cost: 0.0607
Current parameters: [0.5073162  1.89745438 1.06878627 0.97309872 3.07441989 1.75960084
 1.32815376 2.76666513], Cost: 0.0921
Current parameters: [0.49749455 1.90245752 1.06292393 0.97177177 3.07351892 1.7837315
 1.31209605 2.75176114], Cost: 0.0474
Current parameters: [0.49317314 1.90400353 1.0704277  0.97961593 3.07185292 1.81129053
 1.30352822 2.75316711], Cost: 0.0236
```



```
Current parameters: [0.48328418 1.9073319  1.07631561 0.96735668 3.08355717 1.81455912
 1.28048714 2.75520965], Cost: 0.0105
Current parameters: [0.49035042 1.90161204 1.074658   0.97065071 3.08153397 1.82608742
 1.26329031 2.73405333], Cost: 0.0185
Current parameters: [0.48263124 1.91058671 1.08682576 0.96046281 3.0796895  1.8148993
 1.28512443 2.74900058], Cost: 0.0147
Current parameters: [0.45699591 1.91031885 1.07464334 0.97900726 3.09121035 1.81632362
 1.28453448 2.74746567], Cost: 0.0068
Current parameters: [0.46258668 1.92263071 1.07527058 0.98344166 3.09731471 1.81554154
 1.28454157 2.74562994], Cost: 0.0087
Current parameters: [0.44778768 1.92588297 1.0621366  0.9730437  3.07402716 1.82424447
 1.27813358 2.75511899], Cost: 0.0114
Current parameters: [0.45610891 1.91073866 1.08149845 0.98694663 3.08453507 1.80959528
 1.27836012 2.74974579], Cost: 0.0068
Current parameters: [0.45223034 1.9044278  1.08584422 0.98385012 3.10257792 1.83207023
 1.28127738 2.76702197], Cost: 0.0016
Current parameters: [0.44144361 1.89765839 1.08290284 0.97622447 3.11976725 1.81666632
 1.27160202 2.77803174], Cost: 0.0113
Current parameters: [0.45335789 1.89962883 1.083247   0.99330216 3.10026758 1.83463222
 1.29116653 2.77077106], Cost: 0.0049
Current parameters: [0.45373898 1.90227082 1.08193529 0.98681523 3.10353771 1.83404911
 1.27655955 2.76530737], Cost: 0.0009
Current parameters: [0.45202542 1.89963709 1.09059292 0.98581315 3.102898   1.84287243
 1.27301104 2.75709415], Cost: 0.0018
Current parameters: [0.44734082 1.90536544 1.07971564 0.98820409 3.10246776 1.83558609
 1.27636407 2.76495963], Cost: 0.0010
Current parameters: [0.45804656 1.90569002 1.07950978 0.98161199 3.09545725 1.84024765
 1.2714135  2.77243058], Cost: 0.0048
Current parameters: [0.45419054 1.90207868 1.07850866 0.98285196 3.10688414 1.83741214
 1.27964672 2.76416466], Cost: 0.0012
Current parameters: [0.45888496 1.91344648 1.08422247 0.99243495 3.11081844 1.83508136
 1.27583073 2.76414727], Cost: 0.0012
Current parameters: [0.4563277  1.90466343 1.07758328 0.98594666 3.09853373 1.82700059
 1.27739953 2.75362006], Cost: 0.0014
Current parameters: [0.45130253 1.90012995 1.08197337 0.98624497 3.10834972 1.83004365
 1.27324661 2.76518827], Cost: 0.0012
Current parameters: [0.45378545 1.89872131 1.08112357 0.99267045 3.10415306 1.83407866
 1.28017824 2.76517683], Cost: 0.0009
Current parameters: [0.45435349 1.9027292  1.0808181  0.98676986 3.10237326 1.8322198
 1.27683237 2.76236727], Cost: 0.0006
Current parameters: [0.45472517 1.90311159 1.07961549 0.98678646 3.10220537 1.83141379
```



```
 1.2769836  2.76354332], Cost: 0.0008
Current parameters: [0.45523162 1.9018787  1.08122708 0.9873137  3.10059029 1.83332602
 1.27489486 2.7600991 ], Cost: 0.0006
Current parameters: [0.45453245 1.90068535 1.08123974 0.98646834 3.09964324 1.83304838
 1.27527664 2.7603584 ], Cost: 0.0007
Current parameters: [0.45583999 1.90392257 1.08365192 0.98792317 3.10083208 1.83268994
 1.27593257 2.75839028], Cost: 0.0003
Current parameters: [0.45668073 1.90437177 1.08268123 0.98686907 3.10264179 1.83434858
 1.27714258 2.7561979 ], Cost: 0.0004
Current parameters: [0.45695618 1.90300446 1.0841398  0.98755563 3.1016135  1.83189868
 1.27559764 2.75845246], Cost: 0.0003
Current parameters: [0.45504857 1.90320318 1.08436995 0.98845976 3.1034475  1.83042793
 1.27380682 2.75704668], Cost: 0.0002
Current parameters: [0.45498864 1.90404497 1.08465877 0.98701514 3.10338708 1.8305648
 1.27289029 2.7573107 ], Cost: 0.0003
Current parameters: [0.45540559 1.90313502 1.08674621 0.99027419 3.10483952 1.83212631
 1.27313636 2.75800384], Cost: 0.0000
Current parameters: [0.45750512 1.90466798 1.08717198 0.99279813 3.10535402 1.83115608
 1.27228354 2.75812976], Cost: 0.0001
Current parameters: [0.45575587 1.90256704 1.08695072 0.9904407  3.10383892 1.83264132
 1.27222156 2.75690997], Cost: 0.0001
Current parameters: [0.45442756 1.90224382 1.08985583 0.99070791 3.10568681 1.83130853
 1.27463689 2.75811305], Cost: 0.0001
Current parameters: [0.45488025 1.90114282 1.08579685 0.99120066 3.10662212 1.83295996
 1.27202854 2.76004207], Cost: 0.0001
Current parameters: [0.45582368 1.90336653 1.08656417 0.99005635 3.10607393 1.83295008
 1.27370393 2.75701329], Cost: 0.0000
Current parameters: [0.4550842  1.90368549 1.08646555 0.99035811 3.1062253  1.83287836
 1.27342696 2.75669777], Cost: 0.0000
Current parameters: [0.45555785 1.90409532 1.08658578 0.99057475 3.10541731 1.83415587
 1.27456718 2.75757312], Cost: 0.0000
Current parameters: [0.45584292 1.90374581 1.08700944 0.98962836 3.10622887 1.83323625
 1.27319318 2.75726075], Cost: 0.0000
Current parameters: [0.45586291 1.90461037 1.08656902 0.98837915 3.10652902 1.832191
 1.27354921 2.75731506], Cost: 0.0000
Current parameters: [0.45532749 1.90323651 1.08682009 0.98909723 3.10619864 1.83354486
 1.27327982 2.7573444 ], Cost: 0.0000
Current parameters: [0.45605095 1.90327262 1.08657493 0.99022256 3.10747218 1.83331532
 1.27255706 2.75828493], Cost: 0.0000
Current parameters: [0.45606208 1.90337872 1.08751928 0.99036067 3.10759149 1.83322305
 1.27262514 2.75822145], Cost: 0.0000
```



```
Current parameters: [0.45534661 1.90200172 1.0865024  0.99018761 3.10830121 1.83283139
 1.27337204 2.75861849], Cost: 0.0000
Current parameters: [0.45642595 1.90303057 1.08661817 0.99028053 3.10735856 1.83354153
 1.2718458  2.75786153], Cost: 0.0000
Current parameters: [0.45597624 1.90317594 1.08659888 0.99047504 3.10735221 1.83367279
 1.27265274 2.75837347], Cost: 0.0000
Current parameters: [0.45617967 1.90396747 1.0865334  0.99045874 3.10729684 1.83405125
 1.27270149 2.75873754], Cost: 0.0000
Current parameters: [0.45604825 1.90318873 1.0865536  0.99041525 3.1076709  1.83385623
 1.27283207 2.75812837], Cost: 0.0000
Current parameters: [0.45576973 1.90265765 1.08648336 0.99052777 3.10808815 1.8339776
 1.27316727 2.75865303], Cost: 0.0000
Current parameters: [0.45644381 1.90300894 1.0866095  0.99027296 3.10756932 1.83391242
 1.27289962 2.758219  ], Cost: 0.0000
Current parameters: [0.45663946 1.90287472 1.08661827 0.99047247 3.10751594 1.83428811
 1.27217272 2.75778872], Cost: 0.0000
Current parameters: [0.45665105 1.9030484  1.08651499 0.99064713 3.10765805 1.8337257
 1.27294699 2.75821842], Cost: 0.0000
Current parameters: [0.45632873 1.9030527  1.0865923  0.99018729 3.10694058 1.83394729
 1.2734099  2.75769558], Cost: 0.0000
Current parameters: [0.45652976 1.90340418 1.08659446 0.99019317 3.10753448 1.83411819
 1.27292288 2.75837435], Cost: 0.0000
Current parameters: [0.45645157 1.9030448  1.0868448  0.99030826 3.10759001 1.83389326
 1.27291183 2.75819256], Cost: 0.0000
Current parameters: [0.45623682 1.90268493 1.08660381 0.9901709  3.1076831  1.83403261
 1.27304021 2.7584006 ], Cost: 0.0000
Current parameters: [0.45641424 1.90300358 1.08658874 0.99025475 3.10741297 1.83391067
 1.2730208  2.75808187], Cost: 0.0000
Current parameters: [0.45641549 1.9029774  1.08659358 0.99031366 3.10738635 1.83399811
 1.27297577 2.7580635 ], Cost: 0.0000
Current parameters: [0.45643387 1.90294519 1.08656739 0.99019634 3.10737101 1.83396425
 1.27283776 2.75791069], Cost: 0.0000
Current parameters: [0.45639066 1.90292483 1.0865827  0.99019759 3.10719788 1.83387422
 1.27273805 2.75800589], Cost: 0.0000
Current parameters: [0.45645153 1.90288082 1.0865558  0.99026707 3.10739038 1.8338996
 1.27284109 2.75788344], Cost: 0.0000
Current parameters: [0.45656623 1.90295391 1.08643328 0.99027217 3.10734576 1.83387215
 1.27281276 2.75773336], Cost: 0.0000
Current parameters: [0.45654259 1.90282667 1.08657204 0.99022916 3.10736917 1.83391703
 1.27286232 2.75791122], Cost: 0.0000
Current parameters: [0.45645153 1.90288082 1.0865558  0.99026707 3.10739038 1.8338996
```

```
 1.27284109 2.75788344], Cost: 0.0000

   Return from subroutine COBYLA because the MAXFUN limit has been reached.

   NFVALS =  100   F = 1.115160E-07    MAXCV = 0.000000E+00
   X = 4.564515E-01   1.902881E+00   1.086556E+00   9.902671E-01   3.107390E+00
       1.833900E+00   1.272841E+00   2.757883E+00
Simulating for QAOA depth p = 5
Current parameters: [3.98144336 1.30676235 2.26643647 2.06517776 1.12290235 2.63490724
 2.89793079 2.18406569 1.28327509 2.47940213], Cost: 0.6234
Current parameters: [3.98144336 2.30676235 2.26643647 2.06517776 1.12290235 2.63490724
 2.89793079 2.18406569 1.28327509 2.47940213], Cost: 2.0077
Current parameters: [3.98144336 1.30676235 3.26643647 2.06517776 1.12290235 2.63490724
 2.89793079 2.18406569 1.28327509 2.47940213], Cost: 1.2361
Current parameters: [3.98144336 1.30676235 2.26643647 3.06517776 1.12290235 2.63490724
 2.89793079 2.18406569 1.28327509 2.47940213], Cost: 2.1156
Current parameters: [3.98144336 1.30676235 2.26643647 2.06517776 2.12290235 2.63490724
 2.89793079 2.18406569 1.28327509 2.47940213], Cost: 3.1473
Current parameters: [3.98144336 1.30676235 2.26643647 2.06517776 1.12290235 3.63490724
 2.89793079 2.18406569 1.28327509 2.47940213], Cost: 2.3719
Current parameters: [3.98144336 1.30676235 2.26643647 2.06517776 1.12290235 2.63490724
 3.89793079 2.18406569 1.28327509 2.47940213], Cost: 2.1207
Current parameters: [3.98144336 1.30676235 2.26643647 2.06517776 1.12290235 2.63490724
 2.89793079 3.18406569 1.28327509 2.47940213], Cost: 2.2287
Current parameters: [3.98144336 1.30676235 2.26643647 2.06517776 1.12290235 2.63490724
 2.89793079 2.18406569 2.28327509 2.47940213], Cost: 2.7760
Current parameters: [3.98144336 1.30676235 2.26643647 2.06517776 1.12290235 2.63490724
 2.89793079 2.18406569 1.28327509 3.47940213], Cost: 3.0114
Current parameters: [4.20114943 1.05641841 2.1556285  1.7953301  0.66646645 2.31869792
 2.62714853 1.89375498 0.89399512 2.04754951], Cost: 2.8797
Current parameters: [4.0912964  1.18159038 2.21103248 1.93025393 0.8946844  2.47680258
 2.76253966 2.03891034 1.0886351  2.26347582], Cost: 2.8163
Current parameters: [3.98931264 1.55434571 2.26246763 2.05551255 1.10655405 2.62358147
 2.8882321  2.17366755 1.26933213 2.46393434], Cost: 0.4858
Current parameters: [3.98931264 1.55434571 2.26246763 2.05551255 1.19246337 2.62358147
 2.8882321  2.17366755 1.26933213 2.37313462], Cost: 0.6962
Current parameters: [4.0142493  1.57792079 2.24989091 2.02488481 1.25123403 2.58769168
 2.85749829 2.14071726 1.22514882 2.64838845], Cost: 1.0357
Current parameters: [3.98931264 1.55434571 2.26246763 2.05551255 1.10655405 2.52655739
 2.8882321  2.17366755 1.3481441  2.46393434], Cost: 0.2697
Current parameters: [4.01315076 1.57688224 2.25044496 2.02623406 1.07586112 2.64970406
```



```
 2.85885221 2.14216882 1.55354513 2.48036554], Cost: 0.4537
Current parameters: [3.98931264 1.55434571 2.26246763 2.05551255 1.10655405 2.52655739
 2.79682281 2.25892798 1.3481441  2.46393434], Cost: 0.3260
Current parameters: [4.01289045 1.57663614 2.25057624 2.02655377 1.07619628 2.50692622
 3.05885489 2.34378671 1.37718629 2.48018611], Cost: 0.4108
Current parameters: [4.08624688 1.55434571 2.26246763 2.134435   1.10655405 2.52655739
 2.8882321  2.17366755 1.3481441  2.46393434], Cost: 0.1677
Current parameters: [3.94675727 1.5782718  2.24970366 2.33269876 1.07396864 2.50548569
 2.90283408 2.17557399 1.37931739 2.48137866], Cost: 0.3624
Current parameters: [4.0825034  1.55861121 2.38631533 2.13903281 1.11433106 2.53194514
 2.89284584 2.17861402 1.35477687 2.47129248], Cost: 0.2386
Current parameters: [4.15932519 1.55165585 2.26298246 2.10698219 0.92540232 2.40453477
 2.85924116 2.11078932 1.33001274 2.40418454], Cost: 1.1446
Current parameters: [4.04288111 1.55446485 2.26234841 2.09864444 1.1476973  2.45677023
 2.90559336 2.1098475  1.35140846 2.50286165], Cost: 0.4273
Current parameters: [4.20276049 1.58875212 2.22667647 2.19049532 1.19391604 2.58615511
 2.84774354 2.25866856 1.34368941 2.61640948], Cost: 0.8749
Current parameters: [4.08004852 1.56103182 2.25564545 2.11201506 1.09647547 2.5059162
 2.98455732 2.24373396 1.36455769 2.4611437 ], Cost: 0.0859
Current parameters: [4.08059188 1.50696369 2.25798103 2.12191565 1.10086491 2.49461122
 2.98538545 2.25440419 1.34005754 2.46529674], Cost: 0.0557
Current parameters: [4.07552728 1.44511745 2.25151037 2.10834344 1.09115939 2.54001618
 2.99478634 2.23732933 1.43497435 2.46713562], Cost: 0.2484
Current parameters: [4.07204375 1.50112449 2.2646079  2.11230129 1.06095397 2.47596532
 2.98698305 2.2374988  1.33724444 2.42753537], Cost: 0.0553
Current parameters: [4.15781962 1.51423974 2.25185431 2.13245302 1.00648706 2.45311697
 2.97905625 2.28886183 1.30584425 2.44627269], Cost: 0.0554
Current parameters: [4.03981482 1.50594348 2.26203709 2.14886791 1.03282519 2.47587202
 2.98993612 2.23787153 1.34015145 2.45384219], Cost: 0.0655
Current parameters: [4.0679104  1.513131   2.25080573 2.18882855 1.10366897 2.46388981
 2.9938729  2.27382544 1.3316885  2.34976335], Cost: 0.0991
Current parameters: [4.06851191 1.50363234 2.25980285 2.10621067 1.0539721  2.51532788
 3.00948077 2.2345294  1.29623559 2.42183848], Cost: 0.0454
Current parameters: [4.0570697  1.50362054 2.25981465 2.09694238 1.04989922 2.52223628
 2.99292389 2.25468722 1.29591245 2.41798497], Cost: 0.0389
Current parameters: [4.03814199 1.51040044 2.24690184 2.07260526 1.06051403 2.48677817
 2.99978884 2.2625143  1.26260851 2.43045468], Cost: 0.0495
Current parameters: [4.05533586 1.50553405 2.28222846 2.09411838 1.04971502 2.5198558
 3.00968839 2.26718391 1.29988031 2.41892321], Cost: 0.0263
Current parameters: [4.05865178 1.50894882 2.32697161 2.10121626 1.05555178 2.52135285
 2.97946388 2.2537204  1.27378572 2.42330972], Cost: 0.0474
```



```
Current parameters: [4.05809895 1.49783362 2.28489159 2.07590758 1.03866342 2.52447188
 3.00847065 2.25912823 1.30525986 2.437148  ], Cost: 0.0126
Current parameters: [4.06114311 1.45478125 2.29134238 2.05451474 1.02900653 2.54145453
 3.03043366 2.28186449 1.31752545 2.44072403], Cost: 0.0507
Current parameters: [4.03665069 1.49457568 2.28808349 2.0708585  1.05224706 2.53017472
 3.01045294 2.2462714  1.31311825 2.43243424], Cost: 0.0234
Current parameters: [4.06690216 1.54981876 2.28602616 2.05395774 1.02389985 2.53617983
 3.0143357  2.2612487  1.32066456 2.4406088 ], Cost: 0.0099
Current parameters: [4.051476   1.5531535  2.28547245 2.06970773 1.00797431 2.53674129
 3.01722    2.26316637 1.32130888 2.455195  ], Cost: 0.0155
Current parameters: [4.06480041 1.54833598 2.28772771 2.05161412 1.01399589 2.53114492
 3.01451953 2.25705372 1.32035421 2.43122956], Cost: 0.0070
Current parameters: [4.06757276 1.54795259 2.29096766 2.05202682 1.01606776 2.5112961
 3.00418689 2.26040713 1.34091193 2.43560738], Cost: 0.0382
Current parameters: [4.0604878  1.54952735 2.2806214  2.0456183  1.01584174 2.52286441
 3.0200322  2.25998418 1.31579234 2.43366089], Cost: 0.0111
Current parameters: [4.06612082 1.55249012 2.30536415 2.04570045 1.01220223 2.53234568
 3.00702093 2.25220637 1.29779293 2.43548984], Cost: 0.0059
Current parameters: [4.06080662 1.55057939 2.30467291 2.04115147 1.01028172 2.53636332
 2.99966644 2.26274155 1.29786062 2.43300686], Cost: 0.0067
Current parameters: [4.07642313 1.54232331 2.30645109 2.0401823  1.00175834 2.54956337
 3.02393929 2.25424932 1.29279647 2.43167718], Cost: 0.0185
Current parameters: [4.06769371 1.54918167 2.29800852 2.04058336 1.00853339 2.53478328
 3.00043404 2.24487952 1.29815027 2.44130628], Cost: 0.0080
Current parameters: [4.06805644 1.56405132 2.29197559 2.05929636 1.01473141 2.5370238
 2.99939058 2.24920659 1.28574906 2.42017661], Cost: 0.0183
Current parameters: [4.06698684 1.56542073 2.30585305 2.03983376 1.00933181 2.53566559
 3.00862863 2.25192783 1.30218357 2.43606582], Cost: 0.0088
Current parameters: [4.04489934 1.54876326 2.31099774 2.04122668 1.02398836 2.54109385
 3.01035378 2.23849167 1.30136502 2.4297309 ], Cost: 0.0048
Current parameters: [4.04874387 1.54564328 2.31898304 2.04728593 1.02697661 2.53119196
 3.01262317 2.23853849 1.29943446 2.43154471], Cost: 0.0040
Current parameters: [4.04681548 1.54084765 2.33088277 2.03909372 1.02764717 2.52450238
 2.9962781  2.23527487 1.31934037 2.426868  ], Cost: 0.0109
Current parameters: [4.04035575 1.54647981 2.32075565 2.0547178  1.01867342 2.53159922
 3.01282184 2.23872253 1.30069026 2.43817393], Cost: 0.0035
Current parameters: [4.03825227 1.54621878 2.3193974  2.05202939 1.01516459 2.5271525
 3.01448922 2.23785694 1.29924588 2.43504836], Cost: 0.0052
Current parameters: [4.03564958 1.54714763 2.31774479 2.05166367 1.0270217  2.52907793
 3.01482953 2.24333817 1.29917656 2.44814772], Cost: 0.0020
Current parameters: [4.03693832 1.54363226 2.32019511 2.04853276 1.02492899 2.53114507
```



```
 3.0189255  2.24434774 1.30041692 2.4499429 ], Cost: 0.0014
Current parameters: [4.03723761 1.53874207 2.32134399 2.05549941 1.03047857 2.53898803
 3.02447597 2.24762803 1.29517185 2.45292519], Cost: 0.0040
Current parameters: [4.03760785 1.54341292 2.31675273 2.05105641 1.02562013 2.52995048
 3.02213088 2.24552398 1.30564049 2.44872983], Cost: 0.0013
Current parameters: [4.04048458 1.55629732 2.32192252 2.04967387 1.02562434 2.53085866
 3.0277884  2.24581379 1.30728105 2.45109869], Cost: 0.0056
Current parameters: [4.03665314 1.54286113 2.31633635 2.05095234 1.02729145 2.53032929
 3.02386605 2.23831854 1.30621715 2.44992831], Cost: 0.0009
Current parameters: [4.04570201 1.54046165 2.30987624 2.05129212 1.02284241 2.52833961
 3.02031552 2.23659297 1.30540558 2.45856877], Cost: 0.0032
Current parameters: [4.03253504 1.53681598 2.32162515 2.04844349 1.03113751 2.52974579
 3.01673297 2.23832798 1.31559505 2.45148196], Cost: 0.0044
Current parameters: [4.0344265  1.54404187 2.31224439 2.04817855 1.02618683 2.53556149
 3.02295749 2.23849175 1.3069859  2.44947836], Cost: 0.0005
Current parameters: [4.03664806 1.54385264 2.31189866 2.04652272 1.02848382 2.53576587
 3.02279384 2.23850559 1.30677407 2.44805129], Cost: 0.0003
Current parameters: [4.03408197 1.54179733 2.30923757 2.04320207 1.02763444 2.53254987
 3.02556676 2.23843117 1.30413062 2.44552712], Cost: 0.0003
Current parameters: [4.03764634 1.54404664 2.3125161  2.04630005 1.0261063  2.53558689
 3.02200938 2.23727053 1.30767446 2.44578024], Cost: 0.0002
Current parameters: [4.03824084 1.5407172  2.31233571 2.04986632 1.02654086 2.53975456
 3.02381177 2.23696779 1.30496568 2.442846  ], Cost: 0.0004
Current parameters: [4.03869329 1.54679705 2.31243214 2.04646484 1.02548492 2.53586521
 3.02412321 2.23721457 1.30640074 2.44586029], Cost: 0.0002
Current parameters: [4.03701072 1.54806413 2.31554412 2.04263218 1.02764705 2.53850921
 3.02665736 2.23671537 1.30960709 2.44396094], Cost: 0.0004
Current parameters: [4.0403663  1.54502458 2.31266737 2.04518706 1.02403901 2.53648628
 3.02538631 2.23747092 1.30680832 2.44768415], Cost: 0.0004
Current parameters: [4.04053928 1.54934901 2.30721976 2.04556906 1.02749448 2.53614559
 3.02121701 2.23406893 1.30631007 2.44503723], Cost: 0.0002
Current parameters: [4.03894987 1.54546888 2.31209274 2.04820651 1.02598117 2.53801556
 3.02486129 2.2368399  1.30506593 2.44414472], Cost: 0.0001
Current parameters: [4.03872832 1.54548764 2.3129003  2.04758899 1.02591788 2.53832838
 3.02419322 2.23610652 1.30383708 2.44451305], Cost: 0.0002
Current parameters: [4.03949498 1.54607106 2.31167143 2.04797632 1.0257384  2.53854787
 3.02372384 2.24003524 1.30446191 2.44267593], Cost: 0.0003
Current parameters: [4.03973267 1.54593093 2.31286715 2.04905464 1.02630767 2.53864812
 3.02420111 2.23685319 1.30568531 2.44473038], Cost: 0.0002
Current parameters: [4.03984118 1.54484481 2.31254966 2.04933357 1.0273385  2.53541598
 3.02611528 2.23590788 1.3048385  2.44291884], Cost: 0.0001
```

```
Current parameters: [4.03918337 1.5448656  2.31387204 2.04887304 1.0276224  2.53577751
 3.02688016 2.23627559 1.30536041 2.44245318], Cost: 0.0001
Current parameters: [4.03948459 1.54558711 2.31043256 2.04867974 1.02861085 2.5362127
 3.02676033 2.23387259 1.30622598 2.44195251], Cost: 0.0001
Current parameters: [4.04012983 1.5449574  2.31288469 2.04937477 1.02596057 2.53530939
 3.0257445  2.2352994  1.30530045 2.44193213], Cost: 0.0001
Current parameters: [4.03742882 1.54482121 2.31189706 2.05176367 1.02639359 2.53490077
 3.02714691 2.23579914 1.30474667 2.44384874], Cost: 0.0000
Current parameters: [4.03811291 1.54598513 2.31188012 2.05171311 1.02594107 2.53510073
 3.02831098 2.23579807 1.30416524 2.44407517], Cost: 0.0001
Current parameters: [4.0384303  1.54189504 2.31128958 2.0514387  1.0256281  2.53508742
 3.02867193 2.23579371 1.3054479  2.44518308], Cost: 0.0001
Current parameters: [4.03720906 1.54470744 2.31096712 2.0515536  1.02606633 2.53515361
 3.02731975 2.23713823 1.30463449 2.44293339], Cost: 0.0001
Current parameters: [4.03758905 1.54530676 2.31278309 2.05364254 1.02842385 2.5329894
 3.02575312 2.23539711 1.30401143 2.4443606 ], Cost: 0.0000
Current parameters: [4.03669112 1.54445566 2.3120186  2.0512353  1.02635296 2.53514133
 3.02689631 2.23507627 1.3032643  2.44382365], Cost: 0.0001
Current parameters: [4.03721814 1.54494574 2.31179028 2.05123915 1.02627525 2.53415613
 3.02722881 2.23582587 1.30485212 2.44399097], Cost: 0.0000
Current parameters: [4.03691156 1.54564488 2.31182833 2.0510862  1.02604831 2.53462134
 3.02629866 2.236126   1.30553473 2.44522301], Cost: 0.0000
Current parameters: [4.03703441 1.54505375 2.31132007 2.05108394 1.02697103 2.53435642
 3.02749815 2.23564018 1.30499023 2.44410381], Cost: 0.0000
Current parameters: [4.03726027 1.54536459 2.31161529 2.05158065 1.02576191 2.53386469
 3.02690713 2.23464234 1.30588396 2.44323408], Cost: 0.0001
Current parameters: [4.03688804 1.54488843 2.31247212 2.05132613 1.02646533 2.53447654
 3.02748405 2.2359098  1.30521038 2.44381889], Cost: 0.0000
Current parameters: [4.03701571 1.5454274  2.31230711 2.05040333 1.02634157 2.53271438
 3.02759994 2.23637322 1.30474496 2.44420115], Cost: 0.0001
Current parameters: [4.03756793 1.54429176 2.31181311 2.05141119 1.02621359 2.5340641
 3.02744212 2.23578804 1.3051364  2.44447417], Cost: 0.0000
Current parameters: [4.03695948 1.54498692 2.31171689 2.05159307 1.02624063 2.53402384
 3.02731254 2.23585459 1.30478917 2.44408411], Cost: 0.0000
Current parameters: [4.03735408 1.54510261 2.31193766 2.05201486 1.02680733 2.53385261
 3.02688416 2.23597099 1.30472447 2.44400516], Cost: 0.0000
Current parameters: [4.0372846  1.54508069 2.31211801 2.05196539 1.02685169 2.53384448
 3.02682999 2.23562469 1.30450571 2.4441645 ], Cost: 0.0000
Current parameters: [4.03696819 1.5447002  2.3120865  2.05203417 1.02686992 2.5341149
 3.02628104 2.2358456  1.30403514 2.44392364], Cost: 0.0000
Current parameters: [4.0374606  1.54517629 2.31207189 2.05216142 1.02676066 2.53420066
```



```
 3.02692214 2.23556326 1.30440416 2.44416935], Cost: 0.0000
Current parameters: [4.03745703 1.54562964 2.31246761 2.05184236 1.02684225 2.53347502
 3.02711108 2.23591286 1.3040914  2.44428675], Cost: 0.0000
Current parameters: [4.0372846  1.54508069 2.31211801 2.05196539 1.02685169 2.53384448
 3.02682999 2.23562469 1.30450571 2.4441645 ], Cost: 0.0000

   Return from subroutine COBYLA because the MAXFUN limit has been reached.

   NFVALS =  100   F = 6.024475E-06    MAXCV = 0.000000E+00
   X = 4.037285E+00   1.545081E+00   2.312118E+00   2.051965E+00   1.026852E+00
       2.533844E+00   3.026830E+00   2.235625E+00   1.304506E+00   2.444165E+00
Simulating for QAOA depth p = 6
Current parameters: [2.29312569 1.86212495 2.46841542 0.3812062  1.59734156 0.9581625
 1.83863368 2.2441369  2.05583342 2.29195748 3.02106112 2.65451994], Cost: 1.9709
Current parameters: [2.29312569 2.86212495 2.46841542 0.3812062  1.59734156 0.9581625
 1.83863368 2.2441369  2.05583342 2.29195748 3.02106112 2.65451994], Cost: 1.2505
Current parameters: [2.29312569 2.86212495 3.46841542 0.3812062  1.59734156 0.9581625
 1.83863368 2.2441369  2.05583342 2.29195748 3.02106112 2.65451994], Cost: 2.0858
Current parameters: [2.29312569 2.86212495 2.46841542 1.3812062  1.59734156 0.9581625
 1.83863368 2.2441369  2.05583342 2.29195748 3.02106112 2.65451994], Cost: 2.1141
Current parameters: [2.29312569 2.86212495 2.46841542 0.3812062  2.59734156 0.9581625
 1.83863368 2.2441369  2.05583342 2.29195748 3.02106112 2.65451994], Cost: 2.8666
Current parameters: [2.29312569 2.86212495 2.46841542 0.3812062  1.59734156 1.9581625
 1.83863368 2.2441369  2.05583342 2.29195748 3.02106112 2.65451994], Cost: 2.4579
Current parameters: [2.29312569 2.86212495 2.46841542 0.3812062  1.59734156 0.9581625
 2.83863368 2.2441369  2.05583342 2.29195748 3.02106112 2.65451994], Cost: 1.2164
Current parameters: [2.29312569 2.86212495 2.46841542 0.3812062  1.59734156 0.9581625
 2.83863368 3.2441369  2.05583342 2.29195748 3.02106112 2.65451994], Cost: 3.3598
Current parameters: [2.29312569 2.86212495 2.46841542 0.3812062  1.59734156 0.9581625
 2.83863368 2.2441369  3.05583342 2.29195748 3.02106112 2.65451994], Cost: 3.5583
Current parameters: [2.29312569 2.86212495 2.46841542 0.3812062  1.59734156 0.9581625
 2.83863368 2.2441369  2.05583342 3.29195748 3.02106112 2.65451994], Cost: 2.4431
Current parameters: [2.29312569 2.86212495 2.46841542 0.3812062  1.59734156 0.9581625
 2.83863368 2.2441369  2.05583342 2.29195748 4.02106112 2.65451994], Cost: 1.2244
Current parameters: [2.29312569 2.86212495 2.46841542 0.3812062  1.59734156 0.9581625
 2.83863368 2.2441369  2.05583342 2.29195748 3.02106112 3.65451994], Cost: 1.8287
Current parameters: [2.33556558 3.03182136 2.27166787 0.17778449 1.21667069 0.67376753
 2.84665587 1.73928094 1.50420678 2.00302213 3.01919387 2.51030413], Cost: 0.5035
Current parameters: [2.35649024 3.11548888 2.17466301 0.07748898 2.1066907  0.53354876
 2.85061115 1.49036558 1.2322315  1.86056477 3.01827324 2.43919963], Cost: 2.9170
Current parameters: [2.83008957 3.03182136 2.27166787 0.17778449 1.21667069 0.74756468
```



```
 2.84665587 1.73928094 1.50420678 2.00302213 3.01919387 2.51030413], Cost: 0.1632
Current parameters: [2.74295576 3.08662881 2.20812363 0.11208467 1.02149053 1.68230586
 2.84924683 1.57622584 1.326046   1.90970366 3.0185908  2.46372624], Cost: 2.4354
Current parameters: [2.82781831 3.03182136 2.27166787 0.66507325 1.23704307 0.76278466
 2.89118643 1.69475038 1.45967623 1.95849157 2.97466331 2.46577357], Cost: 0.8361
Current parameters: [3.02784593 3.15453473 2.1293929  0.06461795 0.63044778 0.20791685
 2.5262954  1.70036367 1.43146802 2.12024455 3.34400519 2.73217827], Cost: 1.3282
Current parameters: [2.86045861 3.05513905 2.24463314 0.12762805 1.16993246 0.69332472
 2.9271053  1.59056244 1.9299166  1.88397291 2.93959018 2.41114057], Cost: 0.8074
Current parameters: [2.83008957 3.22113271 2.4349505  0.17778449 1.21667069 0.74756468
 2.84665587 1.73928094 1.50420678 2.00302213 3.01919387 2.51030413], Cost: 0.3436
Current parameters: [2.85501641 2.7055263  2.62345427 0.1290674  1.16045337 0.69123442
 2.85921272 1.67609057 1.44287551 1.96183674 3.00726428 2.48386746], Cost: 0.3377
Current parameters: [2.83113969 3.03602029 2.2667996  0.17275108 1.20725147 0.74052769
 2.82606705 1.84981273 1.49055747 1.78252562 3.03978269 2.53089295], Cost: 0.6021
Current parameters: [2.87190047 3.00722293 2.24820542 0.11606221 1.16966318 0.68435892
 3.00934924 2.05889581 1.47554649 2.21634475 2.85772986 2.32040789], Cost: 0.8703
Current parameters: [2.8330171  3.04352713 2.2580961  0.16375233 1.19041177 0.72794694
 3.0654279  1.70445565 1.46615522 1.98309118 3.07659141 2.59206769], Cost: 0.4440
Current parameters: [2.83950239 2.98772832 2.30063666 0.19177008 1.27203311 0.77695558
 2.78265279 1.8129528  1.60124761 2.09052408 2.78558143 2.91258682], Cost: 1.4818
Current parameters: [2.82177445 2.99857326 2.31021603 0.2176403  1.23628611 0.80328534
 2.84508411 1.83819585 1.61228533 2.05963236 3.19033916 2.5385599 ], Cost: 1.6070
Current parameters: [2.88673076 3.05819791 2.1868483  0.05099761 1.50995519 0.59439696
 2.78485985 1.5200116  1.30512909 1.98853613 2.95643577 2.37736622], Cost: 1.2537
Current parameters: [2.81621789 3.04174452 2.24322911 0.13617671 1.0841174  0.91120245
 2.82744233 1.66580054 1.43656515 1.99562514 2.96370485 2.4673649 ], Cost: 0.3066
Current parameters: [2.85882128 3.05473713 2.23615652 0.18760774 1.05492318 0.59235648
 2.77438997 1.71084608 1.47398375 2.03197554 3.05265096 2.53556179], Cost: 0.5761
Current parameters: [2.7074179  3.02979534 2.27217315 0.17374631 1.21505799 0.72729277
 2.84825709 1.73668238 1.50310011 2.00366597 3.00934654 2.504429  ], Cost: 0.1637
Current parameters: [2.83283153 3.03182822 2.27165992 0.17735922 1.20079068 0.74305598
 2.85913758 1.72677915 1.55659982 1.99052907 3.00316505 2.49781692], Cost: 0.1931
Current parameters: [2.84088838 3.03792372 2.25016962 0.14228796 1.28740956 0.71856692
 2.83989652 1.67396842 1.4786346  1.99100573 2.99034049 2.46601136], Cost: 0.1792
Current parameters: [2.82705992 3.0689453  2.22862605 0.19961443 1.2240059  0.75474352
 2.84663456 1.74530432 1.51000979 2.00643705 3.01914082 2.51197074], Cost: 0.1527
Current parameters: [2.82912045 3.04463657 2.25245156 0.30673572 1.22702347 0.75062603
 2.859486   1.72461508 1.4833244  1.99579402 2.98840902 2.48751418], Cost: 0.2607
Current parameters: [2.82329596 3.0666507  2.23128643 0.20700358 1.23257243 0.7633008
 2.82732139 1.71370853 1.51939278 1.99531125 3.02070973 2.5577195 ], Cost: 0.0922
```



```
Current parameters: [2.82986092 3.0628287  2.22658587 0.18927107 1.24554666 0.75734699
 2.83110984 1.74992947 1.51407518 2.02542662 2.92018268 2.60929244], Cost: 0.1933
Current parameters: [2.82541559 3.06601507 2.23202339 0.21207217 1.22406839 0.75218613
 2.77051222 1.7214847  1.52724107 2.00256706 3.01408623 2.54244204], Cost: 0.0885
Current parameters: [2.83851463 3.08047389 2.20682314 0.20732778 1.15673316 0.67012029
 2.77859265 1.6856931  1.49696562 2.01128715 3.02659095 2.57112335], Cost: 0.1876
Current parameters: [2.82567333 3.11400829 2.27133421 0.21083678 1.22346037 0.75045898
 2.77056094 1.71841868 1.523891   2.00081234 3.0087813  2.54156621], Cost: 0.1115
Current parameters: [2.81415669 3.05805525 2.23365907 0.20654011 1.26837781 0.7833079
 2.75142983 1.69613805 1.5622219  2.07560004 3.08064462 2.56425088], Cost: 0.4982
Current parameters: [2.82214012 3.06798547 2.2258369  0.20286363 1.19423383 0.79042243
 2.76763512 1.69623052 1.51268236 2.01746465 2.99974988 2.5309975 ], Cost: 0.0602
Current parameters: [2.81592085 3.07061462 2.20600352 0.1803655  1.17777934 0.82577681
 2.74239224 1.71354851 1.48576596 1.91053531 2.99853895 2.53888658], Cost: 0.2246
Current parameters: [2.82396243 3.03891709 2.22345309 0.2469985  1.20779558 0.77743348
 2.75066899 1.70647508 1.51395075 2.00605292 2.98964839 2.51981364], Cost: 0.0809
Current parameters: [2.81921969 3.09332125 2.26199925 0.26290445 1.16855697 0.82179997
 2.8172739  1.63656146 1.46900279 2.02453852 2.97898305 2.52887821], Cost: 0.1660
Current parameters: [2.82243082 3.05781913 2.22114019 0.19940297 1.21166709 0.79284442
 2.78063255 1.69811523 1.51583565 2.01712792 2.96545457 2.57682632], Cost: 0.0446
Current parameters: [2.83136286 3.07716215 2.22241624 0.17575903 1.18349845 0.78636613
 2.75315774 1.74892037 1.49527055 2.06863782 2.88603784 2.5443344 ], Cost: 0.2495
Current parameters: [2.82434067 3.06132544 2.21741403 0.18673313 1.19025187 0.79453438
 2.78697054 1.68085817 1.56505317 2.01406002 2.95823544 2.55602817], Cost: 0.0907
Current parameters: [2.82706715 3.04058938 2.23494044 0.26026043 1.2440147  0.76544636
 2.81064921 1.70369428 1.53850842 1.96137196 2.9097835  2.62111364], Cost: 0.0454
Current parameters: [2.82898726 3.05543232 2.20247065 0.19876902 1.18286517 0.74871409
 2.77869693 1.6834964  1.50445904 2.01725558 2.9734244  2.5947472 ], Cost: 0.0638
Current parameters: [2.85317156 3.0583765  2.22083371 0.20011434 1.21266307 0.79766637
 2.78017581 1.69821694 1.51589778 2.01686631 2.96767308 2.57792293], Cost: 0.0600
Current parameters: [2.8252445  3.06068249 2.21037133 0.18219925 1.24727845 0.77812881
 2.777263   1.66516911 1.5026809  2.01063368 2.9503589  2.55552898], Cost: 0.0382
Current parameters: [2.82587092 3.0566632  2.2142033  0.19051251 1.25685817 0.77139648
 2.78066521 1.66081042 1.51107134 2.03556517 2.95292904 2.55868622], Cost: 0.0498
Current parameters: [2.80615024 3.04829461 2.19733907 0.17583648 1.27033581 0.79765404
 2.75972786 1.65595138 1.50229102 1.99281096 2.97698061 2.58461565], Cost: 0.0238
Current parameters: [2.8079269  3.04313888 2.19062332 0.16475059 1.27690239 0.78801023
 2.74723163 1.67542318 1.51022962 1.9945726  2.97057508 2.58510618], Cost: 0.0138
Current parameters: [2.78643307 3.04736533 2.18550024 0.15965599 1.280739   0.80619578
 2.72349283 1.68697419 1.48015953 2.000147   2.93573292 2.59861733], Cost: 0.0279
Current parameters: [2.8066639  3.06162299 2.16923058 0.17397309 1.2780834  0.7916977
```

```
 2.7479297  1.67642967 1.51855917 1.99589392 2.96974228 2.5835936 ], Cost: 0.0179
Current parameters: [2.79501923 3.03071118 2.19216464 0.14085787 1.26283453 0.7983635
 2.73809666 1.65663215 1.5530356  1.98196971 2.9544554  2.58071512], Cost: 0.0340
Current parameters: [2.80677201 3.04586273 2.18546686 0.14853853 1.26886812 0.79549512
 2.74263642 1.67457431 1.50415314 2.00885062 2.98397812 2.57435664], Cost: 0.0164
Current parameters: [2.79553207 3.02179408 2.17173262 0.15130623 1.29085193 0.7886939
 2.78973681 1.69825125 1.5023247  1.9829361  2.97415641 2.58002498], Cost: 0.0063
Current parameters: [2.79561593 3.04019755 2.18101163 0.13586337 1.29467883 0.78587817
 2.79887821 1.69923535 1.50465535 1.9837705  2.97388632 2.59416632], Cost: 0.0068
Current parameters: [2.77628523 3.033573   2.18917051 0.15545531 1.30973971 0.7718512
 2.77624634 1.72029585 1.49992295 1.95794272 2.99930706 2.55756619], Cost: 0.0151
Current parameters: [2.79909852 3.01511492 2.16892126 0.15103222 1.28320761 0.76380471
 2.78375133 1.69626672 1.49788695 1.97871608 2.97602357 2.59237781], Cost: 0.0076
Current parameters: [2.76845904 3.03291522 2.13166442 0.13815265 1.27230446 0.79389584
 2.80624923 1.68634666 1.48623056 1.98056248 2.98816587 2.5750475 ], Cost: 0.0129
Current parameters: [2.81913914 3.0190886  2.16296105 0.14508463 1.29715525 0.79750089
 2.78951793 1.70295752 1.49701485 1.97361432 2.98001809 2.58273774], Cost: 0.0028
Current parameters: [2.81822375 3.0167123  2.16587447 0.15180516 1.28076155 0.80656452
 2.79125847 1.72104636 1.50209128 1.97418646 2.98894933 2.59411409], Cost: 0.0071
Current parameters: [2.81498053 3.01513071 2.16109187 0.14600602 1.30511945 0.80001918
 2.78650365 1.69962305 1.49931486 1.9766636  2.98679641 2.59024308], Cost: 0.0025
Current parameters: [2.82673337 3.01035913 2.15564137 0.14589867 1.31732786 0.79312126
 2.79778333 1.70556789 1.4993306  1.99646683 2.98567117 2.58447137], Cost: 0.0205
Current parameters: [2.8173316  3.0132571  2.1610134  0.14347075 1.30249139 0.79805058
 2.78996859 1.69528419 1.51128837 1.97425385 2.99175384 2.58659515], Cost: 0.0063
Current parameters: [2.80870903 3.00660834 2.16015465 0.13327572 1.30704393 0.80864136
 2.77509507 1.70061731 1.50037085 1.96382287 2.96965114 2.59594493], Cost: 0.0060
Current parameters: [2.81433079 3.00768147 2.16897957 0.13986792 1.302686   0.80157627
 2.78949068 1.69718197 1.49293499 1.97815358 2.99109421 2.58831588], Cost: 0.0012
Current parameters: [2.81747913 3.0107062  2.17694703 0.15573341 1.30354214 0.80780315
 2.79678584 1.68203641 1.48349813 1.96478003 2.98961852 2.59539467], Cost: 0.0103
Current parameters: [2.81927519 3.00919852 2.16802792 0.13880409 1.29618978 0.8054613
 2.78474078 1.68990696 1.49360735 1.98625661 2.98828218 2.59124065], Cost: 0.0039
Current parameters: [2.81296628 3.01902727 2.17526852 0.13614553 1.30518615 0.80050048
 2.76897166 1.70078815 1.48662941 1.97389005 3.00506421 2.57841513], Cost: 0.0109
Current parameters: [2.80826642 3.01193337 2.15744037 0.13955485 1.30149206 0.80319424
 2.79130788 1.69543887 1.48787827 1.97839688 2.99498056 2.59053329], Cost: 0.0007
Current parameters: [2.80708671 3.00339346 2.15662497 0.1212365  1.28814988 0.80334111
 2.80394155 1.69279864 1.48536707 1.97078916 2.99397366 2.57803528], Cost: 0.0039
Current parameters: [2.80720167 3.00639148 2.16286765 0.14141797 1.29775751 0.80566985
 2.79395859 1.70632821 1.48962045 1.97855656 2.9982167  2.59502274], Cost: 0.0029
```



```
Current parameters: [2.80779616 3.02551651 2.14106641 0.14354723 1.28365934 0.81340399
 2.78614481 1.69391511 1.48726239 1.97769329 2.99747533 2.5975669 ], Cost: 0.0026
Current parameters: [2.80374485 3.00827469 2.15458829 0.13492983 1.30544717 0.80667954
 2.78551998 1.69659779 1.48857402 1.97368994 2.98639379 2.5952002 ], Cost: 0.0019
Current parameters: [2.80855529 3.00703893 2.15502073 0.14315333 1.29993312 0.80170145
 2.78864591 1.69503636 1.48694769 1.97783608 2.99520544 2.58828506], Cost: 0.0003
Current parameters: [2.81077149 3.00715246 2.15462286 0.14013061 1.29702055 0.78983007
 2.78744974 1.69431225 1.48473567 1.97572603 2.99605504 2.59656069], Cost: 0.0017
Current parameters: [2.80250583 3.00789151 2.15744311 0.14447527 1.29757432 0.79995151
 2.78825292 1.69349946 1.48827328 1.97898641 2.99395094 2.58773739], Cost: 0.0003
Current parameters: [2.80069791 3.00817147 2.15090928 0.14649233 1.30212139 0.7973574
 2.79254225 1.69521095 1.48429052 1.98939466 2.99010906 2.58521806], Cost: 0.0014
Current parameters: [2.80312646 3.00664658 2.15770199 0.14782095 1.29748839 0.80105339
 2.79287017 1.69051869 1.48779393 1.97713758 2.99159346 2.59042221], Cost: 0.0002
Current parameters: [2.80160631 3.01039488 2.1617787  0.15257184 1.30204746 0.80235295
 2.78990098 1.68756756 1.47831406 1.97178669 2.99504347 2.58615751], Cost: 0.0071
Current parameters: [2.8029407  3.00310754 2.1602157  0.14406909 1.29870103 0.79915319
 2.79577023 1.69001182 1.48711506 1.97600943 2.99082878 2.58695613], Cost: 0.0002
Current parameters: [2.80249297 3.0040664  2.1528753  0.13881866 1.28760988 0.80278751
 2.79930846 1.68833227 1.48704135 1.97470807 2.99048125 2.58409425], Cost: 0.0017
Current parameters: [2.80137307 3.00038617 2.15908373 0.14272084 1.30040839 0.80037691
 2.7958546  1.68645207 1.48901389 1.97797656 2.99524371 2.58895975], Cost: 0.0008
Current parameters: [2.80186062 3.00375157 2.15863632 0.14461916 1.29978501 0.79819415
 2.79684201 1.69101695 1.48851705 1.97412294 2.99172649 2.58641173], Cost: 0.0002
Current parameters: [2.806268   3.00313906 2.16099066 0.14498071 1.29868401 0.79806893
 2.79504281 1.6888964  1.49308076 1.97717681 2.98834794 2.58538278], Cost: 0.0005
Current parameters: [2.80208118 3.0029398  2.15898584 0.14285702 1.29997899 0.79962911
 2.79421743 1.68903503 1.48706934 1.9749717  2.98860361 2.58746096], Cost: 0.0006
Current parameters: [2.80176025 2.99945581 2.16148247 0.14426783 1.2978554  0.80093951
 2.79618279 1.69550531 1.48870801 1.97629812 2.98966401 2.58949193], Cost: 0.0006
Current parameters: [2.80360045 3.00296185 2.16127437 0.14333422 1.29740477 0.79778264
 2.79475707 1.68958383 1.48747435 1.97381057 2.99165057 2.58860952], Cost: 0.0003
Current parameters: [2.80354636 3.00308449 2.15792652 0.14366052 1.29879691 0.7935248
 2.79722409 1.69079941 1.48558853 1.97935674 2.98960957 2.58936806], Cost: 0.0008
Current parameters: [2.80374049 3.00430236 2.16360468 0.1484925  1.30003128 0.79948837
 2.79534231 1.69038406 1.48380892 1.97501354 2.99231703 2.58355387], Cost: 0.0011
Current parameters: [2.80308972 3.00345443 2.15876456 0.14332468 1.29575053 0.8001414
 2.79681536 1.68983205 1.48694152 1.97577095 2.99086248 2.58570653], Cost: 0.0004
Current parameters: [2.80142661 3.00337856 2.16084935 0.14435646 1.29813051 0.79873393
 2.79570371 1.68963281 1.48745713 1.97630335 2.99051284 2.58684606], Cost: 0.0002
Current parameters: [2.8031138  3.00562882 2.16150956 0.1424447  1.2993923  0.80003775
```



```
 2.79725743 1.69036749 1.48746655 1.97628802 2.9908072  2.58782738], Cost: 0.0002
Current parameters: [2.80326454 3.00531799 2.16159564 0.14331327 1.29940184 0.80030739
 2.79838862 1.68961516 1.48731355 1.97580967 2.99023087 2.58848372], Cost: 0.0002
Current parameters: [2.8037     3.00667633 2.16326385 0.14410551 1.30016508 0.80033511
 2.79694783 1.69088707 1.48585085 1.97593123 2.99177525 2.58602302], Cost: 0.0003
Current parameters: [2.80278716 3.00515972 2.16176118 0.14233486 1.29963509 0.80123466
 2.79705831 1.68967734 1.48811031 1.97609583 2.99177681 2.58771088], Cost: 0.0002
Current parameters: [2.8031138  3.00562882 2.16150956 0.1424447  1.2993923  0.80003775
 2.79725743 1.69036749 1.48746655 1.97628802 2.9908072  2.58782738], Cost: 0.0002

   Return from subroutine COBYLA because the MAXFUN limit has been reached.

   NFVALS =  100   F = 1.652859E-04    MAXCV = 0.000000E+00
   X = 2.803114E+00   3.005629E+00   2.161510E+00   1.424447E-01   1.299392E+00
       8.000378E-01   2.797257E+00   1.690367E+00   1.487467E+00   1.976288E+00
       2.990807E+00   2.587827E+00
Simulating for QAOA depth p = 7
Current parameters: [1.30702099 1.77101869 0.78472873 2.27153641 2.55139704 1.57322174
 0.95287078 1.03495742 0.36727812 2.29896069 0.83255828 2.40605181
 0.14356907 1.57591722], Cost: 2.0233
Current parameters: [1.30702099 2.77101869 0.78472873 2.27153641 2.55139704 1.57322174
 0.95287078 1.03495742 0.36727812 2.29896069 0.83255828 2.40605181
 0.14356907 1.57591722], Cost: 1.5741
Current parameters: [1.30702099 2.77101869 1.78472873 2.27153641 2.55139704 1.57322174
 0.95287078 1.03495742 0.36727812 2.29896069 0.83255828 2.40605181
 0.14356907 1.57591722], Cost: 1.4823
Current parameters: [1.30702099 2.77101869 1.78472873 3.27153641 2.55139704 1.57322174
 0.95287078 1.03495742 0.36727812 2.29896069 0.83255828 2.40605181
 0.14356907 1.57591722], Cost: 0.4963
Current parameters: [1.30702099 2.77101869 1.78472873 3.27153641 3.55139704 1.57322174
 0.95287078 1.03495742 0.36727812 2.29896069 0.83255828 2.40605181
 0.14356907 1.57591722], Cost: 2.4080
Current parameters: [1.30702099 2.77101869 1.78472873 3.27153641 2.55139704 2.57322174
 0.95287078 1.03495742 0.36727812 2.29896069 0.83255828 2.40605181
 0.14356907 1.57591722], Cost: 1.0833
Current parameters: [1.30702099 2.77101869 1.78472873 3.27153641 2.55139704 1.57322174
 1.95287078 1.03495742 0.36727812 2.29896069 0.83255828 2.40605181
 0.14356907 1.57591722], Cost: 0.5079
Current parameters: [1.30702099 2.77101869 1.78472873 3.27153641 2.55139704 1.57322174
 0.95287078 2.03495742 0.36727812 2.29896069 0.83255828 2.40605181
 0.14356907 1.57591722], Cost: 2.7381
```



```
Current parameters: [1.30702099 2.77101869 1.78472873 3.27153641 2.55139704 1.57322174
 0.95287078 1.03495742 1.36727812 2.29896069 0.83255828 2.40605181
 0.14356907 1.57591722], Cost: 1.7768
Current parameters: [1.30702099 2.77101869 1.78472873 3.27153641 2.55139704 1.57322174
 0.95287078 1.03495742 0.36727812 3.29896069 0.83255828 2.40605181
 0.14356907 1.57591722], Cost: 2.2313
Current parameters: [1.30702099 2.77101869 1.78472873 3.27153641 2.55139704 1.57322174
 0.95287078 1.03495742 0.36727812 2.29896069 1.83255828 2.40605181
 0.14356907 1.57591722], Cost: 1.9806
Current parameters: [1.30702099 2.77101869 1.78472873 3.27153641 2.55139704 1.57322174
 0.95287078 1.03495742 0.36727812 2.29896069 0.83255828 3.40605181
 0.14356907 1.57591722], Cost: 1.0447
Current parameters: [1.30702099 2.77101869 1.78472873 3.27153641 2.55139704 1.57322174
 0.95287078 1.03495742 0.36727812 2.29896069 0.83255828 2.40605181
 1.14356907 1.57591722], Cost: 2.9187
Current parameters: [1.30702099 2.77101869 1.78472873 3.27153641 2.55139704 1.57322174
 0.95287078 1.03495742 0.36727812 2.29896069 0.83255828 2.40605181
 0.14356907 2.57591722], Cost: 1.3899
Current parameters: [ 1.34767143  2.86260004  1.80344013  3.47251194  2.16172569  1.45357055
  0.95051157  0.57799902  0.1062696   1.94529072  0.52999733  2.29426272
 -0.35020891  1.39376923], Cost: 1.5255
Current parameters: [ 1.32734621  2.81680937  1.79408443  3.37202417  2.35656137  1.51339615
  0.95169117  0.80647822  0.23677386  2.12212571  0.6812778   2.35015726
 -0.10331992  1.48484323], Cost: 1.7549
Current parameters: [1.55567166 2.77101869 1.78472873 3.27153641 2.57733623 1.57322174
 0.95287078 1.03495742 0.36727812 2.29896069 0.83255828 2.40605181
 0.14356907 1.57591722], Cost: 0.3634
Current parameters: [1.52055257 2.78376744 1.78733348 3.29951358 3.05937632 1.55656548
 0.95254236 0.97134569 0.33094395 2.24972742 0.79043973 2.39049
 0.0748318  1.55056098], Cost: 1.3219
Current parameters: [1.55567166 2.97480739 1.88712542 3.16913972 2.57733623 1.57322174
 0.95287078 1.03495742 0.36727812 2.29896069 0.83255828 2.40605181
 0.14356907 1.57591722], Cost: 0.8318
Current parameters: [1.56294365 2.46607136 2.06855018 2.99981607 2.56515842 1.56663094
 0.95274082 1.00978658 0.35290087 2.27947934 0.81589218 2.39989408
 0.11637008 1.56588388], Cost: 0.1961
Current parameters: [1.56294365 2.46607136 1.89992144 2.83118734 2.56515842 1.56663094
 0.95274082 1.00978658 0.35290087 2.27947934 0.81589218 2.39989408
 0.04134541 1.56588388], Cost: 0.5077
Current parameters: [1.59059444 2.38749637 1.98703447 2.95721424 2.51885384 1.54157028
 0.95224669 0.91407773 0.29823326 2.205404   0.75252151 2.37648012
```

```
 0.57263084 1.52773342], Cost: 1.7646
Current parameters: [1.56294365 2.46607136 2.06855018 2.99981607 2.56515842 1.56663094
 0.95274082 1.16280167 0.35290087 2.08177657 0.81589218 2.39989408
 0.11637008 1.56588388], Cost: 0.8280
Current parameters: [1.59507698 2.37475837 2.11095388 3.08744208 2.51134729 1.53750764
 0.95216659 1.27962649 0.28937095 2.64689472 0.74224833 2.37268442
 0.03013909 1.52154875], Cost: 2.0171
Current parameters: [1.56294365 2.46607136 2.06855018 2.99981607 2.56515842 1.56663094
 0.95274082 1.00978658 0.16360412 2.27947934 0.97919172 2.39989408
 0.11637008 1.56588388], Cost: 0.4610
Current parameters: [1.58301951 2.40902198 2.09504263 3.05456195 2.53153898 1.54843565
 0.95238206 0.88790463 0.68502381 2.28421546 1.15061586 2.38289437
 0.06249576 1.53818473], Cost: 2.7261
Current parameters: [1.56294365 2.46607136 2.06855018 2.99981607 2.56515842 1.35768033
 0.95274082 1.00978658 0.35290087 2.27947934 0.81589218 2.39989408
 0.11637008 1.70314146], Cost: 0.3551
Current parameters: [1.57188202 2.44067127 2.0803454  3.02419057 2.55019004 1.84299651
 0.95258109 0.95552111 0.32094982 2.281588   0.75647146 2.3923253
 0.09238363 1.97061364], Cost: 1.5575
Current parameters: [1.56294365 2.46607136 2.06855018 2.99981607 2.56515842 1.56663094
 0.70279648 1.00978658 0.35290087 2.27947934 0.81589218 2.40516894
 0.11637008 1.56588388], Cost: 0.2063
Current parameters: [1.56725776 2.453812   2.07424315 3.01158043 2.55793392 1.55891056
 0.96350546 0.98359533 0.33747968 2.28049708 0.78721275 2.89705821
 0.10479301 1.54641415], Cost: 1.9199
Current parameters: [1.69768739 2.41303743 1.98325177 3.11352445 2.4757659  1.55115762
 0.95243573 0.95069259 0.31914718 2.23374264 0.77676492 2.38543749
 0.05251458 1.54232845], Cost: 0.1231
Current parameters: [ 1.51285885  2.4925964   2.15503424  3.06760937  2.41299344  1.48986924
  0.95083391  0.73001122  0.19122315  2.14945568  0.58457388  2.25707925
 -0.11798377  1.41766843], Cost: 1.8826
Current parameters: [1.71490457 2.38323755 1.9867238  3.15815334 2.7073128  1.56407952
 0.98192715 0.91277266 0.32592326 2.24220226 0.73884612 2.36579734
 0.04403226 1.5075415 ], Cost: 0.6641
Current parameters: [ 1.8205482   2.37079905  1.7034311   3.1631266   2.45629523  1.39268695
  0.72928332  1.01878182  0.14202172  2.11972335  0.85565021  2.37920781
 -0.02583547  1.66747518], Cost: 0.7090
Current parameters: [1.85058855 2.602469   2.017856   3.07667847 2.48362496 1.54945944
 0.94918273 0.95599357 0.31877794 2.24919322 0.77344239 2.37459428
 0.0668569  1.54001991], Cost: 0.6956
Current parameters: [1.63706729 2.48141573 1.92968931 3.17491022 2.47568848 1.55819751
```



```
 0.96100817 0.95640565 0.32832757 2.24907058 0.7742029  2.38286683
 0.06367037 1.53733635], Cost: 0.1285
Current parameters: [1.70598788 2.35278728 1.99236236 3.14103816 2.43238071 1.54063386
 0.95592691 1.10039733 0.29607235 2.39550757 0.7231924  2.33780001
 0.03654328 1.50425576], Cost: 0.2339
Current parameters: [1.69745867 2.40982249 1.98531824 3.11515917 2.47455251 1.60351832
 0.95356441 0.94466931 0.32097374 2.23278477 0.77439799 2.30009443
 0.05395614 1.61670337], Cost: 0.1621
Current parameters: [1.68871466 2.39576831 1.99808498 3.13883708 2.45423845 1.6452974
 0.95989628 0.9442749  0.28896013 2.27302563 0.71638603 2.55842595
 0.02534342 1.66510726], Cost: 0.1518
Current parameters: [1.69956058 2.40411524 1.96214878 3.09495534 2.47114237 1.55057611
 0.95510645 0.9385969  0.27577992 2.22783029 0.72231763 2.38270659
 0.15077281 1.53647492], Cost: 0.7363
Current parameters: [1.69523454 2.34774715 1.98278644 3.16062851 2.40880912 1.56379499
 0.94538558 0.912169   0.47457587 2.28030081 0.92597995 2.41399933
 0.03210588 1.54583655], Cost: 0.8017
Current parameters: [ 1.65075125  2.4241687   2.02162358  3.11278341  2.44547562  1.5349288
  0.94178202  0.91715062  0.29028515  2.2227313   0.74519343  2.36819506
 -0.0306348   1.52672194], Cost: 0.2066
Current parameters: [1.6933654  2.41471857 1.99013469 3.11660058 2.48097647 1.50214832
 0.96067032 0.94818297 0.32630072 2.2422045  0.77246116 2.38567374
 0.05754708 1.57635872], Cost: 0.0828
Current parameters: [1.71890086 2.39986256 1.92312119 3.13163003 2.4867019  1.47738967
 0.90774021 0.97775813 0.28381176 2.21230316 0.79728493 2.3892011
 0.01596699 1.60198135], Cost: 0.0267
Current parameters: [1.72450095 2.39765841 1.95662965 3.17340943 2.48298666 1.47674658
 0.89733958 0.98984642 0.27454155 2.19161037 0.80244566 2.3888195
 0.0297986  1.60100236], Cost: 0.0807
Current parameters: [ 1.69408605  2.40451962  1.92246833  3.10286529  2.49097929  1.47821361
  0.94425349  1.0559281   0.32465753  2.14830902  0.78105367  2.39778575
 -0.00568303  1.61327127], Cost: 0.2518
Current parameters: [1.7387947  2.39203252 1.91052756 3.14841817 2.47350381 1.47510516
 0.95665731 0.96903337 0.26969469 2.2055505  0.79938733 2.3860334
 0.00653923 1.59850358], Cost: 0.0561
Current parameters: [1.67894792 2.4130672  1.94741425 3.10112684 2.50719715 1.48097849
 0.93333408 0.99712388 0.2195557  2.23064494 0.87612675 2.39625014
 0.02156737 1.61239487], Cost: 0.0790
Current parameters: [1.68567191 2.35312555 1.91291503 3.14140241 2.48158445 1.47727621
 0.90806139 0.96857668 0.29517944 2.20844331 0.80858187 2.39022732
 0.02160271 1.60027459], Cost: 0.0264
```



```
Current parameters: [ 1.7169723   2.36670684  1.91167508  3.15190543  2.4493547   1.47006302
  0.88638063  0.9457646   0.35974214  2.24722213  0.88024748  2.40306178
 -0.01254615  1.6116229 ], Cost: 0.2992
Current parameters: [1.68459084 2.3660357  1.91095734 3.13693329 2.50335731 1.48042653
 0.90866368 0.93081622 0.30103487 2.17005895 0.81956662 2.40068677
 0.02500365 1.60769954], Cost: 0.0626
Current parameters: [ 1.69389761  2.33022548  1.91716528  3.17096167  2.58518109  1.48074079
  0.91575682  0.97279954  0.29995512  2.24624699  0.79184189  2.38329645
 -0.01539642  1.58721071], Cost: 0.0274
Current parameters: [1.68800427 2.35728508 1.90899094 3.13477016 2.4869185  1.45332137
 0.90621117 0.97007413 0.30217136 2.19854317 0.82303052 2.34762432
 0.02957066 1.56893765], Cost: 0.0606
Current parameters: [1.68698879 2.334758   1.87477415 3.12370415 2.49054484 1.45663429
 0.88274246 0.94726912 0.23572094 2.2410923  0.74730227 2.39933726
 0.080094   1.60774613], Cost: 0.2963
Current parameters: [ 1.67156812  2.36908022  1.92148053  3.12964036  2.47206845  1.46921425
  0.88750175  0.95166     0.28364566  2.21098442  0.78827728  2.38165241
 -0.02003181  1.5960412 ], Cost: 0.0300
Current parameters: [1.63517704e+00 2.30199257e+00 1.95726309e+00 3.19270360e+00
 2.45679208e+00 1.46818254e+00 9.64751622e-01 9.58718277e-01
 2.66035233e-01 2.18615494e+00 8.16022363e-01 2.38714058e+00
 3.05626981e-03 1.59033573e+00], Cost: 0.1196
Current parameters: [1.70125891 2.33642628 1.91724151 3.12686426 2.47802751 1.50438397
 0.89935247 0.97034047 0.2854336  2.19996891 0.812775   2.40881289
 0.01478672 1.55842181], Cost: 0.0371
Current parameters: [1.69816936 2.33831392 1.92520255 3.12880187 2.48192266 1.4777761
 0.90711724 0.96639348 0.29409378 2.20582425 0.80844849 2.38032158
 0.01985732 1.61354061], Cost: 0.0306
Current parameters: [1.6772168  2.35913858 1.89983914 3.1520234  2.47884037 1.52557571
 0.90525385 0.96804195 0.29065807 2.20807705 0.80913808 2.35762193
 0.02096325 1.609323  ], Cost: 0.0624
Current parameters: [1.68992355 2.34631107 1.8979166  3.14805611 2.48201441 1.47120651
 0.89934241 0.97818052 0.28447472 2.19743363 0.81645591 2.39130181
 0.01165778 1.60657404], Cost: 0.0410
Current parameters: [1.67155416 2.35947892 1.91359866 3.11836655 2.48824372 1.48074852
 0.92269519 1.00569501 0.31854867 2.18865777 0.78860505 2.39689145
 0.01197897 1.60394134], Cost: 0.0531
Current parameters: [1.69465375 2.3453137  1.90726214 3.15367547 2.47495075 1.47569338
 0.90452591 0.96288074 0.3086975  2.20197927 0.79057066 2.38841328
 0.02213079 1.59738818], Cost: 0.0501
Current parameters: [1.67610011 2.35027697 1.90143139 3.12082226 2.49086192 1.46834611
```



```
 0.90355944 0.96549207 0.26230145 2.22780319 0.78412522 2.39922116
 0.05184682 1.60272572], Cost: 0.0844
Current parameters: [1.67134069 2.34144239 1.92497324 3.15180288 2.47635072 1.47463612
 0.92328806 0.96688887 0.28682541 2.20410862 0.81041739 2.39057376
 0.01810672 1.59748902], Cost: 0.0394
Current parameters: [1.68451384 2.35567711 1.91217072 3.13760557 2.46840547 1.47680533
 0.90708074 0.96816589 0.29481816 2.20420053 0.8108046  2.39105475
 0.02607086 1.60187464], Cost: 0.0341
Current parameters: [1.68731806 2.35392935 1.91568195 3.1408371  2.48161351 1.47623997
 0.90773164 0.96743172 0.30805313 2.22835727 0.82383794 2.39855861
 0.01206371 1.60297428], Cost: 0.0361
Current parameters: [1.67917496 2.35016223 1.91827716 3.14354221 2.48338784 1.47765965
 0.89696067 0.97085582 0.29807204 2.20715554 0.80977155 2.39092511
 0.02558706 1.60071673], Cost: 0.0243
Current parameters: [1.69232921 2.36640587 1.93528607 3.15390572 2.48510315 1.47748084
 0.89517785 0.97970908 0.29101152 2.20643712 0.80891439 2.39307678
 0.02707584 1.60141393], Cost: 0.0198
Current parameters: [1.69216484 2.36284374 1.93390381 3.15344863 2.48105484 1.47583623
 0.89410768 0.98748601 0.28675589 2.21600753 0.80383272 2.39062784
 0.02913948 1.59973961], Cost: 0.0224
Current parameters: [1.68977803 2.37220989 1.93580079 3.1459162  2.5010144  1.4741507
 0.89259951 0.96960492 0.27861117 2.21121987 0.80382058 2.40414752
 0.03963419 1.60546453], Cost: 0.0325
Current parameters: [1.69047134 2.36668465 1.93378703 3.15688773 2.48368383 1.48994124
 0.89503236 0.97933542 0.28899827 2.20815325 0.80640934 2.395097
 0.02552928 1.60864498], Cost: 0.0194
Current parameters: [1.6873194  2.37559751 1.93285689 3.15750271 2.49136703 1.50005543
 0.89386656 0.97918779 0.28867802 2.20979891 0.81512647 2.37092148
 0.02909409 1.60176942], Cost: 0.0283
Current parameters: [1.68569905 2.37100631 1.93272022 3.15829376 2.48509311 1.4838752
 0.89822859 0.98224514 0.29317618 2.20827786 0.80377508 2.39133443
 0.02615436 1.61880133], Cost: 0.0170
Current parameters: [1.68132136 2.37432733 1.93632369 3.14428201 2.49233584 1.49099414
 0.90338504 1.0005701  0.3013012  2.19994806 0.7979494  2.39800458
 0.02041352 1.61382768], Cost: 0.0120
Current parameters: [1.68517193 2.37005889 1.93397779 3.14767131 2.49446124 1.4929451
 0.90837905 1.00457546 0.30467995 2.19907388 0.80234085 2.40007481
 0.03081292 1.61477562], Cost: 0.0128
Current parameters: [1.68887889 2.3739948  1.9366481  3.15460217 2.49446601 1.49190796
 0.89982156 0.9968721  0.31900984 2.19619012 0.77716345 2.39726439
 0.01878915 1.60958285], Cost: 0.0367
```

```
Current parameters: [1.68583463 2.374612   1.92640054 3.14786029 2.4980055  1.48919062
 0.90387124 1.00375507 0.29612053 2.19712747 0.79761147 2.39794145
 0.01478136 1.61376153], Cost: 0.0160
Current parameters: [1.67453433 2.36890039 1.94352888 3.14349246 2.48697248 1.48816788
 0.88753083 1.01208325 0.30005224 2.19136599 0.81280899 2.40171353
 0.01792031 1.62440384], Cost: 0.0120
Current parameters: [1.674364   2.37013583 1.94244873 3.14963726 2.49327926 1.49007636
 0.91195339 1.00099538 0.29680494 2.19803973 0.7986413  2.39846625
 0.01738343 1.61212152], Cost: 0.0105
Current parameters: [1.6707417  2.37366548 1.94006494 3.15239337 2.49196942 1.48990495
 0.91102793 1.00163739 0.29777479 2.19883542 0.79959095 2.40127474
 0.01828375 1.60929492], Cost: 0.0121
Current parameters: [1.67667765 2.37053267 1.94477733 3.14859783 2.49597069 1.48979603
 0.91521138 1.0009806  0.30152984 2.20733727 0.80551939 2.40166744
 0.01074907 1.61408387], Cost: 0.0080
Current parameters: [1.67859628 2.37127376 1.94370427 3.14878755 2.4900971  1.49018121
 0.91792124 1.00202534 0.30276516 2.20564013 0.80668928 2.39999282
 0.00879005 1.61374031], Cost: 0.0108
Current parameters: [1.67525436 2.37471475 1.94782704 3.1400522  2.49810113 1.48838396
 0.91959835 0.99868882 0.29502105 2.20834931 0.80720228 2.40709827
 0.01418608 1.61895609], Cost: 0.0047
Current parameters: [1.66769962 2.36672673 1.94089852 3.13341958 2.49842869 1.48842054
 0.92094006 0.99453654 0.29797983 2.20887527 0.80855059 2.4068487
 0.01340273 1.619722  ], Cost: 0.0090
Current parameters: [1.67494796 2.37730273 1.94830528 3.14031398 2.50075101 1.49027542
 0.91991258 0.99536836 0.29679456 2.2044788  0.81041826 2.40521688
 0.01374307 1.61882165], Cost: 0.0031
Current parameters: [1.6721515  2.38068086 1.9515439  3.13782119 2.50483181 1.49368218
 0.91977158 0.99998124 0.29309786 2.20993921 0.81377295 2.39544007
 0.01625925 1.61740893], Cost: 0.0025
Current parameters: [1.67129936 2.38080383 1.95079002 3.13942263 2.50412311 1.499906
 0.91965798 0.99972386 0.29221097 2.2107845  0.81237477 2.39643387
 0.01548974 1.62098775], Cost: 0.0033
Current parameters: [1.67331097 2.38363691 1.96291421 3.13745936 2.51293058 1.49176062
 0.91957326 0.99797833 0.29680096 2.20914545 0.81134948 2.3974799
 0.01685589 1.61997342], Cost: 0.0027
Current parameters: [1.67126812 2.38259201 1.94854469 3.13944218 2.5068183  1.49055287
 0.92123381 1.00190042 0.29352677 2.20934399 0.81259593 2.39374863
 0.01514848 1.62181358], Cost: 0.0027
Current parameters: [1.67771642 2.3803875  1.94896885 3.13783096 2.51313402 1.49443322
 0.91919917 1.00144264 0.28484611 2.20745539 0.81736142 2.39872846
```



```
 0.01116764 1.61414927], Cost: 0.0024
Current parameters: [1.67937499 2.38178859 1.94718892 3.13802066 2.51442959 1.49509735
 0.92308534 0.99847042 0.2850958  2.20963132 0.81360815 2.39775931
 0.0116749  1.61150718], Cost: 0.0010
Current parameters: [1.68163117 2.38116185 1.94799988 3.1439409  2.51344389 1.49113737
 0.92127079 0.98883051 0.27974593 2.21410924 0.81763284 2.39511557
 0.01455023 1.6146614 ], Cost: 0.0047
Current parameters: [1.67952666 2.37892437 1.94752376 3.14075405 2.51559675 1.49576472
 0.92726635 1.00036361 0.28559321 2.20880693 0.81573493 2.39879261
 0.01565395 1.61154602], Cost: 0.0012
Current parameters: [1.68768323 2.38473721 1.94200685 3.13349955 2.5165352  1.49681864
 0.91843567 0.99961903 0.29017515 2.21128794 0.8149845  2.39873283
 0.01899122 1.6134757 ], Cost: 0.0012
Current parameters: [1.67799004 2.38420044 1.94660047 3.1393633  2.51467198 1.49487563
 0.92380007 0.99887428 0.287608   2.21386454 0.81697021 2.40083453
 0.00959128 1.61048935], Cost: 0.0058
Current parameters: [1.67459799 2.38757069 1.94563885 3.13876991 2.51533143 1.49531115
 0.92085247 0.99856728 0.28061172 2.20237542 0.80813132 2.39863578
 0.01904432 1.60689819], Cost: 0.0033
Current parameters: [1.683233   2.38563631 1.94933751 3.13963911 2.51068419 1.49518451
 0.92484903 1.00042156 0.28412653 2.20843477 0.81406663 2.39733693
 0.0110451  1.61152843], Cost: 0.0009
Current parameters: [1.68659293 2.38132667 1.94988414 3.13024757 2.51018796 1.49646878
 0.93102626 0.99830184 0.28396789 2.20257633 0.81230605 2.39114547
 0.00939931 1.61318451], Cost: 0.0008
Current parameters: [1.68687998 2.38167575 1.94914307 3.13345599 2.51130665 1.4983049
 0.92969619 0.99676639 0.28752388 2.19948331 0.8139831  2.38789463
 0.00787026 1.61136572], Cost: 0.0011
Current parameters: [1.69166074 2.37434573 1.95133535 3.13352272 2.51038363 1.49527086
 0.92753645 1.00432098 0.28389484 2.20516824 0.8034014  2.38746776
 0.00865791 1.61055424], Cost: 0.0046

   Return from subroutine COBYLA because the MAXFUN limit has been reached.

   NFVALS =  100   F = 7.812662E-04    MAXCV = 0.000000E+00
   X = 1.686593E+00   2.381327E+00   1.949884E+00   3.130248E+00   2.510188E+00
       1.496469E+00   9.310263E-01   9.983018E-01   2.839679E-01   2.202576E+00
       8.123060E-01   2.391145E+00   9.399312E-03   1.613185E+00
Current parameters: [1.68659293 2.38132667 1.94988414 3.13024757 2.51018796 1.49646878
 0.93102626 0.99830184 0.28396789 2.20257633 0.81230605 2.39114547
 0.00939931 1.61318451], Cost: 0.0008
```



```
Simulating for QAOA depth p = 8
Current parameters: [3.56462543 2.18104212 1.47655887 0.06256055 0.90453452 2.72623028
 0.44971515 1.66655662 0.00562242 0.19989412 1.99547192 1.66391046
 0.97172214 1.39214309 0.75928044 1.31136204], Cost: 1.8896
Current parameters: [2.56462543 3.18104212 1.47655887 0.06256055 0.90453452 2.72623028
 0.44971515 1.66655662 0.00562242 0.19989412 1.99547192 1.66391046
 0.97172214 1.39214309 0.75928044 1.31136204], Cost: 1.8447
Current parameters: [2.56462543 2.18104212 2.47655887 0.06256055 0.90453452 2.72623028
 0.44971515 1.66655662 0.00562242 0.19989412 1.99547192 1.66391046
 0.97172214 1.39214309 0.75928044 1.31136204], Cost: 1.9832
Current parameters: [2.56462543 2.18104212 1.47655887 1.06256055 0.90453452 2.72623028
 0.44971515 1.66655662 0.00562242 0.19989412 1.99547192 1.66391046
 0.97172214 1.39214309 0.75928044 1.31136204], Cost: 1.5204
Current parameters: [2.56462543 2.18104212 1.47655887 1.06256055 1.90453452 2.72623028
 0.44971515 1.66655662 0.00562242 0.19989412 1.99547192 1.66391046
 0.97172214 1.39214309 0.75928044 1.31136204], Cost: 1.8493
Current parameters: [2.56462543 2.18104212 1.47655887 1.06256055 0.90453452 3.72623028
 0.44971515 1.66655662 0.00562242 0.19989412 1.99547192 1.66391046
 0.97172214 1.39214309 0.75928044 1.31136204], Cost: 1.4010
Current parameters: [2.56462543 2.18104212 1.47655887 1.06256055 0.90453452 3.72623028
 1.44971515 1.66655662 0.00562242 0.19989412 1.99547192 1.66391046
 0.97172214 1.39214309 0.75928044 1.31136204], Cost: 2.7830
Current parameters: [2.56462543 2.18104212 1.47655887 1.06256055 0.90453452 3.72623028
 0.44971515 2.66655662 0.00562242 0.19989412 1.99547192 1.66391046
 0.97172214 1.39214309 0.75928044 1.31136204], Cost: 2.7540
Current parameters: [2.56462543 2.18104212 1.47655887 1.06256055 0.90453452 3.72623028
 0.44971515 1.66655662 1.00562242 0.19989412 1.99547192 1.66391046
 0.97172214 1.39214309 0.75928044 1.31136204], Cost: 2.1206
Current parameters: [2.56462543 2.18104212 1.47655887 1.06256055 0.90453452 3.72623028
 0.44971515 1.66655662 0.00562242 1.19989412 1.99547192 1.66391046
 0.97172214 1.39214309 0.75928044 1.31136204], Cost: 1.8474
Current parameters: [2.56462543 2.18104212 1.47655887 1.06256055 0.90453452 3.72623028
 0.44971515 1.66655662 0.00562242 0.19989412 2.99547192 1.66391046
 0.97172214 1.39214309 0.75928044 1.31136204], Cost: 0.4341
Current parameters: [2.56462543 2.18104212 1.47655887 1.06256055 0.90453452 3.72623028
 0.44971515 1.66655662 0.00562242 0.19989412 2.99547192 2.66391046
 0.97172214 1.39214309 0.75928044 1.31136204], Cost: 3.3398
Current parameters: [2.56462543 2.18104212 1.47655887 1.06256055 0.90453452 3.72623028
 0.44971515 1.66655662 0.00562242 0.19989412 2.99547192 1.66391046
 1.97172214 1.39214309 0.75928044 1.31136204], Cost: 2.3528
Current parameters: [2.56462543 2.18104212 1.47655887 1.06256055 0.90453452 3.72623028
```



```
 0.44971515 1.66655662 0.00562242 0.19989412 2.99547192 1.66391046
 0.97172214 2.39214309 0.75928044 1.31136204], Cost: 3.1023
Current parameters: [2.56462543 2.18104212 1.47655887 1.06256055 0.90453452 3.72623028
 0.44971515 1.66655662 0.00562242 0.19989412 2.99547192 1.66391046
 0.97172214 1.39214309 1.75928044 1.31136204], Cost: 2.5922
Current parameters: [2.56462543 2.18104212 1.47655887 1.06256055 0.90453452 3.72623028
 0.44971515 1.66655662 0.00562242 0.19989412 2.99547192 1.66391046
 0.97172214 1.39214309 0.75928044 2.31136204], Cost: 1.1393
Current parameters: [ 2.53186166  2.15646998  1.4267399   1.09712391  0.84455608  3.74800166
  0.19772     1.41985204 -0.12558139  0.11849543  3.1717974   1.13405946
  0.62185058  0.90560147  0.3657512   1.18275794], Cost: 0.5999
Current parameters: [ 2.56048497  2.17793686  1.47026309  1.06692843  0.89695486  3.7289816
  0.93452849  1.63537981 -0.01095821  0.18960752  3.01775471  1.59695156
  0.9275078   1.33065734  0.70954895  1.29510995], Cost: 2.0857
Current parameters: [2.31680136 2.18104212 1.47655887 1.06256055 0.90453452 3.72623028
 0.44971515 1.69946907 0.00562242 0.19989412 2.99547192 1.66391046
 0.97172214 1.39214309 0.75928044 1.31136204], Cost: 0.1528
Current parameters: [ 2.3571318   2.17822668  1.47085069  1.06652077  0.89766228  3.72872481
  0.38377146  2.18169665 -0.00941071  0.19056758  3.01567503  1.60320094
  0.93163439  1.33639589  0.71419046  1.29662678], Cost: 0.7016
Current parameters: [2.31680136 1.93531438 1.47655887 1.06256055 0.90453452 3.72623028
 0.44971515 1.69946907 0.05164284 0.19989412 2.99547192 1.66391046
 0.97172214 1.39214309 0.75928044 1.31136204], Cost: 0.0593
Current parameters: [2.30257497 2.02242177 1.47354215 1.06465349 0.90090262 3.72754861
 0.41486462 1.68670767 0.53917854 0.19496515 3.00614905 1.63182613
 0.95053619 1.3626813  0.73545088 1.30357462], Cost: 2.3672
Current parameters: [2.31680136 1.93531438 1.61906559 1.26796706 0.90453452 3.72623028
 0.44971515 1.69946907 0.05164284 0.19989412 2.99547192 1.66391046
 0.97172214 1.39214309 0.75928044 1.31136204], Cost: 0.2385
Current parameters: [2.31089521 1.92964929 1.88443596 0.77558617 0.90302672 3.72677759
 0.43524679 1.69417111 0.03070273 0.19784783 2.99990458 1.6505905
 0.96292669 1.37991189 0.74938749 1.30812906], Cost: 0.1361
Current parameters: [2.31680136 1.93531438 1.47655887 1.06256055 0.98983543 3.96122763
 0.44971515 1.69946907 0.05164284 0.19989412 2.99547192 1.66391046
 0.97172214 1.39214309 0.75928044 1.31136204], Cost: 0.2625
Current parameters: [2.31058906 1.92935564 1.47306109 1.06078218 1.37183103 3.5524399
 0.43449682 1.69389649 0.0296173  0.19774177 3.00013435 1.64990006
 0.96247078 1.37927789 0.74887469 1.30796148], Cost: 0.6460
Current parameters: [2.31755917 1.93588273 1.47771116 1.06176112 0.9059218  3.72572672
 0.45554369 1.70517524 0.05467753 0.4497325  2.99547192 1.66391046
 0.97172214 1.39214309 0.75928044 1.31136204], Cost: 0.8342
```

```
Current parameters: [ 2.24330513  1.86737017  1.42349607  1.0578834   0.80534528  3.72110955
  0.20572444  1.55262251 -0.21453685  0.06575968  3.04147582  1.52567058
  0.88043949  1.26520278  0.65660737  1.27780881], Cost: 2.1930
Current parameters: [2.31680136 1.93531438 1.47655887 1.06256055 0.90453452 3.72623028
 0.44971515 1.69946907 0.05164284 0.19989412 3.23268187 1.74284993
 0.97172214 1.39214309 0.75928044 1.31136204], Cost: 0.4707
Current parameters: [ 2.30133211e+00  1.91905703e+00  1.47434584e+00  1.04903214e+00
  8.89052167e-01  3.71691925e+00  4.47384377e-01  1.73059668e+00
 -8.82360114e-05  1.46410197e-01  2.81481982e+00  2.11676321e+00
  9.38580986e-01  1.34605604e+00  7.22003872e-01  1.29918018e+00], Cost: 1.8921
Current parameters: [2.31680136 1.93531438 1.47655887 1.06256055 0.90453452 3.72623028
 0.44971515 1.69946907 0.05164284 0.19989412 2.99547192 1.66391046
 1.17469241 1.24618739 0.75928044 1.31136204], Cost: 0.0290
Current parameters: [ 2.29931611  1.91693833  1.47405743  1.04726908  0.88703446  3.71570581
  0.44708062  1.73465333 -0.00682999  0.13944001  2.97756501  1.61599145
  1.46010917  1.63904398  0.71714588  1.2975926 ], Cost: 2.5459
Current parameters: [2.31680136 1.93531438 1.47655887 1.06256055 0.90453452 3.72623028
 0.44971515 1.69946907 0.05164284 0.19989412 2.99547192 1.66391046
 1.17469241 1.24618739 0.83693805 1.07372935], Cost: 0.5350
Current parameters: [2.30521881 1.92314174 1.47490187 1.0524312  0.89294216 3.71925868
 0.44796999 1.72277577 0.01290936 0.1598482  2.98361005 1.63216802
 1.1533208  1.21378308 1.2191731  1.48919007], Cost: 1.1625
Current parameters: [2.31843542 1.87612907 1.42266676 1.03160783 0.85998232 3.68266582
 0.64408683 1.71177318 0.04065651 0.13946295 2.95770804 1.5995585
 1.11531068 1.25978844 0.7437659  1.30629194], Cost: 0.3596
Current parameters: [ 2.27358966  2.10270729  1.64717499  1.15118047  1.01474539  3.86372221
  0.6507936   1.66099998 -0.04093895  0.28623741  3.13557026  1.68277869
  1.24447084  1.00374822  0.66507929  1.3520608 ], Cost: 2.4675
Current parameters: [ 2.29455078  1.89221066  1.43574033  1.0542485   0.8543754   3.71120406
  0.31547176  1.63357982 -0.03716     0.14770276  2.98477889  1.56573954
  1.10300099  1.18097448  0.69996889  1.31730634], Cost: 0.9778
Current parameters: [2.33211646 1.93085582 1.47781627 1.06483901 0.90511106 3.72877508
 0.43639127 1.81478872 0.0137729  0.20058869 3.00167192 1.6534784
 1.16858255 1.23325661 0.75004412 1.30834364], Cost: 0.0317
Current parameters: [2.31385214 1.97086359 1.47407509 1.05214925 0.89513908 3.71801297
 0.41446593 1.76271573 0.27700199 0.16265471 2.98674461 1.62915007
 1.15057769 1.21538147 0.73608394 1.32507249], Cost: 0.4771
Current parameters: [2.31669911 1.9226798  1.5690581  0.98426666 0.89496086 3.71736349
 0.44607573 1.69869914 0.04720235 0.18782899 2.98098053 1.67071552
 1.16227317 1.24878801 0.75590564 1.31025917], Cost: 0.0364
Current parameters: [2.30908564 1.91826709 1.43499537 1.06933305 0.88660826 3.71256596
```



```
 0.4412031  1.71552352 0.0429397  0.16134805 2.8965147  1.87834122
 1.14369123 1.2216757  0.73647252 1.3223837 ], Cost: 0.7877
Current parameters: [2.31719927 1.94520941 1.48257893 1.06989988 0.79592215 3.77572096
 0.45350808 1.70246526 0.0530778  0.20986252 3.00382067 1.6687881
 1.18381802 1.24267114 0.78742838 1.3205607 ], Cost: 0.1123
Current parameters: [2.30991127 1.92882595 1.47548317 1.0551384  0.95712028 3.69924164
 0.43948935 1.71125388 0.03958549 0.17519921 2.98760138 1.64439422
 1.16031853 1.22700423 0.98076941 1.39960643], Cost: 0.4488
Current parameters: [2.30608822 1.97870769 1.51966897 1.08425123 0.93830071 3.75819828
 0.50004973 1.69222822 0.03703702 0.22067764 3.03165868 1.66416521
 1.19202602 1.18534584 0.7376694  1.32259413], Cost: 0.1230
Current parameters: [2.25627513 1.93448967 1.47639253 1.06200954 0.90404168 3.72582667
 0.44967084 1.70837807 0.05021374 0.19934753 2.99527651 1.66276361
 1.181557   1.25667134 0.75819413 1.31100704], Cost: 0.0520
Current parameters: [2.34494927 1.94850967 1.49068599 1.06633984 0.91732171 3.73236457
 0.45360355 1.70336121 0.04153154 0.18521949 3.00144548 1.64977662
 1.25663298 1.32564659 0.74045159 1.32802729], Cost: 0.2521
Current parameters: [2.31682836 1.98785608 1.46771571 1.04972653 0.89926609 3.71156203
 0.44992283 1.69967238 0.04191464 0.20879621 2.98068368 1.65898918
 1.16203875 1.25528662 0.75928044 1.31136204], Cost: 0.0522
Current parameters: [2.32043994 1.90170424 1.47439143 1.05074628 0.90752934 3.71292139
 0.45480459 1.70220204 0.06207297 0.31612179 2.98127787 1.65518094
 1.17374692 1.25545445 0.75385678 1.32169907], Cost: 0.2776
Current parameters: [2.31680136 1.93531438 1.4457451  1.01814616 0.91112492 3.7443863
 0.44971515 1.69946907 0.05164284 0.19989412 3.01010914 1.66878147
 1.190374   1.23491077 0.75928044 1.31136204], Cost: 0.0432
Current parameters: [2.31838992 1.93806601 1.47740117 1.06012294 0.89153268 3.6809626
 0.45089369 1.69953818 0.05243418 0.20126528 3.09720803 1.69583499
 1.14058555 1.27485399 0.75666536 1.31634615], Cost: 0.1069
Current parameters: [2.31498124 1.9351197  1.47645431 1.0625938  0.91952471 3.7668621
 0.44979395 1.69918696 0.05274857 0.20238115 2.99616022 1.66485171
 1.13402987 1.265276   0.75999561 1.31159576], Cost: 0.0055
Current parameters: [2.34217095 1.94769919 1.49250826 1.06990759 0.93915024 3.7932934
 0.45328931 1.70273774 0.04281028 0.17849652 3.00245313 1.65139965
 1.19915451 1.35241568 0.74185415 1.32753928], Cost: 0.1436
Current parameters: [2.33061351 1.92997159 1.47981642 1.08068857 0.92147947 3.75448082
 0.43997043 1.70110257 0.10188705 0.18677652 2.98962396 1.65439175
 1.14196558 1.25498095 0.75246294 1.3134486 ], Cost: 0.0237
Current parameters: [2.27410578 1.98592805 1.47551711 1.00854518 0.91861894 3.80340574
 0.45463461 1.74611334 0.10555148 0.21333041 3.01924228 1.6537178
 1.100245   1.27367179 0.7531155  1.31964437], Cost: 0.1579
```



```
Current parameters: [2.31112624 1.93038754 1.47300965 1.06225484 0.9147502  3.7701355
 0.41758713 1.68298585 0.03215991 0.18916922 3.0020653  1.63311301
 1.12047229 1.25204168 0.73818536 1.31855588], Cost: 0.0762
Current parameters: [2.31456569 1.93413148 1.47615566 1.06210449 0.92376196 3.76423269
 0.4494844  1.70022307 0.0516694  0.19971761 2.99500496 1.66335376
 1.13261932 1.26342693 0.77387734 1.33878212], Cost: 0.0129
Current parameters: [2.3167209  1.93628124 1.48154932 1.06565042 0.93420089 3.76663247
 0.44217237 1.69756422 0.0494633  0.19454195 3.00112483 1.65174924
 1.13457311 1.26103753 0.80877028 1.28118834], Cost: 0.0542
Current parameters: [2.31370919 1.94165847 1.48055362 1.06550728 0.92359396 3.77173208
 0.42742818 1.69900893 0.05333521 0.20794377 2.99615658 1.6822916
 1.13884604 1.26284758 0.76066108 1.31277434], Cost: 0.0143
Current parameters: [2.31657366 1.92545772 1.46443767 1.06557382 0.92096907 3.76379312
 0.46793274 1.70063947 0.04440669 0.17251135 2.9714202  1.70195819
 1.11818591 1.25771081 0.75447361 1.30660145], Cost: 0.0411
Current parameters: [2.32138957 1.93495473 1.48129626 1.06980048 0.92309943 3.76777264
 0.45081444 1.7239993  0.04352067 0.20190056 2.99925672 1.66200461
 1.13660363 1.25507858 0.75598444 1.31151696], Cost: 0.0059
Current parameters: [2.3107616  1.95310969 1.49638314 1.07669144 0.93519915 3.78066664
 0.47666119 1.67331413 0.05203456 0.2051048  3.00902808 1.66848711
 1.14332188 1.23715749 0.75211086 1.3155341 ], Cost: 0.0252
Current parameters: [2.31922799 1.92930958 1.50016949 1.048573   0.91703542 3.75786015
 0.44890093 1.69833109 0.05229871 0.19712141 3.00053447 1.66885472
 1.13062576 1.26606928 0.7586755  1.31142208], Cost: 0.0052
Current parameters: [2.32386802 1.92962614 1.48615532 1.06526577 0.91413346 3.73568861
 0.45123616 1.69737706 0.05265787 0.19420667 3.04925842 1.68410611
 1.11729689 1.276479   0.757725   1.3124399 ], Cost: 0.0163
Current parameters: [2.32164972 1.93190199 1.50332299 1.05344179 0.89080236 3.76872008
 0.4494112  1.69846642 0.05230342 0.1906219  3.00353481 1.67065046
 1.13405062 1.26345564 0.7656693  1.31283393], Cost: 0.0068
Current parameters: [2.33283531 1.90099708 1.50356355 1.0561316  0.9034515  3.75226729
 0.45942184 1.69614229 0.0536002  0.24594326 2.9913158  1.67421848
 1.1270354  1.26380179 0.75992308 1.31239645], Cost: 0.0349
Current parameters: [2.30953031 1.9362346  1.48950882 1.0392014  0.91226489 3.76731801
 0.4571298  1.70115246 0.06308378 0.2047813  3.0026966  1.66348409
 1.12076962 1.27523042 0.75464193 1.305149  ], Cost: 0.0201
Current parameters: [2.31716149 1.93976023 1.52822968 1.03307317 0.92663508 3.75863519
 0.44091137 1.72925905 0.06466197 0.19359216 3.00751009 1.68597078
 1.12043792 1.24152273 0.76446152 1.3315539 ], Cost: 0.0129
Current parameters: [2.32819456 1.93060008 1.50861717 1.04930827 0.92317333 3.76466109
 0.45229286 1.70372467 0.05001888 0.19257582 3.00148078 1.67117445
```



```
 1.14510589 1.28604066 0.75443482 1.31770071], Cost: 0.0093
Current parameters: [2.33173775 1.93302166 1.49730034 1.0446212  0.91805221 3.7610379
 0.44723725 1.69569031 0.0510126  0.19731072 3.00089685 1.66759429
 1.12599197 1.2642304  0.75788828 1.31193348], Cost: 0.0063
Current parameters: [2.31308655 1.92191458 1.5049074  1.04993945 0.91531224 3.76011694
 0.43485801 1.69543436 0.04104758 0.19007978 3.00305556 1.65576643
 1.12634055 1.26355463 0.74378382 1.31819755], Cost: 0.0238
Current parameters: [2.31926664 1.93710723 1.50521457 1.05539674 0.91583628 3.75398283
 0.448775   1.698524   0.05091123 0.19842242 2.99554521 1.66740763
 1.12381211 1.27027437 0.75886096 1.31163976], Cost: 0.0079
Current parameters: [2.31988284 1.94253112 1.49473024 1.03839781 0.90993209 3.7403751
 0.44977234 1.69963292 0.04757817 0.19884777 2.99792468 1.66769824
 1.14621922 1.26152019 0.75531088 1.31241352], Cost: 0.0120
Current parameters: [2.31567527 1.93334691 1.50306281 1.04763756 0.91938733 3.76245453
 0.454967   1.69368203 0.04322224 0.20056197 3.00402485 1.67129172
 1.13083841 1.26319488 0.75889753 1.31189473], Cost: 0.0053
Current parameters: [2.32190243 1.93391966 1.50539828 1.05915166 0.92156597 3.75935359
 0.45765129 1.68746405 0.06940375 0.19454369 3.00305679 1.66378208
 1.13805867 1.25503226 0.74957711 1.31199265], Cost: 0.0097
Current parameters: [2.31906728 1.92747092 1.4966082  1.04886814 0.91524366 3.75751291
 0.45334848 1.69830546 0.05139275 0.19271601 2.99505153 1.67762292
 1.12760139 1.26544017 0.75096562 1.31437074], Cost: 0.0075
Current parameters: [2.32145885 1.92516769 1.5001574  1.04967676 0.92548585 3.75641254
 0.45377834 1.69791419 0.04885786 0.18220648 2.99532507 1.67223053
 1.13227008 1.26221082 0.77636434 1.29553407], Cost: 0.0150
Current parameters: [2.32361844 1.92129128 1.50286984 1.05035636 0.91499424 3.75597772
 0.45121785 1.69875033 0.05210504 0.20810233 2.99839327 1.67093765
 1.13175948 1.26464035 0.76065208 1.31168672], Cost: 0.0074
Current parameters: [2.31831966 1.93033144 1.50323607 1.0456438  0.91789467 3.76037933
 0.44812638 1.70072411 0.05352477 0.19736709 2.99641829 1.66866736
 1.13092177 1.26345429 0.75922261 1.3128377 ], Cost: 0.0055
Current parameters: [2.31973433 1.93089407 1.50350597 1.04800249 0.91875564 3.75465764
 0.44797904 1.70461506 0.0551459  0.19643265 3.01003242 1.67505564
 1.12719548 1.26315781 0.7606623  1.31528583], Cost: 0.0022
Current parameters: [2.31978008 1.93203093 1.50467013 1.0484525  0.91861155 3.75580861
 0.44447356 1.70476884 0.05529219 0.19773792 3.01037134 1.67814159
 1.12822006 1.26297484 0.75802933 1.3102712 ], Cost: 0.0024
Current parameters: [2.31748769 1.93319648 1.4985772  1.05074918 0.92135699 3.75666431
 0.44118747 1.69952814 0.05735717 0.19882918 3.00906947 1.68068606
 1.1294077  1.26391941 0.76622635 1.32156788], Cost: 0.0045
Current parameters: [2.31930151 1.92986048 1.50684963 1.04424715 0.91765998 3.7529291
```

```
 0.4475614  1.70006004 0.05664211 0.19555694 3.01027614 1.67617696
 1.12608828 1.26507902 0.76104521 1.31529865], Cost: 0.0032
Current parameters: [2.31766887 1.92547036 1.50607942 1.04963203 0.91934238 3.75471537
 0.44031551 1.7059787  0.04948066 0.193637   3.01476643 1.67015234
 1.12555882 1.25938668 0.7559806  1.31916668], Cost: 0.0080
Current parameters: [2.3178473  1.93209135 1.50206957 1.04632473 0.91690935 3.75641997
 0.44804667 1.70522211 0.0572356  0.20020329 3.01159216 1.67357486
 1.12586813 1.26523947 0.75727383 1.31715349], Cost: 0.0017
Current parameters: [2.31476156 1.93244328 1.49770859 1.04314912 0.91924173 3.75948817
 0.45329701 1.71043457 0.06144    0.19965842 3.01554684 1.67400991
 1.12214529 1.26698681 0.7631898  1.31007615], Cost: 0.0074
Current parameters: [2.31740107 1.93294054 1.50026552 1.0427749  0.91500761 3.75374946
 0.44635348 1.70764853 0.05306287 0.20096495 3.01070934 1.6744642
 1.12643393 1.26692605 0.75869832 1.31717458], Cost: 0.0029
Current parameters: [2.31880914 1.93871242 1.50138862 1.04568326 0.91595101 3.74963703
 0.45125277 1.70407738 0.06150465 0.20100948 3.01365961 1.67175319
 1.13401927 1.2592673  0.75396362 1.3178263 ], Cost: 0.0012
Current parameters: [2.31906529 1.93823023 1.50142826 1.04462395 0.92257805 3.74774409
 0.45144355 1.70453713 0.061296   0.20209911 3.01305173 1.671549
 1.13454535 1.26169015 0.75195973 1.31807134], Cost: 0.0007
Current parameters: [2.32367836 1.93942755 1.50595511 1.04604234 0.92107807 3.75261986
 0.45359894 1.70780139 0.06133386 0.19920165 3.01562082 1.67294829
 1.14259418 1.2697119  0.75133593 1.3214063 ], Cost: 0.0017
Current parameters: [2.32025189 1.94102638 1.50298103 1.04857681 0.92304131 3.74531229
 0.45361433 1.70690809 0.0600924  0.20144951 3.01126861 1.67071085
 1.13199644 1.26191536 0.75405679 1.31885403], Cost: 0.0007
Current parameters: [2.31591827 1.94480889 1.50634594 1.04633449 0.92044176 3.74686857
 0.44629027 1.69997989 0.06455168 0.2071696  3.01249372 1.671258
 1.13099462 1.2671779  0.74649355 1.31669797], Cost: 0.0008
Current parameters: [2.31885887 1.93689454 1.50018132 1.04592835 0.92200893 3.74900539
 0.45392873 1.7046031  0.06220865 0.20089178 3.01138517 1.67493847
 1.13213548 1.26148033 0.74753789 1.32049954], Cost: 0.0014
Current parameters: [2.31863107 1.94319283 1.50007825 1.04003028 0.92144773 3.73874226
 0.45060955 1.70474922 0.0584112  0.1991357  3.01064358 1.67847757
 1.13933302 1.26118469 0.75130689 1.31373109], Cost: 0.0036
Current parameters: [2.32124816 1.93509384 1.50306969 1.04545343 0.92170139 3.74618196
 0.45243952 1.70481059 0.06177246 0.20801472 3.01266074 1.67282223
 1.13542451 1.26095108 0.7526319  1.3179765 ], Cost: 0.0011
Current parameters: [2.32038894 1.93736304 1.50061033 1.04471503 0.92203217 3.74672006
 0.44988331 1.70559376 0.06349639 0.20125442 3.01220087 1.67090115
 1.13432808 1.26233612 0.75187682 1.31797254], Cost: 0.0009
```



```
Current parameters: [2.32498171 1.94079515 1.500422   1.0425792  0.92332989 3.74932877
 0.45103714 1.70290033 0.06024493 0.20278204 3.01386071 1.67126319
 1.13245345 1.26052875 0.75143315 1.31824057], Cost: 0.0006
Current parameters: [2.32457284 1.94152877 1.50186113 1.04101661 0.92370668 3.75055333
 0.45082928 1.70402298 0.06107562 0.20305409 3.01173021 1.67142418
 1.1326547  1.2593972  0.75182782 1.31877898], Cost: 0.0006
Current parameters: [2.32418962 1.94059411 1.50450384 1.04180962 0.92503058 3.75010121
 0.44798406 1.70528336 0.05897829 0.20250721 3.01407037 1.66836642
 1.13257495 1.25615257 0.74937304 1.32094159], Cost: 0.0022
Current parameters: [2.32493869 1.94114429 1.50139226 1.04164685 0.92322963 3.75072959
 0.45082298 1.70268151 0.05971927 0.20300247 3.00927719 1.66986648
 1.13385642 1.25956206 0.75109379 1.31870388], Cost: 0.0009
Current parameters: [2.32345464 1.94258198 1.50034081 1.04086511 0.92568367 3.75309113
 0.45434466 1.70437416 0.06384806 0.20431615 3.01261546 1.66889319
 1.13173729 1.26005537 0.7528923  1.31495349], Cost: 0.0018

   Return from subroutine COBYLA because the MAXFUN limit has been reached.

   NFVALS =  100   F = 5.638996E-04    MAXCV = 0.000000E+00
   X = 2.324573E+00   1.941529E+00   1.501861E+00   1.041017E+00   9.237067E-01
       3.750553E+00   4.508293E-01   1.704023E+00   6.107562E-02   2.030541E-01
       3.011730E+00   1.671424E+00   1.132655E+00   1.259397E+00   7.518278E-01
       1.318779E+00
Current parameters: [2.32457284 1.94152877 1.50186113 1.04101661 0.92370668 3.75055333
 0.45082928 1.70402298 0.06107562 0.20305409 3.01173021 1.67142418
 1.1326547  1.2593972  0.75182782 1.31877898], Cost: 0.0006
Simulating for QAOA depth p = 9
Current parameters: [3.4122854  0.2352302  1.80263983 3.08349539 2.58976169 0.94869244
 0.0580931  0.49043175 2.41794989 2.76301241 0.91775403 1.03379267
 2.34338745 2.34442536 2.67597208 0.31456195 2.82013816 3.03367071], Cost: 0.2964
Current parameters: [3.4122854  1.2352302  1.80263983 3.08349539 2.58976169 0.94869244
 0.0580931  0.49043175 2.41794989 2.76301241 0.91775403 1.03379267
 2.34338745 2.34442536 2.67597208 0.31456195 2.82013816 3.03367071], Cost: 1.8118
Current parameters: [3.4122854  0.2352302  2.80263983 3.08349539 2.58976169 0.94869244
 0.0580931  0.49043175 2.41794989 2.76301241 0.91775403 1.03379267
 2.34338745 2.34442536 2.67597208 0.31456195 2.82013816 3.03367071], Cost: 2.2491
Current parameters: [3.4122854  0.2352302  1.80263983 4.08349539 2.58976169 0.94869244
 0.0580931  0.49043175 2.41794989 2.76301241 0.91775403 1.03379267
 2.34338745 2.34442536 2.67597208 0.31456195 2.82013816 3.03367071], Cost: 2.6981
Current parameters: [3.4122854  0.2352302  1.80263983 3.08349539 3.58976169 0.94869244
 0.0580931  0.49043175 2.41794989 2.76301241 0.91775403 1.03379267
```



```
 2.34338745 2.34442536 2.67597208 0.31456195 2.82013816 3.03367071], Cost: 2.0975
Current parameters: [3.4122854  0.2352302  1.80263983 3.08349539 2.58976169 1.94869244
 0.0580931  0.49043175 2.41794989 2.76301241 0.91775403 1.03379267
 2.34338745 2.34442536 2.67597208 0.31456195 2.82013816 3.03367071], Cost: 2.0715
Current parameters: [3.4122854  0.2352302  1.80263983 3.08349539 2.58976169 0.94869244
 1.0580931  0.49043175 2.41794989 2.76301241 0.91775403 1.03379267
 2.34338745 2.34442536 2.67597208 0.31456195 2.82013816 3.03367071], Cost: 0.7730
Current parameters: [3.4122854  0.2352302  1.80263983 3.08349539 2.58976169 0.94869244
 0.0580931  1.49043175 2.41794989 2.76301241 0.91775403 1.03379267
 2.34338745 2.34442536 2.67597208 0.31456195 2.82013816 3.03367071], Cost: 1.4991
Current parameters: [3.4122854  0.2352302  1.80263983 3.08349539 2.58976169 0.94869244
 0.0580931  0.49043175 3.41794989 2.76301241 0.91775403 1.03379267
 2.34338745 2.34442536 2.67597208 0.31456195 2.82013816 3.03367071], Cost: 0.7281
Current parameters: [3.4122854  0.2352302  1.80263983 3.08349539 2.58976169 0.94869244
 0.0580931  0.49043175 2.41794989 3.76301241 0.91775403 1.03379267
 2.34338745 2.34442536 2.67597208 0.31456195 2.82013816 3.03367071], Cost: 0.8353
Current parameters: [3.4122854  0.2352302  1.80263983 3.08349539 2.58976169 0.94869244
 0.0580931  0.49043175 2.41794989 2.76301241 1.91775403 1.03379267
 2.34338745 2.34442536 2.67597208 0.31456195 2.82013816 3.03367071], Cost: 0.8831
Current parameters: [3.4122854  0.2352302  1.80263983 3.08349539 2.58976169 0.94869244
 0.0580931  0.49043175 2.41794989 2.76301241 0.91775403 2.03379267
 2.34338745 2.34442536 2.67597208 0.31456195 2.82013816 3.03367071], Cost: 3.1515
Current parameters: [3.4122854  0.2352302  1.80263983 3.08349539 2.58976169 0.94869244
 0.0580931  0.49043175 2.41794989 2.76301241 0.91775403 1.03379267
 3.34338745 2.34442536 2.67597208 0.31456195 2.82013816 3.03367071], Cost: 2.9818
Current parameters: [3.4122854  0.2352302  1.80263983 3.08349539 2.58976169 0.94869244
 0.0580931  0.49043175 2.41794989 2.76301241 0.91775403 1.03379267
 2.34338745 3.34442536 2.67597208 0.31456195 2.82013816 3.03367071], Cost: 3.6259
Current parameters: [3.4122854  0.2352302  1.80263983 3.08349539 2.58976169 0.94869244
 0.0580931  0.49043175 2.41794989 2.76301241 0.91775403 1.03379267
 2.34338745 2.34442536 3.67597208 0.31456195 2.82013816 3.03367071], Cost: 1.9966
Current parameters: [3.4122854  0.2352302  1.80263983 3.08349539 2.58976169 0.94869244
 0.0580931  0.49043175 2.41794989 2.76301241 0.91775403 1.03379267
 2.34338745 2.34442536 2.67597208 1.31456195 2.82013816 3.03367071], Cost: 2.1625
Current parameters: [3.4122854  0.2352302  1.80263983 3.08349539 2.58976169 0.94869244
 0.0580931  0.49043175 2.41794989 2.76301241 0.91775403 1.03379267
 2.34338745 2.34442536 2.67597208 0.31456195 3.82013816 3.03367071], Cost: 2.4945
Current parameters: [3.4122854  0.2352302  1.80263983 3.08349539 2.58976169 0.94869244
 0.0580931  0.49043175 2.41794989 2.76301241 0.91775403 1.03379267
 2.34338745 2.34442536 2.67597208 0.31456195 2.82013816 4.03367071], Cost: 1.5717
Current parameters: [ 3.66509934e+00  4.60204361e-02  1.55883657e+00  2.78362771e+00
```



```
  2.36488752e+00  7.27055760e-01 -1.40899989e-03  3.40271074e-01
  2.36405370e+00  2.69572369e+00  8.44501795e-01  6.77307789e-01
  2.00809706e+00  1.92871161e+00  2.46369228e+00  8.15687177e-02
  2.54568631e+00  2.87444030e+00], Cost: 1.5243
Current parameters: [3.53869237 0.14062532 1.6807382  2.93356155 2.4773246  0.8378741
 0.02834205 0.41535141 2.39100179 2.72936805 0.88112791 0.85555023
 2.17574225 2.13656848 2.56983218 0.19806534 2.68291224 2.9540555 ], Cost: 2.4070
Current parameters: [3.16570732 0.22716872 1.79225234 3.07071922 2.58018069 0.93924937
 0.05555795 0.48403399 2.41565358 2.7601455  0.91463304 1.01860425
 2.32910205 2.32671344 2.66692768 0.30463503 2.80844485 3.02688653], Cost: 0.6093
Current parameters: [3.4122854  0.33398063 1.72600203 3.08349539 2.58976169 0.94869244
 0.0580931  0.49043175 2.41794989 2.76301241 0.91775403 1.03379267
 2.34338745 2.34442536 2.67597208 0.31456195 2.82013816 3.03367071], Cost: 0.3286
Current parameters: [3.41580873 0.38399477 1.99719024 3.06712219 2.57748323 0.93659075
 0.0548442  0.48223276 2.41500708 2.75933835 0.91375435 1.01432808
 2.32508011 2.32172679 2.6643813  0.30184019 2.8051527  3.0249765 ], Cost: 0.8927
Current parameters: [3.4122854  0.2352302  1.80263983 3.15848972 2.48975743 0.94869244
 0.0580931  0.49043175 2.41794989 2.76301241 0.91775403 1.03379267
 2.34338745 2.34442536 2.67597208 0.31456195 2.82013816 3.03367071], Cost: 0.4363
Current parameters: [3.41680754 0.22605298 1.79448822 3.2716076  2.74306885 0.93316014
 0.05392319 0.47990849 2.41417284 2.75829681 0.91262051 1.00881021
 2.3198903  2.31529214 2.66109551 0.29823379 2.80090458 3.02251184], Cost: 0.9922
Current parameters: [ 3.4122854   0.2352302   1.80263983  3.08349539  2.58976169  0.98110312
 -0.06263199  0.49043175  2.41794989  2.76301241  0.91775403  1.03379267
  2.34338745  2.34442536  2.67597208  0.31456195  2.82013816  3.03367071], Cost: 0.3507
Current parameters: [3.41507499 0.229569   1.7976113  3.07358019 2.58987653 1.18685587
 0.12445779 0.48394021 2.41561992 2.76010347 0.91458729 1.01838161
 2.32889264 2.3264538  2.6667951  0.30448952 2.80827344 3.02678708], Cost: 0.5845
Current parameters: [3.4122854  0.2352302  1.80263983 3.08349539 2.58976169 0.94869244
 0.0580931  0.53265954 2.30029865 2.76301241 0.91775403 1.03379267
 2.34338745 2.34442536 2.67597208 0.31456195 2.82013816 3.03367071], Cost: 0.2409
Current parameters: [3.41425928 0.23122441 1.79908171 3.07647952 2.58984295 0.94698279
 0.05935051 0.76726781 2.38270243 2.76095408 0.91551328 1.022888
 2.33313111 2.33170891 2.66947856 0.30743482 2.81174284 3.02879994], Cost: 0.5506
Current parameters: [3.4122854  0.2352302  1.80263983 3.08349539 2.58976169 0.94869244
 0.0580931  0.53265954 2.30029865 2.85506846 0.83319232 1.03379267
 2.34338745 2.34442536 2.67597208 0.31456195 2.82013816 3.03367071], Cost: 0.1915
Current parameters: [3.41351699 0.23273082 1.80041977 3.0791179  2.58981239 0.94762572
 0.05887765 0.53153249 2.29876939 3.02453157 1.01628238 1.02698879
 2.33698809 2.33649104 2.6719205  0.31011504 2.81489997 3.03063163], Cost: 0.7216
Current parameters: [3.4122854  0.2352302  1.80263983 3.08349539 2.58976169 0.94869244
```

```
 0.0580931  0.53265954 2.30029865 2.85506846 0.83319232 1.1194328
 2.2523338  2.34442536 2.67597208 0.31456195 2.82013816 3.03367071], Cost: 0.1830
Current parameters: [3.41921625 0.22116474 1.79014627 3.0588607  2.59004701 0.94268939
 0.06250823 0.52631702 2.29169261 2.84305731 0.81228395 1.29310932
 2.41442941 2.29977436 2.65317152 0.28953661 2.79065983 3.01656808], Cost: 0.8029
Current parameters: [3.4122854  0.2352302  1.80263983 3.08349539 2.58976169 0.94869244
 0.0580931  0.53265954 2.30029865 2.85506846 0.83319232 1.1194328
 2.2523338  2.40127259 2.56464647 0.31456195 2.82013816 3.03367071], Cost: 0.3053
Current parameters: [3.42035524 0.21885328 1.78809313 3.05481234 2.5900939  0.94170288
 0.0632338  0.52527471 2.29027833 2.84108344 0.80884795 1.11464031
 2.24636485 2.55084652 2.79853255 0.28542404 2.78581547 3.0137575 ], Cost: 0.3125
Current parameters: [3.4122854  0.2352302  1.80263983 3.08349539 2.58976169 0.94869244
 0.0580931  0.53265954 2.30029865 2.85506846 0.83319232 1.1194328
 2.2523338  2.34442536 2.67597208 0.40985418 2.73924075 3.03367071], Cost: 0.1196
Current parameters: [3.42057399 0.21840936 1.78769882 3.05403484 2.5901029  0.94151341
 0.06337314 0.52507453 2.29000671 2.84070435 0.80818805 1.1145104
 2.24620305 2.3583544  2.70070243 0.57276016 2.91856884 3.01321772], Cost: 0.7395
Current parameters: [3.40352404 0.24178733 1.81108892 3.09388741 2.59755478 0.95637333
 0.06015517 0.53786341 2.30216644 2.85740038 0.8357309  1.13178691
 2.2639534  2.35883206 2.68332871 0.41792864 2.74875197 3.1538974 ], Cost: 0.2906
Current parameters: [3.4769415  0.172032   1.73059782 2.97942793 2.54300972 0.8921317
 0.05296076 0.4905439  2.27462495 2.82090249 0.78293375 1.03769875
 2.17336568 2.2765448  2.66588143 0.36138835 2.66462584 3.06868289], Cost: 1.0048
Current parameters: [3.30773762 0.20034279 1.80711225 3.05027768 2.60272877 0.92400381
 0.08284129 0.52116001 2.32836723 2.85450276 0.83257648 1.11643579
 2.24951497 2.3409304  2.67418742 0.40789538 2.7369334  3.03233204], Cost: 0.1337
Current parameters: [ 3.28521953  0.32981555  1.75838085  3.15472083  2.52918636  1.01307321
 -0.02782445  0.55605864  2.19125169  2.84119121  0.8108573   1.10083456
  2.23368309  2.33791433  2.68624144  0.40043959  2.71845532  3.00745197], Cost: 0.3116
Current parameters: [3.41141692 0.27406476 1.8624514  3.07392008 2.59006971 0.94158811
 0.06238915 0.52887976 2.3053074  2.85428166 0.83233578 1.13517961
 2.1930503  2.29597127 2.71183137 0.37184488 2.77786432 3.03581377], Cost: 0.3899
Current parameters: [3.41316119 0.3277864  1.94721292 3.03770038 2.58942778 0.927136
 0.07085471 0.51782776 2.3013504  2.84096797 0.80991904 1.07058969
 2.33290492 2.41933465 2.62461376 0.46423405 2.6488467  3.00065371], Cost: 0.2213
Current parameters: [3.41225415 0.2206597  1.80307522 3.16470235 2.67364314 0.93155822
 0.0670907  0.5214405  2.30306704 2.8462806  0.81914545 1.10354431
 2.24922722 2.34662674 2.67525355 0.40935326 2.71850947 3.01630013], Cost: 0.2751
Current parameters: [3.41251724 0.22994594 1.80595775 3.07924754 2.59433685 0.99267396
 0.07632934 0.49393012 2.29299437 2.8548267  0.83292913 1.118152
 2.25112915 2.34293176 2.67520939 0.40901707 2.73825468 3.03309862], Cost: 0.1283
```



```
Current parameters: [3.41252997 0.22463005 1.80261549 3.07359367 2.59837274 1.01645362
 0.08588875 0.62168992 2.33726769 2.84884784 0.82339027 1.10721895
 2.2488521  2.34420248 2.67461202 0.40855601 2.72408932 3.02126668], Cost: 0.3341
Current parameters: [3.41229941 0.27791585 1.76946521 3.07264495 2.60416482 0.94400772
 0.07550672 0.52655078 2.3172652  2.85698855 0.83528257 1.11935539
 2.25226099 2.34433509 2.67592598 0.40980359 2.73918115 3.03363613], Cost: 0.0977
Current parameters: [3.41097892 0.27478732 1.7658452  3.07189137 2.60650035 0.94183089
 0.07211694 0.52553131 2.30716935 2.94159636 0.92310041 1.11127681
 2.25599329 2.35475664 2.67948387 0.41398404 2.7248264  3.02019376], Cost: 0.2347
Current parameters: [3.41229941 0.27791585 1.76946521 3.08980117 2.58128715 0.93080966
 0.12466757 0.53408617 2.29627078 2.85698855 0.83528257 1.11935539
 2.25226099 2.34433509 2.67592598 0.40980359 2.73918115 3.03363613], Cost: 0.1136
Current parameters: [3.41124224 0.27897568 1.76641432 3.12447314 2.53732988 0.94500425
 0.06629973 0.49412903 2.40170187 2.85867454 0.83475136 1.1167115
 2.25602725 2.35220225 2.6789699  0.41329651 2.73265614 3.02702818], Cost: 0.1818
Current parameters: [3.41075994 0.28104005 1.77224026 3.07811677 2.60410144 0.9453411
 0.07452604 0.52795957 2.31917677 2.86052322 0.83913051 1.12262717
 2.2675799  2.29701103 2.64174796 0.42216009 2.73995783 3.03743495], Cost: 0.2470
Current parameters: [3.4143499  0.2714749  1.75536042 3.0605377  2.60502727 0.93847416
 0.07352178 0.51922979 2.30909451 2.85072388 0.82349284 1.2090821
 2.31461798 2.35873707 2.7099987  0.38219094 2.72389364 3.00922169], Cost: 0.2089
Current parameters: [3.43482573 0.25086112 1.76169182 3.06333534 2.57974212 0.91684514
 0.05324716 0.53384052 2.30383983 2.85398765 0.83201572 1.11402993
 2.24603884 2.33822176 2.67379665 0.4112042  2.72130496 3.03109878], Cost: 0.1274
Current parameters: [3.40612679 0.29291289 1.72211871 3.02758756 2.62296159 0.95530982
 0.10521109 0.47683339 2.31917815 2.84264839 0.80843197 1.07093685
 2.23000293 2.36643408 2.68783451 0.37812015 2.69899756 3.04900274], Cost: 0.2120
Current parameters: [3.41405564 0.27414495 1.75847461 3.06322435 2.6045321  0.94029088
 0.07487988 0.51990936 2.31224754 2.85284141 0.82723209 1.11341892
 2.2532759  2.3625802  2.68603248 0.44984928 2.77490822 3.02089291], Cost: 0.0340
Current parameters: [3.40436166 0.27152364 1.73935961 3.07300384 2.60086136 0.94799545
 0.06811332 0.52267595 2.30454405 2.85332731 0.82776105 1.11632819
 2.24511679 2.35546825 2.69131223 0.44438601 2.78153022 3.02206474], Cost: 0.0447
Current parameters: [3.39175605 0.30345965 1.77090382 3.06963699 2.58855729 0.94395658
 0.05428282 0.5232058  2.28744074 2.8469606  0.81937084 1.10276225
 2.26168197 2.37948863 2.68214659 0.45992224 2.75935177 3.01041282], Cost: 0.0169
Current parameters: [3.39136639 0.30394969 1.77079312 3.06953633 2.58917318 0.94457235
 0.05499229 0.52281859 2.28782257 2.8264782  0.83812088 1.09862878
 2.26582613 2.37404113 2.69263273 0.46402774 2.75579953 3.01077428], Cost: 0.0197
Current parameters: [3.39258192 0.30248512 1.76734032 3.0677981  2.587637   0.94160423
 0.05224871 0.52123605 2.28527484 2.82531719 0.83703403 1.1162949
```



```
 2.24622942 2.40844498 2.6440591  0.44799718 2.77382665 3.00528895], Cost: 0.0150
Current parameters: [3.39113064 0.30460329 1.76739428 3.06735058 2.58992263 0.94383188
 0.05491222 0.51977124 2.28672923 2.82523469 0.83685704 1.13123444
 2.2290862  2.40743973 2.64489525 0.4636619  2.7603295  3.00664562], Cost: 0.0144
Current parameters: [3.39437534 0.32291112 1.79484546 3.05789227 2.58506486 0.93126693
 0.04936691 0.51476853 2.28283539 2.82232653 0.83372884 1.13450904
 2.19923228 2.40069316 2.66760536 0.45031745 2.78586633 2.99425968], Cost: 0.0455
Current parameters: [3.39233108 0.30222244 1.76534381 3.06315266 2.58982948 0.95345449
 0.05948164 0.53166864 2.29232114 2.82389715 0.8349526  1.1270527
 2.22633769 2.40463771 2.64328003 0.46191786 2.75625951 2.98213438], Cost: 0.0329
Current parameters: [3.39443503 0.30208575 1.77467986 3.0598349  2.58467368 0.9604818
 0.0533087  0.55893361 2.29305714 2.82067039 0.83089559 1.12868454
 2.2275758  2.43267502 2.65615262 0.47256846 2.76882363 3.03694696], Cost: 0.0286
Current parameters: [3.41592761 0.31629398 1.76326228 3.07611896 2.59135005 0.95322426
 0.05300459 0.51752283 2.2811607  2.82527621 0.83676664 1.13132335
 2.22952809 2.4081708  2.6452128  0.46401914 2.76014072 3.0063744 ], Cost: 0.0144
Current parameters: [3.41404062 0.30329269 1.76979036 3.11272796 2.62715137 0.9345931
 0.04820586 0.52141439 2.27905735 2.81816967 0.82666746 1.12160902
 2.22534959 2.42526339 2.65206533 0.46909059 2.75731778 2.99987486], Cost: 0.0020
Current parameters: [3.41422395 0.30128587 1.77442485 3.11711812 2.62645599 0.93318772
 0.04412089 0.52696637 2.27671344 2.83715907 0.84780377 1.12465255
 2.22695331 2.42897259 2.65417721 0.4727215  2.75938708 2.99524606], Cost: 0.0075
Current parameters: [3.41030232 0.3098684  1.74891105 3.09307155 2.64022841 0.93398059
 0.05793246 0.49606915 2.27353939 2.82895065 0.83116618 1.09475608
 2.21665006 2.45529188 2.66242542 0.47035664 2.74646242 3.00499165], Cost: 0.0241
Current parameters: [3.41444848 0.30242314 1.76895574 3.10026534 2.64179268 0.93380448
 0.04996607 0.52768097 2.25978519 2.81721577 0.82557519 1.13258452
 2.23224267 2.42343567 2.65403153 0.46562695 2.75701639 2.99865881], Cost: 0.0068
Current parameters: [3.41320804 0.30289835 1.76249468 3.11965416 2.61548833 0.92982362
 0.0446783  0.50689174 2.29446247 2.81327356 0.81752498 1.15602366
 2.25191551 2.45000285 2.67324848 0.46650062 2.75628748 2.98898679], Cost: 0.1170
Current parameters: [3.41385402 0.32030133 1.75722065 3.11081009 2.63058562 0.91766045
 0.04784771 0.53292933 2.28768676 2.81917956 0.82776684 1.12226469
 2.22684408 2.422361   2.6498653  0.47025183 2.75769764 3.00033534], Cost: 0.0034
Current parameters: [3.40923393 0.30661653 1.77861742 3.11562414 2.6406343  0.93768803
 0.04525395 0.51595783 2.27084741 2.81751638 0.82485568 1.10200642
 2.22777229 2.40426532 2.62050803 0.50370406 2.77550376 3.01123658], Cost: 0.0254
Current parameters: [3.41937005 0.2968919  1.76795128 3.10296838 2.63145055 0.93398034
 0.02128494 0.51981982 2.28512882 2.8174597  0.82589456 1.12034909
 2.22387751 2.42381705 2.65156156 0.46942196 2.75308851 2.99927456], Cost: 0.0024
Current parameters: [3.43333089 0.28022869 1.76386811 3.11211731 2.59813563 0.90310449
```



```
 0.05135892 0.53051171 2.25444815 2.81451705 0.82236986 1.1103625
 2.2169897  2.42437155 2.64869434 0.4776768  2.74632303 3.00005056], Cost: 0.0200
Current parameters: [3.39988184 0.29980816 1.77219664 3.11431338 2.62838585 0.92665631
 0.04875363 0.51353759 2.280137   2.82025564 0.830356   1.13188173
 2.22145478 2.42025364 2.64657864 0.46842918 2.74119884 3.01304704], Cost: 0.0076
Current parameters: [3.42853497 0.31571846 1.78564482 3.12321551 2.63438273 0.94693812
 0.04700862 0.53921424 2.27710263 2.8187624  0.82981408 1.09840911
 2.2208566  2.41732077 2.63999893 0.43091218 2.73867932 3.00831813], Cost: 0.0709
Current parameters: [3.40401176 0.30108292 1.75108916 3.12276917 2.6246932  0.94209469
 0.04115576 0.52415888 2.2702937  2.8183998  0.82690439 1.12215746
 2.21593772 2.41919236 2.65654261 0.46502877 2.76478042 3.00234481], Cost: 0.0047
Current parameters: [3.41595907 0.30078128 1.76887688 3.11227737 2.62810036 0.93401555
 0.04946965 0.52134218 2.28080406 2.82836042 0.8183681  1.12440164
 2.22272683 2.42653791 2.64738414 0.46635664 2.76021503 3.00051767], Cost: 0.0013
Current parameters: [3.42632811 0.28698828 1.76298481 3.10943215 2.63709756 0.93231073
 0.05942161 0.51961966 2.29202189 2.82461651 0.82779883 1.12627679
 2.21876694 2.41744518 2.65189646 0.46390184 2.76747192 3.00687399], Cost: 0.0042
Current parameters: [3.41606065 0.30085315 1.76854534 3.11161905 2.62779035 0.93306192
 0.04885693 0.52063075 2.28018729 2.82292629 0.82272794 1.12795192
 2.21821809 2.43359279 2.63833013 0.46321544 2.76424908 2.99911911], Cost: 0.0013
Current parameters: [3.41342326 0.30845144 1.78230793 3.10710365 2.62746761 0.92401119
 0.04638217 0.51529159 2.27763415 2.82276364 0.82073722 1.12923763
 2.20132847 2.42896425 2.65016648 0.46098362 2.77521939 2.99804573], Cost: 0.0084
Current parameters: [3.41589313 0.30043545 1.7706631  3.11039854 2.62663883 0.93496333
 0.04782083 0.52745161 2.28083648 2.82212225 0.82170724 1.1284826
 2.21790052 2.43974477 2.64117118 0.46592714 2.76653591 3.01027397], Cost: 0.0044
Current parameters: [3.40555797 0.29409149 1.76936234 3.10387272 2.62979554 0.93915295
 0.05443187 0.534605   2.28741715 2.82129948 0.82020618 1.12747785
 2.20633656 2.43399319 2.63777917 0.47168785 2.75732523 2.98609895], Cost: 0.0075
Current parameters: [3.42430005 0.30575069 1.76712608 3.11456023 2.62910973 0.93718266
 0.04910885 0.51929439 2.27872263 2.82291115 0.82262853 1.13326702
 2.21236421 2.43344061 2.63865881 0.46898209 2.7594733  2.99952481], Cost: 0.0014
Current parameters: [3.41290806 0.3020613  1.77079013 3.11242254 2.63416813 0.93444039
 0.04751666 0.51565484 2.27681094 2.82295152 0.82145628 1.12027244
 2.22173383 2.42443794 2.62369464 0.48252931 2.77540031 3.00237646], Cost: 0.0022
Current parameters: [3.41764916 0.29994426 1.77432749 3.1171555  2.62532855 0.93370772
 0.04643267 0.52745383 2.28157072 2.82003325 0.82138412 1.13351691
 2.220366   2.42633064 2.63545331 0.46066884 2.76565209 2.99830913], Cost: 0.0020
Current parameters: [3.42117283 0.30755997 1.77274458 3.11379956 2.63582968 0.94012432
 0.04998374 0.52453815 2.27762822 2.82527377 0.82516922 1.10932838
 2.21315475 2.42994034 2.62978727 0.4491162  2.75665263 3.00344391], Cost: 0.0140
```

```
Current parameters: [3.4174887  0.29564441 1.76940408 3.11998037 2.63312295 0.92568637
 0.0480145  0.52231528 2.27740834 2.82096649 0.81998855 1.12500409
 2.21672702 2.43765459 2.63978135 0.46518606 2.76253603 2.99752674], Cost: 0.0014
Current parameters: [3.42556061 0.29303837 1.76446101 3.10410946 2.60825272 0.92293111
 0.05143669 0.52400994 2.26875076 2.8228318  0.82279832 1.12249302
 2.21458559 2.42844877 2.6337461  0.46667433 2.76037459 3.00033898], Cost: 0.0060
Current parameters: [3.41887402 0.30122554 1.76122572 3.11723737 2.62195997 0.93447617
 0.04835872 0.50705786 2.28821302 2.81879244 0.81364175 1.13539688
 2.23409969 2.44156431 2.64023664 0.46603396 2.76839237 2.98917433], Cost: 0.0227
Current parameters: [3.41640757 0.29986064 1.770506   3.11419168 2.62721638 0.93257175
 0.04679486 0.52271434 2.27929141 2.8324879  0.83308945 1.12928603
 2.21970385 2.43556363 2.63905844 0.46538624 2.76568329 2.99617799], Cost: 0.0048
Current parameters: [3.41477054 0.30360195 1.76808775 3.11382012 2.62700579 0.93199879
 0.05528079 0.52183557 2.27934937 2.82316854 0.82299166 1.12830061
 2.21867755 2.4337319  2.63829362 0.46321903 2.76528943 2.99929583], Cost: 0.0012
Current parameters: [3.41550829 0.3108641  1.76168544 3.11159389 2.62921218 0.92383599
 0.05126564 0.52694772 2.2844382  2.82353162 0.82331336 1.1283807
 2.2193596  2.43215395 2.63707554 0.46390923 2.76496736 2.99927763], Cost: 0.0021
Current parameters: [3.41549793 0.30372535 1.77254424 3.11176783 2.62765452 0.9294646
 0.05691718 0.52035806 2.28147833 2.82334341 0.82333758 1.12925505
 2.22035393 2.43456001 2.63669463 0.46406765 2.76188342 3.0001603 ], Cost: 0.0015
Current parameters: [3.40612793 0.30155202 1.76444103 3.11723363 2.62680774 0.93019179
 0.05396609 0.51795458 2.27817675 2.82410875 0.82434424 1.13303001
 2.21551291 2.43008355 2.6363339  0.46236117 2.75988307 3.00534659], Cost: 0.0023
Current parameters: [3.4126197  0.30642078 1.76923216 3.1128129  2.62698666 0.93225878
 0.05339284 0.52301246 2.27447229 2.82385337 0.82083459 1.12932454
 2.22040848 2.43543127 2.63758261 0.46329008 2.76371003 2.9977871 ], Cost: 0.0024
Current parameters: [3.41763779 0.29927644 1.76541    3.1081626  2.63567499 0.93124181
 0.05882409 0.52309939 2.2744356  2.82165406 0.82490661 1.13365483
 2.22107171 2.43022811 2.63994809 0.46132093 2.76728593 3.00031742], Cost: 0.0016
Current parameters: [3.4118777  0.30137131 1.76733467 3.11014819 2.62766518 0.93385162
 0.05483037 0.52312157 2.28163623 2.82205449 0.82244362 1.12808185
 2.21639976 2.43430108 2.63745255 0.46387084 2.76373951 2.99582445], Cost: 0.0019
Current parameters: [3.414468   0.30364849 1.76988265 3.11971401 2.62737076 0.93160444
 0.06343448 0.52984174 2.27909647 2.82505474 0.822254   1.12942157
 2.21576557 2.43120674 2.64031445 0.47028349 2.76456897 2.99864065], Cost: 0.0015
Current parameters: [3.41702255 0.30126877 1.76666622 3.11162987 2.62216002 0.92954895
 0.05584401 0.52233374 2.27674755 2.82290375 0.82296831 1.12710794
 2.21780455 2.43242611 2.63709722 0.46394715 2.76431962 2.99944252], Cost: 0.0016
Current parameters: [3.41527104 0.3035628  1.76786298 3.11412267 2.62624701 0.93179411
 0.05573516 0.52267671 2.27969493 2.82332754 0.82316116 1.12929554
```



```
 2.21831722 2.43478631 2.64015186 0.46093705 2.763837   2.99889816], Cost: 0.0016
Current parameters: [3.41477054 0.30360195 1.76808775 3.11382012 2.62700579 0.93199879
 0.05528079 0.52183557 2.27934937 2.82316854 0.82299166 1.12830061
 2.21867755 2.4337319  2.63829362 0.46321903 2.76528943 2.99929583], Cost: 0.0012

   Return from subroutine COBYLA because the MAXFUN limit has been reached.

   NFVALS =  100   F = 1.170525E-03    MAXCV = 0.000000E+00
   X = 3.414771E+00   3.036020E-01   1.768088E+00   3.113820E+00   2.627006E+00
       9.319988E-01   5.528079E-02   5.218356E-01   2.279349E+00   2.823169E+00
       8.229917E-01   1.128301E+00   2.218678E+00   2.433732E+00   2.638294E+00
       4.632190E-01   2.765289E+00   2.999296E+00
Simulating for QAOA depth p = 10
Current parameters: [3.53336405 1.07871841 0.95376569 0.90723418 2.1500727  0.85955659
 0.78678161 2.37401902 2.02801192 2.96096553 2.01203439 0.83152326
 1.68170502 1.06413259 1.32704556 1.83120823 1.32418589 3.02488161
 2.8033615  0.54933461], Cost: 2.3665
Current parameters: [2.53336405 2.07871841 0.95376569 0.90723418 2.1500727  0.85955659
 0.78678161 2.37401902 2.02801192 2.96096553 2.01203439 0.83152326
 1.68170502 1.06413259 1.32704556 1.83120823 1.32418589 3.02488161
 2.8033615  0.54933461], Cost: 3.0372
Current parameters: [2.53336405 1.07871841 1.95376569 0.90723418 2.1500727  0.85955659
 0.78678161 2.37401902 2.02801192 2.96096553 2.01203439 0.83152326
 1.68170502 1.06413259 1.32704556 1.83120823 1.32418589 3.02488161
 2.8033615  0.54933461], Cost: 1.5477
Current parameters: [2.53336405 1.07871841 1.95376569 1.90723418 2.1500727  0.85955659
 0.78678161 2.37401902 2.02801192 2.96096553 2.01203439 0.83152326
 1.68170502 1.06413259 1.32704556 1.83120823 1.32418589 3.02488161
 2.8033615  0.54933461], Cost: 2.0221
Current parameters: [2.53336405 1.07871841 1.95376569 0.90723418 3.1500727  0.85955659
 0.78678161 2.37401902 2.02801192 2.96096553 2.01203439 0.83152326
 1.68170502 1.06413259 1.32704556 1.83120823 1.32418589 3.02488161
 2.8033615  0.54933461], Cost: 2.5040
Current parameters: [2.53336405 1.07871841 1.95376569 0.90723418 2.1500727  1.85955659
 0.78678161 2.37401902 2.02801192 2.96096553 2.01203439 0.83152326
 1.68170502 1.06413259 1.32704556 1.83120823 1.32418589 3.02488161
 2.8033615  0.54933461], Cost: 3.0835
Current parameters: [2.53336405 1.07871841 1.95376569 0.90723418 2.1500727  0.85955659
 1.78678161 2.37401902 2.02801192 2.96096553 2.01203439 0.83152326
 1.68170502 1.06413259 1.32704556 1.83120823 1.32418589 3.02488161
 2.8033615  0.54933461], Cost: 3.2749
```



```
Current parameters: [2.53336405 1.07871841 1.95376569 0.90723418 2.1500727  0.85955659
 0.78678161 3.37401902 2.02801192 2.96096553 2.01203439 0.83152326
 1.68170502 1.06413259 1.32704556 1.83120823 1.32418589 3.02488161
 2.8033615  0.54933461], Cost: 3.2038
Current parameters: [2.53336405 1.07871841 1.95376569 0.90723418 2.1500727  0.85955659
 0.78678161 2.37401902 3.02801192 2.96096553 2.01203439 0.83152326
 1.68170502 1.06413259 1.32704556 1.83120823 1.32418589 3.02488161
 2.8033615  0.54933461], Cost: 3.1448
Current parameters: [2.53336405 1.07871841 1.95376569 0.90723418 2.1500727  0.85955659
 0.78678161 2.37401902 2.02801192 3.96096553 2.01203439 0.83152326
 1.68170502 1.06413259 1.32704556 1.83120823 1.32418589 3.02488161
 2.8033615  0.54933461], Cost: 3.2045
Current parameters: [2.53336405 1.07871841 1.95376569 0.90723418 2.1500727  0.85955659
 0.78678161 2.37401902 2.02801192 2.96096553 3.01203439 0.83152326
 1.68170502 1.06413259 1.32704556 1.83120823 1.32418589 3.02488161
 2.8033615  0.54933461], Cost: 3.2719
Current parameters: [2.53336405 1.07871841 1.95376569 0.90723418 2.1500727  0.85955659
 0.78678161 2.37401902 2.02801192 2.96096553 2.01203439 1.83152326
 1.68170502 1.06413259 1.32704556 1.83120823 1.32418589 3.02488161
 2.8033615  0.54933461], Cost: 1.3139
Current parameters: [2.53336405 1.07871841 1.95376569 0.90723418 2.1500727  0.85955659
 0.78678161 2.37401902 2.02801192 2.96096553 2.01203439 1.83152326
 2.68170502 1.06413259 1.32704556 1.83120823 1.32418589 3.02488161
 2.8033615  0.54933461], Cost: 1.9426
Current parameters: [2.53336405 1.07871841 1.95376569 0.90723418 2.1500727  0.85955659
 0.78678161 2.37401902 2.02801192 2.96096553 2.01203439 1.83152326
 1.68170502 2.06413259 1.32704556 1.83120823 1.32418589 3.02488161
 2.8033615  0.54933461], Cost: 1.3200
Current parameters: [2.53336405 1.07871841 1.95376569 0.90723418 2.1500727  0.85955659
 0.78678161 2.37401902 2.02801192 2.96096553 2.01203439 1.83152326
 1.68170502 1.06413259 2.32704556 1.83120823 1.32418589 3.02488161
 2.8033615  0.54933461], Cost: 2.1527
Current parameters: [2.53336405 1.07871841 1.95376569 0.90723418 2.1500727  0.85955659
 0.78678161 2.37401902 2.02801192 2.96096553 2.01203439 1.83152326
 1.68170502 1.06413259 1.32704556 2.83120823 1.32418589 3.02488161
 2.8033615  0.54933461], Cost: 1.9492
Current parameters: [2.53336405 1.07871841 1.95376569 0.90723418 2.1500727  0.85955659
 0.78678161 2.37401902 2.02801192 2.96096553 2.01203439 1.83152326
 1.68170502 1.06413259 1.32704556 1.83120823 2.32418589 3.02488161
 2.8033615  0.54933461], Cost: 2.5073
Current parameters: [2.53336405 1.07871841 1.95376569 0.90723418 2.1500727  0.85955659
```



```
 0.78678161 2.37401902 2.02801192 2.96096553 2.01203439 1.83152326
 1.68170502 1.06413259 1.32704556 1.83120823 1.32418589 4.02488161
 2.8033615  0.54933461], Cost: 2.7718
Current parameters: [2.53336405 1.07871841 1.95376569 0.90723418 2.1500727  0.85955659
 0.78678161 2.37401902 2.02801192 2.96096553 2.01203439 1.83152326
 1.68170502 1.06413259 1.32704556 1.83120823 1.32418589 3.02488161
 3.8033615  0.54933461], Cost: 1.0448
Current parameters: [2.53336405 1.07871841 1.95376569 0.90723418 2.1500727  0.85955659
 0.78678161 2.37401902 2.02801192 2.96096553 2.01203439 1.83152326
 1.68170502 1.06413259 1.32704556 1.83120823 1.32418589 3.02488161
 3.8033615  1.54933461], Cost: 1.4294
Current parameters: [2.44169381 0.85134191 2.02780172 0.8112318  1.9565552  0.54877372
 0.43726609 2.03890209 1.70482624 2.62569423 1.66312873 1.87881468
 1.5544912  1.06290501 1.1573281  1.70267062 1.08271434 2.72987182
 3.85781722 0.47151261], Cost: 1.1741
Current parameters: [2.51875973 1.04249423 1.96556064 0.89193969 2.11924272 1.33607583
 0.73109902 2.32063032 1.97652403 2.90755224 1.95644895 1.83905743
 1.66143812 1.06393702 1.30000725 1.81073044 1.28571618 2.97788253
 3.81203704 0.53693651], Cost: 0.8996
Current parameters: [2.28689447 1.13597418 1.96556064 0.89193969 2.11924272 1.33607583
 0.73109902 2.32063032 1.97652403 2.90755224 1.95644895 1.83905743
 1.66143812 1.06393702 1.30000725 1.81073044 1.28571618 2.97788253
 3.81203704 0.53693651], Cost: 0.9256
Current parameters: [2.7021712  1.48957996 1.97588378 0.8785537  2.09225981 1.34496854
 0.68236469 2.27390364 1.93146097 2.86080404 1.90779966 1.84565146
 1.6437002  1.06376585 1.27634287 1.79280793 1.25204685 2.93674815
 3.81963002 0.52608548], Cost: 1.1241
Current parameters: [2.51875973 1.04249423 1.96556064 0.89193969 2.11924272 1.33607583
 0.90772128 2.32063032 1.97652403 2.90755224 1.77951796 1.83905743
 1.66143812 1.06393702 1.30000725 1.81073044 1.28571618 2.97788253
 3.81203704 0.53693651], Cost: 1.0488
Current parameters: [2.51437259 1.0128151  1.99030108 0.85985881 2.05457546 1.3573881
 1.00291815 2.20864512 1.86852587 2.79551545 2.28481566 1.85486069
 1.61892739 1.0635268  1.24329319 1.76777734 1.20502422 2.87929983
 3.83023437 0.51093092], Cost: 1.1640
Current parameters: [2.51875973 1.04249423 1.96556064 0.89193969 2.11924272 1.33607583
 0.73109902 2.14381292 1.97652403 3.08428822 1.95644895 1.83905743
 1.66143812 1.06393702 1.30000725 1.81073044 1.28571618 2.97788253
 3.81203704 0.53693651], Cost: 1.2713
Current parameters: [2.51319031 1.00481693 1.99696834 0.85121339 2.03714839 1.36313151
 0.66782781 2.69367826 1.83942163 3.10024617 1.96567673 1.85911948
```

```
 1.60747125 1.06341625 1.22800941 1.75620199 1.18327869 2.85273295
 3.83513834 0.50392272], Cost: 0.1647
Current parameters: [2.50664257 0.96052135 2.03389301 0.80333324 1.94063361 1.3949397
 0.59344257 2.87382099 2.21443208 3.06823541 1.97652543 1.88270557
 1.54402472 1.06280401 1.14336465 1.69209523 1.06284734 2.70559998
 3.86229754 0.46510983], Cost: 2.2463
Current parameters: [2.51319031 1.00481693 1.99696834 0.62725755 2.14825097 1.36313151
 0.66782781 2.69367826 1.83942163 3.10024617 1.96567673 1.85911948
 1.60747125 1.06341625 1.22800941 1.75620199 1.18327869 2.85273295
 3.83513834 0.50392272], Cost: 0.3120
Current parameters: [2.5106922  0.98791718 2.01105592 1.07092365 2.42898405 1.37526704
 0.63944818 2.76240672 1.65513271 3.08803335 1.96981575 1.8681181
 1.58326499 1.06318267 1.19571555 1.73174383 1.13733145 2.79659845
 3.84550018 0.48911474], Cost: 0.2058
Current parameters: [2.51319031 1.00481693 1.75448766 0.85121339 2.03714839 1.36313151
 0.66782781 2.69367826 1.83942163 3.10024617 1.96567673 1.85911948
 1.60747125 1.06341625 1.22800941 1.75620199 1.18327869 2.79187969
 3.83513834 0.50392272], Cost: 0.4308
Current parameters: [2.50991563 0.98266365 1.95113873 0.83997329 1.94757552 1.37903954
 0.63062597 2.78377195 1.59784385 3.08423682 1.97110243 1.87091545
 1.57574012 1.06311006 1.18567654 1.72414066 1.12304809 3.25608629
 3.84872131 0.48451146], Cost: 0.9181
Current parameters: [2.51319031 1.00481693 1.99696834 0.85121339 2.03714839 1.36313151
 0.66782781 2.69367826 1.83942163 3.10024617 1.96567673 1.85911948
 1.60747125 1.06341625 1.02347533 1.75620199 1.32703477 2.85273295
 3.83513834 0.50392272], Cost: 0.6131
Current parameters: [2.51069353 0.9879262  2.07379809 0.8426434  1.96885371 1.37526056
 0.63946333 2.76237003 1.65523108 3.08803987 1.96981354 1.86811329
 1.58327791 1.06318279 1.52430563 1.73175689 1.48481438 2.71489164
 3.84549465 0.48912264], Cost: 0.9710
Current parameters: [2.51319031 1.00481693 1.99696834 0.85121339 2.03714839 1.36313151
 0.66782781 2.69367826 1.83942163 3.10024617 1.96567673 1.85911948
 1.42978193 1.06341625 1.22800941 1.93206132 1.18327869 2.85273295
 3.83513834 0.50392272], Cost: 0.1768
Current parameters: [2.5119293  0.99628619 2.03577155 0.84688508 2.00265586 1.36925734
 0.6535022  2.72837135 1.74639537 3.09408132 1.96776605 1.86366185
 1.9402713  1.06329834 1.2051795  2.09112435 1.09017478 2.78311558
 3.84036884 0.49644787], Cost: 1.1558
Current parameters: [2.51319031 1.00481693 1.99696834 0.85121339 2.03714839 1.36313151
 0.66782781 2.69367826 1.83942163 3.10024617 1.96567673 2.07276477
 1.60747125 1.06341625 1.22800941 1.75620199 1.18327869 2.85273295
```



```
 3.83513834 0.63375219], Cost: 1.5491
Current parameters: [2.51261921 1.00095344 2.01454193 0.84925314 2.02152706 1.36590584
 0.66133988 2.70939042 1.79729095 3.09745417 1.96662296 1.56260431
 1.5829571  1.06336285 1.21766997 1.7308255  1.14111284 2.82120393
 3.83750718 0.89800011], Cost: 1.0512
Current parameters: [2.51319031 1.00481693 1.99696834 0.85121339 2.03714839 1.36313151
 0.66782781 2.69367826 1.83942163 3.10024617 1.96567673 1.85911948
 1.60747125 0.81347975 1.22800941 1.75620199 1.18327869 2.85273295
 3.82950411 0.50392272], Cost: 0.3770
Current parameters: [2.51286384 1.00260836 2.00701431 0.85009281 2.02821843 1.36471746
 0.66411897 2.70266014 1.81533756 3.09865012 1.96621765 1.84431821
 1.59345769 1.07888151 1.22209885 1.74169547 1.15917451 2.83470933
 3.33868201 0.4746224 ], Cost: 1.9220
Current parameters: [2.50178757 0.93690301 2.01448484 0.82849978 1.99136327 1.29500576
 0.56697567 2.61644965 1.76295777 3.00202026 1.88593919 1.87030836
 1.57737319 1.06312581 1.18785524 1.72579073 1.12614791 2.78293534
 3.84802225 0.48551048], Cost: 1.2787
Current parameters: [2.35319492 1.19355917 2.03488331 0.84150491 1.9954707  1.26850251
 0.90415173 2.67186522 1.73942692 3.35553973 1.90380935 1.81302215
 1.55308723 1.07577405 1.19782403 1.70010168 1.08832006 2.77458167
 3.91522658 0.40196364], Cost: 0.8715
Current parameters: [2.5728068  0.94075497 1.99788892 0.86768495 2.0604529  1.41319864
 0.58764871 2.57996381 1.864735   3.2622612  2.0028099  1.82822541
 1.61070878 1.06921533 1.25129821 1.75895816 1.19312699 2.88171615
 3.8591869  0.47514909], Cost: 0.4165
Current parameters: [2.47550537 0.91514016 1.99866937 0.84900766 2.03270218 1.36263897
 0.72304309 2.68695681 1.83199619 3.0931789  2.0187901  1.86020604
 1.60454842 1.06338805 1.22411002 1.75324874 1.1777307  2.84595488
 3.8363895  0.5021347 ], Cost: 0.2386
Current parameters: [2.63117577 1.09410231 2.01004965 0.84473086 2.01820907 1.40797144
 0.70832074 2.6444122  1.79971972 3.03169809 2.11662845 1.84879845
 1.58785312 1.06664873 1.21309041 1.73607416 1.14823724 2.81997869
 3.85862524 0.47367647], Cost: 0.4720
Current parameters: [2.51696932 1.01276241 1.99430176 0.85467114 2.04411837 1.25628784
 0.64126901 2.70538034 1.85106191 3.11208256 2.0172861  1.85741617
 1.61205315 1.06346047 1.23412218 1.76083156 1.19197584 2.86335841
 3.83317699 0.50672566], Cost: 0.2120
Current parameters: [2.66265728 0.95610394 1.99345578 0.87024368 2.06739793 1.2998408
 0.79359877 2.68761365 1.87879047 3.08008595 1.86537275 1.83107822
 1.61745591 1.06823897 1.25687442 1.7658685  1.20526547 2.89348512
 3.85170615 0.48482968], Cost: 0.2352
```



```
Current parameters: [2.40522766 1.06505865 1.98756135 0.88736635 2.09660163 1.37502123
 0.68889518 2.59128058 1.92026096 3.01254297 1.96990912 1.81041944
 1.63017023 1.07145675 1.28401604 1.77843875 1.23138629 2.93449364
 3.85991199 0.47610455], Cost: 0.4049
Current parameters: [2.51300724 0.99667446 2.0062937  0.85743525 2.03942848 1.38644993
 0.65066014 2.77683008 1.90121747 3.09976866 1.97376534 1.83243787
 1.59644409 1.0692895  1.2329642  1.74452612 1.16618641 2.84942494
 3.86622719 0.46529333], Cost: 0.2854
Current parameters: [2.51279915 1.00217076 1.98653076 0.84698644 2.02862788 1.36503169
 0.66338413 2.70443976 1.81056567 3.09833389 1.96632482 1.85940011
 1.60671637 1.06270732 1.22700231 1.75543925 1.18184581 2.89432336
 3.86658671 0.50346093], Cost: 0.1483
Current parameters: [2.51400907 1.00543175 1.97891313 0.84765661 2.02265984 1.37515117
 0.66636818 2.72686642 1.78970998 3.10454674 1.96987286 1.8399669
 1.59993559 1.06965872 1.2319826  1.74820707 1.17171292 2.94882045
 3.7662187  0.47657242], Cost: 0.0035
Current parameters: [2.51393333 1.00491935 1.98003264 0.84683812 2.02100995 1.37551912
 0.66550772 2.72895024 1.7841224  3.10417646 1.96999836 1.86194707
 1.63174484 1.06965164 1.23030514 1.78034733 1.16932625 2.94435959
 3.76653287 0.44040222], Cost: 0.0197
Current parameters: [2.51522749 1.00891728 1.98589456 0.84883953 2.01797378 1.38468648
 0.6698139  2.74716859 1.77321777 3.110791   1.97321838 1.76921584
 1.65322572 1.0743758  1.23077651 1.80168383 1.15336255 2.94432561
 3.75790676 0.53612972], Cost: 0.0630
Current parameters: [2.52416913 0.99287447 1.98120018 0.84633927 2.02000439 1.38377522
 0.65017588 2.73704728 1.77790247 3.08881692 1.97577433 1.83861941
 1.60143408 1.06975409 1.26162401 1.74972116 1.21388629 2.93970731
 3.76198814 0.47878983], Cost: 0.0452
Current parameters: [2.47942626 1.0476035  1.99222154 0.84452999 2.00754684 1.36123304
 0.71720259 2.74018989 1.74751755 3.1627562  1.95497152 1.81562652
 1.58596392 1.07512635 1.26108184 1.73363157 1.19238707 2.92485426
 3.77406043 0.44576233], Cost: 0.0770
Current parameters: [2.5143216  1.00754598 1.97715071 0.82271227 1.9723781  1.37363296
 0.6699186  2.71826817 1.81276538 3.10607462 1.96935505 1.8398919
 1.60296391 1.06968795 1.23602271 1.7512669  1.17746113 2.95584314
 3.76492239 0.47906351], Cost: 0.0087
Current parameters: [2.51285178 1.02529994 1.98565282 0.86084428 2.03085385 1.39238701
 0.69105834 2.75588882 1.8029438  3.13187421 1.97453512 1.92105441
 1.60465984 1.0819437  1.25684358 1.75202362 1.16382196 2.98262597
 3.7438577  0.53281872], Cost: 0.3131
Current parameters: [2.51294466 0.99909214 1.98054825 0.84553635 2.01838591 1.3687918
```



```
 0.65695387 2.71965731 1.78257226 3.09537759 1.96242955 1.84101136
 1.59712601 1.06963161 1.27566955 1.74536825 1.13304019 2.94230501
 3.76742138 0.47485369], Cost: 0.0490
Current parameters: [2.50344396 0.98591191 2.0031138  0.85024955 1.9980702  1.35099084
 0.62959942 2.71445578 1.75411882 3.07394218 1.91651718 1.82256578
 1.5847892  1.08935765 1.16456847 1.73135159 1.16995694 2.95074502
 3.73972489 0.45819629], Cost: 0.2653
Current parameters: [2.53278715 1.0197615  2.02653385 0.84618499 2.0196934  1.38225255
 0.67334466 2.71921507 1.78318738 3.09295064 1.9943579  1.83839358
 1.59671886 1.06957491 1.2294866  1.74495687 1.16599676 2.95483204
 3.7699367  0.47176014], Cost: 0.0207
Current parameters: [2.55760132 1.04365633 1.90130316 0.84912516 2.0119297  1.39339157
 0.68415856 2.71556023 1.77042196 3.07874752 2.01973625 1.826152
 1.59021442 1.0785463  1.21599057 1.73767238 1.15663309 2.92554391
 3.76103218 0.45940246], Cost: 0.0545
Current parameters: [2.54373701 0.98449427 1.98070918 0.84445317 2.01620249 1.38210921
 0.64886045 2.72522793 1.78000081 3.14766328 1.97458334 1.8431201
 1.59655882 1.06966231 1.22624703 1.74479517 1.1650362  2.9395833
 3.76605969 0.47645624], Cost: 0.0221
Current parameters: [2.49700333 1.00964887 1.96824717 0.86823738 2.05058904 1.39961937
 0.64730833 2.65272763 1.82381339 3.13130114 1.98317981 1.80047545
 1.6066212  1.07824055 1.25071275 1.75425684 1.1905218  2.99678287
 3.77079499 0.44926391], Cost: 0.1895
Current parameters: [2.51400907 1.00543175 1.97891313 0.89365666 1.99983961 1.37515117
 0.66636818 2.72686642 1.78970998 3.10454674 1.96987286 1.8399669
 1.59993559 1.03403798 1.2319826  1.74820707 1.17171292 2.94882045
 3.76541572 0.47657242], Cost: 0.0348
Current parameters: [2.5151927  1.01058689 1.97352968 0.78140047 2.05423355 1.37682988
 0.668492   2.7309857  1.78627723 3.1034406  1.96700525 1.83269765
 1.59809792 0.97051982 1.22460336 1.74584877 1.16999137 2.95403207
 3.75439555 0.47198209], Cost: 0.1829
Current parameters: [2.52005076 1.00334961 1.97877017 0.84843114 2.02389099 1.37249425
 0.67153194 2.7266205  1.79131229 3.10372684 1.96589194 1.83882564
 1.64416328 1.069855   1.23315739 1.7052305  1.17260778 2.95047906
 3.76689299 0.47579536], Cost: 0.0039
Current parameters: [2.58732084 0.98171336 1.97568915 0.85694087 2.03720739 1.3435227
 0.7293362  2.72504118 1.80811107 3.09433478 1.92098657 1.82415926
 1.58970429 1.07397922 1.24412201 1.7667825  1.18204404 2.97030538
 3.77174175 0.46590915], Cost: 0.0321
Current parameters: [2.51280925 0.99938511 1.98317992 0.85342582 2.02366523 1.39748023
 0.65210441 2.81236668 1.84705886 3.10319946 1.97884555 1.80728095
```

```
 1.58715661 1.08179885 1.22995381 1.73474395 1.15322945 2.95009931
 3.78805787 0.43454515], Cost: 0.0765
Current parameters: [2.49495845 0.96145578 1.97826583 0.84641742 2.0200218  1.37542709
 0.69480807 2.72440295 1.78476882 3.10068785 1.99617751 1.83861494
 1.59788696 1.07156701 1.22789418 1.74613034 1.16842357 2.94676454
 3.76413227 0.47451879], Cost: 0.0126
Current parameters: [2.51438593 1.0054743  1.97424254 0.84641075 2.02014849 1.36073083
 0.6607625  2.73295574 1.77950261 3.10544461 1.97748222 1.83983993
 1.60027714 1.06937355 1.23243826 1.74855218 1.17236123 2.96743126
 3.77886899 0.47678137], Cost: 0.0088
Current parameters: [2.51547824 1.01056393 1.98266148 0.85141341 2.03012141 1.33025828
 0.6588363  2.72617188 1.81020111 3.1102622  1.99087058 1.83759996
 1.60178956 1.07161271 1.23338014 1.75007495 1.17570774 2.93020506
 3.74508028 0.47707121], Cost: 0.0576
Current parameters: [2.51409352 1.00572106 1.97760713 0.83108858 2.0305393  1.37623071
 0.66757168 2.7286662  1.789337   3.10410258 1.96938569 1.83776396
 1.59925298 1.04496445 1.23004764 1.74747355 1.17101603 2.95010192
 3.76348337 0.47491854], Cost: 0.0184
Current parameters: [2.51321452 1.0024526  1.98042449 0.8468053  2.02002388 1.37192776
 0.66168702 2.72372332 1.78576853 3.10016227 1.9653408  1.83267331
 1.59866263 1.07024128 1.22626363 1.746873   1.17180251 2.9458328
 3.76652011 0.47123124], Cost: 0.0130
Current parameters: [2.51135985 1.0067078  1.97940024 0.84633883 2.02096414 1.37907989
 0.66923767 2.73396828 1.78429742 3.1013123  1.96089429 1.85643907
 1.5970382  1.07739794 1.22054715 1.74475023 1.16940429 2.95677691
 3.75489267 0.48602408], Cost: 0.0040
Current parameters: [2.51101268 1.01017659 1.98054402 0.84635519 2.01964029 1.37249539
 0.67313246 2.73193213 1.78326714 3.10966826 1.96774849 1.83901429
 1.59815517 1.06989214 1.23418879 1.74638751 1.17346048 2.94355227
 3.76712374 0.47444697], Cost: 0.0049
Current parameters: [2.50388014 1.01157079 1.97724312 0.84998695 2.02870616 1.38410594
 0.67045717 2.7137021  1.79158389 3.11684213 1.96764045 1.83588245
 1.59853728 1.07620555 1.23455631 1.74633996 1.17783127 2.96328024
 3.76273892 0.47097018], Cost: 0.0166
Current parameters: [2.51394889 1.00616891 1.97851129 0.84680982 2.02313449 1.37217818
 0.66797355 2.71640897 1.78496294 3.10432608 1.96879259 1.84448101
 1.60163147 1.06817892 1.23132025 1.74999782 1.17427344 2.94550018
 3.76986464 0.48273713], Cost: 0.0038
Current parameters: [2.50984509 1.00622515 1.97921109 0.84729145 2.02628909 1.38191096
 0.66776131 2.73388921 1.8023602  3.10449835 1.9670873  1.84569237
 1.59819843 1.07429673 1.22507455 1.74616039 1.17157406 2.94304272
```



```
 3.78910402 0.480327  ], Cost: 0.0089
Current parameters: [2.51775664 1.0082402  1.97192989 0.84760572 2.02202353 1.37589421
 0.66735367 2.72408984 1.78934721 3.10151877 1.97388006 1.84510604
 1.59440821 1.06999426 1.23144849 1.7425944  1.17204399 2.94711685
 3.76650275 0.46975405], Cost: 0.0020
Current parameters: [2.52401743 1.01725998 1.95783815 0.84724859 2.02052836 1.38504832
 0.67322425 2.72668931 1.78398753 3.09794621 1.98208844 1.83365312
 1.60141122 1.07556875 1.22574941 1.74927668 1.16587384 2.94424216
 3.76533933 0.4789777 ], Cost: 0.0009
Current parameters: [2.53274141 1.01395803 1.95723595 0.84814691 2.02171783 1.38429738
 0.68130305 2.72650652 1.78477577 3.09630986 1.97484962 1.83192446
 1.60006598 1.07592    1.22721556 1.75139112 1.16683191 2.94803841
 3.76741564 0.47763637], Cost: 0.0015
Current parameters: [2.52808077 1.0223469  1.95585851 0.84923367 2.0276733  1.36505456
 0.67743805 2.72827002 1.7981473  3.10092676 1.9883586  1.8347958
 1.601366   1.08209589 1.22134159 1.75100168 1.16789135 2.93885991
 3.75585989 0.4831428 ], Cost: 0.0022
Current parameters: [2.52196614 1.01850123 1.9576933  0.84713281 2.02029499 1.38238488
 0.67461528 2.72359548 1.78495577 3.0992935  1.97974324 1.83407786
 1.60066029 1.0755466  1.2276747  1.74851793 1.15156874 2.94481931
 3.76632185 0.47827874], Cost: 0.0056
Current parameters: [2.52647465 1.01488872 1.95398749 0.84512873 2.02294184 1.38733323
 0.66776635 2.73177784 1.78272035 3.08816815 1.98282012 1.83823586
 1.60045624 1.08480692 1.24925831 1.74841536 1.16973135 2.9386652
 3.76196617 0.48691953], Cost: 0.0109
Current parameters: [2.51762807 1.02176002 1.95745213 0.85381108 2.01900219 1.38355285
 0.67698715 2.72704147 1.7860743  3.08867927 1.98107603 1.83297541
 1.60213699 1.07101938 1.22698214 1.75001    1.16730885 2.94622748
 3.76527097 0.47900267], Cost: 0.0034
Current parameters: [2.53333062 1.01320673 1.95452942 0.86737419 2.01110204 1.3886332
 0.66949346 2.72953734 1.78439046 3.11007729 1.98465666 1.83822092
 1.59995852 1.06564541 1.22430781 1.7478883  1.16669359 2.94072089
 3.76298582 0.48495166], Cost: 0.0007
Current parameters: [2.53656176 1.01575522 1.96248474 0.86727106 2.01089415 1.38978294
 0.67084543 2.72784702 1.78429015 3.10815787 1.98882898 1.83394976
 1.59361635 1.06563234 1.22418554 1.74148013 1.16614871 2.94256302
 3.76356565 0.49071212], Cost: 0.0013
Current parameters: [2.54273076 1.01904462 1.9704024  0.86939556 2.0097841  1.3931492
 0.67200386 2.72992473 1.78183988 3.10748661 1.99539499 1.8488867
 1.6099875  1.06834312 1.22258515 1.75808526 1.16543964 2.94021281
 3.76292569 0.4744363 ], Cost: 0.0083
```



```
Current parameters: [2.5308799  1.00724817 1.95482931 0.87260981 2.02163823 1.38899575
 0.67228429 2.73108727 1.77878992 3.10926431 1.98805931 1.83806797
 1.59904892 1.06587783 1.22292326 1.74696615 1.16503987 2.93894609
 3.76300495 0.48415638], Cost: 0.0011
Current parameters: [2.52627439 0.99494486 1.95296821 0.86171222 1.99720541 1.38917493
 0.68291653 2.72736325 1.78893001 3.10957651 1.99678725 1.83927902
 1.5999832  1.06771108 1.22345473 1.74793108 1.16747281 2.94110135
 3.76106112 0.48632376], Cost: 0.0052
Current parameters: [2.53182092 1.01372913 1.95455623 0.86707134 2.01084602 1.38930349
 0.66821018 2.72961109 1.78398675 3.11027938 1.98564912 1.83849169
 1.58889814 1.06543363 1.22400142 1.75862665 1.16646535 2.94031601
 3.76280011 0.48513503], Cost: 0.0015
Current parameters: [2.53712197 1.01450578 1.95042518 0.85277491 2.01961601 1.39212439
 0.67134923 2.73492443 1.78659638 3.11205517 1.98671038 1.84097056
 1.60046534 1.04225285 1.22190189 1.74686201 1.16831643 2.9408207
 3.75797207 0.48822701], Cost: 0.0052
Current parameters: [2.53083387 1.0130079  1.9558404  0.86940203 2.01496712 1.39422592
 0.66908155 2.72741114 1.79053952 3.11498252 1.98578788 1.83382935
 1.60231665 1.06650465 1.22850963 1.75022367 1.16719523 2.94766458
 3.7584573  0.48439604], Cost: 0.0039
Current parameters: [2.53028595 1.02354338 1.94442186 0.86731279 2.01191415 1.39032229
 0.67555828 2.71917855 1.77549531 3.12023184 1.98287601 1.84462805
 1.5936531  1.07172943 1.21702817 1.73939602 1.17921456 2.94187056
 3.76697164 0.48206187], Cost: 0.0021
Current parameters: [2.53120355 1.01278945 1.95515527 0.86528835 2.01340514 1.39425592
 0.66726954 2.73385308 1.79043474 3.11136925 1.98575378 1.83857194
 1.60076964 1.06857443 1.21977089 1.7478708  1.16797995 2.93802377
 3.77256743 0.48698195], Cost: 0.0017
Current parameters: [2.53735433 1.01493052 1.95111502 0.88126188 2.01182887 1.38091925
 0.68142516 2.73045297 1.79620349 3.1067341  1.97348546 1.8471744
 1.5945598  1.0610415  1.23069553 1.74506174 1.16158761 2.94072699
 3.77091611 0.48737181], Cost: 0.0039
Current parameters: [2.53591178 1.00976844 1.95374093 0.86983225 2.00948211 1.38685713
 0.66457191 2.72788079 1.78472238 3.10667649 1.97864916 1.83175022
 1.59875263 1.06573313 1.21687119 1.74597495 1.16864183 2.9367846
 3.76033215 0.48181247], Cost: 0.0070
Current parameters: [2.53402454 1.01351239 1.95390016 0.86750487 2.01122713 1.38705786
 0.66938852 2.72734553 1.78464531 3.11100864 1.98701453 1.83490619
 1.60024673 1.06371528 1.22713562 1.74830867 1.16760193 2.93840377
 3.76629955 0.48248212], Cost: 0.0024
Current parameters: [2.53437182 1.01227967 1.95198658 0.86790286 2.01025109 1.38222483
```



```
 0.6656519  2.73426238 1.78114081 3.1103604  1.9873835  1.83833591
 1.60048021 1.06589322 1.22305218 1.74817141 1.16705877 2.95140035
 3.76747475 0.4864222 ], Cost: 0.0005
Current parameters: [2.53333062 1.01320673 1.95452942 0.86737419 2.01110204 1.3886332
 0.66949346 2.72953734 1.78439046 3.11007729 1.98465666 1.83822092
 1.59995852 1.06564541 1.22430781 1.7478883  1.16669359 2.94072089
 3.76298582 0.48495166], Cost: 0.0007

   Return from subroutine COBYLA because the MAXFUN limit has been reached.

   NFVALS =  100   F = 7.149989E-04    MAXCV = 0.000000E+00
   X = 2.533331E+00   1.013207E+00   1.954529E+00   8.673742E-01   2.011102E+00
       1.388633E+00   6.694935E-01   2.729537E+00   1.784390E+00   3.110077E+00
       1.984657E+00   1.838221E+00   1.599959E+00   1.065645E+00   1.224308E+00
       1.747888E+00   1.166694E+00   2.940721E+00   3.762986E+00   4.849517E-01
Simulating for QAOA depth p = 11
Current parameters: [3.77339736 2.71365013 1.73718062 0.07824775 0.60023537 0.1821141
 2.79221764 0.60586136 2.7428033  1.71546547 2.29646814 2.04217967
 0.97949814 0.62672254 3.05926726 1.86725427 2.04581712 3.09759502
 0.97127495 2.28334467 0.08123238 2.63117383], Cost: 2.5007
Current parameters: [2.77339736 3.71365013 1.73718062 0.07824775 0.60023537 0.1821141
 2.79221764 0.60586136 2.7428033  1.71546547 2.29646814 2.04217967
 0.97949814 0.62672254 3.05926726 1.86725427 2.04581712 3.09759502
 0.97127495 2.28334467 0.08123238 2.63117383], Cost: 2.6394
Current parameters: [2.77339736 2.71365013 2.73718062 0.07824775 0.60023537 0.1821141
 2.79221764 0.60586136 2.7428033  1.71546547 2.29646814 2.04217967
 0.97949814 0.62672254 3.05926726 1.86725427 2.04581712 3.09759502
 0.97127495 2.28334467 0.08123238 2.63117383], Cost: 0.9582
Current parameters: [2.77339736 2.71365013 1.73718062 1.07824775 0.60023537 0.1821141
 2.79221764 0.60586136 2.7428033  1.71546547 2.29646814 2.04217967
 0.97949814 0.62672254 3.05926726 1.86725427 2.04581712 3.09759502
 0.97127495 2.28334467 0.08123238 2.63117383], Cost: 1.9725
Current parameters: [2.77339736 2.71365013 1.73718062 0.07824775 1.60023537 0.1821141
 2.79221764 0.60586136 2.7428033  1.71546547 2.29646814 2.04217967
 0.97949814 0.62672254 3.05926726 1.86725427 2.04581712 3.09759502
 0.97127495 2.28334467 0.08123238 2.63117383], Cost: 1.4081
Current parameters: [2.77339736 2.71365013 1.73718062 0.07824775 0.60023537 1.1821141
 2.79221764 0.60586136 2.7428033  1.71546547 2.29646814 2.04217967
 0.97949814 0.62672254 3.05926726 1.86725427 2.04581712 3.09759502
 0.97127495 2.28334467 0.08123238 2.63117383], Cost: 2.3240
Current parameters: [2.77339736 2.71365013 2.73718062 0.07824775 0.60023537 0.1821141
```

```
 3.79221764 0.60586136 2.7428033  1.71546547 2.29646814 2.04217967
 0.97949814 0.62672254 3.05926726 1.86725427 2.04581712 3.09759502
 0.97127495 2.28334467 0.08123238 2.63117383], Cost: 2.1574
Current parameters: [2.77339736 2.71365013 2.73718062 0.07824775 0.60023537 0.1821141
 2.79221764 1.60586136 2.7428033  1.71546547 2.29646814 2.04217967
 0.97949814 0.62672254 3.05926726 1.86725427 2.04581712 3.09759502
 0.97127495 2.28334467 0.08123238 2.63117383], Cost: 1.9274
Current parameters: [2.77339736 2.71365013 2.73718062 0.07824775 0.60023537 0.1821141
 2.79221764 0.60586136 3.7428033  1.71546547 2.29646814 2.04217967
 0.97949814 0.62672254 3.05926726 1.86725427 2.04581712 3.09759502
 0.97127495 2.28334467 0.08123238 2.63117383], Cost: 2.1860
Current parameters: [2.77339736 2.71365013 2.73718062 0.07824775 0.60023537 0.1821141
 2.79221764 0.60586136 2.7428033  2.71546547 2.29646814 2.04217967
 0.97949814 0.62672254 3.05926726 1.86725427 2.04581712 3.09759502
 0.97127495 2.28334467 0.08123238 2.63117383], Cost: 2.7884
Current parameters: [2.77339736 2.71365013 2.73718062 0.07824775 0.60023537 0.1821141
 2.79221764 0.60586136 2.7428033  1.71546547 3.29646814 2.04217967
 0.97949814 0.62672254 3.05926726 1.86725427 2.04581712 3.09759502
 0.97127495 2.28334467 0.08123238 2.63117383], Cost: 3.0844
Current parameters: [2.77339736 2.71365013 2.73718062 0.07824775 0.60023537 0.1821141
 2.79221764 0.60586136 2.7428033  1.71546547 2.29646814 3.04217967
 0.97949814 0.62672254 3.05926726 1.86725427 2.04581712 3.09759502
 0.97127495 2.28334467 0.08123238 2.63117383], Cost: 0.9580
Current parameters: [2.77339736 2.71365013 2.73718062 0.07824775 0.60023537 0.1821141
 2.79221764 0.60586136 2.7428033  1.71546547 2.29646814 3.04217967
 1.97949814 0.62672254 3.05926726 1.86725427 2.04581712 3.09759502
 0.97127495 2.28334467 0.08123238 2.63117383], Cost: 1.3568
Current parameters: [2.77339736 2.71365013 2.73718062 0.07824775 0.60023537 0.1821141
 2.79221764 0.60586136 2.7428033  1.71546547 2.29646814 3.04217967
 0.97949814 1.62672254 3.05926726 1.86725427 2.04581712 3.09759502
 0.97127495 2.28334467 0.08123238 2.63117383], Cost: 0.7914
Current parameters: [2.77339736 2.71365013 2.73718062 0.07824775 0.60023537 0.1821141
 2.79221764 0.60586136 2.7428033  1.71546547 2.29646814 3.04217967
 0.97949814 1.62672254 4.05926726 1.86725427 2.04581712 3.09759502
 0.97127495 2.28334467 0.08123238 2.63117383], Cost: 0.8023
Current parameters: [2.77339736 2.71365013 2.73718062 0.07824775 0.60023537 0.1821141
 2.79221764 0.60586136 2.7428033  1.71546547 2.29646814 3.04217967
 0.97949814 1.62672254 3.05926726 2.86725427 2.04581712 3.09759502
 0.97127495 2.28334467 0.08123238 2.63117383], Cost: 2.3277
Current parameters: [2.77339736 2.71365013 2.73718062 0.07824775 0.60023537 0.1821141
 2.79221764 0.60586136 2.7428033  1.71546547 2.29646814 3.04217967
```



```
 0.97949814 1.62672254 3.05926726 1.86725427 3.04581712 3.09759502
 0.97127495 2.28334467 0.08123238 2.63117383], Cost: 2.1924
Current parameters: [2.77339736 2.71365013 2.73718062 0.07824775 0.60023537 0.1821141
 2.79221764 0.60586136 2.7428033  1.71546547 2.29646814 3.04217967
 0.97949814 1.62672254 3.05926726 1.86725427 2.04581712 4.09759502
 0.97127495 2.28334467 0.08123238 2.63117383], Cost: 2.6553
Current parameters: [2.77339736 2.71365013 2.73718062 0.07824775 0.60023537 0.1821141
 2.79221764 0.60586136 2.7428033  1.71546547 2.29646814 3.04217967
 0.97949814 1.62672254 3.05926726 1.86725427 2.04581712 3.09759502
 1.97127495 2.28334467 0.08123238 2.63117383], Cost: 1.5693
Current parameters: [2.77339736 2.71365013 2.73718062 0.07824775 0.60023537 0.1821141
 2.79221764 0.60586136 2.7428033  1.71546547 2.29646814 3.04217967
 0.97949814 1.62672254 3.05926726 1.86725427 2.04581712 3.09759502
 0.97127495 3.28334467 0.08123238 2.63117383], Cost: 1.3521
Current parameters: [2.77339736 2.71365013 2.73718062 0.07824775 0.60023537 0.1821141
 2.79221764 0.60586136 2.7428033  1.71546547 2.29646814 3.04217967
 0.97949814 1.62672254 3.05926726 1.86725427 2.04581712 3.09759502
 0.97127495 2.28334467 1.08123238 2.63117383], Cost: 2.9814
Current parameters: [2.77339736 2.71365013 2.73718062 0.07824775 0.60023537 0.1821141
 2.79221764 0.60586136 2.7428033  1.71546547 2.29646814 3.04217967
 0.97949814 1.62672254 3.05926726 1.86725427 2.04581712 3.09759502
 0.97127495 2.28334467 0.08123238 3.63117383], Cost: 2.2097
Current parameters: [ 2.63086392  2.54685087  2.86453511 -0.09921666  0.52153208 -0.05685578
  2.58240276  0.43629523  2.52797769  1.39523649  1.92446395  3.04221729
  0.90973211  1.65588017  3.05735063  1.59844453  1.80068288  2.77147015
  0.83516107  2.18524304 -0.30195116  2.38301796], Cost: 1.3805
Current parameters: [2.751569   2.68810557 2.75668439 0.05106987 0.58818231 0.14551692
 2.76008542 0.57989306 2.70990371 2.19395426 2.23949736 3.04218543
 0.96881378 1.6311879  3.05897373 1.82608725 2.00827591 3.04765045
 0.95042971 2.26832084 0.02254953 2.59316987], Cost: 1.0912
Current parameters: [2.96345754 2.55123976 2.73718062 0.07824775 0.60023537 0.1821141
 2.79221764 0.60586136 2.7428033  1.71546547 2.29646814 3.04217967
 0.97949814 1.62672254 3.05926726 1.86725427 2.04581712 3.09759502
 0.97127495 2.28334467 0.08123238 2.63117383], Cost: 0.7509
Current parameters: [3.28383429 2.92135761 2.7512142  0.05869241 0.59156281 0.15578128
 2.7690975  0.58717635 2.71913101 1.70780435 2.25547587 3.04218382
 0.97181041 1.6299355  3.05905606 1.8376333  2.01880503 3.06165833
 0.95627614 2.27253455 0.03900822 2.60382878], Cost: 0.7060
Current parameters: [ 3.28383429  2.92135761  2.7512142   0.05869241  0.59156281 -0.05455851
  2.7690975   0.58717635  2.71913101  1.70780435  2.39059497  3.04218382
  0.97181041  1.6299355   3.05905606  1.8376333   2.01880503  3.06165833
```



```
  0.95627614  2.27253455  0.03900822  2.60382878], Cost: 1.0141
Current parameters: [ 3.29627697  2.92465463  2.78413303  0.01282107  0.57121944  0.44602158
  2.71486415  0.54334657  2.66360248  1.68983352  2.60417084  3.04219354
  0.95377715  1.63747223  3.05856065  1.76815085  1.95544227  2.97736095
  0.92109317  2.24717706 -0.06003779  2.53968498], Cost: 2.2055
Current parameters: [3.28383429 2.92135761 2.7512142  0.05869241 0.59156281 0.15578128
 2.5902473  0.58717635 2.89380959 1.70780435 2.25547587 3.04218382
 0.97181041 1.6299355  3.05905606 1.8376333  2.01880503 3.06165833
 0.95627614 2.27253455 0.03900822 2.60382878], Cost: 0.9021
Current parameters: [ 3.29173741  2.92345175  2.772123    0.02955666  0.57864147  0.14686824
  3.11864584  0.55933732  3.04477852  1.69638995  2.17610183  3.04218999
  0.96035636  1.63472255  3.05874139  1.79350066  1.97855939  3.0081158
  0.93392924  2.25642843 -0.02390208  2.56308705], Cost: 2.0239
Current parameters: [3.28383429 2.92135761 2.7512142  0.23140101 0.59156281 0.15578128
 2.7690975  0.40642309 2.71913101 1.70780435 2.25547587 3.04218382
 0.97181041 1.6299355  3.05905606 1.8376333  2.01880503 3.06165833
 0.95627614 2.27253455 0.03900822 2.60382878], Cost: 0.7995
Current parameters: [ 3.2899265   2.9229719   2.76733198  0.40330834  0.58160225  0.14891056
  2.74407437  0.92790253  2.66865108  1.69900544  2.19428951  3.04218858
  0.96298093  1.63362565  3.05881349  1.80361316  1.98778123  3.02038448
  0.93904979  2.26011897 -0.00948689  2.57242256], Cost: 1.3305
Current parameters: [3.28383429 2.92135761 2.61978881 0.05869241 0.37889554 0.15578128
 2.7690975  0.58717635 2.71913101 1.70780435 2.25547587 3.04218382
 0.97181041 1.6299355  3.05905606 1.8376333  2.01880503 3.06165833
 0.95627614 2.27253455 0.03900822 2.60382878], Cost: 1.0732
Current parameters: [3.28750411 2.92233003 2.34134887 0.04343109 0.86788324 0.1516425
 2.75402408 0.57949019 2.68872295 1.70250407 2.21861848 3.04218668
 0.96649171 1.63215837 3.05890994 1.81714028 2.00011694 3.03679582
 0.94589934 2.26505566 0.00979578 2.58491033], Cost: 0.9584
Current parameters: [3.28383429 2.92135761 2.7512142  0.05869241 0.59156281 0.15578128
 2.7690975  0.58717635 2.71913101 1.70780435 2.25547587 3.29218378
 0.9719452  1.6299355  3.05905606 1.8376333  2.01880503 3.06165833
 0.95627614 2.27253455 0.03900822 2.60382878], Cost: 0.7595
Current parameters: [3.28554368 2.92181056 2.75896702 0.05158374 0.59749871 0.15385345
 2.76207635 0.58359616 2.70496704 1.7053355  2.23830782 3.04058636
 1.47048943 1.63097091 3.058988   1.82808772 2.01010018 3.05007746
 0.95144267 2.26905091 0.02540116 2.59501663], Cost: 0.9605
Current parameters: [3.28383429 2.92135761 2.7512142  0.05869241 0.59156281 0.15578128
 2.7690975  0.58717635 2.71913101 1.70780435 2.25547587 3.04218382
 0.97181041 1.6299355  3.05905606 1.8376333  2.01880503 2.87127623
 0.95627614 2.27253455 0.20104111 2.60382878], Cost: 0.7714
```



```
Current parameters: [3.29790572e+00 2.92508621e+00 2.81503419e+00 1.74912763e-04
 6.40426159e-01 1.39911700e-01 2.71130052e+00 5.57704810e-01
 2.60253540e+00 1.68748113e+00 2.11415117e+00 3.03126189e+00
 9.44474724e-01 1.63845879e+00 3.05849580e+00 1.75905556e+00
 1.94714805e+00 3.34785631e+00 9.16487703e-01 2.24385775e+00
 3.54660729e-01 2.53128852e+00], Cost: 0.7176
Current parameters: [3.28383429 2.92135761 2.7512142  0.05869241 0.59156281 0.15578128
 2.7690975  0.58717635 2.71913101 1.70780435 2.25547587 3.04218382
 0.97181041 1.6299355  3.05905606 2.00721211 2.01880503 3.06165833
 0.95627614 2.27253455 0.03900822 2.42013603], Cost: 1.3075
Current parameters: [3.29632844 2.92466826 2.80788053 0.00673421 0.634949   0.14169054
 2.71777906 0.56100831 2.61560475 1.68975919 2.12999244 3.03248614
 0.94753882 1.6375034  3.0585586  2.08620355 1.95518018 3.06408188
 0.92094764 2.24707217 0.0235484  2.98200369], Cost: 0.8123
Current parameters: [3.28383429 2.92135761 2.7512142  0.05869241 0.59156281 0.15578128
 2.7690975  0.58717635 2.71913101 1.70780435 2.25547587 3.04218382
 0.97181041 1.6299355  3.05905606 1.8376333  2.14016683 3.06165833
 0.73770965 2.27253455 0.03900822 2.60382878], Cost: 0.9181
Current parameters: [3.29429034 2.92412822 2.79863687 0.01520986 0.62787166 0.14398908
 2.72615034 0.56527696 2.6324924  1.69270279 2.15046184 3.03406807
 0.9514981  1.63626889 3.05863974 1.75228312 2.40536125 3.06368654
 1.20779298 2.2512257  0.02607028 2.64948272], Cost: 1.0378
Current parameters: [ 3.26048637  2.8940348   2.7720757   0.02962257  0.5786707   0.11663644
  2.73472844  0.5594003   2.68394116  1.65534874  2.19453914  3.04218998
  0.96038227  1.6299355   3.05905606  1.7936005   1.97865044  3.00823693
  0.9339798   2.08293352 -0.02375976  2.56317922], Cost: 1.4229
Current parameters: [ 3.24860311  2.87554546  2.81017779 -0.01252938  0.5868935   0.08251578
  2.68898956  0.52863741  2.61645223  1.60996486  2.09946629  3.03826501
  0.94225306  1.63300197  3.05885449  1.72031721  1.92328572  2.9704459
  0.9210926   2.63259045 -0.07558075  2.55578044], Cost: 1.7710
Current parameters: [3.28091875 2.91794572 2.75381925 0.05506236 0.58995293 0.15089312
 2.76480571 0.58370786 2.71473673 1.77171438 2.24786648 3.04218458
 0.97038333 1.87085118 3.05905606 1.83213477 2.01379079 3.05498741
 0.95349192 2.27052788 0.03117016 2.59875273], Cost: 1.4192
Current parameters: [3.27879792 2.9124261  2.77393149 0.03428927 0.59858133 0.13792703
 2.74296147 0.57016477 2.67867837 2.16736482 2.19987192 3.0395815
 0.9611841  1.4643955  3.05892245 1.79441572 1.98699255 3.04310794
 0.95044642 2.26763194 0.0122954  2.60386948], Cost: 1.1346
Current parameters: [3.28377464 2.92128782 2.75126827 0.05861706 0.5915294  0.15621029
 2.76900841 0.58710435 2.7190398  1.70777483 2.25614372 3.04218383
 0.97178078 1.62994788 2.80905757 1.83751917 2.01870095 3.06151986
```

```
 0.95621835 2.2724929  0.03884553 2.60372342], Cost: 0.3751
Current parameters: [3.32351817 2.95501893 2.79277209 0.03129231 0.65446217 0.4412457
 2.74812628 0.58752063 2.64005963 1.65338391 2.58286994 3.03122083
 0.95669415 1.51114482 2.74243997 1.76982324 1.98893244 3.12191958
 0.98947289 2.29351189 0.08912921 2.7092328 ], Cost: 0.2820
Current parameters: [ 3.34415679e+00  2.97917120e+00  2.82579226e+00 -1.56384484e-03
  6.82259843e-01  4.68224540e-01  2.92352175e+00  6.12841357e-01
  2.76095694e+00  1.62972135e+00  2.54899892e+00  2.99022480e+00
  9.39356170e-01  1.47960091e+00  2.78329773e+00  1.73615928e+00
  1.95866734e+00  3.15544525e+00  1.01957044e+00  2.30714729e+00
  6.52534653e-02  2.73396242e+00], Cost: 0.6695
Current parameters: [3.30729826 2.91274979 2.71465679 0.14050446 0.66177517 0.3704892
 2.69283183 0.50208369 2.724884   1.66186243 2.57807437 3.18336749
 1.01027481 1.42362113 2.44604563 1.81969228 2.0908409  3.12992224
 0.93985517 2.28620564 0.32647776 2.82712491], Cost: 1.3787
Current parameters: [ 3.30927793  2.93441534  2.82495469 -0.00516095  0.65816979  0.43095573
  2.70239161  0.556502    2.57999361  1.59512174  2.536628    3.03100252
  0.94136442  1.47351697  2.71836664  1.70525848  1.93987406  3.08180067
  0.97396939  2.47797128  0.04048369  2.69712168], Cost: 0.7996
Current parameters: [3.41607829 2.87658953 2.79471959 0.05125512 0.6551001  0.44108441
 2.7472754  0.60876157 2.63694648 1.65202448 2.57972053 3.02965491
 0.95565598 1.51007504 2.74399191 1.76708912 1.9864557  3.12144346
 0.98988941 2.2940102  0.08615288 2.70884068], Cost: 0.2862
Current parameters: [3.2868416  3.0040143  2.8071559  0.1915517  0.66660163 0.44526673
 2.7324378  0.75719986 2.60369057 1.64046666 2.55839655 3.0201219
 0.94980291 1.49133482 2.74509337 1.75030123 1.97460406 3.13129578
 0.99814065 2.29970165 0.08261975 2.72245425], Cost: 0.6265
Current parameters: [3.31805701 2.95031532 2.78873961 0.03881266 0.64994664 0.44185481
 2.75218658 0.58646148 2.65274842 1.71311067 2.59484748 3.0371808
 0.95879404 1.49943187 2.73655311 1.77309652 1.89912274 3.11507973
 0.93278652 2.28971137 0.09029002 2.70414172], Cost: 1.0068
Current parameters: [3.33291213e+00 2.96041928e+00 2.81811423e+00 1.43283415e-03
 6.69072898e-01 4.48844750e-01 2.73566079e+00 5.85670228e-01
 2.59622045e+00 1.82887552e+00 2.54963395e+00 3.01288150e+00
 9.44004125e-01 1.42870522e+00 2.75160660e+00 1.73363510e+00
 2.09119815e+00 3.12539301e+00 1.07152455e+00 2.29709625e+00
 6.44720850e-02 2.71839979e+00], Cost: 0.1091
Current parameters: [ 3.3411835   2.9695874   2.82367531 -0.0036011   0.67227058  0.45180306
  2.73731442  0.58866606  2.58983639  1.82780167  2.54379717  3.00717161
  1.06687321  1.42360856  2.75722772  1.72767004  2.08660359  3.12839142
  1.07542865  2.30027133  0.05928753  2.72057388], Cost: 0.1318
```



```
Current parameters: [ 3.49180559  3.14189902  2.81575812 -0.0046501   0.66071862  0.4279056
  2.70884208  0.56306125  2.5841031   1.81640149  2.53399573  3.0255927
  0.92189442  1.43669444  2.73345852  1.72799023  2.09586224  3.10380629
  1.06210675  2.28468032  0.05844821  2.70625704], Cost: 0.2932
Current parameters: [ 3.33422981  2.96134558  2.71712077 -0.00414639  0.73860327  0.45166486
  2.73675011  0.58871389  2.58866998  1.82761649  2.54282108  3.00682689
  0.94343634  1.42328199  2.75763441  1.72682194  2.08589177  3.1210817
  1.07550383  2.30035184  0.05006434  2.72035068], Cost: 0.2130
Current parameters: [ 3.32382812  2.95388854  2.81572135  0.03427559  0.64844543  0.46634869
  2.75447241  0.58465239  2.64652731  1.82930513  2.61352065  3.03697464
  0.95619302  1.44038715  2.72130001  1.78986264  2.1531088   2.97257592
  1.08139765  2.29681374 -0.07820736  2.75009642], Cost: 0.2414
Current parameters: [3.32887206 2.95847991 2.80689246 0.01903097 0.66090667 0.45153742
 2.74399271 0.58471774 2.62313662 1.83479399 2.57759366 3.02572459
 0.94952388 1.43921933 2.73888327 1.66098392 2.11146959 3.11504451
 1.06808698 2.29671602 0.07213315 2.63384766], Cost: 0.1226
Current parameters: [ 3.31039457  2.92700533  2.85895698 -0.04457206  0.68031705  0.45150266
  2.7042512   0.56226569  2.53411069  1.73657904  2.50280743  3.00039017
  0.93388164  1.39057371  2.74142711  1.66650308  2.11575335  3.10777072
  1.11017521  2.12263291  0.02340371  2.71751183], Cost: 0.6985
Current parameters: [3.33199421e+00 2.95934509e+00 2.81773398e+00 1.86745507e-03
 6.66623558e-01 4.45389385e-01 2.73267597e+00 5.82927224e-01
 2.59643246e+00 1.84898454e+00 2.54847993e+00 3.01516522e+00
 9.43556057e-01 1.50450947e+00 2.74932314e+00 1.66615461e+00
 2.09072798e+00 3.12155381e+00 1.06865160e+00 2.29646447e+00
 6.34843479e-02 2.78802326e+00], Cost: 0.1762
Current parameters: [ 3.33444773  2.95291954  2.84698426 -0.02483673  0.70456292  0.50598356
  2.74200276  0.5946913   2.55629678  1.8350964   2.56290734  2.99123422
  0.9452048   1.57565224  2.74422802  1.83172468  2.17010363  3.1757788
  1.15381618  2.33496934  0.0888909   2.64659303], Cost: 1.6946
Current parameters: [3.33291141 2.96041845 2.77667853 0.00349516 0.60202187 0.53024338
 2.73352313 0.58351003 2.59830613 1.82887516 2.49735597 3.01586808
 0.94400538 1.42870536 2.74862004 1.73363373 2.09264674 3.123117
 1.06891279 2.29709575 0.06640584 2.71839854], Cost: 0.1272
Current parameters: [ 3.33451092  2.95909119  2.72745803 -0.02680262  0.5206525   0.34392086
  2.76067771  0.61153296  2.56333298  1.80771172  2.63804151  2.97571633
  0.94492523  1.39140722  2.77878336  1.72179598  2.10576548  3.1678099
  1.12684344  2.31086536  0.05649276  2.74712824], Cost: 0.4364
Current parameters: [3.33291177e+00 2.96041886e+00 2.81811456e+00 2.47471188e-03
 6.69072696e-01 4.48847339e-01 2.80099685e+00 5.84578909e-01
 2.53240726e+00 1.82887534e+00 2.54963798e+00 3.01439031e+00
```



```
 9.44004760e-01 1.42870529e+00 2.75009781e+00 1.73363441e+00
 2.13260299e+00 3.12424318e+00 9.96955042e-01 2.29709600e+00
 6.54490054e-02 2.71839916e+00], Cost: 0.0755
Current parameters: [ 3.33406063  2.95948047  2.82151817 -0.04074332  0.67336607  0.45679875
  2.69535391  0.62719849  2.62957715  1.8137769   2.55939385  2.95446139
  0.944648    1.40208796  2.80289884  1.72520132  2.23478486  3.17997133
  0.87115989  2.30692714  0.03812394  2.73891372], Cost: 0.0693
Current parameters: [ 3.33405927  2.95947888  2.82151939  0.01614448  0.67336531  0.45680848
  2.69535189  0.56765747  2.62957508  1.81377623  2.55940901  2.96013573
  0.94465039  1.40208825  2.79722454  1.72519873  2.2347825   3.25132161
  0.87115858  2.30692619 -0.02260816  2.73891133], Cost: 0.1032
Current parameters: [3.33499388 2.95874932 2.8242224  0.06093946 0.67680393 0.4630368
 2.6898783  0.5187332  2.63017253 1.80172562 2.56701038 2.8409281
 0.94513414 1.38082561 2.91073874 1.71849585 2.25711754 3.09761867
 0.87751164 2.31479015 0.12434498 2.75532626], Cost: 0.3058
Current parameters: [ 3.33555136  2.96293139  2.82785949 -0.04944457  0.67304081  0.46939799
  2.69070599  0.6334878   2.61373342  1.81274929  2.57035551  3.02043625
  0.94043474  1.40816212  2.90279716  1.7206865   2.22653805  3.17925435
  0.87493893  2.30746683  0.01980178  2.73016635], Cost: 0.1036
Current parameters: [ 3.33106086  2.94545559  2.80560859 -0.00340114  0.68293056  0.42724608
  2.69848384  0.59314266  2.68729099  1.78797046  2.53898121  3.10843812
  0.96115685  1.32854752  2.75669501  1.72485685  2.31867065  3.18716768
  0.87328238  2.32424007  0.13868875  2.81010474], Cost: 0.4373
Current parameters: [ 3.34344343  2.96711599  2.83332425 -0.04919763  0.6892977   0.52742361
  2.68896723  0.62611292  2.60791035  1.79771473  2.63864844  2.95155618
  0.94018337  1.37082172  2.78548189  1.70552411  2.22520761  3.19346954
  0.87891363  2.31990128  0.04851447  2.76477065], Cost: 0.1946
Current parameters: [ 3.3196736   2.94055468  2.85935064 -0.07950815  0.67365312  0.42907653
  2.65813568  0.60176612  2.57913561  1.7474952   2.48697724  2.95009374
  0.92698288  1.35940831  2.7863356   1.65828426  2.21675797  3.13831709
  0.86906079  2.48517841 -0.0178123   2.72680246], Cost: 0.0780
Current parameters: [ 3.33761784  2.96439251  2.86526497 -0.05556712  0.6927708   0.45742902
  2.76003398  0.64011273  2.69043999  1.80988284  2.54354509  2.9347128
  0.91534726  1.36848861  2.82435774  1.7310955   2.21740545  3.2024969
  0.89911922  2.31628626  0.03418149  2.75269199], Cost: 0.2159
Current parameters: [ 3.36614558  2.99574478  2.72259437 -0.05793346  0.65111469  0.50517864
  2.81495007  0.63541813  2.64247403  1.75727095  2.5307461   2.94067972
  1.02517313  1.45585028  2.80735825  1.61332452  2.28944267  3.15738402
  0.84253966  2.29917577 -0.00878559  2.7419562 ], Cost: 0.1551
Current parameters: [3.29775568 2.99963863 2.82870683 0.03059618 0.6795049  0.45769827
 2.68732475 0.70323714 2.61287179 1.78535177 2.54754525 2.94911989
```



```
 0.9408866  1.3991802  2.80480858 1.71866017 2.24315852 3.1861878
 0.88419161 2.31055356 0.03702349 2.74601778], Cost: 0.1340
Current parameters: [3.45976798 2.86010611 2.81748904 0.02433641 0.66258595 0.45607568
 2.68901397 0.70485298 2.60015287 1.93264955 2.5291742  2.94894721
 0.95607973 1.34239702 2.79852343 1.6730932  2.2050171  3.15803492
 0.83190322 2.30204376 0.00527878 2.74100128], Cost: 0.4640
Current parameters: [ 3.27633034  2.99983536  2.81127408 -0.04853232  0.65802406  0.44892316
  2.69685932  0.61268292  2.64306464  1.83915598  2.5626867   2.96296517
  0.95418766  1.39184808  2.79355907  1.71873369  2.16941481  3.1636246
  0.80686243  2.30060298  0.03334833  2.73488823], Cost: 0.3137
Current parameters: [ 3.31687611  2.97899584  2.8219738  -0.05202284  0.67588408  0.46122211
  2.69500911  0.62222133  2.61832305  1.85293133  2.55264588  2.9505476
  0.94580411  1.38875085  2.80409892  1.71491053  2.26121794  3.18033211
  0.88879605  2.30790008  0.03238181  2.73988133], Cost: 0.1067
Current parameters: [ 3.3359596   2.94407732  2.83055879 -0.05680507  0.68125135  0.47217582
  2.69342354  0.63445407  2.61720836  1.8151063   2.55693516  2.94617413
  0.95243146  1.48424872  2.79423085  1.77710559  2.26261465  3.20067571
  0.87567798  2.34585728  0.05142816  2.69460069], Cost: 0.4420
Current parameters: [ 3.33201974  2.95701457  2.84306693 -0.03952369  0.70666704  0.48102927
  2.68759545  0.61889014  2.62907492  1.80819874  2.53476259  2.96135725
  0.9454187   1.40673523  2.79555764  1.71832762  2.23558449  3.1677003
  0.86234777  2.28442854  0.03432485  2.73209985], Cost: 0.1138
Current parameters: [ 3.3217401   2.93492345  2.82485145 -0.0762606   0.63677737  0.41750092
  2.67837209  0.62553386  2.59539927  1.76314785  2.55939381  2.93654918
  0.93366777  1.37910902  2.80381537  1.71047883  2.24488905  3.18796888
  0.89736714  2.24106102  0.02055039  2.7367117 ], Cost: 0.0900
Current parameters: [ 3.35797309  2.98602587  2.82043815 -0.04818415  0.67599298  0.45134081
  2.68863729  0.62424767  2.61698996  1.81226098  2.54364635  2.95139454
  0.94057836  1.40175529  2.80649281  1.71100202  2.22336374  3.20824274
  0.86686734  2.30531253  0.06663492  2.73055995], Cost: 0.1047
Current parameters: [ 3.39458512  3.02711801  2.83474178 -0.03938992  0.66000534  0.43925193
  2.68033474  0.61838115  2.64094256  1.80246884  2.57405068  2.96369134
  0.92620758  1.40175973  2.78738035  1.75556078  2.24921241  3.13196883
  0.87664624  2.29971944 -0.00384226  2.74119183], Cost: 0.0727
Current parameters: [ 3.33495525  2.96045426  2.7933179  -0.041781    0.6898517   0.46237142
  2.69973563  0.62909827  2.6259026   1.80522353  2.55691409  2.95236825
  0.94968115  1.36916715  2.80498604  1.74857871  2.23586477  3.18002554
  0.8704223   2.30815522  0.03496231  2.70709944], Cost: 0.1240
Current parameters: [ 3.33371705  2.95537402  2.75828972 -0.0532264   0.73843515  0.42784291
  2.67249978  0.62905701  2.63331297  1.81517576  2.56258558  2.94622625
  0.90829664  1.42900544  2.80826374  1.7168308   2.21658739  3.17628921
```

```
  0.89292872  2.30645787  0.0215032   2.78913691], Cost: 0.0893
Current parameters: [ 3.33022429  2.95562096  2.80841503 -0.03769132  0.65635919  0.47427561
  2.68493402  0.62840522  2.64861122  1.81440885  2.55699695  2.95577184
  0.90058065  1.40440033  2.80115963  1.7112582   2.24563326  3.18003859
  0.86429793  2.30634702  0.04016497  2.72139883], Cost: 0.0762
Current parameters: [ 3.337584    2.96219267  2.81644553 -0.05232195  0.66289052  0.46968945
  2.62364085  0.63852343  2.6993172   1.8093237   2.55550053  2.93910825
  1.00149682  1.39745654  2.81548303  1.70982234  2.22838107  3.19191387
  0.89936799  2.30974661  0.02861976  2.73271653], Cost: 0.0990
Current parameters: [ 3.3355674   2.96016038  2.81179749 -0.04769335  0.65395581  0.48337826
  2.69042791  0.62802503  2.62142666  1.81118     2.53192479  2.94908508
  0.94265366  1.39748081  2.80802475  1.75145726  2.23204122  3.18456486
  0.86572577  2.30735079  0.03521454  2.77035037], Cost: 0.0685
Current parameters: [ 3.3346779   2.95973783  2.79590128 -0.02828062  0.62938811  0.50381729
  2.70712694  0.60975074  2.61050781  1.81733889  2.51501628  2.9758281
  0.94306817  1.41124106  2.78359219  1.74654524  2.17449064  3.15905758
  0.94907982  2.30267799  0.04676234  2.75230202], Cost: 0.0857
Current parameters: [ 3.33385147  2.94469566  2.81247736 -0.03886458  0.64970976  0.46197403
  2.69784029  0.62171741  2.63341065  1.8054344   2.538546    2.98305975
  0.95535877  1.39300725  2.79276408  1.75285811  2.24779488  3.17966764
  0.86364882  2.31421091  0.05976808  2.78759556], Cost: 0.1110
Current parameters: [ 3.34072438  3.00110182  2.78100335 -0.05820166  0.66086557  0.51563496
  2.65009736  0.6120517   2.62611507  1.78898373  2.47889468  2.97849119
  0.90680246  1.35387917  2.81571179  1.74181055  2.26619025  3.20362527
  0.88052969  2.28858381  0.02713906  2.78440059], Cost: 0.2041
Current parameters: [ 3.36838639  2.9346119   2.80967726 -0.03412569  0.64707772  0.47992503
  2.68478129  0.64791109  2.61260489  1.83682546  2.52416072  2.94691791
  0.94196295  1.38368594  2.80712202  1.73800847  2.2257365   3.17878658
  0.85884886  2.30194608  0.02244599  2.77430789], Cost: 0.0575
Current parameters: [ 3.36446368  2.93105314  2.82069122 -0.02864573  0.64992281  0.47472251
  2.6692082   0.64311865  2.61062649  1.84324056  2.52641468  2.94467303
  0.93209549  1.376451    2.81032408  1.75108998  2.22040426  3.17860018
  0.86227479  2.3028517   0.03075781  2.77393999], Cost: 0.0547
Current parameters: [ 3.37073177  2.92918756  2.81295116 -0.00577518  0.64517384  0.47545441
  2.67414448  0.61846098  2.60965319  1.83618335  2.52556919  2.91551932
  0.93761509  1.37631411  2.83602127  1.73962628  2.23006056  3.15474776
  0.86186153  2.30230587  0.04491625  2.7801279 ], Cost: 0.0810
Current parameters: [ 3.36266958  2.92738253  2.82608098 -0.03339306  0.64958372  0.47052303
  2.66534237  0.64004868  2.60408076  1.83493449  2.51898439  2.94414851
  0.93084326  1.37275085  2.80777566  1.74320214  2.21822404  3.17316745
  0.86192447  2.32599895  0.02425051  2.7724104 ], Cost: 0.0393
```



```
Current parameters: [ 3.37484134  2.90996521  2.83009565 -0.04166213  0.64193145  0.47010888
  2.65586675  0.64844227  2.5900908   1.83758238  2.51587272  2.93302479
  0.92809913  1.4131221   2.8060537   1.76256987  2.23472467  3.17838653
  0.86942448  2.34078557  0.01784446  2.75883698], Cost: 0.0642
Current parameters: [ 3.35557353  2.9317485   2.81538651 -0.03075627  0.64424731  0.47108109
  2.65093151  0.63585143  2.59205019  1.83825401  2.52284989  2.9494694
  0.93759049  1.37797759  2.80249677  1.7409429   2.21491588  3.16727477
  0.84919301  2.32312137  0.02423215  2.76911153], Cost: 0.0376
Current parameters: [ 3.3406565   2.9348708   2.82532485 -0.04331285  0.62675252  0.45433486
  2.66171939  0.63890047  2.60665237  1.85036291  2.51699672  2.93763086
  0.92892178  1.38122958  2.8038424   1.74194518  2.17979549  3.15986647
  0.82734537  2.32766101  0.00737174  2.77258188], Cost: 0.1398
Current parameters: [ 3.36515523  2.92411601  2.81393616 -0.0485371   0.6446755   0.47820637
  2.6511388   0.61754272  2.59393174  1.84368936  2.53101096  2.95057277
  0.93720674  1.37512971  2.80109107  1.74078658  2.21257737  3.16761094
  0.84703615  2.32271733  0.02491204  2.76921763], Cost: 0.0438
Current parameters: [ 3.35557353  2.9317485   2.81538651 -0.03075627  0.64424731  0.47108109
  2.65093151  0.63585143  2.59205019  1.83825401  2.52284989  2.9494694
  0.93759049  1.37797759  2.80249677  1.7409429   2.21491588  3.16727477
  0.84919301  2.32312137  0.02423215  2.76911153], Cost: 0.0376

   Return from subroutine COBYLA because the MAXFUN limit has been reached.

   NFVALS =  100   F = 3.757239E-02    MAXCV = 0.000000E+00
   X = 3.355574E+00   2.931749E+00   2.815387E+00  -3.075627E-02   6.442473E-01
       4.710811E-01   2.650932E+00   6.358514E-01   2.592050E+00   1.838254E+00
       2.522850E+00   2.949469E+00   9.375905E-01   1.377978E+00   2.802497E+00
       1.740943E+00   2.214916E+00   3.167275E+00   8.491930E-01   2.323121E+00
       2.423215E-02   2.769112E+00
Simulating for QAOA depth p = 12
Current parameters: [3.70311473 2.15876158 2.58563212 1.54521945 2.59065843 2.72292695
 2.89635046 1.51190808 2.51822621 0.11376796 1.0619238  1.74600185
 2.16914469 2.80844431 0.32725991 1.87893248 1.26743057 2.25520384
 1.61179501 2.04006024 1.12510923 0.39538096 2.68389981 1.36730757], Cost: 2.5655
Current parameters: [2.70311473 3.15876158 2.58563212 1.54521945 2.59065843 2.72292695
 2.89635046 1.51190808 2.51822621 0.11376796 1.0619238  1.74600185
 2.16914469 2.80844431 0.32725991 1.87893248 1.26743057 2.25520384
 1.61179501 2.04006024 1.12510923 0.39538096 2.68389981 1.36730757], Cost: 2.2010
Current parameters: [2.70311473 3.15876158 3.58563212 1.54521945 2.59065843 2.72292695
 2.89635046 1.51190808 2.51822621 0.11376796 1.0619238  1.74600185
 2.16914469 2.80844431 0.32725991 1.87893248 1.26743057 2.25520384
```



```
 1.61179501 2.04006024 1.12510923 0.39538096 2.68389981 1.36730757], Cost: 3.1056
Current parameters: [2.70311473 3.15876158 2.58563212 2.54521945 2.59065843 2.72292695
 2.89635046 1.51190808 2.51822621 0.11376796 1.0619238  1.74600185
 2.16914469 2.80844431 0.32725991 1.87893248 1.26743057 2.25520384
 1.61179501 2.04006024 1.12510923 0.39538096 2.68389981 1.36730757], Cost: 1.2097
Current parameters: [2.70311473 3.15876158 2.58563212 2.54521945 3.59065843 2.72292695
 2.89635046 1.51190808 2.51822621 0.11376796 1.0619238  1.74600185
 2.16914469 2.80844431 0.32725991 1.87893248 1.26743057 2.25520384
 1.61179501 2.04006024 1.12510923 0.39538096 2.68389981 1.36730757], Cost: 1.4028
Current parameters: [2.70311473 3.15876158 2.58563212 2.54521945 2.59065843 3.72292695
 2.89635046 1.51190808 2.51822621 0.11376796 1.0619238  1.74600185
 2.16914469 2.80844431 0.32725991 1.87893248 1.26743057 2.25520384
 1.61179501 2.04006024 1.12510923 0.39538096 2.68389981 1.36730757], Cost: 0.7501
Current parameters: [2.70311473 3.15876158 2.58563212 2.54521945 2.59065843 3.72292695
 3.89635046 1.51190808 2.51822621 0.11376796 1.0619238  1.74600185
 2.16914469 2.80844431 0.32725991 1.87893248 1.26743057 2.25520384
 1.61179501 2.04006024 1.12510923 0.39538096 2.68389981 1.36730757], Cost: 2.2504
Current parameters: [2.70311473 3.15876158 2.58563212 2.54521945 2.59065843 3.72292695
 2.89635046 2.51190808 2.51822621 0.11376796 1.0619238  1.74600185
 2.16914469 2.80844431 0.32725991 1.87893248 1.26743057 2.25520384
 1.61179501 2.04006024 1.12510923 0.39538096 2.68389981 1.36730757], Cost: 2.0970
Current parameters: [2.70311473 3.15876158 2.58563212 2.54521945 2.59065843 3.72292695
 2.89635046 1.51190808 3.51822621 0.11376796 1.0619238  1.74600185
 2.16914469 2.80844431 0.32725991 1.87893248 1.26743057 2.25520384
 1.61179501 2.04006024 1.12510923 0.39538096 2.68389981 1.36730757], Cost: 1.3373
Current parameters: [2.70311473 3.15876158 2.58563212 2.54521945 2.59065843 3.72292695
 2.89635046 1.51190808 2.51822621 1.11376796 1.0619238  1.74600185
 2.16914469 2.80844431 0.32725991 1.87893248 1.26743057 2.25520384
 1.61179501 2.04006024 1.12510923 0.39538096 2.68389981 1.36730757], Cost: 2.3898
Current parameters: [2.70311473 3.15876158 2.58563212 2.54521945 2.59065843 3.72292695
 2.89635046 1.51190808 2.51822621 0.11376796 2.0619238  1.74600185
 2.16914469 2.80844431 0.32725991 1.87893248 1.26743057 2.25520384
 1.61179501 2.04006024 1.12510923 0.39538096 2.68389981 1.36730757], Cost: 1.5005
Current parameters: [2.70311473 3.15876158 2.58563212 2.54521945 2.59065843 3.72292695
 2.89635046 1.51190808 2.51822621 0.11376796 1.0619238  2.74600185
 2.16914469 2.80844431 0.32725991 1.87893248 1.26743057 2.25520384
 1.61179501 2.04006024 1.12510923 0.39538096 2.68389981 1.36730757], Cost: 1.3470
Current parameters: [2.70311473 3.15876158 2.58563212 2.54521945 2.59065843 3.72292695
 2.89635046 1.51190808 2.51822621 0.11376796 1.0619238  1.74600185
 3.16914469 2.80844431 0.32725991 1.87893248 1.26743057 2.25520384
 1.61179501 2.04006024 1.12510923 0.39538096 2.68389981 1.36730757], Cost: 1.6522
```



```
Current parameters: [2.70311473 3.15876158 2.58563212 2.54521945 2.59065843 3.72292695
 2.89635046 1.51190808 2.51822621 0.11376796 1.0619238  1.74600185
 2.16914469 3.80844431 0.32725991 1.87893248 1.26743057 2.25520384
 1.61179501 2.04006024 1.12510923 0.39538096 2.68389981 1.36730757], Cost: 1.6702
Current parameters: [2.70311473 3.15876158 2.58563212 2.54521945 2.59065843 3.72292695
 2.89635046 1.51190808 2.51822621 0.11376796 1.0619238  1.74600185
 2.16914469 2.80844431 1.32725991 1.87893248 1.26743057 2.25520384
 1.61179501 2.04006024 1.12510923 0.39538096 2.68389981 1.36730757], Cost: 2.2782
Current parameters: [2.70311473 3.15876158 2.58563212 2.54521945 2.59065843 3.72292695
 2.89635046 1.51190808 2.51822621 0.11376796 1.0619238  1.74600185
 2.16914469 2.80844431 0.32725991 2.87893248 1.26743057 2.25520384
 1.61179501 2.04006024 1.12510923 0.39538096 2.68389981 1.36730757], Cost: 0.8842
Current parameters: [2.70311473 3.15876158 2.58563212 2.54521945 2.59065843 3.72292695
 2.89635046 1.51190808 2.51822621 0.11376796 1.0619238  1.74600185
 2.16914469 2.80844431 0.32725991 1.87893248 2.26743057 2.25520384
 1.61179501 2.04006024 1.12510923 0.39538096 2.68389981 1.36730757], Cost: 1.9513
Current parameters: [2.70311473 3.15876158 2.58563212 2.54521945 2.59065843 3.72292695
 2.89635046 1.51190808 2.51822621 0.11376796 1.0619238  1.74600185
 2.16914469 2.80844431 0.32725991 1.87893248 1.26743057 3.25520384
 1.61179501 2.04006024 1.12510923 0.39538096 2.68389981 1.36730757], Cost: 2.7586
Current parameters: [2.70311473 3.15876158 2.58563212 2.54521945 2.59065843 3.72292695
 2.89635046 1.51190808 2.51822621 0.11376796 1.0619238  1.74600185
 2.16914469 2.80844431 0.32725991 1.87893248 1.26743057 2.25520384
 2.61179501 2.04006024 1.12510923 0.39538096 2.68389981 1.36730757], Cost: 3.1752
Current parameters: [2.70311473 3.15876158 2.58563212 2.54521945 2.59065843 3.72292695
 2.89635046 1.51190808 2.51822621 0.11376796 1.0619238  1.74600185
 2.16914469 2.80844431 0.32725991 1.87893248 1.26743057 2.25520384
 1.61179501 3.04006024 1.12510923 0.39538096 2.68389981 1.36730757], Cost: 2.2628
Current parameters: [2.70311473 3.15876158 2.58563212 2.54521945 2.59065843 3.72292695
 2.89635046 1.51190808 2.51822621 0.11376796 1.0619238  1.74600185
 2.16914469 2.80844431 0.32725991 1.87893248 1.26743057 2.25520384
 1.61179501 2.04006024 2.12510923 0.39538096 2.68389981 1.36730757], Cost: 3.2461
Current parameters: [2.70311473 3.15876158 2.58563212 2.54521945 2.59065843 3.72292695
 2.89635046 1.51190808 2.51822621 0.11376796 1.0619238  1.74600185
 2.16914469 2.80844431 0.32725991 1.87893248 1.26743057 2.25520384
 1.61179501 2.04006024 1.12510923 1.39538096 2.68389981 1.36730757], Cost: 3.4234
Current parameters: [2.70311473 3.15876158 2.58563212 2.54521945 2.59065843 3.72292695
 2.89635046 1.51190808 2.51822621 0.11376796 1.0619238  1.74600185
 2.16914469 2.80844431 0.32725991 1.87893248 1.26743057 2.25520384
 1.61179501 2.04006024 1.12510923 0.39538096 3.68389981 1.36730757], Cost: 2.7055
Current parameters: [2.70311473 3.15876158 2.58563212 2.54521945 2.59065843 3.72292695
```

```
 2.89635046 1.51190808 2.51822621 0.11376796 1.0619238  1.74600185
 2.16914469 2.80844431 0.32725991 1.87893248 1.26743057 2.25520384
 1.61179501 2.04006024 1.12510923 0.39538096 2.68389981 2.36730757], Cost: 0.6934
Current parameters: [ 2.70205854e+00  3.21200269e+00  2.45087036e+00  2.69289927e+00
  2.56188846e+00  3.79139934e+00  2.67284415e+00  1.31124906e+00
  2.43074432e+00 -1.30504162e-01  9.50135051e-01  1.65707914e+00
  2.03474809e+00  2.67137404e+00  9.96092369e-02  1.85894181e+00
  1.08847344e+00  1.95598566e+00  1.25052314e+00  1.81470723e+00
  7.53270626e-01 -2.86995531e-03  2.39259166e+00  2.37574180e+00], Cost: 1.7481
Current parameters: [2.20311785 3.15876158 2.58563212 2.54521945 2.59065843 3.72292695
 2.89635046 1.51190808 2.51822621 0.11376796 1.0619238  1.74600185
 2.16914469 2.80844431 0.32725991 1.87893248 1.26743057 2.25696875
 1.61179501 2.04006024 1.12510923 0.39538096 2.68389981 2.36730757], Cost: 0.6868
Current parameters: [2.20599567 3.17317724 2.54914376 2.58520552 2.58286862 3.74146667
 2.83583345 1.45757725 2.49453944 0.04762836 1.03165566 1.72192496
 2.1327552  2.77133089 0.26562077 1.87351976 1.21897579 3.22301404
 1.51397633 1.97904322 1.02442948 0.28754976 2.60502464 2.36959124], Cost: 1.9769
Current parameters: [2.20311785 3.15876158 3.07855818 2.54521945 2.59065843 3.74268085
 2.87659655 1.49215417 2.49847231 0.09401405 1.04216989 1.72624794
 2.14939078 2.7886904  0.30750601 1.85917857 1.24767666 2.25696875
 1.5920411  2.02030633 1.10535533 0.37562706 2.6641459  2.34755367], Cost: 1.4078
Current parameters: [2.20008605 3.22411612 2.44443739 2.72649941 2.55534271 3.40263428
 3.02633586 1.66993898 2.81518433 0.2182629  1.32904476 2.04119134
 2.40851406 3.0445317  0.45215801 2.25873744 1.45210098 2.08154452
 1.57267039 2.16777878 1.07301373 0.31086381 2.73065708 2.78200467], Cost: 1.9237
Current parameters: [2.20265066 3.16883254 2.5451921  2.26918139 2.88918919 3.98655532
 2.90736906 1.52724841 2.55497487 0.12085854 1.09407463 1.78247796
 2.19701907 2.83581294 0.33749457 1.92844761 1.28687598 2.22993634
 1.5967542  2.05072952 1.10806964 0.37334528 2.68209317 2.42219949], Cost: 0.3542
Current parameters: [2.20265066 2.9839042  2.5451921  2.40589158 2.95949729 4.05295741
 2.91127506 1.53115442 2.55888087 0.12476455 1.09798063 1.78638397
 2.20092507 2.83971895 0.34140057 1.93235362 1.29078199 2.22993634
 1.6006602  2.05463552 1.11197565 0.37725129 2.68599917 2.4261055 ], Cost: 0.3215
Current parameters: [2.20241529 2.6480121  2.52481884 2.09992759 2.81896309 3.91100258
 2.90066329 1.52271989 2.56123163 0.11217388 1.09801506 1.78859742
 2.19880507 2.83734415 0.33039386 1.941136   1.28441556 2.21631769
 1.57691997 2.04384777 1.08722845 0.34998711 2.66892618 2.43759665], Cost: 2.1156
Current parameters: [2.20265066 2.9839042  2.5451921  2.40589158 2.79351639 4.21893831
 2.92564846 1.54552782 2.57325427 0.13913795 1.11235403 1.80075737
 2.21529847 2.85409235 0.35577398 1.94672702 1.30515539 2.22993634
 1.6150336  2.06900893 1.12634905 0.31325777 2.70037257 2.4404789 ], Cost: 0.6106
```



```
Current parameters: [2.20202517 3.06742729 2.49105031 2.46478766 2.9535081  4.18022721
 2.88198196 1.50764733 2.56403561 0.09021255 1.09697972 1.79117382
 2.19419878 2.83231555 0.31105792 1.95460034 1.27277085 2.19374488
 1.53647831 2.02487475 1.04511776 0.826789   2.63953542 2.45555075], Cost: 2.2774
Current parameters: [2.20265066 2.9839042  2.5451921  2.40589158 2.95949729 4.05295741
 2.91190402 1.53178338 2.55950983 0.12539351 1.09860959 1.78701293
 2.20155403 2.84034791 0.34202954 1.93298258 1.29141095 2.22993634
 1.41954345 2.05526449 1.28428694 0.37725129 2.68662813 2.42673446], Cost: 1.4429
Current parameters: [2.20204055 3.06537351 2.49238162 2.46333945 3.0826882  4.04806491
 2.86452616 1.49004926 2.54573276 0.07288607 1.07882823 1.77287995
 2.17618808 2.8143215  0.29362793 1.93587721 1.25503764 2.1946348
 1.99354504 2.00743045 1.28695409 0.24548678 2.62250184 2.43665062], Cost: 0.9355
Current parameters: [ 2.20265066  2.9839042   2.5451921   2.40589158  2.95949729  4.05295741
  2.91642306  1.53630242  2.56402887 -0.07972335  1.10312863  1.79153197
  2.20607308  2.84486695  0.34654858  1.93750162  1.29592999  2.22993634
  1.6006602   2.05978353  1.11197565  0.37725129  2.82871103  2.4312535 ], Cost: 0.3375
Current parameters: [2.20231887 3.02820926 2.51647243 2.43713317 3.0264916  4.05029675
 2.86230036 1.48524893 2.52817913 0.37733621 1.06401361 1.75548867
 2.163921   2.80235571 0.29186914 1.91071836 1.24779184 2.21073848
 1.61591473 2.00541275 0.99709737 0.30559443 3.06174298 2.40828873], Cost: 1.3886
Current parameters: [2.20265066 2.9839042  2.5451921  2.40589158 2.95949729 4.05295741
 2.91164744 1.5315268  2.55925325 0.12476455 1.09835301 1.78675635
 2.20129745 2.84009133 0.5163036  1.932726   1.29115437 2.22993634
 1.6006602  1.87600869 1.11197565 0.37725129 2.68599917 2.42647788], Cost: 0.6426
Current parameters: [2.20241344 3.01558016 2.52465894 2.4282278  3.00739496 4.05105517
 2.85477206 1.47684575 2.51544214 0.10439287 1.05220738 1.74280686
 2.15298049 2.79151757 0.65805    1.89539699 1.23855765 2.2162108
 1.61156644 2.3894428  1.02984327 0.32602012 2.64840944 2.3918789 ], Cost: 0.6046
Current parameters: [2.20265066 2.9839042  2.5451921  2.40589158 2.95949729 4.05295741
 3.07006034 1.3384286  2.56312344 0.12476455 1.1022232  1.79062653
 2.20516764 2.84396151 0.34140057 1.93659618 1.29502455 2.22993634
 1.6006602  2.05463552 1.11197565 0.37725129 2.68599917 2.43034807], Cost: 0.3406
Current parameters: [2.20240781 3.01633187 2.52417166 2.42875786 3.00853163 4.05101002
 3.26805738 1.81694697 2.50492364 0.10390942 1.04163348 1.73228506
 2.14235505 2.78088604 0.3212087  1.88503231 1.22783065 2.21588508
 1.61182526 2.06162895 1.02789417 0.32480435 2.6475174  2.38157901], Cost: 0.9877
Current parameters: [2.20265066 2.9839042  2.5451921  2.40589158 2.95949729 4.05295741
 2.91127506 1.53115442 2.57274652 0.12476455 1.11184628 1.80024962
 2.21479072 2.62799579 0.34140057 1.94621927 1.41931124 2.22993634
 1.6006602  2.05463552 1.11197565 0.37725129 2.68599917 2.43997115], Cost: 0.3656
Current parameters: [2.20248509 3.0060123  2.53086106 2.42148105 2.99292727 4.05162975
```



```
 2.90131255 1.52733208 2.48101428 0.11054622 1.01848468 1.7084208
 2.11991364 3.05466083 0.32763442 1.85901119 1.69116471 2.22035666
 1.60827217 2.05940341 1.05465169 0.34149471 2.65976358 2.35466848], Cost: 1.6492
Current parameters: [2.20265066 2.9839042  2.5451921  2.40589158 2.95949729 4.05295741
 2.91127506 1.53115442 2.5717977  0.12476455 0.89822477 1.79930079
 2.34901363 2.83971895 0.34140057 1.94527044 1.29078199 2.22993634
 1.6006602  2.05463552 1.11197565 0.37725129 2.68599917 2.43902232], Cost: 0.2608
Current parameters: [2.20252358 3.00087278 2.53419263 2.41785693 2.98515572 4.05193839
 2.90362856 1.52822067 2.45096818 0.11385159 1.13514993 1.67839715
 2.71316914 2.84292962 0.33083467 1.82791334 1.27983446 2.22258367
 1.6065026  2.05829501 1.06797793 0.34980712 2.66586263 2.32312768], Cost: 2.1934
Current parameters: [2.20265066 2.9839042  2.5451921  2.40589158 2.95949729 4.05295741
 2.91127506 1.53115442 2.39348955 0.12476455 0.89822477 1.97451903
 2.34901363 2.83971895 0.34140057 1.9468154  1.29078199 2.22993634
 1.6006602  2.05463552 1.11197565 0.37725129 2.68599917 2.44056728], Cost: 0.3899
Current parameters: [2.20254129 2.99850758 2.53572581 2.41618912 2.98157928 4.05208043
 2.90469438 1.52862959 2.84452536 0.11537271 0.89970049 2.07583228
 2.3439246  2.84248209 0.33230742 1.72607806 1.2813604  2.22360853
 1.60568824 2.05778492 1.07411063 0.35363248 2.6686694  2.22108855], Cost: 1.3358
Current parameters: [2.20261723 2.98558891 2.54092782 2.41056462 2.95858692 4.05512409
 2.90420263 1.52480495 2.5690295  0.11703503 0.89468743 1.796487
 2.3447609  2.83538162 0.33419701 1.76973208 1.28511923 2.22046815
 1.58922845 2.04750466 1.10020953 0.3646494  2.67678129 2.61419501], Cost: 0.1108
Current parameters: [ 2.20199470e+00  3.03145883e+00  2.46864983e+00  2.48964967e+00
  2.98157262e+00  4.08278467e+00  2.79886238e+00  1.43562501e+00
  2.56145788e+00 -1.59836698e-03  8.49598517e-01  1.77995088e+00
  2.27961453e+00  2.78161568e+00  2.23093485e-01  1.87017115e+00
  1.19415683e+00  2.08340030e+00  1.44392560e+00  1.95688888e+00
  8.82057769e-01  1.57994924e-01  2.52553494e+00  2.63681852e+00], Cost: 2.5613
Current parameters: [2.20331285 2.97886738 2.51754822 2.3919205  2.96221903 4.10247616
 2.87642311 1.49414103 2.52407732 0.09187713 0.85280392 1.75171672
 2.30573156 2.79668982 0.3069407  1.71625934 1.25171552 2.41753509
 1.5788414  2.01995828 1.0911565  0.35893085 2.65756154 2.55713371], Cost: 0.4698
Current parameters: [2.20336901 3.03230187 2.5680308  2.47152169 3.00142357 3.95564641
 2.94730274 1.5829293  2.70379589 0.1484675  0.98450586 1.91898321
 2.41391751 2.92169291 0.37053618 1.95261761 1.33309949 2.48944241
 1.62364981 2.11223517 1.03451872 0.3245974  2.6782187  2.84053974], Cost: 3.3653
Current parameters: [2.20261981 2.96987    2.77583448 2.41603885 2.99266516 4.06716459
 2.8726134  1.52980712 2.56108415 0.12416292 0.90808921 1.78854902
 2.32356643 2.84444487 0.30058359 1.76144171 1.25588192 2.22119875
 1.59677397 2.04932637 1.07245958 0.36873855 2.64758257 2.60575933], Cost: 0.5996
```



```
Current parameters: [2.20192202 3.09803361 2.61774975 2.28713373 2.8471524  4.13677257
 3.05473391 1.433489   2.54530118 0.02310482 0.68745473 1.75374996
 2.35653256 2.68313933 0.49177348 1.74248281 1.37093543 2.11856451
 1.64924134 2.02006002 1.23330965 0.36861729 2.82890478 2.62483118], Cost: 1.2395
Current parameters: [2.20248521 2.98746735 2.52070999 2.42778102 2.99442021 4.0495907
 2.8707432  1.51537956 2.55181993 0.25576684 0.88294695 1.77594633
 2.30858934 2.82464295 0.29821776 1.75343407 1.24603681 2.19956064
 1.5970468  2.04270675 1.05567881 0.34934718 2.85520118 2.60511254], Cost: 0.6487
Current parameters: [2.07764305 2.985539   2.54112825 2.41193268 2.95710739 4.05381754
 2.90414802 1.52472892 2.56884737 0.11699989 0.89452809 1.79630623
 2.34462276 2.83524598 0.33414628 1.76948668 1.28502286 2.22104327
 1.58930299 2.04745178 1.10029398 0.36475861 2.67679024 2.61392296], Cost: 0.0714
Current parameters: [2.08209313 2.98077143 2.55701161 2.54923064 2.80900149 3.92314057
 2.89971895 1.51772074 2.54956998 0.11196706 0.87472222 1.77594095
 2.32672835 2.81969031 0.32973421 1.74314883 1.27296834 2.22888067
 1.60927663 2.04569879 1.11125797 0.38343283 2.67578865 2.58716665], Cost: 0.1451
Current parameters: [2.07766766 2.98469672 2.54325801 2.41491524 2.95938652 4.05206696
 2.98761219 1.59472256 2.55814516 0.12030955 0.88609847 1.78561837
 2.33309739 2.82664077 0.3340676  1.75811212 1.27315013 2.22246693
 1.55477817 2.05085826 1.06027471 0.3633307  2.67475667 2.60226524], Cost: 0.0935
Current parameters: [2.07537083 2.98386111 2.50002956 2.43372207 3.02274631 4.05040357
 2.93572543 1.5868934  2.55002369 0.11218464 0.88880169 1.77295191
 2.29272    2.82761281 0.28286508 1.75635328 1.2233942  2.18373459
 1.75481514 2.05035436 1.19244629 0.3446018  2.62073175 2.6122818 ], Cost: 0.3660
Current parameters: [2.07769071 2.9695336  2.54525358 2.38848492 2.93420608 4.0485177
 2.92322007 1.53081394 2.58005984 0.11433336 0.89860052 1.80754649
 2.36157671 2.84140808 0.26166926 1.77939682 1.30580195 2.22380088
 1.59049774 1.96420657 1.11463743 0.37217151 2.69011281 2.62328461], Cost: 0.1316
Current parameters: [2.075481   2.82043202 2.51867336 2.27292494 2.90358815 3.98687729
 2.88612983 1.52431455 2.56327221 0.11783299 0.8878834  1.78607706
 2.3196639  2.83271969 0.35561652 1.76328973 1.26090849 2.19562136
 1.60981667 2.09619207 1.06418976 0.39138089 2.65725219 2.6170923 ], Cost: 0.1170
Current parameters: [2.07769982 2.97572022 2.54604189 2.40824249 2.96967501 4.03189801
 2.90446875 1.52883957 2.55225939 0.12083971 0.88069227 1.77975136
 2.32771889 2.8797226  0.33536459 1.75134751 1.36414852 2.22432783
 1.59513512 2.0523102  1.10254835 0.2954908  2.67657419 2.59513049], Cost: 0.2986
Current parameters: [2.07492272 3.00344412 2.50644914 2.43535306 2.96227588 4.10309729
 2.88445429 1.52830748 2.5466484  0.11346125 0.87020093 1.76838391
 2.29718506 2.90003867 0.31209655 1.74883486 1.37704875 2.18516779
 1.60553344 2.05988646 1.04703866 0.54996835 2.64602983 2.60566135], Cost: 0.1590
Current parameters: [2.07763539 2.98630218 2.54046491 2.41284656 2.95867227 4.05393086
```

```
 2.90345672 1.52485155 2.64087355 0.11667133 0.86630145 1.87016917
 2.2987324  2.8357222  0.33364504 1.73731188 1.28419548 2.22059986
 1.58846428 2.04755151 1.09879826 0.36307768 2.67537427 2.58183636], Cost: 0.0658
Current parameters: [2.07437225 2.99241869 2.51860314 2.40432446 2.96156263 4.05611763
 2.90820116 1.51967711 2.65894132 0.1037428  0.95501099 1.8833291
 2.45374543 2.82786844 0.33839716 1.61601958 1.27913882 2.1895115
 1.61590542 2.05834039 1.07554049 0.38047098 2.66857847 2.47354708], Cost: 0.4700
Current parameters: [2.07766371 2.99694364 2.57254824 2.38318052 2.93952393 4.06240821
 2.94305604 1.52713497 2.64004447 0.12260239 0.84430248 1.86942677
 2.30940571 2.82318936 0.37747113 1.72293822 1.31135626 2.24020352
 1.61437896 2.05802564 1.15922865 0.39448225 2.72144074 2.58472097], Cost: 0.3967
Current parameters: [2.07124062 3.03449443 2.51291962 2.39220326 2.91794283 4.0966228
 2.90917005 1.45104917 2.63043059 0.02063448 0.77513238 1.8457492
 2.28212616 2.75119614 0.34548851 1.75925483 1.26420374 2.09699485
 1.56495893 2.01011886 1.04051541 0.31903    2.64895515 2.5882955 ], Cost: 0.2905
Current parameters: [2.02873755 3.01372011 2.54217864 2.42102244 2.97502613 4.07142614
 2.86860359 1.49146718 2.6349946  0.11699299 0.89473742 1.86349895
 2.33999857 2.81992089 0.36993004 1.7757027  1.25351563 2.24265414
 1.58424257 2.07597932 1.10143093 0.36635693 2.68170532 2.62142824], Cost: 0.1717
Current parameters: [2.11906126 2.98670683 2.48526737 2.43090323 2.9473644  3.98445528
 2.9522838  1.56225969 2.72881268 0.08531105 0.86829328 1.93839221
 2.30361318 2.86991582 0.30760224 1.80242823 1.31798623 2.27520766
 1.63414075 2.05653202 0.98520818 0.36174236 2.62989486 2.67084514], Cost: 1.3898
Current parameters: [2.07832579 2.97215202 2.52034248 2.42049137 2.97087964 4.04387786
 2.89088162 1.53199071 2.64236976 0.11667496 0.87111272 1.86580866
 2.28689825 2.84289849 0.31343977 1.73292531 1.26915745 2.19603431
 1.50034996 2.06078613 1.16506327 0.37542924 2.65252178 2.57994711], Cost: 0.3696
Current parameters: [2.07763539 3.02749988 2.54046491 2.38239074 2.95122714 4.03092017
 2.89376765 1.53256612 2.63751001 0.12521391 0.87346753 1.86680563
 2.28867639 2.84416729 0.3361421  1.73394834 1.27579436 2.22059986
 1.58688247 2.0412888  1.09721645 0.36537589 2.66672667 2.57847282], Cost: 0.0842
Current parameters: [2.07781952 2.96179737 2.5350981  2.43021099 2.9781011  4.05040211
 2.89173724 1.53594928 2.63928909 0.12653071 0.87622492 1.86702268
 2.28684031 2.84756689 0.41217148 1.73415844 1.27311859 2.21404803
 1.58667464 1.96413894 1.09526941 0.37000652 2.66179385 2.57934897], Cost: 0.1906
Current parameters: [2.07763539 2.98630218 2.54046491 2.41284656 2.95624276 4.05636037
 2.86913366 1.56775595 2.64233443 0.10290402 0.85322517 1.87163006
 2.30943291 2.82088012 0.33420732 1.73877277 1.29261123 2.22059986
 1.58867466 2.04811379 1.09900864 0.36214099 2.68533968 2.58329725], Cost: 0.0786
Current parameters: [2.07782801 2.98506177 2.53485084 2.41297794 2.88295673 4.12824597
 2.8957651  1.54540755 2.64429224 0.14480204 0.89301001 1.87195386
```



```
 2.28421711 2.86580722 0.33795451 1.7390893  1.27230134 2.21374618
 1.59359055 2.05338316 1.10210519 0.33635835 2.66061617 2.58431052], Cost: 0.1146
Current parameters: [2.07763539 2.98630218 2.54046491 2.41284656 2.95867227 4.05393086
 2.90241805 1.52381289 2.63577297 0.15792897 0.84651011 1.87847885
 2.31403174 2.81332483 0.33260638 1.73903365 1.29612289 2.22059986
 1.58846428 2.04651285 1.09879826 0.36307768 2.64658062 2.58355813], Cost: 0.0460
Current parameters: [2.07773509 2.9856601  2.53755885 2.41291457 2.95755318 4.05432504
 2.902566   1.5235463  2.54742016 0.14400304 0.85195906 1.96472746
 2.30917255 2.81970234 0.33217419 1.73908832 1.29156053 2.21705213
 1.58782073 2.0468686  1.09721295 0.36392613 2.65322448 2.58397346], Cost: 0.1056
Current parameters: [2.07763909 2.98626493 2.54087579 2.41229847 2.95746752 4.05405878
 2.90356008 1.52413631 2.63654279 0.15343533 0.88432803 1.87935664
 2.29174953 2.7766076  0.333832   1.73972185 1.31996348 2.22111235
 1.58820663 2.04668678 1.10027837 0.36357023 2.64083388 2.58413582], Cost: 0.0411
Current parameters: [2.07803082 2.98395417 2.52127824 2.42124919 2.97141596 4.05382064
 2.88477685 1.51639191 2.62861725 0.21866011 0.88635719 1.86865243
 2.26463264 2.75743836 0.31111175 1.73064338 1.30170793 2.1970159
 1.58936506 2.04410919 1.06978001 0.35963218 2.7215222  2.57842822], Cost: 0.3488
Current parameters: [2.0774993  2.98529339 2.58034651 2.41686539 2.96177825 4.04629876
 2.90448347 1.53105543 2.64442519 0.1595595  0.89475085 1.88720348
 2.29647795 2.7857453  0.33435043 1.74927014 1.3223682  2.18151048
 1.59141619 2.05250707 1.09799638 0.36532762 2.64038617 2.59438571], Cost: 0.0287
Current parameters: [2.07823048 2.97291383 2.66209548 2.41372135 2.97242174 4.06875948
 2.88065788 1.52046605 2.62824621 0.14864424 0.8848599  1.86986315
 2.27321606 2.77400987 0.30683291 1.72746777 1.29455319 2.2266767
 1.58818013 2.04468727 1.07462298 0.36959876 2.61051698 2.57321857], Cost: 0.1115
Current parameters: [2.08383484 2.97790219 2.58880562 2.40993376 2.95825577 4.03148172
 2.91413825 1.53444401 2.65262469 0.16082792 0.89843187 1.89676296
 2.30468073 2.77127416 0.33677437 1.76025652 1.30219683 2.19027012
 1.58799187 2.04722055 1.11006867 0.32095494 2.64798579 2.58530498], Cost: 0.0340
Current parameters: [2.07508789 2.98621524 2.57880063 2.41727762 2.96277775 4.05245257
 2.90310567 1.53188262 2.64235664 0.16123699 0.89418767 1.88427945
 2.29417616 2.79267166 0.33515132 1.83388497 1.33148764 2.18007425
 1.59781092 2.05809355 1.09636724 0.39097129 2.63843136 2.50764829], Cost: 0.2041
Current parameters: [2.07066314 2.94669281 2.57313693 2.38344174 2.95241271 4.03298691
 2.8958205  1.52854204 2.63983374 0.15946048 0.89571282 1.88127889
 2.29244134 2.78284003 0.34168673 1.75053258 1.31047233 2.17397162
 1.60192012 2.06792852 1.09288324 0.37455473 2.63376495 2.5982869 ], Cost: 0.0796
Current parameters: [2.06801988 2.99782962 2.56122977 2.43748145 2.99957461 4.05582022
 2.91240555 1.55374494 2.6319417  0.15446247 0.89538107 1.87239096
 2.27697876 2.77682535 0.31110995 1.74591924 1.28420591 2.16143408
```



```
 1.6696334  2.05537945 1.14340332 0.35753468 2.60943437 2.59782384], Cost: 0.1241
Current parameters: [2.08243953 2.98518519 2.57695276 2.38218038 2.99765511 4.07675794
 2.91061544 1.5367439  2.65194105 0.16099233 0.89732866 1.89506534
 2.29804851 2.79167789 0.3319085  1.75322664 1.32963819 2.18000207
 1.58520584 2.04929404 1.09404455 0.35759317 2.64109077 2.59840624], Cost: 0.0305
Current parameters: [2.10018711 2.98770392 2.56906788 2.43499526 2.95572307 4.02110347
 2.9288614  1.54814175 2.6884467  0.14302837 0.89510514 1.9212981
 2.29656633 2.80221774 0.31269846 1.77081144 1.33725082 2.2162492
 1.60639115 2.05580252 1.04321394 0.36148871 2.60497338 2.64517583], Cost: 0.3027
Current parameters: [2.07652795 2.99332889 2.58132149 2.4125441  2.95306137 4.05365505
 2.90700777 1.51883026 2.63882002 0.14527144 0.86291576 1.87993486
 2.26929348 2.7721082  0.33873636 1.77178517 1.32288867 2.16626201
 1.58488646 2.04504616 1.09590018 0.35579815 2.64000435 2.61324693], Cost: 0.0269
Current parameters: [2.07655829 3.01441002 2.56869781 2.40512974 2.93714843 4.07070968
 2.91505489 1.48981013 2.64423403 0.10122753 0.86055927 1.87687931
 2.322185   2.74060831 0.33988517 1.72856115 1.32072517 2.11299754
 1.58703393 2.03445512 1.06183889 0.34099727 2.61957603 2.57983032], Cost: 0.0875
Current parameters: [2.06497278 3.00023919 2.59022175 2.43388502 2.95370696 4.04144791
 2.89530438 1.51526299 2.62758655 0.15252835 0.87075803 1.869608
 2.27572711 2.79666414 0.34484899 1.7552721  1.35836217 2.15754799
 1.58480075 2.0546348  1.09791234 0.34342099 2.64193538 2.61165158], Cost: 0.0077
Current parameters: [2.05278465 2.97509052 2.57256737 2.38856168 2.97675531 4.0439873
 2.88837801 1.50121776 2.61100819 0.13183388 0.84234532 1.84808377
 2.25022113 2.80002039 0.3472501  1.77663429 1.37990784 2.18159101
 1.60242707 2.06419725 1.09056892 0.26356483 2.61667752 2.60247081], Cost: 0.1526
Current parameters: [2.06548756 2.99394687 2.58742778 2.43807806 2.91816396 4.07949667
 2.89811317 1.51773191 2.63232538 0.16017962 0.88570883 1.87184568
 2.26438797 2.81549134 0.34319473 1.75461548 1.35197467 2.153934
 1.58944038 2.06017596 1.09725994 0.32919056 2.63867078 2.61088577], Cost: 0.0193
Current parameters: [2.06279611 3.0421625  2.59354923 2.40556483 2.92705749 4.04678307
 2.85078387 1.57016781 2.60966033 0.19399568 0.86134469 1.86059101
 2.30617607 2.77362836 0.37313709 1.76042492 1.36347664 2.16010058
 1.56814213 2.03543363 1.11487235 0.3417519  2.60313934 2.63021314], Cost: 0.0214
Current parameters: [2.06718774 3.00523761 2.60931306 2.4209138  2.94245979 4.04830172
 2.92072655 1.51339582 2.630993   0.14915657 0.8574267  1.87116296
 2.28091401 2.79133345 0.36357072 1.741906   1.3757647  2.16853865
 1.5993767  2.06089329 1.12335678 0.35364736 2.6592986  2.61366227], Cost: 0.0467
Current parameters: [2.05603034 3.00004801 2.59021237 2.43694873 2.95584893 4.04164039
 2.91305553 1.52892456 2.61975667 0.15256498 0.87100316 1.86128523
 2.27445476 2.7950834  0.34926215 1.75403684 1.35408923 2.15734641
 1.5763262  2.06212704 1.08919672 0.34275377 2.64101576 2.61040822], Cost: 0.0144
```



```
Current parameters: [2.0683901  2.99301652 2.5856442  2.43778477 2.9567288  4.04056545
 2.89527545 1.51145919 2.63373833 0.14489736 0.87022885 1.87025309
 2.27144431 2.80105776 0.3330537  1.74612415 1.35688042 2.14798905
 1.54323715 2.06306563 1.13432639 0.34638921 2.62793948 2.6129824 ], Cost: 0.1409
Current parameters: [2.0392765  2.99970857 2.59059159 2.43118116 2.95118441 4.04016404
 2.89564831 1.51712002 2.63554029 0.15328505 0.86797818 1.87808951
 2.27358608 2.79654937 0.33823149 1.75333925 1.36044849 2.15800187
 1.58477543 2.04512844 1.0992353  0.3441813  2.64236417 2.60967902], Cost: 0.0179
Current parameters: [2.06875582 2.98374974 2.58947402 2.44578111 2.96335949 4.04275175
 2.89764134 1.51495385 2.62941611 0.15121236 0.87608733 1.86838001
 2.26703011 2.80590682 0.38411693 1.74950876 1.354087   2.15459618
 1.58438134 2.01542802 1.09677911 0.34528129 2.63613026 2.60977822], Cost: 0.0305
Current parameters: [2.06979442 2.99395161 2.59082876 2.42977943 2.94834349 4.03928517
 2.9028905  1.51846416 2.61846283 0.15135458 0.87373565 1.85998502
 2.28150741 2.79975041 0.32957613 1.75831438 1.36462863 2.15589585
 1.5855747  2.03847926 1.10024794 0.34454242 2.64428174 2.61447252], Cost: 0.0136
Current parameters: [2.04456253 3.00407011 2.59355007 2.43958709 2.95901995 4.053181
 2.89140423 1.49204554 2.61926849 0.14459281 0.89078248 1.85769996
 2.30071174 2.79479364 0.35394838 1.77543398 1.34712458 2.16881705
 1.5835555  2.05712273 1.10206879 0.34478855 2.64905877 2.63590794], Cost: 0.0066
Current parameters: [2.04456837 3.00402801 2.59376732 2.43917574 2.9580844  4.05328582
 2.89232605 1.49235189 2.59803972 0.13808939 0.89203497 1.87929528
 2.30079641 2.79725378 0.35491927 1.77587664 1.34687767 2.1690904
 1.58334302 2.05733123 1.10312519 0.34518284 2.64686772 2.63627918], Cost: 0.0111
Current parameters: [2.04456253 3.00407011 2.59355007 2.43958709 2.95901995 4.053181
 2.89140423 1.49204554 2.61926849 0.14459281 0.89078248 1.85769996
 2.30071174 2.79479364 0.35394838 1.77543398 1.34712458 2.16881705
 1.5835555  2.05712273 1.10206879 0.34478855 2.64905877 2.63590794], Cost: 0.0066

   Return from subroutine COBYLA because the MAXFUN limit has been reached.

   NFVALS =  100   F = 6.573298E-03    MAXCV = 0.000000E+00
   X = 2.044563E+00   3.004070E+00   2.593550E+00   2.439587E+00   2.959020E+00
       4.053181E+00   2.891404E+00   1.492046E+00   2.619268E+00   1.445928E-01
       8.907825E-01   1.857700E+00   2.300712E+00   2.794794E+00   3.539484E-01
       1.775434E+00   1.347125E+00   2.168817E+00   1.583556E+00   2.057123E+00
       1.102069E+00   3.447886E-01   2.649059E+00   2.635908E+00
```

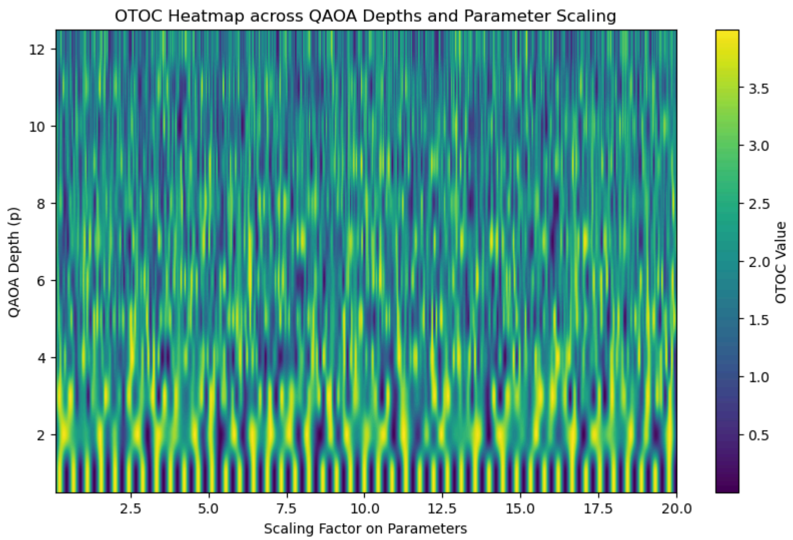


```
Run 4/30 for heatmap generation...
Simulating for QAOA depth p = 1
Current parameters: [2.39365823 1.77675745], Cost: 1.2683
Current parameters: [2.39365823 2.77675745], Cost: 2.9910
Current parameters: [2.89049916 0.90891582], Cost: 2.2283
Current parameters: [2.6420787  1.34283664], Cost: 2.6649
Current parameters: [2.14849409 1.72782338], Cost: 1.4624
Current parameters: [2.40380579 2.02655142], Cost: 1.0360
Current parameters: [2.54367954 2.23375985], Cost: 1.5623
Current parameters: [2.28148998 2.05231654], Cost: 1.0729
Current parameters: [2.39850391 1.96427671], Cost: 1.0036
Current parameters: [2.41918598 1.90529788], Cost: 1.0347
Current parameters: [2.33653694 1.95613149], Cost: 1.0012
Current parameters: [2.3301286  1.98671737], Cost: 1.0057
Current parameters: [2.33266548 1.92512223], Cost: 1.0129
Current parameters: [2.3520578  1.95793246], Cost: 1.0003
Current parameters: [2.35594625 1.95115639], Cost: 1.0012
Current parameters: [2.35397111 1.96550705], Cost: 1.0000
Current parameters: [2.34697757 1.96898922], Cost: 1.0004
Current parameters: [2.35774517 1.96651465], Cost: 1.0001
Current parameters: [2.35306346 1.96723646], Cost: 1.0001
Current parameters: [2.35412212 1.96355977], Cost: 1.0000
Current parameters: [2.3560544  1.96327519], Cost: 1.0000
Current parameters: [2.35624905 1.96133179], Cost: 1.0000
Current parameters: [2.35638665 1.96419349], Cost: 1.0000
Current parameters: [2.35632917 1.96287156], Cost: 1.0000
Current parameters: [2.3555662  1.96326655], Cost: 1.0000
Current parameters: [2.35609975 1.96351508], Cost: 1.0000
Current parameters: [2.35628252 1.96367694], Cost: 1.0000
Current parameters: [2.35589723 1.96365143], Cost: 1.0000
Current parameters: [2.35611795 1.96341675], Cost: 1.0000
Current parameters: [2.35605058 1.96350598], Cost: 1.0000
Current parameters: [2.35615874 1.96359583], Cost: 1.0000

   Normal return from subroutine COBYLA

Current parameters: [2.35615874 1.96359583], Cost: 1.0000
   NFVALS =   32   F = 1.000000E+00    MAXCV = 0.000000E+00
   X = 2.356159E+00   1.963596E+00
Simulating for QAOA depth p = 2
Current parameters: [3.42752526 2.19166501 0.50802619 1.91537205], Cost: 1.4860
```



```
Current parameters: [2.42752526 3.19166501 0.50802619 1.91537205], Cost: 2.3648
Current parameters: [2.42752526 2.19166501 1.50802619 1.91537205], Cost: 2.4197
Current parameters: [2.42752526 2.19166501 0.50802619 2.91537205], Cost: 1.7684
Current parameters: [ 2.08171027  1.60681925 -0.0917743   1.49275305], Cost: 2.0528
Current parameters: [2.25461776 1.89924213 0.20812595 1.70406255], Cost: 1.5153
Current parameters: [2.67246409 2.16046896 0.47603245 1.89282928], Cost: 0.4168
Current parameters: [2.42752526 2.28116202 0.42076059 1.91537205], Cost: 0.3967
Current parameters: [2.41425104 2.28784568 0.72183032 1.82957655], Cost: 0.7966
Current parameters: [2.41930626 2.1464097  0.53291987 2.15984027], Cost: 0.3422
Current parameters: [2.38225352 2.08911441 0.46195043 1.88478003], Cost: 0.2625
Current parameters: [2.42413475 2.17729159 0.51293234 1.97590377], Cost: 0.1659
Current parameters: [2.40633044 2.16899568 0.56957641 1.99356398], Cost: 0.1427
Current parameters: [2.45928956 2.14373601 0.58718734 2.00594889], Cost: 0.1700
Current parameters: [2.41648449 2.19726948 0.57476966 2.0004255 ], Cost: 0.1879
Current parameters: [2.36650854 2.12479417 0.55983197 2.01004848], Cost: 0.0777
Current parameters: [2.33433219 2.08566727 0.56965932 1.97478601], Cost: 0.0439
Current parameters: [2.35823138 2.03037615 0.57484328 1.99063206], Cost: 0.0289
Current parameters: [2.30897949 1.99967277 0.57119394 2.01353371], Cost: 0.0094
Current parameters: [2.29379855 1.98482118 0.62996736 2.0125824 ], Cost: 0.0641
Current parameters: [2.30311102 1.98859932 0.56642309 1.98530711], Cost: 0.0131
Current parameters: [2.30973084 1.98547208 0.51462004 2.03597073], Cost: 0.0428
Current parameters: [2.29004186 2.02380738 0.56679895 2.00951714], Cost: 0.0052
Current parameters: [2.28186022 2.02375827 0.57992471 2.01173375], Cost: 0.0080
Current parameters: [2.2852169  2.01989675 0.56324692 2.0126548 ], Cost: 0.0038
Current parameters: [2.28213295 2.02422522 0.55036703 2.01972605], Cost: 0.0050
Current parameters: [2.27932857 2.01531291 0.55783351 2.00003927], Cost: 0.0049
Current parameters: [2.28881741 2.01334773 0.56247928 2.01479784], Cost: 0.0042
Current parameters: [2.27959196 2.01845507 0.5674989  2.01569411], Cost: 0.0034
Current parameters: [2.27644512 2.02058077 0.56296073 2.02079499], Cost: 0.0029
Current parameters: [2.27061926 2.018476   0.55932879 2.01771708], Cost: 0.0018
Current parameters: [2.26370696 2.01788478 0.55601148 2.01633823], Cost: 0.0014
Current parameters: [2.26187037 2.02120229 0.55325774 2.02258908], Cost: 0.0017
Current parameters: [2.26294337 2.01486589 0.55732701 2.01829563], Cost: 0.0012
Current parameters: [2.26535651 2.01036879 0.55141282 2.01819375], Cost: 0.0027
Current parameters: [2.25823089 2.01580072 0.56347218 2.01785906], Cost: 0.0008
Current parameters: [2.25289109 2.01100615 0.56640395 2.01689028], Cost: 0.0010
Current parameters: [2.26030892 2.01588747 0.56655229 2.01906155], Cost: 0.0013
Current parameters: [2.25765024 2.01587706 0.56314562 2.01969347], Cost: 0.0008
Current parameters: [2.25528457 2.01868893 0.56208472 2.01889958], Cost: 0.0005
Current parameters: [2.25440131 2.01853145 0.55832006 2.01836929], Cost: 0.0005
Current parameters: [2.25202751 2.01685734 0.56302143 2.01825291], Cost: 0.0004
```



```
Current parameters: [2.25056268 2.01811204 0.56332909 2.01824633], Cost: 0.0004
Current parameters: [2.24996669 2.01785375 0.56322701 2.02008545], Cost: 0.0004
Current parameters: [2.25019245 2.01788738 0.56416757 2.02021554], Cost: 0.0005
Current parameters: [2.24901593 2.01777965 0.56152915 2.01993528], Cost: 0.0003
Current parameters: [2.24778946 2.01770417 0.56001287 2.01985997], Cost: 0.0002
Current parameters: [2.24666624 2.01747712 0.55985149 2.02143334], Cost: 0.0002
Current parameters: [2.24537015 2.01888571 0.56012656 2.02115938], Cost: 0.0001
Current parameters: [2.24602118 2.02025596 0.55915889 2.02191892], Cost: 0.0001
Current parameters: [2.24503371 2.01866635 0.5593105  2.02080383], Cost: 0.0001
Current parameters: [2.24381047 2.01965598 0.56100226 2.02130741], Cost: 0.0001
Current parameters: [2.24400679 2.02060942 0.56161096 2.01972734], Cost: 0.0001
Current parameters: [2.24276918 2.01912276 0.56166894 2.0227222 ], Cost: 0.0001
Current parameters: [2.24322654 2.01953948 0.56028748 2.02101037], Cost: 0.0001
Current parameters: [2.24327126 2.02029215 0.55980486 2.02140056], Cost: 0.0001
Current parameters: [2.24316685 2.02005507 0.55975791 2.02181178], Cost: 0.0001
Current parameters: [2.24240509 2.02038765 0.55924802 2.0217588 ], Cost: 0.0001
Current parameters: [2.24256132 2.02029975 0.55829487 2.02164455], Cost: 0.0001
Current parameters: [2.24193985 2.02096543 0.55981624 2.02204256], Cost: 0.0000
Current parameters: [2.24127745 2.0205337  0.56025893 2.02167851], Cost: 0.0000
Current parameters: [2.24117579 2.02029539 0.56021263 2.02208979], Cost: 0.0000
Current parameters: [2.24071104 2.02128745 0.56036471 2.02190986], Cost: 0.0000
Current parameters: [2.24077109 2.02174505 0.56117217 2.02220766], Cost: 0.0001
Current parameters: [2.24020849 2.02138157 0.55955611 2.02171384], Cost: 0.0000
Current parameters: [2.23953604 2.02191264 0.55908835 2.02168863], Cost: 0.0000
Current parameters: [2.23950831 2.02190199 0.55911615 2.02071291], Cost: 0.0000
Current parameters: [2.23988801 2.02219794 0.55890723 2.02167035], Cost: 0.0000
Current parameters: [2.23937909 2.02140916 0.55862735 2.0223691 ], Cost: 0.0000
Current parameters: [2.2394251  2.02210778 0.55942443 2.02196264], Cost: 0.0000
Current parameters: [2.23919953 2.02223549 0.55981517 2.02209883], Cost: 0.0000
Current parameters: [2.23915172 2.02212044 0.55970517 2.02255796], Cost: 0.0000
Current parameters: [2.23901027 2.0222721  0.55957657 2.02255043], Cost: 0.0000
Current parameters: [2.23925433 2.02265673 0.55958598 2.02272599], Cost: 0.0000
Current parameters: [2.23855254 2.02229781 0.55947034 2.02268064], Cost: 0.0000
Current parameters: [2.23848161 2.02220886 0.55899551 2.02267675], Cost: 0.0000
Current parameters: [2.23853075 2.02237247 0.5594615  2.02244939], Cost: 0.0000
Current parameters: [2.2381792  2.02239749 0.55974638 2.02279421], Cost: 0.0000
Current parameters: [2.23856835 2.02208669 0.55957758 2.02262336], Cost: 0.0000
Current parameters: [2.23869911 2.02246039 0.55955446 2.02274853], Cost: 0.0000
Current parameters: [2.23851222 2.02231126 0.55938449 2.02270935], Cost: 0.0000
Current parameters: [2.23847625 2.02225164 0.55931665 2.02273277], Cost: 0.0000
Current parameters: [2.23855654 2.02230862 0.55936187 2.02270519], Cost: 0.0000
```

```
Current parameters: [2.23846086 2.02238318 0.55939414 2.02275514], Cost: 0.0000
Current parameters: [2.23840168 2.02243879 0.55938007 2.02269851], Cost: 0.0000
Current parameters: [2.23832757 2.02249346 0.55941846 2.02270517], Cost: 0.0000
Current parameters: [2.23828163 2.02258172 0.55941745 2.0227151 ], Cost: 0.0000
Current parameters: [2.23824445 2.02264091 0.55947719 2.02267578], Cost: 0.0000
Current parameters: [2.23822051 2.02263183 0.55944961 2.02264286], Cost: 0.0000
Current parameters: [2.23813565 2.02263705 0.5594266  2.0226902 ], Cost: 0.0000
Current parameters: [2.2380675  2.02267517 0.55937553 2.02272619], Cost: 0.0000
Current parameters: [2.2379768  2.02267053 0.55935288 2.02276139], Cost: 0.0000
Current parameters: [2.23791103 2.02268359 0.55928484 2.02273183], Cost: 0.0000
Current parameters: [2.23795991 2.02270018 0.55938354 2.0227415 ], Cost: 0.0000
Current parameters: [2.23792313 2.02274516 0.55939312 2.02282232], Cost: 0.0000
Current parameters: [2.2378516  2.02271575 0.55943763 2.02286746], Cost: 0.0000
Current parameters: [2.23783273 2.0227895  0.55944301 2.02293209], Cost: 0.0000
Current parameters: [2.23786558 2.02278316 0.55947745 2.02294605], Cost: 0.0000
Current parameters: [2.23776931 2.02283195 0.55944858 2.02286771], Cost: 0.0000
Current parameters: [2.23770806 2.02282703 0.55936969 2.02286672], Cost: 0.0000
Current parameters: [2.23776931 2.02283195 0.55944858 2.02286771], Cost: 0.0000

   Return from subroutine COBYLA because the MAXFUN limit has been reached.

   NFVALS =  100   F = 1.029381E-06    MAXCV = 0.000000E+00
   X = 2.237769E+00   2.022832E+00   5.594486E-01   2.022868E+00
Simulating for QAOA depth p = 3
Current parameters: [1.37969433 0.59712819 2.07193866 0.19063118 0.49886425 2.14296912], Cost: 0.3619
Current parameters: [1.37969433 1.59712819 2.07193866 0.19063118 0.49886425 2.14296912], Cost: 1.2818
Current parameters: [1.37969433 0.59712819 3.07193866 0.19063118 0.49886425 2.14296912], Cost: 3.1733
Current parameters: [1.37969433 0.59712819 2.07193866 1.19063118 0.49886425 2.14296912], Cost: 2.2493
Current parameters: [1.37969433 0.59712819 2.07193866 0.19063118 1.49886425 2.14296912], Cost: 2.0045
Current parameters: [1.37969433 0.59712819 2.07193866 0.19063118 0.49886425 3.14296912], Cost: 2.4084
Current parameters: [ 1.62841075  0.39377679  1.45047011 -0.22658365  0.13575323  1.69058944], Cost: 2.0726
Current parameters: [ 1.50405254  0.49545249  1.76120439 -0.01797623  0.31730874  1.91677928], Cost: 1.3597
Current parameters: [1.41295343 0.56993544 2.29892886 0.13483998 0.45030798 2.08247557], Cost: 0.4217
Current parameters: [1.30057314 0.50035611 2.07193866 0.19063118 0.49886425 2.14296912], Cost: 0.4823
Current parameters: [1.20562138 0.76416596 2.08520052 0.15314757 0.46624147 2.10232621], Cost: 0.2110
Current parameters: [1.20562138 0.76416596 2.08520052 0.06126005 0.46624147 2.18707103], Cost: 0.0209
Current parameters: [1.19616525 0.84850206 2.12630577 0.11861807 0.36512681 2.38730651], Cost: 0.1738
Current parameters: [1.1886563  0.77803667 2.12759122 0.0897185  0.57387871 2.21792809], Cost: 0.3260
Current parameters: [ 1.2536684   0.81333424  1.98532532 -0.12890656  0.36905597  2.14026774], Cost: 1.1002
Current parameters: [1.11910352 0.6786033  2.08479951 0.06188361 0.44169937 2.2017621 ], Cost: 0.0821
Current parameters: [1.21397667 0.7550436  2.0308675  0.07778688 0.48062512 2.20499082], Cost: 0.0407
```



```
Current parameters: [1.29293212 0.68512925 2.11988464 0.07000963 0.45619598 2.20642501], Cost: 0.0297
Current parameters: [1.21966539 0.76898099 2.06707661 0.01178104 0.43847456 2.17531598], Cost: 0.1033
Current parameters: [1.20628571 0.78538554 2.09268274 0.0835164  0.42738337 2.2244108 ], Cost: 0.0132
Current parameters: [1.20448827 0.79181229 2.1054601  0.08002982 0.44742182 2.24325416], Cost: 0.0311
Current parameters: [1.21636028 0.79534888 2.09272944 0.0868851  0.43024117 2.21952629], Cost: 0.0163
Current parameters: [1.19507027 0.77849682 2.10070621 0.10785803 0.42444232 2.21267277], Cost: 0.0236
Current parameters: [1.19558027 0.79531853 2.08819138 0.0817243  0.42823462 2.22181054], Cost: 0.0119
Current parameters: [1.19560912 0.79529497 2.08559352 0.06288137 0.40431249 2.21529226], Cost: 0.0399
Current parameters: [1.19841894 0.7862815  2.07821896 0.08295393 0.42643081 2.22889572], Cost: 0.0090
Current parameters: [1.19497943 0.80070066 2.05704262 0.09582571 0.43803816 2.22607266], Cost: 0.0073
Current parameters: [1.19507147 0.80791291 2.05890922 0.10117353 0.42864084 2.23454157], Cost: 0.0069
Current parameters: [1.19432025 0.8107056  2.0396393  0.08708358 0.41020667 2.22689149], Cost: 0.0101
Current parameters: [1.19183151 0.80853836 2.05636182 0.09572685 0.4354229  2.24683385], Cost: 0.0122
Current parameters: [1.18786023 0.80571511 2.05897621 0.10279729 0.42745779 2.23413878], Cost: 0.0061
Current parameters: [1.19075814 0.79854043 2.05778144 0.11538266 0.42431853 2.23031873], Cost: 0.0097
Current parameters: [1.18658984 0.81061177 2.05526832 0.10577522 0.43057969 2.23238343], Cost: 0.0070
Current parameters: [1.18443505 0.81117652 2.07063646 0.09965828 0.42494948 2.22703418], Cost: 0.0075
Current parameters: [1.18733696 0.80592013 2.05469497 0.09921342 0.4224965  2.23191801], Cost: 0.0036
Current parameters: [1.18477951 0.80530153 2.05125475 0.09388156 0.41947015 2.23408349], Cost: 0.0036
Current parameters: [1.18493806 0.80692299 2.05168586 0.09551315 0.41707328 2.23608639], Cost: 0.0034
Current parameters: [1.1861492  0.80353779 2.04942093 0.097901   0.41217611 2.23244019], Cost: 0.0035
Current parameters: [1.18503118 0.80643136 2.0548896  0.09396204 0.41570186 2.23540981], Cost: 0.0041
Current parameters: [1.18257144 0.8127985  2.04970059 0.09569395 0.41666439 2.2319911 ], Cost: 0.0035
Current parameters: [1.18851585 0.80805629 2.05125439 0.09456027 0.41711731 2.23580748], Cost: 0.0038
Current parameters: [1.18171365 0.80607966 2.04604316 0.09474042 0.41653886 2.24023419], Cost: 0.0032
Current parameters: [1.18121275 0.80588412 2.045636   0.09814192 0.41825134 2.24078295], Cost: 0.0026
Current parameters: [1.17957257 0.80547944 2.0458684  0.10133692 0.41934861 2.24175184], Cost: 0.0025
Current parameters: [1.18044532 0.80417184 2.04634349 0.10124169 0.41942012 2.24280222], Cost: 0.0027
Current parameters: [1.18166684 0.80732377 2.04368106 0.10253236 0.42046379 2.24163308], Cost: 0.0026
Current parameters: [1.17926616 0.80602175 2.0463307  0.10053215 0.42085467 2.24229695], Cost: 0.0030
Current parameters: [1.17914096 0.80319893 2.04439613 0.10128817 0.42058467 2.23926709], Cost: 0.0025
Current parameters: [1.17989986 0.80339137 2.0457682  0.1018001  0.42087874 2.2382819 ], Cost: 0.0026
Current parameters: [1.17823608 0.803479   2.04353212 0.10254628 0.41734675 2.23802289], Cost: 0.0019
Current parameters: [1.17648083 0.80432686 2.04266866 0.10341483 0.4145252  2.2366094 ], Cost: 0.0017
Current parameters: [1.17811714 0.80212012 2.04225368 0.10464914 0.41220488 2.23740436], Cost: 0.0016
Current parameters: [1.17701961 0.80109584 2.0431793  0.10390127 0.41193293 2.23767073], Cost: 0.0017
Current parameters: [1.17733564 0.80148632 2.03854201 0.10434242 0.4122736  2.23801352], Cost: 0.0016
Current parameters: [1.17655029 0.8012727  2.03870222 0.10592693 0.41289943 2.23848712], Cost: 0.0015
Current parameters: [1.17697364 0.80052855 2.03750013 0.10815464 0.41288905 2.23563792], Cost: 0.0017
Current parameters: [1.17657119 0.80346237 2.03812552 0.10712203 0.41125563 2.24093676], Cost: 0.0012
Current parameters: [1.17831562 0.80411013 2.03755093 0.10794269 0.41211124 2.24410859], Cost: 0.0011
```



```
Current parameters: [1.1780921  0.80556043 2.03731069 0.10773737 0.41326688 2.2436332 ], Cost: 0.0012
Current parameters: [1.17576763 0.80456265 2.03767976 0.10816406 0.40990038 2.24600813], Cost: 0.0010
Current parameters: [1.17608369 0.80530135 2.03663032 0.1107102  0.40728643 2.24556506], Cost: 0.0007
Current parameters: [1.17698714 0.80585384 2.03450955 0.10836456 0.40525943 2.245735  ], Cost: 0.0007
Current parameters: [1.17769166 0.80664407 2.03598922 0.1080177  0.40464009 2.24571101], Cost: 0.0008
Current parameters: [1.17549023 0.80461356 2.03231793 0.11010132 0.40349937 2.24648492], Cost: 0.0006
Current parameters: [1.1749349  0.80362263 2.03071148 0.10701797 0.4028519  2.24769385], Cost: 0.0010
Current parameters: [1.17635796 0.80496544 2.03202604 0.1105076  0.40394945 2.24806134], Cost: 0.0004
Current parameters: [1.17530082 0.80809718 2.03068274 0.11182243 0.40484383 2.24807997], Cost: 0.0004
Current parameters: [1.17667492 0.80829427 2.02970381 0.11179271 0.40436874 2.24724166], Cost: 0.0003
Current parameters: [1.17774867 0.80738497 2.02943699 0.11497851 0.40610519 2.24744964], Cost: 0.0005
Current parameters: [1.17702459 0.80906387 2.03046665 0.11235085 0.40297099 2.24774465], Cost: 0.0003
Current parameters: [1.17664127 0.80871236 2.02985476 0.11255753 0.40252064 2.24799613], Cost: 0.0003
Current parameters: [1.17685454 0.80930892 2.02894073 0.11155147 0.40243915 2.24924476], Cost: 0.0003
Current parameters: [1.17643706 0.81003928 2.02865188 0.11181331 0.40258716 2.2489763 ], Cost: 0.0003
Current parameters: [1.17551189 0.81024896 2.02807189 0.11099339 0.40149943 2.24812601], Cost: 0.0004
Current parameters: [1.17691934 0.81009614 2.02799882 0.11327809 0.40323239 2.24974473], Cost: 0.0002
Current parameters: [1.17562846 0.81001576 2.02874896 0.11353907 0.40377182 2.25084929], Cost: 0.0003
Current parameters: [1.17744946 0.81033393 2.02652087 0.11440081 0.40307816 2.24965251], Cost: 0.0002
Current parameters: [1.17873341 0.81156796 2.0270283  0.11491697 0.40338604 2.24980946], Cost: 0.0002
Current parameters: [1.17755704 0.81053744 2.02605857 0.11377816 0.40339083 2.25010141], Cost: 0.0002
Current parameters: [1.17747583 0.80972986 2.02620741 0.11453631 0.40463137 2.24869333], Cost: 0.0002
Current parameters: [1.17803017 0.80964534 2.02671619 0.11433811 0.40303942 2.24996671], Cost: 0.0002
Current parameters: [1.17694115 0.81016985 2.02533807 0.11492687 0.40183066 2.25019784], Cost: 0.0001
Current parameters: [1.17699196 0.81019443 2.02504385 0.11389124 0.40041121 2.24939936], Cost: 0.0002
Current parameters: [1.1762554  0.8103309  2.02600221 0.1149029  0.40173408 2.25011722], Cost: 0.0001
Current parameters: [1.17656813 0.80978624 2.0248009  0.11537198 0.40189717 2.25194063], Cost: 0.0001
Current parameters: [1.1765884  0.80991714 2.02489634 0.11538196 0.40225125 2.24976627], Cost: 0.0001
Current parameters: [1.17686444 0.81061553 2.02515687 0.1149406  0.40185105 2.25021938], Cost: 0.0001
Current parameters: [1.17735887 0.81090268 2.02544292 0.11566133 0.40169846 2.25026625], Cost: 0.0001
Current parameters: [1.17716599 0.81083862 2.02531036 0.11453332 0.40261698 2.25041145], Cost: 0.0001
Current parameters: [1.17673384 0.81055888 2.02500639 0.11500365 0.40178519 2.25065205], Cost: 0.0001
Current parameters: [1.17675214 0.81061108 2.02474581 0.11480205 0.40143452 2.25071412], Cost: 0.0001
Current parameters: [1.17656846 0.81055955 2.024312   0.11489176 0.40137581 2.25066575], Cost: 0.0001
Current parameters: [1.17619249 0.81067196 2.02430607 0.11480604 0.40110848 2.25074046], Cost: 0.0001
Current parameters: [1.17651476 0.81053549 2.02427792 0.11469918 0.40150849 2.2506828 ], Cost: 0.0001
Current parameters: [1.17678096 0.81069038 2.02406186 0.11505497 0.40113833 2.25084051], Cost: 0.0001
Current parameters: [1.17691536 0.81039742 2.02393189 0.11515923 0.40082623 2.25093726], Cost: 0.0001
Current parameters: [1.17709304 0.81059181 2.0237397  0.11522895 0.40057349 2.2506855 ], Cost: 0.0001
Current parameters: [1.17680819 0.8103517  2.02377981 0.11527566 0.40091056 2.25089   ], Cost: 0.0001
Current parameters: [1.17684894 0.81044867 2.02372512 0.11561329 0.40088379 2.25122113], Cost: 0.0001
```



```
Current parameters: [1.17692489 0.81048563 2.0233913  0.11553197 0.40092326 2.25155532], Cost: 0.0001
Current parameters: [1.17684894 0.81044867 2.02372512 0.11561329 0.40088379 2.25122113], Cost: 0.0001

   Return from subroutine COBYLA because the MAXFUN limit has been reached.

   NFVALS =  100   F = 6.896690E-05    MAXCV = 0.000000E+00
   X = 1.176849E+00   8.104487E-01   2.023725E+00   1.156133E-01   4.008838E-01
       2.251221E+00
Simulating for QAOA depth p = 4
Current parameters: [4.00549337 0.41937755 2.036414   1.08864573 2.64927478 0.31023492
 2.71837352 1.87699587], Cost: 3.1888
Current parameters: [3.00549337 1.41937755 2.036414   1.08864573 2.64927478 0.31023492
 2.71837352 1.87699587], Cost: 1.5823
Current parameters: [3.00549337 1.41937755 3.036414   1.08864573 2.64927478 0.31023492
 2.71837352 1.87699587], Cost: 0.8123
Current parameters: [3.00549337 1.41937755 3.036414   2.08864573 2.64927478 0.31023492
 2.71837352 1.87699587], Cost: 2.6335
Current parameters: [3.00549337 1.41937755 3.036414   1.08864573 3.64927478 0.31023492
 2.71837352 1.87699587], Cost: 0.6055
Current parameters: [3.00549337 1.41937755 3.036414   1.08864573 3.64927478 1.31023492
 2.71837352 1.87699587], Cost: 2.5877
Current parameters: [3.00549337 1.41937755 3.036414   1.08864573 3.64927478 0.31023492
 3.71837352 1.87699587], Cost: 2.3885
Current parameters: [3.00549337 1.41937755 3.036414   1.08864573 3.64927478 0.31023492
 2.71837352 2.87699587], Cost: 2.1250
Current parameters: [ 2.89834839  1.72754208  3.23549666  0.61781964  3.70272542 -0.20221149
  2.25742465  1.48417107], Cost: 0.7599
Current parameters: [2.97800602 1.49843516 3.08748736 1.53448764 3.66298719 0.17877011
 2.60012007 1.77621921], Cost: 1.6487
Current parameters: [3.24162771 1.50147852 3.036414   1.08864573 3.64927478 0.31023492
 2.71837352 1.87699587], Cost: 0.7536
Current parameters: [3.13707506 0.96313529 3.06962324 1.01226779 3.65819095 0.22475306
 2.64148204 1.81146825], Cost: 0.8692
Current parameters: [2.98606115 1.42620253 2.54625366 1.04047407 3.65489822 0.25632141
 2.66987797 1.83566752], Cost: 0.5478
Current parameters: [2.9553707  1.43698164 2.53978533 0.96439371 3.66377965 0.46940865
 2.59328607 1.77039521], Cost: 0.4492
Current parameters: [2.9553707  1.43698164 2.53978533 0.96439371 3.66377965 0.46940865
 2.67436407 1.67525658], Cost: 0.7119
Current parameters: [2.90058696 1.45622279 2.5282391  0.82858706 3.67963338 0.50104789
 2.57661869 1.967934  ], Cost: 1.0129
```

```
Current parameters: [2.95535138 1.43703722 2.53982124 0.97878705 3.78794811 0.46931622
 2.59320292 1.77032435], Cost: 0.3910
Current parameters: [2.92451046 1.44875305 2.53404542 1.16390643 3.78635973 0.48525135
 2.43064591 1.74964102], Cost: 0.1117
Current parameters: [2.99152971 1.22714918 2.54169261 1.13875143 3.79678646 0.46596734
 2.3470195  1.7225315 ], Cost: 0.0507
Current parameters: [2.94879252 1.25407143 2.56577351 1.08180065 3.80325181 0.4039822
 2.29126347 1.6750157 ], Cost: 0.2443
Current parameters: [3.21491056 1.31579266 2.53856585 1.13696215 3.80541472 0.47446819
 2.27972493 1.71511019], Cost: 0.1643
Current parameters: [2.98623014 1.22901049 2.41918334 1.12561401 3.79832009 0.45047799
 2.33709402 1.71407291], Cost: 0.1208
Current parameters: [2.9543904  1.17378119 2.56052624 1.22754014 3.82654463 0.57073123
 2.29048142 1.90955238], Cost: 0.1618
Current parameters: [2.99496251 1.20032436 2.54845987 1.21702811 3.80312656 0.50378081
 2.38988071 1.64895521], Cost: 0.0876
Current parameters: [3.00607951 1.24003714 2.54052949 1.11582496 3.79945313 0.51379463
 2.32582573 1.70647718], Cost: 0.0170
Current parameters: [3.1180578  1.27400479 2.54691771 1.1453739  3.80443919 0.49239841
 2.30587161 1.71819404], Cost: 0.0689
Current parameters: [3.00303782 1.24116969 2.53994107 1.12729816 3.7402945  0.5154149
 2.30978465 1.7044648 ], Cost: 0.0179
Current parameters: [3.03748806 1.21101838 2.55073349 1.05063002 3.76330375 0.54773237
 2.40679281 1.72715801], Cost: 0.1715
Current parameters: [2.97728823 1.22337086 2.54605474 1.16329511 3.8230556  0.57335871
 2.27530501 1.78115686], Cost: 0.0261
Current parameters: [3.00313897 1.23080233 2.54232014 1.15477786 3.80606933 0.53499504
 2.33708285 1.66558913], Cost: 0.0666
Current parameters: [3.0126918  1.21372188 2.54241079 1.11050281 3.80099217 0.5090015
 2.31333022 1.70128083], Cost: 0.0306
Current parameters: [2.9818232  1.27213033 2.56279902 1.0955268  3.8065406  0.50193942
 2.29256759 1.69736242], Cost: 0.0494
Current parameters: [3.00073191 1.24798968 2.4804089  1.1094684  3.80261319 0.51127916
 2.31386214 1.7061993 ], Cost: 0.0195
Current parameters: [3.00774166 1.24001745 2.53894401 1.09734809 3.79186988 0.52742256
 2.34112042 1.71883586], Cost: 0.0187
Current parameters: [3.01394702 1.24356193 2.54009393 1.110799   3.79701318 0.50730174
 2.3304332  1.69781525], Cost: 0.0198
Current parameters: [3.0286256  1.24926598 2.54075279 1.12393977 3.80184949 0.51680966
 2.3180807  1.72204466], Cost: 0.0208
Current parameters: [3.00568786 1.24116569 2.53909004 1.1235261  3.80048622 0.52449559
```



```
 2.33029386 1.69971019], Cost: 0.0205
Current parameters: [3.00639224 1.23995128 2.54066521 1.11434465 3.80686441 0.51352957
 2.32774134 1.70670838], Cost: 0.0175
Current parameters: [2.99958015 1.24958871 2.54490582 1.11041211 3.79987327 0.51571083
 2.31822248 1.70573043], Cost: 0.0171
Current parameters: [3.00635967 1.23936004 2.54788183 1.11709574 3.79899057 0.51338882
 2.32789505 1.70688427], Cost: 0.0179
Current parameters: [2.99797931 1.24420242 2.53616611 1.12001471 3.79787861 0.50603208
 2.33017648 1.71302848], Cost: 0.0141
Current parameters: [2.98985679 1.23679323 2.53185687 1.11879643 3.79614676 0.49981903
 2.32480281 1.71875019], Cost: 0.0217
Current parameters: [2.99976559 1.24509822 2.53654815 1.12497832 3.79982534 0.5031009
 2.32597705 1.71153123], Cost: 0.0172
Current parameters: [3.00520475 1.25066647 2.53461102 1.11891703 3.7961817  0.50990451
 2.3319663  1.72422883], Cost: 0.0131
Current parameters: [3.00105052 1.25193973 2.53361064 1.11953564 3.79539193 0.51262907
 2.33700904 1.72152759], Cost: 0.0097
Current parameters: [2.99981384 1.2579301  2.52954401 1.12622453 3.79452343 0.52366963
 2.34174813 1.72094388], Cost: 0.0076
Current parameters: [3.00249468 1.26560397 2.52646784 1.1212581  3.79023205 0.52015179
 2.35063355 1.7150962 ], Cost: 0.0099
Current parameters: [3.00291231 1.25293292 2.52736867 1.12884459 3.79390424 0.52273524
 2.34541815 1.72131874], Cost: 0.0092
Current parameters: [2.98758877 1.255269   2.52779946 1.12113149 3.79228289 0.5285852
 2.34389926 1.72592526], Cost: 0.0059
Current parameters: [2.98569881 1.25651359 2.54142087 1.12453582 3.79081507 0.52952424
 2.34957354 1.72850474], Cost: 0.0064
Current parameters: [2.98549669 1.25781467 2.52602312 1.12384704 3.7954922  0.52449064
 2.34530662 1.72918142], Cost: 0.0039
Current parameters: [2.98643697 1.25708973 2.52706008 1.11871268 3.80894796 0.52857772
 2.34932324 1.72799865], Cost: 0.0072
Current parameters: [2.98166987 1.25582265 2.52665892 1.12555505 3.79648274 0.5222244
 2.34515914 1.72344015], Cost: 0.0040
Current parameters: [2.97860315 1.26515843 2.522203   1.13291818 3.79439105 0.52707611
 2.34030692 1.73277533], Cost: 0.0013
Current parameters: [2.9727008  1.26666094 2.52290351 1.13135178 3.79296322 0.51746604
 2.33264347 1.73991539], Cost: 0.0068
Current parameters: [2.9793644  1.26931373 2.52045432 1.13045262 3.79199249 0.52450061
 2.34494303 1.73319071], Cost: 0.0012
Current parameters: [2.97320611 1.27500366 2.5177055  1.13487637 3.79053747 0.5313704
 2.3466805  1.7234755 ], Cost: 0.0033
```



```
Current parameters: [2.97993221 1.26565412 2.51747828 1.13465104 3.79096543 0.524631
 2.34872639 1.73552539], Cost: 0.0017
Current parameters: [2.98066678 1.26920525 2.51735211 1.13000301 3.79249662 0.52393454
 2.34344946 1.73223174], Cost: 0.0014
Current parameters: [2.9842408  1.27191555 2.52303155 1.13470046 3.79277935 0.52258859
 2.34458715 1.73200812], Cost: 0.0014
Current parameters: [2.97850618 1.26994476 2.52009471 1.1305913  3.79552925 0.52401448
 2.34598121 1.73358142], Cost: 0.0011
Current parameters: [2.98217058 1.27116619 2.5205624  1.12806159 3.79682566 0.52935706
 2.34677112 1.73652241], Cost: 0.0018
Current parameters: [2.97961029 1.26807699 2.5208576  1.12936774 3.79604669 0.52398626
 2.3475762  1.73120244], Cost: 0.0016
Current parameters: [2.97578017 1.27117458 2.52192899 1.12962312 3.79546959 0.51926149
 2.34332746 1.73784203], Cost: 0.0020
Current parameters: [2.97646231 1.27202235 2.51964505 1.13196273 3.7951781  0.52550781
 2.34603959 1.73205655], Cost: 0.0009
Current parameters: [2.97606142 1.27093771 2.51816335 1.13451808 3.7945905  0.52577364
 2.34777655 1.73338448], Cost: 0.0009
Current parameters: [2.9748289  1.27027402 2.51759322 1.13340792 3.79439042 0.52614961
 2.34784374 1.73371874], Cost: 0.0009
Current parameters: [2.97392976 1.27280761 2.51883209 1.13553834 3.79435508 0.5241321
 2.34668955 1.73472558], Cost: 0.0004
Current parameters: [2.97302351 1.27543432 2.51932438 1.13600738 3.79449193 0.52506488
 2.34596007 1.73710353], Cost: 0.0003
Current parameters: [2.97040818 1.27690611 2.520454   1.13719837 3.79437457 0.52319329
 2.34591737 1.73690436], Cost: 0.0003
Current parameters: [2.97340202 1.27640798 2.51843818 1.13535186 3.79399672 0.52436554
 2.34683866 1.73705736], Cost: 0.0003
Current parameters: [2.97303865 1.27572285 2.5167712  1.13674929 3.79511401 0.52453439
 2.343234   1.73702424], Cost: 0.0004
Current parameters: [2.97297883 1.27559168 2.51939377 1.13624013 3.7927755  0.52561383
 2.34549121 1.73659201], Cost: 0.0002
Current parameters: [2.9739541  1.27452163 2.52045898 1.13572831 3.79141171 0.52390602
 2.34461202 1.7390853 ], Cost: 0.0007
Current parameters: [2.97278772 1.27504775 2.51865359 1.13751973 3.79248298 0.52574894
 2.34636182 1.73725176], Cost: 0.0002
Current parameters: [2.97494255 1.27670505 2.51971768 1.13960118 3.79316404 0.52652843
 2.34671354 1.73615223], Cost: 0.0002
Current parameters: [2.97418445 1.2770989  2.5195767  1.13933721 3.79341374 0.52815075
 2.34717775 1.73585973], Cost: 0.0003
Current parameters: [2.97503012 1.27709428 2.51988468 1.13955219 3.7930654  0.526705
```



```
 2.34650464 1.73697665], Cost: 0.0002
Current parameters: [2.97596216 1.27637903 2.52096021 1.139594   3.79227313 0.52642213
 2.34644068 1.73772771], Cost: 0.0002
Current parameters: [2.97559973 1.27681866 2.51933129 1.13937744 3.79316029 0.52704056
 2.34619845 1.73700989], Cost: 0.0002
Current parameters: [2.97447018 1.27775086 2.51965326 1.14048119 3.79300429 0.52597577
 2.3452485  1.73717684], Cost: 0.0002
Current parameters: [2.97479515 1.27896478 2.51897398 1.14012763 3.79233545 0.52509505
 2.34588899 1.73730262], Cost: 0.0002
Current parameters: [2.97437831 1.27747101 2.5192818  1.14110058 3.79297017 0.52600728
 2.34571046 1.73753701], Cost: 0.0001
Current parameters: [2.97438407 1.27758114 2.51946923 1.14107049 3.79461634 0.52524387
 2.34590125 1.73819838], Cost: 0.0002
Current parameters: [2.97380401 1.27711814 2.51907763 1.14057434 3.79279445 0.5258232
 2.34566083 1.7378739 ], Cost: 0.0002
Current parameters: [2.97485493 1.27691706 2.51861672 1.14209564 3.79259138 0.52548727
 2.34466896 1.73694573], Cost: 0.0002
Current parameters: [2.97397408 1.27795273 2.51944917 1.1414394  3.79286949 0.526634
 2.34576677 1.73763356], Cost: 0.0001
Current parameters: [2.9740546  1.27798002 2.52017293 1.1416954  3.7924376  0.52667459
 2.34588827 1.73802616], Cost: 0.0001
Current parameters: [2.97373176 1.27792524 2.51958727 1.14153108 3.7928688  0.52644281
 2.34593326 1.73733828], Cost: 0.0001
Current parameters: [2.97366661 1.27805016 2.51954469 1.14157022 3.79289092 0.52722874
 2.3464289  1.73708015], Cost: 0.0001
Current parameters: [2.97360456 1.27772505 2.51966515 1.14168822 3.79302827 0.52739418
 2.34620396 1.73702006], Cost: 0.0001
Current parameters: [2.97339416 1.27854722 2.51926483 1.14216065 3.79266098 0.52732439
 2.34606295 1.7369827 ], Cost: 0.0001
Current parameters: [2.97311948 1.27902165 2.51938248 1.14242901 3.7933479  0.52730549
 2.34609687 1.73728917], Cost: 0.0001
Current parameters: [2.97230002 1.27905104 2.51919321 1.14220769 3.79312361 0.52742571
 2.34602599 1.73764512], Cost: 0.0000
Current parameters: [2.9721592  1.27956055 2.51954871 1.14175977 3.79310511 0.52759078
 2.3455512  1.73733818], Cost: 0.0000
Current parameters: [2.97227467 1.27902111 2.51883424 1.14207107 3.79333785 0.52738428
 2.34596476 1.7374502 ], Cost: 0.0000
Current parameters: [2.97174435 1.27936011 2.5187819  1.14275039 3.79298079 0.52744949
 2.34603159 1.73789946], Cost: 0.0000
Current parameters: [2.97132543 1.27955573 2.51885569 1.14276062 3.79360533 0.52738706
 2.34634338 1.7383926 ], Cost: 0.0000
```

```
Current parameters: [2.97108066 1.27939068 2.5190327  1.14289309 3.79366259 0.52727017
 2.34639008 1.73810406], Cost: 0.0000
Current parameters: [2.9712269  1.27913665 2.51884158 1.14305217 3.79383828 0.52788296
 2.34577031 1.73862616], Cost: 0.0000

   Return from subroutine COBYLA because the MAXFUN limit has been reached.

   NFVALS =  100   F = 1.900725E-05    MAXCV = 0.000000E+00
   X = 2.971325E+00   1.279556E+00   2.518856E+00   1.142761E+00   3.793605E+00
       5.273871E-01   2.346343E+00   1.738393E+00
Current parameters: [2.97132543 1.27955573 2.51885569 1.14276062 3.79360533 0.52738706
 2.34634338 1.7383926 ], Cost: 0.0000
Simulating for QAOA depth p = 5
Current parameters: [2.60212382 3.11308361 1.43127222 1.82218451 2.25520648 2.65326332
 0.85133934 2.67663964 2.07431373 0.58258441], Cost: 2.2883
Current parameters: [2.60212382 4.11308361 1.43127222 1.82218451 2.25520648 2.65326332
 0.85133934 2.67663964 2.07431373 0.58258441], Cost: 2.4893
Current parameters: [2.60212382 3.11308361 2.43127222 1.82218451 2.25520648 2.65326332
 0.85133934 2.67663964 2.07431373 0.58258441], Cost: 1.0064
Current parameters: [2.60212382 3.11308361 2.43127222 2.82218451 2.25520648 2.65326332
 0.85133934 2.67663964 2.07431373 0.58258441], Cost: 1.0184
Current parameters: [2.60212382 3.11308361 2.43127222 1.82218451 3.25520648 2.65326332
 0.85133934 2.67663964 2.07431373 0.58258441], Cost: 3.4078
Current parameters: [2.60212382 3.11308361 2.43127222 1.82218451 2.25520648 3.65326332
 0.85133934 2.67663964 2.07431373 0.58258441], Cost: 1.8694
Current parameters: [2.60212382 3.11308361 2.43127222 1.82218451 2.25520648 2.65326332
 1.85133934 2.67663964 2.07431373 0.58258441], Cost: 1.9118
Current parameters: [2.60212382 3.11308361 2.43127222 1.82218451 2.25520648 2.65326332
 0.85133934 3.67663964 2.07431373 0.58258441], Cost: 1.5546
Current parameters: [2.60212382 3.11308361 2.43127222 1.82218451 2.25520648 2.65326332
 0.85133934 2.67663964 3.07431373 0.58258441], Cost: 1.8546
Current parameters: [2.60212382 3.11308361 2.43127222 1.82218451 2.25520648 2.65326332
 0.85133934 2.67663964 2.07431373 1.58258441], Cost: 3.1493
Current parameters: [2.61353457 3.06054227 2.76642746 1.81903768 1.62735455 2.42763795
 0.61461218 2.53330325 1.85254228 0.02231861], Cost: 1.5792
Current parameters: [2.60782919 3.08681294 2.59884984 1.8206111  1.94128052 2.54045064
 0.73297576 2.60497144 1.963428   0.30245151], Cost: 1.3238
Current parameters: [2.85208254 3.11308361 2.43127222 1.82218451 2.2597493  2.65326332
 0.85133934 2.67663964 2.07431373 0.58258441], Cost: 0.9065
Current parameters: [2.88024281 3.09506865 2.54618759 1.82110555 2.68165388 2.57590269
 0.77017221 2.6274936  1.9982745  0.39048492], Cost: 2.4436
```



```
Current parameters: [2.85208254 2.86610011 2.39255332 1.82218451 2.2597493  2.65326332
 0.85133934 2.67663964 2.07431373 0.58258441], Cost: 0.5976
Current parameters: [2.87054255 2.88829708 1.91438795 1.82167678 2.17169665 2.61685952
 0.8131443  2.65351285 2.03853174 0.49218762], Cost: 1.4286
Current parameters: [2.85208254 2.86610011 2.39255332 1.57220882 2.2597493  2.65674977
 0.85133934 2.67663964 2.07431373 0.58258441], Cost: 0.5730
Current parameters: [2.86379509 2.82738901 2.42593719 1.57639975 2.20388145 3.14614058
 0.82710527 2.6619661  2.05161069 0.52522925], Cost: 0.7597
Current parameters: [2.85208254 2.86610011 2.39255332 1.57220882 2.2597493  2.65674977
 0.98082581 2.46278629 2.07431373 0.58258441], Cost: 0.4571
Current parameters: [2.86708834 2.8165045  2.43532382 1.56868829 2.18817294 2.64634983
 1.40262492 2.69960259 2.04522722 0.50910255], Cost: 1.3527
Current parameters: [2.85208254 2.86610011 2.39255332 1.57220882 2.2597493  2.65674977
 0.98082581 2.46278629 2.30676544 0.49057245], Cost: 1.2182
Current parameters: [2.88477285 2.75805551 2.48572935 1.5645393  2.10381939 2.63409338
 0.89328047 2.36930233 1.99836911 1.00863445], Cost: 0.7443
Current parameters: [2.85846612 2.841775   2.47660643 1.57060894 2.10451262 2.60494631
 0.91886429 2.42043977 1.99493063 0.45576211], Cost: 0.3288
Current parameters: [2.85727945 2.82528744 2.58177873 1.57058549 2.16980649 2.60326477
 0.91710002 2.41937152 1.99327782 0.45158657], Cost: 0.3024
Current parameters: [2.87423535 2.77710102 2.51006567 1.56557735 2.28169565 2.57952444
 0.8516296  2.35398599 1.8182068  0.40230746], Cost: 1.1768
Current parameters: [2.85710927 2.82629567 2.57534731 1.56892618 2.17917017 2.48429348
 0.94712299 2.43755018 1.99706975 0.46116621], Cost: 0.4507
Current parameters: [2.86657613 2.7927261  2.62936908 1.5696632  2.09958754 2.67877408
 1.10057754 2.51959237 1.92474908 0.44445407], Cost: 1.0276
Current parameters: [2.98092369 2.82454637 2.58650593 1.57057153 2.1651689  2.60226372
 0.91246053 2.41656235 1.99923836 0.46664481], Cost: 0.3732
Current parameters: [2.81212614 2.79215171 2.64362709 1.56871817 2.07790373 2.60164467
 0.83447161 2.35918682 2.0074756  0.64246487], Cost: 0.1990
Current parameters: [2.80794338 2.91302889 2.66667094 1.56822061 2.07323166 2.60434919
 0.8252083  2.35063159 1.99269286 0.65009847], Cost: 0.2252
Current parameters: [2.81212614 2.79215171 2.64362709 1.50625689 2.07790373 2.60251583
 0.83447161 2.35918682 2.00935349 0.64172154], Cost: 0.2605
Current parameters: [2.79777983 2.79483303 2.65809027 1.59083845 2.06187912 2.6105885
 0.80269965 2.32984349 2.11341448 0.60664321], Cost: 0.5229
Current parameters: [2.81261606 2.79085365 2.64051158 1.56857238 2.08235612 2.60127221
 0.86463437 2.30496356 2.0025482  0.64183073], Cost: 0.2631
Current parameters: [2.80395095 2.77779749 2.6173585  1.58512898 2.12017921 2.60591765
 0.75468495 2.34953038 1.92925737 0.6456342 ], Cost: 0.1845
Current parameters: [2.80590117 2.77051784 2.63971866 1.58468575 2.07923082 2.59543604
```



```
 0.74739243 2.34347983 1.90700154 0.61436867], Cost: 0.2745
Current parameters: [2.78755023 2.76906918 2.6207574  1.61051595 2.12945733 2.66426195
 0.80226333 2.4112515  1.8861197  0.70219908], Cost: 0.0396
Current parameters: [2.77318754 2.7688464  2.61142678 1.62839683 2.15425465 2.56975081
 0.82983604 2.45516807 1.86741697 0.75145254], Cost: 0.0547
Current parameters: [2.80865391 2.76121808 2.67083867 1.61050252 2.15922267 2.66329911
 0.80062438 2.41025913 1.88638981 0.70288146], Cost: 0.0247
Current parameters: [2.92006739 2.76540993 2.63829815 1.6210044  2.14133874 2.67353938
 0.79608927 2.42220082 1.88061119 0.74035182], Cost: 0.0742
Current parameters: [2.82075877 2.76992006 2.65475711 1.61067657 2.18290301 2.66302773
 0.82317669 2.42713631 1.87487684 0.65885279], Cost: 0.0856
Current parameters: [2.76717084 2.75074151 2.68242762 1.65947903 2.19009903 2.71761972
 0.77084195 2.44637692 1.93777481 0.75313491], Cost: 0.1475
Current parameters: [2.80363479 2.75605285 2.66800079 1.6183097  2.17057466 2.67077896
 0.83110119 2.36471788 1.87071659 0.72164491], Cost: 0.0634
Current parameters: [2.8096767  2.73166059 2.66520387 1.60412398 2.16036511 2.66272845
 0.80288949 2.41235109 1.89019998 0.7009375 ], Cost: 0.0307
Current parameters: [2.80275218 2.7720572  2.67401689 1.55517944 2.16520617 2.67218416
 0.79853954 2.41999627 1.87622405 0.72201106], Cost: 0.0235
Current parameters: [2.80079304 2.76818866 2.66836628 1.55925122 2.17410127 2.67282923
 0.77836491 2.41734761 1.85584106 0.72154879], Cost: 0.0255
Current parameters: [2.79701934 2.76430595 2.70526594 1.557691   2.12278448 2.67300098
 0.80261303 2.43305148 1.84734339 0.71899444], Cost: 0.0922
Current parameters: [2.80667159 2.77233316 2.67620628 1.55017379 2.15892265 2.69375346
 0.79060663 2.40771808 1.88188087 0.70856216], Cost: 0.0244
Current parameters: [2.80544111 2.77890359 2.67703418 1.53976146 2.16492515 2.63265246
 0.77119455 2.39496807 1.90151856 0.71667029], Cost: 0.0360
Current parameters: [2.77466412 2.77083598 2.68140357 1.55607801 2.17257436 2.67348598
 0.79871833 2.4205385  1.88046694 0.71449781], Cost: 0.0166
Current parameters: [2.77110113 2.77485594 2.67943846 1.57095286 2.190719   2.69546617
 0.77701382 2.43900348 1.91294578 0.74569075], Cost: 0.0551
Current parameters: [2.78884602 2.77663464 2.67377757 1.55006369 2.17800974 2.66825535
 0.81804503 2.43321493 1.88303387 0.7215532 ], Cost: 0.0220
Current parameters: [2.77086182 2.78182185 2.67471735 1.55358699 2.20319397 2.67456843
 0.79526528 2.42630025 1.87650777 0.66233782], Cost: 0.0265
Current parameters: [2.76983278 2.77124887 2.67974578 1.55752852 2.18382877 2.67658767
 0.81122045 2.39623578 1.87515403 0.72056111], Cost: 0.0240
Current parameters: [2.77095406 2.78357205 2.67573696 1.55856268 2.16745838 2.67385919
 0.79809395 2.41988004 1.87894161 0.71514732], Cost: 0.0151
Current parameters: [2.76173941 2.76930683 2.65176438 1.55328105 2.15948588 2.67367662
 0.79930163 2.41989263 1.88235668 0.71217069], Cost: 0.0239
```



```
Current parameters: [2.76767102 2.78592929 2.67699823 1.54493149 2.16940595 2.67695169
 0.79505913 2.42077566 1.87605617 0.71809875], Cost: 0.0155
Current parameters: [2.76527137 2.78471611 2.69276352 1.55930234 2.14925482 2.66940733
 0.80522042 2.4250177  1.86509107 0.70946386], Cost: 0.0323
Current parameters: [2.77156036 2.77905739 2.68028718 1.55480666 2.16628949 2.66326522
 0.7932322  2.41715305 1.88550749 0.71425495], Cost: 0.0156
Current parameters: [2.77674681 2.80812122 2.66465742 1.55744221 2.16747469 2.66410144
 0.79845309 2.40924057 1.88153882 0.71617027], Cost: 0.0180
Current parameters: [2.77012609 2.7848773  2.67563681 1.55802339 2.17540331 2.67393378
 0.79622212 2.42220084 1.87874455 0.70213216], Cost: 0.0123
Current parameters: [2.76825697 2.78630351 2.67724698 1.55714568 2.17399398 2.67670404
 0.79924779 2.42171186 1.88437751 0.70096601], Cost: 0.0113
Current parameters: [2.7659492  2.7888583  2.67856703 1.56192931 2.18106405 2.68056927
 0.79116886 2.4264519  1.89104897 0.70420496], Cost: 0.0060
Current parameters: [2.77607522 2.7878849  2.67925231 1.56116128 2.17897148 2.68855679
 0.78432286 2.42394615 1.89396756 0.7010819 ], Cost: 0.0053
Current parameters: [2.7765207  2.78997423 2.67942861 1.56093914 2.17461539 2.68741337
 0.78314885 2.42969714 1.89383863 0.69980157], Cost: 0.0047
Current parameters: [2.77204295 2.79474953 2.68824232 1.56627356 2.17840207 2.69156877
 0.77756343 2.43367054 1.8965988  0.6968116 ], Cost: 0.0026
Current parameters: [2.77867672 2.79509968 2.69001058 1.56548787 2.1867437  2.69171381
 0.78168365 2.44249812 1.90195936 0.6985509 ], Cost: 0.0023
Current parameters: [2.77666909 2.78842093 2.69071302 1.56543669 2.18613911 2.69398813
 0.78173744 2.44494968 1.901593   0.69808446], Cost: 0.0036
Current parameters: [2.77479902 2.80072362 2.68089582 1.56715892 2.1891522  2.6927701
 0.77556614 2.4443014  1.90956607 0.69607106], Cost: 0.0054
Current parameters: [2.77874254 2.79696588 2.68770418 1.56854061 2.18642341 2.6965747
 0.7844732  2.44353274 1.8990444  0.69990348], Cost: 0.0027
Current parameters: [2.78176407 2.79378653 2.69319976 1.57583191 2.18175025 2.68789006
 0.78160994 2.44096667 1.90732752 0.70525841], Cost: 0.0055
Current parameters: [2.77668618 2.79643455 2.69155672 1.56186714 2.18430943 2.69452587
 0.78375194 2.44180101 1.90569227 0.7012444 ], Cost: 0.0053
Current parameters: [2.78049478 2.7968792  2.69179762 1.56259822 2.19062808 2.69036189
 0.77285738 2.44501653 1.89338107 0.70571864], Cost: 0.0013
Current parameters: [2.7857776  2.79621691 2.69205741 1.56189242 2.18909986 2.69415272
 0.76992385 2.44343323 1.89441804 0.70392197], Cost: 0.0012
Current parameters: [2.7893614  2.79975461 2.69709281 1.56299318 2.19231803 2.69014489
 0.77083635 2.44370922 1.88655541 0.69376981], Cost: 0.0016
Current parameters: [2.78873663 2.79583478 2.69368453 1.56653719 2.18809494 2.69000587
 0.76995419 2.44231743 1.89680179 0.70558872], Cost: 0.0013
Current parameters: [2.7837369  2.79526912 2.69352833 1.56958728 2.18604575 2.70005214
```

```
 0.76427371 2.44671075 1.88613113 0.70863717], Cost: 0.0022
Current parameters: [2.7851447  2.7975914  2.68718567 1.56351454 2.19152817 2.69301725
 0.76590817 2.4444169  1.8962436  0.70173836], Cost: 0.0010
Current parameters: [2.78433468 2.79725523 2.68828723 1.56433514 2.19407532 2.69411056
 0.76570718 2.44225953 1.89667474 0.70193581], Cost: 0.0013
Current parameters: [2.78795855 2.79511568 2.68255035 1.5622422  2.19245906 2.6914997
 0.76732311 2.44221614 1.89292322 0.70342461], Cost: 0.0007
Current parameters: [2.78939414 2.7946001  2.68276983 1.56246588 2.19449804 2.69223674
 0.76826931 2.44479459 1.89337601 0.70404466], Cost: 0.0008
Current parameters: [2.78996408 2.79968079 2.68061669 1.55786945 2.19111069 2.68963701
 0.76604905 2.44136061 1.89317024 0.70579294], Cost: 0.0009
Current parameters: [2.78772497 2.79316244 2.68378887 1.56073144 2.19296442 2.69006984
 0.76512696 2.44273921 1.8927346  0.70379957], Cost: 0.0006
Current parameters: [2.78737533 2.78887387 2.6805091  1.55928593 2.18946093 2.6890006
 0.76629633 2.44267707 1.8943484  0.7002951 ], Cost: 0.0006
Current parameters: [2.78677451 2.79226627 2.68279405 1.56121657 2.19193825 2.69151284
 0.76438517 2.44296079 1.8937807  0.70653789], Cost: 0.0014
Current parameters: [2.78923555 2.79408576 2.68437958 1.56194486 2.192499   2.6883012
 0.76408295 2.44346476 1.88694351 0.69960652], Cost: 0.0003
Current parameters: [2.78966328 2.79393259 2.68548009 1.56052225 2.19695873 2.68721123
 0.76521592 2.43875274 1.88746301 0.69603442], Cost: 0.0009
Current parameters: [2.79201154 2.79458458 2.68456502 1.56099087 2.19160686 2.68984972
 0.76261829 2.44285996 1.88764499 0.69920355], Cost: 0.0003
Current parameters: [2.79287492 2.79884318 2.68360456 1.55684599 2.19019273 2.68570441
 0.76368209 2.44465617 1.88806864 0.69977857], Cost: 0.0004
Current parameters: [2.79006361 2.79558472 2.68393903 1.55976731 2.19230765 2.69160691
 0.76144282 2.44400837 1.88782796 0.69769947], Cost: 0.0002
Current parameters: [2.79011824 2.79716639 2.68449426 1.56403743 2.19218975 2.68894248
 0.75858858 2.44533027 1.89203026 0.69539255], Cost: 0.0005
Current parameters: [2.78943408 2.79545686 2.68733279 1.55913763 2.19125447 2.69198822
 0.76233443 2.44449126 1.88848741 0.6972364 ], Cost: 0.0001
Current parameters: [2.78978088 2.79653514 2.6860479  1.56099082 2.18673655 2.69442778
 0.76525043 2.44546637 1.88551449 0.69407228], Cost: 0.0012
Current parameters: [2.78926383 2.79794038 2.68860706 1.56012837 2.19212411 2.69265216
 0.76157253 2.4439329  1.88712472 0.6988208 ], Cost: 0.0001
Current parameters: [2.78859528 2.7954411  2.68721161 1.55893314 2.19021864 2.6917042
 0.76165362 2.4432964  1.88848605 0.69733421], Cost: 0.0001
Current parameters: [2.78922834 2.79372127 2.68815186 1.5577936  2.19166756 2.691942
 0.7602649  2.44581498 1.88833427 0.69910602], Cost: 0.0000
Current parameters: [2.78894107 2.79393301 2.6876896  1.55799815 2.19119594 2.69266346
 0.76076299 2.44606789 1.889417   0.70020353], Cost: 0.0001
```



```
Current parameters: [2.78903966 2.79335786 2.68888383 1.55608719 2.19404503 2.69223632
 0.76056088 2.44391062 1.88980447 0.69927573], Cost: 0.0000
Current parameters: [2.78886243 2.79233853 2.68907685 1.55717188 2.19426889 2.69332182
 0.76036146 2.44348636 1.88963022 0.69904793], Cost: 0.0000
Current parameters: [2.78877514 2.79144404 2.69006044 1.55466391 2.19364732 2.69543543
 0.7605888  2.44390456 1.88817646 0.69904718], Cost: 0.0002
Current parameters: [2.79017657 2.79270264 2.68921086 1.55707698 2.19383816 2.69382849
 0.75943777 2.44330921 1.89030725 0.69866975], Cost: 0.0000
Current parameters: [2.78886243 2.79233853 2.68907685 1.55717188 2.19426889 2.69332182
 0.76036146 2.44348636 1.88963022 0.69904793], Cost: 0.0000

   Return from subroutine COBYLA because the MAXFUN limit has been reached.

   NFVALS =  100   F = 1.283695E-05    MAXCV = 0.000000E+00
   X = 2.788862E+00   2.792339E+00   2.689077E+00   1.557172E+00   2.194269E+00
       2.693322E+00   7.603615E-01   2.443486E+00   1.889630E+00   6.990479E-01
Simulating for QAOA depth p = 6
Current parameters: [1.79014793 1.59254891 1.46263006 0.20317434 2.78560144 1.89399687
 1.22880476 1.55344236 2.16279586 0.37160499 0.80384493 2.14132997], Cost: 2.6705
Current parameters: [0.79014793 2.59254891 1.46263006 0.20317434 2.78560144 1.89399687
 1.22880476 1.55344236 2.16279586 0.37160499 0.80384493 2.14132997], Cost: 2.2618
Current parameters: [0.79014793 1.59254891 2.46263006 0.20317434 2.78560144 1.89399687
 1.22880476 1.55344236 2.16279586 0.37160499 0.80384493 2.14132997], Cost: 2.2392
Current parameters: [0.79014793 1.59254891 1.46263006 1.20317434 2.78560144 1.89399687
 1.22880476 1.55344236 2.16279586 0.37160499 0.80384493 2.14132997], Cost: 2.4536
Current parameters: [0.79014793 1.59254891 1.46263006 0.20317434 3.78560144 1.89399687
 1.22880476 1.55344236 2.16279586 0.37160499 0.80384493 2.14132997], Cost: 2.0903
Current parameters: [0.79014793 1.59254891 1.46263006 0.20317434 2.78560144 2.89399687
 1.22880476 1.55344236 2.16279586 0.37160499 0.80384493 2.14132997], Cost: 3.3453
Current parameters: [0.79014793 1.59254891 1.46263006 0.20317434 2.78560144 1.89399687
 2.22880476 1.55344236 2.16279586 0.37160499 0.80384493 2.14132997], Cost: 1.7858
Current parameters: [0.79014793 1.59254891 1.46263006 0.20317434 2.78560144 1.89399687
 1.22880476 2.55344236 2.16279586 0.37160499 0.80384493 2.14132997], Cost: 0.5885
Current parameters: [0.79014793 1.59254891 1.46263006 0.20317434 2.78560144 1.89399687
 1.22880476 2.55344236 3.16279586 0.37160499 0.80384493 2.14132997], Cost: 2.5924
Current parameters: [0.79014793 1.59254891 1.46263006 0.20317434 2.78560144 1.89399687
 1.22880476 2.55344236 2.16279586 1.37160499 0.80384493 2.14132997], Cost: 2.8054
Current parameters: [0.79014793 1.59254891 1.46263006 0.20317434 2.78560144 1.89399687
 1.22880476 2.55344236 2.16279586 0.37160499 1.80384493 2.14132997], Cost: 2.8334
Current parameters: [0.79014793 1.59254891 1.46263006 0.20317434 2.78560144 1.89399687
 1.22880476 2.55344236 2.16279586 0.37160499 0.80384493 3.14132997], Cost: 1.8950
```



```
Current parameters: [ 0.46793898  1.33676527  1.21053458 -0.08377573  2.5577124   1.46208491
  1.05040658  2.569688    1.83703176  0.01122097  0.43890558  1.92893211], Cost: 1.5245
Current parameters: [1.27023915 1.55480643 1.42543179 0.16083306 2.75197498 1.83026556
 1.20248099 2.5558395  2.11472732 0.31842807 0.74999584 2.10998933], Cost: 0.7003
Current parameters: [0.79014793 1.76803725 1.28457433 0.20317434 2.78560144 1.89399687
 1.22880476 2.55344236 2.16279586 0.37160499 0.80384493 2.14132997], Cost: 0.8206
Current parameters: [0.79164566 1.90925415 1.81397603 0.15009465 2.74344679 1.81410229
 1.19580487 2.55644746 2.10253639 0.30494156 0.73633887 2.10204086], Cost: 0.5260
Current parameters: [ 0.79164566  1.90925415  1.81397603 -0.05813823  2.74344679  1.95244632
  1.19580487  2.55644746  2.10253639  0.30494156  0.73633887  2.10204086], Cost: 0.6346
Current parameters: [0.79451911 1.88741298 1.86767054 0.40786022 2.66257147 2.1567793
 1.13249331 2.56221286 1.98692629 0.17704523 0.6068259  2.02666319], Cost: 2.5313
Current parameters: [0.79164566 1.90925415 1.81397603 0.15009465 2.89755076 1.81410229
 0.99894968 2.55644746 2.10253639 0.30494156 0.73633887 2.10204086], Cost: 0.5838
Current parameters: [0.79306713 1.89844954 1.84053817 0.11405968 3.11634161 1.73745022
 1.49609457 2.55929954 2.04534523 0.24167253 0.67227011 2.06475228], Cost: 2.9765
Current parameters: [0.79164566 1.90925415 1.81397603 0.15009465 2.74344679 1.81410229
 1.19580487 2.55644746 2.10253639 0.12705815 0.91200187 2.10204086], Cost: 0.4695
Current parameters: [0.79399613 1.89138814 1.85789797 0.09050886 2.5733783  1.6873539
 1.07652176 2.56116354 2.00796769 0.41096359 1.21467199 2.04038216], Cost: 1.4298
Current parameters: [0.79164566 1.90925415 1.81397603 0.15009465 2.74344679 1.81410229
 1.19580487 2.55644746 2.23907763 0.12705815 0.91200187 1.89262145], Cost: 1.1981
Current parameters: [0.79343778 1.89563217 1.8474644  0.10466335 2.61377774 1.71746273
 1.10485722 2.56004324 2.41442279 0.06287138 0.85857478 2.43058394], Cost: 0.8771
Current parameters: [0.78310871 1.90247715 1.80729675 0.14249189 2.73740885 1.80265876
 1.1910782  2.80503989 2.09390525 0.11750975 0.90233278 2.09641336], Cost: 0.2308
Current parameters: [ 6.42438679e-01  1.78560804e+00  1.70832504e+00  9.63748934e-04
  2.59266870e+00  1.57986825e+00  1.08142102e+00  2.76430114e+00
  1.91339442e+00 -6.27007490e-02  7.23843027e-01  2.02198414e+00], Cost: 1.3632
Current parameters: [0.78297633 2.08658837 1.67573243 0.18235494 2.68206851 1.75611491
 1.22263284 2.80498935 2.09491872 0.16058255 0.86204015 2.09707414], Cost: 0.3118
Current parameters: [ 0.79127022  1.98891843  1.5978274  -0.00643064  2.91582865  2.00771064
  1.02202592  2.83676769  2.06822976 -0.04538108  1.10120223  2.1751473 ], Cost: 0.6393
Current parameters: [0.78904637 1.91799957 1.83371692 0.26332856 2.68610506 1.89345941
 1.21492478 2.69871552 1.97037393 0.03629212 0.90176635 2.14455165], Cost: 0.9781
Current parameters: [0.7985352  1.86835835 1.84776624 0.20922455 2.61761874 1.98039775
 1.0289212  3.13744016 2.24275966 0.2715554  0.78925479 2.06314293], Cost: 1.3486
Current parameters: [0.79148324 1.90802939 1.83357906 0.10940475 2.89380967 1.71894541
 1.32975686 2.86817655 2.09026326 0.15848298 0.90330661 2.16147759], Cost: 0.9461
Current parameters: [0.66533206 1.91051651 1.81738791 0.15164436 2.7388837  1.81354345
 1.19426477 2.80113022 2.09892968 0.14122111 0.92905703 2.10387523], Cost: 0.2298
```



```
Current parameters: [0.71414977 1.90356352 1.83648646 0.11611012 2.66270931 1.74657118
 1.14193741 2.787114   2.02914466 0.26405926 1.08732929 2.11834427], Cost: 1.1034
Current parameters: [0.66541144 1.83562012 1.73561423 0.18253654 2.73111532 1.81743739
 1.21812457 2.78935759 2.11769539 0.16456157 0.93511581 2.12951173], Cost: 0.1883
Current parameters: [0.66737466 1.85408819 1.77574609 0.16298086 2.67122653 1.76747071
 1.18459855 2.74588758 2.25845944 0.10369227 0.93241311 2.29545816], Cost: 0.6555
Current parameters: [0.63541013 1.81487021 1.70802054 0.13512645 2.72404708 1.74722882
 1.2117816  2.7568145  2.0692116  0.11533567 0.91552138 2.12251627], Cost: 0.3140
Current parameters: [0.62452817 1.78618163 1.70731756 0.18301673 2.68185912 1.86850539
 1.1374611  2.94605965 2.1432026  0.16883542 0.80400027 2.06928675], Cost: 0.1094
Current parameters: [0.62521202 1.78680289 1.70765276 0.10117482 2.64594555 1.92428135
 1.18437551 2.92639033 2.13309653 0.19756772 0.77717445 2.08631749], Cost: 0.0686
Current parameters: [0.6251081  1.82738887 1.5954639  0.0220547  2.74410146 2.03272514
 1.10197278 2.95245514 2.1418218  0.12085801 0.86062771 2.10105706], Cost: 0.3051
Current parameters: [0.6235098  1.78545159 1.70632095 0.09965887 2.59636175 1.92199957
 1.24523435 2.97595835 2.14701609 0.13981857 0.83039465 2.06120676], Cost: 0.1734
Current parameters: [0.61913075 1.77364211 1.70306576 0.09342529 2.66707194 1.94386374
 1.19834836 2.81891276 2.26639062 0.21387722 0.74206736 1.91205782], Cost: 0.5463
Current parameters: [0.62108137 1.78465839 1.7178653  0.16670724 2.63353876 1.98771465
 1.1961654  2.90019266 2.07427304 0.16332649 0.7406106  2.10154692], Cost: 0.3241
Current parameters: [0.61934169 1.78987283 1.70890354 0.1006699  2.68609241 1.91873182
 1.21809791 2.94017742 2.1430176  0.18673145 0.75312679 2.09614353], Cost: 0.1518
Current parameters: [0.64441895 1.76825683 1.71681454 0.08421071 2.64523929 1.90143954
 1.17367288 2.97449031 2.09354724 0.29167531 0.81116333 2.0901106 ], Cost: 0.2053
Current parameters: [0.62532952 1.74231181 1.74178051 0.09051633 2.65701853 1.93360639
 1.1751702  2.92455685 2.13883371 0.18436299 0.78697285 2.09321081], Cost: 0.0718
Current parameters: [0.62814285 1.80417174 1.71582266 0.09496875 2.59689841 1.90283053
 1.15033394 2.92510476 2.19940384 0.19794722 0.76424509 2.16767632], Cost: 0.1936
Current parameters: [0.56620566 1.79073951 1.71258789 0.10556141 2.6466547  1.9294429
 1.18594339 2.92430549 2.13541487 0.20922627 0.79045783 2.09002035], Cost: 0.0801
Current parameters: [0.59732702 1.76062468 1.67840109 0.05118318 2.62930008 1.85400965
 1.16574739 2.90345599 2.08184485 0.16028933 0.75134552 2.08035239], Cost: 0.1755
Current parameters: [0.61764128 1.8039253  1.73108562 0.09043876 2.63957987 1.93233891
 1.16113268 2.95942687 2.13122706 0.19017773 0.75086074 2.06622299], Cost: 0.0600
Current parameters: [0.63314447 1.8737582  1.79996766 0.0669041  2.65875373 1.91115192
 1.16761385 2.91535206 2.10724361 0.17753268 0.79157496 2.07016363], Cost: 0.0911
Current parameters: [0.63100504 1.81772628 1.69544193 0.07282618 2.65644677 1.95841597
 1.14137342 2.95550939 2.13019126 0.18104418 0.7729457  2.06996429], Cost: 0.0730
Current parameters: [0.55133063 1.78602243 1.7723149  0.06351082 2.67584513 1.93902124
 1.12232303 3.02536305 2.13766557 0.15867358 0.7587265  2.07937557], Cost: 0.1568
Current parameters: [0.61475208 1.79886618 1.7350534  0.08272183 2.64095549 1.93556973
```

```
 1.15313123 2.93789737 2.1649697  0.20229053 0.74724977 2.02196448], Cost: 0.0240
Current parameters: [0.6136906  1.79445587 1.74534936 0.11076113 2.62781024 1.97287985
 1.1431226  2.91444552 2.1485007  0.19953307 0.73168175 2.00592504], Cost: 0.0709
Current parameters: [0.61679299 1.79300491 1.73498763 0.08149159 2.65039897 1.93580158
 1.15863004 2.9459972  2.14722838 0.21559437 0.75456336 2.00760438], Cost: 0.0304
Current parameters: [0.63297637 1.79976322 1.73899606 0.07676814 2.62003316 1.92784393
 1.13469966 2.94271069 2.18539298 0.2475369  0.75364431 2.03448535], Cost: 0.0679
Current parameters: [0.61042168 1.79505049 1.73054723 0.07529665 2.64856598 1.92445575
 1.13975165 2.92541605 2.15450939 0.20669384 0.73285108 2.02589459], Cost: 0.0186
Current parameters: [0.60807971 1.78538392 1.7142412  0.05341974 2.61301207 1.90016951
 1.14467693 2.91892416 2.14658822 0.18324629 0.74099231 2.00436045], Cost: 0.0660
Current parameters: [0.60419731 1.79358348 1.73548775 0.05377297 2.64450216 1.93893193
 1.15045217 2.92540471 2.15336037 0.21123338 0.72493856 2.03067778], Cost: 0.0235
Current parameters: [0.5782588  1.78602276 1.75082903 0.06851822 2.67055685 1.92345473
 1.11207774 2.95033849 2.16949833 0.19271211 0.736249   2.02044261], Cost: 0.0162
Current parameters: [0.58037845 1.80187684 1.7693055  0.06236902 2.67164137 1.91953615
 1.10959692 2.93919061 2.16296585 0.19042459 0.74835763 2.02024527], Cost: 0.0161
Current parameters: [0.58931035 1.81697863 1.7702884  0.06280375 2.71262477 1.90462136
 1.1342724  2.93259284 2.18580831 0.18382084 0.72745757 2.01387731], Cost: 0.0230
Current parameters: [0.60244453 1.79456465 1.77946295 0.05914909 2.67114724 1.92441575
 1.10180165 2.94469005 2.16014822 0.18096814 0.73865213 2.01624326], Cost: 0.0140
Current parameters: [0.58786741 1.79600029 1.79019434 0.09203315 2.67350014 1.95722024
 1.09145327 2.91849972 2.15365362 0.17745867 0.71971623 2.00184133], Cost: 0.0754
Current parameters: [0.60376883 1.81662217 1.7666495  0.05436654 2.67051776 1.93108556
 1.09228635 2.95400415 2.15760557 0.18022967 0.73387798 2.00922626], Cost: 0.0152
Current parameters: [0.61597585 1.78257278 1.75497025 0.04341584 2.69596155 1.94540081
 1.08921203 2.92680927 2.16781111 0.17102071 0.76937528 2.02604092], Cost: 0.0356
Current parameters: [0.61859833 1.79711903 1.78898046 0.05325904 2.66382461 1.90878376
 1.08265058 2.9544302  2.18731974 0.22430194 0.73888241 2.02797897], Cost: 0.0346
Current parameters: [0.60346585 1.79815573 1.78002795 0.06294598 2.67529955 1.91907006
 1.10468434 2.95747341 2.14756717 0.17478421 0.73762069 2.03932481], Cost: 0.0279
Current parameters: [0.60134145 1.79676472 1.77847425 0.05837478 2.66432724 1.92280733
 1.09955621 2.94046823 2.16816614 0.17309415 0.73575059 2.02170546], Cost: 0.0141
Current parameters: [0.60149885 1.78887249 1.77482045 0.04706003 2.66295256 1.9059477
 1.10543617 2.9481072  2.15602733 0.17311929 0.74206372 2.00036134], Cost: 0.0287
Current parameters: [0.60620328 1.79639177 1.77915438 0.06960458 2.67008641 1.92280798
 1.10235229 2.94889728 2.16409536 0.17780754 0.74642528 2.01315196], Cost: 0.0124
Current parameters: [0.60510798 1.79839672 1.78683302 0.05997998 2.66319084 1.93872843
 1.11979912 2.95970003 2.17268915 0.17723969 0.75125238 2.01273842], Cost: 0.0140
Current parameters: [0.59938863 1.7955129  1.78399161 0.06805081 2.67862603 1.92134486
 1.09789012 2.954207   2.16949825 0.17398172 0.74549728 2.01134736], Cost: 0.0141
```



```
Current parameters: [0.61633232 1.80503255 1.77627896 0.07069663 2.68505774 1.9154213
 1.11917465 2.94206284 2.17246842 0.17731183 0.73611825 2.01095022], Cost: 0.0141
Current parameters: [0.61002473 1.79047743 1.77612876 0.06306858 2.67780549 1.92867543
 1.10065715 2.94456853 2.16295432 0.17882363 0.75126734 2.01428404], Cost: 0.0153
Current parameters: [0.60894753 1.80567462 1.76514645 0.0741301  2.66487573 1.91889605
 1.09854935 2.95001695 2.17739319 0.16531288 0.7619671  2.01893908], Cost: 0.0194
Current parameters: [0.6105198  1.79667541 1.78193208 0.06811737 2.66810006 1.91958192
 1.09815514 2.95099265 2.17069558 0.18925677 0.74631737 2.01526888], Cost: 0.0085
Current parameters: [0.61104178 1.80063774 1.78655802 0.06659623 2.66837409 1.91862109
 1.09752982 2.94819075 2.16905762 0.1886856  0.74933348 2.01521133], Cost: 0.0089
Current parameters: [0.61050866 1.79714371 1.78581366 0.07647883 2.66529125 1.92587122
 1.09487688 2.94628122 2.17437729 0.19501222 0.74209506 2.01152146], Cost: 0.0058
Current parameters: [0.61199604 1.79311387 1.78757724 0.07448777 2.65885181 1.92029448
 1.09448336 2.94554864 2.1754936  0.19961922 0.74032322 2.00057859], Cost: 0.0075
Current parameters: [0.61603622 1.79639797 1.78784866 0.07660608 2.66465682 1.92667043
 1.09279663 2.94832947 2.17415243 0.19154306 0.74006732 2.0116008 ], Cost: 0.0050
Current parameters: [0.61856365 1.79630877 1.79293625 0.08284362 2.663465   1.92646875
 1.09223541 2.95275006 2.17038955 0.19867476 0.73546648 2.01969083], Cost: 0.0046
Current parameters: [0.61870756 1.79219426 1.79491708 0.08520802 2.66252485 1.92438703
 1.09394301 2.95063825 2.17310584 0.19685885 0.73798938 2.02191283], Cost: 0.0049
Current parameters: [0.62262214 1.80338497 1.78784163 0.08801025 2.6591224  1.92726873
 1.08653572 2.95486735 2.17767519 0.19886031 0.73878356 2.02177939], Cost: 0.0080
Current parameters: [0.61553563 1.79462712 1.79337473 0.08034132 2.6592077  1.92799497
 1.08826983 2.95242651 2.16949582 0.19783353 0.73541258 2.02187961], Cost: 0.0042
Current parameters: [0.61629968 1.79717285 1.79365895 0.07550951 2.65908396 1.92641042
 1.09071023 2.94571523 2.17451349 0.19707945 0.72467509 2.02624845], Cost: 0.0024
Current parameters: [0.61551804 1.79845712 1.79591688 0.07407958 2.65637816 1.92944598
 1.09513836 2.9489016  2.17673614 0.19680505 0.72518022 2.02599546], Cost: 0.0021
Current parameters: [0.62074636 1.79285956 1.79721777 0.06822378 2.66061532 1.93868483
 1.09479334 2.9454474  2.17808576 0.20213623 0.72585894 2.02784638], Cost: 0.0026
Current parameters: [0.61318574 1.79810276 1.79871846 0.07306296 2.65997233 1.92813051
 1.09223328 2.95178975 2.18055153 0.19726641 0.72472612 2.02556889], Cost: 0.0028
Current parameters: [0.61171998 1.79735495 1.7993594  0.07841467 2.65708248 1.93600678
 1.09731721 2.94612811 2.17220882 0.18975873 0.71685233 2.02445415], Cost: 0.0062
Current parameters: [0.61519263 1.79413945 1.79147154 0.07487536 2.65549509 1.92938375
 1.09586326 2.95175603 2.17825534 0.19760428 0.72228424 2.02494553], Cost: 0.0026
Current parameters: [0.61940407 1.79732653 1.80135984 0.06982441 2.64926129 1.92331681
 1.09524314 2.94675942 2.1755967  0.20237804 0.72330024 2.01883156], Cost: 0.0003
Current parameters: [0.62281842 1.79747827 1.80067305 0.06013401 2.6489663  1.91658106
 1.09902283 2.95039682 2.17065286 0.20037527 0.72428061 2.02480621], Cost: 0.0018
Current parameters: [0.61397194 1.79662919 1.80100486 0.06917197 2.65060171 1.92247917
```



```
 1.09812487 2.94496571 2.17395535 0.20602596 0.7239627  2.01900136], Cost: 0.0004
Current parameters: [0.62077304 1.80487548 1.79783049 0.0704619  2.64193102 1.92266836
 1.09358247 2.94269044 2.18206393 0.20937195 0.72539021 2.02079157], Cost: 0.0040
Current parameters: [0.61850131 1.80103514 1.80302238 0.07117849 2.64725322 1.92606196
 1.0987507  2.94587085 2.17345701 0.19912815 0.72420401 2.01995009], Cost: 0.0003
Current parameters: [0.61763984 1.79624953 1.80955002 0.0716642  2.64714746 1.92715819
 1.09801048 2.94264163 2.16873384 0.19625736 0.71429935 2.01388298], Cost: 0.0043
Current parameters: [0.61796397 1.79949587 1.80497222 0.07016484 2.64539553 1.92829375
 1.10080935 2.95101264 2.17509812 0.20056237 0.72622428 2.01758198], Cost: 0.0012
Current parameters: [0.61796082 1.80232754 1.80141039 0.06922202 2.64777837 1.92674655
 1.09833172 2.94565784 2.17335367 0.19829151 0.724325   2.01767426], Cost: 0.0003
Current parameters: [0.61810738 1.79783919 1.8032142  0.07072869 2.64663925 1.92443086
 1.10038627 2.94251553 2.17669787 0.19565277 0.72741308 2.01919083], Cost: 0.0005
Current parameters: [0.61850131 1.80103514 1.80302238 0.07117849 2.64725322 1.92606196
 1.0987507  2.94587085 2.17345701 0.19912815 0.72420401 2.01995009], Cost: 0.0003

   Return from subroutine COBYLA because the MAXFUN limit has been reached.

   NFVALS =  100   F = 2.786239E-04    MAXCV = 0.000000E+00
   X = 6.185013E-01   1.801035E+00   1.803022E+00   7.117849E-02   2.647253E+00
       1.926062E+00   1.098751E+00   2.945871E+00   2.173457E+00   1.991281E-01
       7.242040E-01   2.019950E+00
Simulating for QAOA depth p = 7
Current parameters: [2.43758912 0.71258576 1.01171817 0.64025265 0.49377851 0.95042113
 2.54213201 0.17487476 1.48160414 2.52070123 1.24040454 2.4731065
 2.90500549 1.59139621], Cost: 2.2932
Current parameters: [1.43758912 1.71258576 1.01171817 0.64025265 0.49377851 0.95042113
 2.54213201 0.17487476 1.48160414 2.52070123 1.24040454 2.4731065
 2.90500549 1.59139621], Cost: 1.8197
Current parameters: [1.43758912 0.71258576 2.01171817 0.64025265 0.49377851 0.95042113
 2.54213201 0.17487476 1.48160414 2.52070123 1.24040454 2.4731065
 2.90500549 1.59139621], Cost: 1.6578
Current parameters: [1.43758912 0.71258576 2.01171817 1.64025265 0.49377851 0.95042113
 2.54213201 0.17487476 1.48160414 2.52070123 1.24040454 2.4731065
 2.90500549 1.59139621], Cost: 2.0056
Current parameters: [1.43758912 0.71258576 2.01171817 0.64025265 1.49377851 0.95042113
 2.54213201 0.17487476 1.48160414 2.52070123 1.24040454 2.4731065
 2.90500549 1.59139621], Cost: 2.7975
Current parameters: [1.43758912 0.71258576 2.01171817 0.64025265 0.49377851 1.95042113
 2.54213201 0.17487476 1.48160414 2.52070123 1.24040454 2.4731065
 2.90500549 1.59139621], Cost: 0.8231
```



```
Current parameters: [1.43758912 0.71258576 2.01171817 0.64025265 0.49377851 1.95042113
 3.54213201 0.17487476 1.48160414 2.52070123 1.24040454 2.4731065
 2.90500549 1.59139621], Cost: 0.8355
Current parameters: [1.43758912 0.71258576 2.01171817 0.64025265 0.49377851 1.95042113
 2.54213201 1.17487476 1.48160414 2.52070123 1.24040454 2.4731065
 2.90500549 1.59139621], Cost: 3.0114
Current parameters: [1.43758912 0.71258576 2.01171817 0.64025265 0.49377851 1.95042113
 2.54213201 0.17487476 2.48160414 2.52070123 1.24040454 2.4731065
 2.90500549 1.59139621], Cost: 0.8330
Current parameters: [1.43758912 0.71258576 2.01171817 0.64025265 0.49377851 1.95042113
 2.54213201 0.17487476 1.48160414 3.52070123 1.24040454 2.4731065
 2.90500549 1.59139621], Cost: 3.6648
Current parameters: [1.43758912 0.71258576 2.01171817 0.64025265 0.49377851 1.95042113
 2.54213201 0.17487476 1.48160414 2.52070123 2.24040454 2.4731065
 2.90500549 1.59139621], Cost: 2.8150
Current parameters: [1.43758912 0.71258576 2.01171817 0.64025265 0.49377851 1.95042113
 2.54213201 0.17487476 1.48160414 2.52070123 1.24040454 3.4731065
 2.90500549 1.59139621], Cost: 2.7232
Current parameters: [1.43758912 0.71258576 2.01171817 0.64025265 0.49377851 1.95042113
 2.54213201 0.17487476 1.48160414 2.52070123 1.24040454 2.4731065
 3.90500549 1.59139621], Cost: 2.8675
Current parameters: [1.43758912 0.71258576 2.01171817 0.64025265 0.49377851 1.95042113
 2.54213201 0.17487476 1.48160414 2.52070123 1.24040454 2.4731065
 2.90500549 2.59139621], Cost: 2.8124
Current parameters: [ 1.35231025  0.71241722  2.0406671   0.57772178  0.28890632  2.10045747
  2.53990205 -0.21849175  1.47982329  2.00987653  0.88233866  2.13155425
  2.53750015  1.23379406], Cost: 2.5758
Current parameters: [ 1.39494969  0.71250149  2.02619263  0.60898721  0.39134241  2.0254393
  2.54101703 -0.02180849  1.48071371  2.26528888  1.0613716   2.30233038
  2.72125282  1.41259513], Cost: 2.8699
Current parameters: [1.1932647  0.71258576 2.01171817 0.64025265 0.49377851 1.95042113
 2.54213201 0.22784244 1.48160414 2.52070123 1.24040454 2.4731065
 2.90500549 1.59139621], Cost: 0.7963
Current parameters: [1.29406737 0.71256478 2.01532247 0.63246722 0.46827081 1.96910147
 2.54185437 0.70412873 1.48138241 2.45710078 1.19582339 2.43058139
 2.85924908 1.5468728 ], Cost: 1.8216
Current parameters: [1.1932647  0.46258585 2.01171817 0.64025265 0.49398418 1.95042113
 2.54213201 0.22784244 1.48160414 2.52070123 1.24040454 2.4731065
 2.90500549 1.59139621], Cost: 1.3224
Current parameters: [1.18974827 0.73700197 2.01355544 0.63628407 0.98983891 1.95994332
 2.54199048 0.21738891 1.48149111 2.4882813  1.21767959 2.4514296
```

```
 2.88168145 1.56870069], Cost: 1.1473
Current parameters: [1.1932647  0.71258576 2.23858565 0.74528191 0.49377851 1.95042113
 2.54213201 0.22784244 1.48160414 2.52070123 1.24040454 2.4731065
 2.90500549 1.59139621], Cost: 0.6083
Current parameters: [1.19207705 0.72069351 2.03134764 1.19982376 0.49280432 1.95363718
 2.54208421 0.22431184 1.48156597 2.50975165 1.23272937 2.4657853
 2.89712798 1.58373098], Cost: 1.4805
Current parameters: [1.1932647  0.71258576 2.23858565 0.74528191 0.49377851 1.94670586
 2.29215962 0.22784244 1.48160414 2.52070123 1.24040454 2.4731065
 2.90500549 1.59139621], Cost: 0.6166
Current parameters: [1.1906834  0.73020759 2.24990762 0.73582137 0.49166115 1.45275226
 2.54980602 0.22016884 1.48152117 2.49690281 1.22372289 2.45719419
 2.88788407 1.57473616], Cost: 0.9796
Current parameters: [1.1932647  0.71258576 2.23858565 0.74528191 0.49377851 1.95042113
 2.54213201 0.22784244 1.23160566 2.52157278 1.24040454 2.4731065
 2.90500549 1.59139621], Cost: 0.3464
Current parameters: [1.18337938 0.78007029 2.28194427 0.70905183 0.48566986 1.96482955
 2.54297973 0.19845565 1.19965583 2.9946824  1.17652052 2.41216874
 2.83943733 1.52759492], Cost: 0.2613
Current parameters: [1.18337938 0.78007029 2.28194427 0.70905183 0.48566986 1.96482955
 2.54297973 0.19845565 1.19965583 2.9946824  0.99745916 2.41216874
 3.01389945 1.52759492], Cost: 1.5227
Current parameters: [1.17118155 0.86334174 2.33544593 0.6643463  0.47566431 1.98260859
 2.54402577 0.1621943  1.15834631 3.0376136  1.61647203 2.33697557
 3.00486131 1.44886835], Cost: 1.3186
Current parameters: [1.18337938 0.78007029 2.28194427 0.70905183 0.48566986 1.96482955
 2.54297973 0.19845565 1.19965583 2.9946824  1.17652052 2.23138157
 2.83943733 1.70026801], Cost: 0.9760
Current parameters: [1.17203689 0.85750252 2.33169425 0.66748117 0.47636593 1.98136188
 2.54395241 0.16473705 1.16124305 3.03460315 1.23391699 2.77951148
 2.63228513 1.75988431], Cost: 2.1548
Current parameters: [1.16508188 0.81059295 2.30626473 0.68251528 0.44879233 1.99567087
 2.54300205 0.12139346 1.18421187 2.9276355  1.14112841 2.33515085
 2.69814067 1.38686372], Cost: 0.6805
Current parameters: [1.19317851 0.77997479 2.28184661 0.70926277 0.36958014 1.96432343
 2.54298725 0.24365618 1.19966184 2.9964056  1.1777284  2.41332092
 2.84067706 1.52880124], Cost: 0.4493
Current parameters: [1.2284066  0.79611375 2.29288349 0.69900562 0.58274341 1.97178088
 2.54312758 0.40998752 1.19169529 2.99084787 1.17989609 2.39278679
 2.78805543 1.47590366], Cost: 0.7911
Current parameters: [1.18368035 0.7800706  2.25885575 0.75892396 0.48604112 2.07684632
```



```
 2.54131485 0.19984397 1.19967048 2.99888399 1.17946565 2.41497805
 2.8424601  1.53053624], Cost: 0.3143
Current parameters: [1.17924613 0.79388453 2.19477815 0.90908488 0.49055599 1.85532458
 2.54482456 0.18270738 1.1928459  3.01339019 1.19487936 2.41012789
 2.81083212 1.4982951 ], Cost: 0.1013
Current parameters: [1.0572418  0.78753136 2.19477815 0.90908488 0.49056122 1.85532458
 2.54482456 0.20915699 1.1928459  3.01339019 1.19487936 2.41012789
 2.81083212 1.4982951 ], Cost: 0.1841
Current parameters: [1.19301507 0.55184342 2.19812428 0.91532124 0.49735942 1.84907707
 2.54516638 0.16453767 1.18508215 3.04606021 1.22378998 2.41530108
 2.78613827 1.47258755], Cost: 0.2359
Current parameters: [1.17924613 0.79388453 2.18979086 0.90677599 0.49055599 1.85718042
 2.66968989 0.18270738 1.1928459  3.01339019 1.19487936 2.41012789
 2.81083212 1.4982951 ], Cost: 0.0808
Current parameters: [1.17981684 0.79511729 1.96334869 0.80365519 0.49296655 1.85474173
 2.65922457 0.17703893 1.19001095 3.02531972 1.20543614 2.41201689
 2.80181511 1.48890793], Cost: 0.4730
Current parameters: [1.17950199 0.79213781 2.1886686  0.90771374 0.49076587 1.85680749
 2.66966795 0.18346801 1.31565895 3.01945123 1.19942794 2.39536715
 2.81550062 1.48284074], Cost: 0.1679
Current parameters: [1.18080299 0.82249994 2.22583613 0.92055502 0.50669568 1.84388208
 2.6750226  0.13098142 1.21482383 3.03325188 1.22570175 2.58668757
 2.69056726 1.59619183], Cost: 0.5554
Current parameters: [1.17665243 0.81159101 2.20116722 0.89727    0.48842845 1.86096088
 2.66991232 0.17499693 1.18444355 3.13195954 1.18198352 2.39413914
 2.79759632 1.48155501], Cost: 0.2053
Current parameters: [1.17844045 0.82893882 2.22561271 0.90990291 0.50108805 1.85004879
 2.67387555 0.13787241 1.16340869 3.00819844 1.40223153 2.41068959
 2.909256   1.43339453], Cost: 0.3323
Current parameters: [1.17092465 0.78774082 2.20826306 0.86161347 0.53954641 1.81325324
 2.67024548 0.14283742 1.19592797 3.0143873  1.14943041 2.44508933
 2.87305928 1.49144461], Cost: 0.2369
Current parameters: [1.21170371 0.79897628 2.17335521 1.07647414 0.41198169 1.92303934
 2.67782855 0.2067938  1.1594741  2.9726233  1.0973638  2.41749438
 2.88661553 1.44250096], Cost: 0.2689
Current parameters: [1.18345343 0.79105344 2.19794867 0.92757868 0.50118452 1.84620483
 2.67265134 0.16151693 1.18457043 3.00128368 1.19402847 2.32608429
 2.81856062 1.58237789], Cost: 0.3086
Current parameters: [1.18962889 0.79025477 2.18580371 0.91228752 0.50827761 1.85356089
 2.66968635 0.22972465 1.19470492 3.02622846 1.2024942  2.42255134
 2.83116864 1.51847032], Cost: 0.2827
```



```
Current parameters: [1.19019271 0.8081167  2.21678423 0.89864335 0.53101528 1.84986301
 2.67267379 0.19938121 1.17663898 2.97315964 1.18020029 2.37563278
 2.74590533 1.42738402], Cost: 0.0216
Current parameters: [1.19165683 0.81001078 2.21293302 0.90859114 0.50615163 1.79452551
 2.67352638 0.20659462 1.17569099 2.97462822 1.18195689 2.3745977
 2.74113045 1.42253465], Cost: 0.0410
Current parameters: [1.20003851 0.80459186 2.26241606 0.82677434 0.4499594  1.87539209
 2.67430328 0.21499754 1.17127639 2.94941176 1.16856472 2.36912677
 2.74500601 1.4220305 ], Cost: 0.0589
Current parameters: [1.19307352 0.7483341  2.21688199 0.89996898 0.53163541 1.84920589
 2.67271197 0.19830972 1.17256085 2.97964449 1.18512994 2.36610514
 2.74741176 1.41537454], Cost: 0.0676
Current parameters: [1.18970585 0.80388497 2.23713526 0.92114146 0.52021256 1.85983499
 2.67631323 0.14720165 1.17974617 2.95752577 1.18956259 2.44638156
 2.66749644 1.44285551], Cost: 0.1072
Current parameters: [1.19223194 0.80115189 2.17791775 0.88384576 0.52935033 1.85083086
 2.6708334  0.20711496 1.18007185 2.97282253 1.141552   2.37643279
 2.72573303 1.43938204], Cost: 0.0331
Current parameters: [1.19006009 0.83491233 2.14171896 0.87299184 0.51672234 1.86310908
 2.67142146 0.15899848 1.15740228 2.9526834  1.23688004 2.36373921
 2.75390173 1.37844751], Cost: 0.0667
Current parameters: [1.18858518 0.80928518 2.2126111  0.93651703 0.50630811 1.86613394
 2.68001175 0.19225169 1.16832253 2.96422452 1.15390993 2.37905113
 2.76428477 1.41062288], Cost: 0.0774
Current parameters: [1.21837833 0.85096174 2.24980289 0.88522133 0.58758514 1.84277322
 2.61785446 0.2173898  1.2043376  2.97618999 1.1602115  2.3390557
 2.7235511  1.38122056], Cost: 0.0795
Current parameters: [1.13004632 0.81008144 2.2197999  0.89526584 0.53454662 1.85014643
 2.66875448 0.21055211 1.17728275 2.96950165 1.18080027 2.37080721
 2.73891374 1.42235652], Cost: 0.0454
Current parameters: [1.19328067 0.80968367 2.2136672  0.91040455 0.52263544 1.85871077
 2.67059476 0.2106241  1.17776814 2.97547519 1.1905409  2.36156303
 2.73254969 1.43426914], Cost: 0.0256
Current parameters: [1.1875688  0.81120102 2.22474698 0.89632581 0.53729491 1.84104381
 2.67791098 0.16543731 1.16928644 2.95820043 1.17283128 2.33925878
 2.74877488 1.45672349], Cost: 0.1729
Current parameters: [1.18790463 0.81238811 2.21907865 0.89583583 0.52701925 1.85150107
 2.67619171 0.19094504 1.16927382 3.00009266 1.17641932 2.37197113
 2.74185878 1.42363855], Cost: 0.0415
Current parameters: [1.19517607 0.81052427 2.21904305 0.89799765 0.54185001 1.84847727
 2.66114922 0.2178928  1.12086884 2.96651122 1.18058859 2.38538445
```



```
 2.74278462 1.43135879], Cost: 0.0135
Current parameters: [1.19740344 0.81233943 2.22163756 0.89669999 0.54877938 1.84758153
 2.68739055 0.22325425 1.11865832 2.9615429  1.17896068 2.38121374
 2.73474226 1.42244672], Cost: 0.0127
Current parameters: [1.20502677 0.82019363 2.21286374 0.88950841 0.55672558 1.85223717
 2.69634617 0.26040555 1.13262343 2.97462317 1.19591968 2.41308284
 2.7537889  1.4314116 ], Cost: 0.0721
Current parameters: [1.193137   0.80733022 2.22154201 0.90030725 0.5416413  1.84967751
 2.69540731 0.21167319 1.11608677 2.95902316 1.18598548 2.39940061
 2.72271048 1.43379027], Cost: 0.0071
Current parameters: [1.20544558 0.81542811 2.2369534  0.90331722 0.55539448 1.85096386
 2.67090928 0.20955381 1.12779288 2.95118796 1.18299091 2.41715022
 2.68652329 1.40523857], Cost: 0.0338
Current parameters: [1.19121101 0.81030471 2.20152683 0.91119452 0.55601111 1.84630816
 2.69489225 0.20287038 1.11439004 2.96110909 1.19618546 2.39881713
 2.72392372 1.42777486], Cost: 0.0055
Current parameters: [1.19391838 0.81813844 2.18294552 0.87807375 0.52736706 1.85752603
 2.69363246 0.19510898 1.10566157 2.94569128 1.21853697 2.39601823
 2.72538249 1.40403234], Cost: 0.0072
Current parameters: [1.19731957 0.81509353 2.20837683 0.90297007 0.56741997 1.84434026
 2.69636891 0.19038517 1.12380499 2.94543209 1.19846235 2.40041398
 2.73478457 1.43481431], Cost: 0.0142
Current parameters: [1.18822962 0.81956954 2.19703034 0.91130954 0.55244136 1.84062264
 2.6928591  0.19389927 1.08480103 2.95959305 1.19906508 2.36416778
 2.69820339 1.4579973 ], Cost: 0.0632
Current parameters: [1.19111592 0.81518374 2.20010116 0.92911996 0.54565442 1.85559296
 2.69814341 0.19852177 1.10992328 2.95121111 1.18446556 2.4023649
 2.73376226 1.4213108 ], Cost: 0.0190
Current parameters: [1.20518512 0.80508967 2.20041103 0.91247447 0.55452176 1.84630524
 2.69569179 0.20079405 1.11282437 2.96400601 1.19644508 2.39826672
 2.72439482 1.4281998 ], Cost: 0.0074
Current parameters: [1.18159102 0.78271401 2.20359247 0.91070764 0.55967685 1.84774979
 2.69412058 0.20244771 1.11361643 2.9554706  1.1996912  2.39621579
 2.72682258 1.42128441], Cost: 0.0106
Current parameters: [1.19096467 0.81259743 2.21056916 0.91589676 0.55448518 1.84452203
 2.6952453  0.20198137 1.11179523 2.96363848 1.20600972 2.39726859
 2.72694392 1.42506406], Cost: 0.0045
Current parameters: [1.19083189 0.81123811 2.20713169 0.91831064 0.54476491 1.81642896
 2.69574027 0.2042067  1.11518398 2.95744803 1.20408253 2.39997788
 2.72755771 1.42243411], Cost: 0.0059
Current parameters: [1.1905687  0.81312122 2.21228558 0.91164962 0.55856417 1.84167957
```

```
 2.6943056  0.20059172 1.10033305 2.96540015 1.20226814 2.40300908
 2.73007518 1.42307639], Cost: 0.0078
Current parameters: [1.18474672 0.81250836 2.21222128 0.91056713 0.55020559 1.84248753
 2.70218357 0.18554008 1.12232763 2.98284203 1.19998147 2.39574331
 2.72876319 1.41841847], Cost: 0.0212
Current parameters: [1.18900003 0.81155847 2.20637028 0.9145798  0.5522079  1.84472464
 2.70152963 0.2059577  1.11060206 2.96658914 1.20840418 2.39676378
 2.73650883 1.43225429], Cost: 0.0019
Current parameters: [1.19137154 0.81335749 2.2037429  0.91433999 0.55602173 1.84748861
 2.70053893 0.22568307 1.11938751 2.96986432 1.21967397 2.41475047
 2.73419683 1.43008647], Cost: 0.0278
Current parameters: [1.18829908 0.80966591 2.21097373 0.92285992 0.55935532 1.84188008
 2.70187514 0.20775445 1.11257462 2.97038504 1.20288259 2.39727651
 2.73593379 1.43845178], Cost: 0.0048
Current parameters: [1.18534981 0.81418674 2.2023896  0.91308676 0.54805325 1.84259051
 2.70085345 0.20442072 1.09734271 2.9624641  1.21478744 2.37843377
 2.72503589 1.44682929], Cost: 0.0107
Current parameters: [1.18440814 0.79790697 2.20721858 0.91451985 0.553586   1.845624
 2.70118476 0.20581434 1.11003256 2.9629727  1.21044113 2.39477569
 2.73829771 1.42935323], Cost: 0.0012
Current parameters: [1.1851118  0.79853519 2.20786396 0.9140698  0.55555575 1.84532744
 2.70739103 0.20755498 1.10957755 2.96183348 1.20978718 2.39309349
 2.73673796 1.42674254], Cost: 0.0017
Current parameters: [1.18591044 0.79990851 2.20670233 0.9128627  0.55621826 1.84467113
 2.69522437 0.21154507 1.11115049 2.96439647 1.20768303 2.38548649
 2.7445623  1.42463707], Cost: 0.0028
Current parameters: [1.18314847 0.7975663  2.2056586  0.91855153 0.54986363 1.84725667
 2.70131229 0.20780857 1.10751921 2.96487171 1.20841379 2.39491786
 2.73737147 1.4270689 ], Cost: 0.0017
Current parameters: [1.18512937 0.80064887 2.20789558 0.91949022 0.55176943 1.84905426
 2.70264063 0.20089076 1.11043104 2.9525799  1.20791982 2.3948458
 2.74570121 1.42976417], Cost: 0.0044
Current parameters: [1.18379655 0.79863826 2.20228154 0.91708642 0.55706843 1.84474828
 2.70122191 0.20322493 1.10983721 2.96396181 1.21281914 2.394605
 2.73864805 1.42769769], Cost: 0.0012
Current parameters: [1.17973059 0.79773842 2.20364499 0.91339592 0.55309468 1.84449816
 2.70484804 0.194485   1.11476815 2.97170706 1.2103691  2.39183199
 2.74062792 1.42569361], Cost: 0.0072
Current parameters: [1.18383744 0.79901616 2.20313163 0.91647369 0.55956047 1.85177789
 2.70106601 0.20290981 1.10907221 2.96554214 1.21343428 2.39417686
 2.73845307 1.42829837], Cost: 0.0012
```



```
Current parameters: [1.18350809 0.79812851 2.19988153 0.91569927 0.55692215 1.84590363
 2.70039641 0.21234729 1.1145771  2.96264318 1.2211513  2.40214337
 2.73867194 1.42706485], Cost: 0.0052
Current parameters: [1.18324839 0.79610289 2.20476095 0.91376446 0.55609089 1.84407765
 2.69854652 0.20229677 1.10952048 2.96324727 1.21429492 2.39434941
 2.73706196 1.43242309], Cost: 0.0011
Current parameters: [1.1816146  0.79940387 2.19932321 0.91645114 0.55521902 1.8436991
 2.70047741 0.20379236 1.10263236 2.96056576 1.21811775 2.38529298
 2.73221595 1.43591097], Cost: 0.0028
Current parameters: [1.18966154 0.79408265 2.20257046 0.91567909 0.55584041 1.8423877
 2.6991735  0.20107437 1.10881925 2.96407625 1.21467105 2.39412418
 2.73773043 1.43156443], Cost: 0.0015
Current parameters: [1.18814162 0.79228301 2.21047724 0.90809259 0.55244624 1.85276931
 2.69815258 0.20443033 1.10907471 2.96535464 1.21635718 2.39304656
 2.73516321 1.43817011], Cost: 0.0007
Current parameters: [1.18803472 0.79228814 2.21122086 0.90534192 0.55403597 1.85215157
 2.69779339 0.20445844 1.10283443 2.96596421 1.21562882 2.39575106
 2.73675387 1.43774808], Cost: 0.0016
Current parameters: [1.1882546  0.79188516 2.207971   0.90633218 0.5522538  1.85341466
 2.69804185 0.20450205 1.10953814 2.9644588  1.21445141 2.39337473
 2.73449017 1.43833301], Cost: 0.0007
Current parameters: [1.18802073 0.7902296  2.20873139 0.90913778 0.55584681 1.85304702
 2.69826718 0.20635109 1.11031282 2.96575097 1.21102664 2.39381582
 2.7337393  1.44263628], Cost: 0.0011
Current parameters: [1.18842411 0.79172974 2.20831658 0.90588356 0.5530259  1.85292348
 2.69700728 0.20482693 1.10953745 2.96593374 1.21445999 2.39325655
 2.73159667 1.4368131 ], Cost: 0.0007
Current parameters: [1.18967315 0.7921658  2.21092175 0.90751433 0.55350061 1.85467773
 2.69437761 0.20240017 1.10976919 2.96062296 1.21326337 2.39386698
 2.72990246 1.43591941], Cost: 0.0021
Current parameters: [1.18787208 0.79151648 2.20811369 0.9065098  0.55229115 1.85321904
 2.69902395 0.20318856 1.10927907 2.96616811 1.21493312 2.3952193
 2.73001411 1.43744108], Cost: 0.0007
Current parameters: [1.18667104 0.79097178 2.20886076 0.90389718 0.5517473  1.85272744
 2.69791787 0.20103392 1.11203299 2.96997848 1.21302687 2.39026263
 2.73317321 1.436406  ], Cost: 0.0027
Current parameters: [1.18842411 0.79172974 2.20831658 0.90588356 0.5530259  1.85292348
 2.69700728 0.20482693 1.10953745 2.96593374 1.21445999 2.39325655
 2.73159667 1.4368131 ], Cost: 0.0007

   Return from subroutine COBYLA because the MAXFUN limit has been reached.
```



```
   NFVALS =  100   F = 6.904537E-04    MAXCV = 0.000000E+00
   X = 1.188424E+00   7.917297E-01   2.208317E+00   9.058836E-01   5.530259E-01
       1.852923E+00   2.697007E+00   2.048269E-01   1.109537E+00   2.965934E+00
       1.214460E+00   2.393257E+00   2.731597E+00   1.436813E+00
Simulating for QAOA depth p = 8
Current parameters: [3.39191447 0.35887484 0.16091156 0.27389166 2.0975049  2.5133086
 1.3477285  0.01100539 0.62836316 2.54723826 1.64848375 2.63481657
 2.67283283 1.42305269 0.25732562 2.69930823], Cost: 2.1369
Current parameters: [3.39191447 1.35887484 0.16091156 0.27389166 2.0975049  2.5133086
 1.3477285  0.01100539 0.62836316 2.54723826 1.64848375 2.63481657
 2.67283283 1.42305269 0.25732562 2.69930823], Cost: 1.6309
Current parameters: [3.39191447 1.35887484 1.16091156 0.27389166 2.0975049  2.5133086
 1.3477285  0.01100539 0.62836316 2.54723826 1.64848375 2.63481657
 2.67283283 1.42305269 0.25732562 2.69930823], Cost: 1.3902
Current parameters: [3.39191447 1.35887484 1.16091156 1.27389166 2.0975049  2.5133086
 1.3477285  0.01100539 0.62836316 2.54723826 1.64848375 2.63481657
 2.67283283 1.42305269 0.25732562 2.69930823], Cost: 1.8389
Current parameters: [3.39191447 1.35887484 1.16091156 0.27389166 3.0975049  2.5133086
 1.3477285  0.01100539 0.62836316 2.54723826 1.64848375 2.63481657
 2.67283283 1.42305269 0.25732562 2.69930823], Cost: 1.1862
Current parameters: [3.39191447 1.35887484 1.16091156 0.27389166 3.0975049  3.5133086
 1.3477285  0.01100539 0.62836316 2.54723826 1.64848375 2.63481657
 2.67283283 1.42305269 0.25732562 2.69930823], Cost: 1.2705
Current parameters: [3.39191447 1.35887484 1.16091156 0.27389166 3.0975049  2.5133086
 2.3477285  0.01100539 0.62836316 2.54723826 1.64848375 2.63481657
 2.67283283 1.42305269 0.25732562 2.69930823], Cost: 1.0294
Current parameters: [3.39191447 1.35887484 1.16091156 0.27389166 3.0975049  2.5133086
 2.3477285  1.01100539 0.62836316 2.54723826 1.64848375 2.63481657
 2.67283283 1.42305269 0.25732562 2.69930823], Cost: 2.8460
Current parameters: [3.39191447 1.35887484 1.16091156 0.27389166 3.0975049  2.5133086
 2.3477285  0.01100539 1.62836316 2.54723826 1.64848375 2.63481657
 2.67283283 1.42305269 0.25732562 2.69930823], Cost: 1.2152
Current parameters: [3.39191447 1.35887484 1.16091156 0.27389166 3.0975049  2.5133086
 2.3477285  0.01100539 0.62836316 3.54723826 1.64848375 2.63481657
 2.67283283 1.42305269 0.25732562 2.69930823], Cost: 2.0651
Current parameters: [3.39191447 1.35887484 1.16091156 0.27389166 3.0975049  2.5133086
 2.3477285  0.01100539 0.62836316 2.54723826 2.64848375 2.63481657
 2.67283283 1.42305269 0.25732562 2.69930823], Cost: 2.5187
Current parameters: [3.39191447 1.35887484 1.16091156 0.27389166 3.0975049  2.5133086
 2.3477285  0.01100539 0.62836316 2.54723826 1.64848375 3.63481657
```



```
 2.67283283 1.42305269 0.25732562 2.69930823], Cost: 2.1595
Current parameters: [3.39191447 1.35887484 1.16091156 0.27389166 3.0975049  2.5133086
 2.3477285  0.01100539 0.62836316 2.54723826 1.64848375 2.63481657
 3.67283283 1.42305269 0.25732562 2.69930823], Cost: 2.2364
Current parameters: [3.39191447 1.35887484 1.16091156 0.27389166 3.0975049  2.5133086
 2.3477285  0.01100539 0.62836316 2.54723826 1.64848375 2.63481657
 2.67283283 2.42305269 0.25732562 2.69930823], Cost: 2.0257
Current parameters: [3.39191447 1.35887484 1.16091156 0.27389166 3.0975049  2.5133086
 2.3477285  0.01100539 0.62836316 2.54723826 1.64848375 2.63481657
 2.67283283 1.42305269 1.25732562 2.69930823], Cost: 2.6364
Current parameters: [3.39191447 1.35887484 1.16091156 0.27389166 3.0975049  2.5133086
 2.3477285  0.01100539 0.62836316 2.54723826 1.64848375 2.63481657
 2.67283283 1.42305269 0.25732562 3.69930823], Cost: 2.6037
Current parameters: [ 3.39536077  1.48536512  1.22107823  0.16172301  3.1484852   2.4922351
  2.38694227 -0.4431197   0.58189785  2.28831649  1.27617236  2.35231243
  2.37110129  1.17398631 -0.14440457  2.30574869], Cost: 1.6723
Current parameters: [3.89191447 1.35887484 1.16091156 0.27389166 3.0975049  2.5133086
 2.3477285  0.01100539 0.62836316 2.54723826 1.64848375 2.63481657
 2.67283283 1.42305269 0.25732562 2.69930823], Cost: 0.7059
Current parameters: [ 4.05157595  1.48374322  1.22030675  0.16316127  3.14783152  2.49250531
  2.38643946 -0.43729677  0.58249364  2.29163646  1.28094625  2.35593478
  2.37497019  1.17717992 -0.13925346  2.31079503], Cost: 1.4574
Current parameters: [3.92558432 1.38520743 1.17343698 0.25054053 3.10811791 2.50892155
 2.35589198 0.47411027 0.61869007 2.49333625 1.57097643 2.57600521
 2.61001874 1.37120237 0.17369394 2.6173775 ], Cost: 2.0180
Current parameters: [3.89191447 1.25148864 1.38667295 0.27389166 3.0975049  2.5133086
 2.3477285  0.01100539 0.62836316 2.54723826 1.64848375 2.63481657
 2.67283283 1.42305269 0.25732562 2.69930823], Cost: 0.9511
Current parameters: [ 3.90109038  0.91661757  0.93514237  0.26752787  3.10039722  2.51211301
  2.34995326 -0.01833428  0.62572699  2.53254856  1.62736099  2.61878895
  2.65571436  1.40892213  0.23453382  2.67697998], Cost: 1.9100
Current parameters: [3.89191447 1.35887484 1.16091156 0.37733323 3.32510068 2.5133086
 2.3477285  0.01100539 0.62836316 2.54723826 1.64848375 2.63481657
 2.67283283 1.42305269 0.25732562 2.69930823], Cost: 0.9422
Current parameters: [ 3.89957189  1.38891058  1.16234355  0.72135897  2.88184448  2.51231087
  2.34958509 -0.01347897  0.62616324  2.5349795   1.63085652  2.6214413
  2.65854723  1.41126055  0.23830555  2.680675  ], Cost: 0.8543
Current parameters: [3.89191447 1.35887484 1.16091156 0.27389166 3.0975049  2.28563012
 2.3477285  0.01100539 0.7316226  2.54723826 1.64848375 2.63481657
 2.67283283 1.42305269 0.25732562 2.69930823], Cost: 1.0077
Current parameters: [3.89508098e+00 1.37129526e+00 1.16150372e+00 2.70292651e-01
```

```
 3.09405932e+00 2.72495369e+00 2.34849624e+00 8.80585718e-04
 1.08071751e+00 2.54216900e+00 1.64119451e+00 2.62928561e+00
 2.66692543e+00 1.41817639e+00 2.49460415e-01 2.69160299e+00], Cost: 2.7131
Current parameters: [3.89191447 1.35887484 1.16091156 0.27389166 3.0975049  2.5133086
 2.10054725 0.01100539 0.62836316 2.5098026  1.64848375 2.63481657
 2.67283283 1.42305269 0.25732562 2.69930823], Cost: 0.7826
Current parameters: [ 3.90758269  1.42033239  1.16384162  0.25608338  3.0804558   2.49932939
  2.2839159  -0.03909322  0.52675509  3.01819987  1.61241583  2.60744879
  2.64360238  1.39892422  0.21840774  2.66118188], Cost: 0.5371
Current parameters: [ 3.90758269  1.42033239  1.16384162  0.25608338  3.0804558   2.49932939
  2.2839159  -0.03909322  0.52675509  3.01819987  1.61241583  2.60744879
  2.64360238  1.39892422  0.39335903  2.48259844], Cost: 0.5346
Current parameters: [ 3.94199082  1.55529604  1.17027616  0.21697557  3.04301517  2.46863041
  2.30532688 -0.1491121   0.30361903  2.98579001  1.53320901  2.54734787
  2.57941096  1.34593697  0.6671132   2.75004522], Cost: 3.3666
Current parameters: [ 3.90758269  1.42033239  1.16384162  0.25608338  3.0804558   2.49932939
  2.2839159  -0.03909322  0.52675509  3.01819987  1.76982145  2.60744879
  2.44937707  1.39892422  0.39335903  2.48259844], Cost: 0.5826
Current parameters: [ 3.9251358   1.48918334  1.16712416  0.23613275  3.06135567  2.48366848
  2.29483859 -0.09521873  0.41292348  3.00166616  1.93963012  2.57678864
  2.91549521  1.37189307  0.24240826  2.33434051], Cost: 2.8227
Current parameters: [ 3.90758269  1.42033239  1.16384162  0.25608338  3.0804558   2.49932939
  2.2839159  -0.03909322  0.52675509  3.01819987  1.61241583  2.7727788
  2.64360238  1.21139826  0.39335903  2.48259844], Cost: 1.3060
Current parameters: [ 3.91770351  1.46003068  1.16573428  0.24458019  3.06944299  2.49029958
  2.29021374 -0.07145423  0.46112173  3.00866681  1.50826656  2.91758699
  2.56306534  1.73670498  0.30632339  2.39711545], Cost: 1.3983
Current parameters: [ 3.92231252  1.47231632  1.15594584  0.20621178  3.07076264  2.51537017
  2.32448609 -0.14321532  0.49079925  2.94705717  1.52508867  2.54118628
  2.57283001  1.34050468  0.29913157  2.39028744], Cost: 1.7798
Current parameters: [3.80954629 1.27761357 1.3219076  0.45054773 3.21719982 2.32715036
 2.03978685 0.01115592 0.6271673  3.1529145  1.54255741 2.51703522
 2.59328274 1.42393859 0.35922462 2.44880424], Cost: 0.3692
Current parameters: [3.81729859e+00 1.30363145e+00 1.32127156e+00 4.45641148e-01
 3.20699231e+00 2.43730078e+00 2.03537253e+00 2.15283799e-03
 8.35018301e-01 3.20656591e+00 1.55038403e+00 2.50535794e+00
 2.60259048e+00 1.46296326e+00 3.87093092e-01 2.47593918e+00], Cost: 0.8403
Current parameters: [3.80902606 1.36188322 1.36314366 0.44878253 3.21914497 2.32566472
 2.03960422 0.09113872 0.6264106  3.14717211 1.53377152 2.51036862
 2.5861624  1.41806107 0.34974449 2.43951693], Cost: 0.7636
Current parameters: [3.82696475 1.12763631 1.21589944 0.43765944 3.18910846 2.35413838
```



```
 2.06069796 0.16199923 0.6046956  3.17320157 1.51687388 2.48098997
 2.57525468 1.43851459 0.34976741 2.43938268], Cost: 0.3665
Current parameters: [3.94886456 1.12401358 1.22866553 0.44782452 3.19886097 2.34084928
 2.04713337 0.16004664 0.6070729  3.1753864  1.51546814 2.47992331
 2.57411543 1.43757419 0.35211158 2.44167916], Cost: 0.2394
Current parameters: [3.95713715 1.10061328 1.22021492 0.46334225 3.1952362  2.30633777
 2.02820871 0.12586244 0.52606004 3.19570001 1.49800834 2.43779216
 2.56482661 1.48129377 0.50840421 2.59451965], Cost: 1.4828
Current parameters: [3.95052993 1.13599707 1.2175168  0.5509547  3.13527561 2.35494771
 2.06380855 0.15472167 0.60132133 3.16689241 1.51314738 2.47701439
 2.57223462 1.43500957 0.34753635 2.43719699], Cost: 0.4352
Current parameters: [3.962087   1.08839216 1.2243278  0.4433465  3.21392219 2.32003673
 2.02288065 0.15210545 0.58144021 3.19953576 1.68515828 2.45968798
 2.71493149 1.47453678 0.28804803 2.37873332], Cost: 0.5480
Current parameters: [3.94985223 1.11352187 1.2590519  0.46051753 3.22818379 2.43631425
 2.01377301 0.15688859 0.5569718  3.18231349 1.50868804 2.49872978
 2.56862065 1.45415468 0.34611943 2.43580889], Cost: 0.3639
Current parameters: [3.95583607 1.11699293 1.21073436 0.43962811 3.18836227 2.27857713
 2.05525367 0.15422489 0.61437581 3.18100558 1.47586495 2.6382358
 2.54360005 1.60343406 0.31456908 2.40481137], Cost: 0.2946
Current parameters: [3.95010931 1.17306193 1.13512981 0.46444042 3.24036126 2.34091114
 2.00047476 0.15377563 0.60139382 3.18524509 1.5104566  2.47612063
 2.57005394 1.4342216  0.34670404 2.43638161], Cost: 0.2664
Current parameters: [3.97481186 1.08548846 1.22108364 0.38881459 3.19803582 2.32600788
 2.06567087 0.05571861 0.60892692 3.13805584 1.43159661 2.38086812
 2.51095834 1.43033618 0.24374964 2.3352504 ], Cost: 2.0970
Current parameters: [3.94991933 1.12815088 1.22886278 0.40907119 3.11508455 2.33990821
 1.96973849 0.15667403 0.60023268 3.19602048 1.51304006 2.49404038
 2.57214765 1.41784782 0.34949164 2.43911251], Cost: 0.4541
Current parameters: [3.95243471 1.07064441 1.21956777 0.47375252 3.25737816 2.32239005
 2.06855607 0.17569365 0.63589451 3.19297316 1.53035974 2.64499143
 2.58879788 1.27784732 0.35848207 2.4477731 ], Cost: 0.5321
Current parameters: [3.95371556 1.1410422  1.22823748 0.44222094 3.19319874 2.35105013
 2.08776057 0.14792472 0.6074167  3.29020337 1.50912297 2.473295
 2.56897313 1.43650971 0.34698753 2.43665933], Cost: 0.4720
Current parameters: [3.96142519 1.1152461  1.21395406 0.44243352 3.20100573 2.44042349
 2.02691011 0.16458856 0.82071284 3.16693551 1.54067571 2.48172785
 2.59615437 1.48628546 0.38758674 2.47634227], Cost: 0.5036
Current parameters: [3.94907279 1.1293988  1.23074433 0.44724427 3.19864341 2.34118082
 2.04771844 0.16470456 0.60678254 3.17471431 1.43654769 2.47974557
 2.67075636 1.43716182 0.35162871 2.44120612], Cost: 0.1058
```



```
Current parameters: [3.95923691 1.26571035 1.29886378 0.44049158 3.22278448 2.32088337
 2.05142131 0.35752911 0.60922033 3.15141849 1.44145106 2.48263273
 2.67253758 1.43957501 0.33834375 2.42802678], Cost: 0.4659
Current parameters: [3.94907176 1.12935167 1.23072449 0.44727499 3.19870246 2.34119082
 2.04777112 0.1646631  0.60680978 3.17471694 1.43655143 2.48003866
 2.67076379 1.43683999 0.43911261 2.35192361], Cost: 0.1306
Current parameters: [3.95152291 1.07239562 1.22125963 0.47404395 3.25733622 2.31980062
 2.0630175  0.17982499 0.63161049 3.17464442 1.44482577 2.64486096
 2.69434943 1.27755714 0.35619402 2.44972828], Cost: 0.4834
Current parameters: [3.92556917 1.09494284 1.26886644 0.49473948 3.23146735 2.30901361
 1.98566595 0.17560389 0.65115253 3.20766079 1.42065506 2.457125
 2.65955757 1.44684907 0.34627641 2.43586814], Cost: 0.1458
Current parameters: [3.97155605 1.13609125 1.20106533 0.42239338 3.1878629  2.45547172
 2.05246165 0.16724199 0.80648031 3.15241801 1.46429061 2.49120336
 2.70332506 1.48247099 0.38885597 2.47950694], Cost: 0.5953
Current parameters: [3.97150036 1.09878292 1.24553843 0.43754324 3.21960745 2.30755549
 2.05546679 0.16679262 0.6064785  3.1567299  1.53159331 2.46052959
 2.71627621 1.44986279 0.33977356 2.42951106], Cost: 0.1251
Current parameters: [3.88127575 1.16517239 1.16642945 0.50074103 3.15812133 2.38930204
 1.98454453 0.15553881 0.53482743 3.26372092 1.4465919  2.51943356
 2.81372365 1.48933347 0.30657921 2.40088071], Cost: 0.3330
Current parameters: [3.94840274 1.12118384 1.22626753 0.45162074 3.21022569 2.31232538
 2.04231324 0.162255   0.56631639 3.17355127 1.42950429 2.47583329
 2.68252185 1.4664865  0.42810125 2.51844392], Cost: 0.2944
Current parameters: [3.9534384  1.09188563 1.20424598 0.44400376 3.19162398 2.34792471
 2.05295881 0.20237661 0.60116646 3.17976042 1.43010454 2.47068231
 2.66622116 1.4407908  0.34922667 2.43881366], Cost: 0.1343
Current parameters: [3.95881097 1.11969015 1.2263923  0.42495244 3.22350582 2.31817022
 2.05892795 0.1169337  0.58529354 3.1295146  1.3815448  2.43470081
 2.67317947 1.44083996 0.30487219 2.40120617], Cost: 0.6739
Current parameters: [3.95411586 1.12823198 1.23189491 0.44398728 3.19917143 2.32177695
 2.05343932 0.16937637 0.61073409 3.16985739 1.43146581 2.5176052
 2.65955429 1.47799256 0.34303518 2.43276129], Cost: 0.0416
Current parameters: [3.95695811 1.19988879 1.26619829 0.44176873 3.2106183  2.31178003
 2.053947   0.26323003 0.60365903 3.15981404 1.4333294  2.51385483
 2.66353534 1.47464125 0.33649717 2.42801232], Cost: 0.0992
Current parameters: [3.95315394 1.13311032 1.23683487 0.44821708 3.2086644  2.32395667
 2.05908727 0.16234192 0.61302835 3.16867236 1.42841348 2.54729845
 2.66857282 1.42717237 0.33945701 2.44216756], Cost: 0.0429
Current parameters: [3.95807867 1.06625484 1.20817386 0.45674788 3.22753591 2.28249268
 2.04918394 0.21337731 0.61233803 3.17236593 1.44466222 2.58883812
```



```
 2.65634273 1.50261128 0.35708648 2.40688379], Cost: 0.2223
Current parameters: [3.95938599 1.13530422 1.22521443 0.43762765 3.19920261 2.3492987
 2.05473454 0.17054885 0.65959851 3.16205889 1.43476709 2.52442286
 2.67267865 1.48862617 0.35137259 2.4438122 ], Cost: 0.0581
Current parameters: [3.98238369 1.13001821 1.23160527 0.45007496 3.19798198 2.32327107
 2.04606161 0.16817485 0.60819066 3.1765165  1.42754505 2.51841036
 2.66267496 1.47815743 0.3425291  2.43187733], Cost: 0.0333
Current parameters: [3.96320554 1.14398346 1.2180725  0.46283948 3.18658933 2.3378435
 2.03144807 0.16424709 0.59319958 3.19857927 1.43108727 2.52987515
 2.69841361 1.48812686 0.33087429 2.42424462], Cost: 0.0586
Current parameters: [3.98203904 1.12470103 1.24279208 0.44950051 3.21029599 2.34441258
 2.04124768 0.16800837 0.59765371 3.1716222  1.4302066  2.52251718
 2.65787544 1.48244002 0.34177599 2.43172842], Cost: 0.0271
Current parameters: [3.9833596  1.10523789 1.28386631 0.44835977 3.1770872  2.34714077
 2.05959593 0.17179268 0.59932379 3.17962116 1.42128364 2.52798811
 2.67145596 1.48588404 0.34360769 2.43201708], Cost: 0.0220
Current parameters: [3.98402082 1.10730569 1.28362392 0.45567784 3.19110961 2.34904669
 2.08091945 0.17049369 0.60058105 3.19480567 1.41992395 2.52425141
 2.67181718 1.4895072  0.3432357  2.4315288 ], Cost: 0.0176
Current parameters: [3.9855002  1.11306076 1.28898392 0.4161305  3.18806707 2.35104594
 2.07146082 0.1666093  0.60334128 3.23372545 1.43454231 2.53048423
 2.6527498  1.48538425 0.34203021 2.4333947 ], Cost: 0.0721
Current parameters: [3.98327015 1.11064211 1.28235561 0.4727264  3.17941871 2.3535784
 2.08257606 0.17186012 0.60129115 3.19726666 1.43576445 2.52768378
 2.65716676 1.48804986 0.34644388 2.43533222], Cost: 0.0348
Current parameters: [3.99313273 1.12016595 1.29116448 0.45801524 3.18777732 2.3541509
 2.09492152 0.14185134 0.60115965 3.17193282 1.40181155 2.51562674
 2.66323701 1.49102992 0.30901926 2.41231253], Cost: 0.0795
Current parameters: [3.99057651 1.11166573 1.27288492 0.44413063 3.18399535 2.35342114
 2.09610968 0.17078591 0.58763872 3.18667275 1.42416566 2.53101569
 2.67486858 1.49228995 0.34961305 2.43472455], Cost: 0.0153
Current parameters: [3.98916168 1.12118231 1.28557283 0.45149115 3.1928337  2.34312275
 2.08987725 0.15231592 0.57505409 3.18328797 1.40847507 2.52412495
 2.68178884 1.50204517 0.3755259  2.47663313], Cost: 0.0818
Current parameters: [3.99232015 1.10371616 1.26937472 0.44378306 3.18511776 2.34961366
 2.09723199 0.17733993 0.58746627 3.18640132 1.4261464  2.53631039
 2.67438473 1.49811394 0.32857538 2.45286609], Cost: 0.0083
Current parameters: [3.99873166 1.07461333 1.26303317 0.45080317 3.20124817 2.33181768
 2.09908251 0.19030546 0.58959418 3.18101664 1.42342867 2.57267515
 2.67586581 1.51332052 0.33866855 2.43091969], Cost: 0.0432
Current parameters: [3.98900057 1.11421849 1.26754178 0.4448491  3.18142211 2.35428133
```

```
 2.09682085 0.19269683 0.58643356 3.18902144 1.40559013 2.54237488
 2.66391853 1.4993504  0.32916402 2.45364907], Cost: 0.0153
Current parameters: [4.00022103 1.13083078 1.29254519 0.44175103 3.19782193 2.33778895
 2.09981973 0.21283264 0.58500346 3.17466362 1.44813391 2.5292328
 2.69037151 1.49706537 0.32236584 2.44763563], Cost: 0.0493
Current parameters: [3.98971684 1.09171394 1.26780061 0.44821459 3.19780873 2.35214754
 2.09907138 0.1859104  0.59112347 3.19488166 1.41638035 2.52073669
 2.68420568 1.49183383 0.32878906 2.45247288], Cost: 0.0092
Current parameters: [4.00426623 1.08121229 1.24624935 0.43611161 3.15651781 2.37030819
 2.09834631 0.18971043 0.5711929  3.17102662 1.43768055 2.5237957
 2.66678494 1.47795683 0.32964982 2.45549533], Cost: 0.0548
Current parameters: [3.99583767 1.10610512 1.26580242 0.44054002 3.18490027 2.3636187
 2.0983486  0.17771511 0.61157172 3.18141566 1.42750546 2.53999358
 2.68117214 1.503518   0.33232755 2.45805585], Cost: 0.0213
Current parameters: [3.99230717 1.10267712 1.26796771 0.44288947 3.18260175 2.34925623
 2.09563607 0.17904601 0.58670161 3.18697886 1.42695488 2.52904418
 2.67259131 1.51092971 0.32931917 2.45041992], Cost: 0.0090
Current parameters: [3.98358052 1.10910069 1.26232773 0.45029873 3.17923431 2.35720907
 2.09073894 0.17481758 0.57897906 3.19591848 1.42741984 2.54333335
 2.69302912 1.50180445 0.32182697 2.44863291], Cost: 0.0173
Current parameters: [3.98373617 1.10105074 1.2739462  0.44173389 3.19041    2.35764317
 2.09750632 0.17763218 0.58400178 3.18244954 1.42838489 2.53771631
 2.67152816 1.49978163 0.32842464 2.4530705 ], Cost: 0.0086
Current parameters: [3.97025405 1.10413195 1.26237196 0.43925212 3.17814075 2.33569772
 2.10668268 0.17809207 0.5950684  3.1828275  1.42736011 2.53360884
 2.67535284 1.49540386 0.32872265 2.45310183], Cost: 0.0193
Current parameters: [3.99262852 1.09897695 1.2798097  0.44236755 3.17709114 2.35027338
 2.10152022 0.17819213 0.58807725 3.18971941 1.42418486 2.53773726
 2.6773377  1.49886768 0.32896646 2.45295706], Cost: 0.0111
Current parameters: [3.9929766  1.10421761 1.26859286 0.42382294 3.19090945 2.34901064
 2.09212825 0.17472913 0.58907591 3.20678778 1.43072279 2.53793713
 2.66700228 1.49597776 0.32568579 2.45192334], Cost: 0.0193
Current parameters: [3.99372421 1.10422237 1.26954545 0.4435679  3.1851056  2.35161326
 2.10078642 0.17499726 0.59026796 3.18436709 1.4243016  2.53502983
 2.67238789 1.49706725 0.31843824 2.44312742], Cost: 0.0092
Current parameters: [3.99571858 1.10648704 1.27422906 0.44615905 3.19255208 2.3451113
 2.09843285 0.15711304 0.58056084 3.18110355 1.4126918  2.53086543
 2.67437677 1.50238974 0.32567104 2.46421831], Cost: 0.0156
Current parameters: [3.99625606 1.09833779 1.26371424 0.44213553 3.17926051 2.35404958
 2.0971224  0.17815546 0.58137788 3.18210838 1.42766273 2.53235801
 2.67182752 1.49298185 0.32854522 2.45486813], Cost: 0.0141
```



```
Current parameters: [3.99220929 1.09887979 1.26700674 0.44482713 3.18552632 2.34897738
 2.09712635 0.17554423 0.58796473 3.18696402 1.42428187 2.5401084
 2.67311025 1.49951441 0.3300961  2.4512039 ], Cost: 0.0076
Current parameters: [3.996943   1.09778223 1.26968306 0.44537409 3.19221076 2.34253534
 2.09764673 0.18193909 0.58445595 3.18214526 1.42711232 2.54437712
 2.67602084 1.50167422 0.33025446 2.44791867], Cost: 0.0057
Current parameters: [3.99788868 1.09900276 1.26813051 0.44649698 3.1932827  2.3438077
 2.10404762 0.18181096 0.58305049 3.1842924  1.42849125 2.54475544
 2.67548534 1.50266849 0.33124214 2.44850442], Cost: 0.0053
Current parameters: [3.99797209 1.0980362  1.27150239 0.45806021 3.19466886 2.34195118
 2.10159681 0.18259279 0.58491826 3.18708076 1.43403705 2.54448821
 2.66904154 1.50198631 0.32996085 2.44914871], Cost: 0.0065
Current parameters: [4.0025862  1.09929867 1.26886191 0.44684623 3.19455042 2.34864575
 2.10244024 0.18171063 0.5854368  3.18418813 1.42850608 2.54587177
 2.67643212 1.50403134 0.33177139 2.44929862], Cost: 0.0051
Current parameters: [3.99918943 1.09907346 1.26573506 0.44658016 3.19596594 2.34609384
 2.10650951 0.18314651 0.59655588 3.17800313 1.43151372 2.54771465
 2.67910889 1.5061755  0.3330192  2.45211235], Cost: 0.0160
Current parameters: [4.0043373  1.09832942 1.27019799 0.44255906 3.19647266 2.34713649
 2.10294386 0.18182534 0.58730254 3.18550186 1.4288949  2.54478499
 2.67175854 1.50301066 0.33266821 2.44992882], Cost: 0.0041

   Return from subroutine COBYLA because the MAXFUN limit has been reached.

   NFVALS =  100   F = 5.091256E-03    MAXCV = 0.000000E+00
   X = 4.002586E+00   1.099299E+00   1.268862E+00   4.468462E-01   3.194550E+00
       2.348646E+00   2.102440E+00   1.817106E-01   5.854368E-01   3.184188E+00
       1.428506E+00   2.545872E+00   2.676432E+00   1.504031E+00   3.317714E-01
       2.449299E+00
Current parameters: [4.0025862  1.09929867 1.26886191 0.44684623 3.19455042 2.34864575
 2.10244024 0.18171063 0.5854368  3.18418813 1.42850608 2.54587177
 2.67643212 1.50403134 0.33177139 2.44929862], Cost: 0.0051
Simulating for QAOA depth p = 9
Current parameters: [4.0370518  2.12527307 2.77724725 1.63936009 1.11509891 0.38994505
 0.35511349 1.3108016  2.48892931 0.27301043 1.3488091  0.97556289
 2.27753357 0.7258057  1.78187144 0.33838351 2.98975714 0.25193692], Cost: 2.7929
Current parameters: [3.0370518  3.12527307 2.77724725 1.63936009 1.11509891 0.38994505
 0.35511349 1.3108016  2.48892931 0.27301043 1.3488091  0.97556289
 2.27753357 0.7258057  1.78187144 0.33838351 2.98975714 0.25193692], Cost: 1.4242
Current parameters: [3.0370518  3.12527307 3.77724725 1.63936009 1.11509891 0.38994505
 0.35511349 1.3108016  2.48892931 0.27301043 1.3488091  0.97556289
```



```
 2.27753357 0.7258057  1.78187144 0.33838351 2.98975714 0.25193692], Cost: 1.1283
Current parameters: [3.0370518  3.12527307 3.77724725 2.63936009 1.11509891 0.38994505
 0.35511349 1.3108016  2.48892931 0.27301043 1.3488091  0.97556289
 2.27753357 0.7258057  1.78187144 0.33838351 2.98975714 0.25193692], Cost: 0.8199
Current parameters: [3.0370518  3.12527307 3.77724725 2.63936009 2.11509891 0.38994505
 0.35511349 1.3108016  2.48892931 0.27301043 1.3488091  0.97556289
 2.27753357 0.7258057  1.78187144 0.33838351 2.98975714 0.25193692], Cost: 2.3849
Current parameters: [3.0370518  3.12527307 3.77724725 2.63936009 1.11509891 1.38994505
 0.35511349 1.3108016  2.48892931 0.27301043 1.3488091  0.97556289
 2.27753357 0.7258057  1.78187144 0.33838351 2.98975714 0.25193692], Cost: 0.5919
Current parameters: [3.0370518  3.12527307 3.77724725 2.63936009 1.11509891 1.38994505
 1.35511349 1.3108016  2.48892931 0.27301043 1.3488091  0.97556289
 2.27753357 0.7258057  1.78187144 0.33838351 2.98975714 0.25193692], Cost: 1.9621
Current parameters: [3.0370518  3.12527307 3.77724725 2.63936009 1.11509891 1.38994505
 0.35511349 2.3108016  2.48892931 0.27301043 1.3488091  0.97556289
 2.27753357 0.7258057  1.78187144 0.33838351 2.98975714 0.25193692], Cost: 1.4035
Current parameters: [3.0370518  3.12527307 3.77724725 2.63936009 1.11509891 1.38994505
 0.35511349 1.3108016  3.48892931 0.27301043 1.3488091  0.97556289
 2.27753357 0.7258057  1.78187144 0.33838351 2.98975714 0.25193692], Cost: 1.6920
Current parameters: [3.0370518  3.12527307 3.77724725 2.63936009 1.11509891 1.38994505
 0.35511349 1.3108016  2.48892931 1.27301043 1.3488091  0.97556289
 2.27753357 0.7258057  1.78187144 0.33838351 2.98975714 0.25193692], Cost: 0.4919
Current parameters: [3.0370518  3.12527307 3.77724725 2.63936009 1.11509891 1.38994505
 0.35511349 1.3108016  2.48892931 1.27301043 2.3488091  0.97556289
 2.27753357 0.7258057  1.78187144 0.33838351 2.98975714 0.25193692], Cost: 0.6015
Current parameters: [3.0370518  3.12527307 3.77724725 2.63936009 1.11509891 1.38994505
 0.35511349 1.3108016  2.48892931 1.27301043 1.3488091  1.97556289
 2.27753357 0.7258057  1.78187144 0.33838351 2.98975714 0.25193692], Cost: 1.8128
Current parameters: [3.0370518  3.12527307 3.77724725 2.63936009 1.11509891 1.38994505
 0.35511349 1.3108016  2.48892931 1.27301043 1.3488091  0.97556289
 3.27753357 0.7258057  1.78187144 0.33838351 2.98975714 0.25193692], Cost: 2.4872
Current parameters: [3.0370518  3.12527307 3.77724725 2.63936009 1.11509891 1.38994505
 0.35511349 1.3108016  2.48892931 1.27301043 1.3488091  0.97556289
 2.27753357 1.7258057  1.78187144 0.33838351 2.98975714 0.25193692], Cost: 1.5275
Current parameters: [3.0370518  3.12527307 3.77724725 2.63936009 1.11509891 1.38994505
 0.35511349 1.3108016  2.48892931 1.27301043 1.3488091  0.97556289
 2.27753357 0.7258057  2.78187144 0.33838351 2.98975714 0.25193692], Cost: 1.9640
Current parameters: [3.0370518  3.12527307 3.77724725 2.63936009 1.11509891 1.38994505
 0.35511349 1.3108016  2.48892931 1.27301043 1.3488091  0.97556289
 2.27753357 0.7258057  1.78187144 1.33838351 2.98975714 0.25193692], Cost: 1.9832
Current parameters: [3.0370518  3.12527307 3.77724725 2.63936009 1.11509891 1.38994505
```



```
 0.35511349 1.3108016  2.48892931 1.27301043 1.3488091  0.97556289
 2.27753357 0.7258057  1.78187144 0.33838351 3.98975714 0.25193692], Cost: 2.0845
Current parameters: [3.0370518  3.12527307 3.77724725 2.63936009 1.11509891 1.38994505
 0.35511349 1.3108016  2.48892931 1.27301043 1.3488091  0.97556289
 2.27753357 0.7258057  1.78187144 0.33838351 2.98975714 1.25193692], Cost: 1.0332
Current parameters: [2.82062944 3.20325145 3.84089465 2.70569589 0.77847514 1.43897508
 0.06039452 1.13624291 2.25230862 1.29452658 1.3252261  0.69145341
 1.84835813 0.50304923 1.46524294 0.01761712 2.64720848 0.13550299], Cost: 0.7915
Current parameters: [2.61647471 3.12527307 3.77724725 2.63936009 1.3854966  1.38994505
 0.35511349 1.3108016  2.48892931 1.27301043 1.3488091  0.97556289
 2.27753357 0.7258057  1.78187144 0.33838351 2.98975714 0.25193692], Cost: 0.7830
Current parameters: [3.58068097 3.15721058 3.80331524 2.66652917 1.86582258 1.41002622
 0.23440578 1.2393078  2.39201685 1.28182278 1.33915024 0.8592005
 2.10175668 0.63457159 1.65219027 0.20700759 2.84945988 0.20424921], Cost: 1.7031
Current parameters: [3.0370518  3.60864007 3.77724725 2.63936009 1.11509891 1.38994505
 0.48300542 1.3108016  2.48892931 1.27301043 1.3488091  0.97556289
 2.27753357 0.7258057  1.78187144 0.33838351 2.98975714 0.25193692], Cost: 0.7685
Current parameters: [3.0324919  2.84356431 3.79785911 2.66084257 1.03301824 1.40582316
 1.26919785 1.25427174 2.41230101 1.27997832 1.34117188 0.88355561
 2.13854748 0.65366725 1.67933305 0.23450509 2.87882465 0.21423044], Cost: 1.7569
Current parameters: [3.03576444 3.12025858 3.78306642 2.64542505 1.09192579 1.39442778
 0.33153891 1.80364202 2.46729551 1.27497761 1.34665295 0.94958727
 2.23829483 0.70543948 1.75292266 0.30905642 2.95843853 0.24129158], Cost: 0.7623
Current parameters: [3.0370518  3.12527307 3.59685292 2.81244355 1.11509891 1.38994505
 0.35511349 1.3108016  2.48892931 1.27301043 1.3488091  0.97556289
 2.27753357 0.7258057  1.78187144 0.33838351 2.98975714 0.25193692], Cost: 0.4181
Current parameters: [3.03637296 3.12262883 3.24985693 2.45521183 1.10287929 1.39230888
 0.34268217 1.30607493 2.4775214  1.27404776 1.34767212 0.96186545
 2.25684224 0.71506621 1.7666062  0.32291878 2.97324224 0.24632343], Cost: 1.1236
Current parameters: [3.0370518  3.12527307 3.59685292 2.81244355 1.11509891 1.14514515
 0.35511349 1.3108016  2.43820448 1.27301043 1.3488091  0.97556289
 2.27753357 0.7258057  1.78187144 0.33838351 2.98975714 0.25193692], Cost: 0.4219
Current parameters: [3.03525309 3.11826676 3.61691543 2.84506764 1.08272115 1.29187021
 0.32217482 1.29827755 2.964294   1.275759   1.3457965  0.93926945
 2.22270874 0.69734978 1.74142387 0.29740735 2.94599841 0.23706312], Cost: 1.1992
Current parameters: [2.93614997 3.14247001 3.67541868 2.92364793 0.88411831 1.43335269
 0.14139437 1.20170174 2.28457829 1.27989    1.34126868 0.88472184
 2.14030918 0.65458164 1.68063277 0.23582179 2.88023077 0.21470838], Cost: 2.1463
Current parameters: [3.17357794 3.12291331 3.60988225 2.83281849 1.31029285 1.39601555
 0.32956383 1.29972084 2.46082318 1.27460485 1.34706153 0.95450943
 2.24573025 0.70929871 1.75840822 0.31461366 2.96437315 0.24330878], Cost: 0.5145
```

```
Current parameters: [3.03684064 3.12393821 3.59696547 2.81256086 1.11477047 1.39003175
 0.36015857 1.31049293 2.48851089 1.39789962 1.3488091  0.97556289
 2.27753357 0.7258057  1.78187144 0.33838351 2.98975714 0.25193692], Cost: 0.4531
Current parameters: [3.04334808 3.05567821 3.6083645  2.83089696 1.10859081 1.39504464
 0.58540158 1.30265104 2.46544929 1.25858614 1.34714888 0.95556184
 2.24732    0.71012385 1.75958108 0.31580185 2.96564202 0.24374008], Cost: 0.5017
Current parameters: [3.0370518  3.12527307 3.59685292 2.81244355 1.11509891 1.38994505
 0.35511349 1.3108016  2.48892931 1.27301043 1.22423752 0.98590317
 2.27753357 0.7258057  1.78187144 0.33838351 2.98975714 0.25193692], Cost: 0.3986
Current parameters: [3.04683787 3.11802557 3.60102931 2.82087458 1.12002201 1.39078176
 0.3618189  1.31414341 2.48560572 1.26999719 1.24318723 1.23223574
 2.25844653 0.71589889 1.76778978 0.32411783 2.97452272 0.24675867], Cost: 0.8796
Current parameters: [3.0370518  3.12527307 3.59685292 2.81244355 1.11509891 1.38994505
 0.35511349 1.3108016  2.48892931 1.27301043 1.22423752 0.98590317
 2.33511825 0.61485975 1.78187144 0.33838351 2.98975714 0.25193692], Cost: 0.2170
Current parameters: [3.05507173 3.11192763 3.60454326 2.82796833 1.12416423 1.39148575
 0.36746074 1.31695515 2.48280929 1.26746189 1.21977933 0.96541566
 2.5606981  0.70310811 1.75594168 0.31211489 2.9617047  0.24240176], Cost: 1.2248
Current parameters: [3.0370518  3.12527307 3.59685292 2.81244355 1.11509891 1.38994505
 0.35511349 1.3108016  2.48892931 1.27301043 1.22423752 0.98590317
 2.33511825 0.61485975 1.87083172 0.25057082 2.98975714 0.25193692], Cost: 0.2412
Current parameters: [3.05317051 3.11333566 3.60373188 2.82633036 1.12320778 1.39132319
 0.36615802 1.31630591 2.48345499 1.2680473  1.2202497  0.96757722
 2.30972848 0.57588874 1.94940254 0.51244904 2.96466441 0.24340778], Cost: 0.5364
Current parameters: [3.0370518  3.12527307 3.59685292 2.81244355 1.11509891 1.38994505
 0.35511349 1.3108016  2.48892931 1.27301043 1.22423752 0.98590317
 2.33511825 0.61485975 1.78187144 0.33838351 3.02998488 0.1335869 ], Cost: 0.5003
Current parameters: [3.05103283 3.11491881 3.60281958 2.82448869 1.12213238 1.39114042
 0.36469329 1.31557592 2.484181   1.26870551 1.22077857 0.97000762
 2.31309569 0.5810571  1.7773219  0.3375433  3.20964178 0.35939476], Cost: 0.4566
Current parameters: [2.96568432 3.12428686 3.59580974 2.81135631 1.16528509 1.38914145
 0.35884085 1.2224517  2.49280754 1.27265778 1.22462405 0.99055974
 2.34215245 0.61851073 1.78706099 0.34364089 2.99537152 0.25384528], Cost: 0.1497
Current parameters: [2.81376491 3.12110485 3.60063752 2.81920964 1.26272233 1.39121344
 0.35655474 1.38891774 2.48330229 1.27122821 1.22223078 0.97420993
 2.3184178  0.59535721 1.77521556 0.3334658  2.97794201 0.26376562], Cost: 0.2113
Current parameters: [2.96582998 3.2382467  3.59495006 2.81046032 1.16790703 1.39697843
 0.39151022 1.22346589 2.454986   1.2724352  1.22486801 0.99349877
 2.34659216 0.62081509 1.79033643 0.34695913 2.9989151  0.25504976], Cost: 0.1034
Current parameters: [2.96779931 3.31780278 3.6058249  2.82775578 1.1681189  1.35314853
 0.41133851 1.20910618 2.66755487 1.26925314 1.21998909 0.96109318
```



```
 2.29967557 0.57357386 1.76769179 0.32787456 2.9646679  0.27688413], Cost: 0.0799
Current parameters: [2.95901612 3.31989937 3.68898729 2.91443086 1.15033009 1.35638658
 0.39512348 1.20047686 2.65192794 1.26963665 1.21956873 0.95602901
 2.29202565 0.5696033  1.76204798 0.32215699 2.95856207 0.27480873], Cost: 0.0450
Current parameters: [2.92499009 3.33577993 3.64963227 2.88615214 1.08769507 1.37317838
 0.33752281 1.13759885 2.57312145 1.26317312 1.20877452 0.88234249
 2.18506403 0.46517328 1.70871146 0.2763633  2.88002712 0.31964714], Cost: 1.4355
Current parameters: [2.97896331 3.31891212 3.69025247 2.91574949 1.13873541 1.35671876
 0.395607   1.22340941 2.6503248  1.2696585  1.227995   1.05754205
 2.29025696 0.53285993 1.72598528 0.33088616 2.92840908 0.29814369], Cost: 0.1173
Current parameters: [2.89643852 3.32833235 3.70438513 2.93978968 1.21880706 1.3477435
 0.41910819 1.13180646 2.69527065 1.26265924 1.22648698 1.08056319
 2.25938245 0.64445527 1.88040292 0.28693533 3.03982965 0.23514889], Cost: 0.2307
Current parameters: [2.96251485 3.32406955 3.6992439  2.93042744 1.16408597 1.35200162
 0.4081707  1.20818049 2.67401945 1.26578175 1.21708523 0.94958597
 2.40199439 0.60501082 1.75753082 0.32264741 2.94531664 0.30131856], Cost: 0.1189
Current parameters: [2.93204473 3.31952158 3.68628442 2.91161382 1.10604402 1.35519965
 0.40107696 1.20602633 2.6576561  1.26926886 1.21980547 0.95888109
 2.2972059  0.56828374 1.78214252 0.34757836 2.9587889  0.27864335], Cost: 0.0302
Current parameters: [2.97375108 3.31642838 3.69616243 2.92359025 1.15160837 1.35553195
 0.40478211 1.21474285 2.65634688 1.26816877 1.21816955 0.94660999
 2.2872127  0.54450594 1.84312197 0.42577484 2.93290417 0.29066199], Cost: 0.3233
Current parameters: [2.93306765 3.30819472 3.65442527 2.94698093 1.10498368 1.35599303
 0.43856079 1.20469896 2.65382723 1.26761136 1.21953544 0.95562794
 2.29229171 0.56573311 1.77851703 0.34390549 2.95486661 0.27731015], Cost: 0.0302
Current parameters: [2.93142737 3.29457667 3.75142875 2.86232263 1.10473625 1.35626818
 0.48993435 1.20194229 2.65263596 1.26485568 1.21894433 0.95195764
 2.28891274 0.56362257 1.77416715 0.33666965 2.95192229 0.27821634], Cost: 0.0416
Current parameters: [2.93224126 3.31947155 3.68631601 2.91167578 1.10598977 1.41639258
 0.40111882 1.20620718 2.6703037  1.26926708 1.21979814 0.95879274
 2.29714051 0.56799936 1.78196182 0.34771172 2.95985242 0.27923962], Cost: 0.0111
Current parameters: [2.94585742 3.31703934 3.6909527  2.91899442 1.10274985 1.41402982
 0.40748733 1.22149021 2.67314471 1.26823726 1.21875325 0.95158453
 2.29596278 0.54352262 1.76586937 0.35644845 3.06237023 0.33890984], Cost: 0.1425
Current parameters: [2.95413284 3.31940554 3.6856262  2.91053414 1.09181261 1.41610793
 0.40351238 1.18017838 2.67160342 1.31923995 1.22000675 0.96130585
 2.30105143 0.57097176 1.78425186 0.34884088 2.96207095 0.27911017], Cost: 0.0226
Current parameters: [2.87032941 3.31895331 3.68934267 2.91575088 1.14437239 1.41704851
 0.40462976 1.27343861 2.67019689 1.34370161 1.21903015 0.95296096
 2.29001984 0.56200911 1.77674667 0.34207652 2.95381586 0.28089719], Cost: 0.0199
Current parameters: [2.93126751 3.31945809 3.68630178 2.91166094 1.10667451 1.41638162
```



```
 0.40116968 1.20500172 2.67035661 1.26926227 1.28181029 0.95370929
 2.2964508  0.56956293 1.78324641 0.34658532 2.95828241 0.28411   ], Cost: 0.0052
Current parameters: [2.94130446 3.32553081 3.6903891  2.91513717 1.09542118 1.41569058
 0.40568214 1.2255334  2.6844928  1.26872613 1.29409538 0.95449436
 2.32445496 0.54180956 1.75780442 0.36196774 2.99308207 0.17976207], Cost: 0.0862
Current parameters: [2.91305518 3.31920642 3.68603557 2.91138349 1.11948157 1.41617654
 0.40212086 1.18245563 2.6713463  1.26917227 1.28190893 0.9548976
 2.28970189 0.58695594 1.74866964 0.38336493 2.95971515 0.284597  ], Cost: 0.0154
Current parameters: [2.97027061 3.32555599 3.69056341 2.91536838 1.07531715 1.41606757
 0.40385093 1.26087984 2.68199069 1.26890628 1.28244266 0.95340285
 2.25866771 0.65976748 1.76956156 0.34667217 2.95699285 0.27841854], Cost: 0.1151
Current parameters: [2.93172904 3.32473279 3.68905627 2.91598341 1.10640174 1.40473905
 0.40195652 1.20137951 2.72679832 1.26851493 1.28059205 0.94545459
 2.28447687 0.55777412 1.77732699 0.34152752 2.94950447 0.2892779 ], Cost: 0.0266
Current parameters: [2.92922401 3.20010834 3.68673229 2.9111521  1.10419428 1.4141187
 0.3682753  1.2058604  2.68389259 1.26972604 1.28243182 0.95569084
 2.30084123 0.5757382  1.78307168 0.3437986  2.95980817 0.27894465], Cost: 0.0568
Current parameters: [2.94418397 3.31805346 3.68216908 2.90559995 1.08879803 1.41896287
 0.39354913 1.21832937 2.6575922  1.27067986 1.27964589 0.91987596
 2.30054327 0.54955976 1.75474345 0.35303989 2.93745332 0.29423778], Cost: 0.0666
Current parameters: [2.85972343 3.33833415 3.67696223 2.90707956 1.0867349  1.4319479
 0.39265581 1.15110232 2.65211623 1.26087446 1.28298822 0.96514511
 2.28618521 0.5819634  1.78812373 0.27300902 2.94994141 0.27301156], Cost: 0.0683
Current parameters: [2.93213374 3.32387848 3.69176193 2.91809858 1.12973288 1.42393063
 0.40291273 1.1950717  2.66344542 1.2660258  1.28247528 0.95161105
 2.28457754 0.56215472 1.8273294  0.37158961 2.94154183 0.28002681], Cost: 0.0697
Current parameters: [2.92840936 3.32032511 3.68742197 2.91372447 1.11110929 1.41556792
 0.40336412 1.20233048 2.67444048 1.26860576 1.27991862 0.93488367
 2.31536385 0.58198886 1.78882    0.34516763 2.96135043 0.28451294], Cost: 0.0021
Current parameters: [2.92962982 3.32424269 3.69180419 2.92044266 1.11388316 1.41728797
 0.40904926 1.20801449 2.67998779 1.2657883  1.28276219 0.97250755
 2.35562386 0.58355935 1.7815791  0.34491451 2.94198501 0.29989446], Cost: 0.0880
Current parameters: [2.9292012  3.32607214 3.665444   2.91859335 1.11274371 1.41505832
 0.38358709 1.20398554 2.67686745 1.26962102 1.28015614 0.93692719
 2.317939   0.58340339 1.79118682 0.34824429 2.96345798 0.28478352], Cost: 0.0039
Current parameters: [2.92997993 3.31618367 3.65822421 2.8646083  1.11995802 1.41526791
 0.42530628 1.20374772 2.68018878 1.26731648 1.28015706 0.93636886
 2.31658926 0.58365287 1.7929578  0.34489064 2.9624415  0.28402242], Cost: 0.0201
Current parameters: [2.94399839 3.32041197 3.68674421 2.91283183 1.10144135 1.41548276
 0.40260448 1.1858847  2.67454769 1.24997566 1.28009213 0.93621403
 2.31712045 0.58305537 1.78987101 0.34675913 2.96461088 0.28512977], Cost: 0.0037
```



```
Current parameters: [2.92333673 3.3222832  3.6901433  2.91802363 1.11339053 1.41805393
 0.4072056  1.20556803 2.67235333 1.26966468 1.2800051  0.93134868
 2.31004932 0.57024665 1.79047526 0.34470709 3.01494516 0.31198512], Cost: 0.0406
Current parameters: [2.93773817 3.32340497 3.68766966 2.91346754 1.10342225 1.41629821
 0.40470776 1.21599887 2.67071894 1.26894111 1.28676757 0.94654569
 2.30247025 0.59759379 1.78921132 0.34087238 2.96365633 0.28557005], Cost: 0.0070
Current parameters: [2.92180202 3.32164795 3.69132002 2.91907759 1.10958494 1.41785534
 0.4026314  1.20055777 2.69131605 1.26563722 1.25909748 0.89798815
 2.31165925 0.62144256 1.78991524 0.35150015 2.95922298 0.27445231], Cost: 0.0606
Current parameters: [2.9293968  3.32320308 3.68850653 2.91463312 1.1083518  1.41620845
 0.40486442 1.206659   2.67594844 1.26836084 1.28441748 0.93744732
 2.31980586 0.57555825 1.78540215 0.34688866 2.97191723 0.25768439], Cost: 0.0064
Current parameters: [2.92558449 3.33441823 3.68684134 2.91345126 1.11019355 1.41608505
 0.40633534 1.19957792 2.67215609 1.26839428 1.27955096 0.93401803
 2.31443756 0.58141282 1.78795357 0.34204092 2.96006405 0.28483359], Cost: 0.0031
Current parameters: [2.91371721 3.31078313 3.68520706 2.91209169 1.10479961 1.41804416
 0.39701772 1.1915838  2.66912944 1.26774368 1.28314155 0.9391683
 2.30950377 0.58273416 1.78920372 0.3265213  2.96079144 0.28008196], Cost: 0.0109
Current parameters: [2.92815956 3.3231569  3.69538735 2.90488332 1.11138413 1.41536984
 0.39399185 1.2026611  2.67539644 1.26902022 1.27998607 0.93569633
 2.31659126 0.58262681 1.78972195 0.34608025 2.96233095 0.28484539], Cost: 0.0025
Current parameters: [2.94130391 3.32034291 3.68892885 2.91536816 1.13375599 1.41660753
 0.40044302 1.19916015 2.67158607 1.2686049  1.27970547 0.93309961
 2.31239515 0.58307161 1.77980595 0.33241995 2.96198471 0.2818181 ], Cost: 0.0055
Current parameters: [2.93312515 3.32030452 3.68727825 2.91348785 1.10806471 1.42338939
 0.40388236 1.19677686 2.67634814 1.27931269 1.27996237 0.93541075
 2.31619338 0.58258909 1.78928739 0.34542675 2.96196277 0.28456201], Cost: 0.0029
Current parameters: [2.93322459 3.32007631 3.68749588 2.9137443  1.10833705 1.38935903
 0.40401314 1.1948226  2.66923627 1.28139212 1.27983054 0.935024
 2.31582196 0.58354488 1.7910215  0.34527087 2.96261239 0.28301485], Cost: 0.0042
Current parameters: [2.92770686 3.31938384 3.68696502 2.91301439 1.11230337 1.41831351
 0.40326721 1.20202238 2.66208907 1.2686252  1.2837348  0.93170804
 2.3204354  0.58609858 1.78966404 0.3485875  2.96214093 0.28415117], Cost: 0.0038
Current parameters: [2.92717297 3.3213603  3.68821221 2.91493193 1.11285755 1.41341128
 0.40363524 1.19966892 2.68481393 1.26839145 1.30746466 0.92629127
 2.31490149 0.58226149 1.78865774 0.34592486 2.95866271 0.28745147], Cost: 0.0016
Current parameters: [2.92509296 3.32225315 3.68969946 2.91687696 1.11931211 1.4141374
 0.40512933 1.19585686 2.68582514 1.26767329 1.30791032 0.93137813
 2.31242156 0.58449223 1.79963473 0.34895295 2.95948054 0.28521598], Cost: 0.0008
Current parameters: [2.93229167 3.32164    3.69010995 2.91657013 1.11726127 1.41593772
 0.40123697 1.19965754 2.67787644 1.26819925 1.30901892 0.92482815
```

```
 2.29731888 0.56633089 1.80773222 0.35385353 2.94815192 0.28331279], Cost: 0.0241
Current parameters: [2.92852371 3.32263397 3.69015526 2.91762329 1.11755896 1.41406185
 0.40559806 1.20043799 2.6866977  1.26745195 1.3075682  0.92824653
 2.3194618  0.58344955 1.80824938 0.34051325 2.9591796  0.28572315], Cost: 0.0007
Current parameters: [2.92732849 3.32335835 3.69373826 2.92270005 1.11995127 1.41267579
 0.40770603 1.20078383 2.69007439 1.26680647 1.31071687 0.94803208
 2.33728705 0.58168387 1.80208629 0.34258631 2.94817116 0.29084814], Cost: 0.0235
Current parameters: [2.92878167 3.32245352 3.69009246 2.91742487 1.11684157 1.4138761
 0.40478407 1.19973192 2.68920835 1.26686715 1.30498665 0.92438578
 2.319885   0.59091297 1.80807505 0.34135963 2.94851299 0.27911499], Cost: 0.0043
Current parameters: [2.92285401 3.32325466 3.69430351 2.92397897 1.11860202 1.41354003
 0.40701228 1.20032161 2.69565722 1.26669916 1.300296   0.91372782
 2.31872343 0.59448154 1.80866959 0.34314996 2.97848874 0.29174941], Cost: 0.0058
Current parameters: [2.92629002 3.3168683  3.68479289 2.91190188 1.12005444 1.41253069
 0.41301202 1.20464598 2.69137988 1.26443402 1.30339999 0.92841421
 2.31882715 0.58107267 1.80898612 0.34209258 2.95781179 0.28766733], Cost: 0.0014
Current parameters: [2.93258434 3.33129615 3.68119349 2.89552328 1.11959246 1.41608266
 0.40691328 1.19259639 2.68378225 1.27274186 1.31546594 0.92704682
 2.3225657  0.59056914 1.80961882 0.33760384 2.96733715 0.28300787], Cost: 0.0026
Current parameters: [2.92006177 3.31672194 3.68889043 2.91727131 1.11476524 1.41474812
 0.40354203 1.19571116 2.68554221 1.26637231 1.30814412 0.9299436
 2.31643443 0.5838209  1.80849031 0.33135841 2.9600041  0.28451897], Cost: 0.0016
Current parameters: [2.92987107 3.32268114 3.69002201 2.91735677 1.11686081 1.41408197
 0.40548126 1.20173288 2.68575085 1.26762466 1.30787568 0.92960157
 2.31658793 0.5872028  1.80895881 0.33949239 2.95755648 0.29097363], Cost: 0.0010
Current parameters: [2.93075444 3.32246324 3.69035302 2.91871315 1.11381025 1.41392352
 0.40808887 1.20845787 2.68746798 1.26636014 1.3102471  0.93362933
 2.3153496  0.58688209 1.80729184 0.33927191 2.9640848  0.27796648], Cost: 0.0020
Current parameters: [2.92891125 3.32312751 3.69034175 2.9175668  1.12046793 1.42030071
 0.40487023 1.20147907 2.68733963 1.26474613 1.3078402  0.927921
 2.3192302  0.58335483 1.80686152 0.33922293 2.95900659 0.28580804], Cost: 0.0011
Current parameters: [2.93430712 3.32033002 3.69063736 2.91942729 1.12752321 1.40811043
 0.40592783 1.1991328  2.68577221 1.26906836 1.30610833 0.92790006
 2.3173324  0.5837411  1.80442423 0.33441946 2.96006026 0.28414256], Cost: 0.0001
Current parameters: [2.93144067 3.32135524 3.68731449 2.92030073 1.12962726 1.40812518
 0.40287271 1.20249037 2.68606806 1.2728965  1.30619331 0.92793295
 2.31745242 0.58379902 1.80460985 0.33461711 2.95977287 0.28407539], Cost: 0.0002
Current parameters: [2.93909261 3.31993861 3.68126866 2.92244452 1.12559265 1.40623331
 0.39937444 1.19616733 2.6882058  1.26220921 1.30463692 0.92975112
 2.31786343 0.58392896 1.8053943  0.33569424 2.96199805 0.28427891], Cost: 0.0009
Current parameters: [2.9358611  3.3143804  3.69168918 2.91844589 1.12788448 1.40917743
```



```
 0.40365162 1.19961977 2.685074   1.27038533 1.30774799 0.92759723
 2.31888931 0.58489757 1.80510329 0.33656047 2.96086657 0.28405951], Cost: 0.0004
Current parameters: [2.93164901 3.31994168 3.68699617 2.92229591 1.12936845 1.407414
 0.40975076 1.20081754 2.67365181 1.26466496 1.30633223 0.92549747
 2.32050165 0.58662266 1.80444592 0.33609982 2.96066918 0.28348299], Cost: 0.0011

   Return from subroutine COBYLA because the MAXFUN limit has been reached.

   NFVALS =  100   F = 1.487326E-04    MAXCV = 0.000000E+00
   X = 2.934307E+00   3.320330E+00   3.690637E+00   2.919427E+00   1.127523E+00
       1.408110E+00   4.059278E-01   1.199133E+00   2.685772E+00   1.269068E+00
       1.306108E+00   9.279001E-01   2.317332E+00   5.837411E-01   1.804424E+00
       3.344195E-01   2.960060E+00   2.841426E-01
Current parameters: [2.93430712 3.32033002 3.69063736 2.91942729 1.12752321 1.40811043
 0.40592783 1.1991328  2.68577221 1.26906836 1.30610833 0.92790006
 2.3173324  0.5837411  1.80442423 0.33441946 2.96006026 0.28414256], Cost: 0.0001
Simulating for QAOA depth p = 10
Current parameters: [1.92166387 1.71905878 0.96815404 1.48572621 0.87181057 2.38670433
 2.35311616 0.78507887 1.08676054 0.4586532  0.42730698 1.07723871
 2.50144946 2.49586542 1.45424135 0.85897997 2.42674822 1.01823001
 2.50581113 2.44522036], Cost: 2.7176
Current parameters: [0.92166387 2.71905878 0.96815404 1.48572621 0.87181057 2.38670433
 2.35311616 0.78507887 1.08676054 0.4586532  0.42730698 1.07723871
 2.50144946 2.49586542 1.45424135 0.85897997 2.42674822 1.01823001
 2.50581113 2.44522036], Cost: 1.1138
Current parameters: [0.92166387 2.71905878 1.96815404 1.48572621 0.87181057 2.38670433
 2.35311616 0.78507887 1.08676054 0.4586532  0.42730698 1.07723871
 2.50144946 2.49586542 1.45424135 0.85897997 2.42674822 1.01823001
 2.50581113 2.44522036], Cost: 1.3258
Current parameters: [0.92166387 2.71905878 0.96815404 2.48572621 0.87181057 2.38670433
 2.35311616 0.78507887 1.08676054 0.4586532  0.42730698 1.07723871
 2.50144946 2.49586542 1.45424135 0.85897997 2.42674822 1.01823001
 2.50581113 2.44522036], Cost: 1.3638
Current parameters: [0.92166387 2.71905878 0.96815404 1.48572621 1.87181057 2.38670433
 2.35311616 0.78507887 1.08676054 0.4586532  0.42730698 1.07723871
 2.50144946 2.49586542 1.45424135 0.85897997 2.42674822 1.01823001
 2.50581113 2.44522036], Cost: 1.8759
Current parameters: [0.92166387 2.71905878 0.96815404 1.48572621 0.87181057 3.38670433
 2.35311616 0.78507887 1.08676054 0.4586532  0.42730698 1.07723871
 2.50144946 2.49586542 1.45424135 0.85897997 2.42674822 1.01823001
 2.50581113 2.44522036], Cost: 1.0840
```



```
Current parameters: [0.92166387 2.71905878 0.96815404 1.48572621 0.87181057 3.38670433
 3.35311616 0.78507887 1.08676054 0.4586532  0.42730698 1.07723871
 2.50144946 2.49586542 1.45424135 0.85897997 2.42674822 1.01823001
 2.50581113 2.44522036], Cost: 2.2397
Current parameters: [0.92166387 2.71905878 0.96815404 1.48572621 0.87181057 3.38670433
 2.35311616 1.78507887 1.08676054 0.4586532  0.42730698 1.07723871
 2.50144946 2.49586542 1.45424135 0.85897997 2.42674822 1.01823001
 2.50581113 2.44522036], Cost: 2.0814
Current parameters: [0.92166387 2.71905878 0.96815404 1.48572621 0.87181057 3.38670433
 2.35311616 0.78507887 2.08676054 0.4586532  0.42730698 1.07723871
 2.50144946 2.49586542 1.45424135 0.85897997 2.42674822 1.01823001
 2.50581113 2.44522036], Cost: 0.2571
Current parameters: [0.92166387 2.71905878 0.96815404 1.48572621 0.87181057 3.38670433
 2.35311616 0.78507887 2.08676054 1.4586532  0.42730698 1.07723871
 2.50144946 2.49586542 1.45424135 0.85897997 2.42674822 1.01823001
 2.50581113 2.44522036], Cost: 2.7225
Current parameters: [0.92166387 2.71905878 0.96815404 1.48572621 0.87181057 3.38670433
 2.35311616 0.78507887 2.08676054 0.4586532  1.42730698 1.07723871
 2.50144946 2.49586542 1.45424135 0.85897997 2.42674822 1.01823001
 2.50581113 2.44522036], Cost: 1.9418
Current parameters: [0.92166387 2.71905878 0.96815404 1.48572621 0.87181057 3.38670433
 2.35311616 0.78507887 2.08676054 0.4586532  0.42730698 2.07723871
 2.50144946 2.49586542 1.45424135 0.85897997 2.42674822 1.01823001
 2.50581113 2.44522036], Cost: 2.2936
Current parameters: [0.92166387 2.71905878 0.96815404 1.48572621 0.87181057 3.38670433
 2.35311616 0.78507887 2.08676054 0.4586532  0.42730698 1.07723871
 3.50144946 2.49586542 1.45424135 0.85897997 2.42674822 1.01823001
 2.50581113 2.44522036], Cost: 2.4263
Current parameters: [0.92166387 2.71905878 0.96815404 1.48572621 0.87181057 3.38670433
 2.35311616 0.78507887 2.08676054 0.4586532  0.42730698 1.07723871
 2.50144946 3.49586542 1.45424135 0.85897997 2.42674822 1.01823001
 2.50581113 2.44522036], Cost: 1.3842
Current parameters: [0.92166387 2.71905878 0.96815404 1.48572621 0.87181057 3.38670433
 2.35311616 0.78507887 2.08676054 0.4586532  0.42730698 1.07723871
 2.50144946 2.49586542 2.45424135 0.85897997 2.42674822 1.01823001
 2.50581113 2.44522036], Cost: 3.1204
Current parameters: [0.92166387 2.71905878 0.96815404 1.48572621 0.87181057 3.38670433
 2.35311616 0.78507887 2.08676054 0.4586532  0.42730698 1.07723871
 2.50144946 2.49586542 1.45424135 1.85897997 2.42674822 1.01823001
 2.50581113 2.44522036], Cost: 2.8283
Current parameters: [0.92166387 2.71905878 0.96815404 1.48572621 0.87181057 3.38670433
```



```
 2.35311616 0.78507887 2.08676054 0.4586532  0.42730698 1.07723871
 2.50144946 2.49586542 1.45424135 0.85897997 3.42674822 1.01823001
 2.50581113 2.44522036], Cost: 0.8661
Current parameters: [0.92166387 2.71905878 0.96815404 1.48572621 0.87181057 3.38670433
 2.35311616 0.78507887 2.08676054 0.4586532  0.42730698 1.07723871
 2.50144946 2.49586542 1.45424135 0.85897997 2.42674822 2.01823001
 2.50581113 2.44522036], Cost: 1.8645
Current parameters: [0.92166387 2.71905878 0.96815404 1.48572621 0.87181057 3.38670433
 2.35311616 0.78507887 2.08676054 0.4586532  0.42730698 1.07723871
 2.50144946 2.49586542 1.45424135 0.85897997 2.42674822 1.01823001
 3.50581113 2.44522036], Cost: 2.7341
Current parameters: [0.92166387 2.71905878 0.96815404 1.48572621 0.87181057 3.38670433
 2.35311616 0.78507887 2.08676054 0.4586532  0.42730698 1.07723871
 2.50144946 2.49586542 1.45424135 0.85897997 2.42674822 1.01823001
 2.50581113 3.44522036], Cost: 2.0988
Current parameters: [0.90196841 2.92135292 0.93881128 1.45112622 0.76633289 3.39084089
 2.19313866 0.64700807 2.20122039 0.11739342 0.19411149 0.79534096
 2.20118355 2.33985364 1.0579044  0.50306422 2.34244336 0.79572401
 2.16293875 2.1902885 ], Cost: 2.2705
Current parameters: [0.91181614 2.82020585 0.95348266 1.46842621 0.81907173 3.38877261
 2.27312741 0.71604347 2.14399047 0.28802331 0.31070923 0.93628984
 2.3513165  2.41785953 1.25607287 0.6810221  2.38459579 0.90697701
 2.33437494 2.31775443], Cost: 1.0604
Current parameters: [0.67207919 2.71905878 0.96815404 1.48572621 0.87181057 3.38670433
 2.35311616 0.78507887 2.08676054 0.47305772 0.42730698 1.07723871
 2.50144946 2.49586542 1.45424135 0.85897997 2.42674822 1.01823001
 2.50581113 2.44522036], Cost: 0.2383
Current parameters: [0.69820583 2.74909052 0.96379794 1.48058964 0.8561518  3.38731842
 2.32936657 0.76458146 2.10375277 0.95248807 0.39268775 1.03538936
 2.45687324 2.47270456 1.39540283 0.80614221 2.41423267 0.9851977
 2.45490973 2.40737424], Cost: 1.1077
Current parameters: [0.67207919 2.68317184 0.7207432  1.48572621 0.87181057 3.38670433
 2.35311616 0.78507887 2.08676054 0.47305772 0.42730698 1.07723871
 2.50144946 2.49586542 1.45424135 0.85897997 2.42674822 1.01823001
 2.50581113 2.44522036], Cost: 0.4621
Current parameters: [0.67029479 2.23296415 1.04992265 1.48261427 0.86232386 3.38707637
 2.33872771 0.77266072 2.09705511 0.45833896 0.40633327 1.0518847
 2.47444341 2.48183365 1.41859462 0.82696875 2.4191658  0.99821772
 2.47497303 2.42229167], Cost: 0.7169
Current parameters: [0.67207919 2.71905878 0.96815404 1.24818026 0.9497331  3.38670433
 2.35311616 0.78507887 2.08676054 0.47305772 0.42730698 1.07723871
```

```
 2.50144946 2.49586542 1.45424135 0.85897997 2.42674822 1.01823001
 2.50581113 2.44522036], Cost: 0.3757
Current parameters: [0.67110513 2.72421017 0.9735537  1.64445396 1.34370668 3.38690742
 2.34526187 0.77830011 2.09238008 0.46502312 0.41585796 1.0633986
 2.48670754 2.48820583 1.4347827  0.84150585 2.42260917 1.0073058
 2.48897739 2.43270416], Cost: 0.5663
Current parameters: [0.67207919 2.71905878 0.96815404 1.48572621 0.87181057 3.6366208
 2.35957828 0.78507887 2.08676054 0.47305772 0.42730698 1.07723871
 2.50144946 2.49586542 1.45424135 0.85897997 2.42674822 1.01823001
 2.50581113 2.44522036], Cost: 0.1837
Current parameters: [0.67065853 2.72657207 0.97602943 1.48941302 0.86557169 3.62597738
 2.8550142  0.77519208 2.09495663 0.46133928 0.41060863 1.05705295
 2.47994842 2.48469393 1.42586098 0.83349403 2.42071143 1.00229709
 2.48125918 2.42696553], Cost: 0.3384
Current parameters: [0.67207919 2.71905878 0.96815404 1.48572621 0.87181057 3.6366208
 2.35957828 0.94463155 2.27922597 0.47305772 0.42730698 1.07723871
 2.50144946 2.49586542 1.45424135 0.85897997 2.42674822 1.01823001
 2.50581113 2.44522036], Cost: 0.5397
Current parameters: [0.6704463  2.72769446 0.9772059  1.48996379 0.86463968 3.63912699
 2.35898613 1.15508337 1.75895643 0.4595887  0.40811411 1.05403747
 2.47673645 2.48302506 1.42162132 0.82968677 2.41980961 0.99991693
 2.47759144 2.4242385 ], Cost: 0.5414
Current parameters: [0.67207919 2.71905878 0.96815404 1.48572621 0.87181057 3.6366208
 2.35957828 0.78507887 2.08676054 0.47305772 0.61993885 0.91788702
 2.50144946 2.49586542 1.45424135 0.85897997 2.42674822 1.01823001
 2.50581113 2.44522036], Cost: 0.2481
Current parameters: [0.66846811 2.73815624 0.98817187 1.49509744 0.85595244 3.64216314
 2.35826876 0.75318969 2.06659525 0.44327152 0.72491142 1.44717634
 2.44679761 2.46746948 1.38210347 0.79419921 2.41140377 0.97773136
 2.44340441 2.39881983], Cost: 3.1765
Current parameters: [0.67207919 2.71905878 0.96815404 1.48572621 0.87181057 3.6366208
 2.35957828 0.78507887 2.08676054 0.47305772 0.42730698 1.07723871
 2.61671407 2.27402297 1.45424135 0.85897997 2.42674822 1.01823001
 2.50581113 2.44522036], Cost: 0.6782
Current parameters: [0.66917085 2.73443976 0.98427628 1.49327374 0.85903853 3.64108457
 2.3585236  0.75939552 2.07051954 0.4490681  0.35840632 1.00214666
 2.90341311 2.74994608 1.39614194 0.80680595 2.41438989 0.98561264
 2.45554914 2.40784965], Cost: 1.5022
Current parameters: [0.67207919 2.71905878 0.96815404 1.48572621 0.87181057 3.6366208
 2.35957828 0.78507887 2.08676054 0.47305772 0.42730698 1.07723871
 2.50144946 2.49586542 1.62127824 0.67297278 2.42674822 1.01823001
```



```
 2.50581113 2.44522036], Cost: 0.3518
Current parameters: [0.6672055  2.74483364 0.99517107 1.49837407 0.85040767 3.64410101
 2.35781088 0.74203969 2.05954449 0.43285683 0.31184589 0.95140233
 2.3888216  2.51313925 1.76035503 1.16460733 2.4060386  0.96357108
 2.42158401 2.38259596], Cost: 2.6155
Current parameters: [0.67207919 2.71905878 0.96815404 1.48572621 0.87181057 3.6366208
 2.35957828 0.78507887 2.08676054 0.47305772 0.42730698 1.07723871
 2.50144946 2.49586542 1.45424135 0.85897997 2.19296612 1.10680726
 2.50581113 2.44522036], Cost: 0.4156
Current parameters: [0.67020264 2.72898306 0.97855661 1.49059611 0.86356966 3.63950096
 2.35889777 0.76850718 2.07628135 0.45757888 0.38285016 1.02878702
 2.45808354 2.50251649 1.40276468 0.82458671 2.61066817 1.46936644
 2.47338055 2.42110763], Cost: 1.8772
Current parameters: [0.67207919 2.71905878 0.96815404 1.48572621 0.87181057 3.6366208
 2.35957828 0.78507887 2.08676054 0.47305772 0.42730698 1.07723871
 2.50144946 2.49586542 1.45424135 0.85897997 2.42674822 1.01823001
 2.65497758 2.2445979 ], Cost: 0.1904
Current parameters: [0.66875068 2.73666183 0.98660544 1.49436412 0.85719337 3.64172944
 2.35837123 0.75568508 2.06817323 0.44560235 0.34845235 0.99129823
 2.42452979 2.50766266 1.36293535 0.79797541 2.41728499 0.9324432
 2.87133282 2.71777581], Cost: 1.3346
Current parameters: [0.57842708 2.80229114 1.0457822  1.5515729  0.78773493 3.54843052
 2.3359328  0.76665171 2.10203654 0.39106045 0.39618432 1.03961617
 2.46137547 2.47504383 1.40134556 0.81147887 2.41549675 0.98853399
 2.46005081 2.41119673], Cost: 0.0505
Current parameters: [0.72048203 2.78534082 0.91153072 1.42855509 0.8607922  3.69912513
 2.28660268 0.70537655 2.12945692 0.32440742 0.27679697 0.90040127
 2.32104617 2.43030173 1.22168166 0.66156101 2.38269994 0.86887957
 2.31748285 2.30562119], Cost: 1.8657
Current parameters: [0.56935797 2.81951461 1.06600581 1.5689125  0.76845055 3.55113157
 2.32130217 0.73893258 2.09279089 0.62419068 0.36565125 1.04162736
 2.43150196 2.48747955 1.3652267  0.81238715 2.43167722 0.96121969
 2.42397797 2.41529035], Cost: 0.5355
Current parameters: [0.5235649  2.87862423 1.11601726 1.60181299 0.71640874 3.31501965
 2.40467277 0.90631354 2.17027008 0.40457319 0.47247902 0.85399979
 2.52143772 2.36557001 1.46930653 0.65142229 2.26510172 1.0339743
 2.58573182 2.27522995], Cost: 1.8302
Current parameters: [0.57391118 2.80616203 1.05451415 1.55707162 0.78273103 3.55882484
 2.33256846 0.74791901 2.08942052 0.37303704 0.36345722 1.02126875
 2.66075105 2.60741257 1.36905189 0.80454776 2.42289941 0.95669616
 2.43693839 2.40885729], Cost: 0.1525
```



```
Current parameters: [0.58518077 2.74540976 1.04718319 1.5106263  0.6846579  3.55445854
 2.33632847 0.76730866 2.10245636 0.3904316  0.39680655 1.03920352
 2.46162612 2.47433735 1.40192745 0.8110035  2.41490634 0.98907127
 2.46069865 2.41075319], Cost: 0.1215
Current parameters: [0.53647874 2.60057587 1.11861678 1.62253881 0.86800021 3.51068699
 2.32996354 0.75805464 2.10152767 0.38336056 0.3810062  1.02672086
 2.44811419 2.47606412 1.38124996 0.79925293 2.41493847 0.97335407
 2.44418924 2.40314278], Cost: 0.0296
Current parameters: [0.53689445 2.60011778 1.11834102 1.62229379 0.86833982 3.50787705
 2.33066281 0.76061773 2.10346802 0.38468564 0.44107575 1.09442589
 2.45476803 2.47599449 1.31992482 0.73997588 2.41365549 0.97880086
 2.45182341 2.40491908], Cost: 0.4866
Current parameters: [0.51694299 2.61117435 1.15316984 1.65064729 0.8659109  3.51317471
 2.32731991 0.71951802 2.07418968 0.32045074 0.41405112 1.09400628
 2.41027712 2.52293817 1.50477212 0.94708857 2.42167514 0.89619324
 2.40803322 2.3910288 ], Cost: 1.7201
Current parameters: [0.53705482 2.59991141 1.11823832 1.62220816 0.86849991 3.50715781
 2.33082096 0.76128317 2.1040155  0.38518641 0.39233521 1.03417849
 2.45586598 2.47561139 1.39258342 0.80408697 2.43937695 1.0490758
 2.37107752 2.34382861], Cost: 0.4090
Current parameters: [0.52899982 2.60199045 1.13343835 1.63492546 0.86998975 3.52766847
 2.32345806 0.7270342  2.08175304 0.35854607 0.30669011 0.97211515
 2.42188836 2.50862831 1.34523672 0.80310226 2.49792359 1.10861868
 2.55296163 2.50890718], Cost: 0.3440
Current parameters: [0.54027185 2.6006199  1.11470742 1.61894444 0.86943511 3.50638898
 2.33125542 0.6684747  2.18634369 0.38204599 0.38775529 1.02213137
 2.45306298 2.46947831 1.38816113 0.794538   2.4074764  0.97659344
 2.45053537 2.3977356 ], Cost: 0.0713
Current parameters: [0.60261673 2.59130058 1.05967461 1.56910067 0.90940241 3.58078796
 2.30649558 0.75008592 2.09070716 0.32831192 0.29468487 0.92213989
 2.38873721 2.46759069 1.30469896 0.7367956  2.39445809 0.89771999
 2.39891143 2.36595455], Cost: 0.5678
Current parameters: [0.53644838 2.59991781 1.11874588 1.62278194 0.86863459 3.51534356
 2.45279649 0.75127687 2.09693242 0.3834123  0.37176106 1.02788137
 2.44050638 2.48097268 1.37067248 0.80032283 2.42116605 0.96759707
 2.43496967 2.40608656], Cost: 0.0634
Current parameters: [0.50757354 2.63361758 1.15369866 1.65115513 0.84205974 3.39993769
 2.35727721 0.82686276 2.13123626 0.39497282 0.38477438 0.90433005
 2.49013926 2.43019917 1.42476955 0.73633593 2.33828684 0.97650842
 2.52499045 2.35890956], Cost: 0.2349
Current parameters: [0.53804034 2.59586404 1.11970197 1.62378782 0.87308443 3.5282208
```



```
 2.33151278 0.74813785 2.08548451 0.38454558 0.36478779 1.03220815
 2.50705347 2.37529545 1.37563399 0.81699072 2.43082925 0.96579677
 2.44251495 2.42116764], Cost: 0.1466
Current parameters: [0.54898054 2.59715643 1.09129083 1.67318598 0.85091396 3.52135801
 2.33007791 0.7591217  2.10309842 0.38197817 0.38222739 1.02523152
 2.44781118 2.47590668 1.38211299 0.79748735 2.41308575 0.97379928
 2.4449711  2.40136861], Cost: 0.0234
Current parameters: [0.49134372 2.58124337 0.99851048 1.65191219 0.86058422 3.47173204
 2.32936896 0.7541441  2.09513197 0.38623488 0.37437715 1.02848397
 2.45031078 2.47710941 1.37915073 0.80631132 2.42114108 0.97020993
 2.44379724 2.41032432], Cost: 0.0291
Current parameters: [0.50374179 2.59991078 1.09385973 1.675365   0.8481317  3.56063518
 2.33038649 0.75905072 2.10425391 0.38169673 0.3875721  1.01871318
 2.44648504 2.47521765 1.38589018 0.78976001 2.40497701 0.9757478
 2.44839305 2.39360362], Cost: 0.0079
Current parameters: [0.5009738  2.59291308 1.08766455 1.67093005 0.85586925 3.57603876
 2.33232264 0.83465817 2.19107572 0.38564548 0.36975797 1.02929083
 2.45187819 2.47785051 1.37786045 0.81106118 2.42520598 0.96797054
 2.44378106 2.41516507], Cost: 0.0342
Current parameters: [0.50217288 2.60130514 1.0951602  1.67646811 0.84672322 3.55915776
 2.32999036 0.75874202 2.10450983 0.38032307 0.3902778  1.01541335
 2.4458137  2.47486883 1.35177984 0.8259617  2.37003784 0.98841693
 2.45012537 2.38967269], Cost: 0.0145
Current parameters: [0.51930434 2.5848002  1.08008014 1.66445269 0.86402842 3.57556382
 2.33440675 0.76363581 2.10181308 0.39329796 0.35424259 1.04881161
 2.45567567 2.47983268 1.43152037 0.7614954  2.3279013  1.00830752
 2.43348267 2.43793823], Cost: 0.0894
Current parameters: [0.4983201  2.60472925 1.09835376 1.67917699 0.84326441 3.55552969
 2.32901761 0.75798394 2.10513827 0.37694977 0.41284905 0.99413472
 2.44416509 2.47401225 1.38282795 0.78701009 2.40432564 0.97402864
 2.41613973 2.43145027], Cost: 0.0148
Current parameters: [0.53982676 2.56708958 1.06343251 1.65001114 0.88165701 3.59477631
 2.33955071 0.76704481 2.09842525 0.41210261 0.45440497 0.9831455
 2.46341174 2.48391836 1.40789014 0.80911631 2.40875475 0.98605909
 2.48284459 2.38902184], Cost: 0.0633
Current parameters: [0.50437313 2.59985059 1.09277576 1.67491676 0.84836121 3.55825701
 2.33045605 0.76217357 2.10683667 0.39792478 0.39393868 1.02325602
 2.39671134 2.44381452 1.39186755 0.79148372 2.40400897 0.98206298
 2.4521507  2.39437817], Cost: 0.0130
Current parameters: [0.49654114 2.60827386 1.10499375 1.68634202 0.8399419  3.56173684
 2.32221568 0.7456995  2.09873284 0.49285319 0.35993487 1.0065299
```

```
 2.45686771 2.49762455 1.3686474  0.79222637 2.41177651 0.95366529
 2.43576627 2.39995854], Cost: 0.1150
Current parameters: [0.50740709 2.57174998 1.09380371 1.65475353 0.79776844 3.56279499
 2.33087287 0.76091993 2.10545239 0.38277236 0.39009922 1.01956603
 2.44778457 2.47312767 1.38837035 0.78976034 2.40036668 0.96869594
 2.44435839 2.38903002], Cost: 0.0279
Current parameters: [0.49809892 2.59058686 1.10217959 1.6754625  0.82805431 3.56768839
 2.32641254 0.74692856 2.09500931 0.3697557  0.35185153 0.99167509
 2.43412783 2.49250896 1.36993519 0.79163676 2.44589499 1.04269971
 2.5040918  2.44476387], Cost: 0.1011
Current parameters: [0.5194236  2.59221113 1.07788994 1.66152727 0.85924567 3.59170904
 2.32121463 0.74587495 2.0974463  0.37217696 0.37157057 1.02235838
 2.43012388 2.4815622  1.36561447 0.79076676 2.41557357 0.96398551
 2.42789885 2.39687235], Cost: 0.0307
Current parameters: [0.51450593 2.61002231 1.08382695 1.66764457 0.85471674 3.54608243
 2.33016275 0.78231194 2.11268638 0.36438747 0.35427284 0.93297378
 2.43812001 2.45853675 1.37209424 0.74238827 2.37208684 0.95062327
 2.46408326 2.36397724], Cost: 0.0711
Current parameters: [0.49320611 2.54951527 1.11215522 1.6931767  0.86819059 3.55120183
 2.32888879 0.75680636 2.10405928 0.37961106 0.38397426 1.01578893
 2.44308411 2.4755943  1.38107508 0.78697898 2.4048526  0.97176292
 2.4443483  2.3915622 ], Cost: 0.0055
Current parameters: [0.47877135 2.56097545 1.12525624 1.70859714 0.86808641 3.54854736
 2.32301339 0.74983096 2.09222982 0.3463002  0.37404266 1.01050693
 2.41881549 2.49906025 1.4385049  0.84942015 2.39630261 0.9059851
 2.46682652 2.40451452], Cost: 0.4840
Current parameters: [0.47375741 2.57960425 1.11377496 1.70097156 0.87259314 3.55390529
 2.33213429 0.75475934 2.0995747  0.36954702 0.39568479 1.04062425
 2.46411377 2.4817652  1.38778137 0.76628899 2.41403074 0.96749866
 2.4618062  2.37477083], Cost: 0.0553
Current parameters: [0.51083566 2.51905849 1.10908956 1.6870526  0.86468125 3.54163631
 2.32238171 0.76953628 2.11140999 0.38768452 0.4253478  1.04391326
 2.42109981 2.4711333  1.30205588 0.73737574 2.38408895 0.95796423
 2.46300965 2.42666216], Cost: 0.3673
Current parameters: [0.49392956 2.54677325 1.11249689 1.6939731  0.87080494 3.55937878
 2.3293952  0.75275344 2.09643759 0.37988832 0.37435867 1.01568343
 2.47168898 2.42481257 1.37749956 0.7953204  2.41199442 0.96670053
 2.4452379  2.4015436 ], Cost: 0.0401
Current parameters: [0.49227923 2.5494862  1.11311639 1.6940609  0.86784764 3.55217359
 2.32859115 0.7791556  2.08304991 0.37998757 0.38260628 1.01713298
 2.44206588 2.47721855 1.37966694 0.7882826  2.40737396 0.97302458
```



```
 2.44077582 2.39128706], Cost: 0.0043
Current parameters: [0.4926502  2.54756659 1.11192258 1.6943259  0.86858204 3.547946
 2.32702952 0.77889493 2.0908542  0.37978134 0.38357938 1.01043342
 2.44469349 2.47313929 1.38365711 0.78908684 2.41809837 1.01158674
 2.40592945 2.36100839], Cost: 0.0957
Current parameters: [0.49106152 2.55143753 1.11419868 1.69524353 0.86713465 3.55128181
 2.29940467 0.77938373 2.0834117  0.38977259 0.38235915 1.01623233
 2.44548676 2.47830903 1.38117824 0.78788925 2.40683885 0.97232175
 2.44221709 2.39053994], Cost: 0.0085
Current parameters: [0.48589368 2.55823923 1.11867976 1.7007097  0.86515849 3.55334206
 2.34815954 0.77160116 2.07927156 0.43063206 0.36718664 1.01131678
 2.44892736 2.48873297 1.37237506 0.78743321 2.41344842 0.96293465
 2.43436497 2.39027735], Cost: 0.0243
Current parameters: [0.5076233  2.55261569 1.13492065 1.69849702 0.86678223 3.56578212
 2.32915872 0.78062079 2.08471143 0.38007228 0.38710225 1.01515974
 2.44240159 2.47736047 1.38146468 0.78668585 2.40568296 0.97425244
 2.44255334 2.38862046], Cost: 0.0097
Current parameters: [0.50230488 2.5375741  1.09133215 1.68145241 0.886178   3.56582148
 2.33391066 0.78210244 2.0786558  0.3955414  0.41511211 1.00082079
 2.45475199 2.48278521 1.39328027 0.79653998 2.41206215 0.97781042
 2.45983581 2.3854976 ], Cost: 0.0135
Current parameters: [0.48959626 2.54529931 1.11647937 1.69582238 0.863625   3.55559354
 2.32803097 0.77287818 2.08026414 0.38197019 0.38493968 1.0306706
 2.44137667 2.48252608 1.3797074  0.79872828 2.41938183 0.98704486
 2.44456225 2.40451277], Cost: 0.0116
Current parameters: [0.48882785 2.55572776 1.11327305 1.6928666  0.86279174 3.55242211
 2.32849497 0.78006033 2.08035207 0.37127901 0.35797111 0.98241396
 2.44123721 2.48176327 1.37444008 0.77272344 2.41811123 0.99555921
 2.4718081  2.39871733], Cost: 0.0305
Current parameters: [0.4950136  2.54813059 1.11398311 1.69419073 0.8662099  3.54865188
 2.32800986 0.76083044 2.06187308 0.37964963 0.3854551  1.01576713
 2.43936454 2.47608191 1.38249078 0.78273338 2.39773966 0.97644648
 2.43950934 2.3894976 ], Cost: 0.0017
Current parameters: [0.49847012 2.54647876 1.10622944 1.68979015 0.87662982 3.55875143
 2.33152926 0.77092193 2.06901726 0.38635983 0.36631733 1.03109312
 2.44998238 2.4803483  1.408293   0.76799235 2.36455946 0.99330096
 2.43566328 2.40889602], Cost: 0.0236
Current parameters: [0.49083809 2.55372688 1.11612163 1.69694185 0.86664103 3.54981374
 2.32823353 0.75788922 2.05927397 0.37434359 0.37697089 1.00967501
 2.43924766 2.47964541 1.40274114 0.79959334 2.39980103 0.97041649
 2.43877112 2.38586124], Cost: 0.0334
```



```
Current parameters: [0.50097298 2.52825375 1.1131318  1.69599826 0.86559138 3.53802364
 2.32650028 0.76648943 2.06196504 0.37753895 0.40145916 1.01880377
 2.41332179 2.48101992 1.35717319 0.77850689 2.3791411  0.94942764
 2.45025393 2.41188894], Cost: 0.0279
Current parameters: [0.49107518 2.54714404 1.10806883 1.69314562 0.86841665 3.5476453
 2.33476132 0.77203561 2.05962914 0.3728809  0.37936965 1.0177367
 2.44811125 2.45510504 1.37878888 0.78586801 2.39900193 0.96678223
 2.43609813 2.39241857], Cost: 0.0089
Current parameters: [0.50801691 2.53975964 1.12817078 1.69814759 0.86488368 3.56070459
 2.31222964 0.72834595 2.05545242 0.39758271 0.39039296 1.01136221
 2.45613027 2.44777442 1.37628005 0.78463387 2.40035927 0.98613285
 2.45178302 2.40420593], Cost: 0.0350
Current parameters: [0.50025411 2.54941373 1.105031   1.68788791 0.87398262 3.56710033
 2.32379291 0.75444476 2.05844873 0.37483323 0.37649559 1.01881222
 2.43500235 2.48169369 1.37479481 0.77975912 2.40454208 0.97013853
 2.43326588 2.38909856], Cost: 0.0083
Current parameters: [0.49241599 2.55079737 1.12052432 1.68534049 0.87548026 3.54487401
 2.32786401 0.7603934  2.06144787 0.38001857 0.38544031 1.01561434
 2.43818956 2.4756827  1.38123997 0.78394184 2.3980428  0.9772908
 2.43904937 2.3911773 ], Cost: 0.0018
Current parameters: [0.49185795 2.54014899 1.11842282 1.67281211 0.84790899 3.55162051
 2.32826951 0.76123073 2.06183359 0.38006617 0.38479851 1.01796284
 2.44352287 2.47792318 1.38535855 0.77946424 2.39788268 0.97196043
 2.44082201 2.38558744], Cost: 0.0054
Current parameters: [0.49497815 2.54520658 1.11571345 1.69570249 0.86712804 3.54706536
 2.32777464 0.7599055  2.06133    0.37552762 0.38547648 1.01294331
 2.45002293 2.48298738 1.37834224 0.78454961 2.39586314 0.975444
 2.43634346 2.39160327], Cost: 0.0013
Current parameters: [0.5176907  2.54887522 1.11157997 1.69174997 0.86646127 3.53157229
 2.32790441 0.76010067 2.06055164 0.37602496 0.38006762 1.01948743
 2.45366187 2.48532498 1.38082585 0.78364591 2.40360426 0.9728698
 2.43931352 2.39155341], Cost: 0.0038
Current parameters: [0.49570322 2.54493836 1.11505224 1.6951272  0.86759317 3.54774508
 2.32795039 0.76004925 2.06122377 0.37611752 0.38159903 1.01663376
 2.45032622 2.48313147 1.37276959 0.79155487 2.3896935  0.97804585
 2.44227787 2.38434108], Cost: 0.0020
Current parameters: [0.49813319 2.56655146 1.10948059 1.68938812 0.85752001 3.5493357
 2.32790787 0.76044619 2.06165322 0.37527606 0.39325747 1.00816925
 2.4503154  2.48255294 1.3724187  0.79187719 2.38866533 0.97882433
 2.43156266 2.40031276], Cost: 0.0022
Current parameters: [0.49291463 2.54847212 1.12221017 1.69918514 0.86229201 3.54422754
```



```
 2.32543699 0.75713305 2.06231315 0.37256762 0.37767155 1.0169862
 2.44722938 2.48197977 1.37529238 0.78227449 2.39495344 0.97539963
 2.4322245  2.39308944], Cost: 0.0049
Current parameters: [0.48694039 2.55773169 1.11564913 1.69883754 0.86945256 3.54757842
 2.32914855 0.75856955 2.05872273 0.37175711 0.39592292 1.02569461
 2.45867457 2.48690344 1.3777202  0.77196028 2.39835165 0.97072742
 2.44785903 2.38585378], Cost: 0.0163
Current parameters: [0.49497815 2.54520658 1.11571345 1.69570249 0.86712804 3.54706536
 2.32777464 0.7599055  2.06133    0.37552762 0.38547648 1.01294331
 2.45002293 2.48298738 1.37834224 0.78454961 2.39586314 0.975444
 2.43634346 2.39160327], Cost: 0.0013

   Return from subroutine COBYLA because the MAXFUN limit has been reached.

   NFVALS =  100   F = 1.327097E-03    MAXCV = 0.000000E+00
   X = 4.949781E-01   2.545207E+00   1.115713E+00   1.695702E+00   8.671280E-01
       3.547065E+00   2.327775E+00   7.599055E-01   2.061330E+00   3.755276E-01
       3.854765E-01   1.012943E+00   2.450023E+00   2.482987E+00   1.378342E+00
       7.845496E-01   2.395863E+00   9.754440E-01   2.436343E+00   2.391603E+00
Simulating for QAOA depth p = 11
Current parameters: [3.99262507 3.13012865 0.33890998 2.69281768 1.79657774 1.97862737
 1.60472486 1.29335441 2.31081035 2.64888411 1.59284695 0.85702493
 1.57573352 3.05948305 2.8612679  0.37437169 2.6437981  0.65022823
 0.75863698 0.3353549  1.15692122 2.06014825], Cost: 1.4690
Current parameters: [3.99262507 4.13012865 0.33890998 2.69281768 1.79657774 1.97862737
 1.60472486 1.29335441 2.31081035 2.64888411 1.59284695 0.85702493
 1.57573352 3.05948305 2.8612679  0.37437169 2.6437981  0.65022823
 0.75863698 0.3353549  1.15692122 2.06014825], Cost: 1.9507
Current parameters: [3.99262507 3.13012865 1.33890998 2.69281768 1.79657774 1.97862737
 1.60472486 1.29335441 2.31081035 2.64888411 1.59284695 0.85702493
 1.57573352 3.05948305 2.8612679  0.37437169 2.6437981  0.65022823
 0.75863698 0.3353549  1.15692122 2.06014825], Cost: 1.9623
Current parameters: [3.99262507 3.13012865 0.33890998 3.69281768 1.79657774 1.97862737
 1.60472486 1.29335441 2.31081035 2.64888411 1.59284695 0.85702493
 1.57573352 3.05948305 2.8612679  0.37437169 2.6437981  0.65022823
 0.75863698 0.3353549  1.15692122 2.06014825], Cost: 1.7847
Current parameters: [3.99262507 3.13012865 0.33890998 2.69281768 2.79657774 1.97862737
 1.60472486 1.29335441 2.31081035 2.64888411 1.59284695 0.85702493
 1.57573352 3.05948305 2.8612679  0.37437169 2.6437981  0.65022823
 0.75863698 0.3353549  1.15692122 2.06014825], Cost: 1.3037
Current parameters: [3.99262507 3.13012865 0.33890998 2.69281768 2.79657774 2.97862737
```

```
 1.60472486 1.29335441 2.31081035 2.64888411 1.59284695 0.85702493
 1.57573352 3.05948305 2.8612679  0.37437169 2.6437981  0.65022823
 0.75863698 0.3353549  1.15692122 2.06014825], Cost: 2.5448
Current parameters: [3.99262507 3.13012865 0.33890998 2.69281768 2.79657774 1.97862737
 2.60472486 1.29335441 2.31081035 2.64888411 1.59284695 0.85702493
 1.57573352 3.05948305 2.8612679  0.37437169 2.6437981  0.65022823
 0.75863698 0.3353549  1.15692122 2.06014825], Cost: 2.2637
Current parameters: [3.99262507 3.13012865 0.33890998 2.69281768 2.79657774 1.97862737
 1.60472486 2.29335441 2.31081035 2.64888411 1.59284695 0.85702493
 1.57573352 3.05948305 2.8612679  0.37437169 2.6437981  0.65022823
 0.75863698 0.3353549  1.15692122 2.06014825], Cost: 2.2806
Current parameters: [3.99262507 3.13012865 0.33890998 2.69281768 2.79657774 1.97862737
 1.60472486 1.29335441 3.31081035 2.64888411 1.59284695 0.85702493
 1.57573352 3.05948305 2.8612679  0.37437169 2.6437981  0.65022823
 0.75863698 0.3353549  1.15692122 2.06014825], Cost: 1.6212
Current parameters: [3.99262507 3.13012865 0.33890998 2.69281768 2.79657774 1.97862737
 1.60472486 1.29335441 2.31081035 3.64888411 1.59284695 0.85702493
 1.57573352 3.05948305 2.8612679  0.37437169 2.6437981  0.65022823
 0.75863698 0.3353549  1.15692122 2.06014825], Cost: 1.0929
Current parameters: [3.99262507 3.13012865 0.33890998 2.69281768 2.79657774 1.97862737
 1.60472486 1.29335441 2.31081035 3.64888411 2.59284695 0.85702493
 1.57573352 3.05948305 2.8612679  0.37437169 2.6437981  0.65022823
 0.75863698 0.3353549  1.15692122 2.06014825], Cost: 2.4881
Current parameters: [3.99262507 3.13012865 0.33890998 2.69281768 2.79657774 1.97862737
 1.60472486 1.29335441 2.31081035 3.64888411 1.59284695 1.85702493
 1.57573352 3.05948305 2.8612679  0.37437169 2.6437981  0.65022823
 0.75863698 0.3353549  1.15692122 2.06014825], Cost: 1.1327
Current parameters: [3.99262507 3.13012865 0.33890998 2.69281768 2.79657774 1.97862737
 1.60472486 1.29335441 2.31081035 3.64888411 1.59284695 0.85702493
 2.57573352 3.05948305 2.8612679  0.37437169 2.6437981  0.65022823
 0.75863698 0.3353549  1.15692122 2.06014825], Cost: 1.1675
Current parameters: [3.99262507 3.13012865 0.33890998 2.69281768 2.79657774 1.97862737
 1.60472486 1.29335441 2.31081035 3.64888411 1.59284695 0.85702493
 1.57573352 4.05948305 2.8612679  0.37437169 2.6437981  0.65022823
 0.75863698 0.3353549  1.15692122 2.06014825], Cost: 0.6102
Current parameters: [3.99262507 3.13012865 0.33890998 2.69281768 2.79657774 1.97862737
 1.60472486 1.29335441 2.31081035 3.64888411 1.59284695 0.85702493
 1.57573352 4.05948305 3.8612679  0.37437169 2.6437981  0.65022823
 0.75863698 0.3353549  1.15692122 2.06014825], Cost: 1.8630
Current parameters: [3.99262507 3.13012865 0.33890998 2.69281768 2.79657774 1.97862737
 1.60472486 1.29335441 2.31081035 3.64888411 1.59284695 0.85702493
```



```
 1.57573352 4.05948305 2.8612679  1.37437169 2.6437981  0.65022823
 0.75863698 0.3353549  1.15692122 2.06014825], Cost: 2.0395
Current parameters: [3.99262507 3.13012865 0.33890998 2.69281768 2.79657774 1.97862737
 1.60472486 1.29335441 2.31081035 3.64888411 1.59284695 0.85702493
 1.57573352 4.05948305 2.8612679  0.37437169 3.6437981  0.65022823
 0.75863698 0.3353549  1.15692122 2.06014825], Cost: 2.3479
Current parameters: [3.99262507 3.13012865 0.33890998 2.69281768 2.79657774 1.97862737
 1.60472486 1.29335441 2.31081035 3.64888411 1.59284695 0.85702493
 1.57573352 4.05948305 2.8612679  0.37437169 2.6437981  1.65022823
 0.75863698 0.3353549  1.15692122 2.06014825], Cost: 1.8606
Current parameters: [3.99262507 3.13012865 0.33890998 2.69281768 2.79657774 1.97862737
 1.60472486 1.29335441 2.31081035 3.64888411 1.59284695 0.85702493
 1.57573352 4.05948305 2.8612679  0.37437169 2.6437981  0.65022823
 1.75863698 0.3353549  1.15692122 2.06014825], Cost: 1.5808
Current parameters: [3.99262507 3.13012865 0.33890998 2.69281768 2.79657774 1.97862737
 1.60472486 1.29335441 2.31081035 3.64888411 1.59284695 0.85702493
 1.57573352 4.05948305 2.8612679  0.37437169 2.6437981  0.65022823
 0.75863698 1.3353549  1.15692122 2.06014825], Cost: 2.9415
Current parameters: [3.99262507 3.13012865 0.33890998 2.69281768 2.79657774 1.97862737
 1.60472486 1.29335441 2.31081035 3.64888411 1.59284695 0.85702493
 1.57573352 4.05948305 2.8612679  0.37437169 2.6437981  0.65022823
 0.75863698 0.3353549  2.15692122 2.06014825], Cost: 2.3949
Current parameters: [3.99262507 3.13012865 0.33890998 2.69281768 2.79657774 1.97862737
 1.60472486 1.29335441 2.31081035 3.64888411 1.59284695 0.85702493
 1.57573352 4.05948305 2.8612679  0.37437169 2.6437981  0.65022823
 0.75863698 0.3353549  1.15692122 3.06014825], Cost: 1.9669
Current parameters: [ 4.18059454  3.03725472  0.24380962  2.63195969  2.8284538   1.73933878
  1.41962407  1.10500574  2.24959353  3.6895364   1.32383094  0.84934983
  1.56134695  4.15254593  2.61970931  0.09879205  2.30874409  0.4091381
  0.571486   -0.11413712  0.81281542  1.79856173], Cost: 3.2700
Current parameters: [4.01007458 3.12150701 0.33008166 2.68716813 2.58840625 2.16754441
 1.79867224 1.48700029 2.51625809 3.86378852 1.56787375 0.85631244
 1.57439799 4.06812223 2.83884362 0.34878918 2.61269449 0.62784744
 0.74126345 0.29362782 1.12497731 2.03586474], Cost: 1.1362
Current parameters: [3.88188254 2.90599455 0.33890998 2.69281768 2.79657774 1.97862737
 1.60472486 1.29335441 2.31081035 3.64888411 1.59284695 0.85702493
 1.57573352 4.05948305 2.8612679  0.37437169 2.6437981  0.65022823
 0.75863698 0.3353549  1.15692122 2.06014825], Cost: 0.9823
Current parameters: [3.55829257 3.36805995 0.33197897 2.68838229 2.79391757 1.96617112
 1.59621786 1.28461071 2.31133214 3.65683022 1.5732408  0.85646556
 1.57468501 4.06626557 2.84366287 0.35428717 2.61937904 0.63265735
```



```
 0.74499723 0.30259548 1.13184245 2.04108357], Cost: 0.3252
Current parameters: [3.55829257 3.36805995 0.46673258 2.47780816 2.79391757 1.96617112
 1.59621786 1.28461071 2.31133214 3.65683022 1.5732408  0.85646556
 1.57468501 4.06626557 2.84366287 0.35428717 2.61937904 0.63265735
 0.74499723 0.30259548 1.13184245 2.04108357], Cost: 0.6550
Current parameters: [3.55719535 3.38170249 0.74602322 2.96570119 2.79242392 1.95917712
 1.59144132 1.27970125 2.31162512 3.66129184 1.56223225 0.85615148
 1.57409629 4.07007384 2.83377792 0.34301003 2.60566811 0.62279157
 0.73733873 0.28420158 1.11776111 2.03037904], Cost: 0.9066
Current parameters: [3.55829257 3.36805995 0.33197897 2.68838229 2.77962867 2.11087046
 1.61050676 1.08275579 2.32562103 3.67111911 1.5732408  0.85646556
 1.57468501 4.06626557 2.84366287 0.35428717 2.61937904 0.63265735
 0.74499723 0.30259548 1.13184245 2.04108357], Cost: 0.6636
Current parameters: [3.55652194 3.39007545 0.31196274 2.69551543 2.93596965 2.30285209
 1.44404732 1.50657041 2.16734249 3.51956767 1.55547586 0.85595872
 1.57373497 4.07241113 2.82771114 0.3360888  2.59725317 0.61673655
 0.7326384  0.27291251 1.10911883 2.02380925], Cost: 0.3389
Current parameters: [3.55829257 3.36805995 0.33197897 2.68838229 2.72315047 1.96617112
 1.67116979 1.28461071 2.09484601 3.72759732 1.5732408  0.85646556
 1.57468501 4.06626557 2.84366287 0.35428717 2.61937904 0.63265735
 0.74499723 0.30259548 1.13184245 2.04108357], Cost: 0.1931
Current parameters: [3.55675058 3.38723259 0.31454744 2.69459433 2.93183573 1.95606101
 2.07243879 1.29490325 2.09193371 3.52308311 1.55776985 0.85602417
 1.57385765 4.07161756 2.82977099 0.33843876 2.60011029 0.6187924
 0.7342343  0.27674547 1.11205314 2.02603989], Cost: 0.6915
Current parameters: [3.55829257 3.36805995 0.33197897 2.68838229 2.54950114 1.96617112
 1.67116979 1.28461071 2.09484601 3.55394799 1.52642388 0.85646556
 1.57468501 4.06626557 2.84366287 0.35428717 2.61937904 0.63265735
 0.74499723 0.30259548 1.13184245 2.04108357], Cost: 0.6216
Current parameters: [3.55551081 3.40264752 0.30053237 2.69958885 2.67908712 1.94793241
 1.65369558 1.30317852 2.07879165 3.69028607 2.05818312 0.85566929
 1.57319245 4.07592059 2.81860182 0.32569652 2.58461808 0.60764489
 0.72558082 0.25596189 1.09614239 2.01394466], Cost: 0.6073
Current parameters: [3.55883326 3.3677928  0.33170542 2.68820723 2.72324216 1.96548281
 1.67063736 1.28406893 2.09466993 3.72771425 1.57246698 0.85646556
 1.32468936 4.06626557 2.84366287 0.35428717 2.61937904 0.63265735
 0.74499723 0.30259548 1.13184245 2.04108357], Cost: 0.5010
Current parameters: [3.62404549 3.45835178 0.17726016 2.70519192 2.81839705 1.80195204
 1.53169658 1.27706104 2.01073439 3.85135466 1.43785389 0.85352197
 1.66732609 4.10195751 2.7510191  0.24859548 2.49087743 0.54019324
 0.67322014 0.13020403 0.99986926 1.94075859], Cost: 1.0376
```



```
Current parameters: [3.55829257 3.36805995 0.33197897 2.68838229 2.72315047 1.96617112
 1.67116979 1.28461071 2.09484601 3.72759732 1.5732408  0.606502
 1.57468501 4.06626557 2.84366287 0.35428717 2.61937904 0.63265735
 0.74499723 0.30686362 1.13184245 2.04108357], Cost: 0.7142
Current parameters: [3.5468816  3.4204752  0.29148078 2.70659157 2.75325473 1.95002273
 1.653938   1.31826979 2.07467545 3.76686135 1.57384347 0.92328015
 1.609394   4.07986365 2.80836701 0.31402025 2.57042189 0.59742993
 0.71765126 0.77536324 1.08156268 2.00286128], Cost: 1.7490
Current parameters: [3.55829257 3.36805995 0.33197897 2.68838229 2.72315047 1.96617112
 1.67116979 1.28461071 2.09484601 3.72759732 1.5732408  0.85646556
 1.57468501 4.06626557 2.84366287 0.35428717 2.44026219 0.63265735
 0.74499723 0.30259548 1.30624759 2.04108357], Cost: 0.7565
Current parameters: [3.54620624 3.42357741 0.28908389 2.7076693  2.75503646 1.94906699
 1.65291813 1.32026191 2.07348165 3.7691852  1.57387914 0.91738627
 1.61144826 4.08066846 2.80627802 0.31163704 2.99239237 0.59534499
 0.71603278 0.22657785 1.41852209 2.00059908], Cost: 2.8147
Current parameters: [3.55829257 3.36805995 0.33197897 2.68838229 2.72315047 1.96617112
 1.67116979 1.28461071 2.09484601 3.72759732 1.5732408  0.85646556
 1.57468501 4.06626557 2.84366287 0.52640047 2.61937904 0.63265735
 0.74499723 0.30259548 1.13184245 1.85976337], Cost: 0.3440
Current parameters: [3.55017208 3.40536068 0.30315889 2.70134072 2.74457383 1.9546793
 1.65890698 1.30856381 2.08049186 3.75553913 1.57366968 0.89739659
 1.59938528 4.07594249 2.81854497 0.69172951 2.58507057 0.60758815
 0.72553678 0.25152123 1.0318311  2.37808452], Cost: 1.7207
Current parameters: [3.55829257 3.36805995 0.33197897 2.68838229 2.72315047 1.96617112
 1.67116979 1.28461071 2.09484601 3.72759732 1.5732408  0.85646556
 1.57468501 4.06626557 3.02026791 0.35428717 2.61937904 0.45570916
 0.74499723 0.30259548 1.13184245 2.04108357], Cost: 0.7852
Current parameters: [3.55145608 3.39946274 0.30771588 2.69929175 2.7411864  1.95649637
 1.66084596 1.30477638 2.08276152 3.75112101 1.57360187 0.89092463
 1.5954797  4.07441239 3.15489115 0.30646335 2.59049538 0.99976531
 0.72861384 0.25959702 1.04764476 2.00974157], Cost: 1.1695
Current parameters: [3.55829257 3.36805995 0.33197897 2.68838229 2.72315047 1.96617112
 1.67116979 1.28461071 2.09484601 3.72759732 1.5732408  0.85646556
 1.57468501 4.29011708 2.84366287 0.35428717 2.61937904 0.63265735
 0.85630985 0.30259548 1.13184245 2.04108357], Cost: 0.0818
Current parameters: [3.55426417 3.38656403 0.31768195 2.69481069 2.73377813 1.96047027
 1.66508648 1.29649333 2.08772523 3.74145865 1.57345356 0.87677056
 1.58693828 4.0748143  2.8089158  0.32610699 2.60235934 0.6312604
 1.29922491 0.27725865 1.08222898 2.0226153 ], Cost: 0.9691
Current parameters: [3.56281038 3.36582774 0.32969325 2.68691958 2.63110506 2.05890554
```

```
 1.7629325  1.37856946 2.17410612 3.85276255 1.55912644 0.87635321
 1.5944211  4.27702361 2.80884532 0.31952338 2.61201878 0.62667532
 0.83690996 0.27465395 1.09509587 2.03615932], Cost: 0.0159
Current parameters: [3.58601157 3.43396701 0.23921295 2.70410831 2.56676688 2.04152794
 1.77941831 1.45750132 2.37310704 3.72845039 1.57792773 0.76577226
 1.44340537 4.52204548 2.8853665  0.41101097 2.44687368 0.56720807
 0.86304636 0.22710544 1.07118685 1.87378668], Cost: 0.4582
Current parameters: [3.43186014 3.19309993 0.26596649 2.72684086 2.64811803 1.99504639
 1.74767556 1.41833528 2.23063011 3.84764958 1.5587289  0.88450438
 1.60264304 4.26992958 2.79958023 0.3109477  2.61563457 0.62900115
 0.83277921 0.27223646 1.08706763 2.039313  ], Cost: 0.1401
Current parameters: [3.57027628 3.12463049 0.50404405 2.56269451 2.6063353  2.25125741
 1.78365341 1.26105027 1.97149869 3.85364893 1.56861885 0.87461697
 1.56107646 4.39791045 2.77028572 0.30009815 2.51681435 0.60269782
 0.81295144 0.2087305  0.98889829 1.93861351], Cost: 0.9191
Current parameters: [3.56079421 3.38048822 0.31852138 2.68977382 2.63523383 2.06324087
 1.76210727 1.39312071 2.16885884 3.86966571 1.55944842 0.89513692
 1.60302433 4.29680737 2.78132493 0.48933052 2.58731723 0.63148369
 0.82477043 0.24435968 1.03903386 2.19843997], Cost: 0.4679
Current parameters: [3.66731224 3.30855571 0.32373435 2.70658835 2.63669958 2.04348507
 1.7606583  1.39236916 2.19042205 3.84936479 1.56391674 0.8789043
 1.59711557 4.27321496 2.81054405 0.32274826 2.61920099 0.63179397
 0.83912751 0.28213934 1.09899898 2.04256112], Cost: 0.0556
Current parameters: [3.54459753 3.38825439 0.50619001 2.84656191 2.64250041 2.0191231
 1.74952738 1.39911439 2.20336415 3.85102801 1.55409861 0.88318252
 1.59813885 4.28428598 2.79211667 0.30725673 2.59909149 0.62153099
 0.82719235 0.2568632  1.07070357 2.02683604], Cost: 0.1736
Current parameters: [3.56227804 3.35931524 0.34011114 2.67960411 2.56438399 2.04363826
 1.68270234 1.35634997 2.18194328 3.90520852 1.56462931 0.86765645
 1.58577411 4.2830096  2.8233305  0.33132955 2.61441344 0.62857938
 0.84482696 0.28575027 1.11029129 2.03511566], Cost: 0.0798
Current parameters: [3.56638054 3.36372297 0.3497772  2.65285481 2.58570236 1.92109152
 1.90457827 1.2496272  2.20538127 3.89751776 1.56608432 0.87873019
 1.5936785  4.28540939 2.80764782 0.32268351 2.61073994 0.63037617
 0.83748974 0.27724884 1.08947099 2.03422321], Cost: 0.0965
Current parameters: [3.56231218 3.35674582 0.33911753 2.68427234 2.64065957 2.06548562
 1.76920249 1.37487539 2.17867047 3.86055231 1.44202621 0.86672245
 1.58488222 4.28316019 2.82630813 0.33419707 2.61697643 0.63014281
 0.84676304 0.28944121 1.11435248 2.03646312], Cost: 0.1027
Current parameters: [3.55838164 3.39899003 0.31023314 2.69022552 2.64035338 2.06520943
 1.78153247 1.40168509 2.17144408 3.85944266 1.58890532 0.90237693
```



```
 1.59716981 4.33681022 2.76398135 0.30610508 2.78404462 0.63161784
 0.81679385 0.20878088 1.22598288 2.00295885], Cost: 0.6352
Current parameters: [3.54873066 3.35266244 0.35379986 2.68979915 2.62254099 2.08664592
 1.79208479 1.38783022 2.17888546 3.8450773  1.58886238 0.87110002
 1.58839739 4.27629425 2.88295321 0.33824252 2.62490117 0.69256064
 0.85121927 0.30193371 1.11652295 2.0460439 ], Cost: 0.1007
Current parameters: [3.58230161 3.41094907 0.271642   2.69091084 2.66277976 2.00992472
 1.72204574 1.38277053 2.15279723 3.88846294 1.52484979 0.91969451
 1.62941932 4.28463683 2.86908229 0.24885868 2.5856126  0.75495974
 0.78921313 0.18685285 1.00079346 2.00635022], Cost: 0.9141
Current parameters: [3.5619127  3.36992756 0.3262826  2.68873241 2.63435074 2.05742819
 1.76182251 1.38151647 2.17177804 3.85715814 1.55948124 0.88299133
 1.59979521 4.31645289 2.80337801 0.31457046 2.61508672 0.62881129
 0.74554733 0.34863884 1.0905433  2.0388675 ], Cost: 0.0437
Current parameters: [3.55768475 3.39955216 0.3095165  2.69057459 2.6402565  2.06278055
 1.77427514 1.4000947  2.17253693 3.85924648 1.5809542  0.90255246
 1.59833152 4.24423408 2.738364   0.29970533 2.60875877 0.59827998
 0.9785505  0.44446943 1.05288902 2.00345883], Cost: 0.5324
Current parameters: [3.56831909 3.38372672 0.30913847 2.68855981 2.61413816 2.05741432
 1.76940684 1.3957842  2.22470904 3.81587368 1.56799657 0.84232586
 1.55606185 4.32804328 2.82699306 0.33967376 2.56838324 0.59905941
 0.83965009 0.2635431  1.09752564 1.99808881], Cost: 0.0240
Current parameters: [3.56279537 3.36373216 0.3039569  2.72831271 2.62038513 2.07598543
 1.77161448 1.355859   2.15370688 3.85850115 1.5585539  0.87277736
 1.59085378 4.28049196 2.81111233 0.32149948 2.60865088 0.62389798
 0.83794681 0.2741801  1.09675043 2.03311666], Cost: 0.0202
Current parameters: [3.561964   3.30642664 0.37653568 2.64824997 2.62549489 2.10584943
 1.77147955 1.35304004 2.13047435 3.84573242 1.56825218 0.87192117
 1.58351404 4.30607899 2.79209059 0.31549252 2.58986614 0.60720454
 0.83234837 0.25926515 1.07642045 2.01059046], Cost: 0.0433
Current parameters: [3.56281395 3.36582598 0.32969145 2.68691842 2.62114627 2.03632359
 1.77239422 1.41006126 2.13809718 3.86042849 1.55881206 0.84725891
 1.59276965 4.27850236 2.81001196 0.32066035 2.61083555 0.62550642
 0.83764528 0.27515074 1.09624798 2.03496153], Cost: 0.0065
Current parameters: [3.56267968 3.36552717 0.3312493  2.6850682  2.64447412 2.0587701
 1.76646192 1.38199628 2.16975196 3.8672892  1.56591294 0.74526596
 1.61360667 4.26427428 2.78866611 0.30703603 2.62896368 0.63846779
 0.83066256 0.27670441 1.08059117 2.04820062], Cost: 0.1030
Current parameters: [3.56269162 3.36588642 0.32975334 2.68695803 2.61835622 2.03647932
 1.77251468 1.41018383 2.13813702 3.85763272 1.55824051 0.84725891
 1.6493311  4.29027147 2.81929706 0.32970929 2.60141839 0.61620328
```



```
 0.8434976  0.27515074 1.10541742 2.02542853], Cost: 0.0243
Current parameters: [3.56273288 3.36565395 0.33059783 2.68584479 2.541538   2.0376547
 1.77386095 1.41009929 2.13780784 3.78039694 1.53905735 0.84673487
 1.60629625 4.25968235 2.78928586 0.30463083 2.6298112  0.64137888
 0.82834046 0.27505057 1.07892989 2.05120147], Cost: 0.0853
Current parameters: [3.56281395 3.36582598 0.32969145 2.68691842 2.62114627 2.03632359
 1.77239422 1.41006126 2.13809718 3.86042849 1.55881206 0.84725891
 1.59276965 4.27748688 2.77961403 0.31987957 2.57838018 0.65596342
 0.83714032 0.27515074 1.12784961 2.03578407], Cost: 0.0196
Current parameters: [3.56255066 3.36480734 0.3345118  2.68104629 2.61980066 2.04330958
 1.78016548 1.41009108 2.13647271 3.85679845 1.56744173 0.84442051
 1.58474847 4.23318756 2.84649538 0.27737312 2.56998046 0.56244485
 0.81540251 0.2746082  1.14230274 2.07705966], Cost: 0.2269
Current parameters: [3.5607168  3.37131923 0.33116177 2.67983615 2.62573779 2.03918939
 1.77162919 1.40997789 2.14480573 3.85809519 1.56154154 0.85278701
 1.5954171  4.2352299  2.80547554 0.34627182 2.61239863 0.62277971
 0.82023172 0.27087802 1.09582396 2.00644098], Cost: 0.0131
Current parameters: [3.57691174 3.32682804 0.32542564 2.72848894 2.58795105 2.02508918
 1.78707166 1.41067053 2.09001048 3.8720694  1.55050681 0.80581593
 1.56484854 4.30135491 2.82953992 0.36464311 2.60170808 0.62380646
 0.82114702 0.30386608 1.10519169 1.99495867], Cost: 0.1788
Current parameters: [3.5608469  3.36944076 0.32870209 2.6838647  2.62324564 2.04008219
 1.77454165 1.41363653 2.13795706 3.86225275 1.56290109 0.8522789
 1.5925427  4.2841638  2.79837232 0.363594   2.60559219 0.61965859
 0.83614058 0.26634381 1.08404301 2.07364555], Cost: 0.0674
Current parameters: [3.56371745 3.36456042 0.32988256 2.6887589  2.60871826 2.04727454
 1.78403877 1.42196816 2.14692085 3.876598   1.55649009 0.84869929
 1.59474764 4.27797817 2.80647511 0.31654751 2.60959683 0.62524672
 0.83495608 0.27242935 1.09169564 2.03368861], Cost: 0.0047
Current parameters: [3.55821538 3.37186593 0.37513709 2.72575225 2.61452255 2.03768856
 1.77986539 1.42604922 2.15579943 3.87332087 1.55731519 0.85214603
 1.59574379 4.27833483 2.79988316 0.31386224 2.60707302 0.62139059
 0.83346608 0.2664686  1.08606192 2.03233541], Cost: 0.0066
Current parameters: [3.56372512 3.36398836 0.32670207 2.69411061 2.61339731 2.06436605
 1.76687157 1.43837303 2.1416837  3.87165042 1.55508471 0.84739484
 1.59433597 4.27829932 2.8073464  0.31637028 2.61023941 0.62533473
 0.83455319 0.27269348 1.09312258 2.03314251], Cost: 0.0034
Current parameters: [3.56149016 3.36978856 0.32741536 2.69156315 2.61588599 2.06640261
 1.77338035 1.44189636 2.14405424 3.87158213 1.56360062 0.85367257
 1.5912735  4.2937448  2.79093356 0.31685294 2.65107831 0.6169799
 0.83246284 0.25625077 1.12597734 2.0248601 ], Cost: 0.0466
```



```
Current parameters: [3.56568931 3.36335102 0.32502305 2.69454966 2.61384464 2.06138704
 1.76393327 1.43663951 2.13951043 3.87300895 1.55145361 0.84656824
 1.59563289 4.28233524 2.81719488 0.31436252 2.60863767 0.63466411
 0.81826708 0.25144958 1.09175177 2.03420066], Cost: 0.0192
Current parameters: [3.56581964 3.38534215 0.31733663 2.68875264 2.6285661  2.05738415
 1.76309279 1.44457032 2.13726501 3.8777995  1.56012807 0.86790497
 1.59541274 4.28242281 2.79758954 0.29802427 2.60692205 0.63882254
 0.85424404 0.27508063 1.06144888 2.01181211], Cost: 0.0349
Current parameters: [3.58100137 3.38122436 0.33374623 2.69310801 2.60791569 2.07148929
 1.76970167 1.43377522 2.13088025 3.87355328 1.55374281 0.84243113
 1.59137012 4.28118285 2.81090998 0.32055794 2.60905937 0.6254759
 0.83357552 0.27594778 1.09545623 2.03032741], Cost: 0.0110
Current parameters: [3.55993572 3.33720252 0.31784886 2.71691045 2.60320637 2.05169633
 1.77248943 1.44432404 2.12171596 3.87850915 1.55358867 0.8341849
 1.58096087 4.29044492 2.8110363  0.33222906 2.60714624 0.62681556
 0.83337071 0.2884844  1.08898057 2.00573526], Cost: 0.0289
Current parameters: [3.56336864 3.37076672 0.32282592 2.69145455 2.61250447 2.0637605
 1.76779199 1.44211386 2.15902982 3.86128221 1.55395212 0.8419924
 1.58576479 4.28875873 2.81079811 0.32084978 2.60039128 0.61841189
 0.83671907 0.26853252 1.09370213 2.0246896 ], Cost: 0.0033
Current parameters: [3.55897223 3.36918585 0.32865349 2.68627936 2.62493083 2.06503834
 1.77022698 1.44008227 2.1628819  3.86569744 1.49553535 0.84572545
 1.58347364 4.28748729 2.81294858 0.3245835  2.60625721 0.62063973
 0.84582387 0.27376497 1.09818284 2.02341202], Cost: 0.0319
Current parameters: [3.56489257 3.36726484 0.32380975 2.69274227 2.60568837 2.0636048
 1.76403058 1.44034873 2.15644675 3.86446128 1.554872   0.83777388
 1.58336967 4.28058331 2.81422713 0.32330633 2.59954667 0.61861643
 0.85901351 0.25225417 1.09611865 2.02359689], Cost: 0.0017
Current parameters: [3.55917158 3.37143868 0.32948641 2.68235732 2.58087461 2.05335463
 1.72131421 1.42958845 2.17073345 3.88846022 1.55541905 0.84276715
 1.58054963 4.28670042 2.81484293 0.32615462 2.60209277 0.61786646
 0.85426463 0.26456312 1.09901378 2.02112232], Cost: 0.0224
Current parameters: [3.54960495 3.36369496 0.33478355 2.68102372 2.60433368 2.07545194
 1.76558834 1.43295994 2.14697788 3.86315373 1.55606641 0.83746197
 1.5809368  4.28680918 2.81061964 0.32196804 2.59421779 0.61416869
 0.85774697 0.24812338 1.09176301 2.01768497], Cost: 0.0010
Current parameters: [3.58959595 3.32823538 0.34949893 2.66730995 2.61209833 2.08030227
 1.75972767 1.42936272 2.15698944 3.85766928 1.55866283 0.84794199
 1.58025388 4.2923755  2.80044017 0.31982041 2.59207325 0.6084833
 0.85742673 0.24605852 1.0845898  2.01180583], Cost: 0.0138
Current parameters: [3.54712094 3.36158765 0.34113743 2.67811467 2.60089707 2.07723551
```

```
 1.77011843 1.43838808 2.1477438  3.85949006 1.56029705 0.85582346
 1.576978   4.28852649 2.82694109 0.3272149  2.59400153 0.62416564
 0.86125997 0.25272886 1.09784654 2.01769368], Cost: 0.0162
Current parameters: [3.54440014 3.35925038 0.34421112 2.67698511 2.61743399 2.09052793
 1.76860149 1.42528639 2.16768685 3.86513804 1.56883133 0.8006507
 1.5858491  4.28344266 2.82033159 0.32034159 2.6078135  0.64213829
 0.86423391 0.26004846 1.08451312 2.01802656], Cost: 0.0102
Current parameters: [3.54960179 3.36364946 0.33495473 2.68081488 2.62269243 2.07574644
 1.76588971 1.43291831 2.1469211  3.8814212  1.56136286 0.83736577
 1.57722967 4.28745153 2.81838162 0.32212554 2.58633688 0.60533698
 0.85807749 0.24810116 1.09968511 2.01737902], Cost: 0.0031
Current parameters: [3.54612067 3.36042641 0.33331745 2.68195399 2.59262835 2.07631069
 1.76935848 1.43505218 2.14634525 3.84781083 1.56070447 0.82422359
 1.57712653 4.25927992 2.81851789 0.28427785 2.58022234 0.58939407
 0.85527106 0.25805327 1.09661558 2.02759712], Cost: 0.0353
Current parameters: [3.54245453 3.35632533 0.33432807 2.68193273 2.60833312 2.08277469
 1.75904991 1.43990126 2.14540137 3.86348808 1.55806585 0.84159163
 1.58533652 4.27954301 2.80305281 0.33184114 2.59451406 0.61089203
 0.86201806 0.25421715 1.08752142 2.03704491], Cost: 0.0050
Current parameters: [3.55833314 3.38327899 0.33010239 2.67748754 2.59896775 2.06343505
 1.78504092 1.42574858 2.15040693 3.86224777 1.56382917 0.82258935
 1.5677178  4.3023739  2.80476106 0.35085461 2.58159863 0.60612004
 0.85334314 0.23100253 1.06968901 2.03199983], Cost: 0.0502
Current parameters: [3.54950351 3.36413936 0.33432259 2.68121021 2.60272021 2.07489889
 1.76643202 1.43301973 2.14710936 3.86141324 1.55650692 0.83567845
 1.60911434 4.29250251 2.81494818 0.32566973 2.58875598 0.60860643
 0.86127404 0.248533   1.09467137 2.01264535], Cost: 0.0079
Current parameters: [3.55003699 3.36235067 0.33192553 2.68659684 2.6033082  2.07879497
 1.76566924 1.4275983  2.14713901 3.86344521 1.55547608 0.83885064
 1.58019894 4.2959593  2.81166738 0.31675253 2.59367761 0.6145732
 0.8613796  0.24891104 1.0919006  2.02340254], Cost: 0.0003
Current parameters: [3.54941164 3.36355899 0.32437874 2.69698556 2.60423359 2.09095005
 1.76574449 1.41149449 2.15374077 3.86198401 1.5566556  0.84679351
 1.58033356 4.28419286 2.81050557 0.32369698 2.59296489 0.61271361
 0.85694933 0.24768847 1.0908149  2.01460577], Cost: 0.0038
Current parameters: [3.55139766 3.36054397 0.32073719 2.67745163 2.60186794 2.08116958
 1.76670596 1.42659436 2.14494766 3.86426226 1.55527111 0.8379991
 1.57995349 4.29583399 2.81219063 0.3173863  2.59312125 0.61663361
 0.86172849 0.25038358 1.09444027 2.02376645], Cost: 0.0002
Current parameters: [3.55095534 3.36140488 0.3235399  2.68051059 2.602138   2.08021862
 1.76699195 1.42657881 2.14579682 3.86373643 1.55589658 0.83704972
```



```
 1.579972   4.29507413 2.79619073 0.31626199 2.57604215 0.63092944
 0.8617723  0.25024035 1.10862252 2.02375933], Cost: 0.0067
Current parameters: [3.55054246 3.35355094 0.31881182 2.6828224  2.59964143 2.07880564
 1.76649801 1.42784153 2.13994103 3.86575846 1.55398621 0.83457895
 1.57783383 4.29618873 2.81473438 0.32058955 2.58834684 0.61798464
 0.86146994 0.25560616 1.09021798 2.01952188], Cost: 0.0011
Current parameters: [3.54641155 3.35394319 0.31703331 2.6837048  2.60164737 2.0811976
 1.76983798 1.43129876 2.14062336 3.86491905 1.56131031 0.83519632
 1.57714402 4.30185353 2.80175334 0.31880247 2.61166387 0.6106714
 0.86315697 0.25020588 1.10689819 2.01551159], Cost: 0.0079
Current parameters: [3.55139766 3.36054397 0.32073719 2.67745163 2.60186794 2.08116958
 1.76670596 1.42659436 2.14494766 3.86426226 1.55527111 0.8379991
 1.57995349 4.29583399 2.81219063 0.3173863  2.59312125 0.61663361
 0.86172849 0.25038358 1.09444027 2.02376645], Cost: 0.0002

   Return from subroutine COBYLA because the MAXFUN limit has been reached.

   NFVALS =  100   F = 2.014295E-04    MAXCV = 0.000000E+00
   X = 3.551398E+00   3.360544E+00   3.207372E-01   2.677452E+00   2.601868E+00
       2.081170E+00   1.766706E+00   1.426594E+00   2.144948E+00   3.864262E+00
       1.555271E+00   8.379991E-01   1.579953E+00   4.295834E+00   2.812191E+00
       3.173863E-01   2.593121E+00   6.166336E-01   8.617285E-01   2.503836E-01
       1.094440E+00   2.023766E+00
Simulating for QAOA depth p = 12
Current parameters: [2.23373981 1.50408846 2.61413907 1.94823449 1.98882034 0.15208944
 0.29679727 2.02209508 0.26040949 1.77751638 1.73087743 2.61214673
 2.16562904 2.90969964 1.452708   0.23952993 0.06562486 2.70500833
 1.25225257 2.96386908 0.51951942 2.38630966 1.55764102 2.78654465], Cost: 1.8190
Current parameters: [1.23373981 2.50408846 2.61413907 1.94823449 1.98882034 0.15208944
 0.29679727 2.02209508 0.26040949 1.77751638 1.73087743 2.61214673
 2.16562904 2.90969964 1.452708   0.23952993 0.06562486 2.70500833
 1.25225257 2.96386908 0.51951942 2.38630966 1.55764102 2.78654465], Cost: 1.5763
Current parameters: [1.23373981 2.50408846 3.61413907 1.94823449 1.98882034 0.15208944
 0.29679727 2.02209508 0.26040949 1.77751638 1.73087743 2.61214673
 2.16562904 2.90969964 1.452708   0.23952993 0.06562486 2.70500833
 1.25225257 2.96386908 0.51951942 2.38630966 1.55764102 2.78654465], Cost: 1.2537
Current parameters: [1.23373981 2.50408846 3.61413907 2.94823449 1.98882034 0.15208944
 0.29679727 2.02209508 0.26040949 1.77751638 1.73087743 2.61214673
 2.16562904 2.90969964 1.452708   0.23952993 0.06562486 2.70500833
 1.25225257 2.96386908 0.51951942 2.38630966 1.55764102 2.78654465], Cost: 3.0459
Current parameters: [1.23373981 2.50408846 3.61413907 1.94823449 2.98882034 0.15208944
```



```
 0.29679727 2.02209508 0.26040949 1.77751638 1.73087743 2.61214673
 2.16562904 2.90969964 1.452708   0.23952993 0.06562486 2.70500833
 1.25225257 2.96386908 0.51951942 2.38630966 1.55764102 2.78654465], Cost: 2.5079
Current parameters: [1.23373981 2.50408846 3.61413907 1.94823449 1.98882034 1.15208944
 0.29679727 2.02209508 0.26040949 1.77751638 1.73087743 2.61214673
 2.16562904 2.90969964 1.452708   0.23952993 0.06562486 2.70500833
 1.25225257 2.96386908 0.51951942 2.38630966 1.55764102 2.78654465], Cost: 2.7625
Current parameters: [1.23373981 2.50408846 3.61413907 1.94823449 1.98882034 0.15208944
 1.29679727 2.02209508 0.26040949 1.77751638 1.73087743 2.61214673
 2.16562904 2.90969964 1.452708   0.23952993 0.06562486 2.70500833
 1.25225257 2.96386908 0.51951942 2.38630966 1.55764102 2.78654465], Cost: 1.3372
Current parameters: [1.23373981 2.50408846 3.61413907 1.94823449 1.98882034 0.15208944
 0.29679727 3.02209508 0.26040949 1.77751638 1.73087743 2.61214673
 2.16562904 2.90969964 1.452708   0.23952993 0.06562486 2.70500833
 1.25225257 2.96386908 0.51951942 2.38630966 1.55764102 2.78654465], Cost: 0.5557
Current parameters: [1.23373981 2.50408846 3.61413907 1.94823449 1.98882034 0.15208944
 0.29679727 3.02209508 1.26040949 1.77751638 1.73087743 2.61214673
 2.16562904 2.90969964 1.452708   0.23952993 0.06562486 2.70500833
 1.25225257 2.96386908 0.51951942 2.38630966 1.55764102 2.78654465], Cost: 0.8667
Current parameters: [1.23373981 2.50408846 3.61413907 1.94823449 1.98882034 0.15208944
 0.29679727 3.02209508 0.26040949 2.77751638 1.73087743 2.61214673
 2.16562904 2.90969964 1.452708   0.23952993 0.06562486 2.70500833
 1.25225257 2.96386908 0.51951942 2.38630966 1.55764102 2.78654465], Cost: 2.4546
Current parameters: [1.23373981 2.50408846 3.61413907 1.94823449 1.98882034 0.15208944
 0.29679727 3.02209508 0.26040949 1.77751638 2.73087743 2.61214673
 2.16562904 2.90969964 1.452708   0.23952993 0.06562486 2.70500833
 1.25225257 2.96386908 0.51951942 2.38630966 1.55764102 2.78654465], Cost: 2.4967
Current parameters: [1.23373981 2.50408846 3.61413907 1.94823449 1.98882034 0.15208944
 0.29679727 3.02209508 0.26040949 1.77751638 1.73087743 3.61214673
 2.16562904 2.90969964 1.452708   0.23952993 0.06562486 2.70500833
 1.25225257 2.96386908 0.51951942 2.38630966 1.55764102 2.78654465], Cost: 2.4821
Current parameters: [1.23373981 2.50408846 3.61413907 1.94823449 1.98882034 0.15208944
 0.29679727 3.02209508 0.26040949 1.77751638 1.73087743 2.61214673
 3.16562904 2.90969964 1.452708   0.23952993 0.06562486 2.70500833
 1.25225257 2.96386908 0.51951942 2.38630966 1.55764102 2.78654465], Cost: 3.0430
Current parameters: [1.23373981 2.50408846 3.61413907 1.94823449 1.98882034 0.15208944
 0.29679727 3.02209508 0.26040949 1.77751638 1.73087743 2.61214673
 2.16562904 3.90969964 1.452708   0.23952993 0.06562486 2.70500833
 1.25225257 2.96386908 0.51951942 2.38630966 1.55764102 2.78654465], Cost: 2.1414
Current parameters: [1.23373981 2.50408846 3.61413907 1.94823449 1.98882034 0.15208944
 0.29679727 3.02209508 0.26040949 1.77751638 1.73087743 2.61214673
```



```
 2.16562904 2.90969964 2.452708   0.23952993 0.06562486 2.70500833
 1.25225257 2.96386908 0.51951942 2.38630966 1.55764102 2.78654465], Cost: 2.4806
Current parameters: [1.23373981 2.50408846 3.61413907 1.94823449 1.98882034 0.15208944
 0.29679727 3.02209508 0.26040949 1.77751638 1.73087743 2.61214673
 2.16562904 2.90969964 1.452708   1.23952993 0.06562486 2.70500833
 1.25225257 2.96386908 0.51951942 2.38630966 1.55764102 2.78654465], Cost: 2.5281
Current parameters: [1.23373981 2.50408846 3.61413907 1.94823449 1.98882034 0.15208944
 0.29679727 3.02209508 0.26040949 1.77751638 1.73087743 2.61214673
 2.16562904 2.90969964 1.452708   0.23952993 1.06562486 2.70500833
 1.25225257 2.96386908 0.51951942 2.38630966 1.55764102 2.78654465], Cost: 2.1205
Current parameters: [1.23373981 2.50408846 3.61413907 1.94823449 1.98882034 0.15208944
 0.29679727 3.02209508 0.26040949 1.77751638 1.73087743 2.61214673
 2.16562904 2.90969964 1.452708   0.23952993 0.06562486 3.70500833
 1.25225257 2.96386908 0.51951942 2.38630966 1.55764102 2.78654465], Cost: 1.5082
Current parameters: [1.23373981 2.50408846 3.61413907 1.94823449 1.98882034 0.15208944
 0.29679727 3.02209508 0.26040949 1.77751638 1.73087743 2.61214673
 2.16562904 2.90969964 1.452708   0.23952993 0.06562486 2.70500833
 2.25225257 2.96386908 0.51951942 2.38630966 1.55764102 2.78654465], Cost: 2.0031
Current parameters: [1.23373981 2.50408846 3.61413907 1.94823449 1.98882034 0.15208944
 0.29679727 3.02209508 0.26040949 1.77751638 1.73087743 2.61214673
 2.16562904 2.90969964 1.452708   0.23952993 0.06562486 2.70500833
 1.25225257 3.96386908 0.51951942 2.38630966 1.55764102 2.78654465], Cost: 1.6909
Current parameters: [1.23373981 2.50408846 3.61413907 1.94823449 1.98882034 0.15208944
 0.29679727 3.02209508 0.26040949 1.77751638 1.73087743 2.61214673
 2.16562904 2.90969964 1.452708   0.23952993 0.06562486 2.70500833
 1.25225257 2.96386908 1.51951942 2.38630966 1.55764102 2.78654465], Cost: 2.0533
Current parameters: [1.23373981 2.50408846 3.61413907 1.94823449 1.98882034 0.15208944
 0.29679727 3.02209508 0.26040949 1.77751638 1.73087743 2.61214673
 2.16562904 2.90969964 1.452708   0.23952993 0.06562486 2.70500833
 1.25225257 2.96386908 0.51951942 3.38630966 1.55764102 2.78654465], Cost: 2.5878
Current parameters: [1.23373981 2.50408846 3.61413907 1.94823449 1.98882034 0.15208944
 0.29679727 3.02209508 0.26040949 1.77751638 1.73087743 2.61214673
 2.16562904 2.90969964 1.452708   0.23952993 0.06562486 2.70500833
 1.25225257 2.96386908 0.51951942 2.38630966 2.55764102 2.78654465], Cost: 1.3000
Current parameters: [1.23373981 2.50408846 3.61413907 1.94823449 1.98882034 0.15208944
 0.29679727 3.02209508 0.26040949 1.77751638 1.73087743 2.61214673
 2.16562904 2.90969964 1.452708   0.23952993 0.06562486 2.70500833
 1.25225257 2.96386908 0.51951942 2.38630966 1.55764102 3.78654465], Cost: 0.5629
Current parameters: [ 1.22113623  2.52622618  3.66029368  1.69177514  1.80935206 -0.06380828
  0.2848484   3.12198175  0.21590898  1.50578006  1.45312326  2.33647627
  1.80970818  2.68278656  1.17726331 -0.04271673 -0.15829959  2.56870762
```

```
 1.04512534  2.80142031  0.30520966  2.09551797  1.45113717  2.78551498], Cost: 1.0823
Current parameters: [1.23218519 2.5068191  3.61983214 2.43381254 1.96668335 0.12545897
 0.29532341 3.03441587 0.25492046 1.74399834 1.6966171  2.57814342
 2.12172704 2.88171045 1.41873255 0.20471547 0.0380043  2.68819596
 1.22670391 2.9438314  0.49308482 2.35044118 1.54450402 2.78641764], Cost: 1.5344
Current parameters: [0.98435402 2.50408846 3.61413907 1.94823449 2.00633404 0.15208944
 0.29679727 3.02209508 0.26040949 1.77751638 1.73087743 2.61214673
 2.16562904 2.90969964 1.452708   0.23952993 0.06562486 2.70500833
 1.25225257 2.96386908 0.51951942 2.38630966 1.55764102 2.78654465], Cost: 0.3812
Current parameters: [1.01040635 2.50597228 3.61806662 1.92672082 2.49860211 0.13371753
 0.29578048 3.03059498 0.2566227  1.75439286 1.70724182 2.58868843
 2.1353418  2.89039038 1.42926892 0.21551203 0.04656991 2.69340976
 1.23462699 2.95004543 0.50128264 2.36156461 1.54857803 2.78645703], Cost: 1.4166
Current parameters: [0.98435402 2.75278448 3.61413907 1.94823449 2.00633404 0.17759023
 0.29679727 3.02209508 0.26040949 1.77751638 1.73087743 2.61214673
 2.16562904 2.90969964 1.452708   0.23952993 0.06562486 2.70500833
 1.25225257 2.96386908 0.51951942 2.38630966 1.55764102 2.78654465], Cost: 1.0818
Current parameters: [0.97227531 2.41392044 3.61881374 1.92262837 1.97871003 0.63327214
 0.29558706 3.03221188 0.25590236 1.74999417 1.70274571 2.58422606
 2.12958039 2.88671726 1.4248102  0.2109432  0.04294517 2.69120342
 1.23127415 2.94741581 0.49781354 2.35685747 1.54685401 2.78644036], Cost: 1.0731
Current parameters: [0.9841977  2.50436305 3.86409438 1.94505355 2.00410804 0.1494116
 0.29679727 3.02209508 0.26040949 1.77751638 1.73087743 2.61214673
 2.16562904 2.90969964 1.452708   0.23952993 0.06562486 2.70500833
 1.25225257 2.96386908 0.51951942 2.38630966 1.55764102 2.78654465], Cost: 0.7893
Current parameters: [ 0.93513044  2.37505899  3.52219561  1.74587948  1.82949716 -0.03498828
  0.29240406  3.05882018  0.24404809  1.67760773  1.62875621  2.51079162
  2.03476845  2.82627104  1.3514359   0.13575696 -0.01670492  2.65489497
  1.17609859  2.90414192  0.44072466  2.27939496  1.518483    2.78616607], Cost: 3.0888
Current parameters: [0.98435402 2.50408846 3.61413907 1.94823449 2.00633404 0.15208944
 0.04703862 3.02209508 0.26040949 1.78849884 1.73087743 2.61214673
 2.16562904 2.90969964 1.452708   0.23952993 0.06562486 2.70500833
 1.25225257 2.96386908 0.51951942 2.38630966 1.55764102 2.78654465], Cost: 0.1559
Current parameters: [0.97750146 2.47784576 3.598888   1.95077515 2.00185372 0.15125689
 0.06039635 3.02861885 0.25750309 2.28398316 1.71273683 2.59414222
 2.14238324 2.89487956 1.43471824 0.22109592 0.05099997 2.6961063
 1.23872474 2.95325927 0.50552249 2.36731755 1.55068507 2.7864774 ], Cost: 0.8543
Current parameters: [0.98435402 2.50408846 3.61413907 1.94823449 2.00633404 0.15208944
 0.04703862 2.9203573  0.03204701 1.78849884 1.73087743 2.61214673
 2.16562904 2.90969964 1.452708   0.23952993 0.06562486 2.70500833
 1.25225257 2.96386908 0.51951942 2.38630966 1.55764102 2.78654465], Cost: 0.4305
```



```
Current parameters: [0.98158559 2.4934864  3.60797764 1.94926092 2.00452399 0.15175309
 0.04356655 2.567723   0.4673767  1.78698864 1.72354862 2.6048729
 2.15623773 2.90371233 1.44544014 0.23208258 0.0597164  2.70141191
 1.24678732 2.95958271 0.51386466 2.37863684 1.55483081 2.78651748], Cost: 0.2347
Current parameters: [0.98435402 2.50408846 3.61413907 1.94823449 2.00633404 0.15208944
 0.04703862 3.02209508 0.26040949 1.78849884 1.90698729 2.43470571
 2.16562904 2.90969964 1.452708   0.23952993 0.06562486 2.70500833
 1.25225257 2.96386908 0.51951942 2.38630966 1.55764102 2.78654465], Cost: 0.1569
Current parameters: [0.97381519 2.46372877 3.59068384 1.95214188 1.99944358 0.15080903
 0.03382122 3.02564287 0.27611128 1.78274984 2.07926742 2.95799754
 2.12987841 2.88690726 1.42504084 0.21117954 0.04313267 2.69131754
 1.23144758 2.94755184 0.49799298 2.35710095 1.54694319 2.78644123], Cost: 1.3404
Current parameters: [0.98435402 2.50408846 3.61413907 1.94823449 2.00633404 0.15208944
 0.04703862 3.02209508 0.26040949 1.78849884 1.73087743 2.61214673
 2.30002407 2.69889648 1.452708   0.23952993 0.06562486 2.70500833
 1.25225257 2.96386908 0.51951942 2.38630966 1.55764102 2.78654465], Cost: 0.2395
Current parameters: [0.97218833 2.45749851 3.5870631  1.95274505 1.99837992 0.15061138
 0.03178088 3.02619054 0.27853514 1.78186238 1.72041841 2.6018521
 2.57585667 3.17781296 1.42076991 0.20680314 0.03966059 2.68920412
 1.22823595 2.94503297 0.49466998 2.35259206 1.54529179 2.78642526], Cost: 2.6914
Current parameters: [0.98435402 2.50408846 3.61413907 1.94823449 2.00633404 0.15208944
 0.04703862 3.02209508 0.26040949 1.78849884 1.73087743 2.61214673
 2.16562904 2.90969964 1.63162748 0.0649223  0.06562486 2.70500833
 1.25225257 2.96386908 0.51951942 2.38630966 1.55764102 2.78654465], Cost: 0.4722
Current parameters: [0.97317964 2.46129487 3.58926937 1.95237751 1.99902805 0.15073182
 0.03302414 3.02585682 0.27705818 1.78240315 1.72127065 2.60269095
 2.12273502 2.88839471 1.78164466 0.6041928  0.04177627 2.69049192
 1.23019293 2.94656782 0.49669483 2.35533952 1.54629806 2.78643499], Cost: 1.7632
Current parameters: [0.98435402 2.50408846 3.61413907 1.94823449 2.00633404 0.15208944
 0.04703862 3.02209508 0.26040949 1.78849884 1.73087743 2.61214673
 2.16562904 2.90969964 1.452708   0.23952993 0.19561081 2.49145821
 1.25225257 2.96386908 0.51951942 2.38630966 1.55764102 2.78654465], Cost: 0.1836
Current parameters: [0.97693678 2.47568324 3.59763125 1.95098451 2.00148452 0.15118829
 0.03773619 3.02459202 0.27146044 1.78445269 1.72450071 2.60587024
 2.13715716 2.89555801 1.43216993 0.236807   0.48873563 2.96386102
 1.23760998 2.95238498 0.50436908 2.36575252 1.55011187 2.78647186], Cost: 1.7868
Current parameters: [0.98435402 2.50408846 3.61413907 1.94823449 2.00633404 0.15208944
 0.04703862 3.02209508 0.26040949 1.78849884 1.73087743 2.61214673
 2.16562904 2.90969964 1.452708   0.23952993 0.06562486 2.70500833
 1.40653523 2.7671539  0.51951942 2.38630966 1.55764102 2.78654465], Cost: 0.1306
Current parameters: [0.97695598 2.47575681 3.597674   1.95097739 2.00149708 0.15119062
```



```
 0.03776028 3.02458555 0.27143182 1.78446317 1.72451723 2.6058865
 2.1372309  2.89559463 1.43222312 0.23681405 0.04636863 2.69459299
 1.79736752 3.07238056 0.50440832 2.36580576 1.55013137 2.78647205], Cost: 1.3407
Current parameters: [0.98435402 2.50408846 3.61413907 1.94823449 2.00633404 0.15208944
 0.04703862 3.02209508 0.26040949 1.78849884 1.73087743 2.61214673
 2.16562904 2.90969964 1.452708   0.23952993 0.06562486 2.70500833
 1.40653523 2.7671539  0.72076943 2.23799097 1.55764102 2.78654465], Cost: 0.1691
Current parameters: [0.97382001 2.46374722 3.59069457 1.95214009 1.99944673 0.15080962
 0.03382726 3.02564125 0.2761041  1.78275247 1.72182118 2.60323283
 2.12519313 2.88961562 1.42353972 0.23566281 0.03820605 2.69017801
 1.39198528 2.75388996 0.8099727  2.78415224 1.54694808 2.78644127], Cost: 1.9078
Current parameters: [0.98435402 2.50408846 3.61413907 1.94823449 2.00633404 0.15208944
 0.04703862 3.02209508 0.26040949 1.78849884 1.73087743 2.61214673
 2.16562904 2.90969964 1.452708   0.23952993 0.06562486 2.70500833
 1.40653523 2.7671539  0.51951942 2.38630966 1.56005789 2.53655633], Cost: 0.2024
Current parameters: [0.98135906 2.49261891 3.60747349 1.9493449  2.00437589 0.15172557
 0.04328245 3.0231033  0.26487168 1.78686507 1.72830262 2.60961239
 2.15413257 2.90398949 1.44441507 0.23843045 0.05782933 2.70079187
 1.40239848 2.76338278 0.51421835 2.38017828 2.0569533  2.79254494], Cost: 1.8627
Current parameters: [0.93142259 2.45556135 3.56500818 2.17205846 1.98755329 0.12060307
 0.09810014 3.02522559 0.25901482 1.7777207  1.72217245 2.60350705
 2.15447426 2.90258805 1.4440754  0.23068415 0.05860693 2.70073658
 1.40004373 2.76206265 0.51280283 2.37719607 1.55430312 2.78651238], Cost: 0.1819
Current parameters: [ 1.18844728  2.65003354  3.79715556  2.09802831  2.02490506  0.22376262
 -0.19504153  3.04240423  0.27981139  1.74928517  1.67572897  2.55753453
  2.04747781  2.84375368  1.36477097  0.19227684 -0.01196807  2.66084029
  1.35256953  2.72177797  0.45776876  2.30867069  1.4705544   2.79249734], Cost: 1.8754
Current parameters: [0.96624712 2.47218827 3.58190687 1.93828749 2.23254084 0.13076185
 0.05031493 3.05027458 0.28036165 1.76702892 1.69309172 2.61647947
 2.12958567 2.92193472 1.41437329 0.24276241 0.03209895 2.71936255
 1.38285127 2.78631469 0.51397405 2.37878528 1.55488518 2.78651801], Cost: 0.3500
Current parameters: [1.0218346  2.48840726 3.62412685 1.94247831 2.14985054 0.16052144
 0.17456871 2.87149313 0.15032222 1.85458365 1.89962255 2.52076422
 2.26513368 2.76308208 1.59730209 0.16073669 0.19263403 2.56972789
 1.4937066  2.59935233 0.48735244 2.34785849 1.49400264 2.79167033], Cost: 0.2659
Current parameters: [0.93152909 2.44115264 3.55311221 1.85666625 1.9313769  0.06207324
 0.08751575 3.02649336 0.25807438 1.75303679 1.7014654  2.56463102
 2.09928672 2.85651505 1.40766526 0.19564447 0.02706058 2.667756
 1.37634967 2.72540497 0.4770641  2.33170933 1.50896879 2.789396  ], Cost: 1.2708
Current parameters: [0.99952238 2.52976882 3.62793494 1.95399799 2.01069252 0.04698826
 0.0310813  3.0212884  0.26225203 1.79393079 1.76917295 2.65206374
```



```
 2.16912593 2.91353574 1.45493784 0.24375458 0.06732987 2.70763218
 1.40844665 2.77037477 0.5223622  2.39016695 1.55905377 2.78655831], Cost: 0.2566
Current parameters: [0.96454269 2.46487141 3.59139003 1.94611535 2.00151991 0.24882051
 0.04915438 3.03252667 0.27171624 1.77879796 1.8759296  2.7695201
 2.12923398 2.89959443 1.42188451 0.22991335 0.03861964 2.70029034
 1.38737599 2.76360583 0.50460785 2.36727679 1.53922145 2.78769289], Cost: 0.3808
Current parameters: [0.97982919 2.49959793 3.60965894 1.94866881 2.00587218 0.15194975
 0.0425843  2.9316875  0.31334953 1.84848608 1.71925907 2.61691194
 2.15143852 2.91436996 1.43765887 0.24067002 0.05247068 2.71054237
 1.39722197 2.77454925 0.51726885 2.38325591 1.55652257 2.78653384], Cost: 0.1035
Current parameters: [0.97781929 2.4925207  3.60562695 1.94991827 2.00550319 0.15509741
 0.04826965 3.05054181 0.26535557 2.0614653  1.71284315 2.6134498
 2.13915743 2.90986915 1.42776546 0.23755269 0.04368338 2.70788973
 1.39129573 2.77222134 0.51188575 2.37653704 1.54847872 2.78710286], Cost: 0.5297
Current parameters: [0.9808134  2.50056474 3.61061399 1.94844788 2.00624809 0.1518879
 0.04194885 2.93173048 0.31299833 1.84888023 1.71999472 2.61721474
 2.15045265 2.91338172 1.37964271 0.1807869  0.13211747 2.75866753
 1.39861272 2.77525454 0.51851415 2.38494563 1.55714144 2.78653982], Cost: 0.1204
Current parameters: [0.97425541 2.47196453 3.59518106 1.95313507 2.00601373 0.165227
 0.02000637 2.92801919 0.34018303 1.84357081 1.70871331 2.62380567
 2.10863118 2.90552269 1.54197232 0.38284868 0.17877576 2.80307052
 1.37471008 2.77124708 0.499379   2.36152104 1.52434022 2.78902877], Cost: 0.6813
Current parameters: [0.98134971 2.49822498 3.6125554  1.93119432 1.98694944 0.19320541
 0.03587958 2.9671208  0.30089163 1.82991369 1.693999   2.59612441
 2.2276217  2.96654483 1.44108991 0.24853834 0.05861848 2.7178433
 1.40467947 2.75435688 0.49569758 2.39364892 1.54655371 2.80996935], Cost: 0.2011
Current parameters: [0.97472414 2.45972928 3.58464184 1.99611027 2.05036865 0.08273536
 0.02894069 2.83546746 0.37173298 1.88250304 1.77466354 2.66364829
 2.27001448 2.99019466 1.4018186  0.22860538 0.02895919 2.69166344
 1.3559914  2.80069795 0.53765341 2.32498556 1.52131776 2.73764456], Cost: 0.5384
Current parameters: [0.98352686 2.49209043 3.60869934 1.95055951 2.00753386 0.15769634
 0.03817486 2.92408533 0.31487703 1.84747169 1.7257992  2.61334889
 2.15946104 2.91296928 1.43994723 0.24176869 0.05672159 2.70539726
 1.39321299 2.76244139 0.50811423 2.3711051  1.67843724 2.78853377], Cost: 0.3149
Current parameters: [0.98083528 2.50216764 3.61173519 1.95408385 1.988814   0.14844319
 0.01922842 2.94720763 0.32924978 1.84177777 1.70103025 2.62818177
 2.13662198 2.92963887 1.41699732 0.24933661 0.03560143 2.72467132
 1.38184497 2.79186165 0.51768092 2.37998537 1.56175546 2.78431228], Cost: 0.1461
Current parameters: [ 1.03428514  2.53525386  3.65727573  1.98775526  2.0221649   0.18150266
 -0.0048997   2.917345    0.3092245   1.84015159  1.72132481  2.59466013
  2.14080851  2.88987482  1.43266455  0.22346716  0.04690768  2.68783704
```

```
 1.39457226  2.74886296  0.5009642   2.36394751  1.53379704  2.78891287], Cost: 0.2951
Current parameters: [0.97970883 2.49955614 3.60950599 1.94903848 2.00629963 0.15088673
 0.04267616 2.93089563 0.31378887 1.84896002 1.71980359 2.61756377
 2.15126609 2.91435379 1.43745744 0.24057682 0.05231987 2.71054312
 1.35611436 2.74315331 0.53851875 2.41110253 1.55643511 2.78594749], Cost: 0.2248
Current parameters: [0.98590248 2.48561338 3.60775093 1.95626419 2.00460001 0.16530825
 0.0292154  2.92209846 0.32035306 1.84243327 1.7221183  2.60956391
 2.15038265 2.90860608 1.43171709 0.24027421 0.04811219 2.70053734
 1.46565963 2.81836102 0.56933308 2.45553116 1.55526668 2.78867047], Cost: 0.1894
Current parameters: [1.03003853 2.50011513 3.58246403 1.94884147 2.00241864 0.15276884
 0.06591029 2.93362894 0.31669251 1.84725906 1.71630094 2.61939149
 2.14951349 2.9177011  1.43500855 0.24345762 0.05057971 2.71389113
 1.39506972 2.77741118 0.51711673 2.38349374 1.55658533 2.78679801], Cost: 0.1088
Current parameters: [0.94396295 2.60690947 3.57104066 1.95438618 2.01096907 0.16663572
 0.06826456 2.92527717 0.3228513  1.84651816 1.72110495 2.61984603
 2.15236066 2.91777969 1.43239934 0.24512558 0.0504035  2.71080424
 1.40559055 2.78486477 0.52003469 2.38375024 1.55676487 2.78524532], Cost: 0.2117
Current parameters: [0.97963265 2.49944217 3.62678085 1.94878655 2.00606342 0.15083561
 0.05994534 2.95339107 0.36402977 1.84811231 1.72498404 2.6121132
 2.15516408 2.90792309 1.44278811 0.23527508 0.05613034 2.70406138
 1.4013873  2.76901047 0.51756325 2.38279563 1.55640111 2.78602257], Cost: 0.1052
Current parameters: [0.98179077 2.50237866 3.68885151 1.95138995 2.00615507 0.15648538
 0.11828233 2.91158295 0.28420596 1.84346117 1.70669804 2.63135086
 2.14302083 2.93330365 1.42037282 0.25791228 0.04160787 2.7268139
 1.39421295 2.7972606  0.51895336 2.38482661 1.55703464 2.78667515], Cost: 0.2521
Current parameters: [0.97943178 2.49922374 3.60914904 1.94939365 2.00661238 0.14996323
 0.04261544 2.93029731 0.31443266 1.84931864 1.73151222 2.60664119
 2.12086527 2.96103412 1.44241384 0.23519641 0.06030344 2.69667113
 1.40613702 2.76269459 0.51789896 2.38227077 1.55626262 2.78543958], Cost: 0.1180
Current parameters: [0.9816016  2.50178618 3.61144213 1.95020316 2.00562039 0.15778132
 0.04150362 2.92914462 0.31297838 1.8464449  1.69656388 2.63846385
 2.14777285 2.92536059 1.51816388 0.16333715 0.03613076 2.73756136
 1.38467364 2.80333434 0.51760878 2.38544323 1.55716286 2.78792661], Cost: 0.1037
Current parameters: [0.98349405 2.502963   3.61313435 1.93367113 2.00671201 0.15504441
 0.03923298 2.93220955 0.31287458 1.84875495 1.70192168 2.63443313
 2.15257901 2.91486299 1.43860255 0.24131252 0.07878915 2.66886074
 1.38291013 2.7939253  0.51737825 2.38436599 1.55687643 2.7871078 ], Cost: 0.1151
Current parameters: [1.00331393 2.52125655 3.630159   1.84375915 2.02231152 0.15101542
 0.01679829 2.91577244 0.32429548 1.86108365 1.7437355  2.62038797
 2.15083085 2.91737217 1.4361066  0.24335548 0.03675408 2.73474853
 1.403535   2.77358756 0.52682352 2.37924762 1.55595868 2.77660155], Cost: 0.0857
```



```
Current parameters: [1.0038716  2.5220513  3.63045326 1.84477714 2.02654272 0.14652572
 0.01527272 2.91079407 0.32809961 1.86489436 1.71682089 2.65660632
 2.1496953  2.91839789 1.43469986 0.24398029 0.03550113 2.73572208
 1.42936427 2.74086387 0.52997412 2.3771504  1.55613301 2.77282259], Cost: 0.0800
Current parameters: [0.9799136  2.49396156 3.6012955  1.80192245 1.98585985 0.1209715
 0.03486091 2.90010972 0.3257962  1.83267645 1.69341748 2.62702809
 2.12632549 2.88602778 1.42059168 0.20995558 0.0124124  2.71244356
 1.45043565 2.7320944  0.50724525 2.35436728 1.53005869 2.77924943], Cost: 0.3786
Current parameters: [1.00846699 2.53006491 3.63469189 1.84588494 2.03246441 0.1165141
 0.01161125 2.90150541 0.33223407 1.87244219 1.6845853  2.62515098
 2.15327882 2.92099763 1.44711798 0.25661612 0.04891821 2.74429225
 1.4310138  2.74269032 0.53622554 2.3767223  1.55803632 2.76755438], Cost: 0.0576
Current parameters: [1.00249858e+00 2.51026468e+00 3.62209192e+00 1.84606927e+00
 2.02973498e+00 1.40487289e-01 5.17051034e-04 2.89321244e+00
 3.47497569e-01 1.86250773e+00 1.70073072e+00 2.64681596e+00
 2.12847235e+00 2.90858427e+00 1.49884376e+00 3.17720514e-01
 1.04859874e-01 2.78699414e+00 1.43728120e+00 2.75239257e+00
 5.23305086e-01 2.36180051e+00 1.53478186e+00 2.77026462e+00], Cost: 0.4520
Current parameters: [1.00884667 2.53095651 3.63448822 1.84612556 2.04022041 0.10620513
 0.00985432 2.89409702 0.33788987 1.8777143  1.68578551 2.62937338
 2.15112309 2.92217475 1.44469316 0.25721181 0.04677584 2.74550686
 1.42956302 2.74428669 0.54069306 2.37316319 1.55739735 2.82710048], Cost: 0.1056
Current parameters: [0.99751515 2.51045391 3.61693338 1.8393905  2.13886758 0.12937066
 0.01751347 2.93070819 0.33087111 1.84639615 1.66045717 2.61612874
 2.13900461 2.92359373 1.43219637 0.25454875 0.03398608 2.74837705
 1.42621849 2.75250571 0.52273355 2.38045568 1.55586291 2.76066751], Cost: 0.0435
Current parameters: [0.9962441  2.5077633  3.61571621 1.8388953  2.13862657 0.14139406
 0.01984254 2.94109116 0.32465736 1.8389423  1.65760176 2.61110508
 2.14091543 2.92300592 1.43352259 0.25385337 0.03522878 2.74732588
 1.42688664 2.75137342 0.57017722 2.34511968 1.55563962 2.7604317 ], Cost: 0.0354
Current parameters: [0.99400021 2.48892361 3.60220974 1.85957627 2.16472126 0.10586683
 0.01253456 2.89544002 0.351077   1.85724425 1.6825887  2.62971831
 2.20320574 2.95838203 1.41662403 0.24050111 0.01974429 2.73623651
 1.42173667 2.7722891  0.57727253 2.31701995 1.53513497 2.73063089], Cost: 0.1585
Current parameters: [0.9898294  2.49748433 3.60951852 1.83736187 2.13707032 0.17472941
 0.02409873 2.92635523 0.33529463 1.88012764 1.64119399 2.59966917
 2.13397143 2.92275993 1.42697647 0.25253225 0.02956785 2.74794083
 1.42269071 2.75242229 0.56796913 2.34218966 1.55393621 2.76048825], Cost: 0.0678
Current parameters: [1.00202839 2.51533391 3.61843478 1.8396559  2.14587736 0.10406576
 0.01821688 3.02040797 0.28587306 1.91293562 1.67072983 2.61793112
 2.14210994 2.91614539 1.43612181 0.2457608  0.03106704 2.74225791
```



```
 1.44348276 2.75881243 0.57012405 2.34254926 1.55009407 2.75291949], Cost: 0.0540
Current parameters: [0.98321484 2.49561658 3.61093239 1.84565162 2.14276466 0.13918424
 0.0143431  2.93126293 0.31013166 1.84151662 1.6663352  2.60581452
 2.15086454 2.9055524  1.43787192 0.26047988 0.03104412 2.74973424
 1.43694505 2.74644691 0.5595256  2.33100386 1.60169692 2.7611404 ], Cost: 0.0426
Current parameters: [1.04172556 2.53193738 3.62611038 1.81953506 2.13900549 0.14724303
 0.0265313  2.96516311 0.36146651 1.83434697 1.64433707 2.61505569
 2.13239647 2.96531247 1.427333   0.22315865 0.04726619 2.72669423
 1.41849597 2.75966135 0.58448921 2.36148153 1.63389737 2.74976959], Cost: 0.0723
Current parameters: [ 1.02224613  2.52489454  3.63852226  1.85831133  2.14880575  0.15322289
 -0.00563409  2.93612576  0.31948796  1.83589208  1.65877681  2.59846159
  2.13822985  2.908429    1.431273    0.24285688  0.02953709  2.73580611
  1.43332298  2.74262799  0.5607458   2.33324411  1.54769206  2.75863866], Cost: 0.0725
Current parameters: [1.00439554 2.48663868 3.63472822 1.8377265  2.13753655 0.13910675
 0.02553036 2.93985985 0.31936894 1.83858988 1.65529712 2.61274062
 2.13915409 2.92564633 1.43215681 0.25503174 0.03448391 2.74927606
 1.42469084 2.75279534 0.5699365  2.34528662 1.55569605 2.76067746], Cost: 0.0306
Current parameters: [0.9925825  2.51837167 3.65703904 1.84237767 2.14241788 0.14178974
 0.06264001 2.93001051 0.30367631 1.83758016 1.65181056 2.6173889
 2.13904519 2.92885991 1.42416707 0.26196343 0.02529861 2.75654617
 1.42932464 2.76451805 0.5687158  2.34171369 1.55882741 2.75725579], Cost: 0.0270
Current parameters: [0.99214658 2.52029185 3.65785829 1.8376732  2.13922678 0.14759095
 0.06382536 2.93811391 0.30026074 1.83507903 1.6467742  2.61449695
 2.15365233 2.939615   1.42611364 0.26334988 0.02739406 2.75911858
 1.42185727 2.75497563 0.55574058 2.33140294 1.55708547 2.76138413], Cost: 0.0302
Current parameters: [0.99022757 2.51385055 3.65922605 1.84291826 2.13869554 0.15857322
 0.05577034 2.93606235 0.29386329 1.83020368 1.6458511  2.60558615
 2.16756242 2.93748061 1.42277445 0.26534056 0.02061209 2.75691958
 1.46267518 2.77480699 0.58030479 2.37088224 1.55704744 2.76365176], Cost: 0.0431
Current parameters: [0.99493668 2.51864441 3.65682687 1.84096332 2.14169362 0.14173055
 0.06309936 2.93144113 0.30690535 1.83728976 1.65076994 2.61828437
 2.13741222 2.93180228 1.43866776 0.27588598 0.00626371 2.74340631
 1.42774488 2.76531746 0.56876097 2.3413817  1.55890356 2.75702858], Cost: 0.0320
Current parameters: [0.98622083 2.52142102 3.66110466 1.84638971 2.14863088 0.13887484
 0.0653993  2.92773911 0.29018294 1.83954058 1.65391813 2.61323107
 2.14820738 2.9210479  1.42377345 0.26036758 0.01900134 2.75837119
 1.39696849 2.73228719 0.58974591 2.3700773  1.56257039 2.75287852], Cost: 0.0751
Current parameters: [0.98910824 2.51841551 3.65766906 1.84569258 2.14145378 0.1429911
 0.05931277 2.92881219 0.3004314  1.83662282 1.64535784 2.62262368
 2.13932389 2.92924804 1.44203931 0.24426084 0.01851681 2.76714411
 1.42579588 2.77182383 0.56859604 2.34216776 1.55921391 2.75770974], Cost: 0.0137
```



```
Current parameters: [ 9.79517215e-01  2.52060367e+00  3.66130409e+00  1.85770515e+00
  2.12666518e+00  1.38370790e-01  3.34018468e-02  2.94087038e+00
  3.03003190e-01  1.83046032e+00  1.62950665e+00  2.62941053e+00
  2.13240619e+00  2.93416351e+00  1.41400734e+00  2.58433557e-01
 -2.32291216e-03  2.78721730e+00  1.41349173e+00  2.78412974e+00
  5.69333602e-01  2.34034340e+00  1.56558999e+00  2.75455270e+00], Cost: 0.0422
Current parameters: [0.98968628 2.51720432 3.65596345 1.85079506 2.14138366 0.14115206
 0.06194183 2.92893365 0.30311574 1.83683125 1.65623071 2.61256892
 2.12959636 2.94322639 1.44320282 0.24226254 0.01052293 2.77933705
 1.43426154 2.76050266 0.56884252 2.34138522 1.55898827 2.75704623], Cost: 0.0137
Current parameters: [0.96538574 2.51754349 3.66152785 1.85188875 2.14224181 0.1428783
 0.04289833 2.91980636 0.2749535  1.83662199 1.64196215 2.62356097
 2.12016026 2.96002762 1.4442072  0.24120955 0.03426398 2.74080018
 1.42419995 2.77334938 0.56827695 2.34295847 1.55992742 2.75785502], Cost: 0.0142
Current parameters: [1.00629074 2.51989558 3.64396999 1.83959977 2.14125619 0.14360772
 0.05867298 2.92042726 0.28327716 1.8371099  1.64372612 2.62538718
 2.13730005 2.93286366 1.43921131 0.24729657 0.01564917 2.77201148
 1.42393994 2.77472416 0.56899475 2.34218024 1.55924593 2.75740481], Cost: 0.0109
Current parameters: [0.98910824 2.51841551 3.65766906 1.84569258 2.14145378 0.1429911
 0.05931277 2.92881219 0.3004314  1.83662282 1.64535784 2.62262368
 2.13932389 2.92924804 1.44203931 0.24426084 0.01851681 2.76714411
 1.42579588 2.77182383 0.56859604 2.34216776 1.55921391 2.75770974], Cost: 0.0137

   Return from subroutine COBYLA because the MAXFUN limit has been reached.

   NFVALS =  100   F = 1.370793E-02    MAXCV = 0.000000E+00
   X = 9.891082E-01   2.518416E+00   3.657669E+00   1.845693E+00   2.141454E+00
       1.429911E-01   5.931277E-02   2.928812E+00   3.004314E-01   1.836623E+00
       1.645358E+00   2.622624E+00   2.139324E+00   2.929248E+00   1.442039E+00
       2.442608E-01   1.851681E-02   2.767144E+00   1.425796E+00   2.771824E+00
       5.685960E-01   2.342168E+00   1.559214E+00   2.757710E+00
```

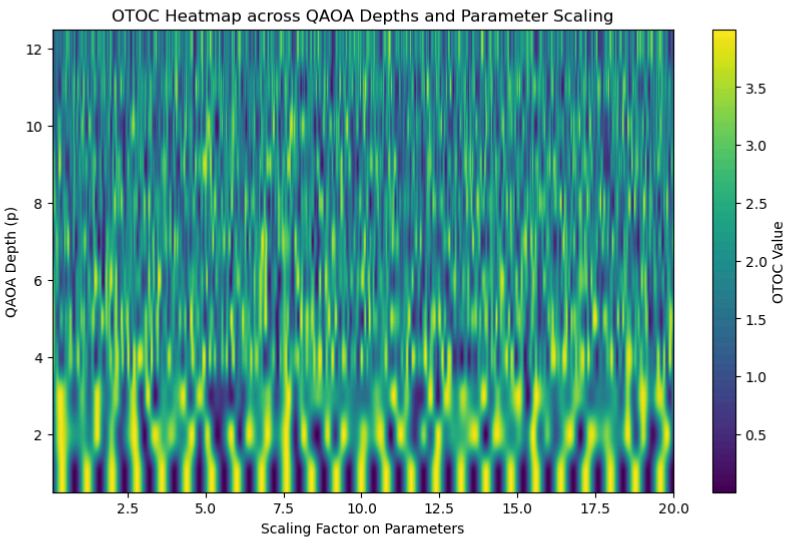


```
Run 5/30 for heatmap generation...
Simulating for QAOA depth p = 1
Current parameters: [1.83723767 2.91690796], Cost: 2.3975
Current parameters: [0.83723767 3.91690796], Cost: 2.0401
Current parameters: [0.0164753  2.34563819], Cost: 2.0014
Current parameters: [0.42685648 2.63127308], Cost: 1.3282
Current parameters: [1.01516327 2.74128768], Cost: 1.1042
Current parameters: [1.23328697 2.61913184], Cost: 1.4574
Current parameters: [0.85178955 2.55205495], Cost: 1.3005
Current parameters: [1.13561327 2.77470619], Cost: 1.2395
Current parameters: [0.98637333 2.68581346], Cost: 1.1088
Current parameters: [0.96114636 2.7727269 ], Cost: 1.0654
Current parameters: [0.90955057 2.80799904], Cost: 1.0576
Current parameters: [0.93369963 2.86564515], Cost: 1.1461
Current parameters: [0.87005618 2.75955895], Cost: 1.0152
Current parameters: [0.82597326 2.7152538 ], Cost: 1.0123
Current parameters: [0.80264907 2.73605151], Cost: 1.0019
Current parameters: [0.77764955 2.75480215], Cost: 1.0004
Current parameters: [0.75334288 2.73516168], Cost: 1.0036
Current parameters: [0.78536797 2.78508397], Cost: 1.0105
Current parameters: [0.78150876 2.76994306], Cost: 1.0036
Current parameters: [0.77181818 2.74960308], Cost: 1.0004
Current parameters: [0.77441772 2.7466874 ], Cost: 1.0003
Current parameters: [0.77874049 2.74017981], Cost: 1.0007
Current parameters: [0.77058704 2.74745203], Cost: 1.0005
Current parameters: [0.77647714 2.75000667], Cost: 1.0002
Current parameters: [0.7803654  2.75038112], Cost: 1.0001
Current parameters: [0.78346711 2.7527556 ], Cost: 1.0001
Current parameters: [0.78121599 2.74862294], Cost: 1.0000
Current parameters: [0.78089969 2.7466956 ], Cost: 1.0001
Current parameters: [0.7831345  2.74898905], Cost: 1.0000
Current parameters: [0.7847084  2.74783253], Cost: 1.0000
Current parameters: [0.785295   2.74861328], Cost: 1.0000
Current parameters: [0.78588851 2.7493888 ], Cost: 1.0000
Current parameters: [0.78568538 2.74831998], Cost: 1.0000
Current parameters: [0.78550057 2.74956796], Cost: 1.0000
Current parameters: [0.78539779 2.74909062], Cost: 1.0000
Current parameters: [0.78516968 2.74917765], Cost: 1.0000
Current parameters: [0.78563974 2.74912319], Cost: 1.0000
Current parameters: [0.7853943  2.74899068], Cost: 1.0000
Current parameters: [0.78536828 2.74889412], Cost: 1.0000
```



```
Current parameters: [0.78526974 2.7488771 ], Cost: 1.0000
Current parameters: [0.78541652 2.74880653], Cost: 1.0000
Current parameters: [0.78541652 2.74880653], Cost: 1.0000

   Normal return from subroutine COBYLA

   NFVALS =   42   F = 1.000000E+00    MAXCV = 0.000000E+00
   X = 7.854165E-01   2.748807E+00
Simulating for QAOA depth p = 2
Current parameters: [3.57863245 1.88156006 1.16898153 0.11886797], Cost: 1.0429
Current parameters: [3.57863245 2.88156006 1.16898153 0.11886797], Cost: 1.1235
Current parameters: [3.57863245 1.88156006 2.16898153 0.11886797], Cost: 2.4641
Current parameters: [3.57863245 1.88156006 1.16898153 1.11886797], Cost: 1.8970
Current parameters: [ 4.28626942  1.84726911  0.56405657 -0.24464248], Cost: 2.3813
Current parameters: [ 3.93245094  1.86441459  0.86651905 -0.06288726], Cost: 1.6227
Current parameters: [3.35534935 1.87610281 1.07271043 0.06101691], Cost: 1.5221
Current parameters: [3.57863245 1.75676041 1.17605596 0.11886797], Cost: 1.1091
Current parameters: [3.63790738 1.93045443 1.3997735  0.06115408], Cost: 1.2619
Current parameters: [3.62214633 1.90770357 1.16640776 0.36364631], Cost: 0.9142
Current parameters: [3.83328753 1.93851079 1.03620798 0.35936544], Cost: 1.3123
Current parameters: [3.39729102 1.96191124 1.13477301 0.45309385], Cost: 1.0697
Current parameters: [3.6365413  1.93070644 1.28780877 0.37591202], Cost: 0.7436
Current parameters: [3.63383985 1.82398486 1.34656175 0.40377407], Cost: 0.7172
Current parameters: [3.69492201 1.81708148 1.34165485 0.39360131], Cost: 0.6945
Current parameters: [3.74472604 1.85926799 1.4432829  0.4257975 ], Cost: 0.7903
Current parameters: [3.70289416 1.81922393 1.31863683 0.45111892], Cost: 0.7619
Current parameters: [3.69987836 1.75574978 1.31881835 0.28721826], Cost: 0.7101
Current parameters: [3.70892171 1.79029507 1.29707701 0.42531192], Cost: 0.7441
Current parameters: [3.69974394 1.86796815 1.31238084 0.37270684], Cost: 0.7119
Current parameters: [3.70698863 1.82133633 1.36988196 0.38959061], Cost: 0.6887
Current parameters: [3.70520232 1.80694994 1.37125181 0.36194053], Cost: 0.6760
Current parameters: [3.72088186 1.78450186 1.37691577 0.34798639], Cost: 0.6695
Current parameters: [3.7042151  1.77193716 1.39428099 0.33251515], Cost: 0.6840
Current parameters: [3.72733891 1.79443104 1.38571129 0.34283882], Cost: 0.6747
Current parameters: [3.72815472 1.7834714  1.34960084 0.33470104], Cost: 0.6681
Current parameters: [3.73667592 1.77648175 1.34602143 0.34518257], Cost: 0.6651
Current parameters: [3.73415859 1.76150854 1.3429017  0.34715081], Cost: 0.6643
Current parameters: [3.72842593 1.76433    1.33428715 0.35851331], Cost: 0.6700
Current parameters: [3.73649287 1.76195266 1.33649607 0.34336177], Cost: 0.6666
Current parameters: [3.74291075 1.75631069 1.35470106 0.34829004], Cost: 0.6588
Current parameters: [3.74250157 1.75113308 1.36866507 0.34358133], Cost: 0.6584
```



```
Current parameters: [3.74088139 1.7439947  1.36655498 0.34531373], Cost: 0.6574
Current parameters: [3.7481931  1.74070878 1.37056188 0.35811322], Cost: 0.6540
Current parameters: [3.76204971 1.734471   1.3682972  0.3552678 ], Cost: 0.6501
Current parameters: [3.77402699 1.72568378 1.36843393 0.36011046], Cost: 0.6461
Current parameters: [3.78779277 1.72516883 1.37565939 0.36158407], Cost: 0.6450
Current parameters: [3.79527629 1.72750087 1.36385771 0.36817341], Cost: 0.6454
Current parameters: [3.78992471 1.72420657 1.37294496 0.35464174], Cost: 0.6445
Current parameters: [3.79252325 1.70880349 1.37294552 0.35481204], Cost: 0.6402
Current parameters: [3.79612473 1.69362627 1.37352905 0.35402137], Cost: 0.6366
Current parameters: [3.80161802 1.68084099 1.37242788 0.3470008 ], Cost: 0.6348
Current parameters: [3.80419759 1.67314591 1.38419592 0.3520311 ], Cost: 0.6341
Current parameters: [3.79309393 1.66299307 1.38146896 0.35461962], Cost: 0.6345
Current parameters: [3.80779006 1.67191113 1.38097693 0.35768972], Cost: 0.6308
Current parameters: [3.81764019 1.66687773 1.37427142 0.36645423], Cost: 0.6259
Current parameters: [3.81921302 1.67076001 1.37195324 0.38132772], Cost: 0.6269
Current parameters: [3.81601184 1.67036196 1.36770311 0.36469323], Cost: 0.6271
Current parameters: [3.82884619 1.65642884 1.37131991 0.36563225], Cost: 0.6218
Current parameters: [3.84347399 1.65761419 1.37539196 0.36214206], Cost: 0.6210
Current parameters: [3.8473708  1.64248882 1.37505673 0.36239948], Cost: 0.6165
Current parameters: [3.8521664  1.62825547 1.37084105 0.36151481], Cost: 0.6126
Current parameters: [3.85643942 1.6145604  1.37697521 0.36235094], Cost: 0.6092
Current parameters: [3.86461644 1.60138637 1.37588627 0.36075842], Cost: 0.6056
Current parameters: [3.87173855 1.59205915 1.38620105 0.36091545], Cost: 0.6044
Current parameters: [3.86771993 1.58111108 1.38358544 0.35085118], Cost: 0.6074
Current parameters: [3.86732533 1.58866605 1.3860966  0.36639584], Cost: 0.6033
Current parameters: [3.87605548 1.58502279 1.38022987 0.37736093], Cost: 0.5971
Current parameters: [3.88396898 1.58205314 1.37657843 0.38998493], Cost: 0.5936
Current parameters: [3.87552226 1.57876151 1.38169356 0.401638  ], Cost: 0.5960
Current parameters: [3.8863404  1.57602965 1.38058968 0.38824166], Cost: 0.5921
Current parameters: [3.88676678 1.56505122 1.36956038 0.38690473], Cost: 0.5897
Current parameters: [3.90071019 1.55840365 1.36733762 0.38767376], Cost: 0.5854
Current parameters: [3.91294929 1.54955855 1.36523544 0.38425471], Cost: 0.5818
Current parameters: [3.91417374 1.53797384 1.35975195 0.37540218], Cost: 0.5826
Current parameters: [3.91108331 1.54455145 1.36972114 0.3877705 ], Cost: 0.5798
Current parameters: [3.91911291 1.53824479 1.37037194 0.39958019], Cost: 0.5758
Current parameters: [3.92829977 1.53431132 1.38221344 0.40159233], Cost: 0.5736
Current parameters: [3.92917198 1.5263318  1.38633025 0.41435004], Cost: 0.5731
Current parameters: [3.94061989 1.53469428 1.3842514  0.42058167], Cost: 0.5747
Current parameters: [3.93302874 1.52143387 1.38204131 0.41240688], Cost: 0.5696
Current parameters: [3.94021394 1.5092157  1.37925895 0.40644968], Cost: 0.5641
Current parameters: [3.94136295 1.49951701 1.36710364 0.40544749], Cost: 0.5617
```

```
Current parameters: [3.95666889 1.50012487 1.36420075 0.40648325], Cost: 0.5589
Current parameters: [3.95780867 1.50551085 1.35979653 0.39253924], Cost: 0.5633
Current parameters: [3.95796898 1.49490351 1.36876617 0.40313074], Cost: 0.5567
Current parameters: [3.96488825 1.48238231 1.3722955  0.40832955], Cost: 0.5513
Current parameters: [3.97251896 1.47905692 1.38451328 0.41338726], Cost: 0.5504
Current parameters: [3.97870992 1.46558405 1.38090329 0.41003136], Cost: 0.5447
Current parameters: [3.97538912 1.450371   1.38073127 0.41131447], Cost: 0.5423
Current parameters: [3.98550192 1.44453616 1.37232911 0.41741613], Cost: 0.5364
Current parameters: [3.99701582 1.43627575 1.36574898 0.41722054], Cost: 0.5316
Current parameters: [4.00606668 1.4295142  1.36342569 0.42776124], Cost: 0.5267
Current parameters: [4.01060038 1.42332637 1.35164184 0.43457577], Cost: 0.5266
Current parameters: [4.01439882 1.42992263 1.34998969 0.43518137], Cost: 0.5285
Current parameters: [4.01890129 1.41305897 1.35999523 0.43476795], Cost: 0.5188
Current parameters: [4.02458767 1.40318996 1.36964463 0.43938286], Cost: 0.5139
Current parameters: [4.03211374 1.39374887 1.37157834 0.4296553 ], Cost: 0.5102
Current parameters: [4.02620623 1.37933477 1.37081698 0.43060187], Cost: 0.5093
Current parameters: [4.03048031 1.37809507 1.36538942 0.43403292], Cost: 0.5067
Current parameters: [4.04366951 1.37108217 1.36453061 0.43853486], Cost: 0.4999
Current parameters: [4.05677737 1.36392498 1.36080041 0.4412153 ], Cost: 0.4937
Current parameters: [4.06820704 1.35499459 1.36402863 0.44604542], Cost: 0.4872
Current parameters: [4.08000733 1.34561588 1.36466879 0.45011036], Cost: 0.4808
Current parameters: [4.09264894 1.33731709 1.36229639 0.45324597], Cost: 0.4743
Current parameters: [4.10501591 1.32852067 1.36098899 0.45672626], Cost: 0.4677
Current parameters: [4.11728722 1.31959121 1.36006304 0.46032667], Cost: 0.4612
Current parameters: [4.12980703 1.31367638 1.35293005 0.46156365], Cost: 0.4561
Current parameters: [4.14011893 1.30238047 1.35604787 0.4608657 ], Cost: 0.4496
Current parameters: [4.14970868 1.2909206  1.35758477 0.46516529], Cost: 0.4424
Current parameters: [4.14011893 1.30238047 1.35604787 0.4608657 ], Cost: 0.4496

   Return from subroutine COBYLA because the MAXFUN limit has been reached.

   NFVALS =  100   F = 4.496087E-01    MAXCV = 0.000000E+00
   X = 4.140119E+00   1.302380E+00   1.356048E+00   4.608657E-01
Simulating for QAOA depth p = 3
Current parameters: [1.40117228 0.84147685 2.18182688 0.9044362  2.14935012 0.26123004], Cost: 2.9962
Current parameters: [0.40117228 1.84147685 2.18182688 0.9044362  2.14935012 0.26123004], Cost: 1.1779
Current parameters: [0.40117228 1.84147685 3.18182688 0.9044362  2.14935012 0.26123004], Cost: 1.8391
Current parameters: [0.40117228 1.84147685 2.18182688 1.9044362  2.14935012 0.26123004], Cost: 1.1861
Current parameters: [0.40117228 1.84147685 2.18182688 0.9044362  3.14935012 0.26123004], Cost: 2.6258
Current parameters: [0.40117228 1.84147685 2.18182688 0.9044362  2.14935012 1.26123004], Cost: 1.9920
Current parameters: [ 0.02700259  2.29179183  1.8819924   0.90071938  1.49282294 -0.10791898], Cost: 1.9296
```



```
Current parameters: [0.88152034 1.90887699 2.13694966 0.90387989 2.05108553 0.20597829], Cost: 1.9131
Current parameters: [0.22118397 1.92572911 2.12572897 0.9037408  2.02651633 0.19216364], Cost: 1.1278
Current parameters: [0.22118397 2.02881107 2.12572897 0.9037408  2.09722072 0.19216364], Cost: 0.8481
Current parameters: [0.12697293 2.09196686 2.07556064 0.9031189  2.30531524 0.13039759], Cost: 1.1989
Current parameters: [0.22118397 2.02881107 2.02870197 0.9037408  2.09722072 0.27097202], Cost: 0.6476
Current parameters: [0.1792908  2.14019061 2.11982237 0.90346426 2.0683155  0.46896456], Cost: 0.1166
Current parameters: [0.29905346 2.32818455 2.06948313 0.90306052 1.98910401 0.53226165], Cost: 0.1730
Current parameters: [0.17943275 2.13996673 2.11983954 0.77846497 2.06864189 0.46898569], Cost: 0.1596
Current parameters: [0.05081834 2.21317057 2.09738627 0.92560954 2.21481505 0.60391953], Cost: 0.4365
Current parameters: [0.13031197 2.10860132 2.11673489 0.90912827 1.96540426 0.5089127 ], Cost: 0.2947
Current parameters: [0.21170756 2.10645081 2.1011739  0.9035895  2.07770426 0.50475702], Cost: 0.1475
Current parameters: [0.221986   2.14121481 2.23663133 0.90982004 2.07792028 0.47387278], Cost: 0.1787
Current parameters: [0.1995436  2.13830986 2.1005905  0.91170469 2.08052923 0.41506102], Cost: 0.1707
Current parameters: [0.1619757  2.11878467 2.12549323 0.90351617 2.08106321 0.46407886], Cost: 0.1140
Current parameters: [0.13962775 2.1525541  2.11884784 0.90992165 2.12238983 0.48583579], Cost: 0.0776
Current parameters: [0.0903292  2.16928933 2.11324409 0.92476494 2.10413475 0.51055142], Cost: 0.0443
Current parameters: [0.0755053  2.18926885 2.1641383  0.93903073 2.11122298 0.53160717], Cost: 0.0847
Current parameters: [0.08607744 2.17057639 2.11694478 0.89510977 2.101936   0.51822408], Cost: 0.0450
Current parameters: [0.05764515 2.18800461 2.06782501 0.92037225 2.11359258 0.49277215], Cost: 0.0199
Current parameters: [0.05666657 2.17047575 2.02929073 0.92649285 2.12298833 0.5373524 ], Cost: 0.0621
Current parameters: [0.04199657 2.16438346 2.07392251 0.91997778 2.12338126 0.48640251], Cost: 0.0209
Current parameters: [0.042059   2.18264404 2.05993496 0.91915991 2.06016121 0.46601487], Cost: 0.0590
Current parameters: [0.03063511 2.22789009 2.09207295 0.92601735 2.14404036 0.48653048], Cost: 0.0341
Current parameters: [0.07421432 2.18687093 2.06060207 0.91134733 2.12801182 0.47381944], Cost: 0.0251
Current parameters: [0.05976757 2.18746778 2.06634632 0.93526468 2.11477938 0.48903473], Cost: 0.0198
Current parameters: [0.04594559 2.2074349  2.07862918 0.93751467 2.12973616 0.48635278], Cost: 0.0210
Current parameters: [0.06750591 2.18495537 2.07922539 0.93628279 2.11453708 0.49234845], Cost: 0.0247
Current parameters: [0.05856308 2.18827745 2.06857601 0.93471594 2.10986314 0.48360457], Cost: 0.0174
Current parameters: [0.05002172 2.19084179 2.06291527 0.93538946 2.09951731 0.47859697], Cost: 0.0158
Current parameters: [0.05426185 2.1922781  2.05772771 0.93101057 2.10266553 0.46563155], Cost: 0.0155
Current parameters: [0.05522725 2.19993699 2.05839915 0.93140085 2.10301425 0.46647994], Cost: 0.0137
Current parameters: [0.0441121  2.20226516 2.06449401 0.93204744 2.1107039  0.4621814 ], Cost: 0.0100
Current parameters: [0.04530234 2.2019548  2.06397123 0.93950529 2.11129281 0.46036964], Cost: 0.0101
Current parameters: [0.04066516 2.21062219 2.07525979 0.93231824 2.10768432 0.4560719 ], Cost: 0.0113
Current parameters: [0.03636868 2.20982081 2.05646359 0.93291977 2.11818877 0.46459396], Cost: 0.0063
Current parameters: [0.04086775 2.21226671 2.05987765 0.93223931 2.13208413 0.46815996], Cost: 0.0106
Current parameters: [0.03840341 2.20991506 2.05382387 0.93070587 2.11972938 0.45806378], Cost: 0.0063
Current parameters: [0.02289707 2.20556943 2.05048372 0.93388335 2.11677858 0.46216373], Cost: 0.0048
Current parameters: [0.02244756 2.21392163 2.03995524 0.93460826 2.10918481 0.46443083], Cost: 0.0055
Current parameters: [0.02556878 2.20091804 2.0460555  0.93356225 2.1185323  0.46524171], Cost: 0.0054
Current parameters: [0.01408141 2.21142555 2.05056077 0.93441447 2.12826082 0.4621333 ], Cost: 0.0035
```



```
Current parameters: [0.00749244 2.21189857 2.05310142 0.92058503 2.12803613 0.46378964], Cost: 0.0034
Current parameters: [0.00549699 2.21176983 2.05577394 0.9228277  2.12646534 0.47030098], Cost: 0.0044
Current parameters: [1.10596492e-03 2.21196882e+00 2.05226467e+00 9.21269450e-01
 2.13263111e+00 4.50333556e-01], Cost: 0.0035
Current parameters: [0.01643147 2.22087361 2.05643913 0.91664509 2.13536749 0.4619811 ], Cost: 0.0058
Current parameters: [0.00582538 2.21625178 2.04746078 0.92052925 2.12531412 0.46406594], Cost: 0.0023
Current parameters: [0.00554403 2.2129961  2.04129843 0.91947882 2.12829357 0.4656156 ], Cost: 0.0021
Current parameters: [0.00508055 2.2106026  2.04096585 0.91951188 2.12535972 0.4648436 ], Cost: 0.0019
Current parameters: [0.00846193 2.21303886 2.03696181 0.91679137 2.12163172 0.46232675], Cost: 0.0019
Current parameters: [0.00668315 2.21315523 2.03763696 0.9133992  2.12161256 0.46267055], Cost: 0.0018
Current parameters: [1.23971970e-03 2.21426146e+00 2.03541755e+00 9.14827250e-01
 2.12155378e+00 4.57852672e-01], Cost: 0.0014
Current parameters: [-0.00286338  2.21603732  2.03281969  0.91607071  2.11745625  0.46184781], Cost: 0.0021
Current parameters: [1.10694076e-03 2.20990169e+00 2.03590828e+00 9.12781878e-01
 2.12245901e+00 4.51789162e-01], Cost: 0.0022
Current parameters: [1.34936711e-03 2.21591096e+00 2.03371696e+00 9.14305451e-01
 2.12460196e+00 4.58117716e-01], Cost: 0.0009
Current parameters: [1.50504499e-03 2.21920114e+00 2.03537322e+00 9.14329642e-01
 2.12551575e+00 4.57206390e-01], Cost: 0.0008
Current parameters: [-2.97423838e-04  2.21821687e+00  2.03715471e+00  9.15627887e-01
  2.12772796e+00  4.58341336e-01], Cost: 0.0008
Current parameters: [0.00224362 2.21932431 2.03497851 0.91608677 2.12551211 0.45710289], Cost: 0.0008
Current parameters: [1.94331403e-03 2.21987387e+00 2.03376681e+00 9.13602881e-01
 2.12847603e+00 4.55550451e-01], Cost: 0.0006
Current parameters: [5.32342157e-04 2.22219097e+00 2.03168454e+00 9.13645347e-01
 2.12887927e+00 4.57394027e-01], Cost: 0.0004
Current parameters: [0.0030571  2.22280163 2.03193505 0.91272361 2.1301766  0.45982632], Cost: 0.0007
Current parameters: [-2.02277299e-03  2.22405879e+00  2.03045177e+00  9.14140252e-01
  2.12890821e+00  4.55529642e-01], Cost: 0.0003
Current parameters: [-0.00352304  2.22204182  2.02746347  0.91421213  2.12891461  0.45559831], Cost: 0.0002
Current parameters: [-0.00290168  2.22241932  2.02686061  0.91317993  2.12760275  0.4552292 ], Cost: 0.0003
Current parameters: [-0.00630531  2.22175883  2.02887906  0.91215043  2.12854381  0.45662091], Cost: 0.0002
Current parameters: [-0.00666166  2.22164663  2.02917012  0.91333461  2.12714328  0.45709701], Cost: 0.0003
Current parameters: [-0.00792408  2.22388189  2.02746741  0.91210452  2.13101032  0.45639191], Cost: 0.0001
Current parameters: [-0.00910324  2.22295791  2.02808197  0.91209578  2.13210731  0.45301057], Cost: 0.0002
Current parameters: [-0.00743998  2.22291522  2.02686532  0.91102214  2.13401415  0.45827151], Cost: 0.0005
Current parameters: [-0.00740864  2.22512011  2.02886677  0.91206029  2.13119417  0.45624396], Cost: 0.0002
Current parameters: [-0.00856286  2.223844    2.02776771  0.9115026   2.13080505  0.45661457], Cost: 0.0001
Current parameters: [-0.00759874  2.2246442   2.0263899   0.91126326  2.1301248   0.45570282], Cost: 0.0001
Current parameters: [-0.00854275  2.2252675   2.02582895  0.91233889  2.12909697  0.4556194 ], Cost: 0.0001
Current parameters: [-0.00885634  2.22573073  2.02545638  0.91227247  2.12978839  0.45575895], Cost: 0.0000
```



```
Current parameters: [-0.00874959  2.22592687  2.02368125  0.91152312  2.12988674  0.45596526], Cost: 0.0000
Current parameters: [-0.0093822   2.22565168  2.02341918  0.91134417  2.13036893  0.4542316 ], Cost: 0.0000
Current parameters: [-0.00871695  2.22576417  2.02320569  0.911939    2.13058381  0.45400163], Cost: 0.0000
Current parameters: [-0.00933245  2.2273275   2.02358896  0.91062316  2.13047089  0.45356467], Cost: 0.0000
Current parameters: [-0.00981411  2.22762721  2.02332656  0.9111808   2.1299697   0.45353557], Cost: 0.0000
Current parameters: [-0.00981377  2.22711847  2.02331722  0.91018514  2.13227714  0.45345948], Cost: 0.0000
Current parameters: [-0.00856414  2.22658904  2.02344342  0.90955664  2.129724    0.45258355], Cost: 0.0001
Current parameters: [-0.00941085  2.22728408  2.0244922   0.91090624  2.13056863  0.45336439], Cost: 0.0000
Current parameters: [-0.00963363  2.22728367  2.02370522  0.91033756  2.13034465  0.45375113], Cost: 0.0000
Current parameters: [-0.00883649  2.227643    2.02362089  0.91064663  2.13055148  0.45433933], Cost: 0.0000
Current parameters: [-0.0084808   2.22754177  2.02351928  0.91039934  2.13039162  0.45427133], Cost: 0.0000
Current parameters: [-0.00883895  2.22785213  2.0236372   0.9105578   2.13057642  0.45425515], Cost: 0.0000
Current parameters: [-0.00881129  2.22781025  2.02342346  0.91044136  2.1309918   0.45431927], Cost: 0.0000
Current parameters: [-0.00886912  2.2278633   2.02321687  0.91047272  2.13089844  0.45435197], Cost: 0.0000
Current parameters: [-0.00875811  2.2280919   2.02290444  0.91071001  2.13089292  0.454493  ], Cost: 0.0000
Current parameters: [-0.00888453  2.22790648  2.02326452  0.9103747   2.13086822  0.45456342], Cost: 0.0000
Current parameters: [-0.00909018  2.22777026  2.02295558  0.91045588  2.13087628  0.45401754], Cost: 0.0000
Current parameters: [-0.00898778  2.22786449  2.02267552  0.9107635   2.13091752  0.45380708], Cost: 0.0000
Current parameters: [-0.00927955  2.22787718  2.02231646  0.91064225  2.13083506  0.45375513], Cost: 0.0000
Current parameters: [-0.00915629  2.22790711  2.02274756  0.91088007  2.1310158   0.45383794], Cost: 0.0000
Current parameters: [-0.00898778  2.22786449  2.02267552  0.9107635   2.13091752  0.45380708], Cost: 0.0000

   Return from subroutine COBYLA because the MAXFUN limit has been reached.

   NFVALS =  100   F = 3.855258E-07    MAXCV = 0.000000E+00
   X =-8.987776E-03   2.227864E+00   2.022676E+00   9.107635E-01   2.130918E+00
       4.538071E-01
Simulating for QAOA depth p = 4
Current parameters: [2.56411847 0.48548704 0.64610422 1.20389394 2.09560823 0.05090338
 2.29356845 0.00297568], Cost: 2.0191
Current parameters: [2.56411847 1.48548704 0.64610422 1.20389394 2.09560823 0.05090338
 2.29356845 0.00297568], Cost: 1.2486
Current parameters: [2.56411847 1.48548704 1.64610422 1.20389394 2.09560823 0.05090338
 2.29356845 0.00297568], Cost: 2.7633
Current parameters: [2.56411847 1.48548704 0.64610422 2.20389394 2.09560823 0.05090338
 2.29356845 0.00297568], Cost: 1.2414
Current parameters: [2.56411847e+00 1.48548704e+00 6.46104220e-01 2.20389394e+00
 3.09560823e+00 5.09033845e-02 2.29356845e+00 2.97568150e-03], Cost: 2.3568
Current parameters: [2.56411847 1.48548704 0.64610422 2.20389394 2.09560823 1.05090338
 2.29356845 0.00297568], Cost: 0.9195
Current parameters: [2.56411847e+00 1.48548704e+00 6.46104220e-01 2.20389394e+00
```

```
 2.09560823e+00 1.05090338e+00 3.29356845e+00 2.97568150e-03], Cost: 1.7493
Current parameters: [2.56411847 1.48548704 0.64610422 2.20389394 2.09560823 1.05090338
 2.29356845 1.00297568], Cost: 2.0157
Current parameters: [ 3.00809807  1.76442497  0.09777223  2.20652544  1.69179105  1.16744852
  1.99314978 -0.39388497], Cost: 0.7969
Current parameters: [ 2.56530329  1.83671994 -0.04434415  2.20720747  1.58712994  1.19765462
  1.91528745 -0.49674309], Cost: 1.5941
Current parameters: [ 3.00809807  1.98725072  0.21112427  2.20652544  1.69179105  1.16744852
  1.99314978 -0.39388497], Cost: 0.8696
Current parameters: [ 3.212195    1.55577446  0.41983327  2.20752379  1.5385891   1.21166393
  1.87917562 -0.54444772], Cost: 1.5873
Current parameters: [ 3.00809807  1.76442497  0.09777223  2.45652013  1.69342015  1.16744852
  1.99314978 -0.39388497], Cost: 0.8682
Current parameters: [ 3.13342261  1.7986125  -0.023531    2.1795266   2.14289979  1.19459873
  1.92316461 -0.48633716], Cost: 1.2200
Current parameters: [ 3.00809807  1.76442497  0.09777223  2.20652544  1.69179105  1.16744852
  1.79382035 -0.24299502], Cost: 1.5947
Current parameters: [ 3.14611671  1.80207534 -0.03581778  2.18055132  1.61168856  1.19734877
  2.43490796 -0.30140985], Cost: 1.6401
Current parameters: [ 3.00809807  1.76442497  0.09777223  2.20652544  1.69179105  1.39647561
  1.95799861 -0.30001812], Cost: 0.6322
Current parameters: [3.09158441 1.78719937 0.01696474 2.19081391 1.64333771 1.24056374
 1.71770295 0.08900169], Cost: 2.4773
Current parameters: [ 3.06920486  1.6187345   0.14533131  2.0987403   1.61386105  1.30242505
  1.90668082 -0.37817588], Cost: 0.7353
Current parameters: [ 3.05639087  1.75283135  0.38647947  2.32752276  1.7016494   1.71310411
  1.97961866 -0.52075088], Cost: 1.4958
Current parameters: [ 3.05094552  1.75045478  0.02003768  2.15752097  1.90255339  1.38533095
  1.9272938  -0.3808703 ], Cost: 0.4833
Current parameters: [ 3.10473     1.79380219 -0.04639979  2.16636977  1.85095622  1.40812163
  1.89390729 -0.42630873], Cost: 0.4736
Current parameters: [ 3.16037726  1.77428831 -0.12696305  2.11262794  1.84357348  1.40996459
  2.11653518 -0.43109282], Cost: 0.8960
Current parameters: [ 3.00433164  1.78990953 -0.07761574  2.14886184  1.83322469  1.41536115
  1.90116091 -0.48819266], Cost: 0.4584
Current parameters: [ 2.93455552  1.80298401 -0.17489766  2.11656311  1.87977021  1.39190771
  1.72411216 -0.37465605], Cost: 1.1728
Current parameters: [ 3.02686005  1.785145    0.05591414  2.20510874  1.8750948   1.5663741
  1.87893663 -0.61438256], Cost: 0.1949
Current parameters: [ 3.02798356  1.96852557  0.1594459   2.10569743  1.88790114  1.4931971
  1.8840445  -0.66657359], Cost: 0.5129
```



```
Current parameters: [ 2.99250625  1.78680211  0.03861802  2.27521438  1.87148642  1.48658282
  1.89780115 -0.66430824], Cost: 0.2474
Current parameters: [ 3.17162993  1.74861094  0.14946513  2.3494443   1.9424309   1.61805614
  1.82127021 -0.60480124], Cost: 0.3331
Current parameters: [ 3.05586018  1.70785669  0.0815901   2.1541707   1.84162995  1.52944099
  1.84898665 -0.66102608], Cost: 0.2970
Current parameters: [ 3.00192348  1.7897434   0.0560405   2.20926556  1.8773013   1.56071824
  1.82336296 -0.60339677], Cost: 0.2409
Current parameters: [ 2.98724780e+00  1.75817652e+00 -2.21026107e-03  2.20627895e+00
  1.89955498e+00  1.64981405e+00  1.92456196e+00 -6.31891396e-01], Cost: 0.4441
Current parameters: [ 3.05212967  1.80708889  0.0255068   2.21108977  1.84265888  1.57803511
  1.86338423 -0.63427823], Cost: 0.1583
Current parameters: [ 3.02542895  1.8061253   0.07520327  2.24182645  1.7460805   1.59852685
  1.88554523 -0.59880631], Cost: 0.2089
Current parameters: [ 3.06899016  1.80266961  0.01741561  2.20115797  1.8496127   1.55665588
  1.89006359 -0.58714947], Cost: 0.1905
Current parameters: [ 3.08493571  1.82377484 -0.05066833  2.23538014  1.79608566  1.55287007
  1.79238198 -0.64010961], Cost: 0.2978
Current parameters: [ 3.06142339  1.85655705  0.05032732  2.19066999  1.84691465  1.5710022
  1.87049862 -0.64916328], Cost: 0.1903
Current parameters: [ 3.07131279  1.80493737  0.03943086  2.22877859  1.84992994  1.57970076
  1.85731697 -0.6321072 ], Cost: 0.1551
Current parameters: [ 3.07114152  1.79847168  0.02794856  2.23859049  1.87081444  1.62813694
  1.8841449  -0.64369749], Cost: 0.1891
Current parameters: [ 3.08201571  1.79771395  0.04431145  2.20843234  1.84541892  1.59359308
  1.84595599 -0.62666433], Cost: 0.1556
Current parameters: [ 3.08233105  1.76766799  0.05358756  2.21818023  1.84378936  1.5564831
  1.87426398 -0.66702333], Cost: 0.1454
Current parameters: [ 3.09094076  1.76892041  0.03647797  2.21267975  1.8629889   1.54930285
  1.86412568 -0.67443347], Cost: 0.1690
Current parameters: [ 3.09312452  1.76411933  0.04284476  2.2395031   1.78893447  1.55604961
  1.86258495 -0.6748616 ], Cost: 0.1461
Current parameters: [ 3.06360214  1.75584775  0.07065875  2.22327069  1.84184531  1.55379756
  1.86218172 -0.66359381], Cost: 0.1693
Current parameters: [ 3.08359214  1.7585621   0.04803197  2.21996414  1.844238    1.55408069
  1.87754297 -0.65658966], Cost: 0.1392
Current parameters: [ 3.09536313  1.76711606  0.05611785  2.21345478  1.83643456  1.54423725
  1.89600381 -0.64400026], Cost: 0.1404
Current parameters: [ 3.09295396  1.75721676  0.05430996  2.22905077  1.84874056  1.55710019
  1.87580137 -0.65605712], Cost: 0.1353
Current parameters: [ 3.0931373   1.7500548   0.04934483  2.23353051  1.84822082  1.57874039
```



```
  1.89576316 -0.65972773], Cost: 0.1132
Current parameters: [ 3.10294321  1.7467853   0.03798474  2.24535197  1.82440053  1.58289403
  1.89418075 -0.66343657], Cost: 0.0988
Current parameters: [ 3.09877687  1.72990122  0.04350573  2.25882929  1.81239314  1.59684234
  1.90434734 -0.65897335], Cost: 0.1115
Current parameters: [ 3.09766414  1.75043445  0.03537714  2.25548026  1.82683693  1.57589937
  1.89975483 -0.66620101], Cost: 0.0969
Current parameters: [ 3.11509301  1.74983436  0.01362287  2.25570108  1.83597414  1.57870363
  1.90751617 -0.67309659], Cost: 0.0844
Current parameters: [ 3.12055335  1.76213516  0.0207434   2.25380032  1.83107821  1.58548039
  1.91274054 -0.69847244], Cost: 0.0771
Current parameters: [ 3.11438351  1.75973098  0.01649889  2.25728836  1.83507636  1.59121718
  1.90302777 -0.70365677], Cost: 0.0710
Current parameters: [ 3.11415551  1.78280717  0.00939679  2.2630864   1.83473561  1.60694652
  1.90145393 -0.69317165], Cost: 0.0921
Current parameters: [ 3.10530109  1.7598184   0.00953994  2.24920707  1.83060444  1.59197083
  1.9081299  -0.70480163], Cost: 0.0721
Current parameters: [ 3.12082897  1.75152405  0.00548859  2.25806263  1.83091282  1.57642281
  1.89015376 -0.72220104], Cost: 0.0722
Current parameters: [ 3.11298447  1.76593841  0.01790153  2.24978954  1.84542694  1.58568792
  1.90027069 -0.70403477], Cost: 0.0757
Current parameters: [ 3.11269751  1.74069448  0.0194177   2.26184823  1.84853764  1.60177558
  1.91494698 -0.7157921 ], Cost: 0.0658
Current parameters: [ 3.11632664  1.74801382  0.00518416  2.27687749  1.85034402  1.62249661
  1.91448008 -0.72275666], Cost: 0.0738
Current parameters: [ 3.10755952  1.74288265  0.0169068   2.27125988  1.84945255  1.59220844
  1.91869463 -0.71920644], Cost: 0.0610
Current parameters: [ 3.11451735  1.72778418  0.0093341   2.27103224  1.84168895  1.57902466
  1.90818608 -0.73647707], Cost: 0.0492
Current parameters: [ 3.09904758  1.71940485  0.02535202  2.27950248  1.83161179  1.57567064
  1.89932358 -0.74861684], Cost: 0.0653
Current parameters: [ 3.11331617  1.721361    0.00626634  2.27694416  1.84217502  1.57848606
  1.90156767 -0.72585874], Cost: 0.0507
Current parameters: [ 3.11466762  1.7158609  -0.00747845  2.26209536  1.85262187  1.56684419
  1.922117   -0.7396207 ], Cost: 0.0445
Current parameters: [ 3.13614249  1.7134034  -0.00431291  2.27016997  1.84048281  1.55794442
  1.93591568 -0.73547737], Cost: 0.0349
Current parameters: [ 3.15320785e+00  1.72238326e+00 -2.18099011e-03  2.27220420e+00
  1.85650158e+00  1.54255278e+00  1.92629133e+00 -7.38621055e-01], Cost: 0.0589
Current parameters: [ 3.13276734  1.71780995 -0.00388531  2.26211851  1.83410815  1.54935516
  1.93379081 -0.73005559], Cost: 0.0443
```



```
Current parameters: [ 3.13870247  1.71769819 -0.02723546  2.27933211  1.82777446  1.56854818
  1.94007127 -0.74261636], Cost: 0.0237
Current parameters: [ 3.15211153  1.70218306 -0.02862187  2.26546795  1.82182186  1.58658233
  1.94080418 -0.74345416], Cost: 0.0249
Current parameters: [ 3.13716837  1.71022236 -0.02577673  2.28143363  1.82244459  1.56304106
  1.93408752 -0.75182579], Cost: 0.0259
Current parameters: [ 3.12979976  1.71048605 -0.02520449  2.29411348  1.82465155  1.57678452
  1.96337432 -0.74404941], Cost: 0.0335
Current parameters: [ 3.14945317  1.72151063 -0.0323777   2.28418248  1.83440496  1.56838382
  1.9381141  -0.74664605], Cost: 0.0213
Current parameters: [ 3.15449966  1.72839581 -0.04069868  2.27982969  1.82746925  1.56307584
  1.93882613 -0.74413677], Cost: 0.0225
Current parameters: [ 3.14764749  1.72346455 -0.0328511   2.28007676  1.83643553  1.568545
  1.94057203 -0.75181183], Cost: 0.0207
Current parameters: [ 3.14268474  1.71764958 -0.04361631  2.2784255   1.84363003  1.56737356
  1.93816194 -0.74895873], Cost: 0.0202
Current parameters: [ 3.14014312  1.72040692 -0.04580379  2.28017534  1.84153583  1.57218205
  1.93505819 -0.75036762], Cost: 0.0200
Current parameters: [ 3.14213097  1.72635997 -0.04218922  2.2756227   1.85149412  1.57396371
  1.92683116 -0.7494018 ], Cost: 0.0244
Current parameters: [ 3.13655211  1.72398182 -0.04539109  2.28405719  1.84288085  1.56793023
  1.9355301  -0.75026272], Cost: 0.0216
Current parameters: [ 3.14377625  1.71657295 -0.0506104   2.28404214  1.8423165   1.58005378
  1.94539836 -0.75331608], Cost: 0.0167
Current parameters: [ 3.14694086  1.70799247 -0.05377547  2.28672075  1.83866574  1.57919777
  1.943536   -0.76453122], Cost: 0.0114
Current parameters: [ 3.14545698  1.69737113 -0.05629054  2.28584976  1.83222293  1.58229846
  1.94669509 -0.77233749], Cost: 0.0105
Current parameters: [ 3.15402727  1.70157093 -0.06651539  2.28393613  1.82707558  1.57864327
  1.9474659  -0.77443345], Cost: 0.0089
Current parameters: [ 3.15273307  1.70420031 -0.06625625  2.27956459  1.83007539  1.57896526
  1.94883269 -0.77915559], Cost: 0.0101
Current parameters: [ 3.16416507  1.69732268 -0.06798967  2.29068186  1.83190013  1.58205591
  1.94235681 -0.77825634], Cost: 0.0060
Current parameters: [ 3.1617873   1.69860257 -0.07101404  2.30290995  1.83275187  1.57581595
  1.94557219 -0.78356432], Cost: 0.0057
Current parameters: [ 3.15846355  1.69678798 -0.07631264  2.30218793  1.83566028  1.5757012
  1.94275634 -0.78226139], Cost: 0.0069
Current parameters: [ 3.15965131  1.704155   -0.07031088  2.30605893  1.82528029  1.58096764
  1.93575295 -0.78798659], Cost: 0.0099
Current parameters: [ 3.16221552  1.69661587 -0.06885409  2.30121461  1.83228169  1.57001033
```

```
  1.94186531 -0.78486742], Cost: 0.0113
Current parameters: [ 3.16688672  1.69865428 -0.07435466  2.30691675  1.8340943   1.58465666
  1.95540603 -0.77978875], Cost: 0.0037
Current parameters: [ 3.16498397  1.70522169 -0.0754364   2.31133719  1.82752473  1.59414669
  1.9495134  -0.7827444 ], Cost: 0.0050
Current parameters: [ 3.16557804  1.69443281 -0.07524381  2.3050876   1.83216095  1.58630252
  1.95733645 -0.78500336], Cost: 0.0039
Current parameters: [ 3.167522    1.70691859 -0.07398121  2.30266633  1.84373263  1.58633391
  1.95771941 -0.78728343], Cost: 0.0043
Current parameters: [ 3.16328365  1.6966263  -0.06924598  2.30917496  1.8368926   1.58681676
  1.95519865 -0.77933846], Cost: 0.0048
Current parameters: [ 3.17159534  1.69406739 -0.08345332  2.30453784  1.83903631  1.59131392
  1.95669114 -0.77330098], Cost: 0.0085
Current parameters: [ 3.16231604  1.70129262 -0.07498189  2.30494799  1.83181484  1.58395841
  1.95971737 -0.77763409], Cost: 0.0061
Current parameters: [ 3.16780324  1.69867985 -0.07247231  2.30404826  1.83327815  1.58558502
  1.95466679 -0.77903229], Cost: 0.0042
Current parameters: [ 3.17173943  1.69880977 -0.07208137  2.31015143  1.8325984   1.58128058
  1.95613368 -0.78255753], Cost: 0.0026
Current parameters: [ 3.1707949   1.69810958 -0.07300179  2.30985478  1.83296472  1.57644681
  1.95067659 -0.78488952], Cost: 0.0030
Current parameters: [ 3.17235772  1.69691394 -0.07222551  2.30903289  1.83449027  1.57950734
  1.95759306 -0.78148186], Cost: 0.0024

   Return from subroutine COBYLA because the MAXFUN limit has been reached.

   NFVALS =  100   F = 2.601249E-03    MAXCV = 0.000000E+00
   X = 3.171739E+00   1.698810E+00  -7.208137E-02   2.310151E+00   1.832598E+00
       1.581281E+00   1.956134E+00  -7.825575E-01
Current parameters: [ 3.17173943  1.69880977 -0.07208137  2.31015143  1.8325984   1.58128058
  1.95613368 -0.78255753], Cost: 0.0026
Simulating for QAOA depth p = 5
Current parameters: [1.44618595 1.84104976 2.70291317 2.72219734 2.53594736 2.33866601
 0.04130204 0.41999636 0.38265007 0.21674583], Cost: 3.3111
Current parameters: [0.44618595 2.84104976 2.70291317 2.72219734 2.53594736 2.33866601
 0.04130204 0.41999636 0.38265007 0.21674583], Cost: 0.9900
Current parameters: [0.44618595 2.84104976 3.70291317 2.72219734 2.53594736 2.33866601
 0.04130204 0.41999636 0.38265007 0.21674583], Cost: 1.2766
Current parameters: [0.44618595 2.84104976 2.70291317 3.72219734 2.53594736 2.33866601
 0.04130204 0.41999636 0.38265007 0.21674583], Cost: 2.9411
Current parameters: [0.44618595 2.84104976 2.70291317 2.72219734 3.53594736 2.33866601
```



```
 0.04130204 0.41999636 0.38265007 0.21674583], Cost: 2.0224
Current parameters: [0.44618595 2.84104976 2.70291317 2.72219734 2.53594736 3.33866601
 0.04130204 0.41999636 0.38265007 0.21674583], Cost: 0.5877
Current parameters: [0.44618595 2.84104976 2.70291317 2.72219734 2.53594736 3.33866601
 1.04130204 0.41999636 0.38265007 0.21674583], Cost: 0.9641
Current parameters: [0.44618595 2.84104976 2.70291317 2.72219734 2.53594736 3.33866601
 0.04130204 1.41999636 0.38265007 0.21674583], Cost: 1.6413
Current parameters: [0.44618595 2.84104976 2.70291317 2.72219734 2.53594736 3.33866601
 0.04130204 0.41999636 1.38265007 0.21674583], Cost: 1.8006
Current parameters: [0.44618595 2.84104976 2.70291317 2.72219734 2.53594736 3.33866601
 0.04130204 0.41999636 0.38265007 1.21674583], Cost: 1.9753
Current parameters: [-0.09821323  2.91713521  2.62629712  2.20062058  2.25995422  3.44621733
 -0.05931667  0.13834552  0.05841093 -0.15420748], Cost: 1.0374
Current parameters: [0.88349708 2.8630368  2.6807728  2.57147303 2.45619136 3.369746
 0.01222543 0.33860541 0.28895203 0.10954841], Cost: 1.4539
Current parameters: [0.44618595 3.0884315  2.70291317 2.75828437 2.53594736 3.33866601
 0.04130204 0.41999636 0.38265007 0.21674583], Cost: 0.5946
Current parameters: [0.3400987  2.77498017 2.67835651 3.15881831 2.44748718 3.37313792
 0.00905215 0.32972279 0.27872628 0.0978494 ], Cost: 1.1656
Current parameters: [0.44618595 2.84104976 2.46202273 2.72219734 2.60281885 3.33866601
 0.04130204 0.41999636 0.38265007 0.21674583], Cost: 0.7426
Current parameters: [0.38321135 2.84386635 2.86203346 2.69321598 2.9913848  3.35912893
 0.02215814 0.36640894 0.32095972 0.14616755], Cost: 1.0495
Current parameters: [0.43448577 2.84157306 2.70711488 2.71681284 2.52920429 3.0899581
 0.03774526 0.41004024 0.37118849 0.20363294], Cost: 0.6579
Current parameters: [0.44618595 2.84104976 2.70291317 2.72219734 2.53594736 3.33866601
 0.15901597 0.37794352 0.38265007 0.21674583], Cost: 0.5499
Current parameters: [0.41082857 2.84263114 2.71561054 2.70592562 2.51557013 3.34300044
 0.24785742 0.60093364 0.34801373 0.17711932], Cost: 0.7009
Current parameters: [0.39819062 2.8477576  2.69615854 2.67621408 2.51161525 3.34814795
 0.15014518 0.35311261 0.28396297 0.21674583], Cost: 0.4881
Current parameters: [0.25572633 2.86232575 2.70420974 2.56771184 2.4354914  3.3720784
 0.14398731 0.29502166 0.4120423  0.1537392 ], Cost: 0.5227
Current parameters: [0.40885003 2.85783041 2.70050546 2.60716353 2.52727412 3.34204588
 0.15585392 0.36909247 0.30235914 0.31530668], Cost: 0.3108
Current parameters: [0.32125302 2.8455958  2.72897848 2.67728681 2.4624927  3.35816236
 0.16661266 0.33417352 0.28957248 0.52265695], Cost: 0.2673
Current parameters: [0.38369425 2.84723223 2.70018806 2.6660689  2.35878128 3.35751051
 0.16722249 0.33588054 0.29153763 0.52490522], Cost: 0.1397
Current parameters: [0.5179742  2.86662648 2.75556702 2.5126133  2.30864651 3.38632515
 0.17880389 0.27129042 0.25807037 0.61821898], Cost: 0.4003
```



```
Current parameters: [0.3768343  2.8484249  2.57901237 2.65485875 2.37047841 3.35874611
 0.17176925 0.33425306 0.29571462 0.54915775], Cost: 0.1075
Current parameters: [0.37783509 2.84835818 2.57865297 2.65531612 2.37105519 3.3024148
 0.16268499 0.30882447 0.29669499 0.55027938], Cost: 0.1313
Current parameters: [0.35084476 2.85099899 2.57526056 2.63374083 2.33776599 3.31469345
 0.21260523 0.43213423 0.28272483 0.55658075], Cost: 0.2718
Current parameters: [0.37227442 2.78764727 2.579328   2.64251121 2.36803387 3.35950337
 0.17117279 0.33258345 0.30068208 0.54701119], Cost: 0.1092
Current parameters: [0.31120463 2.87268508 2.58060953 2.60628409 2.33079929 3.3734851
 0.16325396 0.30629671 0.3679364  0.52667626], Cost: 0.1351
Current parameters: [0.36430578 2.85017589 2.57724917 2.64285546 2.36412685 3.36122124
 0.11925452 0.34570475 0.2699537  0.54915775], Cost: 0.0959
Current parameters: [0.40984913 2.84468969 2.57420272 2.67989486 2.37418915 3.36357802
 0.06257453 0.38043153 0.35309418 0.57564913], Cost: 0.1973
Current parameters: [0.34072289 2.84633216 2.58441424 2.665611   2.34618824 3.3658254
 0.1217014  0.33555235 0.26543244 0.5970501 ], Cost: 0.1312
Current parameters: [0.37263529 2.84228695 2.54269944 2.6976534  2.32456817 3.39523881
 0.11199804 0.30349665 0.23487097 0.47492957], Cost: 0.2404
Current parameters: [0.38881516 2.87733488 2.56615433 2.64795825 2.38202429 3.39354242
 0.14782081 0.34035198 0.26966129 0.56361625], Cost: 0.1131
Current parameters: [0.40363754 2.83434362 2.59521457 2.54392598 2.35445234 3.36086885
 0.09187222 0.30683351 0.24454005 0.57593868], Cost: 0.2030
Current parameters: [0.33268222 2.8459061  2.58214731 2.64185476 2.41661392 3.36300978
 0.11293065 0.34200361 0.26618096 0.5430781 ], Cost: 0.1201
Current parameters: [0.37823308 2.84061192 2.5784772  2.65242045 2.37185775 3.35297366
 0.11850889 0.35462433 0.25089089 0.55433761], Cost: 0.1078
Current parameters: [0.34550423 2.86224885 2.56954503 2.62506277 2.35213891 3.35111786
 0.14454032 0.39158802 0.26970003 0.54278892], Cost: 0.1097
Current parameters: [0.36432504 2.84558615 2.54887447 2.64074347 2.36684451 3.3573178
 0.11216773 0.34611375 0.27459053 0.55632714], Cost: 0.0870
Current parameters: [0.38246319 2.84638694 2.55798897 2.65279857 2.37195868 3.35931794
 0.08594765 0.37162446 0.31899381 0.5591771 ], Cost: 0.1178
Current parameters: [0.36827754 2.84858325 2.56419113 2.62058893 2.35769563 3.36096365
 0.10316086 0.33568935 0.2770832  0.55206655], Cost: 0.0903
Current parameters: [0.3753179  2.83099903 2.51914333 2.60407936 2.38094534 3.3480096
 0.11344279 0.35102987 0.26011408 0.58502535], Cost: 0.0987
Current parameters: [0.3630175  2.84169523 2.53115429 2.64405867 2.35634006 3.36424168
 0.10844397 0.33953409 0.26718676 0.53723629], Cost: 0.0766
Current parameters: [0.36607938 2.85312723 2.52419383 2.64652179 2.35478462 3.3417573
 0.11334023 0.32578273 0.27051323 0.53017394], Cost: 0.0884
Current parameters: [0.36108354 2.85207825 2.52966481 2.64525817 2.35608361 3.36446153
```



```
 0.0998671  0.34228636 0.26095272 0.54020632], Cost: 0.0811
Current parameters: [0.34871444 2.8310751  2.5285919  2.65117835 2.33899509 3.37148998
 0.11086536 0.32982245 0.26769144 0.54940685], Cost: 0.0784
Current parameters: [0.35406559 2.83529163 2.53321707 2.64298192 2.36002434 3.35749651
 0.10177976 0.34076401 0.26628582 0.5338047 ], Cost: 0.0747
Current parameters: [0.34210223 2.83628186 2.5305343  2.64096907 2.37843544 3.3742805
 0.1069405  0.33039523 0.26784702 0.52580731], Cost: 0.0833
Current parameters: [0.34709486 2.83923848 2.53385654 2.63743726 2.35596177 3.35643561
 0.11033689 0.34727871 0.26364296 0.53088245], Cost: 0.0741
Current parameters: [0.34907988 2.83086849 2.52670763 2.63780212 2.34769971 3.36099015
 0.10337537 0.36490732 0.28114866 0.5210519 ], Cost: 0.0612
Current parameters: [0.35548656 2.81577346 2.50915405 2.62098057 2.34826957 3.35986464
 0.10202221 0.37200609 0.27553571 0.52664155], Cost: 0.0696
Current parameters: [0.34822663 2.82902166 2.52152311 2.64853825 2.35255101 3.3588755
 0.10826004 0.37065132 0.27810034 0.52297684], Cost: 0.0632
Current parameters: [0.3558243  2.82030961 2.54245825 2.64431834 2.33693258 3.36605116
 0.10096838 0.3719063  0.27577885 0.50361966], Cost: 0.0504
Current parameters: [0.35802308 2.82024505 2.54221661 2.65034723 2.32400418 3.35260759
 0.1011912  0.36877931 0.28989533 0.48416863], Cost: 0.0403
Current parameters: [0.35932483 2.81505447 2.56171842 2.65252584 2.31942721 3.34828331
 0.1009531  0.38078339 0.30935158 0.48345409], Cost: 0.0446
Current parameters: [0.36341428 2.82443791 2.54315631 2.64654689 2.33497961 3.35178208
 0.10187464 0.37152102 0.29058579 0.47691829], Cost: 0.0386
Current parameters: [0.35258618 2.8211306  2.53827773 2.65015487 2.33619229 3.36963362
 0.10142953 0.36526259 0.2968831  0.45661094], Cost: 0.0471
Current parameters: [0.35442245 2.82262903 2.54446795 2.64602805 2.33826681 3.34566579
 0.09237639 0.37373344 0.28730783 0.47492967], Cost: 0.0353
Current parameters: [0.36230399 2.84116444 2.54100676 2.65028305 2.32703622 3.34497152
 0.07707365 0.38461413 0.28116886 0.47003689], Cost: 0.0284
Current parameters: [0.35231554 2.85053558 2.54712398 2.64130905 2.31862614 3.33548701
 0.09233119 0.39064824 0.26836569 0.46124984], Cost: 0.0311
Current parameters: [0.36108283 2.84780713 2.54904943 2.6577439  2.3296943  3.34457647
 0.07720522 0.37635252 0.27979487 0.4709282 ], Cost: 0.0357
Current parameters: [0.37785335 2.82773691 2.53453713 2.64427757 2.32209439 3.33123543
 0.06614088 0.38471384 0.27264711 0.46159281], Cost: 0.0306
Current parameters: [0.36267506 2.84308185 2.5471428  2.63910971 2.32357715 3.34742624
 0.07240396 0.38007762 0.28527777 0.47079341], Cost: 0.0315
Current parameters: [0.362188   2.83741545 2.54777322 2.6549632  2.32625102 3.33931232
 0.07464672 0.40975184 0.29611099 0.4710861 ], Cost: 0.0269
Current parameters: [0.36028974 2.84194026 2.54086821 2.6549243  2.32495143 3.33094853
 0.0757874  0.40529523 0.30491736 0.46966264], Cost: 0.0305
```

```
Current parameters: [0.35875373 2.84201459 2.5325209  2.64738747 2.33627338 3.34491934
 0.0689366  0.42400747 0.28612764 0.48473195], Cost: 0.0379
Current parameters: [0.35788206 2.83630442 2.54085559 2.65813764 2.32484407 3.34792406
 0.07346724 0.41019325 0.29717854 0.46172837], Cost: 0.0241
Current parameters: [0.35455802 2.82623768 2.55859756 2.65487345 2.32000704 3.35073561
 0.06685433 0.41710944 0.30052696 0.4416121 ], Cost: 0.0173
Current parameters: [0.37493742 2.83429294 2.56149812 2.6566741  2.30488229 3.36142966
 0.073213   0.42406901 0.30368045 0.4350725 ], Cost: 0.0161
Current parameters: [0.37024699 2.83578216 2.56177767 2.66049411 2.29367078 3.36017764
 0.06592382 0.42435866 0.30107262 0.43931156], Cost: 0.0133
Current parameters: [0.36457946 2.81275715 2.56258272 2.66315724 2.27991046 3.36646758
 0.0779102  0.42313477 0.30445764 0.44389531], Cost: 0.0327
Current parameters: [0.37567106 2.83138792 2.5613293  2.66166543 2.29636444 3.36427105
 0.05672395 0.42166015 0.30891813 0.44335563], Cost: 0.0155
Current parameters: [0.36986257 2.84738692 2.57196149 2.68221341 2.30008396 3.36000237
 0.0589762  0.42142321 0.29243313 0.42963585], Cost: 0.0078
Current parameters: [0.36610838 2.86070256 2.57025266 2.67342188 2.30255738 3.36273609
 0.04285026 0.4386321  0.28325822 0.42249516], Cost: 0.0046
Current parameters: [0.36263292 2.87297005 2.56572195 2.67352274 2.29635405 3.34629933
 0.0392393  0.43415831 0.299452   0.40872875], Cost: 0.0054
Current parameters: [0.35912717 2.86718653 2.57607165 2.67253766 2.3034841  3.36937851
 0.04592121 0.43927867 0.29003935 0.42672671], Cost: 0.0046
Current parameters: [0.36662531 2.86709902 2.57490534 2.66014513 2.29849977 3.37015821
 0.03791711 0.4161186  0.27319478 0.41671221], Cost: 0.0322
Current parameters: [0.36868062 2.85792893 2.58300058 2.6693925  2.3019978  3.35615972
 0.04142641 0.44082273 0.28356271 0.42327304], Cost: 0.0043
Current parameters: [0.37619018 2.87077257 2.57854226 2.67809921 2.30613492 3.3434203
 0.04459604 0.45106531 0.28155714 0.44227669], Cost: 0.0098
Current parameters: [0.35891644 2.84523701 2.58498143 2.68340012 2.29787805 3.35760946
 0.04281301 0.46064546 0.28808875 0.41402216], Cost: 0.0046
Current parameters: [0.36443002 2.86061175 2.58820737 2.67027204 2.29560736 3.35984588
 0.03836069 0.43673996 0.27466802 0.41767103], Cost: 0.0102
Current parameters: [0.37414632 2.8597489  2.58368206 2.66944424 2.29943753 3.3589047
 0.04372501 0.44249667 0.28460011 0.42124074], Cost: 0.0041
Current parameters: [0.37552134 2.85979265 2.58437424 2.66714718 2.31241012 3.35915343
 0.04818064 0.44362815 0.28516208 0.41440745], Cost: 0.0065
Current parameters: [0.37631258 2.85986015 2.58518061 2.67351487 2.30160465 3.36055849
 0.03879494 0.44090225 0.28639765 0.42130431], Cost: 0.0058
Current parameters: [0.37498019 2.85416223 2.57907724 2.65918564 2.29657379 3.36099746
 0.04052369 0.44627226 0.29085584 0.42433475], Cost: 0.0019
Current parameters: [0.37599701 2.84604089 2.57888955 2.66078434 2.29348128 3.35963762
```



```
 0.0489893  0.43931199 0.29659046 0.4275205 ], Cost: 0.0035
Current parameters: [0.37384519 2.85670535 2.5781364  2.65888215 2.29488892 3.35741378
 0.03951345 0.4447882  0.29492426 0.42023042], Cost: 0.0018
Current parameters: [0.37580718 2.86187941 2.57360237 2.65820918 2.29349343 3.35015994
 0.04110313 0.45313114 0.29654424 0.42817951], Cost: 0.0026
Current parameters: [0.3762516  2.85988728 2.57779167 2.65536723 2.2958328  3.35704988
 0.03911695 0.43979965 0.29367968 0.42247341], Cost: 0.0030
Current parameters: [0.36764083 2.85456428 2.57842653 2.65331851 2.28523567 3.36034676
 0.03810049 0.45011885 0.29103946 0.41546256], Cost: 0.0014
Current parameters: [0.3671995  2.85340046 2.57782141 2.65019649 2.28989957 3.3605691
 0.04040283 0.45303985 0.29185191 0.41184619], Cost: 0.0017
Current parameters: [0.36939354 2.85294371 2.57697697 2.65355355 2.28501145 3.35869188
 0.03731582 0.44995173 0.28935039 0.41440492], Cost: 0.0014
Current parameters: [0.36842593 2.85286041 2.57899477 2.65486314 2.28503246 3.36273068
 0.03235085 0.4522829  0.29438896 0.41511347], Cost: 0.0013
Current parameters: [0.3697964  2.85270949 2.5823062  2.65435725 2.28464905 3.36148654
 0.03248682 0.45288359 0.29434443 0.41486821], Cost: 0.0009
Current parameters: [0.37381188 2.85547659 2.58231576 2.65699649 2.28249401 3.36459717
 0.03484475 0.45436061 0.29430286 0.41200025], Cost: 0.0014
Current parameters: [0.36880835 2.85472732 2.58283693 2.65534158 2.28592086 3.36113436
 0.03099282 0.45351792 0.29233493 0.41412915], Cost: 0.0006
Current parameters: [0.36510298 2.85258225 2.58697604 2.65951915 2.28541974 3.35855082
 0.03095628 0.453996   0.29188876 0.41325458], Cost: 0.0003
Current parameters: [0.36506979 2.85443447 2.58619959 2.65975157 2.28431607 3.35474759
 0.03083582 0.45943592 0.29257747 0.41659857], Cost: 0.0005
Current parameters: [0.36470318 2.85405704 2.58648665 2.65913143 2.28464877 3.35675082
 0.03042901 0.45292389 0.29382029 0.41137269], Cost: 0.0004
Current parameters: [0.36510298 2.85258225 2.58697604 2.65951915 2.28541974 3.35855082
 0.03095628 0.453996   0.29188876 0.41325458], Cost: 0.0003

   Return from subroutine COBYLA because the MAXFUN limit has been reached.

   NFVALS =  100   F = 3.369688E-04    MAXCV = 0.000000E+00
   X = 3.651030E-01   2.852582E+00   2.586976E+00   2.659519E+00   2.285420E+00
       3.358551E+00   3.095628E-02   4.539960E-01   2.918888E-01   4.132546E-01
Simulating for QAOA depth p = 6
Current parameters: [1.48424643 2.58704813 0.40535687 0.17822603 0.90205154 1.41646232
 0.49878924 0.7543499  0.54918835 1.87004237 1.73902112 0.92089107], Cost: 1.6812
Current parameters: [1.48424643 3.58704813 0.40535687 0.17822603 0.90205154 1.41646232
 0.49878924 0.7543499  0.54918835 1.87004237 1.73902112 0.92089107], Cost: 2.1747
Current parameters: [1.48424643 2.58704813 1.40535687 0.17822603 0.90205154 1.41646232
```



```
 0.49878924 0.7543499  0.54918835 1.87004237 1.73902112 0.92089107], Cost: 2.2167
Current parameters: [1.48424643 2.58704813 0.40535687 1.17822603 0.90205154 1.41646232
 0.49878924 0.7543499  0.54918835 1.87004237 1.73902112 0.92089107], Cost: 1.9491
Current parameters: [1.48424643 2.58704813 0.40535687 0.17822603 1.90205154 1.41646232
 0.49878924 0.7543499  0.54918835 1.87004237 1.73902112 0.92089107], Cost: 1.2544
Current parameters: [1.48424643 2.58704813 0.40535687 0.17822603 1.90205154 2.41646232
 0.49878924 0.7543499  0.54918835 1.87004237 1.73902112 0.92089107], Cost: 3.0815
Current parameters: [1.48424643 2.58704813 0.40535687 0.17822603 1.90205154 1.41646232
 1.49878924 0.7543499  0.54918835 1.87004237 1.73902112 0.92089107], Cost: 2.6298
Current parameters: [1.48424643 2.58704813 0.40535687 0.17822603 1.90205154 1.41646232
 0.49878924 1.7543499  0.54918835 1.87004237 1.73902112 0.92089107], Cost: 1.6429
Current parameters: [1.48424643 2.58704813 0.40535687 0.17822603 1.90205154 1.41646232
 0.49878924 0.7543499  1.54918835 1.87004237 1.73902112 0.92089107], Cost: 1.6951
Current parameters: [1.48424643 2.58704813 0.40535687 0.17822603 1.90205154 1.41646232
 0.49878924 0.7543499  0.54918835 2.87004237 1.73902112 0.92089107], Cost: 1.9959
Current parameters: [1.48424643 2.58704813 0.40535687 0.17822603 1.90205154 1.41646232
 0.49878924 0.7543499  0.54918835 1.87004237 2.73902112 0.92089107], Cost: 2.0225
Current parameters: [1.48424643 2.58704813 0.40535687 0.17822603 1.90205154 1.41646232
 0.49878924 0.7543499  0.54918835 1.87004237 1.73902112 1.92089107], Cost: 0.9813
Current parameters: [ 1.53088906e+00  2.40789537e+00  2.10971845e-01  8.09708875e-02
  2.05694892e+00  7.53281120e-01 -4.21702379e-04  6.13334785e-01
  3.89223396e-01  1.60088689e+00  1.46022785e+00  2.02001815e+00], Cost: 1.3379
Current parameters: [1.50244896 2.51713281 0.32949708 0.1402717  1.96250107 1.82227433
 0.30396951 0.699318   0.48676118 1.76500301 1.63022056 1.95957594], Cost: 1.4699
Current parameters: [1.72618135 2.65003618 0.40535687 0.17822603 1.90205154 1.41646232
 0.49878924 0.7543499  0.54918835 1.87004237 1.73902112 1.92089107], Cost: 0.6918
Current parameters: [1.64991772 3.13646888 0.38280786 0.16694426 1.92001992 1.39432426
 0.44087986 0.73799189 0.53063213 1.83881985 1.70668059 1.93238999], Cost: 1.3848
Current parameters: [ 1.72618135  2.65003618  0.5172179  -0.04535194  1.90205154  1.41646232
  0.49878924  0.7543499   0.54918835  1.87004237  1.73902112  1.92089107], Cost: 0.2410
Current parameters: [1.79278892 2.60455553 0.98163875 0.09470941 1.92158455 1.39239655
 0.43583729 0.73656749 0.52901631 1.83610109 1.70386448 1.93339128], Cost: 0.2058
Current parameters: [1.79278892 2.60455553 0.98163875 0.09470941 1.92158455 1.39239655
 0.55773347 0.73656749 0.52901631 1.83610109 1.48559556 1.93339128], Cost: 2.2049
Current parameters: [ 1.87512295  2.54833661  1.01242946 -0.00397632  1.94572942  1.3626487
  0.33904186  0.71458654  0.50408152  1.79414605  2.16801149  1.94884287], Cost: 2.6244
Current parameters: [1.79278892 2.60455553 0.98163875 0.09470941 1.92158455 1.39239655
 0.55257063 0.73656749 0.52901631 1.61507105 1.70822933 1.93339128], Cost: 0.6637
Current parameters: [ 1.88846166  2.53922873  1.01741779 -0.01996414  1.94964107  1.35782933
  0.65301772  0.71102546  0.5000419   2.08043187  1.36996666  1.95134614], Cost: 2.7678
Current parameters: [1.79534868 2.59472356 0.97097083 0.08937201 1.92158455 1.48722739
```



```
 0.40844041 0.73656749 0.30085251 1.82132973 1.6885642  1.93339128], Cost: 0.5366
Current parameters: [1.82583101 2.55522442 0.95550033 0.04759076 1.92878859 1.69754006
 0.16318055 0.73000909 0.73486346 1.72421102 1.55857713 1.93800153], Cost: 0.3143
Current parameters: [1.79278892 2.60455553 0.98163875 0.09470941 1.75328615 1.39239655
 0.43583729 0.55170092 0.52901631 1.83610109 1.70386448 1.93339128], Cost: 0.3534
Current parameters: [1.80308184 2.6033343  0.99383858 0.08400233 1.56438673 1.31989496
 0.36566915 1.06841066 0.52296619 1.81561092 1.66812618 1.93566802], Cost: 1.4675
Current parameters: [1.7995624  2.57853881 0.95340999 0.08058593 1.94407888 1.29608878
 0.36334144 0.71608915 0.50578605 1.79701409 1.66337787 2.13739647], Cost: 0.0471
Current parameters: [1.82229602 2.51728721 0.89615248 0.04050107 2.01950971 1.02309763
 0.14723817 0.65194868 0.44772026 1.69229832 1.5450373  1.85195631], Cost: 2.1501
Current parameters: [1.74457852 2.78972998 1.00572133 0.10675846 2.00668471 1.27224902
 0.33988446 0.73648808 0.54482058 1.84947731 1.66947628 2.09633386], Cost: 0.2525
Current parameters: [ 1.78825576  2.77419563  0.86211435 -0.03188491  1.79275791  1.40882118
  0.32799674  0.58485587  0.38205136  1.59830517  1.58405123  2.41205937], Cost: 0.3878
Current parameters: [1.56277004 2.5108322  0.97669055 0.07745022 1.94494175 1.31397697
 0.3627852  0.70697794 0.51153051 1.80003436 1.66258198 2.16649226], Cost: 0.0849
Current parameters: [1.78322931 2.59132979 0.83616045 0.08157721 1.95062226 1.29910383
 0.37931418 0.72621803 0.52057961 1.81915875 1.6762884  2.12472211], Cost: 0.0886
Current parameters: [1.81275278 2.56085991 0.94447461 0.31917643 1.94148418 1.32512495
 0.35240552 0.69401564 0.50647699 1.78840585 1.65393998 2.19560205], Cost: 0.1487
Current parameters: [1.79923022 2.57839739 0.95275622 0.08088072 1.94143446 1.29685745
 0.31251399 0.71488144 0.50367361 1.76933305 1.77408823 2.13949369], Cost: 0.2648
Current parameters: [1.84703382 2.53067054 0.99185263 0.03122619 2.01064062 1.36137931
 0.46473077 0.68471536 0.56785204 1.92813869 1.66868883 2.25145517], Cost: 0.2482
Current parameters: [1.80690966 2.56921074 0.94995795 0.07056931 1.95348851 1.37055597
 0.29918388 0.7328908  0.56884011 1.78372774 1.63198021 2.1232116 ], Cost: 0.0311
Current parameters: [1.80899897 2.5672118  0.96911871 0.07321932 1.79356317 1.34910207
 0.30647595 0.90863885 0.59405593 1.83616236 1.61332642 2.16072635], Cost: 0.3512
Current parameters: [1.81977298 2.60529921 0.95474449 0.03713053 1.91572418 1.36222136
 0.30890673 0.70260886 0.51732655 1.73385782 1.58525846 2.17965075], Cost: 0.0725
Current parameters: [1.742739   2.63253481 0.84404674 0.08236346 1.96065487 1.53189455
 0.24698906 0.69051408 0.64027555 1.7766201  1.67496048 2.19678793], Cost: 0.9105
Current parameters: [1.82014677 2.52197881 0.95132805 0.06479252 1.98146379 1.29656163
 0.26512643 0.7092483  0.56619705 1.79555388 1.56967985 2.08887733], Cost: 0.0257
Current parameters: [1.81936519 2.52344889 0.95191393 0.06570879 1.97701012 1.27516658
 0.28411192 0.70274617 0.61243813 1.76666969 1.57295202 2.09510186], Cost: 0.0400
Current parameters: [1.82708033 2.5030693  0.9669162  0.06652589 2.02911836 1.31615568
 0.32435677 0.756072   0.53234515 1.74117463 1.54880327 2.04328238], Cost: 0.4236
Current parameters: [1.82441262 2.52055301 0.96532143 0.06577742 1.94753579 1.30351105
 0.28534079 0.68219822 0.56395279 1.80376636 1.56895375 2.05314926], Cost: 0.0653
```

```
Current parameters: [1.79399932 2.53102894 0.89539792 0.06683385 1.97998713 1.33141084
 0.19986084 0.65212579 0.60674846 1.82217111 1.55945289 2.11190664], Cost: 0.3333
Current parameters: [1.8377925  2.47384196 0.94472034 0.0528923  1.97757219 1.30965509
 0.28020016 0.70105324 0.56633337 1.80076656 1.57668651 2.11201173], Cost: 0.0296
Current parameters: [1.83794117 2.55828756 1.00488084 0.04575499 2.02276807 1.28273464
 0.30510125 0.69404595 0.58367396 1.86868909 1.56045238 2.12487294], Cost: 0.1153
Current parameters: [1.81235828 2.52518009 0.94586004 0.00692662 1.98061383 1.29545348
 0.26008892 0.71891383 0.57209977 1.79678607 1.57891883 2.07423112], Cost: 0.0817
Current parameters: [1.82471159 2.51138434 0.9592979  0.0812237  1.90581676 1.26679475
 0.25176051 0.78728482 0.57878761 1.83038279 1.54936638 2.11117796], Cost: 0.1339
Current parameters: [1.87564023 2.53847573 0.93313591 0.06460464 1.98284939 1.30042377
 0.2587358  0.71420142 0.57290794 1.79495237 1.57116783 2.07919401], Cost: 0.0368
Current parameters: [1.83827635 2.5143345  0.99472583 0.0759794  1.97795547 1.2485795
 0.22301979 0.68949912 0.53618282 1.77788794 1.65568758 2.10077308], Cost: 0.3412
Current parameters: [1.8116228  2.52869273 0.91024981 0.0694295  1.98108349 1.26970916
 0.29186266 0.70606464 0.54972472 1.80942833 1.58269037 2.08944112], Cost: 0.0410
Current parameters: [1.82645301 2.53894653 0.95290442 0.04877416 1.96316557 1.29077984
 0.26834956 0.69321654 0.5409725  1.77189962 1.5435143  2.11722177], Cost: 0.0678
Current parameters: [1.82418443 2.53076833 0.96423718 0.06135325 1.99180143 1.29385336
 0.27572919 0.70588362 0.57113051 1.81427988 1.56614226 2.09776958], Cost: 0.0278
Current parameters: [1.81868333 2.52350922 0.94827423 0.06395827 1.98983815 1.30130196
 0.26407008 0.69815431 0.56634025 1.79270437 1.5713511  2.08749581], Cost: 0.0275
Current parameters: [1.81332484 2.52245452 0.93918133 0.07154399 1.97440646 1.30443777
 0.25382156 0.70755563 0.57939288 1.8082318  1.55894741 2.09551981], Cost: 0.0348
Current parameters: [1.8213127  2.52269029 0.95397939 0.06492806 1.97745957 1.29107876
 0.25736316 0.70408941 0.56607243 1.79799594 1.57873097 2.09266506], Cost: 0.0462
Current parameters: [1.81847812 2.5151581  0.95409075 0.08035818 1.99774865 1.30334218
 0.2719149  0.72422497 0.56308103 1.79047963 1.56284233 2.08519385], Cost: 0.0142
Current parameters: [1.8117867  2.51322918 0.95048477 0.05857776 2.00705954 1.30865779
 0.27281192 0.73914585 0.56676306 1.79081669 1.56051769 2.07607157], Cost: 0.0283
Current parameters: [1.82023314 2.50953191 0.95429253 0.07938521 2.00094474 1.29402402
 0.26771859 0.72101901 0.56235939 1.79158114 1.55465799 2.08135051], Cost: 0.0191
Current parameters: [1.81932194 2.52312795 0.95444528 0.07836566 1.99446312 1.30794327
 0.27775322 0.72437835 0.55053314 1.77722765 1.54586271 2.10005287], Cost: 0.0426
Current parameters: [1.82080002 2.51346944 0.96490483 0.07937256 1.99478013 1.31021741
 0.26751435 0.72252826 0.56665693 1.78802418 1.55975023 2.08183527], Cost: 0.0130
Current parameters: [1.81891749 2.51149353 0.96371094 0.08726139 1.98516569 1.31465456
 0.28540584 0.7168322  0.56431363 1.7953336  1.55915641 2.06230304], Cost: 0.0375
Current parameters: [1.80969704 2.51042878 0.96859501 0.07954969 1.99382569 1.30618541
 0.27167879 0.72055486 0.57239088 1.78373187 1.55995547 2.08470249], Cost: 0.0144
Current parameters: [1.83687206 2.51819666 0.95949259 0.0820892  1.99660295 1.30535545
```



```
 0.27365455 0.72498036 0.58793288 1.77641605 1.56040043 2.08098726], Cost: 0.0134
Current parameters: [1.81578595 2.52527308 0.9652559  0.08296722 1.99504047 1.30785154
 0.26266893 0.72430683 0.56798003 1.7880418  1.55694468 2.07686531], Cost: 0.0120
Current parameters: [1.81077233 2.51980434 0.95295102 0.09038857 1.99149826 1.31916407
 0.25427187 0.72491557 0.58139319 1.80026153 1.54663807 2.08512181], Cost: 0.0087
Current parameters: [1.81182867 2.5161042  0.95971172 0.10354557 1.9992328  1.31772005
 0.23896088 0.73207357 0.58481686 1.80291193 1.56510123 2.09109977], Cost: 0.0183
Current parameters: [1.81213534 2.5237644  0.95349733 0.09246206 1.9908547  1.32230014
 0.2541752  0.72653956 0.57675878 1.79361111 1.5396713  2.0948617 ], Cost: 0.0106
Current parameters: [1.80961129 2.5176516  0.94961655 0.09048567 1.98899597 1.3199021
 0.25158391 0.72596283 0.58030198 1.7956782  1.54753541 2.08271477], Cost: 0.0081
Current parameters: [1.80692845 2.51837552 0.94844595 0.08004703 1.99437197 1.32400986
 0.25080078 0.73440101 0.58276024 1.795623   1.54635488 2.08168887], Cost: 0.0129
Current parameters: [1.81032087 2.51692646 0.95104848 0.09085479 1.98434006 1.31750848
 0.25245503 0.73107488 0.58021268 1.79737331 1.54676735 2.08293631], Cost: 0.0097
Current parameters: [1.80866293 2.51828739 0.94739941 0.09774947 1.98769988 1.3276961
 0.25907526 0.72276511 0.57917615 1.79619203 1.54665911 2.07536038], Cost: 0.0122
Current parameters: [1.80566858 2.51668424 0.95093635 0.08955189 1.98820699 1.32117849
 0.24996837 0.72514542 0.57505874 1.79864798 1.54760295 2.083023  ], Cost: 0.0075
Current parameters: [1.80677603 2.52171425 0.94837854 0.08362158 1.97981313 1.32145046
 0.24680954 0.71676103 0.57709146 1.79994358 1.55128503 2.08541611], Cost: 0.0105
Current parameters: [1.81124927 2.51738128 0.94918842 0.08933423 1.98910125 1.32184992
 0.24739358 0.72565844 0.57220183 1.80086301 1.54639013 2.08112037], Cost: 0.0068
Current parameters: [1.80974409 2.51173873 0.94933196 0.08969098 1.99168824 1.31402968
 0.24332991 0.71976118 0.57237381 1.80038583 1.53734182 2.07818442], Cost: 0.0079
Current parameters: [1.80857273 2.52248841 0.9459069  0.0910385  1.99014025 1.31876048
 0.24690686 0.72688378 0.57158439 1.80164583 1.54628027 2.08025611], Cost: 0.0076
Current parameters: [1.81265645 2.51079424 0.94026194 0.0881057  1.99127477 1.3184213
 0.2534881  0.72484725 0.56835091 1.80262223 1.54987667 2.08688396], Cost: 0.0090
Current parameters: [1.81016981 2.51581441 0.94687363 0.09211348 1.98889943 1.32436096
 0.24421488 0.72637427 0.57559132 1.80391331 1.54547854 2.08351741], Cost: 0.0082
Current parameters: [1.81339362 2.51610522 0.95548259 0.09423058 1.99386867 1.31925108
 0.24119892 0.72518372 0.56886567 1.79767448 1.55560667 2.0811003 ], Cost: 0.0043
Current parameters: [1.81201557 2.51547316 0.95790445 0.08479556 2.00250064 1.32235372
 0.2373306  0.73023824 0.56833133 1.79523995 1.557717   2.07768827], Cost: 0.0060
Current parameters: [1.81636093 2.51779001 0.9568366  0.09339664 1.99303301 1.32195486
 0.24169885 0.72547732 0.56571222 1.79450862 1.55217014 2.08369673], Cost: 0.0030
Current parameters: [1.81121299 2.51938932 0.95675762 0.10007626 1.99722588 1.31962361
 0.23722337 0.72551811 0.56342895 1.78849365 1.55137274 2.09293699], Cost: 0.0023
Current parameters: [1.81040529 2.51765937 0.95717196 0.10426521 1.99992157 1.32170117
 0.24043562 0.72777142 0.56217122 1.7882996  1.54975057 2.09003161], Cost: 0.0039
```



```
Current parameters: [1.81408906 2.52414879 0.95938802 0.10039291 1.99267159 1.3225508
 0.23370875 0.71519749 0.559034   1.78393287 1.55559881 2.0931453 ], Cost: 0.0061
Current parameters: [1.81326446 2.51636007 0.95325358 0.09845297 1.99841941 1.31827875
 0.23995957 0.72511977 0.56046698 1.78854303 1.55348033 2.09587649], Cost: 0.0026
Current parameters: [1.81096973 2.52076994 0.95657967 0.10047953 1.99628871 1.32115166
 0.2378394  0.7274273  0.56353696 1.78883067 1.55360349 2.09380877], Cost: 0.0022
Current parameters: [1.81225357 2.51951631 0.95935013 0.10019961 1.99283766 1.317335
 0.23644645 0.73163894 0.56214989 1.78878191 1.55273181 2.09491353], Cost: 0.0012
Current parameters: [1.81366739 2.52171622 0.96335207 0.09992523 1.99541483 1.31615633
 0.23834526 0.73090546 0.562696   1.79281059 1.55222595 2.09793789], Cost: 0.0011
Current parameters: [1.81476323 2.52290574 0.96337205 0.09999808 1.9959422  1.31457354
 0.23979606 0.7311357  0.56419994 1.79062647 1.55279194 2.09735179], Cost: 0.0009
Current parameters: [1.81922623 2.5212993  0.96222002 0.10116393 1.99603539 1.31610522
 0.23708793 0.73249026 0.56837138 1.79079794 1.55167723 2.09972947], Cost: 0.0021
Current parameters: [1.81588684 2.52489978 0.9617077  0.10058463 1.99669833 1.31370876
 0.23860173 0.73173624 0.5629036  1.79164579 1.55246117 2.09636668], Cost: 0.0009
Current parameters: [1.81194354 2.52559812 0.96132097 0.09713156 1.99364729 1.31331694
 0.24175855 0.73260387 0.56494422 1.79057728 1.54888    2.09951964], Cost: 0.0023
Current parameters: [1.81524926 2.52321369 0.96424285 0.09777183 1.99755917 1.31594362
 0.23910437 0.73228813 0.56325818 1.78927719 1.55233848 2.0973077 ], Cost: 0.0008
Current parameters: [1.81242132 2.52218456 0.96506998 0.09587948 1.99990711 1.31257225
 0.23646856 0.73388018 0.5648186  1.79040763 1.55638778 2.09619283], Cost: 0.0017
Current parameters: [1.81405309 2.52371131 0.96431622 0.09953069 1.9985164  1.31551515
 0.23797656 0.73203327 0.56252746 1.7876512  1.55223306 2.09951126], Cost: 0.0006
Current parameters: [1.81647581 2.52566758 0.96769364 0.0993126  1.99540658 1.31528896
 0.23693264 0.72907424 0.55918915 1.78566907 1.55451301 2.09929916], Cost: 0.0005
Current parameters: [1.81541162 2.52701118 0.96943609 0.10043134 1.99503417 1.31610724
 0.2358476  0.72944427 0.56053672 1.78562984 1.55335796 2.09763809], Cost: 0.0003
Current parameters: [1.8160219  2.52537997 0.97190561 0.10417854 1.99642732 1.31629767
 0.23907396 0.73230811 0.55700262 1.7846907  1.55287183 2.0957235 ], Cost: 0.0011
Current parameters: [1.81496062 2.52854906 0.96903951 0.10065838 1.99401919 1.31764475
 0.23658262 0.73133861 0.56072063 1.78595147 1.55532899 2.09860573], Cost: 0.0003

   Return from subroutine COBYLA because the MAXFUN limit has been reached.

   NFVALS =  100   F = 3.334446E-04    MAXCV = 0.000000E+00
   X = 1.815412E+00   2.527011E+00   9.694361E-01   1.004313E-01   1.995034E+00
       1.316107E+00   2.358476E-01   7.294443E-01   5.605367E-01   1.785630E+00
       1.553358E+00   2.097638E+00
Current parameters: [1.81541162 2.52701118 0.96943609 0.10043134 1.99503417 1.31610724
 0.2358476  0.72944427 0.56053672 1.78562984 1.55335796 2.09763809], Cost: 0.0003
```



```
Simulating for QAOA depth p = 7
Current parameters: [2.51192827 2.49145989 1.44126955 2.08823529 0.94977253 0.07609627
 2.75247171 0.2602367  0.19709492 1.20210554 1.60797202 3.14070654
 2.67629134 0.54921626], Cost: 0.4311
Current parameters: [2.51192827 3.49145989 1.44126955 2.08823529 0.94977253 0.07609627
 2.75247171 0.2602367  0.19709492 1.20210554 1.60797202 3.14070654
 2.67629134 0.54921626], Cost: 1.0700
Current parameters: [2.51192827 2.49145989 2.44126955 2.08823529 0.94977253 0.07609627
 2.75247171 0.2602367  0.19709492 1.20210554 1.60797202 3.14070654
 2.67629134 0.54921626], Cost: 1.0805
Current parameters: [2.51192827 2.49145989 1.44126955 3.08823529 0.94977253 0.07609627
 2.75247171 0.2602367  0.19709492 1.20210554 1.60797202 3.14070654
 2.67629134 0.54921626], Cost: 1.2375
Current parameters: [2.51192827 2.49145989 1.44126955 2.08823529 1.94977253 0.07609627
 2.75247171 0.2602367  0.19709492 1.20210554 1.60797202 3.14070654
 2.67629134 0.54921626], Cost: 1.2218
Current parameters: [2.51192827 2.49145989 1.44126955 2.08823529 0.94977253 1.07609627
 2.75247171 0.2602367  0.19709492 1.20210554 1.60797202 3.14070654
 2.67629134 0.54921626], Cost: 1.2244
Current parameters: [2.51192827 2.49145989 1.44126955 2.08823529 0.94977253 0.07609627
 3.75247171 0.2602367  0.19709492 1.20210554 1.60797202 3.14070654
 2.67629134 0.54921626], Cost: 2.8764
Current parameters: [2.51192827 2.49145989 1.44126955 2.08823529 0.94977253 0.07609627
 2.75247171 1.2602367  0.19709492 1.20210554 1.60797202 3.14070654
 2.67629134 0.54921626], Cost: 0.8146
Current parameters: [2.51192827 2.49145989 1.44126955 2.08823529 0.94977253 0.07609627
 2.75247171 0.2602367  1.19709492 1.20210554 1.60797202 3.14070654
 2.67629134 0.54921626], Cost: 2.3341
Current parameters: [2.51192827 2.49145989 1.44126955 2.08823529 0.94977253 0.07609627
 2.75247171 0.2602367  0.19709492 2.20210554 1.60797202 3.14070654
 2.67629134 0.54921626], Cost: 2.9024
Current parameters: [2.51192827 2.49145989 1.44126955 2.08823529 0.94977253 0.07609627
 2.75247171 0.2602367  0.19709492 1.20210554 2.60797202 3.14070654
 2.67629134 0.54921626], Cost: 1.9660
Current parameters: [2.51192827 2.49145989 1.44126955 2.08823529 0.94977253 0.07609627
 2.75247171 0.2602367  0.19709492 1.20210554 1.60797202 4.14070654
 2.67629134 0.54921626], Cost: 3.2555
Current parameters: [2.51192827 2.49145989 1.44126955 2.08823529 0.94977253 0.07609627
 2.75247171 0.2602367  0.19709492 1.20210554 1.60797202 3.14070654
 3.67629134 0.54921626], Cost: 3.0803
Current parameters: [2.51192827 2.49145989 1.44126955 2.08823529 0.94977253 0.07609627
```

```
 2.75247171 0.2602367  0.19709492 1.20210554 1.60797202 3.14070654
 2.67629134 1.54921626], Cost: 2.5436
Current parameters: [ 2.77753106  2.39455754  1.34278339  1.96593464  0.82985567 -0.04421473
  2.38158967  0.20207371 -0.09153264  0.82727939  1.37517972  2.71232278
  2.27447817  0.22881389], Cost: 1.0219
Current parameters: [2.64472967 2.44300871 1.39202647 2.02708497 0.8898141  0.01594077
 2.56703069 0.2311552  0.05278114 1.01469246 1.49157587 2.92651466
 2.47538476 0.38901507], Cost: 1.9540
Current parameters: [2.26605711 2.48691191 1.43664723 2.08249528 0.9441444  0.07044964
 2.73506486 0.2575069  0.18354858 1.18451358 1.59704623 3.12060092
 2.65743279 0.53417861], Cost: 1.2480
Current parameters: [2.51192827 2.58056178 1.35360055 2.08823529 0.94977253 0.07609627
 2.75247171 0.2602367  0.19709492 1.20210554 1.60797202 3.14070654
 2.67629134 0.54921626], Cost: 0.4573
Current parameters: [2.52113546 2.66545893 1.61916433 2.08538921 0.94698192 0.07329649
 2.74384083 0.25888317 0.1903782  1.19338287 1.60255466 3.13073752
 2.66694066 0.5417601 ], Cost: 0.3179
Current parameters: [2.52113546 2.66545893 1.61916433 2.08538921 0.94698192 0.07329649
 2.74384083 0.25888317 0.1903782  1.19338287 1.60255466 3.2162532
 2.57577011 0.5417601 ], Cost: 0.3322
Current parameters: [2.56852462 2.67165446 1.63087492 2.07074047 0.93261871 0.05888607
 2.69941791 0.25191662 0.15580743 1.14848754 1.57467163 3.29557299
 2.82440995 0.50338346], Cost: 0.4982
Current parameters: [2.52113546 2.66545893 1.61916433 2.08538921 0.94698192 0.07329649
 2.65498623 0.25888317 0.1903782  1.2813025  1.60255466 3.13073752
 2.66694066 0.5417601 ], Cost: 0.2780
Current parameters: [2.58042789 2.67321066 1.6338164  2.06706099 0.92901094 0.05526644
 2.81038827 0.25016675 0.1471239  1.44867211 1.56766793 3.13575427
 2.67522182 0.49374396], Cost: 0.3553
Current parameters: [2.52113546 2.66545893 1.61916433 2.08538921 0.94698192 0.07329649
 2.65498623 0.25888317 0.0975047  1.2813025  1.60255466 3.13073752
 2.66694066 0.62542319], Cost: 0.1113
Current parameters: [2.59179781 2.67469714 1.63662608 2.06354637 0.92556482 0.05180899
 2.66490942 0.24849529 0.22306455 1.30362418 1.56097806 3.13671628
 2.67680982 0.81878139], Cost: 0.6030
Current parameters: [2.52113546 2.66545893 1.61916433 1.97473104 0.94698192 0.07329649
 2.65498623 0.25888317 0.0975047  1.2813025  1.66069045 3.13073752
 2.66694066 0.62542319], Cost: 0.3306
Current parameters: [2.56809375 2.67159813 1.63076844 2.22494497 0.9327493  0.05901709
 2.66158063 0.25197996 0.07027497 1.29613625 1.80028434 3.13471067
 2.67349916 0.63106492], Cost: 1.1426
```



```
Current parameters: [2.52113546 2.66545893 1.61916433 2.08538921 0.85844867 0.16153969
 2.65498623 0.25888317 0.0975047  1.2813025  1.60255466 3.13073752
 2.66694066 0.62542319], Cost: 0.1095
Current parameters: [2.54713112 2.66885753 1.62558826 2.08567267 1.03134015 0.33519617
 2.65863683 0.25506162 0.08243058 1.28951432 1.56550169 3.13293702
 2.67057138 0.6285464 ], Cost: 0.3805
Current parameters: [2.51701767 2.66696126 1.62069122 2.0872853  0.86030781 0.16340494
 2.66073622 0.38292128 0.10197945 1.28711364 1.60616376 3.13737899
 2.67317019 0.63039057], Cost: 0.4257
Current parameters: [2.62938041 2.6533399  1.61315729 2.06158212 0.82015355 0.12353672
 2.58872041 0.2331332  0.00787004 1.22394101 1.47736403 3.04991197
 2.59445921 0.56811176], Cost: 0.7205
Current parameters: [2.44691962 2.66675218 1.62047872 2.11976565 0.89383773 0.12090831
 2.65993599 0.25758373 0.04240028 1.2863049  1.55242173 3.1364547
 2.67230322 0.64455722], Cost: 0.1281
Current parameters: [2.35920182 2.67628061 1.64009567 2.01376663 0.76099194 0.23156116
 2.66510878 0.1656563  0.17488808 1.30665844 1.59419169 3.13568305
 2.67682264 0.60144871], Cost: 0.0378
Current parameters: [2.34689233 2.6810783  1.65029574 2.08318972 0.7479886  0.2329984
 2.66669662 0.11641681 0.15494909 1.31637057 1.68039037 3.133901
 2.67796147 0.60000815], Cost: 0.1331
Current parameters: [2.36237457 2.63347277 1.68239437 2.01278588 0.76003031 0.23059637
 2.66213462 0.16518988 0.17257354 1.30365265 1.59232489 3.14693828
 2.68737976 0.59887936], Cost: 0.0444
Current parameters: [2.37735571 2.70430907 1.62036561 2.00874817 0.7464035  0.22906732
 2.6487723  0.14677399 0.16129005 1.29272319 1.58149583 3.21658938
 2.75360333 0.58497062], Cost: 0.3613
Current parameters: [2.35238571 2.63481987 1.5971786  2.01245503 0.76032278 0.23635995
 2.66713387 0.16665832 0.16806573 1.30870506 1.59999817 3.13748976
 2.67851728 0.58810567], Cost: 0.0748
Current parameters: [2.37380422 2.65205753 1.62569661 2.03007097 0.75757098 0.21624829
 2.67787351 0.13696813 0.23213159 1.32377413 1.57187535 3.12894207
 2.67196093 0.6962266 ], Cost: 0.0726
Current parameters: [2.34718553 2.67406152 1.63631815 2.01738389 0.76477288 0.23457979
 2.62374847 0.16852137 0.18351778 1.26485829 1.59990098 3.13204546
 2.67341067 0.6118456 ], Cost: 0.0447
Current parameters: [2.38396649 2.6727004  1.65513133 2.00460309 0.72213521 0.2234922
 2.65041656 0.12563982 0.1469617  1.30110514 1.54579548 3.07033247
 2.61874752 0.57907942], Cost: 0.3101
Current parameters: [2.35984343 2.67636449 1.64025422 2.01361474 0.76544006 0.23566688
 2.66525468 0.16556198 0.17457249 1.3068059  1.59336065 3.09320708
```



```
 2.72225489 0.60147494], Cost: 0.0352
Current parameters: [2.36038245 2.67876288 1.64920583 2.01455955 0.84636986 0.32578265
 2.67035786 0.14525607 0.17485201 1.31512053 1.58224588 3.09482056
 2.70777653 0.60638428], Cost: 0.0693
Current parameters: [2.35984343 2.67636449 1.64025422 2.02880909 0.75936186 0.24172517
 2.70709373 0.16556198 0.18094867 1.2654071  1.58537808 3.09320708
 2.72225489 0.5957311 ], Cost: 0.0363
Current parameters: [2.34947909 2.67699339 1.64520829 1.92976025 0.79426332 0.2084262
 2.69769217 0.14984797 0.14353268 1.28117582 1.64373111 3.0862963
 2.71626176 0.63818845], Cost: 0.0984
Current parameters: [2.36056927 2.67609968 1.63998507 2.01328051 0.79440392 0.20614248
 2.66424113 0.14369767 0.20451128 1.30578156 1.59272447 3.09203639
 2.72115681 0.57291908], Cost: 0.0439
Current parameters: [2.31664045 2.68051192 1.66036713 2.0202415  0.77567822 0.23547531
 2.68174456 0.2587853  0.22554466 1.32832464 1.6187617  3.07583667
 2.70776359 0.59876632], Cost: 0.4220
Current parameters: [2.39853252 2.67556329 1.63954198 1.99605119 0.74728709 0.25538805
 2.66214612 0.16595981 0.20074856 1.30377685 1.6165652  3.08923179
 2.71856519 0.5910217 ], Cost: 0.0901
Current parameters: [2.37189349 2.67307063 1.65406477 2.00966282 0.73946924 0.22747716
 2.65164474 0.11799981 0.1308689  1.30124221 1.53541745 3.03270721
 2.66844784 0.58664772], Cost: 0.3662
Current parameters: [2.36369202 2.72143154 1.64119634 2.00038539 0.7596083  0.23405762
 2.68096733 0.16780286 0.17185104 1.32205455 1.57990529 3.11888142
 2.70776839 0.5911801 ], Cost: 0.0599
Current parameters: [2.36715325 2.67412139 1.62325313 2.01652989 0.76797594 0.23635999
 2.64141525 0.13958303 0.16516446 1.28398988 1.57622576 3.15882616
 2.81509155 0.61531939], Cost: 0.3483
Current parameters: [2.35429025 2.68908003 1.64667917 2.04731881 0.76024389 0.23568028
 2.67089186 0.1413486  0.16351698 1.31613203 1.6339513  3.09588494
 2.7170246  0.60177202], Cost: 0.0607
Current parameters: [2.36944465 2.67809769 1.6417697  2.01623724 0.77397669 0.23332372
 2.66066491 0.17839253 0.15779093 1.30018328 1.59841891 3.09295684
 2.72408942 0.58554844], Cost: 0.0373
Current parameters: [2.39645674 2.67699285 1.63193072 2.03231546 0.78304703 0.21655489
 2.67273714 0.17418377 0.17643326 1.31262904 1.58718451 3.09446085
 2.71215515 0.63555769], Cost: 0.0446
Current parameters: [2.3596525  2.67659485 1.64208434 2.01396233 0.78581243 0.25806482
 2.66665288 0.16045686 0.17434746 1.30894817 1.59064383 3.09378926
 2.71850283 0.60283375], Cost: 0.0204
Current parameters: [2.35494166 2.67713785 1.64239414 2.01505515 0.7889149  0.2582767
```



```
 2.66892923 0.17058117 0.18072899 1.31092611 1.59644837 3.09737387
 2.72173611 0.60400737], Cost: 0.0380
Current parameters: [2.35316477 2.67433374 1.64977652 2.01732308 0.78653387 0.25943669
 2.66577603 0.16205722 0.17025405 1.31149913 1.57920064 3.07356119
 2.70158795 0.60091942], Cost: 0.0175
Current parameters: [2.35485272 2.67438267 1.64886796 2.02739827 0.7830096  0.26283621
 2.66129373 0.16333991 0.1739917  1.31407703 1.57279994 3.07593064
 2.70446    0.59670124], Cost: 0.0170
Current parameters: [2.35231876 2.67129267 1.6513514  2.02524766 0.78882597 0.26537166
 2.65449306 0.14853774 0.160852   1.30654759 1.55703296 3.07551465
 2.71574105 0.60293117], Cost: 0.0423
Current parameters: [2.35754797 2.66973068 1.65227891 2.02648915 0.78250636 0.26262433
 2.66986581 0.16271073 0.17172049 1.32274026 1.57151424 3.07390542
 2.71034425 0.59387094], Cost: 0.0217
Current parameters: [2.35081705 2.66339602 1.62395204 2.02734377 0.78293144 0.26484751
 2.66474517 0.16150969 0.16818879 1.31803615 1.57168067 3.07967647
 2.69420078 0.59126376], Cost: 0.0345
Current parameters: [2.35555539 2.68188767 1.64145    2.02702307 0.78342101 0.26431088
 2.66174924 0.16341702 0.17511858 1.31454072 1.57366965 3.06638139
 2.71047223 0.59684695], Cost: 0.0202
Current parameters: [2.37387859 2.6703313  1.65387257 2.01879243 0.77486353 0.27514319
 2.65580177 0.15908943 0.18346043 1.30958163 1.578158   3.07117087
 2.69702073 0.59264691], Cost: 0.0188
Current parameters: [2.35320864 2.66852583 1.64980713 2.0355731  0.78317993 0.2631008
 2.65946343 0.15874614 0.17259871 1.31295573 1.58219939 3.07180596
 2.70614456 0.5985654 ], Cost: 0.0147
Current parameters: [2.35392212 2.6882152  1.65551959 2.04314018 0.77914372 0.26539759
 2.66428261 0.14676608 0.16586948 1.31975063 1.58927315 3.07832433
 2.69537362 0.59537432], Cost: 0.0111
Current parameters: [2.36001251 2.68837109 1.65415555 2.04637988 0.77683677 0.26755393
 2.66573963 0.15223725 0.1607     1.32092236 1.58834202 3.07875146
 2.69385969 0.60638345], Cost: 0.0135
Current parameters: [2.36804583 2.68877931 1.65369693 2.05009583 0.79545318 0.24928338
 2.66583362 0.14046212 0.17606647 1.32104732 1.58599145 3.07735307
 2.68956615 0.59785436], Cost: 0.0134
Current parameters: [2.35521371 2.68844836 1.65572345 2.03983445 0.78175562 0.26362366
 2.6535911  0.1484921  0.1620767  1.3288278  1.59187567 3.07829066
 2.6956204  0.59461484], Cost: 0.0081
Current parameters: [2.3646493  2.69026654 1.65763531 2.04445334 0.78920586 0.26246929
 2.65440279 0.16018046 0.14649983 1.31679803 1.59542663 3.07769579
 2.69679485 0.57838447], Cost: 0.0028
```

```
Current parameters: [2.36656269 2.68994995 1.65667169 2.04386967 0.77861331 0.25206314
 2.65287625 0.15861057 0.1441308  1.31504415 1.59442973 3.07603846
 2.69727146 0.57730338], Cost: 0.0047
Current parameters: [2.35689817 2.69656525 1.67122192 2.04994786 0.78949988 0.2580482
 2.64923696 0.17764718 0.15633215 1.3173827  1.60883303 3.07820236
 2.69201394 0.57510469], Cost: 0.0181
Current parameters: [2.36240508 2.69267647 1.66120964 2.04631697 0.78975018 0.2621185
 2.64818328 0.16070491 0.14624173 1.3128583  1.58551438 3.08561495
 2.69629445 0.57931528], Cost: 0.0032
Current parameters: [2.36622902 2.68963262 1.66658018 2.04168067 0.79729507 0.26767268
 2.64716801 0.14182518 0.13267102 1.31047103 1.59805926 3.06893043
 2.70592363 0.58079978], Cost: 0.0162
Current parameters: [2.36245478 2.68609521 1.64754554 2.04563522 0.79000889 0.26434458
 2.65289251 0.15888561 0.14315702 1.31785791 1.59558331 3.08175508
 2.68899425 0.57380669], Cost: 0.0046
Current parameters: [2.36345322 2.68896071 1.6601866  2.04531373 0.78919736 0.26267593
 2.65548786 0.16078834 0.14525594 1.31899186 1.59167799 3.07351727
 2.6936537  0.57723513], Cost: 0.0052
Current parameters: [2.36782289 2.68683016 1.6597894  2.04446925 0.78871761 0.26228504
 2.66002686 0.1602268  0.14521551 1.32428352 1.59400241 3.08566253
 2.70334779 0.57395787], Cost: 0.0086
Current parameters: [2.36022781 2.6921505  1.65538073 2.04635463 0.79128859 0.25974025
 2.65583257 0.16095092 0.14429645 1.31777703 1.5949182  3.07705342
 2.69942689 0.57956886], Cost: 0.0039
Current parameters: [2.36900961 2.69873089 1.65107552 2.0442653  0.78748635 0.26700628
 2.65169842 0.16165068 0.15030945 1.31782025 1.5914435  3.07356216
 2.70042219 0.57441799], Cost: 0.0031
Current parameters: [2.36477282 2.69536008 1.65870124 2.04149402 0.78925266 0.26202123
 2.65682362 0.15872445 0.14467267 1.31772501 1.5960358  3.07934184
 2.69367732 0.57835001], Cost: 0.0028
Current parameters: [2.36473926 2.68706751 1.65481675 2.03214077 0.79279009 0.2604008
 2.6548903  0.16574178 0.14751577 1.31323385 1.59252236 3.07685135
 2.69780469 0.57985442], Cost: 0.0044
Current parameters: [2.36649466 2.69035916 1.65752234 2.04531011 0.79335919 0.25839233
 2.65443476 0.15758351 0.14990361 1.31683481 1.59489305 3.07738571
 2.69582162 0.57655624], Cost: 0.0028
Current parameters: [2.3738585  2.69081465 1.65497548 2.04860464 0.79124422 0.261498
 2.65485652 0.1638053  0.14396187 1.31756877 1.59484074 3.07828719
 2.69390339 0.58839398], Cost: 0.0032
Current parameters: [2.36428203 2.69036682 1.65614595 2.04770072 0.78710975 0.26506229
 2.66424351 0.15859971 0.15022885 1.30663898 1.59381101 3.07823878
```



```
 2.69616313 0.57898435], Cost: 0.0065
Current parameters: [2.36261801 2.68994485 1.65655994 2.04317845 0.78752605 0.26311671
 2.65367053 0.15721572 0.15049017 1.31956897 1.59497704 3.07808
 2.69631803 0.58254127], Cost: 0.0033
Current parameters: [2.36398238 2.69075273 1.65786554 2.04505608 0.78859536 0.26176058
 2.65464001 0.16287561 0.14830051 1.31735999 1.59619129 3.07840949
 2.69573018 0.57795976], Cost: 0.0026
Current parameters: [2.36397938 2.6925201  1.6595857  2.04509628 0.78992532 0.26254539
 2.64980809 0.16289445 0.14856914 1.31544588 1.60119924 3.07798586
 2.69548979 0.5783668 ], Cost: 0.0016
Current parameters: [2.36661919 2.69218865 1.66157776 2.04480081 0.79045063 0.26342425
 2.6491849  0.16318612 0.14879528 1.31842357 1.60389575 3.08217168
 2.69896333 0.57675318], Cost: 0.0042
Current parameters: [2.36339093 2.69259543 1.65986242 2.04560509 0.79205816 0.26507634
 2.65126068 0.16305944 0.14947728 1.31478654 1.60120663 3.07831323
 2.6953629  0.57867772], Cost: 0.0019
Current parameters: [2.3643479  2.69338902 1.65916262 2.04635926 0.78653658 0.26129074
 2.65133374 0.161688   0.15006762 1.30941338 1.60277083 3.07770066
 2.69506254 0.57876567], Cost: 0.0020
Current parameters: [2.36365646 2.69231447 1.65811277 2.04727708 0.78945977 0.26327661
 2.64938071 0.16173291 0.14785886 1.31615215 1.60176783 3.07898431
 2.69394242 0.5772779 ], Cost: 0.0014
Current parameters: [2.36313853 2.69033547 1.65369376 2.04318301 0.79090541 0.26307268
 2.64751567 0.16329566 0.14734962 1.31417873 1.60225618 3.07996755
 2.69135501 0.57660281], Cost: 0.0021
Current parameters: [2.36503145 2.6918112  1.65705622 2.04729351 0.7895557  0.26324027
 2.65087977 0.16198982 0.14762512 1.31717513 1.60391068 3.07715754
 2.69373531 0.57805361], Cost: 0.0017
Current parameters: [2.36724433 2.6932938  1.65773623 2.04971238 0.79047967 0.26269842
 2.6478468  0.1634931  0.14661631 1.31549532 1.59973984 3.08062471
 2.6923087  0.5820588 ], Cost: 0.0009
Current parameters: [2.36827986 2.69548725 1.65594607 2.04966731 0.79010147 0.26374871
 2.64715933 0.16384446 0.14756711 1.31566574 1.59892211 3.07978716
 2.69341094 0.5811598 ], Cost: 0.0010
Current parameters: [2.36678052 2.69377717 1.66060454 2.05119882 0.78990721 0.26322954
 2.64510959 0.16403616 0.14601403 1.31563113 1.5979086  3.07620594
 2.68807482 0.58130597], Cost: 0.0008
Current parameters: [2.36448323 2.69505933 1.65982747 2.0524669  0.79087509 0.26178909
 2.64479452 0.16442967 0.14487608 1.31561861 1.5980955  3.07561509
 2.68883794 0.58207935], Cost: 0.0008
Current parameters: [2.36440924 2.69378391 1.65953911 2.05317681 0.79082771 0.26191443
```



```
 2.64417601 0.16472918 0.1454161  1.31544672 1.59793421 3.07521331
 2.68960422 0.58217639], Cost: 0.0008
Current parameters: [2.3633478  2.6953924  1.65948772 2.05188048 0.79005289 0.26198777
 2.64405919 0.16290418 0.14668645 1.31679966 1.59812424 3.07574098
 2.6882012  0.58425226], Cost: 0.0007
Current parameters: [2.36288821 2.69536815 1.65951642 2.05166605 0.78901428 0.26300722
 2.64405313 0.1635535  0.14583547 1.31679332 1.59825694 3.07582141
 2.68844692 0.58470808], Cost: 0.0006
Current parameters: [2.3632209  2.69645685 1.66104769 2.05261579 0.788347   0.26277245
 2.6421268  0.16364523 0.14514099 1.31796065 1.59935996 3.07764628
 2.68871721 0.58488602], Cost: 0.0003
Current parameters: [2.36327703 2.69739198 1.66136766 2.05378024 0.78641508 0.26214178
 2.64227868 0.16300675 0.14550691 1.31531224 1.60018169 3.0780522
 2.68832716 0.58535719], Cost: 0.0002

   Return from subroutine COBYLA because the MAXFUN limit has been reached.

   NFVALS =  100   F = 3.482753E-04    MAXCV = 0.000000E+00
   X = 2.363221E+00   2.696457E+00   1.661048E+00   2.052616E+00   7.883470E-01
       2.627725E-01   2.642127E+00   1.636452E-01   1.451410E-01   1.317961E+00
       1.599360E+00   3.077646E+00   2.688717E+00   5.848860E-01
Current parameters: [2.3632209  2.69645685 1.66104769 2.05261579 0.788347   0.26277245
 2.6421268  0.16364523 0.14514099 1.31796065 1.59935996 3.07764628
 2.68871721 0.58488602], Cost: 0.0003
Simulating for QAOA depth p = 8
Current parameters: [2.90522073 2.90052269 0.77701525 2.18389994 2.73633721 2.80072956
 0.79742043 2.65880151 2.73240954 0.85643509 2.67062149 0.2607416
 1.11747018 1.01992322 3.09624514 2.27139804], Cost: 0.5691
Current parameters: [2.90522073 3.90052269 0.77701525 2.18389994 2.73633721 2.80072956
 0.79742043 2.65880151 2.73240954 0.85643509 2.67062149 0.2607416
 1.11747018 1.01992322 3.09624514 2.27139804], Cost: 1.1541
Current parameters: [2.90522073 2.90052269 1.77701525 2.18389994 2.73633721 2.80072956
 0.79742043 2.65880151 2.73240954 0.85643509 2.67062149 0.2607416
 1.11747018 1.01992322 3.09624514 2.27139804], Cost: 1.8769
Current parameters: [2.90522073 2.90052269 0.77701525 3.18389994 2.73633721 2.80072956
 0.79742043 2.65880151 2.73240954 0.85643509 2.67062149 0.2607416
 1.11747018 1.01992322 3.09624514 2.27139804], Cost: 1.4297
Current parameters: [2.90522073 2.90052269 0.77701525 2.18389994 3.73633721 2.80072956
 0.79742043 2.65880151 2.73240954 0.85643509 2.67062149 0.2607416
 1.11747018 1.01992322 3.09624514 2.27139804], Cost: 1.4297
Current parameters: [2.90522073 2.90052269 0.77701525 2.18389994 2.73633721 3.80072956
```



```
 0.79742043 2.65880151 2.73240954 0.85643509 2.67062149 0.2607416
 1.11747018 1.01992322 3.09624514 2.27139804], Cost: 0.8327
Current parameters: [2.90522073 2.90052269 0.77701525 2.18389994 2.73633721 2.80072956
 1.79742043 2.65880151 2.73240954 0.85643509 2.67062149 0.2607416
 1.11747018 1.01992322 3.09624514 2.27139804], Cost: 1.7513
Current parameters: [2.90522073 2.90052269 0.77701525 2.18389994 2.73633721 2.80072956
 0.79742043 3.65880151 2.73240954 0.85643509 2.67062149 0.2607416
 1.11747018 1.01992322 3.09624514 2.27139804], Cost: 1.8572
Current parameters: [2.90522073 2.90052269 0.77701525 2.18389994 2.73633721 2.80072956
 0.79742043 2.65880151 3.73240954 0.85643509 2.67062149 0.2607416
 1.11747018 1.01992322 3.09624514 2.27139804], Cost: 1.0959
Current parameters: [2.90522073 2.90052269 0.77701525 2.18389994 2.73633721 2.80072956
 0.79742043 2.65880151 2.73240954 1.85643509 2.67062149 0.2607416
 1.11747018 1.01992322 3.09624514 2.27139804], Cost: 1.1621
Current parameters: [2.90522073 2.90052269 0.77701525 2.18389994 2.73633721 2.80072956
 0.79742043 2.65880151 2.73240954 0.85643509 3.67062149 0.2607416
 1.11747018 1.01992322 3.09624514 2.27139804], Cost: 2.8092
Current parameters: [2.90522073 2.90052269 0.77701525 2.18389994 2.73633721 2.80072956
 0.79742043 2.65880151 2.73240954 0.85643509 2.67062149 1.2607416
 1.11747018 1.01992322 3.09624514 2.27139804], Cost: 2.3092
Current parameters: [2.90522073 2.90052269 0.77701525 2.18389994 2.73633721 2.80072956
 0.79742043 2.65880151 2.73240954 0.85643509 2.67062149 0.2607416
 2.11747018 1.01992322 3.09624514 2.27139804], Cost: 2.6703
Current parameters: [2.90522073 2.90052269 0.77701525 2.18389994 2.73633721 2.80072956
 0.79742043 2.65880151 2.73240954 0.85643509 2.67062149 0.2607416
 1.11747018 2.01992322 3.09624514 2.27139804], Cost: 1.7537
Current parameters: [2.90522073 2.90052269 0.77701525 2.18389994 2.73633721 2.80072956
 0.79742043 2.65880151 2.73240954 0.85643509 2.67062149 0.2607416
 1.11747018 1.01992322 4.09624514 2.27139804], Cost: 1.5104
Current parameters: [2.90522073 2.90052269 0.77701525 2.18389994 2.73633721 2.80072956
 0.79742043 2.65880151 2.73240954 0.85643509 2.67062149 0.2607416
 1.11747018 1.01992322 3.09624514 3.27139804], Cost: 0.8477
Current parameters: [ 3.24527564  2.78353986  0.51549694  2.01182005  2.56425766  2.74803002
  0.56102535  2.40123199  2.62706735  0.73786736  2.2226806  -0.08720904
  0.69730027  0.78304183  2.90802601  2.21569261], Cost: 2.4759
Current parameters: [3.07524818 2.84203128 0.64625609 2.09785999 2.65029743 2.77437979
 0.67922289 2.53001675 2.67973845 0.79715123 2.44665104 0.08676628
 0.90738522 0.90148253 3.00213558 2.24354532], Cost: 0.8069
Current parameters: [2.66756693 2.89087042 0.75543734 2.1697016  2.7221389  2.79638131
 0.77791544 2.63754942 2.72371774 0.84665205 2.63366183 0.23203215
 1.08280191 1.00037811 3.08071516 2.26680178], Cost: 0.6809
```

```
Current parameters: [2.90522073 3.01462696 0.72597393 2.18389994 2.73633721 2.80072956
 0.79742043 2.65880151 2.73240954 0.85643509 2.67062149 0.2607416
 1.11747018 1.01992322 3.09624514 2.27139804], Cost: 0.5784
Current parameters: [2.90933097 2.99778506 0.99718772 2.17094491 2.7233822  2.79676208
 0.77962343 2.63941039 2.72447885 0.84750872 2.63689827 0.23454614
 1.08583769 1.00208961 3.08207507 2.26720426], Cost: 0.6654
Current parameters: [2.90522073 2.90052269 0.77701525 2.2722882  2.64794877 2.80072956
 0.79742043 2.65880151 2.73240954 0.85643509 2.67062149 0.2607416
 1.11747018 1.01992322 3.09624514 2.27139804], Cost: 0.5940
Current parameters: [2.9089367  2.89844002 0.77483638 2.35378191 2.91004642 2.79714265
 0.78133058 2.64127046 2.7252396  0.84836497 2.64013312 0.23705891
 1.088872   1.00380027 3.08343431 2.26760654], Cost: 0.9134
Current parameters: [2.90522073 2.90052269 0.77701525 2.18389994 2.73633721 2.67872449
 0.82461901 2.65880151 2.73240954 0.85643509 2.67062149 0.2607416
 1.11747018 1.01992322 3.09624514 2.27139804], Cost: 0.7463
Current parameters: [2.90891645 2.89845137 0.77484825 2.17079452 2.72703854 2.87218142
 1.02975133 2.64136599 2.72527866 0.84840895 2.64029925 0.23718795
 1.08902783 1.00388813 3.08350412 2.2676272 ], Cost: 0.4000
Current parameters: [2.90891645 2.89845137 0.77484825 2.17079452 2.72703854 2.87218142
 1.02975133 2.64136599 2.72527866 0.84840895 2.7258163  0.23718795
 0.99785856 1.00388813 3.08350412 2.2676272 ], Cost: 0.8466
Current parameters: [2.91995281 2.89226589 0.76837706 2.13165846 2.69927039 2.9343285
 1.04519263 2.58929913 2.70398408 0.82444086 2.65083704 0.16685085
 1.29694615 0.95600331 3.04545622 2.25636652], Cost: 0.2623
Current parameters: [2.91995281 2.89226589 0.76837706 2.13165846 2.69927039 2.9343285
 1.04519263 2.48883078 2.70398408 0.82444086 2.65083704 0.24122227
 1.29694615 0.95600331 3.04545622 2.25636652], Cost: 0.3008
Current parameters: [2.92978261 2.88675665 0.76261334 2.09680095 2.67453799 2.98968135
 1.0589458  2.70799731 2.68501753 0.80309309 2.52527908 0.30858056
 1.35555615 0.91335352 3.01156792 2.24633692], Cost: 0.2545
Current parameters: [2.92978261 2.88675665 0.76261334 2.09680095 2.67453799 2.98968135
 1.0589458  2.70799731 2.68501753 0.80309309 2.52527908 0.30858056
 1.35555615 0.991116   2.91370069 2.24633692], Cost: 0.2235
Current parameters: [2.93721348 2.88259191 0.75825623 2.07045032 2.65584147 3.03152549
 1.06934255 2.69111313 2.67067971 0.78695518 2.43036318 0.27169615
 1.39986254 1.16381794 3.04230257 2.23875502], Cost: 0.9216
Current parameters: [2.92978261 2.88675665 0.76261334 2.09680095 2.67453799 2.98968135
 1.0589458  2.70799731 2.59157127 0.88611596 2.52527908 0.30858056
 1.35555615 0.991116   2.91370069 2.24633692], Cost: 0.2552
Current parameters: [2.93284384 2.88504094 0.76081838 2.08594551 2.66683574 3.00691952
 1.06322886 2.70104168 2.84714956 0.98130041 2.48617738 0.2933856
```



```
 1.37380868 0.95973697 2.88521584 2.24321347], Cost: 0.3085
Current parameters: [2.90880154 2.89397438 0.77874877 2.10741812 2.68515514 2.99293286
 1.07353116 2.72388911 2.69151704 0.81040861 2.55291661 0.33004879
 1.38148024 1.00573136 2.92531363 2.3551029 ], Cost: 0.6519
Current parameters: [3.00117695 2.86177303 0.70806486 2.05472999 2.63448068 2.98992809
 1.01340884 2.65095966 2.65930514 0.77141933 2.40883946 0.2278255
 1.28141291 0.92270647 2.85707621 2.34620821], Cost: 0.4615
Current parameters: [3.04238485 2.89020066 0.77031253 2.13032846 2.70806543 2.99121147
 1.06580948 2.71547578 2.68807611 0.80653567 2.53828493 0.31868322
 1.36775568 0.9979938  2.91916559 2.24795432], Cost: 0.3149
Current parameters: [2.85299038 2.8804543  0.75145914 2.23449363 2.81676551 3.01262762
 1.05682152 2.68896671 2.67186303 0.78213108 2.45334221 0.27453218
 1.36708332 0.93768299 2.86613566 2.18183402], Cost: 0.5007
Current parameters: [2.93176571 2.93417624 0.86862114 2.09055045 2.66828749 3.01978772
 1.04322081 2.69864154 2.68119115 0.79878632 2.50900839 0.29594185
 1.34029419 0.98251169 2.90686395 2.24431352], Cost: 0.1343
Current parameters: [2.93669229 2.95974391 0.92854217 2.05773875 2.63974666 2.81149733
 1.11025973 2.69918378 2.67635472 0.78754548 2.47337454 0.28882049
 1.38126908 0.9491736  2.87556352 2.18756326], Cost: 0.3933
Current parameters: [2.90192057 2.91440487 0.8244217  2.05709708 2.63483419 3.02135956
 1.04295968 2.70857759 2.70180227 0.75385137 2.55675843 0.30936445
 1.32401862 1.00159569 2.86422823 2.19236011], Cost: 0.2102
Current parameters: [3.00278381 2.96830017 0.95397334 2.06463696 2.6563852  3.13479849
 1.03345073 2.70305945 2.63334648 0.8620283  2.57107207 0.27614392
 1.29312187 0.85569738 2.90578468 2.16678558], Cost: 0.6259
Current parameters: [2.88579995 2.92887193 0.85794858 2.15553385 2.73510248 3.02941535
 1.04164484 2.69001211 2.67924414 0.77866079 2.47744334 0.28091588
 1.34433906 0.96231804 2.87565066 2.2057907 ], Cost: 0.2378
Current parameters: [2.93168477 2.9342015  0.86867759 2.05249488 2.70683381 3.01925133
 1.04274599 2.67400204 2.68134733 0.7989621  2.50898499 0.31497969
 1.33892544 0.98286288 2.90714299 2.2443961 ], Cost: 0.1048
Current parameters: [2.93344046 2.93494861 0.87071556 2.09411034 2.6619309  3.02289581
 1.04319317 2.58800933 2.68064878 0.80046606 2.50389147 0.38126213
 1.34106603 0.97952819 2.9064504  2.23963978], Cost: 0.1625
Current parameters: [2.93158385 2.8775222  0.89411595 2.05285274 2.70708772 3.01730021
 1.0364018  2.67447814 2.68154205 0.79918126 2.50981299 0.31562286
 1.3397021  0.98330074 2.90749091 2.24449907], Cost: 0.0928
Current parameters: [2.93326789 2.8911592  0.89338567 2.0532146  2.70949771 2.99004222
 0.9198132  2.68384326 2.68303799 0.80490648 2.5166709  0.32727695
 1.36665647 0.98355196 2.91114408 2.23754865], Cost: 0.1440
Current parameters: [2.9338916  2.87622879 0.8927628  2.04466924 2.7012813  3.03029541
```



```
 1.03963063 2.70497873 2.67708927 0.79416945 2.47818265 0.35215767
 1.35144239 0.97328784 2.89953495 2.24214442], Cost: 0.1107
Current parameters: [2.92831854 2.8815436  0.90107268 2.07005816 2.72120822 2.98883301
 1.02736062 2.69991575 2.69205777 0.81460732 2.50064864 0.34970345
 1.23436042 1.00418224 2.9271506  2.24318294], Cost: 0.0492
Current parameters: [2.91692877 2.88867709 0.91714325 2.07833306 2.72967387 2.9934508
 1.03821899 2.711324   2.6963933  0.82627326 2.51885497 0.36476403
 1.25476135 1.02970192 2.9531846  2.21869923], Cost: 0.0768
Current parameters: [2.9405635  2.87254336 0.88968283 2.04004749 2.70493229 3.00790136
 1.01614435 2.67496801 2.68871833 0.80217354 2.42712057 0.30922956
 1.22045833 1.06308024 2.96668555 2.2196996 ], Cost: 0.1674
Current parameters: [2.92445102 2.8782106  0.89415505 2.06540086 2.71737512 2.99158702
 1.0281932  2.69762655 2.73541263 0.85406461 2.49032727 0.34509529
 1.23759684 0.99908054 2.91517303 2.23700427], Cost: 0.0550
Current parameters: [2.96714823 2.89359953 0.94384245 2.04605496 2.72208179 3.03909688
 1.02190062 2.70069058 2.66636079 0.84361742 2.53297751 0.33783274
 1.19323828 0.94329716 2.92808882 2.21281885], Cost: 0.1825
Current parameters: [2.93676765 2.89471171 0.93723011 2.04904813 2.71077847 2.88657823
 1.0586506  2.69798887 2.68593262 0.81277038 2.4807841  0.34253211
 1.24439868 0.98275193 2.91448979 2.21357554], Cost: 0.0911
Current parameters: [2.90618755 2.88388826 0.91515199 2.0608259  2.72595392 2.98373432
 1.03342294 2.70369447 2.70208131 0.81195702 2.50870445 0.35345443
 1.23085371 1.01286341 2.92235523 2.29544042], Cost: 0.0965
Current parameters: [2.94052183 2.88441702 0.90749629 2.07333056 2.72448061 2.99058679
 1.02726794 2.70022537 2.71209119 0.79706354 2.5011871  0.35012171
 1.23486549 1.00036175 2.93254346 2.2432499 ], Cost: 0.0551
Current parameters: [2.97019796 2.87292705 0.88546843 2.08069273 2.73762877 2.98052552
 1.03429519 2.70463918 2.685783   0.82537371 2.50947755 0.35552398
 1.23806253 1.0273004  2.90655836 2.25173704], Cost: 0.0748
Current parameters: [2.93526947 2.87602866 0.88856144 2.06106712 2.71193528 2.98326276
 1.02973593 2.70246361 2.69277567 0.8185074  2.50747949 0.35366378
 1.2358859  0.99782293 2.94635457 2.24963914], Cost: 0.0578
Current parameters: [2.90858049 2.86787117 0.87687294 2.09542183 2.75662009 2.98202534
 1.03213472 2.69842865 2.69052011 0.80973217 2.49206562 0.34529364
 1.23319764 0.98044289 2.9349806  2.23770857], Cost: 0.0646
Current parameters: [2.92345543 2.87840437 0.89398219 2.05666115 2.72513201 2.98834934
 1.02723135 2.71833126 2.69531502 0.80751137 2.50888006 0.33869899
 1.2314707  1.00772972 2.92083677 2.23626131], Cost: 0.0608
Current parameters: [2.91395804 2.87179373 0.88448776 2.07234137 2.70041948 2.98727506
 1.02924634 2.67333417 2.69889298 0.79779982 2.5178082  0.37719104
 1.22358901 1.00858918 2.90733053 2.22667692], Cost: 0.0646
```



```
Current parameters: [2.92860791 2.88422316 0.90090217 2.05771923 2.73422527 2.98355458
 1.00537263 2.69363718 2.69238859 0.81573695 2.50192434 0.35809214
 1.23896328 1.0043429  2.92792538 2.24190729], Cost: 0.0338
Current parameters: [2.92592224 2.8871899  0.89994772 2.08950016 2.71007977 2.97082713
 0.96387601 2.71092511 2.69218124 0.81740008 2.50515279 0.35093958
 1.24521541 1.00251262 2.92574596 2.24327943], Cost: 0.0282
Current parameters: [2.92652344 2.86329936 0.91013124 2.08736826 2.70856713 2.97363846
 0.96214202 2.71962225 2.69102124 0.81609444 2.49668222 0.36138761
 1.24880946 0.99990414 2.92367334 2.24266602], Cost: 0.0391
Current parameters: [2.92592115 2.91911097 0.8832726  2.08563808 2.70627972 2.97986767
 0.96643117 2.73438396 2.68802216 0.81312626 2.47902006 0.38252342
 1.25276626 0.99290524 2.91703138 2.24341536], Cost: 0.0480
Current parameters: [2.92953888 2.89060523 0.90846257 2.09108447 2.71304634 2.97525286
 0.96513708 2.71537886 2.68912171 0.82420178 2.52295304 0.35617441
 1.24200642 0.98321781 2.91911511 2.24253125], Cost: 0.0484
Current parameters: [2.93443654 2.8938982  0.90258142 2.08128383 2.7076802  2.98589335
 0.93661689 2.68670432 2.67956256 0.82152225 2.48116969 0.33104148
 1.22224895 1.01038022 2.93962274 2.22836012], Cost: 0.0089
Current parameters: [2.9354441  2.89414158 0.89687477 2.07351796 2.70055526 3.00047797
 0.93099715 2.66536658 2.67129277 0.81340126 2.47849624 0.30942566
 1.27058185 1.0031657  2.93156166 2.22802334], Cost: 0.0065
Current parameters: [2.94166366 2.89656384 0.9020775  2.07058346 2.69727446 2.97874924
 0.93668903 2.66413363 2.66790917 0.81336028 2.47242632 0.30701245
 1.27333064 0.99656776 2.92950795 2.21158895], Cost: 0.0046
Current parameters: [2.92118477 2.90873591 0.91937765 2.05924387 2.69880806 2.95107755
 0.92474413 2.64372698 2.66823642 0.81120121 2.45747614 0.29605944
 1.26987336 1.00944214 2.9287569  2.24410245], Cost: 0.0328
Current parameters: [2.9228044  2.90197396 0.91291133 2.06953362 2.69422932 2.98320371
 0.93784785 2.66626746 2.67109915 0.81355204 2.47539846 0.30998297
 1.2788043  0.99695264 2.94547581 2.20040038], Cost: 0.0079
Current parameters: [2.96406458 2.90013505 0.89881964 2.08619027 2.71526405 2.97702169
 0.93170421 2.65911493 2.66100636 0.82906646 2.47938401 0.31299583
 1.28864289 1.03857322 2.93771812 2.19381285], Cost: 0.0300
Current parameters: [2.94363931 2.89811652 0.90500462 2.06176267 2.67781918 2.98068284
 0.93558194 2.65649107 2.67071316 0.80921357 2.4815373  0.3173271
 1.27000985 1.00678301 2.91965248 2.20771285], Cost: 0.0148
Current parameters: [2.91971607 2.88209402 0.86942657 2.08884552 2.70753017 2.97272746
 0.93073559 2.63697679 2.67056852 0.79738996 2.47107368 0.31583967
 1.26350354 0.98474359 2.92599673 2.19396432], Cost: 0.0120
Current parameters: [2.94235414 2.89741517 0.90370432 2.06957747 2.70008935 2.97729588
 0.93625379 2.66973105 2.64767883 0.79249721 2.47943721 0.30656562
```

```
 1.27105707 0.99990614 2.93377773 2.21289857], Cost: 0.0036
Current parameters: [2.93223795 2.89323836 0.8858363  2.04259651 2.70895874 2.97740377
 0.92523994 2.69799287 2.66038344 0.78847808 2.48399571 0.27523313
 1.26485048 1.00817543 2.92142612 2.19554963], Cost: 0.0163
Current parameters: [2.93617158 2.89193794 0.89135723 2.06181564 2.69216902 2.97271341
 0.9376661  2.67090772 2.63151643 0.80919764 2.48283304 0.3084467
 1.27149721 0.99948944 2.94011695 2.21647362], Cost: 0.0087
Current parameters: [2.96912971 2.89420216 0.88856057 2.05883993 2.69053658 2.97047644
 0.93025049 2.66796253 2.66946117 0.77775026 2.48342748 0.30584513
 1.2729969  0.98696601 2.97502048 2.22166677], Cost: 0.0329
Current parameters: [2.93938    2.89961296 0.89790829 2.08430965 2.68693343 2.97507091
 0.95148187 2.6721492  2.65056456 0.78960282 2.47951904 0.29248079
 1.26962065 1.00804369 2.93563999 2.21600335], Cost: 0.0113
Current parameters: [2.95126169 2.88751676 0.92231831 2.07114654 2.7015391  3.00865757
 0.943601   2.6681479  2.62667602 0.79636741 2.44997894 0.2991603
 1.25384941 0.97886931 2.92681194 2.19978597], Cost: 0.0705
Current parameters: [2.95089093 2.90469755 0.90571832 2.07555776 2.70360021 2.97077943
 0.93670367 2.6704483  2.64967881 0.80458618 2.49171139 0.29683367
 1.2698527  0.98217081 2.92719108 2.21356182], Cost: 0.0091
Current parameters: [2.91883047 2.91243872 0.91022246 2.07529138 2.70187138 2.98977853
 0.91969532 2.65793193 2.63762127 0.77586374 2.52297586 0.31334243
 1.26685562 1.01085999 2.93853131 2.21311972], Cost: 0.0212
Current parameters: [2.94079099 2.92018514 0.89229808 2.07132267 2.69873535 2.97830253
 0.93412653 2.67866174 2.6463822  0.79027895 2.47098632 0.31690633
 1.27568294 0.99677645 2.92951542 2.21392778], Cost: 0.0103
Current parameters: [2.94219396 2.89384916 0.90551567 2.07612517 2.69417629 2.97551582
 0.92532743 2.67436344 2.64690613 0.79279676 2.47761436 0.30610364
 1.27224746 0.99930944 2.93344328 2.21249871], Cost: 0.0030
Current parameters: [2.93681816 2.88088399 0.90834958 2.07805277 2.6991114  2.96893014
 0.9337128  2.68564477 2.65140689 0.78879344 2.48331722 0.31766086
 1.28615963 0.99672046 2.92584444 2.21899044], Cost: 0.0096
Current parameters: [2.94205662 2.89420185 0.90452361 2.07391973 2.69252496 2.97830628
 0.92366862 2.66858877 2.64493737 0.79088692 2.47641622 0.30034969
 1.28415797 0.99770782 2.93134857 2.21330925], Cost: 0.0030
Current parameters: [2.93381472 2.8981461  0.91307849 2.07212504 2.70032292 2.95876951
 0.91917222 2.66690119 2.6489356  0.7939052  2.46829537 0.3010001
 1.26863738 1.00456333 2.93174967 2.22802795], Cost: 0.0084
Current parameters: [2.94727814 2.89763626 0.91339483 2.07252283 2.69248045 2.97817245
 0.92616746 2.68078142 2.64516484 0.79605878 2.47935688 0.30481948
 1.27525752 1.0050122  2.93563305 2.21600966], Cost: 0.0052
Current parameters: [2.95125991 2.8925632  0.89948371 2.08628597 2.70849036 2.97000344
```



```
 0.9218444  2.6693705  2.64608021 0.80077623 2.47723828 0.30813247
 1.27929749 1.01509359 2.93496757 2.20196431], Cost: 0.0025
Current parameters: [2.94630711 2.89240634 0.90412495 2.08897719 2.71021838 2.97413793
 0.92413941 2.66967514 2.63973686 0.80422045 2.47381693 0.30725323
 1.27731648 1.01620289 2.92565252 2.19898815], Cost: 0.0025
Current parameters: [2.96170827 2.88121144 0.90371473 2.0877407  2.71461744 2.98075949
 0.91969868 2.66369465 2.64149126 0.79512973 2.46229292 0.30936683
 1.27387181 1.00597144 2.94551102 2.19678583], Cost: 0.0070
Current parameters: [2.95019004 2.89547424 0.90373362 2.09286638 2.70665543 2.97143601
 0.92224341 2.66214556 2.64197468 0.79971454 2.4822762  0.31500026
 1.27997455 1.01473126 2.93819114 2.20568846], Cost: 0.0064
Current parameters: [2.94127629 2.89153319 0.89622386 2.0793469  2.71642897 2.97749507
 0.90907227 2.67236584 2.64619457 0.7906025  2.49136068 0.29987585
 1.27381923 1.02087827 2.93510447 2.19241541], Cost: 0.0035
Current parameters: [2.95257419 2.89029236 0.89753639 2.08749581 2.69964161 2.96814431
 0.91793358 2.66796744 2.6487869  0.80022445 2.47762198 0.31261716
 1.27141602 1.01349718 2.93054382 2.19739758], Cost: 0.0038
Current parameters: [2.96282568 2.89170037 0.90358839 2.06875096 2.7082542  2.96950131
 0.92567502 2.65921476 2.64994777 0.80046599 2.47762671 0.30725749
 1.28930047 1.02721879 2.92387407 2.1982916 ], Cost: 0.0176
Current parameters: [2.94490193 2.89440802 0.90519869 2.08427103 2.70731844 2.96801958
 0.92425571 2.66793124 2.65093163 0.80355138 2.47574262 0.30960671
 1.28153059 1.01341807 2.94031742 2.19311365], Cost: 0.0025
Current parameters: [2.9473518  2.89405686 0.89328905 2.09763653 2.70270508 2.97027995
 0.90817709 2.68165227 2.64545525 0.81362789 2.47284063 0.29736632
 1.28879234 1.01120078 2.93876816 2.20163304], Cost: 0.0021
Current parameters: [2.94560596 2.89312201 0.89088084 2.10382003 2.69896212 2.97030222
 0.91632272 2.68219844 2.64737229 0.80907138 2.47182315 0.28973835
 1.28625575 1.0156084  2.94107709 2.20081264], Cost: 0.0023
Current parameters: [2.94596883 2.89376751 0.89201408 2.09785171 2.7034234  2.97479703
 0.90835583 2.68039463 2.65008932 0.81599688 2.47231153 0.29837534
 1.28793294 1.012122   2.93848934 2.2038202 ], Cost: 0.0029
Current parameters: [2.94622244 2.90441701 0.88748318 2.09774868 2.70440124 2.97145678
 0.90897415 2.68566901 2.64500761 0.80854896 2.46738205 0.30171702
 1.28899994 1.01016919 2.93803138 2.19953316], Cost: 0.0016
Current parameters: [2.94882225 2.90660374 0.89018588 2.10027655 2.70637194 2.97117192
 0.90859382 2.68565299 2.64830456 0.80751138 2.4687806  0.29969538
 1.28885635 1.00731396 2.93573717 2.19901923], Cost: 0.0011

   Return from subroutine COBYLA because the MAXFUN limit has been reached.
```



```
   NFVALS =  100   F = 1.600140E-03    MAXCV = 0.000000E+00
   X = 2.946222E+00   2.904417E+00   8.874832E-01   2.097749E+00   2.704401E+00
       2.971457E+00   9.089742E-01   2.685669E+00   2.645008E+00   8.085490E-01
       2.467382E+00   3.017170E-01   1.289000E+00   1.010169E+00   2.938031E+00
       2.199533E+00
Current parameters: [2.94622244 2.90441701 0.88748318 2.09774868 2.70440124 2.97145678
 0.90897415 2.68566901 2.64500761 0.80854896 2.46738205 0.30171702
 1.28899994 1.01016919 2.93803138 2.19953316], Cost: 0.0016
Simulating for QAOA depth p = 9
Current parameters: [3.87949113 2.75901305 1.44762599 1.83620802 0.41381152 1.14513947
 0.82994606 2.25750172 0.21144248 1.47549137 1.22725106 0.17256377
 1.19778651 2.53406019 2.03546639 0.58541935 0.47120986 0.47911877], Cost: 2.0140
Current parameters: [3.87949113 3.75901305 1.44762599 1.83620802 0.41381152 1.14513947
 0.82994606 2.25750172 0.21144248 1.47549137 1.22725106 0.17256377
 1.19778651 2.53406019 2.03546639 0.58541935 0.47120986 0.47911877], Cost: 0.6069
Current parameters: [3.87949113 3.75901305 2.44762599 1.83620802 0.41381152 1.14513947
 0.82994606 2.25750172 0.21144248 1.47549137 1.22725106 0.17256377
 1.19778651 2.53406019 2.03546639 0.58541935 0.47120986 0.47911877], Cost: 2.8869
Current parameters: [3.87949113 3.75901305 1.44762599 2.83620802 0.41381152 1.14513947
 0.82994606 2.25750172 0.21144248 1.47549137 1.22725106 0.17256377
 1.19778651 2.53406019 2.03546639 0.58541935 0.47120986 0.47911877], Cost: 2.5519
Current parameters: [3.87949113 3.75901305 1.44762599 1.83620802 1.41381152 1.14513947
 0.82994606 2.25750172 0.21144248 1.47549137 1.22725106 0.17256377
 1.19778651 2.53406019 2.03546639 0.58541935 0.47120986 0.47911877], Cost: 1.2868
Current parameters: [3.87949113 3.75901305 1.44762599 1.83620802 0.41381152 2.14513947
 0.82994606 2.25750172 0.21144248 1.47549137 1.22725106 0.17256377
 1.19778651 2.53406019 2.03546639 0.58541935 0.47120986 0.47911877], Cost: 3.2784
Current parameters: [3.87949113 3.75901305 1.44762599 1.83620802 0.41381152 1.14513947
 1.82994606 2.25750172 0.21144248 1.47549137 1.22725106 0.17256377
 1.19778651 2.53406019 2.03546639 0.58541935 0.47120986 0.47911877], Cost: 2.2570
Current parameters: [3.87949113 3.75901305 1.44762599 1.83620802 0.41381152 1.14513947
 0.82994606 3.25750172 0.21144248 1.47549137 1.22725106 0.17256377
 1.19778651 2.53406019 2.03546639 0.58541935 0.47120986 0.47911877], Cost: 1.6541
Current parameters: [3.87949113 3.75901305 1.44762599 1.83620802 0.41381152 1.14513947
 0.82994606 2.25750172 1.21144248 1.47549137 1.22725106 0.17256377
 1.19778651 2.53406019 2.03546639 0.58541935 0.47120986 0.47911877], Cost: 1.8210
Current parameters: [3.87949113 3.75901305 1.44762599 1.83620802 0.41381152 1.14513947
 0.82994606 2.25750172 0.21144248 2.47549137 1.22725106 0.17256377
 1.19778651 2.53406019 2.03546639 0.58541935 0.47120986 0.47911877], Cost: 2.8518
Current parameters: [3.87949113 3.75901305 1.44762599 1.83620802 0.41381152 1.14513947
 0.82994606 2.25750172 0.21144248 1.47549137 2.22725106 0.17256377
```



```
 1.19778651 2.53406019 2.03546639 0.58541935 0.47120986 0.47911877], Cost: 3.7536
Current parameters: [3.87949113 3.75901305 1.44762599 1.83620802 0.41381152 1.14513947
 0.82994606 2.25750172 0.21144248 1.47549137 1.22725106 1.17256377
 1.19778651 2.53406019 2.03546639 0.58541935 0.47120986 0.47911877], Cost: 2.3897
Current parameters: [3.87949113 3.75901305 1.44762599 1.83620802 0.41381152 1.14513947
 0.82994606 2.25750172 0.21144248 1.47549137 1.22725106 0.17256377
 2.19778651 2.53406019 2.03546639 0.58541935 0.47120986 0.47911877], Cost: 2.4251
Current parameters: [3.87949113 3.75901305 1.44762599 1.83620802 0.41381152 1.14513947
 0.82994606 2.25750172 0.21144248 1.47549137 1.22725106 0.17256377
 1.19778651 3.53406019 2.03546639 0.58541935 0.47120986 0.47911877], Cost: 2.7808
Current parameters: [3.87949113 3.75901305 1.44762599 1.83620802 0.41381152 1.14513947
 0.82994606 2.25750172 0.21144248 1.47549137 1.22725106 0.17256377
 1.19778651 2.53406019 3.03546639 0.58541935 0.47120986 0.47911877], Cost: 3.1267
Current parameters: [3.87949113 3.75901305 1.44762599 1.83620802 0.41381152 1.14513947
 0.82994606 2.25750172 0.21144248 1.47549137 1.22725106 0.17256377
 1.19778651 2.53406019 2.03546639 1.58541935 0.47120986 0.47911877], Cost: 2.5335
Current parameters: [3.87949113 3.75901305 1.44762599 1.83620802 0.41381152 1.14513947
 0.82994606 2.25750172 0.21144248 1.47549137 1.22725106 0.17256377
 1.19778651 2.53406019 2.03546639 0.58541935 1.47120986 0.47911877], Cost: 0.7891
Current parameters: [3.87949113 3.75901305 1.44762599 1.83620802 0.41381152 1.14513947
 0.82994606 2.25750172 0.21144248 1.47549137 1.22725106 0.17256377
 1.19778651 2.53406019 2.03546639 0.58541935 0.47120986 1.47911877], Cost: 1.0602
Current parameters: [ 3.954755    3.93969316  1.15485974  1.58645778  0.32650547  0.80209708
  0.61806611  2.1230309   0.05553766  1.18723451  0.8231922  -0.05635893
  0.96431004  2.25491527  1.71191156  0.33802801  0.44781364  0.42090684], Cost: 2.6657
Current parameters: [3.91712307 3.8493531  1.30124287 1.7113329  0.37015849 0.97361828
 0.72400609 2.19026631 0.13349007 1.33136294 1.02522163 0.05810242
 1.08104827 2.39448773 1.87368898 0.46172368 0.45951175 0.45001281], Cost: 3.8379
Current parameters: [4.12161813 3.75901305 1.50987161 1.83620802 0.41381152 1.14513947
 0.82994606 2.25750172 0.21144248 1.47549137 1.22725106 0.17256377
 1.19778651 2.53406019 2.03546639 0.58541935 0.47120986 0.47911877], Cost: 0.9964
Current parameters: [3.74013723 3.77450143 1.9208105  1.81479876 0.40632741 1.11573295
 0.81178314 2.24597452 0.1980779  1.45078122 1.19261404 0.1529399
 1.17777228 2.51013114 2.0077304  0.5642123  0.46920427 0.47412869], Cost: 1.7093
Current parameters: [3.87542175 3.51025303 1.44280285 1.82978912 0.41156764 1.13632284
 0.82450047 2.25404565 0.20743552 1.4680828  1.21686623 0.16668016
 1.19178586 2.52688581 2.02715062 0.57906108 0.47060854 0.47762265], Cost: 0.7969
Current parameters: [3.87949113 3.75901305 1.44762599 1.87745698 0.29581354 1.14513947
 0.82994606 2.25750172 0.21144248 1.47549137 1.22725106 0.17256377
 1.19778651 2.53406019 2.03546639 0.58541935 0.47120986 0.47911877], Cost: 0.5239
Current parameters: [3.87332853 3.7595527  1.44032191 2.11203604 0.37429971 1.13178773
```

```
 0.82169936 2.25226791 0.20537442 1.46427196 1.21152446 0.16365374
 1.18869924 2.52319544 2.02287313 0.5757905  0.47029924 0.47685307], Cost: 1.2137
Current parameters: [3.87949113 3.75901305 1.44762599 1.87745698 0.29581354 1.21082626
 0.72359642 2.25750172 0.21144248 1.47549137 1.22725106 0.17256377
 1.19778651 2.53406019 2.03546639 0.58541935 0.47120986 0.47911877], Cost: 0.7149
Current parameters: [3.86962728 3.75987681 1.43593509 1.86616749 0.28623862 1.34607407
 0.96841755 2.24912449 0.20172995 1.45753361 1.20207909 0.15830239
 1.18324144 2.51667008 2.01530964 0.57000742 0.46975232 0.4754923 ], Cost: 1.0343
Current parameters: [3.87949113 3.75901305 1.44762599 1.87745698 0.29581354 1.14513947
 0.82994606 2.35215691 0.12980061 1.47549137 1.22725106 0.17256377
 1.19778651 2.53406019 2.03546639 0.58541935 0.47120986 0.47911877], Cost: 0.4397
Current parameters: [3.87430346 3.75946733 1.44147742 1.87151953 0.29077782 1.13999474
 0.83432303 2.51650549 0.31600801 1.46604688 1.21401242 0.16506331
 1.19013685 2.52491425 2.0248654  0.57731379 0.4704433  0.47721151], Cost: 0.3977
Current parameters: [3.87430346 3.75946733 1.44147742 1.87151953 0.29077782 1.13999474
 0.83432303 2.51650549 0.31600801 1.46604688 1.29214461 0.16506331
 1.19013685 2.52491425 1.92729306 0.57731379 0.4704433  0.47721151], Cost: 0.2969
Current parameters: [3.85880095 3.76082486 1.42310342 1.8537764  0.27572941 1.12462057
 0.8474029  2.53078027 0.31959611 1.43782358 1.48558862 0.14264943
 1.16727711 2.4975831  2.06921322 0.55309164 0.46815257 0.47151196], Cost: 0.3931
Current parameters: [3.87430346 3.75946733 1.44147742 1.87151953 0.29077782 1.13999474
 0.83432303 2.51650549 0.31600801 1.55300459 1.29214461 0.16506331
 1.19013685 2.43511805 1.92729306 0.57731379 0.4704433  0.47721151], Cost: 0.2814
Current parameters: [3.85890156 3.76081605 1.42322267 1.85389155 0.27582707 1.12472035
 0.84731801 2.53068763 0.31957282 1.72803624 1.31418974 0.14279489
 1.16742546 2.60246412 1.93204783 0.55324884 0.46816744 0.47154895], Cost: 0.8579
Current parameters: [3.87430346 3.75946733 1.44147742 1.87151953 0.29077782 1.13999474
 0.83432303 2.51650549 0.31600801 1.55300459 1.29214461 0.16506331
 1.09922886 2.43511805 1.92729306 0.66310853 0.4704433  0.47721151], Cost: 0.6832
Current parameters: [3.85991895 3.76072696 1.42442851 1.85505599 0.27681467 1.12572932
 0.84645961 2.52975081 0.31933734 1.55016776 1.31273352 0.14426587
 1.38362787 2.43036035 1.93173375 0.72771124 0.46831777 0.471923  ], Cost: 0.1635
Current parameters: [3.85991895 3.76072696 1.42442851 1.85505599 0.27681467 1.12572932
 0.84645961 2.52975081 0.31933734 1.55016776 1.31273352 0.17507131
 1.38362787 2.43036035 1.93173375 0.72771124 0.46831777 0.35077836], Cost: 0.3085
Current parameters: [3.84821676 3.7617517  1.41055875 1.84166245 0.26545525 1.114124
 0.85633306 2.54052625 0.32204584 1.54785992 1.32948317 0.37877825
 1.41605889 2.42648983 1.93534637 0.71763585 0.4665886  0.54291146], Cost: 0.1991
Current parameters: [3.86018394 3.76136309 1.42339776 1.85417669 0.27650729 1.12452156
 0.84571364 2.52927737 0.31878844 1.54915289 1.31131094 0.14345989
 1.38280586 2.42937756 1.9305946  0.72684024 0.34336734 0.47171805], Cost: 0.3906
```



```
Current parameters: [3.83999601 3.8005858  1.32307481 1.76389124 0.22483703 1.02096252
 0.83307058 2.53483277 0.29576349 1.48455039 1.28093895 0.10453416
 1.43377522 2.36189148 1.87676176 0.64701914 0.52559528 0.49601561], Cost: 0.2435
Current parameters: [3.83889248 3.76166999 1.50621865 1.84695149 0.22258417 1.07676102
 0.87324124 2.49232843 0.35023526 1.54870513 1.3106833  0.1431043
 1.3824432  2.42894395 1.93009201 0.72645596 0.46819906 0.47162763], Cost: 0.1194
Current parameters: [3.76530908 3.77791452 1.65697749 1.81116492 0.30055576 1.12091903
 0.82209608 2.58208342 0.27918956 1.52116744 1.31766866 0.13061145
 1.43837924 2.39854578 1.91333171 0.6843578  0.50664719 0.49435245], Cost: 0.1065
Current parameters: [3.66551759 3.77922858 1.62999167 1.87773004 0.31267645 1.10756647
 0.82621869 2.57289562 0.28465063 1.51855554 1.31400747 0.12853718
 1.4362637  2.39601645 1.91039998 0.68211618 0.5064352  0.49382499], Cost: 0.1406
Current parameters: [3.8889124  3.79189068 1.69668953 1.98021977 0.33024026 1.05618078
 0.83992186 2.59251946 0.28300072 1.49545932 1.33386697 0.12093909
 1.50720802 2.36937521 1.90068862 0.64113689 0.55117721 0.52157206], Cost: 0.7062
Current parameters: [3.765309   3.89123154 1.65697739 1.81457935 0.29230377 1.15915862
 0.85619489 2.57533142 0.28501314 1.52116728 1.31766844 0.13061133
 1.43837911 2.39854563 1.91333154 0.68435766 0.50664718 0.49435242], Cost: 0.1580
Current parameters: [3.73987292 3.67509027 1.64873479 1.78421255 0.25111919 1.26061853
 0.96922014 2.56772344 0.28720186 1.49087228 1.30931297 0.11356827
 1.47238554 2.36641654 1.88991428 0.64454132 0.5346299  0.50939971], Cost: 0.1639
Current parameters: [3.76530908 3.77791452 1.65697749 1.84458723 0.20494694 1.15326137
 0.76973249 2.61217298 0.25323675 1.52116744 1.31766866 0.13061145
 1.43837924 2.39854578 1.91333171 0.6843578  0.50664719 0.49435245], Cost: 0.1175
Current parameters: [3.75117926 3.77301348 1.66000477 1.77806335 0.31969511 1.20329832
 0.68557158 2.4535169  0.40129915 1.50989867 1.33266513 0.12799588
 1.4821018  2.38511303 1.91024071 0.66221422 0.53321324 0.51118333], Cost: 0.1314
Current parameters: [3.76920151 3.77389439 1.62958486 1.81681319 0.32051541 1.14433205
 0.81858922 2.60107834 0.27120865 1.58386072 1.33015587 0.09347936
 1.45453542 2.4592573  1.92333099 0.70147686 0.55236868 0.53349946], Cost: 0.2221
Current parameters: [3.70598455 3.7779666  1.70243938 1.72086638 0.22855995 1.04977865
 0.8376799  2.56321427 0.29972827 1.60718372 1.32032924 0.18063759
 1.46895553 2.47631467 1.88233917 0.56655384 0.45861708 0.43554517], Cost: 1.0964
Current parameters: [3.75272705 3.77217416 1.66467966 1.78666799 0.29948682 1.12451972
 0.81481874 2.58306369 0.27883151 1.48961191 1.31651215 0.13297453
 1.37927572 2.36515886 1.89545654 0.7710109  0.53636157 0.51395726], Cost: 0.6296
Current parameters: [3.76469527 3.77781081 1.65425379 1.81042775 0.30085903 1.12132699
 0.82255468 2.62341556 0.32462639 1.51926951 1.31412787 0.12633949
 1.43674007 2.39670784 1.91049639 0.68352443 0.49814526 0.49611599], Cost: 0.0452
Current parameters: [3.76997948 3.77318104 1.63988314 1.8130296  0.32156161 1.14520085
 0.81201801 2.62428232 0.30577009 1.51567327 1.31633376 0.09375884
```



```
 1.45595521 2.39239247 1.90727297 0.6932791  0.39322567 0.532388  ], Cost: 0.1698
Current parameters: [3.76125179 3.77811235 1.65017248 1.80648657 0.29751642 1.11791201
 0.82546004 2.62658634 0.32542339 1.51300043 1.37367139 0.12136082
 1.43166237 2.39063693 1.92132133 0.67814411 0.49763644 0.49484998], Cost: 0.1263
Current parameters: [3.75855043 3.7766687  1.65154029 1.80099273 0.29788214 1.11943419
 0.8234831  2.62941915 0.32781396 1.50573117 1.28225246 0.1209945
 1.44463562 2.38292824 2.02808251 0.67379555 0.50141821 0.50036674], Cost: 0.3839
Current parameters: [3.76552497 3.77744981 1.65247843 1.81143662 0.30301514 1.12375409
 0.82181731 2.62457395 0.32383808 1.56291054 1.31446777 0.12635112
 1.4374775  2.35286499 1.91100816 0.68539232 0.49767162 0.48857644], Cost: 0.0650
Current parameters: [3.77438977 3.77130352 1.63059671 1.82015924 0.33228425 1.15768856
 0.80992781 2.642227   0.3155011  1.5085487  1.3154884  0.12666649
 1.45969931 2.41247964 1.90681933 0.70670637 0.49491858 0.39377381], Cost: 0.1434
Current parameters: [3.76969141 3.76990488 1.67968179 1.83012617 0.31069492 1.14171191
 0.82459605 2.61908761 0.33002642 1.53239267 1.31742689 0.13930026
 1.39923921 2.41065172 1.92054081 0.67735747 0.4872628  0.49285303], Cost: 0.0464
Current parameters: [3.77580067 3.78425685 1.6029009  1.7994116  0.32283962 1.13049059
 0.79965138 2.63774437 0.30478772 1.50249987 1.29610836 0.09831602
 1.38032436 2.38102918 1.88965628 0.60930843 0.51389551 0.49705981], Cost: 0.1191
Current parameters: [3.76833702 3.77570773 1.63592723 1.80671442 0.31315466 1.12812103
 0.81848703 2.63110616 0.31354118 1.51770067 1.31836417 0.17946364
 1.44500152 2.39489729 1.91214327 0.68019674 0.49720965 0.51075244], Cost: 0.0165
Current parameters: [3.74402005 3.79881045 1.60555012 1.84189262 0.28263493 1.16629494
 0.81805827 2.68008545 0.38852684 1.52815886 1.31208943 0.18467857
 1.44786385 2.40667349 1.88508379 0.69063302 0.50806109 0.536194  ], Cost: 0.0694
Current parameters: [3.79317471 3.76924267 1.60161522 1.81822938 0.29746715 1.11273021
 0.83190779 2.60436781 0.32593536 1.52227039 1.31715972 0.18368884
 1.43371879 2.40024452 1.91670266 0.68799569 0.48790775 0.50428941], Cost: 0.0085
Current parameters: [3.85066136 3.76450403 1.63297108 1.89348836 0.31958596 1.09272495
 0.83782145 2.60358324 0.3296499  1.50042566 1.31826399 0.18607618
 1.47624125 2.38004767 1.89279274 0.6592893  0.50823619 0.51852734], Cost: 0.1940
Current parameters: [3.79204993 3.76661051 1.60427029 1.80773406 0.30391128 1.12970459
 0.79865544 2.5666515  0.35129497 1.51917779 1.32104149 0.18508012
 1.44563611 2.39668335 1.91641797 0.67961027 0.49289245 0.50611537], Cost: 0.0163
Current parameters: [3.77487651 3.76655042 1.62123539 1.78038472 0.26819807 1.074754
 0.84242905 2.60036245 0.33652424 1.56306082 1.30647461 0.21719118
 1.44952539 2.443275   1.87666268 0.63492254 0.4588845  0.47852499], Cost: 0.4156
Current parameters: [3.79698546 3.79620991 1.60208903 1.82668337 0.30758942 1.08062975
 0.79461092 2.61389477 0.32841092 1.53054706 1.3190981  0.18620264
 1.42389817 2.40886207 1.92186474 0.70076431 0.4824076  0.50263706], Cost: 0.0361
Current parameters: [3.79631867 3.74869735 1.61045491 1.79184763 0.30443519 1.11719278
```



```
 0.83220955 2.59194073 0.32110568 1.48702063 1.30421388 0.19844339
 1.38336683 2.36960526 1.87328529 0.76747132 0.51060535 0.52310443], Cost: 0.4403
Current parameters: [3.80167663 3.7595083  1.59756996 1.80920849 0.30610531 1.10861998
 0.83373446 2.60150972 0.32086182 1.54068651 1.31114914 0.15992981
 1.44815575 2.43423494 1.91198155 0.70073149 0.51382765 0.52307326], Cost: 0.0212
Current parameters: [3.8009114  3.76246352 1.56835516 1.84093936 0.33193377 1.16632401
 0.82217828 2.60937439 0.28853524 1.58316145 1.3661754  0.21131031
 1.4143621  2.39685583 1.94536879 0.67479872 0.48701468 0.51218865], Cost: 0.1181
Current parameters: [3.7959014  3.76706922 1.59565367 1.81878105 0.30558097 1.121904
 0.82799155 2.60479716 0.31977252 1.51766713 1.31247682 0.16404151
 1.4467181  2.40228437 1.90897064 0.69520451 0.43751352 0.52431797], Cost: 0.0168
Current parameters: [3.77303058 3.75307642 1.59646264 1.83063931 0.30203903 1.1037036
 0.82849653 2.60120098 0.32469663 1.52148459 1.31605898 0.18298923
 1.43371831 2.40018355 1.91581648 0.68783244 0.48830454 0.50430138], Cost: 0.0130
Current parameters: [3.77098412 3.81061483 1.59779446 1.82988102 0.30727042 1.11684365
 0.85264073 2.57687084 0.31428914 1.51971306 1.31247537 0.18129371
 1.43758281 2.40445306 1.91161284 0.69029726 0.49203694 0.50573851], Cost: 0.0286
Current parameters: [3.7918125  3.76852892 1.60899641 1.82606789 0.2762559  1.11724373
 0.8249706  2.60017515 0.31514117 1.52446359 1.31931879 0.18768442
 1.44172556 2.40319833 1.91889072 0.69812053 0.48754493 0.50403462], Cost: 0.0161
Current parameters: [3.79682166 3.77375623 1.58145355 1.81998307 0.28117931 1.11944413
 0.81154954 2.60624321 0.30346462 1.51001881 1.30225274 0.16504356
 1.41498823 2.3994052  1.902079   0.65913002 0.49783283 0.50784459], Cost: 0.0186
Current parameters: [3.79548447 3.76827248 1.59614257 1.82040886 0.30395576 1.12005678
 0.82893159 2.60807343 0.3238884  1.51837928 1.31695694 0.18113318
 1.44012759 2.40628723 1.90776742 0.69455107 0.48755438 0.47998326], Cost: 0.0138
Current parameters: [3.79096513 3.77061794 1.59262388 1.8173574  0.29746959 1.11897858
 0.83227505 2.61174191 0.32961869 1.5090541  1.29077279 0.17535704
 1.44545992 2.40155719 1.96745305 0.68603141 0.48964334 0.49733659], Cost: 0.0468
Current parameters: [3.79935026 3.76281224 1.60932313 1.80864873 0.30564294 1.10382053
 0.83168961 2.59192031 0.30713217 1.51863049 1.316858   0.1841446
 1.4310809  2.39649478 1.92165686 0.68850061 0.48596806 0.49900055], Cost: 0.0206
Current parameters: [3.79155714 3.76339458 1.59624077 1.80993584 0.2998674  1.12652743
 0.83193043 2.60688274 0.33235299 1.50416261 1.30537681 0.18989457
 1.40825254 2.38435543 1.90509761 0.72882169 0.49881215 0.5184863 ], Cost: 0.1218
Current parameters: [3.79517004 3.76773874 1.59281574 1.82381241 0.30612995 1.12659226
 0.82956415 2.60627994 0.31739496 1.53727009 1.3288345  0.19036745
 1.4288378  2.39930054 1.92474593 0.68466515 0.48762109 0.50672895], Cost: 0.0188
Current parameters: [3.80020407 3.76888576 1.60401711 1.82814775 0.30123614 1.11240997
 0.83207609 2.60453893 0.32579164 1.51789658 1.31795634 0.18214925
 1.4374734  2.39593131 1.91614134 0.68755298 0.49152398 0.50705489], Cost: 0.0170
```

```
Current parameters: [3.79526245 3.76694344 1.60604218 1.81718709 0.29379761 1.10779521
 0.83752038 2.6060501  0.33036577 1.53141782 1.31011804 0.19646668
 1.44123783 2.40786545 1.90829782 0.66994489 0.48154018 0.5017821 ], Cost: 0.0302
Current parameters: [3.79252211 3.77065365 1.59430747 1.81380964 0.29677901 1.10933505
 0.83105483 2.60617358 0.32366628 1.52354504 1.3177322  0.18609144
 1.44233543 2.39281527 1.91496874 0.6890645  0.49003022 0.50564222], Cost: 0.0082
Current parameters: [3.78952472 3.77135017 1.59180511 1.80866458 0.29048256 1.10429446
 0.83528555 2.60812741 0.32687952 1.5141366  1.34202928 0.17783582
 1.44453984 2.39839659 1.91783268 0.68248205 0.48907987 0.50430098], Cost: 0.0344
Current parameters: [3.79166334 3.7712578  1.59788689 1.81278593 0.29374176 1.10893225
 0.8335301  2.60573814 0.32798452 1.53106599 1.31726257 0.1773514
 1.43799701 2.38745555 1.91537256 0.6892679  0.48910927 0.50202941], Cost: 0.0089
Current parameters: [3.79396769 3.76793407 1.59066611 1.82278981 0.3007833  1.10702295
 0.82411002 2.60355402 0.31251451 1.53283769 1.31620988 0.18248356
 1.46156936 2.3806221  1.91091293 0.68563674 0.49255343 0.50146271], Cost: 0.0186
Current parameters: [3.7933811  3.77750558 1.59451302 1.81552722 0.29884971 1.10110987
 0.82217715 2.60890815 0.3250095  1.52635193 1.31823265 0.18689493
 1.43964846 2.3953172  1.9163087  0.69134525 0.4880934  0.50499263], Cost: 0.0126
Current parameters: [3.7925223  3.77102777 1.59368796 1.81372069 0.29771033 1.1105006
 0.83103868 2.60601853 0.32323108 1.5230144  1.31711926 0.18355679
 1.44344336 2.39341164 1.91405196 0.689924   0.48359322 0.50816906], Cost: 0.0077
Current parameters: [3.78675918 3.78165277 1.59301269 1.8162363  0.29963529 1.11115532
 0.83667521 2.59938415 0.32116018 1.52297448 1.31606682 0.18343491
 1.44365721 2.39483809 1.91303468 0.68977884 0.48525099 0.50798072], Cost: 0.0093
Current parameters: [3.79576123 3.77000211 1.58922851 1.81543547 0.29589823 1.10855442
 0.83247288 2.6025783  0.32437126 1.52356382 1.3169699  0.18415078
 1.44258032 2.39383476 1.91456226 0.69089574 0.48267069 0.50730579], Cost: 0.0079
Current parameters: [3.79094785 3.76949466 1.59153295 1.81088532 0.29813506 1.11379125
 0.83139605 2.60681558 0.32450108 1.52204497 1.31312101 0.18728005
 1.43908314 2.3881496  1.90747865 0.69884682 0.48489307 0.51152161], Cost: 0.0059
Current parameters: [3.78996869 3.7705408  1.5916701  1.81223359 0.29715708 1.11440464
 0.83097188 2.60721434 0.32487699 1.51947622 1.31329574 0.19008599
 1.43801676 2.38380163 1.90912709 0.69721335 0.48190829 0.50897334], Cost: 0.0058
Current parameters: [3.78844517 3.77030524 1.58839087 1.8098773  0.29728372 1.11718242
 0.83170377 2.60971678 0.32682327 1.51776047 1.30412706 0.18828396
 1.44016901 2.3844203  1.91922226 0.69998582 0.48338076 0.50929378], Cost: 0.0066
Current parameters: [3.78974148 3.76974468 1.59420097 1.8122884  0.29922315 1.1134057
 0.83346195 2.60688397 0.32743491 1.5211839  1.31503867 0.19282309
 1.4409279  2.3838488  1.91065674 0.70031234 0.48064541 0.50837229], Cost: 0.0065
Current parameters: [3.79017714 3.76961892 1.59303261 1.80975524 0.29460275 1.11434257
 0.83390488 2.60893361 0.32780711 1.52333141 1.30532304 0.19598382
```



```
 1.43792062 2.38702464 1.90393631 0.6901713  0.479042   0.50944026], Cost: 0.0063
Current parameters: [3.79045611 3.77045531 1.59153282 1.81335327 0.29682903 1.11190001
 0.83490962 2.61124958 0.32066783 1.51966794 1.31291067 0.19000126
 1.43679921 2.38381543 1.90934449 0.69836191 0.48134225 0.50845448], Cost: 0.0055
Current parameters: [3.79262163 3.7676359  1.59590668 1.80539461 0.30048132 1.10894828
 0.83446245 2.60397986 0.31424632 1.51818244 1.31018896 0.19068398
 1.43327785 2.38280477 1.91127798 0.69826829 0.47971571 0.5067811 ], Cost: 0.0086
Current parameters: [3.79006617 3.77057435 1.5930068  1.81278653 0.29461389 1.10817017
 0.8353542  2.61099726 0.32234514 1.51536837 1.31153214 0.18810882
 1.43937307 2.38347085 1.90727263 0.6991716  0.48186521 0.50771373], Cost: 0.0063
Current parameters: [3.78682543 3.76892278 1.5849875  1.80634064 0.29355658 1.11521069
 0.83899628 2.61537504 0.32239269 1.51765469 1.3207017  0.19009473
 1.43493881 2.38427556 1.90852786 0.69691096 0.48060004 0.51099795], Cost: 0.0014
Current parameters: [3.78459234 3.76562521 1.57756496 1.80518642 0.29298952 1.11827438
 0.83907373 2.61790197 0.31829057 1.5212221  1.32272206 0.18950684
 1.44242633 2.37747508 1.90459612 0.69524913 0.48143216 0.51174039], Cost: 0.0009
Current parameters: [3.78404504 3.76558887 1.5789684  1.80587395 0.2901604  1.11650085
 0.83913044 2.61680405 0.31775545 1.52206437 1.3230095  0.19045262
 1.44240572 2.37547939 1.90666414 0.69453729 0.48189203 0.51765503], Cost: 0.0009
Current parameters: [3.78222188 3.7652403  1.58108407 1.80551596 0.27932715 1.12208392
 0.83704828 2.6162601  0.31349252 1.52342692 1.32458534 0.19226792
 1.44350683 2.379792   1.90658306 0.69963844 0.48012855 0.51479616], Cost: 0.0022

   Return from subroutine COBYLA because the MAXFUN limit has been reached.

   NFVALS =  100   F = 8.750148E-04    MAXCV = 0.000000E+00
   X = 3.784045E+00   3.765589E+00   1.578968E+00   1.805874E+00   2.901604E-01
       1.116501E+00   8.391304E-01   2.616804E+00   3.177555E-01   1.522064E+00
       1.323010E+00   1.904526E-01   1.442406E+00   2.375479E+00   1.906664E+00
       6.945373E-01   4.818920E-01   5.176550E-01
Current parameters: [3.78404504 3.76558887 1.5789684  1.80587395 0.2901604  1.11650085
 0.83913044 2.61680405 0.31775545 1.52206437 1.3230095  0.19045262
 1.44240572 2.37547939 1.90666414 0.69453729 0.48189203 0.51765503], Cost: 0.0009
Simulating for QAOA depth p = 10
Current parameters: [1.69802129 1.26811247 0.29069385 1.14340494 0.89032784 1.57098202
 0.1323546  0.11674599 1.2411198  0.0123113  2.86249523 2.25211452
 1.20792848 1.23622304 0.05870297 0.96564525 0.4890801  0.99834707
 1.95681818 2.77097386], Cost: 2.4454
Current parameters: [1.69802129 2.26811247 0.29069385 1.14340494 0.89032784 1.57098202
 0.1323546  0.11674599 1.2411198  0.0123113  2.86249523 2.25211452
 1.20792848 1.23622304 0.05870297 0.96564525 0.4890801  0.99834707
```



```
 1.95681818 2.77097386], Cost: 2.9960
Current parameters: [1.69802129 1.26811247 1.29069385 1.14340494 0.89032784 1.57098202
 0.1323546  0.11674599 1.2411198  0.0123113  2.86249523 2.25211452
 1.20792848 1.23622304 0.05870297 0.96564525 0.4890801  0.99834707
 1.95681818 2.77097386], Cost: 1.3667
Current parameters: [1.69802129 1.26811247 1.29069385 2.14340494 0.89032784 1.57098202
 0.1323546  0.11674599 1.2411198  0.0123113  2.86249523 2.25211452
 1.20792848 1.23622304 0.05870297 0.96564525 0.4890801  0.99834707
 1.95681818 2.77097386], Cost: 2.4963
Current parameters: [1.69802129 1.26811247 1.29069385 1.14340494 1.89032784 1.57098202
 0.1323546  0.11674599 1.2411198  0.0123113  2.86249523 2.25211452
 1.20792848 1.23622304 0.05870297 0.96564525 0.4890801  0.99834707
 1.95681818 2.77097386], Cost: 1.5640
Current parameters: [1.69802129 1.26811247 1.29069385 1.14340494 0.89032784 2.57098202
 0.1323546  0.11674599 1.2411198  0.0123113  2.86249523 2.25211452
 1.20792848 1.23622304 0.05870297 0.96564525 0.4890801  0.99834707
 1.95681818 2.77097386], Cost: 1.5002
Current parameters: [1.69802129 1.26811247 1.29069385 1.14340494 0.89032784 1.57098202
 1.1323546  0.11674599 1.2411198  0.0123113  2.86249523 2.25211452
 1.20792848 1.23622304 0.05870297 0.96564525 0.4890801  0.99834707
 1.95681818 2.77097386], Cost: 1.6532
Current parameters: [1.69802129 1.26811247 1.29069385 1.14340494 0.89032784 1.57098202
 0.1323546  1.11674599 1.2411198  0.0123113  2.86249523 2.25211452
 1.20792848 1.23622304 0.05870297 0.96564525 0.4890801  0.99834707
 1.95681818 2.77097386], Cost: 1.5075
Current parameters: [1.69802129 1.26811247 1.29069385 1.14340494 0.89032784 1.57098202
 0.1323546  0.11674599 2.2411198  0.0123113  2.86249523 2.25211452
 1.20792848 1.23622304 0.05870297 0.96564525 0.4890801  0.99834707
 1.95681818 2.77097386], Cost: 1.3071
Current parameters: [1.69802129 1.26811247 1.29069385 1.14340494 0.89032784 1.57098202
 0.1323546  0.11674599 2.2411198  1.0123113  2.86249523 2.25211452
 1.20792848 1.23622304 0.05870297 0.96564525 0.4890801  0.99834707
 1.95681818 2.77097386], Cost: 1.0457
Current parameters: [1.69802129 1.26811247 1.29069385 1.14340494 0.89032784 1.57098202
 0.1323546  0.11674599 2.2411198  1.0123113  3.86249523 2.25211452
 1.20792848 1.23622304 0.05870297 0.96564525 0.4890801  0.99834707
 1.95681818 2.77097386], Cost: 2.4408
Current parameters: [1.69802129 1.26811247 1.29069385 1.14340494 0.89032784 1.57098202
 0.1323546  0.11674599 2.2411198  1.0123113  2.86249523 3.25211452
 1.20792848 1.23622304 0.05870297 0.96564525 0.4890801  0.99834707
 1.95681818 2.77097386], Cost: 2.4879
```



```
Current parameters: [1.69802129 1.26811247 1.29069385 1.14340494 0.89032784 1.57098202
 0.1323546  0.11674599 2.2411198  1.0123113  2.86249523 2.25211452
 2.20792848 1.23622304 0.05870297 0.96564525 0.4890801  0.99834707
 1.95681818 2.77097386], Cost: 3.2609
Current parameters: [1.69802129 1.26811247 1.29069385 1.14340494 0.89032784 1.57098202
 0.1323546  0.11674599 2.2411198  1.0123113  2.86249523 2.25211452
 1.20792848 2.23622304 0.05870297 0.96564525 0.4890801  0.99834707
 1.95681818 2.77097386], Cost: 1.1543
Current parameters: [1.69802129 1.26811247 1.29069385 1.14340494 0.89032784 1.57098202
 0.1323546  0.11674599 2.2411198  1.0123113  2.86249523 2.25211452
 1.20792848 1.23622304 1.05870297 0.96564525 0.4890801  0.99834707
 1.95681818 2.77097386], Cost: 1.7495
Current parameters: [1.69802129 1.26811247 1.29069385 1.14340494 0.89032784 1.57098202
 0.1323546  0.11674599 2.2411198  1.0123113  2.86249523 2.25211452
 1.20792848 1.23622304 0.05870297 1.96564525 0.4890801  0.99834707
 1.95681818 2.77097386], Cost: 2.7225
Current parameters: [1.69802129 1.26811247 1.29069385 1.14340494 0.89032784 1.57098202
 0.1323546  0.11674599 2.2411198  1.0123113  2.86249523 2.25211452
 1.20792848 1.23622304 0.05870297 0.96564525 1.4890801  0.99834707
 1.95681818 2.77097386], Cost: 2.8737
Current parameters: [1.69802129 1.26811247 1.29069385 1.14340494 0.89032784 1.57098202
 0.1323546  0.11674599 2.2411198  1.0123113  2.86249523 2.25211452
 1.20792848 1.23622304 0.05870297 0.96564525 0.4890801  1.99834707
 1.95681818 2.77097386], Cost: 2.8866
Current parameters: [1.69802129 1.26811247 1.29069385 1.14340494 0.89032784 1.57098202
 0.1323546  0.11674599 2.2411198  1.0123113  2.86249523 2.25211452
 1.20792848 1.23622304 0.05870297 0.96564525 0.4890801  0.99834707
 2.95681818 2.77097386], Cost: 1.6100
Current parameters: [1.69802129 1.26811247 1.29069385 1.14340494 0.89032784 1.57098202
 0.1323546  0.11674599 2.2411198  1.0123113  2.86249523 2.25211452
 1.20792848 1.23622304 0.05870297 0.96564525 0.4890801  0.99834707
 1.95681818 3.77097386], Cost: 0.4006
Current parameters: [ 1.75276386  1.15269849  1.51682535  0.90661522  0.8489527   1.54298532
  0.07229739  0.08722579  2.25359515  1.06710738  2.57006825  1.94980079
  0.74358405  1.21346839 -0.08881261  0.61416893  0.10589807  0.61246778
  1.83853977  3.90620543], Cost: 1.1787
Current parameters: [2.18948399 1.26811247 1.29069385 1.14340494 0.89032784 1.57098202
 0.1323546  0.11674599 2.2411198  1.0123113  2.95449745 2.25211452
 1.20792848 1.23622304 0.05870297 0.96564525 0.4890801  0.99834707
 1.95681818 3.77097386], Cost: 1.2747
Current parameters: [1.42502348 1.23736806 1.35093162 1.08032799 0.87930617 1.56352415
```

```
 0.11635633 0.10888229 2.24444303 1.02690809 3.79028431 2.17158304
 1.08423464 1.23016157 0.0194072  0.87201764 0.38700661 0.89555507
 1.92531073 3.80699736], Cost: 1.7157
Current parameters: [1.69802129 1.76069573 1.29069385 1.14340494 0.89032784 1.57098202
 0.1323546  0.11674599 2.2411198  1.03944381 2.86249523 2.22498201
 1.18079597 1.20909052 0.03157045 0.93851274 0.46194759 0.97121456
 1.92968567 3.74384135], Cost: 2.5969
Current parameters: [1.50351072 0.81497361 1.4312862  0.99618608 0.86460374 1.55357568
 0.09501533 0.09839245 2.24887608 0.68379782 2.66331809 2.42673928
 1.28181417 1.58465758 0.32957012 1.10970424 0.61342675 1.12101676
 2.2458629  4.2176329 ], Cost: 2.4603
Current parameters: [1.67046137 1.17492601 0.80829027 1.12254572 0.88668303 1.56851574
 0.12706405 0.1141455  2.24221878 1.0183805  2.83427411 2.22424106
 1.16578144 1.23297642 0.04446594 0.93344096 0.4540828  0.96311216
 1.94515671 3.78164453], Cost: 0.3684
Current parameters: [ 1.67046137  1.17492601  0.80829027  1.1840074   0.88668303  1.56851574
 -0.11526313  0.1141455   2.24221878  1.0183805   2.83427411  2.22424106
  1.16578144  1.23297642  0.04446594  0.93344096  0.4540828   0.96311216
  1.94515671  3.78164453], Cost: 0.1406
Current parameters: [ 1.6403352   1.07306245  0.82370297  1.652707    0.88269883  1.56581981
 -0.01536075  0.11130287  2.24342008  1.02501484  2.80342517  2.19377213
  1.11970986  1.2294275   0.02890323  0.89823795  0.41582671  0.92459634
  1.93240937  3.79330881], Cost: 0.3831
Current parameters: [ 1.67046137  1.17492601  0.80829027  1.1840074   0.63959737  1.56851574
 -0.11526313  0.1141455   2.24221878  1.02239254  2.83427411  2.22022902
  1.20188974  1.22896439  0.04045391  0.92942893  0.45007077  0.95910013
  1.94114468  3.7776325 ], Cost: 0.5591
Current parameters: [ 1.63733348  1.06291294  0.82523866  1.15862588  0.99537432  1.56555119
 -0.14256561  0.11101964  2.24353978  1.0773256   2.80035143  2.13908653
  1.57996695  1.17742416 -0.02429714  0.84308066  0.36036521  0.86910896
  1.87948952  3.74282128], Cost: 1.8190
Current parameters: [ 1.67046137  1.17492601  0.80829027  1.1840074   0.88668303  1.56851574
 -0.11526313 -0.1335744   2.24221878  1.02235061  2.83427411  2.22027095
  1.16578144  1.22900631  0.04049583  0.92947085  0.45011269  0.99487305
  1.9411866   3.77767442], Cost: 0.4157
Current parameters: [ 1.64724637  1.09643074  0.82016717  1.16622082  0.90173929  1.56643828
 -0.13439585  0.19601374  2.24314449  1.07574551  2.81050215  2.14850931
  1.07966936  1.17798901 -0.0197792   0.85406116  0.37235032  1.40206293
  1.88308108  3.73838029], Cost: 0.3087
Current parameters: [ 1.67046137  1.17492601  0.80829027  1.1840074   0.88668303  1.32015521
 -0.11526313  0.1141455   2.24221878  1.0222002   2.83427411  2.22042135
```



```
  1.16578144  1.22915672  0.04064624  0.92962126  0.48082071  0.96311216
  1.94133701  3.77782483], Cost: 0.4855
Current parameters: [ 1.64504776  1.08899671  0.821292    1.16453631  0.90316522  1.64639018
 -0.13620785  0.12893207  2.24323217  1.08192802  2.80825079  2.14058734
  1.07151397  1.17203168 -0.0266133   0.8457937   0.88868287  0.92480896
  1.87645244  3.73353322], Cost: 0.5275
Current parameters: [ 1.67046137  1.17492601  0.80829027  1.1840074   0.88668303  1.56851574
 -0.11526313  0.1141455   2.49189959  1.0203292   2.83427411  2.22229236
  1.16578144  1.23102773  0.04251725  0.94513313  0.4540828   0.96311216
  1.94320801  3.77969583], Cost: 0.3713
Current parameters: [ 1.64582009  1.09160815  0.82089687  1.16512805  0.90266432  1.58677396
 -0.13557133  0.1284827   2.2029048   1.08991452  2.80904165  2.13321188
  1.07437881  1.16396607 -0.03437092  1.3732663   0.41017267  0.92597301
  1.86862265  3.7250776 ], Cost: 0.5861
Current parameters: [ 1.67046137  1.17492601  0.80829027  1.1840074   0.88668303  1.56851574
 -0.11526313  0.1141455   2.24221878  0.98866079  2.83427411  2.32589302
  1.16578144  1.26269613 -0.17606486  0.93344096  0.4540828   0.96311216
  1.97487642  3.81136424], Cost: 1.1216
Current parameters: [ 1.64532075  1.08991977  0.82115233  1.16474547  0.90298817  1.58714395
 -0.13598286  0.12877323  2.22882502  1.1469836   2.80853033  2.58761433
  1.0725266   1.10694813  0.21928526  0.89315618  0.40928287  0.92522041
  1.81145225  3.66831182], Cost: 0.7547
Current parameters: [ 1.67046137  1.17492601  0.80829027  1.1840074   0.88668303  1.56851574
 -0.11526313  0.1141455   2.24221878  1.22450962  2.83427411  2.22424106
  1.16578144  1.27046723  0.04446594  0.93344096  0.4540828   0.96311216
  2.07630421  3.81913533], Cost: 0.9075
Current parameters: [ 1.65719653  1.13007452  0.81507662  1.17384431  0.89528604  1.57834447
 -0.12619539  0.12186347  2.23515188  0.8587071   2.82069103  2.18749146
  1.11657777  0.99912861  0.07103213  0.91218567  0.4304452   0.94311949
  2.27593486  3.55449523], Cost: 0.4276
Current parameters: [ 1.67046137  1.16855503  0.80962921  1.18260535  0.88643804  1.56834997
 -0.11561873  0.11397071  2.24229264  1.02903065  2.83427411  2.21212534
  1.15270633  1.39777781  0.0332668   0.92103415  0.44148826  0.95050165
  1.93413068  3.59685774], Cost: 0.3802
Current parameters: [ 1.60909192  1.04809021  0.82273367  1.15474069  0.92958662  1.61608707
 -0.16133839  0.15206567  2.20858868  0.76825285  2.77143232  2.20762755
  1.10369721  1.41653942  0.30917581  0.99219727  0.50419444  1.03028922
  1.99338981  3.96350427], Cost: 2.8232
Current parameters: [ 1.6383389   1.19655912  0.76590436  1.22839107  0.89443835  1.57376342
 -0.10400605  0.11967875  2.23988041  1.00810957  3.00586774  2.28090649
  1.25281778  1.23724153  0.07211614  0.99932139  0.52590612  1.03544106
```



```
  1.96732672  3.75629684], Cost: 0.6241
Current parameters: [ 1.59205013  1.08781027  0.86959406  1.11665632  0.88563625  1.57194728
 -0.14097605  0.11503077  2.2379137   1.02952889  3.16590409  2.08996107
  0.97973305  1.19715241  0.01275987  0.80336342  0.31393184  0.82607895
  1.87262313  3.82210065], Cost: 0.2435
Current parameters: [ 1.63080577  1.6011685   0.74832334  1.23747313  0.92769485  1.60849477
 -0.1271954   0.14822738  2.21620447  1.02377609  2.78844614  2.11898357
  1.0803281   1.1205401   0.07718178  0.89300132  0.41768519  0.93879882
  1.82611202  3.72611736], Cost: 1.7122
Current parameters: [ 1.91372965  1.15902711  0.79643507  1.19457734  0.89479089  1.57641942
 -0.11762208  0.12088334  2.23707586  1.01392988  2.87222175  2.20894942
  1.154405    1.21626556  0.05645102  0.93096353  0.45240446  0.96382283
  1.9271394   3.77618436], Cost: 0.3494
Current parameters: [ 1.66327397  1.15062375  0.6880038   1.17830827  0.8856872   1.56784191
 -0.1167086   0.113435    2.24219596  1.02081266  2.82691427  2.21612252
  1.1545109   1.23128037  0.03990368  0.93080058  0.4447657   0.95373015
  1.94126613  3.78357799], Cost: 0.0868
Current parameters: [ 1.65130583  1.10222879  0.70774939  1.16758887  0.88841167  1.57146061
 -0.1231282   0.1160787   2.22660302  1.05356052  2.81001108  2.16028562
  1.11850635  1.18410552  0.00946997  1.14424819  0.42221724  0.93272545
  1.88981606  3.75014881], Cost: 0.2275
Current parameters: [ 1.64596276  1.25144118  0.71560541  1.18659067  0.90628654  1.587905
 -0.13559043  0.13343733  2.22150051  1.01355627  2.8036182   2.19820285
  1.14871113  1.21022422  0.05077911  0.93184394  0.43594648  0.94415852
  1.91749809  3.79205084], Cost: 0.1892
Current parameters: [ 1.69542021  1.20579067  0.5190084   1.20097195  0.85278406  1.53589315
 -0.06135986  0.07579187  2.28980864  1.05336326  2.86539204  2.17288418
  1.08803973  1.19303942  0.0322354   0.8946589   0.43863387  0.96237685
  1.90579201  3.69713076], Cost: 0.3888
Current parameters: [ 1.65766351  1.11892373  0.68297924  1.1703427   0.90917682  1.57292847
 -0.12055932  0.11705187  2.24078047  1.03149361  2.81552437  2.18130923
  1.24481333  1.20083777  0.03603919  0.89929246  0.42265387  0.93470973
  1.90789875  3.76399383], Cost: 0.2770
Current parameters: [ 1.65852749  1.14207645  0.69504957  1.17571264  0.88696094  1.57013109
 -0.12013048  0.12407395  2.239664    1.02488813  2.82088655  2.20557742
  1.14441624  1.22443776  0.03512347  0.92138282  0.43431459  1.00848246
  1.93229565  3.7815304 ], Cost: 0.0669
Current parameters: [ 1.65167591  1.10757804  0.67306714  1.16489282  0.89592524  1.57893514
 -0.12670701  0.12868639  2.23703991  0.96295099  2.80014676  2.18157726
  1.11376412  1.26412722  0.10382318  0.92450528  0.43528073  1.01396354
  1.93203844  3.81948742], Cost: 0.2281
```



```
Current parameters: [ 1.66036273  1.14325193  0.69078024  1.17672626  0.88537761  1.56847039
 -0.11791585  0.12229873  2.24149024  1.04754717  2.82359271  2.22203987
  1.14679612  1.24859623  0.03756223  0.92239755  0.43629682  1.00952821
  1.88732642  3.80275056], Cost: 0.0715
Current parameters: [ 1.64885404  1.11917548  0.705244    1.16788132  0.89295286  1.57716926
 -0.12922626  0.13059708  2.23347728  1.03158059  2.80511311  2.2881699
  1.1147016   1.17419306  0.07983318  0.90831395  0.41885662  0.99805081
  1.91724303  3.74511591], Cost: 0.0954
Current parameters: [ 1.64629665  1.13329836  0.70937395  1.16682481  0.88835508  1.57222467
 -0.12585853  0.1263026   2.23682273  1.02657774  2.86081995  2.19022462
  1.12205398  1.21928334  0.03090104  0.90560653  0.41620693  0.9917851
  1.92473223  3.78879331], Cost: 0.0621
Current parameters: [ 1.64569324  1.13058039  0.70701706  1.16177039  0.89962419  1.58118787
 -0.13387905  0.13431524  2.23048103  1.12334504  2.85008131  2.17954568
  1.11192681  1.23185852  0.06105182  0.90266967  0.40541311  0.99159783
  1.98627937  3.81189778], Cost: 0.1663
Current parameters: [ 1.6512604   1.14276822  0.70226859  1.16967618  0.88727141  1.56300839
 -0.12247758  0.12424914  2.23924481  1.0177073   2.86592034  2.20436631
  1.13367895  1.2294295   0.03119952  0.91690791  0.36310738  0.99677742
  1.93406301  3.79364704], Cost: 0.1360
Current parameters: [ 1.64380329  1.11646271  0.69976224  1.1551898   0.90807876  1.59371926
 -0.14004259  0.13994749  2.22644554  1.01444097  2.84042761  2.22794889
  1.09038238  1.22460514 -0.06530193  0.88970725  0.43605161  0.9870601
  1.93819441  3.81406461], Cost: 0.1916
Current parameters: [ 1.65123741  1.14501764  0.70373318  1.11010194  0.8871258   1.55964173
 -0.13843313  0.12493377  2.23841206  1.02607974  2.86589121  2.19365476
  1.1280722   1.22049174  0.03260237  0.91002014  0.42236622  0.9968536
  1.92585991  3.78560731], Cost: 0.0616
Current parameters: [ 1.64252204  1.14014644  0.72208664  1.1296378   0.90132586  1.4438026
 -0.14611829  0.13756852  2.22645368  1.02727392  2.8522808   2.19033765
  1.11607968  1.21182697  0.02883377  0.90118846  0.43564825  0.99203742
  1.92537382  3.79638458], Cost: 0.0938
Current parameters: [ 1.65178723  1.14370891  0.70232049  1.12165241  0.88634638  1.55964723
 -0.18451063  0.12416528  2.23917342  1.0278225   2.86645939  2.19169184
  1.12616768  1.24714593  0.03082247  0.90818671  0.42040631  0.99501015
  1.92400784  3.755517  ], Cost: 0.0319
Current parameters: [ 1.6395374   1.14489904  0.73660144  1.10035478  0.90527248  1.55870066
 -0.11632485  0.14218369  2.2214185   1.03117921  2.85110815  2.18687057
  1.11219335  1.30737777  0.0252274   0.8976124   0.41586135  0.98541886
  1.92176615  3.69353044], Cost: 0.1298
Current parameters: [ 1.65340775  1.14289875  0.69751644  1.12195654  0.88416635  1.55974273
```

```
 -0.18447859  0.08921316  2.1879836   1.02797375  2.86813401  2.19159266
  1.12680794  1.24677571  0.03082432  0.9056911   0.42012827  0.99980982
  1.92371421  3.75510147], Cost: 0.0193
Current parameters: [ 1.63840171  1.14605379  0.73795108  1.11809738  0.9084488   1.55858963
 -0.18778548 -0.00553567  2.24656448  1.02997346  2.84827412  2.18821434
  1.11356078  1.24697005  0.02597197  0.8991337   0.41880442  1.00832657
  1.92330969  3.7567444 ], Cost: 0.0328
Current parameters: [ 1.70752379  1.14150112  0.70747379  1.12412288  0.91138567  1.56144591
 -0.18502516  0.09066195  2.18690402  1.02651289  2.8744267   2.18859509
  1.1203603   1.24365593  0.03386188  0.90479531  0.41997221  1.00017179
  1.92024661  3.75453374], Cost: 0.0249
Current parameters: [ 1.6957019   1.13853448  0.74531765  1.11870541  0.78170191  1.55996819
 -0.19287078  0.08543202  2.18873511  1.02909063  2.84762895  2.18293864
  1.11924698  1.24329603  0.0263298   0.88882065  0.42143697  0.99340557
  1.9193829   3.75737185], Cost: 0.0469
Current parameters: [ 1.65470258  1.15727874  0.69767059  1.12762816  0.88406317  1.55925042
 -0.1821593   0.08897298  2.19155044  1.0193883   2.88330667  2.20891819
  1.14150202  1.25837472  0.04007453  0.85915983  0.43055026  1.00989993
  1.93758301  3.76135785], Cost: 0.0764
Current parameters: [ 1.64376905  1.13530277  0.73271239  1.14042842  0.87394768  1.56256784
 -0.19396097  0.08458165  2.18527801  1.0296586   2.93522303  2.20641275
  1.13861237  1.23842646  0.03305872  0.97530539  0.46922236  1.03415273
  1.92518987  3.7381341 ], Cost: 0.3898
Current parameters: [ 1.64891597  1.14120154  0.68314793  1.14494377  0.87850313  1.56272764
 -0.1731347   0.08437783  2.19357888  1.02881163  2.88780056  2.168043
  1.10195719  1.23236448  0.03571454  0.88833074  0.4311729   0.99087424
  1.90350044  3.74023719], Cost: 0.0211
Current parameters: [ 1.68431349  1.17818394  0.61212118  1.09133605  0.86504143  1.5351059
 -0.1821852   0.07022408  2.20704433  1.04831085  2.83968467  2.19989992
  1.10726875  1.24434036  0.0119582   0.91235486  0.40315396  1.01891237
  1.9339468   3.72950512], Cost: 0.1245
Current parameters: [ 1.65355328  1.13605591  0.69973176  1.13090285  0.88860273  1.56258465
 -0.18255012  0.09394244  2.18751329  1.02520325  2.85089708  2.16463305
  1.15560836  1.22623332  0.01789325  0.8977108   0.42313948  1.0266802
  1.93345216  3.73559447], Cost: 0.0426
Current parameters: [ 1.65088657  1.1010654   0.68350719  1.09058606  0.89654172  1.56138025
 -0.207391    0.0815186   2.18628759  1.04731592  2.85671773  2.183453
  1.17690107  1.23690804  0.03692593  0.87777185  0.39517268  0.93561115
  1.8676876   3.76334835], Cost: 0.1768
Current parameters: [ 1.64560042  1.19105593  0.71036159  1.12382168  0.8924394   1.56980451
 -0.19774694  0.09806201  2.17730873  1.02499254  2.85147209  2.18185716
```



```
  1.11957481  1.23581583  0.03486188  0.90593037  0.41683682  0.99350962
  1.91076283  3.76070246], Cost: 0.0149
Current parameters: [ 1.64786464  1.19680919  0.70720593  1.12393092  0.89106605  1.56764209
 -0.19586595  0.09569761  2.17895936  1.02450387  2.85823189  2.16374132
  1.12620824  1.25097476  0.02503414  0.90929462  0.41928799  0.99087784
  1.91150163  3.77256224], Cost: 0.0113
Current parameters: [ 1.6456359   1.18054649  0.69742131  1.11991202  0.89302917  1.57318194
 -0.20354198  0.09815659  2.17655891  0.9932912   2.83579087  2.14637706
  1.10779734  1.26332381  0.0536287   0.91047474  0.42403013  1.00015589
  1.91576849  3.78681066], Cost: 0.0695
Current parameters: [ 1.64809974  1.19589314  0.70583435  1.12311702  0.89128433  1.56800677
 -0.19597934  0.09566759  2.17906297  1.00313176  2.85775335  2.17178288
  1.12502686  1.24984073  0.00659624  0.90863056  0.42153518  0.99045792
  1.90141519  3.77145986], Cost: 0.0077
Current parameters: [ 1.64320575  1.18347645  0.70770139  1.12911585  0.9000443   1.58366128
 -0.20588072  0.10334736  2.17146647  1.02476992  2.84652359  2.17323094
  1.09929309  1.24829921 -0.02555058  0.89571281  0.43001441  0.98458204
  1.92001132  3.78690608], Cost: 0.0143
Current parameters: [ 1.64705492  1.19463337  0.7073024   1.12990552  0.89305016  1.54084088
 -0.20345233  0.09712988  2.17773343  1.00299971  2.85592365  2.17143455
  1.12338358  1.2428312   0.00615184  0.90747261  0.42448057  0.9900619
  1.90167089  3.77914318], Cost: 0.0102
Current parameters: [ 1.64158970e+00  1.19707820e+00  7.25361914e-01  1.11450139e+00
  9.00607228e-01  1.54884912e+00 -1.67464670e-01  1.05186526e-01
  2.16806604e+00  1.00666851e+00  2.84400084e+00  2.16752241e+00
  1.11336566e+00  1.27659917e+00  1.08980376e-03  9.02348244e-01
  4.20812408e-01  9.82531258e-01  1.89851929e+00  3.74471980e+00], Cost: 0.0159
Current parameters: [ 1.65121049  1.19861006  0.70209994  1.13174398  0.89100813  1.56931772
 -0.19278358  0.09173436  2.17991549  0.99835388  2.84983509  2.17450014
  1.13147966  1.24827328  0.00873511  0.91382999  0.43429317  0.97160342
  1.91303998  3.76688604], Cost: 0.0152
Current parameters: [ 1.64360164e+00  1.19788378e+00  7.12590740e-01  1.10162692e+00
  8.91795915e-01  1.56396407e+00 -2.03496330e-01  9.53466894e-02
  2.17734290e+00  9.85345149e-01  2.88043140e+00  2.15736503e+00
  1.11608337e+00  1.23423781e+00  2.97459154e-03  9.04844033e-01
  3.98474970e-01  9.73851183e-01  1.93288528e+00  3.76092127e+00], Cost: 0.0310
Current parameters: [ 1.64762486  1.20356392  0.71108746  1.12983277  0.88794901  1.56770457
 -0.19311881  0.09658626  2.17882004  0.99992936  2.86614576  2.17488748
  1.11514025  1.25140916  0.00555935  0.92176703  0.43145576  1.00575051
  1.91216517  3.76792697], Cost: 0.0373
Current parameters: [ 1.63908536e+00  1.18308209e+00  7.16419768e-01  1.10967402e+00
```



```
  8.98475521e-01  1.57231178e+00 -2.13773338e-01  9.46696160e-02
  2.17039889e+00  1.02214521e+00  2.87191365e+00  2.17231179e+00
  1.14305397e+00  1.24684184e+00 -3.76544867e-04  9.23071546e-01
  4.30040991e-01  9.69449886e-01  1.86635175e+00  3.76852732e+00], Cost: 0.0189
Current parameters: [ 1.64332063  1.18920108  0.71945385  1.14305385  0.89347542  1.57277835
 -0.19320829  0.0979368   2.1759699   0.99810855  2.86152348  2.17253578
  1.12898423  1.25254738  0.00883182  0.91067276  0.40878184  0.98705953
  1.90138231  3.77690417], Cost: 0.0055
Current parameters: [ 1.65200987  1.19921709  0.68952024  1.15513318  0.88879637  1.56727247
 -0.19233158  0.08962732  2.17897236  1.01350215  2.85001976  2.18097704
  1.1286804   1.25724929 -0.00624511  0.91947514  0.3689806   0.98049598
  1.88999932  3.77114953], Cost: 0.0508
Current parameters: [ 1.63362951  1.19044586  0.70903162  1.14133564  0.91632287  1.57272244
 -0.18161189  0.09870467  2.17523758  0.99749752  2.86596473  2.17488623
  1.13109982  1.24770323  0.01019913  0.91480006  0.40890322  0.98890572
  1.90274657  3.78274847], Cost: 0.0073
Current parameters: [ 1.65108009  1.18797559  0.73317986  1.12930049  0.87199071  1.57377864
 -0.15576974  0.09716656  2.17357423  1.00197154  2.85542898  2.17187158
  1.13038133  1.22724913  0.00607619  0.91131856  0.4084904   0.98119203
  1.8935619   3.8051578 ], Cost: 0.0458
Current parameters: [ 1.64712811  1.18927079  0.70940967  1.1443503   0.88743428  1.57303562
 -0.19224932  0.12132346  2.16146712  0.99710183  2.86736175  2.17440494
  1.13314905  1.25318804  0.01058672  0.90952336  0.40972964  0.98556206
  1.90230694  3.7768672 ], Cost: 0.0119
Current parameters: [ 1.63993801  1.20311931  0.72453283  1.14518383  0.89901901  1.57393984
 -0.19832429  0.08887389  2.1801687   0.99849537  2.87599255  2.19118623
  1.14747461  1.26697556  0.01256392  0.86268244  0.41588616  0.98764694
  1.90086891  3.78300744], Cost: 0.0354
Current parameters: [ 1.64477093e+00  1.18685346e+00  7.15941777e-01  1.14120991e+00
  8.94619376e-01  1.57803555e+00 -1.92377655e-01  9.93160151e-02
  2.17717715e+00  9.94862395e-01  2.85231028e+00  2.16131925e+00
  1.14322916e+00  1.24364201e+00  8.61902545e-04  9.03520664e-01
  4.08298642e-01  1.00197174e+00  1.89908046e+00  3.76829117e+00], Cost: 0.0161
Current parameters: [ 1.63228775  1.18254714  0.74574485  1.17261777  0.90433823  1.55607437
 -0.20019234  0.09988067  2.16574866  1.01640876  2.86259948  2.15896745
  1.14053262  1.24577935  0.00859941  0.92182104  0.41604017  0.97370276
  1.92336253  3.76778967], Cost: 0.0206
Current parameters: [ 1.64185337  1.18950042  0.7226956   1.14310797  0.89525029  1.57256386
 -0.19418274  0.11520105  2.20128941  0.99938127  2.86091019  2.17270113
  1.12931583  1.25307489  0.00834772  0.91188192  0.40873453  0.9831374
  1.90037213  3.77703308], Cost: 0.0166
```



```
Current parameters: [ 1.64860243  1.18949541  0.72364272  1.13776175  0.8969407   1.57220191
 -0.1959457   0.09919831  2.17455661  0.99779573  2.85734161  2.17789675
  1.13438558  1.25572792  0.00790668  0.9147271   0.40614594  0.98921044
  1.90591421  3.78039109], Cost: 0.0076
Current parameters: [ 1.66803766  1.18821686  0.72228737  1.1478134   0.90582808  1.57398315
 -0.19211732  0.09764506  2.17604067  0.99816947  2.86773253  2.1674415
  1.12229416  1.2489432   0.01082572  0.90747653  0.41036651  0.98513272
  1.89600372  3.77424692], Cost: 0.0142
Current parameters: [ 1.64530873  1.17750793  0.71643539  1.14266449  0.89137657  1.57016641
 -0.18975734  0.09568481  2.17867556  0.99943668  2.86609873  2.17528103
  1.1311343   1.2550522   0.00728591  0.91059219  0.40951396  0.98845451
  1.90452988  3.77524542], Cost: 0.0081
Current parameters: [ 1.63732895  1.17680932  0.71687289  1.14200473  0.89662695  1.57452553
 -0.20159431  0.0963573   2.17194181  0.98762261  2.85095196  2.16379594
  1.12212729  1.26159516  0.02236824  0.91078316  0.41080591  0.98489769
  1.89835897  3.783917  ], Cost: 0.0119
Current parameters: [ 1.64286581  1.18769096  0.71992462  1.14496153  0.89411073  1.574491
 -0.19418424  0.09787485  2.17503701  1.00383183  2.86002255  2.1758784
  1.13232269  1.25498121  0.00821408  0.914624    0.41548269  0.98840456
  1.8910796   3.77763102], Cost: 0.0054
Current parameters: [ 1.63723297e+00  1.18437541e+00  7.23547507e-01  1.12822273e+00
  8.96196819e-01  1.57811221e+00 -2.05235803e-01  9.55976974e-02
  2.17159634e+00  1.00253823e+00  2.87349852e+00  2.17235657e+00
  1.13524233e+00  1.24958760e+00  3.57364212e-03  9.18659168e-01
  4.11584259e-01  9.77719122e-01  1.88426127e+00  3.77155429e+00], Cost: 0.0043
Current parameters: [ 1.63861874  1.18413933  0.71963988  1.13242211  0.89401096  1.58196791
 -0.21219966  0.09337854  2.17402181  1.00222439  2.8768991   2.17329084
  1.13784277  1.24295992  0.00550275  0.92102279  0.41236113  0.97924967
  1.88669295  3.7785487 ], Cost: 0.0039
Current parameters: [ 1.63723297e+00  1.18437541e+00  7.23547507e-01  1.12822273e+00
  8.96196819e-01  1.57811221e+00 -2.05235803e-01  9.55976974e-02
  2.17159634e+00  1.00253823e+00  2.87349852e+00  2.17235657e+00
  1.13524233e+00  1.24958760e+00  3.57364212e-03  9.18659168e-01
  4.11584259e-01  9.77719122e-01  1.88426127e+00  3.77155429e+00], Cost: 0.0043

   Return from subroutine COBYLA because the MAXFUN limit has been reached.

   NFVALS =  100   F = 4.336054E-03    MAXCV = 0.000000E+00
   X = 1.637233E+00   1.184375E+00   7.235475E-01   1.128223E+00   8.961968E-01
       1.578112E+00  -2.052358E-01   9.559770E-02   2.171596E+00   1.002538E+00
       2.873499E+00   2.172357E+00   1.135242E+00   1.249588E+00   3.573642E-03
```

```
      9.186592E-01   4.115843E-01   9.777191E-01   1.884261E+00   3.771554E+00
Simulating for QAOA depth p = 11
Current parameters: [2.1122611  2.37900618 0.7756055  0.92450517 1.5627562  0.16779522
 1.53427542 0.0310333  1.67034306 0.31812982 1.78202056 1.31210268
 1.09066288 2.86496985 1.75447334 2.61221674 1.37833736 0.48695983
 2.02374465 2.27777634 2.31763471 2.85029636], Cost: 1.4909
Current parameters: [2.1122611  3.37900618 0.7756055  0.92450517 1.5627562  0.16779522
 1.53427542 0.0310333  1.67034306 0.31812982 1.78202056 1.31210268
 1.09066288 2.86496985 1.75447334 2.61221674 1.37833736 0.48695983
 2.02374465 2.27777634 2.31763471 2.85029636], Cost: 2.6449
Current parameters: [2.1122611  2.37900618 1.7756055  0.92450517 1.5627562  0.16779522
 1.53427542 0.0310333  1.67034306 0.31812982 1.78202056 1.31210268
 1.09066288 2.86496985 1.75447334 2.61221674 1.37833736 0.48695983
 2.02374465 2.27777634 2.31763471 2.85029636], Cost: 1.7168
Current parameters: [2.1122611  2.37900618 0.7756055  1.92450517 1.5627562  0.16779522
 1.53427542 0.0310333  1.67034306 0.31812982 1.78202056 1.31210268
 1.09066288 2.86496985 1.75447334 2.61221674 1.37833736 0.48695983
 2.02374465 2.27777634 2.31763471 2.85029636], Cost: 2.2746
Current parameters: [2.1122611  2.37900618 0.7756055  0.92450517 2.5627562  0.16779522
 1.53427542 0.0310333  1.67034306 0.31812982 1.78202056 1.31210268
 1.09066288 2.86496985 1.75447334 2.61221674 1.37833736 0.48695983
 2.02374465 2.27777634 2.31763471 2.85029636], Cost: 2.0640
Current parameters: [2.1122611  2.37900618 0.7756055  0.92450517 1.5627562  1.16779522
 1.53427542 0.0310333  1.67034306 0.31812982 1.78202056 1.31210268
 1.09066288 2.86496985 1.75447334 2.61221674 1.37833736 0.48695983
 2.02374465 2.27777634 2.31763471 2.85029636], Cost: 0.4606
Current parameters: [2.1122611  2.37900618 0.7756055  0.92450517 1.5627562  1.16779522
 2.53427542 0.0310333  1.67034306 0.31812982 1.78202056 1.31210268
 1.09066288 2.86496985 1.75447334 2.61221674 1.37833736 0.48695983
 2.02374465 2.27777634 2.31763471 2.85029636], Cost: 2.5975
Current parameters: [2.1122611  2.37900618 0.7756055  0.92450517 1.5627562  1.16779522
 1.53427542 1.0310333  1.67034306 0.31812982 1.78202056 1.31210268
 1.09066288 2.86496985 1.75447334 2.61221674 1.37833736 0.48695983
 2.02374465 2.27777634 2.31763471 2.85029636], Cost: 1.5956
Current parameters: [2.1122611  2.37900618 0.7756055  0.92450517 1.5627562  1.16779522
 1.53427542 0.0310333  2.67034306 0.31812982 1.78202056 1.31210268
 1.09066288 2.86496985 1.75447334 2.61221674 1.37833736 0.48695983
 2.02374465 2.27777634 2.31763471 2.85029636], Cost: 0.8989
Current parameters: [2.1122611  2.37900618 0.7756055  0.92450517 1.5627562  1.16779522
 1.53427542 0.0310333  1.67034306 1.31812982 1.78202056 1.31210268
 1.09066288 2.86496985 1.75447334 2.61221674 1.37833736 0.48695983
```



```
 2.02374465 2.27777634 2.31763471 2.85029636], Cost: 2.5453
Current parameters: [2.1122611  2.37900618 0.7756055  0.92450517 1.5627562  1.16779522
 1.53427542 0.0310333  1.67034306 0.31812982 2.78202056 1.31210268
 1.09066288 2.86496985 1.75447334 2.61221674 1.37833736 0.48695983
 2.02374465 2.27777634 2.31763471 2.85029636], Cost: 0.6455
Current parameters: [2.1122611  2.37900618 0.7756055  0.92450517 1.5627562  1.16779522
 1.53427542 0.0310333  1.67034306 0.31812982 1.78202056 2.31210268
 1.09066288 2.86496985 1.75447334 2.61221674 1.37833736 0.48695983
 2.02374465 2.27777634 2.31763471 2.85029636], Cost: 2.1126
Current parameters: [2.1122611  2.37900618 0.7756055  0.92450517 1.5627562  1.16779522
 1.53427542 0.0310333  1.67034306 0.31812982 1.78202056 1.31210268
 2.09066288 2.86496985 1.75447334 2.61221674 1.37833736 0.48695983
 2.02374465 2.27777634 2.31763471 2.85029636], Cost: 1.7090
Current parameters: [2.1122611  2.37900618 0.7756055  0.92450517 1.5627562  1.16779522
 1.53427542 0.0310333  1.67034306 0.31812982 1.78202056 1.31210268
 1.09066288 3.86496985 1.75447334 2.61221674 1.37833736 0.48695983
 2.02374465 2.27777634 2.31763471 2.85029636], Cost: 2.6734
Current parameters: [2.1122611  2.37900618 0.7756055  0.92450517 1.5627562  1.16779522
 1.53427542 0.0310333  1.67034306 0.31812982 1.78202056 1.31210268
 1.09066288 2.86496985 2.75447334 2.61221674 1.37833736 0.48695983
 2.02374465 2.27777634 2.31763471 2.85029636], Cost: 1.1158
Current parameters: [2.1122611  2.37900618 0.7756055  0.92450517 1.5627562  1.16779522
 1.53427542 0.0310333  1.67034306 0.31812982 1.78202056 1.31210268
 1.09066288 2.86496985 1.75447334 3.61221674 1.37833736 0.48695983
 2.02374465 2.27777634 2.31763471 2.85029636], Cost: 2.5033
Current parameters: [2.1122611  2.37900618 0.7756055  0.92450517 1.5627562  1.16779522
 1.53427542 0.0310333  1.67034306 0.31812982 1.78202056 1.31210268
 1.09066288 2.86496985 1.75447334 2.61221674 2.37833736 0.48695983
 2.02374465 2.27777634 2.31763471 2.85029636], Cost: 2.5553
Current parameters: [2.1122611  2.37900618 0.7756055  0.92450517 1.5627562  1.16779522
 1.53427542 0.0310333  1.67034306 0.31812982 1.78202056 1.31210268
 1.09066288 2.86496985 1.75447334 2.61221674 1.37833736 1.48695983
 2.02374465 2.27777634 2.31763471 2.85029636], Cost: 2.8650
Current parameters: [2.1122611  2.37900618 0.7756055  0.92450517 1.5627562  1.16779522
 1.53427542 0.0310333  1.67034306 0.31812982 1.78202056 1.31210268
 1.09066288 2.86496985 1.75447334 2.61221674 1.37833736 0.48695983
 3.02374465 2.27777634 2.31763471 2.85029636], Cost: 2.8441
Current parameters: [2.1122611  2.37900618 0.7756055  0.92450517 1.5627562  1.16779522
 1.53427542 0.0310333  1.67034306 0.31812982 1.78202056 1.31210268
 1.09066288 2.86496985 1.75447334 2.61221674 1.37833736 0.48695983
 2.02374465 3.27777634 2.31763471 2.85029636], Cost: 2.4310
```



```
Current parameters: [2.1122611  2.37900618 0.7756055  0.92450517 1.5627562  1.16779522
 1.53427542 0.0310333  1.67034306 0.31812982 1.78202056 1.31210268
 1.09066288 2.86496985 1.75447334 2.61221674 1.37833736 0.48695983
 2.02374465 2.27777634 3.31763471 2.85029636], Cost: 1.9862
Current parameters: [2.1122611  2.37900618 0.7756055  0.92450517 1.5627562  1.16779522
 1.53427542 0.0310333  1.67034306 0.31812982 1.78202056 1.31210268
 1.09066288 2.86496985 1.75447334 2.61221674 1.37833736 0.48695983
 2.02374465 2.27777634 2.31763471 3.85029636], Cost: 2.2767
Current parameters: [ 2.14017168  2.2204849   0.74457659  0.81684959  1.4840355   1.30931705
  1.24074798 -0.12487587  1.61014404  0.03177823  1.75662245  1.08517605
  0.9191783   2.56102107  1.66447754  2.33162485  1.09060838  0.15668792
  1.69633878  2.00711385  2.10807857  2.6008385 ], Cost: 2.3906
Current parameters: [ 2.12621639  2.29974554  0.76009104  0.87067738  1.52339585  1.23855614
  1.3875117  -0.04692129  1.64024355  0.17495403  1.76932151  1.19863936
  1.00492059  2.71299546  1.70947544  2.4719208   1.23447287  0.32182387
  1.86004171  2.14244509  2.21285664  2.72556743], Cost: 2.0380
Current parameters: [2.36113852 2.37900618 0.7756055  0.92450517 1.5627562  1.16779522
 1.55794038 0.0310333  1.67034306 0.31812982 1.78202056 1.31210268
 1.09066288 2.86496985 1.75447334 2.61221674 1.37833736 0.48695983
 2.02374465 2.27777634 2.31763471 2.85029636], Cost: 0.2956
Current parameters: [2.32449242 2.36201997 0.77228062 0.91296944 1.55432095 1.18295987
 2.04597037 0.01432699 1.66389249 0.28744607 1.77929905 1.28778656
 1.0722876  2.83240049 1.74482992 2.58215017 1.34750602 0.45156983
 1.98866177 2.24877374 2.2951799  2.82356592], Cost: 1.2101
Current parameters: [2.36113852 2.42702976 0.53026138 0.92450517 1.5627562  1.16779522
 1.55794038 0.0310333  1.67034306 0.31812982 1.78202056 1.31210268
 1.09066288 2.86496985 1.75447334 2.61221674 1.37833736 0.48695983
 2.02374465 2.27777634 2.31763471 2.85029636], Cost: 0.3639
Current parameters: [2.36729752 2.86614232 0.8732044  0.91817894 1.55813028 1.17611156
 1.54945267 0.0218715  1.66680555 0.30130275 1.78052808 1.29876764
 1.08058582 2.8471087  1.74918486 2.59572813 1.36142935 0.46755183
 2.00450508 2.26187121 2.30532042 2.8356373 ], Cost: 0.8172
Current parameters: [2.36113852 2.37900618 0.7756055  1.07206957 1.36095246 1.16779522
 1.55794038 0.0310333  1.67034306 0.31812982 1.78202056 1.31210268
 1.09066288 2.86496985 1.75447334 2.61221674 1.37833736 0.48695983
 2.02374465 2.27777634 2.31763471 2.85029636], Cost: 0.3031
Current parameters: [2.3662464  2.37435968 0.77655953 1.32624374 1.85676911 1.17469227
 1.5509012  0.02343508 1.662740927 0.3041745  1.78078279 1.30104344
 1.0823056  2.85015694 1.7500874  2.59854213 1.36431492 0.47086406
 2.00778856 2.26458563 2.30742201 2.83813905], Cost: 1.6107
Current parameters: [2.33331424 2.38081114 0.7759588  0.92573097 1.56365254 0.92080562
```



```
 1.55866706 0.02412566 1.66343543 0.31122218 1.77511293 1.30519505
 1.08375525 2.85806221 1.7475657  2.60530911 1.37142973 0.48005219
 2.01683702 2.2708687  2.31072707 2.84338872], Cost: 0.4693
Current parameters: [2.80604052 2.31730142 0.77771135 0.81152769 1.48205219 1.14371785
 1.49375908 0.07400033 1.74881243 0.31271107 1.87339884 1.32872678
 1.12785243 2.85302367 1.82189001 2.60893447 1.37240769 0.46524945
 2.00309739 2.27817724 2.34070215 2.85856282], Cost: 0.4476
Current parameters: [2.358989   2.37497232 0.77661819 0.91273492 1.55439194 1.17023507
 1.55131776 0.02192483 1.66574568 0.56303014 1.77906345 1.29964697
 1.0808203  2.84888386 1.74847156 2.59723163 1.36301586 0.46963316
 2.00655307 2.26325923 2.30599771 2.83677868], Cost: 0.5378
Current parameters: [ 2.37972214  2.37780065  0.77536953  0.92368647  1.56215755  1.16887147
  1.55745503 -0.0754452   1.60969749  0.31595217  1.78663412  1.31671624
  1.09527644  2.86958341  1.7590869   2.6168303   1.38295092  0.49157338
  2.02835821  2.28238989  2.32224826  2.85490992], Cost: 0.4538
Current parameters: [2.3806832  2.37594479 0.77578081 0.91854504 1.55850219 1.17002918
 1.55455966 0.15992093 1.45732712 0.31514346 1.7858312  1.31183118
 1.09151441 2.8631382  1.75697549 2.61085821 1.37683426 0.48459498
 2.02143786 2.27661893 2.31771506 2.84956847], Cost: 0.5631
Current parameters: [2.38166217 2.3776748  0.77534489 0.92360101 1.56209505 1.16898382
 1.55740437 0.02972386 1.66983747 0.31572484 1.69114513 1.23041256
 1.09575807 2.87006504 1.75956853 2.61731193 1.38343255 0.49205502
 2.02883984 2.28287153 2.3227299  2.85539155], Cost: 0.2930
Current parameters: [2.3985199  2.37291064 0.77599754 0.91242258 1.55413434 1.17221557
 1.55108983 0.03382731 1.67901809 0.31232481 1.52564601 1.41432406
 1.09175553 2.86057114 1.75894414 2.6087863  1.37461105 0.48146987
 2.01837352 2.27475752 2.31714908 2.84815659], Cost: 0.4794
Current parameters: [2.37115207 2.37835659 0.77547835 0.92406403 1.56243362 1.16837514
 1.55767886 0.03039442 1.67009638 0.31695642 1.69125436 1.23138856
 1.21453568 2.83335404 1.7569593  2.6147027  1.38082332 0.48944578
 2.02623061 2.28026229 2.32012066 2.85278232], Cost: 0.1675
Current parameters: [2.35707359 2.37402528 0.77689546 0.90977353 1.55228873 1.17078242
 1.54965333 0.03869244 1.68415394 0.31657063 1.69550714 1.2319487
 1.28929675 3.06275051 1.74659234 2.59304637 1.35874424 0.4648468
 2.00180139 2.2591941  2.30267191 2.83297011], Cost: 1.1929
Current parameters: [2.36736963 2.37860196 0.77552638 0.92423066 1.56255547 1.16815609
 1.55777764 0.03063574 1.67018956 0.31739965 1.69129368 1.23173981
 1.21480111 2.83382451 1.75602027 2.61376368 1.3798843  0.48850676
 2.02529159 2.27932327 2.4208799  2.77894374], Cost: 0.6577
Current parameters: [2.34751724 2.37542978 0.77683155 0.91242513 1.55418207 1.16974688
 1.55115851 0.03819516 1.68233827 0.31799496 1.69499215 1.23294792
```

```
 1.21798596 2.8129469  1.7452479  2.59339078 1.3591519  0.46563143
 2.00256062 2.25945053 2.4387941  3.06330924], Cost: 0.2484
Current parameters: [2.36860936 2.37852154 0.77551063 0.92417605 1.56251553 1.16822788
 1.55774526 0.03055665 1.67015902 0.31725438 1.69128079 1.23162469
 1.21471411 2.83367031 1.75632805 2.61407145 1.47698669 0.40963945
 2.02559936 2.27963104 2.32033871 2.85304188], Cost: 0.1271
Current parameters: [2.3567656  2.37094035 0.7776325  0.90095964 1.54602416 1.17267244
 1.54469242 0.04309293 1.69227856 0.31540165 1.69794145 1.23153493
 1.21889946 2.79143625 1.74244761 2.58221809 1.62652292 0.58432734
 1.98933151 2.24871401 2.28176871 2.8839946 ], Cost: 0.7539
Current parameters: [2.38724782 2.37731246 0.77527397 0.92335493 1.56191511 1.16930731
 1.55725849 0.02936749 1.66969987 0.31507031 1.69108707 1.22989386
 1.21340616 2.83135202 1.6341299  2.63033783 1.47479211 0.40712038
 2.03022653 2.28425822 2.31874038 2.85113921], Cost: 0.2865
Current parameters: [2.34792357 2.37440936 0.77706111 0.90958106 1.55216016 1.17038123
 1.54955705 0.03957179 1.68492699 0.31756154 1.69576627 1.23276856
 1.21835678 2.80769276 1.79484938 2.8521292  1.47142117 0.39741345
 1.99869366 2.25622061 2.29625441 2.87458277], Cost: 0.2565
Current parameters: [2.37231674 2.37828104 0.77546356 0.92401272 1.5623961  1.16844259
 1.55764844 0.03032011 1.67006769 0.31681994 1.69124226 1.23128041
 1.21445395 2.83320917 1.75619151 2.61364576 1.47655017 0.40913838
 2.10411562 2.1824469  2.32002079 2.85266342], Cost: 0.1980
Current parameters: [2.34347009 2.37013388 0.77819539 0.89680041 1.54307979 1.17292796
 1.54236873 0.04629599 1.69696919 0.31631188 1.69938641 1.2325318
 1.22050294 2.78435347 1.78642254 2.61194213 1.46524956 0.385444
 2.19715643 2.42903162 2.2749962  2.89129723], Cost: 0.1044
Current parameters: [2.30984834 2.36249481 0.77670013 0.812479   1.48142167 1.18153772
 1.5248989  0.03681095 1.69330686 0.29889112 1.69784127 1.21872628
 1.21007034 2.76586215 1.78094747 2.59487177 1.447745   0.36535127
 2.17723806 2.41256534 2.26224744 2.87612097], Cost: 0.2505
Current parameters: [2.34938156 2.33161636 0.77631933 0.99908224 1.61863295 1.2001184
 1.47447975 0.04372139 1.72311393 0.27251007 1.70622083 1.19998501
 1.20258799 2.67461511 1.81243688 2.56744302 1.40711312 0.30467571
 2.15165731 2.40924725 2.18449222 2.90428877], Cost: 0.2513
Current parameters: [2.33766365 2.26352335 0.75732748 0.89674461 1.54303898 1.17300132
 1.60392986 0.04621517 1.69693798 0.31616345 1.69937325 1.23241417
 1.22041404 2.78419592 1.78637589 2.61179668 1.46510041 0.3852728
 2.19698672 2.42889132 2.27488758 2.89116792], Cost: 0.3126
Current parameters: [2.37473721 2.49660026 0.80610566 0.89139448 1.53955138 1.18290096
 1.7350161  0.05056605 1.71374044 0.30238187 1.70412406 1.22269905
 1.21679484 2.73432567 1.80421139 2.59741282 1.4433823  0.35252229
```



```
 2.18378281 2.42791074 2.23223251 2.90766412], Cost: 0.1420
Current parameters: [2.24195224 2.38362991 0.7808371  0.92245297 1.56183763 1.18700891
 1.55705955 0.0563638  1.64443085 0.31667707 1.67855236 1.22882048
 1.21208102 2.78718628 1.77107698 2.61279044 1.46670426 0.39051334
 2.20198226 2.42903629 2.269809   2.88950025], Cost: 0.1820
Current parameters: [2.43701543 2.36708739 0.77928369 0.87910344 1.53036591 1.16732029
 1.52583924 0.17768798 1.51342107 0.31043348 1.71682526 1.23635345
 1.22920046 2.76448654 1.80895667 2.61170173 1.46088523 0.37394592
 2.19617654 2.4362366  2.261742   2.90845191], Cost: 0.1336
Current parameters: [2.34294086 2.36857745 0.77789074 0.89194821 1.53953172 1.17212923
 1.53961115 0.04189005 1.69423625 0.43392213 1.69610935 1.2281589
 1.23111832 2.76534533 1.7840221  2.60581398 1.44493442 0.38983456
 2.19011269 2.42309768 2.24957415 2.88837898], Cost: 0.0778
Current parameters: [2.35394016 2.39954913 0.79024315 0.90330519 1.54868247 1.18305211
 1.52227274 0.06530735 1.71485399 0.50152118 1.70800997 1.21600408
 1.10715357 2.79369781 1.8132806  2.59784656 1.54066551 0.25634206
 2.19112712 2.44043913 2.34791708 2.91583163], Cost: 0.4592
Current parameters: [2.33416025 2.36967231 0.77810505 0.89394492 1.54099177 1.17187433
 1.54075557 0.0426106  1.693517   0.43519805 1.69618062 1.22849467
 1.27697936 2.84425754 1.82327131 2.5744368  1.44706959 0.35713461
 2.14110265 2.44203166 2.22048896 2.88921954], Cost: 0.3287
Current parameters: [2.37660353 2.41360717 0.79697382 0.89595852 1.54384569 1.18869112
 1.50302805 0.0812429  1.74049385 0.43553592 1.71850798 1.21618326
 1.2342721  2.81539865 1.69934815 2.67923993 1.37292652 0.43052446
 2.33287419 2.37753487 2.18624499 2.95625394], Cost: 1.1510
Current parameters: [2.37116901 2.39418059 0.78290229 0.90233603 1.54712758 1.16258105
 1.50501262 0.03457769 1.73369747 0.40476017 1.70388533 1.23998633
 1.24322959 2.77040035 1.80466004 2.60063111 1.51502931 0.44974593
 2.19250603 2.44923555 2.25697955 2.90106078], Cost: 0.1968
Current parameters: [2.27939128 2.3360612  0.77717073 0.8627352  1.51892977 1.19913055
 1.58932479 0.07970293 1.64050552 0.49886635 1.69193275 1.20086238
 1.20213533 2.64536525 1.79304453 2.55573851 1.52113143 0.46017826
 2.11139358 2.37646952 2.17029162 2.90820705], Cost: 0.9132
Current parameters: [2.33046319 2.37224887 0.77921214 0.89433652 1.54135922 1.04919627
 1.53939482 0.04101594 1.69180207 0.43129663 1.69247432 1.22471926
 1.22427129 2.75569987 1.781997   2.60427935 1.43975687 0.38065714
 2.1902092  2.42136562 2.24402103 2.88999214], Cost: 0.0506
Current parameters: [2.33046319 2.37356935 0.77246602 0.85766924 1.59150407 1.04919627
 1.53939482 0.04101594 1.69180207 0.43129663 1.69247432 1.22471926
 1.22427129 2.75569987 1.781997   2.60427935 1.43975687 0.38065714
 2.1902092  2.42136562 2.24402103 2.88999214], Cost: 0.0385
```



```
Current parameters: [2.33097857 2.39857665 0.65084806 0.86640847 1.58108182 1.04889978
 1.53916436 0.04203382 1.69219557 0.43161357 1.69238721 1.22471035
 1.22289866 2.75320725 1.78255423 2.60503287 1.43905241 0.37835183
 2.19161555 2.42204013 2.24316625 2.89200548], Cost: 0.0758
Current parameters: [2.32726241 2.3726242  0.77282814 0.85427335 1.58909451 1.04974845
 1.53732879 0.04089261 1.69414183 0.43108133 1.67327987 1.24757208
 1.23462436 2.74789008 1.78248551 2.59686572 1.42782952 0.38013423
 2.18088913 2.41778352 2.26288015 2.93642035], Cost: 0.0350
Current parameters: [2.34267868 2.3745445  0.77417867 0.85368099 1.58946279 1.0500444
 1.53528985 0.04316248 1.69602801 0.43034396 1.60062155 1.32668767
 1.21649671 2.7433197  1.78353325 2.60288831 1.43401804 0.36740674
 2.1900481  2.41950347 2.23602412 2.88708389], Cost: 0.1955
Current parameters: [2.33372879 2.37284225 0.7724196  0.85672706 1.59082802 1.04962699
 1.53926796 0.03719925 1.6896547  0.43108127 1.67234359 1.24702984
 1.22921738 2.75386998 1.7646222  2.54184294 1.42483921 0.38538724
 2.19904521 2.4176874  2.26732079 2.93206506], Cost: 0.0331
Current parameters: [ 2.35468684  2.37689444  0.77418339  0.85889336  1.59350765  1.04929221
  1.53774709 -0.0668597   1.62947989  0.43037499  1.67962753  1.24844161
  1.22740457  2.74712081  1.7731866   2.55449365  1.42726717  0.37916318
  2.20965653  2.42584501  2.26675501  2.94393021], Cost: 0.0867
Current parameters: [2.34395325 2.37218307 0.77228991 0.85628242 1.5905028  1.05021727
 1.53900064 0.03654432 1.68940211 0.42988155 1.62690738 1.20619103
 1.23200129 2.75635649 1.767234   2.54437449 1.42725225 0.38804136
 2.20151213 2.4202634  2.2702092  2.93435664], Cost: 0.0453
Current parameters: [2.33090246 2.38598587 0.77678557 0.8658584  1.59983316 1.04548258
 1.53778147 0.03947617 1.68836967 0.43499579 1.6799764  1.24315606
 1.27573148 2.71501292 1.78475257 2.53883258 1.3839125  0.39143424
 2.19616676 2.43276389 2.34684424 2.87927248], Cost: 0.1682
Current parameters: [2.33127649 2.37199257 0.77247944 0.85508596 1.58965844 1.04998608
 1.5390311  0.03818159 1.69044123 0.43159331 1.67264331 1.24695724
 1.26766617 2.74200955 1.7452244  2.54387695 1.4525831  0.35894195
 2.21634412 2.40710293 2.26369647 2.93272547], Cost: 0.0602
Current parameters: [2.34836385 2.3944426  0.78290875 0.85865394 1.59725677 1.04548733
 1.52348248 0.04881038 1.70567825 0.4320137  1.69147049 1.23615571
 1.22447082 2.78322467 1.72822934 2.57912927 1.36319447 0.40100382
 2.26601188 2.39951678 2.25097407 2.96242161], Cost: 0.1394
Current parameters: [2.32180241 2.36928744 0.77210486 0.85243922 1.5877439  1.05059882
 1.54147001 0.03973585 1.68824758 0.43493274 1.67225097 1.24665653
 1.23030792 2.74830917 1.81083891 2.53351139 1.42714621 0.38840427
 2.22810304 2.39419498 2.26127298 2.93513527], Cost: 0.0949
Current parameters: [2.31105034 2.36820824 0.77749645 0.84420291 1.58750313 1.05133747
```



```
 1.55990119 0.05392576 1.66039517 0.46703874 1.68354482 1.22490788
 1.21269991 2.69371162 1.72476407 2.53393738 1.43235162 0.43348043
 2.15723928 2.4041105  2.23151022 2.9492787 ], Cost: 0.3322
Current parameters: [2.34505106 2.37846725 0.77250362 0.86524789 1.59692187 1.04741454
 1.5385045  0.03246529 1.68830479 0.42736253 1.6714773  1.24807486
 1.22813414 2.7676956  1.75858042 2.54375181 1.42990098 0.39186095
 2.18051244 2.36561047 2.28054365 2.92425651], Cost: 0.0591
Current parameters: [2.33922719 2.386013   0.78001511 0.86039161 1.5965803  1.05095191
 1.53166495 0.04883625 1.69928755 0.46331166 1.68492518 1.23556199
 1.15849888 2.75969043 1.76354907 2.54610829 1.45891284 0.32137875
 2.20742966 2.4290477  2.32068958 2.95052315], Cost: 0.2483
Current parameters: [2.32934742 2.36604702 0.76979559 0.83400755 1.57404082 1.04362861
 1.56721835 0.03921464 1.68027537 0.44531062 1.67012469 1.25449644
 1.23364042 2.77876263 1.7582826  2.55217483 1.43784046 0.40453422
 2.2097434  2.42234343 2.28726618 2.92925682], Cost: 0.0533
Current parameters: [2.3624688  2.2752304  0.75478232 0.8872496  1.61480273 1.04357756
 1.5764705  0.03022814 1.71776445 0.4025989  1.68300034 1.24656363
 1.22600463 2.7484359  1.76216505 2.54876851 1.4194206  0.38501529
 2.20519216 2.42213713 2.2722873  2.94003003], Cost: 0.0377
Current parameters: [2.3179338  2.34357093 0.76597764 0.85766172 1.59141563 1.04858813
 1.49365796 0.03966921 1.67584388 0.44512424 1.66928076 1.24837677
 1.23027519 2.76336219 1.75908948 2.54440882 1.42775093 0.3934111
 2.2015474  2.41718454 2.27398715 2.92777332], Cost: 0.0538
Current parameters: [2.33634533 2.35583138 0.7697464  0.7806933  1.53650431 1.04976313
 1.5158742  0.01593164 1.72158207 0.37583625 1.6817538  1.23582443
 1.21641787 2.74222275 1.75645207 2.53392543 1.40973812 0.36960506
 2.18713051 2.40614008 2.27050547 2.92144086], Cost: 0.1937
Current parameters: [2.29894162 2.37508485 0.77246302 0.86357874 1.59578495 1.05262713
 1.54469559 0.00713406 1.72584718 0.4453335  1.66527186 1.24518896
 1.22727388 2.75681796 1.75713984 2.54133774 1.42383138 0.38920322
 2.19911394 2.41534914 2.26700819 2.92749296], Cost: 0.0300
Current parameters: [2.23084795 2.38478603 0.77505778 0.88668128 1.61363515 1.06133645
 1.55849845 0.01944564 1.6879489  0.36180103 1.65567852 1.244029
 1.21194379 2.7683965  1.74292468 2.54942573 1.43350937 0.39139574
 2.2103527  2.42045181 2.28159844 2.93057837], Cost: 0.1121
Current parameters: [2.30615558 2.38822032 0.77502499 0.86999563 1.60047592 1.05229112
 1.54813989 0.01848258 1.72099258 0.43815647 1.67706308 1.25438974
 1.23270232 2.77855153 1.76391645 2.51495768 1.41030362 0.3779678
 2.17646946 2.44098318 2.24598076 2.93022855], Cost: 0.0719
Current parameters: [ 2.27291276  2.33738658  0.76927247  0.861993    1.60056252  1.04258055
  1.54868122 -0.02136314  1.73723324  0.47199975  1.64967562  1.21082429
```

```
 1.28555687  2.82169003  1.77735225  2.57181129  1.42648906  0.37067224
 2.18555668  2.38761746  2.26951147  2.93419836], Cost: 0.1531
Current parameters: [2.31316223e+00 2.38510189e+00 7.75304519e-01 8.70537219e-01
 1.60212586e+00 1.04540146e+00 1.52819118e+00 1.14212462e-03
 1.74755479e+00 4.32648672e-01 1.67030238e+00 1.24740497e+00
 1.23642670e+00 2.76119068e+00 1.76490708e+00 2.54343617e+00
 1.45606804e+00 4.21139938e-01 2.20168589e+00 2.42776186e+00
 2.27120398e+00 2.93638402e+00], Cost: 0.0152
Current parameters: [2.31071921e+00 2.38952282e+00 7.57675566e-01 8.72319866e-01
 1.60094129e+00 1.02054128e+00 1.52811359e+00 1.11546632e-03
 1.74712131e+00 4.32165305e-01 1.66955576e+00 1.24670930e+00
 1.23484247e+00 2.75887671e+00 1.76458028e+00 2.54323727e+00
 1.45491917e+00 4.18948017e-01 2.20191234e+00 2.42751147e+00
 2.26995717e+00 2.93700597e+00], Cost: 0.0146
Current parameters: [2.31515110e+00 2.39779811e+00 7.08035869e-01 8.76099487e-01
 1.59728870e+00 1.05619498e+00 1.52789865e+00 6.93877751e-04
 1.74944519e+00 4.33445155e-01 1.67121374e+00 1.24635424e+00
 1.23783538e+00 2.76161921e+00 1.76464802e+00 2.54591051e+00
 1.45605862e+00 4.22511789e-01 2.20307320e+00 2.42842363e+00
 2.27159924e+00 2.93861010e+00], Cost: 0.0226
Current parameters: [ 2.31656306e+00  2.39023798e+00  7.57382980e-01  8.73402978e-01
  1.60167509e+00  1.02013840e+00  1.52456524e+00 -1.32046680e-03
  1.75162326e+00  4.28071693e-01  1.65398321e+00  1.26418361e+00
  1.23331527e+00  2.76655471e+00  1.76762142e+00  2.54763315e+00
  1.45406522e+00  4.11200130e-01  2.20940051e+00  2.42880603e+00
  2.26998683e+00  2.92568474e+00], Cost: 0.0304
Current parameters: [2.30991538 2.38339586 0.75767237 0.87510502 1.60690054 1.02002437
 1.5380609  0.00453554 1.743246   0.45012366 1.652966   1.25744499
 1.23237879 2.73131306 1.74813749 2.54812026 1.46444119 0.44863458
 2.18823094 2.42549918 2.24240938 2.93506185], Cost: 0.0633
Current parameters: [2.31131394 2.39370526 0.75913179 0.88863291 1.6129556  1.02058882
 1.5322214  0.00654064 1.74247421 0.44733104 1.66944275 1.24704705
 1.2282301  2.76175582 1.76637588 2.54588062 1.46225174 0.41329139
 2.20616496 2.43076943 2.27702244 2.94164282], Cost: 0.0103
Current parameters: [2.31790493 2.3914579  0.75975245 0.8697674  1.60307488 1.02088173
 1.52445334 0.00544349 1.76239055 0.44914469 1.67798035 1.23782931
 1.19187543 2.75355207 1.75923142 2.54507265 1.48331353 0.39254569
 2.19619956 2.4362505  2.29215567 2.95219692], Cost: 0.0618
Current parameters: [2.30764712 2.39103061 0.75762889 0.8892937  1.61330703 1.02070681
 1.5349163  0.00491309 1.73977739 0.44804302 1.66666279 1.24849787
 1.23531373 2.75134368 1.77655932 2.53897815 1.46773546 0.41196614
```



```
 2.19313237 2.43568998 2.29171326 2.92845337], Cost: 0.0204
Current parameters: [2.32263531 2.40770835 0.76480885 0.89406299 1.62040652 1.01705942
 1.52626412 0.01180089 1.75431591 0.45061662 1.68386354 1.23918136
 1.2393962  2.74833516 1.75300035 2.55761759 1.4270298  0.42558309
 2.22218002 2.43987077 2.30117351 2.93593532], Cost: 0.0359
Current parameters: [ 2.32193628e+00  2.37310962e+00  7.55653482e-01  8.96501709e-01
  1.61878389e+00  1.01932997e+00  1.54146571e+00 -2.52539404e-03
  1.74314653e+00  4.38984207e-01  1.67492081e+00  1.24839221e+00
  1.22547264e+00  2.75866161e+00  1.76627658e+00  2.54551612e+00
  1.45917321e+00  4.12187288e-01  2.20801425e+00  2.43513533e+00
  2.27689967e+00  2.94478520e+00], Cost: 0.0093
Current parameters: [ 2.32935597  2.39308059  0.75986799  0.89273381  1.61686684  1.02007916
  1.53401321 -0.04960061  1.71199331  0.44355593  1.67894287  1.24896531
  1.22290359  2.75421076  1.76752276  2.55013348  1.45963028  0.41086875
  2.21028497  2.44204122  2.27542173  2.95029477], Cost: 0.0333
Current parameters: [ 2.33345465  2.36694666  0.75484116  0.89179302  1.61539377  1.01630461
  1.53915589 -0.00775977  1.7517439   0.45919741  1.6757098   1.24499271
  1.23574796  2.76440121  1.77255786  2.54601816  1.45722301  0.4092169
  2.20419173  2.4309291   2.27346028  2.94497804], Cost: 0.0343
Current parameters: [ 2.28801723  2.36318671  0.75597443  0.90585169  1.63033514  1.01726138
  1.54640377 -0.00721937  1.74076581  0.42002403  1.66978371  1.22874668
  1.24106952  2.78433952  1.76230284  2.56558785  1.4666053   0.41516655
  2.20799118  2.42787436  2.28522365  2.95482175], Cost: 0.0362
Current parameters: [ 2.32120928e+00  2.37383545e+00  7.52513490e-01  8.78483819e-01
  1.64273049e+00  1.01954165e+00  1.54201081e+00 -1.74715592e-03
  1.74267793e+00  4.38787075e-01  1.67592382e+00  1.24891761e+00
  1.22474015e+00  2.75734131e+00  1.77242197e+00  2.54318029e+00
  1.45852013e+00  4.12411715e-01  2.21167245e+00  2.43325777e+00
  2.27554493e+00  2.94537997e+00], Cost: 0.0092
Current parameters: [2.30972092 2.37518925 0.75572034 0.88846891 1.6305916  1.02116479
 1.54621108 0.0048406  1.74228767 0.43222155 1.68439526 1.2483749
 1.21613006 2.74422169 1.81357297 2.53203556 1.45620773 0.42104492
 2.24154355 2.42010195 2.26865956 2.95430119], Cost: 0.0665
Current parameters: [ 2.32016996e+00  2.37386107e+00  7.52869546e-01  8.76833719e-01
  1.64157112e+00  1.01996280e+00  1.54129040e+00 -1.14009558e-03
  1.74358299e+00  4.37989124e-01  1.66785403e+00  1.26058951e+00
  1.23416022e+00  2.75118317e+00  1.77041770e+00  2.53840264e+00
  1.45559784e+00  4.08269904e-01  2.20968028e+00  2.43120867e+00
  2.28344887e+00  2.96813024e+00], Cost: 0.0122
Current parameters: [2.32418909 2.3779774  0.75389101 0.87995613 1.6444757  1.02054189
 1.54462136 0.00341508 1.74129547 0.43224088 1.69017992 1.24627261
```



```
 1.25415182 2.74079602 1.75371163 2.53819421 1.48365594 0.3854695
 2.23329121 2.43223181 2.26256984 2.93669407], Cost: 0.0157
Current parameters: [2.32813855e+00 2.38415011e+00 7.54293686e-01 8.85777210e-01
 1.64778314e+00 1.01924821e+00 1.54188473e+00 1.00006333e-03
 1.73920186e+00 4.38298181e-01 1.67888956e+00 1.25206665e+00
 1.22191599e+00 2.76401788e+00 1.76884480e+00 2.53998608e+00
 1.46135848e+00 4.13058963e-01 2.20688091e+00 2.40887662e+00
 2.27574799e+00 2.94571618e+00], Cost: 0.0095
Current parameters: [ 2.32120928e+00  2.37383545e+00  7.52513490e-01  8.78483819e-01
  1.64273049e+00  1.01954165e+00  1.54201081e+00 -1.74715592e-03
  1.74267793e+00  4.38787075e-01  1.67592382e+00  1.24891761e+00
  1.22474015e+00  2.75734131e+00  1.77242197e+00  2.54318029e+00
  1.45852013e+00  4.12411715e-01  2.21167245e+00  2.43325777e+00
  2.27554493e+00  2.94537997e+00], Cost: 0.0092

   Return from subroutine COBYLA because the MAXFUN limit has been reached.

   NFVALS =  100   F = 9.193973E-03    MAXCV = 0.000000E+00
   X = 2.321209E+00   2.373835E+00   7.525135E-01   8.784838E-01   1.642730E+00
       1.019542E+00   1.542011E+00  -1.747156E-03   1.742678E+00   4.387871E-01
       1.675924E+00   1.248918E+00   1.224740E+00   2.757341E+00   1.772422E+00
       2.543180E+00   1.458520E+00   4.124117E-01   2.211672E+00   2.433258E+00
       2.275545E+00   2.945380E+00
Simulating for QAOA depth p = 12
Current parameters: [1.53555198 2.16812015 0.33943921 1.82708278 3.08962839 2.32813268
 1.22928801 2.57118102 1.75031896 2.41034411 1.05949242 2.76177459
 3.11309916 0.40200544 1.3857883  2.38989761 0.35891279 1.63083702
 0.39194828 0.23451778 2.93536303 0.82108005 2.80817384 1.83235281], Cost: 2.8803
Current parameters: [1.53555198 3.16812015 0.33943921 1.82708278 3.08962839 2.32813268
 1.22928801 2.57118102 1.75031896 2.41034411 1.05949242 2.76177459
 3.11309916 0.40200544 1.3857883  2.38989761 0.35891279 1.63083702
 0.39194828 0.23451778 2.93536303 0.82108005 2.80817384 1.83235281], Cost: 2.1608
Current parameters: [1.53555198 3.16812015 1.33943921 1.82708278 3.08962839 2.32813268
 1.22928801 2.57118102 1.75031896 2.41034411 1.05949242 2.76177459
 3.11309916 0.40200544 1.3857883  2.38989761 0.35891279 1.63083702
 0.39194828 0.23451778 2.93536303 0.82108005 2.80817384 1.83235281], Cost: 2.2786
Current parameters: [1.53555198 3.16812015 0.33943921 2.82708278 3.08962839 2.32813268
 1.22928801 2.57118102 1.75031896 2.41034411 1.05949242 2.76177459
 3.11309916 0.40200544 1.3857883  2.38989761 0.35891279 1.63083702
 0.39194828 0.23451778 2.93536303 0.82108005 2.80817384 1.83235281], Cost: 3.6291
Current parameters: [1.53555198 3.16812015 0.33943921 1.82708278 4.08962839 2.32813268
```



```
 1.22928801 2.57118102 1.75031896 2.41034411 1.05949242 2.76177459
 3.11309916 0.40200544 1.3857883  2.38989761 0.35891279 1.63083702
 0.39194828 0.23451778 2.93536303 0.82108005 2.80817384 1.83235281], Cost: 2.2098
Current parameters: [1.53555198 3.16812015 0.33943921 1.82708278 3.08962839 3.32813268
 1.22928801 2.57118102 1.75031896 2.41034411 1.05949242 2.76177459
 3.11309916 0.40200544 1.3857883  2.38989761 0.35891279 1.63083702
 0.39194828 0.23451778 2.93536303 0.82108005 2.80817384 1.83235281], Cost: 1.9495
Current parameters: [1.53555198 3.16812015 0.33943921 1.82708278 3.08962839 3.32813268
 2.22928801 2.57118102 1.75031896 2.41034411 1.05949242 2.76177459
 3.11309916 0.40200544 1.3857883  2.38989761 0.35891279 1.63083702
 0.39194828 0.23451778 2.93536303 0.82108005 2.80817384 1.83235281], Cost: 2.0723
Current parameters: [1.53555198 3.16812015 0.33943921 1.82708278 3.08962839 3.32813268
 1.22928801 3.57118102 1.75031896 2.41034411 1.05949242 2.76177459
 3.11309916 0.40200544 1.3857883  2.38989761 0.35891279 1.63083702
 0.39194828 0.23451778 2.93536303 0.82108005 2.80817384 1.83235281], Cost: 1.7110
Current parameters: [1.53555198 3.16812015 0.33943921 1.82708278 3.08962839 3.32813268
 1.22928801 3.57118102 2.75031896 2.41034411 1.05949242 2.76177459
 3.11309916 0.40200544 1.3857883  2.38989761 0.35891279 1.63083702
 0.39194828 0.23451778 2.93536303 0.82108005 2.80817384 1.83235281], Cost: 1.4134
Current parameters: [1.53555198 3.16812015 0.33943921 1.82708278 3.08962839 3.32813268
 1.22928801 3.57118102 2.75031896 3.41034411 1.05949242 2.76177459
 3.11309916 0.40200544 1.3857883  2.38989761 0.35891279 1.63083702
 0.39194828 0.23451778 2.93536303 0.82108005 2.80817384 1.83235281], Cost: 2.8202
Current parameters: [1.53555198 3.16812015 0.33943921 1.82708278 3.08962839 3.32813268
 1.22928801 3.57118102 2.75031896 2.41034411 2.05949242 2.76177459
 3.11309916 0.40200544 1.3857883  2.38989761 0.35891279 1.63083702
 0.39194828 0.23451778 2.93536303 0.82108005 2.80817384 1.83235281], Cost: 0.6098
Current parameters: [1.53555198 3.16812015 0.33943921 1.82708278 3.08962839 3.32813268
 1.22928801 3.57118102 2.75031896 2.41034411 2.05949242 3.76177459
 3.11309916 0.40200544 1.3857883  2.38989761 0.35891279 1.63083702
 0.39194828 0.23451778 2.93536303 0.82108005 2.80817384 1.83235281], Cost: 1.8143
Current parameters: [1.53555198 3.16812015 0.33943921 1.82708278 3.08962839 3.32813268
 1.22928801 3.57118102 2.75031896 2.41034411 2.05949242 2.76177459
 4.11309916 0.40200544 1.3857883  2.38989761 0.35891279 1.63083702
 0.39194828 0.23451778 2.93536303 0.82108005 2.80817384 1.83235281], Cost: 2.3184
Current parameters: [1.53555198 3.16812015 0.33943921 1.82708278 3.08962839 3.32813268
 1.22928801 3.57118102 2.75031896 2.41034411 2.05949242 2.76177459
 3.11309916 1.40200544 1.3857883  2.38989761 0.35891279 1.63083702
 0.39194828 0.23451778 2.93536303 0.82108005 2.80817384 1.83235281], Cost: 2.3437
Current parameters: [1.53555198 3.16812015 0.33943921 1.82708278 3.08962839 3.32813268
 1.22928801 3.57118102 2.75031896 2.41034411 2.05949242 2.76177459
```

```
 3.11309916 0.40200544 2.3857883  2.38989761 0.35891279 1.63083702
 0.39194828 0.23451778 2.93536303 0.82108005 2.80817384 1.83235281], Cost: 2.7856
Current parameters: [1.53555198 3.16812015 0.33943921 1.82708278 3.08962839 3.32813268
 1.22928801 3.57118102 2.75031896 2.41034411 2.05949242 2.76177459
 3.11309916 0.40200544 1.3857883  3.38989761 0.35891279 1.63083702
 0.39194828 0.23451778 2.93536303 0.82108005 2.80817384 1.83235281], Cost: 1.1101
Current parameters: [1.53555198 3.16812015 0.33943921 1.82708278 3.08962839 3.32813268
 1.22928801 3.57118102 2.75031896 2.41034411 2.05949242 2.76177459
 3.11309916 0.40200544 1.3857883  2.38989761 1.35891279 1.63083702
 0.39194828 0.23451778 2.93536303 0.82108005 2.80817384 1.83235281], Cost: 1.1273
Current parameters: [1.53555198 3.16812015 0.33943921 1.82708278 3.08962839 3.32813268
 1.22928801 3.57118102 2.75031896 2.41034411 2.05949242 2.76177459
 3.11309916 0.40200544 1.3857883  2.38989761 0.35891279 2.63083702
 0.39194828 0.23451778 2.93536303 0.82108005 2.80817384 1.83235281], Cost: 1.4589
Current parameters: [1.53555198 3.16812015 0.33943921 1.82708278 3.08962839 3.32813268
 1.22928801 3.57118102 2.75031896 2.41034411 2.05949242 2.76177459
 3.11309916 0.40200544 1.3857883  2.38989761 0.35891279 1.63083702
 1.39194828 0.23451778 2.93536303 0.82108005 2.80817384 1.83235281], Cost: 2.1167
Current parameters: [1.53555198 3.16812015 0.33943921 1.82708278 3.08962839 3.32813268
 1.22928801 3.57118102 2.75031896 2.41034411 2.05949242 2.76177459
 3.11309916 0.40200544 1.3857883  2.38989761 0.35891279 1.63083702
 0.39194828 1.23451778 2.93536303 0.82108005 2.80817384 1.83235281], Cost: 2.3854
Current parameters: [1.53555198 3.16812015 0.33943921 1.82708278 3.08962839 3.32813268
 1.22928801 3.57118102 2.75031896 2.41034411 2.05949242 2.76177459
 3.11309916 0.40200544 1.3857883  2.38989761 0.35891279 1.63083702
 0.39194828 0.23451778 3.93536303 0.82108005 2.80817384 1.83235281], Cost: 2.7110
Current parameters: [1.53555198 3.16812015 0.33943921 1.82708278 3.08962839 3.32813268
 1.22928801 3.57118102 2.75031896 2.41034411 2.05949242 2.76177459
 3.11309916 0.40200544 1.3857883  2.38989761 0.35891279 1.63083702
 0.39194828 0.23451778 2.93536303 1.82108005 2.80817384 1.83235281], Cost: 1.2214
Current parameters: [1.53555198 3.16812015 0.33943921 1.82708278 3.08962839 3.32813268
 1.22928801 3.57118102 2.75031896 2.41034411 2.05949242 2.76177459
 3.11309916 0.40200544 1.3857883  2.38989761 0.35891279 1.63083702
 0.39194828 0.23451778 2.93536303 0.82108005 3.80817384 1.83235281], Cost: 2.4811
Current parameters: [1.53555198 3.16812015 0.33943921 1.82708278 3.08962839 3.32813268
 1.22928801 3.57118102 2.75031896 2.41034411 2.05949242 2.76177459
 3.11309916 0.40200544 1.3857883  2.38989761 0.35891279 1.63083702
 0.39194828 0.23451778 2.93536303 0.82108005 2.80817384 2.83235281], Cost: 2.9898
Current parameters: [ 1.58425601  3.28432392  0.32040488  1.58994286  3.08170184  3.36225523
  1.20945556  3.6097013   2.7983757   2.18314146  2.18927407  2.56724828
  2.83715559  0.12198231  1.03439543  2.30910216  0.2753447   1.49370695
```



```
  0.14858203 -0.05224841  2.59601966  0.72231199  2.50594936  1.44797438], Cost: 2.0050
Current parameters: [2.03555198 3.16812015 0.33943921 1.82708278 3.08962839 3.32813268
 1.22928801 3.57118102 2.75031896 2.41034411 2.05949242 2.76177459
 3.11309916 0.40200544 1.3857883  2.38989761 0.35891279 1.63083702
 0.39194828 0.23451778 2.93536303 0.82108005 2.80817384 1.83235281], Cost: 1.0216
Current parameters: [ 1.40355962  3.28344408  0.320549    1.59173837  3.08176185  3.36199687
  1.20960572  3.60940964  2.79801184  2.18486173  2.18829143  2.56872114
  2.8392449   0.1241025   1.037056    2.3097139   0.27597744  1.49474523
  0.15042468 -0.05007715  2.59858901  0.72305982  2.50823766  1.4508847 ], Cost: 1.9975
Current parameters: [1.53555198 3.66812015 0.33943921 1.82708278 3.08962839 3.32813268
 1.22928801 3.57118102 2.75031896 2.41034411 2.05949242 2.76177459
 3.11309916 0.40200544 1.3857883  2.38989761 0.35891279 1.63083702
 0.39194828 0.23451778 2.93536303 0.82108005 2.80817384 1.83235281], Cost: 0.9659
Current parameters: [ 1.40354178  3.05395902  0.32054644  1.59170655  3.08176079  3.36200145
  1.20960306  3.60941481  2.79801829  2.18483124  2.18830884  2.56869504
  2.83920788  0.12406493  1.03700885  2.30970306  0.27596623  1.49472683
  0.15039202 -0.05011563  2.59854347  0.72304656  2.5081971   1.45083312], Cost: 1.5789
Current parameters: [ 1.53555198  3.16812015 -0.16056079  1.82708278  3.08962839  3.32813268
  1.22928801  3.57118102  2.75031896  2.41034411  2.05949242  2.76177459
  3.11309916  0.40200544  1.3857883   2.38989761  0.35891279  1.63083702
  0.39194828  0.23451778  2.93536303  0.82108005  2.80817384  1.83235281], Cost: 0.3854
Current parameters: [ 1.40385866  3.05423306 -0.23232624  1.59227155  3.08177968  3.36192015
  1.20965031  3.60932304  2.79790379  2.18537257  2.18799963  2.56915851
  2.83986533  0.1247321   1.03784606  2.30989556  0.27616533  1.49505355
  0.15097185 -0.0494324   2.59935197  0.72328188  2.50891717  1.45174892], Cost: 1.5399
Current parameters: [ 1.53555198  3.16812015 -0.16056079  1.44787367  3.08962839  3.32813268
  1.22928801  3.57118102  2.75031896  2.41034411  2.05949242  2.76177459
  3.43898326  0.40200544  1.3857883   2.38989761  0.35891279  1.63083702
  0.39194828  0.23451778  2.93536303  0.82108005  2.80817384  1.83235281], Cost: 1.9980
Current parameters: [ 1.49328691  3.13156974 -0.18359288  2.56506082  3.08710946  3.33897628
  1.22298558  3.58342215  2.76559063  2.33814274  2.10073495  2.69995724
  3.71787798  0.31301857  1.27412127  2.36422211  0.33235619  1.58725927
  0.31461038  0.14338807  2.82752514  0.78969314  2.71213173  1.71020349], Cost: 3.2603
Current parameters: [ 1.53555198  3.16812015 -0.16056079  1.82708278  2.5899324   3.32813268
  1.22928801  3.57118102  2.75031896  2.4277773   2.05949242  2.76177459
  3.11309916  0.40200544  1.3857883   2.38989761  0.35891279  1.63083702
  0.39194828  0.23451778  2.93536303  0.82108005  2.80817384  1.83235281], Cost: 0.4580
Current parameters: [ 1.51080225  3.14671683 -0.174048    1.84419832  3.12804649  3.33448252
  1.22559741  3.57834923  2.7592618   3.3864062   2.08364336  2.72557537
  2.98430293  0.3498962   1.32039792  2.37486246  0.34336168  1.60531861
  0.34666048  0.18115372  2.87221493  0.8027004   2.75193316  1.76082418], Cost: 3.4205
```



```
Current parameters: [ 1.51768826  3.15025643 -0.14269707  1.82708278  3.08962839  3.32813268
  1.22928801  3.57118102  3.10695349  2.41034411  1.71178975  2.75284273
  3.11309916  0.39307358  1.37685644  2.38096575  0.34998093  1.62190516
  0.38301642  0.22558592  2.92643117  0.81214819  2.79924198  1.82342095], Cost: 0.5828
Current parameters: [ 1.92696149  3.56995967 -0.67114637  1.8804281   3.09300996  3.3479237
  1.21778522  3.59352272  2.70759839  2.11725617  1.97108451  2.8832241
  2.71166987  0.47386693  1.41625545  2.57731084  0.5447179   1.78557626
  0.4850707   0.30246831  2.97281887  0.9980692   2.86715858  1.84368839], Cost: 0.3194
Current parameters: [ 1.89522309  3.54069576 -0.66768218  1.89308414  3.09381223  3.35261907
  1.21505621  3.59882324  2.71591417  2.04772181  1.99395849  2.84973803
  2.61643179  0.42861623  1.36118405  2.5594745   0.52650003  1.75998809
  0.4448641   0.25628974  2.91940552  0.97775977  2.81885291  2.30904168], Cost: 2.1622
Current parameters: [ 1.92384604  3.56684421 -0.66803091  1.8804281   3.09300996  3.59534436
  1.21778522  3.59352272  2.70837726  2.11725617  1.97186337  2.88166638
  2.71166987  0.4723092   1.45130432  2.57575311  0.54316017  1.78401853
  0.48351297  0.30091058  2.97126115  0.99651147  2.86560085  1.84368839], Cost: 0.8999
Current parameters: [ 1.8966576   3.54169707 -0.66416689  1.89086853  3.09367178  3.23715915
  1.21553396  3.59789531  2.71548881  2.05989477  1.99098452  2.85353937
  2.63310455  0.43447713  1.87440403  2.56053613  0.52762845  1.76240679
  0.44984196  0.26231308  2.92669541  0.97925435  2.82524862  1.7703657 ], Cost: 1.1524
Current parameters: [ 1.92504241  3.56804058 -0.66922728  1.8804281   3.09300996  3.3479237
  0.96867761  3.59352272  2.70807816  2.11725617  1.97156428  2.88226456
  2.71166987  0.47290739  1.41625545  2.5763513   0.54375835  1.78461672
  0.48411116  0.30150876  2.99344905  0.99710966  2.86619904  1.84368839], Cost: 0.0701
Current parameters: [ 1.89323876  3.53817661 -0.65958686  1.89034876  3.09363884  3.31403513
  0.99185593  3.59767762  2.71632787  2.06275047  1.99122568  2.85255345
  2.63701587  0.43397437  1.36926861  2.5589075   0.52601549  1.76109652
  0.44913205  0.26184843  3.46428905  0.97772728  2.82487133  1.77401602], Cost: 0.9819
Current parameters: [ 1.92069001  3.56368819 -0.66487489  1.8804281   3.09300996  3.3479237
  0.96867761  3.83931264  2.70916626  2.11725617  1.97265238  2.88008836
  2.71166987  0.47073119  1.41625545  2.5741751   0.54158216  1.78244052
  0.48193496  0.29933257  2.99344905  0.99493346  2.91081108  1.84368839], Cost: 0.2344
Current parameters: [ 1.89172557  3.53638662 -0.6549109   1.88893304  3.09354909  3.31887117
  0.95043779  3.49548547  2.71666353  2.07052865  1.98993284  2.8537675
  2.6476693   0.43650442  1.37597382  2.55837095  0.52552162  1.7614271
  0.45109786  0.26448227  2.95736964  0.97746738  3.33252747  1.78395855], Cost: 1.0261
Current parameters: [ 1.91590679  3.55890497 -0.66009167  1.8804281   3.09300996  3.3479237
  0.96867761  3.59352272  2.54120901  2.11725617  1.80469513  2.87769675
  2.71166987  0.46833958  1.41625545  2.57178349  0.53919055  1.78004891
  0.47954335  0.22142795  2.99344905  0.99254185  2.86619904  1.84368839], Cost: 0.1094
Current parameters: [ 1.88091254  3.525523   -0.64351975  1.88867427  3.09353269  3.31975513
```



```
  0.95099276  3.5915314   2.60266644  2.0719504   1.87563809  2.84872119
  2.6496166   0.43169866  1.37719945  2.55300466  0.52016313  1.7562193
  0.44618897  0.75310957  2.9584674   0.97215165  2.82945626  1.78577591], Cost: 0.8625
Current parameters: [ 1.92872595  3.57172412 -0.67291082  1.8804281   3.09300996  3.3479237
  0.96867761  3.59352272  2.70807816  2.11725617  1.97156428  2.88410633
  2.71166987  0.63325258  1.41625545  2.57819307  0.54560012  1.78645849
  0.29245586  0.30150876  2.99344905  0.99895143  2.86619904  1.84368839], Cost: 0.2464
Current parameters: [ 1.86804276  3.51306235 -0.63532482  1.89076681  3.09366534  3.31260708
  0.94650505  3.59102608  2.71005536  2.06045362  1.98543406  2.83937354
  2.63386997  0.82019876  1.3672886   2.54624452  0.51333991  1.74817748
  0.78690665  0.26675469  2.94959046  0.96498261  2.82013242  1.77108005], Cost: 1.5635
Current parameters: [ 1.92790643  3.5709046  -0.6720913   1.77032934  3.09938711  3.34704548
  0.96918805  3.59253131  2.7068413   2.30004739  1.96822403  2.88369657
  2.58355544  0.48011447  1.42529941  2.57778331  0.54519036  1.78604873
  0.49037479  0.3088894   3.00218289  0.99854167  2.87397753  1.85358131], Cost: 0.4521
Current parameters: [ 1.99933021  3.64662028 -0.7925554   2.18636408  3.10679967  3.26921566
  0.92376444  3.58401876  2.70703258  2.35202794  1.986852    2.88885282
  2.87693745  0.38497997  1.35062967  2.61008328  0.5768286   1.80490353
  0.45248989  0.25900819  2.93735446  1.02655218  2.80136616  1.73146575], Cost: 0.9639
Current parameters: [ 1.93771214  3.58071032 -0.68189702  1.85096532  3.09202515  3.35216316
  0.96621358  3.59830857  2.71404884  2.08902802  1.98768864  2.66339025
  2.67738603  0.4381167   1.37259763  2.58268617  0.55009322  1.79095158
  0.4538748   0.2658803   2.95128828  1.00344452  2.82865001  1.79593236], Cost: 0.2925
Current parameters: [ 1.88013638  3.5247512  -0.64279339  1.81657372  3.09075743  3.33089629
  0.94442532  3.60418901  2.72545741  1.99223876  2.0253206   3.11803703
  2.56552445  0.33622508  1.2615785   2.55261311  0.51977091  1.75581411
  0.38219483  0.17944368  2.84681857  0.97175575  2.73000497  1.65926046], Cost: 1.4184
Current parameters: [ 1.93661927  3.57961745 -0.68080415  1.8804281   3.09300996  3.3479237
  0.96867761  3.59352272  2.70807816  2.11725617  1.97156428  2.88226456
  2.71166987  0.47290739  1.41625545  2.58213973  0.54954679  1.91548516
  0.48411116  0.30150876  2.99344905  0.78520315  2.86619904  1.84368839], Cost: 0.8063
Current parameters: [ 1.84608008  3.48966087 -0.59692207  1.89737671  3.0935339   3.33603719
  0.9632794   3.59087559  2.70624329  2.11048785  1.96906761  2.88769204
  2.70550071  0.47083607  1.41972574  2.53640698  0.5037242   2.18980204
  0.48839194  0.30584159  2.9977887   1.2507899   2.86804487  1.84199527], Cost: 1.5765
Current parameters: [ 1.92658527  3.56958345 -0.67077015  1.8804281   3.09300996  3.3479237
  0.96867761  3.59352272  2.70807816  2.11725617  1.97156428  2.88226456
  2.71166987  0.47290739  1.41625545  2.39501671  0.71583574  1.78461672
  0.48411116  0.30150876  2.99344905  0.99710966  2.86619904  1.84368839], Cost: 0.0598
Current parameters: [ 1.82666522  3.47001875 -0.57491047  1.89076557  3.09332953  3.34067376
  0.96538508  3.59190816  2.70695902  2.11312797  1.97004149  2.88557494
```

```
  2.70790711  0.47164403  1.41837209  2.71685208  1.05782436  1.77640867
  0.48672213  0.30415149  2.99609594  1.00512571  2.86732486  1.8426557 ], Cost: 2.9828
Current parameters: [ 2.09474505  3.3852952  -0.68689862  1.8804281   3.09300996  3.3479237
  0.96867761  3.59352272  2.70807816  2.11725617  1.97156428  2.88226456
  2.71166987  0.47290739  1.41625545  2.39501671  0.71583574  1.78461672
  0.48411116  0.30150876  2.99344905  0.99710966  2.86619904  1.84368839], Cost: 0.1378
Current parameters: [ 2.18657929  3.77749342 -0.3009708   1.90365912  3.09372811  3.33163116
  0.96127842  3.58989436  2.70556315  2.10797901  1.96814216  2.88970387
  2.70321396  0.47006829  1.42101209  2.37972507  0.69927212  1.76617107
  0.48997871  0.30744766  2.9993973   1.01512385  2.86872907  1.84136768], Cost: 1.3679
Current parameters: [ 1.935451    3.57844918 -0.67963587  1.8573979   3.09300996  3.3479237
  0.96867761  3.59352272  2.5310812   2.11725617  2.14412838  2.88669742
  2.73146152  0.47734025  1.42068831  2.39944957  0.7202686   1.78904958
  0.48854402  0.30594163  2.99788191  1.00154252  2.8706319   1.84812125], Cost: 0.0933
Current parameters: [ 1.99081331  3.65227388 -0.83391346  1.56984838  3.09533094  3.29526752
  0.94476404  3.58179616  2.74656203  2.08727314  1.91505946  2.90514041
  3.01576932  0.46256426  1.43046114  2.34442799  0.66113607  1.72383451
  0.50190721  0.31953539  3.01150593  1.05416267  2.87320849  1.83502065], Cost: 1.2209
Current parameters: [ 1.92409707  3.56713572 -0.66874434  1.88063508  3.34217763  3.34543024
  0.96863298  3.59360941  2.70852256  2.10742661  1.97261453  2.88073236
  2.71011235  0.47118278  1.4325144   2.39374044  0.71455323  1.78321367
  0.48246905  0.29976899  2.99159096  0.99579294  2.86442448  1.84179175], Cost: 0.3034
Current parameters: [ 1.89682548  3.55124386 -0.70880973  1.93973484  3.04459717  3.25130966
  0.95409516  3.58929575  2.71539586  2.07160161  1.99375961  2.85445668
  2.64435943  0.42133084  1.86272698  2.3329952   0.65134597  1.71347749
  0.45050106  0.26539749  2.95416833  0.99422846  2.82298641  1.78841628], Cost: 0.6511
Current parameters: [ 2.02413242  3.66975971 -0.79835829  1.89387499  3.09386236  3.35291247
  0.96577807  3.59915445  2.69747481  2.04337679  1.94967197  2.91232044
  2.61048062  0.49046349  1.42337718  2.44170022  0.76211388  1.82306401
  0.50702655  0.31807902  3.00233244  1.04116554  2.88050927  1.85069117], Cost: 0.5066
Current parameters: [ 1.88303957  3.54315992 -0.71889387  1.96948872  3.0752674   3.3057252
  0.94649788  3.58261746  2.71590276  2.09042918  1.99852403  2.85199553
  2.67001648  0.41186265  1.33083254  2.2965049   0.61351065  1.6766792
  0.4491574   0.266795    2.95879489  0.99849936  2.82127069  2.27006298], Cost: 1.6371
Current parameters: [ 1.95579258  3.6005038  -0.71955112  2.0264241   3.09806436  3.35067612
  0.91275376  3.54437482  2.74127005  2.19807612  2.0084357   2.9226884
  2.80542893  0.43362507  1.40727957  2.44785963  0.69156098  1.74147659
  0.52492851  0.30872506  2.98816886  1.04133604  2.84508438  1.81225223], Cost: 0.5668
Current parameters: [ 1.9196696   3.58344051 -0.7692228   1.96677013  3.07736032  3.297329
  1.09805599  3.72946873  2.59597729  2.31620996  1.88141801  2.76505308
  2.67048805  0.53218143  1.31427891  2.1805086   0.71865249  1.82293534
```



```
  0.35254284  0.25851434  2.97597138  0.93032736  2.88894741  1.66193788], Cost: 0.7958
Current parameters: [ 1.90767694  3.55352303 -0.66788564  1.88930362  3.08939175  3.34211212
  0.95422653  3.53777038  2.719222    2.08529223  1.98627868  2.87740594
  2.69323434  0.44929881  1.4009471   2.38041169  0.68674566  1.75177215
  0.47878011  0.290526    2.97994525  0.99700204  3.09559928  1.81502376], Cost: 0.3002
Current parameters: [ 1.93085957  3.57370033 -0.67324568  1.87726509  3.09303822  3.34750262
  0.97274858  3.59670946  2.72946719  2.12395264  1.99221246  2.88281205
  2.7149851   0.51877482  1.41881187  2.39445557  0.7207783   1.79100942
  0.51774782  0.19538738  2.99512123  0.99719812  2.86965589  1.84847138], Cost: 0.1144
Current parameters: [ 1.88784178  3.5358811  -0.65857471  1.89060222  3.08627111  3.33616721
  0.94998009  3.58409743  2.69438264  2.07069446  1.96312543  2.86811672
  2.68334675  0.61377331  1.39248484  2.36032926  0.66537624  1.73007495
  0.61822202  0.38621329  2.97147234  0.99101655  2.83845219  1.79922883], Cost: 0.7686
Current parameters: [ 1.92196649  3.56475623 -0.66376971  1.89539347  3.09347218  3.34493881
  0.97164133  3.59139576  2.70377212  2.13829905  1.9604637   2.84051619
  2.73834689  0.49778472  1.44463706  2.39181937  0.71471535  1.85961299
  0.50341837  0.32431596  3.01820585  1.04070262  2.89160028  1.87736458], Cost: 0.6400
Current parameters: [ 1.88027662  3.5285207  -0.64749803  1.85985457  3.08413509  3.3352951
  0.94667034  3.58508548  2.72229954  2.06495354  1.99651646  2.98095755
  2.67311248  0.40960918  1.36575024  2.35286468  0.65774935  1.88155537
  0.45140215  0.26363507  2.93494571  1.08699394  2.81702686  1.77590967], Cost: 0.4424
Current parameters: [ 1.90802832  3.55403044 -0.66993697  1.88780947  3.08935996  3.34283716
  0.97541327  3.58363474  2.71999126  2.07944103  1.98863122  2.88365087
  2.6850526   0.44128915  1.39470602  2.38042301  0.68671259  1.75217043
  0.47223681  0.28416519  3.22392128  0.99748216  2.8423037   1.80634835], Cost: 0.5397
Current parameters: [ 1.91902186  3.56443397 -0.67732407  1.888987    3.08132335  3.32497663
  0.9673662   3.59508537  2.70896561  2.10677545  1.97658016  2.87715546
  2.69407953  0.45667077  1.52534685  2.37951863  0.70296163  1.76059881
  0.46942775  0.28987392  2.97792641  0.98821566  2.85375512  1.82599052], Cost: 0.1359
Current parameters: [ 1.94130659  3.58464894 -0.68942399  1.84812976  3.09326601  3.34797465
  0.9664134   3.59167291  2.71301541  2.11528269  1.96732977  2.8846591
  2.74197065  0.47158685  1.41843407  2.37452403  0.69062316  1.78105775
  0.48636207  0.3038768   2.99523291  0.99889448  2.86678307  1.84293929], Cost: 0.0946
Current parameters: [ 1.90516347  3.55434031 -0.67369786  1.82594586  3.08371761  3.326508
  0.97337055  3.60356015  2.70556516  2.11351876  1.95786385  2.89090194
  2.74602169  0.46116686  1.40703783  2.4400195   0.78673008  1.74679598
  0.46291881  0.29262088  2.97604681  0.98996893  2.86177016  1.82884077], Cost: 0.0995
Current parameters: [ 1.89977583  3.54866044 -0.71122072  1.88436014  3.09318147  3.34844765
  0.96782305  3.59290906  2.70906154  2.11575539  1.97413062  2.87981617
  2.70827003  0.46897508  1.41029039  2.39073782  0.71023362  1.78009048
  0.48153509  0.29890831  2.99055484  0.99458724  2.86292552  1.87384089], Cost: 0.0311
```



```
Current parameters: [ 1.92466059  3.57108562 -0.66617504  1.89025901  3.08529624  3.33063407
  0.9694436   3.60009341  2.70474248  2.10784953  1.97420069  2.88060211
  2.69196643  0.44678504  1.38450352  2.36803896  0.70368488  1.73246387
  0.45496003  0.28339351  2.96644601  0.98085989  2.84937689  1.94982548], Cost: 0.3051
Current parameters: [ 1.90262881  3.55233707 -0.72348486  1.89413317  3.09425008  3.30318653
  0.97778903  3.6029134   2.70087622  2.13387372  1.96811933  2.8715691
  2.70423261  0.47352311  1.3959696   2.37635242  0.70954736  1.785865
  0.47231278  0.29659198  2.98943737  0.98687892  2.86488888  1.85795488], Cost: 0.0275
Current parameters: [ 1.89823185  3.55267532 -0.74334516  1.90279217  3.09011279  3.38276179
  1.00246302  3.63073005  2.67986613  2.1687679   1.95154754  2.85738019
  2.69485819  0.47472194  1.3852954   2.34256402  0.72042073  1.77474024
  0.43709067  0.28273226  2.97297314  0.9641414   2.86395966  1.8197353 ], Cost: 0.0892
Current parameters: [ 1.90115106  3.55085932 -0.72200712  1.89961613  3.09403823  3.30305448
  0.96807909  3.65946341  2.70751588  2.1278014   1.97444272  2.87967916
  2.71061275  0.46794705  1.39732948  2.38276795  0.70207839  1.78578697
  0.48042391  0.30030818  2.99156112  0.98507295  2.87631193  1.85944241], Cost: 0.0146
Current parameters: [ 1.90063804  3.55241449 -0.72470274  1.90123801  3.0893162   3.30435458
  0.95378129  3.64501624  2.71551842  2.11526813  1.98183537  2.89768991
  2.7211141   0.45431901  1.39616322  2.39591906  0.70198712  1.83457935
  0.4854326   0.30241316  2.98634292  0.87714148  2.87215739  1.85849651], Cost: 0.2209
Current parameters: [ 1.89851393  3.54822219 -0.71936998  1.89623584  3.09483522  3.30202801
  0.97695224  3.65830466  2.70051011  2.15064576  1.96497852  2.92864185
  2.70667933  0.48157819  1.40789999  2.37539894  0.70645143  1.78444277
  0.48125719  0.30630582  3.00108372  0.98372875  2.88543525  1.87100519], Cost: 0.0702
Current parameters: [ 1.90135711  3.55203355 -0.72371523  1.84678497  3.09457793  3.30382389
  0.93646359  3.66178358  2.72820781  2.20076531  1.99523424  2.86007866
  2.65316926  0.44454688  1.39512769  2.40729818  0.68518454  1.78133913
  0.50100513  0.30774957  2.98957549  0.9828641   2.87446301  1.85718835], Cost: 0.2570
Current parameters: [ 1.90031671  3.55002497 -0.72117276  1.89961613  3.09403823  3.30305448
  0.93716551  3.65946341  2.69040616  2.1278014   1.95733299  2.87935771
  2.71061275  0.49065679  1.39732948  2.40494963  0.68040261  1.78546552
  0.45198657  0.29194955  2.99412127  0.98475151  2.87619286  1.85944241], Cost: 0.0247
Current parameters: [ 1.90495558  3.55504212 -0.72639851  1.89845984  3.09322076  3.30334379
  0.97599885  3.66035716  2.7707343   2.12683327  2.03767297  2.88221241
  2.71084279  0.52018204  1.39658565  2.37939927  0.71127605  1.78584842
  0.41878186  0.32987391  2.98921944  0.98600919  2.87576774  1.85868909], Cost: 0.0485
Current parameters: [ 1.86248286  3.59329074 -0.7183219   1.89961613  3.09403823  3.30305448
  0.98529479  3.65946341  2.70748273  2.1278014   1.97440956  2.87974547
  2.71061275  0.46801337  1.39732948  2.3953662   0.69025252  1.78585328
  0.48049022  0.3003745   2.99013537  0.98513926  2.87637825  1.85944241], Cost: 0.0184
Current parameters: [ 1.93244399  3.51345098 -0.72453664  1.89723791  3.09235689  3.30364952
```



```
  1.05057945  3.66130163  2.70664194  2.12581018  1.97359312  2.88034869
  2.71108589  0.46618159  1.39579961  2.44186912  0.64948718  1.78137258
  0.47686601  0.29861533  2.98126146  0.9824578   2.87544771  1.85789301], Cost: 0.0044
Current parameters: [ 1.92185803  3.50323194 -0.71386157  1.86937055  3.09127798  3.30149643
  1.05923865  3.67001925  2.69796736  2.10268774  1.96499665  2.87046108
  2.68772595  0.49393636  1.39444169  2.42675512  0.64771677  1.78278668
  0.48755226  0.30872325  2.97935269  0.97300396  2.87548867  1.85814253], Cost: 0.0038
Current parameters: [ 1.91002423  3.49252844 -0.72426493  1.91209198  3.08448388  3.3004123
  1.04229458  3.65871368  2.69952825  2.0990811   1.97047692  2.88058353
  2.70809003  0.53194478  1.37397102  2.43202047  0.63195723  1.7220933
  0.54105536  0.33719109  2.95030344  0.97188336  2.84939544  1.82086354], Cost: 0.2133
Current parameters: [ 1.90064618  3.48195674 -0.68954528  1.86835324  3.09108403  3.29976927
  1.06108425  3.66754498  2.70167004  2.11146428  1.97155312  2.86365299
  2.70346493  0.48655322  1.39056454  2.41580256  0.63471249  1.77128618
  0.48193871  0.30330943  3.01508674  0.96500654  2.86912458  1.85198311], Cost: 0.0522
Current parameters: [ 1.94602964  3.52825979 -0.76059938  1.87543965  3.08455172  3.30378747
  1.06694811  3.67388776  2.69632023  2.05593682  1.96132917  2.88214443
  2.64680653  0.48366348  1.38321161  2.44173863  0.66348481  1.7629778
  0.48223966  0.30340705  3.05928415  0.97995236  2.86488908  1.83937692], Cost: 0.0355
Current parameters: [ 1.9176513   3.4995368  -0.71179198  1.86766369  3.03399283  3.30070572
  1.05713297  3.66910296  2.69915751  2.10027532  1.96707594  2.87954202
  2.68498126  0.48879952  1.38650282  2.42271728  0.64215354  1.79500595
  0.48527876  0.30567808  2.97460546  0.98358088  2.87166852  1.85273515], Cost: 0.0100
Current parameters: [ 1.90030332  3.48321837 -0.71023168  1.8604419   3.13837778  3.29711835
  1.05494079  3.67102785  2.70185101  2.06658141  1.9735242   2.91532669
  2.6702705   0.46184326  1.37116901  2.41200537  0.63137343  1.8162469
  0.46600269  0.28747107  2.94901276  1.01330889  2.8491529   1.82112389], Cost: 0.1717
Current parameters: [ 1.91901206  3.50048405 -0.71156749  1.87537744  3.09115336  3.30129627
  1.05874093  3.66809904  2.74216734  2.10158684  1.92278465  2.86919637
  2.68219151  0.49202587  1.39281707  2.42515473  0.64561748  1.78055809
  0.48627128  0.30724762  2.97779023  0.97190288  2.88229226  1.8560579 ], Cost: 0.0011
Current parameters: [ 1.91054009  3.49308621 -0.71409888  1.87758586  3.08888539  3.29934715
  1.05372956  3.64252065  2.73441348  2.08055136  1.94129874  2.86928799
  2.67323045  0.47799827  1.38260817  2.42032568  0.63576658  1.75639457
  0.48025644  0.30001335  2.96882663  0.96966966  2.99352438  1.83812622], Cost: 0.1787
Current parameters: [ 1.93180383  3.48555389 -0.71160209  1.8852431   3.09052031  3.30157017
  1.05280869  3.69532542  2.74447718  2.08557855  1.92507339  2.84610962
  2.69565996  0.47374861  1.39275183  2.40586209  0.66441372  1.78322452
  0.50563445  0.30721675  2.97701995  0.98621592  2.88638473  1.85667777], Cost: 0.0041
Current parameters: [ 1.90330251  3.50558574 -0.72666381  1.88753448  3.08887523  3.30382219
  1.08733162  3.66087635  2.72617591  2.11163452  1.89251404  2.83531349
```

```
  2.70765734  0.53082815  1.4189336   2.45429021  0.66362887  1.84305851
  0.47316718  0.33019426  3.00100518  1.01438873  2.88581877  1.88258738], Cost: 0.3374
Current parameters: [ 1.93290477  3.51364743 -0.70415357  1.86191049  3.10867156  3.29899385
  1.06587907  3.67615263  2.71692564  2.09531993  1.93109994  2.85820204
  2.67039836  0.50515466  1.3964794   2.42004311  0.65294859  1.77443113
  0.48450345  0.31115979  2.95662029  0.96029893  2.88616133  1.8868916 ], Cost: 0.0333
Current parameters: [ 1.93312447  3.51660085 -0.67874851  1.90978622  3.05689382  3.29035352
  1.06461094  3.66799282  2.76680211  2.09166812  1.90141575  2.88047645
  2.68073006  0.45580773  1.35733451  2.41078794  0.63115006  1.7361905
  0.44801193  0.28330419  2.98237018  0.97932779  2.85070258  1.9031728 ], Cost: 0.3376
Current parameters: [ 1.91461035  3.49793147 -0.71656469  1.88000932  3.08489111  3.29027418
  1.05969929  3.66927406  2.74223639  2.09531726  1.92388815  2.86631808
  2.67413696  0.48460261  1.44732294  2.41922094  0.64112663  1.76845292
  0.4775726   0.3010695   2.97038764  0.96831787  2.87479515  1.84648499], Cost: 0.0281

   Return from subroutine COBYLA because the MAXFUN limit has been reached.

   NFVALS =  100   F = 1.061014E-03    MAXCV = 0.000000E+00
   X = 1.919012E+00   3.500484E+00  -7.115675E-01   1.875377E+00   3.091153E+00
       3.301296E+00   1.058741E+00   3.668099E+00   2.742167E+00   2.101587E+00
       1.922785E+00   2.869196E+00   2.682192E+00   4.920259E-01   1.392817E+00
       2.425155E+00   6.456175E-01   1.780558E+00   4.862713E-01   3.072476E-01
       2.977790E+00   9.719029E-01   2.882292E+00   1.856058E+00
Current parameters: [ 1.91901206  3.50048405 -0.71156749  1.87537744  3.09115336  3.30129627
  1.05874093  3.66809904  2.74216734  2.10158684  1.92278465  2.86919637
  2.68219151  0.49202587  1.39281707  2.42515473  0.64561748  1.78055809
  0.48627128  0.30724762  2.97779023  0.97190288  2.88229226  1.8560579 ], Cost: 0.0011
```


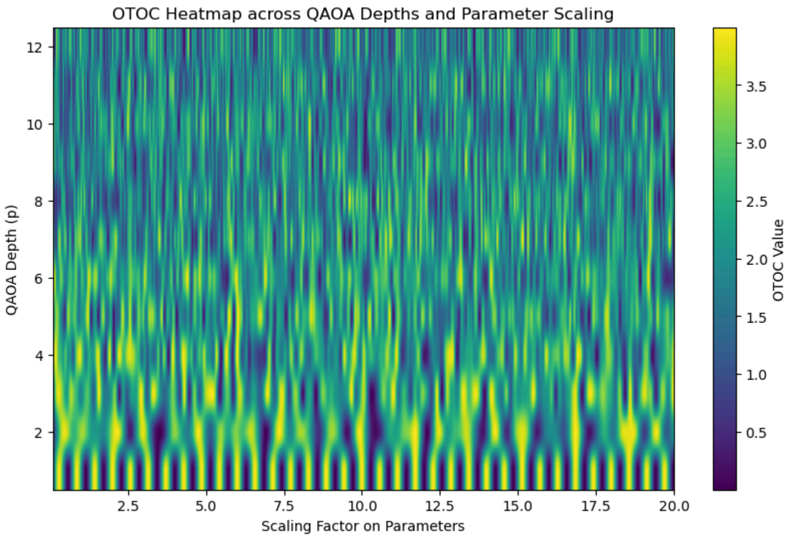


```
Run 6/30 for heatmap generation...
Simulating for QAOA depth p = 1
Current parameters: [2.71095184 0.71672251], Cost: 1.7942
Current parameters: [2.71095184 1.71672251], Cost: 1.5819
Current parameters: [3.23532296 2.56821234], Cost: 1.8602
Current parameters: [2.22503397 1.83455214], Cost: 1.1598
Current parameters: [1.74110726 1.96030962], Cost: 1.6660
Current parameters: [2.28394878 2.07751107], Cost: 1.1116
Current parameters: [1.83655642 2.30075998], Cost: 1.8885
Current parameters: [2.53213452 2.04744719], Cost: 1.1137
Current parameters: [2.29352672 2.20214359], Cost: 1.4266
Current parameters: [2.27622192 2.01549055], Cost: 1.0340
Current parameters: [2.26827706 1.95349757], Cost: 1.0162
Current parameters: [2.22181374 1.91169554], Cost: 1.0565
Current parameters: [2.32719293 1.93263682], Cost: 1.0093
Current parameters: [2.32881738 1.90142907], Cost: 1.0321
Current parameters: [2.34140248 1.96046937], Cost: 1.0005
Current parameters: [2.31858436 1.98182109], Cost: 1.0055
Current parameters: [2.35593159 1.96621789], Cost: 1.0001
Current parameters: [2.36002841 1.95956573], Cost: 1.0002
Current parameters: [2.36069216 1.97241242], Cost: 1.0007
Current parameters: [2.35236233 1.96463071], Cost: 1.0000
Current parameters: [2.35091315 1.96594014], Cost: 1.0001
Current parameters: [2.35291387 1.96275708], Cost: 1.0000
Current parameters: [2.35484537 1.96304691], Cost: 1.0000
Current parameters: [2.35668432 1.96238892], Cost: 1.0000
Current parameters: [2.35508612 1.96399333], Cost: 1.0000
Current parameters: [2.35467461 1.96425616], Cost: 1.0000
Current parameters: [2.35556724 1.96391003], Cost: 1.0000
Current parameters: [2.3556368  1.96342673], Cost: 1.0000
Current parameters: [2.3560595  1.96318229], Cost: 1.0000
Current parameters: [2.35549833 1.96322566], Cost: 1.0000
Current parameters: [2.35567896 1.96351741], Cost: 1.0000
Current parameters: [2.35570468 1.96361404], Cost: 1.0000
Current parameters: [2.35563362 1.96353849], Cost: 1.0000
Current parameters: [2.3557786  1.96352589], Cost: 1.0000
Current parameters: [2.35586662 1.96347843], Cost: 1.0000
Current parameters: [2.35596359 1.96350287], Cost: 1.0000
Current parameters: [2.35605618 1.96346509], Cost: 1.0000
Current parameters: [2.35613621 1.96352505], Cost: 1.0000
Current parameters: [2.35622892 1.96348757], Cost: 1.0000
```



```
Current parameters: [2.35629553 1.96356216], Cost: 1.0000
Current parameters: [2.35629553 1.96356216], Cost: 1.0000

   Normal return from subroutine COBYLA

   NFVALS =   41   F = 1.000000E+00    MAXCV = 0.000000E+00
   X = 2.356296E+00   1.963562E+00
Simulating for QAOA depth p = 2
Current parameters: [4.06758993 1.79751032 2.77555232 1.55674302], Cost: 1.0576
Current parameters: [4.06758993 2.79751032 2.77555232 1.55674302], Cost: 1.0527
Current parameters: [4.06758993 2.79751032 3.77555232 1.55674302], Cost: 2.6118
Current parameters: [4.06758993 2.79751032 2.77555232 2.55674302], Cost: 3.2407
Current parameters: [4.45022587 2.79919408 2.23940349 0.80432603], Cost: 1.3645
Current parameters: [3.59834494 2.79782497 2.67535977 1.41613546], Cost: 1.8088
Current parameters: [4.24143758 2.79783774 2.67129482 1.41043082], Cost: 1.7455
Current parameters: [4.06758993 2.9225097  2.77594488 1.55674302], Cost: 1.0484
Current parameters: [4.07106958 2.9225846  3.02009023 1.50306871], Cost: 1.3431
Current parameters: [4.07469945 2.9242471  2.77027549 1.80657155], Cost: 1.4917
Current parameters: [3.94969535 2.92324517 2.74806412 1.52595358], Cost: 1.0565
Current parameters: [4.11202722 2.92438665 2.69858639 1.46920749], Cost: 1.3229
Current parameters: [4.05522107 2.92318156 2.76681268 1.6173187 ], Cost: 1.0415
Current parameters: [4.04733799 2.92308704 2.79690974 1.62024745], Cost: 1.0806
Current parameters: [4.07277801 2.92507714 2.70685958 1.61751066], Cost: 1.0128
Current parameters: [4.09295646 2.92985026 2.64885271 1.62807004], Cost: 1.0258
Current parameters: [4.1019449  2.92505025 2.7154233  1.62475754], Cost: 1.0341
Current parameters: [4.03318655 2.92782622 2.65904378 1.6108131 ], Cost: 1.0091
Current parameters: [4.03209676 2.95891917 2.66151173 1.61239822], Cost: 1.0087
Current parameters: [3.99473259 2.95386387 2.68379667 1.65698514], Cost: 0.9569
Current parameters: [3.94452844 2.94969335 2.72076849 1.65820376], Cost: 0.9715
Current parameters: [3.99811299 2.95253983 2.66902945 1.71760692], Cost: 0.9611
Current parameters: [3.99601263 2.98508303 2.68349073 1.65654429], Cost: 0.9656
Current parameters: [3.98553623 2.95408434 2.67141612 1.65448692], Cost: 0.9494
Current parameters: [3.98209884 2.93983922 2.64563972 1.64461774], Cost: 0.9530
Current parameters: [3.97074159 2.95176861 2.67574171 1.65557592], Cost: 0.9452
Current parameters: [3.96952744 2.95087885 2.67124756 1.67046475], Cost: 0.9383
Current parameters: [3.96203121 2.96206516 2.66604673 1.67644514], Cost: 0.9360
Current parameters: [3.95468678 2.95405149 2.65678108 1.68277985], Cost: 0.9250
Current parameters: [3.94732959 2.94604166 2.64756946 1.68918308], Cost: 0.9139
Current parameters: [3.94007162 2.93784116 2.63835669 1.69545517], Cost: 0.9027
Current parameters: [3.93297339 2.92929913 2.62926678 1.70163278], Cost: 0.8913
Current parameters: [3.92602838 2.92042683 2.62034562 1.70776489], Cost: 0.8799
```

```
Current parameters: [3.91923093 2.9112421  2.61161482 1.71387629], Cost: 0.8684
Current parameters: [3.91256979 2.90177659 2.60307678 1.71998167], Cost: 0.8569
Current parameters: [3.9060167  2.89209446 2.59470133 1.72608954], Cost: 0.8453
Current parameters: [3.89951146 2.88231798 2.58640131 1.7322012 ], Cost: 0.8338
Current parameters: [3.89292704 2.87269995 2.57797132 1.7383016 ], Cost: 0.8224
Current parameters: [3.8859228  2.86401528 2.56881966 1.74429367], Cost: 0.8115
Current parameters: [3.87745327 2.86549166 2.55619005 1.74756795], Cost: 0.8076
Current parameters: [3.86494106 2.86047896 2.56233936 1.75253224], Cost: 0.8015
Current parameters: [3.86654467 2.856701   2.56131782 1.76757393], Cost: 0.7892
Current parameters: [3.8654223  2.84670431 2.55859558 1.77921594], Cost: 0.7780
Current parameters: [3.86308624 2.84320161 2.55776401 1.79424002], Cost: 0.7727
Current parameters: [3.85379042 2.83069709 2.55892206 1.79408466], Cost: 0.7676
Current parameters: [3.85266786 2.82664926 2.5439101  1.79515083], Cost: 0.7548
Current parameters: [3.85793667 2.81588717 2.53431395 1.79806145], Cost: 0.7440
Current parameters: [3.8551969  2.80914551 2.52100981 1.80182764], Cost: 0.7339
Current parameters: [3.85290402 2.79536364 2.51463283 1.8047047 ], Cost: 0.7254
Current parameters: [3.85624818 2.79151262 2.50858417 1.8181784 ], Cost: 0.7145
Current parameters: [3.86035436 2.78523752 2.4980236  1.82691782], Cost: 0.7042
Current parameters: [3.86068696 2.77365245 2.48935224 1.83280199], Cost: 0.6951
Current parameters: [3.86795835 2.77317544 2.4772292  1.83944066], Cost: 0.6874
Current parameters: [3.86185303 2.77194979 2.47061133 1.85215157], Cost: 0.6785
Current parameters: [3.86576879 2.7655291  2.46886065 1.86573529], Cost: 0.6729
Current parameters: [3.86148407 2.76085397 2.45611563 1.87217652], Cost: 0.6631
Current parameters: [3.85801116 2.75981013 2.44372805 1.88098206], Cost: 0.6543
Current parameters: [3.85800103 2.76040731 2.43533044 1.89414505], Cost: 0.6472
Current parameters: [3.84993921 2.75309882 2.4268906  1.90152765], Cost: 0.6408
Current parameters: [3.85559988 2.74764186 2.41353217 1.90349521], Cost: 0.6327
Current parameters: [3.85485422 2.74781113 2.39843706 1.90745673], Cost: 0.6259
Current parameters: [3.85872808 2.74028837 2.38816765 1.91564704], Cost: 0.6186
Current parameters: [3.85862876 2.73074933 2.375809   1.91501348], Cost: 0.6175
Current parameters: [3.87028456 2.73638574 2.36958251 1.92115713], Cost: 0.6107
Current parameters: [3.86982924 2.73976329 2.36444787 1.93551544], Cost: 0.6011
Current parameters: [3.87436573 2.73933558 2.36188337 1.95023961], Cost: 0.5946
Current parameters: [3.87278683 2.74838699 2.35255448 1.95876554], Cost: 0.5881
Current parameters: [3.86335648 2.74124404 2.34499529 1.96562456], Cost: 0.5849
Current parameters: [3.85559954 2.74746253 2.35190624 1.97550077], Cost: 0.5911
Current parameters: [3.86843452 2.73836759 2.34600167 1.97071984], Cost: 0.5841
Current parameters: [3.87361529 2.73838959 2.3312948  1.97172369], Cost: 0.5752
Current parameters: [3.8786537  2.73480481 2.31694686 1.97152099], Cost: 0.5699
Current parameters: [3.88642777 2.72217708 2.3176542  1.97639383], Cost: 0.5674
Current parameters: [3.89141831 2.72519515 2.31017183 1.98880917], Cost: 0.5585
```



```
Current parameters: [3.90268704 2.73008817 2.30413934 1.99634733], Cost: 0.5505
Current parameters: [3.90886446 2.73243604 2.29557094 2.00761897], Cost: 0.5420
Current parameters: [3.91667195 2.7342291  2.28733288 2.01820686], Cost: 0.5336
Current parameters: [3.92792127 2.73582781 2.28050699 2.02647998], Cost: 0.5256
Current parameters: [3.93396169 2.73733435 2.27180149 2.03786412], Cost: 0.5175
Current parameters: [3.94400802 2.7385239  2.26431468 2.04712395], Cost: 0.5094
Current parameters: [3.94791997 2.7395779  2.25524485 2.05918482], Cost: 0.5021
Current parameters: [3.95883119 2.74052443 2.24815603 2.06778359], Cost: 0.4942
Current parameters: [3.96575844 2.74112229 2.23956122 2.07882558], Cost: 0.4865
Current parameters: [3.97943314 2.74188813 2.23448086 2.08437053], Cost: 0.4790
Current parameters: [3.98888883 2.74236748 2.2266795  2.09404729], Cost: 0.4711
Current parameters: [3.98420743 2.74313677 2.21815591 2.10625309], Cost: 0.4680
Current parameters: [3.9925037  2.74832665 2.22312221 2.11737574], Cost: 0.4802
Current parameters: [3.98408843 2.7502518  2.21576509 2.10408945], Cost: 0.4653
Current parameters: [3.98711066 2.74764046 2.2018424  2.09822889], Cost: 0.4499
Current parameters: [3.98385076 2.74642383 2.18829123 2.09127192], Cost: 0.4453
Current parameters: [3.99743678 2.74796355 2.18103225 2.09339251], Cost: 0.4378
Current parameters: [3.9982037  2.76312525 2.17752813 2.09221078], Cost: 0.4420
Current parameters: [4.00002672 2.74796501 2.18389584 2.0866008 ], Cost: 0.4413
Current parameters: [4.00208473 2.74047233 2.17034128 2.10061207], Cost: 0.4286
Current parameters: [4.0104439  2.73587203 2.16987959 2.1129769 ], Cost: 0.4190
Current parameters: [4.01569882 2.72891976 2.16495745 2.12497543], Cost: 0.4106
Current parameters: [4.03006045 2.7234584  2.1621456  2.12458658], Cost: 0.4025
Current parameters: [4.03787863 2.73280532 2.15423007 2.13033079], Cost: 0.3961
Current parameters: [4.05031345 2.73127722 2.15241574 2.13948989], Cost: 0.3883
Current parameters: [4.05866983 2.72680574 2.14068335 2.14357258], Cost: 0.3770
Current parameters: [4.05031345 2.73127722 2.15241574 2.13948989], Cost: 0.3883

   Return from subroutine COBYLA because the MAXFUN limit has been reached.

   NFVALS =  100   F = 3.883029E-01    MAXCV = 0.000000E+00
   X = 4.050313E+00   2.731277E+00   2.152416E+00   2.139490E+00
Simulating for QAOA depth p = 3
Current parameters: [1.66567349 1.59515237 0.03411297 2.73702122 0.34541825 2.095056  ], Cost: 2.3591
Current parameters: [0.66567349 2.59515237 0.03411297 2.73702122 0.34541825 2.095056  ], Cost: 3.3151
Current parameters: [0.66567349 1.59515237 1.03411297 2.73702122 0.34541825 2.095056  ], Cost: 3.2043
Current parameters: [0.66567349 1.59515237 0.03411297 3.73702122 0.34541825 2.095056  ], Cost: 2.4431
Current parameters: [0.66567349 1.59515237 0.03411297 2.73702122 1.34541825 2.095056  ], Cost: 0.9909
Current parameters: [0.66567349 1.59515237 0.03411297 2.73702122 1.34541825 3.095056  ], Cost: 1.0354
Current parameters: [ 0.31752768  0.97199348 -0.55717645  2.36471459  1.39089748  2.08225988], Cost: 2.4244
Current parameters: [ 1.15042776  1.51370198 -0.0431719   2.68835864  1.35136264  2.09338347], Cost: 1.4428
```



```
Current parameters: [ 0.66567349  1.76723086 -0.14724026  2.73702122  1.34541825  2.095056  ], Cost: 1.2564
Current parameters: [0.58267406 1.76806811 0.44170368 2.52176577 1.37171285 2.0876577 ], Cost: 0.6198
Current parameters: [0.47501045 1.79609204 0.78417668 2.86684545 1.40582116 2.07806093], Cost: 1.2151
Current parameters: [0.57318397 1.75108146 0.42558576 2.51161709 1.62022564 2.0876577 ], Cost: 1.6134
Current parameters: [0.45971722 1.65179878 0.47994565 2.32162154 0.94765798 2.08314417], Cost: 1.6224
Current parameters: [0.59458431 1.7660669  0.43980482 2.52057015 1.3718589  1.83795972], Cost: 0.5937
Current parameters: [1.07229351 1.83077352 0.5421361  2.52322339 1.28948263 1.8564636 ], Cost: 0.4446
Current parameters: [1.04663383 2.06281489 0.47607872 2.53173515 1.25150569 1.90250825], Cost: 0.6027
Current parameters: [1.13345806 1.81138938 0.48926455 2.69345996 1.23443929 1.39703189], Cost: 2.3522
Current parameters: [1.04406653 1.77887416 0.57969834 2.3997539  1.09879191 1.93386585], Cost: 0.5896
Current parameters: [1.09407643 1.82903485 0.47626673 2.42906282 1.32320586 1.82808691], Cost: 0.8037
Current parameters: [1.06375879 1.82924307 0.69866244 2.56304169 1.39341484 2.01626635], Cost: 0.8290
Current parameters: [1.11004274 1.7485777  0.38371301 2.63970077 1.25736767 1.97738761], Cost: 0.6113
Current parameters: [1.06693678 1.82678441 0.57573805 2.59829152 1.23680287 1.77874023], Cost: 0.5570
Current parameters: [1.13233169 1.83270096 0.54011947 2.53146057 1.27851363 1.86674537], Cost: 0.4920
Current parameters: [1.04530858 1.78527812 0.46232858 2.58495006 1.27655578 1.90626779], Cost: 0.4082
Current parameters: [1.0313968  1.78059291 0.49843075 2.60398045 1.30026469 1.94451863], Cost: 0.4459
Current parameters: [1.04572848 1.80891118 0.45356572 2.59667326 1.29047587 1.90312534], Cost: 0.4538
Current parameters: [1.0297896  1.81343077 0.46379351 2.57301638 1.23124642 1.93225516], Cost: 0.3096
Current parameters: [1.00604831 1.80252928 0.4516012  2.53261285 1.19754013 1.94976365], Cost: 0.2932
Current parameters: [0.98951831 1.79590099 0.47022195 2.56063614 1.1534727  1.92706785], Cost: 0.2083
Current parameters: [1.03170722 1.79157093 0.47637523 2.57083211 1.11200618 1.9427619 ], Cost: 0.1907
Current parameters: [1.04059418 1.80257129 0.48626756 2.55321799 1.10981855 1.92369163], Cost: 0.2396
Current parameters: [1.0107987  1.78765316 0.52378005 2.58230504 1.10833387 1.9753411 ], Cost: 0.0925
Current parameters: [0.97859377 1.80271122 0.53024813 2.61783075 1.08405497 2.00270842], Cost: 0.0646
Current parameters: [0.96649961 1.74723467 0.54991526 2.63342697 1.07844849 2.00727607], Cost: 0.0414
Current parameters: [0.95642247 1.73678069 0.54309466 2.59783969 1.07303651 2.05578535], Cost: 0.0275
Current parameters: [0.96773775 1.73520745 0.53624827 2.61258337 1.09264095 2.06983769], Cost: 0.0470
Current parameters: [0.95099441 1.74488478 0.60251468 2.58932022 1.06373833 2.06680048], Cost: 0.0103
Current parameters: [0.9738856  1.73491164 0.60567688 2.59387693 1.00753115 2.07643217], Cost: 0.0310
Current parameters: [0.929952   1.75449281 0.59890093 2.60641255 1.05616877 2.07568642], Cost: 0.0065
Current parameters: [0.88025203 1.73638323 0.61356346 2.58001633 1.0579161  2.06177951], Cost: 0.0379
Current parameters: [0.93203785 1.78300318 0.59632809 2.59412843 1.05481465 2.07582019], Cost: 0.0071
Current parameters: [0.93042328 1.75721866 0.60165738 2.61231427 1.05414866 2.06190313], Cost: 0.0065
Current parameters: [0.93838814 1.76269631 0.610907   2.62415968 1.07319576 2.08512422], Cost: 0.0089
Current parameters: [0.93239577 1.75445977 0.61005433 2.60556182 1.04610114 2.07910522], Cost: 0.0090
Current parameters: [0.94222565 1.75544185 0.57603323 2.61900662 1.04461276 2.07883856], Cost: 0.0074
Current parameters: [0.91678076 1.75364007 0.59207233 2.60636517 1.06026191 2.07312792], Cost: 0.0087
Current parameters: [0.93465433 1.75216775 0.59784596 2.60241536 1.05922754 2.07302733], Cost: 0.0066
Current parameters: [0.93051441 1.75396461 0.59632299 2.60612728 1.05336901 2.07537383], Cost: 0.0073
Current parameters: [0.93317787 1.75633813 0.59856305 2.61158172 1.06019684 2.07772698], Cost: 0.0046
```



```
Current parameters: [0.93591121 1.75770222 0.60305776 2.61523289 1.06377242 2.08004756], Cost: 0.0043
Current parameters: [0.93245287 1.75847102 0.59988734 2.61708593 1.06965155 2.07938841], Cost: 0.0054
Current parameters: [0.93561664 1.75414541 0.60315454 2.61681036 1.06361966 2.08005126], Cost: 0.0053
Current parameters: [0.94055273 1.76185904 0.59974266 2.61692248 1.06125261 2.08146823], Cost: 0.0033
Current parameters: [0.93848949 1.76668837 0.60163869 2.61624068 1.05791058 2.08573753], Cost: 0.0028
Current parameters: [0.93795982 1.77000528 0.60382553 2.61908809 1.05556681 2.0801367 ], Cost: 0.0023
Current parameters: [0.94009808 1.76909789 0.60574949 2.6171741  1.05401753 2.07982358], Cost: 0.0022
Current parameters: [0.94319428 1.7752367  0.60573326 2.61417258 1.05561737 2.07834209], Cost: 0.0010
Current parameters: [0.94761356 1.78148756 0.60591575 2.6133071  1.05465245 2.07749446], Cost: 0.0005
Current parameters: [0.94339737 1.78495146 0.61026914 2.61007711 1.05452687 2.07885816], Cost: 0.0009
Current parameters: [0.94651413 1.78166047 0.60357751 2.61161016 1.0523488  2.07688917], Cost: 0.0010
Current parameters: [0.94979936 1.78128721 0.60677991 2.61147612 1.059757   2.07238941], Cost: 0.0002
Current parameters: [0.95272816 1.78093079 0.60507897 2.60831984 1.0634458  2.07747545], Cost: 0.0003
Current parameters: [0.94808282 1.78305494 0.60484464 2.61298054 1.06153667 2.07249739], Cost: 0.0003
Current parameters: [0.95466686 1.78229191 0.61006975 2.61621257 1.06140631 2.07179277], Cost: 0.0009
Current parameters: [0.94806708 1.77812971 0.6072756  2.61194541 1.06109848 2.07253994], Cost: 0.0002
Current parameters: [0.94901788 1.78206224 0.60821928 2.61103306 1.06014723 2.07281714], Cost: 0.0002
Current parameters: [0.94775114 1.78210129 0.60754352 2.6078518  1.05990533 2.07108017], Cost: 0.0004
Current parameters: [0.94833351 1.78197934 0.60803812 2.61122807 1.05885791 2.07408452], Cost: 0.0003
Current parameters: [0.94871037 1.7821226  0.60846494 2.61148427 1.05971497 2.07218099], Cost: 0.0003
Current parameters: [0.95009808 1.78196453 0.60827522 2.61061979 1.06120128 2.07398049], Cost: 0.0001
Current parameters: [0.95009557 1.78189284 0.60767893 2.60886702 1.06174725 2.07426977], Cost: 0.0000
Current parameters: [0.94942427 1.78269614 0.60670181 2.60950007 1.06283708 2.07468867], Cost: 0.0000
Current parameters: [0.94985404 1.78348744 0.60658498 2.60939187 1.06249956 2.07462908], Cost: 0.0000
Current parameters: [0.95108587 1.78355342 0.60691019 2.60924563 1.06366148 2.07553225], Cost: 0.0000
Current parameters: [0.94989267 1.78364112 0.6068392  2.60931946 1.06298376 2.07383899], Cost: 0.0000
Current parameters: [0.9485921  1.78432403 0.60751931 2.60873203 1.062435   2.07508709], Cost: 0.0000
Current parameters: [0.94932803 1.78350015 0.60497982 2.6087274  1.06212178 2.074015  ], Cost: 0.0000
Current parameters: [0.94972315 1.78365499 0.60670455 2.61027968 1.06221489 2.07447123], Cost: 0.0000
Current parameters: [0.95014856 1.78312126 0.60703051 2.60907727 1.06188573 2.07438702], Cost: 0.0000
Current parameters: [0.94953204 1.78370115 0.60680657 2.60922805 1.06248744 2.07474301], Cost: 0.0000
Current parameters: [0.9497328  1.783477   0.60643633 2.60934658 1.06240593 2.07452006], Cost: 0.0000
Current parameters: [0.95003705 1.7835973  0.60636553 2.60920379 1.06276843 2.07482153], Cost: 0.0000
Current parameters: [0.94975746 1.78329786 0.60660178 2.60940511 1.06261104 2.07466715], Cost: 0.0000
Current parameters: [0.94981699 1.78353395 0.60655204 2.60948559 1.0625367  2.07463989], Cost: 0.0000
Current parameters: [0.94991213 1.7835053  0.60665949 2.60966402 1.06246188 2.07466728], Cost: 0.0000
Current parameters: [0.94986329 1.78354372 0.60660777 2.6094549  1.0625665  2.074552  ], Cost: 0.0000
Current parameters: [0.94970523 1.78357325 0.60651046 2.60961205 1.06268697 2.07456731], Cost: 0.0000
Current parameters: [0.94987773 1.78355745 0.60652164 2.60946349 1.06258796 2.07468136], Cost: 0.0000
Current parameters: [0.94986137 1.78357727 0.60655634 2.60945523 1.06259767 2.07470312], Cost: 0.0000
Current parameters: [0.94988548 1.78361161 0.60647439 2.6094     1.06256093 2.07467781], Cost: 0.0000
```

```
Current parameters: [0.94985903 1.78352863 0.60651639 2.60943264 1.0626064  2.07468199], Cost: 0.0000
Current parameters: [0.94995783 1.78353626 0.60653942 2.60949643 1.06257396 2.07472057], Cost: 0.0000
Current parameters: [0.94987591 1.78359259 0.60649243 2.60952564 1.06265021 2.07466831], Cost: 0.0000
Current parameters: [0.94987591 1.78359259 0.60649243 2.60952564 1.06265021 2.07466831], Cost: 0.0000

   Normal return from subroutine COBYLA

   NFVALS =   94   F = 8.831189E-08    MAXCV = 0.000000E+00
   X = 9.498759E-01   1.783593E+00   6.064924E-01   2.609526E+00   1.062650E+00
       2.074668E+00
Simulating for QAOA depth p = 4
Current parameters: [1.3111851  2.55747175 1.83091417 0.71751936 0.14958477 0.90022828
 1.01736828 2.35406185], Cost: 0.6013
Current parameters: [1.3111851  3.55747175 1.83091417 0.71751936 0.14958477 0.90022828
 1.01736828 2.35406185], Cost: 1.2610
Current parameters: [1.3111851  2.55747175 2.83091417 0.71751936 0.14958477 0.90022828
 1.01736828 2.35406185], Cost: 1.9759
Current parameters: [1.3111851  2.55747175 1.83091417 1.71751936 0.14958477 0.90022828
 1.01736828 2.35406185], Cost: 3.4711
Current parameters: [1.3111851  2.55747175 1.83091417 0.71751936 1.14958477 0.90022828
 1.01736828 2.35406185], Cost: 2.3913
Current parameters: [1.3111851  2.55747175 1.83091417 0.71751936 0.14958477 1.90022828
 1.01736828 2.35406185], Cost: 2.0183
Current parameters: [1.3111851  2.55747175 1.83091417 0.71751936 0.14958477 0.90022828
 2.01736828 2.35406185], Cost: 2.7462
Current parameters: [1.3111851  2.55747175 1.83091417 0.71751936 0.14958477 0.90022828
 1.01736828 3.35406185], Cost: 1.0934
Current parameters: [ 1.38144268  2.41253163  1.52891158  0.08702522 -0.24367657  0.58890257
  0.54613755  2.24593589], Cost: 3.5231
Current parameters: [ 1.34631389  2.48500169  1.67991287  0.40227229 -0.0470459   0.74456543
  0.78175292  2.29999887], Cost: 2.4242
Current parameters: [1.31424448 2.5511603  2.07744359 0.69006436 0.13246012 0.88667153
 0.99684843 2.34935349], Cost: 0.5245
Current parameters: [1.31424448 2.5511603  2.07744359 0.76489758 0.13246012 0.88667153
 0.89672357 2.34935349], Cost: 0.8812
Current parameters: [1.3255019  2.52793645 2.10937606 0.79786399 0.06944761 0.83678762
 1.2028444  2.3320284 ], Cost: 0.6266
Current parameters: [1.31424448 2.5511603  2.07744359 0.69006436 0.21004704 0.78866505
 0.99684843 2.34935349], Cost: 0.7263
Current parameters: [1.325061   2.52884602 2.10812543 0.64215446 0.27072055 1.06578072
 1.0815549  2.33270693], Cost: 1.6533
```



```
Current parameters: [1.31424448 2.62590384 2.07744359 0.69006436 0.13246012 0.88667153
 0.99684843 2.24916166], Cost: 0.4553
Current parameters: [1.31715713 2.82599801 2.08570553 0.67716327 0.09806966 0.87820291
 1.01965803 2.39214573], Cost: 0.8830
Current parameters: [1.18971446 2.62747734 2.08072218 0.69690912 0.13672944 0.89005134
 1.0019642  2.25033549], Cost: 0.4306
Current parameters: [ 1.16618796  2.57387685  2.05379635  0.55251599 -0.03038173  0.8126158
  0.98895805  2.19196129], Cost: 1.0804
Current parameters: [1.19006101 2.59295248 2.10860242 0.62881631 0.14753777 0.9707245
 1.02166263 2.29512354], Cost: 0.3989
Current parameters: [1.19208838 2.6128785  2.09871414 0.66379753 0.30288589 1.07035523
 1.10186344 2.152695  ], Cost: 2.1100
Current parameters: [1.18984607 2.69329407 2.11206079 0.61702032 0.12680314 0.96625098
 1.03443651 2.36435002], Cost: 0.4024
Current parameters: [1.18856949 2.58767411 2.04825238 0.6233418  0.14331627 0.97920922
 1.02914745 2.30275458], Cost: 0.3374
Current parameters: [1.17729768 2.57014475 2.0427256  0.5500031  0.05488166 0.93844713
 1.02037987 2.28783955], Cost: 0.1105
Current parameters: [1.16360566 2.59269792 2.00494529 0.47048077 0.01705761 0.94221109
 1.05950681 2.22301682], Cost: 0.1476
Current parameters: [1.1746726  2.58320093 2.03527938 0.52701605 0.07750667 0.9400606
 0.96946736 2.28163736], Cost: 0.1885
Current parameters: [ 1.16956997  2.5407208   2.02788091  0.58137292 -0.04938049  0.97530434
  0.9936437   2.31131739], Cost: 0.2783
Current parameters: [1.17035086 2.52032613 2.02858665 0.5128162  0.06876718 0.85627208
 1.05513088 2.34579551], Cost: 0.1758
Current parameters: [1.1767721  2.5448777  2.09477665 0.53433858 0.05612578 0.94629211
 1.02340563 2.27232784], Cost: 0.1144
Current parameters: [1.20669908 2.56851315 2.04056638 0.54284452 0.05409525 0.94403169
 1.02126313 2.29241828], Cost: 0.1006
Current parameters: [1.22742211 2.57500255 2.04773291 0.56605435 0.04378234 0.90320483
 1.01526039 2.26024038], Cost: 0.2180
Current parameters: [1.20141829 2.59258445 2.05271748 0.54245267 0.04744989 0.93898395
 1.025186   2.304098  ], Cost: 0.1129
Current parameters: [1.24506027 2.56061326 2.0537028  0.50355833 0.05569587 0.96455359
 1.02257336 2.30761248], Cost: 0.0733
Current parameters: [1.24510034 2.56131281 2.05160864 0.50836621 0.07262183 0.98181696
 1.04143877 2.31046804], Cost: 0.0604
Current parameters: [1.24126425 2.55094723 2.0476533  0.51296987 0.05488432 1.00070894
 1.04312888 2.32243385], Cost: 0.0549
Current parameters: [1.24368278 2.54640162 2.05175417 0.51970662 0.06042752 0.99740922
```



```
 1.03861377 2.33209958], Cost: 0.0684
Current parameters: [1.23228678 2.54137415 2.03806691 0.49749673 0.05457465 0.98930713
 1.05917719 2.33167297], Cost: 0.0630
Current parameters: [1.24907887 2.54858611 2.05086048 0.51870205 0.04933618 0.99606873
 1.05174277 2.3196337 ], Cost: 0.0630
Current parameters: [1.24377164 2.55630971 2.0373841  0.50054425 0.05718379 1.02288788
 1.04100826 2.3090183 ], Cost: 0.0369
Current parameters: [1.23956246 2.5817412  2.0444856  0.49310101 0.05134879 1.03179317
 1.04668239 2.3168096 ], Cost: 0.0542
Current parameters: [1.24158989 2.55059728 2.04996571 0.49468223 0.05777084 1.02549866
 1.04158813 2.30645093], Cost: 0.0358
Current parameters: [1.21988084 2.54632307 2.0451024  0.50494152 0.05969515 1.03182711
 1.04231176 2.28873568], Cost: 0.0325
Current parameters: [1.21702341 2.54695852 2.04492922 0.50167228 0.04953466 1.02714797
 1.03246701 2.28706088], Cost: 0.0233
Current parameters: [1.22512906 2.53639287 2.03432908 0.48844542 0.04341905 1.03074763
 1.01869989 2.27056776], Cost: 0.0313
Current parameters: [1.2230953  2.55037577 2.0504055  0.51060085 0.05070082 1.03160646
 1.02508226 2.28388071], Cost: 0.0336
Current parameters: [1.20305415 2.53695987 2.04103835 0.4992451  0.05591191 1.03128198
 1.01722034 2.30630143], Cost: 0.0294
Current parameters: [1.21609917 2.53988983 2.0451557  0.50597609 0.03781911 1.02606243
 1.03779357 2.2898789 ], Cost: 0.0188
Current parameters: [1.21200805 2.53830332 2.04709573 0.50595644 0.03725661 1.01136786
 1.0388076  2.28791744], Cost: 0.0230
Current parameters: [1.21488257 2.53787096 2.05162201 0.50283099 0.03792349 1.02764206
 1.0382627  2.28885511], Cost: 0.0197
Current parameters: [1.21021287 2.54917628 2.04285865 0.50116279 0.02930839 1.02908974
 1.04105842 2.29147683], Cost: 0.0150
Current parameters: [1.21279426 2.54360467 2.0349062  0.49275954 0.02374702 1.03142094
 1.04012557 2.28553169], Cost: 0.0139
Current parameters: [1.20688398 2.54201726 2.03361441 0.49533613 0.0248257  1.03338737
 1.03938763 2.28241696], Cost: 0.0136
Current parameters: [1.20174823 2.53779154 2.030616   0.49208675 0.02355815 1.03506245
 1.03323765 2.29416958], Cost: 0.0149
Current parameters: [1.20784631 2.54339424 2.03537742 0.49709843 0.02143171 1.03313299
 1.03325767 2.28073211], Cost: 0.0189
Current parameters: [1.20617886 2.54114263 2.02897311 0.49592933 0.02477839 1.03890952
 1.05295437 2.28495313], Cost: 0.0073
Current parameters: [1.20442049 2.54731442 2.02379517 0.48872693 0.03463592 1.04200083
 1.05708811 2.28427702], Cost: 0.0070
```



```
Current parameters: [1.20471999 2.54884919 2.01825274 0.49160697 0.03442801 1.03762111
 1.05686021 2.28482455], Cost: 0.0062
Current parameters: [1.1983947  2.54541283 2.01969857 0.48717741 0.03225893 1.02869297
 1.06613849 2.28449565], Cost: 0.0071
Current parameters: [1.20711276 2.54336796 2.01808754 0.49259419 0.03922101 1.03649438
 1.05699666 2.28487908], Cost: 0.0069
Current parameters: [1.20698141 2.54993126 2.0113163  0.49756149 0.02992609 1.04504237
 1.06571631 2.28554038], Cost: 0.0083
Current parameters: [1.20494338 2.54627488 2.0149549  0.48852502 0.03219178 1.03844251
 1.05519014 2.27977287], Cost: 0.0045
Current parameters: [1.196878   2.54803603 2.01623346 0.49577231 0.03578045 1.03876599
 1.05355078 2.26946948], Cost: 0.0068
Current parameters: [1.20020877 2.54692597 2.01287243 0.48928283 0.0320529  1.03879694
 1.0517352  2.28437744], Cost: 0.0044
Current parameters: [1.19744367 2.53486904 2.00889556 0.48300594 0.03086711 1.04439351
 1.05221918 2.28266033], Cost: 0.0035
Current parameters: [1.1996895  2.53554206 2.0059016  0.47829105 0.02867609 1.04264225
 1.04825509 2.28178684], Cost: 0.0051
Current parameters: [1.19709511 2.53651836 2.0074263  0.48149715 0.03317623 1.04539967
 1.05349759 2.28260396], Cost: 0.0029
Current parameters: [1.19807303 2.53748155 2.00333451 0.48427442 0.03017852 1.04882577
 1.05708604 2.28364978], Cost: 0.0021
Current parameters: [1.19464465 2.53742385 2.00290205 0.48245227 0.02824601 1.04467933
 1.06202997 2.28428293], Cost: 0.0026
Current parameters: [1.19744025 2.53688825 2.00131599 0.48601792 0.03121483 1.04740347
 1.0565758  2.28164107], Cost: 0.0019
Current parameters: [1.19324841 2.53867998 2.00211968 0.48709158 0.03131048 1.05217467
 1.05734908 2.27775737], Cost: 0.0025
Current parameters: [1.1951163  2.5371596  2.0003341  0.48647155 0.03127546 1.04754442
 1.05488409 2.28403463], Cost: 0.0019
Current parameters: [1.19674104 2.53453304 1.99871818 0.48879593 0.03619823 1.04908193
 1.0577542  2.28697531], Cost: 0.0024
Current parameters: [1.19693198 2.53872918 2.00008851 0.48804053 0.03189127 1.04545883
 1.05539053 2.28262355], Cost: 0.0019
Current parameters: [1.19357345 2.54294757 2.0021266  0.48803951 0.03116943 1.04880975
 1.05423661 2.28821207], Cost: 0.0022
Current parameters: [1.19614045 2.53808049 1.99743048 0.48468844 0.03059543 1.04803499
 1.05386052 2.28401217], Cost: 0.0017
Current parameters: [1.19699251 2.53692032 1.99672564 0.48621849 0.02768919 1.04813184
 1.05254542 2.28441693], Cost: 0.0020
Current parameters: [1.19676012 2.53797951 1.99800469 0.48475212 0.03136237 1.04852045
```

```
 1.05237866 2.2837534 ], Cost: 0.0019
Current parameters: [1.19461389 2.53815874 1.99684714 0.48218658 0.03079914 1.04553549
 1.05400573 2.28412444], Cost: 0.0020
Current parameters: [1.1953285  2.53870374 1.99770285 0.48461968 0.029932   1.04842318
 1.05361411 2.28258509], Cost: 0.0018
Current parameters: [1.19604634 2.5377453  1.99526183 0.48524966 0.03223882 1.04980008
 1.05593478 2.2840395 ], Cost: 0.0013
Current parameters: [1.19546098 2.5405336  1.99492407 0.48586848 0.03251364 1.05083052
 1.05642455 2.28633455], Cost: 0.0014
Current parameters: [1.19719936 2.53790588 1.99593002 0.48434482 0.03171166 1.05010572
 1.05682443 2.28421684], Cost: 0.0014
Current parameters: [1.19567545 2.53654567 1.9935999  0.48551009 0.03403889 1.05164406
 1.05738142 2.28258384], Cost: 0.0014
Current parameters: [1.1964205  2.53698309 1.99431802 0.48603697 0.03114515 1.04971413
 1.05608794 2.28463629], Cost: 0.0012
Current parameters: [1.19733401 2.53770936 1.99391167 0.48680476 0.03149745 1.04870814
 1.05645605 2.28398326], Cost: 0.0012
Current parameters: [1.19653868 2.53719297 1.99367816 0.48543205 0.03108412 1.04966149
 1.05575491 2.2845824 ], Cost: 0.0012
Current parameters: [1.19599944 2.53661082 1.99325717 0.48488841 0.0314217  1.04844039
 1.05647624 2.28534848], Cost: 0.0012
Current parameters: [1.19704405 2.53692385 1.99372607 0.48549156 0.03162501 1.04972587
 1.0553853  2.28501447], Cost: 0.0012
Current parameters: [1.19641207 2.53666145 1.99265554 0.48557537 0.0298707  1.04934324
 1.05485832 2.28431349], Cost: 0.0012
Current parameters: [1.19645894 2.53777152 1.99391802 0.48552084 0.03079934 1.04940652
 1.05547007 2.28514803], Cost: 0.0012
Current parameters: [1.19628333 2.53754177 1.99243992 0.48573908 0.03147051 1.05071642
 1.05655259 2.28490156], Cost: 0.0010
Current parameters: [1.1973109  2.53745348 1.99217008 0.48500738 0.03089569 1.05137488
 1.05772065 2.28502166], Cost: 0.0011
Current parameters: [1.19613439 2.53776162 1.99255856 0.48516206 0.03167344 1.05076273
 1.05651199 2.28420073], Cost: 0.0010
Current parameters: [1.19538221 2.53786048 1.99144055 0.48548974 0.03277683 1.051518
 1.05681541 2.28424413], Cost: 0.0010
Current parameters: [1.19495593 2.53955435 1.99133289 0.48521365 0.03261207 1.05166189
 1.05668835 2.28502641], Cost: 0.0010
Current parameters: [1.19492672 2.53748235 1.99160507 0.48512092 0.03250787 1.05199514
 1.05656461 2.284522  ], Cost: 0.0010
Current parameters: [1.19462958 2.53745316 1.99024143 0.4853486  0.03148478 1.05203278
 1.05597255 2.28387739], Cost: 0.0009
```



```
Current parameters: [1.19368543 2.53754656 1.98989313 0.48626075 0.03055994 1.05252685
 1.05630229 2.28300959], Cost: 0.0008
Current parameters: [1.19277085 2.53716811 1.98921828 0.48567569 0.03032036 1.05132004
 1.05698342 2.28325373], Cost: 0.0008
Current parameters: [1.19234987 2.53716278 1.98944043 0.48599756 0.03059942 1.05093927
 1.05635226 2.28320633], Cost: 0.0008
Current parameters: [1.19277085 2.53716811 1.98921828 0.48567569 0.03032036 1.05132004
 1.05698342 2.28325373], Cost: 0.0008

   Return from subroutine COBYLA because the MAXFUN limit has been reached.

   NFVALS =  100   F = 7.855047E-04    MAXCV = 0.000000E+00
   X = 1.192771E+00   2.537168E+00   1.989218E+00   4.856757E-01   3.032036E-02
       1.051320E+00   1.056983E+00   2.283254E+00
Simulating for QAOA depth p = 5
Current parameters: [2.22452024 2.2261654  2.2231957  0.98336511 1.02662665 0.64587426
 0.60578622 1.16032972 2.80832296 2.16652728], Cost: 2.0698
Current parameters: [2.22452024 3.2261654  2.2231957  0.98336511 1.02662665 0.64587426
 0.60578622 1.16032972 2.80832296 2.16652728], Cost: 1.6821
Current parameters: [2.22452024 3.2261654  3.2231957  0.98336511 1.02662665 0.64587426
 0.60578622 1.16032972 2.80832296 2.16652728], Cost: 0.3036
Current parameters: [2.22452024 3.2261654  3.2231957  1.98336511 1.02662665 0.64587426
 0.60578622 1.16032972 2.80832296 2.16652728], Cost: 3.1772
Current parameters: [2.22452024 3.2261654  3.2231957  0.98336511 2.02662665 0.64587426
 0.60578622 1.16032972 2.80832296 2.16652728], Cost: 3.0815
Current parameters: [2.22452024 3.2261654  3.2231957  0.98336511 1.02662665 1.64587426
 0.60578622 1.16032972 2.80832296 2.16652728], Cost: 2.3067
Current parameters: [2.22452024 3.2261654  3.2231957  0.98336511 1.02662665 0.64587426
 1.60578622 1.16032972 2.80832296 2.16652728], Cost: 2.6022
Current parameters: [2.22452024 3.2261654  3.2231957  0.98336511 1.02662665 0.64587426
 0.60578622 2.16032972 2.80832296 2.16652728], Cost: 2.4422
Current parameters: [2.22452024 3.2261654  3.2231957  0.98336511 1.02662665 0.64587426
 0.60578622 1.16032972 3.80832296 2.16652728], Cost: 2.2147
Current parameters: [2.22452024 3.2261654  3.2231957  0.98336511 1.02662665 0.64587426
 0.60578622 1.16032972 2.80832296 3.16652728], Cost: 1.7524
Current parameters: [2.35302196 3.28883535 3.44600379 0.51889629 0.57763721 0.32211651
 0.23425507 0.81465741 2.49942902 1.93236014], Cost: 2.5047
Current parameters: [2.2887711  3.25750038 3.33459974 0.7511307  0.80213193 0.48399539
 0.42002065 0.98749356 2.65387599 2.04944371], Cost: 3.2409
Current parameters: [2.4654688  3.2261654  3.2231957  1.05002686 1.02662665 0.64587426
 0.60578622 1.16032972 2.80832296 2.16652728], Cost: 0.1772
```



```
Current parameters: [2.34676351 3.23470235 3.25354677 1.52083734 0.965465   0.60177176
 0.55517599 1.11324199 2.76624521 2.13462886], Cost: 1.6987
Current parameters: [2.4654688  3.46682668 3.15550413 1.05002686 1.02662665 0.64587426
 0.60578622 1.16032972 2.80832296 2.16652728], Cost: 0.5719
Current parameters: [2.47429058 3.07575879 2.74976661 1.03807828 0.9974195  0.62481354
 0.5816178  1.13784343 2.78822914 2.1512945 ], Cost: 0.2780
Current parameters: [2.34863543 3.22398525 3.21544471 1.03509789 1.23713747 0.61672824
 0.5766402  1.13118369 2.77917694 2.13738125], Cost: 0.9185
Current parameters: [2.93958067 3.16249167 3.24832375 1.05696581 1.15572476 0.66872007
 0.6158828  1.17732721 2.83513545 2.21328202], Cost: 0.2869
Current parameters: [2.45613823 3.21370902 3.22150537 1.0397495  1.01890873 0.89236537
 0.58513723 1.14084948 2.79050501 2.15208679], Cost: 0.2005
Current parameters: [2.46699041 3.2261938  3.22329665 1.05022129 1.02642323 0.64572758
 0.68991428 1.06789188 2.80870255 2.16690687], Cost: 0.1574
Current parameters: [2.45925992 3.20069296 3.22057206 1.03047742 1.00894793 0.66522541
 0.87110071 1.22914748 2.77479455 2.13997159], Cost: 0.6159
Current parameters: [2.47322221 3.22631008 3.22371008 1.05101759 1.02559011 0.64512683
 0.68922489 1.06725046 2.88092781 2.06508882], Cost: 0.2176
Current parameters: [2.43786256 3.20335531 3.21941957 1.03000793 1.01404334 0.66502388
 0.6991605  1.07434552 3.00583655 2.31248089], Cost: 1.2441
Current parameters: [2.50816773 3.19391737 3.2657035  0.92405459 0.89992423 0.6118773
 0.62485411 1.00299187 2.69561687 2.10636566], Cost: 0.9399
Current parameters: [2.57958966 3.21946206 3.2249169  1.07084474 1.07515894 0.65127211
 0.68868649 1.06760906 2.80189189 2.17129529], Cost: 0.2007
Current parameters: [2.46466802 3.24294818 3.28286289 1.05657587 1.02941121 0.64788216
 0.69238679 1.07019229 2.81075821 2.16846523], Cost: 0.1865
Current parameters: [2.45559449 3.21162256 3.21476742 1.16500026 0.99350916 0.6434699
 0.68059045 1.05778515 2.78415108 2.15443428], Cost: 0.3247
Current parameters: [2.48071764 3.27955662 3.20926986 1.05197536 1.00168947 0.64915206
 0.69333877 1.07131636 2.81212704 2.17033135], Cost: 0.1684
Current parameters: [2.42513236 3.2772395  3.20050618 1.04884661 1.11875982 0.642312
 0.67852033 1.05404986 2.77180112 2.14399196], Cost: 0.4823
Current parameters: [2.46916451 3.22919834 3.22369941 1.05269152 1.0282961  0.58422058
 0.69496644 1.072667   2.81432834 2.1713383 ], Cost: 0.2263
Current parameters: [2.46227962 3.20913899 3.21464277 1.04705427 1.02340534 0.6758384
 0.67914005 1.05491527 2.90039903 2.24186375], Cost: 0.3920
Current parameters: [2.48136369 3.21285577 3.2308223  1.01877152 0.99338718 0.64741246
 0.67072822 1.04863715 2.78543716 2.15682293], Cost: 0.1465
Current parameters: [2.48709363 3.20299375 3.23304037 1.01771441 0.97953695 0.64972712
 0.68938568 1.06574479 2.78661579 2.15786475], Cost: 0.0650
Current parameters: [2.48327341 3.19831679 3.22753454 0.99453824 0.98127547 0.66657851
```



```
 0.72532823 1.09505879 2.75960436 2.14626533], Cost: 0.0115
Current parameters: [2.48876795 3.17669269 3.22572397 0.97581818 0.95935598 0.68848462
 0.74333773 1.10848154 2.78731957 2.17493155], Cost: 0.0702
Current parameters: [2.48060666 3.19716451 3.22699675 1.00392513 0.97862619 0.66652783
 0.72465935 1.09444658 2.74072909 2.16897219], Cost: 0.0137
Current parameters: [2.48343257 3.18269671 3.22465747 1.03454255 0.95399463 0.67518975
 0.73536642 1.10213766 2.74780796 2.11552144], Cost: 0.0453
Current parameters: [2.45675382 3.20037352 3.22720888 0.98999228 0.96993606 0.65601196
 0.72649139 1.09572186 2.76207434 2.14588548], Cost: 0.0130
Current parameters: [2.50605652 3.19272466 3.22779437 0.98788477 0.98085968 0.61393877
 0.74245876 1.10940461 2.75318851 2.14607664], Cost: 0.0223
Current parameters: [2.48326028 3.20101686 3.23713401 0.9956058  0.98170765 0.66689015
 0.74564104 1.0735461  2.76002467 2.1466057 ], Cost: 0.0126
Current parameters: [2.48271122 3.21105464 3.28426537 0.99530125 0.98007148 0.67023918
 0.7195654  1.11669892 2.75681313 2.1455132 ], Cost: 0.0254
Current parameters: [2.49081462 3.22406249 3.22090897 0.99531963 0.96741131 0.66819216
 0.7275922  1.09731808 2.76156591 2.14812173], Cost: 0.0212
Current parameters: [2.47127941 3.20791168 3.21650359 0.96658666 1.00807783 0.67910348
 0.73913469 1.10372115 2.72342311 2.12845017], Cost: 0.0242
Current parameters: [2.48911516 3.19383299 3.23098952 0.98191971 0.9705276  0.66392616
 0.70858752 1.07939472 2.75052562 2.14172903], Cost: 0.0351
Current parameters: [2.476344   3.20558129 3.22439811 1.00761693 1.00318466 0.65947993
 0.71853056 1.09013738 2.76895481 2.14883858], Cost: 0.0313
Current parameters: [2.48338879 3.19246243 3.22601024 0.98910687 0.98034269 0.67179091
 0.73123849 1.0996209  2.76632431 2.15327173], Cost: 0.0069
Current parameters: [2.48268097 3.18570752 3.22397738 0.99733232 0.97545692 0.67596461
 0.73710061 1.10403967 2.76553059 2.14775726], Cost: 0.0127
Current parameters: [2.48416747 3.19241551 3.23208577 0.99144304 0.97831074 0.67128861
 0.72946719 1.10079478 2.76906411 2.15455769], Cost: 0.0073
Current parameters: [2.48082086 3.19571506 3.23314045 0.98313554 0.98556338 0.67578813
 0.73486992 1.10659032 2.76062956 2.15152628], Cost: 0.0092
Current parameters: [2.48578166 3.19194266 3.22587213 0.9889886  0.98090493 0.66554194
 0.73420459 1.10220587 2.76600823 2.15351798], Cost: 0.0063
Current parameters: [2.49013243 3.18456877 3.22683385 0.97817363 0.97665386 0.66327555
 0.73079236 1.09850198 2.76504418 2.15537518], Cost: 0.0017
Current parameters: [2.49055068 3.17929755 3.22513569 0.97219216 0.98406311 0.65983678
 0.72798108 1.09558835 2.77212428 2.16202918], Cost: 0.0014
Current parameters: [2.4916069  3.18096191 3.22491108 0.96994223 0.98396521 0.65993584
 0.72826601 1.09585919 2.77683824 2.15658478], Cost: 0.0014
Current parameters: [2.49615648 3.18640197 3.22140443 0.96626107 0.97652598 0.66013059
 0.72872572 1.09597575 2.77344494 2.16936399], Cost: 0.0036
```

```
Current parameters: [2.49710796 3.17857613 3.22515134 0.97379774 0.98667195 0.66256591
 0.72786528 1.09554359 2.7713567  2.16174646], Cost: 0.0007
Current parameters: [2.4993723  3.16490721 3.22324783 0.9732684  0.98168744 0.66404557
 0.73124929 1.09718335 2.77161262 2.15909503], Cost: 0.0013
Current parameters: [2.4971047  3.17823553 3.22284876 0.97383946 0.98661719 0.66219337
 0.72247592 1.10063887 2.7708718  2.16126523], Cost: 0.0008
Current parameters: [2.50093606 3.17716193 3.22716914 0.97241582 0.98741697 0.65565487
 0.72134358 1.08743579 2.76521622 2.15652983], Cost: 0.0073
Current parameters: [2.49690506 3.17899473 3.22891731 0.96932546 0.98710311 0.66430102
 0.72904224 1.09822577 2.76768627 2.16056873], Cost: 0.0002
Current parameters: [2.49492337 3.17649103 3.22924746 0.96694226 0.99804949 0.66664849
 0.73303079 1.10334265 2.77342489 2.16584128], Cost: 0.0046
Current parameters: [2.50114285 3.18189006 3.22723771 0.97052007 0.98625698 0.66559191
 0.73130313 1.09552563 2.76993742 2.16382341], Cost: 0.0008
Current parameters: [2.49740993 3.18056742 3.22713039 0.9604297  0.97713702 0.66475792
 0.72792806 1.10490025 2.76521717 2.16333759], Cost: 0.0025
Current parameters: [2.49938775 3.1784905  3.23022814 0.96832305 0.98829957 0.66107768
 0.72623261 1.09394762 2.7647017  2.15833975], Cost: 0.0012
Current parameters: [2.49668446 3.17822189 3.22589375 0.96869508 0.98762359 0.66482585
 0.73036161 1.09766175 2.76642858 2.15962715], Cost: 0.0002
Current parameters: [2.49866272 3.17300183 3.23023764 0.97147177 0.98410831 0.66530415
 0.73069389 1.09796849 2.76887787 2.15934735], Cost: 0.0004
Current parameters: [2.49603703 3.17849893 3.22894022 0.96839212 0.98701928 0.66696572
 0.72725092 1.09659426 2.7681986  2.16095221], Cost: 0.0003
Current parameters: [2.49548646 3.18200784 3.22993537 0.97376364 0.98611836 0.66753159
 0.72951003 1.09913079 2.76514072 2.15736155], Cost: 0.0006
Current parameters: [2.497139   3.17944264 3.22867032 0.9673547  0.98439777 0.66445891
 0.72869096 1.09982553 2.766726   2.16090047], Cost: 0.0004
Current parameters: [2.4966333  3.17857284 3.22897227 0.96988347 0.98715266 0.66427978
 0.72898    1.09816931 2.76652427 2.16194287], Cost: 0.0002
Current parameters: [2.49602249 3.17775902 3.22967046 0.96901876 0.98958949 0.66513384
 0.72983741 1.10036583 2.76708542 2.16261065], Cost: 0.0005
Current parameters: [2.49588531 3.1787613  3.22978834 0.96928244 0.98691334 0.66421249
 0.73020034 1.09738387 2.76636893 2.16196346], Cost: 0.0002
Current parameters: [2.49396906 3.17840416 3.22774823 0.97083584 0.98634962 0.66277806
 0.72851069 1.098073   2.76806137 2.16242729], Cost: 0.0005
Current parameters: [2.49761248 3.17929277 3.22856635 0.97018039 0.98701122 0.6646702
 0.72964361 1.09772371 2.76716458 2.16287201], Cost: 0.0003
Current parameters: [2.49690326 3.17774708 3.22906278 0.96988573 0.98687183 0.66421607
 0.72914494 1.09810081 2.76679688 2.16198251], Cost: 0.0002
Current parameters: [2.49648265 3.17857999 3.2293667  0.97113005 0.98666628 0.6651764
```



```
 0.72931563 1.09844979 2.76608814 2.16113022], Cost: 0.0002
Current parameters: [2.49653501 3.17846431 3.22948347 0.97153428 0.98731575 0.66499843
 0.72928417 1.09792961 2.76618319 2.16096765], Cost: 0.0001
Current parameters: [2.49719651 3.17844423 3.23006022 0.9713767  0.98737007 0.66441954
 0.72862512 1.0973025  2.76502961 2.16024461], Cost: 0.0001
Current parameters: [2.49614114 3.17837219 3.22984239 0.97212557 0.98707518 0.66333974
 0.7279955  1.09675315 2.7650832  2.1598793 ], Cost: 0.0002
Current parameters: [2.49738083 3.17852826 3.23008135 0.97156228 0.98750151 0.66380981
 0.72910268 1.0977988  2.76493174 2.16017576], Cost: 0.0001
Current parameters: [2.49739054 3.17806443 3.23085893 0.9711744  0.98893448 0.66436347
 0.72904239 1.0983443  2.76455463 2.15997326], Cost: 0.0001
Current parameters: [2.49751719 3.17830868 3.23157464 0.97135066 0.98879559 0.66426286
 0.72876657 1.0984503  2.76491348 2.16027187], Cost: 0.0001
Current parameters: [2.498921   3.1788469  3.23158257 0.97162224 0.989402   0.66474784
 0.72930106 1.09778854 2.76469148 2.16059542], Cost: 0.0001
Current parameters: [2.49736845 3.17812465 3.23159487 0.97159391 0.98867041 0.66432458
 0.72875682 1.09853715 2.76431052 2.16094077], Cost: 0.0001
Current parameters: [2.49798266 3.1786406  3.23197405 0.97098203 0.9879203  0.66509259
 0.72889618 1.09936308 2.76429426 2.15956162], Cost: 0.0001
Current parameters: [2.4979359  3.17805831 3.23119144 0.97164606 0.98885935 0.6642837
 0.7281992  1.09882268 2.76504184 2.16026935], Cost: 0.0001
Current parameters: [2.49807477 3.1767286  3.23206431 0.97123235 0.98831861 0.66418988
 0.72931942 1.09813462 2.76524952 2.16022357], Cost: 0.0000
Current parameters: [2.49830794 3.17678171 3.23192712 0.97042717 0.98829175 0.66397128
 0.72921193 1.09824943 2.76528444 2.16061714], Cost: 0.0000
Current parameters: [2.4986121  3.17589498 3.23282792 0.97008771 0.98889958 0.66309045
 0.72896443 1.09770576 2.76492817 2.16000059], Cost: 0.0001
Current parameters: [2.4981584  3.1766528  3.23181879 0.970431   0.98842017 0.66437312
 0.72966325 1.09858888 2.76584266 2.16092446], Cost: 0.0000
Current parameters: [2.4984298  3.17685601 3.23196394 0.97047894 0.9882346  0.66377146
 0.72943928 1.09856266 2.76517777 2.16051898], Cost: 0.0000
Current parameters: [2.49858892 3.1769106  3.23214409 0.97023008 0.98774858 0.66442965
 0.72937307 1.09877655 2.76497598 2.16025307], Cost: 0.0000
Current parameters: [2.49808235 3.1767308  3.23195657 0.9704183  0.98806316 0.66364268
 0.72930543 1.09872004 2.76524082 2.16044379], Cost: 0.0000
Current parameters: [2.49860014 3.17704291 3.23161316 0.97067722 0.98755345 0.66356759
 0.72964167 1.09825985 2.76544296 2.16065634], Cost: 0.0000
Current parameters: [2.49821108 3.17691923 3.23213542 0.97041082 0.98817591 0.66377769
 0.72968336 1.09842238 2.76497276 2.16068564], Cost: 0.0000
Current parameters: [2.49854898 3.17652779 3.23179087 0.97084309 0.988133   0.66384598
 0.72919894 1.09883673 2.76476735 2.16111226], Cost: 0.0000
```



```
Current parameters: [2.49838406 3.17668617 3.23162367 0.9703878  0.98828889 0.66381917
 0.72959321 1.09850033 2.76500994 2.16036924], Cost: 0.0000
Current parameters: [2.49820549 3.17765853 3.23162272 0.97054963 0.98845982 0.66385118
 0.72927504 1.09877985 2.76510446 2.16055496], Cost: 0.0000
Current parameters: [2.49863706 3.17690388 3.23216106 0.97047668 0.98846648 0.66355129
 0.72949163 1.0986123  2.76536495 2.16041027], Cost: 0.0000
Current parameters: [2.49884936 3.17679747 3.23221376 0.97067322 0.98859304 0.66342716
 0.72939366 1.09880431 2.76528901 2.16064893], Cost: 0.0000
Current parameters: [2.49870105 3.17691933 3.23213469 0.97026801 0.98844009 0.66352337
 0.72946085 1.0986477  2.76536428 2.16049604], Cost: 0.0000
Current parameters: [2.49863706 3.17690388 3.23216106 0.97047668 0.98846648 0.66355129
 0.72949163 1.0986123  2.76536495 2.16041027], Cost: 0.0000

   Return from subroutine COBYLA because the MAXFUN limit has been reached.

   NFVALS =  100   F = 1.321338E-05    MAXCV = 0.000000E+00
   X = 2.498637E+00   3.176904E+00   3.232161E+00   9.704767E-01   9.884665E-01
       6.635513E-01   7.294916E-01   1.098612E+00   2.765365E+00   2.160410E+00
Simulating for QAOA depth p = 6
Current parameters: [2.43541485 1.55846823 0.24625476 1.22228517 1.70780381 2.75498672
 0.10650252 0.60049858 0.94982876 2.84679221 2.79155909 3.13308206], Cost: 2.4749
Current parameters: [1.43541485 2.55846823 0.24625476 1.22228517 1.70780381 2.75498672
 0.10650252 0.60049858 0.94982876 2.84679221 2.79155909 3.13308206], Cost: 2.2387
Current parameters: [1.43541485 1.55846823 1.24625476 1.22228517 1.70780381 2.75498672
 0.10650252 0.60049858 0.94982876 2.84679221 2.79155909 3.13308206], Cost: 1.4885
Current parameters: [1.43541485 1.55846823 1.24625476 2.22228517 1.70780381 2.75498672
 0.10650252 0.60049858 0.94982876 2.84679221 2.79155909 3.13308206], Cost: 2.0982
Current parameters: [1.43541485 1.55846823 1.24625476 1.22228517 2.70780381 2.75498672
 0.10650252 0.60049858 0.94982876 2.84679221 2.79155909 3.13308206], Cost: 0.5361
Current parameters: [1.43541485 1.55846823 1.24625476 1.22228517 2.70780381 3.75498672
 0.10650252 0.60049858 0.94982876 2.84679221 2.79155909 3.13308206], Cost: 0.5594
Current parameters: [1.43541485 1.55846823 1.24625476 1.22228517 2.70780381 2.75498672
 1.10650252 0.60049858 0.94982876 2.84679221 2.79155909 3.13308206], Cost: 2.0536
Current parameters: [1.43541485 1.55846823 1.24625476 1.22228517 2.70780381 2.75498672
 0.10650252 1.60049858 0.94982876 2.84679221 2.79155909 3.13308206], Cost: 0.1872
Current parameters: [1.43541485 1.55846823 1.24625476 1.22228517 2.70780381 2.75498672
 0.10650252 1.60049858 1.94982876 2.84679221 2.79155909 3.13308206], Cost: 3.1307
Current parameters: [1.43541485 1.55846823 1.24625476 1.22228517 2.70780381 2.75498672
 0.10650252 1.60049858 0.94982876 3.84679221 2.79155909 3.13308206], Cost: 1.7231
Current parameters: [1.43541485 1.55846823 1.24625476 1.22228517 2.70780381 2.75498672
 0.10650252 1.60049858 0.94982876 2.84679221 3.79155909 3.13308206], Cost: 1.8046
```



```
Current parameters: [1.43541485 1.55846823 1.24625476 1.22228517 2.70780381 2.75498672
 0.10650252 1.60049858 0.94982876 2.84679221 2.79155909 4.13308206], Cost: 1.9943
Current parameters: [ 1.33105326  1.5054644   1.35635808  1.08973327  2.91486344  2.74993888
 -0.22342286  1.67636228  0.30985917  2.51285876  2.43990261  2.7401872 ], Cost: 2.3704
Current parameters: [1.42312551 1.55222664 1.25922024 1.20667621 2.7321866  2.7543923
 0.59398854 1.60943208 0.87446766 2.8074691  2.75014896 3.08681582], Cost: 2.3357
Current parameters: [1.54862234 1.33556901 1.24625476 1.22228517 2.70780381 2.75498672
 0.10650252 1.60049858 0.94982876 2.84679221 2.79155909 3.13308206], Cost: 0.3664
Current parameters: [1.87688718 1.78901416 1.25024154 1.21748554 2.71530132 2.75480394
 0.07821264 1.60324556 0.92665585 2.83470068 2.77882582 3.11885558], Cost: 0.3687
Current parameters: [1.43541485 1.55846823 1.05394525 1.0625446  2.70780381 2.75498672
 0.10650252 1.60049858 0.94982876 2.84679221 2.79155909 3.13308206], Cost: 0.6109
Current parameters: [1.4281978  1.56419221 0.94500968 1.61593169 2.71892831 2.75471552
 0.06452713 1.60457443 0.91544573 2.82885129 2.77266598 3.11197338], Cost: 0.9930
Current parameters: [1.43541485 1.55846823 1.24625476 1.22228517 2.70171096 2.50506098
 0.10650252 1.60049858 0.94982876 2.84679221 2.79155909 3.13308206], Cost: 0.1818
Current parameters: [1.42687356 1.56524249 1.27853425 1.22009073 2.20878571 2.51677595
 0.05682514 1.60532231 0.90913685 2.82555934 2.76919931 3.10810018], Cost: 0.5651
Current parameters: [1.32113573 1.63061107 1.385788   1.235959   2.73114574 2.62044922
 0.02783759 1.62951641 0.92081093 2.81777437 2.76254125 3.10406423], Cost: 0.3201
Current parameters: [ 1.54762034  1.43824131  1.23053572  1.11950494  2.81372652  2.28367294
 -0.20121469  1.57492483  0.76254162  2.77498538  2.71306461  3.03902661], Cost: 2.2114
Current parameters: [1.44157772 1.56263437 1.26313207 1.22611645 2.69985608 2.49507293
 0.07802301 1.6032475  1.19426633 2.82643735 2.76999514 3.10870476], Cost: 1.0216
Current parameters: [1.42831552 1.56420899 1.25510638 1.22467065 2.70163135 2.51339863
 0.1043489  1.4765779  0.95070254 2.85099485 2.78947578 3.13099875], Cost: 0.1315
Current parameters: [1.42360344 1.57204691 1.27179309 1.22885202 2.70073159 2.51772202
 0.08956021 1.4851531  0.94371364 3.09890003 2.77780476 3.11808933], Cost: 0.3703
Current parameters: [1.42761491 1.56477553 1.25597991 1.22490607 2.70162349 2.51422144
 0.10413637 1.47656768 0.95078876 2.85103984 2.88373628 3.04891589], Cost: 0.1776
Current parameters: [1.42187585 1.57678616 1.28291908 1.23158849 2.70000941 2.51874633
 0.07794995 1.46974957 0.93764527 2.85243093 2.94741396 3.31964434], Cost: 0.2744
Current parameters: [1.5261632  1.63568679 1.27703948 1.22866031 2.70389805 2.53277016
 0.10234733 1.47723497 0.94515964 2.84810258 2.78643001 3.12759582], Cost: 0.0264
Current parameters: [1.52358699 1.65978093 1.33883951 1.23258502 2.71471516 2.53446797
 0.32911437 1.46667474 0.86856766 2.82747941 2.77543016 3.13088885], Cost: 0.4649
Current parameters: [1.51064184 1.65981201 1.22614702 1.33459289 2.69973342 2.56141867
 0.11338766 1.47940123 0.94973174 2.84901902 2.78755816 3.12921656], Cost: 0.0740
Current parameters: [ 1.59310148  1.6123383   1.23948739  1.25392258  2.75852088  2.45130195
 -0.02337147  1.44908102  0.78523752  2.80238562  2.75212244  3.10848941], Cost: 1.2083
Current parameters: [1.53423001 1.63470026 1.270246   1.22974538 2.59441537 2.5066544
```

```
 0.10272236 1.52321718 0.91822539 2.84650945 2.78713445 3.12797274], Cost: 0.0214
Current parameters: [1.5052748  1.6504181  1.33186666 1.21780113 2.48054063 2.6159135
 0.06683642 1.35360576 0.95765648 2.82979775 2.76580859 3.11297114], Cost: 0.0996
Current parameters: [1.54050783 1.68797432 1.21407443 1.14605047 2.61965729 2.5497965
 0.09842186 1.52452616 0.92381718 2.8496991  2.7906048  3.13188288], Cost: 0.0521
Current parameters: [1.41380883 1.62916125 1.12868134 1.28229271 2.47180752 2.52133884
 0.1591533  1.5609316  0.84669263 2.83458952 2.78408292 3.13451533], Cost: 0.2901
Current parameters: [1.55165416 1.6557297  1.26569307 1.23426865 2.55122137 2.45556304
 0.12790826 1.58305348 1.14665904 2.82033103 2.77625116 3.1340164 ], Cost: 1.0402
Current parameters: [1.4786478  1.67812361 1.33120545 1.23544007 2.60450487 2.5436763
 0.07101664 1.58386058 0.90232576 2.83093289 2.7751148  3.11765991], Cost: 0.0678
Current parameters: [1.54914649 1.60630257 1.26421716 1.22822455 2.59541262 2.53328543
 0.10772141 1.53854099 0.92047351 2.84446653 2.75888508 3.09539376], Cost: 0.0997
Current parameters: [1.55872628 1.58772446 1.28073097 1.23243519 2.59092816 2.56771302
 0.09796767 1.54737703 0.90954017 2.8474312  2.84555741 3.19772151], Cost: 0.0263
Current parameters: [1.53118362 1.63207316 1.26135446 1.23553319 2.58744441 2.50629574
 0.06909747 1.52847175 0.9250567  2.89667402 2.7853252  3.12594307], Cost: 0.0517
Current parameters: [1.53536265 1.65320961 1.31302789 1.21922101 2.6043018  2.51482914
 0.18783698 1.51093367 0.88698016 2.91567651 2.78670196 3.13239822], Cost: 0.1126
Current parameters: [1.56756763 1.63955927 1.2760137  1.2300116  2.62236238 2.48002465
 0.05276431 1.48502402 0.82700612 2.82333663 2.78733118 3.14038151], Cost: 0.3449
Current parameters: [1.54161069 1.64668725 1.27759322 1.23046226 2.58634331 2.48882
 0.11124283 1.53502134 0.969951   2.83730363 2.79403098 3.14380257], Cost: 0.0651
Current parameters: [1.53365572 1.63292088 1.25777419 1.23297647 2.60323335 2.49423849
 0.10901677 1.54487989 0.91086485 2.84805563 2.78931668 3.1301967 ], Cost: 0.0236
Current parameters: [1.50237851 1.63821987 1.24315207 1.24156901 2.5544923  2.51509703
 0.11616111 1.51908954 0.91163831 2.83968315 2.7896526  3.13478222], Cost: 0.0502
Current parameters: [1.54481789 1.64428812 1.26963915 1.23635111 2.58819226 2.50917848
 0.10310092 1.52603533 0.91633613 2.84615038 2.80643642 3.11067879], Cost: 0.0156
Current parameters: [1.51170089 1.61830285 1.27311698 1.21737898 2.60374496 2.50010257
 0.10372587 1.51787626 0.9259306  2.84547851 2.83691763 3.09198615], Cost: 0.0549
Current parameters: [1.53979298 1.64811666 1.26377167 1.2538668  2.60690345 2.51814548
 0.10472238 1.51523544 0.92261552 2.8452549  2.80523771 3.11267289], Cost: 0.0107
Current parameters: [1.54700816 1.66374235 1.2390505  1.27439927 2.60248194 2.52362161
 0.11448708 1.55308963 0.92665009 2.85740129 2.83139265 3.10036069], Cost: 0.0312
Current parameters: [1.55489538 1.63750746 1.24730687 1.2529884  2.60444772 2.50891854
 0.11199702 1.50182181 0.92473955 2.84981829 2.80887633 3.11388734], Cost: 0.0185
Current parameters: [1.5512031  1.66919464 1.2686475  1.2664799  2.61614375 2.5019516
 0.07693608 1.5052104  0.88892862 2.82243728 2.81520344 3.12586163], Cost: 0.0438
Current parameters: [1.53895858 1.63561532 1.27786596 1.27472047 2.60048851 2.50814012
 0.10956124 1.51658834 0.92013538 2.84821426 2.80512813 3.11052028], Cost: 0.0107
```



```
Current parameters: [1.54083435 1.67007169 1.28495544 1.24433507 2.61599373 2.52267043
 0.14282487 1.50413943 0.90445563 2.87348685 2.80651475 3.1214508 ], Cost: 0.0322
Current parameters: [1.54573285 1.63522679 1.26823653 1.25315275 2.60708336 2.53461932
 0.10339122 1.51878377 0.92026193 2.8443516  2.8172148  3.13053622], Cost: 0.0026
Current parameters: [1.56669478 1.61593696 1.27392473 1.25397053 2.61467716 2.56767245
 0.10834332 1.52682494 0.91353904 2.82833701 2.78607876 3.10705754], Cost: 0.0316
Current parameters: [1.53654931 1.64109739 1.26565185 1.25212008 2.61308474 2.54073384
 0.08521147 1.51536762 0.92797578 2.85892817 2.80527253 3.1260821 ], Cost: 0.0048
Current parameters: [1.56976213 1.61212749 1.2760008  1.26135046 2.58830851 2.53229668
 0.08896846 1.52189375 0.90505207 2.88252923 2.82957101 3.1463683 ], Cost: 0.0260
Current parameters: [1.55318297 1.64058815 1.27814741 1.25337053 2.60541883 2.53119407
 0.1037501  1.52080246 0.94494622 2.83916132 2.81533155 3.14217813], Cost: 0.0148
Current parameters: [1.5436719  1.63895127 1.26389675 1.25721136 2.59709056 2.53846159
 0.1051841  1.51689018 0.91850312 2.84305929 2.81125454 3.13592215], Cost: 0.0029
Current parameters: [1.5225648  1.62382199 1.26723022 1.24942448 2.60213008 2.53932745
 0.10484566 1.51127742 0.92335506 2.83950045 2.82960568 3.12879689], Cost: 0.0097
Current parameters: [1.54478248 1.63604934 1.27585397 1.24791173 2.60933996 2.53447752
 0.10850811 1.51018768 0.91675387 2.84729857 2.81290686 3.13404764], Cost: 0.0039
Current parameters: [1.5413474  1.65147598 1.26625531 1.25601223 2.61210284 2.54307598
 0.11931324 1.52840161 0.91893676 2.85652963 2.82589501 3.12692205], Cost: 0.0132
Current parameters: [1.54498364 1.64820196 1.26913446 1.2586825  2.6148113  2.53077432
 0.08900226 1.5136874  0.90554739 2.83067718 2.82323211 3.13592382], Cost: 0.0104
Current parameters: [1.54106288 1.63102015 1.26424291 1.25221611 2.61260098 2.52987501
 0.10521302 1.52551441 0.91810946 2.84444568 2.81185064 3.13780401], Cost: 0.0041
Current parameters: [1.54510819 1.63592125 1.26792387 1.25244784 2.60932712 2.53718992
 0.10515383 1.51908505 0.92114018 2.83962631 2.81382777 3.12734932], Cost: 0.0047
Current parameters: [1.55238402 1.62572459 1.27040388 1.25542056 2.60469492 2.54036464
 0.10024505 1.520875   0.91465049 2.84802518 2.81594121 3.13011214], Cost: 0.0014
Current parameters: [1.55376571 1.62428224 1.26724139 1.25924335 2.60787698 2.54091992
 0.10173533 1.5168595  0.91609106 2.84857833 2.8165354  3.1310699 ], Cost: 0.0019
Current parameters: [1.55811406 1.62443074 1.26177741 1.2469366  2.60070138 2.53576075
 0.09939963 1.51815057 0.91269788 2.85032611 2.81574938 3.12714322], Cost: 0.0039
Current parameters: [1.55057453 1.62521871 1.27089631 1.25878088 2.60381926 2.53655384
 0.10121685 1.52033042 0.91206557 2.84913271 2.81426859 3.12578376], Cost: 0.0018
Current parameters: [1.55748243 1.62318647 1.27720333 1.25901083 2.59996863 2.53518791
 0.09829448 1.52252952 0.92377956 2.84822628 2.81445392 3.13347537], Cost: 0.0011
Current parameters: [1.55723485 1.62319342 1.27673104 1.25846558 2.59928779 2.53703735
 0.10321772 1.52496855 0.92626788 2.85245939 2.81454474 3.13197101], Cost: 0.0042
Current parameters: [1.56132833 1.62903752 1.28075168 1.26385133 2.60111625 2.53405953
 0.09105359 1.52379391 0.91675    2.84661797 2.81975473 3.1382055 ], Cost: 0.0023
Current parameters: [1.55799705 1.62098327 1.27773909 1.25899268 2.59818233 2.53408054
```



```
 0.10270661 1.5227994  0.92217204 2.84442497 2.81816361 3.13441138], Cost: 0.0020
Current parameters: [1.54820268 1.61668568 1.27831586 1.25840545 2.59703363 2.53686641
 0.09011338 1.51816188 0.92575008 2.84783497 2.81793838 3.1342147 ], Cost: 0.0012
Current parameters: [1.55554037 1.62116113 1.27668932 1.25706308 2.6019694  2.53539066
 0.09817433 1.52709847 0.9219911  2.84811021 2.81248313 3.13760739], Cost: 0.0008
Current parameters: [1.55500138 1.61785909 1.27221199 1.26292388 2.60489515 2.52380313
 0.09735796 1.52406204 0.92331328 2.84845417 2.80810818 3.13980445], Cost: 0.0019
Current parameters: [1.55622941 1.62166809 1.28077352 1.25470884 2.60273568 2.53503335
 0.09942298 1.52229584 0.92082216 2.84938144 2.8096725  3.13897083], Cost: 0.0009
Current parameters: [1.54858858 1.61638492 1.27838106 1.25574678 2.60313136 2.53594127
 0.10089178 1.53052572 0.91483134 2.84968648 2.81778781 3.14571517], Cost: 0.0014
Current parameters: [1.55510305 1.61941341 1.27781011 1.25683142 2.60382609 2.53774316
 0.09655105 1.5282593  0.92122275 2.84236212 2.80942005 3.13689885], Cost: 0.0017
Current parameters: [1.55618738 1.62024135 1.27755993 1.25575325 2.60434531 2.53437257
 0.09768231 1.52753875 0.92237664 2.84846496 2.81397556 3.13622848], Cost: 0.0007
Current parameters: [1.55863141 1.61941736 1.27419663 1.25068049 2.6016818  2.53330959
 0.09536758 1.527281   0.92058569 2.84855457 2.81402942 3.13691493], Cost: 0.0009
Current parameters: [1.55538453 1.62039468 1.27932282 1.25555222 2.6025761  2.53269348
 0.09743619 1.52908573 0.92164791 2.84862511 2.81321973 3.13484597], Cost: 0.0007
Current parameters: [1.55204105 1.6252543  1.27876529 1.25273682 2.60340888 2.53075541
 0.09780081 1.52905655 0.92394336 2.84622785 2.81418304 3.13606072], Cost: 0.0010
Current parameters: [1.55622876 1.61912221 1.28043284 1.25512712 2.60163971 2.53251188
 0.09765783 1.52991491 0.92425061 2.84833506 2.81324345 3.13658058], Cost: 0.0009
Current parameters: [1.55816103 1.62375169 1.28153046 1.25793043 2.60285024 2.53236821
 0.09299904 1.52966639 0.92003821 2.84909488 2.81514446 3.13703784], Cost: 0.0002
Current parameters: [1.56007901 1.62471537 1.28120969 1.25786161 2.60367171 2.5317589
 0.09525715 1.53129306 0.91916449 2.84876062 2.81437969 3.13757879], Cost: 0.0002
Current parameters: [1.55781048 1.62225002 1.27943069 1.2609847  2.60434117 2.52653196
 0.09171125 1.52822709 0.91991405 2.84931526 2.81339042 3.13836614], Cost: 0.0007
Current parameters: [1.55504437 1.62197927 1.28258278 1.25750413 2.6033195  2.53274262
 0.09402869 1.5313474  0.91592994 2.85053732 2.81826645 3.14077138], Cost: 0.0012
Current parameters: [1.5588113  1.62536616 1.2812478  1.25819639 2.60301163 2.53320059
 0.09227685 1.53022405 0.92087189 2.85175938 2.8143771  3.13557363], Cost: 0.0002
Current parameters: [1.5591717  1.6256091  1.28118467 1.25847579 2.60226709 2.53268486
 0.09306647 1.52954963 0.92101644 2.85218562 2.81554401 3.13528931], Cost: 0.0002
Current parameters: [1.56022961 1.62606187 1.28194469 1.2589678  2.60298204 2.53422108
 0.09133625 1.52991394 0.92042603 2.8496027  2.81415932 3.13339147], Cost: 0.0001
Current parameters: [1.55971628 1.62646178 1.28096725 1.25921681 2.60290322 2.53410326
 0.09109851 1.53099505 0.92093712 2.84906192 2.81489605 3.13321252], Cost: 0.0001
Current parameters: [1.55866132 1.62927946 1.28162884 1.25766186 2.60352548 2.53337301
 0.09108534 1.53005144 0.92170231 2.84817773 2.81464267 3.13354182], Cost: 0.0001
```



```
Current parameters: [1.55923836 1.62877955 1.28160114 1.25647856 2.6042375  2.53280143
 0.09059139 1.5302119  0.92165359 2.84834296 2.8152987  3.13302469], Cost: 0.0001
Current parameters: [1.55956639 1.62978573 1.27977081 1.25621059 2.60121882 2.53354186
 0.09004178 1.52975462 0.92072673 2.84796954 2.81384139 3.13422109], Cost: 0.0003
Current parameters: [1.55791602 1.62959466 1.28121024 1.25774367 2.60398138 2.53353381
 0.09119018 1.52985199 0.92016423 2.8484449  2.81493251 3.13312658], Cost: 0.0002
Current parameters: [1.55866058 1.62980309 1.28400829 1.25807718 2.60363912 2.53455674
 0.0904941  1.53207493 0.92279956 2.84804902 2.8156676  3.1345523 ], Cost: 0.0002
Current parameters: [1.55823092 1.62922046 1.28230606 1.25775968 2.60278247 2.532166
 0.09091447 1.53068525 0.92135864 2.84826572 2.8141433  3.13295641], Cost: 0.0001
Current parameters: [1.55923289 1.62940943 1.28077358 1.25974446 2.60515023 2.53163325
 0.08993517 1.52936007 0.92290636 2.84788906 2.81388182 3.1340586 ], Cost: 0.0002
Current parameters: [1.5576911  1.62878395 1.28178017 1.25768654 2.60311368 2.53367895
 0.08997459 1.52923584 0.9221457  2.84834292 2.81501713 3.13326241], Cost: 0.0001
Current parameters: [1.55866132 1.62927946 1.28162884 1.25766186 2.60352548 2.53337301
 0.09108534 1.53005144 0.92170231 2.84817773 2.81464267 3.13354182], Cost: 0.0001

   Return from subroutine COBYLA because the MAXFUN limit has been reached.

   NFVALS =  100   F = 7.998527E-05    MAXCV = 0.000000E+00
   X = 1.558661E+00   1.629279E+00   1.281629E+00   1.257662E+00   2.603525E+00
       2.533373E+00   9.108534E-02   1.530051E+00   9.217023E-01   2.848178E+00
       2.814643E+00   3.133542E+00
Simulating for QAOA depth p = 7
Current parameters: [3.13570402 2.27976499 3.01162836 2.32607629 1.87718153 0.52344315
 1.33771993 2.30128277 0.22727179 2.96719724 1.96619427 2.17755305
 0.92837679 1.4768974 ], Cost: 3.4250
Current parameters: [2.13570402 3.27976499 3.01162836 2.32607629 1.87718153 0.52344315
 1.33771993 2.30128277 0.22727179 2.96719724 1.96619427 2.17755305
 0.92837679 1.4768974 ], Cost: 0.4803
Current parameters: [2.13570402 3.27976499 4.01162836 2.32607629 1.87718153 0.52344315
 1.33771993 2.30128277 0.22727179 2.96719724 1.96619427 2.17755305
 0.92837679 1.4768974 ], Cost: 2.6535
Current parameters: [2.13570402 3.27976499 3.01162836 3.32607629 1.87718153 0.52344315
 1.33771993 2.30128277 0.22727179 2.96719724 1.96619427 2.17755305
 0.92837679 1.4768974 ], Cost: 2.5839
Current parameters: [2.13570402 3.27976499 3.01162836 2.32607629 2.87718153 0.52344315
 1.33771993 2.30128277 0.22727179 2.96719724 1.96619427 2.17755305
 0.92837679 1.4768974 ], Cost: 1.8573
Current parameters: [2.13570402 3.27976499 3.01162836 2.32607629 1.87718153 1.52344315
 1.33771993 2.30128277 0.22727179 2.96719724 1.96619427 2.17755305
```

```
 0.92837679 1.4768974 ], Cost: 2.8723
Current parameters: [2.13570402 3.27976499 3.01162836 2.32607629 1.87718153 0.52344315
 2.33771993 2.30128277 0.22727179 2.96719724 1.96619427 2.17755305
 0.92837679 1.4768974 ], Cost: 0.1791
Current parameters: [2.13570402 3.27976499 3.01162836 2.32607629 1.87718153 0.52344315
 2.33771993 3.30128277 0.22727179 2.96719724 1.96619427 2.17755305
 0.92837679 1.4768974 ], Cost: 3.1003
Current parameters: [2.13570402 3.27976499 3.01162836 2.32607629 1.87718153 0.52344315
 2.33771993 2.30128277 1.22727179 2.96719724 1.96619427 2.17755305
 0.92837679 1.4768974 ], Cost: 3.1717
Current parameters: [2.13570402 3.27976499 3.01162836 2.32607629 1.87718153 0.52344315
 2.33771993 2.30128277 0.22727179 3.96719724 1.96619427 2.17755305
 0.92837679 1.4768974 ], Cost: 3.1332
Current parameters: [2.13570402 3.27976499 3.01162836 2.32607629 1.87718153 0.52344315
 2.33771993 2.30128277 0.22727179 2.96719724 2.96619427 2.17755305
 0.92837679 1.4768974 ], Cost: 3.5387
Current parameters: [2.13570402 3.27976499 3.01162836 2.32607629 1.87718153 0.52344315
 2.33771993 2.30128277 0.22727179 2.96719724 1.96619427 3.17755305
 0.92837679 1.4768974 ], Cost: 3.2337
Current parameters: [2.13570402 3.27976499 3.01162836 2.32607629 1.87718153 0.52344315
 2.33771993 2.30128277 0.22727179 2.96719724 1.96619427 2.17755305
 1.92837679 1.4768974 ], Cost: 1.9879
Current parameters: [2.13570402 3.27976499 3.01162836 2.32607629 1.87718153 0.52344315
 2.33771993 2.30128277 0.22727179 2.96719724 1.96619427 2.17755305
 0.92837679 2.4768974 ], Cost: 1.2625
Current parameters: [ 1.94080367  3.43060127  2.75647083  2.07909832  1.71551305  0.24259423
  2.37308805  1.95829565 -0.12409575  2.62035657  1.57174341  1.81891296
  0.71600091  1.34969839], Cost: 1.8928
Current parameters: [2.11431625 3.29631731 3.50024049 2.29897368 1.85944053 0.49262364
 2.34160112 2.2636444  0.18871378 2.929136   1.92290843 2.13819697
 0.90507131 1.46293897], Cost: 1.3466
Current parameters: [2.28871286 3.47747259 3.01162836 2.32607629 1.87718153 0.52344315
 2.33771993 2.30128277 0.22727179 2.96719724 1.96619427 2.17755305
 0.92837679 1.4768974 ], Cost: 0.6824
Current parameters: [2.50685638 2.96072119 2.98794338 2.29979823 1.85998027 0.49356127
 2.34148304 2.26478949 0.18988684 2.93029394 1.92422533 2.13939431
 0.90578033 1.46336363], Cost: 0.9943
Current parameters: [2.13570402 3.27976499 3.01162836 2.46299712 1.6680101  0.52344315
 2.33771993 2.30128277 0.22727179 2.96719724 1.96619427 2.17755305
 0.92837679 1.4768974 ], Cost: 0.3673
Current parameters: [2.10054291 3.26757341 2.98228359 2.72438046 2.15182914 0.48642066
```



```
 2.34238228 2.256069   0.18095329 2.92147548 1.91419637 2.13027585
 0.90038065 1.46012958], Cost: 1.7121
Current parameters: [2.10247926 3.26824481 2.98389963 2.28833014 1.86562968 1.00867361
 2.34212552 2.25855896 0.18350408 2.92399341 1.91705993 2.13287944
 0.90192242 1.461053  ], Cost: 1.8996
Current parameters: [2.11538095 3.27271827 2.99466713 2.30298756 1.87011544 0.49670559
 2.34041476 2.54007879 0.20049976 2.94077013 1.93613956 2.15022689
 0.91219506 1.46720563], Cost: 0.6621
Current parameters: [2.13570402 3.27976499 3.01162836 2.32607629 1.87718153 0.52344315
 2.46209145 2.30128277 0.23979084 2.96719724 1.96619427 2.17755305
 0.92837679 1.4768974 ], Cost: 0.2223
Current parameters: [2.11352811 3.27207583 2.99312078 2.30088257 1.86947123 0.49426794
 2.3105301  2.28663427 0.46367741 2.93836078 1.93339948 2.14773558
 0.91071978 1.46632203], Cost: 0.7834
Current parameters: [2.13570402 3.27976499 3.01162836 2.32607629 1.87718153 0.52344315
 2.33771993 2.30128277 0.22727179 3.06106901 1.88365282 2.17755305
 0.92837679 1.4768974 ], Cost: 0.3936
Current parameters: [2.09750067 3.26651856 2.97974459 2.28267404 1.86389868 0.47318183
 2.33502865 2.2760472  0.19592801 3.09155733 2.15132742 2.12618529
 0.89795835 1.45867878], Cost: 0.8588
Current parameters: [2.13570402 3.27976499 3.01162836 2.32607629 1.87718153 0.52344315
 2.33771993 2.30128277 0.22727179 2.96719724 1.96619427 2.2412447
 0.82082042 1.4768974 ], Cost: 0.1629
Current parameters: [2.10098632 3.26772715 2.98265365 2.28663404 1.8651106  0.47776764
 2.3352742  2.27834968 0.1987878  2.93052354 1.96420144 2.44137665
 0.93702497 1.46034104], Cost: 0.5312
Current parameters: [2.14803294 3.27022346 3.027769   2.34169951 1.88740828 0.54120896
 2.33548263 2.32297929 0.24949844 2.98913752 1.99114626 2.26393139
 0.83425479 1.58500951], Cost: 0.3036
Current parameters: [2.08122918 3.30032331 2.94945929 2.25957297 1.84128184 0.44736194
 2.3427497  2.22660559 0.14799925 2.88419539 1.89384842 2.17365279
 0.77951336 1.59291782], Cost: 1.2326
Current parameters: [2.11619812 3.27111021 3.00237856 2.30708497 1.88719908 0.62094586
 2.30202501 2.28387933 0.20680224 2.92764045 1.97809647 2.22344981
 0.85394077 1.47737479], Cost: 0.3105
Current parameters: [2.14836831 3.28802763 2.99973195 2.33015458 1.8264663  0.64048667
 2.4517564  2.3374795  0.26531365 3.05596018 1.93954779 2.30755072
 0.71046561 1.40677824], Cost: 0.5971
Current parameters: [2.12530392 3.27622758 3.13356859 2.31413618 1.87290324 0.51763183
 2.33831791 2.29485466 0.21918439 2.95722508 1.96538811 2.24059243
 0.81796313 1.46191423], Cost: 0.2473
```



```
Current parameters: [2.17888869 3.24522726 3.01074551 2.30171435 1.86228982 0.52239248
 2.33618848 2.29904388 0.22485405 2.96390099 1.9651198  2.23892257
 0.82149761 1.47639294], Cost: 0.1890
Current parameters: [2.16450668 3.23726239 3.0110122  2.41341829 1.94036878 0.51373264
 2.33907162 2.29081473 0.21392285 2.9507082  1.96545542 2.24101068
 0.81586849 1.45069821], Cost: 0.2507
Current parameters: [2.13492983 3.27935517 3.01110704 2.32504297 1.87745882 0.52262135
 2.34008102 2.34961363 0.18788103 2.96464923 1.96571325 2.23959166
 0.82169622 1.47659952], Cost: 0.1636
Current parameters: [2.1195828  3.26530963 3.00646926 2.3162394  1.88531787 0.50590625
 2.31305317 2.36790058 0.31238984 2.92882167 1.96179161 2.22539846
 0.82605929 1.45246497], Cost: 0.3268
Current parameters: [2.1006235  3.23916226 3.0074564  2.32154465 1.87369046 0.5168665
 2.33899873 2.29798072 0.22333465 2.98696476 1.98734553 2.23497067
 0.81608467 1.47451351], Cost: 0.1460
Current parameters: [2.13032894 3.28420837 3.00220064 2.31034838 1.88566712 0.49602794
 2.31851479 2.29475696 0.21585987 3.02349626 2.08089088 2.21657423
 0.82441673 1.44283103], Cost: 0.5090
Current parameters: [2.10050526 3.23948225 3.00662775 2.28856455 1.92170905 0.52050071
 2.35180034 2.29844583 0.22477117 2.99684398 1.97907381 2.24086383
 0.80460424 1.47427582], Cost: 0.1256
Current parameters: [2.09963168 3.2368443  3.00194197 2.30529089 1.88867731 0.57969729
 2.40868015 2.31413478 0.24123007 3.03544143 1.95744183 2.27481543
 0.75097843 1.44294331], Cost: 0.2749
Current parameters: [2.10406601 3.24393655 3.00823597 2.29223317 1.92156284 0.52482517
 2.35361494 2.29763974 0.22495478 2.99624743 1.97563332 2.19005104
 0.77239476 1.48853774], Cost: 0.3734
Current parameters: [2.064326   3.23228898 2.98105398 2.26739062 1.91257104 0.46957422
 2.36918986 2.2684997  0.19254406 2.95459894 1.9454637  2.31146856
 0.82052978 1.48398502], Cost: 0.0877
Current parameters: [2.06621756 3.23040895 2.98413088 2.26113787 1.92900322 0.46953038
 2.34607345 2.27157692 0.19361791 2.99334913 1.91813786 2.31203299
 0.8474694  1.47616043], Cost: 0.0902
Current parameters: [2.03662029 3.22792073 2.96177399 2.30738029 1.82809715 0.44448217
 2.36368376 2.24766547 0.16732731 2.95590481 1.88299259 2.31226266
 0.81873709 1.4922607 ], Cost: 0.3444
Current parameters: [2.06808209 3.28295967 2.96833298 2.26917041 1.91201296 0.46015305
 2.36401237 2.24419188 0.2021332  2.96588666 1.9537992  2.3165752
 0.8133554  1.47468493], Cost: 0.0593
Current parameters: [2.08152172 3.27456557 2.96301282 2.24997763 1.90988379 0.43319621
 2.36202673 2.25820913 0.17210847 2.98719777 2.04129635 2.36139121
```



```
 0.85043655 1.43405572], Cost: 0.2397
Current parameters: [2.05661747 3.28689348 2.96079078 2.26500703 1.91330332 0.44836045
 2.35326194 2.26940695 0.24759025 2.94583576 1.9477521  2.32284299
 0.81741239 1.45944276], Cost: 0.0221
Current parameters: [2.05618029 3.28354586 2.9612677  2.2638412  1.91376477 0.45289915
 2.3302     2.27000456 0.24298084 2.94433664 1.94918518 2.33278394
 0.8005051  1.46001652], Cost: 0.0255
Current parameters: [2.04175241 3.29527348 2.95062532 2.25794121 1.91728186 0.49194202
 2.34454437 2.25432847 0.24598101 2.92223304 1.94823348 2.32245952
 0.84229561 1.4514413 ], Cost: 0.1125
Current parameters: [2.05193946 3.29259371 2.96319706 2.2484717  1.90129014 0.4523362
 2.35278466 2.27553729 0.25179642 2.95239035 1.95162981 2.32547818
 0.82001005 1.47830181], Cost: 0.0350
Current parameters: [2.06468604 3.28778443 2.96138872 2.29980118 1.93470978 0.44869193
 2.35484506 2.26758873 0.26515841 2.94640496 1.95215451 2.34781927
 0.82530356 1.49336584], Cost: 0.0377
Current parameters: [2.05484828 3.28885723 2.96743086 2.26400085 1.91684797 0.43358417
 2.33986028 2.26400638 0.24321445 2.93461831 1.9520345  2.31026416
 0.82698712 1.4679032 ], Cost: 0.0221
Current parameters: [2.04749753 3.29849674 3.02392666 2.26407535 1.91533231 0.43477581
 2.34679551 2.25649218 0.24973623 2.93121946 1.94592955 2.3243838
 0.82374901 1.45533215], Cost: 0.0211
Current parameters: [2.02345553 3.30449418 3.02368897 2.27172164 1.9229956  0.43471832
 2.34863631 2.26677903 0.24244355 2.93694701 1.95035674 2.32023132
 0.82012194 1.45569197], Cost: 0.0189
Current parameters: [2.03220417 3.30858796 3.02570406 2.25200487 1.95935747 0.42337288
 2.35969706 2.26778183 0.26016239 2.94031456 1.94246768 2.29920126
 0.78834271 1.45817639], Cost: 0.0227
Current parameters: [2.01102172 3.29032491 3.02214664 2.27175702 1.91950523 0.43244324
 2.34792354 2.24837097 0.25452837 2.94504092 1.95680402 2.31811805
 0.81808947 1.45508143], Cost: 0.0157
Current parameters: [2.00275319 3.29620416 3.01491126 2.29020777 1.89288262 0.4004474
 2.34992921 2.24743651 0.25883848 2.94523685 1.91961089 2.3157129
 0.80183508 1.45398241], Cost: 0.0324
Current parameters: [2.01772714 3.29282313 3.02785578 2.28430579 1.91153587 0.44395428
 2.34174199 2.25333368 0.26300044 2.95045446 1.95988522 2.29931517
 0.81463355 1.45568973], Cost: 0.0048
Current parameters: [2.03156712 3.29166281 3.03140608 2.29029332 1.90653327 0.41593843
 2.33896587 2.27226591 0.2713137  2.96842884 1.99738886 2.30142099
 0.81512027 1.42950825], Cost: 0.1387
Current parameters: [2.01659876 3.29385337 3.02619713 2.28734161 1.90337505 0.44458686
```

```
 2.35319301 2.25155871 0.26236417 2.93079823 1.97355645 2.29901778
 0.80149776 1.45952026], Cost: 0.0113
Current parameters: [2.00062915 3.30778714 3.01800459 2.2830704  1.92098017 0.47955919
 2.33365535 2.23449541 0.27450386 2.92801944 1.9362667  2.28780415
 0.8259723  1.44796494], Cost: 0.0515
Current parameters: [2.01510624 3.26813777 3.03417089 2.28422823 1.9126825  0.44762176
 2.34436606 2.26547999 0.26103636 2.94413773 1.95176141 2.29500711
 0.81631576 1.46017437], Cost: 0.0025
Current parameters: [2.01448257 3.2674868  3.03475311 2.27699827 1.90787105 0.44865319
 2.34515723 2.26331015 0.25252395 2.94592297 1.95131542 2.28868386
 0.81415578 1.45379688], Cost: 0.0012
Current parameters: [2.02064578 3.26526805 3.03909361 2.28730531 1.91286315 0.45342041
 2.35116149 2.25070901 0.23705473 2.95500826 1.94861185 2.28753636
 0.81071665 1.4687568 ], Cost: 0.0209
Current parameters: [2.01409444 3.26983512 3.03483095 2.27544294 1.9061087  0.44696906
 2.35624195 2.26380685 0.25529065 2.94748738 1.95111969 2.28421705
 0.82265996 1.45593613], Cost: 0.0012
Current parameters: [2.00866323 3.27423599 3.03774476 2.25915189 1.89488259 0.45151226
 2.35008791 2.26986312 0.25962453 2.95322637 1.9510966  2.2869999
 0.81867939 1.47483806], Cost: 0.0064
Current parameters: [2.01319863 3.27082743 3.03813743 2.27497168 1.90783357 0.43952865
 2.3495436  2.26110653 0.25310768 2.94187765 1.95319829 2.27792553
 0.82742295 1.46016456], Cost: 0.0050
Current parameters: [2.01048629 3.2725936  3.03121187 2.28422397 1.89286583 0.43242946
 2.35728255 2.26393524 0.25764489 2.94734647 1.93110785 2.28253189
 0.81435717 1.4557232 ], Cost: 0.0109
Current parameters: [2.01227464 3.27223934 3.04895638 2.27548476 1.90569053 0.44722024
 2.35796306 2.26196245 0.25691499 2.9466642  1.94966823 2.28777926
 0.82188286 1.45274222], Cost: 0.0022
Current parameters: [2.01874948 3.26269739 3.03583096 2.27539646 1.90285611 0.44164561
 2.35364926 2.27877768 0.26207009 2.95922476 1.96655231 2.2828933
 0.82455519 1.44158549], Cost: 0.0319
Current parameters: [2.0114022  3.26915386 3.03411385 2.28033765 1.89730887 0.45042557
 2.35340425 2.26327136 0.25099258 2.94618069 1.95268023 2.28928528
 0.83074447 1.45518134], Cost: 0.0026
Current parameters: [2.005986   3.2742361  3.0294918  2.27144511 1.9127108  0.46606279
 2.35323833 2.25855778 0.26385916 2.93728283 1.94027964 2.27705831
 0.82648553 1.44951086], Cost: 0.0103
Current parameters: [2.00043213 3.2704733  3.03378354 2.27738518 1.91025402 0.44516134
 2.3578052  2.26557547 0.25236315 2.9504994  1.95361508 2.28444042
 0.82115571 1.45594614], Cost: 0.0016
```



```
Current parameters: [2.00999833 3.25580524 3.02993467 2.2638309  1.90642818 0.43910983
 2.35889444 2.24517977 0.26146759 2.9475297  1.95160117 2.29607302
 0.825098   1.45410191], Cost: 0.0081
Current parameters: [2.01789996 3.27712938 3.03140286 2.26514631 1.91496065 0.44236022
 2.36084761 2.27500299 0.24195692 2.93809352 1.94379774 2.29848161
 0.82704161 1.45431828], Cost: 0.0090
Current parameters: [2.01344148 3.27005523 3.03381459 2.27651188 1.90219586 0.44792717
 2.36202351 2.26348221 0.25534306 2.93745847 1.95791175 2.2839144
 0.81610895 1.45735344], Cost: 0.0025
Current parameters: [2.01456419 3.27601944 3.0331393  2.27515591 1.90567499 0.44592178
 2.35541713 2.26116066 0.25622111 2.94879033 1.95321221 2.2853133
 0.82234127 1.45438318], Cost: 0.0012
Current parameters: [2.01760398 3.27048068 3.03584229 2.27992895 1.90932829 0.45157706
 2.35982392 2.25715905 0.24811999 2.95224168 1.95070576 2.28361501
 0.82028087 1.46237207], Cost: 0.0061
Current parameters: [2.01577614 3.2683429  3.03509411 2.27579979 1.90597593 0.44690985
 2.35600899 2.2674552  0.2569297  2.95055212 1.95513322 2.2829792
 0.82251956 1.45237828], Cost: 0.0019
Current parameters: [2.01370851 3.27015849 3.03535472 2.27315091 1.90564494 0.44832559
 2.35551538 2.26447417 0.25564699 2.94813536 1.95227527 2.28464189
 0.82269752 1.45803016], Cost: 0.0010
Current parameters: [2.01285293 3.27101865 3.03475665 2.27322083 1.90109229 0.44615156
 2.35517345 2.26649459 0.25727055 2.95004846 1.94874942 2.2839907
 0.81976411 1.45936886], Cost: 0.0023
Current parameters: [2.01304976 3.26825784 3.03515802 2.27287567 1.9049618  0.44794199
 2.3554263  2.26186478 0.25726631 2.94892045 1.95291076 2.28463677
 0.8225474  1.45798259], Cost: 0.0007
Current parameters: [2.01363055 3.26666084 3.03351309 2.26859765 1.90607318 0.44529667
 2.35698058 2.26198837 0.25627815 2.94853869 1.95245773 2.28943254
 0.82316816 1.45665441], Cost: 0.0010
Current parameters: [2.01263063 3.26824832 3.03517383 2.27102412 1.90400324 0.44875078
 2.35547621 2.26115813 0.25547115 2.94898934 1.95247789 2.28287412
 0.82219636 1.45626947], Cost: 0.0005
Current parameters: [2.01102101 3.26858354 3.03320844 2.26964455 1.90603688 0.45301218
 2.35483395 2.26101169 0.25953074 2.94708424 1.95031623 2.28259875
 0.82325369 1.45430465], Cost: 0.0006
Current parameters: [2.01599852 3.26811804 3.03552806 2.2705283  1.90302272 0.44898809
 2.35489737 2.26064202 0.25613616 2.94808162 1.95191723 2.28263964
 0.82270549 1.45638729], Cost: 0.0007
Current parameters: [2.0108095  3.26817915 3.03581656 2.26928291 1.90572808 0.44506829
 2.35193774 2.26054915 0.25560537 2.94648804 1.95376612 2.27969732
```



```
 0.82467197 1.45709816], Cost: 0.0010
Current parameters: [2.01253131 3.26883611 3.03519037 2.270639   1.90354959 0.44833306
 2.35826592 2.26128116 0.25616281 2.94934768 1.9524511  2.28175684
 0.82430119 1.45680873], Cost: 0.0006
Current parameters: [2.01198141 3.268117   3.03441317 2.27096816 1.90228558 0.44915458
 2.35797095 2.26116856 0.25593339 2.94377189 1.95604196 2.28266296
 0.81896714 1.45652828], Cost: 0.0007
Current parameters: [2.01175122 3.26803497 3.03490139 2.27186989 1.90169405 0.44924695
 2.35454949 2.26150118 0.25495067 2.94834867 1.95287945 2.28410874
 0.82428013 1.45564789], Cost: 0.0005
Current parameters: [2.01253814 3.26712959 3.03404425 2.27188123 1.90556864 0.45265707
 2.35568755 2.25701799 0.25179225 2.95063253 1.95394728 2.28308421
 0.82275106 1.45830027], Cost: 0.0017
Current parameters: [2.01139017 3.26915434 3.04214227 2.27064508 1.90417786 0.44933613
 2.35615782 2.25979139 0.25621576 2.94855695 1.952105   2.28467094
 0.82190698 1.4545161 ], Cost: 0.0007
Current parameters: [2.01323206 3.26734314 3.0353474  2.27093717 1.90418636 0.44902452
 2.3552549  2.26265262 0.2562518  2.95048826 1.95468895 2.28230086
 0.82218572 1.45437861], Cost: 0.0002
Current parameters: [2.01311075 3.26580133 3.03576724 2.27100998 1.90427353 0.44927563
 2.35545969 2.2633233  0.25602674 2.95017137 1.954149   2.28202366
 0.82225159 1.45477238], Cost: 0.0002
Current parameters: [2.01227035 3.26550035 3.03556916 2.27050708 1.90215443 0.44870818
 2.35496671 2.26438628 0.25720137 2.95179261 1.95322144 2.28147468
 0.82058909 1.45454775], Cost: 0.0006
Current parameters: [2.01319233 3.26548978 3.03542437 2.26992276 1.90447111 0.44847052
 2.35578773 2.26358711 0.25597368 2.94998825 1.95393412 2.28312811
 0.8223358  1.45428503], Cost: 0.0002
Current parameters: [2.01342224 3.26408289 3.03486798 2.26950808 1.90554282 0.45007702
 2.35609763 2.26231937 0.25432448 2.95130704 1.95542116 2.28383728
 0.82269132 1.45417189], Cost: 0.0001
Current parameters: [2.01309635 3.26424196 3.03518896 2.26837704 1.90558769 0.45011069
 2.35532579 2.26257131 0.25446644 2.95122076 1.95606584 2.28361461
 0.82304597 1.45518183], Cost: 0.0001
Current parameters: [2.01293414 3.26308798 3.03477843 2.26991713 1.90695967 0.44826613
 2.35492795 2.26152605 0.25383669 2.9505403  1.95610974 2.28233982
 0.82372202 1.4527696 ], Cost: 0.0002
Current parameters: [2.01342224 3.26408289 3.03486798 2.26950808 1.90554282 0.45007702
 2.35609763 2.26231937 0.25432448 2.95130704 1.95542116 2.28383728
 0.82269132 1.45417189], Cost: 0.0001
```



```
   Return from subroutine COBYLA because the MAXFUN limit has been reached.

   NFVALS =  100   F = 1.141748E-04    MAXCV = 0.000000E+00
   X = 2.013422E+00   3.264083E+00   3.034868E+00   2.269508E+00   1.905543E+00
       4.500770E-01   2.356098E+00   2.262319E+00   2.543245E-01   2.951307E+00
       1.955421E+00   2.283837E+00   8.226913E-01   1.454172E+00
Simulating for QAOA depth p = 8
Current parameters: [1.1043875  2.09457375 1.29429767 2.12748515 1.8374286  2.2376549
 0.78601725 3.07957845 2.44126926 1.5125033  0.9774504  2.59690529
 0.80988902 1.69676253 0.81390418 2.7446489 ], Cost: 1.6703
Current parameters: [1.1043875  3.09457375 1.29429767 2.12748515 1.8374286  2.2376549
 0.78601725 3.07957845 2.44126926 1.5125033  0.9774504  2.59690529
 0.80988902 1.69676253 0.81390418 2.7446489 ], Cost: 0.9078
Current parameters: [1.1043875  3.09457375 2.29429767 2.12748515 1.8374286  2.2376549
 0.78601725 3.07957845 2.44126926 1.5125033  0.9774504  2.59690529
 0.80988902 1.69676253 0.81390418 2.7446489 ], Cost: 1.2120
Current parameters: [1.1043875  3.09457375 1.29429767 3.12748515 1.8374286  2.2376549
 0.78601725 3.07957845 2.44126926 1.5125033  0.9774504  2.59690529
 0.80988902 1.69676253 0.81390418 2.7446489 ], Cost: 3.2193
Current parameters: [1.1043875  3.09457375 1.29429767 2.12748515 2.8374286  2.2376549
 0.78601725 3.07957845 2.44126926 1.5125033  0.9774504  2.59690529
 0.80988902 1.69676253 0.81390418 2.7446489 ], Cost: 0.8550
Current parameters: [1.1043875  3.09457375 1.29429767 2.12748515 2.8374286  3.2376549
 0.78601725 3.07957845 2.44126926 1.5125033  0.9774504  2.59690529
 0.80988902 1.69676253 0.81390418 2.7446489 ], Cost: 2.0376
Current parameters: [1.1043875  3.09457375 1.29429767 2.12748515 2.8374286  2.2376549
 1.78601725 3.07957845 2.44126926 1.5125033  0.9774504  2.59690529
 0.80988902 1.69676253 0.81390418 2.7446489 ], Cost: 2.6452
Current parameters: [1.1043875  3.09457375 1.29429767 2.12748515 2.8374286  2.2376549
 0.78601725 4.07957845 2.44126926 1.5125033  0.9774504  2.59690529
 0.80988902 1.69676253 0.81390418 2.7446489 ], Cost: 1.6720
Current parameters: [1.1043875  3.09457375 1.29429767 2.12748515 2.8374286  2.2376549
 0.78601725 3.07957845 3.44126926 1.5125033  0.9774504  2.59690529
 0.80988902 1.69676253 0.81390418 2.7446489 ], Cost: 2.4097
Current parameters: [1.1043875  3.09457375 1.29429767 2.12748515 2.8374286  2.2376549
 0.78601725 3.07957845 2.44126926 2.5125033  0.9774504  2.59690529
 0.80988902 1.69676253 0.81390418 2.7446489 ], Cost: 2.3193
Current parameters: [1.1043875  3.09457375 1.29429767 2.12748515 2.8374286  2.2376549
 0.78601725 3.07957845 2.44126926 1.5125033  1.9774504  2.59690529
 0.80988902 1.69676253 0.81390418 2.7446489 ], Cost: 3.1748
Current parameters: [1.1043875  3.09457375 1.29429767 2.12748515 2.8374286  2.2376549
```

```
 0.78601725 3.07957845 2.44126926 1.5125033  0.9774504  3.59690529
 0.80988902 1.69676253 0.81390418 2.7446489 ], Cost: 1.6443
Current parameters: [1.1043875  3.09457375 1.29429767 2.12748515 2.8374286  2.2376549
 0.78601725 3.07957845 2.44126926 1.5125033  0.9774504  2.59690529
 1.80988902 1.69676253 0.81390418 2.7446489 ], Cost: 2.0867
Current parameters: [1.1043875  3.09457375 1.29429767 2.12748515 2.8374286  2.2376549
 0.78601725 3.07957845 2.44126926 1.5125033  0.9774504  2.59690529
 0.80988902 2.69676253 0.81390418 2.7446489 ], Cost: 2.7857
Current parameters: [1.1043875  3.09457375 1.29429767 2.12748515 2.8374286  2.2376549
 0.78601725 3.07957845 2.44126926 1.5125033  0.9774504  2.59690529
 0.80988902 1.69676253 1.81390418 2.7446489 ], Cost: 2.1568
Current parameters: [1.1043875  3.09457375 1.29429767 2.12748515 2.8374286  2.2376549
 0.78601725 3.07957845 2.44126926 1.5125033  0.9774504  2.59690529
 0.80988902 1.69676253 0.81390418 3.7446489 ], Cost: 2.2053
Current parameters: [1.38132595 3.22698377 1.24147777 1.72610548 2.84660836 2.03229638
 0.47514794 2.9376919  2.17128116 1.25821463 0.57461568 2.45984228
 0.59600497 1.36149689 0.58784867 2.51017015], Cost: 2.5673
Current parameters: [0.61586154 3.1092495  1.28844334 2.08299797 2.83844604 2.21489385
 0.75156184 3.06385236 2.41134495 1.48431904 0.93280195 2.58171382
 0.78618304 1.65960314 0.78884917 2.7186603 ], Cost: 1.7391
Current parameters: [1.1043875  3.00194399 1.06209145 2.12748515 2.8374286  2.2376549
 0.78601725 3.07957845 2.44126926 1.5125033  0.9774504  2.59690529
 0.80988902 1.69676253 0.81390418 2.7446489 ], Cost: 0.9088
Current parameters: [1.1173968  2.63373914 1.48038417 2.10499885 2.83794287 2.2261502
 0.76860157 3.07162961 2.42614384 1.4982574  0.95488258 2.58922667
 0.7979067  1.67798011 0.80123998 2.73151281], Cost: 1.1163
Current parameters: [1.14428558 3.10586884 1.29670533 2.60327322 2.83900582 2.20237125
 0.73260527 3.05520023 2.39488128 1.46881271 0.90823739 2.57335582
 0.77314056 1.6391589  0.77506447 2.70436196], Cost: 2.0770
Current parameters: [1.2118937  3.13727422 1.2849943  1.98034452 2.8391922  2.19820184
 0.72629367 3.0523195  2.3893997  1.46364988 0.90005862 2.57057303
 0.76879806 1.63235199 0.77047486 2.69960133], Cost: 1.6932
Current parameters: [1.1043875  3.09457375 1.29429767 2.12748515 2.96237414 2.2376549
 0.7897068  3.07957845 2.44126926 1.5125033  0.9774504  2.59690529
 0.80988902 1.69676253 0.81390418 2.7446489 ], Cost: 0.9666
Current parameters: [1.11012352 3.09509564 1.29606249 2.1213853  2.82254777 2.22758532
 1.03229736 3.07262117 2.42803063 1.50003447 0.95769775 2.59018452
 0.79940141 1.68032308 0.80281975 2.73315144], Cost: 1.2281
Current parameters: [1.1043875  3.09457375 1.29429767 2.12748515 2.8374286  2.30870973
 0.78601725 2.97673774 2.44126926 1.5125033  0.9774504  2.59690529
 0.80988902 1.69676253 0.81390418 2.7446489 ], Cost: 0.7998
```



```
Current parameters: [1.10912242 3.09500456 1.29575448 2.12244989 2.83125587 2.51439254
 0.78236856 3.11507856 2.43034114 1.50221064 0.96114515 2.59135749
 0.80123179 1.68319222 0.80475429 2.73515807], Cost: 1.1616
Current parameters: [1.1043875  3.09457375 1.29429767 2.12748515 2.8374286  2.30870973
 0.78601725 2.97673774 2.52697241 1.42150895 0.9774504  2.59690529
 0.80988902 1.69676253 0.81390418 2.7446489 ], Cost: 0.7672
Current parameters: [1.111906   3.09525782 1.29661091 2.11948976 2.82762703 2.30591527
 0.78022357 2.968821   2.70776514 1.58778632 0.95155958 2.58809604
 0.79614236 1.67521449 0.79937524 2.72957859], Cost: 2.3088
Current parameters: [1.1043875  3.09457375 1.29429767 2.12748515 2.8374286  2.30870973
 0.78601725 2.97673774 2.52697241 1.42150895 1.01771436 2.47856758
 0.80988902 1.69676253 0.81390418 2.7446489 ], Cost: 0.9747
Current parameters: [1.11223207 3.09528749 1.29671124 2.119143   2.82720194 2.30579408
 0.7799723  2.96847765 2.49093167 1.38338782 1.1988865  2.69266726
 0.79554617 1.67427995 0.79874512 2.72892499], Cost: 0.0607
Current parameters: [1.12022366 3.0960146  1.29917004 2.1106445  2.8167836  2.30282378
 0.77381405 2.96006275 2.45421545 1.3445522  1.22989585 2.72401994
 0.7809345  1.91223329 0.78330195 2.71290638], Cost: 0.3247
Current parameters: [1.11223207 3.09528749 1.29671124 2.119143   2.82720194 2.30579408
 0.7799723  2.96847765 2.49093167 1.38338782 1.1988865  2.69266726
 0.79554617 1.67427995 0.70875519 2.81568219], Cost: 0.0867
Current parameters: [1.1202805  3.09601977 1.29918753 2.11058406 2.8167095  2.30280266
 0.77377026 2.9600029  2.45395431 1.344276   1.2301164  2.72424293
 0.78083058 1.66053583 0.96600962 2.89884769], Cost: 0.9591
Current parameters: [1.10846304 3.09348544 1.2974301  2.0673458  2.82702995 2.3096415
 0.78579648 2.97113591 2.49598993 1.38815195 1.20643366 2.69523515
 0.9081505  1.6805612  0.8029803  2.73331798], Cost: 0.1070
Current parameters: [1.13654673 3.10300396 1.29710606 2.30379825 2.81468777 2.28752782
 0.75022141 2.94781827 2.42546186 1.31634982 1.20959507 2.72259271
 0.88868694 1.63334322 0.74813672 2.6719467 ], Cost: 0.4473
Current parameters: [1.07479259 3.20595356 1.25256525 2.12135555 2.82715133 2.30692609
 0.78168593 2.96925978 2.49241994 1.38478955 1.20110708 2.6934228
 0.79672518 1.67612806 0.79999122 2.73021752], Cost: 0.1110
Current parameters: [0.89558219 3.03260948 1.3238535  2.07444284 2.81958987 2.29238863
 0.75838597 2.95426386 2.44777235 1.33942721 1.20125738 2.70966281
 0.77224687 1.64554759 0.76520079 2.69136865], Cost: 0.3072
Current parameters: [1.21834091 3.13034144 1.28567605 1.97900576 2.81727731 2.27465866
 0.73105748 2.94014136 2.41480641 1.30712616 1.17731851 2.70763953
 0.75024991 1.61401274 0.73857928 2.66271069], Cost: 0.4356
Current parameters: [1.11548607 3.09436632 1.29775003 2.11670071 2.82101047 2.30250066
 0.9019766  2.96464827 2.47858865 1.37072353 1.2014221  2.6986102
```



```
 0.78918589 1.66675457 0.78922398 2.71818231], Cost: 0.1538
Current parameters: [1.11229437 3.11842831 1.35472609 2.11907675 2.82720328 2.30555524
 0.77992673 2.96831263 2.49252789 1.38489122 1.19867196 2.69259426
 0.79543226 1.67410139 0.79862473 2.72880011], Cost: 0.1024
Current parameters: [1.11800336 3.08575831 1.2872645  2.11761396 2.81564542 2.29839844
 0.77451815 2.95633448 2.56611992 1.44950112 1.24120913 2.73006202
 0.78313494 1.66588949 0.77295887 2.69825464], Cost: 0.5445
Current parameters: [1.11284528 3.09534328 1.29689991 2.11849089 2.77377084 2.3322035
 0.77794558 2.98672448 2.48951638 1.38205483 1.19677483 2.69194877
 0.79442498 1.67252248 0.79756013 2.72769584], Cost: 0.0645
Current parameters: [1.11489283 3.09321065 1.29576567 2.11764796 2.89107859 2.39280707
 0.77965353 3.02656003 2.4827075  1.37428001 1.20773499 2.70233678
 0.79014293 1.66901434 0.78936575 2.71806002], Cost: 0.1832
Current parameters: [1.11223207 3.09528749 1.29671124 2.119143   2.82720194 2.27145898
 0.7799723  3.01817232 2.49093167 1.38338782 1.19371472 2.70786736
 0.79554617 1.67427995 0.79874512 2.72892499], Cost: 0.0707
Current parameters: [1.11290342 3.09293897 1.29508459 2.11981574 2.82668    2.28745637
 0.77919826 2.99345022 2.48721756 1.37847456 1.25571443 2.58635863
 0.79375927 1.67480828 0.79304442 2.72183134], Cost: 0.1947
Current parameters: [1.10433318 3.09313931 1.2976317  2.11750908 2.82697875 2.3053314
 0.7792335  2.96801092 2.5319273  1.33702461 1.19748764 2.6926721
 0.7948813  1.67323775 0.79804241 2.7281961 ], Cost: 0.0597
Current parameters: [0.99717629 3.0613308  1.30848777 2.09590559 2.82338118 2.29885615
 0.76832933 2.96021272 2.49595918 1.32714906 1.19740284 2.70079695
 0.78391974 1.65956242 0.78244148 2.71077029], Cost: 0.1287
Current parameters: [1.11177366 3.08146315 1.28514675 2.15788686 2.83588229 2.30042558
 0.76729352 2.94199972 2.5074357  1.3358861  1.19530059 2.69811986
 0.80792663 1.66501752 0.79839742 2.71669144], Cost: 0.0495
Current parameters: [1.10945173 3.11269675 1.31530312 2.20382165 2.8008929  2.29859526
 0.77395772 2.9940842  2.51331895 1.27691926 1.20519635 2.70739033
 0.85242509 1.65421112 0.75742694 2.70369895], Cost: 0.2356
Current parameters: [1.1141698  3.08212802 1.28633335 2.15503586 2.83225655 2.29954905
 0.76558583 2.94011453 2.496391   1.32339039 1.24028965 2.71987405
 0.80355146 1.65485938 0.81984176 2.7393314 ], Cost: 0.1043
Current parameters: [1.10778642 3.0713298  1.27943183 2.16886037 2.83400356 2.30013165
 0.76550886 2.94207193 2.49802958 1.32342854 1.11615714 2.67217178
 0.8087611  1.66332958 0.8627366  2.78011701], Cost: 0.0850
Current parameters: [1.09944596 3.07581661 1.28691633 2.17548973 2.83550816 2.30393258
 0.77235489 2.94328161 2.50973714 1.3386342  1.20429658 2.70245453
 0.81027637 1.7215212  0.8039373  2.72200726], Cost: 0.0136
Current parameters: [1.1438322  3.08211567 1.27477294 2.12241749 2.83091683 2.2895188
```



```
 0.74714205 2.92174718 2.45768937 1.29194921 1.18002399 2.71063926
 0.78757011 1.77190952 0.78843174 2.70002506], Cost: 0.2416
Current parameters: [1.10581889 3.06852956 1.27739111 2.18013015 2.84349166 2.29306546
 0.76525053 2.93832109 2.49929335 1.34435109 1.21606081 2.65083387
 0.80930732 1.70955366 0.79563698 2.70511167], Cost: 0.0604
Current parameters: [1.07682521 3.07970295 1.30333427 2.17299578 2.8176424  2.3032358
 0.78784895 2.98614691 2.52124693 1.31674983 1.25399824 2.66550693
 0.80596309 1.77500739 0.84639124 2.77942473], Cost: 0.2401
Current parameters: [1.09885908 3.06285585 1.27672445 2.18670554 2.83179821 2.300253
 0.76547364 2.92750469 2.54603085 1.37584042 1.19515376 2.72017769
 0.80621793 1.71911229 0.80258976 2.71417387], Cost: 0.0707
Current parameters: [1.09989159 3.07805736 1.28857618 2.18767506 2.83734201 2.30791227
 0.77191719 2.94859822 2.50946622 1.33515363 1.20274224 2.70301318
 0.78296515 1.71954841 0.80135056 2.72098503], Cost: 0.0262
Current parameters: [1.10106299 3.06666439 1.27949278 2.17250033 2.87206876 2.3469789
 0.76871906 2.96099598 2.50529755 1.34422556 1.2025778  2.70712489
 0.82169117 1.72036971 0.80514929 2.71650154], Cost: 0.0278
Current parameters: [1.090892   3.10021731 1.27517544 2.17630587 2.8376722  2.30477295
 0.76030381 2.94191832 2.51015648 1.3415751  1.20479167 2.7016011
 0.81124886 1.7227948  0.80594255 2.72279472], Cost: 0.0140
Current parameters: [1.09405789 3.09065205 1.26852836 2.177214   2.84122941 2.30526671
 0.82685142 2.93076976 2.50133377 1.34289791 1.20092254 2.70472349
 0.8100775  1.71906258 0.80515634 2.7155105 ], Cost: 0.0274
Current parameters: [1.09450968 3.07576578 1.29097736 2.17444129 2.83217537 2.30313873
 0.77172937 2.94695817 2.51065894 1.33549201 1.20376397 2.7036552
 0.80970233 1.72076899 0.82466426 2.70042011], Cost: 0.0127
Current parameters: [1.04087938 3.05839605 1.29399523 2.16562573 2.83234543 2.30119548
 0.76709248 2.94045084 2.49409281 1.33441887 1.19852952 2.70877973
 0.80615544 1.71412971 0.81097355 2.69970217], Cost: 0.0310
Current parameters: [1.09532219 3.07206562 1.28856986 2.1758904  2.83490821 2.30243261
 0.76977719 2.94187176 2.50599939 1.33657377 1.18384792 2.69692278
 0.80827806 1.71607839 0.84052041 2.71370022], Cost: 0.0156
Current parameters: [1.09330781 3.08562455 1.30004682 2.20530478 2.82010489 2.30341413
 0.77357718 2.96362912 2.51441937 1.31555838 1.17954842 2.70605339
 0.83772787 1.70497569 0.80861462 2.69337684], Cost: 0.0696
Current parameters: [1.10514518 3.06643887 1.29490819 2.16417514 2.82748293 2.29712124
 0.76941459 2.94014031 2.50282169 1.32788112 1.1979381  2.70975359
 0.81764295 1.73224989 0.8137608  2.70684759], Cost: 0.0183
Current parameters: [1.10145792 3.09504535 1.26900396 2.159263   2.83995547 2.30978789
 0.76122484 2.94063641 2.49892003 1.32749786 1.18821538 2.71229505
 0.78556293 1.7335531  0.83141197 2.66566068], Cost: 0.0220
```

```
Current parameters: [1.08821535 3.07796718 1.28908071 2.17073963 2.82859824 2.30555013
 0.77541652 2.95666458 2.515365   1.33087225 1.21196618 2.69869371
 0.80770172 1.74162614 0.83660489 2.70886964], Cost: 0.0237
Current parameters: [1.09545014 3.07608488 1.29113594 2.17447752 2.82135958 2.31208924
 0.77139438 2.94557456 2.50614355 1.34001579 1.20401345 2.70193874
 0.80954314 1.72051946 0.82449601 2.70024559], Cost: 0.0122
Current parameters: [1.09528369 3.08799388 1.3148592  2.17381491 2.82982595 2.31443378
 0.77215909 2.93626704 2.50236239 1.34766026 1.20442332 2.70068135
 0.80873369 1.72555552 0.8267192  2.69896341], Cost: 0.0127
Current parameters: [1.09489742 3.07398251 1.28902284 2.18261139 2.82275142 2.31502409
 0.76948753 2.93628628 2.50528122 1.3345281  1.20867823 2.70342734
 0.81096157 1.72257335 0.82718219 2.70001998], Cost: 0.0163
Current parameters: [1.09774255 3.07485372 1.2879328  2.18564746 2.82351859 2.30197473
 0.76930665 2.95472306 2.48774223 1.35275643 1.20836323 2.71061586
 0.81139674 1.72510928 0.82895712 2.69980292], Cost: 0.0205
Current parameters: [1.09514486 3.07897055 1.29262914 2.17194388 2.81970332 2.31433851
 0.77215447 2.94647808 2.50713433 1.33750747 1.20494363 2.71447874
 0.80833183 1.71643438 0.82836463 2.70464598], Cost: 0.0152
Current parameters: [1.10279372 3.0771983  1.29068407 2.16073993 2.82276401 2.30779165
 0.76823932 2.94315043 2.49968818 1.33807499 1.21949954 2.68962669
 0.80845874 1.70496947 0.82838055 2.70137903], Cost: 0.0260
Current parameters: [1.09590414 3.07351993 1.29456146 2.17512398 2.82045595 2.31252481
 0.75727964 2.94859728 2.5096658  1.3404601  1.20431741 2.70227273
 0.80817774 1.72091135 0.82546989 2.69999956], Cost: 0.0113
Current parameters: [1.09801557 3.06760052 1.28965686 2.17160241 2.81892289 2.309032
 0.75919127 2.93798151 2.52724143 1.36117849 1.20305647 2.70639289
 0.80805276 1.72290343 0.82525827 2.69700615], Cost: 0.0198
Current parameters: [1.09443171 3.07824143 1.29618587 2.17802772 2.81139245 2.30272181
 0.75715    2.94572665 2.5109615  1.33802129 1.20354254 2.70133891
 0.80475895 1.72039792 0.82616735 2.6992452 ], Cost: 0.0104
Current parameters: [1.09625992 3.08713247 1.30092877 2.1873091  2.80826417 2.30677691
 0.7575712  2.95593387 2.51829284 1.33291289 1.19095149 2.69755553
 0.82073186 1.71459068 0.82025497 2.69038921], Cost: 0.0251
Current parameters: [1.10320741 3.08401818 1.29145681 2.17620447 2.81297509 2.30431936
 0.75566729 2.94519268 2.51048479 1.33631254 1.20068663 2.70249803
 0.79970154 1.72399642 0.82887867 2.69276418], Cost: 0.0063
Current parameters: [1.08334234 3.08454443 1.28693911 2.1664444  2.81502623 2.30579574
 0.75035043 2.94123416 2.50218208 1.33436293 1.19214781 2.70510274
 0.79101262 1.72562669 0.82477772 2.67979246], Cost: 0.0147
Current parameters: [1.1047716  3.08123464 1.29092974 2.17763641 2.80962385 2.30758555
 0.7626586  2.94811132 2.51003294 1.33785038 1.19714783 2.69734013
```



```
 0.79203263 1.72470766 0.82186974 2.69424152], Cost: 0.0117
Current parameters: [1.10919313 3.082636   1.30084885 2.18606073 2.82474584 2.30144496
 0.74421343 2.94412566 2.51427822 1.32939507 1.20614078 2.71352004
 0.78445923 1.71995626 0.82965436 2.69616977], Cost: 0.0111
Current parameters: [1.10095089 3.08648425 1.29161323 2.17386822 2.81233631 2.30492898
 0.75543381 2.94969948 2.51414272 1.33362692 1.20444683 2.70050157
 0.79874582 1.73451312 0.83568063 2.69590111], Cost: 0.0075
Current parameters: [1.1038784  3.07961619 1.29394483 2.17599751 2.81225053 2.30399104
 0.75745053 2.94566513 2.51022316 1.33536627 1.19903244 2.70234416
 0.79933463 1.72330577 0.83321739 2.6899752 ], Cost: 0.0066
Current parameters: [1.1033091  3.08536314 1.28889758 2.17699607 2.8152006  2.30406012
 0.75297109 2.94229894 2.50871512 1.337377   1.19134454 2.69910348
 0.79910946 1.72214267 0.83492068 2.70119784], Cost: 0.0073
Current parameters: [1.10485494 3.08192314 1.29277888 2.17525556 2.81216351 2.30325248
 0.75503897 2.94382838 2.50956132 1.33467726 1.19798468 2.70526224
 0.80152002 1.72806142 0.82622389 2.69413638], Cost: 0.0063
Current parameters: [1.11158241 3.08204195 1.29294077 2.16574068 2.81143781 2.29988157
 0.7522367  2.94153171 2.50597795 1.3318917  1.20317494 2.70049037
 0.80309344 1.72494458 0.82644973 2.69499704], Cost: 0.0097
Current parameters: [1.10499898 3.08483679 1.29807798 2.17511923 2.81360303 2.30361884
 0.75617919 2.94226497 2.50736652 1.33782729 1.19786455 2.70419282
 0.80147419 1.72953685 0.82639332 2.69352837], Cost: 0.0064
Current parameters: [1.10684167 3.08100263 1.28843415 2.17963207 2.81191987 2.29775674
 0.75379453 2.94982377 2.50097171 1.34064554 1.19980675 2.70827217
 0.80270745 1.73035763 0.82831096 2.69327608], Cost: 0.0102
Current parameters: [1.10505698 3.07975006 1.2912636  2.17350153 2.81213424 2.30198814
 0.75536578 2.94049847 2.51227371 1.33941844 1.19916848 2.70651404
 0.79962537 1.72912624 0.82686521 2.69454359], Cost: 0.0021
Current parameters: [1.1082998  3.08198235 1.29025203 2.1736007  2.81177057 2.30114613
 0.75386103 2.9378679  2.52342646 1.3451583  1.19617599 2.70850858
 0.80469651 1.72941055 0.8276936  2.68997888], Cost: 0.0024
Current parameters: [1.10918233 3.07914877 1.29363027 2.17696528 2.81364375 2.30155046
 0.75479989 2.94099143 2.51416139 1.33858587 1.20138228 2.70779303
 0.79798465 1.728124   0.82721102 2.69744424], Cost: 0.0032
Current parameters: [1.09931731 3.07881202 1.29158142 2.17091575 2.81685863 2.30077438
 0.74904026 2.93597077 2.51349423 1.33905556 1.19881285 2.71204652
 0.79110936 1.72966888 0.82673398 2.69079741], Cost: 0.0004
Current parameters: [1.09925071 3.07689805 1.29053253 2.17435169 2.81843468 2.30030997
 0.7474703  2.93309901 2.51325806 1.33822514 1.19982144 2.70760863
 0.791947   1.73193018 0.82604756 2.68897907], Cost: 0.0007
Current parameters: [1.0989245  3.08024196 1.28816553 2.17268964 2.81275415 2.30665814
```



```
 0.75301917 2.92623635 2.5113892  1.33824308 1.20238885 2.71766722
 0.79138991 1.73035515 0.83060746 2.69101133], Cost: 0.0017
Current parameters: [1.09865896 3.07946307 1.28993548 2.17011922 2.82073999 2.29735835
 0.75424972 2.93514859 2.51373259 1.33775569 1.1987138  2.71236618
 0.79169677 1.72989166 0.8266192  2.69093637], Cost: 0.0005
Current parameters: [1.10170614 3.08231612 1.29557554 2.17554841 2.82294199 2.30005732
 0.74408419 2.93513476 2.5201163  1.33429946 1.19797909 2.71842777
 0.78854068 1.7274604  0.82708195 2.68825311], Cost: 0.0021
Current parameters: [1.10033147 3.07785607 1.29080514 2.1704245  2.81583843 2.30192561
 0.75122387 2.93580396 2.51557161 1.34072314 1.1969607  2.71087834
 0.78567736 1.73078599 0.82385357 2.69041801], Cost: 0.0009
Current parameters: [1.09848568 3.07965394 1.29223244 2.17172277 2.8145607  2.29829511
 0.74908838 2.93529865 2.51383621 1.33855208 1.19877733 2.71175617
 0.79054679 1.72934993 0.82675838 2.69096193], Cost: 0.0006
Current parameters: [1.09498284 3.07526348 1.29344117 2.17041534 2.8177244  2.30094296
 0.74893203 2.93522657 2.51578505 1.34127804 1.20024933 2.71266212
 0.79246785 1.72852715 0.82541939 2.69346121], Cost: 0.0005
Current parameters: [1.09893032 3.0789658  1.29208557 2.17038704 2.81633642 2.30092282
 0.74950783 2.93724813 2.51442281 1.33841969 1.20118705 2.71236185
 0.79106608 1.73172902 0.82669504 2.68972211], Cost: 0.0005
Current parameters: [1.09931731 3.07881202 1.29158142 2.17091575 2.81685863 2.30077438
 0.74904026 2.93597077 2.51349423 1.33905556 1.19881285 2.71204652
 0.79110936 1.72966888 0.82673398 2.69079741], Cost: 0.0004

   Return from subroutine COBYLA because the MAXFUN limit has been reached.

   NFVALS =  100   F = 4.274875E-04    MAXCV = 0.000000E+00
   X = 1.099317E+00   3.078812E+00   1.291581E+00   2.170916E+00   2.816859E+00
       2.300774E+00   7.490403E-01   2.935971E+00   2.513494E+00   1.339056E+00
       1.198813E+00   2.712047E+00   7.911094E-01   1.729669E+00   8.267340E-01
       2.690797E+00
Simulating for QAOA depth p = 9
Current parameters: [1.86948589 1.0976567  1.64286913 0.42952351 3.04974231 1.26125098
 1.40100715 1.36627399 2.21447844 2.57178494 2.63264442 2.01798838
 0.01241099 2.65233396 2.7977811  1.10145284 0.65645445 2.95006852], Cost: 2.7202
Current parameters: [0.86948589 2.0976567  1.64286913 0.42952351 3.04974231 1.26125098
 1.40100715 1.36627399 2.21447844 2.57178494 2.63264442 2.01798838
 0.01241099 2.65233396 2.7977811  1.10145284 0.65645445 2.95006852], Cost: 1.9386
Current parameters: [0.86948589 1.0976567  2.64286913 0.42952351 3.04974231 1.26125098
 1.40100715 1.36627399 2.21447844 2.57178494 2.63264442 2.01798838
 0.01241099 2.65233396 2.7977811  1.10145284 0.65645445 2.95006852], Cost: 2.0040
```



```
Current parameters: [0.86948589 1.0976567  1.64286913 1.42952351 3.04974231 1.26125098
 1.40100715 1.36627399 2.21447844 2.57178494 2.63264442 2.01798838
 0.01241099 2.65233396 2.7977811  1.10145284 0.65645445 2.95006852], Cost: 1.7573
Current parameters: [0.86948589 1.0976567  1.64286913 1.42952351 4.04974231 1.26125098
 1.40100715 1.36627399 2.21447844 2.57178494 2.63264442 2.01798838
 0.01241099 2.65233396 2.7977811  1.10145284 0.65645445 2.95006852], Cost: 2.2149
Current parameters: [0.86948589 1.0976567  1.64286913 1.42952351 3.04974231 2.26125098
 1.40100715 1.36627399 2.21447844 2.57178494 2.63264442 2.01798838
 0.01241099 2.65233396 2.7977811  1.10145284 0.65645445 2.95006852], Cost: 2.1459
Current parameters: [0.86948589 1.0976567  1.64286913 1.42952351 3.04974231 1.26125098
 2.40100715 1.36627399 2.21447844 2.57178494 2.63264442 2.01798838
 0.01241099 2.65233396 2.7977811  1.10145284 0.65645445 2.95006852], Cost: 2.2417
Current parameters: [0.86948589 1.0976567  1.64286913 1.42952351 3.04974231 1.26125098
 1.40100715 2.36627399 2.21447844 2.57178494 2.63264442 2.01798838
 0.01241099 2.65233396 2.7977811  1.10145284 0.65645445 2.95006852], Cost: 1.9453
Current parameters: [0.86948589 1.0976567  1.64286913 1.42952351 3.04974231 1.26125098
 1.40100715 1.36627399 3.21447844 2.57178494 2.63264442 2.01798838
 0.01241099 2.65233396 2.7977811  1.10145284 0.65645445 2.95006852], Cost: 1.7956
Current parameters: [0.86948589 1.0976567  1.64286913 1.42952351 3.04974231 1.26125098
 1.40100715 1.36627399 2.21447844 3.57178494 2.63264442 2.01798838
 0.01241099 2.65233396 2.7977811  1.10145284 0.65645445 2.95006852], Cost: 1.9903
Current parameters: [0.86948589 1.0976567  1.64286913 1.42952351 3.04974231 1.26125098
 1.40100715 1.36627399 2.21447844 2.57178494 3.63264442 2.01798838
 0.01241099 2.65233396 2.7977811  1.10145284 0.65645445 2.95006852], Cost: 1.7737
Current parameters: [0.86948589 1.0976567  1.64286913 1.42952351 3.04974231 1.26125098
 1.40100715 1.36627399 2.21447844 2.57178494 2.63264442 3.01798838
 0.01241099 2.65233396 2.7977811  1.10145284 0.65645445 2.95006852], Cost: 2.1608
Current parameters: [0.86948589 1.0976567  1.64286913 1.42952351 3.04974231 1.26125098
 1.40100715 1.36627399 2.21447844 2.57178494 2.63264442 2.01798838
 1.01241099 2.65233396 2.7977811  1.10145284 0.65645445 2.95006852], Cost: 1.7243
Current parameters: [0.86948589 1.0976567  1.64286913 1.42952351 3.04974231 1.26125098
 1.40100715 1.36627399 2.21447844 2.57178494 2.63264442 2.01798838
 1.01241099 3.65233396 2.7977811  1.10145284 0.65645445 2.95006852], Cost: 2.2100
Current parameters: [0.86948589 1.0976567  1.64286913 1.42952351 3.04974231 1.26125098
 1.40100715 1.36627399 2.21447844 2.57178494 2.63264442 2.01798838
 1.01241099 2.65233396 3.7977811  1.10145284 0.65645445 2.95006852], Cost: 3.2409
Current parameters: [0.86948589 1.0976567  1.64286913 1.42952351 3.04974231 1.26125098
 1.40100715 1.36627399 2.21447844 2.57178494 2.63264442 2.01798838
 1.01241099 2.65233396 2.7977811  2.10145284 0.65645445 2.95006852], Cost: 0.5917
Current parameters: [0.86948589 1.0976567  1.64286913 1.42952351 3.04974231 1.26125098
 1.40100715 1.36627399 2.21447844 2.57178494 2.63264442 2.01798838
```

```
 1.01241099 2.65233396 2.7977811  2.10145284 1.65645445 2.95006852], Cost: 2.3587
Current parameters: [0.86948589 1.0976567  1.64286913 1.42952351 3.04974231 1.26125098
 1.40100715 1.36627399 2.21447844 2.57178494 2.63264442 2.01798838
 1.01241099 2.65233396 2.7977811  2.10145284 0.65645445 3.95006852], Cost: 2.6247
Current parameters: [0.63675172 1.08473901 1.61156459 1.46760868 2.92103997 1.15194903
 1.26476119 1.31338016 2.20369824 2.50624526 2.62801732 1.90450959
 1.0216742  2.51573637 2.37124791 2.42000561 0.15948717 2.37829283], Cost: 1.9241
Current parameters: [0.82989406 1.09545919 1.63754372 1.43600241 3.02784796 1.24265695
 1.37782951 1.3572759  2.21264456 2.56063559 2.63185728 1.99868381
 1.01398681 2.6290965  3.27351788 2.15564381 0.57191229 2.85280025], Cost: 3.4502
Current parameters: [0.88334058 0.8480409  1.64286913 1.42952351 3.04974231 1.26125098
 1.40100715 1.36627399 2.21447844 2.57178494 2.63264442 2.01798838
 1.01241099 2.65233396 2.7977811  2.10145284 0.65645445 2.95006852], Cost: 0.5836
Current parameters: [1.37108216 0.87450696 1.64079001 1.43205296 3.04119446 1.25399161
 1.39195828 1.36276101 2.21376247 2.56743208 2.63233711 2.01045161
 1.01302621 2.64326174 2.70812161 2.12260973 0.62344804 2.91209366], Cost: 1.5035
Current parameters: [0.88334058 0.8480409  1.3999516  1.42952351 3.10882763 1.26125098
 1.40100715 1.36627399 2.21447844 2.57178494 2.63264442 2.01798838
 1.01241099 2.65233396 2.7977811  2.10145284 0.65645445 2.95006852], Cost: 0.5038
Current parameters: [0.86098789 0.84647511 1.51411489 1.43088155 3.59173412 1.2573535
 1.3961489  1.36438791 2.21409404 2.56944794 2.63247943 2.01394197
 1.01274129 2.64746318 2.74964382 2.11281177 0.63873364 2.9296802 ], Cost: 0.3907
Current parameters: [0.86098789 0.84647511 1.51411489 1.43088155 3.59173412 1.2573535
 1.55614549 1.36438791 2.21409404 2.56944794 2.63247943 1.82184539
 1.01274129 2.64746318 2.74964382 2.11281177 0.63873364 2.9296802 ], Cost: 0.8802
Current parameters: [0.83081458 0.84436148 1.50972387 1.43271473 3.59196797 1.25209239
 1.75755998 1.36184193 2.21357515 2.56629327 2.63225671 2.34945579
 1.01318716 2.64088823 2.68466456 2.12814488 0.61481278 2.90215854], Cost: 0.7026
Current parameters: [0.86098789 0.84647511 1.51411489 1.43088155 3.59173412 1.3859176
 1.3961489  1.36438791 2.21409404 2.35503881 2.63247943 2.01394197
 1.01274129 2.64746318 2.74964382 2.11281177 0.63873364 2.9296802 ], Cost: 1.0023
Current parameters: [0.83962366 0.84497855 1.51100582 1.43217953 3.5918997  1.67031156
 1.37595444 1.36258523 2.21372664 2.84440884 2.63232173 2.02154683
 1.01305699 2.64280778 2.7036352  2.12366839 0.62179646 2.91019347], Cost: 1.0735
Current parameters: [0.86098789 0.84647511 1.51411489 1.63376221 3.59173412 1.2573535
 1.3961489  1.51046815 2.21409404 2.56944794 2.63247943 2.01394197
 1.01274129 2.64746318 2.74964382 2.11281177 0.63873364 2.9296802 ], Cost: 0.6157
Current parameters: [0.85034415 0.84572952 1.51256594 1.13592871 3.59181661 1.24382047
 1.38608794 1.76667134 2.213911   2.57495555 2.63240086 2.01773074
 1.01289858 2.64514384 2.72672215 2.11822058 0.63029547 2.91997183], Cost: 1.5772
Current parameters: [0.86098789 0.84647511 1.51411489 1.43088155 3.59173412 1.2573535
```



```
 1.3961489  1.36438791 1.96486896 2.56944794 2.63247943 2.01394197
 1.01274129 2.66713188 2.74964382 2.11281177 0.63873364 2.9296802 ], Cost: 0.4597
Current parameters: [0.83915172 0.84494549 1.51093714 1.43884058 3.59190336 1.22958982
 1.37550834 1.33824298 2.25567792 2.58074707 2.63231825 2.02171482
 1.01306397 3.14002654 2.70261887 2.12390821 0.62142231 2.909763  ], Cost: 1.2908
Current parameters: [0.86098789 0.84647511 1.51411489 1.43088155 3.59173412 1.2573535
 1.3961489  1.36438791 2.21409404 2.56944794 2.38248761 2.01394197
 1.01274129 2.64746318 2.74964382 2.11281177 0.63873364 2.93170326], Cost: 0.9434
Current parameters: [0.77776527 0.84064541 1.50200378 1.46121523 3.5923791  1.15153981
 1.31748305 1.26474365 2.21708196 2.61251149 2.71823584 2.04356608
 1.01397108 2.55439974 2.57042107 2.15510284 0.57275625 3.32671816], Cost: 1.9344
Current parameters: [0.86098789 0.84647511 1.51411489 1.43088155 3.59173412 1.2573535
 1.3961489  1.36438791 2.21409404 2.56944794 2.63247943 2.01394197
 1.26269787 2.64746318 2.74964382 2.11281177 0.6433927  2.9296802 ], Cost: 1.0523
Current parameters: [0.80834061 0.84278719 1.5064533  1.45007087 3.59214214 1.19041493
 1.34638427 1.30135217 2.21598422 2.59669028 2.68340449 2.03268241
 0.94221062 2.58859052 2.6362661  2.13956543 1.06843743 2.76996075], Cost: 1.7206
Current parameters: [0.82811863 0.84465073 1.50969372 1.43626035 3.57355737 1.24191668
 1.37690676 1.35691766 2.21257154 2.56019171 2.63182594 1.99791525
 1.01404954 2.62817137 2.68940414 2.32394652 0.56854649 2.84892779], Cost: 1.3326
Current parameters: [0.7772244  0.84130062 1.50245013 1.4518419  3.56566306 1.18905899
 1.33411804 1.31031968 2.21318341 2.57471567 2.66646346 2.00356272
 0.97386872 2.57911278 2.584544   1.80004704 0.42343414 2.70305543], Cost: 1.7587
Current parameters: [0.87061833 0.84645149 1.51498743 1.4370126  3.6185773  1.23110876
 1.38797901 1.32935321 2.21769522 2.60282191 2.6705512  2.05100701
 0.96751022 2.6327273  2.97972774 2.09932388 0.62058209 2.93119944], Cost: 0.9308
Current parameters: [0.87005892 0.72180556 1.51410574 1.43089267 3.59169653 1.25732157
 1.39610911 1.36437246 2.21409089 2.56942879 2.63247808 2.01390882
 1.012744   2.64742328 2.74924953 2.11290481 0.63858849 2.9295132 ], Cost: 0.5382
Current parameters: [1.10448453 0.87129705 1.51423847 1.43319293 3.60020178 1.24775976
 1.3925908  1.35210114 2.2152663  2.58050403 2.64548401 2.02599329
 0.99731229 2.64165583 2.7115028  2.10997672 0.62974208 2.92700285], Cost: 0.5745
Current parameters: [0.85910224 0.84636363 1.39291832 1.43135908 3.6195038  1.25598301
 1.39444058 1.3637247  2.21395888 2.56862617 2.63242141 2.01251911
 1.01285744 2.64575044 2.74603767 2.10593384 0.6325024  2.92251097], Cost: 0.3866
Current parameters: [0.82651514 0.85660291 1.41418206 1.44269646 3.60313512 1.21825911
 1.36213536 1.33207653 2.21394985 2.57545534 2.6543203  2.01191857
 0.98759806 2.60943722 2.68525927 1.94614733 0.52165186 2.8084081 ], Cost: 0.4724
Current parameters: [0.84612444 0.8691091  1.41719087 1.35456454 3.61978393 1.24397575
 1.38192359 1.45014858 2.22558856 2.57520985 2.64430104 2.01747823
 1.00099251 2.64285777 2.74013543 2.09603666 0.6347372  2.92968057], Cost: 0.4123
```



```
Current parameters: [0.93660498 0.73010839 1.23270561 1.42887194 3.62926358 1.2599039
 1.42699694 1.45476665 2.14325221 2.56700169 2.62158919 2.01555851
 1.01051519 2.64542602 2.7694007  2.0690076  0.60969935 2.9057489 ], Cost: 0.5782
Current parameters: [0.84644317 0.84565337 1.37861004 1.43994996 3.62176809 1.23495074
 1.38163454 1.346161   2.20936284 2.57837474 2.65002046 2.02027916
 0.99193132 2.62718532 2.7089199  2.08484849 0.61596797 3.02692062], Cost: 0.4561
Current parameters: [0.86441097 0.84312479 1.38977719 1.43289253 3.6197627  1.25767098
 1.34850011 1.36468308 2.21234304 2.56753435 2.63063523 1.97190711
 1.01461937 2.64821133 2.75410504 2.10575352 0.63479945 2.92477322], Cost: 0.5088
Current parameters: [0.85482163 0.8486081  1.38931054 1.43191946 3.62084895 1.24540006
 1.3978758  1.35822945 2.22328746 2.57408024 2.64143841 2.02552328
 1.00187857 2.7667023  2.73454553 2.09363617 0.62740014 2.92498005], Cost: 0.1790
Current parameters: [0.85601881 0.847078   1.38770723 1.43393988 3.62085809 1.19487067
 1.39850051 1.35680379 2.22233423 2.54284132 2.64059645 2.02499391
 1.00234242 2.76487578 2.7356195  2.09415307 0.6457943  2.92061013], Cost: 0.3317
Current parameters: [0.84301203 0.85217559 1.37898782 1.43405027 3.62369184 1.28103208
 1.40080106 1.3456402  2.22284861 2.62184666 2.66131578 2.05043156
 0.97540209 2.77692021 2.7024759  2.06722994 0.71484638 2.90396633], Cost: 0.0670
Current parameters: [0.84247179 0.85662865 1.36917104 1.43200524 3.56262801 1.28007572
 1.39942865 1.3446544  2.22510287 2.62387992 2.66355751 2.05219797
 0.97315111 2.77706838 2.70586533 2.07145768 0.71564983 2.90471296], Cost: 0.0481
Current parameters: [0.8241631  0.85924818 1.35544861 1.42931204 3.56710086 1.28827901
 1.3988669  1.33743267 2.22373473 2.66283063 2.68901164 2.08034876
 0.94169088 2.79520166 2.66153479 2.15121104 0.67928454 2.87275358], Cost: 0.2636
Current parameters: [0.8428554  0.85595937 1.36849824 1.432318   3.56282105 1.25607605
 1.39966016 1.34481469 2.22475394 2.66386968 2.6632097  2.05193859
 1.01473211 2.7770636  2.7060247  2.07140238 0.7163599  2.90467331], Cost: 0.0628
Current parameters: [0.83514206 0.87184226 1.36713438 1.42146937 3.55538379 1.34383927
 1.40383176 1.33883314 2.23083598 2.58175017 2.69287855 2.08958038
 1.04669713 2.80246152 2.69274957 2.0459875  0.71916054 2.91477614], Cost: 0.2383
Current parameters: [0.84137216 0.85588268 1.36827881 1.42938495 3.55858146 1.28419016
 1.39755949 1.34895968 2.22489199 2.61862971 2.61436732 2.05013459
 0.98025957 2.7793438  2.66950568 2.07353766 0.71845819 2.90477328], Cost: 0.1689
Current parameters: [0.83539514 0.87827361 1.3748421  1.42642925 3.56485924 1.29084375
 1.40670646 1.32656944 2.23383185 2.66295594 2.62097873 2.09515175
 0.93765877 2.79525254 2.78760668 2.04223715 0.71050482 2.91606036], Cost: 0.0622
Current parameters: [0.84650553 0.84940696 1.36190157 1.38507061 3.56441669 1.27984991
 1.41222947 1.31059222 2.22135012 2.6239927  2.66374596 2.03673047
 0.97293043 2.77717433 2.70506186 2.07100886 0.71513442 2.90423319], Cost: 0.0440
Current parameters: [0.83482336 0.87264652 1.37343898 1.41014443 3.55766391 1.29420082
 1.49629806 1.3282918  2.23187821 2.64403596 2.68001681 1.9666489
```



```
 0.95845169 2.7936628  2.7093868  2.05363538 0.71634399 2.91210982], Cost: 0.1510
Current parameters: [0.84700149 0.86453688 1.37650174 1.38502466 3.56112353 1.27996529
 1.41242017 1.3106054  2.16280193 2.62415568 2.66382567 2.03697308
 0.97283003 2.78197545 2.70533078 2.07181033 0.71585226 2.90510186], Cost: 0.0573
Current parameters: [0.96019147 0.88327565 1.38351895 1.38621042 3.56247685 1.27925779
 1.41354704 1.30473378 2.23714134 2.63481258 2.67438172 2.04768275
 0.96159253 2.77805741 2.68838611 2.06603853 0.71208084 2.9061559 ], Cost: 0.0645
Current parameters: [0.86142425 0.83541546 1.34194765 1.38188245 3.56897887 1.28870309
 1.42265832 1.32708245 2.21449265 2.62232591 2.65779671 2.03719281
 0.97835001 2.78533975 2.72106354 2.10347828 0.73798451 2.92843682], Cost: 0.0808
Current parameters: [0.87246279 0.79188019 1.30024925 1.38508227 3.5631856  1.28057144
 1.42390153 1.34292386 2.19112924 2.63562342 2.67295114 2.04781256
 0.95848586 2.77836453 2.70298913 2.0126398  0.68267842 2.87728038], Cost: 0.1038
Current parameters: [0.84931329 0.80935996 1.40105059 1.39342602 3.54560259 1.28305095
 1.41106695 1.31524104 2.22213806 2.6206514  2.67490493 2.03941602
 0.96280025 2.77843289 2.70842187 2.0719669  0.71961589 2.90922818], Cost: 0.0693
Current parameters: [0.84404792 0.82186761 1.39202848 1.33574566 3.61484094 1.28398635
 1.43320669 1.26961717 2.22356543 2.67566732 2.62309773 2.05060533
 1.01183877 2.79895775 2.6924128  2.06126541 0.70634316 2.90340358], Cost: 0.1833
Current parameters: [0.84942468 0.84992442 1.35750756 1.38495254 3.56656029 1.29118616
 1.36781667 1.30602137 2.22251246 2.63502709 2.66850193 2.00229274
 0.97005759 2.79175167 2.71659442 2.06487784 0.72030548 2.90286281], Cost: 0.0452
Current parameters: [0.84844068 0.8440412  1.3550073  1.40372071 3.56454658 1.28083679
 1.41404448 1.28892283 2.21824167 2.62329781 2.66249284 2.036243
 0.97389328 2.7762483  2.70323813 2.07149907 0.71318768 2.9115631 ], Cost: 0.0607
Current parameters: [0.83743538 0.85454413 1.35925224 1.37407542 3.56617334 1.27094912
 1.40308876 1.31685047 2.22227173 2.63385499 2.67641954 2.04227927
 0.95933054 2.77200639 2.6878483  2.05721306 0.70879642 2.95359574], Cost: 0.0717
Current parameters: [0.84705392 0.84937311 1.36309819 1.38438675 3.56413602 1.29624357
 1.41134365 1.3121291  2.21968954 2.63164761 2.66211852 2.03419085
 0.97506977 2.75268259 2.70713578 2.07340593 0.71101794 2.90495129], Cost: 0.0603
Current parameters: [0.84423286 0.85661977 1.35926009 1.37883947 3.56859678 1.24596983
 1.40526685 1.30294407 2.22343031 2.61349839 2.6753667  2.03781713
 0.966455   2.7529038  2.72143175 2.06162049 0.74781655 2.88923172], Cost: 0.0588
Current parameters: [0.84975755 0.8463138  1.36407067 1.38441878 3.5665567  1.2635734
 1.41153696 1.31257612 2.22045986 2.62258861 2.65351953 2.02648031
 0.96831409 2.77191922 2.71194892 2.07966766 0.6981672  2.90662902], Cost: 0.0254
Current parameters: [0.85009611 0.85701198 1.36675851 1.37831053 3.5621562  1.2651943
 1.40533767 1.31361043 2.22656911 2.58843775 2.6701045  2.03075835
 0.99745467 2.77657358 2.73593538 2.07436197 0.67191665 2.91165503], Cost: 0.0330
Current parameters: [0.85170072 0.84020123 1.36264215 1.38184894 3.57069413 1.2593084
```

```
 1.41103747 1.3139235  2.21786108 2.61654953 2.66396003 2.01505085
 0.97970252 2.76774431 2.69204256 2.08694749 0.69900471 2.90355379], Cost: 0.0356
Current parameters: [0.83960606 0.85595065 1.35802119 1.38312103 3.56563282 1.25969051
 1.40381055 1.30905989 2.22465546 2.63459018 2.6792034  2.04187202
 0.94512909 2.78054904 2.7145832  2.11301479 0.67817375 2.88675132], Cost: 0.0530
Current parameters: [0.85340291 0.83547463 1.34827279 1.38065105 3.56541799 1.26444698
 1.415208   1.32177643 2.21663306 2.62283928 2.65716345 2.03207451
 0.96581034 2.77738034 2.70982548 2.06463433 0.69200135 2.89576925], Cost: 0.0288
Current parameters: [0.8665057  0.82907722 1.35924241 1.3995048  3.57264307 1.2473589
 1.39920766 1.30874014 2.2002177  2.63208501 2.65262831 2.01043469
 0.95974672 2.74407038 2.73548701 2.0776188  0.68226105 2.92051009], Cost: 0.0545
Current parameters: [0.84621948 0.85457349 1.36637542 1.3890151  3.56508723 1.26341919
 1.43036111 1.31707784 2.22471399 2.62577347 2.65958393 2.00828595
 0.96403577 2.77654741 2.71694852 2.07419646 0.69719389 2.90566339], Cost: 0.0241
Current parameters: [0.83232657 0.84971568 1.3640612  1.3889804  3.56583491 1.2634041
 1.43009633 1.31725817 2.22229847 2.62508934 2.65780516 2.0066934
 0.96565053 2.77585363 2.71926995 2.07508552 0.69739075 2.90611127], Cost: 0.0217
Current parameters: [0.83204124 0.84634067 1.37224255 1.3742824  3.57900641 1.25923825
 1.43537586 1.30773309 2.22508685 2.6365697  2.65075202 2.0068681
 0.97420009 2.7833032  2.72287098 2.07114515 0.69380732 2.90245014], Cost: 0.0208
Current parameters: [0.83249979 0.84618344 1.36949325 1.36975564 3.56641802 1.25662427
 1.43607176 1.30413359 2.22472709 2.64100491 2.65036282 2.00538127
 0.97776263 2.78271721 2.72325912 2.07228665 0.69392482 2.90312025], Cost: 0.0242
Current parameters: [0.82766186 0.85276363 1.3755188  1.37435565 3.57668607 1.25533597
 1.43201665 1.31306287 2.23034787 2.62703835 2.62697305 2.00192328
 0.96929853 2.78749558 2.71752752 2.06814253 0.68988665 2.89595935], Cost: 0.0208
Current parameters: [0.83367249 0.84406641 1.37012397 1.36643185 3.57950268 1.2636569
 1.43780908 1.30240874 2.2235905  2.62984191 2.65043087 2.00386073
 0.96714473 2.78292549 2.72394012 2.07315067 0.69466731 2.9043666 ], Cost: 0.0277
Current parameters: [0.83669144 0.84438558 1.36384641 1.37428656 3.5831938  1.2581822
 1.44010459 1.32239099 2.22685629 2.63752761 2.64347505 2.00816358
 0.98169714 2.79339199 2.73170155 2.08458613 0.70440804 2.90853878], Cost: 0.0282
Current parameters: [0.83007241 0.85467892 1.36443704 1.37113145 3.58283618 1.25708891
 1.43432298 1.30764005 2.2248739  2.63876253 2.65061395 2.00733962
 0.97389615 2.78189995 2.71946074 2.06881933 0.69181069 2.90961235], Cost: 0.0215
Current parameters: [0.82689192 0.83826708 1.38597973 1.37026266 3.57496101 1.25325058
 1.4333572  1.3176571  2.23158804 2.63773476 2.65566047 2.01170896
 0.96733061 2.78982788 2.71747331 2.0637482  0.69322653 2.91857835], Cost: 0.0174
Current parameters: [0.82666438 0.83559863 1.38231726 1.37027246 3.57618937 1.24969148
 1.4325192  1.31593634 2.24422358 2.63672002 2.65675909 2.01151679
 0.96664701 2.78480224 2.71946266 2.06270239 0.69646007 2.91698043], Cost: 0.0165
```



```
Current parameters: [0.82320741 0.84396108 1.38737591 1.36503418 3.57716865 1.23391486
 1.4321034  1.31790628 2.23490096 2.63107065 2.6615672  2.01633944
 0.96476069 2.78226817 2.72460877 2.05573837 0.71311375 2.90921964], Cost: 0.0176
Current parameters: [0.82722859 0.83602331 1.38139123 1.36922354 3.57666494 1.25214839
 1.42164474 1.31614128 2.24452501 2.63916071 2.65901339 2.00405105
 0.96594488 2.79012573 2.72198406 2.06025157 0.69933014 2.91607214], Cost: 0.0209
Current parameters: [0.83118631 0.82760776 1.38518989 1.37957111 3.58081947 1.23633369
 1.43088541 1.31782595 2.23707398 2.64151621 2.65075485 2.00590656
 0.96320652 2.77127638 2.72941632 2.05905566 0.68631397 2.92328016], Cost: 0.0112
Current parameters: [0.83019045 0.82603677 1.38412201 1.38050087 3.58184943 1.23475117
 1.43149511 1.31764265 2.23611127 2.65028358 2.64934612 2.00604653
 0.95419389 2.77124681 2.72418723 2.0638724  0.68990489 2.92008008], Cost: 0.0280
Current parameters: [0.82549023 0.83461952 1.38954357 1.38016144 3.58082953 1.230148
 1.42787181 1.32015587 2.24233735 2.63298123 2.65995172 2.01295047
 0.96769603 2.77440839 2.74103407 2.06398094 0.66663059 2.91988945], Cost: 0.0113
Current parameters: [0.83168948 0.82572109 1.38235045 1.38636607 3.58097387 1.23078251
 1.43183541 1.30967209 2.23656835 2.63854385 2.65085046 2.00659555
 0.96276481 2.77963884 2.72806663 2.05841168 0.6870228  2.92561086], Cost: 0.0085
Current parameters: [0.82900438 0.82628584 1.38790746 1.39722592 3.58132197 1.23797257
 1.43539369 1.30577501 2.23851382 2.63195796 2.64661873 2.0113269
 0.97773147 2.77489168 2.73239423 2.04060059 0.68878689 2.93382738], Cost: 0.0131
Current parameters: [0.83026268 0.82989732 1.38961902 1.38774742 3.58216772 1.22969428
 1.43001502 1.30552048 2.23797315 2.63747084 2.65077131 2.00213604
 0.96575177 2.77638859 2.72592901 2.06669336 0.69008009 2.93027671], Cost: 0.0123
Current parameters: [0.83450505 0.81614822 1.37873911 1.38338526 3.58521423 1.22204077
 1.43359356 1.31668056 2.23388582 2.62923175 2.65775344 1.99695654
 0.97581465 2.77830011 2.71231548 2.05569479 0.68189695 2.92112566], Cost: 0.0020
Current parameters: [0.83630831 0.82137269 1.38043947 1.38561744 3.58431425 1.22196999
 1.44276644 1.31881259 2.23655479 2.63094546 2.66113701 1.9885582
 0.97333656 2.78071445 2.71414666 2.05282326 0.68137632 2.92054524], Cost: 0.0038
Current parameters: [0.86230106 0.82278276 1.38372605 1.38236797 3.58459591 1.21963354
 1.42923527 1.31649365 2.23772482 2.62774873 2.65767773 2.00478243
 0.97560803 2.77869521 2.70781895 2.05324365 0.67985439 2.92130173], Cost: 0.0017
Current parameters: [0.86244705 0.82093516 1.3873078  1.37496332 3.59133396 1.2175727
 1.43215354 1.31240328 2.23914739 2.6335185  2.6539628  2.00493407
 0.98021638 2.78287932 2.71011169 2.05201952 0.67865314 2.91986197], Cost: 0.0031
Current parameters: [0.86776186 0.81909297 1.37514251 1.383477   3.5864294  1.21621237
 1.43346958 1.33253553 2.23830524 2.62317917 2.65163343 2.00621249
 0.98387266 2.78657694 2.71866455 2.06382653 0.68787805 2.93044819], Cost: 0.0018
Current parameters: [0.86453858 0.81945209 1.38226253 1.38239594 3.58714089 1.22211152
 1.43097808 1.31391806 2.2350412  2.63169679 2.6696432  2.00726376
```



```
 0.97730737 2.77663265 2.71051796 2.05473651 0.68185678 2.9245921 ], Cost: 0.0065
Current parameters: [0.86578689 0.81642447 1.3817449  1.36860411 3.58535061 1.22273863
 1.43312227 1.30924018 2.2345229  2.6076494  2.65464853 1.99936608
 0.96502927 2.77667031 2.71279684 2.05135045 0.67642557 2.92792005], Cost: 0.0062
Current parameters: [0.86230106 0.82278276 1.38372605 1.38236797 3.58459591 1.21963354
 1.42923527 1.31649365 2.23772482 2.62774873 2.65767773 2.00478243
 0.97560803 2.77869521 2.70781895 2.05324365 0.67985439 2.92130173], Cost: 0.0017

   Return from subroutine COBYLA because the MAXFUN limit has been reached.

   NFVALS =  100   F = 1.697876E-03    MAXCV = 0.000000E+00
   X = 8.623011E-01   8.227828E-01   1.383726E+00   1.382368E+00   3.584596E+00
       1.219634E+00   1.429235E+00   1.316494E+00   2.237725E+00   2.627749E+00
       2.657678E+00   2.004782E+00   9.756080E-01   2.778695E+00   2.707819E+00
       2.053244E+00   6.798544E-01   2.921302E+00
Simulating for QAOA depth p = 10
Current parameters: [3.46092929 1.40262883 1.5131475  0.5864987  0.52540361 2.6271266
 2.00683254 1.92013389 2.05980407 2.73733896 1.76107992 0.93731721
 0.12361653 0.76940301 0.96736754 1.99947122 2.5726242  0.51896401
 2.41444065 1.37212016], Cost: 1.8014
Current parameters: [3.46092929 2.40262883 1.5131475  0.5864987  0.52540361 2.6271266
 2.00683254 1.92013389 2.05980407 2.73733896 1.76107992 0.93731721
 0.12361653 0.76940301 0.96736754 1.99947122 2.5726242  0.51896401
 2.41444065 1.37212016], Cost: 2.4761
Current parameters: [3.46092929 1.40262883 2.5131475  0.5864987  0.52540361 2.6271266
 2.00683254 1.92013389 2.05980407 2.73733896 1.76107992 0.93731721
 0.12361653 0.76940301 0.96736754 1.99947122 2.5726242  0.51896401
 2.41444065 1.37212016], Cost: 2.1514
Current parameters: [3.46092929 1.40262883 1.5131475  1.5864987  0.52540361 2.6271266
 2.00683254 1.92013389 2.05980407 2.73733896 1.76107992 0.93731721
 0.12361653 0.76940301 0.96736754 1.99947122 2.5726242  0.51896401
 2.41444065 1.37212016], Cost: 1.9986
Current parameters: [3.46092929 1.40262883 1.5131475  0.5864987  1.52540361 2.6271266
 2.00683254 1.92013389 2.05980407 2.73733896 1.76107992 0.93731721
 0.12361653 0.76940301 0.96736754 1.99947122 2.5726242  0.51896401
 2.41444065 1.37212016], Cost: 1.5133
Current parameters: [3.46092929 1.40262883 1.5131475  0.5864987  1.52540361 3.6271266
 2.00683254 1.92013389 2.05980407 2.73733896 1.76107992 0.93731721
 0.12361653 0.76940301 0.96736754 1.99947122 2.5726242  0.51896401
 2.41444065 1.37212016], Cost: 2.4065
Current parameters: [3.46092929 1.40262883 1.5131475  0.5864987  1.52540361 2.6271266
```



```
 3.00683254 1.92013389 2.05980407 2.73733896 1.76107992 0.93731721
 0.12361653 0.76940301 0.96736754 1.99947122 2.5726242  0.51896401
 2.41444065 1.37212016], Cost: 0.6754
Current parameters: [3.46092929 1.40262883 1.5131475  0.5864987  1.52540361 2.6271266
 3.00683254 2.92013389 2.05980407 2.73733896 1.76107992 0.93731721
 0.12361653 0.76940301 0.96736754 1.99947122 2.5726242  0.51896401
 2.41444065 1.37212016], Cost: 2.5422
Current parameters: [3.46092929 1.40262883 1.5131475  0.5864987  1.52540361 2.6271266
 3.00683254 1.92013389 3.05980407 2.73733896 1.76107992 0.93731721
 0.12361653 0.76940301 0.96736754 1.99947122 2.5726242  0.51896401
 2.41444065 1.37212016], Cost: 2.7544
Current parameters: [3.46092929 1.40262883 1.5131475  0.5864987  1.52540361 2.6271266
 3.00683254 1.92013389 2.05980407 3.73733896 1.76107992 0.93731721
 0.12361653 0.76940301 0.96736754 1.99947122 2.5726242  0.51896401
 2.41444065 1.37212016], Cost: 0.7962
Current parameters: [3.46092929 1.40262883 1.5131475  0.5864987  1.52540361 2.6271266
 3.00683254 1.92013389 2.05980407 2.73733896 2.76107992 0.93731721
 0.12361653 0.76940301 0.96736754 1.99947122 2.5726242  0.51896401
 2.41444065 1.37212016], Cost: 1.7668
Current parameters: [3.46092929 1.40262883 1.5131475  0.5864987  1.52540361 2.6271266
 3.00683254 1.92013389 2.05980407 2.73733896 1.76107992 1.93731721
 0.12361653 0.76940301 0.96736754 1.99947122 2.5726242  0.51896401
 2.41444065 1.37212016], Cost: 2.4126
Current parameters: [3.46092929 1.40262883 1.5131475  0.5864987  1.52540361 2.6271266
 3.00683254 1.92013389 2.05980407 2.73733896 1.76107992 0.93731721
 1.12361653 0.76940301 0.96736754 1.99947122 2.5726242  0.51896401
 2.41444065 1.37212016], Cost: 2.1379
Current parameters: [3.46092929 1.40262883 1.5131475  0.5864987  1.52540361 2.6271266
 3.00683254 1.92013389 2.05980407 2.73733896 1.76107992 0.93731721
 0.12361653 1.76940301 0.96736754 1.99947122 2.5726242  0.51896401
 2.41444065 1.37212016], Cost: 3.0836
Current parameters: [3.46092929 1.40262883 1.5131475  0.5864987  1.52540361 2.6271266
 3.00683254 1.92013389 2.05980407 2.73733896 1.76107992 0.93731721
 0.12361653 0.76940301 1.96736754 1.99947122 2.5726242  0.51896401
 2.41444065 1.37212016], Cost: 1.6601
Current parameters: [3.46092929 1.40262883 1.5131475  0.5864987  1.52540361 2.6271266
 3.00683254 1.92013389 2.05980407 2.73733896 1.76107992 0.93731721
 0.12361653 0.76940301 0.96736754 2.99947122 2.5726242  0.51896401
 2.41444065 1.37212016], Cost: 0.1409
Current parameters: [3.46092929 1.40262883 1.5131475  0.5864987  1.52540361 2.6271266
 3.00683254 1.92013389 2.05980407 2.73733896 1.76107992 0.93731721
```

```
 0.12361653 0.76940301 0.96736754 2.99947122 3.5726242  0.51896401
 2.41444065 1.37212016], Cost: 0.1872
Current parameters: [3.46092929 1.40262883 1.5131475  0.5864987  1.52540361 2.6271266
 3.00683254 1.92013389 2.05980407 2.73733896 1.76107992 0.93731721
 0.12361653 0.76940301 0.96736754 2.99947122 2.5726242  1.51896401
 2.41444065 1.37212016], Cost: 2.9844
Current parameters: [3.46092929 1.40262883 1.5131475  0.5864987  1.52540361 2.6271266
 3.00683254 1.92013389 2.05980407 2.73733896 1.76107992 0.93731721
 0.12361653 0.76940301 0.96736754 2.99947122 2.5726242  0.51896401
 3.41444065 1.37212016], Cost: 1.2399
Current parameters: [3.46092929 1.40262883 1.5131475  0.5864987  1.52540361 2.6271266
 3.00683254 1.92013389 2.05980407 2.73733896 1.76107992 0.93731721
 0.12361653 0.76940301 0.96736754 2.99947122 2.5726242  0.51896401
 2.41444065 2.37212016], Cost: 1.1093
Current parameters: [ 3.5605189   1.28690988  1.45311232  0.55266631  1.57481224  2.47393566
  3.15053831  1.599964    1.70322578  2.71662175  1.57389955  0.63936519
 -0.12722028  0.35637199  0.79847867  3.09115137  2.56468335  0.03125734
  2.22594345  1.20601857], Cost: 2.0484
Current parameters: [3.47356571 1.38794583 1.50552993 0.58220588 1.53167282 2.60768896
 3.02506665 2.40546941 2.01455963 2.73471026 1.73732953 0.89951157
 0.0917891  0.71699557 0.94593808 3.01110405 2.57161663 0.45708135
 2.39052318 1.35104436], Cost: 0.3750
Current parameters: [3.27144072 1.2395519  1.5131475  0.5864987  1.52540361 2.6271266
 3.00683254 1.92013389 2.05980407 2.73733896 1.76107992 0.93731721
 0.12361653 0.76940301 0.96736754 2.99947122 2.5726242  0.51896401
 2.41444065 1.37212016], Cost: 0.4428
Current parameters: [3.14707847 1.7873557  1.50935807 0.5843632  1.52852229 2.61745716
 3.01590327 1.9202976  2.03729679 2.73603129 1.74926506 0.91851043
 0.10778367 0.74333242 0.95670725 3.00525808 2.57212297 0.48817989
 2.40254268 1.3616358 ], Cost: 0.2928
Current parameters: [3.46092929 1.40262883 1.63588531 0.36870194 1.52540361 2.6271266
 3.00683254 1.92013389 2.05980407 2.73733896 1.76107992 0.93731721
 0.12361653 0.76940301 0.96736754 2.99947122 2.5726242  0.51896401
 2.41444065 1.37212016], Cost: 0.2104
Current parameters: [3.46573972 1.40615764 1.94741153 0.83279607 1.52682225 2.62272812
 3.01095868 1.92020836 2.04956584 2.73674412 1.75570551 0.92876229
 0.1164144  0.75754389 0.96251833 3.00210357 2.5723962  0.50496078
 2.40902844 1.36735098], Cost: 0.7074
Current parameters: [3.46092929 1.40262883 1.5131475  0.5864987  1.28747291 2.55038687
 3.00683254 1.92013389 2.05980407 2.73733896 1.76107992 0.93731721
 0.12361653 0.76940301 0.96736754 2.99947122 2.5726242  0.51896401
```



```
 2.41444065 1.37212016], Cost: 0.4243
Current parameters: [3.47221275 1.41090608 1.50114987 0.58342243 1.38572909 3.10285308
 3.0165109  1.92030857 2.03578906 2.73594369 1.7484736  0.9172506
 0.10672306 0.741586   0.95599313 3.00564573 2.5720894  0.48611771
 2.40174565 1.36093347], Cost: 1.0159
Current parameters: [3.46092929 1.40262883 1.5131475  0.5864987  1.52540361 2.6271266
 2.77495508 1.92013389 1.96635438 2.73733896 1.76107992 0.93731721
 0.12361653 0.76940301 0.96736754 2.99947122 2.5726242  0.51896401
 2.41444065 1.37212016], Cost: 0.1312
Current parameters: [3.48922007 1.42338221 1.48306609 0.57878563 1.57045619 2.59442003
 2.59666103 1.92057186 2.40576091 2.73384062 1.72947237 0.88700461
 0.08125987 0.699658   0.93884873 3.01495246 2.5712833  0.43660917
 2.38261075 1.34407201], Cost: 0.8486
Current parameters: [3.46092929 1.40262883 1.5131475  0.5864987  1.52540361 2.6271266
 2.77495508 1.92013389 1.96635438 2.48885631 1.78858209 0.93731721
 0.12361653 0.76940301 0.96736754 2.99947122 2.5726242  0.51896401
 2.41444065 1.37212016], Cost: 0.2568
Current parameters: [3.47498921 1.41294283 1.49819767 0.58266547 1.5477938  2.61087212
 2.77965092 1.92035155 1.95321363 2.79884154 2.25104409 0.91231291
 0.10256616 0.73474121 0.95319429 3.00716507 2.5719578  0.47803539
 2.39862186 1.35818082], Cost: 0.8880
Current parameters: [ 3.46092929  1.40262883  1.5131475   0.5864987   1.52540361  2.6271266
  2.77495508  1.92013389  1.96635438  2.73733896  1.76107992  1.0983248
 -0.06763345  0.76940301  0.96736754  2.99947122  2.5726242   0.51896401
  2.41444065  1.37212016], Cost: 1.0923
Current parameters: [3.49098621 1.42467781 1.48118816 0.57830411 1.57326874 2.59237822
 2.78499372 1.9205992  1.93826244 2.74840946 1.7206231  1.21254185
 0.50995432 0.69530396 0.93706835 3.01591892 2.57119959 0.43146792
 2.38062367 1.34232102], Cost: 1.2919
Current parameters: [3.46092929 1.40262883 1.5131475  0.5864987  1.52540361 2.6271266
 2.77495508 1.92013389 1.96635438 2.73733896 1.76107992 0.93731721
 0.12361653 0.86402361 0.73596542 2.99947122 2.5726242  0.51896401
 2.41444065 1.37212016], Cost: 0.4051
Current parameters: [3.49512035 1.4277105  1.47679236 0.577177   1.57985229 2.5875988
 2.78637448 1.9206632  1.93439857 2.74993214 1.71505851 0.7805969
 0.16758193 1.16674406 1.17126882 3.0181812  2.57100364 0.41943337
 2.37597235 1.33822233], Cost: 1.8655
Current parameters: [3.55918764 1.42977925 1.42872189 0.64473753 1.62730948 2.61148812
 2.86153748 1.88187588 1.99813549 2.73718338 1.75967423 0.93507966
 0.12173281 0.76630124 0.96609922 3.02251766 2.54957776 0.65623299
 2.39139421 1.34907372], Cost: 0.2620
```



```
Current parameters: [3.34664287 1.39079018 1.60253456 0.48140135 1.4308506  2.6022769
 2.65062638 1.98211035 1.87507608 2.7534892  1.70522307 0.74301822
 0.18214859 0.59862641 0.94984638 2.98616387 2.6075097  0.84297041
 2.40279994 1.36625041], Cost: 0.6738
Current parameters: [3.47602588 1.4094261  1.4990043  0.59047732 1.5442498  2.61899896
 2.78511553 1.91658069 1.96401276 2.73943924 1.75321658 0.91078227
 0.13082141 0.74573045 0.96464584 3.00480763 2.57015804 0.50651054
 2.65903325 1.36423287], Cost: 0.7145
Current parameters: [3.46987053 1.40509945 1.50546499 0.59179828 1.53467677 2.62570354
 2.78283385 1.91665251 1.96924638 2.7373248  1.760952   0.9371136
 0.12344512 0.76912076 0.96725213 2.94727986 2.6846627  0.51863073
 2.41431184 1.370023  ], Cost: 0.1797
Current parameters: [3.4678013  1.40556193 1.50678134 0.58861455 1.53378686 2.62377798
 2.77977286 1.91837388 1.96573155 2.73816474 1.75796951 0.92684472
 0.12643659 0.76004979 0.96628359 2.77507653 2.46470153 0.51402441
 2.40822661 1.36879641], Cost: 0.1433
Current parameters: [3.45932408 1.4091632  1.5132828  0.59007104 1.52636352 2.63407273
 2.77081496 1.9344672  1.98477539 2.73834308 1.77015211 0.95175825
 0.13577401 0.78942166 0.97555319 2.99502769 2.57300908 0.54260202
 2.42357667 1.48781947], Cost: 0.3960
Current parameters: [3.49653477 1.39115905 1.48516848 0.58255951 1.55802119 2.58715565
 2.8110905  1.85964066 1.89587841 2.73716495 1.71433469 0.83931476
 0.09083618 0.65561585 0.93255085 3.01990088 2.56804533 0.41069056
 2.35419768 1.44207811], Cost: 0.8579
Current parameters: [3.39378359 1.50349828 1.49866876 0.59544764 1.53872673 2.63437392
 2.79078367 1.91415033 1.96695197 2.73704999 1.75846904 0.93316124
 0.12011775 0.76364186 0.9650118  3.00075001 2.57251344 0.51216126
 2.41181141 1.3698033 ], Cost: 0.1547
Current parameters: [3.49408056 1.40288029 1.4902729  0.59571858 1.47504057 2.86410679
 2.79062144 1.9117499  1.97352246 2.7400526  1.75599948 0.91377111
 0.13637449 0.75122139 0.96760633 3.00034386 2.57070787 0.51458743
 2.40069598 1.35123878], Cost: 0.3254
Current parameters: [3.44857029 1.3981285  1.53972687 0.58735503 1.51224385 2.62784749
 2.7593248  1.80311814 1.97222409 2.73794719 1.76657525 0.94606464
 0.13098073 0.78152899 0.97232587 2.99677962 2.57285733 0.53328236
 2.41997465 1.37699666], Cost: 0.1934
Current parameters: [3.48623409 1.42414585 1.7061509  0.72932719 1.55759959 2.63459423
 2.80213001 1.95261552 1.97508818 2.73879671 1.75686801 0.9215338
 0.12946319 0.75601497 0.96639957 3.00058529 2.57144666 0.5131935
 2.40513171 1.35876687], Cost: 0.2842
Current parameters: [3.46545207 1.40757766 1.50087301 0.57996559 1.53744815 2.60982851
```



```
 2.6851765  1.92692641 2.18426028 2.73906933 1.74567716 0.89658883
 0.12349264 0.72810543 0.9585271  3.00532116 2.5701873  0.48812278
 2.3898817  1.34128316], Cost: 0.1876
Current parameters: [3.46117061 1.40386441 1.51143601 0.58288664 1.52748903 2.62302818
 2.77241312 1.92361663 1.96348988 2.75253977 1.88224029 0.9227655
 0.12917539 0.7571345  0.96654535 3.0004654  2.57153189 0.51382045
 2.40586411 1.35976884], Cost: 0.1405
Current parameters: [3.44999107 1.39988881 1.52147915 0.57962581 1.51480242 2.62716368
 2.76599658 1.92472083 1.96162213 2.73715821 1.75944679 0.90019561
 0.09236498 0.76952283 0.96741654 2.99748534 2.57530814 0.5514383
 2.41561945 1.37348565], Cost: 0.2235
Current parameters: [3.44060917 1.40123875 1.52380675 0.56188625 1.51154313 2.61428469
 2.74970263 1.93993351 1.94820918 2.74171847 1.74975221 0.96503858
 0.20297965 0.7310009  0.96485973 2.9987787  2.57436168 0.56534582
 2.38971543 1.33588222], Cost: 0.0428
Current parameters: [3.48160966 1.43644981 1.53185478 0.54633677 1.49517668 2.60802532
 2.73369107 1.9399426  1.9411283  2.74163465 1.74899485 0.96383513
 0.2019665  0.73807593 0.96775271 2.99910011 2.57433384 0.56363603
 2.3890546  1.3352999 ], Cost: 0.0673
Current parameters: [3.42621182 1.39073095 1.51471246 0.55791483 1.52634673 2.60555218
 2.74828409 1.950512   1.94412342 2.74725809 1.74431107 0.94186263
 0.24455182 0.8160085  1.01400552 3.0014999  2.56994606 0.5450107
 2.36255807 1.29541489], Cost: 0.1234
Current parameters: [3.44060917 1.40123875 1.53605276 0.54015585 1.55902161 2.62959791
 2.72656734 1.93993351 1.93888536 2.74171847 1.74975221 0.97351435
 0.19291185 0.7310009  0.96485973 2.9987787  2.57436168 0.56534582
 2.38971543 1.33588222], Cost: 0.0702
Current parameters: [3.42819351 1.39415935 1.51787851 0.56668563 1.48315022 2.60332936
 2.7498006  1.95339166 1.95370092 2.75017255 1.75478013 1.05751748
 0.14996935 0.72797956 0.98212288 2.99717481 2.56912578 0.5663437
 2.36528659 1.293092  ], Cost: 0.2696
Current parameters: [3.44126727 1.40106981 1.50165155 0.60023712 1.51215862 2.61360062
 2.70949065 1.93890462 1.9305771  2.74163208 1.74897164 0.96336758
 0.20244383 0.72914288 0.96409998 2.99920844 2.57438354 0.56357504
 2.38876356 1.33742989], Cost: 0.0511
Current parameters: [3.44950353 1.38874499 1.51084034 0.54811313 1.52203844 2.58739339
 2.77111645 1.92010756 1.91622862 2.74733787 1.72955716 0.93048349
 0.21919123 0.6680219  0.95750541 3.01105495 2.56985974 0.50918017
 2.3346948  1.34328148], Cost: 0.1554
Current parameters: [3.44231738 1.40198584 1.52221625 0.56237813 1.51364886 2.61341306
 2.75087866 1.93951195 1.94800483 2.74163086 1.74896064 0.96225581
```

```
 0.20373202 0.7072921  1.01647985 2.99936747 2.57407361 0.56421807
 2.41531003 1.33502621], Cost: 0.0688
Current parameters: [3.440642   1.39598945 1.51302883 0.5560314  1.51643344 2.59956211
 2.75469148 1.94452402 1.94188264 2.74974572 1.74532588 0.95962009
 0.23892521 0.72958292 0.90722264 3.00322348 2.56746499 0.54736485
 2.48198778 1.29733345], Cost: 0.0692
Current parameters: [3.44066191 1.39710903 1.48206833 0.53507934 1.49769057 2.64853469
 2.7467728  1.93263048 1.94763179 2.74172908 1.74984807 0.96463696
 0.20376801 0.73094408 0.96510179 2.99864507 2.57436828 0.56593579
 2.38949102 1.33578761], Cost: 0.0420
Current parameters: [3.45906021 1.40086877 1.52982772 0.58025665 1.48682084 2.74126527
 2.76257702 1.94271236 1.95176837 2.74596577 1.7464199  0.95359312
 0.22957306 0.71536525 0.95775656 3.00091676 2.57170937 0.55747977
 2.38155088 1.31121579], Cost: 0.0301
Current parameters: [3.47607178 1.40556935 1.51521103 0.5903396  1.50446391 2.73855777
 2.77756712 1.93608871 1.95727067 2.79075235 1.74121656 0.95320573
 0.22924693 0.71482824 0.95753697 3.00490682 2.56771932 0.58124529
 2.37756083 1.30722573], Cost: 0.0377
Current parameters: [3.49392378 1.40831751 1.49972123 0.60138868 1.52277589 2.73610192
 2.7953888  1.93130961 1.96172066 2.66047158 1.75498723 0.95378688
 0.24153662 0.71008133 0.95208276 3.01027665 2.56112312 0.60262188
 2.37277499 1.28940891], Cost: 0.0458
Current parameters: [3.45746306 1.4003668  1.53101108 0.57952109 1.48514488 2.74153306
 2.7582702  1.94333071 1.95947438 2.74582512 1.74670175 0.95485267
 0.22892442 0.71635285 0.95775789 3.05671263 2.59823075 0.55774935
 2.38228732 1.31144404], Cost: 0.0348
Current parameters: [3.46125759 1.40325276 1.52346601 0.57772404 1.49327982 2.73311416
 2.71933684 1.94623409 2.0598405  2.74656066 1.73901767 0.93417253
 0.23688145 0.69347249 0.95118611 2.98811195 2.56310785 0.5415838
 2.36927138 1.29546325], Cost: 0.0906
Current parameters: [3.44327203 1.39419929 1.54679501 0.58751264 1.47902984 2.73257303
 2.7821665  1.95293939 1.96813225 2.74534682 1.75169185 0.96021904
 0.24803472 0.72075284 0.94361352 2.99847195 2.57261391 0.57677489
 2.37299579 1.34706745], Cost: 0.0355
Current parameters: [3.49146077 1.41577815 1.49160363 0.57343725 1.50426639 2.77736693
 2.72721384 1.95854208 1.92884839 2.74497922 1.75084621 0.97390284
 0.28097811 0.72025166 0.97678265 3.00634909 2.55795569 0.54024848
 2.41647074 1.37457827], Cost: 0.0494
Current parameters: [3.45472701 1.39870312 1.54101863 0.58051785 1.48105713 2.7442197
 2.7535076  1.88492642 1.9521205  2.74592547 1.7495729  0.9600972
 0.24168353 0.72067237 0.95957958 3.00040742 2.57009397 0.56275108
```



```
 2.38656686 1.31399474], Cost: 0.0429
Current parameters: [3.47513792 1.37845199 1.53224551 0.57882245 1.4848366  2.73947994
 2.75987707 1.9436781  1.95194966 2.74602921 1.74699315 0.95450561
 0.23034126 0.71663018 0.95827378 2.99493196 2.58396436 0.55897339
 2.38212816 1.31150794], Cost: 0.0347
Current parameters: [3.44952791 1.42489395 1.5212719  0.58455108 1.49474373 2.74456021
 2.76858882 1.94005049 1.95094904 2.74559041 1.74614684 0.95447525
 0.23619829 0.71329956 0.95660418 2.97817    2.62117533 0.55415813
 2.38290799 1.31005323], Cost: 0.0474
Current parameters: [3.4587252  1.40049144 1.53046434 0.58098588 1.48603385 2.74228951
 2.76298668 1.941958   1.95236473 2.74216582 1.71612704 0.956294
 0.2274001  0.71843152 0.95796307 3.00061865 2.57204953 0.55957975
 2.38372192 1.31433402], Cost: 0.0354
Current parameters: [3.4498912  1.40123319 1.53196243 0.5710746  1.47691388 2.75160537
 2.74654285 1.94967695 1.93560569 2.74483487 1.7495249  0.91360349
 0.22249181 0.71214111 0.95520155 3.00046958 2.5709299  0.5917542
 2.39162442 1.31388093], Cost: 0.0765
Current parameters: [3.4567462  1.40326842 1.53061361 0.58295396 1.4820519  2.74672268
 2.75658582 1.94796655 1.95821792 2.74566162 1.75100893 0.96974478
 0.22132277 0.72737901 0.96198041 2.99825883 2.57209843 0.56920399
 2.39192066 1.30526089], Cost: 0.0434
Current parameters: [3.45492844 1.39467732 1.52273624 0.5795202  1.47306061 2.73917966
 2.77405793 1.94587551 1.9301896  2.74903627 1.7477688  0.98480855
 0.23054644 0.68504444 0.94634118 3.00660323 2.56142874 0.54171504
 2.35892533 1.30320781], Cost: 0.1206
Current parameters: [3.4646416  1.40269229 1.52934627 0.5849936  1.48675195 2.74522964
 2.7666374  1.93756398 1.9553073  2.74427256 1.74921884 0.95331385
 0.20927629 0.70604504 0.9495231  3.00054823 2.57221232 0.5536239
 2.39072675 1.3243051 ], Cost: 0.0666
Current parameters: [3.45498022 1.39417642 1.52545415 0.58899853 1.49276232 2.74167623
 2.77977462 1.94299387 1.95192565 2.74610535 1.74949988 0.93357889
 0.23336998 0.76193641 0.97017682 3.00362014 2.56636998 0.53365411
 2.37380335 1.30860717], Cost: 0.0282
Current parameters: [3.4543993  1.39478314 1.53011248 0.58521782 1.50280849 2.74881539
 2.77343167 1.94194357 1.95118393 2.74438168 1.75047325 0.93676547
 0.2233093  0.76249513 0.98176581 3.00265077 2.56785768 0.53756288
 2.3535877  1.31691815], Cost: 0.0163
Current parameters: [3.44347541 1.38603597 1.55118862 0.56747739 1.54802059 2.75223552
 2.77042379 1.94589709 1.94616723 2.74602032 1.75034259 0.94266046
 0.22758859 0.76864452 0.95871593 3.00381109 2.56546636 0.53397217
 2.36491288 1.30839125], Cost: 0.0045
```



```
Current parameters: [3.42906682 1.3733544  1.54688964 0.57348019 1.55455606 2.75465603
 2.77613225 1.9456442  1.94845622 2.7460202  1.75034151 0.94212718
 0.22821853 0.7579623  0.9752206  3.00389768 2.56537886 0.53419878
 2.37385111 1.30833143], Cost: 0.0062
Current parameters: [3.46215019 1.40477455 1.58618145 0.55256606 1.55136592 2.73482748
 2.78417701 1.95132371 1.94699587 2.74580366 1.75036926 0.94029272
 0.23494427 0.76390252 0.99084431 3.00502824 2.56351041 0.53179298
 2.37472674 1.30612005], Cost: 0.0414
Current parameters: [3.44363903 1.3872943  1.55835411 0.59023529 1.55269535 2.74076735
 2.75986238 1.94789066 1.93464321 2.74585747 1.75043054 0.94321473
 0.22681329 0.76912937 0.95869242 3.00467723 2.5659003  0.53401183
 2.36527893 1.3097108 ], Cost: 0.0106
Current parameters: [3.44135925 1.38612223 1.54772136 0.57148019 1.5576187  2.7470368
 2.74040896 1.95026006 1.99146611 2.7470316  1.74710153 0.93096148
 0.24125056 0.76349575 0.95747815 2.99859876 2.55878148 0.52342955
 2.35188247 1.30021951], Cost: 0.0214
Current parameters: [3.44149176 1.38679446 1.5511961  0.56232321 1.5437306  2.75829625
 2.7589033  1.95048943 1.94098325 2.76287141 1.74934523 0.94575414
 0.23301301 0.77049493 0.96259581 3.00315298 2.56561523 0.52293459
 2.37179833 1.32244634], Cost: 0.0050
Current parameters: [3.46527161 1.3932701  1.52534584 0.56960999 1.5681164  2.76458099
 2.76664588 1.94885196 1.93911065 2.72020648 1.75491008 0.94979526
 0.25494266 0.77148524 0.9647863  3.01007103 2.55626591 0.54183142
 2.37229236 1.32588552], Cost: 0.0250
Current parameters: [3.43827361 1.385743   1.53916248 0.55566659 1.55087472 2.72884009
 2.76621502 1.94377107 1.94440353 2.74475994 1.75200917 0.94475582
 0.22211713 0.77172698 0.96072395 3.00320439 2.56503094 0.53536201
 2.36607669 1.31371925], Cost: 0.0040
Current parameters: [3.43915485 1.36128063 1.53975474 0.55782196 1.55384241 2.73532553
 2.77021851 1.93437518 1.95516874 2.75264091 1.73072356 0.95426829
 0.21040171 0.80739964 0.95826609 3.00466647 2.59105843 0.55088999
 2.36537567 1.32311704], Cost: 0.0695
Current parameters: [3.4394627  1.38636631 1.54360393 0.54830358 1.54697164 2.74096371
 2.77750871 1.94255866 1.94178613 2.74163545 1.75282882 0.94711039
 0.2201707  0.77213849 0.94459027 3.0038362  2.56386059 0.53684429
 2.35443552 1.30019072], Cost: 0.0132
Current parameters: [3.41878731 1.37683225 1.52641841 0.55434313 1.54927576 2.71364079
 2.75750217 1.95351098 1.9221649  2.75385191 1.75448154 0.96539287
 0.24122693 0.75722988 0.97527862 3.00658312 2.55267259 0.51845649
 2.3416553  1.30773091], Cost: 0.0151
Current parameters: [3.43020465 1.38543611 1.53906131 0.54936557 1.54859055 2.73161276
```



```
 2.7567948  1.94817899 1.93433862 2.74520936 1.75396775 0.92357704
 0.22456269 0.77102876 0.96229301 3.00295439 2.56288431 0.54885625
 2.36576914 1.31277023], Cost: 0.0111
Current parameters: [3.44305889 1.37987364 1.53963501 0.5547996  1.54957308 2.72948157
 2.76319235 1.94262176 1.94403225 2.7451588  1.7502459  0.94499766
 0.21715111 0.77288813 0.96219566 3.00882908 2.55495941 0.53512119
 2.36791604 1.311461  ], Cost: 0.0040
Current parameters: [3.44881724 1.38604562 1.53002073 0.55275049 1.55508507 2.73420146
 2.75508922 1.9369484  1.93694842 2.74601526 1.74656024 0.94280705
 0.20938485 0.77182253 0.96911699 3.0058717  2.5651032  0.52585825
 2.37231433 1.2952521 ], Cost: 0.0063
Current parameters: [3.44045499 1.37997518 1.54290678 0.55399763 1.54764213 2.72992133
 2.76033464 1.92901131 1.94330892 2.74330956 1.75213504 0.94593896
 0.22142109 0.77342362 0.96268929 3.00837644 2.55399857 0.53504858
 2.36838683 1.31184048], Cost: 0.0044
Current parameters: [3.43690629 1.37498283 1.54592919 0.55092502 1.5411438  2.73144996
 2.75627556 1.95021936 1.9427962  2.72321706 1.74987877 0.94640521
 0.21316025 0.77551171 0.96257613 3.00760766 2.55912899 0.52364859
 2.3714766  1.3143683 ], Cost: 0.0077
Current parameters: [3.44430556 1.37822598 1.53958076 0.55521804 1.5494847  2.72973265
 2.76381144 1.9430235  1.94270871 2.74490804 1.74820844 0.94591092
 0.2169928  0.7750613  0.96299863 2.99482597 2.5495238  0.53590463
 2.36847103 1.31088574], Cost: 0.0027
Current parameters: [3.44211941 1.3825108  1.53603949 0.55782385 1.55559666 2.73006967
 2.76689088 1.93787649 1.94184283 2.74671654 1.75701654 0.93967564
 0.2030658  0.76275134 0.95714174 2.99091854 2.54258117 0.52526536
 2.36880361 1.32443545], Cost: 0.0112
Current parameters: [3.44813304 1.37237327 1.53973212 0.55532983 1.54928488 2.72932683
 2.76356562 1.94401555 1.94304464 2.74643474 1.76108596 0.94508523
 0.21764151 0.77808871 0.9641798  2.99336344 2.55223322 0.53487095
 2.36808826 1.30906366], Cost: 0.0016
Current parameters: [3.43757519 1.38555586 1.5387436  0.5569902  1.54733924 2.73390969
 2.75726239 1.94810821 1.94771559 2.74777821 1.77177593 0.95902102
 0.21034047 0.78153105 0.96667868 2.99348248 2.54622838 0.54443006
 2.37545844 1.30447682], Cost: 0.0101
Current parameters: [3.45238715 1.36907276 1.541734   0.55270502 1.54876231 2.72969222
 2.75855621 1.94403923 1.9429629  2.7462165  1.7606625  0.94994556
 0.21581355 0.76843973 0.96298517 2.99138699 2.55618003 0.54075772
 2.36750511 1.31065921], Cost: 0.0061
Current parameters: [3.44833118 1.36945298 1.53414963 0.55801554 1.54038401 2.7207631
 2.76680029 1.94540944 1.9432049  2.74958665 1.76083502 0.94334016
```

```
 0.22744159 0.77256263 0.95186499 2.99282816 2.55090173 0.53103136
 2.38828689 1.30209515], Cost: 0.0036
Current parameters: [3.44982228 1.37378897 1.54297814 0.55320338 1.54772952 2.72825399
 2.77086255 1.94367028 1.9326912  2.74650073 1.76186724 0.94766519
 0.21551043 0.77864324 0.96821282 2.99464922 2.55345851 0.53675667
 2.37244191 1.31152259], Cost: 0.0045
Current parameters: [3.44813304 1.37237327 1.53973212 0.55532983 1.54928488 2.72932683
 2.76356562 1.94401555 1.94304464 2.74643474 1.76108596 0.94508523
 0.21764151 0.77808871 0.9641798  2.99336344 2.55223322 0.53487095
 2.36808826 1.30906366], Cost: 0.0016

   Return from subroutine COBYLA because the MAXFUN limit has been reached.

   NFVALS =  100   F = 1.570093E-03    MAXCV = 0.000000E+00
   X = 3.448133E+00   1.372373E+00   1.539732E+00   5.553298E-01   1.549285E+00
       2.729327E+00   2.763566E+00   1.944016E+00   1.943045E+00   2.746435E+00
       1.761086E+00   9.450852E-01   2.176415E-01   7.780887E-01   9.641798E-01
       2.993363E+00   2.552233E+00   5.348709E-01   2.368088E+00   1.309064E+00
Simulating for QAOA depth p = 11
Current parameters: [1.95815246 0.98129441 2.68331272 2.17947635 2.39727579 0.97483206
 1.20076328 2.81453107 1.67052343 1.45070826 1.96064666 0.04930292
 1.97623656 0.29578322 1.92389988 0.25060929 2.35232439 0.49958904
 1.608623   0.39057825 0.5242464  2.00979671], Cost: 3.7444
Current parameters: [0.95815246 1.98129441 2.68331272 2.17947635 2.39727579 0.97483206
 1.20076328 2.81453107 1.67052343 1.45070826 1.96064666 0.04930292
 1.97623656 0.29578322 1.92389988 0.25060929 2.35232439 0.49958904
 1.608623   0.39057825 0.5242464  2.00979671], Cost: 3.5979
Current parameters: [0.95815246 0.98129441 3.68331272 2.17947635 2.39727579 0.97483206
 1.20076328 2.81453107 1.67052343 1.45070826 1.96064666 0.04930292
 1.97623656 0.29578322 1.92389988 0.25060929 2.35232439 0.49958904
 1.608623   0.39057825 0.5242464  2.00979671], Cost: 2.7244
Current parameters: [0.95815246 0.98129441 2.68331272 3.17947635 2.39727579 0.97483206
 1.20076328 2.81453107 1.67052343 1.45070826 1.96064666 0.04930292
 1.97623656 0.29578322 1.92389988 0.25060929 2.35232439 0.49958904
 1.608623   0.39057825 0.5242464  2.00979671], Cost: 2.3073
Current parameters: [0.95815246 0.98129441 2.68331272 3.17947635 3.39727579 0.97483206
 1.20076328 2.81453107 1.67052343 1.45070826 1.96064666 0.04930292
 1.97623656 0.29578322 1.92389988 0.25060929 2.35232439 0.49958904
 1.608623   0.39057825 0.5242464  2.00979671], Cost: 2.2640
Current parameters: [0.95815246 0.98129441 2.68331272 3.17947635 3.39727579 1.97483206
 1.20076328 2.81453107 1.67052343 1.45070826 1.96064666 0.04930292
```



```
 1.97623656 0.29578322 1.92389988 0.25060929 2.35232439 0.49958904
 1.608623   0.39057825 0.5242464  2.00979671], Cost: 2.9137
Current parameters: [0.95815246 0.98129441 2.68331272 3.17947635 3.39727579 0.97483206
 2.20076328 2.81453107 1.67052343 1.45070826 1.96064666 0.04930292
 1.97623656 0.29578322 1.92389988 0.25060929 2.35232439 0.49958904
 1.608623   0.39057825 0.5242464  2.00979671], Cost: 2.8381
Current parameters: [0.95815246 0.98129441 2.68331272 3.17947635 3.39727579 0.97483206
 1.20076328 3.81453107 1.67052343 1.45070826 1.96064666 0.04930292
 1.97623656 0.29578322 1.92389988 0.25060929 2.35232439 0.49958904
 1.608623   0.39057825 0.5242464  2.00979671], Cost: 1.6789
Current parameters: [0.95815246 0.98129441 2.68331272 3.17947635 3.39727579 0.97483206
 1.20076328 3.81453107 2.67052343 1.45070826 1.96064666 0.04930292
 1.97623656 0.29578322 1.92389988 0.25060929 2.35232439 0.49958904
 1.608623   0.39057825 0.5242464  2.00979671], Cost: 2.0425
Current parameters: [0.95815246 0.98129441 2.68331272 3.17947635 3.39727579 0.97483206
 1.20076328 3.81453107 1.67052343 2.45070826 1.96064666 0.04930292
 1.97623656 0.29578322 1.92389988 0.25060929 2.35232439 0.49958904
 1.608623   0.39057825 0.5242464  2.00979671], Cost: 3.1054
Current parameters: [0.95815246 0.98129441 2.68331272 3.17947635 3.39727579 0.97483206
 1.20076328 3.81453107 1.67052343 1.45070826 2.96064666 0.04930292
 1.97623656 0.29578322 1.92389988 0.25060929 2.35232439 0.49958904
 1.608623   0.39057825 0.5242464  2.00979671], Cost: 1.7111
Current parameters: [0.95815246 0.98129441 2.68331272 3.17947635 3.39727579 0.97483206
 1.20076328 3.81453107 1.67052343 1.45070826 1.96064666 1.04930292
 1.97623656 0.29578322 1.92389988 0.25060929 2.35232439 0.49958904
 1.608623   0.39057825 0.5242464  2.00979671], Cost: 2.2394
Current parameters: [0.95815246 0.98129441 2.68331272 3.17947635 3.39727579 0.97483206
 1.20076328 3.81453107 1.67052343 1.45070826 1.96064666 0.04930292
 2.97623656 0.29578322 1.92389988 0.25060929 2.35232439 0.49958904
 1.608623   0.39057825 0.5242464  2.00979671], Cost: 0.6818
Current parameters: [0.95815246 0.98129441 2.68331272 3.17947635 3.39727579 0.97483206
 1.20076328 3.81453107 1.67052343 1.45070826 1.96064666 0.04930292
 2.97623656 1.29578322 1.92389988 0.25060929 2.35232439 0.49958904
 1.608623   0.39057825 0.5242464  2.00979671], Cost: 3.5279
Current parameters: [0.95815246 0.98129441 2.68331272 3.17947635 3.39727579 0.97483206
 1.20076328 3.81453107 1.67052343 1.45070826 1.96064666 0.04930292
 2.97623656 0.29578322 2.92389988 0.25060929 2.35232439 0.49958904
 1.608623   0.39057825 0.5242464  2.00979671], Cost: 3.4121
Current parameters: [0.95815246 0.98129441 2.68331272 3.17947635 3.39727579 0.97483206
 1.20076328 3.81453107 1.67052343 1.45070826 1.96064666 0.04930292
 2.97623656 0.29578322 1.92389988 1.25060929 2.35232439 0.49958904
```



```
 1.608623   0.39057825 0.5242464  2.00979671], Cost: 2.9019
Current parameters: [0.95815246 0.98129441 2.68331272 3.17947635 3.39727579 0.97483206
 1.20076328 3.81453107 1.67052343 1.45070826 1.96064666 0.04930292
 2.97623656 0.29578322 1.92389988 0.25060929 3.35232439 0.49958904
 1.608623   0.39057825 0.5242464  2.00979671], Cost: 2.0801
Current parameters: [0.95815246 0.98129441 2.68331272 3.17947635 3.39727579 0.97483206
 1.20076328 3.81453107 1.67052343 1.45070826 1.96064666 0.04930292
 2.97623656 0.29578322 1.92389988 0.25060929 2.35232439 1.49958904
 1.608623   0.39057825 0.5242464  2.00979671], Cost: 1.5439
Current parameters: [0.95815246 0.98129441 2.68331272 3.17947635 3.39727579 0.97483206
 1.20076328 3.81453107 1.67052343 1.45070826 1.96064666 0.04930292
 2.97623656 0.29578322 1.92389988 0.25060929 2.35232439 0.49958904
 2.608623   0.39057825 0.5242464  2.00979671], Cost: 2.0671
Current parameters: [0.95815246 0.98129441 2.68331272 3.17947635 3.39727579 0.97483206
 1.20076328 3.81453107 1.67052343 1.45070826 1.96064666 0.04930292
 2.97623656 0.29578322 1.92389988 0.25060929 2.35232439 0.49958904
 1.608623   1.39057825 0.5242464  2.00979671], Cost: 1.1875
Current parameters: [0.95815246 0.98129441 2.68331272 3.17947635 3.39727579 0.97483206
 1.20076328 3.81453107 1.67052343 1.45070826 1.96064666 0.04930292
 2.97623656 0.29578322 1.92389988 0.25060929 2.35232439 0.49958904
 1.608623   0.39057825 1.5242464  2.00979671], Cost: 2.9021
Current parameters: [0.95815246 0.98129441 2.68331272 3.17947635 3.39727579 0.97483206
 1.20076328 3.81453107 1.67052343 1.45070826 1.96064666 0.04930292
 2.97623656 0.29578322 1.92389988 0.25060929 2.35232439 0.49958904
 1.608623   0.39057825 0.5242464  3.00979671], Cost: 2.5150
Current parameters: [ 0.74925096  0.79510342  2.6325691   3.19339696  3.40400188  0.87408713
  1.11173652  3.9052503   1.6141472   1.22951462  1.95564817 -0.03760242
  3.13084337 -0.14552643  1.5005458  -0.09364163  2.13550698  0.36592013
  1.39382535  0.31216709  0.17996406  1.72553845], Cost: 1.8610
Current parameters: [0.45815246 0.98129441 2.68331272 3.17947635 3.39727579 0.97483206
 1.20076328 3.81453107 1.67052343 1.45070826 1.96064666 0.04930292
 2.97623656 0.29578322 1.92389988 0.25060929 2.35232439 0.49958904
 1.608623   0.39057825 0.5242464  2.00979671], Cost: 1.1665
Current parameters: [ 1.11007392  0.79311271  2.63202656  3.19354579  3.40407379  0.87300999
  1.11078466  3.90622025  1.61354444  1.22714966  1.95559473 -0.03853159
  3.13249639 -0.15024481  1.49601939 -0.09732229  2.13318882  0.36449097
  1.39152878  0.31132874  0.17628307  1.72249922], Cost: 1.6139
Current parameters: [0.95815246 0.48129441 2.68331272 3.17947635 3.39727579 0.97483206
 1.20076328 3.81453107 1.67052343 1.45070826 1.96064666 0.04930292
 2.97623656 0.29578322 1.92389988 0.25060929 2.35232439 0.49958904
 1.608623   0.39057825 0.5242464  2.00979671], Cost: 1.0808
```



```
Current parameters: [ 1.11159851  1.10760822  2.63151188  3.19368699  3.40414202  0.87198817
  1.10988169  3.90714038  1.61297263  1.22490617  1.95554403 -0.03941305
  3.13406452 -0.15472088  1.49172544 -0.10081391  2.13098971  0.36313521
  1.38935015  0.31053344  0.17279112  1.71961608], Cost: 1.8118
Current parameters: [0.95815246 0.98129441 2.18331272 3.17947635 3.39727579 0.97483206
 1.20076328 3.81453107 1.67052343 1.45070826 1.96064666 0.04930292
 2.97623656 0.29578322 1.92389988 0.25060929 2.35232439 0.49958904
 1.608623   0.39057825 0.5242464  2.00979671], Cost: 0.7867
Current parameters: [ 1.1117199   1.10770815  2.71655042  3.19369823  3.40414745  0.87190681
  1.1098098   3.90721365  1.6129271   1.22472753  1.95553999 -0.03948323
  3.13418937 -0.15507727  1.49138355 -0.10109193  2.13081461  0.36302726
  1.38917669  0.31047011  0.17251309  1.71938652], Cost: 1.9167
Current parameters: [1.03493618 1.04450128 2.69993157 3.18658729 3.40071162 0.92336943
 1.15528654 3.86087236 1.64172527 1.3377179  1.95809333 0.00490984
 3.05521297 0.07035297 1.70764172 0.07475868 2.2415695  0.43130815
 1.49889984 0.35052418 0.34837974 1.86459161], Cost: 2.9766
Current parameters: [0.95815246 0.98129441 2.68331272 3.42935206 3.39727579 0.97483206
 1.20076328 3.81453107 1.67052343 1.45070826 1.96064666 0.04930292
 2.97623656 0.30366526 1.92389988 0.25060929 2.35232439 0.49958904
 1.608623   0.39057825 0.5242464  2.00979671], Cost: 1.0025
Current parameters: [0.98009416 0.99935639 2.68806172 3.13524459 3.39825761 0.96012611
 1.18776786 3.82777355 1.66229407 1.41842015 1.95991702 0.03661716
 2.99880485 0.77722148 1.86210198 0.20035825 2.32067509 0.4800771
 1.57726853 0.37913241 0.47399077 1.96830292], Cost: 0.2286
Current parameters: [0.98009416 0.99935639 2.68806172 3.13524459 3.39825761 0.73261296
 1.18776786 3.82777355 1.66229407 1.52204336 1.95991702 0.03661716
 2.99880485 0.77722148 1.86210198 0.20035825 2.32067509 0.4800771
 1.57726853 0.37913241 0.47399077 1.96830292], Cost: 0.3845
Current parameters: [0.99429808 1.01104878 2.69113597 3.11555579 3.39889319 1.17253321
 1.17935529 3.83634605 1.65696681 1.86273907 1.95944469 0.02840506
 3.01341438 0.80532058 1.82209723 0.16782833 2.30018698 0.46744609
 1.55697127 0.37172296 0.44145789 1.941442  ], Cost: 1.2422
Current parameters: [0.98009416 0.99935639 2.68806172 3.13524459 3.64754715 0.96012611
 1.20660202 3.82777355 1.66229407 1.41842015 1.95991702 0.03661716
 2.99880485 0.77722148 1.86210198 0.20035825 2.32067509 0.4800771
 1.57726853 0.37913241 0.47399077 1.96830292], Cost: 0.3962
Current parameters: [0.9851906  1.00355168 2.68916477 3.12818015 3.35715353 0.9599088
 1.68504882 3.8308494  1.66038263 1.41003568 1.95974755 0.03367062
 3.00404682 0.78730357 1.84774809 0.18868636 2.31332386 0.47554503
 1.56998578 0.37647386 0.46231782 1.9586651 ], Cost: 0.4709
Current parameters: [ 0.98009416  0.99935639  2.68806172  3.13524459  3.39825761  0.96012611
```

```
 1.18776786  3.6548324   1.66229407  1.41842015  1.95991702 -0.14391361
 2.99880485  0.77722148  1.86210198  0.20035825  2.32067509  0.4800771
 1.57726853  0.37913241  0.47399077  1.96830292], Cost: 0.5836
Current parameters: [0.98731301 1.00529881 2.68962414 3.12523816 3.39345013 0.9598183
 1.18514704 3.47558716 1.6595866  1.40654395 1.95967697 0.3886406
 3.00622984 0.79150227 1.84177039 0.18382559 2.31026243 0.47365765
 1.56695287 0.37536671 0.4574566  1.95465141], Cost: 1.1367
Current parameters: [0.98009416 0.99935639 2.68806172 3.13524459 3.39825761 0.96012611
 1.18776786 3.82777355 1.68437323 1.41842015 1.71089391 0.03661716
 2.99880485 0.77722148 1.86210198 0.20035825 2.32067509 0.4800771
 1.57726853 0.37913241 0.47399077 1.96830292], Cost: 0.4897
Current parameters: [0.98328997 1.00198712 2.68875341 3.13081471 3.39612933 0.95998984
 1.18660761 3.83476463 2.15957786 1.41316252 2.00746405 0.03640256
 3.00209192 0.78354363 1.85310113 0.19303919 2.31606537 0.47723519
 1.57270176 0.37746532 0.46667105 1.96225936], Cost: 0.9184
Current parameters: [0.98009416 0.99935639 2.68806172 3.13524459 3.39825761 0.96012611
 1.18776786 3.82777355 1.66229407 1.41842015 1.95991702 0.03661716
 2.99880485 0.77722148 2.01983489 0.20035825 2.32067509 0.4800771
 1.57726853 0.37913241 0.28003116 1.96830292], Cost: 0.2767
Current parameters: [1.01585505 1.02879406 2.6958017  3.08567455 3.37444223 0.95860131
 1.17478481 3.90600334 1.61141532 1.3595876  1.99408198 0.03421578
 3.03558694 0.84796595 2.21055295 0.11845842 2.26909263 0.44827628
 1.52616659 0.36047781 0.76651148 1.90067579], Cost: 3.1704
Current parameters: [0.98009416 0.99935639 2.68806172 3.13524459 3.39825761 0.96012611
 1.18776786 3.82777355 1.66229407 1.41842015 1.95991702 0.03661716
 2.99880485 0.77722148 1.86210198 0.35953769 2.32067509 0.4800771
 1.57726853 0.37913241 0.47399077 1.77552869], Cost: 1.0818
Current parameters: [1.00573177 1.02046077 2.69361065 3.09970696 3.38118395 0.95903295
 1.17846008 3.88385785 1.6258182  1.37624207 1.98441048 0.03489557
 3.02517458 0.82793943 1.77087057 0.49847442 2.28369472 0.45727854
 1.54063265 0.3657586  0.40635993 2.33151092], Cost: 2.2484
Current parameters: [0.98009416 0.99935639 2.68806172 3.13524459 3.39825761 0.96012611
 1.18776786 3.82777355 1.66229407 1.41842015 1.95991702 0.03661716
 2.99880485 0.77722148 1.86210198 0.20035825 2.49662263 0.4800771
 1.39966655 0.37913241 0.47399077 1.96830292], Cost: 0.5056
Current parameters: [0.99605128 1.01249198 2.69151543 3.11312559 3.38763078 0.95944572
 1.1819746  3.86268101 1.63959111 1.39216807 1.975162   0.03554562
 3.01521765 0.80878887 1.80531859 0.11487425 2.65008413 0.46588703
 1.92928026 0.37080841 0.43189662 1.97056601], Cost: 0.7737
Current parameters: [0.98009416 0.99935639 2.68806172 3.13524459 3.39825761 0.96012611
 1.18776786 3.82777355 1.66229407 1.41842015 1.95991702 0.03661716
```



```
 2.83529841 0.77722148 1.86210198 0.20035825 2.32067509 0.29095902
 1.57726853 0.37913241 0.47399077 1.96830292], Cost: 0.1126
Current parameters: [0.99083729 1.00819992 2.69038693 3.12035298 3.3911031  0.95966803
 1.18386755 3.85127499 1.64700931 1.40074594 1.9701807  0.03589575
 2.45991494 0.79847422 1.82387258 0.14280617 2.30154636 0.60870845
 1.57560118 0.37352828 0.4456509  1.96982655], Cost: 3.0520
Current parameters: [0.98009416 0.99935639 2.68806172 3.13503713 3.3844797  0.9698556
 1.17901668 3.84361571 1.65327815 1.40504551 1.97357216 0.0413795
 2.84759115 0.78379813 1.84339376 0.18003761 2.30127666 0.29936419
 1.57831809 0.6240783  0.47733481 1.97019572], Cost: 1.0358
Current parameters: [ 1.00233693  1.0176662   2.69287588  3.10694597  3.55168926  0.84036919
  1.28655474  3.68298005  1.74074328  1.54514721  1.81442181 -0.02303029
  2.78196504  0.74091489  2.01140047  0.32934023  2.51794829  0.09057985
  1.5610001   0.38782337  0.37448075  1.9483442 ], Cost: 0.7861
Current parameters: [ 1.00767027  1.02205651  2.69403021  3.10229778  3.41875436  0.89890984
  1.17075722  3.87684729  1.62785138  1.33344712  1.94831366 -0.00738875
  2.97514508  0.66445557  1.66987683 -0.01032319  2.24944644  0.10207532
  1.49726172  0.23578406  0.27545777  1.87066097], Cost: 2.8752
Current parameters: [0.98331379 1.00200672 2.68875856 3.38284079 3.40019146 0.95985734
 1.19186021 3.82730915 1.6621219  1.42360146 1.95890356 0.03741285
 2.84107013 0.80214801 1.86856391 0.19926446 2.32716232 0.27803247
 1.58259298 0.3677495  0.47431751 1.9763109 ], Cost: 0.2180
Current parameters: [0.90065082 1.09586419 2.68806172 3.13524459 3.39825761 0.96012611
 1.18776786 3.82777355 1.66229407 1.41842015 1.95991702 0.03661716
 2.83529841 0.77722148 1.86210198 0.20035825 2.32067509 0.29095902
 1.57726853 0.37913241 0.47399077 1.96830292], Cost: 0.1266
Current parameters: [0.78861251 0.84125445 2.68875366 3.1340801  3.40017786 0.95985923
 1.19183142 3.82731242 1.66212311 1.42356501 1.95891069 0.03740726
 2.84102953 0.79418074 1.86851846 0.19927215 2.32711669 0.2781234
 1.58255552 0.36782957 0.47431571 1.97625457], Cost: 0.2031
Current parameters: [0.98030259 0.99952797 2.81068697 3.13497545 3.39698293 0.96084773
 1.18678474 3.81150995 1.66147157 1.41675932 1.96119449 0.05405976
 2.83639254 0.77700658 1.8605029  0.19858074 2.31893897 0.29179122
 1.57744203 0.37902368 0.47442412 1.96857961], Cost: 0.1777
Current parameters: [0.98268447 1.00107578 2.63835226 3.13152661 3.38707183 0.96716725
 1.18137167 3.66349002 1.65385823 1.40612895 1.97192047 0.2130654
 2.85127475 0.78970685 1.85153147 0.1815084  2.30874544 0.28825632
 1.58358428 0.36827238 0.47863781 1.97796034], Cost: 0.1740
Current parameters: [0.98160112 1.00059689 2.68838788 3.13308414 3.38139004 0.97464489
 1.30768352 3.83383888 1.6574207  1.4315139  1.96730106 0.04158835
 2.84194038 0.78247167 1.85199996 0.18938166 2.31019289 0.29550853
```



```
 1.5778433  0.37834631 0.47581062 1.96933601], Cost: 0.1488
Current parameters: [0.98571454 1.0034807  2.68741337 3.12745235 3.39249794 1.06811843
 1.14123292 3.83781588 1.65382052 1.63360343 1.96975257 0.0434033
 2.85406451 0.79243004 1.84597927 0.17651433 2.3078362  0.28176462
 1.57970368 0.36463357 0.47066646 1.97293442], Cost: 0.3297
Current parameters: [0.97959351 0.99894426 2.68795336 3.1358587  3.39365498 0.9646274
 1.18754081 3.83029998 1.78296613 1.41718914 1.9752409  0.04057627
 2.83779633 0.77876291 1.86127253 0.17752844 2.31648117 0.29488905
 1.5795982  0.37939357 0.47925389 1.95542583], Cost: 0.1095
Current parameters: [0.9896201  1.0058933  2.6852797  3.12188675 3.37358619 0.97703113
 1.18368264 3.85791878 1.82192252 1.39645839 2.00245733 0.05157948
 2.87940388 0.81142723 1.82311913 0.31993867 2.2936994  0.27225716
 1.5911529  0.34411823 0.46885108 2.13136251], Cost: 0.3881
Current parameters: [0.98616902 1.0043571  2.68937654 3.12349732 3.39723958 0.95724992
 1.18429659 3.83277523 1.77944168 1.40902522 1.97158534 0.03359366
 2.84238171 0.89469805 1.84651634 0.16357501 2.3374706  0.28652263
 1.59570464 0.37596347 0.46296163 1.93958553], Cost: 0.0753
Current parameters: [0.99051517 1.00752361 2.68879075 3.11951271 3.37164882 0.97809777
 1.18218094 3.8580787  1.76913897 1.39316993 1.99751536 0.04785748
 2.8693233  0.84193808 1.82755232 0.12937632 2.48216928 0.29291974
 1.77694767 0.36422798 0.47888888 1.95473568], Cost: 0.2660
Current parameters: [0.98917547 1.00683195 2.69002725 3.11870152 3.39123238 0.96503643
 1.18663946 3.83647588 1.77719245 1.41209526 1.97722235 0.03809934
 2.83806522 0.92056743 1.93544709 0.17073168 2.3368544  0.29793973
 1.60327769 0.38008101 0.54277573 1.95303443], Cost: 0.5820
Current parameters: [0.99899041 1.01174802 2.68040685 3.10834634 3.40039291 0.93046118
 1.16758648 3.86659294 1.75969988 1.35722366 1.97613892 0.02124789
 2.94298713 0.86240968 1.81250486 0.05040791 2.28855884 0.16432224
 1.55804113 0.27361948 0.41943019 1.92132178], Cost: 0.6126
Current parameters: [0.99008318 1.00757916 2.69022371 3.11860661 3.42882736 0.93338301
 1.2053394  3.79931903 1.79713538 1.43643986 1.93994103 0.01982939
 2.86759878 0.88548375 1.87876649 0.20120236 2.38082317 0.22098695
 1.59208392 0.39635937 0.44654627 1.92775134], Cost: 0.2174
Current parameters: [0.99153425 1.00534996 2.67782666 3.1176244  3.43982569 0.91495842
 1.19838644 3.80769706 1.7956259  1.4238518  1.93819425 0.0218676
 2.66238587 0.88327267 1.80488747 0.15042838 2.37184405 0.34918326
 1.58304119 0.30361032 0.35845709 1.95629669], Cost: 1.1665
Current parameters: [1.02711738 0.98371243 2.6519193  3.10507826 3.39427825 0.95202724
 1.14798    3.85624323 1.74198163 1.38383348 1.97397522 0.01899027
 2.82796701 0.88613527 1.84581007 0.22627521 2.3363707  0.27876912
 1.60262291 0.35572487 0.46971984 1.88629572], Cost: 0.1298
```



```
Current parameters: [0.91186932 1.03587675 2.74725571 3.1603012  3.35886686 1.00278583
 1.23776994 3.82866835 1.84097701 1.42777712 2.01596187 0.09751922
 2.89671791 0.90377476 1.78755184 0.24267368 2.29947363 0.31482232
 1.61371854 0.36155556 0.45167332 1.8017707 ], Cost: 0.7803
Current parameters: [ 0.98704989  1.00323748  2.68742367  3.12348585  3.37819886  0.97401729
  1.18104471  3.75331751  1.77666388  1.39959719  1.9911637  -0.05050337
  2.85446029  0.89377912  1.83078996  0.16287491  2.32301614  0.29859646
  1.60930517  0.36971296  0.47411184  1.94081394], Cost: 0.1435
Current parameters: [0.98535695 1.00476651 2.69011938 3.12386261 3.34084912 0.94918488
 1.18075199 3.83250059 1.78191382 1.41324529 1.95203432 0.0340824
 2.83679705 0.89486787 1.85219357 0.16322409 2.34380962 0.27988631
 1.58919083 0.37636483 0.45586369 1.93956145], Cost: 0.0707
Current parameters: [0.98755768 1.0031918  2.68731635 3.12313764 3.35870731 0.97656341
 1.17944738 3.83339822 1.78924069 1.39846868 1.84494976 0.03270372
 2.85607765 0.89390171 1.83021925 0.16343085 2.32141208 0.30122809
 1.61134345 0.37127264 0.47747562 1.94045464], Cost: 0.1583
Current parameters: [0.98645543 1.0042127  2.68911455 3.12336849 3.34112037 0.90063897
 1.17980425 3.8328721  1.78094092 1.43461642 1.95173752 0.03342128
 2.84435136 0.89463816 1.84451404 0.16369877 2.32400531 0.28886318
 1.60933716 0.37582191 0.465465   1.93959402], Cost: 0.1077
Current parameters: [0.98957788 1.0018555  2.68492873 3.12240994 3.34196667 0.92693284
 1.17591414 3.83418622 1.77786515 1.41914961 1.95124739 0.03145086
 2.8728031  0.8931336  1.81159463 0.16379539 2.40901709 0.32003086
 1.52267677 0.36753843 0.49675668 1.94108015], Cost: 0.1846
Current parameters: [0.98369103 1.00622892 2.69217378 3.1241679  3.33985576 0.95779492
 1.17932128 3.8326122  1.78317044 1.42953316 1.95336009 0.03580531
 2.81119878 0.89639805 1.82415153 0.16053533 2.34237889 0.24762031
 1.58950275 0.37727986 0.4883121  1.93982367], Cost: 0.0517
Current parameters: [1.00040356 1.00147907 2.67949504 3.1139392  3.34002639 1.00539955
 1.14416766 3.84312611 1.76661309 1.52720142 1.95516584 0.03064462
 2.83407548 0.90038406 1.83007966 0.15318165 2.3379591  0.25997004
 1.5891307  0.36432153 0.46342289 1.94357133], Cost: 0.1096
Current parameters: [ 0.97769965  1.0085719   2.70933103  3.1371468   3.34158518  0.95701134
  1.18640444  3.87182763  1.79031935  1.43320578  1.95154451 -0.0053929
  2.80821179  0.89512906  1.82644405  0.16332317  2.3448227   0.24794609
  1.58844588  0.38017382  0.48756505  1.9374695 ], Cost: 0.0624
Current parameters: [1.00105931 0.99999719 2.67317093 3.24209259 3.34285894 0.9556747
 1.1654924  3.8213111  1.7680223  1.42941506 1.95079422 0.04571878
 2.8136648  0.90755038 1.82671193 0.16290084 2.34644181 0.23985126
 1.59171359 0.36801505 0.48662322 1.94593185], Cost: 0.1129
Current parameters: [0.94169046 0.96250865 2.68540172 3.123992   3.34183812 0.95645087
```

```
 1.173747   3.83266659 1.77678601 1.43042827 1.95153101 0.0344264
 2.81180085 0.90002184 1.82672873 0.16262549 2.3451931  0.24384456
 1.59059129 0.37044517 0.48790962 1.9423705 ], Cost: 0.0265
Current parameters: [0.94373614 0.9445713  2.66742663 3.12151329 3.33658946 0.96040006
 1.15136921 3.84467383 1.75881587 1.41647935 1.95857682 0.03316324
 2.81923359 0.89927443 1.79730581 0.14873315 2.33361725 0.23802072
 1.58716658 0.47999518 0.46940748 1.95104851], Cost: 0.2508
Current parameters: [0.95037252 0.96138055 2.68020766 3.11763673 3.34052203 0.95505089
 1.1681257  3.83974547 1.78007163 1.42331887 1.95407087 0.03144753
 2.81714528 0.90607191 1.820023   0.18686024 2.34257072 0.23547555
 1.59126669 0.36202332 0.48386408 1.99469681], Cost: 0.0144
Current parameters: [0.91540482 0.97458775 2.70582083 3.13004731 3.31713026 0.98251602
 1.19045843 3.84365662 1.81504902 1.42622868 1.98260262 0.06397693
 2.85044185 0.91640005 1.77994916 0.25013682 2.31947996 0.24099462
 1.60055735 0.35489658 0.4723965  1.96570226], Cost: 0.1111
Current parameters: [0.94829792 0.96012208 2.68016579 3.12004789 3.3461989  0.95423269
 1.17345024 3.8289505  1.78690237 1.43597238 1.9480098  0.03178777
 2.77647917 0.91025627 1.81829114 0.20847356 2.35691799 0.26494101
 1.59599416 0.37414338 0.47559527 1.99917943], Cost: 0.0329
Current parameters: [0.94264534 0.96295158 2.67820797 3.10638658 3.35159538 0.94134317
 1.17231126 3.84363604 1.78659766 1.4070722  1.95463483 0.03286359
 2.77386267 0.8928475  1.78263216 0.14455745 2.33292995 0.21445725
 1.57260719 0.29608758 0.41824937 1.99763679], Cost: 0.6118
Current parameters: [0.95052993 0.96168094 2.68039651 3.11708255 3.35465848 0.94405121
 1.17440274 3.82517976 1.78729034 1.43425111 1.93986368 0.02461474
 2.81860884 0.91458687 1.83294969 0.20442363 2.32470669 0.21583279
 1.55269852 0.37015057 0.47596003 1.98821341], Cost: 0.0217
Current parameters: [0.95177312 0.96469183 2.68108496 3.11219995 3.35893323 0.94252407
 1.18542262 3.81915975 1.79394403 1.44048767 1.93675009 0.02527434
 2.84622337 0.88199588 1.83089604 0.20811031 2.41801877 0.17997392
 1.63811449 0.37524056 0.46506625 1.98784266], Cost: 0.0766
Current parameters: [ 0.94847316  0.96241272  2.68199867  3.11819393  3.34901354  0.96458108
  1.16992066  3.80144863  1.7804325   1.41877626  1.9680835  -0.00718581
  2.82593225  0.91316486  1.8054205   0.18083843  2.33566091  0.24233709
  1.59971392  0.35999354  0.48524135  1.99722458], Cost: 0.0857
Current parameters: [9.59224678e-01 9.52494124e-01 2.66647698e+00 3.11344020e+00
 3.24339603e+00 9.54313111e-01 1.15102032e+00 3.82094189e+00
 1.77260261e+00 1.41991387e+00 1.93783170e+00 3.61866257e-03
 2.80562693e+00 8.66243323e-01 1.84829149e+00 2.17858341e-01
 2.33908532e+00 2.28685328e-01 1.58567796e+00 3.55410333e-01
 5.02593101e-01 1.98407404e+00], Cost: 0.0820
```



```
Current parameters: [0.93884157 0.96686061 2.68520754 3.10970685 3.35426187 0.97276358
 1.17899427 3.84819767 1.78974062 1.42482129 1.9732838  0.049333
 2.81873764 0.94689378 1.90098408 0.18945847 2.34402664 0.23722597
 1.60451568 0.36896249 0.55569653 2.0113726 ], Cost: 0.4842
Current parameters: [0.95149873 0.95968291 2.67755076 3.11636053 3.33913018 0.94413526
 1.1657889  3.84162091 1.77872235 1.42622406 1.95391063 0.03168017
 2.83534417 0.90459693 1.79960326 0.18829586 2.37570731 0.25428032
 1.55780566 0.35757159 0.50409543 1.99512705], Cost: 0.0222
Current parameters: [0.93435036 0.98063248 2.68147605 3.11779696 3.34277267 0.9530528
 1.15549399 3.83866819 1.76785182 1.42205044 1.95168973 0.03068951
 2.81619299 0.90535346 1.8211589  0.19040646 2.34410006 0.23459013
 1.59096224 0.36207753 0.48312174 1.99594869], Cost: 0.0114
Current parameters: [0.94693162 0.96469532 2.73457865 3.11768642 3.34430596 0.95090758
 1.13893604 3.83084136 1.75221377 1.41970048 1.94928523 0.03763551
 2.81563164 0.90416348 1.8217645  0.19456746 2.34587395 0.23344812
 1.59023018 0.36207038 0.48238245 1.99757619], Cost: 0.0142
Current parameters: [0.92965618 0.98320083 2.68594193 3.11984307 3.3359142  0.95460422
 1.17773286 3.83638885 1.75344438 1.42774628 1.94846742 0.03275288
 2.8178749  0.90422933 1.8207046  0.18519612 2.34337407 0.23516402
 1.58890714 0.36439345 0.48152908 2.00463823], Cost: 0.0204
Current parameters: [0.95122583 0.97290656 2.66693892 3.10865463 3.35651076 0.95487563
 1.16349503 3.84930774 1.72960978 1.41262144 1.95721228 0.02518226
 2.81361072 0.91151362 1.81602687 0.21688123 2.34290323 0.23260182
 1.59618343 0.35344162 0.48616124 1.97602196], Cost: 0.0119
Current parameters: [0.93295058 0.97956785 2.68128398 3.12035573 3.35209952 0.95374186
 1.15702433 3.83812844 1.76739631 1.42422926 1.96636301 0.03216539
 2.81462666 0.88132269 1.82506671 0.19335902 2.34048354 0.23574325
 1.58693131 0.36317523 0.48599241 1.99916218], Cost: 0.0115
Current parameters: [0.93400627 0.97845324 2.68001126 3.118811   3.3586685  0.96874191
 1.1556007  3.83971827 1.77139402 1.41538884 1.90558029 0.03238104
 2.82577765 0.88507221 1.81188061 0.19442236 2.33020188 0.24628533
 1.59896496 0.35991678 0.49735644 1.99833986], Cost: 0.0593
Current parameters: [0.93431094 0.98042189 2.68050649 3.13995168 3.34325801 0.94414648
 1.15947844 3.83482763 1.76824509 1.40433345 1.9509222  0.03332355
 2.81244637 0.90655289 1.82051748 0.19214268 2.34562725 0.23098091
 1.59138137 0.36283033 0.48734613 1.99632144], Cost: 0.0061
Current parameters: [0.9461018  0.97604831 2.66931658 3.17924013 3.34336436 0.9598316
 1.14189104 3.83521904 1.75691219 1.43767114 1.95051872 0.03630156
 2.82176139 0.91149407 1.82304711 0.19189002 2.34645193 0.23186706
 1.59152407 0.35468218 0.47836161 1.99926791], Cost: 0.0114
Current parameters: [0.93407514 0.97806263 2.67881655 3.14107749 3.33836026 0.94773401
```



```
 1.15353596 3.84063998 1.76350446 1.39917794 1.95565039 0.03475518
 2.80693214 0.91217638 1.81563725 0.18649193 2.32725189 0.24326038
 1.58042342 0.36950463 0.49065223 1.99869504], Cost: 0.0205
Current parameters: [0.93631651 0.97237587 2.67238778 3.13934111 3.34166977 0.94272737
 1.15086785 3.8391221  1.76210846 1.40193223 1.95029771 0.03449258
 2.82355936 0.89988893 1.80781472 0.19375028 2.35760733 0.21613104
 1.59797112 0.41504617 0.47461373 1.99730956], Cost: 0.0261
Current parameters: [0.92656276 0.98436878 2.68750786 3.14304766 3.35430099 0.94924803
 1.16631088 3.83924961 1.77585327 1.40562327 1.95868052 0.04443486
 2.82105174 0.91488244 1.8091097  0.19927964 2.3420032  0.23318062
 1.59415911 0.36321949 0.48286877 1.99250804], Cost: 0.0120
Current parameters: [0.93431094 0.98042189 2.68050649 3.13995168 3.34325801 0.94414648
 1.15947844 3.83482763 1.76824509 1.40433345 1.9509222  0.03332355
 2.81244637 0.90655289 1.82051748 0.19214268 2.34562725 0.23098091
 1.59138137 0.36283033 0.48734613 1.99632144], Cost: 0.0061

   Return from subroutine COBYLA because the MAXFUN limit has been reached.

   NFVALS =  100   F = 6.056883E-03    MAXCV = 0.000000E+00
   X = 9.343109E-01   9.804219E-01   2.680506E+00   3.139952E+00   3.343258E+00
       9.441465E-01   1.159478E+00   3.834828E+00   1.768245E+00   1.404333E+00
       1.950922E+00   3.332355E-02   2.812446E+00   9.065529E-01   1.820517E+00
       1.921427E-01   2.345627E+00   2.309809E-01   1.591381E+00   3.628303E-01
       4.873461E-01   1.996321E+00
Simulating for QAOA depth p = 12
Current parameters: [3.26577728 0.05165197 1.38843968 0.47641421 3.0448943  1.65839884
 0.97305346 0.43108744 0.16779391 2.09495121 1.72076417 0.48475438
 0.9932965  0.44149458 2.35617943 1.73966342 0.93089444 1.43258378
 0.92203514 3.11451862 3.11281849 2.94365735 0.77826897 1.71769913], Cost: 1.2278
Current parameters: [3.26577728 1.05165197 1.38843968 0.47641421 3.0448943  1.65839884
 0.97305346 0.43108744 0.16779391 2.09495121 1.72076417 0.48475438
 0.9932965  0.44149458 2.35617943 1.73966342 0.93089444 1.43258378
 0.92203514 3.11451862 3.11281849 2.94365735 0.77826897 1.71769913], Cost: 0.0986
Current parameters: [3.26577728 1.05165197 2.38843968 0.47641421 3.0448943  1.65839884
 0.97305346 0.43108744 0.16779391 2.09495121 1.72076417 0.48475438
 0.9932965  0.44149458 2.35617943 1.73966342 0.93089444 1.43258378
 0.92203514 3.11451862 3.11281849 2.94365735 0.77826897 1.71769913], Cost: 2.1004
Current parameters: [3.26577728 1.05165197 1.38843968 1.47641421 3.0448943  1.65839884
 0.97305346 0.43108744 0.16779391 2.09495121 1.72076417 0.48475438
 0.9932965  0.44149458 2.35617943 1.73966342 0.93089444 1.43258378
 0.92203514 3.11451862 3.11281849 2.94365735 0.77826897 1.71769913], Cost: 1.2736
```



```
Current parameters: [3.26577728 1.05165197 1.38843968 0.47641421 4.0448943  1.65839884
 0.97305346 0.43108744 0.16779391 2.09495121 1.72076417 0.48475438
 0.9932965  0.44149458 2.35617943 1.73966342 0.93089444 1.43258378
 0.92203514 3.11451862 3.11281849 2.94365735 0.77826897 1.71769913], Cost: 1.3434
Current parameters: [3.26577728 1.05165197 1.38843968 0.47641421 3.0448943  2.65839884
 0.97305346 0.43108744 0.16779391 2.09495121 1.72076417 0.48475438
 0.9932965  0.44149458 2.35617943 1.73966342 0.93089444 1.43258378
 0.92203514 3.11451862 3.11281849 2.94365735 0.77826897 1.71769913], Cost: 1.7856
Current parameters: [3.26577728 1.05165197 1.38843968 0.47641421 3.0448943  1.65839884
 1.97305346 0.43108744 0.16779391 2.09495121 1.72076417 0.48475438
 0.9932965  0.44149458 2.35617943 1.73966342 0.93089444 1.43258378
 0.92203514 3.11451862 3.11281849 2.94365735 0.77826897 1.71769913], Cost: 2.0426
Current parameters: [3.26577728 1.05165197 1.38843968 0.47641421 3.0448943  1.65839884
 0.97305346 1.43108744 0.16779391 2.09495121 1.72076417 0.48475438
 0.9932965  0.44149458 2.35617943 1.73966342 0.93089444 1.43258378
 0.92203514 3.11451862 3.11281849 2.94365735 0.77826897 1.71769913], Cost: 2.7771
Current parameters: [3.26577728 1.05165197 1.38843968 0.47641421 3.0448943  1.65839884
 0.97305346 0.43108744 1.16779391 2.09495121 1.72076417 0.48475438
 0.9932965  0.44149458 2.35617943 1.73966342 0.93089444 1.43258378
 0.92203514 3.11451862 3.11281849 2.94365735 0.77826897 1.71769913], Cost: 2.6617
Current parameters: [3.26577728 1.05165197 1.38843968 0.47641421 3.0448943  1.65839884
 0.97305346 0.43108744 0.16779391 3.09495121 1.72076417 0.48475438
 0.9932965  0.44149458 2.35617943 1.73966342 0.93089444 1.43258378
 0.92203514 3.11451862 3.11281849 2.94365735 0.77826897 1.71769913], Cost: 2.5218
Current parameters: [3.26577728 1.05165197 1.38843968 0.47641421 3.0448943  1.65839884
 0.97305346 0.43108744 0.16779391 2.09495121 2.72076417 0.48475438
 0.9932965  0.44149458 2.35617943 1.73966342 0.93089444 1.43258378
 0.92203514 3.11451862 3.11281849 2.94365735 0.77826897 1.71769913], Cost: 2.7152
Current parameters: [3.26577728 1.05165197 1.38843968 0.47641421 3.0448943  1.65839884
 0.97305346 0.43108744 0.16779391 2.09495121 1.72076417 1.48475438
 0.9932965  0.44149458 2.35617943 1.73966342 0.93089444 1.43258378
 0.92203514 3.11451862 3.11281849 2.94365735 0.77826897 1.71769913], Cost: 0.4834
Current parameters: [3.26577728 1.05165197 1.38843968 0.47641421 3.0448943  1.65839884
 0.97305346 0.43108744 0.16779391 2.09495121 1.72076417 0.48475438
 1.9932965  0.44149458 2.35617943 1.73966342 0.93089444 1.43258378
 0.92203514 3.11451862 3.11281849 2.94365735 0.77826897 1.71769913], Cost: 0.0692
Current parameters: [3.26577728 1.05165197 1.38843968 0.47641421 3.0448943  1.65839884
 0.97305346 0.43108744 0.16779391 2.09495121 1.72076417 0.48475438
 1.9932965  1.44149458 2.35617943 1.73966342 0.93089444 1.43258378
 0.92203514 3.11451862 3.11281849 2.94365735 0.77826897 1.71769913], Cost: 2.9005
Current parameters: [3.26577728 1.05165197 1.38843968 0.47641421 3.0448943  1.65839884
```

```
 0.97305346 0.43108744 0.16779391 2.09495121 1.72076417 0.48475438
 1.9932965  0.44149458 3.35617943 1.73966342 0.93089444 1.43258378
 0.92203514 3.11451862 3.11281849 2.94365735 0.77826897 1.71769913], Cost: 1.4350
Current parameters: [3.26577728 1.05165197 1.38843968 0.47641421 3.0448943  1.65839884
 0.97305346 0.43108744 0.16779391 2.09495121 1.72076417 0.48475438
 1.9932965  0.44149458 2.35617943 2.73966342 0.93089444 1.43258378
 0.92203514 3.11451862 3.11281849 2.94365735 0.77826897 1.71769913], Cost: 0.8014
Current parameters: [3.26577728 1.05165197 1.38843968 0.47641421 3.0448943  1.65839884
 0.97305346 0.43108744 0.16779391 2.09495121 1.72076417 0.48475438
 1.9932965  0.44149458 2.35617943 1.73966342 1.93089444 1.43258378
 0.92203514 3.11451862 3.11281849 2.94365735 0.77826897 1.71769913], Cost: 0.5461
Current parameters: [3.26577728 1.05165197 1.38843968 0.47641421 3.0448943  1.65839884
 0.97305346 0.43108744 0.16779391 2.09495121 1.72076417 0.48475438
 1.9932965  0.44149458 2.35617943 1.73966342 0.93089444 2.43258378
 0.92203514 3.11451862 3.11281849 2.94365735 0.77826897 1.71769913], Cost: 3.3547
Current parameters: [3.26577728 1.05165197 1.38843968 0.47641421 3.0448943  1.65839884
 0.97305346 0.43108744 0.16779391 2.09495121 1.72076417 0.48475438
 1.9932965  0.44149458 2.35617943 1.73966342 0.93089444 1.43258378
 1.92203514 3.11451862 3.11281849 2.94365735 0.77826897 1.71769913], Cost: 2.1211
Current parameters: [3.26577728 1.05165197 1.38843968 0.47641421 3.0448943  1.65839884
 0.97305346 0.43108744 0.16779391 2.09495121 1.72076417 0.48475438
 1.9932965  0.44149458 2.35617943 1.73966342 0.93089444 1.43258378
 0.92203514 4.11451862 3.11281849 2.94365735 0.77826897 1.71769913], Cost: 2.5498
Current parameters: [3.26577728 1.05165197 1.38843968 0.47641421 3.0448943  1.65839884
 0.97305346 0.43108744 0.16779391 2.09495121 1.72076417 0.48475438
 1.9932965  0.44149458 2.35617943 1.73966342 0.93089444 1.43258378
 0.92203514 3.11451862 4.11281849 2.94365735 0.77826897 1.71769913], Cost: 2.4614
Current parameters: [3.26577728 1.05165197 1.38843968 0.47641421 3.0448943  1.65839884
 0.97305346 0.43108744 0.16779391 2.09495121 1.72076417 0.48475438
 1.9932965  0.44149458 2.35617943 1.73966342 0.93089444 1.43258378
 0.92203514 3.11451862 3.11281849 3.94365735 0.77826897 1.71769913], Cost: 1.7492
Current parameters: [3.26577728 1.05165197 1.38843968 0.47641421 3.0448943  1.65839884
 0.97305346 0.43108744 0.16779391 2.09495121 1.72076417 0.48475438
 1.9932965  0.44149458 2.35617943 1.73966342 0.93089444 1.43258378
 0.92203514 3.11451862 3.11281849 2.94365735 1.77826897 1.71769913], Cost: 1.6809
Current parameters: [3.26577728 1.05165197 1.38843968 0.47641421 3.0448943  1.65839884
 0.97305346 0.43108744 0.16779391 2.09495121 1.72076417 0.48475438
 1.9932965  0.44149458 2.35617943 1.73966342 0.93089444 1.43258378
 0.92203514 3.11451862 3.11281849 2.94365735 0.77826897 2.71769913], Cost: 1.9950
Current parameters: [ 3.28289664  1.1719327   1.17520026  0.35125423  2.91229767  1.47869475
  0.76597309  0.14577292 -0.1052348   1.8368253   1.44204114  0.4437656
```



```
  1.99643031  0.1398986   2.21068985  1.66166234  0.88008696  1.08260718
  0.70346127  2.85028051  2.85799037  2.76470185  0.60658982  1.51255967], Cost: 2.8793
Current parameters: [3.26773377 1.06539833 1.36406949 0.46211023 3.02974042 1.63786125
 0.94938716 0.91894197 0.13659067 2.06545113 1.68891015 0.48006995
 1.99365465 0.40702651 2.33955207 1.73074902 0.92508787 1.3925865
 0.8970553  3.08432001 3.08369531 2.92320531 0.75864852 1.69425464], Cost: 0.7781
Current parameters: [3.51328294 1.0164249  1.38843968 0.47641421 3.0448943  1.65839884
 0.97305346 0.43108744 0.16779391 2.09495121 1.72076417 0.48475438
 1.9932965  0.44149458 2.35617943 1.73966342 0.93089444 1.43258378
 0.92203514 3.11451862 3.11281849 2.94365735 0.77826897 1.71769913], Cost: 0.1792
Current parameters: [3.19334648 0.55916997 1.37791942 0.47023938 3.03835258 1.64953305
 0.96283705 0.42717219 0.15432392 2.08221644 1.70701324 0.48273218
 1.9934511  0.4266152  2.34900163 1.7358152  0.92838783 1.41531752
 0.9112517  3.10148231 3.10024642 2.9348285  0.7697991  1.70757848], Cost: 0.1452
Current parameters: [3.26577728 1.05165197 1.51498811 0.26080925 3.0448943  1.65839884
 0.97305346 0.43108744 0.16779391 2.09495121 1.72076417 0.48475438
 1.9932965  0.44149458 2.35617943 1.73966342 0.93089444 1.43258378
 0.92203514 3.11451862 3.11281849 2.94365735 0.77826897 1.71769913], Cost: 0.4264
Current parameters: [3.26072204 1.05083125 1.80333561 0.73833935 3.03106855 1.63966123
 0.95146135 0.42281267 0.13932542 2.06803662 1.69170194 0.48048051
 1.99362326 0.4100474  2.34100935 1.73153031 0.92559678 1.39609199
 0.89924461 3.08696671 3.08624776 2.9249978  0.76036812 1.69630939], Cost: 0.9042
Current parameters: [3.26577728 1.05165197 1.38843968 0.47641421 3.24606034 1.50996628
 0.97305346 0.43108744 0.16779391 2.09495121 1.72076417 0.48475438
 1.9932965  0.44149458 2.35617943 1.73966342 0.93089444 1.43258378
 0.92203514 3.11451862 3.11281849 2.94365735 0.77826897 1.71769913], Cost: 0.0809
Current parameters: [3.2611078  1.05089387 1.37154292 0.48349606 3.33672323 2.05471326
 0.95310904 0.42344411 0.14149785 2.07009047 1.69391967 0.48080665
 1.99359832 0.41244713 2.34216697 1.73215094 0.92600104 1.39887667
 0.90098375 3.08906919 3.08827537 2.9264217  0.76173413 1.69794164], Cost: 0.3643
Current parameters: [3.26577728 1.05165197 1.38843968 0.47641421 3.0448943  1.65839884
 1.17224204 0.43108744 0.01671807 2.09495121 1.72076417 0.48475438
 1.9932965  0.44149458 2.35617943 1.73966342 0.93089444 1.43258378
 0.92203514 3.11451862 3.11281849 2.94365735 0.77826897 1.71769913], Cost: 0.4006
Current parameters: [3.25798713 1.05038723 1.36025056 0.48822896 3.04384107 1.65831899
 1.24534392 0.41833597 0.56435459 2.05347567 1.6759791  0.47816833
 1.99380004 0.3930343  2.33280224 1.72713025 0.92273072 1.37634972
 0.8869148  3.07206097 3.07187284 2.91490287 0.75068365 1.68473742], Cost: 0.4129
Current parameters: [3.26577728 1.05165197 1.38843968 0.47641421 3.0448943  1.65839884
 0.97305346 0.43108744 0.16779391 2.27837529 1.55089479 0.48475438
 1.9932965  0.44149458 2.35617943 1.73966342 0.93089444 1.43258378
```



```
 0.92203514 3.11451862 3.11281849 2.94365735 0.77826897 1.71769913], Cost: 0.1372
Current parameters: [3.25579543 1.0500314  1.35231979 0.49155294 3.04354476 1.65829653
 0.951998   0.41474845 0.18815323 2.41265525 2.07260155 0.47631539
 1.9939417  0.37940039 2.32622526 1.72360414 0.92043392 1.36052872
 0.87703398 3.06011586 3.06035311 2.90681303 0.74292275 1.67546392], Cost: 0.5664
Current parameters: [3.26577728 1.05165197 1.38843968 0.47641421 3.0448943  1.65839884
 0.97305346 0.43108744 0.16779391 2.09495121 1.72076417 0.2370317
 1.9932965  0.47516164 2.35617943 1.73966342 0.93089444 1.43258378
 0.92203514 3.11451862 3.11281849 2.94365735 0.77826897 1.71769913], Cost: 0.1002
Current parameters: [3.25665207 1.05017048 1.35541959 0.49025374 3.04366057 1.65830531
 0.95380497 0.41615066 0.186406   2.09191639 1.72551569 0.55210465
 1.99388633 0.91855993 2.32879592 1.72498235 0.92133164 1.36671248
 0.88089598 3.0647847  3.06485569 2.90997501 0.74595616 1.67908854], Cost: 2.4929
Current parameters: [3.26377826 1.05132743 1.38120612 0.47944597 3.04462403 1.65837835
 0.96883678 0.4278153  0.17187118 2.09428638 1.72180507 0.48316789
 1.99342571 0.4257687  2.60402709 1.7364473  0.92879956 1.41815365
 0.91302297 3.10362363 3.10231149 2.93627871 0.77119034 1.70924088], Cost: 0.1687
Current parameters: [3.26577728 1.05165197 1.38843968 0.47641421 3.0448943  1.65839884
 0.97305346 0.43108744 0.16779391 2.09495121 1.72076417 0.48475438
 2.11819573 0.44149458 2.35617943 1.74468144 0.93089444 1.43258378
 0.92203514 3.11451862 3.11281849 2.94365735 0.77826897 1.71769913], Cost: 0.0355
Current parameters: [3.26469089 1.0514756  1.38450854 0.47806185 3.04474742 1.6583877
 0.97076187 0.42930917 0.17000974 2.0945899  1.72132986 0.48389219
 2.10882767 0.43294821 2.35613289 1.9938778  0.92975596 1.42474159
 0.91713739 3.10859764 3.10710837 2.93964736 0.77442203 1.71310241], Cost: 0.2386
Current parameters: [3.26593007 1.05272549 1.38653651 0.47529715 3.04371086 1.65679497
 0.97120526 0.42854099 0.16535711 2.09264742 1.71827655 0.48438855
 2.1182237  0.43880282 2.35488093 1.74398528 0.80608219 1.43258378
 0.92203514 3.11451862 3.11281849 2.94365735 0.77826897 1.71769913], Cost: 0.0820
Current parameters: [3.26078131 1.07674824 1.30968673 0.46211985 3.01447286 1.61868675
 0.90889483 0.35382342 0.12548367 2.03512127 1.66387656 0.46875634
 2.12454358 0.30600089 2.32372533 1.71604826 0.94946777 1.36927269
 0.88249492 3.0667177  3.06671985 2.91128413 0.74721205 1.68058921], Cost: 0.7533
Current parameters: [3.26577728 1.05165197 1.38843968 0.47641421 3.0448943  1.65839884
 0.97305346 0.43108744 0.16779391 2.09495121 1.72076417 0.48475438
 2.11819573 0.44149458 2.35617943 1.74468144 0.93089444 1.49879846
 0.81601337 3.11451862 3.11281849 2.94365735 0.77826897 1.71769913], Cost: 0.1550
Current parameters: [3.25981756 1.04747745 1.37438473 0.48816024 3.04777066 1.66324719
 0.96711957 0.42988401 0.18644832 2.10016955 1.73123128 0.48151406
 2.12165278 0.40651774 2.35991497 1.7396492  0.93556287 1.62907535
 1.0583568  3.08458365 3.08394956 2.92338387 0.75881981 1.69445932], Cost: 0.6354
```



```
Current parameters: [3.26577728 1.05165197 1.38843968 0.47641421 3.0448943  1.65839884
 0.97305346 0.43108744 0.16779391 2.09495121 1.72076417 0.48475438
 2.11819573 0.44149458 2.35617943 1.74468144 0.93089444 1.43258378
 0.92203514 3.20129034 3.02284256 2.94365735 0.77826897 1.71769913], Cost: 0.0466
Current parameters: [3.25958893 1.0473173  1.37384555 0.48861084 3.04788101 1.66343318
 0.96689194 0.42983785 0.18716394 2.10036974 1.73163283 0.48138975
 2.1217854  0.40517595 2.36005827 1.73945615 0.93574196 1.42156854
 0.92928272 3.28779236 3.2814738  2.92260613 0.7580737  1.69356779], Cost: 0.8628
Current parameters: [3.26577728 1.05165197 1.38843968 0.47641421 3.0448943  1.65839884
 0.97305346 0.43108744 0.16779391 2.09495121 1.72076417 0.48475438
 2.11819573 0.44149458 2.35617943 1.74468144 0.93089444 1.43258378
 0.92203514 3.11451862 3.11281849 3.03019283 0.68806581 1.71769913], Cost: 0.0233
Current parameters: [3.26140846 1.04859181 1.3781366  0.48502471 3.04700284 1.66195296
 0.96870358 0.43020526 0.18146865 2.09877655 1.72843716 0.48237904
 2.12072995 0.41585456 2.35891779 1.74099252 0.93431666 1.42480731
 0.92715175 3.1069131  3.1065796  3.2087514  0.85816833 1.70066303], Cost: 0.8556
Current parameters: [3.28321016 1.1741355  1.3911205  0.4779877  3.04656129 1.66065806
 0.97565685 0.43075356 0.1712264  2.09819633 1.72426824 0.48526969
 2.11815634 0.44528621 2.35800851 1.74566206 0.93153318 1.43698364
 0.92478302 3.11784058 3.11602215 3.03244264 0.69022414 1.73121087], Cost: 0.0526
Current parameters: [3.25869389 1.02487814 1.37933662 0.48326743 3.046319   1.6609092
 0.96891914 0.43041945 0.17849318 2.09749223 1.72645545 0.48267602
 2.12031215 0.41941053 2.35809612 1.74141807 0.93361559 1.42524297
 0.92574893 3.10753327 3.10699366 3.02133057 0.67856889 1.96384257], Cost: 0.3474
Current parameters: [3.26529847 1.04828785 1.3915582  0.47824461 3.04683346 1.66102692
 0.97608191 0.31548812 0.17178683 2.09872617 1.72484036 0.49081515
 2.11813873 0.48608979 2.35882582 1.74610025 0.9318186  1.4389497
 0.9260109  3.11932499 3.1174537  3.03344795 0.69118858 1.72143052], Cost: 0.0541
Current parameters: [3.25647259 1.05189063 1.34040355 0.48302137 3.03524188 1.64697085
 0.93983275 0.50553705 0.17007795 2.07549758 1.70816889 0.5154673
 2.12447546 0.64810129 2.35047991 1.72938156 0.93475889 1.38477034
 0.91585319 3.07430133 3.07663511 2.99116666 0.64778723 1.70029683], Cost: 0.6548
Current parameters: [3.2658328  1.05204205 1.45020518 0.51266721 3.04289168 1.65568476
 0.96992591 0.43322553 0.16367034 2.02677498 1.64714784 0.4858587
 2.1181113  0.44962014 2.36009919 1.74678294 0.93226328 1.4420128
 0.92792393 3.12163768 3.11968403 3.03501423 0.69269117 1.72322597], Cost: 0.0501
Current parameters: [3.2607476  1.04958811 1.54761366 0.58748981 3.04011431 1.65275928
 0.95638587 0.42512781 0.16918916 2.17661665 1.81321141 0.48228565
 2.12159681 0.41350169 2.3432413  1.73153551 0.92934539 1.38416615
 0.90380505 3.07587447 3.07686993 2.99804248 0.65578283 1.69470663], Cost: 0.5409
Current parameters: [3.26578847 1.05173061 1.38897404 0.47672785 3.10225465 1.73613755
```

```
 0.92519895 0.43151846 0.10469931 2.09635317 1.72227801 0.484977
 2.11817871 0.44313265 2.35696963 1.74510509 0.93117039 1.43448461
 0.92322229 3.11595378 3.11420254 3.0311648  0.68899825 1.71881331], Cost: 0.0078
Current parameters: [3.26332211 1.0501574  1.37340286 0.4759452  3.19213387 1.85834514
 1.02820049 0.42542989 0.25156953 2.06986552 1.69569638 0.48212083
 2.11991811 0.41746341 2.34485463 1.73566975 0.92834551 1.39715282
 0.90559317 3.08677898 3.08669144 3.00857531 0.66664549 1.69948221], Cost: 0.2802
Current parameters: [3.2657724  1.05161772 1.38965576 0.47712798 3.10245095 1.73640359
 0.92545022 0.43222765 0.10503059 2.18781845 1.63825971 0.48523492
 2.12429942 0.44503039 2.35757559 1.74266311 0.93710558 1.42974937
 0.92735989 3.11066391 3.11746127 3.02769355 0.6940073  1.71960969], Cost: 0.0180
Current parameters: [3.26075743 1.05328766 1.36193521 0.48043226 3.09631407 1.72901657
 0.90390558 0.42198784 0.10254554 2.1073805  1.69440917 0.48268924
 2.01093008 0.41539905 2.34981252 1.78439107 0.82851228 1.51002084
 0.85547752 3.20166145 3.04721307 3.08056748 0.58406761 1.70450241], Cost: 0.1902
Current parameters: [3.26243805 1.02819054 1.42314833 0.36998123 3.08995594 1.71946949
 0.93457405 0.45252984 0.11706007 2.10492784 1.73063989 0.48263204
 2.12238774 0.42587838 2.35703419 1.74324044 0.93512178 1.4303889
 0.92553455 3.11168443 3.11582395 3.03760616 0.66450006 1.70406269], Cost: 0.1034
Current parameters: [3.27130233 1.14226174 1.51506665 0.42603642 3.14597657 1.79669777
 0.86490156 0.35335338 0.06157504 2.05927175 1.68475076 0.49678953
 2.09290598 0.51475263 2.36068481 1.75098848 0.90904632 1.4468451
 0.91487083 3.13043737 3.08926276 2.99535763 0.75220013 1.77413533], Cost: 0.4966
Current parameters: [3.26430227 1.05386759 1.38742806 0.47830838 3.10250239 1.73679032
 1.02192225 0.43025253 0.02617735 2.09457781 1.72021492 0.48586854
 2.1155899  0.44597728 2.35792642 1.7449616  0.92912907 1.43403283
 0.92314381 3.11587123 3.10951247 3.0278999  0.68368947 1.71981079], Cost: 0.0665
Current parameters: [3.26578219 1.05168651 1.38843963 0.47695183 3.10224689 1.73612703
 0.92488743 0.43127672 0.10500053 2.09630405 1.72235491 0.54398475
 2.11818826 0.43393539 2.37528033 1.74486749 0.93101562 1.43341853
 0.92255648 3.11514887 3.1134263  3.03061967 0.68847529 1.71818842], Cost: 0.0097
Current parameters: [3.2652672  1.05213793 1.38503176 0.47868188 3.10228481 1.73628099
 0.92299921 0.42955184 0.10511608 2.09546242 1.72210583 0.44746885
 2.11740262 0.44160402 2.47521764 1.74352828 0.9295131  1.42747186
 0.91890845 3.11074268 3.10768537 3.02659735 0.6839123  1.71511117], Cost: 0.0769
Current parameters: [3.27232261 1.05080062 1.38897404 0.47672785 3.05224434 1.77303821
 0.92519895 0.43151846 0.10469931 2.09635317 1.72227801 0.484977
 2.11817871 0.44313265 2.35696963 1.74510509 0.93117039 1.43448461
 0.92322229 3.11595378 3.11420254 3.0311648  0.68899825 1.71881331], Cost: 0.0303
Current parameters: [3.3888324  1.03487723 1.38846662 0.47725899 3.11340449 1.72819648
 0.92500696 0.43109544 0.10307979 2.09574878 1.72157052 0.48516373
```



```
 2.11729428 0.44416531 2.35659309 1.74506522 0.93047923 1.43437128
 0.92322106 3.1159565  3.11263072 3.03007079 0.68720542 1.71917794], Cost: 0.0377
Current parameters: [3.26572311 1.05010725 1.39053078 0.4770241  3.10292772 1.73702039
 0.92744648 0.43458532 0.10684494 2.09865255 1.72461163 0.48532435
 2.12046773 0.44603223 2.35805064 1.80653545 0.935229   1.43067539
 0.92644736 3.11172337 3.11672627 3.02866792 0.69208766 1.71895377], Cost: 0.0371
Current parameters: [3.26450631 1.06450166 1.34927427 0.47123469 3.09000722 1.71586551
 0.89611889 0.39592457 0.07754657 2.06656145 1.69312928 0.47849539
 2.12680094 0.38612949 2.34001161 1.7475303  0.94597593 1.39292847
 0.91314084 3.08127095 3.0911262  3.00318383 0.67360805 1.70319661], Cost: 0.2162
Current parameters: [3.26545167 1.05125053 1.38806379 0.47719409 3.10229198 1.73623567
 0.92503871 0.43171088 0.10581292 2.0967456  1.72294355 0.48478599
 2.11983552 0.44117005 2.35716453 1.7449172  0.93282282 1.47342565
 0.94948537 3.08532078 3.08694983 3.02906767 0.68888794 1.71745035], Cost: 0.0207
Current parameters: [3.26283294 1.05051311 1.37261406 0.48842695 3.10808998 1.73716008
 0.92170608 0.43102732 0.1066439  2.09713065 1.72602243 0.48409348
 2.13184306 0.42582772 2.35679983 1.73915382 0.94625548 1.47909019
 0.97668135 3.17014036 3.18206759 2.99341311 0.67070615 1.70315483], Cost: 0.3882
Current parameters: [3.26554354 1.05300238 1.35139694 0.45625944 3.10235587 1.73635014
 0.92351199 0.44180201 0.10457702 2.07761598 1.7024293  0.48967106
 2.11828087 0.47678771 2.35882821 1.7455375  0.93185083 1.43782789
 0.9254511  3.117948   3.11629167 3.03369527 0.69287361 1.72270397], Cost: 0.0245
Current parameters: [3.25963872 1.03905808 1.40480405 0.51225876 3.09246567 1.71686241
 0.91398109 0.47117783 0.10476044 2.11994053 1.75202838 0.49604056
 2.12032396 0.51089863 2.34706609 1.73159097 0.93058961 1.39981841
 0.91244854 3.08990201 3.0885212  3.01123695 0.64737948 1.69599944], Cost: 0.2353
Current parameters: [3.26409201 1.04266709 1.38571985 0.47633254 3.1006012  1.73396865
 0.92604367 0.43493395 0.10781884 2.09846068 1.72491976 0.48446982
 2.11925873 0.43855732 2.3569025  1.74456875 0.93211571 1.43272369
 0.92336945 3.11384084 3.11363415 3.01571049 0.67032324 1.7749423 ], Cost: 0.0196
Current parameters: [3.26052651 1.03030553 1.3735357  0.48916648 3.09943277 1.72375957
 0.9273919  0.44726901 0.10980458 2.10912239 1.73854274 0.48381381
 2.11708285 0.42388392 2.35320136 1.7368198  0.93085309 1.42185244
 0.92551327 3.11171215 3.10445771 3.12294067 0.74952004 1.75134032], Cost: 0.2793
Current parameters: [3.26710886 1.07391569 1.4217151  0.46528964 3.11431251 1.74983305
 0.91132133 0.41274832 0.09257676 2.08810151 1.71412802 0.48831308
 2.11150945 0.46184132 2.35653733 1.74414017 0.92519716 1.43588138
 0.92013298 3.11926247 3.10686231 3.02514961 0.70629895 1.73281143], Cost: 0.0338
Current parameters: [3.26545649 1.05201862 1.38733737 0.47715716 3.09530526 1.7267853
 0.91602932 0.43094941 0.09445002 2.0993621  1.72124504 0.48494625
 2.10735092 0.44213235 2.35708889 1.74971088 0.92103433 1.44492868
```



```
 0.91720729 3.12643317 3.10953511 3.03754953 0.68014798 1.71871508], Cost: 0.0027
Current parameters: [3.26428853 1.05371536 1.38266378 0.47937905 3.11513181 1.75165192
 0.93397018 0.4269496  0.12286719 2.09535484 1.71154094 0.48455268
 2.09048815 0.43477545 2.35249098 1.7520417  0.90390848 1.44769444
 0.90216799 3.13353767 3.09218402 3.04180282 0.66217399 1.7139056 ], Cost: 0.0283
Current parameters: [3.26514899 1.04985817 1.38632448 0.47656265 3.0943881  1.7255423
 0.91697319 0.43312958 0.09569448 2.1005944  1.72257566 0.48484123
 2.11814929 0.44135965 2.35704233 1.75011972 0.93176872 1.44737225
 0.91244441 3.14344554 3.09131728 3.03136949 0.68619893 1.71830213], Cost: 0.0032
Current parameters: [3.26516219 1.05075667 1.38752196 0.47753979 3.09608437 1.72666133
 0.91705547 0.43243828 0.09520105 2.10039527 1.7224065  0.48510464
 2.11706456 0.44224162 2.3564201  1.74938061 0.93082556 1.47326508
 0.86949907 3.10932353 3.12584152 3.03248809 0.68673551 1.71886824], Cost: 0.0175
Current parameters: [3.26582494 1.05482541 1.38862788 0.47790362 3.09647312 1.72837355
 0.9165052  0.42813046 0.09150577 2.09775417 1.71949759 0.48509585
 2.13228025 0.44316873 2.35716536 1.75074291 0.90719762 1.44541002
 0.91751383 3.12680049 3.10981226 3.04542971 0.67227797 1.71925721], Cost: 0.0030
Current parameters: [3.26464133 1.0531187  1.38769079 0.47724931 3.09605057 1.72709435
 0.96461394 0.43038118 0.0558373  2.09876302 1.72031029 0.48553654
 2.10194551 0.44386401 2.35710303 1.74893469 0.92125188 1.44468692
 0.91590403 3.12634732 3.10648824 3.03538304 0.67903564 1.7193736 ], Cost: 0.0211
Current parameters: [3.26632531 1.05812296 1.38933791 0.47833137 3.09726726 1.72944433
 0.91315533 0.45509846 0.09066075 2.09564032 1.71722628 0.48372474
 2.10735904 0.43314448 2.35671196 1.7495088  0.91092719 1.44427252
 0.91679749 3.12593776 3.10905734 3.03027252 0.6870618  1.71880283], Cost: 0.0074
Current parameters: [3.26293552 1.0345955  1.37834205 0.4719786  3.08768198 1.71601971
 0.92280619 0.41388011 0.10327964 2.10822947 1.7308372  0.48549794
 2.10703572 0.44580544 2.35590484 1.74892986 0.88406542 1.44359859
 0.91570823 3.12542123 3.10817883 3.01186971 0.70612963 1.71654535], Cost: 0.0094
Current parameters: [3.26247499 1.03107061 1.39808465 0.48346524 3.0940857  1.72513247
 0.91549033 0.43139056 0.09373937 2.08641492 1.70726474 0.48505608
 2.10734252 0.44294051 2.35747875 1.7499199  0.92117047 1.44586649
 0.91779298 3.12714123 3.11021795 3.03802906 0.68060802 1.71738872], Cost: 0.0021
Current parameters: [3.26633493 1.05852343 1.41048092 0.49085766 3.07127288 1.69371543
 0.93508665 0.43110134 0.11945509 2.07376997 1.69363061 0.48540289
 2.10693852 0.44504771 2.35802833 1.75004623 0.92105322 1.44788685
 0.91828547 3.12865777 3.11124267 3.0393506  0.68196007 1.72118834], Cost: 0.0100
Current parameters: [3.26276496 1.0346615  1.39919376 0.48374587 3.09587015 1.72759002
 0.91499813 0.42870975 0.09335996 2.08472206 1.70546916 0.48528609
 2.10918625 0.44417401 2.35777744 1.75010368 0.92298974 1.4545459
 0.92459011 3.13578843 3.12137567 3.01793896 0.66846988 1.70969237], Cost: 0.0119
```



```
Current parameters: [3.25799828 1.02609391 1.39293131 0.49499656 3.1017231  1.7256678
 0.91713706 0.43119167 0.09877704 2.09168791 1.7145853  0.485515
 2.10757836 0.43135067 2.35371572 1.74244001 0.9225738  1.46589486
 0.93539089 3.15305226 3.13399909 3.06508365 0.70375848 1.72794629], Cost: 0.1777
Current parameters: [3.26239029 1.03047554 1.39881983 0.48054002 3.09371134 1.7246251
 0.91567604 0.43177047 0.09398422 2.06390413 1.72837258 0.48492989
 2.10593236 0.442012   2.35732982 1.7504776  0.91979962 1.44693522
 0.91682561 3.12834325 3.10945077 3.03906251 0.67871429 1.71680028], Cost: 0.0022
Current parameters: [3.26048316 1.01975385 1.41690643 0.43029153 3.08980689 1.71730057
 0.9207885  0.44095978 0.1005751  2.09592811 1.70717222 0.48422664
 2.10883213 0.43461455 2.35657319 1.74747839 0.92255043 1.44519987
 0.91848425 3.12666089 3.11194726 3.04325608 0.67049405 1.71096103], Cost: 0.0150
Current parameters: [3.2627397  1.03200101 1.39630499 0.48159817 3.0950106  1.72636252
 0.91580646 0.42828621 0.09389833 2.08473641 1.70533961 0.47539121
 2.10697008 0.43802918 2.38625172 1.75009882 0.92074371 1.44658884
 0.91743823 3.12786619 3.11052632 3.03838948 0.68223832 1.71809282], Cost: 0.0038
Current parameters: [3.25819981 1.02484675 1.4107858  0.49760609 3.09192155 1.71642537
 0.91074085 0.44984279 0.09587521 2.09975761 1.72264642 0.48498361
 2.10605705 0.47824292 2.37119259 1.740981   0.91857524 1.43049276
 0.90976695 3.11635944 3.09674268 3.03273897 0.6618331  1.70627471], Cost: 0.0569
Current parameters: [3.23202801 1.03568103 1.39681648 0.48314133 3.09092938 1.72636235
 0.91451901 0.43024869 0.0931883  2.08552053 1.70641849 0.48477983
 2.10786846 0.44068608 2.35697641 1.7501269  0.92186939 1.44442591
 0.91744459 3.12591296 3.10979922 3.03731062 0.68051525 1.71674963], Cost: 0.0073
Current parameters: [3.27264861 1.03552162 1.38383105 0.47953015 3.09090608 1.71191669
 0.8993813  0.41311756 0.08049167 2.073825   1.69428814 0.48175618
 2.10971158 0.41774836 2.34949576 1.74724058 0.92698931 1.42194255
 0.90830027 3.1091581  3.09548391 3.02637349 0.67109598 1.70746295], Cost: 0.0693
Current parameters: [3.26383839 1.04375642 1.40421536 0.47779213 3.09389578 1.72495336
 0.90304979 0.41942527 0.08886673 2.08989741 1.71029558 0.48637595
 2.09124235 0.45111403 2.35789366 1.7510335  0.92380679 1.44643034
 0.92152773 3.12666635 3.11252618 3.04219337 0.68002744 1.72261561], Cost: 0.0079
Current parameters: [3.2628168  1.0383107  1.42998226 0.4771194  3.10878943 1.74255124
 0.91708279 0.42673874 0.08619396 2.07040463 1.69185445 0.48727887
 2.12450405 0.45452679 2.35632829 1.74781592 0.90925562 1.44563593
 0.90679339 3.13055339 3.09532296 3.02379695 0.70241792 1.72673068], Cost: 0.0361
Current parameters: [3.2626956  1.02988798 1.39922675 0.48355171 3.0947764  1.72491191
 0.91650596 0.43295869 0.09486636 2.08771889 1.70849271 0.48512704
 2.10860947 0.44430981 2.35798886 1.78058776 0.92307299 1.44317213
 0.91863925 3.12466889 3.11087873 3.03689136 0.68176825 1.71687838], Cost: 0.0075
Current parameters: [3.26261385 1.03106206 1.39821778 0.48340944 3.09303679 1.72591047
```

```
 0.91556793 0.43145078 0.09366433 2.08642715 1.70724558 0.47035635
 2.10734014 0.44523169 2.35291727 1.74997909 0.92120903 1.44613206
 0.91795885 3.12734174 3.11041133 3.03816486 0.68073829 1.71754439], Cost: 0.0034
Current parameters: [3.26598107 1.03029295 1.39836001 0.48342783 3.06907742 1.74327317
 0.91540356 0.43142563 0.0938087  2.08654934 1.70733075 0.48760638
 2.10745412 0.44248359 2.35827707 1.75023567 0.92108655 1.44516684
 0.91717158 3.12679559 3.10971915 3.03807824 0.68029653 1.71689889], Cost: 0.0085
Current parameters: [3.26250432 1.03135973 1.39253167 0.48024997 3.09409375 1.72514547
 0.91526468 0.43292145 0.09350017 2.08347841 1.70410455 0.4858101
 2.10702999 0.44846405 2.35772372 1.75002308 0.92094704 1.43866454
 0.91293179 3.13351218 3.11593394 3.03883047 0.68122114 1.71825226], Cost: 0.0041
Current parameters: [3.26252939 1.03112974 1.38306045 0.47538266 3.09390711 1.72512235
 0.91469582 0.43560488 0.09409143 2.0791757  1.69959618 0.48695461
 2.10800534 0.4557153  2.35831439 1.75034387 0.92188379 1.45871453
 0.92630343 3.11809652 3.10211775 3.03845569 0.68184767 1.71807866], Cost: 0.0119

   Return from subroutine COBYLA because the MAXFUN limit has been reached.

   NFVALS =  100   F = 2.090843E-03    MAXCV = 0.000000E+00
   X = 3.262475E+00   1.031071E+00   1.398085E+00   4.834652E-01   3.094086E+00
       1.725132E+00   9.154903E-01   4.313906E-01   9.373937E-02   2.086415E+00
       1.707265E+00   4.850561E-01   2.107343E+00   4.429405E-01   2.357479E+00
       1.749920E+00   9.211705E-01   1.445866E+00   9.177930E-01   3.127141E+00
       3.110218E+00   3.038029E+00   6.806080E-01   1.717389E+00
Current parameters: [3.26247499 1.03107061 1.39808465 0.48346524 3.0940857  1.72513247
 0.91549033 0.43139056 0.09373937 2.08641492 1.70726474 0.48505608
 2.10734252 0.44294051 2.35747875 1.7499199  0.92117047 1.44586649
 0.91779298 3.12714123 3.11021795 3.03802906 0.68060802 1.71738872], Cost: 0.0021
```


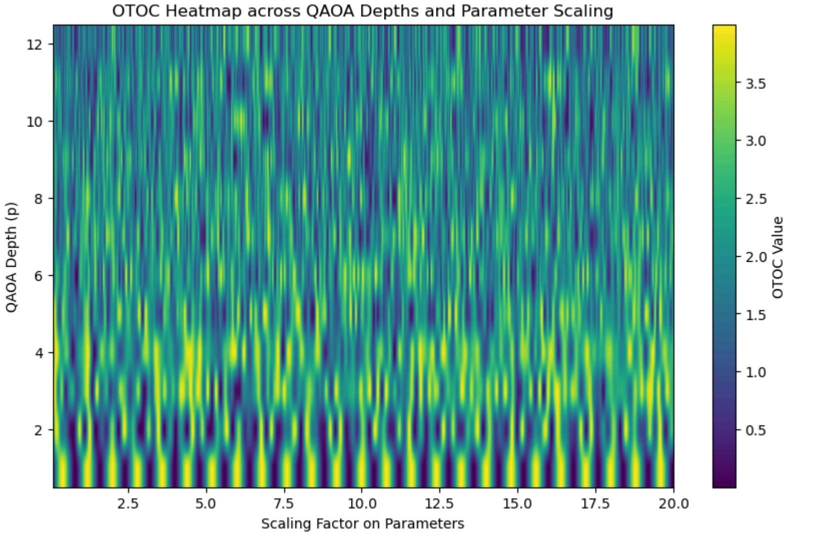


```
Run 7/30 for heatmap generation...
Simulating for QAOA depth p = 1
Current parameters: [3.08381259 0.00952254], Cost: 1.9956
Current parameters: [2.08381259 1.00952254], Cost: 2.6681
Current parameters: [ 2.04362791 -0.98966973], Cost: 1.4087
Current parameters: [ 1.99319517 -1.98839719], Cost: 2.7441
Current parameters: [ 2.01841154 -1.48903346], Cost: 1.7493
Current parameters: [ 1.79394604 -0.97706155], Cost: 1.7006
Current parameters: [ 2.48764225 -0.7597757 ], Cost: 2.0988
Current parameters: [ 1.81140763 -0.89707523], Cost: 1.8000
Current parameters: [ 2.16318981 -0.95320124], Cost: 1.4238
Current parameters: [ 2.0746266  -1.11076507], Cost: 1.1849
Current parameters: [ 2.10295512 -1.23251274], Cost: 1.1462
Current parameters: [ 1.98869416 -1.28320233], Cost: 1.3228
Current parameters: [ 2.22791719 -1.22943385], Cost: 1.0531
Current parameters: [ 2.3506725  -1.25301635], Cost: 1.0446
Current parameters: [ 2.34190784 -1.31489875], Cost: 1.1464
Current parameters: [ 2.36498488 -1.19217717], Cost: 1.0017
Current parameters: [ 2.38273884 -1.13225183], Cost: 1.0182
Current parameters: [ 2.39540447 -1.19933336], Cost: 1.0067
Current parameters: [ 2.36530709 -1.129678  ], Cost: 1.0189
Current parameters: [ 2.36514598 -1.16092759], Cost: 1.0025
Current parameters: [ 2.34951777 -1.19439285], Cost: 1.0022
Current parameters: [ 2.36433733 -1.18439155], Cost: 1.0004
Current parameters: [ 2.36465389 -1.17658547], Cost: 1.0002
Current parameters: [ 2.37204815 -1.17406353], Cost: 1.0006
Current parameters: [ 2.35771507 -1.1729955 ], Cost: 1.0002
Current parameters: [ 2.36118448 -1.17479048], Cost: 1.0001
Current parameters: [ 2.36179058 -1.17293378], Cost: 1.0003
Current parameters: [ 2.36014379 -1.17644325], Cost: 1.0001
Current parameters: [ 2.35900738 -1.17803173], Cost: 1.0000
Current parameters: [ 2.35711364 -1.17850971], Cost: 1.0000
Current parameters: [ 2.35702449 -1.1804608 ], Cost: 1.0000
Current parameters: [ 2.35664208 -1.17765455], Cost: 1.0000
Current parameters: [ 2.35618091 -1.17781499], Cost: 1.0000
Current parameters: [ 2.35569495 -1.17786259], Cost: 1.0000
Current parameters: [ 2.35610069 -1.1775844 ], Cost: 1.0000
Current parameters: [ 2.35613862 -1.17830143], Cost: 1.0000
Current parameters: [ 2.35638064 -1.17833354], Cost: 1.0000
Current parameters: [ 2.35593659 -1.17843851], Cost: 1.0000
Current parameters: [ 2.3561382  -1.17820143], Cost: 1.0000
```



```
Current parameters: [ 2.35612214 -1.17810273], Cost: 1.0000
Current parameters: [ 2.35603021 -1.17806338], Cost: 1.0000
Current parameters: [ 2.35619284 -1.17803201], Cost: 1.0000

   Normal return from subroutine COBYLA

Current parameters: [ 2.35619284 -1.17803201], Cost: 1.0000
   NFVALS =   43   F = 1.000000E+00    MAXCV = 0.000000E+00
   X = 2.356193E+00  -1.178032E+00
Simulating for QAOA depth p = 2
Current parameters: [1.72400772 0.45163847 0.78936708 0.5305481 ], Cost: 2.8082
Current parameters: [0.72400772 1.45163847 0.78936708 0.5305481 ], Cost: 3.0045
Current parameters: [0.72400772 0.45163847 1.78936708 0.5305481 ], Cost: 2.7478
Current parameters: [0.72400772 0.45163847 0.78936708 1.5305481 ], Cost: 1.9569
Current parameters: [ 0.19535378 -0.23253503  0.30857804  1.67640263], Cost: 2.1996
Current parameters: [1.1727159  0.27383834 0.66442165 1.56845216], Cost: 2.3331
Current parameters: [0.72400772 0.59537854 0.58482176 1.5305481 ], Cost: 2.6231
Current parameters: [0.62868688 0.27467256 1.24600535 1.56338858], Cost: 1.0688
Current parameters: [ 0.95112917 -0.0564445   1.4352766   1.5872394 ], Cost: 1.5679
Current parameters: [0.55388946 0.17787121 1.17798017 1.77052964], Cost: 1.5186
Current parameters: [0.35916172 0.01145361 1.18500516 1.2403546 ], Cost: 1.7987
Current parameters: [0.57114054 0.61330323 1.60906189 1.54897893], Cost: 2.0777
Current parameters: [0.51407001 0.20679908 1.22956601 1.77430541], Cost: 1.7072
Current parameters: [0.6956905  0.3185385  1.15306079 1.53945945], Cost: 0.9890
Current parameters: [0.72341129 0.41966641 1.20499088 1.4954939 ], Cost: 0.9011
Current parameters: [0.69136746 0.4164304  1.18299567 1.37673392], Cost: 0.8544
Current parameters: [0.73771281 0.38510846 1.20576404 1.36084673], Cost: 0.8723
Current parameters: [0.71080789 0.52166855 1.14675912 1.32326511], Cost: 0.7857
Current parameters: [0.67120727 0.61286686 1.19377855 1.26386113], Cost: 0.6771
Current parameters: [0.63129142 0.70253915 1.24536214 1.20615724], Cost: 0.6730
Current parameters: [0.60756746 0.67490646 1.22366625 1.1602318 ], Cost: 0.7095
Current parameters: [0.69580078 0.78146699 1.17355984 1.21500989], Cost: 0.4987
Current parameters: [0.70445268 0.86457227 1.09217737 1.25996043], Cost: 0.3471
Current parameters: [0.76976625 0.94697933 1.02713695 1.27834406], Cost: 0.2617
Current parameters: [0.69480361 0.99229786 0.9731676  1.34933098], Cost: 0.4536
Current parameters: [0.74850735 0.99019268 1.05166945 1.2469582 ], Cost: 0.1910
Current parameters: [0.72953432 0.97272171 0.97244884 1.15377084], Cost: 0.1689
Current parameters: [0.80401314 1.02357915 1.01567678 1.07878569], Cost: 0.0869
Current parameters: [0.78792744 1.14101166 1.01410941 1.03911959], Cost: 0.1013
Current parameters: [0.76336073 0.98844856 1.10279661 1.00703362], Cost: 0.2693
Current parameters: [0.85791328 1.02909824 0.9852468  1.08546252], Cost: 0.0717
```

```
Current parameters: [0.89724928 1.01216914 1.00646412 1.04518648], Cost: 0.0901
Current parameters: [0.84828226 1.02140911 0.96284933 1.06748931], Cost: 0.1341
Current parameters: [0.87855748 1.04408676 1.01935992 1.1311977 ], Cost: 0.0228
Current parameters: [0.89909806 1.0568343  1.0331472  1.1871597 ], Cost: 0.0577
Current parameters: [0.88407453 1.01396464 1.02445474 1.13477898], Cost: 0.0413
Current parameters: [0.90784138 1.06111132 1.05977409 1.09764829], Cost: 0.0324
Current parameters: [0.85331437 1.04535595 1.03764455 1.13304427], Cost: 0.0333
Current parameters: [0.88653999 1.04829773 1.02894463 1.12278217], Cost: 0.0205
Current parameters: [0.88392414 1.04655922 1.02611757 1.11621118], Cost: 0.0209
Current parameters: [0.89176362 1.0615468  1.02272197 1.12117127], Cost: 0.0127
Current parameters: [0.90020539 1.07292228 1.01639997 1.11929834], Cost: 0.0072
Current parameters: [0.91332765 1.07695891 1.0218215  1.12442276], Cost: 0.0067
Current parameters: [0.91172192 1.08041419 1.02715429 1.1201708 ], Cost: 0.0067
Current parameters: [0.91024467 1.09016631 1.01669373 1.1302457 ], Cost: 0.0042
Current parameters: [0.91613725 1.09837098 1.0070935  1.12317908], Cost: 0.0020
Current parameters: [0.91863493 1.11156317 0.99916232 1.12416131], Cost: 0.0028
Current parameters: [0.91738609 1.10496707 1.00312791 1.12367019], Cost: 0.0021
Current parameters: [0.91933957 1.09912166 1.0086785  1.12456767], Cost: 0.0020
Current parameters: [0.91995477 1.10560715 1.00437171 1.12478372], Cost: 0.0020
Current parameters: [0.92017749 1.09724298 1.00607237 1.12662566], Cost: 0.0028
Current parameters: [0.92121852 1.10045604 1.01066421 1.11737176], Cost: 0.0012
Current parameters: [0.91840376 1.10451341 1.01636549 1.11533571], Cost: 0.0008
Current parameters: [0.91970786 1.10638196 1.01924039 1.10843803], Cost: 0.0011
Current parameters: [0.92037946 1.10407588 1.01911304 1.11723702], Cost: 0.0013
Current parameters: [0.91926011 1.10612576 1.01576529 1.11568423], Cost: 0.0007
Current parameters: [0.9163514  1.10830227 1.0143875  1.1160876 ], Cost: 0.0004
Current parameters: [0.9163122  1.10845673 1.01051118 1.11563199], Cost: 0.0005
Current parameters: [0.91601019 1.11021285 1.01501568 1.11275632], Cost: 0.0004
Current parameters: [0.91540686 1.11182731 1.01526423 1.11364088], Cost: 0.0003
Current parameters: [0.91347786 1.11182837 1.01555237 1.11374407], Cost: 0.0003
Current parameters: [0.9136245  1.11169002 1.01650409 1.11382918], Cost: 0.0004
Current parameters: [0.91309848 1.1127746  1.01388981 1.11385052], Cost: 0.0002
Current parameters: [0.91273954 1.11239455 1.01267111 1.11241658], Cost: 0.0003
Current parameters: [0.9130007  1.11199069 1.01358826 1.11433905], Cost: 0.0002
Current parameters: [0.91317096 1.11308116 1.01275623 1.11571902], Cost: 0.0001
Current parameters: [0.91185463 1.11367221 1.01331396 1.11691132], Cost: 0.0001
Current parameters: [0.91281278 1.11418773 1.01377275 1.11846708], Cost: 0.0002
Current parameters: [0.91078516 1.11420497 1.01177718 1.11675195], Cost: 0.0001
Current parameters: [0.91001831 1.11254082 1.01133819 1.11726628], Cost: 0.0001
Current parameters: [0.90961411 1.11510749 1.01223672 1.11556131], Cost: 0.0001
Current parameters: [0.90959708 1.1159676  1.01189566 1.1158732 ], Cost: 0.0001
```



```
Current parameters: [0.91000186 1.11630334 1.01129092 1.11531516], Cost: 0.0001
Current parameters: [0.90928962 1.11580828 1.0115551  1.11592338], Cost: 0.0001
Current parameters: [0.90922735 1.11632234 1.01242166 1.11651698], Cost: 0.0000
Current parameters: [0.90905991 1.11704658 1.01281727 1.11701157], Cost: 0.0000
Current parameters: [0.9087731  1.11712385 1.01298885 1.11666408], Cost: 0.0000
Current parameters: [0.90900198 1.11644571 1.01346268 1.11742717], Cost: 0.0001
Current parameters: [0.90869137 1.11691746 1.01256637 1.11716316], Cost: 0.0000
Current parameters: [0.90837141 1.11750613 1.01193014 1.11747936], Cost: 0.0000
Current parameters: [0.90839678 1.11717001 1.01107529 1.11714877], Cost: 0.0000
Current parameters: [0.90782591 1.11751399 1.01208686 1.11827401], Cost: 0.0000
Current parameters: [0.90746011 1.11766958 1.01219733 1.11801286], Cost: 0.0000
Current parameters: [0.90755197 1.11784738 1.01233282 1.11804744], Cost: 0.0000
Current parameters: [0.90712216 1.11763982 1.0118608  1.11791253], Cost: 0.0000
Current parameters: [0.90676216 1.11735687 1.0118014  1.11775368], Cost: 0.0000
Current parameters: [0.90670684 1.11723968 1.01146087 1.11807875], Cost: 0.0000
Current parameters: [0.9069136  1.11725533 1.01169482 1.1176312 ], Cost: 0.0000
Current parameters: [0.90647802 1.11771111 1.01167165 1.11762976], Cost: 0.0000
Current parameters: [0.90612487 1.11779356 1.01196995 1.11776365], Cost: 0.0000
Current parameters: [0.90626897 1.11816498 1.01212359 1.11800049], Cost: 0.0000
Current parameters: [0.90569778 1.11772868 1.01207854 1.11756362], Cost: 0.0000
Current parameters: [0.90591133 1.11776112 1.01202424 1.11766363], Cost: 0.0000
Current parameters: [0.90617186 1.11783418 1.0120466  1.11769177], Cost: 0.0000
Current parameters: [0.90604708 1.11786575 1.01190411 1.11797343], Cost: 0.0000
Current parameters: [0.90603768 1.11770774 1.01196015 1.11815064], Cost: 0.0000
Current parameters: [0.90607663 1.11779982 1.01180959 1.11794609], Cost: 0.0000
Current parameters: [0.9059958  1.11793398 1.01164256 1.11803078], Cost: 0.0000
Current parameters: [0.90615092 1.11808074 1.01153969 1.11808928], Cost: 0.0000
Current parameters: [0.90578702 1.11796349 1.01154394 1.11795719], Cost: 0.0000
Current parameters: [0.9059958  1.11793398 1.01164256 1.11803078], Cost: 0.0000

   Return from subroutine COBYLA because the MAXFUN limit has been reached.

   NFVALS =  100   F = 3.464816E-06    MAXCV = 0.000000E+00
   X = 9.059958E-01   1.117934E+00   1.011643E+00   1.118031E+00
Simulating for QAOA depth p = 3
Current parameters: [1.81426384 2.79449742 2.22556094 3.10541099 1.59652648 1.47416026], Cost: 1.7507
Current parameters: [1.81426384 3.79449742 2.22556094 3.10541099 1.59652648 1.47416026], Cost: 1.9526
Current parameters: [1.81426384 2.79449742 3.22556094 3.10541099 1.59652648 1.47416026], Cost: 2.0739
Current parameters: [1.81426384 2.79449742 2.22556094 4.10541099 1.59652648 1.47416026], Cost: 2.5693
Current parameters: [1.81426384 2.79449742 2.22556094 3.10541099 2.59652648 1.47416026], Cost: 2.2278
Current parameters: [1.81426384 2.79449742 2.22556094 3.10541099 1.59652648 2.47416026], Cost: 1.6210
```



```
Current parameters: [2.23847197 2.61687022 1.94122017 2.38533194 1.17685587 2.58821774], Cost: 1.7796
Current parameters: [1.35008109 2.75804589 2.16721033 2.95764092 1.51040433 2.49756641], Cost: 2.3747
Current parameters: [1.81426384 3.00652574 2.09310721 3.10541099 1.59652648 2.47416026], Cost: 1.6194
Current parameters: [1.95028836 3.25649296 2.49201625 3.02030367 1.54692493 2.4876409 ], Cost: 2.5214
Current parameters: [1.81426384 3.00652574 2.09310721 3.2312947  1.38053275 2.47416026], Cost: 2.3294
Current parameters: [1.97604135 2.87938543 1.88811962 3.10577663 2.0032036  2.49019313], Cost: 0.6622
Current parameters: [1.70848543 2.78200045 1.73065278 2.88616893 2.31241107 2.50743829], Cost: 1.4533
Current parameters: [1.87666638 2.93566607 1.97821213 3.28144302 2.10558406 2.49019313], Cost: 1.8553
Current parameters: [2.37734573 2.92885761 1.96657032 2.82569963 2.04613992 2.4983254 ], Cost: 0.6347
Current parameters: [2.37734573 2.92885761 1.96657032 2.83144087 2.03628899 2.74806526], Cost: 0.7616
Current parameters: [2.55395874 2.70637256 1.60975352 2.90446825 1.86078289 2.46053269], Cost: 0.8764
Current parameters: [2.33283711 3.12774324 1.85876035 2.78814834 1.95712651 2.49567755], Cost: 1.3477
Current parameters: [2.5775328  2.64445961 2.21350827 2.64180204 2.22101905 2.43779828], Cost: 1.1491
Current parameters: [2.44659646 2.96362791 1.99112299 3.0399768  2.14528548 2.48616051], Cost: 0.9289
Current parameters: [2.40779788 2.93419839 2.03469797 2.85164065 1.94952003 2.4939179 ], Cost: 0.9626
Current parameters: [2.1667802  2.80426504 1.95852955 2.87090868 2.06512416 2.48528946], Cost: 0.2894
Current parameters: [2.15121285 2.65569636 1.97508439 2.74795399 2.22138069 2.46587333], Cost: 0.4013
Current parameters: [2.11931212 2.87670878 2.03692124 2.8480086  2.10320173 2.48731786], Cost: 0.2436
Current parameters: [1.97105166 2.97434477 1.86861721 2.8100949  2.12556359 2.4604422 ], Cost: 0.5226
Current parameters: [2.12642473 2.8793324  2.04005877 2.94899844 2.17619204 2.48173664], Cost: 0.3153
Current parameters: [2.11642105 2.87620174 2.03045332 2.84695501 2.10995672 2.54903545], Cost: 0.2930
Current parameters: [2.07192363 2.83382418 2.1131717  2.86927454 2.03352325 2.46685379], Cost: 0.3370
Current parameters: [2.10097949 2.83536555 2.03997398 2.82430695 2.13874122 2.48214495], Cost: 0.2337
Current parameters: [2.18976722 2.851432   2.0767694  2.75993665 2.16573346 2.44668247], Cost: 0.3712
Current parameters: [2.08747816 2.88048051 2.01679181 2.81489274 2.12601961 2.45213288], Cost: 0.2819
Current parameters: [2.02668438 2.75979466 2.00308351 2.81330216 2.18459696 2.51059694], Cost: 0.3631
Current parameters: [2.08407977 2.8219086  2.08053539 2.83644635 2.10028766 2.46917229], Cost: 0.2578
Current parameters: [2.09026792 2.84915549 2.05720132 2.82900505 2.15729901 2.48504618], Cost: 0.2009
Current parameters: [2.10548392 2.84622613 2.0529751  2.8829403  2.17597786 2.46528838], Cost: 0.1575
Current parameters: [2.13073029 2.85434844 2.06597036 2.87507692 2.1754576  2.45878985], Cost: 0.1444
Current parameters: [2.14179084 2.84460399 2.09224354 2.88287631 2.22939563 2.45344772], Cost: 0.2174
Current parameters: [2.13771705 2.86823318 2.06787174 2.8884568  2.16922232 2.48144922], Cost: 0.1865
Current parameters: [2.13287735 2.86198484 2.05491999 2.87150702 2.18225253 2.45835355], Cost: 0.1532
Current parameters: [2.12089505 2.86896112 2.07268467 2.8790429  2.16587496 2.43612574], Cost: 0.1084
Current parameters: [2.12867144 2.85863369 2.06510838 2.88212117 2.14918939 2.41458159], Cost: 0.0956
Current parameters: [2.11921594 2.86090192 2.07340047 2.857596   2.13652048 2.4074215 ], Cost: 0.1399
Current parameters: [2.12674076 2.85209127 2.0681474  2.87974354 2.16035606 2.40696407], Cost: 0.0836
Current parameters: [2.13024324 2.85580475 2.07869705 2.90367865 2.16849411 2.39281864], Cost: 0.0622
Current parameters: [2.13513491 2.8714695  2.05863686 2.90269584 2.18129827 2.38098962], Cost: 0.0692
Current parameters: [2.11633599 2.85444006 2.0735985  2.90816817 2.16706903 2.39199112], Cost: 0.0599
Current parameters: [2.12248509 2.83132456 2.06763878 2.92456077 2.17705202 2.39271825], Cost: 0.0762
```



```
Current parameters: [2.11140358 2.85569091 2.08307381 2.90657032 2.16660665 2.38077957], Cost: 0.0471
Current parameters: [2.10541108 2.86110533 2.09248926 2.90723986 2.17570206 2.38344214], Cost: 0.0469
Current parameters: [2.10694974 2.864514   2.09362311 2.91347837 2.1730887  2.38332245], Cost: 0.0482
Current parameters: [2.09977555 2.86159771 2.09860263 2.90096712 2.16891108 2.37399222], Cost: 0.0385
Current parameters: [2.09809489 2.85258707 2.1035183  2.90365717 2.17137253 2.36291678], Cost: 0.0286
Current parameters: [2.09760818 2.85769021 2.09894109 2.90211143 2.17679566 2.35006721], Cost: 0.0255
Current parameters: [2.109243   2.85743848 2.10716649 2.89706541 2.17770166 2.34622431], Cost: 0.0264
Current parameters: [2.09463123 2.86040248 2.10360638 2.90245913 2.18136987 2.35148399], Cost: 0.0233
Current parameters: [2.08498278 2.85544183 2.11084793 2.90262899 2.18275593 2.34299576], Cost: 0.0169
Current parameters: [2.07796934 2.86342766 2.11085134 2.8973059  2.1854946  2.33323138], Cost: 0.0178
Current parameters: [2.08429307 2.85194257 2.10878016 2.89731444 2.186413   2.34455151], Cost: 0.0180
Current parameters: [2.08396602 2.85049225 2.11335839 2.91317881 2.19146486 2.33797961], Cost: 0.0123
Current parameters: [2.07656137 2.84509101 2.12437616 2.919401   2.19154103 2.33813975], Cost: 0.0108
Current parameters: [2.07224442 2.83733201 2.11667353 2.92417984 2.18521183 2.33157652], Cost: 0.0100
Current parameters: [2.07666441 2.83490645 2.11987053 2.92179863 2.18387321 2.32734183], Cost: 0.0073
Current parameters: [2.0855444  2.83053763 2.12528524 2.92707844 2.18610347 2.31817502], Cost: 0.0044
Current parameters: [2.08381661 2.82486166 2.13705651 2.92548603 2.17823628 2.31573625], Cost: 0.0027
Current parameters: [2.08324639 2.81355883 2.13760166 2.91830673 2.18612774 2.314341  ], Cost: 0.0036
Current parameters: [2.07971153 2.82804452 2.13807004 2.92410285 2.18025039 2.31053473], Cost: 0.0028
Current parameters: [2.09325298 2.82617588 2.13633195 2.91961545 2.17032947 2.30826216], Cost: 0.0092
Current parameters: [2.08107019 2.82135303 2.13280174 2.92818052 2.1752861  2.31306888], Cost: 0.0039
Current parameters: [2.08163008 2.82443366 2.14619276 2.93598468 2.18410277 2.31906313], Cost: 0.0035
Current parameters: [2.07894545 2.82594574 2.13768781 2.92259865 2.17669922 2.32073961], Cost: 0.0038
Current parameters: [2.08362785 2.82467916 2.13925412 2.92826944 2.17978615 2.31619549], Cost: 0.0019
Current parameters: [2.08571006 2.82529573 2.14214392 2.92958393 2.1801331  2.31560914], Cost: 0.0014
Current parameters: [2.0871474  2.82683523 2.14170346 2.93126008 2.18286558 2.31620315], Cost: 0.0017
Current parameters: [2.08589147 2.82429485 2.1425915  2.92850385 2.18131928 2.31568725], Cost: 0.0014
Current parameters: [2.08545529 2.82298527 2.14369747 2.93182716 2.17955336 2.31416901], Cost: 0.0012
Current parameters: [2.08411362 2.82350996 2.14670982 2.93200568 2.17891666 2.31608523], Cost: 0.0012
Current parameters: [2.08514627 2.82272379 2.14636008 2.93222817 2.17834579 2.31736293], Cost: 0.0013
Current parameters: [2.08543783 2.82427922 2.14874806 2.9310109  2.17753282 2.31366564], Cost: 0.0009
Current parameters: [2.08374442 2.82415649 2.1501458  2.9304065  2.17614552 2.31081388], Cost: 0.0012
Current parameters: [2.08708955 2.82584487 2.15014633 2.93286145 2.1795946  2.31299575], Cost: 0.0006
Current parameters: [2.08668531 2.82838438 2.15188209 2.93143198 2.17960057 2.31489048], Cost: 0.0007
Current parameters: [2.08711886 2.8247065  2.15100272 2.93203395 2.18063644 2.31311563], Cost: 0.0007
Current parameters: [2.08889277 2.82713217 2.14819842 2.93117833 2.18089752 2.31157269], Cost: 0.0006
Current parameters: [2.09238162 2.82674554 2.1488084  2.93223368 2.17975046 2.31194101], Cost: 0.0006
Current parameters: [2.09251359 2.82705953 2.14816689 2.93361192 2.18089807 2.31220673], Cost: 0.0007
Current parameters: [2.09235775 2.82702332 2.14936194 2.93246893 2.17986789 2.31123362], Cost: 0.0005
Current parameters: [2.09228328 2.82715032 2.14970925 2.93232857 2.1789409  2.30956228], Cost: 0.0004
Current parameters: [2.0923771  2.82866096 2.15079228 2.93178906 2.17872326 2.30967388], Cost: 0.0005
```

```
Current parameters: [2.09271455 2.82722721 2.14942032 2.93176659 2.17945588 2.30925043], Cost: 0.0004
Current parameters: [2.09216644 2.82584748 2.15102248 2.932214   2.17939527 2.30916291], Cost: 0.0004
Current parameters: [2.09303981 2.82575945 2.15115747 2.93251386 2.17913988 2.30926183], Cost: 0.0004
Current parameters: [2.0929332  2.82571539 2.15116974 2.93398192 2.17981942 2.30817359], Cost: 0.0002
Current parameters: [2.09278481 2.8251993  2.15109147 2.93533578 2.18009614 2.30690446], Cost: 0.0002
Current parameters: [2.09279847 2.82637432 2.15234669 2.93589688 2.18067093 2.3064429 ], Cost: 0.0002
Current parameters: [2.09240875 2.82627986 2.15255481 2.936447   2.18035483 2.30703197], Cost: 0.0002
Current parameters: [2.09322806 2.82609854 2.15193757 2.93626135 2.18243404 2.3068241 ], Cost: 0.0004
Current parameters: [2.09366447 2.8264259  2.15233991 2.93620208 2.1803733  2.30658177], Cost: 0.0002
Current parameters: [2.09247398 2.8276909  2.15110811 2.93622608 2.18022896 2.30607108], Cost: 0.0001
Current parameters: [2.0923503  2.827693   2.15154611 2.93582735 2.17907568 2.30461556], Cost: 0.0001
Current parameters: [2.09247308 2.82862828 2.15192301 2.9343906  2.17858828 2.30530912], Cost: 0.0002

   Return from subroutine COBYLA because the MAXFUN limit has been reached.

   NFVALS =  100   F = 9.301511E-05    MAXCV = 0.000000E+00
   X = 2.092350E+00   2.827693E+00   2.151546E+00   2.935827E+00   2.179076E+00
       2.304616E+00
Current parameters: [2.0923503  2.827693   2.15154611 2.93582735 2.17907568 2.30461556], Cost: 0.0001
Simulating for QAOA depth p = 4
Current parameters: [3.74912846 0.45253772 2.04427139 1.31958613 2.9236173  1.43759082
 1.9157916  3.0205903 ], Cost: 1.5484
Current parameters: [2.74912846 1.45253772 2.04427139 1.31958613 2.9236173  1.43759082
 1.9157916  3.0205903 ], Cost: 1.9419
Current parameters: [2.74912846 0.45253772 3.04427139 1.31958613 2.9236173  1.43759082
 1.9157916  3.0205903 ], Cost: 2.1222
Current parameters: [2.74912846 0.45253772 2.04427139 2.31958613 2.9236173  1.43759082
 1.9157916  3.0205903 ], Cost: 1.3555
Current parameters: [2.74912846 0.45253772 2.04427139 1.31958613 3.9236173  1.43759082
 1.9157916  3.0205903 ], Cost: 0.7529
Current parameters: [2.74912846 0.45253772 2.04427139 1.31958613 3.9236173  2.43759082
 1.9157916  3.0205903 ], Cost: 2.1269
Current parameters: [2.74912846 0.45253772 2.04427139 1.31958613 3.9236173  1.43759082
 2.9157916  3.0205903 ], Cost: 1.7018
Current parameters: [2.74912846 0.45253772 2.04427139 1.31958613 3.9236173  1.43759082
 1.9157916  4.0205903 ], Cost: 1.4999
Current parameters: [2.55746713 0.09294275 1.60771205 1.21027245 4.07148371 0.85114715
 1.51081442 2.70178521], Cost: 2.4812
Current parameters: [2.72815425 0.94199949 1.99649709 1.30762353 3.93979887 1.37341412
 1.87147345 2.98570228], Cost: 1.9598
Current parameters: [2.97803925 0.45253772 1.94377338 1.31958613 3.9236173  1.43759082
```



```
 1.9157916  3.0205903 ], Cost: 0.6316
Current parameters: [3.19441735 0.34167392 2.36705813 1.30483239 3.94357439 1.35844025
 1.86113303 2.97756212], Cost: 0.7546
Current parameters: [2.97425081 0.44542985 1.93514422 1.06986496 3.9236173  1.43759082
 1.9157916  3.0205903 ], Cost: 0.5152
Current parameters: [2.95826458 0.05779922 1.74028224 1.02676149 3.96906823 1.25733075
 1.79131039 2.92259653], Cost: 1.7636
Current parameters: [2.97425081 0.44542985 1.93514422 1.06986496 4.16603033 1.49871304
 1.9157916  3.0205903 ], Cost: 0.7904
Current parameters: [3.01480166 0.38429992 1.96671877 1.04645212 3.75104595 1.89541
 1.86803303 2.98299392], Cost: 1.9149
Current parameters: [2.97425081 0.44542985 1.93514422 1.06986496 3.9236173  1.43759082
 2.07042949 2.82415425], Cost: 0.8078
Current parameters: [3.00638646 0.39698574 1.96016635 1.05131081 3.89823943 1.35866078
 2.26911647 3.35813682], Cost: 1.8455
Current parameters: [2.99192028 0.64554648 1.93332985 1.04774092 3.90947434 1.33689362
 1.81340657 2.99185505], Cost: 0.5843
Current parameters: [2.92835032 0.47560207 1.83059371 1.07331527 3.91895015 1.45610094
 1.94412923 3.04289818], Cost: 0.4876
Current parameters: [2.96660184 0.51325627 1.8759334  1.05397906 3.90132218 1.37024306
 2.09099653 3.21004208], Cost: 1.0799
Current parameters: [2.90306201 0.3339402  1.73026882 1.06471943 3.91283354 1.3211001
 1.83361599 3.00858158], Cost: 0.6612
Current parameters: [2.93855881 0.47600139 1.82045905 1.06062203 3.86398841 1.54962615
 1.88528639 3.03774448], Cost: 0.6719
Current parameters: [2.98071066 0.47560207 1.80760611 1.07331527 3.94340797 1.46226776
 1.94412923 3.04289818], Cost: 0.5443
Current parameters: [2.8696841  0.48258247 1.84782227 1.06683831 4.02181319 1.48066163
 1.91944652 3.04448122], Cost: 0.6091
Current parameters: [2.93073708 0.50881486 1.82879144 1.02638754 3.91651614 1.43877092
 1.93084689 3.03244209], Cost: 0.4406
Current parameters: [2.93160245 0.58617773 1.82950708 1.10400537 3.90685773 1.40112081
 1.88991411 3.0117566 ], Cost: 0.4410
Current parameters: [2.90839589 0.49946328 1.82685287 1.04687683 3.90076621 1.44127382
 1.9698494  2.99896703], Cost: 0.4195
Current parameters: [2.94305423 0.53223385 1.82631962 0.98742249 3.92610246 1.43052237
 1.97201282 2.90382195], Cost: 0.4283
Current parameters: [2.91935502 0.53534283 1.85105868 1.0444031  3.90383797 1.46712135
 2.00085759 3.01532939], Cost: 0.5392
Current parameters: [2.87956012 0.46633502 1.80019463 1.02418016 3.86531913 1.33538595
 1.96989895 2.9977518 ], Cost: 0.4217
```



```
Current parameters: [2.86588663 0.49342268 1.82794294 1.04998151 3.94399172 1.43959435
 1.95853891 2.99170722], Cost: 0.3868
Current parameters: [2.8610039  0.50396945 1.80043162 1.04558402 3.94351409 1.44560846
 1.96209369 2.99573734], Cost: 0.3816
Current parameters: [2.83630872 0.48968378 1.79435346 1.04249561 3.91729676 1.46833659
 1.92459085 2.97486976], Cost: 0.3463
Current parameters: [2.85123283 0.45386005 1.76574986 1.07003983 3.93480477 1.47155629
 1.91116152 2.95681187], Cost: 0.3982
Current parameters: [2.83608063 0.47034743 1.79417744 1.02308092 3.91968959 1.47770394
 1.93484394 2.98007136], Cost: 0.3684
Current parameters: [2.80621537 0.49022988 1.78675341 1.03126784 3.89739565 1.41999585
 1.91569888 2.97273206], Cost: 0.2525
Current parameters: [2.76063715 0.51162213 1.7891025  1.02708511 3.87731314 1.39187416
 1.9047288  2.96692221], Cost: 0.2256
Current parameters: [2.76025044 0.48405123 1.75916386 1.04680182 3.8787133  1.35641127
 1.88611717 2.95095642], Cost: 0.2998
Current parameters: [2.75451836 0.50465218 1.78942626 1.03937596 3.87278156 1.39421774
 1.90028257 2.99325805], Cost: 0.2257
Current parameters: [2.77590237 0.52722302 1.7916337  0.99565047 3.89123172 1.38307075
 1.86193897 2.98517311], Cost: 0.2489
Current parameters: [2.76236325 0.52258911 1.76269754 1.02242441 3.87036659 1.39786283
 1.90981792 2.97185645], Cost: 0.2150
Current parameters: [2.78929089 0.5258645  1.77708067 1.01753781 3.81708797 1.38872761
 1.91373507 2.97336066], Cost: 0.2968
Current parameters: [2.77215946 0.54022924 1.76518339 1.03573607 3.87912381 1.38313761
 1.91808638 2.97677115], Cost: 0.2414
Current parameters: [2.7432964  0.50737175 1.76143201 0.9851332  3.88392234 1.38554114
 1.94514674 2.99016472], Cost: 0.1897
Current parameters: [2.70189321 0.52122007 1.76090881 0.97784796 3.89868154 1.42668905
 1.95038722 2.98725697], Cost: 0.1599
Current parameters: [2.66963887 0.4919907  1.72379294 0.98045871 3.9157183  1.41945179
 1.94114641 2.97314578], Cost: 0.1085
Current parameters: [2.62847238 0.46642354 1.69639775 0.96409364 3.89805471 1.4281091
 1.94480719 2.96134639], Cost: 0.0866
Current parameters: [2.64572563 0.43334185 1.67644325 0.96171777 3.92672789 1.46239926
 1.95540702 2.96151126], Cost: 0.2046
Current parameters: [2.63151138 0.4664566  1.69655497 0.94434871 3.90089627 1.4300118
 1.92189312 2.96772526], Cost: 0.0831
Current parameters: [2.59392061 0.49800261 1.68696662 0.94423791 3.91021339 1.394967
 1.9127885  2.97059648], Cost: 0.0419
Current parameters: [2.56906214 0.53175095 1.71153527 0.9303433  3.89432132 1.42633055
```



```
 1.9221623  2.9759425 ], Cost: 0.0362
Current parameters: [2.57516097 0.53859615 1.7115108  0.91810607 3.89893134 1.42391925
 1.92655056 2.94955243], Cost: 0.0320
Current parameters: [2.55129344 0.51350446 1.75686728 0.92312331 3.90814852 1.41081183
 1.91424978 2.93483694], Cost: 0.0256
Current parameters: [2.5493689  0.5186899  1.7516681  0.94094347 3.90633124 1.42219023
 1.89606216 2.92309726], Cost: 0.0284
Current parameters: [2.54876693 0.52171717 1.76003605 0.91741229 3.85743318 1.37832914
 1.90391053 2.92737433], Cost: 0.1050
Current parameters: [2.53308559 0.49829334 1.74194913 0.91506217 3.90223137 1.41801991
 1.91695022 2.92919211], Cost: 0.0205
Current parameters: [2.50866394 0.50784946 1.73348134 0.90921792 3.95727193 1.42516474
 1.92146134 2.93262623], Cost: 0.0596
Current parameters: [2.53448419 0.49998898 1.75589548 0.90825591 3.89580208 1.44339644
 1.92367316 2.9298273 ], Cost: 0.0304
Current parameters: [2.53515167 0.4979558  1.74180789 0.90527987 3.90283842 1.41852704
 1.90537215 2.93225272], Cost: 0.0199
Current parameters: [2.52561985 0.5046724  1.74213542 0.90049345 3.88046057 1.40172052
 1.90283284 2.9269705 ], Cost: 0.0313
Current parameters: [2.54076276 0.49848218 1.74115746 0.89995992 3.90534776 1.41751804
 1.9074825  2.9191422 ], Cost: 0.0276
Current parameters: [2.54607838 0.47310023 1.73816481 0.91420606 3.89851033 1.41592967
 1.90198406 2.94271518], Cost: 0.0285
Current parameters: [2.52849777 0.49232164 1.75267088 0.90641662 3.90725728 1.4155882
 1.90244068 2.92877863], Cost: 0.0187
Current parameters: [2.51163117 0.49816366 1.74408456 0.90689125 3.92738936 1.4150012
 1.90211115 2.94212955], Cost: 0.0169
Current parameters: [2.5122045  0.50244076 1.74599099 0.90197533 3.92323911 1.426419
 1.90095726 2.94911492], Cost: 0.0250
Current parameters: [2.49830184 0.48563486 1.75649662 0.91697519 3.93570669 1.40952977
 1.91466955 2.93075646], Cost: 0.0182
Current parameters: [2.50461919 0.50170327 1.74269821 0.90737284 3.9196986  1.40434179
 1.89956908 2.94315763], Cost: 0.0079
Current parameters: [2.50123802 0.50415869 1.73956654 0.91706524 3.91850248 1.40692187
 1.89029304 2.93779136], Cost: 0.0092
Current parameters: [2.50657834 0.50734417 1.74690105 0.90869981 3.92164348 1.40293516
 1.89986164 2.9428328 ], Cost: 0.0084
Current parameters: [2.50708483 0.49756859 1.74173717 0.91171763 3.91829193 1.39277203
 1.89681407 2.9507561 ], Cost: 0.0090
Current parameters: [2.50378129 0.49986935 1.74595654 0.90355213 3.92149037 1.40355114
 1.89436064 2.94226775], Cost: 0.0087
```

```
Current parameters: [2.50179065 0.5062738  1.73515992 0.90154482 3.91886978 1.39642293
 1.90222307 2.93580783], Cost: 0.0077
Current parameters: [2.49711545 0.50737071 1.73369208 0.90185538 3.92394973 1.39660599
 1.90263003 2.93892527], Cost: 0.0058
Current parameters: [2.48663911 0.50384751 1.74074797 0.90517032 3.92166743 1.39397294
 1.90901432 2.93604451], Cost: 0.0050
Current parameters: [2.48407456 0.50716166 1.74103393 0.90318975 3.91674103 1.39473856
 1.90748234 2.93954704], Cost: 0.0040
Current parameters: [2.47502808 0.51043424 1.73641969 0.90063784 3.91964798 1.39904573
 1.91323183 2.94753015], Cost: 0.0026
Current parameters: [2.46764423 0.51412153 1.73131139 0.90974297 3.91921141 1.40165152
 1.9059862  2.94478332], Cost: 0.0015
Current parameters: [2.469222   0.51371548 1.72924052 0.9137682  3.91769553 1.39728453
 1.90783928 2.94840099], Cost: 0.0020
Current parameters: [2.45958921 0.51190349 1.73319631 0.90508332 3.92318332 1.39612386
 1.89709567 2.94965156], Cost: 0.0007
Current parameters: [2.45875578 0.52449901 1.74027277 0.90688075 3.92831354 1.39402429
 1.89751008 2.95042159], Cost: 0.0036
Current parameters: [2.45843029 0.51412598 1.72951222 0.90215676 3.92249432 1.39216032
 1.89839867 2.94581318], Cost: 0.0005
Current parameters: [2.45018018 0.50805632 1.7239392  0.8982227  3.91463211 1.39727687
 1.8962684  2.94622414], Cost: 0.0010
Current parameters: [2.45456929 0.51123182 1.73266328 0.90444518 3.9233219  1.39065116
 1.90193396 2.94314391], Cost: 0.0008
Current parameters: [2.45949901 0.50860687 1.72130518 0.90245607 3.93421279 1.39432474
 1.8975276  2.94428782], Cost: 0.0020
Current parameters: [2.46112006 0.51178402 1.73100167 0.90295045 3.9203744  1.3901438
 1.89364735 2.94202747], Cost: 0.0021
Current parameters: [2.45906923 0.51366435 1.73113486 0.89913982 3.92313284 1.39335728
 1.89891185 2.94491942], Cost: 0.0004
Current parameters: [2.46084535 0.51133961 1.73090743 0.89677196 3.92201401 1.38987439
 1.90231153 2.94959675], Cost: 0.0008
Current parameters: [2.46102639 0.5133166  1.73020111 0.9001144  3.92184149 1.39480597
 1.90097977 2.94371815], Cost: 0.0004
Current parameters: [2.45661532 0.51978432 1.73208992 0.89789171 3.92346596 1.39597791
 1.90143841 2.94623373], Cost: 0.0006
Current parameters: [2.45893432 0.51290523 1.7291838  0.89885809 3.92627365 1.39425677
 1.89923505 2.9449285 ], Cost: 0.0003
Current parameters: [2.45784952 0.51220048 1.7285037  0.89839218 3.92538459 1.39502675
 1.8991051  2.94517636], Cost: 0.0003
Current parameters: [2.45816895 0.51181673 1.72825387 0.89668382 3.92564377 1.39570931
```



```
 1.89643066 2.9430924 ], Cost: 0.0007
Current parameters: [2.45707416 0.51226304 1.72817228 0.89809921 3.92535556 1.39399258
 1.89986541 2.94400781], Cost: 0.0003
Current parameters: [2.45549812 0.51048544 1.73046554 0.89945444 3.92625    1.39468028
 1.90090194 2.94359309], Cost: 0.0003
Current parameters: [2.4577562  0.51076568 1.72804934 0.89788303 3.92520565 1.39304554
 1.89978537 2.94435393], Cost: 0.0003
Current parameters: [2.45608736 0.51355054 1.72836593 0.89644399 3.92568028 1.39391228
 1.90237116 2.94586778], Cost: 0.0002
Current parameters: [2.45433677 0.51470381 1.72675099 0.89879184 3.92565741 1.39296277
 1.90285871 2.94713426], Cost: 0.0001
Current parameters: [2.45346858 0.51487164 1.7272415  0.8981911  3.92622861 1.39229496
 1.90162519 2.94750503], Cost: 0.0001
Current parameters: [2.45103838 0.51695381 1.72690394 0.89639258 3.92702797 1.3928652
 1.90186692 2.9467011 ], Cost: 0.0001
Current parameters: [2.45346858 0.51487164 1.7272415  0.8981911  3.92622861 1.39229496
 1.90162519 2.94750503], Cost: 0.0001

   Return from subroutine COBYLA because the MAXFUN limit has been reached.

   NFVALS =  100   F = 9.463895E-05    MAXCV = 0.000000E+00
   X = 2.453469E+00   5.148716E-01   1.727241E+00   8.981911E-01   3.926229E+00
       1.392295E+00   1.901625E+00   2.947505E+00
Simulating for QAOA depth p = 5
Current parameters: [1.06187196 1.730533   0.35742801 0.86998783 1.97215335 2.75876393
 0.02462957 2.43633421 0.97053791 1.95851443], Cost: 1.1373
Current parameters: [1.06187196 2.730533   0.35742801 0.86998783 1.97215335 2.75876393
 0.02462957 2.43633421 0.97053791 1.95851443], Cost: 2.6381
Current parameters: [1.06187196 1.730533   1.35742801 0.86998783 1.97215335 2.75876393
 0.02462957 2.43633421 0.97053791 1.95851443], Cost: 2.7085
Current parameters: [1.06187196 1.730533   0.35742801 1.86998783 1.97215335 2.75876393
 0.02462957 2.43633421 0.97053791 1.95851443], Cost: 2.8801
Current parameters: [1.06187196 1.730533   0.35742801 0.86998783 2.97215335 2.75876393
 0.02462957 2.43633421 0.97053791 1.95851443], Cost: 2.6702
Current parameters: [1.06187196 1.730533   0.35742801 0.86998783 1.97215335 3.75876393
 0.02462957 2.43633421 0.97053791 1.95851443], Cost: 2.8853
Current parameters: [1.06187196 1.730533   0.35742801 0.86998783 1.97215335 2.75876393
 1.02462957 2.43633421 0.97053791 1.95851443], Cost: 3.3795
Current parameters: [1.06187196 1.730533   0.35742801 0.86998783 1.97215335 2.75876393
 0.02462957 3.43633421 0.97053791 1.95851443], Cost: 2.8194
Current parameters: [1.06187196 1.730533   0.35742801 0.86998783 1.97215335 2.75876393
```



```
 0.02462957 2.43633421 1.97053791 1.95851443], Cost: 2.1464
Current parameters: [1.06187196 1.730533   0.35742801 0.86998783 1.97215335 2.75876393
 0.02462957 2.43633421 0.97053791 2.95851443], Cost: 1.4119
Current parameters: [ 1.26943711  1.41834423  0.03058727  0.50745641  1.65328158  2.39514689
 -0.44178811  2.08642821  0.7606298   1.90138867], Cost: 1.8978
Current parameters: [ 1.16565453  1.57443861  0.19400764  0.68872212  1.81271746  2.57695541
 -0.20857927  2.26138121  0.86558386  1.92995155], Cost: 2.5953
Current parameters: [1.07240522e+00 1.97584046e+00 3.40841906e-01 8.51590536e-01
 1.95597164e+00 2.74031155e+00 9.60387879e-04 2.41857761e+00
 9.59885756e-01 1.95561549e+00], Cost: 1.1617
Current parameters: [0.95635226 1.730533   0.29041613 0.86998783 1.97215335 2.75876393
 0.02462957 2.43633421 0.97053791 1.95851443], Cost: 1.3844
Current parameters: [ 0.9572013   1.73743799  0.57604565  0.8445597   1.94978753  2.73325966
 -0.00808519  2.41179163  0.95581486  1.95450761], Cost: 1.3126
Current parameters: [1.06187196 1.730533   0.35742801 0.95254357 1.87829414 2.75876393
 0.02462957 2.43633421 0.97053791 1.95851443], Cost: 1.1405
Current parameters: [ 1.09917225  1.74061065  0.37721139  1.0459942   2.12769535  2.72154114
 -0.02311673  2.40051499  0.94905001  1.95266658], Cost: 2.2419
Current parameters: [ 1.06187196  1.730533    0.35742801  0.86998783  1.97215335  2.85734606
 -0.0522246   2.43633421  0.97053791  1.95851443], Cost: 1.1757
Current parameters: [1.10681264 1.7426749  0.3812637  0.80832541 1.91879897 2.89066792
 0.20665925 2.39317798 0.94464855 1.95146873], Cost: 0.9840
Current parameters: [1.10681264 1.7426749  0.3812637  0.80832541 1.91879897 2.89066792
 0.20665925 2.45748195 0.83745714 1.95146873], Cost: 1.0689
Current parameters: [1.15062554 1.7545121  0.40450125 0.74821038 1.86678351 2.90130079
 0.23281036 2.58081934 1.07704002 1.94459984], Cost: 1.6294
Current parameters: [1.10923253 1.73903526 0.37745324 0.80409885 1.91508142 2.8864287
 0.20122154 2.38909861 0.94220135 1.82701182], Cost: 0.9019
Current parameters: [1.19109886 1.69270652 0.33966497 0.6773234  1.80442314 2.8293515
 0.14036985 2.269843   0.89024817 1.83196811], Cost: 0.3020
Current parameters: [1.33641097 1.66342084 0.3354103  0.69418755 1.82135008 2.80152005
 0.13513528 2.08452523 0.82734899 1.80304158], Cost: 1.1079
Current parameters: [1.17368862 1.67485492 0.44252328 0.64740074 1.83196778 2.77700944
 0.13901161 2.26536812 0.88756369 1.83123754], Cost: 0.3273
Current parameters: [1.0619184  1.74105654 0.40459684 0.66106013 1.65183336 2.92756122
 0.10441432 2.20659188 0.86432832 1.82197264], Cost: 1.0878
Current parameters: [1.12570704 1.66027245 0.27480829 0.62890446 1.83021882 2.77956982
 0.16332611 2.26125935 0.89080291 1.83072372], Cost: 0.2165
Current parameters: [1.12468805 1.60254956 0.27952133 0.63990044 1.82245032 2.79787675
 0.16550959 2.26593165 0.89360582 1.83117907], Cost: 0.2568
Current parameters: [1.17284962 1.65797931 0.25999312 0.56451313 1.7973539  2.75246971
```



```
 0.16302931 2.32948107 0.94112708 1.82522351], Cost: 0.0973
Current parameters: [1.17557593 1.65871589 0.2614391  0.58239861 1.7695299  2.72866669
 0.1988671  2.34264931 0.91324155 1.82479608], Cost: 0.1535
Current parameters: [1.18021006 1.65330378 0.24992338 0.52881498 1.82606132 2.77057859
 0.11850959 2.38312391 0.85078677 1.8247458 ], Cost: 0.0142
Current parameters: [1.17335494 1.65005919 0.24463543 0.55196141 1.81796973 2.74677986
 0.08580072 2.38913744 0.85439428 1.78557808], Cost: 0.0084
Current parameters: [1.17019654 1.64482681 0.23078362 0.57899384 1.80694804 2.70216717
 0.03034123 2.40085516 0.86216335 1.88190997], Cost: 0.2477
Current parameters: [1.21368805 1.63950067 0.23348412 0.56457046 1.85498532 2.72783545
 0.09518249 2.38138804 0.85324071 1.7830837 ], Cost: 0.0239
Current parameters: [1.19686006 1.65688027 0.22332762 0.46159231 1.80044349 2.75981734
 0.0729553  2.34726825 0.84028922 1.72384273], Cost: 0.0797
Current parameters: [1.18031088 1.65992595 0.1941811  0.56953103 1.80656886 2.77405657
 0.08775478 2.39387026 0.85682232 1.78583804], Cost: 0.0215
Current parameters: [1.13570637 1.67433069 0.25345531 0.53881675 1.88031502 2.76828041
 0.1115038  2.46434208 0.88598743 1.74429632], Cost: 0.2239
Current parameters: [1.20929415 1.66747702 0.27626421 0.56672849 1.8042896  2.77267683
 0.0726109  2.39533384 0.85465154 1.78082791], Cost: 0.0285
Current parameters: [1.179715   1.62510719 0.24451044 0.54737092 1.80991925 2.75068265
 0.0866893  2.40096324 0.86284312 1.78497956], Cost: 0.0053
Current parameters: [1.161799   1.59544881 0.25344609 0.56805504 1.82050623 2.77721005
 0.08463807 2.37275091 0.83991902 1.7774613 ], Cost: 0.0568
Current parameters: [1.1820388  1.62437525 0.24289172 0.5454792  1.80738821 2.74720132
 0.08841    2.41587881 0.83600255 1.78475322], Cost: 0.0029
Current parameters: [1.18421203 1.62642854 0.24799059 0.56061247 1.77415107 2.72622085
 0.13174946 2.40654364 0.83757423 1.77337406], Cost: 0.0276
Current parameters: [1.17565381 1.62288426 0.24673406 0.56603152 1.8091772  2.74189642
 0.08690281 2.42569838 0.83953109 1.80356601], Cost: 0.0138
Current parameters: [1.19337979 1.62253651 0.23085433 0.51879443 1.78337004 2.72314427
 0.05737873 2.40009688 0.8318967  1.80783555], Cost: 0.0625
Current parameters: [1.16580576 1.63848664 0.24495472 0.52408888 1.83013471 2.74988309
 0.0988262  2.45489205 0.85294961 1.76152656], Cost: 0.0741
Current parameters: [1.20240319 1.61929775 0.23726407 0.55052942 1.82576513 2.73695214
 0.0930375  2.41248173 0.83568424 1.78299654], Cost: 0.0042
Current parameters: [1.18290459 1.62412145 0.24919379 0.53841179 1.81038966 2.74989134
 0.09569389 2.4140257  0.83493238 1.79368535], Cost: 0.0037
Current parameters: [1.18714588 1.62974926 0.22033672 0.54784751 1.80385844 2.76386884
 0.09597532 2.41726276 0.83663908 1.79233236], Cost: 0.0174
Current parameters: [1.19192323 1.63097461 0.24937655 0.54702539 1.8032778  2.75080738
 0.08555821 2.41984541 0.83812322 1.78432844], Cost: 0.0021
```

```
Current parameters: [1.19237046 1.6197347  0.26086239 0.55688824 1.80651956 2.7667566
 0.0811026  2.40772228 0.82679904 1.77652587], Cost: 0.0157
Current parameters: [1.19874958 1.62802234 0.24683088 0.53490797 1.79274029 2.73804452
 0.06813522 2.41028344 0.83469983 1.79367156], Cost: 0.0113
Current parameters: [1.18914325 1.63318949 0.25200355 0.53961132 1.80910013 2.74880178
 0.08431001 2.42842899 0.84155412 1.77764201], Cost: 0.0056
Current parameters: [1.19352393 1.62501947 0.24950647 0.5463912  1.80030038 2.75109661
 0.08712347 2.42239785 0.8402273  1.78382921], Cost: 0.0043
Current parameters: [1.19131263 1.6364555  0.25184443 0.54885127 1.79772413 2.74357611
 0.09461362 2.41617493 0.83707481 1.77936891], Cost: 0.0028
Current parameters: [1.19266472 1.63101214 0.24809788 0.54666464 1.80249181 2.75061807
 0.08627671 2.42359275 0.83153277 1.7845974 ], Cost: 0.0028
Current parameters: [1.19362995 1.63766401 0.23850935 0.54568816 1.80229461 2.75778034
 0.0893698  2.41859769 0.84131678 1.78517851], Cost: 0.0059
Current parameters: [1.18669142 1.63218365 0.25088666 0.54641307 1.7987903  2.75317683
 0.08436762 2.42098912 0.83831113 1.78534498], Cost: 0.0031
Current parameters: [1.19048324 1.63335374 0.25023467 0.55707032 1.80709904 2.74553833
 0.08570819 2.42433475 0.84090151 1.79236023], Cost: 0.0040
Current parameters: [1.19361083 1.63170292 0.24870944 0.54421199 1.80164149 2.74668906
 0.08136398 2.41719822 0.83760049 1.78651612], Cost: 0.0007
Current parameters: [1.19470473 1.6314611  0.25143049 0.54579402 1.80318254 2.74729297
 0.07801047 2.41206376 0.83478333 1.78525579], Cost: 0.0024
Current parameters: [1.19350826 1.63193718 0.24663457 0.54587828 1.80081468 2.7463886
 0.07976776 2.41763706 0.83799839 1.78440922], Cost: 0.0007
Current parameters: [1.19553825 1.63551877 0.24163498 0.5439154  1.80067851 2.74828632
 0.08089025 2.41567399 0.83925102 1.7866307 ], Cost: 0.0011
Current parameters: [1.19339003 1.63221673 0.24686057 0.54301012 1.80131218 2.74691097
 0.07952961 2.41781589 0.83799894 1.78190319], Cost: 0.0004
Current parameters: [1.19260306 1.63529243 0.2473658  0.54219218 1.80486873 2.74268413
 0.07771802 2.42108446 0.84025389 1.7825859 ], Cost: 0.0002
Current parameters: [1.19463767 1.63720986 0.249055   0.54273902 1.80316824 2.74247717
 0.07838786 2.42150439 0.84062518 1.78181595], Cost: 0.0004
Current parameters: [1.18991729 1.63588345 0.24633949 0.54200526 1.80385856 2.73791825
 0.0818535  2.41828467 0.83956094 1.78078898], Cost: 0.0009
Current parameters: [1.19531029 1.63347465 0.24671074 0.54233368 1.80626223 2.74167452
 0.07859045 2.42111215 0.84061804 1.78201971], Cost: 0.0006
Current parameters: [1.19139083 1.63243113 0.24727881 0.5388712  1.80070016 2.74178685
 0.07529858 2.4231754  0.84339271 1.78402886], Cost: 0.0008
Current parameters: [1.19289131 1.63552901 0.24657841 0.54155276 1.80441513 2.74224452
 0.07753743 2.42297372 0.83710164 1.78297738], Cost: 0.0001
Current parameters: [1.19333433 1.63561899 0.24685037 0.54129705 1.80435444 2.74162751
```



```
 0.07634638 2.42179412 0.83661304 1.78317194], Cost: 0.0001
Current parameters: [1.19202091 1.63637657 0.2478021  0.54143428 1.80617163 2.74318496
 0.0743807  2.42184057 0.83529309 1.7823359 ], Cost: 0.0002
Current parameters: [1.19340506 1.63594476 0.24554962 0.54198717 1.80394617 2.74159461
 0.07564035 2.42189012 0.83687992 1.78228389], Cost: 0.0001
Current parameters: [1.19393878 1.63759716 0.24506099 0.53965346 1.80488435 2.74281727
 0.07663281 2.42094918 0.83684914 1.78476221], Cost: 0.0001
Current parameters: [1.19373662 1.63545994 0.24690137 0.54246867 1.80436761 2.7420236
 0.07582037 2.42217126 0.83674646 1.78445976], Cost: 0.0001
Current parameters: [1.19123197 1.63641797 0.24632706 0.5424746  1.80416955 2.73959441
 0.07726436 2.42048777 0.83551605 1.78395916], Cost: 0.0003
Current parameters: [1.19315624 1.63614931 0.24697477 0.54089349 1.80493185 2.74053614
 0.0757839  2.42271756 0.83737124 1.78340633], Cost: 0.0001
Current parameters: [1.19240103 1.63398621 0.2467189  0.53954123 1.80191226 2.74228642
 0.07494076 2.42188464 0.83679116 1.78362748], Cost: 0.0002
Current parameters: [1.19443937 1.63455915 0.24636707 0.54113741 1.80524209 2.74135208
 0.07646354 2.4215395  0.83622152 1.78283189], Cost: 0.0001
Current parameters: [1.19360247 1.6358635  0.24683331 0.54120176 1.80408068 2.74150445
 0.07637035 2.42227662 0.83591382 1.78318733], Cost: 0.0001
Current parameters: [1.19472448 1.63660686 0.24776592 0.54142682 1.80351403 2.74162732
 0.07654334 2.42183446 0.83622767 1.7825372 ], Cost: 0.0000
Current parameters: [1.19456961 1.63697358 0.24770384 0.54191465 1.80385952 2.74124088
 0.07694157 2.42156822 0.83599495 1.78256958], Cost: 0.0000
Current parameters: [1.19454526 1.63644682 0.24797044 0.54130148 1.80202962 2.74109558
 0.07655193 2.42089848 0.83564734 1.78252005], Cost: 0.0001
Current parameters: [1.19444053 1.63625417 0.24826096 0.54180653 1.80359896 2.74154621
 0.07628063 2.42204182 0.83603695 1.7820931 ], Cost: 0.0000
Current parameters: [1.19429253 1.6371358  0.24849362 0.54147276 1.80415729 2.7426329
 0.07564312 2.42156677 0.83525783 1.78174922], Cost: 0.0001
Current parameters: [1.19440643 1.6362354  0.24826034 0.54193526 1.80338517 2.74189073
 0.07698866 2.4224538  0.8361479  1.78179674], Cost: 0.0000
Current parameters: [1.19433099 1.6363533  0.24826492 0.54150148 1.80366618 2.74132717
 0.07632731 2.42207148 0.83609983 1.78184009], Cost: 0.0000
Current parameters: [1.1947698  1.63648077 0.24851952 0.54211916 1.80357401 2.74122705
 0.07592104 2.42243419 0.83629792 1.78251199], Cost: 0.0000
Current parameters: [1.19443292 1.63616314 0.2485815  0.54163268 1.80370904 2.74155192
 0.07645807 2.42201552 0.83596672 1.78231184], Cost: 0.0000
Current parameters: [1.19515587 1.63577883 0.24810716 0.54186589 1.80389945 2.74140449
 0.07629192 2.42191392 0.83583189 1.78195195], Cost: 0.0000
Current parameters: [1.19440118 1.63620439 0.24828342 0.54186825 1.80386196 2.74166605
 0.07627598 2.42193933 0.83640437 1.78206111], Cost: 0.0000
```



```
Current parameters: [1.19433691 1.63598568 0.24841402 0.54201873 1.80306074 2.74138822
 0.0761892  2.42138858 0.8362105  1.78224329], Cost: 0.0000
Current parameters: [1.1939639  1.63581469 0.24783853 0.54186023 1.8038142  2.74147993
 0.07612288 2.42225387 0.83590784 1.78255655], Cost: 0.0000
Current parameters: [1.19444224 1.63642396 0.24832737 0.54184262 1.80371025 2.74183272
 0.07605046 2.42191371 0.83584178 1.78213694], Cost: 0.0000
Current parameters: [1.19448063 1.63633231 0.2483427  0.54172208 1.80362167 2.74193292
 0.0759587  2.42198453 0.83590113 1.78212583], Cost: 0.0000
Current parameters: [1.19445899 1.63643885 0.24835555 0.54207553 1.80361778 2.74212499
 0.07630209 2.42207229 0.83582199 1.78213017], Cost: 0.0000
Current parameters: [1.19441211 1.6363397  0.24845064 0.54228485 1.80333483 2.74220281
 0.07632831 2.42179255 0.83586848 1.78220962], Cost: 0.0000
Current parameters: [1.19466621 1.63642038 0.24839537 0.54208263 1.80363646 2.74211523
 0.07632981 2.42201299 0.83580314 1.78203216], Cost: 0.0000
Current parameters: [1.19445538 1.63615146 0.24815897 0.54223813 1.80375983 2.74223082
 0.0763151  2.42215899 0.83566971 1.78229892], Cost: 0.0000
Current parameters: [1.19440826 1.6360892  0.24829561 0.54233848 1.8037809  2.74217846
 0.07622798 2.4222387  0.83564681 1.78221457], Cost: 0.0000
Current parameters: [1.19455108 1.63626365 0.24836351 0.54252212 1.8037422  2.74214906
 0.0761362  2.42245123 0.83577008 1.78249405], Cost: 0.0000
Current parameters: [1.19440826 1.6360892  0.24829561 0.54233848 1.8037809  2.74217846
 0.07622798 2.4222387  0.83564681 1.78221457], Cost: 0.0000

   Return from subroutine COBYLA because the MAXFUN limit has been reached.

   NFVALS =  100   F = 4.384807E-06    MAXCV = 0.000000E+00
   X = 1.194408E+00   1.636089E+00   2.482956E-01   5.423385E-01   1.803781E+00
       2.742178E+00   7.622798E-02   2.422239E+00   8.356468E-01   1.782215E+00
Simulating for QAOA depth p = 6
Current parameters: [1.51061484 2.99577727 3.12326217 0.70786484 1.46087843 0.2138001
 2.43603957 1.39978644 2.59836956 2.1489994  0.0343038  2.42530306], Cost: 1.2842
Current parameters: [1.51061484 3.99577727 3.12326217 0.70786484 1.46087843 0.2138001
 2.43603957 1.39978644 2.59836956 2.1489994  0.0343038  2.42530306], Cost: 2.4813
Current parameters: [1.51061484 2.99577727 4.12326217 0.70786484 1.46087843 0.2138001
 2.43603957 1.39978644 2.59836956 2.1489994  0.0343038  2.42530306], Cost: 1.8889
Current parameters: [1.51061484 2.99577727 3.12326217 1.70786484 1.46087843 0.2138001
 2.43603957 1.39978644 2.59836956 2.1489994  0.0343038  2.42530306], Cost: 1.6224
Current parameters: [1.51061484 2.99577727 3.12326217 0.70786484 2.46087843 0.2138001
 2.43603957 1.39978644 2.59836956 2.1489994  0.0343038  2.42530306], Cost: 2.0649
Current parameters: [1.51061484 2.99577727 3.12326217 0.70786484 1.46087843 1.2138001
 2.43603957 1.39978644 2.59836956 2.1489994  0.0343038  2.42530306], Cost: 2.0439
```



```
Current parameters: [1.51061484 2.99577727 3.12326217 0.70786484 1.46087843 0.2138001
 3.43603957 1.39978644 2.59836956 2.1489994  0.0343038  2.42530306], Cost: 2.6248
Current parameters: [1.51061484 2.99577727 3.12326217 0.70786484 1.46087843 0.2138001
 2.43603957 2.39978644 2.59836956 2.1489994  0.0343038  2.42530306], Cost: 2.2282
Current parameters: [1.51061484 2.99577727 3.12326217 0.70786484 1.46087843 0.2138001
 2.43603957 1.39978644 3.59836956 2.1489994  0.0343038  2.42530306], Cost: 2.2182
Current parameters: [1.51061484 2.99577727 3.12326217 0.70786484 1.46087843 0.2138001
 2.43603957 1.39978644 2.59836956 3.1489994  0.0343038  2.42530306], Cost: 0.6634
Current parameters: [1.51061484 2.99577727 3.12326217 0.70786484 1.46087843 0.2138001
 2.43603957 1.39978644 2.59836956 3.1489994  1.0343038  2.42530306], Cost: 2.4021
Current parameters: [1.51061484 2.99577727 3.12326217 0.70786484 1.46087843 0.2138001
 2.43603957 1.39978644 2.59836956 3.1489994  0.0343038  3.42530306], Cost: 2.5982
Current parameters: [ 1.60023865  2.67497795  2.9612082   0.61722683  1.25167085  0.01021739
  2.07679013  1.14681478  2.34806524  3.31537896 -0.43165119  1.90678438], Cost: 1.0197
Current parameters: [ 1.52446347  3.4738786   3.09822168  0.6938595   1.42855178  0.1823426
  2.38052853  1.36069739  2.55969266  3.17470829 -0.03769533  2.34518181], Cost: 1.8949
Current parameters: [1.29184314 2.99577727 3.00227067 0.70786484 1.46087843 0.2138001
 2.43603957 1.39978644 2.59836956 3.1489994  0.0343038  2.42530306], Cost: 0.9429
Current parameters: [ 1.29340474e+00  2.94846426e+00  3.56504429e+00  7.00687762e-01
  1.44431253e+00  1.97679604e-01  2.40759276e+00  1.37975513e+00
  2.57854946e+00  3.16217400e+00 -2.59238159e-03  2.38424467e+00], Cost: 1.0795
Current parameters: [1.51061484 2.99577727 3.12326217 0.93726119 1.36149374 0.2138001
 2.43603957 1.39978644 2.59836956 3.1489994  0.0343038  2.42530306], Cost: 0.6511
Current parameters: [ 1.54763532  2.937707    3.11650345  1.13219441  1.80820868  0.19401439
  2.40112499  1.37520074  2.57404309  3.16516943 -0.01098123  2.37490948], Cost: 1.7741
Current parameters: [1.51061484 2.99577727 3.12326217 0.93726119 1.36149374 0.43130356
 2.31278274 1.39978644 2.59836956 3.1489994  0.0343038  2.42530306], Cost: 1.4095
Current parameters: [ 1.5724376   2.89880218  3.11197537  0.89897062  1.26772996  0.31510312
  2.88242497  1.35872927  2.5577453   3.17600272 -0.04132045  2.34114774], Cost: 0.5467
Current parameters: [ 1.5724376   2.89880218  3.11197537  0.89897062  1.26772996  0.31510312
  2.88242497  1.53456659  2.38003419  3.17600272 -0.04132045  2.34114774], Cost: 0.5452
Current parameters: [ 1.62771618  2.81209229  3.10188332  0.86473325  1.18389145  0.19154573
  2.90368463  1.85625407  2.69800279  3.20014763 -0.10893958  2.2659006 ], Cost: 3.2110
Current parameters: [ 1.5724376   2.89880218  3.11197537  0.89897062  1.26772996  0.31510312
  2.88242497  1.53456659  2.38003419  3.17600272 -0.22727087  2.50824785], Cost: 0.7480
Current parameters: [1.64058193 2.7919111  3.09953446 0.85676472 1.16437856 0.16278851
 2.90863269 1.31615281 2.16352028 3.2057672  0.19944143 2.55088896], Cost: 1.5629
Current parameters: [ 1.57085654  2.97430366  3.11483418  0.90056957  1.27142062  0.31869455
  2.88876253  1.53902929  2.38444983  3.41376091 -0.03310049  2.35029499], Cost: 1.1358
Current parameters: [ 1.60305504  3.30413973  3.12234285  0.88385493  1.22931996  0.24702454
  2.93732307  1.43267725  2.27897999  2.98784736 -0.11574565  2.22380504], Cost: 1.3284
```

```
Current parameters: [1.50755306 2.96403818 3.22929685 0.91855218 1.31292746 0.35908541
 2.96003771 1.58921893 2.43411027 3.14005785 0.0593451  2.45316923], Cost: 1.9365
Current parameters: [ 1.39613268  2.76421687  3.45420224  0.87136139  1.20274784  0.24091962
  2.813937    1.43621861  2.2826379   3.2074186  -0.18208447  2.17219341], Cost: 1.3470
Current parameters: [ 1.57412513  2.89030561  3.10892407  1.0750648   1.38566811  0.18331693
  2.88250743  1.53096932  2.37647485  3.17836864 -0.04794635  2.33377439], Cost: 0.7100
Current parameters: [ 1.68454693  2.76201649  2.9804675   0.77903225  1.58085284  0.50432038
  2.9271626   1.4213386   2.26774098  3.14941511 -0.12719772  2.20818561], Cost: 1.8933
Current parameters: [ 1.36714569  2.88912636  2.97422784  0.89592958  1.24825323  0.30202322
  2.89775686  1.52205762  2.36761771  3.16886103 -0.04570384  2.33058252], Cost: 1.0105
Current parameters: [ 1.58597186  2.87757236  3.10950446  0.94865982  1.22204389  0.34472558
  2.97592872  1.52557834  2.37114071  3.18191429 -0.05787614  2.32272444], Cost: 0.8301
Current parameters: [ 1.59819654  2.90196603  3.08736308  1.02017031  1.19875903  0.42806357
  2.71513904  1.51865569  2.36421025  3.1549522  -0.03236896  2.33944662], Cost: 0.7316
Current parameters: [1.61144488e+00 2.86670609e+00 3.09156056e+00 8.75123559e-01
 1.22814252e+00 2.56862449e-01 2.86824029e+00 1.49145201e+00
 2.33732026e+00 3.17147313e+00 3.40751250e-04 2.37972183e+00], Cost: 0.3747
Current parameters: [ 1.61106418e+00  2.87205438e+00  3.09385816e+00  8.80092618e-01
  1.19188439e+00  2.29369451e-01  2.86242471e+00  1.52093597e+00
  2.36649334e+00  3.16998386e+00 -2.69303144e-03  2.37634582e+00], Cost: 0.4615
Current parameters: [ 1.67455421  2.81956852  3.04526289  0.82480046  1.26814344  0.25069721
  2.84275783  1.49693291  2.34262501  3.15505383 -0.02104868  2.33883597], Cost: 0.4928
Current parameters: [ 1.59360808e+00  2.81705804e+00  3.11933720e+00  8.74732203e-01
  1.22747890e+00  2.58306951e-01  2.85887233e+00  1.49443261e+00
  2.34026943e+00  3.18699921e+00 -7.26086103e-04  2.37853465e+00], Cost: 0.4511
Current parameters: [ 1.60173261  2.90301451  3.14437187  0.85388628  1.21110332  0.22492613
  2.84716442  1.44452471  2.29083196  3.14936083 -0.03967111  2.32714215], Cost: 0.3855
Current parameters: [1.60547054 2.85570378 3.1023631  0.8766684  1.23170828 0.26033234
 2.87436337 1.49576369 2.34158648 3.11324762 0.00828254 2.38855953], Cost: 0.3774
Current parameters: [1.63502115 2.9190013  3.1289812  0.83562833 1.26175266 0.25640695
 2.85734438 1.47766445 2.32346544 3.18237702 0.07233007 2.42914059], Cost: 0.4111
Current parameters: [1.59112901 2.86573808 3.07792382 0.87503792 1.22635911 0.25540677
 2.8697578  1.49020764 2.33608901 3.1707692  0.0423101  2.34055524], Cost: 0.4292
Current parameters: [ 1.53894063  2.87889176  3.02874852  0.8457579   1.23303551  0.24161803
  2.85475148  1.46674042  2.3127322   3.16385082 -0.02662961  2.43424643], Cost: 0.5876
Current parameters: [1.61508347e+00 2.86835144e+00 3.08863529e+00 8.57777265e-01
 1.19331861e+00 3.00323294e-01 2.84686775e+00 1.48970162e+00
 2.33558833e+00 3.16816058e+00 2.54148829e-03 2.38052037e+00], Cost: 0.3549
Current parameters: [1.64240593 2.87474832 3.0798254  0.94836787 1.20351061 0.30624718
 2.7727288  1.47134958 2.31735602 3.15829535 0.0116409  2.38879081], Cost: 0.5119
Current parameters: [1.61510121e+00 2.86832360e+00 3.08863205e+00 8.57842421e-01
```



```
 1.19325870e+00 3.00362137e-01 2.84699036e+00 1.53364901e+00
 2.29114904e+00 3.16816833e+00 2.51977910e-03 2.38049621e+00], Cost: 0.3547
Current parameters: [1.66459312e+00 2.86351873e+00 3.09230278e+00 8.74478308e-01
 1.15504007e+00 3.24901902e-01 2.93981500e+00 1.50064496e+00
 2.25881651e+00 3.17093835e+00 1.73153532e-03 2.38682133e+00], Cost: 0.2977
Current parameters: [ 1.64240712  2.87127372  3.05604523  0.87797478  1.16500205  0.31424614
  2.94736172  1.48636424  2.24468338  3.18237748 -0.01963048  2.41450605], Cost: 0.3593
Current parameters: [1.64362127 2.85298081 3.11703046 0.80521208 1.09364573 0.36020065
 2.94786271 1.48887521 2.24703461 3.11331027 0.03313438 2.38622433], Cost: 0.2990
Current parameters: [ 1.65806616  2.88759712  3.10897238  0.87407095  1.17329881  0.36333762
  2.95885566  1.513691    2.2717341   3.16893265 -0.01567299  2.37308809], Cost: 0.3031
Current parameters: [ 1.67780149  2.8572614   3.0826839   0.85361646  1.16448464  0.32084419
  2.94401563  1.50368414  2.26180372  3.17111523 -0.00557567  2.38047224], Cost: 0.3107
Current parameters: [1.7012104  2.86269219 3.09230483 0.89885037 1.15346624 0.3479934
 2.91564465 1.48847318 2.24670738 3.14907996 0.00653438 2.37939136], Cost: 0.2407
Current parameters: [1.73360879e+00 2.88407082e+00 3.09777609e+00 9.15953166e-01
 1.11827991e+00 3.39902641e-01 2.91085183e+00 1.50595635e+00
 2.26396714e+00 3.13797908e+00 2.00131063e-03 2.38119685e+00], Cost: 0.1787
Current parameters: [1.76033318 2.91120807 3.11995699 0.90655299 1.1303324  0.33900936
 2.90586756 1.50168648 2.25962645 3.14669575 0.02945421 2.41013907], Cost: 0.1795
Current parameters: [1.73419032 2.87481843 3.08446533 0.91974722 1.11966191 0.3512837
 2.91483885 1.51730304 2.27520758 3.13808444 0.01587914 2.39139546], Cost: 0.2803
Current parameters: [ 1.74691729  2.88867173  3.13504367  0.92108918  1.10368553  0.32510389
  2.89910958  1.48939098  2.24753368  3.13801442 -0.0277999   2.36416009], Cost: 0.1210
Current parameters: [ 1.74650665  2.92247893  3.12248871  0.9244437   1.09997248  0.31056146
  2.90253336  1.47810882  2.23635166  3.13444696 -0.00725632  2.32329209], Cost: 0.1636
Current parameters: [ 1.74411716  2.89202266  3.13074156  0.90688798  1.08656596  0.33990731
  2.88977321  1.48587085  2.24404098  3.14864907 -0.02752454  2.3637593 ], Cost: 0.1343
Current parameters: [ 1.73454844  2.89453467  3.14245477  0.89934151  1.09240227  0.32498809
  2.9028213   1.48298608  2.24115898  3.08509294 -0.02828469  2.37709539], Cost: 0.1487
Current parameters: [ 1.74686083  2.88866416  3.1350372   0.92109979  1.10370602  0.32509092
  2.89904836  1.51140798  2.22535698  3.13801464 -0.02780972  2.36413966], Cost: 0.1211
Current parameters: [ 1.73374056  2.9011279   3.13509     0.91837037  1.12156355  0.31547596
  2.84862295  1.50522783  2.26346057  3.13292104 -0.03625004  2.36150799], Cost: 0.1802
Current parameters: [ 1.7491522   2.87721504  3.12530755  0.92144027  1.09517481  0.30581727
  2.89011119  1.48238232  2.24059433  3.13949267 -0.02020177  2.37208641], Cost: 0.1540
Current parameters: [ 1.75034737  2.91595261  3.10967333  0.93489517  1.11020862  0.32718828
  2.9064901   1.47612786  2.23435603  3.1411988  -0.0640769   2.38737409], Cost: 0.1600
Current parameters: [ 1.76777883  2.88244425  3.12575226  0.90632932  1.11246077  0.32629354
  2.89781929  1.48963361  2.24773997  3.13334873 -0.03379597  2.35638285], Cost: 0.1434
Current parameters: [ 1.73645373  2.89861139  3.1451169   0.89189696  1.09664353  0.29484411
```



```
  2.92455738  1.50618028  2.26416983  3.15699976 -0.04218899  2.36647236], Cost: 0.0815
Current parameters: [ 1.72083531  2.89206864  3.1450358   0.88188058  1.11427351  0.29593299
  2.92617094  1.49516485  2.2532839   3.16250772 -0.04166046  2.36437   ], Cost: 0.0851
Current parameters: [ 1.74723736  2.89650774  3.2011282   0.89947812  1.0947203   0.29800973
  2.94053129  1.50215261  2.26017187  3.17208031 -0.05008023  2.36636323], Cost: 0.0427
Current parameters: [ 1.72588152  2.87205944  3.20791575  0.91472318  1.0874534   0.31302101
  2.97612447  1.52177214  2.27972117  3.17807552 -0.05827506  2.35376335], Cost: 0.2150
Current parameters: [ 1.74897677  2.90315961  3.20168015  0.90231001  1.10630053  0.3053256
  2.94710978  1.50344558  2.26985066  3.17470424 -0.03014913  2.38016833], Cost: 0.0842
Current parameters: [ 1.76789753  2.91359555  3.20998138  0.86294021  1.08252692  0.28117405
  2.93093378  1.51126478  2.22934422  3.18228992 -0.0565323   2.3747623 ], Cost: 0.0229
Current parameters: [ 1.76186965  2.92043994  3.22031671  0.86405801  1.0914183   0.2762694
  2.90841164  1.51757905  2.23793254  3.1822703  -0.06412516  2.3728458 ], Cost: 0.0181
Current parameters: [ 1.76128748  2.92141524  3.22279945  0.86191157  1.0897689   0.27363144
  2.90784256  1.51833465  2.23831475  3.18094934 -0.05437808  2.36168298], Cost: 0.0197
Current parameters: [ 1.76192571  2.94328446  3.21768115  0.87485642  1.09367666  0.2714332
  2.91390947  1.50651431  2.23213336  3.18376758 -0.07172729  2.36534065], Cost: 0.0211
Current parameters: [ 1.75830185  2.92395171  3.2239874   0.85945493  1.09040359  0.279792
  2.91092829  1.51697335  2.2380638   3.16963419 -0.06547862  2.37450637], Cost: 0.0220
Current parameters: [ 1.75957283  2.91285858  3.22549616  0.8707662   1.08413615  0.25577653
  2.90305086  1.50411811  2.23495098  3.17614655 -0.05965363  2.38247268], Cost: 0.0349
Current parameters: [ 1.76858737  2.91759392  3.21967404  0.86487501  1.10055889  0.26993863
  2.91240249  1.5215469   2.23770156  3.17717302 -0.06627411  2.37087624], Cost: 0.0175
Current parameters: [ 1.78530509  2.91368462  3.22003762  0.85609373  1.1017885   0.27747929
  2.90531682  1.50311629  2.24657946  3.17708364 -0.07212081  2.36429766], Cost: 0.0238
Current parameters: [ 1.77225292  2.91623244  3.22024003  0.87108677  1.10228784  0.27615552
  2.90632892  1.52171771  2.22824126  3.17360984 -0.06324213  2.37083933], Cost: 0.0145
Current parameters: [ 1.77132283  2.90799647  3.22710368  0.87312269  1.094688    0.27983132
  2.90681874  1.53414123  2.21839594  3.17945961 -0.08147768  2.35791365], Cost: 0.0344
Current parameters: [ 1.78000868  2.91974804  3.21977848  0.8761139   1.09390659  0.27583577
  2.90560258  1.52729827  2.23404714  3.17081105 -0.06274267  2.37236755], Cost: 0.0100
Current parameters: [ 1.78970132  2.9310227   3.22961894  0.87590491  1.10406819  0.28295509
  2.90763417  1.53553851  2.23676765  3.18104239 -0.04996786  2.38491227], Cost: 0.0091
Current parameters: [ 1.79327659  2.935302    3.22498641  0.86638489  1.10142538  0.27860781
  2.90345623  1.53816905  2.22998231  3.18163941 -0.05054177  2.38690471], Cost: 0.0066
Current parameters: [ 1.79085922  2.93921823  3.2001285   0.87058916  1.10786592  0.28389353
  2.89460468  1.54606395  2.23773892  3.17866083 -0.04435402  2.38844312], Cost: 0.0128
Current parameters: [ 1.79078853  2.93102396  3.22493967  0.86936192  1.09758538  0.27723808
  2.89611155  1.53560616  2.23100883  3.18941546 -0.05267386  2.39512559], Cost: 0.0096
Current parameters: [ 1.80056584  2.93714925  3.22949031  0.87201104  1.10083852  0.25849023
  2.90338612  1.5334984   2.22566482  3.16681459 -0.03732225  2.39219861], Cost: 0.0078
```



```
Current parameters: [ 1.79854272  2.93560946  3.22901936  0.86815945  1.09825218  0.27789264
  2.90401344  1.54882582  2.22439937  3.18172878 -0.05482525  2.38211757], Cost: 0.0062
Current parameters: [ 1.79909395  2.93716975  3.23166104  0.86723624  1.10075711  0.27719285
  2.89884635  1.54840076  2.22712445  3.1804215  -0.05677056  2.38014203], Cost: 0.0036
Current parameters: [ 1.81161953  2.93573317  3.23177201  0.86363576  1.09998338  0.2808343
  2.89721638  1.54222806  2.22907157  3.17993235 -0.05834887  2.37681461], Cost: 0.0015
Current parameters: [ 1.81117453  2.93096055  3.2330762   0.86027969  1.09951895  0.28231135
  2.8964844   1.544845    2.23044957  3.17717549 -0.05661897  2.37849439], Cost: 0.0021
Current parameters: [ 1.81125872  2.94401323  3.23528067  0.86213399  1.09689261  0.28631272
  2.89605005  1.54018732  2.22821695  3.17209116 -0.06426421  2.38112604], Cost: 0.0036
Current parameters: [ 1.81019409  2.93660957  3.23288119  0.8641607   1.09701454  0.28344107
  2.89437205  1.54126386  2.22705303  3.18028029 -0.05392201  2.37393233], Cost: 0.0015
Current parameters: [ 1.81183073  2.94047363  3.23432021  0.85718114  1.0957028   0.27532306
  2.89866673  1.54320017  2.23782211  3.183539   -0.05756243  2.37119994], Cost: 0.0069
Current parameters: [ 1.81372211  2.93842468  3.23427869  0.86335859  1.10292466  0.28264719
  2.89776206  1.54406079  2.22949975  3.18264209 -0.05514375  2.37991569], Cost: 0.0039
Current parameters: [ 1.81019755  2.93207249  3.23244358  0.86048623  1.10837476  0.27952866
  2.89388123  1.53798732  2.22094321  3.17870275 -0.06208386  2.37084903], Cost: 0.0052
Current parameters: [ 1.8100723   2.93612244  3.22830367  0.86222969  1.10086159  0.28571671
  2.89673611  1.54425947  2.23042946  3.18246326 -0.06059295  2.37532078], Cost: 0.0013
Current parameters: [ 1.81423435  2.93738505  3.21920635  0.86796634  1.10219779  0.28370553
  2.89067714  1.54754329  2.2330458   3.17601407 -0.05800107  2.37428463], Cost: 0.0005
Current parameters: [ 1.81480588  2.93765172  3.21741573  0.86527322  1.09995191  0.28159947
  2.8867176   1.54772868  2.23077461  3.17873957 -0.05890559  2.3776878 ], Cost: 0.0002
Current parameters: [ 1.81508076  2.93147889  3.22069742  0.87458526  1.09687736  0.28340206
  2.88118908  1.54561372  2.23494353  3.18253429 -0.06404564  2.37928092], Cost: 0.0001
Current parameters: [ 1.81766583  2.93151677  3.22251294  0.87553841  1.09510979  0.28309723
  2.88184919  1.55095895  2.23195811  3.18267556 -0.0660376   2.37704006], Cost: 0.0001
Current parameters: [ 1.81763807  2.92712359  3.21261807  0.87749098  1.08992219  0.28567218
  2.88866535  1.54228795  2.23064365  3.18286491 -0.06316929  2.38153118], Cost: 0.0005
Current parameters: [ 1.81649592  2.93253105  3.220589    0.87420257  1.09486106  0.27786782
  2.88303014  1.54403047  2.23804379  3.18315187 -0.0657343   2.3771993 ], Cost: 0.0003

   Return from subroutine COBYLA because the MAXFUN limit has been reached.

   NFVALS =  100   F = 9.006812E-05    MAXCV = 0.000000E+00
   X = 1.815081E+00   2.931479E+00   3.220697E+00   8.745853E-01   1.096877E+00
       2.834021E-01   2.881189E+00   1.545614E+00   2.234944E+00   3.182534E+00
      -6.404564E-02   2.379281E+00
Current parameters: [ 1.81508076  2.93147889  3.22069742  0.87458526  1.09687736  0.28340206
  2.88118908  1.54561372  2.23494353  3.18253429 -0.06404564  2.37928092], Cost: 0.0001
```

```
Simulating for QAOA depth p = 7
Current parameters: [3.56960925 1.98669991 0.8931192  0.15045247 2.85196302 0.87560857
 2.25533667 0.3333034  1.77497699 2.97787091 0.06534674 0.3058676
 2.85372307 2.79920371], Cost: 2.9328
Current parameters: [2.56960925 2.98669991 0.8931192  0.15045247 2.85196302 0.87560857
 2.25533667 0.3333034  1.77497699 2.97787091 0.06534674 0.3058676
 2.85372307 2.79920371], Cost: 2.2635
Current parameters: [2.56960925 1.98669991 1.8931192  0.15045247 2.85196302 0.87560857
 2.25533667 0.3333034  1.77497699 2.97787091 0.06534674 0.3058676
 2.85372307 2.79920371], Cost: 2.0972
Current parameters: [2.56960925 1.98669991 0.8931192  1.15045247 2.85196302 0.87560857
 2.25533667 0.3333034  1.77497699 2.97787091 0.06534674 0.3058676
 2.85372307 2.79920371], Cost: 2.1391
Current parameters: [2.56960925 1.98669991 0.8931192  0.15045247 3.85196302 0.87560857
 2.25533667 0.3333034  1.77497699 2.97787091 0.06534674 0.3058676
 2.85372307 2.79920371], Cost: 2.0832
Current parameters: [2.56960925 1.98669991 0.8931192  0.15045247 2.85196302 1.87560857
 2.25533667 0.3333034  1.77497699 2.97787091 0.06534674 0.3058676
 2.85372307 2.79920371], Cost: 1.9774
Current parameters: [2.56960925 1.98669991 0.8931192  0.15045247 2.85196302 0.87560857
 3.25533667 0.3333034  1.77497699 2.97787091 0.06534674 0.3058676
 2.85372307 2.79920371], Cost: 1.9966
Current parameters: [2.56960925 1.98669991 0.8931192  0.15045247 2.85196302 0.87560857
 2.25533667 1.3333034  1.77497699 2.97787091 0.06534674 0.3058676
 2.85372307 2.79920371], Cost: 3.7772
Current parameters: [2.56960925 1.98669991 0.8931192  0.15045247 2.85196302 0.87560857
 2.25533667 0.3333034  2.77497699 2.97787091 0.06534674 0.3058676
 2.85372307 2.79920371], Cost: 0.7609
Current parameters: [2.56960925 1.98669991 0.8931192  0.15045247 2.85196302 0.87560857
 2.25533667 0.3333034  2.77497699 3.97787091 0.06534674 0.3058676
 2.85372307 2.79920371], Cost: 2.2544
Current parameters: [2.56960925 1.98669991 0.8931192  0.15045247 2.85196302 0.87560857
 2.25533667 0.3333034  2.77497699 2.97787091 1.06534674 0.3058676
 2.85372307 2.79920371], Cost: 2.8790
Current parameters: [2.56960925 1.98669991 0.8931192  0.15045247 2.85196302 0.87560857
 2.25533667 0.3333034  2.77497699 2.97787091 0.06534674 1.3058676
 2.85372307 2.79920371], Cost: 1.5394
Current parameters: [2.56960925 1.98669991 0.8931192  0.15045247 2.85196302 0.87560857
 2.25533667 0.3333034  2.77497699 2.97787091 0.06534674 0.3058676
 3.85372307 2.79920371], Cost: 3.2749
Current parameters: [2.56960925 1.98669991 0.8931192  0.15045247 2.85196302 0.87560857
```



```
 2.25533667 0.3333034  2.77497699 2.97787091 0.06534674 0.3058676
 2.85372307 3.79920371], Cost: 1.2396
Current parameters: [ 2.31911206  1.88301604  0.82590888  0.07406393  2.78782975  0.83468447
  2.21019245 -0.10241479  3.00088798  2.65027843 -0.39925377  0.1351178
  2.30228362  2.69420383], Cost: 2.3082
Current parameters: [3.06082527 1.97670652 0.88664125 0.14308989 2.84578165 0.87166417
 2.25098552 0.29130745 2.79675103 2.94629647 0.02056701 0.28941018
 2.80057352 2.78908348], Cost: 1.5543
Current parameters: [2.56960925 2.12268491 0.68333817 0.15045247 2.85196302 0.87560857
 2.25533667 0.3333034  2.77497699 2.97787091 0.06534674 0.3058676
 2.85372307 2.79920371], Cost: 1.0571
Current parameters: [2.55693127 2.39781292 1.17392919 0.14692046 2.84899766 0.87371635
 2.25324931 0.31315691 2.78542253 2.96272387 0.04386481 0.29797257
 2.82822593 2.79434879], Cost: 1.8194
Current parameters: [2.56960925 1.98669991 0.8931192  0.31120184 2.66049595 0.87560857
 2.25533667 0.3333034  2.77497699 2.97787091 0.06534674 0.3058676
 2.85372307 2.79920371], Cost: 0.7622
Current parameters: [2.55969036 1.9683281  0.89241125 0.53211119 3.17244189 0.87412815
 2.25370358 0.31754137 2.78314928 2.9660203  0.04853989 0.29969076
 2.83377484 2.79540536], Cost: 1.2362
Current parameters: [2.56960925 1.98669991 0.8931192  0.15045247 2.85196302 1.0608305
 2.08742943 0.3333034  2.77497699 2.97787091 0.06534674 0.3058676
 2.85372307 2.79920371], Cost: 0.5981
Current parameters: [2.56360768 1.97558378 0.89269085 0.14711125 2.84918865 1.39854249
 2.45531094 0.32376635 2.77992175 2.97070052 0.05517751 0.30213021
 2.8416531  2.79690546], Cost: 2.2342
Current parameters: [2.53239297 1.90500699 0.94134354 0.06264511 2.88910041 0.99771565
 2.14421859 0.51527777 2.79620255 2.95664534 0.04412118 0.28464204
 2.83249751 2.77797815], Cost: 1.1498
Current parameters: [2.60871062 2.0956799  0.75015054 0.36441097 2.7143645  1.14981138
 1.77107643 0.51484753 2.76979648 2.9580148  0.01175571 0.32463022
 2.77875005 2.83415566], Cost: 1.0616
Current parameters: [2.4901289  1.90441685 0.87890585 0.11710826 2.82493236 1.00674069
 2.00228164 0.17184021 2.80314756 2.93708893 0.00757185 0.28453859
 2.78517767 2.78602957], Cost: 1.1823
Current parameters: [2.57057922 1.98908135 0.8916408  0.15303197 2.85081752 1.06269252
 2.08570769 0.3331244  2.88142649 3.04320595 0.06598072 0.30650158
 2.85435705 2.79983769], Cost: 0.5699
Current parameters: [2.56881553 1.96974032 0.897237   0.14458844 2.85172268 1.04678422
 2.06974288 0.32783    2.75487111 3.25445908 0.04813083 0.29897932
 2.83345514 2.79462626], Cost: 0.0596
```



```
Current parameters: [ 2.56922291  1.97074052  0.89661607  0.14567182  2.85124157  1.04756626
  2.06901975  0.32775482  2.75491009  3.25440256 -0.04817295  0.29924558
  2.91311905  2.79489253], Cost: 0.2486
Current parameters: [2.57365066 1.94144564 0.89764867 0.14740399 2.84286126 1.02255838
 2.01006512 0.3119002  2.72610694 3.30706271 0.21800571 0.28509622
 2.98992656 2.78692643], Cost: 1.0741
Current parameters: [2.56646619 2.00614011 0.91805366 0.14670414 2.85045465 1.04835907
 2.06800867 0.32620579 2.75571323 3.25323793 0.04639895 0.18134757
 2.83139956 2.79520914], Cost: 0.1472
Current parameters: [2.54284305 2.13622011 1.04415568 0.12005025 2.85649705 1.01501093
 2.05261949 0.31210111 2.7427999  3.27791437 0.03481972 0.38553198
 2.79009055 2.78438696], Cost: 0.2213
Current parameters: [2.6016398  1.97121311 0.89338224 0.14823966 2.84951865 1.04950433
 2.06673463 0.32495633 2.75636105 3.25229853 0.04506667 0.29785318
 2.82981825 2.67438953], Cost: 0.2101
Current parameters: [2.79194724 1.94490946 0.92493535 0.1114734  2.85851564 1.01249765
 2.06287791 0.30233356 2.75248961 3.26250249 0.02361337 0.29903584
 2.78309341 2.86607674], Cost: 0.2377
Current parameters: [2.56689032 1.95630254 0.91232873 0.22034054 2.94140076 1.01086776
 2.05986111 0.32477066 2.75645732 3.25215893 0.0448687  0.29778042
 2.82958327 2.79388901], Cost: 0.0860
Current parameters: [2.55390964 1.96170978 0.89220649 0.20886391 2.8697115  1.21840931
 2.19510256 0.30258396 2.72581625 3.30899091 0.02842152 0.31622993
 2.75262534 2.8244272 ], Cost: 0.1457
Current parameters: [2.58624003 2.00138911 0.87118836 0.18571241 2.83523098 1.08283822
 2.00526581 0.3696398  2.69705481 3.22367375 0.0445932  0.30570567
 2.82688429 2.8036492 ], Cost: 0.0792
Current parameters: [ 2.53101803e+00  1.96434412e+00  8.33148464e-01  1.87730895e-01
  2.79212397e+00  1.00376649e+00  2.01171598e+00  4.03693536e-01
  2.87973207e+00  3.35861862e+00 -9.98588870e-04  3.04099968e-01
  2.72744081e+00  2.81536909e+00], Cost: 0.1407
Current parameters: [2.5694426  2.0287074  0.81042402 0.114988   2.89129411 1.01927279
 2.0999792  0.31909069 2.73794516 3.25901254 0.04864952 0.30292525
 2.82512401 2.80000732], Cost: 0.1286
Current parameters: [2.56597983 1.96550627 0.89839199 0.11396488 2.88472312 1.07511285
 2.0411888  0.33051197 2.76837393 3.26035542 0.04625797 0.29788202
 2.83135308 2.79370251], Cost: 0.0458
Current parameters: [2.52822691 1.93110954 0.88983982 0.10244904 2.87183001 1.05973541
 2.0051073  0.25219621 2.73225804 3.24049012 0.01938712 0.29477222
 2.78106194 2.79730761], Cost: 0.1693
Current parameters: [2.55243558 1.93977137 0.91162236 0.098321   2.87337475 1.05497741
```



```
 2.06467852 0.36039092 2.74730698 3.25093247 0.05088722 0.30186666
 2.82254267 2.79750581], Cost: 0.0380
Current parameters: [2.55662916 1.95285992 0.90781792 0.07293819 2.94928785 1.08025632
 2.04135809 0.42147071 2.73103128 3.25487875 0.00820203 0.27921974
 2.78698803 2.78262543], Cost: 0.0727
Current parameters: [2.5450762  1.97333072 0.94645938 0.09231665 2.87039104 1.0441488
 2.0608781  0.35539576 2.73180957 3.27691319 0.04834088 0.32047686
 2.81613323 2.79625656], Cost: 0.0713
Current parameters: [2.5392274  1.97736093 0.93886759 0.09515233 2.87408568 1.062978
 2.07895226 0.36996982 2.80540128 3.16211543 0.05432626 0.32963835
 2.79645186 2.81157628], Cost: 0.0933
Current parameters: [2.55777593 1.93982058 0.92303759 0.09949152 2.88878743 1.08982889
 2.09467999 0.34179409 2.71890399 3.23932235 0.05695671 0.30302742
 2.8308007  2.79769936], Cost: 0.0241
Current parameters: [2.5392852  1.92324742 0.90885513 0.1403945  2.86208206 1.14076067
 2.13845153 0.35863905 2.74915913 3.29252362 0.04501803 0.31541934
 2.77484574 2.81901852], Cost: 0.0137
Current parameters: [2.48632598 1.93015527 0.90019419 0.14763098 2.86616809 1.15025272
 2.13392271 0.36513266 2.74821411 3.2923735  0.05579135 0.3134176
 2.7927092  2.80121653], Cost: 0.0112
Current parameters: [2.50236477 1.89778474 0.89452752 0.14006862 2.8880795  1.17610904
 2.11774892 0.39070792 2.75374404 3.31614568 0.15012159 0.32111824
 2.81671203 2.84634893], Cost: 0.2472
Current parameters: [2.48326058 1.92190722 0.90165271 0.14839611 2.85965585 1.15451022
 2.14290574 0.37060248 2.75260253 3.29333953 0.01174854 0.32196146
 2.83028007 2.81572283], Cost: 0.0124
Current parameters: [2.49085183 1.92328788 0.89396319 0.1471427  2.8695239  1.15141389
 2.13176688 0.36569671 2.74794734 3.29230739 0.05560808 0.33877069
 2.79234396 2.78675079], Cost: 0.0329
Current parameters: [2.49771016 1.92937511 0.91187124 0.15216798 2.84541357 1.15373934
 2.15119264 0.36871273 2.75599726 3.29239662 0.04844266 0.28278722
 2.79763006 2.75893044], Cost: 0.0327
Current parameters: [2.48697498 1.93929783 0.88779375 0.12586525 2.85294063 1.15928588
 2.13461021 0.36628342 2.74608388 3.29313836 0.05629255 0.31413182
 2.79238047 2.8020438 ], Cost: 0.0125
Current parameters: [2.48580618 1.95059306 0.86851104 0.15460473 2.88158188 1.12155172
 2.1697882  0.35965342 2.74468368 3.2951566  0.05277287 0.30885756
 2.79289505 2.80404228], Cost: 0.0074
Current parameters: [2.49240599 1.96038045 0.86311914 0.17082095 2.87161613 1.12412579
 2.16411986 0.37271596 2.73134994 3.28837417 0.05398601 0.31073443
 2.79487247 2.80598576], Cost: 0.0015
```

```
Current parameters: [2.47507834 1.94771238 0.85122265 0.17836621 2.85935509 1.12047202
 2.15354017 0.34386256 2.70628632 3.27308612 0.03062241 0.29799924
 2.77187454 2.8123163 ], Cost: 0.0801
Current parameters: [2.48969636 1.96266979 0.86787871 0.17567392 2.85547554 1.11952866
 2.17092365 0.360992   2.74182985 3.27666027 0.06348129 0.31931176
 2.79902579 2.81092077], Cost: 0.0030
Current parameters: [2.48790074 1.98225568 0.87324494 0.16570673 2.89072269 1.13529745
 2.17024515 0.3947824  2.75334745 3.25268698 0.04070766 0.31210783
 2.77714341 2.80978571], Cost: 0.0766
Current parameters: [2.48497355 1.95167501 0.87352937 0.16252069 2.86575128 1.11308919
 2.17512276 0.38669331 2.71932791 3.28674669 0.05594964 0.31479464
 2.78985291 2.80769506], Cost: 0.0011
Current parameters: [2.48125463 1.99023306 0.90298862 0.16354629 2.86242477 1.10703511
 2.17206431 0.37532369 2.7080787  3.31894266 0.04998881 0.32709358
 2.79008316 2.80700006], Cost: 0.0165
Current parameters: [2.47967056 1.94776385 0.86816124 0.17194394 2.85672404 1.11888832
 2.18009781 0.39396462 2.73149955 3.30133705 0.05205986 0.31750624
 2.77520986 2.8126806 ], Cost: 0.0050
Current parameters: [2.48347605 1.95498508 0.86499122 0.15535981 2.85481163 1.07084227
 2.14145179 0.40113994 2.73998432 3.29022973 0.05132158 0.30936586
 2.79432669 2.80419773], Cost: 0.0119
Current parameters: [2.49583736 1.94091855 0.86916782 0.16493959 2.86818714 1.11667844
 2.18048532 0.39759882 2.71601285 3.30927036 0.09195865 0.3017559
 2.82102204 2.83060076], Cost: 0.0502
Current parameters: [2.49182881 1.95095696 0.87795075 0.16533654 2.85557458 1.11379505
 2.18444899 0.38832878 2.72212384 3.28841155 0.05330515 0.30123309
 2.79378059 2.78553804], Cost: 0.0033
Current parameters: [2.47188663 1.9532185  0.87118984 0.16431206 2.86687564 1.11548467
 2.17391788 0.38832168 2.71907781 3.28670588 0.05870093 0.31499583
 2.79424404 2.80282202], Cost: 0.0021
Current parameters: [2.49267002 1.94504598 0.8661721  0.16292919 2.86850729 1.11201525
 2.17490673 0.38675279 2.71731832 3.28960546 0.0573039  0.33901465
 2.79155683 2.79331695], Cost: 0.0124
Current parameters: [2.4856736  1.95367382 0.87314207 0.16234536 2.86732648 1.11202955
 2.17281746 0.38521813 2.71814522 3.28644941 0.06687343 0.31261421
 2.78037591 2.80409011], Cost: 0.0036
Current parameters: [2.47923197 1.94257485 0.86702066 0.16704288 2.85717208 1.10997447
 2.17040796 0.37074002 2.70351265 3.28471322 0.04555598 0.30834362
 2.78425771 2.80934489], Cost: 0.0180
Current parameters: [2.48460827 1.94726405 0.87985738 0.17333582 2.87250596 1.10870929
 2.17478661 0.38630902 2.7205927  3.28605539 0.05558957 0.31445589
```



```
 2.78986403 2.80731033], Cost: 0.0037
Current parameters: [2.48464285 1.96384735 0.87637599 0.15818441 2.87203117 1.12006546
 2.17940108 0.39740984 2.72628533 3.26717504 0.04591208 0.31414219
 2.7830606  2.80886037], Cost: 0.0202
Current parameters: [2.48460999 1.95888417 0.86760016 0.16423428 2.86930939 1.10576343
 2.18365783 0.38464903 2.71785188 3.28933207 0.05486866 0.31441477
 2.78990994 2.80833723], Cost: 0.0007
Current parameters: [2.4831261  1.97602541 0.88585341 0.1632039  2.86563269 1.1068764
 2.17834228 0.3799553  2.71256743 3.30342662 0.05104102 0.32068732
 2.79007158 2.80753172], Cost: 0.0031
Current parameters: [2.48561906 1.95896142 0.86659865 0.15998511 2.86534812 1.09649168
 2.17501953 0.38865776 2.72236176 3.28964664 0.05260092 0.31344529
 2.79195887 2.80933337], Cost: 0.0003
Current parameters: [2.47600227 1.96825124 0.85886835 0.17582841 2.87215973 1.0849922
 2.17451225 0.3876835  2.72800396 3.29216731 0.05650841 0.30997877
 2.78546499 2.79520804], Cost: 0.0035
Current parameters: [2.49000471 1.95995417 0.86355683 0.1630115  2.86651951 1.09635371
 2.17837533 0.39599727 2.72209487 3.30162721 0.0693643  0.30436238
 2.80848292 2.81888588], Cost: 0.0152
Current parameters: [2.48207376 1.95490583 0.86911467 0.15217687 2.87068011 1.09481532
 2.17808613 0.38228113 2.72928616 3.29338498 0.05220267 0.31193818
 2.79130828 2.80801309], Cost: 0.0005
Current parameters: [2.48604956 1.96037011 0.86804797 0.15924655 2.86592851 1.09440924
 2.17488892 0.38544098 2.72095055 3.28508768 0.05489004 0.31376312
 2.79537558 2.80815416], Cost: 0.0006
Current parameters: [2.48351106 1.95895359 0.8677286  0.16353087 2.85572176 1.0959755
 2.17912299 0.38541194 2.72997015 3.28800643 0.0556028  0.31779592
 2.7907574  2.81328055], Cost: 0.0006
Current parameters: [2.48347362 1.95967794 0.8652603  0.1601098  2.86874988 1.09598438
 2.17292338 0.38816773 2.7218997  3.28919756 0.05386234 0.31686558
 2.79078615 2.81408403], Cost: 0.0010
Current parameters: [2.48233051 1.95479655 0.86329161 0.160606   2.86009869 1.09464946
 2.17287877 0.38050067 2.7155661  3.29022082 0.04720644 0.30924437
 2.78952058 2.81117258], Cost: 0.0040
Current parameters: [2.48736098 1.9562546  0.86473351 0.16012873 2.86487614 1.09569662
 2.17455657 0.38759443 2.7211762  3.2923671  0.05360853 0.31848819
 2.79327494 2.80650669], Cost: 0.0019
Current parameters: [2.48704375 1.96285054 0.86645553 0.15543766 2.86620755 1.09930562
 2.1778031  0.39299941 2.72640604 3.28170615 0.04455816 0.3166394
 2.78881944 2.80671058], Cost: 0.0026
Current parameters: [2.48831053 1.96064563 0.86421331 0.16116712 2.8659909  1.09982197
```



```
 2.17211277 0.38554287 2.72535319 3.29012423 0.0516786  0.31247927
 2.79374215 2.80960002], Cost: 0.0002
Current parameters: [2.48993772 1.96280824 0.86360459 0.15980814 2.86644818 1.10066225
 2.17677353 0.39153445 2.72762347 3.29329751 0.0569068  0.3052399
 2.80109893 2.81479358], Cost: 0.0029
Current parameters: [2.48786557 1.96460983 0.86912075 0.16083004 2.8646416  1.09985143
 2.17110686 0.38613043 2.72391531 3.29384954 0.05045011 0.31261444
 2.79331822 2.80929258], Cost: 0.0003
Current parameters: [2.4886298  1.96634162 0.86348723 0.16039546 2.86905685 1.10230247
 2.17148264 0.37492024 2.72834289 3.28884986 0.0523823  0.31980002
 2.79765241 2.81091923], Cost: 0.0006
Current parameters: [2.48593007 1.96343183 0.86258898 0.16494935 2.86780719 1.09720835
 2.17223721 0.38550306 2.72724789 3.29060874 0.05256998 0.31114293
 2.7921967  2.80617635], Cost: 0.0002
Current parameters: [2.48575206 1.96067895 0.86369714 0.16021991 2.86515263 1.10151907
 2.17099813 0.38611436 2.72531651 3.28991629 0.05246034 0.31257956
 2.7945543  2.8085651 ], Cost: 0.0002
Current parameters: [2.48818888 1.96172824 0.86363871 0.15792725 2.86449541 1.10243762
 2.16935716 0.38517793 2.72546123 3.28933026 0.05699853 0.31162494
 2.78992839 2.80777703], Cost: 0.0004
Current parameters: [2.48557366 1.95888216 0.86527234 0.16198288 2.86566136 1.10165724
 2.16950311 0.38525274 2.7254567  3.29045983 0.05391347 0.31217307
 2.79519882 2.80894783], Cost: 0.0003
Current parameters: [2.48629011 1.96055256 0.86665092 0.16054152 2.86653725 1.10348225
 2.17012618 0.38827007 2.72840281 3.28579265 0.0494941  0.3133892
 2.79306157 2.80698052], Cost: 0.0006
Current parameters: [2.48506205 1.96057377 0.86408419 0.16109923 2.86232988 1.10123814
 2.17209386 0.38575074 2.7269417  3.28958319 0.05281172 0.31307029
 2.79392619 2.80954384], Cost: 0.0001
Current parameters: [2.48331149 1.96029137 0.862872   0.16109265 2.8606844  1.10100541
 2.17089393 0.38194557 2.72449907 3.28846605 0.04911415 0.30946317
 2.79208911 2.81022792], Cost: 0.0013
Current parameters: [2.48412529 1.95985303 0.8648152  0.15910653 2.8634664  1.1008204
 2.17303724 0.38418109 2.72875535 3.29061879 0.0526132  0.31285276
 2.79380839 2.80927141], Cost: 0.0001
Current parameters: [2.48584086 1.96415844 0.8631254  0.16171866 2.86327893 1.10262376
 2.17165197 0.38167199 2.72766402 3.28857645 0.05358475 0.31738134
 2.79623535 2.81030111], Cost: 0.0003
Current parameters: [2.48467833 1.96049339 0.86456021 0.16205572 2.86303728 1.10347663
 2.17461087 0.38537942 2.72587386 3.28970061 0.05323795 0.31275108
 2.79322672 2.80931523], Cost: 0.0001
```



```
Current parameters: [2.48562605 1.96285819 0.86399678 0.16140368 2.8634664  1.10408827
 2.17682162 0.38824866 2.72805999 3.29022088 0.05556627 0.30931775
 2.79675049 2.8116494 ], Cost: 0.0006
Current parameters: [2.4857008  1.95936897 0.86383466 0.16210202 2.86211551 1.10344186
 2.17557223 0.38606775 2.72584429 3.29140918 0.05334943 0.31434934
 2.79355086 2.80717524], Cost: 0.0002
Current parameters: [2.48466377 1.96045439 0.86388065 0.16234363 2.86332151 1.10420362
 2.17412408 0.38565852 2.72643251 3.29063728 0.05279744 0.31292071
 2.79260248 2.81005156], Cost: 0.0000
Current parameters: [2.48309276 1.96025291 0.86402302 0.16198315 2.86429399 1.10294667
 2.17477537 0.38690527 2.72544619 3.29048112 0.05368538 0.31478667
 2.79161104 2.81140997], Cost: 0.0002
Current parameters: [2.48479534 1.95945852 0.86278106 0.16244249 2.86370359 1.10426814
 2.17433435 0.38558365 2.7268937  3.28957566 0.05300501 0.31291468
 2.79265912 2.81007831], Cost: 0.0001
Current parameters: [2.48466377 1.96045439 0.86388065 0.16234363 2.86332151 1.10420362
 2.17412408 0.38565852 2.72643251 3.29063728 0.05279744 0.31292071
 2.79260248 2.81005156], Cost: 0.0000

   Return from subroutine COBYLA because the MAXFUN limit has been reached.

   NFVALS =  100   F = 4.720467E-05    MAXCV = 0.000000E+00
   X = 2.484664E+00   1.960454E+00   8.638807E-01   1.623436E-01   2.863322E+00
       1.104204E+00   2.174124E+00   3.856585E-01   2.726433E+00   3.290637E+00
       5.279744E-02   3.129207E-01   2.792602E+00   2.810052E+00
Simulating for QAOA depth p = 8
Current parameters: [2.43477707 2.26834147 1.5717784  1.36759096 1.32483571 1.31190884
 0.5024131  1.66529481 2.13363666 1.73357812 0.71221244 0.53680235
 1.95211138 0.78050706 1.83370902 2.89594829], Cost: 1.1910
Current parameters: [2.43477707 3.26834147 1.5717784  1.36759096 1.32483571 1.31190884
 0.5024131  1.66529481 2.13363666 1.73357812 0.71221244 0.53680235
 1.95211138 0.78050706 1.83370902 2.89594829], Cost: 1.7403
Current parameters: [2.43477707 2.26834147 2.5717784  1.36759096 1.32483571 1.31190884
 0.5024131  1.66529481 2.13363666 1.73357812 0.71221244 0.53680235
 1.95211138 0.78050706 1.83370902 2.89594829], Cost: 1.2633
Current parameters: [2.43477707 2.26834147 1.5717784  2.36759096 1.32483571 1.31190884
 0.5024131  1.66529481 2.13363666 1.73357812 0.71221244 0.53680235
 1.95211138 0.78050706 1.83370902 2.89594829], Cost: 2.7699
Current parameters: [2.43477707 2.26834147 1.5717784  1.36759096 2.32483571 1.31190884
 0.5024131  1.66529481 2.13363666 1.73357812 0.71221244 0.53680235
 1.95211138 0.78050706 1.83370902 2.89594829], Cost: 1.7588
```

```
Current parameters: [2.43477707 2.26834147 1.5717784  1.36759096 1.32483571 2.31190884
 0.5024131  1.66529481 2.13363666 1.73357812 0.71221244 0.53680235
 1.95211138 0.78050706 1.83370902 2.89594829], Cost: 1.7101
Current parameters: [2.43477707 2.26834147 1.5717784  1.36759096 1.32483571 1.31190884
 1.5024131  1.66529481 2.13363666 1.73357812 0.71221244 0.53680235
 1.95211138 0.78050706 1.83370902 2.89594829], Cost: 3.2531
Current parameters: [2.43477707 2.26834147 1.5717784  1.36759096 1.32483571 1.31190884
 0.5024131  2.66529481 2.13363666 1.73357812 0.71221244 0.53680235
 1.95211138 0.78050706 1.83370902 2.89594829], Cost: 1.6285
Current parameters: [2.43477707 2.26834147 1.5717784  1.36759096 1.32483571 1.31190884
 0.5024131  1.66529481 3.13363666 1.73357812 0.71221244 0.53680235
 1.95211138 0.78050706 1.83370902 2.89594829], Cost: 3.5561
Current parameters: [2.43477707 2.26834147 1.5717784  1.36759096 1.32483571 1.31190884
 0.5024131  1.66529481 2.13363666 2.73357812 0.71221244 0.53680235
 1.95211138 0.78050706 1.83370902 2.89594829], Cost: 2.3681
Current parameters: [2.43477707 2.26834147 1.5717784  1.36759096 1.32483571 1.31190884
 0.5024131  1.66529481 2.13363666 1.73357812 1.71221244 0.53680235
 1.95211138 0.78050706 1.83370902 2.89594829], Cost: 2.1000
Current parameters: [2.43477707 2.26834147 1.5717784  1.36759096 1.32483571 1.31190884
 0.5024131  1.66529481 2.13363666 1.73357812 0.71221244 1.53680235
 1.95211138 0.78050706 1.83370902 2.89594829], Cost: 1.5768
Current parameters: [2.43477707 2.26834147 1.5717784  1.36759096 1.32483571 1.31190884
 0.5024131  1.66529481 2.13363666 1.73357812 0.71221244 0.53680235
 2.95211138 0.78050706 1.83370902 2.89594829], Cost: 2.8415
Current parameters: [2.43477707 2.26834147 1.5717784  1.36759096 1.32483571 1.31190884
 0.5024131  1.66529481 2.13363666 1.73357812 0.71221244 0.53680235
 1.95211138 1.78050706 1.83370902 2.89594829], Cost: 2.0410
Current parameters: [2.43477707 2.26834147 1.5717784  1.36759096 1.32483571 1.31190884
 0.5024131  1.66529481 2.13363666 1.73357812 0.71221244 0.53680235
 1.95211138 0.78050706 2.83370902 2.89594829], Cost: 1.6973
Current parameters: [2.43477707 2.26834147 1.5717784  1.36759096 1.32483571 1.31190884
 0.5024131  1.66529481 2.13363666 1.73357812 0.71221244 0.53680235
 1.95211138 0.78050706 1.83370902 3.89594829], Cost: 1.4153
Current parameters: [2.52761046 2.14462677 1.55550377 1.01197849 1.19695082 1.19499116
 0.03796394 1.56675929 1.6009395  1.4684616  0.50747464 0.44992032
 1.58037891 0.5890712  1.71968215 2.84544037], Cost: 0.7773
Current parameters: [ 2.55976145  2.10178065  1.54986738  0.88881921  1.15266044  1.15449906
 -0.12288876  1.53263348  2.00602183  1.37664378  0.43656778  0.41983047
  1.45163678  0.52277121  1.68019122  2.82794797], Cost: 0.9009
Current parameters: [2.52761046 2.14462677 1.55550377 1.01197849 1.19695082 1.19499116
 0.19418207 1.56675929 1.6009395  1.4684616  0.50747464 0.44992032
```



```
 1.38519721 0.5890712  1.71968215 2.84544037], Cost: 0.4855
Current parameters: [2.58166038 2.07259698 1.54602827 0.80493202 1.12249304 1.12691876
 0.53699635 1.50938945 1.54160043 1.31410413 0.38827117 0.39933544
 1.46349492 0.47761246 1.65329285 2.8160334 ], Cost: 1.5439
Current parameters: [2.52761046 2.14462677 1.55550377 1.16140331 1.19695082 1.19499116
 0.19418207 1.56675929 1.6009395  1.26803151 0.50747464 0.44992032
 1.38519721 0.5890712  1.71968215 2.84544037], Cost: 1.3428
Current parameters: [2.56146313 2.09951291 1.54956906 1.10892042 1.15031625 1.1523559
 0.12211189 1.53082728 1.56377412 1.89205285 0.43281485 0.41823788
 1.20470397 0.51926211 1.67810105 2.82702213], Cost: 1.0430
Current parameters: [2.52761046 2.14462677 1.55550377 1.01197849 1.19695082 1.19499116
 0.19418207 1.56675929 1.6009395  1.4684616  0.67821966 0.44992032
 1.38519721 0.40646195 1.71968215 2.84544037], Cost: 0.3230
Current parameters: [2.54521426 2.12116701 1.55241765 0.81207682 1.17270027 1.1728203
 0.1567047  1.54807421 1.58161305 1.50212091 1.01924873 0.43344507
 1.29133852 0.6873334  1.69805947 2.83586266], Cost: 0.7786
Current parameters: [2.52761046 1.96494473 1.55550377 1.01197849 1.37077362 1.19499116
 0.19418207 1.56675929 1.6009395  1.4684616  0.67821966 0.44992032
 1.38519721 0.40646195 1.71968215 2.84544037], Cost: 0.3269
Current parameters: [2.53779711 2.47698274 1.55371795 0.89630293 1.53996221 1.18216172
 0.17249533 1.55594694 1.58975601 1.48793898 0.64504415 0.44038671
 1.33088472 0.35345227 1.70716992 2.83989811], Cost: 0.0412
Current parameters: [2.53779711 2.47698274 1.55371795 0.89630293 1.53996221 1.35671197
 0.17249533 1.55594694 1.58975601 1.48793898 0.64504415 0.44038671
 1.33088472 0.35345227 1.52819447 2.83989811], Cost: 0.5957
Current parameters: [2.54726161 2.46997107 1.55205872 0.78882793 1.53220535 1.48895048
 0.15234602 1.5459011  1.57936535 1.50603555 0.61422054 0.43152897
 1.2804226  0.30420058 2.07751912 2.83474876], Cost: 1.3096
Current parameters: [2.71976038 2.47698274 1.55371795 0.89630293 1.53996221 1.18216172
 0.17249533 1.72738021 1.58975601 1.48793898 0.64504415 0.44038671
 1.33088472 0.35345227 1.70716992 2.83989811], Cost: 0.1395
Current parameters: [2.20067196 2.4718852  1.55251168 0.81816775 1.53432292 1.11162853
 0.15784662 1.90420796 1.58220192 1.50109533 0.62263514 0.43394706
 1.29419836 0.3176459  1.69010427 2.83615449], Cost: 0.7207
Current parameters: [2.53779711 2.47698274 1.55371795 0.89630293 1.53996221 1.18216172
 0.17249533 1.55594694 1.58975601 1.48793898 0.64504415 0.56603274
 1.33088472 0.35345227 1.70716992 2.62376603], Cost: 1.0414
Current parameters: [2.55527521 2.47343911 1.55287939 0.84198617 1.53604198 1.13312959
 0.16231207 1.53074154 1.58450468 1.4970848  0.62946621 0.8402739
 1.30538168 0.32856097 1.6953065  3.12607645], Cost: 0.3484
Current parameters: [2.5337925  2.4823195  1.30746679 0.91164322 1.54547887 1.18720528
```



```
 0.19253058 1.56019753 1.61273532 1.49937548 0.65387606 0.4441346
 1.34692039 0.36171036 1.71208877 2.84207691], Cost: 0.1451
Current parameters: [2.50515878 2.54029586 1.64339667 1.03400891 1.60515144 1.19615369
 0.41331626 1.58391305 1.87241485 1.64041503 0.74007481 0.44397525
 1.50621804 0.43194721 1.75689783 2.89588003], Cost: 1.9755
Current parameters: [2.55376845 2.45569849 1.55091801 0.82251621 1.51796051 1.16204685
 0.10381585 1.53899459 1.79145263 1.44860936 0.60123476 0.42543927
 1.25981399 0.32969932 1.68755239 2.83120858], Cost: 0.1363
Current parameters: [ 2.60773138  2.472682    1.53315687  0.9037034   1.53435889  0.97245674
 -0.02410437  1.45176561  1.45056564  1.44523552  0.74295885  0.25401052
  1.37439789  0.10034026  1.66450569  2.97331539], Cost: 2.1173
Current parameters: [2.56235151 2.45274629 1.54862092 0.74154025 1.5147983  1.13962664
 0.3200619  1.5270252  1.54988164 1.47614933 0.55571939 0.40586306
 1.35706595 0.35578038 1.68113709 2.84290678], Cost: 0.2203
Current parameters: [2.54043947 2.47346139 1.55325472 0.94260896 1.53632218 1.17883385
 0.17770415 1.55314228 1.58685508 1.55342234 0.56396484 0.43791376
 1.30325993 0.31162349 1.70392433 2.83846049], Cost: 0.1320
Current parameters: [2.55193865 2.47097836 1.55003971 0.85970555 1.53358823 1.14678208
 0.14710036 1.53661024 1.55468391 1.59639571 0.69283403 0.40968388
 1.19303102 0.50498284 1.69604541 2.85697277], Cost: 0.0395
Current parameters: [2.55193865 2.47097836 1.55003971 0.88682846 1.53358823 1.14678208
 0.21075023 1.53661024 1.55468391 1.56001455 0.73140991 0.40968388
 1.11350575 0.46372651 1.69604541 2.85697277], Cost: 0.0731
Current parameters: [2.54714338 2.49474337 1.549258   0.94360305 1.55792805 1.12904993
 0.13819931 1.53584617 1.53350145 1.44822674 0.57608667 0.39053745
 1.16593663 0.62844291 1.71038564 2.89309405], Cost: 0.5429
Current parameters: [2.55198879 2.56075242 1.55003092 0.85975925 1.4466079  1.14671893
 0.14703906 1.53655702 1.55466598 1.59638933 0.69290546 0.40963696
 1.19309143 0.50487954 1.69598382 2.85694549], Cost: 0.0840
Current parameters: [2.58850701 2.46002718 1.53941933 0.84512389 1.53628932 1.0390465
 0.0432109  1.48260663 1.48219232 1.58765429 0.76229314 0.31413577
 1.2078579  0.40075723 1.67305452 2.92403172], Cost: 0.4957
Current parameters: [2.55060823 2.48246119 1.56199793 0.90160994 1.54553197 1.08258189
 0.18438075 1.53802238 1.59559628 1.61338009 0.71245258 0.41092902
 1.22785277 0.52629214 1.63343258 2.85769661], Cost: 0.0673
Current parameters: [2.55072542 2.49011471 1.58979764 0.86906527 1.56585525 1.21294218
 0.21821872 1.52370594 1.64360722 1.67146745 0.74951667 0.35352074
 1.24683187 0.5241003  1.83726258 2.93887962], Cost: 1.4592
Current parameters: [2.49971326 2.47091726 1.5500334  0.85929671 1.53352312 1.14654289
 0.14702371 1.59204348 1.55464439 1.59646455 0.69271678 0.32399399
 1.19283906 0.50479548 1.69581214 2.80715788], Cost: 0.1490
```



```
Current parameters: [2.42711713 2.46276507 1.54939717 0.80532664 1.52902981 1.09785053
 0.12645458 1.66447948 1.53669992 1.60351524 0.68628247 0.52032037
 1.16657815 0.48252533 1.67328571 2.958576  ], Cost: 0.0893
Current parameters: [2.54300627 2.39081572 1.55737985 0.88972421 1.45086531 1.14764351
 0.1500737  1.53658765 1.55370276 1.58291629 0.71150039 0.41034073
 1.21368694 0.51882912 1.70598866 2.86489277], Cost: 0.0188
Current parameters: [2.62327026 2.3322989  1.47108251 0.86534291 1.3983481  1.13471682
 0.15864863 1.56174769 1.5755211  1.68882167 0.59574733 0.41130824
 1.11811832 0.51127978 1.62731061 2.84280674], Cost: 0.3827
Current parameters: [2.56585374 2.38265635 1.55847993 0.85182675 1.50083259 1.16331875
 0.11397028 1.50410993 1.53230034 1.6006419  0.70815445 0.46901185
 1.21788879 0.53450068 1.72915653 2.8059646 ], Cost: 0.2276
Current parameters: [2.47659609 2.39510389 1.57531308 0.95039998 1.34466061 1.03562489
 0.18145156 1.58200624 1.54468029 1.51754037 0.77144648 0.45957535
 1.21145225 0.48466003 1.68746087 2.74118211], Cost: 0.5609
Current parameters: [2.52870681 2.39044694 1.56980416 0.89142752 1.44645303 1.22363038
 0.14104967 1.53197598 1.53911885 1.5643672  0.73632035 0.41296004
 1.22290861 0.51757249 1.61621267 2.87129222], Cost: 0.0739
Current parameters: [2.54360844 2.39639997 1.55130654 0.93185716 1.4573423  1.15510137
 0.11398965 1.546468   1.56669191 1.58853576 0.71681869 0.41622367
 1.19782613 0.5229131  1.71170657 2.86601262], Cost: 0.0424
Current parameters: [2.54596385 2.39494227 1.55641721 0.90575099 1.46565085 1.13473721
 0.14910776 1.52605316 1.52701183 1.50487069 0.64757958 0.40336378
 1.20434577 0.57561852 1.70112611 2.88877458], Cost: 0.1711
Current parameters: [2.55133675 2.38825082 1.61463072 0.88899275 1.44748848 1.14308382
 0.14261387 1.53973556 1.55325793 1.590974   0.69946173 0.40661679
 1.19949942 0.51429865 1.70305829 2.86496879], Cost: 0.0304
Current parameters: [2.5655338  2.38441608 1.54406145 0.87576462 1.45709322 1.09973639
 0.09312013 1.50536911 1.51133559 1.57505149 0.73899986 0.37274067
 1.21939925 0.46864409 1.68884103 2.90067116], Cost: 0.1143
Current parameters: [2.49994367 2.38921962 1.5571973  0.89192691 1.44875961 1.14406325
 0.14734775 1.49791593 1.55975741 1.59575768 0.69921428 0.40997141
 1.2010999  0.51658293 1.70404737 2.86697894], Cost: 0.0250
Current parameters: [2.49374667 2.38600533 1.55581128 0.85220358 1.46451414 1.13182668
 0.1322746  1.59984872 1.54091399 1.59019707 0.70627763 0.4690057
 1.20453426 0.51210099 1.7024022  2.92130677], Cost: 0.0057
Current parameters: [2.49069781 2.39017865 1.55645705 0.8768324  1.46737448 1.13391193
 0.14881623 1.60278813 1.49343202 1.61585011 0.70409204 0.4698244
 1.20961862 0.51629717 1.70569916 2.92362087], Cost: 0.0175
Current parameters: [2.49315016 2.38814248 1.57018034 0.85047803 1.46705843 1.16650291
 0.17123013 1.59642842 1.58501083 1.61707993 0.73848336 0.44494659
```

```
 1.23249763 0.51737751 1.74978456 2.9943433 ], Cost: 0.1860
Current parameters: [2.49235931 2.38656436 1.55627089 0.8393232  1.45558602 1.12018765
 0.13045761 1.59947434 1.53535749 1.59701626 0.73854816 0.4603577
 1.17956579 0.55314302 1.69821368 2.92075107], Cost: 0.0186
Current parameters: [2.455298   2.38785074 1.56789582 0.88174745 1.4087828  1.08612408
 0.16095973 1.62609981 1.54817153 1.55758737 0.742124   0.4943404
 1.21973906 0.4813704  1.69868768 2.87455717], Cost: 0.1333
Current parameters: [2.51973625 2.37023288 1.52439283 0.84067225 1.44347592 1.13007552
 0.15848794 1.61380904 1.54749687 1.60469725 0.6946237  0.46197403
 1.20440993 0.51151712 1.6964411  2.91359389], Cost: 0.0042
Current parameters: [2.5068517  2.36204775 1.53575208 0.85703946 1.43146305 1.12262664
 0.1218491  1.61254097 1.5650509  1.65193217 0.64274693 0.47310623
 1.13613241 0.49313337 1.64449627 2.93356418], Cost: 0.2427
Current parameters: [2.49378367 2.36930606 1.52741277 0.82692478 1.46640566 1.13889654
 0.13714027 1.63597255 1.55324384 1.59724781 0.68012376 0.43364291
 1.21048416 0.501223   1.70084179 2.89877634], Cost: 0.0173
Current parameters: [2.51876175 2.35936107 1.501547   0.84888787 1.41489951 1.12533906
 0.20765387 1.64440853 1.51391897 1.65019842 0.7219851  0.39765489
 1.19896406 0.54837029 1.70384357 2.87898576], Cost: 0.1771
Current parameters: [2.53224056 2.36152494 1.52173736 0.81776705 1.46273698 1.13970151
 0.14990953 1.60116914 1.54189318 1.61323887 0.69866778 0.49448985
 1.2152431  0.51571671 1.71160295 2.88355241], Cost: 0.0401
Current parameters: [2.51880183 2.34650757 1.52609815 0.84866467 1.45778258 1.12492196
 0.16539137 1.61338642 1.54938475 1.59958518 0.69754693 0.46190218
 1.20185093 0.51314698 1.69238409 2.91533299], Cost: 0.0105
Current parameters: [2.52613662 2.37833085 1.51784197 0.83989759 1.44328835 1.12625231
 0.16350562 1.61070789 1.53593523 1.56432237 0.66312352 0.46392433
 1.20950718 0.54023283 1.70300659 2.92088775], Cost: 0.0404
Current parameters: [2.51941357 2.36324519 1.5262339  0.84218732 1.43699187 1.12111444
 0.14616093 1.61214559 1.54938686 1.61226122 0.69209133 0.46029616
 1.2254873  0.52046198 1.68999228 2.91659598], Cost: 0.0106
Current parameters: [2.53796365 2.36925507 1.50984294 0.8264827  1.44736035 1.10961206
 0.14235442 1.60010289 1.52435095 1.59128929 0.71051559 0.44546618
 1.20514015 0.48254088 1.69742681 2.92760555], Cost: 0.0509
Current parameters: [2.52303221 2.38412556 1.52314799 0.84198526 1.45832313 1.11065378
 0.16516565 1.61468139 1.55557579 1.61141584 0.69175585 0.46162228
 1.20572272 0.51281544 1.6968628  2.91157419], Cost: 0.0088
Current parameters: [2.52314362 2.38616614 1.52155825 0.83588776 1.45728289 1.15165057
 0.1697196  1.613523   1.54661208 1.59532849 0.70798683 0.46271433
 1.2195838  0.51233954 1.64820768 2.9120336 ], Cost: 0.0273
Current parameters: [2.50348783 2.3710785  1.49984353 0.8441785  1.44519693 1.13077018
```



```
 0.15676885 1.60818704 1.54923754 1.60627938 0.69739317 0.46710785
 1.20469817 0.51354498 1.69761612 2.91729969], Cost: 0.0095
Current parameters: [2.54698657 2.37709294 1.51168048 0.87721159 1.4498658  1.14143599
 0.13267234 1.63288974 1.55789932 1.59797446 0.70661516 0.46201957
 1.20245419 0.51598675 1.70347706 2.90659659], Cost: 0.0071
Current parameters: [2.51966516 2.36883896 1.52229336 0.83219433 1.44490153 1.12690053
 0.15853582 1.63707728 1.540811   1.60684016 0.69790915 0.47359925
 1.20421264 0.51347521 1.69617703 2.9256616 ], Cost: 0.0066
Current parameters: [2.50044705 2.37396698 1.53547733 0.85295622 1.42191011 1.11182977
 0.16486617 1.62424759 1.55692352 1.5791866  0.70825234 0.48113371
 1.21507616 0.49114523 1.69747889 2.89176531], Cost: 0.0267
Current parameters: [2.51847748 2.3749038  1.53762698 0.83986211 1.44807677 1.15059347
 0.17089091 1.60827914 1.57576435 1.60448685 0.70593876 0.45973341
 1.22331757 0.50569438 1.72326566 2.94580221], Cost: 0.0439
Current parameters: [2.52050926 2.36860604 1.51964971 0.84215027 1.43577615 1.13102607
 0.17253325 1.62140916 1.53996742 1.61321498 0.70281826 0.44751387
 1.2056506  0.5210807  1.70203565 2.90341439], Cost: 0.0201
Current parameters: [2.52226128 2.3688897  1.52125011 0.84474653 1.4433706  1.13243144
 0.16296648 1.61559442 1.54456501 1.60771147 0.68630409 0.46317804
 1.20955013 0.50340786 1.69668266 2.91329192], Cost: 0.0052
Current parameters: [2.51725301 2.369465   1.52977895 0.84503232 1.43570953 1.13101808
 0.15216234 1.61431175 1.55371493 1.61740508 0.6838928  0.46541597
 1.18445509 0.51074297 1.68688299 2.91532873], Cost: 0.0134
Current parameters: [2.52502683 2.36726533 1.51853467 0.83448244 1.43946018 1.12917522
 0.16044103 1.61553943 1.55601304 1.60025738 0.69103891 0.46068574
 1.20383194 0.51246871 1.69525325 2.91234173], Cost: 0.0028
Current parameters: [2.52495137 2.37285097 1.52009578 0.83879921 1.43906578 1.12875469
 0.16191088 1.61375771 1.54695304 1.58361727 0.67413568 0.46277859
 1.20220287 0.52798535 1.69854812 2.91624893], Cost: 0.0094
Current parameters: [2.52719862 2.36966473 1.52082718 0.83192211 1.43904515 1.12673194
 0.15544202 1.60941048 1.54862459 1.59650426 0.69637845 0.45678875
 1.20044251 0.50833591 1.69663403 2.9155531 ], Cost: 0.0049
Current parameters: [2.53564914 2.35979882 1.50083783 0.83293832 1.43657081 1.11766718
 0.15990094 1.62265093 1.5584057  1.60046215 0.68143702 0.45626231
 1.20399736 0.50125877 1.68965598 2.91746228], Cost: 0.0115
Current parameters: [2.52563282 2.36826547 1.52137935 0.83446081 1.43923044 1.13368668
 0.16300263 1.61420466 1.5636996  1.60042491 0.6917615  0.45949267
 1.20626161 0.5106496  1.70146981 2.92219142], Cost: 0.0073
Current parameters: [2.52210851 2.36826305 1.52001285 0.83718235 1.43611028 1.12666001
 0.16156873 1.61723939 1.55725963 1.59700866 0.6923887  0.46066889
 1.20427973 0.51016507 1.69436059 2.91201885], Cost: 0.0026
```



```
Current parameters: [2.52345177 2.36688131 1.52141125 0.83351769 1.43531659 1.12619994
 0.15988243 1.61550554 1.55884997 1.59505151 0.69322491 0.47117888
 1.2057948  0.50698668 1.69906028 2.90387348], Cost: 0.0021
Current parameters: [2.52660802 2.36770192 1.52069651 0.83485085 1.43352365 1.12626208
 0.16297272 1.61316442 1.55827575 1.59547601 0.69542566 0.47439183
 1.20604443 0.50819102 1.69587147 2.90545616], Cost: 0.0026
Current parameters: [2.52194618 2.37073381 1.52243137 0.82939444 1.44020455 1.13276519
 0.15884371 1.61718102 1.56043666 1.59082246 0.69407278 0.47066359
 1.20903654 0.50429812 1.68970413 2.90049002], Cost: 0.0014
Current parameters: [2.51870504 2.36958075 1.52351378 0.82488434 1.43937589 1.13112555
 0.16229259 1.61513779 1.55939291 1.59186955 0.6921738  0.47072385
 1.20944337 0.50367972 1.6886826  2.90141535], Cost: 0.0016
Current parameters: [2.52243085 2.37152831 1.5220296  0.82735331 1.43630779 1.13616058
 0.16463331 1.62096993 1.55773575 1.59274479 0.69927082 0.46521334
 1.20950344 0.50726201 1.69221734 2.89147203], Cost: 0.0045
Current parameters: [2.52167658 2.37183335 1.52217446 0.8297017  1.44099728 1.13446339
 0.16097502 1.61778469 1.56095067 1.59079298 0.69313684 0.47159232
 1.20234226 0.50223805 1.68985908 2.9001916 ], Cost: 0.0017
Current parameters: [2.52003554 2.37027857 1.52849906 0.83012121 1.43451991 1.13333755
 0.15099378 1.61620322 1.56315251 1.59724294 0.69161133 0.47190053
 1.20612891 0.50648462 1.68394702 2.89822043], Cost: 0.0009
Current parameters: [2.52311156 2.36630391 1.53335532 0.83067844 1.43647908 1.13232624
 0.15253137 1.61733499 1.56319614 1.59613131 0.69139834 0.47093915
 1.20572547 0.50627608 1.68306081 2.89780024], Cost: 0.0009
Current parameters: [2.51546203 2.35766456 1.53022768 0.83447479 1.44105506 1.13307517
 0.15090356 1.61342274 1.56427871 1.59478104 0.69628504 0.47267632
 1.20402023 0.50887739 1.6806884  2.89633713], Cost: 0.0029
Current parameters: [2.52205312 2.37030946 1.5356224  0.83145446 1.44020437 1.12930944
 0.15345223 1.61596552 1.56462898 1.59768826 0.69278182 0.47100466
 1.20558418 0.50830376 1.68373213 2.89659662], Cost: 0.0010
Current parameters: [2.52726473 2.3658673  1.52713418 0.82925724 1.4380695  1.12348289
 0.15026    1.6203231  1.56701862 1.59813847 0.68730166 0.47025832
 1.20611751 0.50143447 1.67809847 2.89514502], Cost: 0.0020
Current parameters: [2.52224878 2.36599801 1.53374549 0.82864755 1.43726905 1.13149056
 0.15170138 1.62222921 1.56190135 1.59813483 0.69486946 0.4733294
 1.20529779 0.50622312 1.68282594 2.9003027 ], Cost: 0.0006
Current parameters: [2.52138078 2.36837011 1.53493882 0.8281108  1.43747022 1.12995711
 0.14967437 1.62661986 1.55764059 1.59112708 0.68747742 0.47799323
 1.20450791 0.51426173 1.68186255 2.90096779], Cost: 0.0047
Current parameters: [2.52224878 2.36599801 1.53374549 0.82864755 1.43726905 1.13149056
 0.15170138 1.62222921 1.56190135 1.59813483 0.69486946 0.4733294
```



```
 1.20529779 0.50622312 1.68282594 2.9003027 ], Cost: 0.0006

   Return from subroutine COBYLA because the MAXFUN limit has been reached.

   NFVALS =  100   F = 5.661726E-04    MAXCV = 0.000000E+00
   X = 2.522249E+00   2.365998E+00   1.533745E+00   8.286475E-01   1.437269E+00
       1.131491E+00   1.517014E-01   1.622229E+00   1.561901E+00   1.598135E+00
       6.948695E-01   4.733294E-01   1.205298E+00   5.062231E-01   1.682826E+00
       2.900303E+00
Simulating for QAOA depth p = 9
Current parameters: [3.152406   0.26457338 2.29049095 1.46911031 2.65208947 1.64592439
 2.93913091 2.34870068 2.05932692 3.00693741 1.13308742 0.39271492
 2.28985703 2.99852627 2.61959634 0.00925865 3.07970128 1.31366007], Cost: 3.0579
Current parameters: [2.152406   1.26457338 2.29049095 1.46911031 2.65208947 1.64592439
 2.93913091 2.34870068 2.05932692 3.00693741 1.13308742 0.39271492
 2.28985703 2.99852627 2.61959634 0.00925865 3.07970128 1.31366007], Cost: 2.9287
Current parameters: [2.152406   0.26457338 3.29049095 1.46911031 2.65208947 1.64592439
 2.93913091 2.34870068 2.05932692 3.00693741 1.13308742 0.39271492
 2.28985703 2.99852627 2.61959634 0.00925865 3.07970128 1.31366007], Cost: 1.8400
Current parameters: [2.152406   0.26457338 2.29049095 2.46911031 2.65208947 1.64592439
 2.93913091 2.34870068 2.05932692 3.00693741 1.13308742 0.39271492
 2.28985703 2.99852627 2.61959634 0.00925865 3.07970128 1.31366007], Cost: 1.6130
Current parameters: [2.152406   0.26457338 2.29049095 1.46911031 3.65208947 1.64592439
 2.93913091 2.34870068 2.05932692 3.00693741 1.13308742 0.39271492
 2.28985703 2.99852627 2.61959634 0.00925865 3.07970128 1.31366007], Cost: 1.3966
Current parameters: [2.152406   0.26457338 2.29049095 1.46911031 2.65208947 2.64592439
 2.93913091 2.34870068 2.05932692 3.00693741 1.13308742 0.39271492
 2.28985703 2.99852627 2.61959634 0.00925865 3.07970128 1.31366007], Cost: 2.2991
Current parameters: [2.152406   0.26457338 2.29049095 1.46911031 2.65208947 1.64592439
 3.93913091 2.34870068 2.05932692 3.00693741 1.13308742 0.39271492
 2.28985703 2.99852627 2.61959634 0.00925865 3.07970128 1.31366007], Cost: 1.2477
Current parameters: [2.152406   0.26457338 2.29049095 1.46911031 2.65208947 1.64592439
 2.93913091 3.34870068 2.05932692 3.00693741 1.13308742 0.39271492
 2.28985703 2.99852627 2.61959634 0.00925865 3.07970128 1.31366007], Cost: 1.2181
Current parameters: [2.152406   0.26457338 2.29049095 1.46911031 2.65208947 1.64592439
 2.93913091 2.34870068 3.05932692 3.00693741 1.13308742 0.39271492
 2.28985703 2.99852627 2.61959634 0.00925865 3.07970128 1.31366007], Cost: 1.0017
Current parameters: [2.152406   0.26457338 2.29049095 1.46911031 2.65208947 1.64592439
 2.93913091 2.34870068 2.05932692 4.00693741 1.13308742 0.39271492
 2.28985703 2.99852627 2.61959634 0.00925865 3.07970128 1.31366007], Cost: 3.7418
Current parameters: [2.152406   0.26457338 2.29049095 1.46911031 2.65208947 1.64592439
```

```
 2.93913091 2.34870068 2.05932692 3.00693741 2.13308742 0.39271492
 2.28985703 2.99852627 2.61959634 0.00925865 3.07970128 1.31366007], Cost: 3.7791
Current parameters: [2.152406   0.26457338 2.29049095 1.46911031 2.65208947 1.64592439
 2.93913091 2.34870068 2.05932692 3.00693741 1.13308742 1.39271492
 2.28985703 2.99852627 2.61959634 0.00925865 3.07970128 1.31366007], Cost: 1.5127
Current parameters: [2.152406   0.26457338 2.29049095 1.46911031 2.65208947 1.64592439
 2.93913091 2.34870068 2.05932692 3.00693741 1.13308742 0.39271492
 3.28985703 2.99852627 2.61959634 0.00925865 3.07970128 1.31366007], Cost: 2.3013
Current parameters: [2.152406   0.26457338 2.29049095 1.46911031 2.65208947 1.64592439
 2.93913091 2.34870068 2.05932692 3.00693741 1.13308742 0.39271492
 2.28985703 3.99852627 2.61959634 0.00925865 3.07970128 1.31366007], Cost: 2.8247
Current parameters: [2.152406   0.26457338 2.29049095 1.46911031 2.65208947 1.64592439
 2.93913091 2.34870068 2.05932692 3.00693741 1.13308742 0.39271492
 2.28985703 2.99852627 3.61959634 0.00925865 3.07970128 1.31366007], Cost: 3.6159
Current parameters: [2.152406   0.26457338 2.29049095 1.46911031 2.65208947 1.64592439
 2.93913091 2.34870068 2.05932692 3.00693741 1.13308742 0.39271492
 2.28985703 2.99852627 2.61959634 1.00925865 3.07970128 1.31366007], Cost: 3.4979
Current parameters: [2.152406   0.26457338 2.29049095 1.46911031 2.65208947 1.64592439
 2.93913091 2.34870068 2.05932692 3.00693741 1.13308742 0.39271492
 2.28985703 2.99852627 2.61959634 0.00925865 4.07970128 1.31366007], Cost: 1.1694
Current parameters: [2.152406   0.26457338 2.29049095 1.46911031 2.65208947 1.64592439
 2.93913091 2.34870068 2.05932692 3.00693741 1.13308742 0.39271492
 2.28985703 2.99852627 2.61959634 0.00925865 3.07970128 2.31366007], Cost: 1.8535
Current parameters: [ 1.84829031 -0.02191665  2.15253285  1.36212014  2.57461529  1.44532293
  2.881971    2.29557577  2.03572745  2.60952001  0.73057529  0.29939923
  2.08895616  2.72622651  2.23935437 -0.35489194  3.03323096  1.173852  ], Cost: 2.2174
Current parameters: [ 2.00034815  0.12132836  2.2215119   1.41561523  2.61335238  1.54562366
  2.91055096  2.32213822  2.04752719  2.80822871  0.93183135  0.34605708
  2.18940659  2.86237639  2.42947535 -0.17281665  3.05646612  1.24375604], Cost: 2.2206
Current parameters: [ 2.39630553  0.24806577  2.28254178  1.46294552  2.64762539  1.6343657
  2.93583735  2.34563961  2.05796711  2.98403815  1.1098946   0.38733806
  2.27828108  2.98283631  2.59768674 -0.01172377  3.07702365  1.30560431], Cost: 1.7106
Current parameters: [2.152406   0.31880622 2.17786858 1.46911031 2.65208947 1.64592439
 2.93913091 2.34870068 2.05932692 3.00693741 1.13308742 0.39271492
 2.28985703 2.99852627 2.61959634 0.00925865 3.07970128 1.31366007], Cost: 1.0248
Current parameters: [ 2.13004834  0.47789789  2.40732215  1.46275736  2.64748914  1.63401291
  2.93573682  2.34554618  2.05792561  2.98333923  1.10918672  0.38717395
  2.27792777  2.98235743  2.59701802 -0.01236419  3.07694193  1.30535843], Cost: 1.4842
Current parameters: [2.152406   0.26457338 2.29049095 1.54242313 2.55084604 1.64592439
 2.93913091 2.34870068 2.05932692 3.00693741 1.13308742 0.39271492
 2.28985703 2.99852627 2.61959634 0.00925865 3.07970128 1.31366007], Cost: 0.7273
```



```
Current parameters: [2.14337267e+00 2.56465097e-01 2.29228565e+00 1.74547448e+00
 2.69460096e+00 1.64111170e+00 2.93775957e+00 2.34742614e+00
 2.05876074e+00 2.99740286e+00 1.12343065e+00 3.90476163e-01
 2.28503716e+00 2.99199345e+00 2.61047386e+00 5.22216203e-04
 3.07858640e+00 1.31030590e+00], Cost: 0.8490
Current parameters: [2.152406   0.26457338 2.29049095 1.54242313 2.55084604 1.68017876
 2.81891595 2.34870068 2.05932692 3.00693741 1.13308742 0.39271492
 2.28985703 2.99852627 2.61959634 0.00925865 3.07970128 1.31366007], Cost: 0.9360
Current parameters: [ 2.13784568  0.25150409  2.29338372  1.54378474  2.546546    1.88083494
  3.01522809  2.34664633  2.05841432  2.9915692   1.1175222   0.38910639
  2.28208815  2.98799638  2.60489232 -0.00482312  3.07790426  1.30825367], Cost: 1.0394
Current parameters: [2.152406   0.26457338 2.29049095 1.54242313 2.55084604 1.64592439
 2.93913091 2.39944714 1.94509122 3.00693741 1.13308742 0.39271492
 2.28985703 2.99852627 2.61959634 0.00925865 3.07970128 1.31366007], Cost: 0.8592
Current parameters: [2.14836991 0.26095059 2.29129282 1.54280057 2.54965408 1.643937
 2.94110407 2.57631297 2.16212728 3.00267737 1.12877277 0.39171464
 2.28770351 2.9956074  2.61552041 0.00535521 3.07920315 1.31216143], Cost: 0.4001
Current parameters: [2.14836991 0.26095059 2.29129282 1.54280057 2.54965408 1.643937
 2.94110407 2.57631297 2.16212728 2.91372788 1.21659639 0.39171464
 2.28770351 2.9956074  2.61552041 0.00535521 3.07920315 1.31216143], Cost: 0.3456
Current parameters: [ 2.10187684  0.21921861  2.30052984  1.54714836  2.53592348  1.62104363
  2.9638335   2.59041374  2.18784809  3.0654667   1.38073754  0.38019211
  2.26289646  2.96198408  2.56856849 -0.03960977  3.07346504  1.29489806], Cost: 1.9917
Current parameters: [ 2.14836991  0.26095059  2.29129282  1.54280057  2.54965408  1.643937
  2.94110407  2.57631297  2.16212728  2.91372788  1.21659639  0.39171464
  2.28770351  2.9956074   2.70197789 -0.08492272  3.07920315  1.31216143], Cost: 0.2942
Current parameters: [2.10874194 0.2253807  2.29916591 1.54650637 2.53795092 1.62442403
 2.9604773  2.58833164 2.18405019 2.85880683 1.16988429 0.38189351
 2.26655943 2.96694886 2.86953091 0.06736962 3.07431232 1.29744714], Cost: 1.0316
Current parameters: [ 2.14836991  0.26095059  2.29129282  1.54280057  2.54965408  1.643937
  2.94110407  2.57631297  2.16212728  2.91372788  1.21659639  0.39171464
  2.18711723  3.06981926  2.70197789 -0.08492272  3.07920315  1.31216143], Cost: 0.3529
Current parameters: [ 2.12249068  0.23772151  2.29643438  1.54522066  2.54201128  1.63119396
  2.95375585  2.58416182  2.17644414  2.87786143  1.18609083  0.38530091
  2.43391767  3.18637099  2.68905082 -0.10263822  3.07600917  1.3025522 ], Cost: 1.1592
Current parameters: [ 2.14836991  0.26095059  2.29129282  1.54280057  2.54965408  1.643937
  2.94110407  2.57631297  2.16212728  2.91372788  1.21659639  0.28774624
  2.28770351  2.9956074   2.70197789 -0.08492272  3.07920315  1.38155574], Cost: 0.3089
Current parameters: [ 2.13622791  0.25005199  2.29370513  1.54393602  2.54606824  1.63795823
  2.94704002  2.57999549  2.16884445  2.89690009  1.20228382  0.52961387
  2.28168047  2.98396525  2.69591278 -0.09323446  3.0777046   1.51783109], Cost: 1.3844
```



```
Current parameters: [ 2.1529614   0.26527597  2.29337569  1.54441589  2.55082377  1.64696564
  2.94196706  2.57711504  2.16248358  2.91972802  1.22267345  0.39312351
  2.29073668  2.99971854  2.70771872 -0.07942484  3.20329     1.31427223], Cost: 0.4073
Current parameters: [ 2.069595    0.18764258  2.26878562  1.5241175   2.52876322  1.59536628
  2.94002321  2.57224472  2.16880647  2.80918065  1.11526993  0.35749175
  2.23900127  2.92378705  2.61870934 -0.16884826  3.10243774  1.25932677], Cost: 1.1626
Current parameters: [ 2.20779914  0.26232     2.29195225  1.55326805  2.48743601  1.60034143
  2.98746463  2.52695714  2.20206171  2.91121929  1.21405564  0.39112561
  2.28643538  2.99388858  2.69957771 -0.08722133  3.07890982  1.31127893], Cost: 0.2829
Current parameters: [ 2.41926654  0.24124042  2.29992312  1.54578879  2.55516779  1.64283967
  2.94726025  2.5959428   2.17065708  2.88321264  1.19215028  0.37084611
  2.27815297  2.97443375  2.69652124 -0.09607025  3.06513447  1.2786846 ], Cost: 0.7311
Current parameters: [ 2.18877155  0.34446865  2.3315106   1.55115332  2.50591226  1.61449891
  2.97464858  2.54154484  2.19080987  2.91522861  1.21811636  0.41183029
  2.30704886  2.96160019  2.71614764 -0.12086213  3.04083121  1.27789026], Cost: 0.3831
Current parameters: [ 2.23250739  0.39310397  2.37438038  1.55979045  2.4430187   1.5576125
  3.02664552  2.50845387  2.24037579  2.85950979  1.168617    0.31673965
  2.22189464  3.04244476  2.64495361 -0.02680407  3.15431173  1.35238208], Cost: 0.9037
Current parameters: [ 2.21124457  0.3160917   2.1796461   1.55558894  2.4876691   1.60120602
  2.99100659  2.53044898  2.20551684  2.90923607  1.21349101  0.38637611
  2.28559794  2.99463465  2.70219992 -0.08297537  3.07871203  1.30764763], Cost: 0.4027
Current parameters: [ 2.20972281  0.26404667  2.29157007  1.49944143  2.45945446  1.60688467
  2.98006872  2.53495101  2.19586774  2.91324969  1.21611207  0.39160236
  2.28746178  2.99527976  2.70152036 -0.08536089  3.07914723  1.3119932 ], Cost: 0.3385
Current parameters: [ 2.20287596  0.2563016   2.29302143  1.56367161  2.46987029  1.70809146
  3.04035691  2.51067167  2.21738638  2.90317718  1.20664165  0.38706803
  2.28245357  2.98944423  2.69417186 -0.09151999  3.07798672  1.30696425], Cost: 0.3577
Current parameters: [ 2.20766723  0.2622016   2.29197846  1.54374694  2.50056254  1.6087374
  2.95783571  2.51360402  2.25222642  2.91108007  1.21391463  0.39109292
  2.28636499  2.99379319  2.6994445  -0.0873489   3.07889354  1.31122995], Cost: 0.2776
Current parameters: [ 2.21102956  0.26282727  2.29144667  1.59370613  2.43422125  1.63173835
  2.87781013  2.48612195  2.22084549  2.91037224  1.2142915   0.38768195
  2.28613224  2.99490257  2.70219072 -0.08340495  3.07883661  1.30875889], Cost: 0.2670
Current parameters: [ 2.21445722  0.26590392  2.29076568  1.57779655  2.45676166  1.63342614
  2.87613443  2.4369438   2.19756483  2.90260333  1.20642299  0.38853144
  2.28796112  2.99738142  2.70565221 -0.08008995  3.07925965  1.31003161], Cost: 0.3836
Current parameters: [ 2.20462873  0.24154068  2.29360324  1.6055805   2.43035279  1.62074291
  2.88591907  2.47305738  2.22870348  2.98502603  1.29700765  0.35852392
  2.26955064  2.98168412  2.6915087  -0.0850992   3.07498107  1.28221931], Cost: 0.4995
Current parameters: [ 2.22445643  0.27476229  2.29580039  1.59732854  2.43685961  1.63929258
  2.87923539  2.48673849  2.22089216  2.92694132  1.23107299  0.41376338
```



```
  2.29225224  3.00557035  2.7146582  -0.06951457  3.074677    1.35037227], Cost: 0.2788
Current parameters: [ 2.18458221  0.22977611  2.28111711  1.60292953  2.42545127  1.6171134
  2.87005746  2.49692441  2.22628059  2.88973051  1.20043074  0.43113589
  2.26029352  2.94028122  2.66308921 -0.13604441  3.07161814  1.36008845], Cost: 0.7344
Current parameters: [ 2.21458396  0.26574411  2.2908222   1.59346923  2.43484845  1.6331076
  2.8764874   2.48488998  2.21927919  2.91448628  1.21845828  0.38793394
  2.31130703  3.03013691  2.66973441 -0.11357316  3.0798877   1.31032624], Cost: 0.3452
Current parameters: [ 2.18038248  0.22780582  2.2929764   1.61258744  2.42630447  1.62104219
  2.88064884  2.50946842  2.23763922  2.88955357  1.20107175  0.38798401
  2.33879753  3.03639725  2.76720816 -0.04468164  3.05226481  1.2852717 ], Cost: 0.2203
Current parameters: [ 2.14231507  0.25112278  2.30338493  1.61496227  2.40687192  1.60677587
  2.89397768  2.49341289  2.24912779  2.88765764  1.19915152  0.3816204
  2.33159649  3.04666221  2.76030281 -0.03483329  3.06491574  1.29715299], Cost: 0.2574
Current parameters: [ 2.25309087e+00  2.55771498e-01  2.33083182e+00  1.63399026e+00
  2.42235968e+00  1.61121854e+00  2.88163895e+00  2.54321555e+00
  2.25566618e+00  2.86026528e+00  1.18049136e+00  3.43961669e-01
  2.31944467e+00  3.05596308e+00  2.77027229e+00 -2.91584300e-03
  3.06954023e+00  1.27755665e+00], Cost: 0.7062
Current parameters: [ 2.18093043  0.23894665  2.27036254  1.61303635  2.42651612  1.62134064
  2.88124979  2.51030534  2.23823725  2.84865902  1.24099528  0.38718473
  2.33865813  3.03649196  2.76778916 -0.04389205  3.05236389  1.28437754], Cost: 0.2330
Current parameters: [ 2.17930782  0.26957595  2.18913073  1.62084     2.42440734  1.62041871
  2.8829822   2.51784163  2.24406102  2.92662042  1.16624297  0.3831762
  2.34229379  3.03793038  2.77844369 -0.03621112  3.04770921  1.27734298], Cost: 0.2387
Current parameters: [ 2.17551896  0.22928294  2.29349206  1.61481347  2.42928217  1.62719859
  2.87398486  2.51947507  2.2350034   2.8910049   1.20210977  0.39663125
  2.34738727  3.02573749  2.78057176 -0.05021685  3.10779533  1.2743767 ], Cost: 0.3667
Current parameters: [ 2.17335841  0.20642301  2.28450817  1.60216382  2.46713869  1.61397297
  2.90236023  2.47544679  2.23171809  2.91530989  1.22625059  0.42308093
  2.3967976   3.09230906  2.78214799 -0.0479146   3.02177318  1.32234014], Cost: 0.2376
Current parameters: [ 2.16944021  0.26591139  2.31133598  1.61352369  2.43556206  1.62695603
  2.87497296  2.51720012  2.23335994  2.89330338  1.20416673  0.39851753
  2.35199803  3.02255492  2.77866631 -0.05963078  3.0272509   1.2684008 ], Cost: 0.1646
Current parameters: [ 2.17015697  0.26592791  2.31134394  1.61364994  2.43481166  1.62643024
  2.8755321   2.51660485  2.23384158  2.89327312  1.20413608  0.39725649
  2.33549753  3.03469686  2.79489246 -0.0766319   3.02724736  1.2692271 ], Cost: 0.1464
Current parameters: [ 2.1542929   0.26491314  2.31086088  1.61212976  2.450575    1.63682917
  2.86421655  2.52934551  2.22449345  2.89492631  1.20541629  0.42370513
  2.3087583   3.05736932  2.77917644 -0.05631194  3.02351035  1.25162472], Cost: 0.1435
Current parameters: [ 2.14437736  0.26503194  2.31068485  1.60977101  2.46232421  1.63794115
  2.85313776  2.53877481  2.21673689  2.89583028  1.20628864  0.40619321
```

```
  2.30921033  3.05792571  2.7799061  -0.05566507  3.02361503  1.26394618], Cost: 0.1535
Current parameters: [ 2.14535595  0.2608856   2.3108563   1.6174835   2.44738754  1.69246571
  2.88642104  2.52626327  2.22944797  2.89273755  1.20297779  0.41408876
  2.30878755  3.05733647  2.77992471 -0.0575296   3.01749352  1.25433902], Cost: 0.1831
Current parameters: [ 2.15150932  0.26204971  2.31023297  1.63784628  2.46288725  1.63283988
  2.86690361  2.52660638  2.2276152   2.89124494  1.20202366  0.42634289
  2.3057837   3.05196884  2.77588623 -0.06054424  3.02316161  1.25445555], Cost: 0.1484
Current parameters: [ 2.13581086  0.24780646  2.30326862  1.59966021  2.43516524  1.62946171
  2.85742888  2.54237135  2.22749022  2.8857525   1.19545846  0.4476385
  2.30231889  3.03729019  2.7802256  -0.07367302  2.99499061  1.2666508 ], Cost: 0.0508
Current parameters: [ 2.12395802  0.2313493   2.30075931  1.60424884  2.42695513  1.62071257
  2.85766135  2.54117788  2.23241577  2.9176136   1.22911877  0.43828145
  2.28998951  3.0215942   2.78367055 -0.07548768  2.97603     1.24964688], Cost: 0.0652
Current parameters: [ 2.13613706  0.24797767  2.30346331  1.59492931  2.44175845  1.63362229
  2.84264742  2.53574601  2.25260233  2.88558375  1.19531289  0.447412
  2.30209241  3.03727918  2.78000576 -0.07363696  2.99513207  1.2666538 ], Cost: 0.0585
Current parameters: [ 2.1548895   0.254991    2.31394111  1.59896964  2.4261861   1.61453635
  2.8596132   2.56484695  2.23370698  2.8750105   1.18233822  0.45286035
  2.29237006  3.04299434  2.78763995 -0.07063566  2.95093885  1.27520793], Cost: 0.0400
Current parameters: [ 2.15317251  0.25429338  2.31348902  1.58545576  2.44266419  1.60863007
  2.87756685  2.56695841  2.23885242  2.87262913  1.17941804  0.45029998
  2.28661136  3.0353977   2.78790878 -0.07027298  2.95225139  1.27107662], Cost: 0.0319
Current parameters: [ 2.15064431  0.25168376  2.31088191  1.57522393  2.44759364  1.60634703
  2.86411546  2.52655969  2.21515438  2.85925274  1.16374935  0.45039991
  2.27498276  3.02080168  2.80029829 -0.06588078  2.93309836  1.26300743], Cost: 0.0429
Current parameters: [ 2.14385001  0.2445946   2.31221543  1.58755084  2.44048653  1.60444344
  2.87738892  2.57068624  2.24155997  2.86408593  1.1721549   0.44172623
  2.29779939  3.04466663  2.79020137 -0.07274228  2.9491029   1.25334398], Cost: 0.1112
Current parameters: [ 2.16340968  0.2660328   2.3110041   1.57353864  2.44413157  1.60763166
  2.87640728  2.56083594  2.23151102  2.87898476  1.18083691  0.45305157
  2.27747926  3.03603662  2.74593297 -0.10800599  2.944354    1.27721408], Cost: 0.0935
Current parameters: [ 2.15308888  0.24296206  2.33846752  1.58296836  2.44530013  1.60863785
  2.87785778  2.56353109  2.23688013  2.86493625  1.18917366  0.45346572
  2.2888854   3.03749221  2.78627112 -0.0726821   2.95323905  1.27453796], Cost: 0.0296
Current parameters: [ 2.14837218  0.24104732  2.32585088  1.57663816  2.46321932  1.60419963
  2.88833573  2.55169228  2.2343002   2.88186136  1.1994764   0.48038097
  2.31206074  3.05647369  2.80259858 -0.07227458  2.93952229  1.29932421], Cost: 0.0784
Current parameters: [ 2.17094159  0.23184245  2.33348614  1.58209082  2.45508437  1.61630867
  2.87057897  2.57254211  2.23098812  2.86603346  1.19023187  0.45761233
  2.29343082  3.03120748  2.79125972 -0.07813533  2.9539296   1.26744095], Cost: 0.0282
Current parameters: [ 2.16658191  0.24544863  2.34485706  1.57828073  2.44455398  1.61565275
```



```
  2.86552295  2.58906381  2.23050811  2.86612723  1.18912775  0.48191145
  2.2721139   2.99883638  2.81247383 -0.07578672  2.97451282  1.28067056], Cost: 0.0498
Current parameters: [ 2.16948008  0.2256904   2.34418851  1.58262859  2.45447194  1.62403454
  2.87348103  2.57165457  2.23143132  2.88526794  1.17075781  0.45647155
  2.29353803  3.03115441  2.79097235 -0.07854956  2.95319602  1.26842403], Cost: 0.0294
Current parameters: [ 2.16612201  0.2312285   2.32825949  1.58536849  2.44950808  1.66740781
  2.89136961  2.57231559  2.2357673   2.85165541  1.1989815   0.45184379
  2.28604633  3.02480172  2.79376197 -0.07665903  2.93835241  1.27660663], Cost: 0.0457
Current parameters: [ 2.17454022  0.22141194  2.32846064  1.5819398   2.45191937  1.61424799
  2.87260232  2.56992507  2.23256385  2.86498037  1.18935794  0.45367052
  2.27601233  3.04515656  2.8016901  -0.08825702  2.96078268  1.27275617], Cost: 0.0299
Current parameters: [ 2.18448395  0.20136863  2.32094206  1.57812967  2.44467818  1.60745467
  2.87304295  2.57078424  2.23358088  2.86277385  1.1868451   0.4572105
  2.2887208   3.02028103  2.76705875 -0.04422692  2.95926851  1.28959473], Cost: 0.0215
Current parameters: [ 2.1752228   0.20088329  2.32067796  1.57704196  2.45415016  1.61275941
  2.86586759  2.57843042  2.22785155  2.86372057  1.18760273  0.46781084
  2.27555124  3.03158122  2.75938429 -0.03407817  2.95743387  1.28263466], Cost: 0.0276
Current parameters: [ 2.1688955   0.20213301  2.32150155  1.57276651  2.46442769  1.60668598
  2.85235875  2.5887143   2.21953483  2.86425548  1.18811587  0.42066619
  2.29012171  3.01510358  2.7718239  -0.0451521   2.95532227  1.31941319], Cost: 0.0167
Current parameters: [ 2.16515048  0.19838066  2.32047692  1.59774046  2.47588852  1.60232323
  2.85468009  2.58666878  2.22280338  2.86010471  1.18421166  0.42455227
  2.28681895  3.00866665  2.7686     -0.05028203  2.95347648  1.32302376], Cost: 0.0230
Current parameters: [ 2.20214945  0.22565849  2.33589837  1.5858138   2.47795202  1.60688381
  2.85889909  2.5784251   2.21582694  2.87503549  1.19874205  0.40134908
  2.27878648  3.02205041  2.77652379 -0.01973041  2.96397659  1.31804653], Cost: 0.0509
Current parameters: [ 2.17449508  0.2104958   2.32221019  1.56998008  2.46869583  1.61134143
  2.85221617  2.58876123  2.21671673  2.84908924  1.17188945  0.42500387
  2.29652886  3.02326352  2.76706381 -0.04696296  2.96578197  1.32741596], Cost: 0.0681
Current parameters: [ 2.17762167  0.20772755  2.32021468  1.5612621   2.45995764  1.60174062
  2.85081841  2.58479378  2.21402417  2.88297515  1.20369049  0.4199769
  2.27225578  3.00239326  2.73520572 -0.07643076  2.94290512  1.32437351], Cost: 0.0851
Current parameters: [ 2.16856106  0.20742013  2.30787722  1.5669604   2.47625649  1.60404866
  2.86363989  2.58877112  2.22279123  2.87576047  1.19050922  0.43084275
  2.29512105  3.01850609  2.77727659 -0.04389513  2.9525553   1.3306925 ], Cost: 0.0138
Current parameters: [ 2.16106135  0.18419156  2.32630431  1.57294456  2.46811709  1.60097202
  2.84951063  2.57117249  2.21066805  2.88189824  1.21295772  0.44646977
  2.31341913  3.02821309  2.79892172 -0.03583056  2.93570843  1.34376203], Cost: 0.0568
Current parameters: [ 2.16647117  0.20798753  2.30751863  1.57140527  2.46914304  1.60009011
  2.87815307  2.59705903  2.19868651  2.87617562  1.19044948  0.43237728
  2.29451609  3.01599065  2.77917137 -0.04398052  2.95607871  1.33034527], Cost: 0.0110
```



```
Current parameters: [ 2.1584921   0.21642025  2.31335645  1.56598364  2.45953611  1.59705775
  2.87023357  2.61431975  2.21194871  2.88059708  1.19179585  0.44930758
  2.27357115  2.98070364  2.80480386 -0.03556924  2.9737278   1.33657824], Cost: 0.0824
Current parameters: [ 2.16172447  0.20451112  2.30591286  1.57002364  2.46256919  1.62424142
  2.88528876  2.5989778   2.20054253  2.86990074  1.19630136  0.42419812
  2.29103929  3.01225537  2.78206742 -0.04106314  2.94776715  1.33095732], Cost: 0.0146
Current parameters: [ 2.18065053  0.22119248  2.29683212  1.59321894  2.49193238  1.61183893
  2.90500293  2.58288566  2.20331503  2.87580246  1.18896258  0.4578094
  2.29336069  3.01794042  2.77019482 -0.05070486  2.95190343  1.35364051], Cost: 0.0790
Current parameters: [ 2.16170138  0.19936006  2.29806176  1.57238553  2.47292045  1.58998281
  2.88813464  2.60393513  2.20543381  2.87898626  1.18888896  0.42073847
  2.30158001  3.02044264  2.7806415  -0.04003531  2.95095377  1.31549219], Cost: 0.0212
Current parameters: [ 2.17123112  0.20992844  2.31031607  1.57046555  2.46632672  1.59643132
  2.87824927  2.60273831  2.19990935  2.87410279  1.18782205  0.43346684
  2.29147358  3.01704291  2.77947418 -0.044769    2.94485419  1.33246093], Cost: 0.0098
Current parameters: [ 2.1710155   0.207772    2.30938183  1.56228068  2.46183902  1.5939118
  2.87533774  2.60028116  2.1955374   2.8848747   1.19837751  0.42770661
  2.28563511  3.01159126  2.76234941 -0.05897724  2.94059815  1.32997111], Cost: 0.0344
Current parameters: [ 2.16929582  0.21069591  2.31416027  1.57373414  2.46705092  1.59871702
  2.87746938  2.61277997  2.20269823  2.8766006   1.19271848  0.43464199
  2.29297404  3.02338491  2.77654858 -0.04589378  2.94834032  1.33428576], Cost: 0.0148
Current parameters: [ 2.15877138  0.21592768  2.30861362  1.56788838  2.48328254  1.59975968
  2.86939257  2.60433058  2.18905502  2.87443861  1.18516657  0.43466489
  2.28030804  3.02339413  2.7819876  -0.04194403  2.93746751  1.32646528], Cost: 0.0088
Current parameters: [ 2.15715668  0.22195219  2.30915287  1.56920923  2.48333629  1.60122442
  2.86698375  2.60570947  2.18803922  2.87573742  1.18506     0.43654012
  2.28907461  3.01702004  2.77700741 -0.03684819  2.93394599  1.32392527], Cost: 0.0130
Current parameters: [ 2.15877138  0.21592768  2.30861362  1.56788838  2.48328254  1.59975968
  2.86939257  2.60433058  2.18905502  2.87443861  1.18516657  0.43466489
  2.28030804  3.02339413  2.7819876  -0.04194403  2.93746751  1.32646528], Cost: 0.0088

   Return from subroutine COBYLA because the MAXFUN limit has been reached.

   NFVALS =  100   F = 8.841736E-03    MAXCV = 0.000000E+00
   X = 2.158771E+00   2.159277E-01   2.308614E+00   1.567888E+00   2.483283E+00
       1.599760E+00   2.869393E+00   2.604331E+00   2.189055E+00   2.874439E+00
       1.185167E+00   4.346649E-01   2.280308E+00   3.023394E+00   2.781988E+00
      -4.194403E-02   2.937468E+00   1.326465E+00
Simulating for QAOA depth p = 10
Current parameters: [1.05162003 0.7899579  1.89844474 0.71412117 1.32153735 1.80568988
 2.47079708 2.42520352 2.18699007 0.70393923 0.01241239 0.93839749
```



```
 2.5070113  0.9523177  1.37590685 2.24997041 2.80750985 2.54129753
 0.92122184 0.09402042], Cost: 2.0459
Current parameters: [0.05162003 1.7899579  1.89844474 0.71412117 1.32153735 1.80568988
 2.47079708 2.42520352 2.18699007 0.70393923 0.01241239 0.93839749
 2.5070113  0.9523177  1.37590685 2.24997041 2.80750985 2.54129753
 0.92122184 0.09402042], Cost: 2.0452
Current parameters: [0.05162003 0.7899579  2.89844474 0.71412117 1.32153735 1.80568988
 2.47079708 2.42520352 2.18699007 0.70393923 0.01241239 0.93839749
 2.5070113  0.9523177  1.37590685 2.24997041 2.80750985 2.54129753
 0.92122184 0.09402042], Cost: 2.8973
Current parameters: [0.05162003 0.7899579  1.89844474 1.71412117 1.32153735 1.80568988
 2.47079708 2.42520352 2.18699007 0.70393923 0.01241239 0.93839749
 2.5070113  0.9523177  1.37590685 2.24997041 2.80750985 2.54129753
 0.92122184 0.09402042], Cost: 1.7812
Current parameters: [0.05162003 0.7899579  1.89844474 0.71412117 2.32153735 1.80568988
 2.47079708 2.42520352 2.18699007 0.70393923 0.01241239 0.93839749
 2.5070113  0.9523177  1.37590685 2.24997041 2.80750985 2.54129753
 0.92122184 0.09402042], Cost: 1.9856
Current parameters: [0.05162003 0.7899579  1.89844474 0.71412117 1.32153735 2.80568988
 2.47079708 2.42520352 2.18699007 0.70393923 0.01241239 0.93839749
 2.5070113  0.9523177  1.37590685 2.24997041 2.80750985 2.54129753
 0.92122184 0.09402042], Cost: 2.8314
Current parameters: [0.05162003 0.7899579  1.89844474 0.71412117 1.32153735 1.80568988
 3.47079708 2.42520352 2.18699007 0.70393923 0.01241239 0.93839749
 2.5070113  0.9523177  1.37590685 2.24997041 2.80750985 2.54129753
 0.92122184 0.09402042], Cost: 1.5817
Current parameters: [0.05162003 0.7899579  1.89844474 0.71412117 1.32153735 1.80568988
 2.47079708 3.42520352 2.18699007 0.70393923 0.01241239 0.93839749
 2.5070113  0.9523177  1.37590685 2.24997041 2.80750985 2.54129753
 0.92122184 0.09402042], Cost: 1.1159
Current parameters: [0.05162003 0.7899579  1.89844474 0.71412117 1.32153735 1.80568988
 2.47079708 2.42520352 3.18699007 0.70393923 0.01241239 0.93839749
 2.5070113  0.9523177  1.37590685 2.24997041 2.80750985 2.54129753
 0.92122184 0.09402042], Cost: 2.8825
Current parameters: [0.05162003 0.7899579  1.89844474 0.71412117 1.32153735 1.80568988
 2.47079708 2.42520352 2.18699007 1.70393923 0.01241239 0.93839749
 2.5070113  0.9523177  1.37590685 2.24997041 2.80750985 2.54129753
 0.92122184 0.09402042], Cost: 0.3399
Current parameters: [0.05162003 0.7899579  1.89844474 0.71412117 1.32153735 1.80568988
 2.47079708 2.42520352 2.18699007 0.70393923 1.01241239 0.93839749
 2.5070113  0.9523177  1.37590685 2.24997041 2.80750985 2.54129753
```

```
 0.92122184 0.09402042], Cost: 0.2037
Current parameters: [0.05162003 0.7899579  1.89844474 0.71412117 1.32153735 1.80568988
 2.47079708 2.42520352 2.18699007 0.70393923 0.01241239 1.93839749
 2.5070113  0.9523177  1.37590685 2.24997041 2.80750985 2.54129753
 0.92122184 0.09402042], Cost: 3.0941
Current parameters: [0.05162003 0.7899579  1.89844474 0.71412117 1.32153735 1.80568988
 2.47079708 2.42520352 2.18699007 0.70393923 0.01241239 0.93839749
 3.5070113  0.9523177  1.37590685 2.24997041 2.80750985 2.54129753
 0.92122184 0.09402042], Cost: 2.1495
Current parameters: [0.05162003 0.7899579  1.89844474 0.71412117 1.32153735 1.80568988
 2.47079708 2.42520352 2.18699007 0.70393923 0.01241239 0.93839749
 2.5070113  1.9523177  1.37590685 2.24997041 2.80750985 2.54129753
 0.92122184 0.09402042], Cost: 1.6784
Current parameters: [0.05162003 0.7899579  1.89844474 0.71412117 1.32153735 1.80568988
 2.47079708 2.42520352 2.18699007 0.70393923 0.01241239 0.93839749
 2.5070113  0.9523177  2.37590685 2.24997041 2.80750985 2.54129753
 0.92122184 0.09402042], Cost: 3.1674
Current parameters: [0.05162003 0.7899579  1.89844474 0.71412117 1.32153735 1.80568988
 2.47079708 2.42520352 2.18699007 0.70393923 0.01241239 0.93839749
 2.5070113  0.9523177  1.37590685 3.24997041 2.80750985 2.54129753
 0.92122184 0.09402042], Cost: 3.2948
Current parameters: [0.05162003 0.7899579  1.89844474 0.71412117 1.32153735 1.80568988
 2.47079708 2.42520352 2.18699007 0.70393923 0.01241239 0.93839749
 2.5070113  0.9523177  1.37590685 2.24997041 3.80750985 2.54129753
 0.92122184 0.09402042], Cost: 1.4033
Current parameters: [0.05162003 0.7899579  1.89844474 0.71412117 1.32153735 1.80568988
 2.47079708 2.42520352 2.18699007 0.70393923 0.01241239 0.93839749
 2.5070113  0.9523177  1.37590685 2.24997041 2.80750985 3.54129753
 0.92122184 0.09402042], Cost: 3.2149
Current parameters: [0.05162003 0.7899579  1.89844474 0.71412117 1.32153735 1.80568988
 2.47079708 2.42520352 2.18699007 0.70393923 0.01241239 0.93839749
 2.5070113  0.9523177  1.37590685 2.24997041 2.80750985 2.54129753
 1.92122184 0.09402042], Cost: 1.8500
Current parameters: [0.05162003 0.7899579  1.89844474 0.71412117 1.32153735 1.80568988
 2.47079708 2.42520352 2.18699007 0.70393923 0.01241239 0.93839749
 2.5070113  0.9523177  1.37590685 2.24997041 2.80750985 2.54129753
 0.92122184 1.09402042], Cost: 1.0894
Current parameters: [-0.15081404  0.58760003  1.60357033  0.54042492  1.12564605  1.51796686
  2.31875322  2.32373823  1.89372603  0.686716    0.00998409  0.62215319
  2.29332338  0.78977798  1.05170998  1.91194299  2.6748402   2.21194386
  0.74005674 -0.00456692], Cost: 3.2511
```



```
Current parameters: [-0.04959701  0.68877897  1.75100754  0.62727305  1.2235917   1.66182837
  2.39477515  2.37447087  2.04035805  0.69532761  0.01119824  0.78027534
  2.40016734  0.87104784  1.21380841  2.0809567   2.74117502  2.37662069
  0.83063929  0.04472675], Cost: 2.1878
Current parameters: [0.04078928 0.77913123 2.14316167 0.70482798 1.31105666 1.79029595
 2.46266234 2.41977486 2.17129969 0.70301774 0.01228247 0.92147761
 2.49557844 0.94362141 1.35856148 2.23188508 2.80041168 2.52367626
 0.91152904 0.08874574], Cost: 0.9173
Current parameters: [0.13999173 0.70155291 1.89844474 0.71412117 1.32153735 1.80568988
 2.47079708 2.42520352 2.18699007 0.70393923 0.01241239 0.93839749
 2.5070113  0.9523177  1.37590685 2.24997041 2.80750985 2.54129753
 0.92122184 0.09402042], Cost: 0.1794
Current parameters: [0.31337914 0.87474859 1.88699237 0.7049661  1.31121243 1.79052475
 2.46278325 2.41985555 2.1715329  0.70303144 0.0122844  0.92172909
 2.49574837 0.94375066 1.35881929 2.23215388 2.80051718 2.52393817
 0.91167311 0.08882414], Cost: 0.6949
Current parameters: [0.13999173 0.70155291 1.89844474 0.80764906 1.23860644 1.80568988
 2.47079708 2.42520352 2.18699007 0.70393923 0.01241239 0.93839749
 2.5070113  0.9523177  1.37590685 2.24997041 2.80750985 2.54129753
 0.92122184 0.09402042], Cost: 0.1441
Current parameters: [0.13571961 0.69716337 1.88766512 0.97192912 1.42158578 1.79141561
 2.46325401 2.42016971 2.17244091 0.70308476 0.01229191 0.92270825
 2.49640999 0.94425392 1.35982307 2.23320048 2.80092795 2.52495791
 0.91223403 0.08912939], Cost: 0.3687
Current parameters: [0.13999173 0.70155291 1.89844474 0.80764906 1.23860644 1.86409179
 2.36027913 2.42520352 2.18699007 0.70393923 0.01241239 0.93839749
 2.5070113  0.9523177  1.37590685 2.24997041 2.80750985 2.54129753
 0.92122184 0.09402042], Cost: 0.1954
Current parameters: [0.13426993 0.69567384 1.88400721 0.80900572 1.23706595 2.01962943
 2.58720429 2.41846155 2.16750385 0.70279481 0.01225103 0.91738432
 2.49281259 0.94151759 1.35436526 2.22750984 2.79869448 2.51941329
 0.90918415 0.08746968], Cost: 0.6142
Current parameters: [0.13999173 0.70155291 1.89844474 0.80764906 1.23860644 1.80568988
 2.47079708 2.5433329  2.14611895 0.70393923 0.01241239 0.93839749
 2.5070113  0.9523177  1.37590685 2.24997041 2.80750985 2.54129753
 0.92122184 0.09402042], Cost: 0.1086
Current parameters: [0.13417018 0.69557135 1.88375551 0.80902938 1.23703909 1.79982564
 2.47111002 2.62513067 2.37617049 0.70277486 0.01224822 0.91701798
 2.49256505 0.9413293  1.35398971 2.22711827 2.7985408  2.51903177
 0.90897429 0.08735548], Cost: 0.6268
Current parameters: [0.13999173 0.70155291 1.89844474 0.80764906 1.23860644 1.80568988
```



```
 2.47079708 2.5433329  2.14611895 0.5791242  0.01241239 0.94519514
 2.5070113  0.9523177  1.37590685 2.24997041 2.80750985 2.54129753
 0.92122184 0.09402042], Cost: 0.0828
Current parameters: [0.13363832 0.69502487 1.8824135  0.80915548 1.2368959  1.79928989
 2.47113861 2.543366   2.13926517 0.59074476 0.01223322 1.1889723
 2.49124525 0.9403254  1.35198737 2.2250305  2.79772138 2.51699758
 0.90785535 0.08674657], Cost: 0.8486
Current parameters: [ 0.13999173  0.70155291  1.89844474  0.80764906  1.23860644  1.80568988
  2.47079708  2.5433329   2.14611895  0.5791242  -0.11257954  0.94519514
  2.50843167  0.9523177   1.37590685  2.24997041  2.80750985  2.54129753
  0.92122184  0.09402042], Cost: 0.1177
Current parameters: [0.13568145 0.69712416 1.88756883 0.80867104 1.23744597 1.801348
 2.47102878 2.54335535 2.14146921 0.57753887 0.0167386  0.93671438
 2.75433538 0.94418189 1.3596794  2.23305069 2.80086916 2.52481197
 0.91215375 0.0890857 ], Cost: 0.3345
Current parameters: [0.13999173 0.70155291 1.89844474 0.80764906 1.23860644 1.80568988
 2.47079708 2.5433329  2.14611895 0.5791242  0.01241239 0.94519514
 2.5070113  1.06406019 1.3198835  2.24997041 2.80750985 2.54129753
 0.92122184 0.09402042], Cost: 0.2291
Current parameters: [0.13180611 0.6931423  1.87779038 0.80958991 1.23640261 1.79744425
 2.4712371  2.54337554 2.13728866 0.57611351 0.01528741 0.92908938
 2.50671188 1.05020095 1.59809336 2.21783828 2.79489857 2.5099899
 0.90400069 0.08464893], Cost: 0.5466
Current parameters: [0.13999173 0.70155291 1.89844474 0.80764906 1.23860644 1.80568988
 2.47079708 2.5433329  2.14611895 0.5791242  0.01241239 0.94519514
 2.5070113  0.9523177  1.37590685 2.29563913 2.69115106 2.54129753
 0.92122184 0.09402042], Cost: 0.1730
Current parameters: [0.13043697 0.69173552 1.87433569 0.80991454 1.23603399 1.79606507
 2.4713107  2.54338268 2.13581169 0.57560994 0.01576829 0.9263955
 2.5066618  0.93947775 1.38175614 2.47291842 2.90435466 2.50475332
 0.90112024 0.08308143], Cost: 0.8793
Current parameters: [0.13999173 0.70155291 1.89844474 0.80764906 1.23860644 1.80568988
 2.47079708 2.5433329  2.14611895 0.5791242  0.01241239 0.94519514
 2.5070113  0.9523177  1.37590685 2.24997041 2.80750985 2.60154263
 0.81169775 0.09402042], Cost: 0.1340
Current parameters: [0.12855394 0.68980074 1.86958433 0.81036101 1.23552701 1.79416823
 2.47141192 2.54339249 2.13378036 0.57491736 0.01642967 0.92269051
 2.50659292 0.93694728 1.3829089  2.23318856 2.81210608 2.75264806
 1.04421334 0.0809256 ], Cost: 0.7849
Current parameters: [0.14546155 0.70702067 1.90641231 0.81234237 1.24389947 1.81346421
 2.47490534 2.54607451 2.154043   0.57958957 0.012478   0.95374013
```



```
 2.51278519 0.95670956 1.38466672 2.25910399 2.81109461 2.55019674
 0.92611696 0.21609419], Cost: 0.1504
Current parameters: [0.08578344 0.64702693 1.80654025 0.77442483 1.19462521 1.73366604
 2.43984833 2.52230742 2.07207679 0.57108155 0.01616953 0.8555918
 2.46215424 0.90222907 1.31595361 2.1619108  2.78481163 2.46774808
 0.88791059 0.13899078], Cost: 0.8480
Current parameters: [0.13159448 0.69302767 2.01938533 0.80559852 1.23376805 1.79570327
 2.46827828 2.54150767 2.13569514 0.57707904 0.01402128 0.93016893
 2.50294352 0.94302941 1.37287806 2.23690209 2.80698264 2.53331652
 0.91960354 0.08876911], Cost: 0.3094
Current parameters: [0.15802838 0.71958277 1.89974811 0.76996248 1.19610424 1.80741579
 2.47170912 2.54394154 2.14787809 0.57922751 0.01242695 0.94709213
 2.50829311 0.9532927  1.37785155 2.25199807 2.80830567 2.54327316
 0.92230856 0.0946118 ], Cost: 0.0738
Current parameters: [0.23643161 0.79784853 1.89516053 0.80135553 1.22942933 1.79514317
 2.46749473 2.54101029 2.13516754 0.57730134 0.01371728 0.9302691
 2.50193558 0.94335756 1.37079339 2.23648015 2.80600365 2.53206549
 0.91842548 0.08890055], Cost: 0.2029
Current parameters: [0.15794706 0.71950148 1.89954573 0.76996013 1.19610159 1.84697604
 2.49261427 2.52967553 2.1066452  0.57921147 0.01242469 0.94679758
 2.50809407 0.9531413  1.37754958 2.25168323 2.8081821  2.54296639
 0.92213982 0.09451997], Cost: 0.0610
Current parameters: [0.15743922 0.71884887 1.89239202 0.77181947 1.19540663 1.91842894
 2.53342178 2.55860811 2.18457809 0.57654649 0.01414603 0.92276319
 2.4985804  0.93905821 1.36658956 2.22930147 2.80448425 2.52641145
 0.91609726 0.0864007 ], Cost: 0.2936
Current parameters: [0.20211251 0.67528729 1.89950578 0.76995966 1.19610107 1.84696393
 2.49260787 2.52967026 2.10662997 0.57921129 0.01243454 0.94679429
 2.50896097 0.95311142 1.37748997 2.25162107 2.8081577  2.54290584
 0.92210651 0.09450184], Cost: 0.0599
Current parameters: [0.20002768 0.67561413 1.89503296 0.77068489 1.19580194 1.84574029
 2.49318118 2.52976287 2.10462082 0.57844202 0.01456887 0.94263129
 2.63236744 0.94627194 1.37070174 2.24016049 2.80569477 2.53384085
 0.91834599 0.09052254], Cost: 0.0960
Current parameters: [0.19725675 0.67043872 1.89263832 0.76563342 1.19122201 1.83993583
 2.48889395 2.52720394 2.09950159 0.57876455 0.01237453 0.93859157
 2.50368036 0.92670322 1.32481697 2.24929669 2.80724542 2.5406411
 0.92086076 0.10282465], Cost: 0.2047
Current parameters: [0.18284314 0.65567962 1.85910825 0.75865256 1.17889178 1.81900926
 2.4827035  2.52194318 2.07521112 0.57467529 0.01522167 0.90325812
 2.49719798 0.9677569  1.43404968 2.19824866 2.79533094 2.49928879
```

```
 0.90300573 0.10593957], Cost: 0.2410
Current parameters: [0.2025809  0.67576855 1.90068763 0.76997338 1.19611654 1.84732235
 2.49279727 2.52982613 2.10708047 0.57916952 0.01243588 0.94602732
 2.50907915 0.95320126 1.37766916 2.28941765 2.82299217 2.50130341
 0.89922258 0.09503808], Cost: 0.1019
Current parameters: [0.1985298  0.67123078 1.8774244  0.77356693 1.19359415 1.84022131
 2.49622561 2.53051421 2.09566728 0.57441741 0.01683255 0.9174098
 2.51683498 0.95354128 1.41868927 2.317595   2.84603063 2.60791897
 0.96508307 0.07492045], Cost: 0.1838
Current parameters: [0.20094285 0.67373417 1.88434762 0.77340006 1.19382668 1.84232388
 2.4968772  2.53118693 2.09846918 0.58268324 0.0164541  1.06543058
 2.50779261 0.94701833 1.4030974  2.24050136 2.81500624 2.54317311
 0.92900711 0.0786736 ], Cost: 0.2243
Current parameters: [0.19844838 0.67144543 1.95937456 0.76959649 1.19344436 1.8415857
 2.49221993 2.52883694 2.09964116 0.57788362 0.01390859 0.94245673
 2.50909759 0.95131537 1.38769497 2.24581005 2.80997107 2.54357617
 0.92494115 0.08881748], Cost: 0.1009
Current parameters: [0.20211251 0.67528729 1.89950578 0.79268945 1.17594663 1.84696393
 2.49260787 2.52967026 2.10662997 0.57921129 0.01243454 0.94679429
 2.50896097 0.95966332 1.3742051  2.25162107 2.8081577  2.54290584
 0.92210651 0.09450184], Cost: 0.0631
Current parameters: [0.20225668 0.67534942 1.90164783 0.7588886  1.20593743 1.84604112
 2.49383061 2.53018266 2.10491876 0.57855066 0.01347635 0.94863103
 2.51033072 1.01010364 1.35782025 2.25125311 2.81086663 2.54552366
 0.92526507 0.09123217], Cost: 0.0716
Current parameters: [0.20211251 0.67528729 1.89950578 0.76995966 1.19610107 1.85525759
 2.4769132  2.5539754  2.09822073 0.57921129 0.01243454 0.94679429
 2.50896097 0.95311142 1.37748997 2.25162107 2.8081577  2.54290584
 0.92210651 0.09450184], Cost: 0.0569
Current parameters: [0.2021756  0.67531448 1.9004431  0.76970979 1.19633124 1.87916865
 2.43143558 2.5204325  2.10915489 0.57892221 0.01289042 0.94759802
 2.50956035 0.95529753 1.38029018 2.25146006 2.80934308 2.54405135
 0.92348865 0.09307109], Cost: 0.0736
Current parameters: [0.2029378  0.67611227 1.90070793 0.77066779 1.19689968 1.85643059
 2.47753305 2.55438905 2.09941632 0.57928151 0.01244444 0.94808357
 2.50983214 0.95377406 1.37881167 2.25299916 2.80869857 2.53223952
 0.94467722 0.11292043], Cost: 0.0570
Current parameters: [0.20442229 0.6775464  1.90404388 0.77151954 1.19857096 1.85835196
 2.4783855  2.55495498 2.1005547  0.57899907 0.01309402 0.95138153
 2.51213874 0.95793033 1.38493592 2.25510823 2.81125956 2.56663946
 0.89233036 0.14211028], Cost: 0.0610
```



```
Current parameters: [0.20211251 0.67528729 1.89950578 0.76995966 1.19610107 1.85525759
 2.4769132  2.5539754  2.09822073 0.60429218 0.03102489 0.94542834
 2.50874972 0.95311142 1.37748997 2.25162107 2.8081577  2.54290584
 0.92210651 0.09450184], Cost: 0.0568
Current parameters: [ 0.20219878  0.67534748  1.9002451   0.76981302  1.19630731  1.85528161
  2.47684072  2.55392549  2.09781645  0.6414045  -0.01913058  0.94404252
  2.50980122  0.95473071  1.37958733  2.25157179  2.80904464  2.54414168
  0.92253054  0.09436667], Cost: 0.0740
Current parameters: [0.19106104 0.66425523 1.90015618 0.76546323 1.19132394 1.85699473
 2.47751023 2.55439062 2.10001982 0.60456456 0.03084256 0.94780883
 2.50965022 0.95451703 1.37849054 2.26305502 2.7849477  2.54449287
 0.92265701 0.09530991], Cost: 0.0648
Current parameters: [0.2354107  0.70842876 1.90036668 0.78281884 1.21113476 1.85016597
 2.47485736 2.55254751 2.09132144 0.60341213 0.03187262 0.94073749
 2.50788432 0.95505347 1.38245186 2.25891169 2.7747978  2.54285372
 0.92207083 0.09157884], Cost: 0.0775
Current parameters: [0.20262923 0.67519046 1.90033189 0.7698184  1.19610944 1.85833985
 2.47835885 2.55503985 2.10163911 0.60437938 0.0305665  0.94553404
 2.47808138 0.95425383 1.37873977 2.25358211 2.80862187 2.54450108
 0.92279999 0.09516938], Cost: 0.0926
Current parameters: [0.20543315 0.67874593 1.89952761 0.77187455 1.19887254 1.89079628
 2.49701716 2.56855204 2.13527747 0.60260948 0.03279143 0.93564717
 2.52551927 0.95444502 1.38352533 2.23933171 2.80627193 2.54018343
 0.92088207 0.08862613], Cost: 0.0816
Current parameters: [0.20280751 0.67599486 1.90140132 0.76998329 1.19634312 1.85610168
 2.47712399 2.55414248 2.09914271 0.60572762 0.0308679  0.96986406
 2.50735637 0.95246547 1.37487995 2.23845847 2.80259917 2.53201883
 0.9158816  0.09521154], Cost: 0.1056
Current parameters: [0.20305701 0.676164   1.88661715 0.77239192 1.19721295 1.85234011
 2.48047531 2.55652678 2.09172841 0.60202279 0.03559124 0.96262087
 2.52639926 0.95469926 1.40255525 2.27609408 2.82749292 2.57267003
 0.94184383 0.08228171], Cost: 0.0240
Current parameters: [0.19632517 0.66935978 1.86507294 0.76762055 1.1913289  1.83992479
 2.47749618 2.55465569 2.07567395 0.59809741 0.03903564 0.93547603
 2.53570811 0.96687017 1.440769   2.25694704 2.82706247 2.56667097
 0.94053356 0.08454898], Cost: 0.0574
Current parameters: [0.21190749 0.68501132 1.88705473 0.75345078 1.1758515  1.85298358
 2.48081534 2.55675407 2.09238536 0.6020605  0.03559665 0.96331332
 2.52687527 0.95436735 1.40189323 2.27703393 2.82786179 2.57358575
 0.94234753 0.08283478], Cost: 0.0288
Current parameters: [0.20028162 0.67333666 1.87482262 0.77322087 1.20111671 1.84736019
```



```
 2.48015006 2.55666707 2.08057967 0.5982998  0.03939519 0.94179369
 2.54780593 0.93537747 1.36565849 2.28485612 2.83680948 2.59705025
 0.95462722 0.08542244], Cost: 0.0914
Current parameters: [0.20392992 0.67714519 1.88806838 0.77248084 1.19766753 1.85068519
 2.47921385 2.55562651 2.08984853 0.60470396 0.04102581 0.95606295
 2.53163202 0.95500998 1.40100121 2.2922239  2.83976801 2.55440931
 0.9314105  0.08331276], Cost: 0.0290
Current parameters: [0.20227665 0.67477088 1.87731802 0.77442998 1.2002963  1.8641366
 2.49096883 2.56425926 2.09944148 0.58407747 0.00919861 0.98447842
 2.52230026 0.95839945 1.42343252 2.31090953 2.81380963 2.57293029
 0.9435517  0.07391697], Cost: 0.0812
Current parameters: [0.20161077 0.67466442 1.91518903 0.77268338 1.19689863 1.84998798
 2.48093517 2.55686702 2.08822644 0.60027493 0.03602192 0.95925441
 2.5302902  0.95476845 1.41021937 2.27607793 2.82948548 2.57694713
 0.94537651 0.07849195], Cost: 0.0260
Current parameters: [0.2030524  0.67615794 1.88625771 0.77437445 1.19545222 1.84588473
 2.47674998 2.5588217  2.09894755 0.60214678 0.03540032 0.96233294
 2.52618124 0.94424507 1.4062249  2.27621436 2.82701619 2.57224071
 0.9412921  0.08289746], Cost: 0.0220
Current parameters: [0.20326886 0.67636391 1.88692767 0.77099951 1.19905724 1.83093178
 2.46964371 2.56463614 2.11369557 0.60144801 0.0356243  0.9624923
 2.52838578 0.96363919 1.40100507 2.27742906 2.82845108 2.57495593
 0.94335225 0.08138641], Cost: 0.0425
Current parameters: [0.2140715  0.66512814 1.88626285 0.77437226 1.19545407 1.84626644
 2.47601664 2.5582778  2.09912066 0.60214208 0.03541013 0.96234507
 2.52640717 0.94427341 1.40625488 2.27619626 2.82702922 2.57224406
 0.94130607 0.08286987], Cost: 0.0218
Current parameters: [0.21281325 0.666704   1.88658937 0.77466312 1.19576707 1.85857757
 2.45375041 2.54166822 2.10427544 0.60155197 0.03550675 0.96219135
 2.52822904 0.94490167 1.40907242 2.27726531 2.82803994 2.57440174
 0.94283913 0.08177265], Cost: 0.0147
Current parameters: [0.21279555 0.66671728 1.88563224 0.77460826 1.19558052 1.86218413
 2.45583717 2.54317114 2.10828165 0.60198362 0.03641057 0.95885303
 2.52958018 0.94398267 1.40800439 2.27495135 2.82797173 2.56906021
 0.94912794 0.07090446], Cost: 0.0156
Current parameters: [0.21507012 0.66903292 1.88773864 0.77634095 1.19753222 1.87399363
 2.46230974 2.54746473 2.11999566 0.60257867 0.0391332  0.95516841
 2.53664671 0.94634143 1.41344352 2.27344386 2.83105328 2.58040148
 0.93531931 0.09153788], Cost: 0.0183
Current parameters: [0.2211388  0.67502312 1.88675856 0.77796365 1.19945971 1.85710669
 2.45315294 2.54115457 2.10245527 0.60071859 0.03877394 0.95949654
```



```
 2.52931279 0.94521884 1.40988684 2.27887848 2.82087221 2.57439172
 0.94283643 0.08112864], Cost: 0.0133
Current parameters: [0.21754148 0.67137588 1.88633392 0.77665054 1.19783646 1.8581704
 2.45367268 2.54155904 2.10358462 0.58005805 0.0604756  0.96491523
 2.52554718 0.94447124 1.40959437 2.27927376 2.82007609 2.57401895
 0.94279367 0.0811774 ], Cost: 0.0167
Current parameters: [0.22097697 0.6748521  1.88665199 0.78922105 1.18936324 1.85712494
 2.45318644 2.54117348 2.10246541 0.60033878 0.03855446 0.95855424
 2.52901632 0.94854651 1.40848277 2.27970555 2.82088623 2.57500718
 0.94319021 0.08107528], Cost: 0.0187
Current parameters: [0.22436517 0.67861356 1.88853957 0.78114889 1.20087552 1.85769472
 2.45123039 2.53948628 2.0993855  0.60724113 0.04273173 0.98019358
 2.54211306 0.94166663 1.4105211  2.26530765 2.82283854 2.56703023
 0.93893088 0.08025135], Cost: 0.0303
Current parameters: [0.22136006 0.67521887 1.88684545 0.77802881 1.1995429  1.86134347
 2.44537565 2.55327063 2.09841273 0.60118042 0.03907032 0.95934607
 2.52792995 0.94523231 1.40979458 2.27862563 2.82122412 2.57427943
 0.94275603 0.08120262], Cost: 0.0114
Current parameters: [0.22662251 0.68009812 1.88855689 0.77952449 1.20377506 1.86426061
 2.44600895 2.55027945 2.09918833 0.61101591 0.04565213 0.95866845
 2.50371766 0.94231119 1.41371179 2.27542978 2.83192239 2.57674074
 0.94416833 0.08078098], Cost: 0.0111
Current parameters: [0.22619205 0.67966788 1.88796129 0.77916247 1.20336781 1.86367787
 2.44569875 2.5500724  2.09859562 0.61101306 0.04566833 0.95801997
 2.50327717 0.94198155 1.4130439  2.27475041 2.83160856 2.58206612
 0.93287714 0.07157725], Cost: 0.0146
Current parameters: [0.21868124 0.67220168 1.8913902  0.77585198 1.20123931 1.86696071
 2.44532185 2.54957883 2.10064905 0.62778958 0.05675977 0.95766937
 2.50074837 0.94169419 1.41282889 2.28065422 2.81299068 2.57464235
 0.94321687 0.08324855], Cost: 0.0089
Current parameters: [0.21866757 0.67220602 1.89497484 0.77546904 1.20084149 1.86797382
 2.44465238 2.54906445 2.10293663 0.62370362 0.05190436 0.95652044
 2.49533731 0.94324492 1.41153422 2.27502519 2.80834421 2.56652373
 0.93816016 0.08532343], Cost: 0.0135
Current parameters: [0.21772434 0.6713312  1.89208108 0.77407139 1.20613517 1.86997437
 2.44151688 2.54619136 2.09420269 0.62719155 0.05684568 0.94491496
 2.50161744 0.92565235 1.39469427 2.27988912 2.81292578 2.58184933
 0.94915843 0.08897997], Cost: 0.0227
Current parameters: [0.22006266 0.67352745 1.89470772 0.77686145 1.20257566 1.87085156
 2.44714237 2.55085448 2.10480106 0.6265234  0.05303876 0.96567665
 2.49843979 0.93965044 1.40746877 2.28880335 2.81162178 2.57569877
```

```
 0.94357159 0.08182821], Cost: 0.0093
Current parameters: [0.21651613 0.66984333 1.88343883 0.77341043 1.20498233 1.87338316
 2.44568764 2.54909508 2.09505463 0.62437064 0.0494477  0.95678525
 2.49738893 0.93779586 1.43576426 2.29186617 2.80711804 2.57603194
 0.94863007 0.08267964], Cost: 0.0384
Current parameters: [0.21830331 0.67177047 1.89052016 0.77538636 1.20143772 1.86601134
 2.44508827 2.55070192 2.10333776 0.62642173 0.05414898 0.96089703
 2.49844192 0.94387089 1.41288729 2.2729818  2.80719594 2.58378106
 0.94848998 0.08260098], Cost: 0.0150
Current parameters: [0.21937623 0.67300528 1.89240361 0.77135381 1.20845046 1.8535875
 2.43488199 2.55316553 2.11322654 0.63217402 0.06159462 0.95383085
 2.50206777 0.95677113 1.40688656 2.28897632 2.81960907 2.57202754
 0.94428428 0.08398633], Cost: 0.0098
Current parameters: [0.22105973 0.67460395 1.87840593 0.77204416 1.19723905 1.86816143
 2.44517747 2.54946729 2.10237835 0.62859639 0.05656382 0.95934356
 2.49906094 0.94159815 1.40919479 2.28084393 2.81215081 2.57286359
 0.94169869 0.08508933], Cost: 0.0072
Current parameters: [0.22885923 0.68240669 1.89020609 0.75395279 1.17832542 1.86762745
 2.44442549 2.54951926 2.10184013 0.62921299 0.05775533 0.95790237
 2.50058429 0.94097198 1.41125814 2.28285329 2.81411044 2.57512062
 0.94402161 0.08461748], Cost: 0.0009
Current parameters: [0.23983171 0.67142453 1.89024438 0.75376763 1.17848977 1.86860332
 2.4440391  2.54876588 2.10134622 0.62919686 0.05778271 0.95794103
 2.50082938 0.94196506 1.41094931 2.28282416 2.81416741 2.57516359
 0.94408644 0.08453318], Cost: 0.0009
Current parameters: [0.22719672 0.68487746 1.89111694 0.74891486 1.18206735 1.88035303
 2.45103601 2.5447878  2.08752564 0.62998926 0.05895982 0.95787755
 2.5006376  0.96164769 1.40361393 2.28356751 2.81575271 2.5757933
 0.94552364 0.08380554], Cost: 0.0047

   Return from subroutine COBYLA because the MAXFUN limit has been reached.

   NFVALS =  100   F = 8.709526E-04    MAXCV = 0.000000E+00
   X = 2.288592E-01   6.824067E-01   1.890206E+00   7.539528E-01   1.178325E+00
       1.867627E+00   2.444425E+00   2.549519E+00   2.101840E+00   6.292130E-01
       5.775533E-02   9.579024E-01   2.500584E+00   9.409720E-01   1.411258E+00
       2.282853E+00   2.814110E+00   2.575121E+00   9.440216E-01   8.461748E-02
Current parameters: [0.22885923 0.68240669 1.89020609 0.75395279 1.17832542 1.86762745
 2.44442549 2.54951926 2.10184013 0.62921299 0.05775533 0.95790237
 2.50058429 0.94097198 1.41125814 2.28285329 2.81411044 2.57512062
 0.94402161 0.08461748], Cost: 0.0009
```



```
Simulating for QAOA depth p = 11
Current parameters: [3.46315111 2.74499757 2.71581317 2.44511033 0.4209131  1.87821697
 2.18952179 0.42946008 0.39134069 2.23150232 2.92931397 2.03393868
 1.92760028 1.48955087 1.4368936  1.88871848 0.61275793 2.21681887
 0.0545086  0.50763975 1.16314868 2.38891297], Cost: 1.7795
Current parameters: [2.46315111 3.74499757 2.71581317 2.44511033 0.4209131  1.87821697
 2.18952179 0.42946008 0.39134069 2.23150232 2.92931397 2.03393868
 1.92760028 1.48955087 1.4368936  1.88871848 0.61275793 2.21681887
 0.0545086  0.50763975 1.16314868 2.38891297], Cost: 1.3937
Current parameters: [2.46315111 2.74499757 3.71581317 2.44511033 0.4209131  1.87821697
 2.18952179 0.42946008 0.39134069 2.23150232 2.92931397 2.03393868
 1.92760028 1.48955087 1.4368936  1.88871848 0.61275793 2.21681887
 0.0545086  0.50763975 1.16314868 2.38891297], Cost: 0.8204
Current parameters: [2.46315111 2.74499757 2.71581317 3.44511033 0.4209131  1.87821697
 2.18952179 0.42946008 0.39134069 2.23150232 2.92931397 2.03393868
 1.92760028 1.48955087 1.4368936  1.88871848 0.61275793 2.21681887
 0.0545086  0.50763975 1.16314868 2.38891297], Cost: 1.1100
Current parameters: [2.46315111 2.74499757 2.71581317 2.44511033 1.4209131  1.87821697
 2.18952179 0.42946008 0.39134069 2.23150232 2.92931397 2.03393868
 1.92760028 1.48955087 1.4368936  1.88871848 0.61275793 2.21681887
 0.0545086  0.50763975 1.16314868 2.38891297], Cost: 1.7026
Current parameters: [2.46315111 2.74499757 2.71581317 2.44511033 0.4209131  2.87821697
 2.18952179 0.42946008 0.39134069 2.23150232 2.92931397 2.03393868
 1.92760028 1.48955087 1.4368936  1.88871848 0.61275793 2.21681887
 0.0545086  0.50763975 1.16314868 2.38891297], Cost: 0.5760
Current parameters: [2.46315111 2.74499757 2.71581317 2.44511033 0.4209131  2.87821697
 3.18952179 0.42946008 0.39134069 2.23150232 2.92931397 2.03393868
 1.92760028 1.48955087 1.4368936  1.88871848 0.61275793 2.21681887
 0.0545086  0.50763975 1.16314868 2.38891297], Cost: 2.9016
Current parameters: [2.46315111 2.74499757 2.71581317 2.44511033 0.4209131  2.87821697
 2.18952179 1.42946008 0.39134069 2.23150232 2.92931397 2.03393868
 1.92760028 1.48955087 1.4368936  1.88871848 0.61275793 2.21681887
 0.0545086  0.50763975 1.16314868 2.38891297], Cost: 0.5001
Current parameters: [2.46315111 2.74499757 2.71581317 2.44511033 0.4209131  2.87821697
 2.18952179 1.42946008 1.39134069 2.23150232 2.92931397 2.03393868
 1.92760028 1.48955087 1.4368936  1.88871848 0.61275793 2.21681887
 0.0545086  0.50763975 1.16314868 2.38891297], Cost: 1.3550
Current parameters: [2.46315111 2.74499757 2.71581317 2.44511033 0.4209131  2.87821697
 2.18952179 1.42946008 0.39134069 3.23150232 2.92931397 2.03393868
 1.92760028 1.48955087 1.4368936  1.88871848 0.61275793 2.21681887
 0.0545086  0.50763975 1.16314868 2.38891297], Cost: 2.5175
```



```
Current parameters: [2.46315111 2.74499757 2.71581317 2.44511033 0.4209131  2.87821697
 2.18952179 1.42946008 0.39134069 2.23150232 3.92931397 2.03393868
 1.92760028 1.48955087 1.4368936  1.88871848 0.61275793 2.21681887
 0.0545086  0.50763975 1.16314868 2.38891297], Cost: 1.8418
Current parameters: [2.46315111 2.74499757 2.71581317 2.44511033 0.4209131  2.87821697
 2.18952179 1.42946008 0.39134069 2.23150232 2.92931397 3.03393868
 1.92760028 1.48955087 1.4368936  1.88871848 0.61275793 2.21681887
 0.0545086  0.50763975 1.16314868 2.38891297], Cost: 2.6654
Current parameters: [2.46315111 2.74499757 2.71581317 2.44511033 0.4209131  2.87821697
 2.18952179 1.42946008 0.39134069 2.23150232 2.92931397 2.03393868
 2.92760028 1.48955087 1.4368936  1.88871848 0.61275793 2.21681887
 0.0545086  0.50763975 1.16314868 2.38891297], Cost: 2.2474
Current parameters: [2.46315111 2.74499757 2.71581317 2.44511033 0.4209131  2.87821697
 2.18952179 1.42946008 0.39134069 2.23150232 2.92931397 2.03393868
 1.92760028 2.48955087 1.4368936  1.88871848 0.61275793 2.21681887
 0.0545086  0.50763975 1.16314868 2.38891297], Cost: 1.8053
Current parameters: [2.46315111 2.74499757 2.71581317 2.44511033 0.4209131  2.87821697
 2.18952179 1.42946008 0.39134069 2.23150232 2.92931397 2.03393868
 1.92760028 1.48955087 2.4368936  1.88871848 0.61275793 2.21681887
 0.0545086  0.50763975 1.16314868 2.38891297], Cost: 3.3826
Current parameters: [2.46315111 2.74499757 2.71581317 2.44511033 0.4209131  2.87821697
 2.18952179 1.42946008 0.39134069 2.23150232 2.92931397 2.03393868
 1.92760028 1.48955087 1.4368936  2.88871848 0.61275793 2.21681887
 0.0545086  0.50763975 1.16314868 2.38891297], Cost: 2.5534
Current parameters: [2.46315111 2.74499757 2.71581317 2.44511033 0.4209131  2.87821697
 2.18952179 1.42946008 0.39134069 2.23150232 2.92931397 2.03393868
 1.92760028 1.48955087 1.4368936  1.88871848 1.61275793 2.21681887
 0.0545086  0.50763975 1.16314868 2.38891297], Cost: 2.9834
Current parameters: [2.46315111 2.74499757 2.71581317 2.44511033 0.4209131  2.87821697
 2.18952179 1.42946008 0.39134069 2.23150232 2.92931397 2.03393868
 1.92760028 1.48955087 1.4368936  1.88871848 0.61275793 3.21681887
 0.0545086  0.50763975 1.16314868 2.38891297], Cost: 0.6237
Current parameters: [2.46315111 2.74499757 2.71581317 2.44511033 0.4209131  2.87821697
 2.18952179 1.42946008 0.39134069 2.23150232 2.92931397 2.03393868
 1.92760028 1.48955087 1.4368936  1.88871848 0.61275793 2.21681887
 1.0545086  0.50763975 1.16314868 2.38891297], Cost: 2.8545
Current parameters: [2.46315111 2.74499757 2.71581317 2.44511033 0.4209131  2.87821697
 2.18952179 1.42946008 0.39134069 2.23150232 2.92931397 2.03393868
 1.92760028 1.48955087 1.4368936  1.88871848 0.61275793 2.21681887
 0.0545086  1.50763975 1.16314868 2.38891297], Cost: 2.6774
Current parameters: [2.46315111 2.74499757 2.71581317 2.44511033 0.4209131  2.87821697
```



```
 2.18952179 1.42946008 0.39134069 2.23150232 2.92931397 2.03393868
 1.92760028 1.48955087 1.4368936  1.88871848 0.61275793 2.21681887
 0.0545086  0.50763975 2.16314868 2.38891297], Cost: 2.5529
Current parameters: [2.46315111 2.74499757 2.71581317 2.44511033 0.4209131  2.87821697
 2.18952179 1.42946008 0.39134069 2.23150232 2.92931397 2.03393868
 1.92760028 1.48955087 1.4368936  1.88871848 0.61275793 2.21681887
 0.0545086  0.50763975 1.16314868 3.38891297], Cost: 2.5539
Current parameters: [ 2.3348392   2.66595556  2.71000859  2.40231157  0.30241315  2.90363496
  1.89245559  1.43914641  0.28214432  1.97381026  2.75793334  1.75735353
  1.70440955  1.32283186  1.06870065  1.626447    0.29555897  2.20103299
 -0.24622745  0.22951916  0.90094129  2.12657585], Cost: 1.8573
Current parameters: [2.44615832 2.73452976 2.71504445 2.43944234 0.40521974 2.88158316
 2.6732667  1.43074288 0.37687944 2.19737527 2.90661744 1.99730955
 1.89804236 1.46747169 1.38813254 1.85398496 0.57075017 2.21472829
 0.01468109 0.47080728 1.12842364 2.35417076], Cost: 2.2683
Current parameters: [2.59427271 2.53214286 2.71581317 2.44511033 0.4209131  2.87821697
 2.18952179 1.42946008 0.39134069 2.23150232 2.92931397 2.03393868
 1.92760028 1.48955087 1.4368936  1.88871848 0.61275793 2.21681887
 0.0545086  0.50763975 1.16314868 2.38891297], Cost: 0.8653
Current parameters: [2.87895692 3.01504041 2.71544498 2.44239557 0.41339655 2.87982925
 2.16491766 1.43007449 0.38441428 2.2151567  2.91844316 2.01639466
 1.91344311 1.47897575 1.41353882 1.87208238 0.59263774 2.21581756
 0.03543267 0.48999834 1.14651665 2.37227271], Cost: 0.6170
Current parameters: [2.46315111 2.74499757 2.4680812  2.478709   0.4209131  2.87821697
 2.18952179 1.42946008 0.39134069 2.23150232 2.92931397 2.03393868
 1.92760028 1.48955087 1.4368936  1.88871848 0.61275793 2.21681887
 0.0545086  0.50763975 1.16314868 2.38891297], Cost: 1.0201
Current parameters: [2.46110035 2.74774768 2.78778025 2.93954024 0.41874288 2.87868247
 2.18241794 1.42963748 0.38934086 2.22678292 2.92617529 2.02887327
 1.92351274 1.48649756 1.43015047 1.88391521 0.60694871 2.21652977
 0.04900089 0.50254622 1.15834658 2.3841085 ], Cost: 0.4030
Current parameters: [2.46110035 2.74774768 2.78778025 2.93954024 0.47117475 3.12312244
 2.18241794 1.42963748 0.38934086 2.22678292 2.92617529 2.02887327
 1.92351274 1.48649756 1.43015047 1.88391521 0.60694871 2.21652977
 0.04900089 0.50254622 1.15834658 2.3841085 ], Cost: 1.1032
Current parameters: [2.45530249 2.75552269 2.8015922  2.93902909 0.90043102 2.75641609
 2.16233419 1.43013901 0.38368699 2.21344039 2.9173017  2.01455251
 1.91195659 1.47786534 1.41108653 1.87033557 0.59052509 2.21571242
 0.03342967 0.48814596 1.14477026 2.37052546], Cost: 1.4979
Current parameters: [2.46110035 2.74774768 2.78778025 2.93954024 0.41874288 2.87868247
 2.18241794 1.42963748 0.38934086 2.22678292 2.92617529 2.02887327
```

```
 1.92351274 1.48649756 1.59332376 1.88391521 0.41754312 2.21652977
 0.04900089 0.50254622 1.15834658 2.3841085 ], Cost: 0.6481
Current parameters: [ 2.43613888  2.78122139  2.84724478  2.93733956  0.33927693  2.81416276
  2.09595145  1.43179671  0.36499929  2.16933937  2.88797189  1.96721816
  1.87376007  1.44933331  1.74594225  1.82545083  0.91584708  2.21301085
 -0.01803783  0.44054884  1.0998965   2.32562949], Cost: 1.9262
Current parameters: [2.46110035 2.74774768 2.78778025 2.93954024 0.41874288 2.87868247
 2.18241794 1.42963748 0.38934086 2.22678292 2.92617529 2.02887327
 1.92351274 1.48649756 1.43015047 1.88391521 0.60694871 2.21652977
 0.21874156 0.31900303 1.15834658 2.3841085 ], Cost: 0.7865
Current parameters: [2.44006872 2.77595141 2.8378829  2.93768603 0.35178776 2.82432051
 2.10956442 1.43145677 0.36883154 2.17838307 2.89398649 1.97692491
 1.88159295 1.45518431 1.35523603 1.83465525 0.57345245 2.21356486
 0.35956562 0.83987478 1.10909866 2.33483621], Cost: 3.5015
Current parameters: [2.46110035 2.74774768 2.78778025 2.93954024 0.41874288 2.87868247
 2.18241794 1.42963748 0.38934086 2.22678292 2.92617529 2.20091622
 1.92351274 1.48649756 1.43015047 1.88391521 0.60694871 2.21652977
 0.04900089 0.50254622 1.15834658 2.20272156], Cost: 0.3847
Current parameters: [ 2.44439491  2.77014992  2.82757682  2.93806744  0.36556038  2.83550273
  2.12455033  1.43108254  0.37305029  2.18833888  2.9006077   2.53675501
  1.89021582  1.46162542  1.37064588  1.84478799  0.58034261  2.21417474
 -0.05852509  0.44291533  1.11922892  2.5193356 ], Cost: 1.0487
Current parameters: [2.46110035 2.74774768 2.78778025 2.93954024 0.41874288 2.87868247
 2.18241794 1.42963748 0.38934086 2.22678292 2.92617529 2.20091622
 1.92351274 1.48649756 1.43015047 2.0606703  0.60694871 2.21652977
 0.04900089 0.50254622 0.98154828 2.20272156], Cost: 0.3539
Current parameters: [ 2.44448066  2.77003493  2.82737254  2.938075    0.36583337  2.83572437
  2.12484737  1.43107513  0.37313391  2.18853622  2.90073895  2.16932729
  1.89038674  1.4617531   1.37095132  2.38979166  0.58047918  2.21418682
 -0.05797314  0.44322142  1.30728691  2.17084546], Cost: 1.1741
Current parameters: [2.46110035 2.74774768 2.78778025 2.93954024 0.41874288 2.87868247
 2.18241794 1.42963748 0.38934086 2.39045592 2.92617529 2.20091622
 1.73453881 1.48649756 1.43015047 2.0606703  0.60694871 2.21652977
 0.04900089 0.50254622 0.98154828 2.20272156], Cost: 0.9880
Current parameters: [ 2.4457958   2.76827131  2.82423955  2.93819094  0.37002017  2.8391237
  2.12940301  1.43096136  0.37441639  2.55069174  2.90275176  2.17182696
  2.26263834  1.46371116  1.37563583  2.02996103  0.58257375  2.21437223
 -0.04950815  0.44791587  0.94780553  2.17336786], Cost: 0.2070
Current parameters: [ 2.42707951  2.7567418   2.82339286  2.93194808  0.35273511  2.84283131
  2.08607127  1.43096136  0.37441639  2.51310334  2.69611064  2.13148271
  2.23008249  1.46371116  1.32192915  1.99170464  0.53630534  2.21437223
```



```
 -0.09337519  0.40734765  0.90955849  2.1351019 ], Cost: 0.4788
Current parameters: [ 2.39494766  2.7600143   2.85055046  2.92412266  0.29642312  2.81639672
  1.99813976  1.43198091  0.36292278  2.44116283  3.19167365  2.06522435
  2.21289011  1.44616292  1.22156461  1.9264683   0.4672377   2.21271066
 -0.21692446  0.32117615  0.84199617  2.0708991 ], Cost: 0.0961
Current parameters: [ 2.39494766  2.7600143   2.85055046  2.92412266  0.29642312  2.81639672
  1.99813976  1.43198091  0.15378833  2.44116283  3.19167365  2.06522435
  2.21289011  1.58314023  1.22156461  1.9264683   0.4672377   2.21271066
 -0.21692446  0.32117615  0.84199617  2.0708991 ], Cost: 0.1750
Current parameters: [ 2.39586496  2.77683809  2.8707592   2.92646728  0.2784944   2.79309535
  1.99071932  1.43269956  0.63720601  2.43778243  3.20714512  2.0693191
  2.24176356  1.85947327  1.21844382  1.92865456  0.47681152  2.21153947
 -0.24877688  0.31151669  0.84253113  2.07382592], Cost: 1.5157
Current parameters: [ 2.39465098  2.75983154  2.85053704  2.9240237   0.29614913  2.81645549
  2.00658552  1.43198091  0.3626703   2.440567    3.19127739  2.06458484
  2.21237406  1.44577744  1.22071328  1.92586188  0.46650428  1.96286311
 -0.21761982  0.32053309  0.8413899   2.07029253], Cost: 0.3493
Current parameters: [ 2.38022923  2.7861776   2.89358386  2.92363074  0.24214586  2.76921573
  2.45764342  1.43353812  0.33071907  2.40028683  3.2028843   2.03808596
  2.24639512  1.38516337  1.16686404  1.89705812  0.44668371  2.25022363
 -0.32509911  0.26403463  0.80901624  2.04308506], Cost: 1.1001
Current parameters: [ 2.39470132  2.75986255  2.85053932  2.92404049  0.29619561  2.81644552
  1.99781175  1.68196171  0.36283044  2.44094492  3.19152873  2.06499046
  2.21270138  1.44602194  1.22085772  1.92624652  0.46662871  2.21269731
 -0.21482918  0.32311387  0.84177444  2.07067726], Cost: 0.2648
Current parameters: [2.35440234 2.80437232 2.93471852 2.91929243 0.17890949 2.72583587
 1.90981352 1.39937675 0.31082022 2.38732685 3.23143401 2.04037562
 2.30187695 1.34332866 1.08068588 1.89567051 0.39816279 2.25041982
 0.02445652 0.62883127 0.80416632 2.04324938], Cost: 2.2283
Current parameters: [ 2.18561806  2.63106422  2.81902415  2.92976978  0.30015153  2.81559698
  2.01034406  1.43167615  0.36635846  2.44927069  3.19706586  2.07392664
  2.21991244  1.45140846  1.23314919  1.93472024  0.47721784  2.21320734
 -0.2074623   0.32992676  0.85024609  2.0791531 ], Cost: 0.2619
Current parameters: [ 2.44415029  2.81524839  2.36913433  2.99627216  0.26638671  2.78122315
  1.98237033  1.42254293  0.34869207  2.43242749  3.21302826  2.06768788
  2.25407553  1.41606517  1.20885187  1.92620348  0.47508385  2.22691688
 -0.2529608   0.31819946  0.83920081  2.07176866], Cost: 0.7107
Current parameters: [ 2.39399297  2.75942619  2.87466072  2.96821501  0.22807629  2.73533195
  1.97190853  1.43263596  0.35553831  2.42373625  3.18008394  1.98210992
  2.19779671  1.43488845  1.26449802  1.90873204  0.62776415  2.21164313
 -0.30081017  0.37108378  0.82426424  2.12106682], Cost: 0.2150
```



```
Current parameters: [ 2.38180287  2.81376171  2.89425492  2.82949358  0.33068789  2.89819923
  1.90215177  1.40998253  0.3116056   2.38171363  3.21953816  2.16026214
  2.28236569  1.34704309  1.55514176  1.88736553  0.55293623  2.24564624
 -0.22268838  0.13489787  0.79662382  1.89810077], Cost: 2.3690
Current parameters: [ 2.52771118  2.55006792  2.85107385  2.92378422  0.2901487   2.80860995
  1.99526816  1.42853099  0.35860722  2.4398787   3.2005495   2.07197181
  2.22924002  1.43657577  1.21519425  1.928251    0.46103139  2.21791217
 -0.22384629  0.31795622  0.842872    2.06899255], Cost: 0.1988
Current parameters: [ 2.39503235  2.75990073  2.83497516  2.81077678  0.32524995  2.79100135
  1.99843312  1.43197359  0.36300536  2.44135772  3.19180327  2.05250636
  2.21305891  1.44628901  1.20958236  1.92666665  0.48170937  2.2127226
 -0.2294512   0.33517778  0.84219447  2.08472677], Cost: 0.2350
Current parameters: [ 2.39601163  2.76378045  2.87011128  3.00960912  0.51067494  2.73919464
  1.9842779   1.42831105  0.35502095  2.43266493  3.19711785  2.05135355
  2.22544351  1.4306794   1.19095666  1.92134277  0.46693329  2.21821153
 -0.24685516  0.32486637  0.83583821  2.07634221], Cost: 0.3825
Current parameters: [ 2.39439053  2.76076142  2.85187769  2.92407354  0.29484813  2.81588281
  1.99620986  1.4320291   0.36237948  2.43988071  3.19082097  2.13269601
  2.21177965  1.44533343  1.21944319  1.86817918  0.46650931  2.21263212
 -0.22041413  0.31908324  0.78372129  2.13604283], Cost: 0.0594
Current parameters: [ 2.38872201  2.77561509  2.8811531   2.93159858  0.25377035  2.79380622
  1.95206861  1.42752292  0.34436765  2.41154885  3.18747419  2.22470694
  2.21505085  1.41305836  1.16649573  1.98015058  0.44084314  2.21930565
 -0.30211776  0.27035605  0.89422176  2.23210969], Cost: 0.1005
Current parameters: [ 2.39439053  2.76076142  2.85187769  2.92407354  0.30520267  2.8641562
  1.99620986  1.4320291   0.36237948  2.43988071  3.19082097  2.15262376
  2.21177965  1.44533343  1.23834357  1.86817918  0.44457044  2.21263212
 -0.29325764  0.39785005  0.78372129  2.11503276], Cost: 0.0446
Current parameters: [ 2.39950937  2.76031692  2.85364514  2.93162881  0.33980664  3.05122123
  1.99224795  1.42716312  0.35631962  2.43812782  3.20337273  2.08242363
  2.23488019  1.43185431  1.15462755  1.87014782  0.52267373  2.21996892
 -0.28211049  0.37118771  0.78441153  2.19466143], Cost: 0.4813
Current parameters: [ 2.39439053  2.76076142  2.85187769  2.92407354  0.30520267  2.8641562
  1.99620986  1.4320291   0.36237948  2.4168191   3.19082097  2.09426251
  2.23840617  1.44533343  1.29369601  1.86817918  0.38031935  2.21263212
 -0.29325764  0.39785005  0.78372129  2.17656373], Cost: 0.1899
Current parameters: [ 2.40200963  2.7600998   2.85450843  2.93531913  0.2962165   2.86057414
  1.99031278  1.42478637  0.35335973  2.61157742  3.20950353  2.13063807
  2.0449131   1.42527055  1.25195251  1.87110939  0.4003873   2.22355253
 -0.30600833  0.38989327  0.78474867  2.14657138], Cost: 0.4704
Current parameters: [ 2.39336378  2.76009692  2.85179155  2.92372705  0.30429139  2.86440511
```



```
  1.99385152  1.43202769  0.36239543  2.43743968  3.17932934  2.15040635
  2.20960284  1.44535778  1.2354086   1.95390748  0.44201783  2.21263443
 -0.2955972   0.39564745  0.69377666  2.11293147], Cost: 0.1755
Current parameters: [ 2.37909951  2.74843982  2.8514584   2.92267543  0.28480148  2.86711061
  1.95094089  1.42886238  0.35875654  2.40331169  2.99080072  2.11751726
  2.17714032  1.43707335  1.17725563  1.80791952  0.39523844  2.21740995
 -0.34115882  0.35450846  0.76973065  2.07527477], Cost: 0.9190
Current parameters: [ 2.39806588  2.75521334  2.84235381  2.92431991  0.31761858  2.87453689
  2.00930485  1.4316838   0.35912443  2.35922987  3.19684632  2.1595288
  2.12664664  1.44036367  1.25165428  1.87554207  0.45014569  2.21319487
 -0.26830562  0.41141311  0.79187556  2.12204193], Cost: 0.1638
Current parameters: [ 2.40883847  2.76846069  2.86139346  2.9396123   0.29418596  2.85176991
  1.99943133  1.42385321  0.49830469  2.43779483  3.22777453  2.17888525
  2.2148689   1.64556695  1.2314733   1.87546964  0.45240704  2.22493253
 -0.30535504  0.3972066   0.8036532   2.12228572], Cost: 0.5620
Current parameters: [ 2.39555455  2.75956364  2.8478902   2.91059395  0.32871452  2.89042353
  1.99360571  1.43321902  0.3633545   2.43952044  3.18866225  2.17024409
  2.2077223   1.44682207  1.31195034  1.86714983  0.47025653  2.21252801
 -0.33245437  0.32182833  0.78288661  2.09397205], Cost: 0.2654
Current parameters: [ 2.3978985   2.79385519  2.91181006  2.92516183  0.25527857  2.84514102
  1.92882798  1.40996769  0.32101513  2.43902605  3.28227962  2.22876727
  2.28911596  1.36448459  1.27407138  1.85420931  0.45349799  2.24068923
 -0.193242    0.47381032  0.79698714  2.05811968], Cost: 0.3507
Current parameters: [ 2.38136869  2.75941251  2.85956544  2.92056676  0.2854708   2.85724723
  1.97596043  1.43232405  0.35851553  2.41158519  3.26286445  2.12542331
  2.20032405  1.43943402  1.19861611  1.84169009  0.41537777  2.21266814
 -0.337733    0.3648444   0.75656119  2.08884076], Cost: 0.0536
Current parameters: [ 2.40024758  2.77855313  2.87994931  2.93656401  0.2714925   2.83538188
  2.22689534  1.42387205  0.3411319   2.43873049  3.20958885  2.16972464
  2.24586673  1.40550175  1.21225134  1.86197339  0.4416387   2.23358972
 -0.32206851  0.3944445   0.78876417  2.10618511], Cost: 0.1404
Current parameters: [ 2.40975908  2.79629082  2.76061228  2.96390447  0.27964085  2.87867528
  1.99555699  1.41231989  0.37698579  2.44012634  3.19473747  2.14258111
  2.21872813  1.43166333  1.23900312  1.88759032  0.42865356  2.23234584
 -0.28644773  0.38864928  0.80277804  2.08537882], Cost: 0.0899
Current parameters: [ 2.39730146  2.70938004  2.70893886  2.887097    0.33790735  2.78496673
  1.99289717  1.4777099   0.30162947  2.44549589  3.23083754  2.20986844
  2.24476149  1.44513103  1.21627165  1.81600386  0.49656425  2.17145312
 -0.32808991  0.43987028  0.74465222  2.19756962], Cost: 0.1425
Current parameters: [ 2.39746811  2.77497914  2.86438951  2.9784997   0.40505516  2.8305141
  1.9915086   1.42211653  0.36413396  2.43886418  3.19973843  2.14546739
```

```
 2.22097666  1.43153454  1.22202815  1.87394935  0.43868784  2.22335998
-0.30086689  0.40047652  0.79177553  2.10590742], Cost: 0.2132
Current parameters: [ 2.35676841  2.7377188   2.84623161  2.92530007  0.30589572  2.8640972
 1.99847245  1.38851571  0.36312099  2.44138661  3.19181798  2.15415969
 2.21307586  1.44624518  1.24055726  1.8698205   0.44636762  2.21284501
-0.29184669  0.39903964  0.78536264  2.11637829], Cost: 0.0478
Current parameters: [ 2.32110548  2.72960513  2.84407399  2.9481696   0.30241918  2.8730536
 2.00232505  1.51556844  0.37446305  2.44519273  3.19651693  2.15119188
 2.21612682  1.43946301  1.2418966   1.88383034  0.43687715  2.2272924
-0.27977622  0.39856376  0.80100867  2.09878309], Cost: 0.0113
Current parameters: [ 2.32333929  2.72650873  2.8375198   2.93049061  0.32004931  2.89020803
 2.00891632  1.51536435  0.37266034  2.44913346  3.19928892  2.17423507
 2.21981124  1.44411257  1.23078349  1.88419144  0.4000896   2.20959091
-0.26136131  0.38757201  0.8013556   2.09210521], Cost: 0.0228
Current parameters: [ 2.31392914  2.73926876  2.85064148  2.97883163  0.28574519  2.86867468
 2.00547692  1.517285    0.38398589  2.44599096  3.20105955  2.13532594
 2.21697663  1.42870303  1.24788686  1.89414373  0.4518104   2.11331012
-0.28261185  0.40504159  0.81408112  2.0874492 ], Cost: 0.0630
Current parameters: [ 2.29717265  2.76844597  2.84773311  2.93987785  0.29885409  2.86845045
 1.99520283  1.51540053  0.36819376  2.44052236  3.19436618  2.16926156
 2.21379626  1.43814912  1.23282475  1.89856226  0.43476155  2.22752474
-0.29378596  0.39187456  0.81564799  2.12113179], Cost: 0.0088
Current parameters: [ 2.33800939  2.70559746  2.86065573  2.95734172  0.27710237  2.86040191
 1.97904583  1.51317104  0.36659285  2.43090473  3.19834281  2.19746464
 2.22157685  1.41811269  1.2129163   1.94900429  0.42234674  2.23161948
-0.32278639  0.37319854  0.86622705  2.14357117], Cost: 0.0269
Current parameters: [ 2.29736199  2.76847042  2.8494204   2.9532752   0.29581699  2.87158537
 1.99575432  1.51541194  0.31900056  2.44110851  3.19609583  2.16306382
 2.21404133  1.47053984  1.23493283  1.9058598   0.43356063  2.22750808
-0.29151735  0.39080019  0.82262375  2.11221655], Cost: 0.0322
Current parameters: [ 2.28370416  2.75989698  2.85042353  2.94005623  0.26840419  2.85089383
 1.97811889  1.52213667  0.35852092  2.4206319   3.10273377  2.17401025
 2.21992877  1.42504682  1.19464006  1.85493219  0.4142591   2.2286327
-0.30772708  0.39175476  0.81017661  2.09533414], Cost: 0.1649
Current parameters: [ 2.29836793  2.76957723  2.84862001  2.94042996  0.29091949  2.83125242
 1.99659244  1.51511709  0.38940184  2.43875856  3.19733778  2.18530273
 2.20928759  1.47025902  1.24871817  1.90086722  0.42015558  2.22794872
-0.29734267  0.39700608  0.81732277  2.10808744], Cost: 0.0425
Current parameters: [ 2.2981686   2.76753332  2.85488512  2.9460611   0.28542948  2.92142603
 1.99838817  1.51894212  0.41633574  2.43981236  3.21224678  2.16785289
 2.23843584  1.50209297  1.18261983  1.88671281  0.47000017  2.23537138
```



```
-0.28541379  0.40246269  0.82508056  2.15200641], Cost: 0.0459
Current parameters: [ 2.29782333  2.7637078   2.84011383  2.94426414  0.30235895  2.86907104
 2.00396786  1.51734289  0.37280255  2.47448433  3.18439814  2.15541221
 2.17541543  1.44670962  1.22362945  1.89681547  0.42516179  2.22526096
-0.30923747  0.38363461  0.81620127  2.13558122], Cost: 0.0489
Current parameters: [ 2.29529626  2.77735825  2.87665355  2.9349763   0.25629508  2.87299881
 1.96876444  1.51326663  0.35439704  2.49612576  3.24265295  2.1974271
 2.20546463  1.39938907  1.23886199  1.88339548  0.43704257  2.24594685
-0.24950514  0.43111471  0.82277974  2.1189227 ], Cost: 0.0879
Current parameters: [ 2.29670982  2.75681841  2.81794475  2.93160612  0.31033407  2.86046782
 1.9528156   1.52442796  0.36192789  2.44161063  3.19771952  2.17566322
 2.21317538  1.44524116  1.23396592  1.8911284   0.44391927  2.21693493
-0.29440414  0.3994202   0.80826391  2.13641622], Cost: 0.0187
Current parameters: [ 2.2982678   2.75493414  2.80613034  2.92456157  0.29178739  2.83693243
 2.07366439  1.53234526  0.34068198  2.44029932  3.21953439  2.19653557
 2.24060921  1.42253193  1.21780142  1.87774571  0.45757646  2.22230501
-0.31320793  0.4069189   0.80197633  2.15104575], Cost: 0.0194
Current parameters: [ 2.31105853  2.77343524  2.84990579  2.89774032  0.30326902  2.86236416
 1.99603246  1.52296552  0.36676688  2.44914827  3.18913899  2.1503864
 2.21755083  1.44094878  1.22435149  1.90941461  0.44373393  2.22457612
-0.29844984  0.40853747  0.83785989  2.10563077], Cost: 0.0240
Current parameters: [ 2.22426633  2.74035639  2.81429985  2.93092895  0.30517606  2.8601382
 2.00000121  1.48632607  0.37986309  2.41638397  3.23334545  2.12695065
 2.21704562  1.42175963  1.22410733  1.93286799  0.43572777  2.24775789
-0.27270009  0.3668137   0.82460847  2.10624477], Cost: 0.1055
Current parameters: [ 2.29765071  2.76775352  2.84959019  2.92767143  0.30147445  2.88699494
 1.99551194  1.52033673  0.36986574  2.44742094  3.19664754  2.18504476
 2.21925395  1.43773008  1.26320419  1.88946586  0.45709528  2.22914596
-0.31160186  0.35910664  0.81993491  2.1098797 ], Cost: 0.0653
Current parameters: [ 2.29705566  2.77040368  2.84917957  2.9485471   0.31420355  2.86320727
 1.99451703  1.51352765  0.36853614  2.44423122  3.19597601  2.17921416
 2.20959284  1.43589981  1.21958126  1.90011446  0.44612307  2.22931543
-0.29464717  0.3919724   0.81642869  2.10776409], Cost: 0.0090
Current parameters: [ 2.30491582  2.77759734  2.86112541  2.95585779  0.33518087  2.8611682
 1.99486431  1.51928624  0.36980726  2.4418236   3.20007111  2.15384932
 2.23870229  1.43120501  1.24219636  1.88865707  0.42015626  2.23344033
-0.29820964  0.40011746  0.82607709  2.14027635], Cost: 0.0889
Current parameters: [ 2.2958403   2.76916905  2.84923477  2.93991594  0.29628128  2.8665008
 1.99236972  1.51522677  0.3688552   2.45501101  3.19150706  2.16749704
 2.22920637  1.43902616  1.22976867  1.91162108  0.4332493   2.22749749
-0.29868138  0.3887431   0.79907541  2.11938193], Cost: 0.0069
```



```
Current parameters: [ 2.30858268  2.77247777  2.85710343  2.9339467   0.3015058   2.87685139
 1.99817325  1.52533712  0.36788907  2.43017788  3.18912257  2.17493708
 2.20751069  1.43571738  1.23412407  1.94112361  0.44140902  2.22882343
-0.29257096  0.40338063  0.76845729  2.12699611], Cost: 0.0363
Current parameters: [ 2.29126665  2.7689897   2.84126552  2.94377839  0.2960878   2.8787617
 1.992192    1.51141676  0.3703169   2.4546144   3.1938339   2.17068538
 2.22972354  1.43771604  1.2334727   1.91309323  0.42620979  2.22948133
-0.31540712  0.40533018  0.8005426   2.11257784], Cost: 0.0150
Current parameters: [ 2.32304002  2.79587734  2.81156208  2.95485364  0.28502449  2.86933278
 1.99337974  1.51545861  0.37489336  2.45558655  3.1876356   2.16378573
 2.23329738  1.4325463   1.23000812  1.92133094  0.43311263  2.23603795
-0.28430424  0.37988318  0.806526    2.11285267], Cost: 0.0128
Current parameters: [ 2.2998819   2.77353622  2.85201638  2.93968919  0.291787    2.8653419
 1.98768065  1.52898216  0.36825913  2.44875909  3.20688561  2.1612401
 2.22658016  1.43733393  1.22083175  1.90607821  0.42628524  2.2279568
-0.30809398  0.38140273  0.79344513  2.11287837], Cost: 0.0029
Current parameters: [ 2.31982868  2.7809763   2.85506863  2.92842122  0.28933905  2.86123343
 1.98295388  1.47870189  0.36289001  2.44138016  3.21755003  2.15841306
 2.22384959  1.43831475  1.2129332   1.89586378  0.4272138   2.22242346
-0.3193239   0.37634411  0.78076377  2.11726954], Cost: 0.0087
Current parameters: [ 2.30481623  2.77859711  2.85674666  2.94758003  0.28746837  2.86601121
 1.98836332  1.53192335  0.36975148  2.45068041  3.20554891  2.16172867
 2.22742877  1.43558324  1.22303673  1.90527183  0.43053399  2.20048499
-0.31016102  0.38535099  0.79531941  2.11226337], Cost: 0.0131
Current parameters: [ 2.26555525  2.76256057  2.8368215   2.93989076  0.28800957  2.86016919
 1.99068764  1.51566158  0.37534821  2.43603161  3.22657328  2.13887632
 2.22603484  1.42764694  1.21465497  1.92606871  0.43074836  2.21665426
-0.29570372  0.37034155  0.7969493   2.10386024], Cost: 0.0147
Current parameters: [ 2.2901223   2.78840478  2.84944541  2.93600469  0.29577089  2.87052601
 1.99167421  1.52981024  0.37175535  2.45085653  3.20652307  2.15460798
 2.22645199  1.44704506  1.22194741  1.89292982  0.43128252  2.22748306
-0.30064149  0.38661587  0.78203081  2.10923341], Cost: 0.0029
Current parameters: [ 2.2998819   2.77353622  2.85201638  2.93968919  0.291787    2.8653419
 1.98768065  1.52898216  0.36825913  2.44875909  3.20688561  2.1612401
 2.22658016  1.43733393  1.22083175  1.90607821  0.42628524  2.2279568
-0.30809398  0.38140273  0.79344513  2.11287837], Cost: 0.0029

   Return from subroutine COBYLA because the MAXFUN limit has been reached.

   NFVALS =  100   F = 2.933120E-03    MAXCV = 0.000000E+00
   X = 2.299882E+00   2.773536E+00   2.852016E+00   2.939689E+00   2.917870E-01
```



```
       2.865342E+00   1.987681E+00   1.528982E+00   3.682591E-01   2.448759E+00
       3.206886E+00   2.161240E+00   2.226580E+00   1.437334E+00   1.220832E+00
       1.906078E+00   4.262852E-01   2.227957E+00  -3.080940E-01   3.814027E-01
       7.934451E-01   2.112878E+00
Simulating for QAOA depth p = 12
Current parameters: [3.06830733 0.28260508 0.41935626 3.04511251 1.20597356 0.32784605
 0.05713576 1.28176726 2.67997777 2.4934801  1.62336167 1.15129591
 2.48932653 3.13738673 0.15506462 0.47981424 1.59003128 0.38744703
 2.77720598 0.52190428 1.38415951 1.9132206  1.11659809 3.04731346], Cost: 1.7891
Current parameters: [3.06830733 1.28260508 0.41935626 3.04511251 1.20597356 0.32784605
 0.05713576 1.28176726 2.67997777 2.4934801  1.62336167 1.15129591
 2.48932653 3.13738673 0.15506462 0.47981424 1.59003128 0.38744703
 2.77720598 0.52190428 1.38415951 1.9132206  1.11659809 3.04731346], Cost: 1.9326
Current parameters: [3.06830733 0.28260508 1.41935626 3.04511251 1.20597356 0.32784605
 0.05713576 1.28176726 2.67997777 2.4934801  1.62336167 1.15129591
 2.48932653 3.13738673 0.15506462 0.47981424 1.59003128 0.38744703
 2.77720598 0.52190428 1.38415951 1.9132206  1.11659809 3.04731346], Cost: 1.9470
Current parameters: [3.06830733 0.28260508 0.41935626 4.04511251 1.20597356 0.32784605
 0.05713576 1.28176726 2.67997777 2.4934801  1.62336167 1.15129591
 2.48932653 3.13738673 0.15506462 0.47981424 1.59003128 0.38744703
 2.77720598 0.52190428 1.38415951 1.9132206  1.11659809 3.04731346], Cost: 1.3712
Current parameters: [3.06830733 0.28260508 0.41935626 4.04511251 2.20597356 0.32784605
 0.05713576 1.28176726 2.67997777 2.4934801  1.62336167 1.15129591
 2.48932653 3.13738673 0.15506462 0.47981424 1.59003128 0.38744703
 2.77720598 0.52190428 1.38415951 1.9132206  1.11659809 3.04731346], Cost: 2.4269
Current parameters: [3.06830733 0.28260508 0.41935626 4.04511251 1.20597356 1.32784605
 0.05713576 1.28176726 2.67997777 2.4934801  1.62336167 1.15129591
 2.48932653 3.13738673 0.15506462 0.47981424 1.59003128 0.38744703
 2.77720598 0.52190428 1.38415951 1.9132206  1.11659809 3.04731346], Cost: 2.4028
Current parameters: [3.06830733 0.28260508 0.41935626 4.04511251 1.20597356 0.32784605
 1.05713576 1.28176726 2.67997777 2.4934801  1.62336167 1.15129591
 2.48932653 3.13738673 0.15506462 0.47981424 1.59003128 0.38744703
 2.77720598 0.52190428 1.38415951 1.9132206  1.11659809 3.04731346], Cost: 1.7338
Current parameters: [3.06830733 0.28260508 0.41935626 4.04511251 1.20597356 0.32784605
 0.05713576 2.28176726 2.67997777 2.4934801  1.62336167 1.15129591
 2.48932653 3.13738673 0.15506462 0.47981424 1.59003128 0.38744703
 2.77720598 0.52190428 1.38415951 1.9132206  1.11659809 3.04731346], Cost: 2.3664
Current parameters: [3.06830733 0.28260508 0.41935626 4.04511251 1.20597356 0.32784605
 0.05713576 1.28176726 3.67997777 2.4934801  1.62336167 1.15129591
 2.48932653 3.13738673 0.15506462 0.47981424 1.59003128 0.38744703
 2.77720598 0.52190428 1.38415951 1.9132206  1.11659809 3.04731346], Cost: 1.6869
```

```
Current parameters: [3.06830733 0.28260508 0.41935626 4.04511251 1.20597356 0.32784605
 0.05713576 1.28176726 2.67997777 3.4934801  1.62336167 1.15129591
 2.48932653 3.13738673 0.15506462 0.47981424 1.59003128 0.38744703
 2.77720598 0.52190428 1.38415951 1.9132206  1.11659809 3.04731346], Cost: 1.4475
Current parameters: [3.06830733 0.28260508 0.41935626 4.04511251 1.20597356 0.32784605
 0.05713576 1.28176726 2.67997777 2.4934801  2.62336167 1.15129591
 2.48932653 3.13738673 0.15506462 0.47981424 1.59003128 0.38744703
 2.77720598 0.52190428 1.38415951 1.9132206  1.11659809 3.04731346], Cost: 2.1670
Current parameters: [3.06830733 0.28260508 0.41935626 4.04511251 1.20597356 0.32784605
 0.05713576 1.28176726 2.67997777 2.4934801  1.62336167 2.15129591
 2.48932653 3.13738673 0.15506462 0.47981424 1.59003128 0.38744703
 2.77720598 0.52190428 1.38415951 1.9132206  1.11659809 3.04731346], Cost: 1.1006
Current parameters: [3.06830733 0.28260508 0.41935626 4.04511251 1.20597356 0.32784605
 0.05713576 1.28176726 2.67997777 2.4934801  1.62336167 2.15129591
 3.48932653 3.13738673 0.15506462 0.47981424 1.59003128 0.38744703
 2.77720598 0.52190428 1.38415951 1.9132206  1.11659809 3.04731346], Cost: 1.2430
Current parameters: [3.06830733 0.28260508 0.41935626 4.04511251 1.20597356 0.32784605
 0.05713576 1.28176726 2.67997777 2.4934801  1.62336167 2.15129591
 2.48932653 4.13738673 0.15506462 0.47981424 1.59003128 0.38744703
 2.77720598 0.52190428 1.38415951 1.9132206  1.11659809 3.04731346], Cost: 2.1731
Current parameters: [3.06830733 0.28260508 0.41935626 4.04511251 1.20597356 0.32784605
 0.05713576 1.28176726 2.67997777 2.4934801  1.62336167 2.15129591
 2.48932653 3.13738673 1.15506462 0.47981424 1.59003128 0.38744703
 2.77720598 0.52190428 1.38415951 1.9132206  1.11659809 3.04731346], Cost: 2.6441
Current parameters: [3.06830733 0.28260508 0.41935626 4.04511251 1.20597356 0.32784605
 0.05713576 1.28176726 2.67997777 2.4934801  1.62336167 2.15129591
 2.48932653 3.13738673 0.15506462 1.47981424 1.59003128 0.38744703
 2.77720598 0.52190428 1.38415951 1.9132206  1.11659809 3.04731346], Cost: 1.4519
Current parameters: [3.06830733 0.28260508 0.41935626 4.04511251 1.20597356 0.32784605
 0.05713576 1.28176726 2.67997777 2.4934801  1.62336167 2.15129591
 2.48932653 3.13738673 0.15506462 0.47981424 2.59003128 0.38744703
 2.77720598 0.52190428 1.38415951 1.9132206  1.11659809 3.04731346], Cost: 3.6320
Current parameters: [3.06830733 0.28260508 0.41935626 4.04511251 1.20597356 0.32784605
 0.05713576 1.28176726 2.67997777 2.4934801  1.62336167 2.15129591
 2.48932653 3.13738673 0.15506462 0.47981424 1.59003128 1.38744703
 2.77720598 0.52190428 1.38415951 1.9132206  1.11659809 3.04731346], Cost: 3.1149
Current parameters: [3.06830733 0.28260508 0.41935626 4.04511251 1.20597356 0.32784605
 0.05713576 1.28176726 2.67997777 2.4934801  1.62336167 2.15129591
 2.48932653 3.13738673 0.15506462 0.47981424 1.59003128 0.38744703
 3.77720598 0.52190428 1.38415951 1.9132206  1.11659809 3.04731346], Cost: 3.2257
Current parameters: [3.06830733 0.28260508 0.41935626 4.04511251 1.20597356 0.32784605
```



```
 0.05713576 1.28176726 2.67997777 2.4934801  1.62336167 2.15129591
 2.48932653 3.13738673 0.15506462 0.47981424 1.59003128 0.38744703
 2.77720598 1.52190428 1.38415951 1.9132206  1.11659809 3.04731346], Cost: 1.7450
Current parameters: [3.06830733 0.28260508 0.41935626 4.04511251 1.20597356 0.32784605
 0.05713576 1.28176726 2.67997777 2.4934801  1.62336167 2.15129591
 2.48932653 3.13738673 0.15506462 0.47981424 1.59003128 0.38744703
 2.77720598 0.52190428 2.38415951 1.9132206  1.11659809 3.04731346], Cost: 1.6350
Current parameters: [3.06830733 0.28260508 0.41935626 4.04511251 1.20597356 0.32784605
 0.05713576 1.28176726 2.67997777 2.4934801  1.62336167 2.15129591
 2.48932653 3.13738673 0.15506462 0.47981424 1.59003128 0.38744703
 2.77720598 0.52190428 1.38415951 2.9132206  1.11659809 3.04731346], Cost: 2.2756
Current parameters: [3.06830733 0.28260508 0.41935626 4.04511251 1.20597356 0.32784605
 0.05713576 1.28176726 2.67997777 2.4934801  1.62336167 2.15129591
 2.48932653 3.13738673 0.15506462 0.47981424 1.59003128 0.38744703
 2.77720598 0.52190428 1.38415951 1.9132206  2.11659809 3.04731346], Cost: 1.1500
Current parameters: [3.06830733 0.28260508 0.41935626 4.04511251 1.20597356 0.32784605
 0.05713576 1.28176726 2.67997777 2.4934801  1.62336167 2.15129591
 2.48932653 3.13738673 0.15506462 0.47981424 1.59003128 0.38744703
 2.77720598 0.52190428 1.38415951 1.9132206  1.11659809 4.04731346], Cost: 0.6551
Current parameters: [ 3.27355146  0.25467081  0.38861307  4.12646936  1.00044058  0.12699801
 -0.01345297  1.08801251  2.6185025   2.47862155  1.46842772  2.20397136
  2.46159888  2.92859988 -0.14541779  0.41142972  1.0972163  -0.00469838
  2.36348264  0.39644083  1.28012004  1.68447064  1.10698691  4.13403768], Cost: 3.2465
Current parameters: [3.09043386 0.2795936  0.41604196 3.87960736 1.3580918  0.48046934
 0.22380179 1.43515526 2.84762628 2.66615417 1.78093478 2.33125054
 2.48633732 3.11487827 0.12267083 0.47244198 1.53690289 0.34517142
 2.73260414 0.50837857 1.37294343 1.88856    1.11556194 4.05666285], Cost: 1.1830
Current parameters: [3.0345925  0.03488889 0.41935626 4.04511251 1.20597356 0.32784605
 0.05713576 1.28176726 2.67997777 2.4934801  1.62336167 2.15129591
 2.48932653 3.13738673 0.15506462 0.47981424 1.59003128 0.38744703
 2.77720598 0.52190428 1.38415951 1.9132206  1.11659809 4.04731346], Cost: 0.5773
Current parameters: [2.54326548 0.0972577  0.41709398 4.04575954 1.19618884 0.31840607
 0.05728113 1.27284925 2.68079376 2.49772646 1.61730035 2.16051186
 2.48728615 3.12202281 0.13295313 0.47478205 1.55376668 0.35859037
 2.74676147 0.51267184 1.37650359 1.89638766 1.11589083 4.05369521], Cost: 0.6806
Current parameters: [3.0345925  0.03488889 0.17885524 4.05315689 1.27032854 0.31980167
 0.04909139 1.27372289 2.6719334  2.48543573 1.6153173  2.14325153
 2.48932653 3.13738673 0.15506462 0.47981424 1.59003128 0.38744703
 2.77720598 0.52190428 1.38415951 1.9132206  1.11659809 4.04731346], Cost: 0.7243
Current parameters: [3.03281359e+00 3.26314579e-02 5.59973296e-01 4.10236365e+00
 1.65567643e+00 2.65713092e-01 3.24556686e-04 1.21992410e+00
```



```
 2.62353889e+00 2.43894575e+00 1.56310454e+00 2.09952066e+00
 2.48819372e+00 3.12885674e+00 1.42788410e-01 4.77020391e-01
 1.56989732e+00 3.71425924e-01 2.76030331e+00 5.16778465e-01
 1.37990897e+00 1.90387502e+00 1.11620542e+00 4.05085658e+00], Cost: 0.6294
Current parameters: [3.0345925  0.03488889 0.41935626 4.27813741 1.20597356 0.34166737
 0.09366969 1.31830119 2.7165117  2.53001403 1.6598956  2.18782983
 2.48932653 3.13738673 0.15506462 0.47981424 1.59003128 0.38744703
 2.77720598 0.52190428 1.38415951 1.9132206  1.11659809 4.04731346], Cost: 0.6571
Current parameters: [ 3.03270626  0.03249525  0.42436752  4.08309095  1.20416168  0.79179157
 -0.01542093  1.20387501  2.60781587  2.42333765  1.54715114  2.08407903
  2.48812537  3.12834207  0.1420477   0.47685182  1.56868249  0.37045925
  2.75928344  0.51646919  1.37965251  1.90331113  1.11618173  4.05107036], Cost: 0.5400
Current parameters: [ 3.03270626  0.03249525  0.42436752  4.08309095  1.20416168  0.79179157
 -0.00418144  1.35601574  2.61905536  2.43457713  1.35005245  2.09531852
  2.48812537  3.12834207  0.1420477   0.47685182  1.56868249  0.37045925
  2.75928344  0.51646919  1.37965251  1.90331113  1.11618173  4.05107036], Cost: 0.5147
Current parameters: [ 3.02982063  0.0288334   0.43203386  4.08150086  1.20138982  0.79312595
 -0.15185431  1.69142071  2.47198646  2.29059762  1.57432315  1.95581463
  2.4862878   3.11450532  0.12213409  0.47231983  1.53602259  0.34447094
  2.73186512  0.50815446  1.37275759  1.88815138  1.11554478  4.05681776], Cost: 0.8098
Current parameters: [3.03270626 0.03249525 0.42436752 4.08309095 1.20416168 0.79179157
 0.16263247 1.35601574 2.433329   2.44403336 1.35005245 2.10477474
 2.48812537 3.12834207 0.1420477  0.47685182 1.56868249 0.37045925
 2.75928344 0.51646919 1.37965251 1.90331113 1.11618173 4.05107036], Cost: 0.5669
Current parameters: [3.0313054  0.03071756 0.42808923 4.08231902 1.20281605 0.79243936
 0.2694864  1.3538022  2.84116742 2.18357669 1.34730917 1.84649082
 2.4872333  3.12162486 0.13238041 0.47465171 1.55282738 0.35784294
 2.74597291 0.51243271 1.37630529 1.89595166 1.11587251 4.0538605 ], Cost: 0.3181
Current parameters: [3.0313054  0.03071756 0.42808923 4.08231902 1.20281605 0.79243936
 0.2694864  1.3538022  2.84116742 2.35952926 1.34730917 1.67053826
 2.4872333  3.12162486 0.13238041 0.47465171 1.52871532 0.35784294
 2.74597291 0.51243271 1.37630529 1.89595166 1.11587251 4.0538605 ], Cost: 0.6643
Current parameters: [3.02186666 0.01873982 0.45316546 4.07711791 1.19374941 0.79680408
 0.26964701 1.33888773 2.85126192 2.15555026 1.32882545 1.83698572
 2.48122269 3.07636551 0.06724393 0.4598278  2.02355783 0.27283639
 2.65628886 0.48523562 1.35375234 1.84636484 1.11378907 4.07265997], Cost: 0.8206
Current parameters: [3.0313054  0.03071756 0.42808923 4.08231902 1.20281605 0.79243936
 0.2694864  1.3538022  2.84116742 2.18357669 1.34730917 1.84649082
 2.4872333  3.12162486 0.13238041 0.47465171 1.55282738 0.17639791
 2.9179546  0.51243271 1.37630529 1.89595166 1.11587251 4.0538605 ], Cost: 0.4341
Current parameters: [3.01629176 0.01166528 0.4679765  4.07404592 1.18839429 0.79938206
```



```
 0.26974187 1.33007864 2.85722415 2.08779566 1.31790821 1.88257268
 2.47767258 3.04963349 0.02877168 0.45107219 1.55433279 0.69381459
 3.05517509 0.46917191 1.34043166 1.81707686 1.1125585  4.0837637 ], Cost: 3.1482
Current parameters: [3.0313054  0.03071756 0.42808923 4.08231902 1.20281605 0.79243936
 0.2694864  1.3538022  2.84116742 2.18357669 1.34730917 1.84649082
 2.4872333  3.12162486 0.28381203 0.47465171 1.55282738 0.35784294
 2.74597291 0.51243271 1.37630529 1.69703343 1.11587251 4.0538605 ], Cost: 0.6571
Current parameters: [3.02326093 0.02050914 0.44946126 4.0778862  1.19508871 0.79615933
 0.26962328 1.34109086 2.84977079 2.13225617 1.33155582 1.86582387
 2.48211056 3.0830511  0.48152331 0.46201755 1.55363399 0.28916352
 2.6492655  0.4892531  1.35708381 2.2230277  1.11409683 4.06988297], Cost: 0.6364
Current parameters: [3.0313054  0.03071756 0.42808923 4.08231902 1.20281605 0.79243936
 0.2694864  1.3538022  2.84116742 2.18357669 1.34730917 1.84649082
 2.4872333  3.25039317 0.13238041 0.47465171 1.55282738 0.35784294
 2.74597291 0.29814616 1.37630529 1.89595166 1.11587251 4.0538605 ], Cost: 0.1892
Current parameters: [3.02566617 0.0235614  0.44307116 4.07921159 1.19739914 0.79504709
 0.26958236 1.34489147 2.84719844 2.14760068 1.33626597 1.86004341
 2.48364223 3.67636915 0.10989958 0.46579508 1.55339282 0.30969823
 2.6781804  0.53895211 1.36283091 1.92179665 1.11462775 4.06509235], Cost: 0.1113
Current parameters: [3.02566617 0.0235614  0.44307116 4.07921159 1.19739914 0.79504709
 0.26958236 1.34489147 2.84719844 2.14760068 1.33626597 1.86004341
 2.48364223 3.67636915 0.10989958 0.2568832  1.55339282 0.30969823
 2.6781804  0.53895211 1.50014743 1.92179665 1.11462775 4.06509235], Cost: 0.8897
Current parameters: [3.02261779 0.019693   0.45116991 4.07753181 1.19447092 0.79645674
 0.26963423 1.34007461 2.85045861 2.12815319 1.33029637 1.86736951
 2.48170101 3.68592592 0.09774715 0.77318545 1.55369848 0.28367273
 2.64153393 0.53649451 1.75322158 1.93576762 1.11395487 4.07116393], Cost: 1.2063
Current parameters: [3.02843616 0.02318439 0.44265625 4.08030959 1.19462525 0.79233643
 0.26862969 1.34227654 2.84636876 2.14740015 1.33417497 1.86075432
 2.23400372 3.67355135 0.10584424 0.46487216 1.54674175 0.30440581
 2.67259676 0.53725885 1.36142678 1.91870942 1.11462775 4.06509235], Cost: 0.1491
Current parameters: [ 3.09270823e+00  1.95924970e-03  4.55291174e-01  4.10441312e+00
  1.11283995e+00  7.24857445e-01  2.43621855e-01  1.25918743e+00
  2.83395515e+00  2.08545233e+00  1.26156416e+00  1.90087017e+00
  2.51415041e+00  3.62697603e+00 -3.66533560e-02  4.94530496e-01
  1.37198514e+00  8.88247662e-02  2.41838531e+00  4.85382522e-01
  1.18145607e+00  1.87787783e+00  1.11266760e+00  4.08277932e+00], Cost: 2.2107
Current parameters: [3.02617887 0.02349162 0.44299436 4.07537668 1.20092386 0.79858351
 0.27344417 1.34844561 2.85108301 2.1516017  1.33991709 1.86421313
 2.48357296 3.6758476  0.10914898 0.46562426 1.55216178 0.30871866
 2.67714693 0.53863871 1.36257102 1.92122524 0.86489624 4.06509235], Cost: 1.0612
```

```
Current parameters: [3.02885769 0.01904616 0.44987409 3.91256313 1.35596929 0.95788924
 0.43499965 1.50201205 3.01594309 2.29609536 1.49344128 2.03605216
 2.48030069 3.67927322 0.08939702 0.48143544 1.53904723 0.27325581
 2.63097808 0.53288434 1.31300798 1.92831339 1.1866658  4.07087699], Cost: 1.4292
Current parameters: [ 3.24663786 -0.00651343  0.44408986  4.07892024  1.20180516  0.79929787
  0.2695169   1.34890721  2.846831    2.14568856  1.33899536  1.8558935
  2.484561    3.68328746  0.11985629  0.46806105  1.56972262  0.32269227
  2.69188944  0.54310944  1.36627834  1.92937645  1.11494622  3.95636944], Cost: 0.6630
Current parameters: [2.79896974 0.05263347 0.44656737 4.0831414  1.19206534 0.79149855
 0.26493082 1.33872331 2.84384395 2.1346069  1.32945787 1.85790882
 2.48265729 3.68120746 0.10608537 0.47379382 1.55555593 0.30049131
 2.66464416 0.53844804 1.34285479 1.9285538  1.135064   3.62218607], Cost: 1.2668
Current parameters: [3.02637607 0.02346478 0.33148987 4.05250468 0.98782488 0.82415249
 0.29607312 1.37385449 2.87350627 2.17297279 1.36444976 1.88405994
 2.48419879 3.68056005 0.11593105 0.46716774 1.56328491 0.31756962
 2.68648491 0.5414705  1.36491925 1.92638826 1.11482067 4.06335157], Cost: 0.1029
Current parameters: [ 3.01671328  0.02267692 -0.11263106  4.08275388  1.19282612  0.79811829
  0.26463287  1.34450514  2.84330963  2.12993111  1.33312196  1.85170627
  2.48391004  3.69284878  0.12089737  0.47876248  1.58136577  0.3190597
  2.68354545  0.54482879  1.34462177  1.94157061  1.13924218  4.08579384], Cost: 0.5331
Current parameters: [3.02660825 0.02343318 0.33073044 4.28365564 0.98809946 0.8390379
 0.33628995 1.41121794 2.91287829 2.1945398  1.4014697  1.92633403
 2.48438082 3.67454504 0.10282303 0.46761668 1.56085081 0.32670201
 2.67527591 0.55458485 1.36560227 1.93443078 1.11488377 4.06278223], Cost: 0.2202
Current parameters: [2.98529847 0.02064022 0.37338965 4.11552052 0.95423043 0.81585186
 0.22472733 1.35762559 2.81957942 2.33434568 1.34428548 1.77429509
 2.48115305 3.82672418 0.34242382 0.50890322 1.68741772 0.19803996
 2.85621964 0.36126651 1.27660433 1.87304755 1.2118904  4.15906862], Cost: 1.3149
Current parameters: [2.96792785 0.03141973 0.38361204 4.0681771  1.03404186 0.80396592
 0.28112027 1.35250622 2.85595311 2.1028716  1.34149965 1.87318372
 2.55748735 3.69958494 0.08447856 0.50905803 1.56384201 0.42971112
 2.74412652 0.6015957  1.28762924 1.95489906 1.18916586 4.14498115], Cost: 0.8959
Current parameters: [ 3.1454421  -0.0102255   0.23965371  3.98271827  0.75356744  0.86893899
  0.3412811   1.39189857  2.93199222  2.13511525  1.37966397  1.96598645
  2.21864267  3.59951247  0.05359247  0.4035939   1.53841492  0.47215762
  2.80499814  0.46496677  1.44942188  1.97984164  1.05429132  3.97824665], Cost: 0.2941
Current parameters: [3.01833252 0.02176897 0.33323278 3.96621137 1.06298075 0.90609547
 0.37754506 1.45163222 2.95746936 2.24881914 1.44206752 1.97106763
 2.47796677 3.6846745  0.10913199 0.47608934 1.55831228 0.30373134
 2.67305034 0.53286057 1.33623635 1.93015832 1.15533134 4.08347477], Cost: 0.2203
Current parameters: [ 3.01117673 -0.08619027  0.33237301  4.05226761  0.98750557  0.82366649
```



```
  0.27902188  1.41281002  2.8565246   2.15634701  1.3907121   1.86794973
  2.48398711  3.67896611  0.11363708  0.46664567  1.55952262  0.31457587
  2.68332644  0.54051267  1.36412499  1.92464192  1.1147473   4.06401364], Cost: 0.1474
Current parameters: [3.0465581  0.13855412 0.329837   4.04681077 0.97345981 0.82785912
 0.23476521 1.5231455  2.81338702 2.1064499  1.46510897 1.82647832
 2.46857966 3.67512438 0.10886917 0.46467885 1.55790354 0.30125538
 2.66690231 0.53496568 1.36381796 1.92810477 1.11628149 4.06440857], Cost: 0.1118
Current parameters: [3.02593199 0.02290123 0.33266969 4.06144606 0.9873983  0.93338053
 0.30704609 1.35551609 2.82528103 2.15804932 1.34650729 1.86982525
 2.483916   3.67843064 0.11286643 0.46647029 1.5582587  0.31357013
 2.68226536 0.5401909  1.36385816 1.92405525 1.11472265 4.06423607], Cost: 0.0379
Current parameters: [3.02812313 0.02346199 0.3301582  4.05344339 0.98332512 0.85696477
 0.47778567 1.36913455 2.66369069 2.17766354 1.35969036 1.89127115
 2.47918631 3.68016081 0.11560983 0.4668011  1.56482236 0.31478737
 2.68276035 0.54015248 1.36525541 1.92850491 1.11537192 4.06311763], Cost: 0.1865
Current parameters: [3.02624138 0.02329386 0.33184771 4.06161655 0.9876955  0.93323746
 0.30121394 1.2808176  2.81917604 2.15393262 1.4445186  1.86455513
 2.48411302 3.6811458  0.11751633 0.4669562  1.54188376 0.31460068
 2.68682844 0.53903286 1.36459743 1.92458995 1.11479094 4.06361983], Cost: 0.0296
Current parameters: [3.02783434 0.01863069 0.33758262 4.05457862 0.97062167 0.94065511
 0.30595834 1.2524449  2.82963705 2.12993477 1.4679242  1.86264716
 2.46668282 3.64624418 0.05833959 0.46009128 1.76500851 0.29615655
 2.61952483 0.55130491 1.35684523 1.9259614  1.11564128 4.07022118], Cost: 0.1288
Current parameters: [3.02564433 0.0225362  0.33343391 4.06128755 0.98712198 0.93351355
 0.3675054  1.27987418 2.87325105 2.10121044 1.44334941 1.79518546
 2.48373282 3.68457352 0.13470503 0.46601851 1.5367374  0.30009248
 2.68927532 0.52684414 1.36317083 1.92232597 1.11465915 4.064809  ], Cost: 0.0625
Current parameters: [3.02594282 0.01909915 0.33833043 4.05693345 0.97678887 0.93803028
 0.29126628 1.27592851 2.81640869 2.12426056 1.43839303 1.9012487
 2.47339405 3.64215233 0.26715024 0.46114042 1.5442707  0.30792261
 2.61499686 0.56465481 1.35728607 2.09740155 1.11500972 4.0697939 ], Cost: 0.2064
Current parameters: [3.02624138 0.02329386 0.33184771 4.06161655 0.9876955  0.93323746
 0.30121394 1.2808176  2.81917604 2.2154879  1.4445186  1.80299985
 2.48411302 3.68161902 0.06342645 0.4669562  1.5334484  0.31393387
 2.68746047 0.53824536 1.36459743 1.99564159 1.11479094 4.06361983], Cost: 0.2707
Current parameters: [3.03828173 0.02525802 0.31971279 4.05248406 0.96220413 0.94391208
 0.30231135 1.28442906 2.82457746 2.2398126  1.44876249 1.79011465
 2.45504077 3.62330573 0.19385714 0.46720857 1.52790666 0.4010334
 2.59568065 0.64034887 1.3703436  1.81479754 1.11844204 4.05910754], Cost: 0.4726
Current parameters: [3.02552922 0.02396284 0.32963024 4.05990985 0.98871237 0.93724183
 0.30293425 1.28551931 2.82029536 2.15720238 1.44863951 1.8629709
```



```
 2.47907457 3.64186302 0.12482659 0.46727737 1.55117043 0.26336216
 2.75844909 0.61640433 1.37205423 1.92705358 1.11106241 4.05943492], Cost: 0.1403
Current parameters: [3.05883566 0.02159615 0.32522152 4.05696813 0.92307381 0.92071756
 0.28823518 1.24667939 2.82094352 2.10958833 1.41694726 1.91876305
 2.46578979 3.63767371 0.05156422 0.46463661 1.45617662 0.17385989
 2.59416021 0.51519488 1.31049523 1.88269656 1.15605704 4.09117829], Cost: 0.8645
Current parameters: [3.06545153 0.01646308 0.32727929 4.05854944 0.97352738 0.93642748
 0.30369361 1.2818227  2.82275832 2.14950889 1.44505103 1.86934831
 2.467072   3.76931742 0.11382317 0.45638585 1.54288597 0.30623464
 2.67479662 0.58915601 1.3787358  1.92987926 1.09810257 4.11170759], Cost: 0.1035
Current parameters: [2.9305891  0.03762877 0.32972302 4.05450057 0.97240709 0.94317673
 0.29992354 1.28110945 2.82331464 2.13513257 1.44435837 1.88129512
 2.46559912 3.79100172 0.11525158 0.46649543 1.54710199 0.30384438
 2.66912117 0.58177381 1.34886311 1.92564309 1.12850135 3.87134775], Cost: 0.3319
Current parameters: [3.01732901 0.02439152 0.33359636 4.05507549 0.99622221 0.9330018
 0.30690782 1.27990125 2.82390097 2.1330501  1.44380693 1.87576874
 2.49223609 3.66870771 0.09244832 0.53540163 1.52902373 0.31974885
 2.661717   0.55565227 1.45428796 1.93232459 1.12298831 4.06164857], Cost: 0.2810
Current parameters: [3.0183603  0.02793373 0.33924414 4.08100243 0.95796741 0.95073563
 0.2693933  1.28297034 2.80267931 2.21748609 1.4453414  1.84295682
 2.4558386  3.74251959 0.23123748 0.52196793 1.60840904 0.26607746
 2.77079166 0.41419348 1.40202843 1.88552776 1.15039928 4.09304236], Cost: 0.3118
Current parameters: [3.03579966 0.02103619 0.43245328 4.05227222 0.92867183 0.94139039
 0.31118578 1.28765666 2.8297982  2.15636426 1.45148864 1.87803413
 2.47168767 3.6726571  0.10925294 0.4582489  1.53562001 0.33358217
 2.70050569 0.53554816 1.37386931 1.92720079 1.10254709 4.05572819], Cost: 0.0463
Current parameters: [3.08097517 0.0149586  0.22125015 4.04196579 0.9438448  0.958542
 0.31012424 1.28530565 2.83901896 2.10346114 1.44746007 1.91230707
 2.38478048 3.64923458 0.09455063 0.4134733  1.54438816 0.42470177
 2.7677008  0.48857541 1.38272786 1.94413193 1.07001282 4.03677971], Cost: 0.3287
Current parameters: [3.04925945 0.05190385 0.30730528 4.03788153 0.94414493 0.89051184
 0.30427088 1.25574461 2.84466685 2.17516445 1.42871507 1.87414951
 2.55822908 3.68311713 0.12848066 0.45190224 1.55100853 0.31044608
 2.69135547 0.53453167 1.38478507 1.9237958  1.09053684 4.04554153], Cost: 0.1485
Current parameters: [ 3.05209034 -0.04705731  0.33071739  4.09430488  0.99991033  1.06645975
  0.30545902  1.36631287  2.77454603  2.12067006  1.50761742  1.86458818
  2.62863025  3.68596854  0.12193051  0.42667093  1.54969009  0.32674408
  2.69001975  0.5246576   1.35614607  1.91522653  1.0968356   4.05650658], Cost: 0.2320
Current parameters: [2.99558564 0.03090535 0.34344951 4.0628996  1.01111826 0.92720177
 0.29680371 1.27513955 2.81706283 2.15236248 1.43991873 1.86401034
 2.48083114 3.6799435  0.11894633 0.37360967 1.54044287 0.30938762
```



```
 2.68372258 0.53312308 1.42298479 1.91969092 1.15230667 4.07717469], Cost: 0.1277
Current parameters: [2.98067001 0.02696136 0.32178722 4.06027655 0.96857035 0.9393614
 0.30425239 1.28556293 2.82046291 2.15576378 1.44869374 1.86761144
 2.48444098 3.68007386 0.11532459 0.46699336 1.53899648 0.31321479
 2.68465475 0.53884276 1.36309846 1.92365141 1.08829888 4.08642766], Cost: 0.0436
Current parameters: [3.03626621 0.03159666 0.36421232 4.06182161 1.05795734 0.92246986
 0.2917085  1.27034313 2.81593462 2.15188586 1.43650295 1.8652317
 2.47428153 3.68044708 0.12627742 0.45863071 1.54451489 0.30416743
 2.68310405 0.52226089 1.36244706 1.91249267 1.02433621 4.05420036], Cost: 0.1960
Current parameters: [3.02527591 0.01732669 0.33334255 4.08689976 0.97124077 0.92247436
 0.28043092 1.26720933 2.79290009 2.13008713 1.42886907 1.84073959
 2.48772443 3.68031099 0.11723631 0.4661465  1.54220156 0.32093406
 2.6907638  0.54301584 1.36853782 1.92451458 1.10782141 4.06181735], Cost: 0.0798
Current parameters: [3.00331022 0.04713861 0.35497048 4.04622567 1.02011945 0.91754147
 0.29862527 1.26783353 2.82918063 2.13041575 1.43573701 1.88086157
 2.51361002 3.69488112 0.11525516 0.48148473 1.55209079 0.37043674
 2.72055611 0.5505568  1.31285442 1.92654127 1.15261169 4.10118642], Cost: 0.3399
Current parameters: [ 3.01988805 -0.01546384  0.33242714  4.06964942  0.99091833  0.94437579
  0.31611138  1.24846775  2.82743755  2.16683507  1.42239066  1.87607856
  2.4877306   3.68180812  0.11817152  0.46742356  1.54201047  0.31770968
  2.69026707  0.54064187  1.36459263  1.92392107  1.11449468  4.06356298], Cost: 0.0485
Current parameters: [3.04575532 0.02115897 0.32671128 4.05966728 0.96070374 0.93807448
 0.29372404 1.27351158 2.81822858 2.13561807 1.43833049 1.89268573
 2.46764183 3.66012356 0.09297724 0.45807315 1.5036945  0.24311585
 2.65031231 0.51240999 1.31992614 1.8998289  1.13575357 4.07497822], Cost: 0.2257
Current parameters: [3.01692446 0.01191985 0.33203733 4.01058351 0.98626828 0.94251289
 0.29169126 1.27505363 2.80352126 2.144813   1.43728116 1.85235147
 2.48243564 3.69087032 0.11939162 0.46665374 1.54160796 0.31074914
 2.68730956 0.53989507 1.36307273 1.92321732 1.11578419 4.0487176 ], Cost: 0.0421
Current parameters: [2.98514373 0.03796211 0.3294692  4.09492802 0.98105188 0.9363468
 0.30711279 1.28870843 2.83206104 2.15204748 1.45244788 1.88339771
 2.47126104 3.73320884 0.11719694 0.46318001 1.54509274 0.30784964
 2.67877677 0.55580582 1.34849998 1.92402276 1.12212584 3.97272554], Cost: 0.0975
Current parameters: [3.02532137 0.02366557 0.33151092 4.06004794 0.9892351  0.91514039
 0.34355728 1.28532119 2.77945954 2.15978901 1.44883673 1.86861785
 2.48367863 3.68372558 0.12114424 0.46712318 1.54728127 0.32072331
 2.69020759 0.5396212  1.36701597 1.92645539 1.11335315 4.06191287], Cost: 0.0036
Current parameters: [3.04453322 0.02235002 0.32605091 4.05981637 0.96238902 0.91068529
 0.35760862 1.28046721 2.75939871 2.14435124 1.44509105 1.90016735
 2.46650423 3.66395764 0.0978164  0.4580866  1.51103675 0.2508194
 2.65445678 0.51294388 1.32240898 1.90214269 1.13411931 4.07185566], Cost: 0.2318
```

```
Current parameters: [3.04194548 0.0127504  0.32747652 4.07056959 0.99663136 0.94617342
 0.33736084 1.30987032 2.77681605 2.156591   1.47291671 1.87135672
 2.51506184 3.67957946 0.12129561 0.46216529 1.54601296 0.32257366
 2.68722743 0.53293581 1.36289793 1.92329655 1.11601146 4.06087148], Cost: 0.0098
Current parameters: [ 2.98275286 -0.01600962  0.3446837   4.04929886  0.96269665  0.93959881
  0.38947614  1.28461279  2.73406136  2.14056482  1.41897201  1.85557753
  2.51549615  3.71083846  0.12765961  0.44353224  1.56538474  0.32355833
  2.70652786  0.55476423  1.38218949  1.93247669  1.07503328  4.05038515], Cost: 0.0684
Current parameters: [3.03921626 0.020372   0.30393568 4.055599   0.97846062 0.92365446
 0.34820424 1.2891524  2.78319899 2.14802587 1.4513906  1.88077704
 2.45710641 3.6761516  0.11532652 0.45481524 1.54786994 0.34692422
 2.70947671 0.52726603 1.37150178 1.93160377 1.10238549 4.05390709], Cost: 0.0185
Current parameters: [3.02553218 0.02344481 0.33246336 4.06006604 0.98916132 0.91552653
 0.34184235 1.28494111 2.77931864 2.15315161 1.44857602 1.87618185
 2.48321325 3.68074003 0.13679794 0.46651432 1.54786743 0.31697997
 2.68454038 0.53879335 1.3655039  1.95024637 1.11370496 4.06283278], Cost: 0.0075
Current parameters: [3.02756258 0.02223104 0.32766984 4.05811267 0.98110659 0.92156492
 0.34682412 1.2882267  2.77721218 2.17804978 1.45004726 1.85525637
 2.47262854 3.6688666  0.15297273 0.46281006 1.54509869 0.34179619
 2.66445769 0.56067233 1.36225748 1.90997908 1.11293797 4.05847259], Cost: 0.0148
Current parameters: [3.0242295  0.02400908 0.33119597 4.06087526 0.99048844 0.91439678
 0.34317024 1.28841481 2.777475   2.16372277 1.44517113 1.86786228
 2.48563295 3.68937065 0.12972792 0.46897794 1.52078083 0.32216987
 2.70013715 0.53695121 1.36949288 1.92627554 1.11325473 4.0615522 ], Cost: 0.0107
Current parameters: [3.02479644 0.02315011 0.33158626 4.06485615 0.98098213 0.92254442
 0.33841557 1.28824463 2.77499208 2.1763809  1.45241312 1.86284912
 2.4723631  3.69666253 0.14786851 0.48142621 1.57172265 0.30884069
 2.70684716 0.51034019 1.37641786 1.91670909 1.12226261 4.06770832], Cost: 0.0134
Current parameters: [3.02557636 0.01386227 0.33104137 4.05981691 0.99008758 0.90497279
 0.34074266 1.30756947 2.78481408 2.16082019 1.43405146 1.87048128
 2.48311386 3.68232455 0.12063113 0.46730327 1.55073323 0.32055775
 2.68866788 0.53874882 1.36570927 1.92580418 1.11456259 4.0621272 ], Cost: 0.0050
Current parameters: [ 3.01722491 -0.01127937  0.33170633  4.05617732  0.9864273   0.88346117
  0.33783233  1.28045931  2.78319193  2.15747193  1.48691207  1.86590281
  2.48269499  3.68675972  0.12538108  0.4650801   1.54500185  0.3223578
  2.69278694  0.53990082  1.36719922  1.92507064  1.11043692  4.06020886], Cost: 0.0097
Current parameters: [3.0301903  0.02485678 0.34988147 4.0591523  0.97937759 0.91820782
 0.3435122  1.28982509 2.78363727 2.16143766 1.45281184 1.872715
 2.47944248 3.67387557 0.12139534 0.4659279  1.54753904 0.31577761
 2.70366669 0.54988753 1.36799895 1.92632168 1.1124818  4.05984961], Cost: 0.0043
```



```
   Return from subroutine COBYLA because the MAXFUN limit has been reached.

   NFVALS =  100   F = 3.600477E-03    MAXCV = 0.000000E+00
   X = 3.025321E+00   2.366557E-02   3.315109E-01   4.060048E+00   9.892351E-01
       9.151404E-01   3.435573E-01   1.285321E+00   2.779460E+00   2.159789E+00
       1.448837E+00   1.868618E+00   2.483679E+00   3.683726E+00   1.211442E-01
       4.671232E-01   1.547281E+00   3.207233E-01   2.690208E+00   5.396212E-01
       1.367016E+00   1.926455E+00   1.113353E+00   4.061913E+00
Current parameters: [3.02532137 0.02366557 0.33151092 4.06004794 0.9892351  0.91514039
 0.34355728 1.28532119 2.77945954 2.15978901 1.44883673 1.86861785
 2.48367863 3.68372558 0.12114424 0.46712318 1.54728127 0.32072331
 2.69020759 0.5396212  1.36701597 1.92645539 1.11335315 4.06191287], Cost: 0.0036
```


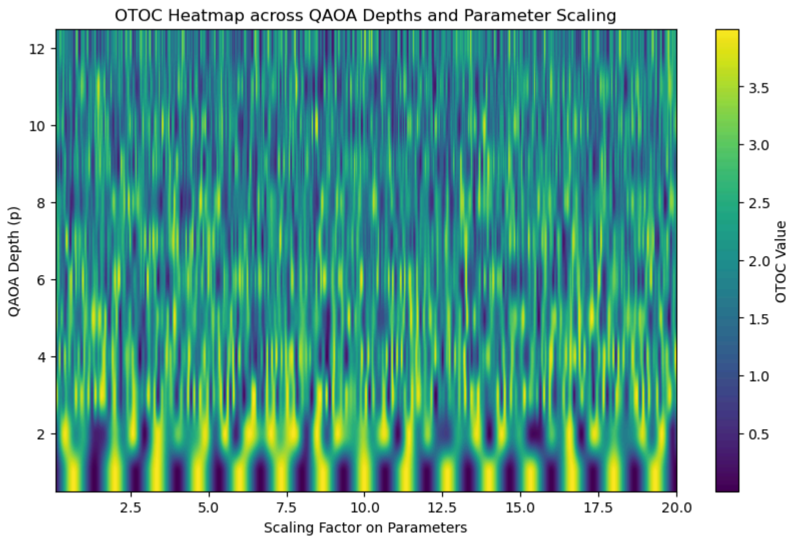


```
Run 8/30 for heatmap generation...
Simulating for QAOA depth p = 1
Current parameters: [1.95323982 2.24962424], Cost: 1.7137
Current parameters: [1.95323982 3.24962424], Cost: 1.7100
Current parameters: [2.95322503 3.25506283], Cost: 1.8387
Current parameters: [1.45344408 3.2639148 ], Cost: 2.1093
Current parameters: [2.20323717 3.25077578], Cost: 1.5967
Current parameters: [2.45322893 3.25280533], Cost: 1.5778
Current parameters: [2.60472903 3.45167141], Cost: 1.1686
Current parameters: [2.61208951 3.70156304], Cost: 1.3161
Current parameters: [2.72746986 3.42801367], Cost: 1.3288
Current parameters: [2.58688941 3.51157131], Cost: 1.1083
Current parameters: [2.53226904 3.54195001], Cost: 1.0618
Current parameters: [2.50979837 3.60027084], Cost: 1.0798
Current parameters: [2.50574173 3.52543142], Cost: 1.0450
Current parameters: [2.48624936 3.50100581], Cost: 1.0422
Current parameters: [2.4605913 3.5188447], Cost: 1.0236
Current parameters: [2.43351448 3.53444626], Cost: 1.0119
Current parameters: [2.40252767 3.53039898], Cost: 1.0044
Current parameters: [2.37994693 3.55200159], Cost: 1.0036
Current parameters: [2.36821653 3.54167985], Cost: 1.0007
Current parameters: [2.35600381 3.53193357], Cost: 1.0000
Current parameters: [2.34200837 3.53888112], Cost: 1.0006
Current parameters: [2.35639384 3.51631344], Cost: 1.0026
Current parameters: [2.35619882 3.52412351], Cost: 1.0008
Current parameters: [2.35284571 3.53423253], Cost: 1.0000
Current parameters: [2.35399519 3.53581158], Cost: 1.0000
Current parameters: [2.34898528 3.53482909], Cost: 1.0001
Current parameters: [2.35091549 3.53453081], Cost: 1.0001
Current parameters: [2.35354838 3.53355435], Cost: 1.0000
Current parameters: [2.35382899 3.53261897], Cost: 1.0000
Current parameters: [2.35429738 3.53418098], Cost: 1.0000
Current parameters: [2.35518242 3.53459375], Cost: 1.0000
Current parameters: [2.35492725 3.53553639], Cost: 1.0000
Current parameters: [2.35585001 3.53388101], Cost: 1.0000
Current parameters: [2.35628172 3.53410916], Cost: 1.0000
Current parameters: [2.3567498  3.53424814], Cost: 1.0000
Current parameters: [2.35639579 3.53389331], Cost: 1.0000
Current parameters: [2.35631187 3.53459651], Cost: 1.0000
Current parameters: [2.35651322 3.53403162], Cost: 1.0000
Current parameters: [2.35628085 3.53420916], Cost: 1.0000
```

```
Current parameters: [2.35622873 3.5342945 ], Cost: 1.0000
Current parameters: [2.35630073 3.53436391], Cost: 1.0000
Current parameters: [2.35630073 3.53436391], Cost: 1.0000

   Normal return from subroutine COBYLA

   NFVALS =   42   F = 1.000000E+00    MAXCV = 0.000000E+00
   X = 2.356301E+00   3.534364E+00
Simulating for QAOA depth p = 2
Current parameters: [2.67085063 1.28479136 2.60088325 0.25697804], Cost: 2.6969
Current parameters: [1.67085063 2.28479136 2.60088325 0.25697804], Cost: 2.0336
Current parameters: [1.67085063 2.28479136 3.60088325 0.25697804], Cost: 1.1553
Current parameters: [1.67085063 2.28479136 3.60088325 1.25697804], Cost: 1.4746
Current parameters: [ 1.15029705  2.36740492  4.39956307 -0.03338296], Cost: 1.2893
Current parameters: [2.11403095 2.30719029 3.81742846 0.17825277], Cost: 0.9331
Current parameters: [2.29666107 2.75140142 3.77331983 0.04643042], Cost: 1.4217
Current parameters: [ 2.11403095  2.23252923  3.7427674  -0.04835582], Cost: 1.5824
Current parameters: [2.18993634 2.23999568 4.00888851 0.62888156], Cost: 1.7973
Current parameters: [2.2288662  2.25760294 3.60987533 0.23969917], Cost: 0.4888
Current parameters: [ 2.38442467  1.93190604  3.38165851 -0.02037808], Cost: 1.1604
Current parameters: [2.29300774 2.47029673 3.49619098 0.22476333], Cost: 0.7471
Current parameters: [2.12074057 2.2564054  3.5477693  0.23102412], Cost: 0.7173
Current parameters: [2.3055032  2.20004975 3.62794304 0.4698903 ], Cost: 0.1598
Current parameters: [2.49032121 2.03422126 3.60227143 0.48346169], Cost: 0.2471
Current parameters: [2.32438381 2.23245114 3.85774185 0.56091874], Cost: 0.7387
Current parameters: [2.36637613 2.28661592 3.56651666 0.49543601], Cost: 0.3961
Current parameters: [2.22769224 2.13806415 3.56850346 0.51674302], Cost: 0.2047
Current parameters: [2.34386751 2.15345895 3.62013596 0.45565033], Cost: 0.1449
Current parameters: [2.35207157 2.14978373 3.63409646 0.48212396], Cost: 0.1370
Current parameters: [2.3371947  2.12512086 3.68655214 0.46409417], Cost: 0.0704
Current parameters: [2.33661873 2.10176257 3.74238431 0.44850322], Cost: 0.0775
Current parameters: [2.35790534 2.1432944  3.69762467 0.45436004], Cost: 0.0935
Current parameters: [2.30740164 2.07983387 3.71765198 0.46480992], Cost: 0.0373
Current parameters: [2.32187704 2.072594   3.77438018 0.44416512], Cost: 0.1005
Current parameters: [2.33035006 2.06024496 3.71100318 0.46950042], Cost: 0.0369
Current parameters: [2.32190695 2.06187615 3.71942071 0.53083106], Cost: 0.1102
Current parameters: [2.30235927 2.03241137 3.67327094 0.43909755], Cost: 0.0197
Current parameters: [2.3091979  2.01696996 3.70281792 0.38667597], Cost: 0.0306
Current parameters: [2.28263799 2.01167523 3.67231853 0.45161711], Cost: 0.0156
Current parameters: [2.27178639 2.00733844 3.64337097 0.45304418], Cost: 0.0488
Current parameters: [2.29443532 2.00222034 3.66944588 0.45432203], Cost: 0.0212
```



```
Current parameters: [2.2802845  2.01849038 3.69781102 0.46819149], Cost: 0.0075
Current parameters: [2.27426324 2.00796623 3.71815351 0.44780198], Cost: 0.0054
Current parameters: [2.24991479 2.01990996 3.73196799 0.45488877], Cost: 0.0139
Current parameters: [2.28250768 2.01948476 3.72158177 0.44216813], Cost: 0.0078
Current parameters: [2.27766852 2.00637844 3.72309469 0.45254569], Cost: 0.0092
Current parameters: [2.27185675 2.00253152 3.70752029 0.43801689], Cost: 0.0028
Current parameters: [2.25748819 2.00761187 3.70538556 0.43531172], Cost: 0.0022
Current parameters: [2.25839398 2.01040883 3.69992474 0.44006263], Cost: 0.0016
Current parameters: [2.25867611 2.00785822 3.68657898 0.43235249], Cost: 0.0080
Current parameters: [2.2556059  2.00399681 3.69913064 0.44345632], Cost: 0.0017
Current parameters: [2.25588268 2.01699898 3.70871781 0.45088326], Cost: 0.0013
Current parameters: [2.25391458 2.02222036 3.70404901 0.45372954], Cost: 0.0008
Current parameters: [2.25692937 2.02625754 3.70005563 0.45816802], Cost: 0.0015
Current parameters: [2.25513327 2.02492618 3.70517254 0.45145143], Cost: 0.0009
Current parameters: [2.24624655 2.02305591 3.70401675 0.45248964], Cost: 0.0003
Current parameters: [2.23990643 2.02503375 3.70091614 0.45519377], Cost: 0.0001
Current parameters: [2.23546993 2.02327997 3.70682486 0.45702786], Cost: 0.0008
Current parameters: [2.23918562 2.02281937 3.7006886  0.45206583], Cost: 0.0000
Current parameters: [2.23787721 2.02265876 3.69311621 0.4506672 ], Cost: 0.0009
Current parameters: [2.23899445 2.02875901 3.70262307 0.44737785], Cost: 0.0003
Current parameters: [2.24272063 2.021583   3.7016784  0.45156168], Cost: 0.0001
Current parameters: [2.23749979 2.02102961 3.703679   0.45258647], Cost: 0.0001
Current parameters: [2.2391824  2.02413527 3.7014983  0.45087105], Cost: 0.0000
Current parameters: [2.23886161 2.0224093  3.6990996  0.45105755], Cost: 0.0001
Current parameters: [2.23868068 2.02229117 3.70133638 0.45207561], Cost: 0.0000
Current parameters: [2.23830855 2.02252391 3.70123325 0.45226306], Cost: 0.0000
Current parameters: [2.23857762 2.02269069 3.70163648 0.45309424], Cost: 0.0000
Current parameters: [2.23828872 2.02219299 3.70094474 0.45247584], Cost: 0.0000
Current parameters: [2.23776589 2.02246597 3.7008887  0.45153017], Cost: 0.0000
Current parameters: [2.23737415 2.02266906 3.70102336 0.45169013], Cost: 0.0000
Current parameters: [2.23705298 2.02300145 3.70102156 0.45184755], Cost: 0.0000
Current parameters: [2.23672035 2.02291495 3.70136482 0.45189727], Cost: 0.0000
Current parameters: [2.23712883 2.02313964 3.70114952 0.45171198], Cost: 0.0000
Current parameters: [2.23699287 2.02305889 3.70054677 0.45192555], Cost: 0.0000
Current parameters: [2.23712255 2.02296029 3.70104542 0.45207668], Cost: 0.0000
Current parameters: [2.23742813 2.02331248 3.70116825 0.45215358], Cost: 0.0000
Current parameters: [2.23707306 2.02293388 3.70061298 0.45229638], Cost: 0.0000
Current parameters: [2.23727912 2.0227748  3.70103823 0.45205159], Cost: 0.0000
Current parameters: [2.23700096 2.02285431 3.70116552 0.45221511], Cost: 0.0000
Current parameters: [2.23711862 2.02295952 3.70095286 0.45211433], Cost: 0.0000
Current parameters: [2.23718024 2.02301431 3.70092179 0.4521616 ], Cost: 0.0000
```



```
Current parameters: [2.2371831  2.02303784 3.70082895 0.45213296], Cost: 0.0000
Current parameters: [2.23714531 2.02304861 3.70092663 0.45217057], Cost: 0.0000
Current parameters: [2.23713052 2.02301543 3.7009031  0.45226072], Cost: 0.0000
Current parameters: [2.23718207 2.02305196 3.70093571 0.45233104], Cost: 0.0000
Current parameters: [2.23718207 2.02305196 3.70093571 0.45233104], Cost: 0.0000

   Normal return from subroutine COBYLA

   NFVALS =   78   F = 2.043789E-07    MAXCV = 0.000000E+00
   X = 2.237182E+00   2.023052E+00   3.700936E+00   4.523310E-01
Simulating for QAOA depth p = 3
Current parameters: [3.95732295 0.52763781 2.43353801 0.82537545 0.5280769  1.06379257], Cost: 2.9782
Current parameters: [2.95732295 1.52763781 2.43353801 0.82537545 0.5280769  1.06379257], Cost: 1.1866
Current parameters: [2.95732295 1.52763781 3.43353801 0.82537545 0.5280769  1.06379257], Cost: 2.4550
Current parameters: [2.95732295 1.52763781 2.43353801 1.82537545 0.5280769  1.06379257], Cost: 1.1714
Current parameters: [2.95732295 1.52763781 2.43353801 1.82537545 1.5280769  1.06379257], Cost: 1.3217
Current parameters: [2.95732295 1.52763781 2.43353801 1.82537545 0.5280769  2.06379257], Cost: 0.3050
Current parameters: [2.71321348 2.14836054 1.82127233 1.83269464 0.45552085 2.48203489], Cost: 1.5984
Current parameters: [2.57193123 1.21538272 2.38176029 1.82599442 0.52194102 2.09916224], Cost: 0.8328
Current parameters: [3.0823982  1.40256256 2.2568678  1.82537545 0.5280769  2.06379257], Cost: 0.5668
Current parameters: [3.16825896 1.52956183 2.85647632 1.82818852 0.50019057 2.22454059], Cost: 0.9667
Current parameters: [2.95676783 1.52904936 2.43214569 2.07536697 0.5280769  2.06379257], Cost: 0.2827
Current parameters: [2.8306282  1.95645668 2.23845456 2.08349534 0.50797627 2.17966067], Cost: 0.4134
Current parameters: [2.95676783 1.52904936 2.43214569 2.07536697 0.28175591 2.02106117], Cost: 1.2376
Current parameters: [2.94309478 1.62528577 2.46855732 2.0818544  0.88485643 1.72928984], Cost: 1.3691
Current parameters: [2.74247664 1.48663589 2.47264522 1.96074145 0.528261   2.06273132], Cost: 0.3343
Current parameters: [2.88752711 1.35490803 2.34472808 2.26499418 0.72041949 2.43024111], Cost: 1.6400
Current parameters: [3.03416476 1.6040324  2.6042442  1.96763714 0.55241414 2.15903618], Cost: 0.3520
Current parameters: [2.95991883 1.55865101 2.44580958 2.0721063  0.61057962 1.97584496], Cost: 0.4406
Current parameters: [2.96720579 1.47742055 2.46218352 2.0856742  0.52997451 2.05285396], Cost: 0.3241
Current parameters: [2.93691423 1.51691748 2.40360846 2.11575303 0.59501478 2.1541118 ], Cost: 0.2828
Current parameters: [2.95291447 1.55783282 2.48344673 2.08990683 0.52554333 2.07839708], Cost: 0.2855
Current parameters: [3.01386458 1.5300626  2.45129423 1.978231   0.55122441 2.10880438], Cost: 0.2167
Current parameters: [3.02631295 1.54295261 2.434815   1.9621906  0.51006979 2.14571744], Cost: 0.1534
Current parameters: [3.04424356 1.56865234 2.41461966 1.93790257 0.48131016 2.17887449], Cost: 0.1111
Current parameters: [3.06512647 1.53700974 2.42270343 1.93095982 0.44537022 2.21148847], Cost: 0.0854
Current parameters: [3.10095176 1.54687458 2.41785338 1.89195021 0.43810756 2.24194277], Cost: 0.0556
Current parameters: [3.09104211 1.57782261 2.38901785 1.89903418 0.39815522 2.26124066], Cost: 0.0629
Current parameters: [3.10586599 1.54989768 2.42499645 1.88182823 0.42137424 2.21936421], Cost: 0.1034
Current parameters: [3.06341606 1.53350743 2.41966268 1.86393936 0.45220515 2.27843957], Cost: 0.0268
Current parameters: [3.0776277  1.50594012 2.3756943  1.86827531 0.47506144 2.3001166 ], Cost: 0.0651
```



```
Current parameters: [3.06770318 1.51426434 2.43022271 1.87290623 0.43517754 2.28873656], Cost: 0.0169
Current parameters: [3.07455145 1.53808531 2.46488489 1.89167317 0.45235231 2.32672702], Cost: 0.0418
Current parameters: [3.05202494 1.51009446 2.42950767 1.89687663 0.44261338 2.27962701], Cost: 0.0216
Current parameters: [3.01978479 1.51016648 2.43344236 1.84854961 0.40643824 2.30153211], Cost: 0.0196
Current parameters: [3.06601711 1.49757558 2.44687935 1.8607554  0.44931783 2.28035974], Cost: 0.0248
Current parameters: [3.06895884 1.52066916 2.43857494 1.87373142 0.42832346 2.27956469], Cost: 0.0225
Current parameters: [3.07810854 1.4980944  2.40942055 1.87396943 0.42316691 2.2940946 ], Cost: 0.0176
Current parameters: [3.06993701 1.51631442 2.43351916 1.87662378 0.43640271 2.30318509], Cost: 0.0131
Current parameters: [3.05789612 1.51583076 2.43287481 1.87138944 0.43025165 2.30895349], Cost: 0.0090
Current parameters: [3.05366014 1.52306119 2.42529692 1.8667233  0.43481558 2.31754949], Cost: 0.0108
Current parameters: [3.06100231 1.51458888 2.4351473  1.86476488 0.43047723 2.30981634], Cost: 0.0090
Current parameters: [3.0514001  1.50479025 2.43827337 1.87332482 0.43402345 2.31469221], Cost: 0.0094
Current parameters: [3.06217548 1.50980268 2.42617151 1.87265413 0.41952544 2.314236  ], Cost: 0.0064
Current parameters: [3.06077391 1.51438534 2.43432662 1.87436718 0.40809306 2.31882246], Cost: 0.0053
Current parameters: [3.05390628 1.51027439 2.43091423 1.86980201 0.39815157 2.31183976], Cost: 0.0140
Current parameters: [3.07263364 1.51467787 2.4367728  1.88178926 0.4099724  2.32505134], Cost: 0.0036
Current parameters: [3.06977435 1.52082028 2.43114364 1.87915489 0.41237913 2.3374536 ], Cost: 0.0019
Current parameters: [3.07136122 1.52693398 2.42848401 1.8803629  0.41153543 2.33400471], Cost: 0.0020
Current parameters: [3.06152194 1.52233591 2.43299145 1.89139842 0.41680566 2.33836394], Cost: 0.0031
Current parameters: [3.06986188 1.52384721 2.43629462 1.87655429 0.41665644 2.33693016], Cost: 0.0036
Current parameters: [3.07474233 1.51841911 2.42352161 1.88127344 0.40364381 2.34610258], Cost: 0.0005
Current parameters: [3.06815661 1.51852243 2.42176575 1.89475286 0.40426204 2.35005207], Cost: 0.0006
Current parameters: [3.07885121 1.51651538 2.42070213 1.88272006 0.40916309 2.34594924], Cost: 0.0017
Current parameters: [3.06514576 1.51618455 2.41404592 1.87543136 0.39889029 2.34536858], Cost: 0.0017
Current parameters: [3.07477099 1.51280741 2.42501829 1.88296014 0.40188097 2.34148192], Cost: 0.0008
Current parameters: [3.08092724 1.52488934 2.42609617 1.88404714 0.39157975 2.34814224], Cost: 0.0014
Current parameters: [3.07251336 1.51654251 2.42897074 1.87942264 0.40426769 2.35046578], Cost: 0.0007
Current parameters: [3.07189476 1.52300769 2.42653818 1.88127133 0.40563196 2.3417645 ], Cost: 0.0007
Current parameters: [3.07219597 1.5183237  2.42141282 1.87965313 0.40235075 2.3462487 ], Cost: 0.0004
Current parameters: [3.07309866 1.51833741 2.42163423 1.87801138 0.40221337 2.34576247], Cost: 0.0004
Current parameters: [3.07295441 1.51995178 2.42164666 1.88028729 0.39901997 2.34694295], Cost: 0.0003
Current parameters: [3.07426455 1.52159024 2.41903984 1.88060711 0.39804743 2.34867909], Cost: 0.0002
Current parameters: [3.07491078 1.52014239 2.41779931 1.8817137  0.39506422 2.34763895], Cost: 0.0007
Current parameters: [3.07437987 1.52047411 2.41944302 1.8802926  0.3978083  2.35017475], Cost: 0.0002
Current parameters: [3.07308938 1.52299178 2.42096582 1.87934772 0.39813009 2.35215956], Cost: 0.0001
Current parameters: [3.07582657 1.52371906 2.42274954 1.88088982 0.39929356 2.35272903], Cost: 0.0001
Current parameters: [3.07404328 1.52313272 2.42120353 1.87777031 0.39773342 2.351732  ], Cost: 0.0001
Current parameters: [3.07367199 1.52307645 2.41985213 1.87620114 0.40083804 2.35282314], Cost: 0.0003
Current parameters: [3.07361924 1.52353279 2.42174706 1.877892   0.39601053 2.35217501], Cost: 0.0001
Current parameters: [3.07469533 1.52351291 2.42081758 1.87805478 0.39765865 2.35211734], Cost: 0.0001
Current parameters: [3.07549551 1.52357991 2.42243528 1.87867949 0.39805917 2.35206946], Cost: 0.0001
```

```
Current parameters: [3.0744713  1.52435435 2.42083725 1.87807178 0.39780997 2.35170274], Cost: 0.0001
Current parameters: [3.07500091 1.52405864 2.42034945 1.87881611 0.39719569 2.35019362], Cost: 0.0001
Current parameters: [3.07417209 1.52546128 2.42082591 1.87732171 0.39865674 2.35280734], Cost: 0.0001
Current parameters: [3.0743217  1.52490782 2.42083158 1.87769674 0.39823336 2.35225504], Cost: 0.0001
Current parameters: [3.07459613 1.52423855 2.42068218 1.87805846 0.39820729 2.35153739], Cost: 0.0001
Current parameters: [3.07439135 1.5249026  2.42086731 1.87819959 0.39701812 2.35165334], Cost: 0.0000
Current parameters: [3.07413085 1.52536333 2.42104319 1.8778447  0.39715119 2.35235971], Cost: 0.0000
Current parameters: [3.07444326 1.52555994 2.42189666 1.87810366 0.39726056 2.35245962], Cost: 0.0000
Current parameters: [3.07431562 1.52558439 2.42178119 1.87852261 0.39734359 2.3526202 ], Cost: 0.0000
Current parameters: [3.0734209  1.52556556 2.42216575 1.87846805 0.39729979 2.35262112], Cost: 0.0000
Current parameters: [3.07331845 1.52534145 2.42227757 1.87840858 0.39688848 2.35346239], Cost: 0.0000
Current parameters: [3.07331059 1.52564611 2.42224389 1.87848423 0.39652639 2.35337539], Cost: 0.0000
Current parameters: [3.07299853 1.52646803 2.42219857 1.87840354 0.39676098 2.35371767], Cost: 0.0000
Current parameters: [3.07242532 1.52695138 2.42236554 1.87889595 0.39641304 2.35372616], Cost: 0.0000
Current parameters: [3.07230383 1.52743048 2.42306076 1.8785563  0.39609116 2.35364207], Cost: 0.0000
Current parameters: [3.07210695 1.52686007 2.42218392 1.87861682 0.39632603 2.35362493], Cost: 0.0000
Current parameters: [3.07203889 1.52716815 2.42140464 1.87903822 0.39614097 2.35381208], Cost: 0.0000
Current parameters: [3.0720574  1.52670522 2.42224411 1.87857647 0.39617479 2.35405372], Cost: 0.0000
Current parameters: [3.07155631 1.52671021 2.42227486 1.87898664 0.39702099 2.35362506], Cost: 0.0000
Current parameters: [3.07208956 1.52657109 2.4222198  1.87884782 0.39609909 2.35340479], Cost: 0.0000
Current parameters: [3.07222342 1.52671621 2.42207002 1.87857057 0.3964268  2.35363361], Cost: 0.0000
Current parameters: [3.07216759 1.52719909 2.42218673 1.87833412 0.39652507 2.35364114], Cost: 0.0000
Current parameters: [3.07225919 1.52745289 2.42226194 1.87811895 0.39656339 2.3533062 ], Cost: 0.0000
Current parameters: [3.0722432  1.5272876  2.42202325 1.87836073 0.39638989 2.35365962], Cost: 0.0000
Current parameters: [3.07194899 1.52738955 2.42199769 1.87850777 0.39682144 2.35366639], Cost: 0.0000
Current parameters: [3.07214392 1.52726204 2.42223102 1.87820715 0.39652776 2.35383348], Cost: 0.0000
Current parameters: [3.07212412 1.52714548 2.42212008 1.87826157 0.39652919 2.35362074], Cost: 0.0000
Current parameters: [3.07230578 1.5270772  2.42206286 1.87825749 0.3966644  2.35363993], Cost: 0.0000
Current parameters: [3.07208743 1.52719147 2.42211667 1.87833932 0.39660256 2.35362112], Cost: 0.0000
Current parameters: [3.07212412 1.52714548 2.42212008 1.87826157 0.39652919 2.35362074], Cost: 0.0000

   Return from subroutine COBYLA because the MAXFUN limit has been reached.

   NFVALS =  100   F = 2.366929E-06    MAXCV = 0.000000E+00
   X = 3.072124E+00   1.527145E+00   2.422120E+00   1.878262E+00   3.965292E-01
       2.353621E+00
Simulating for QAOA depth p = 4
Current parameters: [1.12422642 2.10608684 0.45808756 2.45301777 1.2725602  3.03834889
 1.30709965 0.26017843], Cost: 1.6457
Current parameters: [1.12422642 3.10608684 0.45808756 2.45301777 1.2725602  3.03834889
 1.30709965 0.26017843], Cost: 0.7536
```



```
Current parameters: [1.12422642 3.10608684 1.45808756 2.45301777 1.2725602  3.03834889
 1.30709965 0.26017843], Cost: 1.7691
Current parameters: [1.12422642 3.10608684 0.45808756 3.45301777 1.2725602  3.03834889
 1.30709965 0.26017843], Cost: 1.5708
Current parameters: [1.12422642 3.10608684 0.45808756 2.45301777 2.2725602  3.03834889
 1.30709965 0.26017843], Cost: 1.8101
Current parameters: [1.12422642 3.10608684 0.45808756 2.45301777 1.2725602  4.03834889
 1.30709965 0.26017843], Cost: 1.8222
Current parameters: [1.12422642 3.10608684 0.45808756 2.45301777 1.2725602  3.03834889
 2.30709965 0.26017843], Cost: 1.0277
Current parameters: [1.12422642 3.10608684 0.45808756 2.45301777 1.2725602  3.03834889
 1.30709965 1.26017843], Cost: 2.4613
Current parameters: [ 1.28225838  3.42262198  0.09775777  2.16306755  0.89767436  2.6591904
  1.20983846 -0.34576086], Cost: 0.9788
Current parameters: [ 1.2032424   3.26435441  0.27792266  2.30804266  1.08511728  2.84876964
  1.25846905 -0.04279121], Cost: 1.6590
Current parameters: [1.35317521 3.10608684 0.55849897 2.45301777 1.2725602  3.03834889
 1.30709965 0.26017843], Cost: 1.0943
Current parameters: [0.87411555 3.14644919 0.87486346 2.41604535 1.22475738 2.99000126
 1.29469758 0.1829133 ], Cost: 0.2736
Current parameters: [0.7910307  3.18951634 0.90051535 2.37659528 1.17375114 2.93841369
 1.28146441 0.39759858], Cost: 0.5686
Current parameters: [0.87411555 3.14644919 0.87486346 2.41604535 1.13586963 3.07788736
 1.29469758 0.1829133 ], Cost: 0.2721
Current parameters: [0.79061291 3.18973291 0.90064434 2.37639691 1.29162073 3.23619766
 1.28139787 0.13909852], Cost: 1.5850
Current parameters: [0.87411555 3.06201637 0.87486346 2.32387106 1.13586963 3.07788736
 1.29469758 0.1829133 ], Cost: 0.1892
Current parameters: [0.83441832 2.88597404 0.88711971 2.46438189 1.06691771 3.00852233
 1.28837489 0.16208372], Cost: 0.4171
Current parameters: [0.87703045 3.06785488 0.86821716 2.31852291 1.12895483 3.07089375
 1.17105294 0.17173671], Cost: 0.0330
Current parameters: [0.86553694 3.14086305 0.82019406 2.23196818 1.00149398 2.94233377
 1.17816063 0.05167295], Cost: 0.7791
Current parameters: [0.89279776 3.07662855 0.98957222 2.31048611 1.1185638  3.0603843
 1.16835706 0.15494138], Cost: 0.0259
Current parameters: [1.12767715 3.0470396  0.97258758 2.32075099 1.118528   3.06065063
 1.14265195 0.22843626], Cost: 0.2678
Current parameters: [0.85401696 3.08880986 1.00333005 2.29389708 1.09282342 3.03444812
 1.15339166 0.26425274], Cost: 0.1559
Current parameters: [0.89839213 3.11615842 0.987845   2.27427625 1.1406725  3.08274497
```



```
 1.1692481  0.1578768 ], Cost: 0.0946
Current parameters: [0.85586853 3.00999175 1.00912686 2.3467144  1.1715948  3.1144655
 1.12232219 0.13852117], Cost: 0.1368
Current parameters: [0.87721082 3.06864842 0.97062169 2.28622929 1.05886342 3.00057066
 1.13927529 0.07504587], Cost: 0.2270
Current parameters: [0.95068445 3.06177262 0.98543829 2.31733481 1.11758678 3.05951758
 1.15837676 0.16795586], Cost: 0.0526
Current parameters: [0.89279776 3.07699011 0.98957222 2.31088082 1.09634512 3.0823526
 1.16835706 0.15494138], Cost: 0.0267
Current parameters: [0.8940865  3.11442304 0.9894872  2.35973876 1.11858198 3.05871707
 1.16228843 0.15166623], Cost: 0.0446
Current parameters: [0.8940078  3.06471899 0.99334352 2.32277143 1.12101956 3.06243443
 1.22824763 0.15411504], Cost: 0.0804
Current parameters: [0.8842383  3.0606572  0.99460528 2.31958058 1.13263088 3.07449256
 1.15665854 0.15241311], Cost: 0.0342
Current parameters: [0.89450163 3.07780634 1.00481046 2.3093703  1.11709193 3.05889631
 1.16791882 0.15358905], Cost: 0.0301
Current parameters: [0.88225821 3.07421178 0.99050273 2.3114443  1.11239281 3.05395435
 1.16345162 0.18240711], Cost: 0.0323
Current parameters: [0.88528268 3.06932447 0.98247573 2.30808139 1.10408986 3.0454049
 1.15640132 0.13965162], Cost: 0.0178
Current parameters: [0.88011034 3.08415355 0.9740996  2.28671094 1.10759938 3.04828817
 1.14406516 0.13415239], Cost: 0.0157
Current parameters: [0.8676263  3.08605623 0.97540453 2.28646004 1.10805362 3.04872078
 1.15266687 0.13123342], Cost: 0.0104
Current parameters: [0.86110647 3.07976171 0.96148205 2.27532784 1.10252528 3.0417539
 1.17121028 0.11883526], Cost: 0.0222
Current parameters: [0.87047141 3.09501313 0.96749521 2.29425596 1.11264629 3.04766993
 1.15566976 0.13010564], Cost: 0.0101
Current parameters: [0.8554917  3.1064656  0.97613789 2.3069027  1.10000308 3.04466468
 1.1431974  0.12223581], Cost: 0.0153
Current parameters: [0.86272777 3.08757352 0.96814904 2.29569133 1.12052958 3.05435037
 1.15181158 0.12794209], Cost: 0.0081
Current parameters: [0.87270997 3.08937401 0.94671576 2.30820526 1.13233057 3.05584426
 1.14493995 0.11963676], Cost: 0.0187
Current parameters: [0.86055148 3.09126001 0.96179937 2.2961368  1.12045979 3.0493459
 1.15394312 0.14042025], Cost: 0.0069
Current parameters: [0.84039881 3.07558595 0.9637261  2.29490063 1.12261306 3.05334748
 1.13876865 0.14870288], Cost: 0.0072
Current parameters: [0.86017672 3.09057076 0.96598467 2.29528274 1.12834045 3.03690146
 1.15229819 0.13805287], Cost: 0.0068
```



```
Current parameters: [0.85582098 3.10179668 0.98190067 2.29131236 1.14408327 3.04848715
 1.16050562 0.1486858 ], Cost: 0.0204
Current parameters: [0.86171486 3.08296205 0.96978465 2.30614557 1.1266156  3.03535181
 1.15855094 0.14069257], Cost: 0.0072
Current parameters: [0.86133535 3.0861812  0.96376428 2.29113733 1.13053428 3.03752774
 1.15591458 0.13821558], Cost: 0.0066
Current parameters: [0.85239874 3.08675001 0.96072743 2.28916765 1.12286109 3.03205517
 1.16184568 0.13303413], Cost: 0.0088
Current parameters: [0.85931824 3.08711064 0.95955803 2.29467945 1.13475478 3.03893508
 1.15647068 0.13581537], Cost: 0.0068
Current parameters: [0.86947587 3.08182355 0.95554265 2.29213983 1.12764357 3.03345013
 1.14786793 0.13895811], Cost: 0.0123
Current parameters: [0.86629494 3.09026545 0.96324042 2.29143006 1.13010037 3.0364895
 1.15981668 0.13651153], Cost: 0.0076
Current parameters: [0.85562443 3.08871413 0.97167607 2.28832617 1.13759093 3.04279589
 1.15851096 0.14530927], Cost: 0.0074
Current parameters: [0.86184038 3.08420474 0.96746628 2.29067495 1.13091473 3.04032769
 1.15487132 0.13239473], Cost: 0.0069
Current parameters: [0.86137466 3.08538737 0.96506597 2.29183747 1.1322484  3.03446633
 1.15604578 0.1378767 ], Cost: 0.0065
Current parameters: [0.86193553 3.08184959 0.96638701 2.2970109  1.13026174 3.03593259
 1.15927533 0.13965669], Cost: 0.0066
Current parameters: [0.86334527 3.08149099 0.96330903 2.29189267 1.13302081 3.03412171
 1.15060298 0.14079914], Cost: 0.0076
Current parameters: [0.85848661 3.08478502 0.96458721 2.29113136 1.13050969 3.03318547
 1.15691894 0.13724331], Cost: 0.0065
Current parameters: [0.85740493 3.08474591 0.9654349  2.29089664 1.13132872 3.03384683
 1.15681904 0.13811008], Cost: 0.0059
Current parameters: [0.85574954 3.08568438 0.96731683 2.29005251 1.13278614 3.03467913
 1.15812156 0.13980424], Cost: 0.0050
Current parameters: [0.85375887 3.08479547 0.9684716  2.28939345 1.13418686 3.03550848
 1.15712547 0.14206167], Cost: 0.0046
Current parameters: [0.85170659 3.08590126 0.97005924 2.28832425 1.13487497 3.03568207
 1.15916888 0.14327872], Cost: 0.0042
Current parameters: [0.84851707 3.08459024 0.96964897 2.28760492 1.13357078 3.03470368
 1.15903182 0.14334412], Cost: 0.0036
Current parameters: [0.84622383 3.08376122 0.97031224 2.28646942 1.134491   3.03220086
 1.15853059 0.14381436], Cost: 0.0032
Current parameters: [0.84515984 3.08153495 0.97137059 2.28851118 1.13393313 3.03175287
 1.15995296 0.14497474], Cost: 0.0030
Current parameters: [0.84402665 3.08036499 0.97351827 2.28800679 1.13416806 3.03298061
```

```
 1.15962879 0.14251073], Cost: 0.0027
Current parameters: [0.84396237 3.07961695 0.97244909 2.28769394 1.13523399 3.03331481
 1.16044033 0.14218899], Cost: 0.0027
Current parameters: [0.84157011 3.08096605 0.97182448 2.28995288 1.13631977 3.03361395
 1.15980432 0.14144742], Cost: 0.0025
Current parameters: [0.84136185 3.08050874 0.97188833 2.28974449 1.13677247 3.03464474
 1.15878777 0.14254917], Cost: 0.0025
Current parameters: [0.8380528  3.08037021 0.97197945 2.28924575 1.13526746 3.03364195
 1.16056175 0.14087613], Cost: 0.0021
Current parameters: [0.83669767 3.07821857 0.97098757 2.28990151 1.13430304 3.03255913
 1.15886958 0.13932274], Cost: 0.0024
Current parameters: [0.83773398 3.08031534 0.97258109 2.28875918 1.1363831  3.03233741
 1.16027799 0.1411665 ], Cost: 0.0020
Current parameters: [0.83559223 3.08114009 0.97463658 2.28847896 1.13753215 3.03278415
 1.16231075 0.14135601], Cost: 0.0017
Current parameters: [0.83245177 3.07993921 0.97575772 2.28862497 1.13810233 3.03171132
 1.16245561 0.14027026], Cost: 0.0014
Current parameters: [0.83018405 3.08135004 0.97578881 2.28643745 1.13876302 3.03157277
 1.16183409 0.13868988], Cost: 0.0013
Current parameters: [0.8273246  3.08147813 0.97519084 2.28662291 1.13870294 3.02986815
 1.16298542 0.14025162], Cost: 0.0009
Current parameters: [0.82617788 3.08274503 0.97512419 2.28846668 1.13952077 3.02778751
 1.16435887 0.13881964], Cost: 0.0009
Current parameters: [0.82747302 3.08068645 0.97415798 2.28620501 1.13970452 3.03014434
 1.16385163 0.13994387], Cost: 0.0011
Current parameters: [0.8256307  3.08090612 0.97731293 2.28544167 1.13698478 3.02838759
 1.16345081 0.14114771], Cost: 0.0007
Current parameters: [0.82307353 3.08110249 0.97836353 2.28643332 1.13778355 3.02881386
 1.1617922  0.14288645], Cost: 0.0005
Current parameters: [0.82209415 3.08413278 0.97806688 2.2845434  1.1377409  3.02855247
 1.16148492 0.14402336], Cost: 0.0005
Current parameters: [0.82257039 3.08145891 0.97857561 2.2867749  1.1371884  3.03017443
 1.16280672 0.14269089], Cost: 0.0005
Current parameters: [0.82069356 3.08093737 0.97691382 2.28738353 1.13474439 3.02996303
 1.16137962 0.14216384], Cost: 0.0006
Current parameters: [0.82299467 3.08144962 0.97797792 2.2874666  1.1367931  3.02982228
 1.16343385 0.14414874], Cost: 0.0005
Current parameters: [0.82211379 3.08171958 0.98219353 2.28776129 1.13697433 3.03052162
 1.16204366 0.14310496], Cost: 0.0005
Current parameters: [0.82138647 3.08057913 0.97871303 2.28618825 1.13805593 3.03033142
 1.16344159 0.14300014], Cost: 0.0004
```



```
Current parameters: [0.82163613 3.08024063 0.97844166 2.28593868 1.13867237 3.03180719
 1.16261847 0.1435163 ], Cost: 0.0006
Current parameters: [0.82110153 3.08090941 0.97850809 2.28682952 1.13858876 3.03029549
 1.16334747 0.14287317], Cost: 0.0004
Current parameters: [0.82114972 3.08055401 0.97914274 2.28581772 1.13810424 3.02960719
 1.16484405 0.14202939], Cost: 0.0004
Current parameters: [0.82124946 3.07979404 0.9790902  2.28630766 1.13794114 3.02964245
 1.16480446 0.14172285], Cost: 0.0004
Current parameters: [0.81965379 3.08066044 0.9788524  2.28561145 1.13706072 3.02917719
 1.16444823 0.14193866], Cost: 0.0003
Current parameters: [0.81918561 3.08069656 0.98059058 2.2860428  1.13687603 3.02892409
 1.16419526 0.14241269], Cost: 0.0003
Current parameters: [0.81910903 3.08083724 0.98049857 2.28638551 1.13651923 3.02936712
 1.16478962 0.14276659], Cost: 0.0003
Current parameters: [0.81890993 3.08037524 0.98030635 2.28603877 1.13675877 3.02742538
 1.16433284 0.14354224], Cost: 0.0003
Current parameters: [0.81870306 3.08024564 0.98060387 2.28573791 1.13737049 3.02925395
 1.16433257 0.14264118], Cost: 0.0002
Current parameters: [0.81727127 3.08020928 0.9811929  2.28491827 1.13713669 3.02981819
 1.16389811 0.14221363], Cost: 0.0002
Current parameters: [0.81729802 3.08010811 0.98301321 2.28493695 1.13767219 3.03010332
 1.1638221  0.14255426], Cost: 0.0002
Current parameters: [0.81703172 3.08034487 0.98130519 2.28502892 1.13739755 3.02945863
 1.16452126 0.14169651], Cost: 0.0002
Current parameters: [0.81608964 3.08091827 0.9809268  2.2860917  1.13821373 3.02968545
 1.16374259 0.14173898], Cost: 0.0002
Current parameters: [0.81599965 3.08018941 0.98101364 2.28659183 1.13793718 3.02965472
 1.163642   0.14147607], Cost: 0.0002
Current parameters: [0.81508071 3.07947741 0.98040872 2.28583982 1.13831217 3.03049176
 1.16337938 0.14068764], Cost: 0.0002
Current parameters: [0.81492605 3.07990388 0.98098408 2.28638381 1.13759128 3.02844353
 1.1635936  0.14244945], Cost: 0.0001
Current parameters: [0.81599965 3.08018941 0.98101364 2.28659183 1.13793718 3.02965472
 1.163642   0.14147607], Cost: 0.0002

   Return from subroutine COBYLA because the MAXFUN limit has been reached.

   NFVALS =  100   F = 1.608518E-04    MAXCV = 0.000000E+00
   X = 8.159996E-01   3.080189E+00   9.810136E-01   2.286592E+00   1.137937E+00
       3.029655E+00   1.163642E+00   1.414761E-01
Simulating for QAOA depth p = 5
```



```
Current parameters: [1.72541295 1.79184308 1.83566324 0.33700772 0.67497813 0.6209824
 2.49343408 0.31948117 0.12658612 0.2094743 ], Cost: 0.7522
Current parameters: [1.72541295 2.79184308 1.83566324 0.33700772 0.67497813 0.6209824
 2.49343408 0.31948117 0.12658612 0.2094743 ], Cost: 2.8185
Current parameters: [1.72541295 1.79184308 2.83566324 0.33700772 0.67497813 0.6209824
 2.49343408 0.31948117 0.12658612 0.2094743 ], Cost: 1.8540
Current parameters: [1.72541295 1.79184308 1.83566324 1.33700772 0.67497813 0.6209824
 2.49343408 0.31948117 0.12658612 0.2094743 ], Cost: 2.0826
Current parameters: [1.72541295 1.79184308 1.83566324 0.33700772 1.67497813 0.6209824
 2.49343408 0.31948117 0.12658612 0.2094743 ], Cost: 1.9764
Current parameters: [1.72541295 1.79184308 1.83566324 0.33700772 0.67497813 1.6209824
 2.49343408 0.31948117 0.12658612 0.2094743 ], Cost: 1.7933
Current parameters: [1.72541295 1.79184308 1.83566324 0.33700772 0.67497813 0.6209824
 3.49343408 0.31948117 0.12658612 0.2094743 ], Cost: 2.5278
Current parameters: [1.72541295 1.79184308 1.83566324 0.33700772 0.67497813 0.6209824
 2.49343408 1.31948117 0.12658612 0.2094743 ], Cost: 2.7661
Current parameters: [1.72541295 1.79184308 1.83566324 0.33700772 0.67497813 0.6209824
 2.49343408 0.31948117 1.12658612 0.2094743 ], Cost: 1.9527
Current parameters: [1.72541295 1.79184308 1.83566324 0.33700772 0.67497813 0.6209824
 2.49343408 0.31948117 0.12658612 1.2094743 ], Cost: 1.8051
Current parameters: [ 1.8269215   1.32745918  1.5880445   0.03801187  0.39984268  0.38700627
  2.09438796 -0.13313273 -0.14321086 -0.02716496], Cost: 2.4171
Current parameters: [ 1.77616722  1.55965113  1.71185387  0.1875098   0.53741041  0.50399434
  2.29391102  0.09317422 -0.00831237  0.09115467], Cost: 1.6974
Current parameters: [1.7346397  2.02852771 1.81315561 0.30983011 0.64996934 0.59971484
 2.45716227 0.27834025 0.10206258 0.18796467], Cost: 0.8799
Current parameters: [1.84107192 1.79184308 1.88307635 0.33700772 0.67497813 0.6209824
 2.49343408 0.31948117 0.12658612 0.2094743 ], Cost: 0.7858
Current parameters: [1.62920361 1.79392546 2.06105327 0.3195178  0.65888394 0.60729584
 2.47009166 0.29300528 0.1108042  0.19563196], Cost: 1.0859
Current parameters: [1.72541295 1.79184308 1.83566324 0.42164966 0.58299584 0.6209824
 2.49343408 0.31948117 0.12658612 0.2094743 ], Cost: 0.7272
Current parameters: [1.72723787 1.79526692 1.81591743 0.60027681 0.741494   0.59847898
 2.4550545  0.27594954 0.10063751 0.18671474], Cost: 1.0169
Current parameters: [1.72541295 1.79184308 1.83566324 0.42164966 0.58299584 0.72881345
 2.43020857 0.31948117 0.12658612 0.2094743 ], Cost: 1.3782
Current parameters: [1.72753673 1.79582763 1.81268373 0.41759764 0.57243095 0.60974445
 2.73326162 0.26882052 0.096388   0.18298749], Cost: 1.2650
Current parameters: [1.72541295 1.79184308 1.83566324 0.42164966 0.58299584 0.6209824
 2.49343408 0.38348389 0.01921456 0.2094743 ], Cost: 0.8659
Current parameters: [1.72740344 1.79557756 1.81412592 0.41785194 0.573094   0.46797903
```



```
 2.47522648 0.47101479 0.24735666 0.18464978], Cost: 0.7551
Current parameters: [1.72541295 1.79184308 1.83566324 0.42164966 0.58299584 0.56794579
 2.49622128 0.2955857  0.11234236 0.3191578 ], Cost: 0.3158
Current parameters: [1.72877659 1.79815381 1.79926829 0.41523207 0.56626316 0.6523152
 2.44743163 0.33542245 0.18753346 0.5290716 ], Cost: 0.4394
Current parameters: [1.72895618 1.7756334  1.82701991 0.43076549 0.5119813  0.62163211
 2.45571455 0.25114829 0.15387614 0.27527083], Cost: 0.6543
Current parameters: [1.7435016  1.72424555 1.77184973 0.3502696  0.59315127 0.39747326
 2.43492212 0.22557123 0.03509036 0.38730909], Cost: 0.1897
Current parameters: [1.6967602  1.72660126 1.87886739 0.34483955 0.59399385 0.36484902
 2.42573583 0.21537983 0.0344886  0.41374319], Cost: 0.4390
Current parameters: [1.7416422  1.6674736  1.77638551 0.35615372 0.59618252 0.40375896
 2.45734575 0.23153002 0.04068402 0.38980458], Cost: 0.1861
Current parameters: [1.75205147 1.69945957 1.74159838 0.35358163 0.58352026 0.37646648
 2.56214742 0.20256287 0.04629092 0.41255651], Cost: 0.0842
Current parameters: [1.75101854 1.69896787 1.74411809 0.36662716 0.60539648 0.40455213
 2.562677   0.16495328 0.0154155  0.42219914], Cost: 0.1387
Current parameters: [1.76148718 1.70406304 1.71130767 0.44252219 0.63538562 0.32760029
 2.55494068 0.20695415 0.08546984 0.41091464], Cost: 0.3387
Current parameters: [1.69466021 1.69945957 1.71807148 0.35570461 0.58121316 0.37779674
 2.56207751 0.20637284 0.04126201 0.40980544], Cost: 0.0984
Current parameters: [ 1.7758915   1.7023207   1.72542341  0.36648633  0.5396936   0.37329065
  2.5825712   0.27107529 -0.04023645  0.42211563], Cost: 0.1004
Current parameters: [1.75281986 1.7009012  1.73328431 0.35759145 0.5737471  0.39230867
 2.55118222 0.21011728 0.06254609 0.46760516], Cost: 0.1451
Current parameters: [1.76626374 1.70350933 1.71124701 0.26017554 0.6440311  0.38905559
 2.58764035 0.23672694 0.04628772 0.40143954], Cost: 0.0923
Current parameters: [1.75710374 1.68029115 1.724149   0.33588557 0.57927421 0.33730042
 2.54226128 0.18002677 0.03021144 0.42603876], Cost: 0.1202
Current parameters: [1.77237694 1.69630721 1.71230412 0.32323383 0.50258298 0.4162841
 2.58105923 0.17461348 0.07320187 0.35404595], Cost: 0.0669
Current parameters: [1.75655361 1.70407563 1.75879573 0.30167074 0.4869925  0.40312185
 2.60446181 0.17347845 0.07277414 0.35214138], Cost: 0.0937
Current parameters: [1.77148387 1.66789787 1.71445664 0.32673028 0.50445586 0.41920644
 2.59202194 0.1776414  0.07619753 0.35524788], Cost: 0.0883
Current parameters: [1.78544462 1.72301796 1.69692502 0.3475681  0.51214543 0.39282235
 2.59555548 0.18394752 0.09264455 0.32444577], Cost: 0.0791
Current parameters: [1.76678436 1.6936143  1.7185206  0.3197175  0.51282024 0.41748249
 2.56951785 0.16186715 0.09483797 0.34992355], Cost: 0.0433
Current parameters: [1.76805845 1.69104961 1.73373047 0.31721682 0.51190565 0.42778907
 2.52423425 0.1864199  0.09201691 0.32005731], Cost: 0.0378
```

```
Current parameters: [1.77041753 1.69186358 1.72694503 0.29516046 0.52873595 0.43114998
 2.5314252  0.19167845 0.08850879 0.31287499], Cost: 0.0558
Current parameters: [1.77283614 1.70885611 1.72826155 0.31323063 0.49971472 0.44383228
 2.50604669 0.19952564 0.13165965 0.35231341], Cost: 0.0473
Current parameters: [1.76566497 1.69891699 1.74170871 0.32819409 0.5196973  0.451868
 2.53268226 0.18548075 0.090235   0.31708162], Cost: 0.0708
Current parameters: [1.7757751  1.69076938 1.72567585 0.31460467 0.50518857 0.40421887
 2.48915289 0.14249738 0.08825288 0.32049726], Cost: 0.0537
Current parameters: [1.78934283 1.6928187  1.75475292 0.31194195 0.51062192 0.42371308
 2.52918465 0.18490552 0.09373238 0.32168092], Cost: 0.0295
Current parameters: [1.75199226 1.70438244 1.78470506 0.29475448 0.49065594 0.39968428
 2.53686006 0.18751507 0.10378296 0.31518748], Cost: 0.0130
Current parameters: [1.74555864 1.70435614 1.79047976 0.30436405 0.50888294 0.38764657
 2.5298365  0.19642237 0.09805316 0.32825837], Cost: 0.0086
Current parameters: [1.76645831 1.72732118 1.81924191 0.27718158 0.52952383 0.38408072
 2.53556793 0.17534834 0.10977452 0.34622491], Cost: 0.0140
Current parameters: [1.75310394 1.71256599 1.7866789  0.31475681 0.51282018 0.37188022
 2.53508595 0.20104304 0.11338576 0.31477885], Cost: 0.0288
Current parameters: [1.74335036 1.69292475 1.79336418 0.30447932 0.51301344 0.38913176
 2.53002461 0.19326567 0.10642613 0.32731994], Cost: 0.0095
Current parameters: [1.75095551 1.69018471 1.79853551 0.29708138 0.49461614 0.37991818
 2.53069463 0.20592653 0.08884819 0.3420282 ], Cost: 0.0207
Current parameters: [1.74445486 1.7074049  1.78600613 0.30305965 0.50511197 0.38910077
 2.52227674 0.19921963 0.1070938  0.33524907], Cost: 0.0189
Current parameters: [1.74251664 1.70197989 1.78930053 0.30598527 0.50619731 0.37652137
 2.51406124 0.17471554 0.08870322 0.32387729], Cost: 0.0106
Current parameters: [1.74856963 1.70409411 1.79576182 0.30068901 0.510504   0.39014229
 2.52058315 0.20265087 0.09716938 0.32054191], Cost: 0.0135
Current parameters: [1.73484849 1.70925472 1.80561206 0.32011186 0.50762401 0.40262861
 2.53590698 0.19020499 0.09047084 0.32961271], Cost: 0.0031
Current parameters: [1.72303578 1.71188825 1.80862763 0.31346334 0.50555036 0.39797533
 2.5380868  0.19184912 0.09209977 0.32673046], Cost: 0.0022
Current parameters: [1.71459452 1.70925238 1.79728418 0.3060668  0.52215281 0.40773755
 2.54745477 0.18816177 0.07652379 0.32646985], Cost: 0.0174
Current parameters: [1.71822702 1.7068114  1.80141855 0.32122781 0.50128848 0.39734333
 2.53684076 0.19766239 0.09014766 0.32170417], Cost: 0.0049
Current parameters: [1.72986229 1.72044299 1.81778564 0.32573317 0.50939793 0.38227473
 2.55282348 0.18842479 0.09823565 0.31700924], Cost: 0.0041
Current parameters: [1.72602183 1.70769004 1.81037008 0.31103897 0.50038571 0.39236691
 2.54386066 0.19593214 0.08445171 0.33321554], Cost: 0.0022
Current parameters: [1.72377746 1.69505193 1.83381507 0.31316254 0.49004054 0.39817791
```



```
 2.53729641 0.18835436 0.0884717  0.33281044], Cost: 0.0061
Current parameters: [1.72028927 1.71598972 1.81113693 0.30624386 0.49999696 0.39359165
 2.53935855 0.19417343 0.09250866 0.33859002], Cost: 0.0017
Current parameters: [1.72454872 1.71978584 1.80560605 0.32345068 0.48967801 0.38784734
 2.54344752 0.19909901 0.09438658 0.35940729], Cost: 0.0179
Current parameters: [1.72402732 1.72132916 1.81632835 0.30387017 0.50195862 0.39341529
 2.53300025 0.20426211 0.0893204  0.33500966], Cost: 0.0035
Current parameters: [1.72122276 1.71568064 1.81134742 0.30521317 0.49887724 0.39779637
 2.54369887 0.19747455 0.095671   0.33804957], Cost: 0.0014
Current parameters: [1.72553027 1.7206433  1.80794943 0.30043413 0.48893208 0.39884043
 2.54374218 0.19222237 0.09356054 0.33217293], Cost: 0.0012
Current parameters: [1.72821885 1.71740934 1.8064057  0.298459   0.48827398 0.39513164
 2.541928   0.1933915  0.09781078 0.33178165], Cost: 0.0028
Current parameters: [1.72243289 1.72026854 1.80727697 0.29114587 0.4972632  0.40282205
 2.54792457 0.18700606 0.09048455 0.3292993 ], Cost: 0.0035
Current parameters: [1.72479126 1.7171127  1.80664633 0.29850674 0.48693897 0.4025728
 2.54029444 0.19316117 0.09144239 0.33490295], Cost: 0.0011
Current parameters: [1.72293011 1.71301958 1.8192355  0.30000208 0.48298337 0.4050467
 2.53886008 0.18820387 0.09397228 0.33200459], Cost: 0.0014
Current parameters: [1.72979415 1.71793437 1.80886656 0.29730646 0.48745627 0.40235274
 2.5416813  0.19163926 0.09049659 0.33975175], Cost: 0.0014
Current parameters: [1.71888446 1.72149989 1.80440252 0.30741399 0.47995231 0.40562032
 2.5445265  0.19354167 0.08924316 0.3398665 ], Cost: 0.0017
Current parameters: [1.72250026 1.71746378 1.80908466 0.29511584 0.48486724 0.39914008
 2.54188292 0.19674052 0.08889983 0.33548978], Cost: 0.0015
Current parameters: [1.72861283 1.72459966 1.81013617 0.3011633  0.48900758 0.40779403
 2.53348014 0.20048688 0.08871976 0.33100531], Cost: 0.0024
Current parameters: [1.72757864 1.71396806 1.80650243 0.30137092 0.48691032 0.40196389
 2.54301227 0.1937416  0.08720344 0.33188817], Cost: 0.0018
Current parameters: [1.72173861 1.70685192 1.80268665 0.29588999 0.48727941 0.40792847
 2.53500075 0.19588241 0.09333873 0.34116301], Cost: 0.0012
Current parameters: [1.72322958 1.71731847 1.8061705  0.29973777 0.487833   0.39909451
 2.53566056 0.189788   0.088302   0.33599895], Cost: 0.0003
Current parameters: [1.72189027 1.71716196 1.80726127 0.3007565  0.49089387 0.39898126
 2.53666431 0.19032506 0.0883844  0.33672592], Cost: 0.0001
Current parameters: [1.71873284 1.71848886 1.80597645 0.29766038 0.49351029 0.40101495
 2.53709591 0.18567555 0.08619885 0.33601463], Cost: 0.0011
Current parameters: [1.72323462 1.71754868 1.80813995 0.29943734 0.49244185 0.39964549
 2.53466648 0.1915946  0.08787567 0.33528009], Cost: 0.0003
Current parameters: [1.72025556 1.72032916 1.80760174 0.30597356 0.48977634 0.4000343
 2.53392005 0.19239065 0.08779452 0.3392744 ], Cost: 0.0002
```



```
Current parameters: [1.72228285 1.71723179 1.8040793  0.3002035  0.49183727 0.39858533
 2.53676299 0.19177463 0.08784997 0.33783304], Cost: 0.0001
Current parameters: [1.72041726 1.71086702 1.80782934 0.30070816 0.49069438 0.3997004
 2.53379207 0.19070969 0.08972803 0.33958144], Cost: 0.0002
Current parameters: [1.72213657 1.71741882 1.80711383 0.30159338 0.49158905 0.39610543
 2.5362244  0.18985732 0.09063719 0.3363148 ], Cost: 0.0001
Current parameters: [1.72274488 1.71667175 1.80672486 0.30185464 0.49132967 0.39962914
 2.53668655 0.18964231 0.08836351 0.33620285], Cost: 0.0002
Current parameters: [1.72170279 1.71601618 1.807954   0.30163398 0.49044191 0.39696239
 2.53762057 0.19132992 0.085945   0.33601456], Cost: 0.0001
Current parameters: [1.72292499 1.71737435 1.80774041 0.30058689 0.49109779 0.39861002
 2.53656537 0.18965691 0.0878733  0.3379684 ], Cost: 0.0000
Current parameters: [1.7239198  1.71740322 1.808929   0.30056327 0.49074218 0.39904736
 2.5391042  0.19134926 0.08930578 0.33905263], Cost: 0.0001
Current parameters: [1.72203219 1.71601563 1.80801594 0.30028572 0.49092791 0.39894772
 2.53605478 0.18986747 0.08839173 0.33850521], Cost: 0.0000
Current parameters: [1.72433854 1.7144872  1.80779745 0.30136915 0.49137065 0.39760244
 2.5348704  0.18960206 0.08764735 0.34003779], Cost: 0.0000
Current parameters: [1.72242571 1.71524585 1.80781891 0.29912886 0.49139056 0.39865646
 2.53681606 0.18911711 0.0883968  0.33800657], Cost: 0.0001
Current parameters: [1.7199472  1.71701665 1.80842333 0.30050887 0.49179375 0.39821767
 2.5362943  0.18819    0.08726433 0.34057133], Cost: 0.0001
Current parameters: [1.72211747 1.71636546 1.80888371 0.29962021 0.4919454  0.39902318
 2.53513763 0.19053992 0.08810267 0.33823829], Cost: 0.0001
Current parameters: [1.72213754 1.71595714 1.80875128 0.30050503 0.49067178 0.39897571
 2.53600973 0.18940446 0.08845813 0.33825395], Cost: 0.0000
Current parameters: [1.72208217 1.71639788 1.80756067 0.29942612 0.48946311 0.39864915
 2.5354351  0.1898666  0.0882167  0.33877067], Cost: 0.0001
Current parameters: [1.72228741 1.71627545 1.80787467 0.30017264 0.49111011 0.39935002
 2.53582764 0.18949425 0.08896419 0.33877843], Cost: 0.0000
Current parameters: [1.72236308 1.71610522 1.80854194 0.3002671  0.49068423 0.39894702
 2.53726831 0.19078719 0.08885721 0.33941382], Cost: 0.0000
Current parameters: [1.72248185 1.71602148 1.8085624  0.30019755 0.49062109 0.39965083
 2.53737399 0.19069763 0.08824881 0.33961035], Cost: 0.0000
Current parameters: [1.72127012 1.71651975 1.80852639 0.30050975 0.49112868 0.398387
 2.53765224 0.19012515 0.08844259 0.34047232], Cost: 0.0000
Current parameters: [1.72236308 1.71610522 1.80854194 0.3002671  0.49068423 0.39894702
 2.53726831 0.19078719 0.08885721 0.33941382], Cost: 0.0000

   Return from subroutine COBYLA because the MAXFUN limit has been reached.
```



```
   NFVALS =  100   F = 2.104206E-05    MAXCV = 0.000000E+00
   X = 1.722363E+00   1.716105E+00   1.808542E+00   3.002671E-01   4.906842E-01
       3.989470E-01   2.537268E+00   1.907872E-01   8.885721E-02   3.394138E-01
Simulating for QAOA depth p = 6
Current parameters: [2.43183132 2.02695233 2.02115633 2.79615025 0.60574902 2.94370556
 2.93023011 0.82668125 1.64161156 0.11473273 0.52009753 0.91016907], Cost: 3.3175
Current parameters: [1.43183132 3.02695233 2.02115633 2.79615025 0.60574902 2.94370556
 2.93023011 0.82668125 1.64161156 0.11473273 0.52009753 0.91016907], Cost: 2.6818
Current parameters: [1.43183132 3.02695233 3.02115633 2.79615025 0.60574902 2.94370556
 2.93023011 0.82668125 1.64161156 0.11473273 0.52009753 0.91016907], Cost: 2.4894
Current parameters: [1.43183132 3.02695233 3.02115633 3.79615025 0.60574902 2.94370556
 2.93023011 0.82668125 1.64161156 0.11473273 0.52009753 0.91016907], Cost: 1.9366
Current parameters: [1.43183132 3.02695233 3.02115633 3.79615025 1.60574902 2.94370556
 2.93023011 0.82668125 1.64161156 0.11473273 0.52009753 0.91016907], Cost: 2.7432
Current parameters: [1.43183132 3.02695233 3.02115633 3.79615025 0.60574902 3.94370556
 2.93023011 0.82668125 1.64161156 0.11473273 0.52009753 0.91016907], Cost: 2.1982
Current parameters: [1.43183132 3.02695233 3.02115633 3.79615025 0.60574902 2.94370556
 3.93023011 0.82668125 1.64161156 0.11473273 0.52009753 0.91016907], Cost: 2.3502
Current parameters: [1.43183132 3.02695233 3.02115633 3.79615025 0.60574902 2.94370556
 2.93023011 1.82668125 1.64161156 0.11473273 0.52009753 0.91016907], Cost: 2.4233
Current parameters: [1.43183132 3.02695233 3.02115633 3.79615025 0.60574902 2.94370556
 2.93023011 0.82668125 2.64161156 0.11473273 0.52009753 0.91016907], Cost: 2.4066
Current parameters: [1.43183132 3.02695233 3.02115633 3.79615025 0.60574902 2.94370556
 2.93023011 0.82668125 1.64161156 1.11473273 0.52009753 0.91016907], Cost: 2.1458
Current parameters: [1.43183132 3.02695233 3.02115633 3.79615025 0.60574902 2.94370556
 2.93023011 0.82668125 1.64161156 0.11473273 1.52009753 0.91016907], Cost: 1.6071
Current parameters: [1.43183132 3.02695233 3.02115633 3.79615025 0.60574902 2.94370556
 2.93023011 0.82668125 1.64161156 0.11473273 1.52009753 1.91016907], Cost: 3.3026
Current parameters: [1.34088249 3.2218161  3.10767442 4.04470864 0.24306167 2.8260697
 2.74426292 0.60784097 1.43028532 0.02067014 1.66823884 0.147816  ], Cost: 1.2629
Current parameters: [ 1.29911041  3.31131549  3.1474115   4.15886954  1.14683125  2.77204047
  2.65884965  0.50732935  1.33322484 -0.02253207  1.73627897 -0.20232676], Cost: 1.8892
Current parameters: [1.79396301 3.4332825  3.10767442 4.04470864 0.24306167 2.8260697
 2.74426292 0.60784097 1.43028532 0.02067014 1.66823884 0.147816  ], Cost: 1.2379
Current parameters: [ 2.20830602  2.55959285  3.13053535  4.11038594  0.15599815  2.79498643
  2.69512427  0.55001616  1.37444597 -0.0041843   1.70738264 -0.05362278], Cost: 2.3998
Current parameters: [1.79669297 3.42743338 2.60912403 4.04470864 0.25394826 2.8260697
 2.74426292 0.60784097 1.43028532 0.02067014 1.6936093  0.12244553], Cost: 0.5179
Current parameters: [ 1.58799978  3.91661183  2.07704675  4.2409851  -0.17343854  2.73317743
  2.59741218  0.43503168  1.26340957 -0.05360728  1.42094763 -0.08991089], Cost: 1.4343
Current parameters: [1.78392581 3.45894766 2.56485915 3.5601229  0.24309409 2.81687808
```

```
 2.72973216 0.59074163 1.4137731  0.01332045 1.73988774 0.02817494], Cost: 0.3252
Current parameters: [1.78392581 3.45894766 2.56485915 3.5601229  0.24309409 2.81687808
 2.72973216 0.76440394 1.23393594 0.01332045 1.73988774 0.02817494], Cost: 0.4329
Current parameters: [ 1.76755948  3.49934605  2.50811564  3.55757007  0.22918005  2.80509528
  2.71110506  0.91893927  1.75767523  0.00389882  1.79921244 -0.09267128], Cost: 1.0450
Current parameters: [ 1.78122988  3.46472386  2.56742374  3.56749073  0.23234323  2.81687808
  2.96404257  0.58425471  1.40750891  0.01332045  1.71483357 -0.05351057], Cost: 0.9751
Current parameters: [ 1.77379411  3.49022051  2.44478811  3.50300268  0.29480215  2.79574291
  2.6649487   0.50474443  1.37911168 -0.00357941  2.02496537  0.3939214 ], Cost: 0.8118
Current parameters: [1.78213675 3.46278084 2.56656105 3.56501232 0.32077864 2.58032876
 2.72607398 0.5864368  1.40961608 0.01332045 1.72607668 0.01317862], Cost: 0.3698
Current parameters: [ 1.76053331  3.5139068   2.52148875  3.58114593  0.63851558  2.97177622
  2.57033121  0.50328044  1.35379319  0.00477036  1.72903271 -0.13587105], Cost: 1.5557
Current parameters: [ 1.80042257  3.42360227  2.5647346   3.55976509  0.2439477   2.81704742
  2.72999986  0.59105666  1.41407731 -0.23333862  1.72824751  0.02927238], Cost: 0.8879
Current parameters: [ 1.94803629  3.11310571  2.49721665  3.5395875   0.06015209  2.76708117
  2.57362882  0.52653022  1.3809804   0.18491996  1.72027624 -0.02766997], Cost: 1.4519
Current parameters: [1.81232844 3.39809317 2.55288229 3.5093214  0.32680545 2.84092104
 2.76774098 0.63546921 1.45696492 0.03254539 1.58115659 0.15780593], Cost: 0.5610
Current parameters: [1.64267993 3.76624663 2.48498072 3.55789078 0.01909592 2.76980486
 2.59273856 0.52615874 1.37503267 0.05933308 1.52111756 0.07740659], Cost: 1.2141
Current parameters: [1.94252806 3.54568508 2.73188463 3.54019143 0.23757462 2.81534943
 2.71306348 0.59313352 1.42146351 0.0311503  1.75817693 0.05051913], Cost: 0.2717
Current parameters: [1.8723379  3.50956835 2.80987391 3.52779037 0.19658886 2.79933427
 2.72234175 0.60405189 1.432007   0.03127758 1.76551602 0.0796936 ], Cost: 0.2163
Current parameters: [1.78648644 3.51823424 2.89554665 3.52722138 0.35984127 2.86456787
 2.6367195  0.56325629 1.40727265 0.07930648 1.7851466  0.01255089], Cost: 0.5098
Current parameters: [1.92082898 3.40568312 2.81458608 3.52656758 0.20780151 2.79984289
 2.72325662 0.60512847 1.43304662 0.04744066 1.80615705 0.05930295], Cost: 0.3619
Current parameters: [1.87356549 3.52771763 2.79540464 3.51823545 0.04071493 2.7645814
 2.5926336  0.54678333 1.40002194 0.12073664 1.68093315 0.07075648], Cost: 0.7752
Current parameters: [1.87083586 3.51254507 2.81457778 3.53325698 0.18827328 2.79986791
 2.70130247 0.67807796 1.50349133 0.03170428 1.7419818  0.01719659], Cost: 0.3150
Current parameters: [1.84767315 3.58041031 2.75879569 3.5128215  0.20589964 2.78807176
 2.71460019 0.6613063  1.50313737 0.07288147 1.92956728 0.20925151], Cost: 1.2164
Current parameters: [1.86898295 3.51778525 2.79911534 3.4084936  0.19311643 2.79706121
 2.73592721 0.59934766 1.42746429 0.02946002 1.77512369 0.05039178], Cost: 0.2209
Current parameters: [ 1.86058136  3.54327594  2.84916314  3.5717031   0.17612536  2.80466244
  2.93045463  0.56247131  1.40527735  0.07842086  1.72264779 -0.00686218], Cost: 0.8259
Current parameters: [ 1.8771032   3.50358368  2.8257322   3.52739637  0.19216385  2.80164705
  2.70679821  0.58690131  1.42186561 -0.08896065  1.75223145  0.07549818], Cost: 0.5848
```



```
Current parameters: [1.87528798 3.5032476  2.80862992 3.52251379 0.20528367 2.80183153
 2.7262896  0.5692064  1.47738854 0.03327441 1.74902917 0.09315793], Cost: 0.2352
Current parameters: [1.88376056 3.48694598 2.81126373 3.50399826 0.23350767 2.81152612
 2.73320261 0.65543164 1.40819077 0.04614896 1.68839439 0.13808162], Cost: 0.2455
Current parameters: [1.87268562 3.50881746 2.80957298 3.52744689 0.17695482 2.85828199
 2.72770726 0.6049059  1.4329835  0.03183253 1.76859328 0.08187975], Cost: 0.2174
Current parameters: [1.86679971 3.52561848 2.82897761 3.54847697 0.19217277 2.79169064
 2.82630239 0.58909806 1.41502601 0.0608307  1.73995252 0.0394538 ], Cost: 0.4495
Current parameters: [1.90771668 3.53135434 2.8542971  3.52292576 0.20257323 2.80189013
 2.71445302 0.60280213 1.4327643  0.03776143 1.77083578 0.08210721], Cost: 0.2326
Current parameters: [1.81400675 3.50920962 2.83263481 3.52957169 0.27356857 2.83101108
 2.67964582 0.58002396 1.41161954 0.05866104 1.76407088 0.04499568], Cost: 0.3043
Current parameters: [1.87469861 3.5084793  2.80534658 3.52565445 0.16104516 2.79162993
 2.69210656 0.58833442 1.4184881  0.05696629 1.74233437 0.07646013], Cost: 0.2292
Current parameters: [1.8616983  3.52858598 2.7948788  3.52322318 0.1926634  2.79513304
 2.71509933 0.61735713 1.44165559 0.0555391  1.79728217 0.11479801], Cost: 0.1863
Current parameters: [1.8570706  3.53454135 2.79302339 3.52077308 0.18662737 2.79357107
 2.70443877 0.61658246 1.43825062 0.03216753 1.80100429 0.12841549], Cost: 0.1351
Current parameters: [1.84843145 3.54733668 2.79142834 3.52075485 0.17647583 2.78939587
 2.70753803 0.61417622 1.43181217 0.0138084  1.80465803 0.14283491], Cost: 0.1050
Current parameters: [ 1.85179923e+00  3.53405470e+00  2.78907180e+00  3.51774388e+00
  1.71092901e-01  2.78699279e+00  2.69757978e+00  6.14258372e-01
  1.42787954e+00 -1.46973757e-03  1.81908430e+00  1.56372338e-01], Cost: 0.0975
Current parameters: [ 1.85680053  3.53420493  2.78743752  3.51801646  0.16334808  2.78412223
  2.6997678   0.62273951  1.43551142 -0.00392448  1.81719895  0.15447638], Cost: 0.1144
Current parameters: [ 1.83353757  3.54470222  2.79131034  3.51667551  0.17946557  2.79152992
  2.6790725   0.61668612  1.42842133 -0.01062367  1.81948908  0.15681923], Cost: 0.0800
Current parameters: [ 1.84703017  3.55765527  2.77133442  3.51871354  0.1882763   2.79509829
  2.67362907  0.61126207  1.4220139  -0.0163679   1.81967389  0.1570214 ], Cost: 0.0625
Current parameters: [ 1.84766448  3.55620461  2.77268916  3.53298874  0.18941576  2.79561352
  2.67216094  0.61281153  1.42213874 -0.01585784  1.81700364  0.16176388], Cost: 0.0607
Current parameters: [ 1.84856651  3.55342341  2.77180297  3.52233936  0.19780935  2.79838197
  2.67349864  0.62335944  1.41423902 -0.0155002   1.79957588  0.17898   ], Cost: 0.0619
Current parameters: [ 1.84144688  3.55104758  2.76407435  3.53304857  0.18970186  2.79553719
  2.67429912  0.60736027  1.42940306 -0.01675964  1.81330513  0.16350466], Cost: 0.0679
Current parameters: [ 1.8577772   3.56555833  2.78606299  3.52943586  0.19332678  2.79703936
  2.66775729  0.59703852  1.43510797 -0.01827022  1.81549538  0.17331882], Cost: 0.0664
Current parameters: [ 1.84762947  3.5563678   2.77266517  3.5330276   0.19352829  2.78076648
  2.67016426  0.61225546  1.42160021 -0.01543476  1.81573087  0.16114885], Cost: 0.0617
Current parameters: [ 1.84731548  3.55779002  2.76984651  3.53202605  0.17043125  2.79436291
  2.65608455  0.60443286  1.4130503  -0.00594655  1.80740517  0.16291633], Cost: 0.0785
```



```
Current parameters: [ 1.84965184  3.55231372  2.77617229  3.53377406  0.1895162   2.79643706
  2.6724158   0.60936701  1.41922523 -0.02534889  1.80959439  0.15496539], Cost: 0.0754
Current parameters: [ 1.83876513  3.56925982  2.76959192  3.53683433  0.19872996  2.79725121
  2.67817504  0.61224991  1.40861414 -0.01586171  1.83416667  0.17172071], Cost: 0.0394
Current parameters: [ 1.84193336  3.56378132  2.76696524  3.53706705  0.21841567  2.80434032
  2.66564653  0.61645882  1.40681611 -0.00438633  1.84754718  0.16994843], Cost: 0.0430
Current parameters: [ 1.83604703  3.5757138   2.7710935   3.53824622  0.203021    2.79904086
  2.6825685   0.61038946  1.40889658 -0.0078738   1.8275263   0.16551668], Cost: 0.0399
Current parameters: [ 1.83875368  3.58986764  2.76275555  3.5398761   0.19700411  2.79611821
  2.67915651  0.631281    1.41635819 -0.02020098  1.84047729  0.17486091], Cost: 0.0552
Current parameters: [ 1.8486681   3.5661226   2.7688309   3.54061385  0.19502412  2.79837301
  2.68592451  0.61455098  1.40853902 -0.01001037  1.83673821  0.17213065], Cost: 0.0499
Current parameters: [ 1.83203583  3.57334156  2.76352928  3.52586093  0.20866085  2.78679356
  2.68494539  0.59636766  1.39631503 -0.01986227  1.83753421  0.180251  ], Cost: 0.0262
Current parameters: [ 1.81208553  3.5674551   2.78230261  3.52249456  0.20762994  2.78304282
  2.69033503  0.59881228  1.39549633 -0.01483214  1.84510971  0.18707827], Cost: 0.0294
Current parameters: [ 1.82960289  3.57504639  2.76267209  3.52761469  0.1993566   2.78816908
  2.67560705  0.59408237  1.39053467 -0.01670606  1.837046    0.18289744], Cost: 0.0280
Current parameters: [ 1.83266544e+00  3.58446460e+00  2.75576097e+00  3.51859471e+00
  2.20262500e-01  2.78013422e+00  2.69267200e+00  5.86335544e-01
  1.38700299e+00 -2.20829554e-03  1.83642262e+00  1.80881929e-01], Cost: 0.0184
Current parameters: [ 1.83072583  3.58914164  2.76002755  3.52348486  0.23205029  2.78430415
  2.69621775  0.57792143  1.36608209 -0.01448553  1.83472107  0.17206214], Cost: 0.0303
Current parameters: [ 1.82569807e+00  3.57849600e+00  2.74641690e+00  3.51982417e+00
  2.18902053e-01  2.77892217e+00  2.69555122e+00  5.91473237e-01
  1.38358392e+00 -1.38961523e-03  1.83580436e+00  1.76365226e-01], Cost: 0.0164
Current parameters: [ 1.81514751e+00  3.57654782e+00  2.73434500e+00  3.52742674e+00
  2.23357705e-01  2.78699432e+00  2.69836566e+00  5.74634300e-01
  1.39718067e+00 -5.12308525e-04  1.83484210e+00  1.86105465e-01], Cost: 0.0186
Current parameters: [ 1.82495449e+00  3.58054180e+00  2.74622956e+00  3.51905324e+00
  2.14942171e-01  2.77680775e+00  2.69557502e+00  5.86009000e-01
  1.38693422e+00 -1.67267579e-03  1.84497553e+00  1.66703543e-01], Cost: 0.0162
Current parameters: [ 1.82345055e+00  3.57979313e+00  2.74287769e+00  3.49366856e+00
  2.11135081e-01  2.79381464e+00  2.69815788e+00  5.85672106e-01
  1.38721743e+00 -2.48083126e-03  1.84303918e+00  1.64934977e-01], Cost: 0.0200
Current parameters: [1.82743788e+00 3.56997417e+00 2.74917734e+00 3.51716813e+00
 2.15477225e-01 2.77376466e+00 2.69316019e+00 5.79337759e-01
 1.38695333e+00 9.61732894e-04 1.83917999e+00 1.63009876e-01], Cost: 0.0222
Current parameters: [ 1.81808282  3.59271364  2.73659177  3.50950084  0.21944494  2.7575894
  2.68807929  0.59305875  1.39511287 -0.00635116  1.84251482  0.16929493], Cost: 0.0191
Current parameters: [ 1.82478551  3.58389768  2.74723321  3.51942223  0.20613864  2.77341517
```



```
  2.70452296  0.58324252  1.38710135 -0.00452199  1.83813736  0.16696592], Cost: 0.0177
Current parameters: [1.83749313 3.57717095 2.73591808 3.51954807 0.21146792 2.77238482
 2.70597572 0.59172889 1.38331506 0.00478946 1.86274875 0.17927899], Cost: 0.0309
Current parameters: [1.82360483 3.58604963 2.74727654 3.52320362 0.2159942  2.78098144
 2.69617124 0.5916188  1.39135029 0.00701702 1.84135713 0.16077025], Cost: 0.0196
Current parameters: [ 1.82203812e+00  3.57887585e+00  2.75002001e+00  3.51784159e+00
  2.17843017e-01  2.77565008e+00  2.69968608e+00  5.87225256e-01
  1.38870312e+00 -1.78274894e-03  1.84655239e+00  1.67098522e-01], Cost: 0.0180
Current parameters: [1.81598037e+00 3.58178514e+00 2.75017174e+00 3.51885288e+00
 2.08688957e-01 2.77622947e+00 2.69045535e+00 5.84606888e-01
 1.38163019e+00 3.45348073e-03 1.84719817e+00 1.71121081e-01], Cost: 0.0143
Current parameters: [1.81585586 3.58453152 2.74921044 3.51806793 0.21243107 2.77547049
 2.69240082 0.58159697 1.37717114 0.00531026 1.84676696 0.17012297], Cost: 0.0120
Current parameters: [ 1.81140461  3.58846188  2.74922407  3.52241977  0.21475059  2.7792662
  2.68949736  0.58093041  1.36931387 -0.00422412  1.84717078  0.16721187], Cost: 0.0135
Current parameters: [1.81884406 3.58453235 2.75179875 3.51631913 0.2111226  2.7733831
 2.692179   0.58585851 1.37356566 0.00539435 1.84747983 0.16798891], Cost: 0.0118
Current parameters: [1.82251566 3.58635027 2.74457662 3.51830532 0.20997269 2.77280859
 2.69548877 0.58525172 1.36773442 0.00964078 1.85564127 0.17428426], Cost: 0.0098
Current parameters: [1.82163689 3.58788786 2.74136513 3.50588661 0.20820531 2.781161
 2.69650259 0.58514097 1.36618162 0.00978403 1.85596516 0.17433825], Cost: 0.0106
Current parameters: [1.81894476 3.58476065 2.73970301 3.51741837 0.21005714 2.76957394
 2.69550654 0.58781671 1.36740814 0.00954693 1.85463214 0.17254214], Cost: 0.0094
Current parameters: [1.82016896 3.59417677 2.7385204  3.51290352 0.21299727 2.76146245
 2.68985957 0.5866018  1.36939233 0.00880482 1.85508076 0.17698086], Cost: 0.0125
Current parameters: [1.82005642 3.58006428 2.73994366 3.5153207  0.20993921 2.76727688
 2.69452047 0.58408832 1.36565961 0.00675386 1.85490936 0.17402075], Cost: 0.0101
Current parameters: [1.81987696 3.58259363 2.73686835 3.52077791 0.21064699 2.77095841
 2.69409039 0.58449254 1.36159675 0.02110525 1.85153955 0.16734577], Cost: 0.0073
Current parameters: [1.81975291 3.58644797 2.7361993  3.5221445  0.20173273 2.76804044
 2.70298686 0.58102126 1.35913641 0.02069846 1.84509411 0.1672182 ], Cost: 0.0067
Current parameters: [1.81921654 3.58737488 2.73632046 3.52169103 0.19992026 2.76691937
 2.70323157 0.57836241 1.36090905 0.02055092 1.84976164 0.16241748], Cost: 0.0067
Current parameters: [1.8135066  3.58429371 2.74021214 3.52219922 0.20722499 2.76656656
 2.71270965 0.58191877 1.35589628 0.02664131 1.84835868 0.16769668], Cost: 0.0073
Current parameters: [1.82451673 3.58524165 2.73450679 3.52090661 0.20407115 2.7671099
 2.70608246 0.58134716 1.36280255 0.02157833 1.84404497 0.16614564], Cost: 0.0078
Current parameters: [1.82006148 3.58916495 2.73127999 3.52871558 0.20526014 2.77263149
 2.70420403 0.5807923  1.35067595 0.01482449 1.84516855 0.16191328], Cost: 0.0063
Current parameters: [1.82305214 3.58902614 2.73391384 3.52700896 0.20376308 2.77058539
 2.70388358 0.58520137 1.34730187 0.01481405 1.84590242 0.15983273], Cost: 0.0069
```

```
Current parameters: [1.81662378 3.5852376  2.72659118 3.53646275 0.19659422 2.7773325
 2.70226127 0.58276194 1.35035017 0.01958678 1.8473077  0.16355105], Cost: 0.0036
Current parameters: [1.81222925 3.59080164 2.72055812 3.54207425 0.19236464 2.77601962
 2.69651575 0.58079881 1.34716915 0.02673098 1.8478802  0.16638289], Cost: 0.0035
Current parameters: [1.81199601 3.58977719 2.72329316 3.54745124 0.19337887 2.77227092
 2.69547335 0.58095531 1.34889816 0.02592691 1.84631243 0.16526382], Cost: 0.0036
Current parameters: [1.80510148 3.58713659 2.72232881 3.54200812 0.18821622 2.78385043
 2.69415441 0.58098074 1.348333   0.02800819 1.84104179 0.15983508], Cost: 0.0055
Current parameters: [1.81222925 3.59080164 2.72055812 3.54207425 0.19236464 2.77601962
 2.69651575 0.58079881 1.34716915 0.02673098 1.8478802  0.16638289], Cost: 0.0035

   Return from subroutine COBYLA because the MAXFUN limit has been reached.

   NFVALS =  100   F = 3.519737E-03    MAXCV = 0.000000E+00
   X = 1.812229E+00   3.590802E+00   2.720558E+00   3.542074E+00   1.923646E-01
       2.776020E+00   2.696516E+00   5.807988E-01   1.347169E+00   2.673098E-02
       1.847880E+00   1.663829E-01
Simulating for QAOA depth p = 7
Current parameters: [1.55555671 0.04759428 0.07384749 0.84100427 1.53202818 0.00348515
 2.66465492 2.13299261 0.36795554 1.47022709 1.59797337 3.09867439
 0.08902481 1.99585789], Cost: 1.5807
Current parameters: [1.55555671 1.04759428 0.07384749 0.84100427 1.53202818 0.00348515
 2.66465492 2.13299261 0.36795554 1.47022709 1.59797337 3.09867439
 0.08902481 1.99585789], Cost: 2.4000
Current parameters: [1.55555671 0.04759428 1.07384749 0.84100427 1.53202818 0.00348515
 2.66465492 2.13299261 0.36795554 1.47022709 1.59797337 3.09867439
 0.08902481 1.99585789], Cost: 1.8110
Current parameters: [1.55555671 0.04759428 0.07384749 1.84100427 1.53202818 0.00348515
 2.66465492 2.13299261 0.36795554 1.47022709 1.59797337 3.09867439
 0.08902481 1.99585789], Cost: 1.9312
Current parameters: [1.55555671 0.04759428 0.07384749 0.84100427 2.53202818 0.00348515
 2.66465492 2.13299261 0.36795554 1.47022709 1.59797337 3.09867439
 0.08902481 1.99585789], Cost: 2.1200
Current parameters: [1.55555671 0.04759428 0.07384749 0.84100427 1.53202818 1.00348515
 2.66465492 2.13299261 0.36795554 1.47022709 1.59797337 3.09867439
 0.08902481 1.99585789], Cost: 2.3975
Current parameters: [1.55555671e+00 4.75942804e-02 7.38474937e-02 8.41004272e-01
 1.53202818e+00 3.48515161e-03 3.66465492e+00 2.13299261e+00
 3.67955537e-01 1.47022709e+00 1.59797337e+00 3.09867439e+00
 8.90248093e-02 1.99585789e+00], Cost: 1.8231
Current parameters: [1.55555671 0.04759428 0.07384749 0.84100427 1.53202818 0.00348515
```



```
 2.66465492 3.13299261 0.36795554 1.47022709 1.59797337 3.09867439
 0.08902481 1.99585789], Cost: 1.3905
Current parameters: [1.55555671 0.04759428 0.07384749 0.84100427 1.53202818 0.00348515
 2.66465492 3.13299261 1.36795554 1.47022709 1.59797337 3.09867439
 0.08902481 1.99585789], Cost: 3.2101
Current parameters: [1.55555671 0.04759428 0.07384749 0.84100427 1.53202818 0.00348515
 2.66465492 3.13299261 0.36795554 2.47022709 1.59797337 3.09867439
 0.08902481 1.99585789], Cost: 3.1749
Current parameters: [1.55555671 0.04759428 0.07384749 0.84100427 1.53202818 0.00348515
 2.66465492 3.13299261 0.36795554 1.47022709 2.59797337 3.09867439
 0.08902481 1.99585789], Cost: 3.3766
Current parameters: [1.55555671e+00 4.75942804e-02 7.38474937e-02 8.41004272e-01
 1.53202818e+00 3.48515161e-03 2.66465492e+00 3.13299261e+00
 3.67955537e-01 1.47022709e+00 1.59797337e+00 4.09867439e+00
 8.90248093e-02 1.99585789e+00], Cost: 1.6644
Current parameters: [1.55555671 0.04759428 0.07384749 0.84100427 1.53202818 0.00348515
 2.66465492 3.13299261 0.36795554 1.47022709 1.59797337 3.09867439
 1.08902481 1.99585789], Cost: 1.6734
Current parameters: [1.55555671 0.04759428 0.07384749 0.84100427 1.53202818 0.00348515
 2.66465492 3.13299261 0.36795554 1.47022709 1.59797337 3.09867439
 0.08902481 2.99585789], Cost: 1.7491
Current parameters: [ 1.59955317 -0.182762    0.00910383  0.74245569  1.38040452 -0.22615966
  2.59651586  3.18649093 -0.14370256  0.96846468  1.03951777  3.02164218
  0.00946213  1.89501696], Cost: 0.6582
Current parameters: [ 1.63629671 -0.37514349 -0.04496668  0.66015308  1.25377639 -0.41794696
  2.53960969  3.23116993 -0.57101259  0.54941902  1.76066325  2.95730892
 -0.05698444  1.81079989], Cost: 1.3361
Current parameters: [ 1.59955317  0.29563234  0.00910383  0.74245569  1.38040452 -0.22615966
  2.59651586  3.24354222 -0.20075385  0.9114134   1.07963963  2.9645909
 -0.04758915  1.83796567], Cost: 0.5775
Current parameters: [ 1.65384911  0.47133412 -0.07079618  0.62083711  1.1932861  -0.5095638
  2.5124256   2.94739563 -0.47002122  0.65435827  0.44815177  3.23169437
  0.21639147  2.07568684], Cost: 1.9440
Current parameters: [ 1.60726201e+00  2.77033824e-01 -2.24022693e-03  7.25188505e-01
  1.35383779e+00  2.50178435e-01  2.58457688e+00  3.25771726e+00
 -2.95205338e-01  8.18695779e-01  1.02952036e+00  2.94629237e+00
 -6.63310595e-02  1.81549550e+00], Cost: 1.4376
Current parameters: [ 1.59955317  0.29563234  0.00910383  0.53284049  1.516645   -0.22615966
  2.59651586  3.24354222 -0.20075385  0.9114134   1.07963963  2.9645909
 -0.04758915  1.83796567], Cost: 0.4661
Current parameters: [ 1.60498967e+00  2.82516135e-01  1.10367716e-03  7.83904704e-01
```



```
  1.93134937e+00 -2.89378550e-01  2.58809615e+00  3.25353886e+00
 -2.67363738e-01  8.46026283e-01  1.04429409e+00  2.95168625e+00
 -6.08064803e-02  1.82211906e+00], Cost: 1.1527
Current parameters: [ 1.59955317  0.29563234 -0.17213068  0.53284049  1.516645   -0.22615966
  2.76871938  3.24354222 -0.20075385  0.9114134   1.07963963  2.9645909
 -0.04758915  1.83796567], Cost: 0.6936
Current parameters: [ 1.60217408  0.28930909  0.36147272  0.51193343  1.49818166 -0.25663714
  2.94523681  3.24836155 -0.23286611  0.87989062  1.06259973  2.95836964
 -0.05396115  1.83032611], Cost: 0.6111
Current parameters: [ 1.35161144  0.29451081  0.00878862  0.53236069  1.51590679 -0.22727772
  2.59618411  3.23967602 -0.23140139  0.9152796   1.07692068  2.9684571
 -0.04372295  1.84183187], Cost: 0.5876
Current parameters: [ 1.57566863  0.28972036  0.03579768  0.45831022  1.45618221 -0.32767723
  2.5370803   3.32845136  0.19417931  0.72087377  1.05854152  2.87413615
 -0.13864018  1.74190056], Cost: 2.0337
Current parameters: [ 1.60177484  0.28400015  0.0058345   0.52786413  1.50898854 -0.23775592
  2.59307508  3.20344314 -0.22659079  0.93471879  1.05143957  3.00468998
 -0.00749007  1.60282821], Cost: 0.7719
Current parameters: [ 1.65382525  0.29990061  0.03718247  0.46641449  1.46582864 -0.31254934
  2.54309532  3.35806288 -0.37053368  1.299712    1.08357391  2.84481339
 -0.1679319   1.92384804], Cost: 2.5119
Current parameters: [ 1.60468020e+00  2.68788298e-01  1.55907631e-03  5.21356358e-01
  1.49897588e+00 -2.52920789e-01  2.58857543e+00  3.17580064e+00
 -2.60378755e-01  8.52941660e-01  1.11251734e+00  3.03233248e+00
 -2.55199225e-01  1.82621441e+00], Cost: 0.3001
Current parameters: [ 1.63595377e+00  2.21400282e-01 -9.16473738e-04  4.73549751e-01
  1.44615124e+00 -3.36729483e-01  2.55249840e+00  3.17288580e+00
 -4.37950591e-01  6.43851862e-01  1.46980558e+00  3.03312665e+00
 -8.31026223e-02  1.85515036e+00], Cost: 0.9878
Current parameters: [ 1.60535381e+00  2.65261422e-01  5.67816579e-04  5.19847527e-01
  1.49665445e+00 -2.56436773e-01  2.58753219e+00  3.36069057e+00
 -2.68212512e-01  8.45259412e-01  1.10396709e+00  3.19991339e+00
 -2.56417372e-01  1.82467048e+00], Cost: 0.8135
Current parameters: [ 1.6016659   0.37725931  0.04068368  0.54582774  1.55313975 -0.1739145
  2.60308799  2.9124865  -0.07705461  1.00487687  1.26789274  3.29085158
 -0.20905289  1.91327484], Cost: 0.4098
Current parameters: [ 1.67100133  0.3038822   0.01426272  0.44756445  1.48969883 -0.27607557
  2.51946153  3.13216963 -0.29092746  0.79708738  0.98258574  3.07040843
 -0.15514307  1.93375294], Cost: 1.1884
Current parameters: [ 1.41247467  0.25527952 -0.06974796  0.69770316  1.3958454  -0.38029181
  2.7766042   2.97703254 -0.3607194   0.79445675  1.04198656  3.08587811
```



```
 -0.43411369  1.67914991], Cost: 2.0576
Current parameters: [ 1.60718717  0.25263819  0.01292043  0.52235213  1.50464703 -0.01359855
  2.5888295   3.17482927 -0.28303019  0.81417209  1.12699407  2.99743823
 -0.28945591  1.81288615], Cost: 0.4281
Current parameters: [ 1.60575916e+00  3.88296563e-01  1.36753361e-03  5.19885941e-01
  1.49803328e+00 -2.53518098e-01  2.58732973e+00  3.19138520e+00
 -2.77009699e-01  8.44089286e-01  1.12259887e+00  3.01670352e+00
 -2.70040815e-01  1.81307633e+00], Cost: 0.2523
Current parameters: [ 1.64059544  0.37833876  0.02254609  0.48506614  1.49638176 -0.26267643
  2.55471005  3.22420243 -0.3200866   1.0391665   1.18863529  2.92681526
 -0.33160352  1.84709537], Cost: 0.8079
Current parameters: [ 1.61942133  0.38878127  0.0944077   0.50851865  1.50447206 -0.25850921
  2.66631829  3.19779039 -0.28229038  0.83909612  1.11827728  3.00998087
 -0.26243536  1.82453506], Cost: 0.2643
Current parameters: [ 1.62788953  0.38601843  0.01151369  0.63330852  1.71297426 -0.27272097
  2.56164743  3.19923389 -0.28726386  0.81667984  1.12737047  2.9901214
 -0.27332618  1.82537577], Cost: 0.5483
Current parameters: [ 1.60899506  0.33560625  0.01138293  0.50156043  1.4979724  -0.28957221
  2.57561175  3.19326547 -0.18754658  0.82245548  1.13242066  2.97840554
 -0.30021387  1.80906505], Cost: 0.2560
Current parameters: [ 1.58824604  0.56923544  0.01065546  0.50305211  1.46859    -0.17602641
  2.58054014  3.24740606 -0.17502353  0.76954814  1.16879824  3.00076122
 -0.2630146   1.78791686], Cost: 0.4619
Current parameters: [ 1.55570176  0.38879313 -0.01535986  0.55997118  1.46626495 -0.27326778
  2.63327477  3.14329141 -0.28891205  0.82521025  1.11064397  3.0197182
 -0.32547865  1.77201912], Cost: 0.5435
Current parameters: [ 1.60508316  0.39038774  0.00549333  0.52313974  1.50464467 -0.24950806
  2.59040192  3.15809175 -0.25460208  0.86259833  1.14834185  3.04938171
 -0.26355497  1.82195385], Cost: 0.2354
Current parameters: [ 1.60816328e+00  3.68796852e-01  2.82267128e-03  5.14460912e-01
  1.48962251e+00 -2.71519843e-01  2.58636713e+00  3.15522170e+00
 -2.95700363e-01  8.11642036e-01  1.24134968e+00  3.03487679e+00
 -2.29227604e-01  1.81924040e+00], Cost: 0.3046
Current parameters: [ 1.55863559  0.38999712  0.00682156  0.48852087  1.52725477 -0.2496431
  2.5892237   3.15791233 -0.25963606  0.86365521  1.14792589  3.04925649
 -0.26181547  1.82443646], Cost: 0.2350
Current parameters: [ 1.62094752  0.39771809 -0.02859514  0.4218007   1.56275747 -0.24879373
  2.61866786  3.14457887 -0.2690132   0.85361236  1.14892627  3.0683371
 -0.29120919  1.77697138], Cost: 0.2905
Current parameters: [ 1.55995546  0.39088123  0.04203911  0.48637535  1.52504228 -0.25113598
  2.55342413  3.15006607 -0.26788442  0.86214736  1.14511275  3.05994628
```

```
 -0.26853113  1.791711  ], Cost: 0.2059
Current parameters: [ 1.55161068  0.37741993  0.02940561  0.49559168  1.52832934 -0.2503815
  2.57401821  3.15166565 -0.24468374  0.89765105  1.13523428  3.03647057
 -0.18465108  1.71954298], Cost: 0.3712
Current parameters: [ 1.56040412  0.3696652   0.04466366  0.4868055   1.52658556 -0.19807985
  2.55363114  3.13418269 -0.26986457  0.85539359  1.14719244  3.04265022
 -0.27360278  1.7910317 ], Cost: 0.1905
Current parameters: [ 1.54882279  0.3225279   0.04555443  0.49697835  1.52393587 -0.16990031
  2.56186126  3.2146231  -0.26186104  0.85594175  1.16301735  3.11394943
 -0.28987956  1.77830889], Cost: 0.4207
Current parameters: [ 1.56001197  0.36432264  0.04325687  0.4553302   1.47623676 -0.19524759
  2.55457605  3.12854941 -0.26877338  0.86003005  1.136997    3.05180034
 -0.26544385  1.79671526], Cost: 0.2262
Current parameters: [ 1.58843758  0.36516242  0.06092635  0.4832397   1.56773567 -0.20220481
  2.50868261  3.09771661 -0.26890645  0.80957145  1.09665757  3.05437581
 -0.23570683  1.84806866], Cost: 0.6143
Current parameters: [ 1.55383844  0.40954388  0.04539923  0.48592874  1.520382   -0.18064541
  2.55518682  3.14365212 -0.24417628  0.82327055  1.15178309  3.04645958
 -0.26705979  1.78285764], Cost: 0.1997
Current parameters: [ 1.57138978  0.41901856  0.06292899  0.47056725  1.52267762 -0.18247652
  2.52956811  3.1522754  -0.26675664  0.92493111  1.19448934  2.99094085
 -0.3105059   1.8047549 ], Cost: 0.2084
Current parameters: [ 1.53689186  0.37685013  0.04118068  0.50675952  1.51293999 -0.20671889
  2.57079686  3.10699019 -0.27721214  0.85209431  1.14763449  3.03673873
 -0.30856117  1.7744644 ], Cost: 0.2516
Current parameters: [ 1.55963789  0.3825315   0.04213068  0.49126383  1.52640013 -0.18937275
  2.55650517  3.13368268 -0.29274841  0.86011346  1.14596957  3.05201084
 -0.2656159   1.79199555], Cost: 0.2591
Current parameters: [ 1.56186238  0.35744925  0.04501332  0.48183654  1.51924728 -0.21125039
  2.54995005  3.13166107 -0.28525321  0.82999518  1.19522629  3.03083811
 -0.26124145  1.78985262], Cost: 0.1818
Current parameters: [ 1.56179654  0.35773556  0.02041644  0.48110992  1.51953581 -0.20998746
  2.53219321  3.1294067  -0.28446257  0.83068184  1.19632637  3.03347205
 -0.26464562  1.78455709], Cost: 0.1960
Current parameters: [ 1.59374608  0.36109939  0.03924795  0.44776492  1.53737918 -0.21255768
  2.57201029  3.1247627  -0.28834076  0.82474135  1.19711289  3.03646673
 -0.27781826  1.76816489], Cost: 0.1878
Current parameters: [ 1.5579868   0.35532139  0.04129926  0.48451343  1.51761035 -0.21076057
  2.55760137  3.13367129 -0.28028479  0.84135047  1.19705542  3.02390353
 -0.24564487  1.76930424], Cost: 0.2010
Current parameters: [ 1.57462919  0.34693587  0.06336949  0.47556559  1.53771304 -0.22502967
```



```
  2.53089043  3.1103203  -0.25757429  0.8178295   1.18532743  3.00682536
 -0.2511862   1.80458589], Cost: 0.1104
Current parameters: [ 1.57855506  0.31386037  0.08378457  0.47441508  1.55073503 -0.22597375
  2.51971021  3.13295245 -0.22413687  0.80988553  1.19456982  3.01431902
 -0.26585555  1.81034262], Cost: 0.0661
Current parameters: [ 1.56232261  0.30559001  0.0937855   0.48806405  1.54584982 -0.24329207
  2.52715719  3.10499289 -0.20690054  0.79710771  1.19493135  2.99827958
 -0.30277346  1.80353291], Cost: 0.0625
Current parameters: [ 1.56048164  0.29507587  0.08978718  0.49096075  1.54587993 -0.24717266
  2.53007598  3.11148618 -0.21478871  0.77797401  1.17807373  2.99798201
 -0.29809235  1.79736816], Cost: 0.1155
Current parameters: [ 1.56890072  0.31764861  0.10150881  0.4772145   1.54135025 -0.24371685
  2.51386585  3.13398057 -0.21234113  0.80700154  1.19470453  2.95345387
 -0.32168268  1.80642976], Cost: 0.1013
Current parameters: [ 1.53909178  0.30538841  0.09419843  0.47077168  1.55716949 -0.24334894
  2.52682341  3.10495914 -0.20935846  0.79764685  1.19474346  2.99814075
 -0.30185591  1.80500744], Cost: 0.0617
Current parameters: [ 1.5412725   0.30006621  0.06727153  0.47174305  1.56485862 -0.2440216
  2.55735171  3.10594519 -0.1869216   0.80116823  1.20434819  2.98725986
 -0.29734545  1.84252864], Cost: 0.0450
Current parameters: [ 1.5412691   0.30589435  0.06731374  0.48809632  1.58917342 -0.24319726
  2.55761895  3.10854485 -0.19321776  0.80011456  1.20897307  2.98519199
 -0.29927999  1.84002157], Cost: 0.0475
Current parameters: [ 1.55094033  0.30088969  0.09309634  0.46492535  1.575521   -0.23862531
  2.54361486  3.07791721 -0.16595432  0.81719548  1.22651531  3.00196545
 -0.2915453   1.86584928], Cost: 0.0359
Current parameters: [ 1.55145849  0.29272176  0.09403487  0.46435048  1.57501091 -0.21184719
  2.54339334  3.07309149 -0.16997217  0.81499464  1.22687299  2.99030897
 -0.29481772  1.86577131], Cost: 0.0270
Current parameters: [ 1.56112191  0.3071176   0.10676187  0.4508908   1.57389132 -0.21773149
  2.52846711  3.09672691 -0.16634382  0.83093109  1.23378702  2.94950196
 -0.31167559  1.87921917], Cost: 0.1304
Current parameters: [ 1.55368696  0.27285359  0.09282825  0.46547697  1.57763037 -0.22017979
  2.54437563  3.0689713  -0.18127266  0.8333218   1.22494558  2.98686589
 -0.29438209  1.86503135], Cost: 0.0239
Current parameters: [ 1.54623837  0.26957357  0.09242179  0.47029277  1.57903399 -0.21575362
  2.55544875  3.06703723 -0.14964622  0.84790145  1.23435939  2.97443345
 -0.26565136  1.82778488], Cost: 0.1215
Current parameters: [ 1.55436481  0.26709751  0.08578921  0.46288863  1.57420676 -0.2261061
  2.53741359  3.06710534 -0.18839773  0.821381    1.24823095  2.98199065
 -0.28946996  1.86291623], Cost: 0.0248
```



```
Current parameters: [ 1.55543709  0.27104895  0.05133547  0.46453688  1.58489412 -0.21482628
  2.50813601  3.05956157 -0.16638023  0.84051675  1.21576219  2.99662758
 -0.3096202   1.86207447], Cost: 0.0300
Current parameters: [ 1.53722541  0.27147594  0.09493014  0.48275607  1.56779453 -0.2189437
  2.53416669  3.07331938 -0.18090258  0.83646029  1.22442253  2.98507173
 -0.28653013  1.87523742], Cost: 0.0328
Current parameters: [ 1.54507188  0.26810314  0.08865974  0.46931833  1.5676602  -0.20479287
  2.56270448  3.07932747 -0.19115662  0.84412941  1.24822149  3.02336182
 -0.32451024  1.86646282], Cost: 0.0793
Current parameters: [ 1.55818902  0.28952052  0.08696337  0.47332942  1.56719572 -0.22124519
  2.54950641  3.05231181 -0.18915264  0.8412124   1.22706256  2.98113936
 -0.29615898  1.8630867 ], Cost: 0.0167
Current parameters: [ 1.53950753  0.29606485  0.07826089  0.44872674  1.58277819 -0.21704269
  2.55281648  3.02971498 -0.21734604  0.83086816  1.21092578  2.98967382
 -0.27459416  1.88182717], Cost: 0.0922
Current parameters: [ 1.57179435  0.27413961  0.09731934  0.46980404  1.57608363 -0.22650411
  2.5559861   3.05360202 -0.18958683  0.84003353  1.21084588  2.98133729
 -0.29999288  1.86545552], Cost: 0.0178
Current parameters: [ 1.5792489   0.28441926  0.08126272  0.50937075  1.54603095 -0.22318268
  2.54473048  3.03045334 -0.18277621  0.80935782  1.22872866  2.98477536
 -0.30096321  1.87073386], Cost: 0.0373
Current parameters: [ 1.56634816  0.29772639  0.09080166  0.46926107  1.56417292 -0.22449551
  2.54043345  3.06057978 -0.19035913  0.84538241  1.23047048  2.96275438
 -0.30888838  1.87359799], Cost: 0.0407
Current parameters: [ 1.5492662   0.28753858  0.08919542  0.47255059  1.56862108 -0.22514423
  2.55129646  3.04624034 -0.18569327  0.8385438   1.22697141  2.97732133
 -0.3039643   1.8613235 ], Cost: 0.0163
Current parameters: [ 1.55267587  0.283737    0.0892146   0.47449788  1.5699108  -0.22263298
  2.55786962  3.04379812 -0.1686091   0.84651159  1.22952681  2.96818747
 -0.29204208  1.84394247], Cost: 0.0381
Current parameters: [ 1.54905461  0.28549652  0.0822056   0.47296437  1.56983537 -0.22463026
  2.56059486  3.04824898 -0.1815732   0.83995273  1.22982655  2.9769447
 -0.30510521  1.86969656], Cost: 0.0191
Current parameters: [ 1.54905793  0.28312965  0.09505244  0.47393478  1.56378656 -0.21785922
  2.55689638  3.05037315 -0.19162202  0.84567053  1.23494276  2.99522195
 -0.31999628  1.85301463], Cost: 0.0272
Current parameters: [ 1.54886244  0.28472003  0.0888546   0.46409573  1.55662852 -0.22558208
  2.55133186  3.04489508 -0.1831482   0.83875484  1.22422872  2.97837536
 -0.30269106  1.86267697], Cost: 0.0148
Current parameters: [ 1.54095276  0.2878906   0.086208    0.45313056  1.56366836 -0.22066477
  2.55366451  3.02979516 -0.19876285  0.83365222  1.21776652  2.98447414
```



```
 -0.29321103  1.86823027], Cost: 0.0263
Current parameters: [ 1.55282069  0.28438024  0.09438637  0.46586328  1.55719687 -0.22475328
  2.54761516  3.03671415 -0.1775935   0.83950336  1.22943444  2.98226166
 -0.30167579  1.86886022], Cost: 0.0116
Current parameters: [ 1.56005527  0.28167789  0.08733159  0.48314151  1.54540375 -0.22600981
  2.54774077  3.02937323 -0.17988345  0.82089507  1.22554476  2.98325562
 -0.30406839  1.86802519], Cost: 0.0199
Current parameters: [ 1.55155149  0.28751469  0.10341137  0.46742719  1.55401043 -0.22918845
  2.55535692  3.03891012 -0.18079842  0.83700316  1.23310682  2.980888
 -0.29708214  1.86996922], Cost: 0.0105
Current parameters: [ 1.55186005  0.2948293   0.09780915  0.46647528  1.556071   -0.25432296
  2.5518772   3.03994934 -0.17634118  0.84021283  1.230736    2.99513085
 -0.29563     1.86911063], Cost: 0.0107
Current parameters: [ 1.5427083   0.28530618  0.10813227  0.47500156  1.5569763  -0.23107748
  2.54888629  3.03919436 -0.18219831  0.83806344  1.22895211  2.97879862
 -0.29694322  1.87150488], Cost: 0.0121
Current parameters: [ 1.54950685  0.27755973  0.09978588  0.46964551  1.53957384 -0.22439316
  2.55934799  3.04732151 -0.1767098   0.84937948  1.22971649  2.99052943
 -0.28621745  1.88200012], Cost: 0.0196
Current parameters: [ 1.54952871  0.29357705  0.10656299  0.46573212  1.55557918 -0.22446947
  2.55544554  3.04381501 -0.17330413  0.83034625  1.22854114  2.98520377
 -0.29758842  1.8711894 ], Cost: 0.0111
Current parameters: [ 1.5480838   0.2905605   0.10082865  0.46795199  1.55205599 -0.22805532
  2.55336958  3.03907678 -0.18066707  0.83664444  1.23778312  2.9808672
 -0.29592151  1.86938166], Cost: 0.0113
Current parameters: [ 1.55148179  0.27831871  0.10411203  0.46317056  1.55718641 -0.2315042
  2.55187041  3.04368773 -0.18068842  0.82978228  1.23870127  2.98213111
 -0.29474899  1.87026781], Cost: 0.0084
Current parameters: [ 1.55247603  0.27990738  0.10422756  0.46190392  1.55584985 -0.23209783
  2.54996094  3.04565937 -0.1836103   0.8293493   1.23887192  2.98149412
 -0.29836294  1.87516805], Cost: 0.0081
Current parameters: [ 1.55824258  0.28121746  0.10945501  0.45710343  1.55670438 -0.23584234
  2.54559261  3.04627195 -0.17778526  0.83058173  1.24154269  2.97253385
 -0.29779731  1.87398411], Cost: 0.0170
Current parameters: [ 1.55311584  0.27882867  0.10624721  0.46404961  1.55449297 -0.2318308
  2.55003473  3.04816095 -0.18249613  0.83105725  1.24110399  2.98340017
 -0.30294048  1.87218879], Cost: 0.0135
Current parameters: [ 1.54962178  0.28047368  0.10586437  0.45520222  1.56037757 -0.23025724
  2.55104923  3.0383295  -0.19134092  0.82687332  1.23812308  2.98743591
 -0.29621353  1.87687197], Cost: 0.0045
Current parameters: [ 1.55247603  0.27990738  0.10422756  0.46190392  1.55584985 -0.23209783
```

```
  2.54996094  3.04565937 -0.1836103   0.8293493   1.23887192  2.98149412
 -0.29836294  1.87516805], Cost: 0.0081

   Return from subroutine COBYLA because the MAXFUN limit has been reached.

   NFVALS =  100   F = 8.066618E-03    MAXCV = 0.000000E+00
   X = 1.552476E+00   2.799074E-01   1.042276E-01   4.619039E-01   1.555850E+00
      -2.320978E-01   2.549961E+00   3.045659E+00  -1.836103E-01   8.293493E-01
       1.238872E+00   2.981494E+00  -2.983629E-01   1.875168E+00
Simulating for QAOA depth p = 8
Current parameters: [3.87731619 2.57092213 1.73303306 2.90454104 1.72532003 2.29466328
 2.91883894 1.05190845 1.01869953 0.79392011 0.97608519 3.08639307
 0.32439791 2.28463107 0.52242923 1.53317996], Cost: 2.1430
Current parameters: [2.87731619 3.57092213 1.73303306 2.90454104 1.72532003 2.29466328
 2.91883894 1.05190845 1.01869953 0.79392011 0.97608519 3.08639307
 0.32439791 2.28463107 0.52242923 1.53317996], Cost: 2.6393
Current parameters: [2.87731619 2.57092213 2.73303306 2.90454104 1.72532003 2.29466328
 2.91883894 1.05190845 1.01869953 0.79392011 0.97608519 3.08639307
 0.32439791 2.28463107 0.52242923 1.53317996], Cost: 1.3752
Current parameters: [2.87731619 2.57092213 2.73303306 3.90454104 1.72532003 2.29466328
 2.91883894 1.05190845 1.01869953 0.79392011 0.97608519 3.08639307
 0.32439791 2.28463107 0.52242923 1.53317996], Cost: 1.6042
Current parameters: [2.87731619 2.57092213 2.73303306 2.90454104 2.72532003 2.29466328
 2.91883894 1.05190845 1.01869953 0.79392011 0.97608519 3.08639307
 0.32439791 2.28463107 0.52242923 1.53317996], Cost: 1.5037
Current parameters: [2.87731619 2.57092213 2.73303306 2.90454104 1.72532003 3.29466328
 2.91883894 1.05190845 1.01869953 0.79392011 0.97608519 3.08639307
 0.32439791 2.28463107 0.52242923 1.53317996], Cost: 2.5236
Current parameters: [2.87731619 2.57092213 2.73303306 2.90454104 1.72532003 2.29466328
 3.91883894 1.05190845 1.01869953 0.79392011 0.97608519 3.08639307
 0.32439791 2.28463107 0.52242923 1.53317996], Cost: 2.1290
Current parameters: [2.87731619 2.57092213 2.73303306 2.90454104 1.72532003 2.29466328
 2.91883894 2.05190845 1.01869953 0.79392011 0.97608519 3.08639307
 0.32439791 2.28463107 0.52242923 1.53317996], Cost: 1.4444
Current parameters: [2.87731619 2.57092213 2.73303306 2.90454104 1.72532003 2.29466328
 2.91883894 1.05190845 2.01869953 0.79392011 0.97608519 3.08639307
 0.32439791 2.28463107 0.52242923 1.53317996], Cost: 0.7607
Current parameters: [2.87731619 2.57092213 2.73303306 2.90454104 1.72532003 2.29466328
 2.91883894 1.05190845 2.01869953 1.79392011 0.97608519 3.08639307
 0.32439791 2.28463107 0.52242923 1.53317996], Cost: 2.6869
Current parameters: [2.87731619 2.57092213 2.73303306 2.90454104 1.72532003 2.29466328
```



```
 2.91883894 1.05190845 2.01869953 0.79392011 1.97608519 3.08639307
 0.32439791 2.28463107 0.52242923 1.53317996], Cost: 1.5819
Current parameters: [2.87731619 2.57092213 2.73303306 2.90454104 1.72532003 2.29466328
 2.91883894 1.05190845 2.01869953 0.79392011 0.97608519 4.08639307
 0.32439791 2.28463107 0.52242923 1.53317996], Cost: 1.5710
Current parameters: [2.87731619 2.57092213 2.73303306 2.90454104 1.72532003 2.29466328
 2.91883894 1.05190845 2.01869953 0.79392011 0.97608519 3.08639307
 1.32439791 2.28463107 0.52242923 1.53317996], Cost: 2.8366
Current parameters: [2.87731619 2.57092213 2.73303306 2.90454104 1.72532003 2.29466328
 2.91883894 1.05190845 2.01869953 0.79392011 0.97608519 3.08639307
 0.32439791 3.28463107 0.52242923 1.53317996], Cost: 0.7775
Current parameters: [2.87731619 2.57092213 2.73303306 2.90454104 1.72532003 2.29466328
 2.91883894 1.05190845 2.01869953 0.79392011 0.97608519 3.08639307
 0.32439791 2.28463107 1.52242923 1.53317996], Cost: 0.9014
Current parameters: [2.87731619 2.57092213 2.73303306 2.90454104 1.72532003 2.29466328
 2.91883894 1.05190845 2.01869953 0.79392011 0.97608519 3.08639307
 0.32439791 2.28463107 0.52242923 2.53317996], Cost: 1.7963
Current parameters: [ 2.82891242  2.38804206  2.89272659  2.84246126  1.69048438  1.98342442
  2.71455247  1.03314125  2.18522362  0.27192502  0.75352285  2.86679853
 -0.23817165  2.28007039  0.4842815   1.25253384], Cost: 2.1360
Current parameters: [2.87156477 2.54919202 2.75200811 2.89716461 1.7211808  2.78174549
 2.89456528 1.0496785  2.0384862  0.73189579 0.94963996 3.06030049
 0.25755246 2.28408917 0.51789645 1.49983313], Cost: 1.5357
Current parameters: [2.63563803 2.63488827 2.73303306 2.90454104 1.72532003 2.29466328
 2.91883894 1.05190845 2.01869953 0.79392011 0.97608519 3.08639307
 0.32439791 2.28463107 0.52242923 1.53317996], Cost: 0.7261
Current parameters: [2.75981616 3.11430517 2.74421063 2.90019583 1.72288175 2.27252729
 2.90454014 1.05059486 2.03035519 0.75738365 0.96050719 3.0710228
 0.28502149 2.28431185 0.51975912 1.51353646], Cost: 0.5615
Current parameters: [2.75981616 3.11430517 2.94117241 2.90019583 1.72288175 2.27252729
 3.05850786 1.05059486 2.03035519 0.75738365 0.96050719 3.0710228
 0.28502149 2.28431185 0.51975912 1.51353646], Cost: 0.3081
Current parameters: [2.75974234 3.12894862 2.66174234 2.89386658 1.71933013 2.24028385
 3.46143219 1.04868148 2.04733292 0.70416439 0.93781616 3.04863435
 0.22766551 2.28384688 0.51586982 1.48492358], Cost: 0.9006
Current parameters: [2.75981616 3.11430517 2.94117241 2.65170422 1.72288175 2.27252729
 3.05850786 1.05059486 2.03035519 0.75738365 0.96050719 3.0710228
 0.31244264 2.28431185 0.51975912 1.51353646], Cost: 0.7225
Current parameters: [2.75963566 3.15011343 3.03534773 3.05947119 1.71419682 2.193681
 3.04921492 1.04591598 2.07187157 0.62724427 0.90501984 3.01627536
 0.70734409 2.28317482 0.51024845 1.44356815], Cost: 2.1853
```



```
Current parameters: [2.75981616 3.11430517 2.94117241 2.90019583 1.4734366  2.27252729
 3.05850786 1.05059486 2.03035519 0.77403052 0.96050719 3.0710228
 0.28502149 2.28431185 0.51975912 1.51353646], Cost: 0.5800
Current parameters: [2.75967742 3.14182851 3.01355849 2.96768835 1.80862943 2.21192358
 3.05136501 1.04699853 2.06226597 1.19420383 0.91785789 3.02894221
 0.11184049 2.2834379  0.51244893 1.45975664], Cost: 1.1260
Current parameters: [2.75942395 3.11282334 2.94246636 2.89969282 1.72259949 2.27000541
 3.05685258 0.80069913 2.03035519 0.75315407 0.96050719 3.0710228
 0.28046315 2.28431185 0.51975912 1.51353646], Cost: 0.2332
Current parameters: [ 2.74112084  3.06528879  3.05829325  2.92837775  1.74789138  2.10629056
  2.97455064  0.80267124  2.05495156  0.47846324  0.92763373  3.0385877
 -0.06450608  2.28363822  0.51412453  1.47208375], Cost: 1.3149
Current parameters: [2.75942395 3.11282334 2.94246636 2.89969282 1.72259949 2.27000541
 3.05685258 0.80069913 2.03035519 0.75315407 0.76462629 3.0710228
 0.28046315 2.28431185 0.51975912 1.66887696], Cost: 0.6846
Current parameters: [2.76466645 3.1449544  2.95677475 2.9361501  1.74829918 2.27747843
 3.07609718 0.79348628 2.04437639 0.76562133 1.29949739 3.05253311
 0.26600868 2.28392785 0.51654711 1.8747011 ], Cost: 0.8277
Current parameters: [2.75942395 3.11282334 2.94246636 2.89969282 1.72259949 2.27000541
 3.05685258 0.80069913 2.22955635 0.75315407 0.96050719 3.22208206
 0.28046315 2.28431185 0.51975912 1.51353646], Cost: 0.8729
Current parameters: [2.76353166 3.13799926 2.95367753 2.9282585  1.74273618 2.27586081
 3.07193146 0.79504759 1.6952574  0.76292265 0.98112961 3.43720543
 0.26913751 2.28401097 0.51724239 1.48759664], Cost: 0.1640
Current parameters: [2.76327178 3.13701739 2.95453492 2.9279252  1.74254915 2.29786954
 3.07083466 0.79494683 1.69615146 0.76012009 0.97993468 3.43602644
 0.26611711 2.28401097 0.26826523 1.48608987], Cost: 0.1306
Current parameters: [2.7639961  3.1544018  2.98970962 2.96419489 1.7691321  2.77311836
 3.07046712 0.78423866 1.65173233 0.715552   0.98589712 3.37975535
 0.18493432 2.28355455 0.30862472 1.41481217], Cost: 0.5830
Current parameters: [2.76323753 3.13688798 2.95464792 2.92802259 1.74253341 2.29772665
 3.07069011 0.79493835 1.69622669 0.75988424 0.97983412 3.43592722
 0.26699968 2.03401289 0.26824799 1.48596307], Cost: 0.7653
Current parameters: [2.763575   3.17337197 3.0438285  3.0755489  1.80933943 2.22055041
 3.06388279 0.77076581 1.57555356 0.69633459 1.0127469  3.3190008
 0.61870357 2.45101635 0.24994632 1.33806203], Cost: 1.9708
Current parameters: [2.76380747 3.13904133 2.95276758 2.92127981 1.75453028 2.27447139
 3.07309551 0.71392584 1.66883304 0.93965134 1.00661623 3.3408993
 0.20589633 2.28370515 0.26570724 1.39219751], Cost: 0.2994
Current parameters: [2.76318118 3.16422502 3.02548615 2.99241174 1.81208153 2.28026478
 3.0640113  1.04538819 1.71201425 0.96900708 0.89122527 3.63853898
```



```
 0.07961853 2.41257712 0.2582196  1.65335328], Cost: 0.5888
Current parameters: [2.76385225 3.13921054 2.86177695 2.98061755 1.79651819 2.28448572
 3.27972148 0.83433227 1.69586197 0.73430514 0.97842896 3.41115676
 0.23699933 2.28381597 0.26663412 1.45963571], Cost: 0.1602
Current parameters: [2.76121836 3.15683198 2.65855299 2.72082068 1.5138643  2.29975503
 3.07693843 0.65545283 1.64530105 0.7222233  0.96670232 3.54484501
 0.1812379  2.41305    0.26127267 1.57191807], Cost: 0.5814
Current parameters: [2.75489652 3.11126865 3.00917344 2.93672563 1.755179   2.22702581
 3.0342731  0.78852552 1.66811639 0.62893999 0.99351323 3.3947727
 0.09460134 2.31136796 0.26484376 1.4403787 ], Cost: 0.7315
Current parameters: [2.64925581 3.16647441 2.96479454 2.89217703 1.75560009 2.29794691
 3.07893525 0.80434926 1.69427636 0.75936973 0.98198563 3.43242655
 0.27033992 2.28401352 0.26828658 1.48277087], Cost: 0.2352
Current parameters: [2.84747726 3.11705093 3.00923117 2.72698879 1.81414622 2.30212323
 3.11661339 0.84911291 1.67992545 0.75842474 0.99304224 3.42192633
 0.28986656 2.2931052  0.26809081 1.47244379], Cost: 0.2451
Current parameters: [2.74322879 3.05763258 2.94661743 2.92247277 1.73416178 2.30012131
 3.06694279 0.80555437 1.70120647 0.76411347 0.91112519 3.46077056
 0.27479992 2.28412495 0.26921864 1.42986711], Cost: 0.2349
Current parameters: [2.71914114 2.95975785 2.95680854 2.93988334 1.75559355 2.31250818
 3.08647189 0.78005222 1.67467965 0.7799515  1.10182839 3.41269297
 0.277629   2.29339451 0.26816044 1.59404876], Cost: 0.2232
Current parameters: [2.76327409 3.13707359 2.89006482 2.92804266 1.83579609 2.29778339
 3.02034702 0.78353125 1.6985803  0.75375821 0.97608013 3.44091678
 0.26585373 2.28400949 0.26825286 1.48909956], Cost: 0.1649
Current parameters: [2.7665879  3.14068949 3.04170304 2.93011516 1.80772412 2.3004594
 3.14024438 0.61746492 1.72853285 0.7561731  0.92028502 3.51921393
 0.26219587 2.29311925 0.2677961  1.53766015], Cost: 0.4108
Current parameters: [2.76367641 3.13949736 2.95563928 2.93073908 1.74453273 2.29844633
 3.07232002 0.79439012 1.71737351 0.76108235 1.0545887  3.51322236
 0.26500147 2.28398133 0.26801732 1.42594236], Cost: 0.2407
Current parameters: [2.77185561 3.13442041 2.93789999 2.91596673 1.72219701 2.30780786
 3.06356908 0.80800845 1.93047395 0.76471688 0.9851639  3.36423041
 0.27207097 2.31325905 0.2685403  1.50228625], Cost: 0.1333
Current parameters: [2.78255026 3.15953216 2.95308275 2.96294331 1.73530032 2.28658719
 3.066619   0.8095476  1.67827773 0.76126508 0.98171634 3.40882234
 0.36078614 2.33927072 0.26311422 1.48700724], Cost: 0.1710
Current parameters: [2.76335053 3.13723734 2.95407609 2.92800482 1.74115591 2.34945277
 3.07118519 0.77966174 1.69072814 0.74158944 0.98559819 3.41886679
 0.26900995 2.2840203  0.27313706 1.4694159 ], Cost: 0.1212
Current parameters: [2.77457651 3.15466143 2.96364482 2.95141165 1.74857562 2.41204156
```

```
 3.06805178 0.83871186 1.68905675 0.79104218 0.96647658 3.4465549
 0.23629054 2.31559235 0.2764625  1.50720144], Cost: 0.2028
Current parameters: [2.77214019 3.14603416 2.92387092 2.90905511 1.70553807 2.33766596
 3.07014699 0.77066809 1.68259215 0.74472718 0.98027761 3.43118657
 0.25619715 2.28836473 0.26748372 1.49220873], Cost: 0.0833
Current parameters: [2.75670637 3.13935193 2.8565818  2.87782565 1.69972639 2.39760392
 3.07771483 0.73709766 1.68730035 0.73132122 0.99427657 3.42187913
 0.29496293 2.33223176 0.28738558 1.46536122], Cost: 0.0330
Current parameters: [2.75796764 3.14363706 2.84720882 2.87623926 1.69765096 2.41020244
 3.08385509 0.73844113 1.69234086 0.75296551 0.99186216 3.42945774
 0.32512673 2.28683559 0.28784748 1.47344591], Cost: 0.0674
Current parameters: [2.78291008 3.15163562 2.81504643 2.88119465 1.66006757 2.40466534
 3.0518497  0.73905946 1.67419624 0.65184429 1.00699086 3.36399199
 0.27023966 2.32088468 0.29116788 1.46583535], Cost: 0.0483
Current parameters: [2.75542539 3.13865095 2.8661492  2.87917349 1.70577578 2.40484335
 3.08534864 0.73437891 1.69746971 0.69062691 0.98179474 3.45242632
 0.30889966 2.33395028 0.28869372 1.49003568], Cost: 0.0398
Current parameters: [2.7818152  3.15822747 2.86778475 2.88111303 1.70100571 2.3967227
 3.09893492 0.62788888 1.68891489 0.74638818 0.97382911 3.40379779
 0.27542324 2.35641325 0.28558059 1.47871141], Cost: 0.0394
Current parameters: [2.77739841 3.17228453 2.86359909 2.84606593 1.70821436 2.39641383
 3.08128007 0.74782772 1.68952289 0.72658823 0.97217683 3.42439906
 0.29675659 2.33204615 0.28782618 1.44068798], Cost: 0.0572
Current parameters: [2.79060893 3.08401128 2.875121   2.80302417 1.71698779 2.39564814
 3.09883884 0.74405404 1.66949808 0.73360983 1.03490528 3.39627906
 0.29985562 2.34423096 0.28319557 1.50853505], Cost: 0.0297
Current parameters: [2.79256009 3.08515964 2.877667   2.80426111 1.71707598 2.38709159
 3.09961168 0.7432041  1.64956571 0.73494814 1.03428855 3.40171272
 0.2985374  2.34105144 0.34125973 1.51104124], Cost: 0.0799
Current parameters: [2.78303814 3.08413476 2.8739098  2.81008978 1.73087777 2.41131371
 3.10196757 0.73195715 1.55981369 0.72241064 1.03552704 3.42258295
 0.3048803  2.33803003 0.24199248 1.49087008], Cost: 0.0804
Current parameters: [2.78631896 3.08239871 2.89579197 2.78967714 1.70649399 2.40504637
 3.04591989 0.73327972 1.6682813  0.73759162 1.03263986 3.39939995
 0.31074893 2.34598814 0.28407768 1.51171764], Cost: 0.0183
Current parameters: [2.81275356 3.08673612 2.89287245 2.78975676 1.68156709 2.39551967
 3.03781834 0.7151226  1.701619   0.73518456 1.12004229 3.45694073
 0.29843652 2.35867505 0.26678256 1.47836425], Cost: 0.0607
Current parameters: [2.75317321 3.12091232 2.90220411 2.77899561 1.71408668 2.40431242
 3.05014719 0.7328316  1.66582252 0.73589789 1.05773392 3.38935022
 0.30918443 2.34595888 0.28375025 1.53044363], Cost: 0.0282
```



```
Current parameters: [2.71395117 3.034728   2.88411331 2.75160719 1.66296013 2.38244167
 3.05067907 0.72442685 1.69603967 0.73471952 0.99553675 3.38967789
 0.29701264 2.36782967 0.25331229 1.49905591], Cost: 0.0657
Current parameters: [2.78633126 3.08608356 2.89256615 2.81077588 1.69427235 2.38385482
 3.04377221 0.74453459 1.66049036 0.74935365 1.03814851 3.38865545
 0.3439874  2.38136515 0.28158942 1.51186078], Cost: 0.0792
Current parameters: [2.84249337 3.10305164 2.91315853 2.74641755 1.70129758 2.41025041
 3.05925515 0.71023701 1.69611739 0.69448076 1.00178799 3.39409668
 0.33839688 2.30215605 0.25224283 1.55407973], Cost: 0.1073
Current parameters: [2.78625537 3.0787768  2.86242162 2.78678211 1.7463742  2.38771009
 3.02147121 0.72858833 1.67511473 0.73927381 1.02981266 3.404591
 0.30261136 2.34711398 0.27880071 1.52069774], Cost: 0.0147
Current parameters: [2.7913854  3.08496402 2.85298438 2.78382265 1.73458844 2.39404125
 3.02055775 0.73534066 1.67184982 0.74883739 1.02461009 3.41381518
 0.29235052 2.35407732 0.27729548 1.53547662], Cost: 0.0156
Current parameters: [2.78367416 3.06843761 2.8729785  2.7998686  1.76844651 2.43229918
 3.01575413 0.73672549 1.68968321 0.74315137 1.03109546 3.39231741
 0.28202866 2.35767736 0.27522846 1.51784195], Cost: 0.0261
Current parameters: [2.78351692 3.07806704 2.87627888 2.78230671 1.75766325 2.38476018
 3.02913452 0.72800755 1.67875335 0.7539363  1.02384465 3.42048038
 0.30808687 2.35033792 0.27742277 1.52234305], Cost: 0.0284
Current parameters: [2.78960754 3.07548358 2.88787242 2.8084099  1.74297134 2.35872579
 3.01064003 0.74149152 1.67257637 0.74222454 1.02977691 3.39746618
 0.27037034 2.33041933 0.26645563 1.5305302 ], Cost: 0.0500
Current parameters: [2.78451403 3.0768695  2.86357564 2.77896866 1.74890462 2.38754692
 3.01837834 0.75491076 1.67532042 0.72817255 1.03129042 3.41147186
 0.3067945  2.3460012  0.27925698 1.51807301], Cost: 0.0190
Current parameters: [2.77446008 3.07017784 2.84436415 2.79608597 1.73884499 2.40020184
 3.01783219 0.73709957 1.68030057 0.76707691 1.03898625 3.40348781
 0.32070164 2.30515685 0.27607718 1.52202991], Cost: 0.0230
Current parameters: [2.79544509 3.07206885 2.86385488 2.76850537 1.74879328 2.38500843
 3.02360014 0.73194218 1.66887165 0.75151842 1.03890861 3.39071134
 0.29952411 2.34886997 0.27765109 1.51873144], Cost: 0.0135
Current parameters: [2.80028238 3.10798444 2.85724607 2.76252542 1.7474812  2.38488123
 3.01600377 0.73668659 1.6742232  0.76384952 1.01812499 3.38527265
 0.29035262 2.34381565 0.27590039 1.47778548], Cost: 0.0130
Current parameters: [2.7922503  3.10455572 2.85534384 2.76586299 1.75042315 2.38881977
 3.01521493 0.73493676 1.64892997 0.76448568 1.02066232 3.39270323
 0.28658909 2.34497697 0.26779665 1.46914282], Cost: 0.0119
Current parameters: [2.81558017 3.11312268 2.85083663 2.74511158 1.75445488 2.40690197
 3.0172697  0.71631837 1.64037508 0.73830864 1.00756954 3.38956653
```



```
 0.29677496 2.32505793 0.24333063 1.47937813], Cost: 0.0171
Current parameters: [2.77659671 3.0977895  2.8543285  2.76043294 1.74925246 2.38720095
 3.01778965 0.73716915 1.64598938 0.76452027 0.99935344 3.38036936
 0.28688521 2.34554267 0.26812099 1.47406945], Cost: 0.0059
Current parameters: [2.75497741 3.07892249 2.83986646 2.73534141 1.72802953 2.38648423
 3.01810156 0.71961439 1.66671145 0.75394776 1.00786929 3.39191194
 0.26843799 2.34954757 0.25864315 1.4569855 ], Cost: 0.0315
Current parameters: [2.77439218 3.10026156 2.8471957  2.76937819 1.75230098 2.38272424
 3.01428954 0.72013037 1.64295302 0.75197534 0.99809061 3.37678074
 0.28859547 2.34512466 0.28556397 1.47027905], Cost: 0.0035
Current parameters: [2.78351672 3.09587591 2.85146954 2.74609843 1.75470309 2.39856208
 3.01478186 0.73357175 1.62454829 0.76138889 0.991148   3.38353606
 0.27576722 2.32996086 0.32849346 1.47716357], Cost: 0.0119
Current parameters: [2.77908341 3.1004647  2.83862044 2.77198267 1.74795688 2.38471806
 3.00851527 0.72497313 1.63486293 0.75595235 0.99497694 3.3655315
 0.30602935 2.36153483 0.28748559 1.47062656], Cost: 0.0232
Current parameters: [2.78134374 3.09326785 2.8425919  2.77358364 1.75259366 2.38131885
 3.02218504 0.72198457 1.64361629 0.75164052 0.99373329 3.37816216
 0.28710104 2.34484154 0.28548079 1.46622704], Cost: 0.0035
Current parameters: [2.76598802 3.11367001 2.83937206 2.76894358 1.75942297 2.38205429
 3.03523053 0.7240665  1.6414039  0.74939123 1.00602507 3.37045372
 0.284214   2.34430371 0.28594617 1.47552085], Cost: 0.0068
Current parameters: [2.77544476 3.10234737 2.84639121 2.76602048 1.74676858 2.37079225
 3.01552032 0.7204641  1.63912625 0.75258797 0.99786367 3.38012697
 0.29204234 2.34174066 0.28564612 1.47187508], Cost: 0.0047
Current parameters: [2.77674681 3.09966834 2.85645157 2.78180327 1.75328042 2.37490772
 3.00740512 0.72215905 1.64003414 0.74821994 0.99520987 3.36570947
 0.27021935 2.33593801 0.28104468 1.47486713], Cost: 0.0112
Current parameters: [2.77726099 3.10214613 2.8533032  2.76565193 1.7550368  2.37962498
 3.01629663 0.71916999 1.64183642 0.74776148 0.99534026 3.37912292
 0.2847708  2.35571877 0.2859868  1.47046863], Cost: 0.0065
Current parameters: [2.76771842 3.09984363 2.85556882 2.77445172 1.76719503 2.38138595
 3.01897936 0.70882978 1.64751966 0.76444679 0.98959266 3.38645109
 0.296451   2.33749621 0.28492216 1.47076998], Cost: 0.0142
Current parameters: [2.77429027 3.09904821 2.83960713 2.76681743 1.76179834 2.37877032
 3.00808732 0.72435569 1.64439701 0.75058371 0.99779684 3.37923242
 0.28730437 2.34532014 0.28439285 1.47205794], Cost: 0.0037
Current parameters: [2.76693262 3.09979617 2.85594669 2.7732639  1.75305037 2.38422689
 3.01483407 0.73574985 1.64555636 0.73380454 0.99698248 3.38201861
 0.29854937 2.33886895 0.28802869 1.46179197], Cost: 0.0070
Current parameters: [2.77531249 3.10994138 2.84485244 2.76892169 1.75103892 2.38329046
```



```
 3.01222136 0.72152119 1.64446525 0.75482858 0.9920369  3.37697231
 0.28586691 2.34422692 0.28511018 1.46132352], Cost: 0.0037
Current parameters: [2.77114083 3.10593065 2.84015596 2.78058412 1.74266284 2.38933033
 3.01286926 0.72205185 1.64423185 0.74886339 0.98975791 3.39294327
 0.2844268  2.34866899 0.28557008 1.48591738], Cost: 0.0068
Current parameters: [2.78107289 3.1032367  2.84679616 2.76546683 1.75428053 2.38713746
 3.01479698 0.71645591 1.64000016 0.74609254 0.99433573 3.37533781
 0.29199232 2.34006816 0.28009969 1.47368076], Cost: 0.0056
Current parameters: [2.76406545 3.09034964 2.83888092 2.75600482 1.74234789 2.38516996
 3.01430186 0.71049818 1.65170512 0.74536129 1.00159972 3.37935742
 0.27852763 2.34192596 0.28157092 1.4625958 ], Cost: 0.0046
Current parameters: [2.77637776 3.09873177 2.84795703 2.76343839 1.75249234 2.38690962
 3.0143292  0.72343805 1.6389754  0.75447037 0.99731601 3.37843602
 0.28471522 2.34078638 0.29617642 1.47154907], Cost: 0.0039
Current parameters: [2.77308202 3.09970587 2.84677049 2.77043217 1.75307565 2.38376352
 3.01398529 0.71960403 1.63684817 0.75213057 0.99962545 3.37913771
 0.28765576 2.34538657 0.28355395 1.46793978], Cost: 0.0035
Current parameters: [2.78193631 3.10311388 2.84726832 2.77150168 1.75212935 2.38348471
 3.01296456 0.71887328 1.64702765 0.75188813 1.00904832 3.38184041
 0.28777854 2.34389182 0.28661222 1.46802851], Cost: 0.0045
Current parameters: [2.77169954 3.09775404 2.84511468 2.76603563 1.74981553 2.38330316
 3.01431125 0.71772669 1.64510841 0.75031234 0.99881625 3.37734309
 0.28611193 2.34436349 0.28450318 1.46838792], Cost: 0.0023
Current parameters: [2.77439218 3.10026156 2.8471957  2.76937819 1.75230098 2.38272424
 3.01428954 0.72013037 1.64295302 0.75197534 0.99809061 3.37678074
 0.28859547 2.34512466 0.28556397 1.47027905], Cost: 0.0035

   Return from subroutine COBYLA because the MAXFUN limit has been reached.

   NFVALS =  100   F = 3.455189E-03    MAXCV = 0.000000E+00
   X = 2.774392E+00   3.100262E+00   2.847196E+00   2.769378E+00   1.752301E+00
       2.382724E+00   3.014290E+00   7.201304E-01   1.642953E+00   7.519753E-01
       9.980906E-01   3.376781E+00   2.885955E-01   2.345125E+00   2.855640E-01
       1.470279E+00
Simulating for QAOA depth p = 9
Current parameters: [3.79918725 2.0915078  0.14828162 1.70901721 0.4357638  2.37695462
 0.14868245 2.4294332  2.93589655 1.50533215 1.11534406 1.67606645
 2.78976471 0.44274981 0.49625002 2.17355436 1.90260098 0.0852818 ], Cost: 2.6188
Current parameters: [2.79918725 3.0915078  0.14828162 1.70901721 0.4357638  2.37695462
 0.14868245 2.4294332  2.93589655 1.50533215 1.11534406 1.67606645
 2.78976471 0.44274981 0.49625002 2.17355436 1.90260098 0.0852818 ], Cost: 3.0850
```

```
Current parameters: [2.79918725 2.0915078  1.14828162 1.70901721 0.4357638  2.37695462
 0.14868245 2.4294332  2.93589655 1.50533215 1.11534406 1.67606645
 2.78976471 0.44274981 0.49625002 2.17355436 1.90260098 0.0852818 ], Cost: 2.2965
Current parameters: [2.79918725 2.0915078  1.14828162 2.70901721 0.4357638  2.37695462
 0.14868245 2.4294332  2.93589655 1.50533215 1.11534406 1.67606645
 2.78976471 0.44274981 0.49625002 2.17355436 1.90260098 0.0852818 ], Cost: 1.4868
Current parameters: [2.79918725 2.0915078  1.14828162 2.70901721 1.4357638  2.37695462
 0.14868245 2.4294332  2.93589655 1.50533215 1.11534406 1.67606645
 2.78976471 0.44274981 0.49625002 2.17355436 1.90260098 0.0852818 ], Cost: 1.7340
Current parameters: [2.79918725 2.0915078  1.14828162 2.70901721 0.4357638  3.37695462
 0.14868245 2.4294332  2.93589655 1.50533215 1.11534406 1.67606645
 2.78976471 0.44274981 0.49625002 2.17355436 1.90260098 0.0852818 ], Cost: 2.5242
Current parameters: [2.79918725 2.0915078  1.14828162 2.70901721 0.4357638  2.37695462
 1.14868245 2.4294332  2.93589655 1.50533215 1.11534406 1.67606645
 2.78976471 0.44274981 0.49625002 2.17355436 1.90260098 0.0852818 ], Cost: 1.9789
Current parameters: [2.79918725 2.0915078  1.14828162 2.70901721 0.4357638  2.37695462
 0.14868245 3.4294332  2.93589655 1.50533215 1.11534406 1.67606645
 2.78976471 0.44274981 0.49625002 2.17355436 1.90260098 0.0852818 ], Cost: 2.2835
Current parameters: [2.79918725 2.0915078  1.14828162 2.70901721 0.4357638  2.37695462
 0.14868245 2.4294332  3.93589655 1.50533215 1.11534406 1.67606645
 2.78976471 0.44274981 0.49625002 2.17355436 1.90260098 0.0852818 ], Cost: 1.5801
Current parameters: [2.79918725 2.0915078  1.14828162 2.70901721 0.4357638  2.37695462
 0.14868245 2.4294332  2.93589655 2.50533215 1.11534406 1.67606645
 2.78976471 0.44274981 0.49625002 2.17355436 1.90260098 0.0852818 ], Cost: 1.5673
Current parameters: [2.79918725 2.0915078  1.14828162 2.70901721 0.4357638  2.37695462
 0.14868245 2.4294332  2.93589655 1.50533215 2.11534406 1.67606645
 2.78976471 0.44274981 0.49625002 2.17355436 1.90260098 0.0852818 ], Cost: 1.5250
Current parameters: [2.79918725 2.0915078  1.14828162 2.70901721 0.4357638  2.37695462
 0.14868245 2.4294332  2.93589655 1.50533215 1.11534406 2.67606645
 2.78976471 0.44274981 0.49625002 2.17355436 1.90260098 0.0852818 ], Cost: 0.4921
Current parameters: [2.79918725 2.0915078  1.14828162 2.70901721 0.4357638  2.37695462
 0.14868245 2.4294332  2.93589655 1.50533215 1.11534406 2.67606645
 3.78976471 0.44274981 0.49625002 2.17355436 1.90260098 0.0852818 ], Cost: 3.4844
Current parameters: [2.79918725 2.0915078  1.14828162 2.70901721 0.4357638  2.37695462
 0.14868245 2.4294332  2.93589655 1.50533215 1.11534406 2.67606645
 2.78976471 1.44274981 0.49625002 2.17355436 1.90260098 0.0852818 ], Cost: 2.7582
Current parameters: [2.79918725 2.0915078  1.14828162 2.70901721 0.4357638  2.37695462
 0.14868245 2.4294332  2.93589655 1.50533215 1.11534406 2.67606645
 2.78976471 0.44274981 1.49625002 2.17355436 1.90260098 0.0852818 ], Cost: 1.2934
Current parameters: [2.79918725 2.0915078  1.14828162 2.70901721 0.4357638  2.37695462
 0.14868245 2.4294332  2.93589655 1.50533215 1.11534406 2.67606645
```



```
 2.78976471 0.44274981 0.49625002 3.17355436 1.90260098 0.0852818 ], Cost: 1.3148
Current parameters: [2.79918725 2.0915078  1.14828162 2.70901721 0.4357638  2.37695462
 0.14868245 2.4294332  2.93589655 1.50533215 1.11534406 2.67606645
 2.78976471 0.44274981 0.49625002 2.17355436 2.90260098 0.0852818 ], Cost: 2.5973
Current parameters: [2.79918725 2.0915078  1.14828162 2.70901721 0.4357638  2.37695462
 0.14868245 2.4294332  2.93589655 1.50533215 1.11534406 2.67606645
 2.78976471 0.44274981 0.49625002 2.17355436 1.90260098 1.0852818 ], Cost: 2.2952
Current parameters: [ 2.77777432  1.98063402  1.18870768  2.8643922   0.3883414   2.17788263
  0.05425758  2.27656458  2.91799792  1.48989318  1.10801433  2.86694789
  2.21556969  0.00790823  0.34249442  2.01569134  1.49862349 -0.26071648], Cost: 1.5095
Current parameters: [ 2.78848078  2.03607091  1.16849465  2.78670471  0.4120526   2.27741863
  0.10147001  2.35299889  2.92694724  1.49761267  1.11167919  2.77150717
  2.5026672   0.22532902  0.41937222  2.09462285  1.70061224 -0.08771734], Cost: 3.2669
Current parameters: [2.5493609  2.0915078  1.14828162 2.70901721 0.4357638  2.37695462
 0.14868245 2.4294332  2.93589655 1.50533215 1.11534406 2.67606645
 2.79908126 0.44274981 0.49625002 2.17355436 1.90260098 0.0852818 ], Cost: 0.3798
Current parameters: [2.55357782 2.07379217 1.15474098 2.73384333 0.42818656 2.34514651
 0.13359506 2.40500755 2.93303668 1.50286528 1.1141729  2.70656586
 3.2816587  0.37326998 0.47168266 2.1483307  1.83805268 0.02999752], Cost: 0.7545
Current parameters: [2.5493609  1.8730982  1.14828162 2.70901721 0.4357638  2.49859854
 0.14868245 2.4294332  2.93589655 1.50533215 1.11534406 2.67606645
 2.79908126 0.44274981 0.49625002 2.17355436 1.90260098 0.0852818 ], Cost: 0.3511
Current parameters: [2.54366427 2.1133818  1.1511668  2.72010621 0.4323793  2.93326276
 0.14194342 2.41852308 2.93461914 1.50423028 1.11482094 2.68968952
 2.81136707 0.41171549 0.4852766  2.16228779 1.87376941 0.06058816], Cost: 0.3930
Current parameters: [2.5493609  1.8730982  1.37810466 2.70901721 0.4357638  2.49859854
 0.24707642 2.4294332  2.93589655 1.50533215 1.11534406 2.67606645
 2.79908126 0.44274981 0.49625002 2.17355436 1.90260098 0.0852818 ], Cost: 0.5674
Current parameters: [2.54671881 1.87429386 0.94673528 2.71416029 0.43419407 2.50224762
 0.60547496 2.42437308 2.93530409 1.50482111 1.11510144 2.68238483
 2.80477942 0.42835609 0.49116055 2.16832893 1.8892289  0.07382889], Cost: 0.2188
Current parameters: [2.54671881 1.87429386 0.94673528 2.53882678 0.43419407 2.50224762
 0.60547496 2.24616489 2.93530409 1.50482111 1.11510144 2.68238483
 2.80477942 0.42835609 0.49116055 2.16832893 1.8892289  0.07382889], Cost: 0.5242
Current parameters: [2.5409265  1.87691513 0.93258711 2.37232685 0.43075273 2.51024754
 0.6078993  2.7845607  2.93400523 1.50370073 1.11456954 2.69623667
 2.81727155 0.39680061 0.48000285 2.15687316 1.85991315 0.04872056], Cost: 0.9812
Current parameters: [2.54671881 1.87429386 0.94673528 2.71416029 0.18566761 2.50224762
 0.60547496 2.42437308 2.93530409 1.50482111 1.11510144 2.68238483
 2.80477942 0.45545958 0.49116055 2.16832893 1.8892289  0.07382889], Cost: 0.3592
Current parameters: [2.53403497 1.88003386 0.91575397 2.76001332 0.5038851  2.51976563
```



```
 0.61078372 2.43152597 2.93245987 1.50236774 1.11393669 2.71271725
 2.83213437 0.90944521 0.46672769 2.14324337 1.82503401 0.0188473 ], Cost: 2.2705
Current parameters: [2.54671881 1.87429386 0.94673528 2.71416029 0.43419407 2.50224762
 0.60547496 2.42437308 2.68554911 1.50482111 1.11510144 2.68238483
 2.80477942 0.42835609 0.49116055 2.16832893 1.90029455 0.07382889], Cost: 0.2820
Current parameters: [2.53537289 1.87942839 0.91902194 2.75517665 0.44194948 2.5179178
 0.61022374 2.43077147 2.96375704 1.50262653 1.11405955 2.70951772
 2.82924892 0.35818443 0.46930492 2.14588944 2.37559378 0.02464687], Cost: 0.7208
Current parameters: [2.54671881 1.87429386 0.94673528 2.71416029 0.43419407 2.50224762
 0.60547496 2.42437308 2.93530409 1.25506962 1.11510144 2.68238483
 2.80477942 0.42835609 0.49116055 2.16832893 1.8892289  0.08497318], Cost: 0.4383
Current parameters: [2.53594328 1.87917026 0.92041517 2.75311465 0.44155959 2.51713002
 0.60998501 2.4304498  2.9419734  1.54939012 1.11411193 2.70815368
 2.82801877 0.36171216 0.47040366 2.14701754 1.89176436 0.56244745], Cost: 1.2020
Current parameters: [2.54671881 1.87429386 0.94673528 2.71416029 0.43419407 2.50224762
 0.60547496 2.42437308 2.93530409 1.50482111 0.86528555 2.67279205
 2.80477942 0.42835609 0.49116055 2.16832893 1.8892289  0.07382889], Cost: 1.0081
Current parameters: [2.54082086 1.87696294 0.93232906 2.73548182 0.43822556 2.51039345
 0.60794352 2.42769915 2.93895452 1.51669661 1.17883279 2.18938529
 2.81749939 0.39187877 0.47979934 2.15666421 1.89061668 0.06070881], Cost: 1.3949
Current parameters: [2.54671881 1.87429386 0.94673528 2.71416029 0.43419407 2.50224762
 0.60547496 2.42437308 2.93530409 1.50482111 1.11510144 2.68238483
 2.80477942 0.42835609 0.31206934 2.3427604  1.8892289  0.07382889], Cost: 0.1783
Current parameters: [2.54007523 1.87730037 0.93050779 2.73817733 0.43873523 2.51142326
 0.6082556  2.42811964 2.93941601 1.51819794 1.16425358 2.71655431
 2.81910748 0.38726723 0.65421694 2.69775523 1.89079212 0.05905015], Cost: 2.7220
Current parameters: [2.54529245e+00 1.86690834e+00 9.34122427e-01 2.80923437e+00
 5.03921284e-01 2.48898704e+00 6.34935333e-01 2.44052153e+00
 3.01018578e+00 1.57991653e+00 1.19087096e+00 2.74691528e+00
 2.76653115e+00 3.46506864e-01 3.48837612e-01 2.26844708e+00
 1.81283203e+00 2.73762699e-03], Cost: 0.1823
Current parameters: [ 2.51596394  1.85145991  0.9283835   2.69133223  0.30056821  2.47502525
  0.52025649  2.3424016   2.80317622  1.40713699  1.1501037   2.7686874
  2.68199687  0.24444225 -0.02069332  2.22331559  1.86394023  0.00393091], Cost: 2.4130
Current parameters: [2.54118904 1.8772283  0.93076574 2.74566413 0.4771855  2.51070418
 0.61313587 2.43174754 2.94957263 1.52688637 1.16728089 2.71619426
 2.81892814 0.65236782 0.2745155  2.29173377 1.88839689 0.05872704], Cost: 0.3241
Current parameters: [2.54713241 1.81391691 0.94620924 2.71213851 0.43481114 2.39384182
 0.60670364 2.42636225 2.93553699 1.505022   1.11519681 2.67990103
 2.8158704  0.43401436 0.31407004 2.34481455 1.89448556 0.0783311 ], Cost: 0.2789
Current parameters: [2.55415638 1.88326619 0.94648719 2.72771444 0.43031132 2.51916363
```



```
 0.59855714 2.4138765  2.93286184 1.50452503 1.12299831 2.70144095
 3.04530708 0.39485055 0.28855339 2.32247743 1.86302073 0.04809668], Cost: 0.1180
Current parameters: [2.66503149 1.88326619 0.89356107 2.72771444 0.43031132 2.51916363
 0.57589792 2.4138765  2.93286184 1.50452503 1.12299831 2.70144095
 3.04117232 0.39485055 0.28855339 2.32247743 1.86302073 0.04809668], Cost: 0.1099
Current parameters: [2.77993721 1.88488384 1.09529091 2.72849043 0.4304042  2.52311421
 0.66551456 2.41679611 2.93162965 1.50581111 1.13403623 2.70551382
 3.04041732 0.39842335 0.27467831 2.313241   1.87272188 0.05214314], Cost: 0.7548
Current parameters: [2.6649823  1.77434362 0.89339588 2.72864782 0.43093191 2.57990875
 0.57602369 2.41388017 2.93347617 1.50514324 1.12396691 2.69334474
 3.04127841 0.39447887 0.28892944 2.32192158 1.86203817 0.04720758], Cost: 0.1219
Current parameters: [2.65681972 1.90294619 0.87326384 2.75701493 0.44906651 2.51893858
 0.57867529 2.41899838 2.94915505 1.52525714 1.17097026 2.46583252
 3.05066258 0.38984667 0.27600309 2.28996569 1.85023221 0.0284013 ], Cost: 0.2256
Current parameters: [2.66472039 1.88340698 0.89313191 2.79815142 0.42874705 2.51959331
 0.57525561 2.34276063 2.93169061 1.50656265 1.12167682 2.70213528
 3.04184326 0.39244144 0.28702723 2.32276492 1.86581767 0.12278657], Cost: 0.3509
Current parameters: [2.65319496 1.88981559 0.86308496 2.64877285 0.45789667 2.53222907
 0.58331371 2.54114865 2.96053901 1.54500983 1.17228586 2.71415514
 3.05865668 0.36689308 0.27731686 2.27499776 1.85149374 0.21948762], Cost: 0.4074
Current parameters: [2.66448428 1.88351383 0.93515747 2.73832116 0.44406462 2.5199194
 0.47584649 2.42341051 2.94935819 1.51864004 1.13717258 2.70329577
 3.04235247 0.38965966 0.29769676 2.31146306 1.91374506 0.0450901 ], Cost: 0.1736
Current parameters: [2.65762456 1.88698639 0.83388452 2.74450764 0.43951665 2.52838029
 0.6757607  2.41712503 2.95411072 1.51657289 1.14104433 2.71446269
 3.05466371 0.36323238 0.28013175 2.29818067 2.07442148 0.01963566], Cost: 0.7401
Current parameters: [2.66845705 1.88692922 0.89388695 2.73527525 0.4473231  2.52431924
 0.58835166 2.42702774 2.94773249 1.51956922 1.13795369 2.6944805
 3.0606098  0.41545875 0.37887235 2.39417327 1.86705999 0.05599176], Cost: 0.6781
Current parameters: [ 2.62291331  1.8905331   0.83752534  2.68828927  0.36943462  2.52916114
  0.49485347  2.40592225  2.86181241  1.45551576  1.15543345  2.73270739
  3.00531651  0.35459463  0.15822963  2.3050124   1.76923541 -0.03599123], Cost: 0.4722
Current parameters: [2.65975856 1.88580668 0.88558738 2.73847675 0.44969204 2.52210538
 0.56644069 2.43544013 2.94906019 1.52484068 1.01173584 2.69540625
 3.04148732 0.39897092 0.27940499 2.29254974 1.84673781 0.03713297], Cost: 0.2907
Current parameters: [2.66503149 1.88326619 0.89356107 2.7039522  0.42446567 2.51916363
 0.57589792 2.38972467 2.92698729 1.55599624 1.12299831 2.70144095
 3.04117232 0.39548805 0.28434094 2.32658028 1.863281   0.04579996], Cost: 0.1470
Current parameters: [2.6629237  1.88428172 0.89037369 2.66311369 0.4734215  2.52033956
 0.57211751 2.35246374 2.97487471 1.45848267 1.1242335  2.70078393
 3.04129824 0.39264104 0.31037943 2.28569426 1.8549373  0.04613092], Cost: 0.0175
```

```
Current parameters: [2.6629237  1.88428172 0.89037369 2.66311369 0.44443958 2.52033956
 0.57211751 2.35246374 3.0256739  1.45848267 1.1242335  2.70078393
 3.04129824 0.39580171 0.2948377  2.30083161 1.85268659 0.04613092], Cost: 0.0189
Current parameters: [2.66174341 1.88485038 0.88858886 2.66098016 0.39010529 2.52099804
 0.57000061 2.3526735  2.9656959  1.45635346 1.12492517 2.70041602
 3.04136875 0.40312264 0.37175583 2.21722129 1.85185985 0.04397473], Cost: 0.0452
Current parameters: [2.66324214 1.88614753 0.89341587 2.63957126 0.45629221 2.5237115
 0.56480804 2.34847894 2.95625988 1.43986803 1.1056574  2.68489019
 3.05095371 0.41463489 0.30093292 2.30391439 1.87402312 0.06379279], Cost: 0.0498
Current parameters: [2.65890089 1.88621079 0.88040652 2.67773636 0.49543855 2.52495285
 0.5740139  2.3565657  2.98247801 1.46816937 1.15170968 2.71795201
 3.04759498 0.501778   0.28748416 2.25506728 1.84937176 0.03515624], Cost: 0.1919
Current parameters: [2.66476972 1.87773894 0.89023832 2.66607801 0.47257779 2.50878579
 0.57063292 2.35022912 2.97432165 1.4584405  1.126144   2.70500322
 3.10066844 0.38540845 0.30501934 2.2811184  1.84939961 0.04059888], Cost: 0.0447
Current parameters: [2.66048395 1.82553092 0.88746101 2.65528025 0.47352737 2.41406735
 0.57088668 2.35614759 2.97446815 1.455047   1.12311039 2.69428921
 3.01557098 0.39869046 0.30949097 2.28535383 1.85811647 0.05100475], Cost: 0.0843
Current parameters: [2.68356622 1.88423032 0.93481697 2.65934531 0.47231286 2.51948373
 0.56972579 2.35420405 2.97133814 1.45721603 1.12401155 2.69627214
 3.03905519 0.39858039 0.30923729 2.28756379 1.81819281 0.05321862], Cost: 0.0096
Current parameters: [2.71877942 1.88723933 0.9825328  2.64899971 0.46991317 2.52414788
 0.62858009 2.35418883 2.97350434 1.44342721 1.13251812 2.70231046
 3.02925518 0.36905107 0.27188552 2.25173673 1.88008043 0.02637978], Cost: 0.1459
Current parameters: [2.68393393 1.8834194  0.93884724 2.67660619 0.4634721  2.51747753
 0.56298811 2.32444102 2.96169202 1.44596146 1.11580017 2.69452992
 3.03425054 0.40418611 0.31088828 2.30317514 1.81578451 0.00871093], Cost: 0.0093
Current parameters: [ 2.66204955  1.89051247  0.90888477  2.64273899  0.442947    2.52899686
  0.52434846  2.33188713  2.9373623   1.41435536  1.14724468  2.71029657
  3.0121359   0.36096859  0.25717667  2.29041037  1.76502313 -0.00307683], Cost: 0.1946
Current parameters: [2.6862588  1.87886832 0.94386029 2.66970085 0.45966922 2.51770692
 0.56290647 2.32383404 2.95839289 1.44156712 1.10462593 2.75278744
 3.03287166 0.40647621 0.31858922 2.31489771 1.81917143 0.01401106], Cost: 0.0698
Current parameters: [ 2.6884072   1.89416226  0.93561002  2.65266219  0.48557492  2.52637458
  0.58345895  2.35125852  2.98328086  1.43790018  1.14298761  2.65775876
  3.03922984  0.39291597  0.39419985  2.35762166  1.80757415 -0.01257434], Cost: 0.3090
Current parameters: [2.67522715 1.8910603  0.92085635 2.694901   0.49322989 2.53132852
 0.56688572 2.30293633 2.95548743 1.44911824 1.11760225 2.69995092
 3.0097042  0.38158565 0.32181586 2.29595292 1.82576581 0.01835808], Cost: 0.0145
Current parameters: [2.69592768 1.87847994 0.95942091 2.72921095 0.42792189 2.50344721
 0.56373447 2.26389113 2.97356004 1.42990694 1.12006262 2.67456861
```



```
 3.05832314 0.41019893 0.29941136 2.30914522 1.7993581  0.08131015], Cost: 0.0439
Current parameters: [2.68299362 1.88511798 0.93762197 2.68323968 0.47025603 2.51812295
 0.55871967 2.33079288 2.97363065 1.45037571 1.06058725 2.68464105
 3.0354144  0.40156707 0.30594317 2.28710595 1.80349646 0.00736301], Cost: 0.0430
Current parameters: [2.68392073 1.91019219 0.94081017 2.67686704 0.46395522 2.5025879
 0.55833343 2.32488073 2.96230344 1.44646189 1.11621724 2.69660481
 3.03427901 0.40403753 0.31121844 2.30280269 1.8183701  0.00879043], Cost: 0.0116
Current parameters: [2.6843139  1.87343254 0.960355   2.68255238 0.46944644 2.523315
 0.5135395  2.3277082  2.97158265 1.45103341 1.12175659 2.69196555
 3.03523332 0.39982932 0.31519813 2.29689372 1.83900572 0.00860118], Cost: 0.0058
Current parameters: [2.71186902 1.87343254 0.9472016  2.68126321 0.4691293  2.523315
 0.50790814 2.32639788 2.97126394 1.45382588 1.12175659 2.69196555
 3.03420573 0.39986391 0.31496959 2.29711632 1.83901984 0.00847657], Cost: 0.0063
Current parameters: [2.6788901  1.87315733 0.96508285 2.65927554 0.46264199 2.52327238
 0.51333436 2.30153628 2.96786912 1.50125017 1.12059608 2.6886889
 3.03595079 0.39828838 0.3107531  2.29996049 1.83789442 0.0079727 ], Cost: 0.0126
Current parameters: [2.68398179 1.87337046 0.95977855 2.6689625  0.47974724 2.52316088
 0.51257937 2.3143333  2.98243294 1.4412104  1.12450225 2.6931691
 3.03751619 0.41174345 0.31709346 2.28588296 1.83598023 0.00753193], Cost: 0.0140
Current parameters: [2.6805752  1.87654865 0.9537507  2.70994906 0.4780193  2.53093814
 0.51368546 2.33897441 2.96569381 1.47061139 1.13441649 2.69479018
 3.02967876 0.4431228  0.30006144 2.28714679 1.8390846  0.00482249], Cost: 0.0341
Current parameters: [2.68542271 1.86935673 0.96263447 2.68148995 0.48716984 2.51600982
 0.51347331 2.32769291 2.97492141 1.45044244 1.12077463 2.69092968
 3.03652408 0.39876712 0.29918359 2.31448132 1.83886142 0.01026517], Cost: 0.0072
Current parameters: [2.67981916 1.84645268 0.95283464 2.68436163 0.46737497 2.4750179
 0.51332809 2.32945074 2.9668524  1.45364592 1.12352987 2.68887824
 3.01369102 0.40122964 0.32413939 2.28452882 1.84318481 0.00893812], Cost: 0.0259
Current parameters: [2.69126301 1.87324627 0.97056294 2.68264746 0.46689778 2.5235173
 0.52408739 2.32522701 2.96997777 1.44882158 1.12150981 2.69485835
 3.03267499 0.39430298 0.29888958 2.28366676 1.85279902 0.00461159], Cost: 0.0115
Current parameters: [ 2.69732282e+00  1.87716190e+00  9.78373294e-01  2.68331983e+00
  4.76426477e-01  2.52950118e+00  5.35035684e-01  2.33758587e+00
  2.98670915e+00  1.45532655e+00  1.13518794e+00  2.66328856e+00
  3.03739730e+00  3.94218683e-01  3.45807136e-01  2.31315415e+00
  1.85160323e+00 -1.64734740e-03], Cost: 0.0721
Current parameters: [ 2.6802825   1.87528911  0.95401705  2.6686683   0.4763803   2.52728816
  0.51089555  2.34243634  2.96803203  1.45312119  1.12363986  2.69448069
  3.02771859  0.39549427  0.31389717  2.29402827  1.83854753 -0.00939709], Cost: 0.0122
Current parameters: [2.67771422 1.8763599  0.95485354 2.68378628 0.45019615 2.52911484
 0.49387817 2.31972556 2.9658703  1.43767685 1.14384055 2.67788042
```



```
 3.03056878 0.37916905 0.28975491 2.29453553 1.80965861 0.01899042], Cost: 0.0230
Current parameters: [2.68114892 1.87544587 0.95568392 2.67304834 0.46555241 2.52723673
 0.51006575 2.32595917 2.95685013 1.4431887  1.11484949 2.68225587
 3.03067319 0.40842304 0.31364935 2.30326015 1.85254885 0.01654539], Cost: 0.0053
Current parameters: [2.68423853 1.87509899 0.96212264 2.67265558 0.47105472 2.52651322
 0.50964185 2.32609565 2.94630559 1.44300281 1.11491641 2.6818204
 3.03342789 0.40828686 0.31626597 2.30031319 1.84737559 0.01728635], Cost: 0.0064
Current parameters: [2.67551253 1.87316022 0.94258296 2.67576643 0.46740261 2.5237137
 0.50900454 2.32434518 2.95176435 1.44331552 1.11713678 2.6815232
 3.05489327 0.40282579 0.31265201 2.29973272 1.86065771 0.01305284], Cost: 0.0093
Current parameters: [2.68163403 1.87454319 0.95656086 2.67081908 0.46363063 2.5271028
 0.51094235 2.32459319 2.95397323 1.44174241 1.12510265 2.69113739
 3.03031226 0.4092818  0.31556698 2.30801373 1.85550082 0.01742504], Cost: 0.0124
Current parameters: [2.67990189 1.87437321 0.95583299 2.67384151 0.46149831 2.5304939
 0.50358162 2.32714733 2.9577878  1.4415806  1.09088132 2.6984032
 3.03293422 0.40537866 0.31266882 2.30490271 1.84818753 0.02173858], Cost: 0.0139
Current parameters: [2.68356966 1.87361601 0.96047192 2.66847749 0.45831818 2.52381677
 0.50956802 2.3314469  2.9586888  1.4424394  1.11466041 2.68039622
 3.03678791 0.41400572 0.31144659 2.30527524 1.85030085 0.01394482], Cost: 0.0070
Current parameters: [2.68548958 1.87883609 0.96160866 2.67729692 0.46807722 2.5344345
 0.51719794 2.32789649 2.96489992 1.44310589 1.11041978 2.66393058
 3.03183184 0.39937686 0.32767565 2.31109975 1.85685377 0.01689408], Cost: 0.0228
Current parameters: [2.68122965 1.87525514 0.9566689  2.67744564 0.46337085 2.52675755
 0.50838528 2.31855177 2.95442207 1.44043244 1.11283627 2.68182977
 3.02945524 0.40996155 0.31401524 2.30713056 1.85194482 0.00540751], Cost: 0.0042
Current parameters: [2.67868047 1.87806011 0.95250497 2.69390151 0.46690468 2.53370123
 0.50698404 2.32391342 2.95221178 1.44977942 1.11428621 2.68002056
 3.02752584 0.43023492 0.30669494 2.30321412 1.85077669 0.00541421], Cost: 0.0087
Current parameters: [2.68098955 1.86071567 0.95526936 2.67741112 0.46303568 2.5312974
 0.51068094 2.31843898 2.95386787 1.44028786 1.11273133 2.68065208
 3.02823723 0.41013198 0.31435286 2.30661308 1.85089654 0.00538712], Cost: 0.0024
Current parameters: [2.67793626 1.85307233 0.95085578 2.68191455 0.46156985 2.50574142
 0.50829919 2.31965897 2.95177446 1.4419685  1.11000306 2.67682389
 3.01740326 0.41092259 0.3188167  2.30077333 1.85267981 0.00683874], Cost: 0.0046
Current parameters: [2.68177964 1.86102675 0.9537294  2.68262002 0.46335239 2.5317841
 0.50928339 2.32389244 2.95438661 1.4273144  1.1147885  2.68038638
 3.02762822 0.4089358  0.31320666 2.3054417  1.84891792 0.00610321], Cost: 0.0055
Current parameters: [2.67598985 1.8610438  0.95220122 2.67497731 0.45330178 2.53381589
 0.50102954 2.30931132 2.95079734 1.44484182 1.11877963 2.6708287
 3.02476734 0.40086366 0.30103635 2.30468661 1.83690819 0.00994865], Cost: 0.0086
Current parameters: [2.68104802 1.85826686 0.96046945 2.67745529 0.46556552 2.53270901
```



```
 0.49848825 2.31784228 2.95741248 1.44050906 1.1144729  2.68014981
 3.02871444 0.41030265 0.31560352 2.30393449 1.85626157 0.00524934], Cost: 0.0025
Current parameters: [2.67954985 1.86081459 0.95131369 2.66517431 0.47230952 2.53024659
 0.51418315 2.30368226 2.96379328 1.43213721 1.11129165 2.67961338
 3.02970692 0.42320807 0.31627458 2.29606198 1.84490224 0.00534549], Cost: 0.0067
Current parameters: [ 2.67792337e+00  1.86229859e+00  9.50000218e-01  2.67091183e+00
  4.67041186e-01  2.53395868e+00  5.09510393e-01  2.32558442e+00
  2.95395564e+00  1.44183866e+00  1.11231379e+00  2.67995971e+00
  3.02225766e+00  4.07860136e-01  3.12893983e-01  2.30402944e+00
  1.85180455e+00 -3.53041094e-04], Cost: 0.0021
Current parameters: [ 2.68011277  1.8568553   0.95729931  2.66770595  0.46598017  2.53042239
  0.50629369  2.32720895  2.94332799  1.44402642  1.11204142  2.68589883
  3.03142601  0.41022738  0.31860503  2.30830176  1.84316619 -0.02234701], Cost: 0.0221
Current parameters: [ 2.67792337e+00  1.86229859e+00  9.50000218e-01  2.67091183e+00
  4.67041186e-01  2.53395868e+00  5.09510393e-01  2.32558442e+00
  2.95395564e+00  1.44183866e+00  1.11231379e+00  2.67995971e+00
  3.02225766e+00  4.07860136e-01  3.12893983e-01  2.30402944e+00
  1.85180455e+00 -3.53041094e-04], Cost: 0.0021

   Return from subroutine COBYLA because the MAXFUN limit has been reached.

   NFVALS =  100   F = 2.139114E-03    MAXCV = 0.000000E+00
   X = 2.677923E+00   1.862299E+00   9.500002E-01   2.670912E+00   4.670412E-01
       2.533959E+00   5.095104E-01   2.325584E+00   2.953956E+00   1.441839E+00
       1.112314E+00   2.679960E+00   3.022258E+00   4.078601E-01   3.128940E-01
       2.304029E+00   1.851805E+00  -3.530411E-04
Simulating for QAOA depth p = 10
Current parameters: [3.74504799 2.12856801 2.9840405  0.46246307 2.94922167 0.71190658
 2.87735093 1.01812127 2.03156207 1.01649773 1.58433724 0.50063513
 2.36430258 2.62480908 2.62492652 0.45296495 0.04802359 2.26146411
 0.7264756  2.95695793], Cost: 1.6918
Current parameters: [3.74504799 3.12856801 2.9840405  0.46246307 2.94922167 0.71190658
 2.87735093 1.01812127 2.03156207 1.01649773 1.58433724 0.50063513
 2.36430258 2.62480908 2.62492652 0.45296495 0.04802359 2.26146411
 0.7264756  2.95695793], Cost: 1.2867
Current parameters: [3.74504799 3.12856801 3.9840405  0.46246307 2.94922167 0.71190658
 2.87735093 1.01812127 2.03156207 1.01649773 1.58433724 0.50063513
 2.36430258 2.62480908 2.62492652 0.45296495 0.04802359 2.26146411
 0.7264756  2.95695793], Cost: 2.0110
Current parameters: [3.74504799 3.12856801 2.9840405  1.46246307 2.94922167 0.71190658
 2.87735093 1.01812127 2.03156207 1.01649773 1.58433724 0.50063513
```

```
 2.36430258 2.62480908 2.62492652 0.45296495 0.04802359 2.26146411
 0.7264756  2.95695793], Cost: 2.0695
Current parameters: [3.74504799 3.12856801 2.9840405  0.46246307 3.94922167 0.71190658
 2.87735093 1.01812127 2.03156207 1.01649773 1.58433724 0.50063513
 2.36430258 2.62480908 2.62492652 0.45296495 0.04802359 2.26146411
 0.7264756  2.95695793], Cost: 2.5073
Current parameters: [3.74504799 3.12856801 2.9840405  0.46246307 2.94922167 1.71190658
 2.87735093 1.01812127 2.03156207 1.01649773 1.58433724 0.50063513
 2.36430258 2.62480908 2.62492652 0.45296495 0.04802359 2.26146411
 0.7264756  2.95695793], Cost: 0.2624
Current parameters: [3.74504799 3.12856801 2.9840405  0.46246307 2.94922167 1.71190658
 3.87735093 1.01812127 2.03156207 1.01649773 1.58433724 0.50063513
 2.36430258 2.62480908 2.62492652 0.45296495 0.04802359 2.26146411
 0.7264756  2.95695793], Cost: 1.1134
Current parameters: [3.74504799 3.12856801 2.9840405  0.46246307 2.94922167 1.71190658
 2.87735093 2.01812127 2.03156207 1.01649773 1.58433724 0.50063513
 2.36430258 2.62480908 2.62492652 0.45296495 0.04802359 2.26146411
 0.7264756  2.95695793], Cost: 1.1088
Current parameters: [3.74504799 3.12856801 2.9840405  0.46246307 2.94922167 1.71190658
 2.87735093 1.01812127 3.03156207 1.01649773 1.58433724 0.50063513
 2.36430258 2.62480908 2.62492652 0.45296495 0.04802359 2.26146411
 0.7264756  2.95695793], Cost: 2.4816
Current parameters: [3.74504799 3.12856801 2.9840405  0.46246307 2.94922167 1.71190658
 2.87735093 1.01812127 2.03156207 2.01649773 1.58433724 0.50063513
 2.36430258 2.62480908 2.62492652 0.45296495 0.04802359 2.26146411
 0.7264756  2.95695793], Cost: 0.4138
Current parameters: [3.74504799 3.12856801 2.9840405  0.46246307 2.94922167 1.71190658
 2.87735093 1.01812127 2.03156207 1.01649773 2.58433724 0.50063513
 2.36430258 2.62480908 2.62492652 0.45296495 0.04802359 2.26146411
 0.7264756  2.95695793], Cost: 1.6671
Current parameters: [3.74504799 3.12856801 2.9840405  0.46246307 2.94922167 1.71190658
 2.87735093 1.01812127 2.03156207 1.01649773 1.58433724 1.50063513
 2.36430258 2.62480908 2.62492652 0.45296495 0.04802359 2.26146411
 0.7264756  2.95695793], Cost: 1.6097
Current parameters: [3.74504799 3.12856801 2.9840405  0.46246307 2.94922167 1.71190658
 2.87735093 1.01812127 2.03156207 1.01649773 1.58433724 0.50063513
 3.36430258 2.62480908 2.62492652 0.45296495 0.04802359 2.26146411
 0.7264756  2.95695793], Cost: 2.1168
Current parameters: [3.74504799 3.12856801 2.9840405  0.46246307 2.94922167 1.71190658
 2.87735093 1.01812127 2.03156207 1.01649773 1.58433724 0.50063513
 2.36430258 3.62480908 2.62492652 0.45296495 0.04802359 2.26146411
```



```
 0.7264756  2.95695793], Cost: 3.3728
Current parameters: [3.74504799 3.12856801 2.9840405  0.46246307 2.94922167 1.71190658
 2.87735093 1.01812127 2.03156207 1.01649773 1.58433724 0.50063513
 2.36430258 2.62480908 3.62492652 0.45296495 0.04802359 2.26146411
 0.7264756  2.95695793], Cost: 3.4488
Current parameters: [3.74504799 3.12856801 2.9840405  0.46246307 2.94922167 1.71190658
 2.87735093 1.01812127 2.03156207 1.01649773 1.58433724 0.50063513
 2.36430258 2.62480908 2.62492652 1.45296495 0.04802359 2.26146411
 0.7264756  2.95695793], Cost: 0.6166
Current parameters: [3.74504799 3.12856801 2.9840405  0.46246307 2.94922167 1.71190658
 2.87735093 1.01812127 2.03156207 1.01649773 1.58433724 0.50063513
 2.36430258 2.62480908 2.62492652 0.45296495 1.04802359 2.26146411
 0.7264756  2.95695793], Cost: 1.2895
Current parameters: [3.74504799 3.12856801 2.9840405  0.46246307 2.94922167 1.71190658
 2.87735093 1.01812127 2.03156207 1.01649773 1.58433724 0.50063513
 2.36430258 2.62480908 2.62492652 0.45296495 0.04802359 3.26146411
 0.7264756  2.95695793], Cost: 1.3460
Current parameters: [3.74504799 3.12856801 2.9840405  0.46246307 2.94922167 1.71190658
 2.87735093 1.01812127 2.03156207 1.01649773 1.58433724 0.50063513
 2.36430258 2.62480908 2.62492652 0.45296495 0.04802359 2.26146411
 1.7264756  2.95695793], Cost: 1.7003
Current parameters: [3.74504799 3.12856801 2.9840405  0.46246307 2.94922167 1.71190658
 2.87735093 1.01812127 2.03156207 1.01649773 1.58433724 0.50063513
 2.36430258 2.62480908 2.62492652 0.45296495 0.04802359 2.26146411
 0.7264756  3.95695793], Cost: 1.5849
Current parameters: [ 3.86295702  3.18959192  2.87492879  0.34453546  2.76534601  1.86620277
  2.74915884  0.89062013  1.69726249  0.99369831  1.37273899  0.29767568
  2.0849509   2.15625823  2.14492527  0.39961679 -0.10669632  2.09822899
  0.50987583  2.75774034], Cost: 1.8258
Current parameters: [ 3.80400251  3.15907997  2.92948465  0.40349926  2.85728384  1.78905467
  2.81325488  0.9543707   1.86441228  1.00509802  1.47853811  0.3991554
  2.22462674  2.39053365  2.38492589  0.42629087 -0.02933636  2.17984655
  0.61817571  2.85734913], Cost: 3.0944
Current parameters: [3.98081307 3.12856801 2.9840405  0.46246307 2.94922167 1.71190658
 2.87735093 1.01812127 2.11471752 1.01649773 1.58433724 0.50063513
 2.36430258 2.62480908 2.62492652 0.45296495 0.04802359 2.26146411
 0.7264756  2.95695793], Cost: 0.6013
Current parameters: [3.56246616 3.13471149 2.97305587 0.45059091 2.9307103  1.72744008
 2.86444541 1.00528532 2.48741548 1.01420244 1.56303495 0.48020254
 2.33617933 2.57763852 2.57660322 0.44759421 0.03244743 2.24503069
 0.70466979 2.93690204], Cost: 0.2767
```



```
Current parameters: [3.74504799 3.34676154 3.10607159 0.46246307 2.94922167 1.71190658
 2.87735093 1.01812127 2.03156207 1.01649773 1.58433724 0.50063513
 2.36430258 2.62480908 2.62492652 0.45296495 0.04802359 2.26146411
 0.7264756  2.95695793], Cost: 0.7349
Current parameters: [3.73591984 2.87586077 3.41396463 0.45803065 2.94231052 1.71770595
 2.8725327  1.01332902 2.0343665  1.01564079 1.57638412 0.49300671
 2.35380288 2.60719814 2.60688521 0.45095981 0.0422083  2.25532876
 0.71833449 2.94947015], Cost: 0.9477
Current parameters: [3.74504799 3.12856801 2.9840405  0.67290238 2.8142576  1.71190658
 2.87735093 1.01812127 2.03156207 1.01649773 1.58433724 0.50063513
 2.36430258 2.62480908 2.62492652 0.45296495 0.04802359 2.26146411
 0.7264756  2.95695793], Cost: 0.3627
Current parameters: [3.72891839 3.11713311 2.96575245 0.72665746 3.36859516 1.72215417
 2.86883704 1.00965328 2.03651755 1.01498351 1.57028393 0.48715557
 2.34574942 2.59369024 2.59304721 0.44942183 0.03774786 2.25062284
 0.71209011 2.94372688], Cost: 1.4789
Current parameters: [3.74231726 3.12715472 2.98656749 0.46519423 2.95348016 1.46210942
 2.87735093 1.01812127 2.03930434 1.01649773 1.58433724 0.50063513
 2.36430258 2.62480908 2.62492652 0.45296495 0.04802359 2.26146411
 0.7264756  2.95695793], Cost: 0.2393
Current parameters: [ 3.68696199  3.07794844  2.8166124   0.32493215  2.78429167  1.44714779
  2.8206552   0.96173113  1.9247156   1.00641419  1.49075334  0.41087192
  2.24075326  2.41758233  2.41263558  0.42937057 -0.02040464  2.18926985
  0.63067967  2.86884964], Cost: 2.4745
Current parameters: [3.70930362 3.10094151 2.94935124 0.4202296  2.95939852 1.50200563
 2.86708983 1.00786018 2.0095092  1.00623664 1.57407615 0.49037404
 2.59568567 2.61454798 2.61466543 0.44270385 0.0377625  2.25120302
 0.71621451 2.94669684], Cost: 0.1805
Current parameters: [3.84010158 3.21062864 3.10154057 0.63696729 2.87905937 1.26420118
 2.89540718 1.03634944 2.21000419 1.06077625 1.58164159 0.50008886
 2.77702727 2.55818223 2.55545075 0.48964278 0.05947958 2.27080147
 0.72253554 2.95734265], Cost: 0.2842
Current parameters: [3.71010101 3.10157465 2.95025014 0.42131565 2.95925557 1.501042
 2.86733767 1.00810802 2.01022885 1.00648448 1.57432399 0.49062188
 2.59563692 2.61479582 2.61491327 0.4429517  0.03801034 2.25145086
 0.88180129 2.7594112 ], Cost: 0.6609
Current parameters: [3.68393969 3.08282878 2.92240585 0.39909329 2.94417712 1.50495006
 2.85491973 0.99574981 2.01526706 1.0031782  1.55469521 0.47173996
 2.60818451 2.57295235 2.57207985 0.43700434 0.02329895 2.23600329
 1.06833563 3.29078021], Cost: 0.4289
Current parameters: [3.70970226 3.10125805 2.94980063 0.42077256 2.95932705 1.50152387
```



```
 2.86721374 1.00798409 2.00986898 1.00636054 1.74549014 0.30839603
 2.5956613  2.61467189 2.61478933 0.44282776 0.03788641 2.25132692
 0.71619561 2.94667946], Cost: 0.1852
Current parameters: [3.68225076 3.08160672 2.92059863 0.39758019 2.94331935 1.50536448
 2.85414313 0.99497672 2.01542468 1.00293652 1.93176911 0.82733298
 2.6089305  2.57044826 2.56951765 0.43660761 0.02238771 2.23504882
 0.70127147 2.96913303], Cost: 2.0083
Current parameters: [3.70911759 3.10079381 2.94914153 0.41997623 2.95943187 1.50223044
 2.86703201 1.00780236 2.00934131 1.00617882 1.57408476 0.4903823
 2.59569705 2.79370656 2.44030391 0.44264604 0.03770468 2.2511452
 0.71622333 2.94670495], Cost: 0.2159
Current parameters: [3.67953339 3.08464604 2.92175993 0.42826729 2.89311084 1.43759356
 2.8423055  0.98329228 2.08644222 1.01446559 1.47592319 0.3997164
 2.63717286 2.92315559 2.94127741 0.44136369 0.00466857 2.21544177
 0.66209768 3.02074104], Cost: 3.4539
Current parameters: [3.71886811 3.10851648 2.95998961 0.43525642 2.96096347 1.49060257
 2.87004085 1.0108112  2.01787963 1.00918766 1.57353932 0.48985913
 2.59497695 2.61335926 2.61344765 0.19418511 0.04071352 2.25415404
 0.71566499 2.94619142], Cost: 0.4588
Current parameters: [3.67466317 3.07370605 2.89367393 0.6550133  3.36992844 1.52581166
 2.84749936 0.98835275 1.98362741 0.99931327 1.52512834 0.44476296
 2.59670335 2.5382866  2.5399191  0.47311741 0.01498366 2.22740074
 0.68389644 2.96448096], Cost: 0.7482
Current parameters: [3.70877327 3.10051884 2.94874164 0.41967073 2.95976163 1.50265924
 2.86692322 1.00769357 2.00900917 1.00607002 1.57409304 0.49039024
 2.59570797 2.61458538 2.61470373 0.44270811 0.22067822 2.08079234
 0.71623179 2.94671274], Cost: 0.7892
Current parameters: [3.67985584 3.07911248 2.91673456 0.40174827 2.96601733 1.51577929
 2.85431797 0.99513713 2.00494864 1.00091081 1.55249003 0.47045593
 2.60577164 2.58563328 2.58703021 0.45770832 0.35443029 2.62954319
 0.70457363 2.9639169 ], Cost: 0.5896
Current parameters: [3.70936167 3.10098778 2.94941797 0.42029077 2.95935877 1.50193408
 3.04320045 0.83041998 2.00956393 1.00625488 1.5740743  0.49037227
 2.59568323 2.61454389 2.61466123 0.44270339 0.03776115 2.25120159
 0.71621261 2.9466951 ], Cost: 0.1748
Current parameters: [3.67885175 3.07792769 2.9152917  0.39774864 2.9698791  1.52229458
 3.39391538 1.17819857 1.99856357 0.99967527 1.55780103 0.47536572
 2.60374007 2.59296762 2.59407829 0.4543087  0.02231916 2.26352592
 0.70755966 2.96011794], Cost: 0.4826
Current parameters: [3.71939427 3.09554269 2.94352323 0.41506436 2.96534245 1.50788377
 3.04459109 0.83180312 1.9581811  1.2501886  1.57636974 0.49257399
```

```
 2.59871366 2.61962677 2.61986833 0.44328211 0.03943957 2.25297238
 0.71856231 2.94885623], Cost: 0.2200
Current parameters: [3.92794299 3.13764516 3.02195997 0.49330086 2.9363281  1.41166388
 3.05502925 0.84205834 1.59947486 0.91432031 1.58478247 0.50096539
 2.62561846 2.6459877  2.64768668 0.45380379 0.04369613 2.27276705
 0.73145492 2.97214891], Cost: 0.1715
Current parameters: [3.89657887e+00 3.00050600e+00 3.19545067e+00 4.48888149e-01
 2.93192277e+00 1.44850611e+00 3.02754552e+00 8.61058432e-01
 1.58315539e+00 9.12838114e-01 1.54613130e+00 5.09521294e-01
 2.64710091e+00 2.60512820e+00 2.65288349e+00 4.83644157e-01
 3.25378298e-03 2.27770018e+00 6.92476753e-01 2.98116068e+00], Cost: 0.2332
Current parameters: [3.94180902 3.05143933 3.2083638  0.60250163 3.00515329 1.35005194
 3.19312042 0.72249373 1.64960125 0.92434271 1.72257426 0.38282562
 2.50170577 2.75035198 2.49936723 0.32023729 0.20038963 2.26698202
 0.89500877 2.94334796], Cost: 1.0901
Current parameters: [ 3.93698576  3.15222844  2.97300917  0.46406648  2.85710164  1.38055367
  3.0433183   0.81070411  1.57190588  0.91405642  1.52281628  0.42414357
  2.58529697  2.52526321  2.50825804  0.45981615 -0.00515033  2.2707684
  0.68713738  2.96456183], Cost: 2.3789
Current parameters: [3.93204091 3.14245495 3.02676724 0.49935957 2.93365249 1.40536112
 3.05674791 0.84071001 1.60753153 0.91611968 1.50598741 0.42389187
 2.62547701 2.68722433 2.68688999 0.45300596 0.04581929 2.27173915
 0.73503017 2.97246586], Cost: 0.2355
Current parameters: [ 3.90514367e+00  3.11267086e+00  3.01503006e+00  5.13594445e-01
  2.95403543e+00  1.39487888e+00  3.05095713e+00  8.41948138e-01
  1.62369488e+00  9.08013392e-01  1.66502402e+00  5.81869525e-01
  2.65789552e+00  2.77971211e+00  2.79426297e+00  4.80626837e-01
 -1.60350371e-03  2.30753079e+00  7.10737618e-01  3.01523345e+00], Cost: 2.1756
Current parameters: [3.93327746 3.13968897 3.03171328 0.44306914 2.84588268 1.4031927
 3.06012929 0.84531261 1.60402624 0.91566896 1.59636484 0.51009346
 2.62473503 2.66343598 2.66377807 0.45020104 0.09415235 2.23643661
 0.73295767 2.97176696], Cost: 0.1018
Current parameters: [3.9158251  3.12068084 3.01195517 0.48178871 2.94175375 1.42150814
 3.03475601 0.86542549 1.58226386 0.90877638 1.56645773 0.5262548
 2.64955722 2.62813093 2.67481828 0.48707508 0.23095125 2.08335504
 0.68885141 2.98774497], Cost: 0.2056
Current parameters: [3.93603367 3.14084303 3.03458294 0.44810439 2.84927336 1.40087016
 3.06488876 0.84362482 1.60345114 0.91616404 1.60147182 0.50876542
 2.6198727  2.67259849 2.66660988 0.44764333 0.13402647 2.28354928
 0.6553216  2.89725113], Cost: 0.1700
Current parameters: [3.90984051 3.12321061 3.00705407 0.41792991 2.85060251 1.42561607
```



```
 3.03236133 0.8556683  1.59397292 0.90934835 1.56436449 0.51692651
 2.65189612 2.62374598 2.66211242 0.47708884 0.22887494 2.39226314
 0.79302511 3.06528203], Cost: 0.8148
Current parameters: [3.97078463 3.16549704 3.06729662 0.49275247 2.82699334 1.35270797
 3.05913743 0.85933193 1.65407498 0.9285669  1.59063007 0.51984744
 2.67705634 2.64170918 2.65632115 0.47188176 0.09899738 2.24076179
 0.7271761  2.98198493], Cost: 0.2694
Current parameters: [3.8703655  3.33206324 3.1458707  0.3916042  2.88314916 1.46538223
 3.06615961 0.84649543 1.5830782  0.91670984 1.60123764 0.51026407
 2.62918134 2.66477539 2.66090466 0.45210569 0.09688505 2.22956718
 0.73208752 2.9744197 ], Cost: 0.1649
Current parameters: [3.99365578 3.14460572 3.03846925 0.42307683 2.85884275 1.42705195
 2.98886739 0.77325657 1.62441144 0.9170293  1.60035622 0.51252704
 2.62256984 2.66839989 2.66752987 0.44874677 0.09733121 2.23391774
 0.73325893 2.97073276], Cost: 0.1501
Current parameters: [4.08438404 3.13334629 3.02515729 0.36575906 2.8962552  1.49380655
 3.16452308 0.9431351  1.65172086 0.91263853 1.5969536  0.50522871
 2.62323506 2.66179106 2.65692648 0.45040989 0.09178681 2.23198954
 0.73264357 2.97417309], Cost: 0.1636
Current parameters: [3.93502994 3.14082025 3.03175679 0.51570047 2.79789136 1.48818176
 3.06406397 0.84185365 1.60208978 0.91592111 1.60012713 0.50646587
 2.61988617 2.66734896 2.66029743 0.44519392 0.09440409 2.23668835
 0.71584632 2.99155383], Cost: 0.1526
Current parameters: [3.92764873 3.13397576 3.02864409 0.46668655 2.82922282 1.45252106
 3.03382197 0.8732345  1.59342214 0.91195994 1.57165934 0.53966798
 2.66100893 2.63624773 2.69185459 0.49138949 0.09462269 2.22382562
 0.88193303 2.8009395 ], Cost: 0.2938
Current parameters: [3.91934451 3.12862454 3.01599546 0.4242485  2.84863635 1.42004247
 3.07732938 0.81928097 1.5914312  0.91133671 1.6127074  0.48322541
 2.72786529 2.68068778 2.63779959 0.42596853 0.08981771 2.23210196
 0.72833666 2.96714955], Cost: 0.2054
Current parameters: [3.94018284 3.14445742 3.04194007 0.45356592 2.85076068 1.39618155
 3.01782608 0.89542733 1.6075915  0.91666458 1.55237354 0.55854698
 2.63514901 2.61595085 2.70931807 0.22871402 0.09773906 2.23192348
 0.73489032 2.9680953 ], Cost: 0.4682
Current parameters: [3.93359938 3.13838935 3.03490868 0.44498742 2.84694326 1.40205226
 3.09820893 0.80734472 1.60505797 0.9159766  1.63343863 0.47115545
 2.62276291 2.59212544 2.73245875 0.44814228 0.09666676 2.23639037
 0.73552286 2.97130101], Cost: 0.1276
Current parameters: [3.93914253 3.09696502 3.12703248 0.49373433 2.88411144 1.37607862
 3.12616901 0.79451877 1.62672858 0.9215324  1.6628462  0.45992653
```



```
 2.55815602 2.72868279 2.58282414 0.38669677 0.17033925 2.22165604
 0.81498622 2.95344796], Cost: 0.1280
Current parameters: [3.93315721 3.13961075 3.03149736 0.44284391 2.8457636  1.40329313
 3.12232807 0.78248279 1.60389724 0.91564277 1.53565603 0.57430279
 2.62467455 2.6631867  2.66349903 0.45016733 0.09402517 2.23638779
 0.73288778 2.97175857], Cost: 0.1133
Current parameters: [3.9397528  3.15661341 2.99853551 0.40253486 2.78703293 1.38226847
 3.068771   0.82390745 1.56868426 0.91270023 1.55659694 0.4370526
 2.54601489 2.56169407 2.51702855 0.46151096 0.04538885 2.17098363
 0.70217128 2.94017332], Cost: 1.8861
Current parameters: [3.92791821 3.14208663 3.03459936 0.44587481 2.84309669 1.40006974
 3.05937906 0.84461886 1.63014064 0.79374317 1.596025   0.50982114
 2.62357608 2.66241562 2.66284835 0.45021485 0.09279536 2.23593341
 0.73153917 2.97116286], Cost: 0.0763
Current parameters: [3.90345559 3.11980138 3.04130691 0.45402263 2.87628944 1.3911285
 3.07509538 0.85181421 1.64504917 0.79021611 1.68701565 0.58448346
 2.61681591 2.81243024 2.79931105 0.48267572 0.04483045 2.20926642
 0.72169205 2.99091224], Cost: 2.4830
Current parameters: [3.98257774 3.1512564  3.05275615 0.46415479 2.8373428  1.37747987
 3.0622407  0.84763135 1.52763001 0.77076288 1.59860554 0.51257151
 2.63108741 2.67017426 2.67120199 0.45250375 0.09428405 2.24133137
 0.73535048 2.97752559], Cost: 0.1038
Current parameters: [3.93403205 3.14599386 3.04590303 0.45597145 2.84716041 1.39162806
 3.01876225 0.89322246 1.63490621 0.79435927 1.5530684  0.55537422
 2.62880966 2.61445185 2.7020678  0.22616476 0.09569474 2.22779925
 0.73419045 2.96584649], Cost: 0.5256
Current parameters: [3.91467947 3.13000943 3.02221773 0.4596455  2.88852866 1.40353096
 3.04320719 0.86298227 1.61550028 0.79572298 1.58122543 0.51800481
 2.62932719 2.64965516 2.66928389 0.46869309 0.15758513 2.1554238
 0.71165594 2.98248864], Cost: 0.1542
Current parameters: [3.98712004 3.17536008 3.07591334 0.52381907 2.89497933 1.48213559
 3.11122682 0.73489148 1.67858942 0.71836633 1.58313878 0.49471369
 2.70065087 2.5959289  2.64313781 0.45116231 0.13623051 2.27177719
 0.70509591 2.91724924], Cost: 0.2889
Current parameters: [3.92985077 3.15026114 3.01784759 0.42404757 2.81184126 1.38937272
 3.0622405  0.83580707 1.6144153  0.79285249 1.57422286 0.47120728
 2.58598694 2.61050217 2.58741305 0.45865942 0.0708793  2.20934794
 0.71940919 2.95695715], Cost: 0.5483
Current parameters: [3.93493132 3.17569454 2.9906714  0.45602869 2.84416014 1.39114991
 3.06536513 0.84055236 1.63375328 0.79422856 1.60487829 0.50786469
 2.61854392 2.67181419 2.66150798 0.44345719 0.10599047 2.23821201
```



```
 0.74275513 2.97133324], Cost: 0.1387
Current parameters: [3.91098603 3.12464522 3.0329384  0.42210099 2.84237629 1.41187815
 3.04452619 0.8566419  1.62037223 0.79264887 1.57787593 0.51106222
 2.62668325 2.63938941 2.65449433 0.46590121 0.15659738 2.3091079
 0.76448066 3.01771841], Cost: 0.2121
Current parameters: [3.89199356 3.14490716 3.03800261 0.46847792 2.83199706 1.38066142
 3.03529538 0.81536161 1.6200199  0.79139613 1.59601355 0.51107449
 2.6272427  2.66094063 2.66481932 0.44889838 0.09374954 2.23867273
 0.73055988 2.96812686], Cost: 0.0763
Current parameters: [3.89643145 3.14586891 3.04550917 0.4718019  2.83311144 1.37881907
 3.01591437 0.83839714 1.62238324 0.79009488 1.57187653 0.53163303
 2.62956579 2.63130494 2.67961994 0.3371198  0.09626943 2.23752207
 0.73179464 2.96544717], Cost: 0.1841
Current parameters: [3.89159699 3.13242302 3.05949367 0.47815317 2.83895656 1.37279827
 3.04835851 0.81064009 1.62317866 0.79677073 1.61114969 0.49967679
 2.60906831 2.67818217 2.64564706 0.43565988 0.11234609 2.2322883
 0.75954376 2.95829752], Cost: 0.0431
Current parameters: [3.8862572  3.13045164 3.04831457 0.47996836 2.82497149 1.39538873
 3.02709633 0.83704474 1.61349015 0.79898438 1.59236215 0.51816359
 2.62592478 2.66072672 2.66436675 0.46156054 0.10639694 2.22524198
 0.82901786 2.87896382], Cost: 0.0657
Current parameters: [3.90902718 3.085425   3.03186476 0.49439808 2.8308435  1.36010115
 3.04828156 0.80465561 1.63020595 0.79317089 1.6099898  0.4997791
 2.61167374 2.67586752 2.64708188 0.43476156 0.11145641 2.2359862
 0.75698703 2.95634792], Cost: 0.0708
Current parameters: [3.87220824 3.11439223 3.09028991 0.47441703 2.85651208 1.38423109
 3.06044035 0.80631043 1.63441414 0.78415955 1.65649046 0.54049191
 2.60090591 2.74438015 2.7103685  0.44554094 0.08840615 2.23349321
 0.76401661 2.96289814], Cost: 0.6732
Current parameters: [3.90962133 3.13255019 3.07886622 0.48853267 2.85185767 1.39769643
 3.06507796 0.79337092 1.64438948 0.77592106 1.6084424  0.49677857
 2.62739316 2.66539875 2.64333968 0.43563998 0.11295341 2.23970107
 0.75112272 2.94404631], Cost: 0.0274
Current parameters: [3.89731731 3.17377791 3.04866956 0.54298822 2.86131109 1.38593879
 3.05155125 0.73255058 1.60428101 0.77966907 1.60708243 0.49336702
 2.64298981 2.64549859 2.63394441 0.43810681 0.16969798 2.25127008
 0.75894795 2.94260721], Cost: 0.1989
Current parameters: [3.91241574 3.15351    3.10317464 0.4977344  2.84247965 1.36404368
 3.06262373 0.81278711 1.65582023 0.79570381 1.61212302 0.49626901
 2.6303309  2.66787833 2.62976145 0.43948014 0.12206803 2.23335038
 0.76074466 2.95868805], Cost: 0.0477
```

```
Current parameters: [3.955485   3.1118338  3.074174   0.52683992 2.83625432 1.3893492
 3.06188245 0.79927893 1.69185958 0.75997814 1.59180983 0.52343368
 2.68955009 2.63320499 2.67069805 0.46891156 0.09502711 2.25339554
 0.73502815 2.92899356], Cost: 0.1384
Current parameters: [3.91143681 3.13492719 3.08182899 0.49231288 2.85058288 1.39465066
 3.06592659 0.79179124 1.64851137 0.77625811 1.56912391 0.45884769
 2.62763224 2.68612505 2.663886   0.43554161 0.11433099 2.23977464
 0.75327152 2.94408696], Cost: 0.0271
Current parameters: [3.91230562 3.13463769 3.08244617 0.48067784 2.82998231 1.39637236
 3.06940877 0.78604206 1.64886007 0.77382122 1.57312357 0.45858852
 2.6375715  2.69059997 2.66621718 0.43206697 0.1263306  2.23213419
 0.75129827 2.94243304], Cost: 0.0168
Current parameters: [3.90035534 3.12788703 3.07478779 0.47353599 2.84706429 1.39596575
 3.07323033 0.79144288 1.64015141 0.7819297  1.57934648 0.44415629
 2.68338802 2.7012024  2.64759139 0.42352913 0.11227553 2.23171048
 0.75802517 2.94551882], Cost: 0.0327
Current parameters: [3.91354022 3.1349533  3.08273035 0.46532255 2.84103946 1.37793094
 3.06963762 0.78174283 1.65008177 0.77080513 1.57232769 0.4599241
 2.63790586 2.68890759 2.66910816 0.43279307 0.13122782 2.23726988
 0.74644    2.92980916], Cost: 0.0164
Current parameters: [3.908004   3.13330938 3.08428667 0.47855185 2.83437961 1.38754528
 3.06931012 0.80500925 1.64516046 0.78623695 1.57764183 0.45537367
 2.63669535 2.70292904 2.66092139 0.43153878 0.14482799 2.25679954
 0.71269454 2.90271414], Cost: 0.0282
Current parameters: [3.91569595 3.13338403 3.084661   0.46410009 2.8418868  1.379425
 3.07034369 0.78117219 1.64934675 0.78346388 1.58649909 0.44537335
 2.63774103 2.67525878 2.68323883 0.43228308 0.13043106 2.23734247
 0.74669123 2.92940536], Cost: 0.0229
Current parameters: [3.94100531 3.1355789  3.08672562 0.45491875 2.84701512 1.39019063
 3.02513003 0.76666715 1.65721367 0.78665852 1.57594786 0.4592729
 2.63790614 2.70399364 2.65973824 0.43246215 0.13063654 2.23651514
 0.74587219 2.92899824], Cost: 0.0310
Current parameters: [3.91119596 3.13739357 3.08075791 0.46815619 2.8406635  1.37547023
 3.057166   0.79090594 1.65222859 0.74928011 1.58291819 0.44905004
 2.63799815 2.68901027 2.66937151 0.43282979 0.13404252 2.23750892
 0.74715397 2.93056628], Cost: 0.0134
Current parameters: [3.91415241 3.13463952 3.0832751  0.45456744 2.82458396 1.37677264
 3.06140231 0.78768557 1.65084643 0.74339678 1.57103801 0.42682054
 2.62422984 2.66485543 2.63371159 0.43886383 0.11720815 2.22484838
 0.73877128 2.91858552], Cost: 0.1904
Current parameters: [3.91177596 3.14064034 3.08120626 0.4647617  2.82980309 1.3739997
```



```
 3.05845305 0.78623834 1.65280765 0.7483148  1.58500305 0.44732574
 2.63701485 2.69018323 2.66724348 0.42979821 0.12666354 2.26314414
 0.75553011 2.93256255], Cost: 0.0226
Current parameters: [3.90585707 3.11775103 3.08777628 0.45075687 2.85061652 1.3906357
 3.05322748 0.80675505 1.65429305 0.75018516 1.56950319 0.45016985
 2.6410836  2.67455919 2.66591322 0.44511752 0.1686098  2.25387485
 0.75505298 2.95181312], Cost: 0.0227
Current parameters: [3.92752098 3.13527607 3.08985917 0.46862804 2.84050116 1.37347268
 3.05982475 0.79572684 1.62886679 0.74407596 1.58229619 0.44994209
 2.63994522 2.68967336 2.67138113 0.43343888 0.13274866 2.23818037
 0.74685801 2.93238225], Cost: 0.0146
Current parameters: [3.9121428  3.13737457 3.08454146 0.46981549 2.84143886 1.37567613
 3.04748912 0.80174    1.65520299 0.74802257 1.56960284 0.45867607
 2.64013953 2.67365409 2.67645416 0.37721298 0.13684479 2.23674576
 0.74781391 2.92867604], Cost: 0.0615
Current parameters: [3.915218   3.13500508 3.08125139 0.46908913 2.84389559 1.37163958
 3.0625758  0.78303879 1.65657113 0.74634552 1.58607395 0.44540661
 2.63701255 2.6908528  2.6665062  0.42760377 0.13390899 2.24058726
 0.72732862 2.94911783], Cost: 0.0134
Current parameters: [3.91119596 3.13739357 3.08075791 0.46815619 2.8406635  1.37547023
 3.057166   0.79090594 1.65222859 0.74928011 1.58291819 0.44905004
 2.63799815 2.68901027 2.66937151 0.43282979 0.13404252 2.23750892
 0.74715397 2.93056628], Cost: 0.0134

   Return from subroutine COBYLA because the MAXFUN limit has been reached.

   NFVALS =  100   F = 1.342496E-02    MAXCV = 0.000000E+00
   X = 3.911196E+00   3.137394E+00   3.080758E+00   4.681562E-01   2.840663E+00
       1.375470E+00   3.057166E+00   7.909059E-01   1.652229E+00   7.492801E-01
       1.582918E+00   4.490500E-01   2.637998E+00   2.689010E+00   2.669372E+00
       4.328298E-01   1.340425E-01   2.237509E+00   7.471540E-01   2.930566E+00
Simulating for QAOA depth p = 11
Current parameters: [2.40238256 0.49605756 1.99017856 1.73122928 1.00443071 0.59063359
 0.1158631  2.7394948  1.25173905 2.23549529 0.99232023 1.58224049
 0.189892   2.84602747 0.34540945 0.76914586 2.91324813 2.06802414
 2.59391702 1.74476959 0.11879669 2.55085284], Cost: 1.8313
Current parameters: [2.40238256 1.49605756 1.99017856 1.73122928 1.00443071 0.59063359
 0.1158631  2.7394948  1.25173905 2.23549529 0.99232023 1.58224049
 0.189892   2.84602747 0.34540945 0.76914586 2.91324813 2.06802414
 2.59391702 1.74476959 0.11879669 2.55085284], Cost: 2.2048
Current parameters: [2.40238256 0.49605756 2.99017856 1.73122928 1.00443071 0.59063359
```



```
 0.1158631  2.7394948  1.25173905 2.23549529 0.99232023 1.58224049
 0.189892   2.84602747 0.34540945 0.76914586 2.91324813 2.06802414
 2.59391702 1.74476959 0.11879669 2.55085284], Cost: 2.5157
Current parameters: [2.40238256 0.49605756 1.99017856 2.73122928 1.00443071 0.59063359
 0.1158631  2.7394948  1.25173905 2.23549529 0.99232023 1.58224049
 0.189892   2.84602747 0.34540945 0.76914586 2.91324813 2.06802414
 2.59391702 1.74476959 0.11879669 2.55085284], Cost: 1.6757
Current parameters: [2.40238256 0.49605756 1.99017856 2.73122928 2.00443071 0.59063359
 0.1158631  2.7394948  1.25173905 2.23549529 0.99232023 1.58224049
 0.189892   2.84602747 0.34540945 0.76914586 2.91324813 2.06802414
 2.59391702 1.74476959 0.11879669 2.55085284], Cost: 1.8493
Current parameters: [2.40238256 0.49605756 1.99017856 2.73122928 1.00443071 1.59063359
 0.1158631  2.7394948  1.25173905 2.23549529 0.99232023 1.58224049
 0.189892   2.84602747 0.34540945 0.76914586 2.91324813 2.06802414
 2.59391702 1.74476959 0.11879669 2.55085284], Cost: 1.4615
Current parameters: [2.40238256 0.49605756 1.99017856 2.73122928 1.00443071 1.59063359
 1.1158631  2.7394948  1.25173905 2.23549529 0.99232023 1.58224049
 0.189892   2.84602747 0.34540945 0.76914586 2.91324813 2.06802414
 2.59391702 1.74476959 0.11879669 2.55085284], Cost: 2.7574
Current parameters: [2.40238256 0.49605756 1.99017856 2.73122928 1.00443071 1.59063359
 0.1158631  3.7394948  1.25173905 2.23549529 0.99232023 1.58224049
 0.189892   2.84602747 0.34540945 0.76914586 2.91324813 2.06802414
 2.59391702 1.74476959 0.11879669 2.55085284], Cost: 2.7148
Current parameters: [2.40238256 0.49605756 1.99017856 2.73122928 1.00443071 1.59063359
 0.1158631  2.7394948  2.25173905 2.23549529 0.99232023 1.58224049
 0.189892   2.84602747 0.34540945 0.76914586 2.91324813 2.06802414
 2.59391702 1.74476959 0.11879669 2.55085284], Cost: 1.4571
Current parameters: [2.40238256 0.49605756 1.99017856 2.73122928 1.00443071 1.59063359
 0.1158631  2.7394948  2.25173905 3.23549529 0.99232023 1.58224049
 0.189892   2.84602747 0.34540945 0.76914586 2.91324813 2.06802414
 2.59391702 1.74476959 0.11879669 2.55085284], Cost: 2.2455
Current parameters: [2.40238256 0.49605756 1.99017856 2.73122928 1.00443071 1.59063359
 0.1158631  2.7394948  2.25173905 2.23549529 1.99232023 1.58224049
 0.189892   2.84602747 0.34540945 0.76914586 2.91324813 2.06802414
 2.59391702 1.74476959 0.11879669 2.55085284], Cost: 2.4373
Current parameters: [2.40238256 0.49605756 1.99017856 2.73122928 1.00443071 1.59063359
 0.1158631  2.7394948  2.25173905 2.23549529 0.99232023 2.58224049
 0.189892   2.84602747 0.34540945 0.76914586 2.91324813 2.06802414
 2.59391702 1.74476959 0.11879669 2.55085284], Cost: 2.4565
Current parameters: [2.40238256 0.49605756 1.99017856 2.73122928 1.00443071 1.59063359
 0.1158631  2.7394948  2.25173905 2.23549529 0.99232023 1.58224049
```



```
 1.189892   2.84602747 0.34540945 0.76914586 2.91324813 2.06802414
 2.59391702 1.74476959 0.11879669 2.55085284], Cost: 1.9774
Current parameters: [2.40238256 0.49605756 1.99017856 2.73122928 1.00443071 1.59063359
 0.1158631  2.7394948  2.25173905 2.23549529 0.99232023 1.58224049
 0.189892   3.84602747 0.34540945 0.76914586 2.91324813 2.06802414
 2.59391702 1.74476959 0.11879669 2.55085284], Cost: 1.8956
Current parameters: [2.40238256 0.49605756 1.99017856 2.73122928 1.00443071 1.59063359
 0.1158631  2.7394948  2.25173905 2.23549529 0.99232023 1.58224049
 0.189892   2.84602747 1.34540945 0.76914586 2.91324813 2.06802414
 2.59391702 1.74476959 0.11879669 2.55085284], Cost: 2.1788
Current parameters: [2.40238256 0.49605756 1.99017856 2.73122928 1.00443071 1.59063359
 0.1158631  2.7394948  2.25173905 2.23549529 0.99232023 1.58224049
 0.189892   2.84602747 0.34540945 1.76914586 2.91324813 2.06802414
 2.59391702 1.74476959 0.11879669 2.55085284], Cost: 2.3888
Current parameters: [2.40238256 0.49605756 1.99017856 2.73122928 1.00443071 1.59063359
 0.1158631  2.7394948  2.25173905 2.23549529 0.99232023 1.58224049
 0.189892   2.84602747 0.34540945 0.76914586 3.91324813 2.06802414
 2.59391702 1.74476959 0.11879669 2.55085284], Cost: 1.3755
Current parameters: [2.40238256 0.49605756 1.99017856 2.73122928 1.00443071 1.59063359
 0.1158631  2.7394948  2.25173905 2.23549529 0.99232023 1.58224049
 0.189892   2.84602747 0.34540945 0.76914586 3.91324813 3.06802414
 2.59391702 1.74476959 0.11879669 2.55085284], Cost: 0.6769
Current parameters: [2.40238256 0.49605756 1.99017856 2.73122928 1.00443071 1.59063359
 0.1158631  2.7394948  2.25173905 2.23549529 0.99232023 1.58224049
 0.189892   2.84602747 0.34540945 0.76914586 3.91324813 3.06802414
 3.59391702 1.74476959 0.11879669 2.55085284], Cost: 2.4817
Current parameters: [2.40238256 0.49605756 1.99017856 2.73122928 1.00443071 1.59063359
 0.1158631  2.7394948  2.25173905 2.23549529 0.99232023 1.58224049
 0.189892   2.84602747 0.34540945 0.76914586 3.91324813 3.06802414
 2.59391702 2.74476959 0.11879669 2.55085284], Cost: 2.3460
Current parameters: [2.40238256 0.49605756 1.99017856 2.73122928 1.00443071 1.59063359
 0.1158631  2.7394948  2.25173905 2.23549529 0.99232023 1.58224049
 0.189892   2.84602747 0.34540945 0.76914586 3.91324813 3.06802414
 2.59391702 1.74476959 1.11879669 2.55085284], Cost: 1.7986
Current parameters: [2.40238256 0.49605756 1.99017856 2.73122928 1.00443071 1.59063359
 0.1158631  2.7394948  2.25173905 2.23549529 0.99232023 1.58224049
 0.189892   2.84602747 0.34540945 0.76914586 3.91324813 3.06802414
 2.59391702 1.74476959 0.11879669 3.55085284], Cost: 2.1829
Current parameters: [ 2.66203766  0.41208429  1.83633454  2.76620345  0.96539797  1.63878884
 -0.17545555  2.45775723  2.25272077  2.05826782  0.77197581  1.35758759
  0.07294134  2.74745982  0.18318301  0.55971175  3.93158958  3.22506281
```

```
 2.18821325  1.36957034 -0.13335898  2.21233083], Cost: 1.7484
Current parameters: [2.2485268  0.02031771 1.99017856 2.73122928 1.00443071 1.59063359
 0.1158631  2.7394948  2.25173905 2.23549529 0.99232023 1.58224049
 0.189892   2.84602747 0.34540945 0.76914586 3.91324813 3.06802414
 2.59391702 1.74476959 0.11879669 2.55085284], Cost: 0.5120
Current parameters: [1.31519909 0.30438103 1.95506759 2.73921124 0.99552248 1.6016238
 0.04937705 2.67519539 2.2519631  2.19504764 0.94203223 1.53096919
 0.163201   2.82353192 0.30838541 0.72134787 3.9174341  3.10386421
 2.50132548 1.65913993 0.06124859 2.47359383], Cost: 0.8606
Current parameters: [2.2485268  0.02031771 2.1010181  3.21878912 1.00443071 1.59063359
 0.1158631  2.7394948  2.25173905 2.23549529 0.99232023 1.58224049
 0.189892   2.84602747 0.34540945 0.76914586 3.91324813 3.06802414
 2.59391702 1.74476959 0.11879669 2.55085284], Cost: 1.3462
Current parameters: [2.24849658 0.00972629 2.94448074 2.46194511 0.99911859 1.59718723
 0.07621638 2.701152   2.25187266 2.21137569 0.96233267 1.55166657
 0.17397572 2.83261301 0.3233314  0.74064313 3.91574429 3.08939616
 2.53870317 1.69370721 0.08447981 2.50478202], Cost: 1.4286
Current parameters: [2.2485268  0.02031771 1.99017856 2.73122928 0.50885926 1.59063359
 0.18226294 2.7394948  2.25173905 2.23549529 0.99232023 1.58224049
 0.189892   2.84602747 0.34540945 0.76914586 3.91324813 3.06802414
 2.59391702 1.74476959 0.11879669 2.55085284], Cost: 0.4196
Current parameters: [2.24846906e+00 8.22335332e-05 1.92036277e+00 2.64710704e+00
 6.26387379e-01 1.60315467e+00 1.14079178e+00 2.66623887e+00
 2.25199431e+00 2.18941352e+00 9.35027417e-01 1.52382741e+00
 1.59483100e-01 2.82039843e+00 3.03228179e-01 7.14689896e-01
 3.91801718e+00 3.10885651e+00 2.48842804e+00 1.64721223e+00
 5.32324858e-02 2.46283211e+00], Cost: 1.7611
Current parameters: [2.2485268  0.02031771 1.99017856 2.73122928 0.50885926 2.08348621
 0.18226294 2.82373433 2.25173905 2.23549529 0.99232023 1.58224049
 0.189892   2.84602747 0.34540945 0.76914586 3.91324813 3.06802414
 2.59391702 1.74476959 0.11879669 2.55085284], Cost: 0.5008
Current parameters: [2.24847037e+00 5.39201374e-04 1.92193939e+00 2.64900673e+00
 4.89406030e-01 1.41847931e+00 1.16601735e-01 3.69162412e+00
 2.25198855e+00 2.19045417e+00 9.36321233e-01 1.52514652e+00
 1.60169809e-01 2.82097719e+00 3.04180738e-01 7.15919648e-01
 3.91790948e+00 3.10793442e+00 2.49081025e+00 1.64941532e+00
 5.47130898e-02 2.46481984e+00], Cost: 1.8600
Current parameters: [2.26271027 0.09520176 1.99556087 2.73000569 0.51022484 1.58894886
 0.19245485 2.74935152 2.25173905 2.72212447 0.99232023 1.58224049
 0.189892   2.84602747 0.34540945 0.76914586 3.91324813 3.10583167
 2.55610949 1.70696205 0.08098916 2.51304531], Cost: 1.0515
```



```
Current parameters: [ 2.12038695 -0.70839385  1.75884173  2.52205032  0.44443411  1.6142675
 -0.08557013  2.45370368  2.25240729  2.21805051  0.8423356   1.42932316
  0.11028572  2.77893418  0.23498473  0.62658769  3.92573284  2.8337504
  2.65892874  1.83054521  0.28832593  2.66159402], Cost: 1.4689
Current parameters: [ 2.27975289 -0.05313955  1.90473723  2.62737018  0.62789403  1.59077679
  0.16543206  2.73865701  2.25173905  2.23496827  1.44685163  1.58224049
  0.189892    2.84602747  0.34540945  0.76914586  3.91324813  3.0979897
  2.56395146  1.71480402  0.08883112  2.52088727], Cost: 1.0725
Current parameters: [ 2.06280176  0.2905276   2.28942865  3.10530346 -0.18560786  1.59908356
  0.10900299  2.5613002   2.2523223   2.03970988  1.18491582  1.44877102
  0.12040996  2.78746704  0.24902843  0.64471809  3.92414505  2.95636549
  2.55784021  1.72681595  0.17394498  2.55468967], Cost: 0.9988
Current parameters: [2.23468509 0.01366581 1.97890277 2.71995    0.45927521 1.59275825
 0.14894094 2.69677537 2.25187625 2.18893484 0.94635387 2.06650966
 0.173547   2.83225168 0.32273672 0.7398754  3.91581153 3.07040094
 2.55678684 1.71190271 0.10312635 2.52311196], Cost: 1.1273
Current parameters: [2.2485268  0.02031771 1.99017856 2.73122928 0.50885926 1.59063359
 0.18226294 2.7394948  2.50173024 2.23549529 0.99232023 1.58224049
 0.19199051 2.84602747 0.34540945 0.76914586 3.91324813 3.06802414
 2.59391702 1.74476959 0.11879669 2.55085284], Cost: 0.5263
Current parameters: [2.24164966 0.01701277 1.98457628 2.72562526 0.48422383 1.59168921
 0.16570718 2.71827    2.24091798 2.21236211 0.96948221 1.56054979
 0.68552924 2.83918308 0.33414469 0.75460307 3.91452174 3.06920503
 2.57546919 1.72843995 0.11101101 2.53707001], Cost: 1.2585
Current parameters: [2.2485268  0.02031771 1.99017856 2.73122928 0.50885926 1.59063359
 0.18226294 2.7394948  2.25173905 2.23549529 0.99232023 1.58224049
 0.189892   3.05968168 0.21559465 0.76914586 3.91324813 3.06802414
 2.59391702 1.74476959 0.11879669 2.55085284], Cost: 0.4416
Current parameters: [2.23765328 0.01509223 1.98132073 2.72236871 0.46990794 1.59230264
 0.15608646 2.70593605 2.24156014 2.19891919 0.95621082 1.54794511
 0.14794291 3.09748855 0.76346703 0.74615211 3.91526184 3.06989126
 2.56474897 1.71895063 0.10648668 2.52906067], Cost: 2.2450
Current parameters: [2.2485268  0.02031771 1.99017856 2.73122928 0.50885926 1.59063359
 0.18226294 2.7394948  2.25173905 2.23549529 0.99232023 1.58224049
 0.189892   2.84602747 0.34540945 0.79095644 4.16229491 3.06802414
 2.59391702 1.74476959 0.11879669 2.55085284], Cost: 0.6543
Current parameters: [2.23808531 0.01529985 1.98167268 2.72272077 0.47145558 1.59223632
 0.15712652 2.70726943 2.24196458 2.20037246 0.95764554 1.54930776
 0.14960966 2.79828613 0.27086155 1.2460988  3.84914185 3.06981708
 2.5659079  1.71997649 0.10697579 2.52992654], Cost: 1.8777
Current parameters: [2.3051732  0.08595519 1.9561954  2.70593646 0.5379237  1.59414631
```



```
 0.15673674 2.71894324 2.25181066 2.2225673  0.97624704 1.56585301
 0.18136095 2.83883738 0.33357572 0.75386854 3.91458606 2.87437097
 2.53787685 1.68872941 0.06275651 2.49481266], Cost: 0.5629
Current parameters: [2.38657957 0.09683077 1.89777834 2.69526013 0.51844303 1.60807224
 0.05982899 2.62196443 2.24544    2.1514853  0.89397395 1.48361326
 0.12284807 2.78016425 0.23974146 0.639184   3.90947339 3.35653199
 2.49023711 1.64327188 0.02610118 2.45197892], Cost: 2.0016
Current parameters: [2.24545105 0.01421602 1.99203647 2.73359776 0.50614522 1.59065423
 0.18249948 2.73937405 2.25173947 2.23541933 0.99222579 1.5821442
 0.18984188 2.84598523 0.34533992 0.7690561  3.91325599 3.06809144
 2.75839842 1.55678619 0.12353344 2.55558959], Cost: 0.8165
Current parameters: [2.25673456 0.13184145 1.9708553  2.6777641  0.52553891 1.58532273
 0.19361967 2.74903022 2.24238681 2.22749499 0.99085019 1.5830227
 0.16848982 2.81499053 0.29812306 0.71706645 3.89675517 3.01525266
 2.91571239 2.06379514 0.02616074 2.44963437], Cost: 1.2247
Current parameters: [2.26291562 0.04886237 1.98148699 2.72014915 0.52155595 1.59053703
 0.18115636 2.74005971 2.25173708 2.23585064 0.99276203 1.58269093
 0.1901265  2.84622511 0.34573473 0.7695658  3.91321136 3.06770926
 2.59473049 1.74552189 0.32267472 2.41103648], Cost: 0.2381
Current parameters: [2.31699951 0.24182161 1.93589629 2.63609615 0.57776802 1.58544665
 0.18777989 2.75047009 2.24331856 2.22980791 0.99287372 1.5848565
 0.17163681 2.81894953 0.30425742 0.72408449 3.89825694 3.01922054
 2.57820019 1.779108   0.5523684  2.7793274 ], Cost: 2.1235
Current parameters: [2.04401369 0.11539283 1.96823435 2.66053835 0.57994439 1.59317249
 0.15710353 2.72464071 2.25179081 2.2261513  0.98070297 1.57039608
 0.18372599 2.84083068 0.33685636 0.75810384 3.91421515 3.07630371
 2.57252704 1.7249879  0.30887469 2.39250977], Cost: 0.1466
Current parameters: [2.08397668 0.05517861 2.40941304 2.46232211 0.65796939 1.59359611
 0.13689861 2.71411512 2.24834539 2.21429953 0.96913516 1.55945438
 0.16988074 2.82430461 0.3110753  0.72817118 3.90896966 3.06443826
 2.54427577 1.71916351 0.30591807 2.36420118], Cost: 0.1206
Current parameters: [ 2.08397754  0.05548062  2.41027584  2.46278946  0.63329163  1.56454768
 -0.04241056  2.88406609  2.24834158  2.21498728  0.96999023  1.56032617
  0.17033458  2.82468711  0.31170484  0.72898391  3.90889848  3.06382886
  2.54585014  1.72061952  0.30689659  2.36551485], Cost: 0.1363
Current parameters: [ 1.99720453 -0.07455793  2.40891221  2.48987626  0.61818642  1.53863263
  0.42981332  3.00885374  2.23685905  2.1745051   0.93021996  1.52262005
  0.12353398  2.76904814  0.22486061  0.62804006  3.8915086   3.02512193
  2.44944331  1.69915571  0.29566492  2.26928286], Cost: 2.0693
Current parameters: [ 2.09716691 -0.09815819  2.32506387  2.36730222  0.76698648  1.59522099
  0.11202889  2.70460856  2.24833679  2.24542173  0.88220106  1.56142194
```



```
  0.17090502  2.82516789  0.31249611  0.73000545  3.90880902  3.04822648
  2.56266541  1.73728599  0.3229629   2.38200243], Cost: 0.0844
Current parameters: [ 1.96317272 -0.31040408  2.37233052  2.49755871  0.57500066  1.62629093
 -0.05202624  2.50669949  2.24165194  2.29033648  0.93442037  1.48875968
  0.11674271  2.7697828   0.22418401  0.62271123  3.90243581  2.91233027
  2.56199408  1.78845893  0.40766899  2.39638654], Cost: 0.9280
Current parameters: [ 2.08367159 -0.11416912  2.37334627  2.53920553  0.92269825  1.57907511
  0.0984635   2.70933032  2.21814757  2.23391984  0.87232612  1.56552079
  0.16483586  2.79884089  0.32740122  0.72441072  3.87894413  3.0629685
  2.54655292  1.76512198  0.31334719  2.4150004 ], Cost: 0.1026
Current parameters: [ 2.155158   -0.01449457  2.32038371  2.41682679  0.92857171  1.6929291
  0.03360561  2.65913879  2.4220513   2.28317131  0.91005988  1.6223473
  0.2232083   2.98265609  0.21613073  0.771924    4.0640264   2.94826949
  2.66274146  1.63104067  0.3898952   2.13961837], Cost: 0.9058
Current parameters: [ 2.0819463  -0.1086834   2.33790241  2.36471407  0.75298708  1.59418041
  0.09818894  2.68608182  2.23897844  2.22804478  0.86490993  1.54448583
  0.14895152  2.9461093   0.52193331  0.72298391  3.8935427   3.04935479
  2.54723066  1.75555653  0.31793605  2.36138317], Cost: 0.6826
Current parameters: [ 2.12226027 -0.08378423  2.31127182  2.36292315  0.77530933  1.59807075
  0.09621428  2.69093016  2.24939779  2.23631825  0.87066814  1.54900751
  0.16471599  2.82334884  0.30755908  0.62576131  3.9189512   3.09313853
  2.5513452   1.72426576  0.31025026  2.36852913], Cost: 0.4807
Current parameters: [ 2.12418886 -0.08747564  2.31409258  2.3560358   0.75943321  1.5958416
  0.06094638  2.65011592  2.23002504  2.20101787  0.83334892  1.51166083
  0.12689916  2.78030025  0.26475448  0.88727282  3.86849682  3.14640383
  2.51045123  1.73678985  0.28538036  2.33469942], Cost: 0.2747
Current parameters: [ 2.0946688  -0.1171804   2.32736132  2.3753125   0.75996728  1.59704238
  0.10731181  2.69952469  2.24946169  2.24241371  0.87813807  1.5569745
  0.26058767  2.83053347  0.31771098  0.73612792  3.91015767  3.05742602
  2.50298452  1.68416961  0.33874052  2.39561122], Cost: 0.1117
Current parameters: [ 2.085663   -0.07315403  2.33099153  2.35046902  0.76191008  1.5832459
  0.10862724  2.69563728  2.2194342   2.23025547  0.8697992   1.54994113
  0.33349144  2.79250071  0.30596239  0.73854744  3.87584368  3.04501163
  2.6613219   1.8711839   0.27482306  2.34856065], Cost: 0.3436
Current parameters: [ 2.08960843 -0.08453445  2.33899058  2.38454261  0.73750433  1.59656421
  0.11001266  2.69838139  2.24890801  2.24452721  0.79149247  1.63670444
  0.16834917  2.82347971  0.30865893  0.72546814  3.90975823  3.04315102
  2.56214959  1.73665804  0.32587996  2.38228639], Cost: 0.0810
Current parameters: [ 2.08339396 -0.12344425  2.31867512  2.34870681  0.75538088  1.58763142
  0.09059009  2.67758349  2.22357948  2.22335004  0.92325792  1.82269597
  0.16372791  2.7993054   0.31293376  0.73957556  3.87928125  3.07104851
```

```
  2.52583848  1.74920584  0.29738343  2.36893785], Cost: 0.5032
Current parameters: [ 2.08690368 -0.08698736  2.34087404  2.38600539  0.7358239   1.71639001
  0.11135572  2.7199554   2.23798343  2.25414584  0.7921795   1.6374049
  0.16862214  2.814453    0.31483605  0.7251683   3.89882078  3.05232645
  2.55667214  1.74658161  0.31850998  2.3899316 ], Cost: 0.0342
Current parameters: [ 2.08678347 -0.05766086  2.35129768  2.38714708  0.73501976  1.69037926
  0.11573714  2.71900004  2.22171313  2.49324543  0.79196894  1.63736766
  0.16967979  2.80233775  0.32316815  0.74127669  3.87868606  3.08203856
  2.53014646  1.75026291  0.29168627  2.37587482], Cost: 0.1194
Current parameters: [ 2.09836284 -0.06199029  2.33301267  2.37563861  0.74473072  1.71630341
  0.1094183   2.71902284  2.25907732  2.25262371  0.79085353  1.63620544
  0.16672251  2.82995514  0.29975917  0.7224821   3.9202139   3.03168016
  2.47997844  1.82492437  0.34493011  2.39916262], Cost: 0.1772
Current parameters: [ 2.09654504 -0.00464679  2.34473843  2.34509288  0.76480383  1.72615205
  0.10605986  2.71584849  2.21884338  2.26257998  0.78744736  1.63441615
  0.16249016  2.79133509  0.30004134  0.776605    3.86424138  3.03272577
  2.62897086  1.69982839  0.42473957  2.5595641 ], Cost: 0.8647
Current parameters: [ 2.09095718 -0.0826657   2.33477774  2.38405089  0.73817341  1.71419158
  0.12210971  2.73159146  2.26513732  2.25094198  0.78867877  1.63396559
  0.1658223   2.83443859  0.29548268  0.70944792  3.78960334  3.02890118
  2.54622024  1.7395395   0.33319362  2.36472753], Cost: 0.0132
Current parameters: [ 2.03409437 -0.15664031  2.3450321   2.40466596  0.71709357  1.69843722
  0.26498061  2.87571486  2.24795567  2.24262497  0.76383203  1.61097166
  0.14518819  2.79853832  0.25539432  0.74176157  3.8214546   3.0086367
  2.52745243  1.7246142   0.32127226  2.30668044], Cost: 0.4894
Current parameters: [ 2.05968354 -0.12192011  2.35024351  2.40357527  0.71283954  1.71510917
  0.11987251  2.72500281  2.29936724  2.25975657  0.79944572  1.63520562
  0.16552399  2.86255044  0.27583672  0.70761191  3.78857126  3.09777603
  2.56932662  1.76656439  0.38983739  2.37024771], Cost: 0.1420
Current parameters: [ 2.03793416 -0.16958777  2.35198615  2.44187617  0.65485614  1.73425815
  0.04281738  2.63829076  2.2356615   2.2720106   0.80422858  1.59093416
  0.13728089  2.7824414   0.25997797  0.68993626  3.78401432  2.89690855
  2.53799502  1.74104605  0.33158141  2.3566742 ], Cost: 0.5279
Current parameters: [ 2.09263497 -0.07933728  2.33376427  2.38275891  0.7396539   1.71418032
  0.12198068  2.73165733  2.33526548  2.25098341  0.78873029  1.63401811
  0.16643832  2.7496137   0.34707362  0.70949688  3.78959906  3.02886447
  2.5463151   1.73962722  0.3569666   2.34842439], Cost: 0.0750
Current parameters: [ 2.07761983 -0.10520853  2.34569905  2.39110657  0.73079066  1.7160783
  0.12651656  2.73507237  2.41114782  2.25191746  0.78559771  1.63221304
  0.16701962  2.87524375  0.26875742  0.72587563  3.78829646  3.02461995
  2.54911513  1.73553359  0.16971488  2.46866178], Cost: 0.2582
```



```
Current parameters: [ 2.11627638 -0.1022685   2.44500088  2.33914738  0.75338902  1.71411088
  0.1187988   2.73007016  2.26428004  2.24868926  0.78666261  1.63212114
  0.16284338  2.8307247   0.28972154  0.70284032  3.78823321  3.02535646
  2.5407858   1.73954309  0.33343328  2.35901989], Cost: 0.0121
Current parameters: [ 2.32984391 -0.16407833  2.4287812   2.41158274  0.69107294  1.71226076
  0.14565334  2.74784291  2.26304853  2.2597052   0.79824807  1.64448316
  0.17023103  2.83736164  0.29958998  0.72369523  3.78702045  3.015674
  2.56637315  1.75883571  0.3460529   2.37669764], Cost: 0.1385
Current parameters: [ 2.12531726 -0.09715906  2.43563664  2.3301595   0.79252858  1.73490985
  0.11121494  2.73090851  2.30522086  2.26691492  0.81907737  1.63493008
  0.14788887  2.86459842  0.2638511   0.73233277  3.82423743  2.98322568
  2.57990409  1.72484414  0.3533235   2.31280676], Cost: 0.0340
Current parameters: [ 2.13277995 -0.03230798  2.48218566  2.42718214  0.77584341  1.74121251
  0.08125525  2.69787388  2.27192856  2.22206539  0.68147848  1.6786489
  0.27496778  2.86336134  0.28425329  0.70043363  3.80355571  3.06740669
  2.50763552  1.67355658  0.31363997  2.26415688], Cost: 0.8342
Current parameters: [ 2.12068048 -0.07582416  2.39935856  2.33357197  0.76205024  1.71212195
  0.12110868  2.73342602  2.22473316  2.24236349  0.78458865  1.62638721
  0.15080193  2.89823417  0.34762243  0.71194949  3.7432311   3.00052887
  2.53508224  1.73709345  0.32457861  2.3464213 ], Cost: 0.1306
Current parameters: [ 2.07416814 -0.18878913  2.55124683  2.32253252  0.71997459  1.71753719
  0.11897113  2.72335029  2.32164464  2.25170515  0.78983866  1.60251091
  0.12544466  2.85226213  0.45246685  0.74381436  3.85372764  3.04610448
  2.55820729  1.77435386  0.34044398  2.3568187 ], Cost: 0.6563
Current parameters: [ 2.10813502 -0.10227691  2.4395238   2.32354967  0.75218704  1.73004055
  0.11992243  2.73291194  2.23969253  2.23847324  0.79508438  1.62121967
  0.22844638  2.81463474  0.29434114  0.70998498  3.78513176  3.02208662
  2.60962036  1.797473    0.30043281  2.34217434], Cost: 0.0279
Current parameters: [ 2.12211208 -0.06296358  2.47163736  2.30292949  0.76530369  1.56864454
  0.13972017  2.72653446  2.3346962   2.30280119  0.77912672  1.6129503
  0.19921538  2.8318391   0.23838814  0.71122531  3.71166845  2.97048922
  2.50068936  1.8436802   0.39233114  2.36262168], Cost: 0.2929
Current parameters: [ 2.1307958  -0.10242601  2.4330173   2.31778017  0.75393231  1.71314953
  0.09750738  2.70813003  2.25632503  2.23159093  0.77666005  1.59554818
  0.12677596  2.80817576  0.26448156  0.78161653  3.7692514   3.06786631
  2.52853576  1.74818969  0.31344164  2.33370455], Cost: 0.0909
Current parameters: [ 2.11686134 -0.10129763  2.44338336  2.3336213   0.75358625  1.72191072
  0.16346405  2.68761338  2.26534267  2.24869372  0.78589869  1.63237489
  0.16378784  2.83154787  0.28901471  0.70174527  3.78940374  3.02556176
  2.54014624  1.73748024  0.33353815  2.35752365], Cost: 0.0102
Current parameters: [ 2.12417116 -0.08680904  2.42522805  2.25841038  0.66886333  1.72744907
```



```
  0.15648897  2.69555658  2.28002131  2.25214189  0.77718954  1.63945111
  0.17939011  2.84325013  0.28021995  0.69135942  3.80512327  3.02503489
  2.53646414  1.71308821  0.33861253  2.34157264], Cost: 0.0466
Current parameters: [ 2.1212603  -0.13461152  2.4249989   2.31495092  0.77583454  1.72321734
  0.15696068  2.6845626   2.26687006  2.25611791  0.75011531  1.62806972
  0.17086848  2.83317124  0.28887942  0.69407486  3.79186455  3.0238706
  2.54065959  1.73579294  0.33961407  2.36254505], Cost: 0.0089
Current parameters: [ 2.11831586 -0.17058249  2.40999064  2.28658457  0.79483195  1.71518121
  0.150266    2.67813492  2.25385752  2.25243243  0.79971055  1.72225879
  0.17284233  2.81615529  0.28557293  0.70008216  3.77616031  3.03492333
  2.52145315  1.73877475  0.32702694  2.35793308], Cost: 0.1273
Current parameters: [ 2.12186137 -0.14051838  2.42404754  2.31344963  0.77621166  1.71954002
  0.15644014  2.68363647  2.27763695  2.1980166   0.74928573  1.62821931
  0.17067099  2.8382325   0.28391143  0.69065665  3.79190995  3.01254798
  2.54046939  1.7405344   0.35142944  2.36542631], Cost: 0.0175
Current parameters: [ 2.12306554 -0.10026497  2.42714763  2.29273554  0.79129897  1.74181121
  0.16273984  2.6937562   2.24313411  2.25716124  0.74948712  1.63135025
  0.16901368  2.80944065  0.27726307  0.71913779  3.7867172   3.01764882
  2.58226357  1.70320592  0.37972819  2.44542235], Cost: 0.1457
Current parameters: [ 2.12032544 -0.12261527  2.42848254  2.31772662  0.76866153  1.72308445
  0.15756203  2.68393296  2.26668079  2.25666154  0.72056643  1.65484137
  0.13693132  2.83058011  0.28552794  0.68735114  3.79197744  3.02004484
  2.56205746  1.7546969   0.33445556  2.35726907], Cost: 0.0303
Current parameters: [ 2.1451892  -0.12252073  2.41023934  2.29339038  0.80275916  1.71930466
  0.15576616  2.68692193  2.26378063  2.25179469  0.78658744  1.58656847
  0.13602886  2.81942873  0.27457997  0.60956558  3.79665594  3.0573729
  2.55464976  1.73709295  0.32447789  2.35390517], Cost: 0.5451
Current parameters: [ 2.11906512 -0.13817535  2.42821637  2.31433426  0.77492509  1.72021194
  0.16719685  2.69468665  2.23488313  2.25548126  0.74909541  1.62611753
  0.1684824   2.81789243  0.2923182   0.69311043  3.79859623  3.02403464
  2.53778584  1.73769243  0.37840542  2.33393432], Cost: 0.0103
Current parameters: [ 2.09582087 -0.15791305  2.41325016  2.32421864  0.77073404  1.72048897
  0.22703241  2.75674244  2.26660354  2.25265225  0.73632119  1.62347109
  0.16696386  2.83120568  0.25941082  0.71760132  3.79644146  3.00466219
  2.52980143  1.72029594  0.29906893  2.36148203], Cost: 0.1608
Current parameters: [ 2.11656344 -0.13747042  2.40856838  2.33008528  0.76447966  1.72862562
  0.14230444  2.66859733  2.24871928  2.25947971  0.75180247  1.62390733
  0.17096509  2.82344521  0.25763487  0.68461944  3.77870178  2.99293872
  2.53653086  1.72873146  0.33415386  2.36144682], Cost: 0.0785
Current parameters: [ 2.08551368 -0.19183368  2.47181831  2.31778878  0.74065491  1.72929283
  0.14066515  2.66122717  2.27745874  2.26658035  0.75646056  1.61213057
```



```
  0.1525918   2.82950835  0.34693142  0.72094203  3.81046451  2.98964996
  2.54680081  1.74490571  0.34557486  2.37517662], Cost: 0.1204
Current parameters: [ 2.12162044 -0.13106147  2.42618016  2.30004233  0.77657787  1.68896004
  0.16477374  2.68653492  2.28118178  2.27032639  0.7571353   1.61990582
  0.16978404  2.83254485  0.27904433  0.69653169  3.77294224  3.00786557
  2.5343855   1.76511626  0.35446075  2.37091032], Cost: 0.0548
Current parameters: [ 2.12180208 -0.1022849   2.46584507  2.3454649   0.78548444  1.70977803
  0.14449902  2.66853215  2.29033144  2.25799722  0.69713044  1.65731535
  0.23949361  2.84352844  0.28896751  0.71760246  3.79123782  3.03195038
  2.5196342   1.71956009  0.35322515  2.33514501], Cost: 0.2365
Current parameters: [ 2.07546171 -0.1147431   2.41792381  2.29758066  0.7916613   1.72302176
  0.15161616  2.68145297  2.25767734  2.25215926  0.74705015  1.62397869
  0.16637097  2.84770551  0.30054681  0.6916403   3.78128274  3.01996671
  2.53376863  1.73075405  0.334611    2.35558184], Cost: 0.0122
Current parameters: [ 2.17460976 -0.12761253  2.38784071  2.32694373  0.76812266  1.7221478
  0.16522859  2.69129824  2.23202946  2.25324065  0.74842804  1.63031195
  0.16613493  2.89377579  0.3484035   0.71449194  3.75299568  2.99926983
  2.54254076  1.73325577  0.33682577  2.35909586], Cost: 0.1443
Current parameters: [ 2.12431163 -0.12309731  2.41828633  2.31280839  0.77903142  1.7514567
  0.15489193  2.68746718  2.26232509  2.25791068  0.74906094  1.62964227
  0.17201468  2.83711726  0.28778133  0.6982622   3.81735492  3.01781719
  2.50946908  1.76784748  0.34194139  2.37433805], Cost: 0.0292
Current parameters: [ 2.12090626 -0.13757045  2.43080823  2.31374157  0.77521617  1.74250756
  0.1586257   2.68951084  2.27516375  2.25760269  0.75022462  1.62760798
  0.17031687  2.83222135  0.28782729  0.69100813  3.7720795   3.02563858
  2.53921954  1.73794825  0.34554776  2.35890034], Cost: 0.0133
Current parameters: [ 2.1212603  -0.13461152  2.4249989   2.31495092  0.77583454  1.72321734
  0.15696068  2.6845626   2.26687006  2.25611791  0.75011531  1.62806972
  0.17086848  2.83317124  0.28887942  0.69407486  3.79186455  3.0238706
  2.54065959  1.73579294  0.33961407  2.36254505], Cost: 0.0089

   Return from subroutine COBYLA because the MAXFUN limit has been reached.

   NFVALS =  100   F = 8.895853E-03    MAXCV = 0.000000E+00
   X = 2.121260E+00  -1.346115E-01   2.424999E+00   2.314951E+00   7.758345E-01
       1.723217E+00   1.569607E-01   2.684563E+00   2.266870E+00   2.256118E+00
       7.501153E-01   1.628070E+00   1.708685E-01   2.833171E+00   2.888794E-01
       6.940749E-01   3.791865E+00   3.023871E+00   2.540660E+00   1.735793E+00
       3.396141E-01   2.362545E+00
Simulating for QAOA depth p = 12
Current parameters: [3.16472813 1.3739492  3.11351795 2.14862898 0.56178559 0.71257851
```

```
 0.3704145  2.04043024 0.83945203 0.58921618 3.05888431 1.7769265
 0.81358283 1.88227998 0.45720571 2.01231696 3.12758377 1.18082061
 2.88025404 1.46977744 2.30594583 0.33789464 2.95850829 0.58589306], Cost: 2.4813
Current parameters: [2.16472813 2.3739492  3.11351795 2.14862898 0.56178559 0.71257851
 0.3704145  2.04043024 0.83945203 0.58921618 3.05888431 1.7769265
 0.81358283 1.88227998 0.45720571 2.01231696 3.12758377 1.18082061
 2.88025404 1.46977744 2.30594583 0.33789464 2.95850829 0.58589306], Cost: 1.4428
Current parameters: [2.16472813 1.3739492  4.11351795 2.14862898 0.56178559 0.71257851
 0.3704145  2.04043024 0.83945203 0.58921618 3.05888431 1.7769265
 0.81358283 1.88227998 0.45720571 2.01231696 3.12758377 1.18082061
 2.88025404 1.46977744 2.30594583 0.33789464 2.95850829 0.58589306], Cost: 0.8308
Current parameters: [2.16472813 1.3739492  3.11351795 3.14862898 0.56178559 0.71257851
 0.3704145  2.04043024 0.83945203 0.58921618 3.05888431 1.7769265
 0.81358283 1.88227998 0.45720571 2.01231696 3.12758377 1.18082061
 2.88025404 1.46977744 2.30594583 0.33789464 2.95850829 0.58589306], Cost: 1.6381
Current parameters: [2.16472813 1.3739492  3.11351795 2.14862898 1.56178559 0.71257851
 0.3704145  2.04043024 0.83945203 0.58921618 3.05888431 1.7769265
 0.81358283 1.88227998 0.45720571 2.01231696 3.12758377 1.18082061
 2.88025404 1.46977744 2.30594583 0.33789464 2.95850829 0.58589306], Cost: 2.0500
Current parameters: [2.16472813 1.3739492  3.11351795 2.14862898 0.56178559 1.71257851
 0.3704145  2.04043024 0.83945203 0.58921618 3.05888431 1.7769265
 0.81358283 1.88227998 0.45720571 2.01231696 3.12758377 1.18082061
 2.88025404 1.46977744 2.30594583 0.33789464 2.95850829 0.58589306], Cost: 2.0759
Current parameters: [2.16472813 1.3739492  3.11351795 2.14862898 0.56178559 0.71257851
 1.3704145  2.04043024 0.83945203 0.58921618 3.05888431 1.7769265
 0.81358283 1.88227998 0.45720571 2.01231696 3.12758377 1.18082061
 2.88025404 1.46977744 2.30594583 0.33789464 2.95850829 0.58589306], Cost: 1.2262
Current parameters: [2.16472813 1.3739492  3.11351795 2.14862898 0.56178559 0.71257851
 0.3704145  3.04043024 0.83945203 0.58921618 3.05888431 1.7769265
 0.81358283 1.88227998 0.45720571 2.01231696 3.12758377 1.18082061
 2.88025404 1.46977744 2.30594583 0.33789464 2.95850829 0.58589306], Cost: 1.4295
Current parameters: [2.16472813 1.3739492  3.11351795 2.14862898 0.56178559 0.71257851
 0.3704145  2.04043024 1.83945203 0.58921618 3.05888431 1.7769265
 0.81358283 1.88227998 0.45720571 2.01231696 3.12758377 1.18082061
 2.88025404 1.46977744 2.30594583 0.33789464 2.95850829 0.58589306], Cost: 1.7386
Current parameters: [2.16472813 1.3739492  3.11351795 2.14862898 0.56178559 0.71257851
 0.3704145  2.04043024 0.83945203 1.58921618 3.05888431 1.7769265
 0.81358283 1.88227998 0.45720571 2.01231696 3.12758377 1.18082061
 2.88025404 1.46977744 2.30594583 0.33789464 2.95850829 0.58589306], Cost: 2.2729
Current parameters: [2.16472813 1.3739492  3.11351795 2.14862898 0.56178559 0.71257851
 0.3704145  2.04043024 0.83945203 0.58921618 4.05888431 1.7769265
```



```
 0.81358283 1.88227998 0.45720571 2.01231696 3.12758377 1.18082061
 2.88025404 1.46977744 2.30594583 0.33789464 2.95850829 0.58589306], Cost: 1.9290
Current parameters: [2.16472813 1.3739492  3.11351795 2.14862898 0.56178559 0.71257851
 0.3704145  2.04043024 0.83945203 0.58921618 3.05888431 2.7769265
 0.81358283 1.88227998 0.45720571 2.01231696 3.12758377 1.18082061
 2.88025404 1.46977744 2.30594583 0.33789464 2.95850829 0.58589306], Cost: 2.5061
Current parameters: [2.16472813 1.3739492  3.11351795 2.14862898 0.56178559 0.71257851
 0.3704145  2.04043024 0.83945203 0.58921618 3.05888431 1.7769265
 1.81358283 1.88227998 0.45720571 2.01231696 3.12758377 1.18082061
 2.88025404 1.46977744 2.30594583 0.33789464 2.95850829 0.58589306], Cost: 3.0423
Current parameters: [2.16472813 1.3739492  3.11351795 2.14862898 0.56178559 0.71257851
 0.3704145  2.04043024 0.83945203 0.58921618 3.05888431 1.7769265
 0.81358283 2.88227998 0.45720571 2.01231696 3.12758377 1.18082061
 2.88025404 1.46977744 2.30594583 0.33789464 2.95850829 0.58589306], Cost: 1.1397
Current parameters: [2.16472813 1.3739492  3.11351795 2.14862898 0.56178559 0.71257851
 0.3704145  2.04043024 0.83945203 0.58921618 3.05888431 1.7769265
 0.81358283 1.88227998 1.45720571 2.01231696 3.12758377 1.18082061
 2.88025404 1.46977744 2.30594583 0.33789464 2.95850829 0.58589306], Cost: 1.0376
Current parameters: [2.16472813 1.3739492  3.11351795 2.14862898 0.56178559 0.71257851
 0.3704145  2.04043024 0.83945203 0.58921618 3.05888431 1.7769265
 0.81358283 1.88227998 0.45720571 3.01231696 3.12758377 1.18082061
 2.88025404 1.46977744 2.30594583 0.33789464 2.95850829 0.58589306], Cost: 2.7427
Current parameters: [2.16472813 1.3739492  3.11351795 2.14862898 0.56178559 0.71257851
 0.3704145  2.04043024 0.83945203 0.58921618 3.05888431 1.7769265
 0.81358283 1.88227998 0.45720571 2.01231696 4.12758377 1.18082061
 2.88025404 1.46977744 2.30594583 0.33789464 2.95850829 0.58589306], Cost: 0.8964
Current parameters: [2.16472813 1.3739492  3.11351795 2.14862898 0.56178559 0.71257851
 0.3704145  2.04043024 0.83945203 0.58921618 3.05888431 1.7769265
 0.81358283 1.88227998 0.45720571 2.01231696 3.12758377 2.18082061
 2.88025404 1.46977744 2.30594583 0.33789464 2.95850829 0.58589306], Cost: 1.9242
Current parameters: [2.16472813 1.3739492  3.11351795 2.14862898 0.56178559 0.71257851
 0.3704145  2.04043024 0.83945203 0.58921618 3.05888431 1.7769265
 0.81358283 1.88227998 0.45720571 2.01231696 3.12758377 1.18082061
 3.88025404 1.46977744 2.30594583 0.33789464 2.95850829 0.58589306], Cost: 2.1338
Current parameters: [2.16472813 1.3739492  3.11351795 2.14862898 0.56178559 0.71257851
 0.3704145  2.04043024 0.83945203 0.58921618 3.05888431 1.7769265
 0.81358283 1.88227998 0.45720571 2.01231696 3.12758377 1.18082061
 2.88025404 2.46977744 2.30594583 0.33789464 2.95850829 0.58589306], Cost: 1.4755
Current parameters: [2.16472813 1.3739492  3.11351795 2.14862898 0.56178559 0.71257851
 0.3704145  2.04043024 0.83945203 0.58921618 3.05888431 1.7769265
 0.81358283 1.88227998 0.45720571 2.01231696 3.12758377 1.18082061
```



```
 2.88025404 1.46977744 3.30594583 0.33789464 2.95850829 0.58589306], Cost: 2.0044
Current parameters: [2.16472813 1.3739492  3.11351795 2.14862898 0.56178559 0.71257851
 0.3704145  2.04043024 0.83945203 0.58921618 3.05888431 1.7769265
 0.81358283 1.88227998 0.45720571 2.01231696 3.12758377 1.18082061
 2.88025404 1.46977744 2.30594583 1.33789464 2.95850829 0.58589306], Cost: 1.2343
Current parameters: [2.16472813 1.3739492  3.11351795 2.14862898 0.56178559 0.71257851
 0.3704145  2.04043024 0.83945203 0.58921618 3.05888431 1.7769265
 0.81358283 1.88227998 0.45720571 2.01231696 3.12758377 1.18082061
 2.88025404 1.46977744 2.30594583 0.33789464 3.95850829 0.58589306], Cost: 1.1581
Current parameters: [2.16472813 1.3739492  3.11351795 2.14862898 0.56178559 0.71257851
 0.3704145  2.04043024 0.83945203 0.58921618 3.05888431 1.7769265
 0.81358283 1.88227998 0.45720571 2.01231696 3.12758377 1.18082061
 2.88025404 1.46977744 2.30594583 0.33789464 2.95850829 1.58589306], Cost: 2.7792
Current parameters: [1.87485815 1.25688105 3.09826816 1.99906111 0.34369085 0.49016127
 0.28938809 1.92556919 0.67315621 0.33402    2.86091804 1.48293932
 0.43037784 1.81563428 0.40755383 1.67895798 3.10141712 0.98365116
 2.64820137 1.34727115 2.09543312 0.2555064  2.88881247 0.24645695], Cost: 0.2910
Current parameters: [1.80991947 1.23065463 3.09485179 1.96555388 0.29483175 0.44033381
 0.271236   1.89983722 0.63590146 0.27684918 2.81656827 1.41707828
 0.88555017 1.80070385 0.39643047 1.60427659 3.09555508 0.93947989
 2.59621531 1.31982643 2.0482726  0.23704922 2.87319873 0.17041411], Cost: 2.9141
Current parameters: [1.87485815 1.25688105 3.09826816 1.99906111 0.34369085 0.49016127
 0.28938809 1.92556919 0.67315621 0.33402    2.86091804 1.48293932
 0.43037784 1.81563428 0.40755383 1.5005918  3.10141712 0.98365116
 2.64820137 1.34727115 2.09543312 0.2555064  2.88881247 0.42162973], Cost: 1.5480
Current parameters: [1.81247028 1.23168481 3.09498599 1.96687006 0.29675096 0.44229106
 0.27194902 1.90084798 0.63736484 0.27909488 2.81831034 1.41966533
 0.25010734 1.80129032 0.3968674  2.08421319 3.09578535 0.94121496
 2.59825735 1.32090447 2.05012509 0.23777422 2.87381204 0.40213383], Cost: 3.5038
Current parameters: [1.6968393  1.25688105 3.09826816 1.99906111 0.34369085 0.49016127
 0.28938809 1.92556919 0.67315621 0.33402    2.86091804 1.65846507
 0.43037784 1.81563428 0.40755383 1.67895798 3.10141712 0.98365116
 2.64820137 1.34727115 2.09543312 0.2555064  2.88881247 0.24645695], Cost: 0.4485
Current parameters: [2.18594471 1.242563   3.09640303 1.98076818 0.31701673 0.46295848
 0.27947814 1.91152108 0.65281736 0.30280818 2.83670574 1.78018787
 0.32793709 1.80748316 0.40148114 1.61703354 3.0982168  0.95953631
 2.61982011 1.33228799 2.06968632 0.24542989 2.88028831 0.03737973], Cost: 0.2134
Current parameters: [2.18594471 1.242563   3.09640303 1.98076818 0.31701673 0.46295848
 0.27947814 1.91152108 0.65281736 0.47099866 2.83670574 1.78018787
 0.32793709 1.80748316 0.40148114 1.61703354 3.0982168  0.95953631
 2.43485536 1.33228799 2.06968632 0.24542989 2.88028831 0.03737973], Cost: 0.7389
```



```
Current parameters: [ 2.14521148  1.23131413  3.0949377   1.96639647  0.29606039  0.4415868
  0.27169246  1.90048429  0.63683829  0.61532182  2.81768351  1.72453295
  0.24745524  1.80107929  0.39671018  1.56838304  3.09570249  0.94059065
  2.94942286  1.32051657  2.04945852  0.23751335  2.87359136 -0.12688032], Cost: 1.3501
Current parameters: [2.18594471 1.242563   3.09640303 1.98076818 0.13851401 0.63799214
 0.27947814 1.91152108 0.65281736 0.30280818 2.83670574 1.78018787
 0.32793709 1.80748316 0.40148114 1.61703354 3.0982168  0.95953631
 2.61982011 1.33228799 2.06968632 0.24542989 2.88028831 0.03737973], Cost: 0.2238
Current parameters: [ 2.15338082  1.23357017  3.09523159  1.96927882  0.64256     0.79419257
  0.27325393  1.90269779  0.640043    0.22633871  2.82149855  1.73569494
  0.26359644  1.80236364  0.39766703  1.57814024  3.09620676  0.94439033
  2.58660481  1.32287741  2.05351535  0.23910107  2.87493448 -0.09393681], Cost: 0.5805
Current parameters: [2.18594471 1.242563   3.09640303 1.98076818 0.31701673 0.46295848
 0.27947814 1.91152108 0.65281736 0.30280818 2.65458523 1.78018787
 0.32793709 1.80748316 0.40148114 1.61703354 3.0982168  0.95953631
 2.61982011 1.33228799 2.24095254 0.24542989 2.88028831 0.03737973], Cost: 0.5894
Current parameters: [ 2.15755985  1.23472425  3.09538192  1.97075328  0.28250156  0.42709568
  0.2740527   1.90383011  0.64168238  0.23615228  3.17136703  1.74140486
  0.27185348  1.80302064  0.39815651  1.58313155  3.09646471  0.94633407
  2.59086744  1.3240851   2.40109741  0.23991327  2.87562155 -0.07708454], Cost: 1.1599
Current parameters: [2.18594471 1.242563   3.09640303 1.98076818 0.31701673 0.46295848
 0.27947814 1.91152108 0.46171301 0.30280818 2.83670574 1.78018787
 0.32793709 1.80748316 0.40148114 1.61703354 3.0982168  1.12071673
 2.61982011 1.33228799 2.06968632 0.24542989 2.88028831 0.03737973], Cost: 0.3420
Current parameters: [ 2.16249949  1.23608838  3.09555962  1.97249611  0.28850802  0.43333666
  0.27499686  1.90516853  0.96444489  0.247752    2.81227798  1.74815403
  0.28161337  1.80379723  0.39873507  1.5890313   3.09676962  1.32167573
  2.59590589  1.3255126   2.0235071   0.24087329  2.87643368 -0.05716503], Cost: 1.9796
Current parameters: [2.18594471 1.242563   3.09640303 2.1391803  0.31701673 0.46295848
 0.27947814 1.91152108 0.65281736 0.30280818 2.83670574 1.78018787
 0.32793709 1.80748316 0.40148114 1.61703354 3.0982168  0.95953631
 2.61982011 1.13888272 2.06968632 0.24542989 2.88028831 0.03737973], Cost: 0.6419
Current parameters: [ 2.16987054  1.23812397  3.09582478  2.3511819   0.297471    0.4426496
  0.27640575  1.90716573  0.62726441  0.26506136  2.81995794  1.7582253
  0.2961773   1.80495606  0.39959842  1.59783505  3.0972246   0.92420475
  2.6034244   1.64966095  2.03802559  0.24230586  2.87764555 -0.02744067], Cost: 0.2002
Current parameters: [ 2.16987054  1.41321048  3.09582478  2.3511819   0.297471    0.4426496
  0.27640575  1.72871487  0.62726441  0.26506136  2.81995794  1.7582253
  0.2961773   1.80495606  0.39959842  1.59783505  3.0972246   0.92420475
  2.6034244   1.64966095  2.03802559  0.24230586  2.87764555 -0.02744067], Cost: 0.5111
Current parameters: [ 2.15646378  1.58417797  3.09534249  2.33435121  0.28116877  0.42571085
```

```
  0.2738432   2.25586415  0.60595183  0.2335784   2.80598933  1.73990728
  0.26968784  1.80284832  0.39802813  1.58182244  3.09639706  0.89473625
  2.58974945  1.64753459  2.01161877  0.23970025  2.87544135 -0.08150452], Cost: 0.5300
Current parameters: [ 2.16987054  1.23812397  3.09582478  2.3511819   0.297471    0.4426496
  0.09816206  1.90716573  0.62726441  0.26506136  2.81995794  1.7582253
  0.2961773   1.80495606  0.39959842  1.59783505  3.0972246   0.92420475
  2.6034244   1.64966095  2.03802559  0.41760328  2.87764555 -0.02744067], Cost: 0.7222
Current parameters: [ 2.16059092  1.22710247  3.09549096  2.33953237  0.28618725  0.43092528
  0.62936644  1.90269817  0.6125127   0.24327012  2.81028943  1.7455463
  0.27784236  1.80349717  0.39851153  1.58675176  3.09665181  0.90380784
  2.59395915  1.64818917  2.01974786  0.59035289  2.87611989 -0.06486149], Cost: 1.8091
Current parameters: [ 2.16987054  1.23812397  3.09582478  2.3511819   0.297471    0.4426496
  0.27640575  1.90716573  0.62726441  0.26506136  2.81995794  1.7582253
  0.2961773   1.62426987  0.39959842  1.59783505  3.0972246   0.92420475
  2.6034244   1.64966095  2.03802559  0.24230586  3.05042431 -0.02744067], Cost: 0.6024
Current parameters: [ 2.16235245  1.22919467  3.09555433  2.34174378  0.28832923  0.43315089
  0.2705971   1.90354624  0.61531299  0.24740672  2.81212479  1.74795313
  0.28132286  2.15180984  0.39871785  1.58885569  3.09676054  0.90767975
  2.59575592  1.64846855  2.02321749  0.22761248  3.23350385 -0.05775795], Cost: 3.2567
Current parameters: [ 2.16987054  1.23812397  3.09582478  2.3511819   0.297471    0.4426496
  0.27640575  1.90716573  0.62726441  0.26506136  2.81995794  1.7582253
  0.2961773   1.80495606  0.51615388  1.59783505  2.87605764  0.92420475
  2.6034244   1.64966095  2.03802559  0.24230586  2.87764555 -0.02744067], Cost: 0.1041
Current parameters: [ 2.1656637   1.23312747  3.09567345  2.34590069  0.29235561  0.43733448
  0.27315545  1.9051404   0.62057685  0.25518249  2.8155748   1.75247738
  0.28786531  1.79815143  0.95778844  1.59281054  3.10808246  0.91495799
  2.59913341  1.64899373  2.02973954  0.234084    2.86668549 -0.04440508], Cost: 3.0661
Current parameters: [ 2.15530711  1.23224232  3.34072559  2.34366742  0.28651365  0.43147508
  0.27233488  1.90139497  0.61890951  0.25223999  2.81001187  1.74345502
  0.2769246   1.8016077   0.51365931  1.58108669  2.874743    0.91429871
  2.59176578  1.64350608  2.02744918  0.23816657  2.87414395 -0.04449436], Cost: 0.1281
Current parameters: [2.31782494 1.29315567 3.19591174 2.42306969 0.40599757 0.55318627
 0.31479259 1.96527954 0.70602426 0.38647515 2.91884084 1.90616003
 0.48783277 1.83061435 0.52840176 1.76771987 2.88148485 1.0157
 2.72054587 1.71378062 2.13798442 0.27429717 2.89897375 0.12857518], Cost: 2.1780
Current parameters: [2.21668653 1.2324056  3.09507988 2.2506543  0.33425152 0.34919954
 0.27244789 1.90155517 0.66442513 0.1776036  2.8544526  1.80570624
 0.29466906 1.80170065 0.51372856 1.64816315 2.87477949 0.82604919
 2.64782617 1.68584164 1.94086608 0.23828148 2.87424115 0.02380491], Cost: 0.4551
Current parameters: [ 2.12014762  1.18196652  3.06405217  2.39941159  0.22157615  0.50269473
  0.24146402  1.9063959   0.5070666   0.24154222  2.75480087  1.67982266
```



```
  0.08682822  1.69820202  0.34604864  1.83203567  2.77286206  0.90808364
  2.53881982  1.63218183  2.04264743  0.11392518  2.69341517 -0.01271463], Cost: 1.5335
Current parameters: [ 2.17000332  1.13518213  3.09460289  2.37647252  0.29771789  0.43053647
  0.30728816  1.93880931  0.62831336  0.25302917  2.82028672  1.75835997
  0.40975581  1.83641246  0.4680326   1.55791994  2.95772333  0.91202077
  2.60418441  1.68156606  2.0257383   0.13867653  2.77354239 -0.06808343], Cost: 0.5710
Current parameters: [ 2.12941613  1.3601218   3.07576235  2.28498936  0.24277048  0.44227753
  0.19334704  1.84085849  0.54790211  0.21036301  2.77297343  1.69927782
  0.59228332  1.67658736  0.48161749  1.56179014  2.67101834  0.87563726
  2.5568787   1.58847273  2.00206022  0.32315242  2.92495809 -0.20746242], Cost: 2.6412
Current parameters: [2.18676395 1.24898659 3.08858466 2.35413185 0.30012765 0.46204589
 0.26725808 1.9093445  0.61401778 0.2656299  2.82353777 1.76787728
 0.24273066 1.77551716 0.48241093 1.40749313 2.8360861  0.9205048
 2.61137696 1.65500265 2.04108539 0.23159467 2.85120974 0.10546554], Cost: 1.1972
Current parameters: [ 2.25420077  1.23785102  3.09580498  2.34854632  0.29597616  0.43761175
  0.27634886  1.90706941  0.64736597  0.2598113   2.8186308   1.67099105
  0.2950898   1.8049171   0.51603235  1.59717767  2.87613195  0.94422291
  2.60197519  1.65068187  2.03302368  0.24207346  2.8774277  -0.0296602 ], Cost: 0.1709
Current parameters: [ 2.11636034  1.24127281  3.0921066   2.33147386  0.28677648  0.41174128
  0.27145209  1.90746349  0.78212624  0.22319372  2.81106359  1.79112513
  0.26117272  1.79017116  0.49859027  1.59172225  2.85700425  1.08309023
  2.59569974  1.6604828   1.9993748   0.23517457  2.86290089 -0.07099844], Cost: 0.1067
Current parameters: [ 2.16829359  1.23801196  3.09579679  2.35298393  0.30217702  0.45043726
  0.27621593  1.90690999  0.6253133   0.32585631  2.75322019  1.75635196
  0.29463988  1.80479327  0.51611128  1.5969057   2.87591751  0.92513663
  2.65587798  1.64843242  1.97011235  0.24226132  2.87762595 -0.03057847], Cost: 0.0566
Current parameters: [ 2.1480862   1.23481201  3.09230925  2.31342795  0.29907809  0.40549818
  0.26948093  1.90289903  0.64097148  0.41343065  2.86307198  1.73088136
  0.233791    1.79103947  0.50011308  1.57174564  2.86095573  0.89601024
  2.7838653   1.66068876  2.03746915  0.23248729  2.86246922 -0.13322984], Cost: 0.3024
Current parameters: [2.17392087e+00 1.24059513e+00 3.09608514e+00 2.36327208e+00
 2.18967716e-01 3.76314478e-01 2.77600387e-01 1.90892050e+00
 6.22544960e-01 3.61801901e-01 2.75136366e+00 1.76488166e+00
 3.10477370e-01 1.80590810e+00 5.17224496e-01 1.60647931e+00
 2.87608141e+00 9.33512723e-01 2.64440718e+00 1.64714323e+00
 1.97910999e+00 2.44202437e-01 2.87933296e+00 1.74517583e-03], Cost: 0.2938
Current parameters: [ 2.1793278   1.23659218  3.09432816  2.2991654   0.36829767  0.44426804
  0.27498253  1.90766756  0.66502974  0.46606447  2.78987029  1.76761111
  0.27871386  1.79982812  0.50817046  1.5930353   2.87058141  0.8911503
  2.48852421  1.6743509   1.93051791  0.23692939  2.86938352 -0.05241453], Cost: 0.6338
Current parameters: [ 2.23779752  1.23595203  3.09536216  2.34003167  0.27711857  0.4619655
```



```
  0.27339665  1.90307902  0.60014981  0.31783747  2.72823513  1.82258877
  0.27076811  1.80239175  0.51529847  1.58247545  2.87402884  0.935572
  2.64906253  1.67455611  1.98144288  0.2412371   2.87698381 -0.07929974], Cost: 0.1539
Current parameters: [ 2.22292264  1.23524062  3.0944003   2.46870787  0.3342348   0.41236199
  0.27442836  1.90643633  0.66179998  0.31986877  2.78652867  1.81019613
  0.27220987  1.80003125  0.50876885  1.58817296  2.87143998  0.8905822
  2.65195928  1.46583487  1.93040002  0.23657469  2.86963199 -0.06741374], Cost: 0.3705
Current parameters: [ 2.16713634  1.24115277  3.09575516  2.3773369   0.2505067   0.49826144
  0.27461243  1.9050857   0.69755721  0.32313874  2.74472775  1.75477077
  0.29235335  1.80325042  0.51760837  1.59552352  2.87274812  0.86010969
  2.65469758  1.66938557  1.97468536  0.245625    2.88107941 -0.03524518], Cost: 0.0695
Current parameters: [ 2.16626972  1.23538954  3.09478315  2.3175111   0.38018008  0.37943761
  0.27754008  1.91048125  0.68481323  0.3278124   2.60299877  1.75643512
  0.29300945  1.80278292  0.50872647  1.60072584  2.8745736   0.88205831
  2.65814787  1.61710498  2.11468944  0.23577641  2.86848571 -0.02500024], Cost: 0.1803
Current parameters: [ 2.16533818  1.25396418  3.09569047  2.42957077  0.33527941  0.41072033
  0.23910655  1.90071635  0.62061512  0.31891615  2.75014093  1.7523139
  0.2888005   1.79826868  0.52303497  1.59337585  2.86194035  0.91864054
  2.65286345  1.71373243  1.96429118  0.22676632  2.89336501 -0.0424964 ], Cost: 0.0636
Current parameters: [ 2.15175125  1.21619217  3.09259053  2.40424067  0.32034576  0.4210211
  0.44871117  1.90815067  0.61362216  0.30562931  2.74186075  1.74165516
  0.26852821  1.79904866  0.49247353  1.59445912  2.87504736  0.91605314
  2.65103387  1.69360857  1.95775386  0.39022369  2.84630543 -0.05915413], Cost: 0.4524
Current parameters: [ 2.17151322  1.16370059  3.09591261  2.35702582  0.306092    0.4545051
  0.26590067  1.81259025  0.63043152  0.33341694  2.75657475  1.76075103
  0.30100132  1.79562706  0.52361323  1.60075113  2.86259668  0.93221349
  2.65916202  1.64894307  1.97645394  0.2535672   2.89015244 -0.01759504], Cost: 0.1502
Current parameters: [ 2.13924006  1.20991899  3.08830423  2.34870296  0.29481669  0.45796997
  0.29255429  1.91982643  0.61519183  0.30360397  2.73745823  1.73617512
  0.25346522  1.98489089  0.46426686  1.60494884  2.864744    0.92862753
  2.65594513  1.642556    1.96282381  0.20686248  3.01941563 -0.05358362], Cost: 0.5775
Current parameters: [ 2.12871763  1.23997194  3.06817704  2.36236745  0.29550484  0.45869304
  0.28116977  1.90825692  0.59779598  0.31530625  2.77640502  1.78080517
  0.27891949  1.81351932  0.60028341  1.60949141  2.89030316  0.91017309
  2.62706547  1.6386329   2.01396525  0.24788488  2.88734637 -0.03996533], Cost: 0.1370
Current parameters: [ 2.19214076  1.2338546   3.13581027  2.32936216  0.30416403  0.45230715
  0.26348706  1.90634922  0.66412096  0.30926101  2.67913098  1.6719387
  0.25005505  1.78710708  0.64386647  1.59102604  2.97199413  0.96751079
  2.71576214  1.65669035  1.87428081  0.20448502  2.84616941 -0.04643797], Cost: 0.5650
Current parameters: [ 2.15058813  1.22929941  3.08865476  2.36441191  0.28961189  0.47267969
  0.26558298  1.91014455  0.59826461  0.31497325  2.74076346  1.74245758
```



```
  0.23808415  1.78084624  0.48092354  1.66886793  2.85625344  0.92586467
  2.64813245  1.64588475  1.97566417  0.2141795   2.838098   -0.02314453], Cost: 0.1653
Current parameters: [ 2.18454851  1.23544877  3.10643304  2.36479953  0.31577799  0.4614765
  0.28614899  1.91676265  0.63799015  0.34177464  2.76529683  1.77368625
  0.29010174  1.81634452  0.52099726  1.61507025  2.88857613  0.93796883
  2.669703    1.65808877  1.98261213  0.23977133  2.87693232 -0.00338192], Cost: 0.0937
Current parameters: [ 2.16391849  1.27882826  3.10688157  2.34972502  0.31138925  0.48466996
  0.26323251  1.90703101  0.62131517  0.31943567  2.75003557  1.75770043
  0.37404566  1.78356106  0.52417087  1.6355669   2.82905171  0.94375884
  2.67813575  1.64452147  1.97742832  0.25664325  2.89092645 -0.05305085], Cost: 0.1318
Current parameters: [ 2.16947902  1.25057288  3.09550355  2.35293632  0.3024342   0.45119869
  0.31307199  1.89453551  0.62474889  0.32563272  2.75342944  1.75692727
  0.29201608  1.79034296  0.52182475  1.58608089  2.85909432  0.92466123
  2.65652505  1.64896833  1.96983124  0.20457842  2.88802371 -0.0230663 ], Cost: 0.1190
Current parameters: [2.15322731 1.25114481 3.08761849 2.3546712  0.30722717 0.48421533
 0.23656122 1.92159386 0.60973854 0.31075065 2.75433186 1.76213002
 0.28003546 1.79464201 0.50628872 1.52070467 2.84594348 0.93336836
 2.66894025 1.64725416 1.98367329 0.26643257 2.85952255 0.02636308], Cost: 0.2745
Current parameters: [ 2.1725007   1.20375416  3.09598732  2.34454227  0.30525456  0.44254817
  0.27590671  1.94077462  0.62866641  0.31867935  2.75580847  1.75997906
  0.29456797  1.78706665  0.5269742   1.60105513  2.854737    0.91713361
  2.65979962  1.6515077   1.96158511  0.24179782  2.89390968 -0.02640904], Cost: 0.0609
Current parameters: [ 2.17459915e+00  1.26779474e+00  3.08588624e+00  2.30626190e+00
  3.20803852e-01  4.16344463e-01  2.67712714e-01  1.88159332e+00
  6.32225450e-01  2.75431551e-01  2.77873028e+00  1.79015373e+00
  2.93757781e-01  1.81751585e+00  5.14824769e-01  1.62422175e+00
  2.87654752e+00  8.75222675e-01  2.67344786e+00  1.66275922e+00
  1.94038974e+00  2.48745835e-01  2.86529543e+00 -1.99456372e-03], Cost: 0.1572
Current parameters: [ 2.14763961  1.24096171  3.08849085  2.34815326  0.29960891  0.44342184
  0.27458512  1.90654912  0.65989113  0.31066496  2.75867676  1.77362048
  0.28500737  1.79684876  0.5104612   1.59686686  2.8747697   0.96065624
  2.64889661  1.65052839  1.96937383  0.24276154  2.88013685 -0.03896006], Cost: 0.0435
Current parameters: [ 2.1408751   1.24940193  3.08375792  2.34743861  0.30251437  0.4532934
  0.26774237  1.90727854  0.64409479  0.30454687  2.76348385  1.77738775
  0.28812865  1.73257294  0.47412504  1.60099471  2.94637851  0.94903195
  2.65130922  1.64849884  1.97895651  0.24991279  2.94396828 -0.03273051], Cost: 0.1068
Current parameters: [ 2.16055903  1.24154592  3.03057932  2.35002157  0.30256886  0.44628879
  0.27574792  1.90810737  0.66395236  0.31996703  2.75323691  1.76691024
  0.29009929  1.79785016  0.51067585  1.60034912  2.87567762  0.96530676
  2.6574986   1.65219592  1.96343422  0.24294111  2.88016668 -0.03584275], Cost: 0.0488
Current parameters: [ 2.12452936  1.19804658  3.08018928  2.3584831   0.30252962  0.44761424
```

```
  0.28053538  1.92492012  0.65436314  0.28533294  2.77045139  1.79036927
  0.34738242  1.80746569  0.48680493  1.58072895  2.91200561  0.95445513
  2.64787513  1.66618711  1.97817885  0.19626931  2.82976811 -0.05663657], Cost: 0.1611
Current parameters: [ 2.13864143  1.23989575  3.0885373   2.34320491  0.29839579  0.4482448
  0.27114609  1.90827241  0.6452523   0.31055812  2.74973914  1.75706013
  0.28492227  1.8411593   0.49222551  1.59317499  2.88262057  0.95861706
  2.64836356  1.64167697  1.96116431  0.24268139  2.90356801 -0.0499713 ], Cost: 0.0668
Current parameters: [ 2.14133136  1.22462324  3.08433448  2.35881619  0.29886127  0.4519239
  0.28145751  1.91976204  0.70154712  0.27231574  2.76851932  1.81028182
  0.26450593  1.81086041  0.51767448  1.62512647  2.82723737  0.98225478
  2.66461055  1.67814775  1.98944252  0.21330479  2.94003735 -0.02537901], Cost: 0.1479
Current parameters: [ 2.15923337  1.24163985  3.08857441  2.37742704  0.30859826  0.43765118
  0.2708262   1.90815419  0.66882458  0.30487215  2.76561249  1.78681859
  0.28201241  1.79621778  0.50752444  1.5958844   2.87235526  0.95376154
  2.65114022  1.60401564  1.95865217  0.241163    2.87989592 -0.04842477], Cost: 0.0089
Current parameters: [ 2.15770162  1.24238375  3.08539068  2.37463459  0.31559497  0.43865261
  0.27104703  1.90905888  0.66963327  0.31720908  2.77447134  1.79474442
  0.28441833  1.796998    0.49638108  1.59620549  2.86383634  0.94768179
  2.63238908  1.605434    1.96192125  0.24248706  2.88200567 -0.04823859], Cost: 0.0292
Current parameters: [ 2.16419053  1.24263796  3.09808372  2.36954165  0.32617822  0.44401501
  0.25818558  1.91308221  0.69118639  0.3168745   2.75614554  1.77576287
  0.27463023  1.79063533  0.53293592  1.59949865  2.88478492  0.960912
  2.63816652  1.6006043   1.92732727  0.23179078  2.87836476 -0.06013001], Cost: 0.0411
Current parameters: [ 2.16065701  1.24056512  3.08871211  2.38491424  0.3095819   0.44050591
  0.281882    1.9086646   0.66672723  0.29405962  2.75301196  1.78930632
  0.28696048  1.79711749  0.50811187  1.59868291  2.87360047  0.95655289
  2.6368468   1.60566926  1.95084186  0.25072368  2.8798838  -0.03856741], Cost: 0.0098
Current parameters: [ 2.15180874  1.23845684  3.09046788  2.38629713  0.31996396  0.43131906
  0.30311999  1.91247694  0.67529641  0.30778015  2.77461946  1.778248
  0.26921367  1.7920663   0.50380153  1.59490791  2.87024509  0.95152442
  2.66887615  1.60971436  1.95437477  0.27331174  2.87287354 -0.07429858], Cost: 0.0180
Current parameters: [ 2.15983165  1.24183519  3.08861919  2.38244939  0.29504897  0.44278725
  0.2709621   1.90780935  0.66199167  0.30600107  2.78419592  1.7878984
  0.28265899  1.79606611  0.50888134  1.59618277  2.87195612  0.95963411
  2.65036848  1.6082599   1.9417268   0.2416181   2.88139666 -0.04707825], Cost: 0.0232
Current parameters: [ 2.15822434  1.24551361  3.09263291  2.38388157  0.27801314  0.39515769
  0.27037022  1.91533577  0.67889004  0.32415895  2.75136698  1.78552335
  0.29043658  1.7962157   0.50947284  1.6034265   2.87381899  0.95766661
  2.65166002  1.59346661  1.96195966  0.24888442  2.87996792 -0.04293826], Cost: 0.0118
Current parameters: [ 2.17426019  1.24146943  3.08931226  2.37625483  0.30724947  0.43316791
  0.27265011  1.90768868  0.67297486  0.29508575  2.77614798  1.77077779
```



```
  0.28414674  1.79737745  0.50737137  1.60078636  2.87478892  0.95629492
  2.64129245  1.603708    1.96784893  0.2405346   2.8805979  -0.05257335], Cost: 0.0111
Current parameters: [ 2.16034996  1.24988107  3.08881595  2.38282402  0.30959975  0.44069449
  0.25161326  1.92042244  0.67242473  0.29620717  2.76475434  1.77324829
  0.27238758  1.79224158  0.50577074  1.5566375   2.86186054  0.96223079
  2.65581943  1.59202093  1.96729132  0.26096022  2.86953898 -0.02764625], Cost: 0.0395
Current parameters: [ 2.15513752  1.23476149  3.08929244  2.38804633  0.30308383  0.44800676
  0.27028196  1.91335659  0.66325969  0.3123192   2.75880991  1.77858619
  0.27374926  1.78966964  0.5023546   1.60294475  2.86850259  0.9634589
  2.64674473  1.60105959  1.96529309  0.23576834  2.87603485 -0.05299878], Cost: 0.0035
Current parameters: [ 2.14915976  1.24489212  3.08543692  2.37828608  0.30349233  0.43859932
  0.25808931  1.90987206  0.65578278  0.29394235  2.75647256  1.77455684
  0.25031308  1.7870411   0.48625077  1.6349626   2.86476567  0.94723919
  2.65153806  1.5945012   1.95442678  0.23657001  2.85138049 -0.04390212], Cost: 0.0309
Current parameters: [ 2.16648318  1.24787753  3.0891937   2.38513523  0.29808396  0.44926661
  0.27173403  1.92987031  0.65811747  0.30954391  2.75394922  1.78926175
  0.26847679  1.79085827  0.50093302  1.59980718  2.87052591  0.96401098
  2.64499146  1.60552343  1.96612076  0.23346639  2.87372487 -0.06369419], Cost: 0.0015
Current parameters: [ 2.19377898  1.22461105  3.09125788  2.38123703  0.2889305   0.4485803
  0.25971831  1.89665024  0.64938403  0.31023742  2.73896094  1.80641298
  0.26021575  1.78879263  0.4985994   1.59364322  2.8678144   0.96972367
  2.64617821  1.60533568  1.96841736  0.2473799   2.87069358 -0.08122125], Cost: 0.0165
Current parameters: [ 2.16038391  1.24086328  3.0824584   2.38712393  0.29521174  0.44430726
  0.27496746  1.93010712  0.65438414  0.30858409  2.75829115  1.79304517
  0.25171264  1.79566092  0.51353317  1.59477865  2.88081109  0.95824417
  2.63606993  1.60458526  1.97190404  0.23159324  2.87320974 -0.06155565], Cost: 0.0035
Current parameters: [ 2.152407    1.26420258  3.08411008  2.3865372   0.29671863  0.45976274
  0.26089963  1.92740618  0.64150738  0.30494406  2.75487179  1.79275079
  0.28829138  1.79027976  0.53291359  1.61611156  2.86381974  0.96367084
  2.64329297  1.59095119  1.98347413  0.25214233  2.87466455 -0.08029387], Cost: 0.0477
Current parameters: [ 2.16580871  1.24889452  3.08885808  2.39021722  0.28432776  0.46168899
  0.26946896  1.92805027  0.67271445  0.30894472  2.75098105  1.7870076
  0.27006781  1.79240389  0.49989087  1.59858099  2.87211411  0.94594431
  2.64409136  1.60906424  1.96730575  0.2359355   2.87182166 -0.06667969], Cost: 0.0037
Current parameters: [ 2.16648318  1.24787753  3.0891937   2.38513523  0.29808396  0.44926661
  0.27173403  1.92987031  0.65811747  0.30954391  2.75394922  1.78926175
  0.26847679  1.79085827  0.50093302  1.59980718  2.87052591  0.96401098
  2.64499146  1.60552343  1.96612076  0.23346639  2.87372487 -0.06369419], Cost: 0.0015

   Return from subroutine COBYLA because the MAXFUN limit has been reached.
```



```
NFVALS =  100   F = 1.506047E-03    MAXCV = 0.000000E+00
X = 2.166483E+00   1.247878E+00   3.089194E+00   2.385135E+00   2.980840E-01
    4.492666E-01   2.717340E-01   1.929870E+00   6.581175E-01   3.095439E-01
    2.753949E+00   1.789262E+00   2.684768E-01   1.790858E+00   5.009330E-01
    1.599807E+00   2.870526E+00   9.640110E-01   2.644991E+00   1.605523E+00
    1.966121E+00   2.334664E-01   2.873725E+00  -6.369419E-02
```

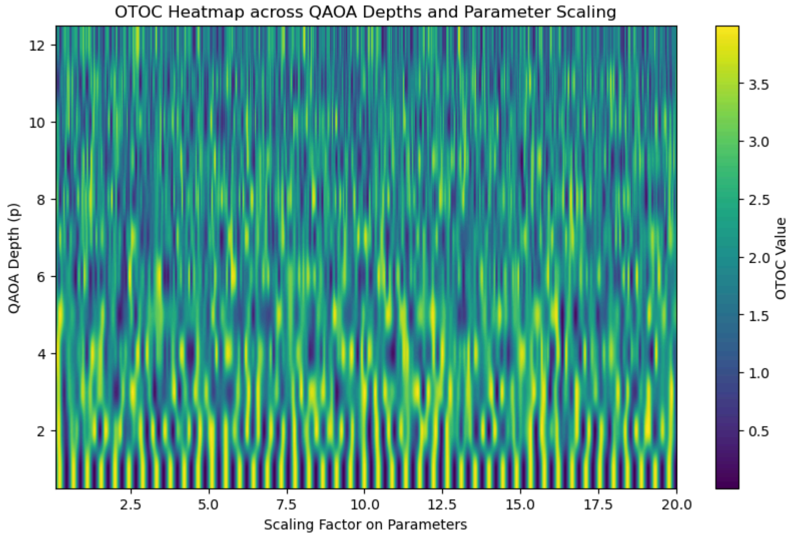


```
Run 9/30 for heatmap generation...
Simulating for QAOA depth p = 1
Current parameters: [1.21785843 1.70628627], Cost: 2.3346
Current parameters: [0.21785843 2.70628627], Cost: 1.5841
Current parameters: [0.03642667 3.6896898 ], Cost: 2.0592
Current parameters: [0.12714255 3.19798804], Cost: 2.0563
Current parameters: [-0.02799246  2.66092833], Cost: 2.0525
Current parameters: [0.6977746  2.56599917], Cost: 1.2673
Current parameters: [0.93705317 3.00502738], Cost: 1.5042
Current parameters: [0.94156599 2.12946079], Cost: 2.7495
Current parameters: [0.81967029 2.34772998], Cost: 2.0338
Current parameters: [0.74760287 2.68063836], Cost: 1.0398
Current parameters: [0.68491036 2.78878016], Cost: 1.0326
Current parameters: [0.79138478 2.8542645 ], Cost: 1.0876
Current parameters: [0.62723318 2.76470543], Cost: 1.0515
Current parameters: [0.70865709 2.80909408], Cost: 1.0403
Current parameters: [0.70171617 2.76243386], Cost: 1.0154
Current parameters: [0.70990304 2.73227532], Cost: 1.0136
Current parameters: [0.74033667 2.73937158], Cost: 1.0048
Current parameters: [0.77153918 2.74109377], Cost: 1.0009
Current parameters: [0.78761092 2.71429337], Cost: 1.0096
Current parameters: [0.77964575 2.771274  ], Cost: 1.0041
Current parameters: [0.78223504 2.72970347], Cost: 1.0030
Current parameters: [0.7743268  2.74839201], Cost: 1.0002
Current parameters: [0.77004968 2.7549297 ], Cost: 1.0008
Current parameters: [0.7820448  2.74960348], Cost: 1.0000
Current parameters: [0.78644366 2.74314707], Cost: 1.0003
Current parameters: [0.78350144 2.75322798], Cost: 1.0002
Current parameters: [0.78010853 2.74934745], Cost: 1.0001
Current parameters: [0.78290307 2.74784904], Cost: 1.0000
Current parameters: [0.78384043 2.74812293], Cost: 1.0000
Current parameters: [0.78479291 2.74833848], Cost: 1.0000
Current parameters: [0.78571434 2.748015  ], Cost: 1.0000
Current parameters: [0.78494285 2.74930346], Cost: 1.0000
Current parameters: [0.78454153 2.74958159], Cost: 1.0000
Current parameters: [0.78543108 2.74931036], Cost: 1.0000
Current parameters: [0.78545077 2.74906701], Cost: 1.0000
Current parameters: [0.78549308 2.74882657], Cost: 1.0000
Current parameters: [0.78573587 2.7488009 ], Cost: 1.0000
Current parameters: [0.78531155 2.74866332], Cost: 1.0000
Current parameters: [0.78541873 2.7487597 ], Cost: 1.0000
```

```
Current parameters: [0.78552652 2.74878939], Cost: 1.0000
Current parameters: [0.78547998 2.74892571], Cost: 1.0000
Current parameters: [0.78538006 2.74892977], Cost: 1.0000
Current parameters: [0.78535177 2.74902568], Cost: 1.0000

   Normal return from subroutine COBYLA

Current parameters: [0.78535177 2.74902568], Cost: 1.0000
   NFVALS =   44   F = 1.000000E+00    MAXCV = 0.000000E+00
   X = 7.853518E-01   2.749026E+00
Simulating for QAOA depth p = 2
Current parameters: [1.26711648 0.95399378 0.56892424 1.34788673], Cost: 2.0299
Current parameters: [0.26711648 1.95399378 0.56892424 1.34788673], Cost: 2.6955
Current parameters: [0.26711648 0.95399378 1.56892424 1.34788673], Cost: 1.4956
Current parameters: [0.26711648 0.95399378 1.56892424 2.34788673], Cost: 2.0191
Current parameters: [-0.08560038  0.10487433  1.61472869  0.95738842], Cost: 1.9968
Current parameters: [0.75053413 0.83811331 1.57517523 1.2945949 ], Cost: 2.0494
Current parameters: [-0.0168951   1.33143725  1.58802217  1.1850705 ], Cost: 1.5077
Current parameters: [0.15907716 0.96560179 1.57618918 1.57292002], Cost: 2.0134
Current parameters: [0.26178374 0.94115591 1.69300846 1.34198276], Cost: 1.6625
Current parameters: [0.16206304 0.81253266 1.47726126 1.19606307], Cost: 0.9960
Current parameters: [0.19349647 0.75588403 1.33580106 1.00037995], Cost: 1.1907
Current parameters: [0.23241908 0.71825889 1.47253028 1.23807618], Cost: 0.9689
Current parameters: [0.04696734 0.61728669 1.39220051 1.34512476], Cost: 1.1971
Current parameters: [0.2069882  0.66857648 1.57373325 1.19045384], Cost: 1.0198
Current parameters: [0.23421856 0.73942567 1.5060836  1.28633749], Cost: 1.0686
Current parameters: [0.29365201 0.70914949 1.40927272 1.14980939], Cost: 0.9239
Current parameters: [0.22306158 0.64270696 1.3309611  1.1400794 ], Cost: 0.9188
Current parameters: [0.20972717 0.61051952 1.37390792 1.11095927], Cost: 0.9863
Current parameters: [0.26530825 0.70709232 1.2352884  1.11680969], Cost: 0.8842
Current parameters: [0.30920065 0.69147488 1.19073127 1.22390411], Cost: 0.7503
Current parameters: [0.28861599 0.7352637  1.15288433 1.33276834], Cost: 0.8018
Current parameters: [0.40561737 0.61841611 1.16081278 1.23370792], Cost: 0.7646
Current parameters: [0.32208438 0.72404157 1.15420966 1.18721839], Cost: 0.7352
Current parameters: [0.28522836 0.6925551  1.12099649 1.16592588], Cost: 0.8223
Current parameters: [0.32499437 0.71701838 1.17292447 1.16337462], Cost: 0.7635
Current parameters: [0.3482201  0.76972059 1.15530444 1.22091413], Cost: 0.6595
Current parameters: [0.36638407 0.81391931 1.14224247 1.25902158], Cost: 0.6168
Current parameters: [0.40047321 0.84692495 1.18176813 1.24940232], Cost: 0.5957
Current parameters: [0.42153778 0.8980393  1.15344181 1.24251195], Cost: 0.5348
Current parameters: [0.4427363  0.94896338 1.12493502 1.23537237], Cost: 0.4706
```



```
Current parameters: [0.46352845 0.99703034 1.09203034 1.22638427], Cost: 0.4026
Current parameters: [0.4822684  1.04160269 1.05365492 1.21660182], Cost: 0.3397
Current parameters: [0.50560265 1.09102136 1.02496816 1.20677185], Cost: 0.2880
Current parameters: [0.54021326 1.13261364 1.05489624 1.19767524], Cost: 0.2612
Current parameters: [0.5534447  1.16986325 1.03909674 1.24343572], Cost: 0.2804
Current parameters: [0.51434988 1.14838043 1.06201334 1.19477644], Cost: 0.3026
Current parameters: [0.58810142 1.12576232 1.03052575 1.16649652], Cost: 0.1805
Current parameters: [0.64416047 1.10418418 1.02848454 1.14935448], Cost: 0.1262
Current parameters: [0.65410526 1.07230248 0.97603664 1.14301999], Cost: 0.1402
Current parameters: [0.63187134 1.0896041  1.03778825 1.12641104], Cost: 0.1534
Current parameters: [0.6838344  1.14811686 1.01936636 1.1314937 ], Cost: 0.0891
Current parameters: [0.73716196 1.15189746 1.03562794 1.15948815], Cost: 0.0619
Current parameters: [0.75442765 1.2013668  1.02252729 1.19094134], Cost: 0.0754
Current parameters: [0.75778763 1.15262922 1.04915501 1.14031454], Cost: 0.0609
Current parameters: [0.76385104 1.14894557 1.03560162 1.13713459], Cost: 0.0446
Current parameters: [0.78068109 1.12943268 1.01837867 1.14112572], Cost: 0.0271
Current parameters: [0.78210088 1.12656352 0.99292236 1.12328501], Cost: 0.0311
Current parameters: [0.78743105 1.14199775 1.01405286 1.14581453], Cost: 0.0237
Current parameters: [0.80928235 1.12958885 1.00865633 1.16359035], Cost: 0.0210
Current parameters: [0.82038257 1.13238971 1.01615692 1.15605232], Cost: 0.0169
Current parameters: [0.83184187 1.14087062 1.02249273 1.15692227], Cost: 0.0182
Current parameters: [0.82237388 1.13490224 1.00956232 1.15335635], Cost: 0.0140
Current parameters: [0.82891711 1.12827286 1.00361094 1.14231284], Cost: 0.0099
Current parameters: [0.82307923 1.13524261 1.00399108 1.12961096], Cost: 0.0109
Current parameters: [0.83446093 1.12630018 0.98919025 1.14106435], Cost: 0.0125
Current parameters: [0.82676971 1.12133777 1.00571365 1.14033622], Cost: 0.0105
Current parameters: [0.83530043 1.12880081 1.00767584 1.14044567], Cost: 0.0082
Current parameters: [0.83926617 1.13127078 1.01365899 1.14229208], Cost: 0.0084
Current parameters: [0.83707258 1.12707841 1.00637013 1.14317456], Cost: 0.0083
Current parameters: [0.8404734  1.1293913  1.00485854 1.13534766], Cost: 0.0067
Current parameters: [0.84208172 1.12347278 1.00702236 1.13101898], Cost: 0.0064
Current parameters: [0.84644748 1.1250322  1.00351001 1.12580297], Cost: 0.0060
Current parameters: [0.84090898 1.12649974 1.0034174  1.1204928 ], Cost: 0.0079
Current parameters: [0.84752642 1.12695488 1.0066686  1.12515392], Cost: 0.0054
Current parameters: [0.85448993 1.12701279 1.00954535 1.12721924], Cost: 0.0039
Current parameters: [0.86080213 1.1283222  1.01395761 1.12712565], Cost: 0.0033
Current parameters: [0.86650843 1.1262529  1.00917161 1.12825943], Cost: 0.0023
Current parameters: [0.8675489  1.11899516 1.01145711 1.12969267], Cost: 0.0025
Current parameters: [0.86623594 1.12704061 1.00940847 1.13206836], Cost: 0.0025
Current parameters: [0.87304262 1.12704773 1.00602196 1.12546883], Cost: 0.0017
Current parameters: [0.8717516  1.1251728  0.99866883 1.12413296], Cost: 0.0033
```



```
Current parameters: [0.87687031 1.12972532 1.01166308 1.12274996], Cost: 0.0013
Current parameters: [0.88116752 1.13137942 1.01041386 1.11656348], Cost: 0.0014
Current parameters: [0.87952012 1.13119368 1.01161773 1.12521555], Cost: 0.0012
Current parameters: [0.88140585 1.1279473  1.01264124 1.12555654], Cost: 0.0011
Current parameters: [0.88137359 1.12848816 1.01450054 1.1253033 ], Cost: 0.0013
Current parameters: [0.88243499 1.12621406 1.00952782 1.12678217], Cost: 0.0009
Current parameters: [0.88454118 1.12400876 1.00801492 1.12869803], Cost: 0.0009
Current parameters: [0.88128366 1.12583031 1.00994095 1.12825567], Cost: 0.0010
Current parameters: [0.88564065 1.12561033 1.00794206 1.12533188], Cost: 0.0007
Current parameters: [0.88863723 1.12390526 1.00616265 1.1257855 ], Cost: 0.0007
Current parameters: [0.88663181 1.12645999 1.008346   1.12672731], Cost: 0.0007
Current parameters: [0.88784077 1.1232401  1.00585041 1.1246797 ], Cost: 0.0007
Current parameters: [0.88531879 1.12820648 1.00515761 1.12451821], Cost: 0.0009
Current parameters: [0.8864473  1.12567703 1.00926225 1.12414165], Cost: 0.0005
Current parameters: [0.88691457 1.12699698 1.00892406 1.12282266], Cost: 0.0005
Current parameters: [0.88620641 1.12745537 1.00940891 1.1229062 ], Cost: 0.0006
Current parameters: [0.88806963 1.12593148 1.0096586  1.12192504], Cost: 0.0005
Current parameters: [0.88841156 1.12452075 1.00934802 1.12065572], Cost: 0.0004
Current parameters: [0.88906517 1.124491   1.00872031 1.12101845], Cost: 0.0004
Current parameters: [0.89071353 1.12431889 1.00974977 1.12110886], Cost: 0.0003
Current parameters: [0.89243669 1.12427236 1.01066703 1.12106542], Cost: 0.0002
Current parameters: [0.89401817 1.12470426 1.00960597 1.12103015], Cost: 0.0002
Current parameters: [0.8948529  1.12351403 1.00974225 1.12232734], Cost: 0.0002
Current parameters: [0.8962416  1.12254033 1.01032493 1.12155364], Cost: 0.0001
Current parameters: [0.89790825 1.12220901 1.01127904 1.12142375], Cost: 0.0001
Current parameters: [0.89861606 1.1206737  1.01061069 1.12070973], Cost: 0.0001
Current parameters: [0.89795045 1.1193947  1.01188423 1.12037222], Cost: 0.0001
Current parameters: [0.89856419 1.12103003 1.01070209 1.11980658], Cost: 0.0001
Current parameters: [0.90008668 1.12046027 1.00979411 1.11921694], Cost: 0.0001
Current parameters: [0.89856419 1.12103003 1.01070209 1.11980658], Cost: 0.0001

   Return from subroutine COBYLA because the MAXFUN limit has been reached.

   NFVALS =  100   F = 5.861593E-05    MAXCV = 0.000000E+00
   X = 8.985642E-01   1.121030E+00   1.010702E+00   1.119807E+00
Simulating for QAOA depth p = 3
Current parameters: [1.65745208 3.0473118  1.85198788 2.9235027  0.50788207 2.11535004], Cost: 1.3492
Current parameters: [1.65745208 4.0473118  1.85198788 2.9235027  0.50788207 2.11535004], Cost: 1.4494
Current parameters: [1.65745208 3.0473118  2.85198788 2.9235027  0.50788207 2.11535004], Cost: 1.5141
Current parameters: [1.65745208 3.0473118  1.85198788 3.9235027  0.50788207 2.11535004], Cost: 2.9193
Current parameters: [1.65745208 3.0473118  1.85198788 2.9235027  1.50788207 2.11535004], Cost: 2.8577
```



```
Current parameters: [1.65745208 3.0473118  1.85198788 2.9235027  0.50788207 3.11535004], Cost: 2.1324
Current parameters: [ 1.89528713  3.0054174   1.78300891  2.26671919 -0.12316271  1.78772348], Cost: 1.2060
Current parameters: [ 2.00529278  2.98604002  1.75110412  2.62497884 -0.41503933  1.63618663], Cost: 1.8249
Current parameters: [ 1.89528713  3.0054174   1.78300891  2.26671919 -0.00796756  1.56584496], Cost: 1.7928
Current parameters: [ 1.99658559  2.98757378  1.75362945  2.08232178 -0.16300817  2.23824208], Cost: 1.2298
Current parameters: [ 1.82564967  3.0054174   1.54290346  2.26671919 -0.12316271  1.78772348], Cost: 1.7322
Current parameters: [ 1.49603449  2.99980788  2.02155275  2.20874993 -0.28500303  1.85183256], Cost: 1.9549
Current parameters: [ 1.89090748  2.75624568  1.78427913  2.26671919 -0.10468877  1.78055607], Cost: 1.7600
Current parameters: [2.15870762 3.29050553 1.97425753 2.14032024 0.07943797 1.71199608], Cost: 0.9228
Current parameters: [ 1.85486299  3.50402983  2.27459604  2.04010009 -0.02121162  1.75332623], Cost: 1.2642
Current parameters: [ 2.22368419  3.27905998  1.95541247  1.99869397 -0.09296357  1.62248845], Cost: 1.0132
Current parameters: [ 2.38503279  3.42275553  2.06078538  2.48538301 -0.14110372  1.79005274], Cost: 0.9775
Current parameters: [2.34462127 2.9666797  2.26873423 2.14676319 0.23124323 1.68424599], Cost: 1.2039
Current parameters: [2.23361632 3.40703082 1.81571372 2.14748752 0.21296215 1.69484014], Cost: 1.6404
Current parameters: [2.13422339 3.28921235 1.98474475 2.18635489 0.0898115  1.59935919], Cost: 1.6545
Current parameters: [2.24725793 3.17757221 2.06822828 2.07050193 0.09707564 1.87899525], Cost: 0.1948
Current parameters: [2.15432008 3.2826802  2.1524077  1.99244634 0.039659   2.04128927], Cost: 0.2466
Current parameters: [2.1770952  3.13067429 2.03756261 1.99787598 0.14324633 1.8665    ], Cost: 0.2160
Current parameters: [ 2.21737189  3.05953605  1.94023504  2.14642845 -0.00865137  1.9987825 ], Cost: 0.0994
Current parameters: [2.25075459 3.1239691  1.85545787 2.13185969 0.03651076 2.02911305], Cost: 0.1201
Current parameters: [2.38559882 2.9414826  1.98726839 2.04967017 0.00793122 2.09050926], Cost: 0.3118
Current parameters: [2.18013067 3.04552297 1.95852271 2.23381896 0.06182887 2.03198972], Cost: 0.4263
Current parameters: [2.23965560e+00 3.03548958e+00 1.91797990e+00 2.16434086e+00
 2.40622329e-03 1.95527345e+00], Cost: 0.1667
Current parameters: [ 2.19500327  3.10141676  1.92132932  2.09732163 -0.11064935  1.98470561], Cost: 0.4321
Current parameters: [ 2.25963749  3.07796547  1.95789626  2.17697726 -0.02966811  2.00844549], Cost: 0.0529
Current parameters: [ 2.32916733  3.02037292  1.98285942  2.11465148 -0.01213578  2.06000765], Cost: 0.1202
Current parameters: [ 2.25516753e+00  3.10350029e+00  1.99387273e+00  2.15111729e+00
 -2.68309575e-03  1.98514298e+00], Cost: 0.0218
Current parameters: [ 2.35070029e+00  3.15381207e+00  1.99278140e+00  2.19742594e+00
 -2.17123480e-03  1.94246660e+00], Cost: 0.0864
Current parameters: [ 2.24472658  3.12761228  1.99900307  2.11976875 -0.04940865  1.98029332], Cost: 0.0840
Current parameters: [ 2.25437079  3.0875389   2.01350403  2.16076369 -0.01394021  1.97437273], Cost: 0.0271
Current parameters: [2.23233477 3.12073159 2.01976823 2.17822938 0.02255384 2.01747643], Cost: 0.0643
Current parameters: [2.2892942  3.07935663 2.01049135 2.1202947  0.00909336 2.01331758], Cost: 0.0182
Current parameters: [2.31438938 3.09162453 2.01113199 2.1314141  0.01125471 2.0050975 ], Cost: 0.0162
Current parameters: [2.31409225 3.08398358 2.00715798 2.13487747 0.02282816 2.00020507], Cost: 0.0231
Current parameters: [2.31842987e+00 3.10221013e+00 2.01064795e+00 2.10758635e+00
 1.79361201e-03 1.99128905e+00], Cost: 0.0290
Current parameters: [2.31045015 3.09848102 2.02170671 2.13038866 0.01954351 2.00492065], Cost: 0.0128
Current parameters: [2.31036897 3.11114321 2.02882288 2.14169491 0.01636424 2.02997351], Cost: 0.0218
```

```
Current parameters: [2.31844409 3.09010109 2.02890003 2.12464041 0.02049147 2.00985315], Cost: 0.0134
Current parameters: [2.30518758 3.09109557 2.03990197 2.14425003 0.00533639 1.99191004], Cost: 0.0123
Current parameters: [ 2.30615327  3.10596288  2.04652197  2.12315115 -0.00332762  1.97810638], Cost: 0.0201
Current parameters: [2.30447813e+00 3.09772742e+00 2.04420317e+00 2.15070550e+00
 2.80301468e-03 2.00344539e+00], Cost: 0.0118
Current parameters: [2.31066638 3.10021729 2.04407789 2.15370418 0.00416431 2.00106206], Cost: 0.0153
Current parameters: [2.29203275 3.09234958 2.04633496 2.15127688 0.00918657 2.00728092], Cost: 0.0135
Current parameters: [2.30094907e+00 3.10115749e+00 2.03963280e+00 2.15362428e+00
 1.51958579e-03 2.00104522e+00], Cost: 0.0121
Current parameters: [ 2.3048719   3.09010532  2.03829323  2.14928056 -0.00798888  2.00914331], Cost: 0.0095
Current parameters: [ 2.30078948  3.09578509  2.04202484  2.13840756 -0.01592808  2.00933449], Cost: 0.0095
Current parameters: [ 2.29942979  3.09385069  2.04655929  2.14188509 -0.01932487  2.00598011], Cost: 0.0094
Current parameters: [ 2.2877821   3.08582031  2.04719308  2.14075778 -0.01735304  2.01217885], Cost: 0.0076
Current parameters: [ 2.28443616  3.07570548  2.04576933  2.14062757 -0.02393755  2.02141117], Cost: 0.0122
Current parameters: [ 2.28701519  3.0904583   2.05033051  2.14300664 -0.01799626  2.01703904], Cost: 0.0058
Current parameters: [ 2.2795773   3.09775     2.05811971  2.13482671 -0.0158507   2.0151781 ], Cost: 0.0054
Current parameters: [ 2.27578084  3.10070422  2.05354895  2.13592362 -0.01954606  2.01371387], Cost: 0.0062
Current parameters: [ 2.27566177  3.10059986  2.05980023  2.14434576 -0.00457057  2.01527659], Cost: 0.0032
Current parameters: [ 2.27297587e+00  3.10183632e+00  2.06726221e+00  2.15016729e+00
 -1.26533787e-03  2.02689131e+00], Cost: 0.0088
Current parameters: [ 2.27432316  3.09840207  2.06415911  2.14769403 -0.00792345  2.01167652], Cost: 0.0032
Current parameters: [ 2.26768197e+00  3.08834349e+00  2.06090290e+00  2.14385100e+00
 -1.16102499e-03  2.00640795e+00], Cost: 0.0033
Current parameters: [ 2.28193692e+00  3.10340027e+00  2.06398461e+00  2.14788748e+00
 -2.21084651e-03  2.00034128e+00], Cost: 0.0069
Current parameters: [ 2.26965535  3.10128423  2.05974212  2.14969291 -0.01031681  2.01037025], Cost: 0.0027
Current parameters: [ 2.27147932  3.0994994   2.0577291   2.1518008  -0.01071431  2.01067501], Cost: 0.0030
Current parameters: [ 2.26429396  3.10172106  2.06166829  2.15153984 -0.00952891  2.01530561], Cost: 0.0028
Current parameters: [ 2.26938522  3.09988552  2.05935083  2.14767752 -0.01307843  2.01154839], Cost: 0.0020
Current parameters: [ 2.26696178  3.1008852   2.05875381  2.14251667 -0.01826017  2.01098142], Cost: 0.0028
Current parameters: [ 2.2708498   3.10229455  2.06026827  2.14878749 -0.01468783  2.01317518], Cost: 0.0021
Current parameters: [ 2.26470111  3.09782838  2.06074192  2.14925563 -0.01238924  2.01702213], Cost: 0.0017
Current parameters: [ 2.26545504  3.0959056   2.06392259  2.14940601 -0.01305736  2.01638369], Cost: 0.0017
Current parameters: [ 2.26573146  3.09764699  2.0608312   2.14835637 -0.01136173  2.01794287], Cost: 0.0018
Current parameters: [ 2.26250605  3.09758325  2.06063682  2.14697671 -0.01465286  2.0172499 ], Cost: 0.0011
Current parameters: [ 2.26154453  3.09564436  2.05911063  2.1464202  -0.01738982  2.01791704], Cost: 0.0013
Current parameters: [ 2.26181446  3.09653926  2.06022537  2.14663012 -0.013593    2.01633678], Cost: 0.0011
Current parameters: [ 2.25907912  3.09826598  2.06172131  2.14510034 -0.01374744  2.01589714], Cost: 0.0009
Current parameters: [ 2.26000228  3.09799041  2.06201387  2.1419526  -0.0127009   2.01769756], Cost: 0.0010
Current parameters: [ 2.25887717  3.09928641  2.06010591  2.14496939 -0.01344353  2.01601377], Cost: 0.0009
Current parameters: [ 2.26040868  3.10004875  2.06002007  2.14337258 -0.01467087  2.01313826], Cost: 0.0014
```



```
Current parameters: [ 2.25627078  3.09987111  2.06067953  2.146332   -0.01190853  2.01790631], Cost: 0.0008
Current parameters: [ 2.2538803   3.10233803  2.06164421  2.14619187 -0.01106868  2.01656352], Cost: 0.0008
Current parameters: [ 2.25369681  3.09883779  2.05966177  2.14567304 -0.0142514   2.01868514], Cost: 0.0005
Current parameters: [ 2.25145885  3.09636796  2.05806185  2.14550483 -0.01328885  2.01788785], Cost: 0.0006
Current parameters: [ 2.25235115  3.09962822  2.05924998  2.14637183 -0.01694114  2.02090566], Cost: 0.0005
Current parameters: [ 2.25019686  3.10157714  2.06045651  2.14482257 -0.01744732  2.01926024], Cost: 0.0005
Current parameters: [ 2.252494    3.10032     2.05810858  2.14525786 -0.01637162  2.02157473], Cost: 0.0005
Current parameters: [ 2.25232767  3.09795128  2.06153988  2.14436704 -0.01696686  2.02268965], Cost: 0.0006
Current parameters: [ 2.2532963   3.09923993  2.05889053  2.14554442 -0.01806188  2.02006854], Cost: 0.0007
Current parameters: [ 2.25007999  3.10023414  2.05914784  2.14899684 -0.0160843   2.02235416], Cost: 0.0005
Current parameters: [ 2.25099422  3.10112035  2.06001927  2.14944701 -0.01498663  2.02219113], Cost: 0.0006
Current parameters: [ 2.24993296  3.09999041  2.05873462  2.14911025 -0.01558163  2.02169357], Cost: 0.0005
Current parameters: [ 2.24939917  3.09903121  2.05990757  2.14825037 -0.01546959  2.02298015], Cost: 0.0004
Current parameters: [ 2.24996933  3.09853165  2.05959416  2.14860795 -0.01538235  2.02336138], Cost: 0.0005
Current parameters: [ 2.24799315  3.09929924  2.06004158  2.14780785 -0.0166644   2.02262718], Cost: 0.0003
Current parameters: [ 2.24688816  3.10026233  2.06108822  2.14739279 -0.01706981  2.02214335], Cost: 0.0002
Current parameters: [ 2.24560563  3.10023739  2.06121008  2.1472229  -0.01852662  2.02208692], Cost: 0.0002
Current parameters: [ 2.24578977  3.09979104  2.06182007  2.14771637 -0.01867648  2.02179973], Cost: 0.0002
Current parameters: [ 2.24508974  3.09971384  2.06323072  2.14701864 -0.01791296  2.02230863], Cost: 0.0002
Current parameters: [ 2.24414804  3.09932735  2.06297487  2.14691797 -0.0170464   2.02091149], Cost: 0.0001
Current parameters: [ 2.24456297  3.09910949  2.06306448  2.14617968 -0.01730118  2.02057085], Cost: 0.0002
Current parameters: [ 2.24342968  3.10057655  2.06365476  2.14790556 -0.01652541  2.02074085], Cost: 0.0001

   Return from subroutine COBYLA because the MAXFUN limit has been reached.

   NFVALS =  100   F = 1.425734E-04    MAXCV = 0.000000E+00
   X = 2.244148E+00   3.099327E+00   2.062975E+00   2.146918E+00  -1.704640E-02
       2.020911E+00
Current parameters: [ 2.24414804  3.09932735  2.06297487  2.14691797 -0.0170464   2.02091149], Cost: 0.0001
Simulating for QAOA depth p = 4
Current parameters: [3.88477707 2.0448113  0.82850748 2.72756376 0.91784119 3.06032856
 0.94434745 0.74983301], Cost: 1.2136
Current parameters: [3.88477707 3.0448113  0.82850748 2.72756376 0.91784119 3.06032856
 0.94434745 0.74983301], Cost: 1.6096
Current parameters: [3.88477707 2.0448113  1.82850748 2.72756376 0.91784119 3.06032856
 0.94434745 0.74983301], Cost: 1.5670
Current parameters: [3.88477707 2.0448113  0.82850748 3.72756376 0.91784119 3.06032856
 0.94434745 0.74983301], Cost: 1.7825
Current parameters: [3.88477707 2.0448113  0.82850748 2.72756376 1.91784119 3.06032856
 0.94434745 0.74983301], Cost: 2.9558
Current parameters: [3.88477707 2.0448113  0.82850748 2.72756376 0.91784119 4.06032856
```



```
 0.94434745 0.74983301], Cost: 2.1505
Current parameters: [3.88477707 2.0448113  0.82850748 2.72756376 0.91784119 3.06032856
 1.94434745 0.74983301], Cost: 2.9537
Current parameters: [3.88477707 2.0448113  0.82850748 2.72756376 0.91784119 3.06032856
 0.94434745 1.74983301], Cost: 2.9482
Current parameters: [4.19343606 1.92887759 0.72501926 2.56099611 0.40778866 2.78603327
 0.43490146 0.24198687], Cost: 2.8087
Current parameters: [4.03910657 1.98684444 0.77676337 2.64427993 0.66281492 2.92318092
 0.68962445 0.49590994], Cost: 2.5883
Current parameters: [3.63838346 2.03965427 0.82390406 2.7201544  0.89515275 3.04812721
 0.921686   0.72724272], Cost: 1.7019
Current parameters: [3.88477707 2.12805251 0.73525567 2.72756376 0.91784119 3.06032856
 0.94434745 0.74983301], Cost: 1.2587
Current parameters: [3.89357165 2.2290876  0.99602919 2.72400759 0.90695173 3.05447245
 0.93347095 0.73899066], Cost: 1.1717
Current parameters: [3.89357165 2.2290876  0.99602919 2.72400759 0.99528748 3.05447245
 0.84503003 0.73899066], Cost: 1.1771
Current parameters: [3.94721561 2.23080586 1.01602956 2.70231619 1.06882888 3.01875212
 1.09746757 0.67285599], Cost: 0.6885
Current parameters: [4.02459308 2.23328433 1.04487863 2.67102792 1.16361939 2.96722817
 1.1954802  0.85574647], Cost: 2.0829
Current parameters: [3.94721561 2.23080586 1.01602956 2.59547314 1.06882888 3.08363327
 1.09746757 0.67285599], Cost: 0.5598
Current parameters: [3.97095597 2.23156629 1.02488081 2.69707938 1.09791178 3.28440367
 1.12753905 0.57551748], Cost: 0.7254
Current parameters: [4.02404223 2.21503305 1.01299892 2.52002332 1.03423241 3.00461024
 1.06429553 0.46766074], Cost: 0.6674
Current parameters: [3.83271611 2.23273631 1.02142678 2.58069351 1.0888924  3.07433282
 1.1179672  0.63605529], Cost: 0.7130
Current parameters: [3.94700185 2.18883493 1.06215949 2.59555958 1.06688709 3.08377562
 1.09552808 0.67587516], Cost: 0.5637
Current parameters: [4.01274714 2.2393383  1.0222259  2.54251781 1.12216931 3.0767316
 1.15320084 0.72201005], Cost: 1.0794
Current parameters: [3.94820534 2.2763977  1.05706781 2.59507294 1.05700373 3.08297424
 1.09669315 0.67163581], Cost: 0.4852
Current parameters: [3.94180729 2.26863089 1.04623132 2.59356446 0.94458041 3.09292108
 1.14751697 0.68074801], Cost: 0.4052
Current parameters: [3.93207796 2.26831925 1.04260388 2.56955134 0.93201017 3.05337748
 1.11983544 0.70783547], Cost: 0.5297
Current parameters: [3.94101856 2.28127055 1.05245405 2.62466297 0.90416288 3.17912795
 1.07642601 0.6623184 ], Cost: 0.2472
```



```
Current parameters: [3.98228304 2.27848141 1.05491001 2.59985595 0.87837144 3.15135796
 1.05902828 0.5548289 ], Cost: 0.4627
Current parameters: [3.97142726 2.31587    1.07946837 2.63989128 0.90287156 3.27264031
 1.11658069 0.70903977], Cost: 0.2429
Current parameters: [3.91479607 2.32292183 1.08644824 2.64151966 0.90487354 3.28930381
 1.12693288 0.69457561], Cost: 0.3105
Current parameters: [3.97153395 2.33681837 1.05644419 2.64143575 0.90384074 3.27160518
 1.11754871 0.70753285], Cost: 0.2481
Current parameters: [3.97483474 2.3173437  1.08615025 2.6995364  0.89599042 3.25764915
 1.11598535 0.71326351], Cost: 0.2655
Current parameters: [3.9697412  2.30306058 1.06706917 2.63964737 0.88032857 3.27504829
 1.12825571 0.71139516], Cost: 0.2600
Current parameters: [3.97724652 2.28294571 1.05149727 2.6496561  0.93725457 3.29467529
 1.10153822 0.71311855], Cost: 0.3136
Current parameters: [3.96105539 2.31700028 1.07813255 2.63717597 0.90003628 3.26819008
 1.10496887 0.7354192 ], Cost: 0.2829
Current parameters: [4.00522938 2.33326432 1.09942459 2.62789588 0.88143214 3.27698942
 1.09453531 0.67814638], Cost: 0.0810
Current parameters: [4.04268277 2.35165473 1.12146761 2.61363836 0.86357648 3.2763926
 1.0801906  0.64730543], Cost: 0.0249
Current parameters: [4.03450408 2.36024509 1.12478111 2.60807507 0.8396186  3.28248486
 1.03190006 0.67524399], Cost: 0.0812
Current parameters: [4.05036429 2.35978385 1.12774687 2.61754144 0.86373669 3.30010845
 1.0900194  0.65904166], Cost: 0.0202
Current parameters: [4.06136264 2.33493837 1.10670626 2.63013426 0.87908966 3.33231432
 1.0657726  0.63247358], Cost: 0.0315
Current parameters: [4.04000068 2.35991685 1.12606155 2.59028465 0.86720574 3.30961836
 1.09188785 0.65486988], Cost: 0.0192
Current parameters: [4.00123273 2.3767744  1.14401164 2.61137839 0.85171172 3.32273979
 1.09453838 0.62432965], Cost: 0.0127
Current parameters: [4.00063851 2.35299924 1.16241363 2.60982999 0.84420429 3.32443274
 1.09702054 0.62645849], Cost: 0.0145
Current parameters: [4.00629622 2.36854978 1.1110512  2.60893412 0.80356473 3.3276841
 1.11355779 0.62029419], Cost: 0.0298
Current parameters: [4.03033182 2.39467919 1.16887266 2.60362107 0.86390155 3.3164408
 1.12139487 0.59040141], Cost: 0.0196
Current parameters: [4.00643072 2.38954274 1.15596461 2.60555324 0.8409369  3.32569954
 1.07287225 0.62825759], Cost: 0.0070
Current parameters: [3.99269483 2.40395664 1.1693347  2.60700406 0.84907728 3.32986762
 1.06630895 0.64476661], Cost: 0.0166
Current parameters: [4.01112279 2.38868703 1.15500863 2.60905491 0.84344775 3.33990654
```

```
 1.07276199 0.62850633], Cost: 0.0021
Current parameters: [4.01285094 2.38040199 1.14967639 2.61193559 0.85166404 3.34574394
 1.05107624 0.61129155], Cost: 0.0088
Current parameters: [4.0061322  2.38851177 1.15322652 2.59543955 0.84380235 3.34476699
 1.07423178 0.62632921], Cost: 0.0014
Current parameters: [4.01319692 2.3931459  1.14412128 2.59664329 0.8186846  3.35768479
 1.07898197 0.62702765], Cost: 0.0010
Current parameters: [4.01103668 2.39874061 1.15161605 2.59910969 0.81781397 3.35901484
 1.08386489 0.61609382], Cost: 0.0009
Current parameters: [4.03779728 2.40343481 1.1573715  2.5883798  0.8248084  3.35970539
 1.09020486 0.61683351], Cost: 0.0043
Current parameters: [4.01214591 2.3883715  1.16148891 2.60182179 0.81466028 3.36201468
 1.08527831 0.61919547], Cost: 0.0016
Current parameters: [4.00290588 2.39101013 1.15166704 2.57440249 0.80867308 3.35416034
 1.07877527 0.60573923], Cost: 0.0038
Current parameters: [4.00400155 2.4062246  1.15470913 2.59729064 0.81987027 3.36747317
 1.08282305 0.62308697], Cost: 0.0041
Current parameters: [4.00918015 2.39794283 1.15031512 2.59789334 0.81826217 3.3577188
 1.09085993 0.61781685], Cost: 0.0027
Current parameters: [4.01242979 2.39305581 1.14478091 2.6041909  0.822065   3.3656609
 1.08053777 0.6080917 ], Cost: 0.0014
Current parameters: [4.01283261 2.39993511 1.14936293 2.59961397 0.81139321 3.36191222
 1.0850417  0.61630879], Cost: 0.0012
Current parameters: [4.01252334 2.40071952 1.15240629 2.60726911 0.81444991 3.34887086
 1.0765016  0.61455155], Cost: 0.0066
Current parameters: [4.01762713 2.39993537 1.15292845 2.59625238 0.81999935 3.36011719
 1.08427538 0.61573686], Cost: 0.0008
Current parameters: [4.01654134 2.39885895 1.1529094  2.59301727 0.81931549 3.36057962
 1.08372673 0.61452346], Cost: 0.0005
Current parameters: [4.01785589 2.39881372 1.15373092 2.59215869 0.81710982 3.35583756
 1.07890831 0.61181724], Cost: 0.0012
Current parameters: [4.01588998 2.40174735 1.15071189 2.59226616 0.82018399 3.36032715
 1.08332713 0.61417604], Cost: 0.0006
Current parameters: [4.01455696 2.40024212 1.15646842 2.59195708 0.81999842 3.36541913
 1.08005036 0.61652113], Cost: 0.0011
Current parameters: [4.01600712 2.40013339 1.15462882 2.59371445 0.81918595 3.36102287
 1.08496533 0.61166701], Cost: 0.0006
Current parameters: [4.01729237 2.39521945 1.14920004 2.59460928 0.82170576 3.36376671
 1.0820708  0.61101372], Cost: 0.0007
Current parameters: [4.01588002 2.39743411 1.15250267 2.59091164 0.81993274 3.36000742
 1.08638646 0.61516349], Cost: 0.0008
```



```
Current parameters: [4.01825122 2.39920887 1.15312986 2.59233493 0.81955988 3.36098503
 1.0838822  0.61444521], Cost: 0.0006
Current parameters: [4.01660852 2.39902516 1.15162214 2.59321545 0.81592582 3.36186695
 1.08432383 0.61468694], Cost: 0.0004
Current parameters: [4.01665432 2.39868204 1.15160818 2.59297864 0.81503589 3.36064466
 1.0832751  0.61418418], Cost: 0.0004
Current parameters: [4.01647514 2.39913282 1.15218073 2.59272886 0.81479933 3.36052258
 1.08326843 0.61469138], Cost: 0.0005
Current parameters: [4.01623436 2.39783014 1.15114278 2.59306983 0.8151352  3.36173607
 1.08215875 0.61369385], Cost: 0.0002
Current parameters: [4.01642761 2.39711594 1.15024582 2.59456338 0.8151673  3.36181193
 1.08172097 0.61351054], Cost: 0.0002
Current parameters: [4.01587881 2.39583647 1.14938593 2.5937955  0.81520746 3.36184057
 1.08223089 0.61297669], Cost: 0.0001
Current parameters: [4.01548003 2.39594103 1.14984406 2.59415103 0.81501868 3.36218838
 1.0825418  0.61122879], Cost: 0.0001
Current parameters: [4.01536533 2.39641394 1.14884231 2.59377203 0.81535128 3.36169667
 1.08210731 0.61291498], Cost: 0.0001
Current parameters: [4.01652235 2.3964741  1.14784858 2.59299755 0.81556273 3.36219194
 1.08162337 0.61231425], Cost: 0.0001
Current parameters: [4.01538291 2.39627737 1.14905647 2.59375805 0.81619295 3.36134934
 1.0819169  0.6127622 ], Cost: 0.0001
Current parameters: [4.01427001 2.39516688 1.14879531 2.59338872 0.81632017 3.36184013
 1.08099445 0.61293655], Cost: 0.0000
Current parameters: [4.01433374 2.3950795  1.14883378 2.59326215 0.81586827 3.36112629
 1.08058226 0.61273456], Cost: 0.0001
Current parameters: [4.0139274  2.39460825 1.14804367 2.59353871 0.81718533 3.36233725
 1.08229725 0.6132604 ], Cost: 0.0000
Current parameters: [4.01417125 2.39469508 1.14776459 2.59513156 0.8175643  3.36302026
 1.08162846 0.61353467], Cost: 0.0000
Current parameters: [4.01376233 2.39495124 1.14850307 2.59319311 0.81733778 3.36293496
 1.08203056 0.61342372], Cost: 0.0000
Current parameters: [4.01397156 2.39456064 1.14741815 2.59351239 0.81791033 3.3617691
 1.08386705 0.61358568], Cost: 0.0001
Current parameters: [4.01365412 2.39436421 1.14766969 2.59350028 0.81648211 3.3627208
 1.08241222 0.61341287], Cost: 0.0000
Current parameters: [4.01335146 2.39437095 1.14793125 2.59370891 0.81641588 3.36265418
 1.08255185 0.6133322 ], Cost: 0.0000
Current parameters: [4.01340784 2.39431371 1.14779204 2.59368784 0.81645878 3.3629746
 1.08264947 0.61243034], Cost: 0.0000
Current parameters: [4.01367732 2.3942681  1.14817441 2.59376494 0.81628192 3.36275146
```



```
 1.08272717 0.61336743], Cost: 0.0000
Current parameters: [4.01319343 2.3934878  1.14807722 2.59360008 0.8166551  3.36264643
 1.08231875 0.61339535], Cost: 0.0000
Current parameters: [4.01341146 2.39439526 1.14804663 2.59371345 0.81616206 3.36251118
 1.08220191 0.61321573], Cost: 0.0000
Current parameters: [4.01341837 2.39439328 1.14790105 2.59373352 0.81561302 3.36174088
 1.08221745 0.61340804], Cost: 0.0000
Current parameters: [4.01352482 2.39439437 1.14784744 2.59455402 0.81619825 3.36270474
 1.08184776 0.61339004], Cost: 0.0000
Current parameters: [4.01329464 2.39456573 1.14826911 2.59356417 0.81606947 3.36272396
 1.08199089 0.61337558], Cost: 0.0000
Current parameters: [4.01335159 2.3946272  1.14836857 2.59357532 0.81622681 3.36260546
 1.08195547 0.61342573], Cost: 0.0000
Current parameters: [4.0133301  2.39466855 1.14837288 2.59352639 0.81609295 3.36279812
 1.08185244 0.61294063], Cost: 0.0000
Current parameters: [4.01325745 2.39435989 1.14831899 2.59353856 0.81614598 3.36276094
 1.08191691 0.613372  ], Cost: 0.0000
Current parameters: [4.01323147 2.39452441 1.14829587 2.59349008 0.81575745 3.36240729
 1.0818866  0.61350975], Cost: 0.0000
Current parameters: [4.01333369 2.3945954  1.14819423 2.59396735 0.81603125 3.36281128
 1.08177391 0.61348371], Cost: 0.0000
Current parameters: [4.01344334 2.39457018 1.1481452  2.59345801 0.81608381 3.36275185
 1.08190121 0.6134183 ], Cost: 0.0000
Current parameters: [4.01337729 2.39454089 1.1483328  2.59357822 0.81602893 3.36274402
 1.0820244  0.61338099], Cost: 0.0000
Current parameters: [4.01329464 2.39456573 1.14826911 2.59356417 0.81606947 3.36272396
 1.08199089 0.61337558], Cost: 0.0000

   Return from subroutine COBYLA because the MAXFUN limit has been reached.

   NFVALS =  100   F = 1.301877E-06    MAXCV = 0.000000E+00
   X = 4.013295E+00   2.394566E+00   1.148269E+00   2.593564E+00   8.160695E-01
       3.362724E+00   1.081991E+00   6.133756E-01
Simulating for QAOA depth p = 5
Current parameters: [2.67393646 2.01183393 3.11860273 1.28091223 1.43663727 2.6348918
 0.70958089 2.80170049 2.50570428 0.49690131], Cost: 2.2223
Current parameters: [1.67393646 3.01183393 3.11860273 1.28091223 1.43663727 2.6348918
 0.70958089 2.80170049 2.50570428 0.49690131], Cost: 1.4464
Current parameters: [1.67393646 2.01183393 4.11860273 1.28091223 1.43663727 2.6348918
 0.70958089 2.80170049 2.50570428 0.49690131], Cost: 1.1373
Current parameters: [1.67393646 2.01183393 3.11860273 2.28091223 1.43663727 2.6348918
```



```
 0.70958089 2.80170049 2.50570428 0.49690131], Cost: 1.2575
Current parameters: [1.67393646 2.01183393 3.11860273 1.28091223 2.43663727 2.6348918
 0.70958089 2.80170049 2.50570428 0.49690131], Cost: 1.9044
Current parameters: [1.67393646 2.01183393 3.11860273 1.28091223 1.43663727 3.6348918
 0.70958089 2.80170049 2.50570428 0.49690131], Cost: 3.2094
Current parameters: [1.67393646 2.01183393 3.11860273 1.28091223 1.43663727 2.6348918
 1.70958089 2.80170049 2.50570428 0.49690131], Cost: 1.7247
Current parameters: [1.67393646 2.01183393 3.11860273 1.28091223 1.43663727 2.6348918
 0.70958089 3.80170049 2.50570428 0.49690131], Cost: 1.6509
Current parameters: [1.67393646 2.01183393 3.11860273 1.28091223 1.43663727 2.6348918
 0.70958089 2.80170049 3.50570428 0.49690131], Cost: 2.2700
Current parameters: [1.67393646 2.01183393 3.11860273 1.28091223 1.43663727 2.6348918
 0.70958089 2.80170049 2.50570428 1.49690131], Cost: 1.4686
Current parameters: [1.29284477 1.88543879 3.09368495 1.21653998 1.15989383 1.92971456
 0.49182351 2.60817366 2.10892796 0.36324326], Cost: 2.3716
Current parameters: [1.48339062 1.94863636 3.10614384 1.2487261  1.29826555 2.28230318
 0.6007022  2.70493708 2.30731612 0.43007228], Cost: 1.3545
Current parameters: [1.91088507 2.0009352  3.11645413 1.27536157 1.41277439 2.57408617
 0.69080423 2.78501317 2.47149127 0.48537632], Cost: 1.2699
Current parameters: [1.67393646 2.03601132 2.9959632  1.28091223 1.43663727 2.6348918
 0.70958089 2.80170049 2.50570428 0.49690131], Cost: 1.0657
Current parameters: [1.66836442 2.25560016 3.16699519 1.27904511 1.42861033 2.61443814
 0.70326484 2.79608725 2.49419578 0.49302456], Cost: 0.8671
Current parameters: [1.66836442 2.25560016 3.16699519 1.27904511 1.42861033 2.67573419
 0.70326484 2.79608725 2.38525637 0.49302456], Cost: 0.5858
Current parameters: [1.62267352 2.32023174 3.18249129 1.2637347  1.36278919 2.8677292
 0.65147304 2.75005849 2.29169116 0.46123509], Cost: 1.0592
Current parameters: [1.66836442 2.25560016 3.16699519 1.27904511 1.50590738 2.67573419
 0.60502958 2.79608725 2.38525637 0.49302456], Cost: 0.8493
Current parameters: [1.65254161 2.27798215 3.1723615  1.2737431  1.56872231 2.64659023
 0.88603126 2.78014744 2.29904613 0.48201583], Cost: 0.1788
Current parameters: [1.64194417 2.29297264 3.17595563 1.27019204 1.55506423 2.62707086
 0.92385401 3.01837229 2.24130623 0.47464266], Cost: 0.5138
Current parameters: [1.65254161 2.27798215 3.1723615  1.16112394 1.56872231 2.64659023
 0.88603126 2.78014744 2.29904613 0.53625535], Cost: 0.3378
Current parameters: [1.64521977 2.28833917 3.17484471 1.39336275 1.55928586 2.63310417
 0.91216323 2.75440717 2.25915327 0.69371442], Cost: 0.5026
Current parameters: [1.61351338 2.26503781 3.16980963 1.2660627  1.51727193 2.56859621
 0.86688058 2.7570573  2.23592466 0.46606878], Cost: 0.4564
Current parameters: [1.64211421 2.327602   3.18397612 1.30054243 1.74942217 2.67888428
 0.97793064 2.71249633 2.3540719  0.38542034], Cost: 0.1870
```

```
Current parameters: [1.77171814 2.28352683 3.17369189 1.27538458 1.56087508 2.6385292
 0.90041102 2.7658881  2.27487637 0.46570928], Cost: 0.2699
Current parameters: [1.65260357 2.26319398 3.23308604 1.27376386 1.56881157 2.64681768
 0.88610149 2.78020986 2.29917411 0.48205894], Cost: 0.1811
Current parameters: [1.65168644 2.16405809 3.1442214  1.27888728 1.57706492 2.67887702
 0.91158985 2.77787551 2.29906893 0.47447855], Cost: 0.3307
Current parameters: [1.64635932 2.28672724 3.17445823 1.2716715  1.57414483 2.68637968
 0.87371351 2.77391943 2.2547602  0.4777145 ], Cost: 0.3446
Current parameters: [1.64074133 2.30866016 3.179286   1.27841243 1.48990186 2.68286676
 0.94332326 2.76602029 2.35467282 0.46278214], Cost: 0.1161
Current parameters: [1.64444131 2.3034264  3.17803115 1.27965224 1.48635667 2.68968172
 0.90768385 2.7199784  2.37483204 0.4653564 ], Cost: 0.1704
Current parameters: [1.64924408 2.32856433 3.18307449 1.30094982 1.49617754 2.67345253
 0.90949737 2.84898995 2.43282281 0.44352981], Cost: 0.1324
Current parameters: [1.64715033 2.30419503 3.1781782  1.30245638 1.46645359 2.68713938
 0.93547835 2.77634902 2.34856754 0.51270531], Cost: 0.1150
Current parameters: [1.63471756 2.3374946  3.18547714 1.34463479 1.52817409 2.65943545
 1.00328685 2.74926251 2.3841745  0.54948597], Cost: 0.0459
Current parameters: [1.61642047 2.33984524 3.16199633 1.35458746 1.51504728 2.60922642
 0.99303478 2.74607669 2.37617055 0.54293158], Cost: 0.1231
Current parameters: [1.64349111 2.33487828 3.280453   1.34838713 1.49179648 2.67090795
 1.07266503 2.75368455 2.39742005 0.54289762], Cost: 0.1115
Current parameters: [1.63517097 2.34281611 3.18099433 1.29352451 1.5308404  2.65023417
 1.01011473 2.75471113 2.39968878 0.57840994], Cost: 0.1091
Current parameters: [1.60602214 2.32993626 3.1884332  1.3444633  1.52940857 2.66717758
 1.00147004 2.75168653 2.38725748 0.55214922], Cost: 0.0401
Current parameters: [1.61776853 2.27918012 3.16736752 1.35206808 1.53601046 2.67097851
 1.02181813 2.75489233 2.4013601  0.54910744], Cost: 0.0272
Current parameters: [1.61478875 2.29056809 3.15134774 1.35286056 1.5200629  2.68173566
 1.03267826 2.75142538 2.40921506 0.5447448 ], Cost: 0.0248
Current parameters: [1.61612696 2.29909562 3.13894949 1.37397647 1.55375502 2.71878203
 1.04173848 2.76494295 2.38778454 0.54405838], Cost: 0.0384
Current parameters: [1.61300219 2.2853767  3.15034084 1.34750284 1.51828844 2.68450626
 1.03826616 2.72802576 2.39178872 0.54958547], Cost: 0.0236
Current parameters: [1.61949744 2.28249445 3.15654233 1.364441   1.52665146 2.70368858
 1.01174323 2.69322761 2.42610505 0.55029638], Cost: 0.1072
Current parameters: [1.61630484 2.28287848 3.14972873 1.35918188 1.50607522 2.68686346
 1.03380249 2.73340476 2.38845231 0.57424172], Cost: 0.0314
Current parameters: [1.61999117 2.26675914 3.15040212 1.32898388 1.49168811 2.70736508
 1.01296694 2.74530135 2.37034696 0.52635863], Cost: 0.0587
Current parameters: [1.61139828 2.28245383 3.19033692 1.3593071  1.5043862  2.68519393
```



```
 1.08084255 2.73477012 2.38936509 0.53967322], Cost: 0.0295
Current parameters: [1.6023679  2.2848268  3.1379394  1.35870379 1.51693251 2.66422163
 1.04209114 2.73064781 2.3821476  0.54209189], Cost: 0.0155
Current parameters: [1.58828013 2.28135265 3.13918893 1.35629538 1.51766069 2.66760972
 1.04150836 2.73208736 2.38436885 0.54471653], Cost: 0.0187
Current parameters: [1.60614285 2.28841884 3.12862137 1.34018073 1.52265956 2.65280177
 1.05705438 2.73824454 2.38048817 0.55057157], Cost: 0.0152
Current parameters: [1.60926908 2.27570362 3.12391723 1.34238671 1.52406837 2.65389102
 1.06081302 2.73741313 2.38570402 0.54986412], Cost: 0.0172
Current parameters: [1.60566447 2.30224336 3.12580664 1.34668123 1.52929111 2.63631633
 1.05817589 2.7211077  2.3909087  0.54660006], Cost: 0.0556
Current parameters: [1.606444   2.28490652 3.13130665 1.33372935 1.51224845 2.64641451
 1.05279593 2.73667428 2.38361243 0.54874588], Cost: 0.0262
Current parameters: [1.60866069 2.28666624 3.12090131 1.33910829 1.52437073 2.66518906
 1.05679032 2.75247502 2.35998282 0.5393029 ], Cost: 0.0041
Current parameters: [1.61193775 2.28614774 3.11674143 1.35216057 1.5153507  2.66736263
 1.05639171 2.76149626 2.35085162 0.56225681], Cost: 0.0036
Current parameters: [1.61055063 2.2900462  3.11695794 1.35434494 1.50706569 2.67157177
 1.06726325 2.76129861 2.35351078 0.55901006], Cost: 0.0076
Current parameters: [1.61340095 2.27838296 3.14037514 1.3567882  1.5225092  2.66027039
 1.0665423  2.76831836 2.3418263  0.56099732], Cost: 0.0109
Current parameters: [1.61281849 2.28866596 3.1171051  1.35472299 1.51618055 2.66587999
 1.0537249  2.77346653 2.35929886 0.55987283], Cost: 0.0016
Current parameters: [1.61467043 2.30091508 3.10181814 1.3651491  1.53595958 2.66894392
 1.04959083 2.77634112 2.35319229 0.55569489], Cost: 0.0046
Current parameters: [1.61658436 2.29448873 3.12249482 1.34915427 1.5205365  2.67413646
 1.05431307 2.77284673 2.36245602 0.56604967], Cost: 0.0031
Current parameters: [1.62471383 2.27085278 3.11147176 1.3520676  1.51196564 2.68130204
 1.04351287 2.78050493 2.35723335 0.55179372], Cost: 0.0139
Current parameters: [1.59896541 2.28576614 3.11616702 1.35243259 1.51861771 2.66965416
 1.05325196 2.77677686 2.3595406  0.56237748], Cost: 0.0027
Current parameters: [1.614329   2.28728242 3.11387755 1.35054733 1.51756402 2.66381425
 1.05770252 2.77573297 2.35886881 0.56116193], Cost: 0.0017
Current parameters: [1.611064   2.2968637  3.11742007 1.34881388 1.51114981 2.65860195
 1.04709108 2.77572567 2.35594817 0.56034834], Cost: 0.0014
Current parameters: [1.61090328 2.29709305 3.11288948 1.34840908 1.50964411 2.6586881
 1.0453319  2.77268785 2.36065555 0.56222172], Cost: 0.0020
Current parameters: [1.61064979 2.29486676 3.12394104 1.35287468 1.51610843 2.64752665
 1.04748735 2.77506391 2.35994091 0.56449336], Cost: 0.0034
Current parameters: [1.61198557 2.29654814 3.11638285 1.35201347 1.50879197 2.65929123
 1.04693085 2.77802346 2.35366851 0.56602603], Cost: 0.0021
```



```
Current parameters: [1.61635363 2.28839968 3.11811569 1.3452299  1.50995026 2.66034957
 1.03853783 2.78038363 2.35514995 0.55476202], Cost: 0.0023
Current parameters: [1.61234836 2.30280975 3.11025923 1.35352114 1.52034653 2.65887303
 1.04429636 2.77743477 2.3518269  0.55572986], Cost: 0.0015
Current parameters: [1.61102216 2.29931595 3.11745113 1.35006157 1.50757465 2.65907396
 1.05070404 2.77774479 2.35694436 0.55562236], Cost: 0.0031
Current parameters: [1.61017614 2.29531898 3.11628269 1.34955224 1.51015092 2.65716284
 1.04736931 2.77407072 2.35405781 0.55946122], Cost: 0.0014
Current parameters: [1.61162749 2.29647092 3.11800235 1.34629683 1.51192114 2.65960088
 1.04732713 2.76939859 2.35062831 0.56063472], Cost: 0.0026
Current parameters: [1.60876623 2.29585772 3.11782079 1.35149017 1.50958988 2.6583585
 1.04539511 2.77311867 2.35428046 0.55894974], Cost: 0.0015
Current parameters: [1.60378154 2.29374422 3.11521633 1.34727459 1.51204848 2.65829465
 1.04734974 2.77647293 2.35367135 0.5601943 ], Cost: 0.0008
Current parameters: [1.60261845 2.29523798 3.11602338 1.34827011 1.51303927 2.6566136
 1.04916292 2.77541512 2.35428631 0.56139425], Cost: 0.0005
Current parameters: [1.60210062 2.29199723 3.11829675 1.34879551 1.51656832 2.65185598
 1.04686901 2.77679777 2.35587415 0.56158444], Cost: 0.0006
Current parameters: [1.6021796  2.29731282 3.1161009  1.34681418 1.51169739 2.65480537
 1.04756401 2.77600734 2.35347859 0.5614686 ], Cost: 0.0008
Current parameters: [1.59952622 2.29562715 3.11658676 1.3498746  1.51252055 2.65609376
 1.0539381  2.77795728 2.35621122 0.55753154], Cost: 0.0010
Current parameters: [1.60236891 2.29374745 3.11790646 1.34733634 1.5106921  2.65658765
 1.04991318 2.77527072 2.35495413 0.56281457], Cost: 0.0006
Current parameters: [1.60215615 2.29502043 3.11451897 1.3524233  1.51305108 2.65682268
 1.04966415 2.77863991 2.35175474 0.5663094 ], Cost: 0.0008
Current parameters: [1.60183154 2.29509285 3.11374197 1.34830596 1.51283267 2.65643104
 1.04867661 2.77417489 2.35685955 0.56236386], Cost: 0.0005
Current parameters: [1.60256148 2.29484596 3.11300681 1.34728002 1.51314516 2.65587987
 1.04966019 2.77456569 2.35668725 0.56264072], Cost: 0.0005
Current parameters: [1.60140928 2.29492808 3.11357153 1.34622954 1.51530399 2.65681106
 1.05022762 2.7722685  2.35594993 0.56355272], Cost: 0.0005
Current parameters: [1.60229407 2.29412051 3.11247012 1.34770934 1.5125966  2.65513321
 1.04979912 2.77367706 2.3557575  0.56217949], Cost: 0.0006
Current parameters: [1.60539108 2.29591411 3.1138414  1.34848866 1.51349761 2.65555824
 1.05015113 2.77308355 2.35780463 0.56284053], Cost: 0.0008
Current parameters: [1.60227574 2.29495325 3.11334905 1.34662807 1.51308882 2.65576836
 1.05010828 2.77426548 2.3574216  0.56111312], Cost: 0.0004
Current parameters: [1.60003139 2.29535336 3.11317372 1.34744662 1.51365316 2.65612915
 1.05231964 2.77591401 2.35834026 0.5604345 ], Cost: 0.0002
Current parameters: [1.59930128 2.29391863 3.11247839 1.34643638 1.51427639 2.65832404
```



```
 1.05206619 2.77730684 2.35922097 0.55865199], Cost: 0.0002
Current parameters: [1.60012559 2.29631337 3.11261809 1.34720495 1.51267826 2.65716546
 1.05276343 2.77561796 2.35788595 0.56039324], Cost: 0.0003
Current parameters: [1.59810654 2.29694241 3.11367202 1.34605532 1.51406195 2.65401341
 1.05152841 2.77698139 2.35889854 0.56008218], Cost: 0.0002
Current parameters: [1.59967294 2.29456145 3.11415899 1.34698497 1.51271563 2.65613209
 1.05286131 2.77589914 2.35887913 0.56107655], Cost: 0.0002
Current parameters: [1.59990919 2.29533657 3.11261845 1.34749496 1.51358973 2.65606383
 1.05220701 2.77558234 2.35900468 0.5606698 ], Cost: 0.0002
Current parameters: [1.60064431 2.29555842 3.11385882 1.34820796 1.51433307 2.65583376
 1.05316756 2.77584375 2.35925637 0.55994945], Cost: 0.0002
Current parameters: [1.60028743 2.29526147 3.11298214 1.34774633 1.51306992 2.65582703
 1.05219448 2.77638917 2.358429   0.56016545], Cost: 0.0002
Current parameters: [1.59916682 2.29453181 3.11286407 1.34777518 1.51377896 2.65551291
 1.05286561 2.77505921 2.35767681 0.55986534], Cost: 0.0001
Current parameters: [1.59953197 2.2944083  3.11249629 1.3472626  1.51393524 2.65523739
 1.05335713 2.77525479 2.35759022 0.5600047 ], Cost: 0.0001
Current parameters: [1.59850746 2.2941019  3.11274251 1.34863218 1.51453293 2.65559817
 1.05295441 2.77557146 2.35727314 0.56106407], Cost: 0.0001
Current parameters: [1.59883288 2.29437839 3.11290437 1.34878635 1.51435713 2.65510178
 1.05284172 2.77514934 2.35701556 0.56149629], Cost: 0.0001
Current parameters: [1.59847136 2.29286729 3.1129585  1.34813022 1.51540343 2.65597447
 1.05206822 2.77517188 2.35730471 0.56068306], Cost: 0.0001
Current parameters: [1.59714461 2.29334492 3.11325402 1.34740546 1.51596616 2.65525977
 1.05176302 2.77551499 2.35755812 0.5603497 ], Cost: 0.0001
Current parameters: [1.59688364 2.29336986 3.11289113 1.34703434 1.5155388  2.65552088
 1.05144538 2.77551287 2.35708977 0.56056056], Cost: 0.0001
Current parameters: [1.59633131 2.29330202 3.11332442 1.34735172 1.51659188 2.65646774
 1.05249164 2.77491205 2.35760354 0.55972012], Cost: 0.0001
Current parameters: [1.59714461 2.29334492 3.11325402 1.34740546 1.51596616 2.65525977
 1.05176302 2.77551499 2.35755812 0.5603497 ], Cost: 0.0001

   Return from subroutine COBYLA because the MAXFUN limit has been reached.

   NFVALS =  100   F = 5.895989E-05    MAXCV = 0.000000E+00
   X = 1.597145E+00   2.293345E+00   3.113254E+00   1.347405E+00   1.515966E+00
       2.655260E+00   1.051763E+00   2.775515E+00   2.357558E+00   5.603497E-01
Simulating for QAOA depth p = 6
Current parameters: [1.14239931 2.28271999 0.02125375 2.88306986 0.15063413 0.02977125
 2.23171804 1.210444   0.53782152 2.86825874 0.83124884 2.50973388], Cost: 1.6437
Current parameters: [0.14239931 3.28271999 0.02125375 2.88306986 0.15063413 0.02977125
```

```
 2.23171804 1.210444   0.53782152 2.86825874 0.83124884 2.50973388], Cost: 2.4910
Current parameters: [0.14239931 2.28271999 1.02125375 2.88306986 0.15063413 0.02977125
 2.23171804 1.210444   0.53782152 2.86825874 0.83124884 2.50973388], Cost: 2.2549
Current parameters: [0.14239931 2.28271999 0.02125375 3.88306986 0.15063413 0.02977125
 2.23171804 1.210444   0.53782152 2.86825874 0.83124884 2.50973388], Cost: 1.8184
Current parameters: [0.14239931 2.28271999 0.02125375 2.88306986 1.15063413 0.02977125
 2.23171804 1.210444   0.53782152 2.86825874 0.83124884 2.50973388], Cost: 1.9369
Current parameters: [0.14239931 2.28271999 0.02125375 2.88306986 0.15063413 1.02977125
 2.23171804 1.210444   0.53782152 2.86825874 0.83124884 2.50973388], Cost: 2.0085
Current parameters: [0.14239931 2.28271999 0.02125375 2.88306986 0.15063413 0.02977125
 3.23171804 1.210444   0.53782152 2.86825874 0.83124884 2.50973388], Cost: 1.6204
Current parameters: [0.14239931 2.28271999 0.02125375 2.88306986 0.15063413 0.02977125
 2.23171804 2.210444   0.53782152 2.86825874 0.83124884 2.50973388], Cost: 3.3037
Current parameters: [0.14239931 2.28271999 0.02125375 2.88306986 0.15063413 0.02977125
 2.23171804 1.210444   1.53782152 2.86825874 0.83124884 2.50973388], Cost: 3.2515
Current parameters: [0.14239931 2.28271999 0.02125375 2.88306986 0.15063413 0.02977125
 2.23171804 1.210444   0.53782152 3.86825874 0.83124884 2.50973388], Cost: 2.6182
Current parameters: [0.14239931 2.28271999 0.02125375 2.88306986 0.15063413 0.02977125
 2.23171804 1.210444   0.53782152 2.86825874 1.83124884 2.50973388], Cost: 2.5285
Current parameters: [0.14239931 2.28271999 0.02125375 2.88306986 0.15063413 0.02977125
 2.23171804 1.210444   0.53782152 2.86825874 0.83124884 3.50973388], Cost: 2.5349
Current parameters: [ 0.09578669  1.99381384 -0.20012313  2.78649314  0.02017753 -0.12116714
  2.19176331  0.68913574  0.03144842  2.5429843   0.5316198   2.20828576], Cost: 0.9785
Current parameters: [ 0.06271036  1.78880596 -0.35721221  2.71796228 -0.0723945  -0.22827307
  2.16341143  1.48494264 -0.32787406  2.31216948  0.31900296  1.9943781 ], Cost: 1.7656
Current parameters: [ 0.01292109  1.91094824 -0.28298872  2.70362754 -0.06268807 -0.20403274
  2.10889772  0.73957759  0.45428237  2.46011871  0.4487542   2.12542017], Cost: 2.3635
Current parameters: [ 0.37490902  2.01628424 -0.10612164  3.01269048  0.21048726  0.04744703
  2.47793808  0.19207942 -0.65229805  2.52693122  0.54273187  2.21747095], Cost: 1.9283
Current parameters: [ 0.10980782  1.86253803 -0.29090343  2.77055214 -0.01608032 -0.16970738
  2.20977701  0.49539359  0.37912798  2.38989941  0.39391377  2.06948888], Cost: 0.7833
Current parameters: [ 0.07021619  1.7574142  -0.37776288  2.71744692 -0.07834891 -0.2375156
  2.17198611  0.43596112  0.4173637   2.82426581  0.28588976  1.96097287], Cost: 0.7726
Current parameters: [ 0.07039754  1.75759555 -0.37758153  2.71762827 -0.07816756 -0.23733425
  2.17216746  0.43585073  0.41725647  2.82419694  0.10820831  2.13683942], Cost: 0.6106
Current parameters: [-0.00538016  1.57358517 -0.53142654  2.61953157 -0.19139843 -0.35971435
  2.09936384  0.34401593  0.48654882  2.79639133  0.29573377  2.44479099], Cost: 2.1291
Current parameters: [ 0.07905966  1.89921441 -0.58248541  2.72629039 -0.06950544 -0.22867214
  2.18082958  0.43057794  0.41213474  2.82090693  0.1051777   2.13379041], Cost: 0.5055
Current parameters: [ 0.01481665  2.25361298 -0.39011618  2.64808251 -0.15718267 -0.32207398
  2.11844744  0.38336179  0.4654414   2.80990117 -0.0872482   2.01283775], Cost: 0.5707
```



```
Current parameters: [ 0.08521508  1.89713788 -0.58407656  2.73244581 -0.25995223 -0.06721804
  2.186985    0.42683101  0.40849516  2.81856901  0.1030241   2.13162374], Cost: 0.4316
Current parameters: [-0.02883547  1.90883663 -0.5901427   2.60999347  0.00540923  0.28186628
  2.07405402  0.44432111  0.48514866  2.8405854   0.01363339  2.08539419], Cost: 0.8965
Current parameters: [-0.1205824   1.88700795 -0.59183871  2.86673054 -0.26452644 -0.07251041
  2.2170131   0.40855235  0.39074017  2.80716389  0.09251819  2.12105404], Cost: 0.1456
Current parameters: [ 0.03444486  1.9311995  -0.57023919  3.26582665 -0.25413741 -0.0503371
  2.02217368  0.48533962  0.51400514  2.86413462  0.05552128  2.11948856], Cost: 0.5907
Current parameters: [-0.14092092  1.92904975 -0.56718284  2.85925566 -0.26327869 -0.06598946
  2.34538745  0.5328198   0.53561734  2.85856898  0.13732068  2.16632487], Cost: 1.6929
Current parameters: [-0.03844966  1.83579799 -0.6196061   2.97319119 -0.23420817 -0.04249166
  2.33403518  0.13016348  0.09671741  2.74417405 -0.07948102  1.99651019], Cost: 2.0283
Current parameters: [-0.12546425  1.78061373 -0.54096537  2.85234217 -0.28536105 -0.09724202
  2.21339803  0.55288901  0.35656753  2.76521193 -0.06077367  2.11447308], Cost: 0.2969
Current parameters: [-0.208677    1.93867083 -0.88072828  2.80295535 -0.35061993 -0.15293796
  1.99479938  0.54101074  0.32489426  2.70048008  0.19950835  1.91110887], Cost: 0.7951
Current parameters: [-0.13872209  1.95262151 -0.53730749  2.87015196 -0.25208054 -0.05184644
  2.17712923  0.48797007  0.21860216  2.89393261  0.15059504  2.19765318], Cost: 0.3984
Current parameters: [-0.13062219  1.86024207 -0.61358256  2.85327354 -0.2803006  -0.08968539
  2.20742867  0.39354065  0.40042557  2.9157033   0.06574164  2.09457206], Cost: 0.3682
Current parameters: [-0.17848612  1.82742668 -0.5488163   2.83031335 -0.3087014  -0.11035317
  2.08094956  0.31765486  0.42543643  2.75849061  0.09575054  2.25772   ], Cost: 0.2930
Current parameters: [-0.11078836  1.80608325 -0.64662789  2.87948245 -0.22768885 -0.03079655
  2.226413    0.42087415  0.38170403  2.81094326  0.12818622  2.15437705], Cost: 0.2422
Current parameters: [-0.18057543  1.92649474 -0.5581851   2.81186855 -0.14169164  0.08169779
  2.13415555  0.40050377  0.42766284  2.8070263   0.0339367   2.07001069], Cost: 0.3520
Current parameters: [-0.0715292   1.89197568 -0.58392771  2.96009035 -0.25984318 -0.06936381
  2.15636105  0.41816179  0.41012234  2.81759338  0.08384196  2.11378078], Cost: 0.1414
Current parameters: [-0.11336739  1.97386354 -0.56089317  3.01132303 -0.28239007 -0.07250739
  2.25121364  0.50850973  0.56812621  2.79060122  0.12812501  2.18831783], Cost: 1.3335
Current parameters: [-0.08313256  1.93158915 -0.57185875  2.95733847 -0.27212649 -0.08863184
  2.22564433  0.36325057  0.37809029  2.75794871  0.07868075  2.08514211], Cost: 0.2075
Current parameters: [ 7.89837607e-03  1.80178690e+00 -6.13375135e-01  3.04265545e+00
 -2.28198458e-01 -2.44788053e-02  2.07791993e+00  3.18349536e-01
  2.93630791e-01  2.82862297e+00 -2.91354885e-03  2.10897481e+00], Cost: 0.7208
Current parameters: [-0.09259671  1.90764039 -0.65242723  2.94462723 -0.28347707 -0.09235068
  2.10160563  0.44663418  0.39312668  2.77892254  0.11026815  2.06253562], Cost: 0.1878
Current parameters: [-0.07066624  1.88235303 -0.57110341  2.9609533  -0.30474758 -0.03234862
  2.157224    0.4176365   0.40961209  2.81726561  0.09498782  2.10213802], Cost: 0.1315
Current parameters: [-0.07489514  1.83342522 -0.50433525  2.96131837 -0.27331317 -0.06397101
  2.14625043  0.4178189   0.41068472  2.8155295   0.15482168  2.04680032], Cost: 0.1508
```



```
Current parameters: [-0.06620515  1.90534832 -0.58358092  2.96627422 -0.29674901 -0.02247518
  2.15016831  0.3858406   0.41254089  2.83740482  0.13375625  2.10798474], Cost: 0.1465
Current parameters: [-0.0893075   1.9200505  -0.53430433  2.95840199 -0.30674282 -0.03236538
  2.15057499  0.46485907  0.31859434  2.83662935  0.11558988  2.13581189], Cost: 0.1567
Current parameters: [-0.03996565  1.89216674 -0.58320771  2.96497977 -0.29017798 -0.0185271
  2.18170145  0.44075114  0.40431136  2.83221899  0.09526939  2.07186976], Cost: 0.1391
Current parameters: [ 0.01179481  1.89391712 -0.55037319  2.88238159 -0.31859715 -0.04708926
  2.13596095  0.41372032  0.41426402  2.79311605  0.10279676  2.12550079], Cost: 0.1401
Current parameters: [-0.08606847  1.90040055 -0.55645588  2.94314289 -0.2695129  -0.00368376
  2.13995149  0.41697616  0.41901584  2.81084064  0.0787013   2.09474526], Cost: 0.1286
Current parameters: [-0.11030987  1.89258054 -0.56283322  2.91154521 -0.33820322  0.05926981
  2.1464365   0.39969501  0.39206559  2.77538639  0.09732265  2.04390396], Cost: 0.1161
Current parameters: [-0.12758801  1.91466079 -0.55611809  2.90242115 -0.34926518  0.05453666
  2.16671554  0.42666463  0.42628989  2.76901774  0.11869737  2.05020684], Cost: 0.0827
Current parameters: [-0.11231277  1.91461966 -0.53221667  2.96000217 -0.37588193  0.10410569
  2.15354093  0.46730834  0.47809268  2.7151864   0.12809747  2.07926004], Cost: 0.1670
Current parameters: [-0.14513507  1.89183308 -0.55626163  2.90904694 -0.38684429  0.03598992
  2.16874257  0.4240868   0.43866289  2.78253447  0.08994921  2.06041221], Cost: 0.1036
Current parameters: [-0.13368736  1.97234665 -0.5294695   2.84396909 -0.34279976  0.06605539
  2.16278683  0.43424924  0.45184456  2.83898356  0.12368373  2.00211542], Cost: 0.2115
Current parameters: [-0.14001845  1.88279379 -0.57480751  2.87736084 -0.33122695  0.06789528
  2.18878465  0.43464099  0.42585162  2.7686533   0.13167671  2.07335048], Cost: 0.1128
Current parameters: [-0.12441346  1.91736635 -0.5396181   2.90473012 -0.34794597  0.0592372
  2.18737915  0.41318833  0.42491042  2.76873429  0.11403171  2.05524678], Cost: 0.0834
Current parameters: [-0.141228    1.93407803 -0.57294734  2.90233522 -0.34392799  0.02298662
  2.18098545  0.41836398  0.41964168  2.72926435  0.11365424  2.0395506 ], Cost: 0.0658
Current parameters: [-0.14214309  1.95053677 -0.58168855  2.90134993 -0.3495874   0.0304917
  2.18220615  0.42466443  0.40688281  2.7328091   0.10474938  2.05514752], Cost: 0.0543
Current parameters: [-0.15963565  1.94654609 -0.55032607  2.90608076 -0.35381058  0.01315223
  2.17891013  0.44692536  0.36976985  2.73077497  0.12345702  2.05983357], Cost: 0.0378
Current parameters: [-0.17790296  1.94819848 -0.55658259  2.92461558 -0.34656288  0.02039404
  2.1835337   0.44538815  0.36926733  2.73942591  0.12524533  2.05273064], Cost: 0.0535
Current parameters: [-1.58471857e-01  1.95754617e+00 -5.59371447e-01  2.91076422e+00
 -3.70723643e-01  1.12747781e-03  2.16963330e+00  4.14356545e-01
  3.69216658e-01  2.73639420e+00  1.62763081e-01  2.08276518e+00], Cost: 0.0723
Current parameters: [-0.15377634  1.94315981 -0.55766322  2.90498206 -0.37175843  0.02188668
  2.18969294  0.44902641  0.36122173  2.72882884  0.13082668  2.04481844], Cost: 0.0362
Current parameters: [-0.120695    1.94439221 -0.56888452  2.92554055 -0.36135337 -0.00736231
  2.21118042  0.47138134  0.36071235  2.74650708  0.13325989  2.04904506], Cost: 0.0311
Current parameters: [-0.12590131  1.95163358 -0.56952274  2.90785581 -0.35811122 -0.01590973
  2.21421897  0.46635843  0.35698255  2.76504783  0.13200308  2.03872134], Cost: 0.0376
```



```
Current parameters: [-0.10724707  1.97731112 -0.54906056  2.91701285 -0.3676258  -0.00489237
  2.21025147  0.50776853  0.36128797  2.72126413  0.12177855  2.04301658], Cost: 0.0192
Current parameters: [-0.08307775  1.97033309 -0.54987635  2.92072362 -0.35675904 -0.00612505
  2.19314285  0.49930818  0.31892583  2.70753548  0.09450366  2.03607889], Cost: 0.0947
Current parameters: [-0.10370732  1.97757028 -0.5541782   2.90743788 -0.34767962  0.00618564
  2.21555549  0.51100269  0.35624373  2.71578528  0.13681042  2.04536418], Cost: 0.0238
Current parameters: [-0.12830143  1.95764295 -0.5560159   2.89650206 -0.3833504  -0.01355651
  2.23631658  0.53417062  0.3742633   2.71307241  0.11458628  2.06793797], Cost: 0.0501
Current parameters: [-0.11060193  1.97342245 -0.56496293  2.91481227 -0.36827034 -0.01096723
  2.18989415  0.52037199  0.36376685  2.72035726  0.12718085  2.036255  ], Cost: 0.0284
Current parameters: [-0.10897685  1.96499518 -0.53165765  2.91899152 -0.35735121 -0.03181737
  2.22070175  0.49623981  0.38259449  2.69392876  0.13447269  2.01123885], Cost: 0.0357
Current parameters: [-0.09929378  1.96359989 -0.53043428  2.91086972 -0.36258731 -0.00311002
  2.199367    0.51367345  0.36752426  2.73100595  0.12200566  2.04800506], Cost: 0.0423
Current parameters: [-0.10451273  1.99415921 -0.55404439  2.93773703 -0.39247136  0.0260306
  2.22722677  0.51310014  0.37453306  2.73574593  0.14141576  2.02066281], Cost: 0.0297
Current parameters: [-0.10449471  1.99224692 -0.54731284  2.91641264 -0.37545794 -0.01477856
  2.20550563  0.49880441  0.36224921  2.71832287  0.13725373  2.05823107], Cost: 0.0122
Current parameters: [-0.09631872  1.99271013 -0.55288183  2.91105079 -0.37639935 -0.01252729
  2.20527825  0.49425639  0.37048874  2.71674384  0.13277709  2.05868067], Cost: 0.0123
Current parameters: [-0.11556963  2.00670198 -0.54879546  2.92744555 -0.36020804 -0.01481117
  2.20365681  0.49784368  0.37516068  2.72062939  0.12884305  2.06466297], Cost: 0.0266
Current parameters: [-0.09772604  1.99384385 -0.55004364  2.91800571 -0.37244644 -0.02314157
  2.2110633   0.50310624  0.36145591  2.72579649  0.13759353  2.05734899], Cost: 0.0185
Current parameters: [-0.11512038  2.00343662 -0.54449635  2.89543527 -0.37706619 -0.01670614
  2.20541387  0.49226355  0.35875415  2.72901035  0.13550115  2.04765453], Cost: 0.0096
Current parameters: [-0.1088193   2.00700097 -0.54293557  2.90004191 -0.37343733 -0.01528607
  2.19858574  0.48636657  0.3526543   2.72946576  0.13424735  2.04195325], Cost: 0.0073
Current parameters: [-0.1095381   2.01166204 -0.54813614  2.89677388 -0.38656689 -0.01369567
  2.20323373  0.47832042  0.33366854  2.71441671  0.12692262  2.04579391], Cost: 0.0219
Current parameters: [-0.10633814  2.01193964 -0.53663089  2.89851457 -0.3731798  -0.01857157
  2.19969348  0.49229704  0.35576525  2.72044628  0.13381845  2.03620515], Cost: 0.0077
Current parameters: [-0.11466108  1.99587185 -0.54282421  2.90350147 -0.37089561 -0.02593416
  2.20371036  0.46804722  0.35954183  2.72339311  0.14450007  2.03031443], Cost: 0.0146
Current parameters: [-0.11146528  2.00742589 -0.53911917  2.90575796 -0.3802927  -0.00794953
  2.1971021   0.48809231  0.35202581  2.72869305  0.14329634  2.04103445], Cost: 0.0065
Current parameters: [-1.02492370e-01  2.01925691e+00 -5.48436968e-01  2.90497162e+00
 -3.86160710e-01  9.77240714e-04  2.20756026e+00  4.86826977e-01
  3.62557130e-01  2.74102318e+00  1.41508927e-01  2.02771505e+00], Cost: 0.0093
Current parameters: [-0.10596209  2.00598591 -0.53914782  2.90409804 -0.37423873 -0.0077941
  2.19095301  0.48402793  0.3497683   2.73746258  0.13788731  2.04363485], Cost: 0.0092
```

```
Current parameters: [-0.10680756  2.00257226 -0.51608206  2.89585185 -0.39142506 -0.00305294
  2.19497048  0.49038806  0.36182496  2.7285302   0.15003683  2.04284181], Cost: 0.0123
Current parameters: [-0.11270939  2.00675277 -0.5459788   2.90300877 -0.3833621  -0.01037272
  2.18652831  0.49344999  0.35407217  2.727831    0.14682114  2.03724375], Cost: 0.0082
Current parameters: [-0.10916434  2.00640139 -0.54045834  2.90221904 -0.37704274 -0.00569924
  2.19869267  0.48883703  0.34997493  2.7275827   0.14732153  2.04088513], Cost: 0.0064
Current parameters: [-1.15740209e-01  2.01412861e+00 -5.39665750e-01  2.90445789e+00
 -3.69374193e-01 -6.98158908e-04  2.19665649e+00  4.88574187e-01
  3.54889188e-01  2.72479724e+00  1.49345799e-01  2.04427764e+00], Cost: 0.0107
Current parameters: [-1.11300219e-01  2.00279987e+00 -5.40266852e-01  2.90179124e+00
 -3.76071462e-01 -1.49678236e-03  2.19839490e+00  4.89666110e-01
  3.50076989e-01  2.72645247e+00  1.43767083e-01  2.03772593e+00], Cost: 0.0071
Current parameters: [-0.10913704  2.0043285  -0.53793416  2.90940907 -0.37484015 -0.00957979
  2.20279105  0.49815246  0.34510845  2.73195564  0.1499943   2.03799687], Cost: 0.0091
Current parameters: [-0.10511277  2.00388645 -0.54190096  2.90580743 -0.37645043 -0.00484531
  2.19852175  0.48971131  0.35345868  2.72538046  0.14723644  2.04299938], Cost: 0.0075
Current parameters: [-0.10708121  2.00958745 -0.5435163   2.90159562 -0.38453246 -0.00447878
  2.20051188  0.4866149   0.33995453  2.72147703  0.14431467  2.04359599], Cost: 0.0045
Current parameters: [-0.10855181  2.0057407  -0.54507354  2.9029719  -0.38539211 -0.00999477
  2.20295769  0.47538326  0.3381312   2.71823879  0.14821662  2.03807073], Cost: 0.0131
Current parameters: [-0.10848685  2.00643497 -0.53920636  2.90078096 -0.38412556 -0.00599563
  2.19778749  0.48700418  0.33847132  2.71893946  0.14522128  2.04681789], Cost: 0.0043
Current parameters: [-1.03769242e-01  2.00920582e+00 -5.31644220e-01  2.89490233e+00
 -3.92695379e-01 -1.21953085e-03  2.19910063e+00  4.90430007e-01
  3.41139974e-01  2.72110450e+00  1.46035645e-01  2.04734432e+00], Cost: 0.0027
Current parameters: [-1.06401826e-01  2.00677006e+00 -5.33473588e-01  2.89625026e+00
 -3.95077111e-01  1.73419190e-03  2.19932562e+00  4.89368716e-01
  3.40658345e-01  2.72448728e+00  1.48673992e-01  2.05031048e+00], Cost: 0.0027
Current parameters: [-0.10661513  2.00757967 -0.53569134  2.88724083 -0.39352979 -0.00568535
  2.20534865  0.49509233  0.34356679  2.7214328   0.1408309   2.05396383], Cost: 0.0030
Current parameters: [-1.05510150e-01  2.01332306e+00 -5.31276972e-01  2.89168276e+00
 -3.89567968e-01 -1.22442269e-03  2.19905603e+00  4.89162713e-01
  3.41466464e-01  2.72177440e+00  1.50181039e-01  2.04853756e+00], Cost: 0.0022
Current parameters: [-0.1090866   2.02141482 -0.52989501  2.89571014 -0.38929805  0.00621917
  2.19743644  0.49398444  0.3411837   2.71733391  0.14612389  2.05402692], Cost: 0.0031
Current parameters: [-9.93195183e-02  2.01441759e+00 -5.31006173e-01  2.88839687e+00
 -3.85994837e-01 -2.33445505e-04  2.19316480e+00  4.87162446e-01
  3.36415335e-01  2.73007896e+00  1.44703317e-01  2.05263711e+00], Cost: 0.0031
Current parameters: [-1.05897174e-01  2.01376509e+00 -5.34513548e-01  2.88985257e+00
 -3.91802872e-01 -2.14567305e-03  2.19399675e+00  4.92383674e-01
  3.41606773e-01  2.72122682e+00  1.51862651e-01  2.04715801e+00], Cost: 0.0021
```



```
Current parameters: [-0.10585241  2.01391118 -0.53389599  2.8907954  -0.39122111 -0.0028995
  2.19501223  0.49494365  0.33990685  2.72247462  0.15280627  2.04678845], Cost: 0.0024

   Return from subroutine COBYLA because the MAXFUN limit has been reached.

   NFVALS =  100   F = 2.094714E-03    MAXCV = 0.000000E+00
   X =-1.058972E-01   2.013765E+00  -5.345135E-01   2.889853E+00  -3.918029E-01
      -2.145673E-03   2.193997E+00   4.923837E-01   3.416068E-01   2.721227E+00
       1.518627E-01   2.047158E+00
Current parameters: [-1.05897174e-01  2.01376509e+00 -5.34513548e-01  2.88985257e+00
 -3.91802872e-01 -2.14567305e-03  2.19399675e+00  4.92383674e-01
  3.41606773e-01  2.72122682e+00  1.51862651e-01  2.04715801e+00], Cost: 0.0021
Simulating for QAOA depth p = 7
Current parameters: [3.39033654 1.62554484 0.02338994 1.40254014 1.30844308 0.82437948
 0.08956421 0.75044532 2.98960387 2.09761716 0.66181681 1.41439284
 3.03177198 0.66462473], Cost: 1.6774
Current parameters: [3.39033654 2.62554484 0.02338994 1.40254014 1.30844308 0.82437948
 0.08956421 0.75044532 2.98960387 2.09761716 0.66181681 1.41439284
 3.03177198 0.66462473], Cost: 1.3205
Current parameters: [3.39033654 2.62554484 1.02338994 1.40254014 1.30844308 0.82437948
 0.08956421 0.75044532 2.98960387 2.09761716 0.66181681 1.41439284
 3.03177198 0.66462473], Cost: 2.0863
Current parameters: [3.39033654 2.62554484 0.02338994 2.40254014 1.30844308 0.82437948
 0.08956421 0.75044532 2.98960387 2.09761716 0.66181681 1.41439284
 3.03177198 0.66462473], Cost: 1.3538
Current parameters: [3.39033654 2.62554484 0.02338994 1.40254014 2.30844308 0.82437948
 0.08956421 0.75044532 2.98960387 2.09761716 0.66181681 1.41439284
 3.03177198 0.66462473], Cost: 1.4320
Current parameters: [3.39033654 2.62554484 0.02338994 1.40254014 1.30844308 1.82437948
 0.08956421 0.75044532 2.98960387 2.09761716 0.66181681 1.41439284
 3.03177198 0.66462473], Cost: 1.3580
Current parameters: [3.39033654 2.62554484 0.02338994 1.40254014 1.30844308 0.82437948
 1.08956421 0.75044532 2.98960387 2.09761716 0.66181681 1.41439284
 3.03177198 0.66462473], Cost: 1.6462
Current parameters: [3.39033654 2.62554484 0.02338994 1.40254014 1.30844308 0.82437948
 0.08956421 1.75044532 2.98960387 2.09761716 0.66181681 1.41439284
 3.03177198 0.66462473], Cost: 0.9849
Current parameters: [3.39033654 2.62554484 0.02338994 1.40254014 1.30844308 0.82437948
 0.08956421 1.75044532 3.98960387 2.09761716 0.66181681 1.41439284
 3.03177198 0.66462473], Cost: 1.3076
Current parameters: [3.39033654 2.62554484 0.02338994 1.40254014 1.30844308 0.82437948
```



```
 0.08956421 1.75044532 2.98960387 3.09761716 0.66181681 1.41439284
 3.03177198 0.66462473], Cost: 1.2763
Current parameters: [3.39033654 2.62554484 0.02338994 1.40254014 1.30844308 0.82437948
 0.08956421 1.75044532 2.98960387 2.09761716 1.66181681 1.41439284
 3.03177198 0.66462473], Cost: 2.1264
Current parameters: [3.39033654 2.62554484 0.02338994 1.40254014 1.30844308 0.82437948
 0.08956421 1.75044532 2.98960387 2.09761716 0.66181681 2.41439284
 3.03177198 0.66462473], Cost: 2.5582
Current parameters: [3.39033654 2.62554484 0.02338994 1.40254014 1.30844308 0.82437948
 0.08956421 1.75044532 2.98960387 2.09761716 0.66181681 1.41439284
 4.03177198 0.66462473], Cost: 1.6981
Current parameters: [3.39033654 2.62554484 0.02338994 1.40254014 1.30844308 0.82437948
 0.08956421 1.75044532 2.98960387 2.09761716 0.66181681 1.41439284
 3.03177198 1.66462473], Cost: 1.4531
Current parameters: [ 3.55746241  2.77367103 -0.29446008  1.38871194  1.26217657  0.8088381
 -0.04561292  1.88973115  2.85567668  1.9766669   0.18803206  0.76137658
  2.73575691  0.47030643], Cost: 3.4387
Current parameters: [3.40402038 2.63767303 0.51732655 1.40140792 1.3046549  0.82310699
 0.07849625 1.76184969 2.97863826 2.08771406 0.62302453 1.36092555
 3.00753502 0.64871445], Cost: 1.8604
Current parameters: [3.22451474 2.81263608 0.02338994 1.40254014 1.30844308 0.82437948
 0.08956421 1.75044532 2.98960387 2.09761716 0.66181681 1.41439284
 3.03177198 0.66462473], Cost: 1.0616
Current parameters: [ 3.0233139   2.29099385 -0.00589856  1.40178797  1.30592648  0.82353413
  0.08221145  1.75802157  2.9823191   2.09103825  0.63604598  1.37887298
  3.01567067  0.65405508], Cost: 1.7853
Current parameters: [3.39033654 2.62554484 0.02338994 1.15383804 1.30844308 0.82437948
 0.11500565 1.75044532 2.98960387 2.09761716 0.66181681 1.41439284
 3.03177198 0.66462473], Cost: 0.8545
Current parameters: [3.40620462 2.63423682 0.00638598 1.19776612 1.30698203 0.8238887
 0.61161969 1.75484385 2.98537457 2.09379765 0.6468551  1.39377115
 3.02242408 0.65848833], Cost: 0.4148
Current parameters: [3.40620462 2.63423682 0.00638598 1.19776612 1.38658832 0.5869017
 0.61161969 1.75484385 2.98537457 2.09379765 0.6468551  1.39377115
 3.02242408 0.65848833], Cost: 0.5092
Current parameters: [3.41207533e+00 2.63745259e+00 9.50210771e-05 1.19572936e+00
 1.78015547e+00 9.84763803e-01 6.16570048e-01 1.75647117e+00
 2.98380985e+00 2.09238455e+00 6.41319713e-01 1.38614175e+00
 3.01896564e+00 6.56218045e-01], Cost: 1.8882
Current parameters: [ 3.42082251  2.64719288 -0.02141521  1.19655662  1.30293526  0.82252935
  0.59979623  1.75484385  2.98537457  2.09379765  0.6468551   1.14641297
```



```
  3.02242408  0.65848833], Cost: 1.6740
Current parameters: [ 3.50059874  2.6874022  -0.09834469  1.16615642  1.09073208  0.78088501
  0.68409312  1.77981422  2.96136486  2.07211438  0.56191767  1.79316503
  2.96935619  0.62365205], Cost: 0.7867
Current parameters: [3.40972028 2.6373528  0.13328837 1.19747523 1.30600876 0.82356177
 0.6087761  1.75484385 2.98537457 2.09379765 0.43196957 1.38003432
 3.02242408 0.65848833], Cost: 0.8216
Current parameters: [3.41062892 2.62751285 0.40327075 1.18909276 1.23939565 0.81153062
 0.65998833 1.76355986 2.97699388 2.08622902 0.92455686 1.48019777
 3.00390051 0.64632858], Cost: 1.1010
Current parameters: [3.40620462 2.63423682 0.00638598 1.19776612 1.30698203 0.8238887
 0.61161969 1.75484385 2.98537457 2.09379765 0.6468551  1.39377115
 3.15961701 0.44949527], Cost: 0.3308
Current parameters: [ 3.4033356   2.62763281 -0.00669046  1.1948343   1.28245955  0.81959788
  0.63375793  1.75816898  2.98217736  2.09091024  0.65433149  1.44454787
  3.57605927  0.71888488], Cost: 2.1715
Current parameters: [3.40620462 2.63423682 0.00638598 1.19776612 1.30698203 0.8238887
 0.61161969 1.58156767 2.80516534 2.09379765 0.6468551  1.39377115
 3.15961701 0.44949527], Cost: 0.2459
Current parameters: [ 3.40472971e+00  2.63084183e+00 -3.36367475e-04  1.19625893e+00
  1.29437549e+00  8.21682872e-01  6.23000535e-01  1.22095917e+00
  3.14950070e+00  2.09231329e+00  6.50698566e-01  1.41987449e+00
  3.14516683e+00  4.37962007e-01], Cost: 1.4716
Current parameters: [3.40337222e+00 2.63172642e+00 1.11655871e-03 1.19834421e+00
 1.30232142e+00 8.29489462e-01 6.17270839e-01 1.58152589e+00
 2.80520551e+00 2.34331854e+00 6.51057740e-01 1.40070289e+00
 3.15597785e+00 4.55232566e-01], Cost: 0.5515
Current parameters: [3.49543898 2.68954734 0.12708498 1.15895794 1.33786946 0.58909504
 0.52968985 1.67533074 2.6876771  2.04309259 0.53330193 1.43158846
 3.13126701 0.10335243], Cost: 2.2556
Current parameters: [ 3.22696873  2.4722439  -0.01907412  1.19530991  1.28555742  0.82175213
  0.62680305  1.59729788  2.78643866  2.07899521  0.64158447  1.41805373
  3.12927052  0.42878651], Cost: 0.4624
Current parameters: [3.47979982 2.55321447 0.00738042 1.25443557 1.30866813 0.82172901
 0.63151247 1.58184484 2.80489883 2.09355697 0.64436767 1.38990937
 3.16044608 0.4469483 ], Cost: 0.2994
Current parameters: [3.33197668 2.73051298 0.00578997 1.40033291 1.30517289 0.81547109
 0.69038005 1.58720602 2.79833927 2.08845579 0.64094484 1.39637313
 3.15392631 0.43435853], Cost: 0.5322
Current parameters: [3.41175385 2.63755061 0.00902985 1.15273524 1.31331398 0.81542345
 0.72554352 1.58288144 2.80390211 2.09265683 0.6363846  1.37854508
```

```
 3.16233509 0.43926854], Cost: 0.3246
Current parameters: [ 3.4584382   2.66513471 -0.12214098  1.17782694  1.21928244  0.87448143
  0.62937866  1.60680163  2.77616105  2.07038093  0.66610231  1.53741435
  3.07187012  0.47407815], Cost: 0.3120
Current parameters: [ 3.40542896  2.63537821 -0.06831912  1.1992575   1.38498081  0.83628446
  0.6033225   1.58007025  2.80660515  2.09509795  0.58762113  1.38800051
  3.16998074  0.44064459], Cost: 0.5866
Current parameters: [3.40682501 2.63087981 0.10221346 1.18928837 1.45012842 0.92138397
 0.62224017 1.59601595 2.78704901 2.07991092 0.7651973  1.46907874
 3.10991095 0.46524193], Cost: 0.3847
Current parameters: [3.40355693 2.62891513 0.02571021 1.18555255 1.27024934 0.80966609
 0.61334506 1.60515484 2.77454379 2.07079521 0.6759264  1.4694011
 3.35711042 0.56261337], Cost: 0.6566
Current parameters: [3.42810245 2.64690779 0.05249428 1.17887644 1.28665268 0.76974507
 0.58400382 1.60608783 2.77470115 2.07973171 0.64283927 1.41610345
 3.14343819 0.37274296], Cost: 0.4091
Current parameters: [ 3.40503835e+00  2.63307369e+00 -3.68683363e-04  1.19776160e+00
  1.32133715e+00  7.79675686e-01  6.13194692e-01  1.55928306e+00
  2.82659261e+00  2.09370562e+00  6.60576389e-01  1.41070655e+00
  3.14977261e+00  4.62411797e-01], Cost: 0.2456
Current parameters: [3.40725756 2.63348306 0.02182408 1.19080877 1.28560225 0.82617022
 0.60866292 1.48481332 2.89403712 2.08240178 0.65504455 1.42373165
 3.1586101  0.42895814], Cost: 0.4188
Current parameters: [ 3.40540662  2.63340009 -0.02801429  1.19773113  1.3212352   0.77964144
  0.61289682  1.55928306  2.82659261  2.09370562  0.70391939  1.39524614
  3.1673332   0.43566085], Cost: 0.2148
Current parameters: [ 3.41492418  2.63975235 -0.02555773  1.18501549  1.28963433  0.77353203
  0.59618865  1.55989426  2.81828759  2.07330964  0.7438382   1.31657471
  3.11531382  0.49077142], Cost: 0.4249
Current parameters: [ 3.41604984  2.61698003 -0.01172769  1.16535337  1.35252997  0.79999255
  0.60377072  1.56143959  2.82451905  2.09345707  0.72531621  1.40858114
  3.15922169  0.44040534], Cost: 0.1393
Current parameters: [3.39132792 2.6545858  0.02546353 1.23684509 1.39335085 0.82794756
 0.63380102 1.56944417 2.81334285 2.08278035 0.77207018 1.43479195
 3.13527784 0.43785324], Cost: 0.2428
Current parameters: [ 3.44466805  2.63788669 -0.03913706  1.16089744  1.33424     0.81179005
  0.60805742  1.56627035  2.81987414  2.09135738  0.7301192   1.43942791
  3.141673    0.44782709], Cost: 0.1580
Current parameters: [ 3.34307962  2.54661878 -0.03912654  1.15697519  1.32078905  0.80795162
  0.61168893  1.57496937  2.80917573  2.08271538  0.73663027  1.44723343
  3.12453327  0.43439955], Cost: 0.1071
```



```
Current parameters: [ 3.34389113  2.54605135 -0.03893873  1.15540802  1.3208987   0.80552213
  0.61161452  1.57818633  2.80608253  2.02716348  0.73648037  1.45427695
  3.14913948  0.4460541 ], Cost: 0.0249
Current parameters: [ 3.33653892  2.53295638 -0.02015172  1.1394502   1.29981451  0.79999788
  0.60726319  1.59279443  2.7852655   2.06179047  0.7698692   1.49359581
  3.23395832  0.49701559], Cost: 0.2772
Current parameters: [ 3.34384318  2.54612191 -0.04355667  1.15550021  1.32572025  0.80628838
  0.61110162  1.53510706  2.76146485  2.02724386  0.73281878  1.45392024
  3.14978012  0.44550699], Cost: 0.0343
Current parameters: [ 3.33243341  2.53613902 -0.10665214  1.14264737  1.40348702  0.81918306
  0.58597531  1.5959653   2.81297889  1.9968949   0.69753596  1.45268602
  3.15388137  0.43335829], Cost: 0.1367
Current parameters: [ 3.35318852  2.54717849 -0.01849098  1.14220184  1.31655897  0.78035392
  0.59759811  1.59277734  2.79049745  2.01149883  0.73705239  1.46519456
  3.14045246  0.4075686 ], Cost: 0.0790
Current parameters: [ 3.32945499  2.56326873 -0.03877275  1.13736253  1.32144002  0.80477889
  0.62351311  1.57831367  2.80596009  2.02705291  0.73552307  1.45293543
  3.14935008  0.44514337], Cost: 0.0291
Current parameters: [ 3.36581654  2.52180528 -0.03889003  1.15074466  1.32722328  0.80234787
  0.66295332  1.58026941  2.80514632  2.0215411   0.73303795  1.44700789
  3.1500439   0.44077943], Cost: 0.0439
Current parameters: [ 3.34291601  2.5452099  -0.03583522  1.15600446  1.31907286  0.81473938
  0.61321885  1.56474661  2.81937821  2.02809393  0.73051509  1.46772813
  3.15859372  0.43156678], Cost: 0.0501
Current parameters: [ 3.3464678   2.53897868 -0.03342083  1.14166492  1.30649988  0.82032682
  0.60166087  1.55951599  2.82277686  1.99933816  0.75375528  1.42619176
  3.1278381   0.45879014], Cost: 0.0153
Current parameters: [ 3.34585163  2.53844737 -0.03675222  1.14175844  1.31372309  0.79825215
  0.60248781  1.54838352  2.83347666  1.99927875  0.76066764  1.43469106
  3.122886    0.46524621], Cost: 0.0159
Current parameters: [ 3.33282794  2.55426265 -0.01626452  1.17567907  1.32714715  0.83542084
  0.61549739  1.56402236  2.81785896  1.98441634  0.77850554  1.43464388
  3.11241944  0.45969146], Cost: 0.0265
Current parameters: [ 3.34830915  2.53572382 -0.01754532  1.13555888  1.31246251  0.82418951
  0.60007371  1.55992362  2.82238491  1.99929118  0.7377366   1.4358689
  3.11817615  0.47206979], Cost: 0.0197
Current parameters: [ 3.33351189  2.54956719 -0.04009763  1.17129937  1.2787393   0.80343602
  0.60927263  1.55835303  2.82569758  1.98350617  0.72157292  1.4124742
  3.12267507  0.46765258], Cost: 0.0503
Current parameters: [ 3.34193666  2.5324165  -0.04763283  1.13860622  1.29445006  0.82571283
  0.60457638  1.56367996  2.818328    1.99660134  0.75748763  1.44367678
```



```
  3.11546927  0.4600784 ], Cost: 0.0141
Current parameters: [ 3.38718226  2.57076326 -0.06017607  1.13833068  1.29102222  0.83242665
  0.60502837  1.56308759  2.82025995  1.99341822  0.75813322  1.45193289
  3.11171562  0.46886978], Cost: 0.0276
Current parameters: [ 3.34346749  2.53296907 -0.03227407  1.13936806  1.27593421  0.82230969
  0.6091731   1.56228562  2.81505723  2.0032881   0.76867968  1.44847419
  3.12468548  0.46852375], Cost: 0.0044
Current parameters: [ 3.3369303   2.52130063 -0.04532631  1.12945779  1.28572746  0.82679959
  0.59741651  1.57619098  2.80816574  1.98928597  0.78577043  1.44881527
  3.15766539  0.5057757 ], Cost: 0.0826
Current parameters: [ 3.34679957  2.52883882 -0.03621494  1.15150256  1.2774433   0.8199455
  0.59990432  1.5736304   2.81456187  2.02350873  0.77082772  1.44356211
  3.11652438  0.4761839 ], Cost: 0.0069
Current parameters: [ 3.33485687  2.538831   -0.02446563  1.11650059  1.2658105   0.83384751
  0.62625103  1.53689556  2.82089551  2.03245002  0.78053235  1.43259531
  3.10044068  0.4552199 ], Cost: 0.0375
Current parameters: [ 3.34896531  2.53194707 -0.02195188  1.13441112  1.27214452  0.80906206
  0.59932885  1.57259025  2.80630074  1.99668032  0.77345053  1.44938646
  3.11681894  0.45384716], Cost: 0.0134
Current parameters: [ 3.33760114  2.53913454 -0.03173216  1.13963581  1.27405027  0.8247639
  0.59784258  1.55888742  2.81728927  2.00443234  0.76859443  1.4520701
  3.12584826  0.46739845], Cost: 0.0072
Current parameters: [ 3.35287192  2.52051461 -0.02829316  1.14946488  1.27396014  0.82751173
  0.61060167  1.56021789  2.82937814  2.00693016  0.76602633  1.45994922
  3.13205697  0.45561914], Cost: 0.0114
Current parameters: [ 3.33948723  2.53744908 -0.02893245  1.14044911  1.27729737  0.82450531
  0.61263333  1.57057288  2.82358099  2.00036479  0.77305147  1.44848302
  3.12185955  0.46903666], Cost: 0.0040
Current parameters: [ 3.33666403  2.54042985 -0.02228702  1.15883081  1.28720936  0.83060882
  0.61721545  1.56262368  2.81016052  1.99586659  0.78294158  1.45358766
  3.11615851  0.46791592], Cost: 0.0155
Current parameters: [ 3.33700742  2.54163725 -0.02954846  1.13715925  1.28263941  0.82265303
  0.61655237  1.57457225  2.81984951  2.00740769  0.77194091  1.45511692
  3.12677827  0.46543621], Cost: 0.0074
Current parameters: [ 3.33165481  2.54442288 -0.0308951   1.15297791  1.26312289  0.81367066
  0.621231    1.57204675  2.823891    1.99799467  0.75586958  1.44526064
  3.12165323  0.46910106], Cost: 0.0067
Current parameters: [ 3.3513298   2.54520982 -0.03206834  1.13959001  1.27561036  0.82524526
  0.61547258  1.56849056  2.82146292  1.99939778  0.77257071  1.4497388
  3.12208905  0.47220212], Cost: 0.0075
Current parameters: [ 3.33897502  2.54974718 -0.0275662   1.14605589  1.27639136  0.82861353
```



```
  0.59905614  1.58144541  2.84136206  2.00181136  0.77564325  1.45229781
  3.11330091  0.46137343], Cost: 0.0100
Current parameters: [ 3.33642968  2.53267579 -0.03140271  1.13856607  1.27498035  0.82408376
  0.61060326  1.5754284   2.82569529  1.99599569  0.77622839  1.44649746
  3.1298082   0.4773035 ], Cost: 0.0060
Current parameters: [ 3.33885246  2.53831583 -0.03259332  1.14108729  1.27567202  0.82392843
  0.61370646  1.56952427  2.82371393  2.00131092  0.77711989  1.44554277
  3.123359    0.46550287], Cost: 0.0030
Current parameters: [ 3.33540952  2.53621499 -0.02966743  1.13358411  1.27126127  0.82641258
  0.61819861  1.56494252  2.82709003  2.00693913  0.77822946  1.44034702
  3.11855016  0.46684088], Cost: 0.0026
Current parameters: [ 3.33545197  2.53609778 -0.02965862  1.13340786  1.2696165   0.83207735
  0.61807055  1.56775592  2.82429617  2.00669072  0.77630054  1.4385529
  3.11924794  0.46511806], Cost: 0.0025
Current parameters: [ 3.33307168  2.53196511 -0.04081197  1.13055557  1.27076557  0.83332585
  0.61947014  1.56792639  2.82411074  2.00331087  0.77434036  1.44240899
  3.11194064  0.46446249], Cost: 0.0060
Current parameters: [ 3.33483221  2.53714459 -0.03141656  1.13457703  1.27413641  0.83406304
  0.61858451  1.56580109  2.82691965  2.00726724  0.77366814  1.43563886
  3.12092468  0.46620998], Cost: 0.0044
Current parameters: [ 3.33810548  2.53368568 -0.02595184  1.12848658  1.27158935  0.82517563
  0.61560011  1.57006588  2.821326    2.00283043  0.77528219  1.43041158
  3.11792453  0.45898911], Cost: 0.0034
Current parameters: [ 3.334232    2.53742419 -0.02817564  1.13118647  1.26910647  0.83332792
  0.61956929  1.56540822  2.82612051  2.00195871  0.77604706  1.44063191
  3.12139795  0.46259962], Cost: 0.0020
Current parameters: [ 3.34035979  2.52756291 -0.02661412  1.13451877  1.26601456  0.83380478
  0.62166957  1.56422453  2.83070143  2.00509697  0.77477097  1.44325561
  3.12502965  0.45733951], Cost: 0.0035
Current parameters: [ 3.3336605   2.53496462 -0.02747725  1.13256871  1.27038903  0.83323558
  0.62289806  1.56230214  2.82109515  2.00086108  0.77677299  1.44020652
  3.12220801  0.46403333], Cost: 0.0016
Current parameters: [ 3.3297068   2.53688143 -0.0205313   1.13925108  1.2686176   0.83603695
  0.62196611  1.56255872  2.82099194  1.99867721  0.78608789  1.4399406
  3.11750177  0.46153379], Cost: 0.0021
Current parameters: [ 3.33237292  2.53615749 -0.02734642  1.13519997  1.26826295  0.83093377
  0.62762498  1.56497502  2.82229424  1.99975533  0.77411483  1.43901093
  3.12150725  0.46427899], Cost: 0.0018
Current parameters: [ 3.32858439  2.53687594 -0.02752919  1.13511585  1.26201875  0.83015882
  0.61658355  1.55578945  2.81739134  2.00232759  0.77182285  1.44012642
  3.12432237  0.4627826 ], Cost: 0.0020
```

```
Current parameters: [ 3.33770372  2.53660124 -0.02759018  1.13278466  1.26661388  0.83515136
  0.62322339  1.56439921  2.81802581  2.00302935  0.77771448  1.44031238
  3.12202473  0.46614774], Cost: 0.0015
Current parameters: [ 3.34478593  2.54323412 -0.0279054   1.13020047  1.26560209  0.83229802
  0.62604472  1.55609971  2.82046863  1.99642915  0.7801661   1.43909595
  3.1222268   0.46652551], Cost: 0.0023
Current parameters: [ 3.33645662  2.53446948 -0.02769167  1.1299074   1.27176777  0.83470532
  0.62423382  1.56182531  2.81623393  2.00327378  0.77885984  1.44171985
  3.12425011  0.46529721], Cost: 0.0023
Current parameters: [ 3.33545165  2.54502913 -0.02483704  1.13066927  1.26207305  0.83215574
  0.62715191  1.56942929  2.81606703  2.00905667  0.77966265  1.4443652
  3.12524696  0.46228924], Cost: 0.0026
Current parameters: [ 3.33602417  2.53491121 -0.02786972  1.13150442  1.26413276  0.83454599
  0.62249388  1.5662774   2.81907365  2.00069885  0.78033859  1.43898561
  3.12584663  0.46979142], Cost: 0.0016
Current parameters: [ 3.33689539  2.53599685 -0.02653097  1.13084389  1.2656503   0.83582631
  0.62425635  1.56334157  2.81886184  2.00435886  0.77763254  1.43926821
  3.12068424  0.46679693], Cost: 0.0014
Current parameters: [ 3.33755945  2.5359558  -0.02171487  1.13026827  1.26600043  0.83573542
  0.62304063  1.5642984   2.81850823  2.00256748  0.77459219  1.44234859
  3.11952964  0.47013495], Cost: 0.0018
Current parameters: [ 3.33604014  2.53639465 -0.02798108  1.13161469  1.26539392  0.83714965
  0.62503482  1.56242217  2.81989083  2.00465956  0.77784217  1.44163022
  3.12064026  0.46807351], Cost: 0.0023
Current parameters: [ 3.33653196  2.53406977 -0.02929715  1.12848736  1.26329564  0.83496841
  0.62393239  1.56519159  2.81638939  2.00183744  0.77800493  1.43947646
  3.11628596  0.46532076], Cost: 0.0014
Current parameters: [ 3.33859673  2.53457053 -0.02746989  1.13006037  1.26470916  0.83512147
  0.62484511  1.5634287   2.81952211  2.00510912  0.77567593  1.43993479
  3.12199217  0.4664572 ], Cost: 0.0014

   Return from subroutine COBYLA because the MAXFUN limit has been reached.

   NFVALS =  100   F = 1.396584E-03    MAXCV = 0.000000E+00
   X = 3.336895E+00   2.535997E+00  -2.653097E-02   1.130844E+00   1.265650E+00
       8.358263E-01   6.242564E-01   1.563342E+00   2.818862E+00   2.004359E+00
       7.776325E-01   1.439268E+00   3.120684E+00   4.667969E-01
Current parameters: [ 3.33689539  2.53599685 -0.02653097  1.13084389  1.2656503   0.83582631
  0.62425635  1.56334157  2.81886184  2.00435886  0.77763254  1.43926821
  3.12068424  0.46679693], Cost: 0.0014
Simulating for QAOA depth p = 8
```



```
Current parameters: [3.13547982 2.82267308 0.46267539 2.730079   0.7925801  1.71231803
 2.67363144 1.77088424 1.08684772 1.07293491 2.16723278 2.26549304
 1.04309708 0.59342085 0.43223634 3.10029603], Cost: 3.1412
Current parameters: [2.13547982 3.82267308 0.46267539 2.730079   0.7925801  1.71231803
 2.67363144 1.77088424 1.08684772 1.07293491 2.16723278 2.26549304
 1.04309708 0.59342085 0.43223634 3.10029603], Cost: 1.7889
Current parameters: [2.13547982 3.82267308 1.46267539 2.730079   0.7925801  1.71231803
 2.67363144 1.77088424 1.08684772 1.07293491 2.16723278 2.26549304
 1.04309708 0.59342085 0.43223634 3.10029603], Cost: 1.3440
Current parameters: [2.13547982 3.82267308 1.46267539 3.730079   0.7925801  1.71231803
 2.67363144 1.77088424 1.08684772 1.07293491 2.16723278 2.26549304
 1.04309708 0.59342085 0.43223634 3.10029603], Cost: 1.4122
Current parameters: [2.13547982 3.82267308 1.46267539 2.730079   1.7925801  1.71231803
 2.67363144 1.77088424 1.08684772 1.07293491 2.16723278 2.26549304
 1.04309708 0.59342085 0.43223634 3.10029603], Cost: 1.7319
Current parameters: [2.13547982 3.82267308 1.46267539 2.730079   0.7925801  2.71231803
 2.67363144 1.77088424 1.08684772 1.07293491 2.16723278 2.26549304
 1.04309708 0.59342085 0.43223634 3.10029603], Cost: 1.5243
Current parameters: [2.13547982 3.82267308 1.46267539 2.730079   0.7925801  1.71231803
 3.67363144 1.77088424 1.08684772 1.07293491 2.16723278 2.26549304
 1.04309708 0.59342085 0.43223634 3.10029603], Cost: 3.2207
Current parameters: [2.13547982 3.82267308 1.46267539 2.730079   0.7925801  1.71231803
 2.67363144 2.77088424 1.08684772 1.07293491 2.16723278 2.26549304
 1.04309708 0.59342085 0.43223634 3.10029603], Cost: 1.3716
Current parameters: [2.13547982 3.82267308 1.46267539 2.730079   0.7925801  1.71231803
 2.67363144 1.77088424 2.08684772 1.07293491 2.16723278 2.26549304
 1.04309708 0.59342085 0.43223634 3.10029603], Cost: 2.4488
Current parameters: [2.13547982 3.82267308 1.46267539 2.730079   0.7925801  1.71231803
 2.67363144 1.77088424 1.08684772 2.07293491 2.16723278 2.26549304
 1.04309708 0.59342085 0.43223634 3.10029603], Cost: 2.3753
Current parameters: [2.13547982 3.82267308 1.46267539 2.730079   0.7925801  1.71231803
 2.67363144 1.77088424 1.08684772 1.07293491 3.16723278 2.26549304
 1.04309708 0.59342085 0.43223634 3.10029603], Cost: 2.4830
Current parameters: [2.13547982 3.82267308 1.46267539 2.730079   0.7925801  1.71231803
 2.67363144 1.77088424 1.08684772 1.07293491 2.16723278 3.26549304
 1.04309708 0.59342085 0.43223634 3.10029603], Cost: 2.2890
Current parameters: [2.13547982 3.82267308 1.46267539 2.730079   0.7925801  1.71231803
 2.67363144 1.77088424 1.08684772 1.07293491 2.16723278 2.26549304
 2.04309708 0.59342085 0.43223634 3.10029603], Cost: 2.2716
Current parameters: [2.13547982 3.82267308 1.46267539 2.730079   0.7925801  1.71231803
 2.67363144 1.77088424 1.08684772 1.07293491 2.16723278 2.26549304
```



```
 1.04309708 1.59342085 0.43223634 3.10029603], Cost: 1.7739
Current parameters: [2.13547982 3.82267308 1.46267539 2.730079   0.7925801  1.71231803
 2.67363144 1.77088424 1.08684772 1.07293491 2.16723278 2.26549304
 1.04309708 0.59342085 1.43223634 3.10029603], Cost: 3.0523
Current parameters: [2.13547982 3.82267308 1.46267539 2.730079   0.7925801  1.71231803
 2.67363144 1.77088424 1.08684772 1.07293491 2.16723278 2.26549304
 1.04309708 0.59342085 0.43223634 4.10029603], Cost: 1.0829
Current parameters: [ 1.88898919  3.94517465  1.58406484  2.71148515  0.68675208  1.66313624
  2.16156913  1.76335971  0.78540623  0.79153558  1.85645175  2.00764144
  0.78999504  0.47612186 -0.03386467  4.17154953], Cost: 2.8993
Current parameters: [2.08728639 3.84662438 1.48640925 2.72644356 0.77188878 1.70270209
 3.14458378 1.76941305 1.02791039 1.01791619 2.10646939 2.21507833
 0.993611   0.57048675 0.34110505 4.11422739], Cost: 1.8980
Current parameters: [2.24674249 4.04654942 1.46267539 2.730079   0.7925801  1.71231803
 2.67363144 1.77088424 1.08684772 1.07293491 2.16723278 2.26549304
 1.04309708 0.59342085 0.43223634 4.10029603], Cost: 1.2204
Current parameters: [2.56860354 3.58955907 1.47560738 2.72809814 0.78130591 1.70707855
 2.65021872 1.77008263 1.05473424 1.04295659 2.13412433 2.23802332
 1.01613335 0.58092463 0.38258118 4.10788688], Cost: 0.8539
Current parameters: [2.56860354 3.58955907 1.47560738 2.72809814 0.78130591 1.70707855
 2.65021872 1.77008263 1.05473424 1.04295659 1.92612144 2.23802332
 1.01613335 0.58092463 0.52127075 4.10788688], Cost: 0.3809
Current parameters: [2.60697345 3.52420811 1.50912075 2.72296472 0.75208876 1.69350037
 2.58954442 1.76800524 0.97151184 0.96526746 2.05170719 2.16683528
 0.94625658 0.54854056 0.96653949 4.12755865], Cost: 1.2971
Current parameters: [2.56860354 3.58955907 1.47560738 2.72809814 0.78130591 1.70707855
 2.65021872 1.77008263 1.22533128 0.86020908 1.92612144 2.23802332
 1.01613335 0.58092463 0.52127075 4.10788688], Cost: 0.3644
Current parameters: [2.59784235 3.53976003 1.50114539 2.72418635 0.75904173 1.69673164
 2.60398342 1.76849961 1.57035669 1.17711714 1.7950904  2.18377629
 0.96288553 0.55624717 0.52053222 4.12287725], Cost: 1.0302
Current parameters: [2.56860354 3.58955907 1.47560738 2.72809814 0.78130591 1.70707855
 2.65021872 1.77008263 1.22533128 0.86020908 1.92612144 2.41314923
 0.83772114 0.58092463 0.52127075 4.10788688], Cost: 0.8812
Current parameters: [2.59494034 3.54470268 1.49861069 2.7245746  0.76125149 1.69775859
 2.60857236 1.76865673 1.19431141 0.82658812 1.80809549 2.4951461
 1.41827203 0.55869646 0.52060553 4.12138943], Cost: 1.8681
Current parameters: [2.56860354 3.58955907 1.30188565 2.72809814 0.78130591 1.70707855
 2.65021872 1.77008263 1.22533128 0.86020908 1.92612144 2.23802332
 1.01613335 0.40114487 0.52127075 4.10788688], Cost: 0.9325
Current parameters: [2.57866335 3.57242539 1.15449638 2.72675226 0.77364577 1.70351863
```



```
 2.63431116 1.76953798 1.21348269 0.84736696 1.88103935 2.1688258
 1.00541086 0.95361595 0.52101666 4.11304442], Cost: 1.5104
Current parameters: [2.56860354 3.58955907 1.47560738 2.72809814 0.88666719 1.48036507
 2.65021872 1.77008263 1.22533128 0.86020908 1.92612144 2.23802332
 1.01613335 0.58092463 0.52127075 4.10788688], Cost: 0.3654
Current parameters: [2.57501012 3.57864751 1.51977181 2.72724102 1.22999861 1.91565604
 2.64008801 1.76973577 1.21778552 0.8520306  1.89741097 2.19395498
 1.00930475 0.57798827 0.52110893 4.11117145], Cost: 0.4045
Current parameters: [2.56631858 3.59069466 1.47673265 2.47826912 0.78032489 1.70662263
 2.64547192 1.77008263 1.22253693 0.85760052 1.92324051 2.23563305
 1.0137871  0.57983727 0.51695001 4.10788688], Cost: 0.3326
Current parameters: [2.49839451 3.60091144 1.65433071 2.49047539 0.72608011 1.68158119
 2.43296203 1.76902601 1.09261716 0.73286306 1.72553443 2.00991908
 0.90319991 0.52928168 0.35111227 4.11789253], Cost: 1.7148
Current parameters: [2.56591285 3.5908963  1.47693246 2.47823852 0.7801507  1.70654168
 2.64943677 1.52011804 1.22204075 0.85713733 1.92272895 2.23520862
 1.01337049 0.57964419 0.5161828  4.10788688], Cost: 0.0130
Current parameters: [2.57340491 3.57089636 1.57291097 2.46890928 0.7660246  1.70009291
 3.12174804 1.49427354 1.19880724 0.83305914 1.85468921 2.13610968
 0.99282457 0.5706032  0.50449253 4.11480511], Cost: 0.6045
Current parameters: [2.6136214  3.6868927  1.47693246 2.47823852 0.7801507  1.70654168
 2.64943677 1.52011804 1.22204075 0.85713733 1.92272895 2.23520862
 1.01337049 0.57964419 0.5161828  4.33373769], Cost: 0.5197
Current parameters: [2.78486164 3.97347346 1.50313152 2.47618812 0.78271827 1.70777733
 2.64608409 1.50753389 1.23556608 0.86889046 1.92056732 2.21783492
 1.02434761 0.58490718 0.54594969 3.87730939], Cost: 0.8299
Current parameters: [2.35571469 3.70308752 1.49567527 2.50284543 0.76427804 1.72537242
 2.66001711 1.52046484 1.25447095 0.854899   1.94763066 2.22824326
 1.03983578 0.59458198 0.55352971 4.10460284], Cost: 0.0934
Current parameters: [2.45233442 3.5663149  1.33067308 2.24792531 0.99544246 1.55892488
 2.65325923 1.47514552 1.12361549 1.06953462 1.84350099 2.37417574
 0.93658067 0.51942122 0.51714323 4.06352661], Cost: 0.2559
Current parameters: [2.58507285 3.44646815 1.56645231 2.43316133 0.79711511 1.67762246
 2.59055371 1.47117358 1.18424486 0.86442309 2.11035354 2.14284938
 0.98128561 0.56166672 0.92186358 4.0834654 ], Cost: 0.9580
Current parameters: [2.56390155 3.53962582 1.53542378 2.4816979  0.77633989 1.71322868
 2.63936907 1.4966864  1.21715332 0.84056352 1.87507302 2.36450727
 1.20282331 0.59029604 0.50581234 4.08990731], Cost: 0.8469
Current parameters: [2.56762616 3.58814962 1.4881147  2.49356496 0.88200129 1.77101923
 2.64724742 1.52003297 1.22917924 0.84253239 1.92072281 2.21784666
 1.01811397 0.59045868 0.51317404 4.10869242], Cost: 0.0659
```

```
Current parameters: [2.55950982 3.56663987 1.33144021 2.48591339 0.75723342 1.70658747
 2.65119166 1.50531119 1.21552291 0.83680569 1.89926708 2.18626908
 1.01226653 0.77010215 0.52033441 4.0936701 ], Cost: 0.4977
Current parameters: [2.57841874 3.57868346 1.47876561 2.48461082 0.77607438 1.71426608
 2.65722429 1.51984883 1.31704371 0.93389084 1.92531169 2.23138367
 1.02183889 0.59000273 0.52005631 4.11043614], Cost: 0.1226
Current parameters: [2.52130414 3.5567246  1.56567906 2.4725948  0.75631558 1.6841035
 2.55639518 1.4940357  1.21532856 0.86326974 1.82058325 2.111254
 0.93511159 0.54268078 0.43124181 4.08693301], Cost: 0.1246
Current parameters: [2.56195181 3.57829348 1.49950868 2.47336306 0.77741088 1.70195465
 2.76506657 1.5154387  1.21220934 0.85162681 1.90609679 2.20880794
 0.99837989 0.57016338 0.50909511 4.10233319], Cost: 0.0872
Current parameters: [2.57777427 3.58095302 1.49961837 2.49879656 0.75845555 1.71945156
 2.64548078 1.51998497 1.2293536  0.83544007 1.9399094  2.21327926
 1.02106208 0.5867175  0.54194956 4.10960761], Cost: 0.0202
Current parameters: [2.55323423 3.56387344 1.47935427 2.43660338 0.80989413 1.67711989
 2.63543857 1.50625615 1.19487773 0.88453932 1.95290514 2.22579665
 0.97951492 0.55586872 0.59723146 4.09850558], Cost: 0.1784
Current parameters: [2.54636977 3.55668209 1.47547421 2.47758379 0.7802264  1.70577522
 2.64973221 1.52013671 1.22038499 0.85658616 1.95871957 2.23600283
 1.0131314  0.58004239 0.48967516 4.12655767], Cost: 0.0279
Current parameters: [2.60095235 3.65250022 1.49254546 2.4680054  0.78155125 1.69682909
 2.64688834 1.50890898 1.21404924 0.86179024 1.99186771 2.21752302
 1.00820487 0.57916242 0.47166387 4.05461436], Cost: 0.1827
Current parameters: [2.56591894 3.59089327 1.47739256 2.47890449 0.76195713 1.74569692
 2.64944943 1.52011804 1.19122132 0.89016674 1.92273663 2.23474815
 1.01385235 0.58012634 0.51619432 4.10788688], Cost: 0.0173
Current parameters: [2.56360017 3.58799097 1.45163161 2.42330963 0.74957112 1.76704267
 2.64732279 1.51329872 1.26205163 0.80805028 1.92057606 2.25848074
 0.97527331 0.54514354 0.51439832 4.0975945 ], Cost: 0.1030
Current parameters: [2.56594883 3.59087842 1.50616545 2.48217241 0.78016614 1.70654886
 2.64951152 1.52011804 1.22208475 0.8571784  1.92277432 2.26749098
 0.98055762 0.60993205 0.51625083 4.10788688], Cost: 0.0415
Current parameters: [2.56427104 3.58605646 1.43888748 2.57481718 0.77913996 1.7031042
 2.64977882 1.51060595 1.21828229 0.85636198 1.92172318 2.2575201
 0.97388516 0.53023177 0.51668909 4.0935303 ], Cost: 0.0629
Current parameters: [2.55440818 3.58210223 1.49762224 2.47858723 0.77419832 1.70089369
 2.62540251 1.51473532 1.2210049  0.85870569 1.89368078 2.20443614
 0.99735249 0.57137397 0.49459698 4.10484986], Cost: 0.0264
Current parameters: [2.59140645 3.5772898  1.47465428 2.47525648 0.78208017 1.70426139
 2.64803329 1.52769707 1.21812453 0.85742279 1.91972585 2.23606593
```



```
 1.01017494 0.57783925 0.51167882 4.10828499], Cost: 0.0170
Current parameters: [2.55126958 3.59646978 1.4780895  2.48033286 0.77861573 1.70638193
 2.6485609  1.57998437 1.2223978  0.85641225 1.92368356 2.23191903
 1.01094578 0.57639304 0.51839021 4.10203865], Cost: 0.0563
Current parameters: [2.57083233 3.60368002 1.47335905 2.47802717 0.78038351 1.70613315
 2.65005184 1.52154955 1.22233933 0.85814987 1.9256404  2.22730936
 1.00179622 0.57899344 0.51681636 4.13169228], Cost: 0.0461
Current parameters: [2.56561317 3.59009893 1.49574274 2.48213173 0.7760879  1.70266527
 2.64367648 1.51402943 1.21269821 0.8487032  1.9046879  2.26235824
 1.05427944 0.56381448 0.5119364  4.12436726], Cost: 0.0100
Current parameters: [2.55834216 3.57688943 1.48273865 2.48427719 0.77417368 1.70410315
 2.64449179 1.51510166 1.21253852 0.84573766 1.88958638 2.26243653
 1.0548939  0.57667502 0.52108928 4.13339612], Cost: 0.0421
Current parameters: [2.57592052 3.61012624 1.48583645 2.48476712 0.76837084 1.69366448
 2.63611708 1.50091959 1.19866744 0.83903142 1.91682128 2.25750359
 1.0765162  0.58519808 0.4862995  4.09514261], Cost: 0.0583
Current parameters: [2.56144076 3.5889544  1.49578732 2.4754439  0.78128597 1.69753617
 2.64013757 1.51224663 1.18992652 0.83668779 1.90821736 2.26176459
 1.04812479 0.55863824 0.52196751 4.12261839], Cost: 0.0241
Current parameters: [2.5671765  3.58041596 1.50783624 2.4702249  0.78390039 1.68728372
 2.63430942 1.50099087 1.2298407  0.88525197 1.91663137 2.26408482
 1.06446864 0.54716403 0.5364194  4.10984599], Cost: 0.0543
Current parameters: [2.5659609  3.59192395 1.48749307 2.47446902 0.77170976 1.71304806
 2.62128467 1.51385241 1.22082212 0.84205912 1.90766553 2.27102162
 1.05117296 0.5602189  0.51296509 4.12387437], Cost: 0.0156
Current parameters: [2.56519061 3.58705314 1.50052354 2.46028105 0.76149792 1.72171946
 2.67938017 1.50473199 1.22640987 0.83031811 1.89785888 2.26662467
 1.04770751 0.54008853 0.50294437 4.11089379], Cost: 0.0238
Current parameters: [2.56626785 3.59067151 1.50405026 2.46768953 0.79397619 1.71434834
 2.64324457 1.51569029 1.21463103 0.84631814 1.90429545 2.25516238
 1.06235235 0.57407873 0.51139625 4.12693761], Cost: 0.0225
Current parameters: [2.56664035 3.58838178 1.49114477 2.52314474 0.80971699 1.72305545
 2.64398401 1.50681517 1.21519903 0.84409732 1.90317201 2.26765924
 1.04880886 0.54437357 0.50677398 4.11398317], Cost: 0.0400
Current parameters: [2.55192971 3.59624891 1.49113327 2.48175588 0.77860864 1.70028651
 2.646158   1.53616353 1.21661522 0.84768875 1.90463033 2.26296973
 1.05823251 0.56221025 0.51606048 4.11132625], Cost: 0.0137
Current parameters: [2.5858336  3.58335826 1.52290949 2.48889251 0.75800767 1.70619482
 2.63758011 1.55024337 1.2032342  0.85280826 1.90762333 2.2640449
 1.05335387 0.55047632 0.49446009 4.14381397], Cost: 0.0062
Current parameters: [2.58630566 3.60510221 1.51665181 2.4880713  0.75864214 1.7022316
```



```
 2.6423014  1.55088046 1.20786307 0.85425224 1.91125743 2.25852998
 1.04906812 0.54430525 0.49561267 4.16113421], Cost: 0.0165
Current parameters: [2.59190657 3.58989209 1.52692863 2.48964442 0.75730189 1.70691615
 2.63688194 1.55054141 1.20281175 0.85302459 1.89881837 2.26660094
 1.05057268 0.55292333 0.50017325 4.13986349], Cost: 0.0047
Current parameters: [2.58311511 3.59197978 1.53814366 2.4907289  0.75862934 1.70358674
 2.64017129 1.5496321  1.20646881 0.85319235 1.90711375 2.28354186
 1.03645464 0.56521497 0.49562644 4.14164666], Cost: 0.0078
Current parameters: [2.59042152 3.5900999  1.52394961 2.48488209 0.76131126 1.70408283
 2.6353054  1.55061105 1.19982114 0.85864192 1.89160519 2.26936672
 1.04799214 0.55006924 0.49188536 4.13959412], Cost: 0.0052
Current parameters: [2.57951765 3.59069422 1.53893281 2.49328663 0.75260241 1.70340556
 2.62739146 1.54615835 1.20858225 0.84889476 1.8915918  2.24863801
 1.04259408 0.55141397 0.4953132  4.13729889], Cost: 0.0151
Current parameters: [2.5966099  3.58742756 1.52713548 2.48913968 0.7615731  1.69735218
 2.63632547 1.5515924  1.20862338 0.84510771 1.89748417 2.26803725
 1.05181676 0.55194918 0.49903894 4.1405286 ], Cost: 0.0053
Current parameters: [2.60047173 3.59966668 1.52402529 2.49274105 0.74762582 1.7027445
 2.63612593 1.5470356  1.19659174 0.85109712 1.90491172 2.26678654
 1.06118782 0.5628707  0.48511716 4.12770354], Cost: 0.0121
Current parameters: [2.58615817 3.59230402 1.53168166 2.49086523 0.75617342 1.70619315
 2.63569465 1.54723463 1.2018924  0.85186246 1.89562096 2.27241759
 1.06047866 0.54958486 0.5009792  4.1431621 ], Cost: 0.0035
Current parameters: [2.59222638 3.58779079 1.5390206  2.48577663 0.75437906 1.69704448
 2.63350791 1.54352239 1.20844598 0.87164347 1.90368697 2.27492801
 1.06408828 0.54112071 0.51043097 4.13577776], Cost: 0.0172
Current parameters: [2.58675111 3.59322012 1.53073396 2.50096714 0.76411585 1.70525566
 2.62859811 1.54814288 1.19980095 0.85532172 1.8973134  2.27213747
 1.06127019 0.55147398 0.50155477 4.14418486], Cost: 0.0034
Current parameters: [2.59053102 3.59236137 1.5322782  2.51275055 0.77065404 1.7188188
 2.63955165 1.5439625  1.20361946 0.8494952  1.8962769  2.27805988
 1.05796433 0.5355946  0.49492691 4.13615673], Cost: 0.0328
Current parameters: [2.58719937 3.59108828 1.52809904 2.50446016 0.76135133 1.70789283
 2.63038213 1.54917462 1.21019365 0.86022611 1.89266143 2.27242498
 1.06421131 0.55642929 0.49880277 4.14680077], Cost: 0.0097
Current parameters: [2.58438667 3.58320928 1.52373339 2.50053661 0.75701562 1.69748556
 2.62665946 1.55197852 1.18169119 0.85081855 1.89035521 2.27543632
 1.06026445 0.56043788 0.51292494 4.15279409], Cost: 0.0069
Current parameters: [2.58672554 3.59250315 1.53619235 2.50152753 0.76975594 1.70289146
 2.6387367  1.54856174 1.19648407 0.85787072 1.89587363 2.26671637
 1.06433191 0.55520504 0.50097503 4.14493307], Cost: 0.0030
```



```
Current parameters: [2.58971917 3.59337578 1.54191537 2.48369874 0.78151488 1.71494681
 2.62764075 1.55229593 1.19533113 0.85314769 1.89801173 2.26528421
 1.07132035 0.56506919 0.50224485 4.14912133], Cost: 0.0035
Current parameters: [2.58807033 3.59242622 1.54121949 2.50295891 0.76628236 1.70312847
 2.6379469  1.56187426 1.19527312 0.85855602 1.8965198  2.26723834
 1.06504811 0.55183271 0.49801201 4.1462542 ], Cost: 0.0068
Current parameters: [2.57037539 3.59951497 1.52591793 2.49851921 0.77599574 1.69937243
 2.64165023 1.5597278  1.20035657 0.85585978 1.8955414  2.2672881
 1.06851287 0.55803642 0.50945707 4.12931104], Cost: 0.0073
Current parameters: [2.58725046 3.60344855 1.53321148 2.5021433  0.77143659 1.70016923
 2.63393153 1.548443   1.19609663 0.85821446 1.90094935 2.26581554
 1.06161282 0.55121982 0.50222604 4.15038985], Cost: 0.0018
Current parameters: [2.58781467 3.6107879  1.52985628 2.49342772 0.76761175 1.69820437
 2.65399928 1.54761995 1.19798293 0.85890573 1.89411402 2.26024475
 1.06399776 0.55780545 0.504955   4.16697238], Cost: 0.0045
Current parameters: [2.59044307 3.60230631 1.53408063 2.5045907  0.76955407 1.69709308
 2.63755362 1.54074817 1.19187316 0.8584425  1.90141876 2.2640546
 1.06574179 0.55743621 0.49673815 4.14459832], Cost: 0.0019
Current parameters: [2.59111031 3.621432   1.52709251 2.49634818 0.760646   1.69787563
 2.62363916 1.55909084 1.1928473  0.85351632 1.90864056 2.26805165
 1.0684289  0.55576732 0.49442767 4.14916968], Cost: 0.0039
Current parameters: [2.58172473 3.60496773 1.53926468 2.50150656 0.77093622 1.69874895
 2.6358126  1.54714157 1.19740386 0.85681584 1.90438296 2.27514593
 1.05610502 0.55638871 0.49991546 4.15174989], Cost: 0.0031
Current parameters: [2.59729542 3.601517   1.52756167 2.50654452 0.78108269 1.69554783
 2.63159943 1.55616186 1.19590385 0.87068633 1.90943671 2.25954028
 1.04890683 0.56533928 0.50583219 4.15103054], Cost: 0.0066
Current parameters: [2.58611173 3.60733203 1.5382747  2.50368475 0.76502265 1.70575379
 2.63294348 1.54719119 1.19594854 0.85671503 1.8993981  2.25918079
 1.05614807 0.55408816 0.50730456 4.14688239], Cost: 0.0036
Current parameters: [2.57510409 3.60096531 1.53822025 2.4990473  0.77496557 1.68477922
 2.62211987 1.5455822  1.19876706 0.85551461 1.89492413 2.2513784
 1.06069473 0.55016826 0.49258212 4.15647872], Cost: 0.0038
Current parameters: [2.58866321 3.60297795 1.53747889 2.49579484 0.76811098 1.69512569
 2.63254728 1.54533926 1.19710237 0.86567087 1.90417587 2.26722617
 1.06511081 0.54770732 0.50842402 4.14855557], Cost: 0.0040
Current parameters: [2.58645684 3.60111542 1.53144383 2.50252242 0.76941074 1.69865577
 2.63214294 1.54833822 1.19158284 0.85686635 1.89958136 2.26733164
 1.06144601 0.55288677 0.5053507  4.15216665], Cost: 0.0005
Current parameters: [2.58836023 3.60220354 1.52884465 2.50337432 0.77503635 1.70015506
 2.63173281 1.54334627 1.18308176 0.84993838 1.89958787 2.26948284
```

```
 1.06042211 0.54625348 0.50631187 4.15358214], Cost: 0.0014
Current parameters: [2.5911531  3.59994847 1.5331499  2.50333379 0.76854078 1.6993328
 2.63029513 1.54770147 1.19121155 0.85839854 1.90078492 2.26691242
 1.05922324 0.55316919 0.5035019  4.15622452], Cost: 0.0014
Current parameters: [2.58712777 3.6007721  1.5254605  2.50428473 0.77692963 1.69481695
 2.6321633  1.5522704  1.18936749 0.86039922 1.90266126 2.265055
 1.05676111 0.55918042 0.50937495 4.14931648], Cost: 0.0021
Current parameters: [2.58539366 3.6021436  1.53101047 2.49989457 0.76969401 1.69827047
 2.63092716 1.5480577  1.1895945  0.85992322 1.89513803 2.26741018
 1.05877283 0.55218443 0.50246556 4.15118774], Cost: 0.0006
Current parameters: [2.58004249 3.59898138 1.53361296 2.50290972 0.77110657 1.69090981
 2.62678236 1.5469464  1.19320336 0.85224924 1.89877506 2.26012685
 1.06290106 0.55279071 0.50267931 4.15594465], Cost: 0.0010
Current parameters: [2.58645684 3.60111542 1.53144383 2.50252242 0.76941074 1.69865577
 2.63214294 1.54833822 1.19158284 0.85686635 1.89958136 2.26733164
 1.06144601 0.55288677 0.5053507  4.15216665], Cost: 0.0005

   Return from subroutine COBYLA because the MAXFUN limit has been reached.

   NFVALS =  100   F = 5.141135E-04    MAXCV = 0.000000E+00
   X = 2.586457E+00   3.601115E+00   1.531444E+00   2.502522E+00   7.694107E-01
       1.698656E+00   2.632143E+00   1.548338E+00   1.191583E+00   8.568663E-01
       1.899581E+00   2.267332E+00   1.061446E+00   5.528868E-01   5.053507E-01
       4.152167E+00
Simulating for QAOA depth p = 9
Current parameters: [2.16885608 2.05879269 0.71100305 1.43561497 1.39360366 0.59005363
 1.59219654 1.4941505  2.74854803 1.95808086 1.78083736 2.90447067
 1.69827454 1.6334499  3.12371546 0.57899688 0.12792523 2.93193321], Cost: 1.5089
Current parameters: [2.16885608 3.05879269 0.71100305 1.43561497 1.39360366 0.59005363
 1.59219654 1.4941505  2.74854803 1.95808086 1.78083736 2.90447067
 1.69827454 1.6334499  3.12371546 0.57899688 0.12792523 2.93193321], Cost: 1.0288
Current parameters: [2.16885608 3.05879269 1.71100305 1.43561497 1.39360366 0.59005363
 1.59219654 1.4941505  2.74854803 1.95808086 1.78083736 2.90447067
 1.69827454 1.6334499  3.12371546 0.57899688 0.12792523 2.93193321], Cost: 3.8495
Current parameters: [2.16885608 3.05879269 0.71100305 2.43561497 1.39360366 0.59005363
 1.59219654 1.4941505  2.74854803 1.95808086 1.78083736 2.90447067
 1.69827454 1.6334499  3.12371546 0.57899688 0.12792523 2.93193321], Cost: 3.3928
Current parameters: [2.16885608 3.05879269 0.71100305 1.43561497 2.39360366 0.59005363
 1.59219654 1.4941505  2.74854803 1.95808086 1.78083736 2.90447067
 1.69827454 1.6334499  3.12371546 0.57899688 0.12792523 2.93193321], Cost: 3.0311
Current parameters: [2.16885608 3.05879269 0.71100305 1.43561497 1.39360366 1.59005363
```



```
 1.59219654 1.4941505  2.74854803 1.95808086 1.78083736 2.90447067
 1.69827454 1.6334499  3.12371546 0.57899688 0.12792523 2.93193321], Cost: 3.2888
Current parameters: [2.16885608 3.05879269 0.71100305 1.43561497 1.39360366 0.59005363
 2.59219654 1.4941505  2.74854803 1.95808086 1.78083736 2.90447067
 1.69827454 1.6334499  3.12371546 0.57899688 0.12792523 2.93193321], Cost: 3.2225
Current parameters: [2.16885608 3.05879269 0.71100305 1.43561497 1.39360366 0.59005363
 1.59219654 2.4941505  2.74854803 1.95808086 1.78083736 2.90447067
 1.69827454 1.6334499  3.12371546 0.57899688 0.12792523 2.93193321], Cost: 0.8800
Current parameters: [2.16885608 3.05879269 0.71100305 1.43561497 1.39360366 0.59005363
 1.59219654 2.4941505  3.74854803 1.95808086 1.78083736 2.90447067
 1.69827454 1.6334499  3.12371546 0.57899688 0.12792523 2.93193321], Cost: 1.1958
Current parameters: [2.16885608 3.05879269 0.71100305 1.43561497 1.39360366 0.59005363
 1.59219654 2.4941505  2.74854803 2.95808086 1.78083736 2.90447067
 1.69827454 1.6334499  3.12371546 0.57899688 0.12792523 2.93193321], Cost: 2.8491
Current parameters: [2.16885608 3.05879269 0.71100305 1.43561497 1.39360366 0.59005363
 1.59219654 2.4941505  2.74854803 1.95808086 2.78083736 2.90447067
 1.69827454 1.6334499  3.12371546 0.57899688 0.12792523 2.93193321], Cost: 2.8281
Current parameters: [2.16885608 3.05879269 0.71100305 1.43561497 1.39360366 0.59005363
 1.59219654 2.4941505  2.74854803 1.95808086 1.78083736 3.90447067
 1.69827454 1.6334499  3.12371546 0.57899688 0.12792523 2.93193321], Cost: 1.9248
Current parameters: [2.16885608 3.05879269 0.71100305 1.43561497 1.39360366 0.59005363
 1.59219654 2.4941505  2.74854803 1.95808086 1.78083736 2.90447067
 2.69827454 1.6334499  3.12371546 0.57899688 0.12792523 2.93193321], Cost: 1.9697
Current parameters: [2.16885608 3.05879269 0.71100305 1.43561497 1.39360366 0.59005363
 1.59219654 2.4941505  2.74854803 1.95808086 1.78083736 2.90447067
 1.69827454 2.6334499  3.12371546 0.57899688 0.12792523 2.93193321], Cost: 2.7220
Current parameters: [2.16885608 3.05879269 0.71100305 1.43561497 1.39360366 0.59005363
 1.59219654 2.4941505  2.74854803 1.95808086 1.78083736 2.90447067
 1.69827454 1.6334499  4.12371546 0.57899688 0.12792523 2.93193321], Cost: 2.6411
Current parameters: [2.16885608 3.05879269 0.71100305 1.43561497 1.39360366 0.59005363
 1.59219654 2.4941505  2.74854803 1.95808086 1.78083736 2.90447067
 1.69827454 1.6334499  3.12371546 1.57899688 0.12792523 2.93193321], Cost: 1.1440
Current parameters: [2.16885608 3.05879269 0.71100305 1.43561497 1.39360366 0.59005363
 1.59219654 2.4941505  2.74854803 1.95808086 1.78083736 2.90447067
 1.69827454 1.6334499  3.12371546 0.57899688 1.12792523 2.93193321], Cost: 2.8034
Current parameters: [2.16885608 3.05879269 0.71100305 1.43561497 1.39360366 0.59005363
 1.59219654 2.4941505  2.74854803 1.95808086 1.78083736 2.90447067
 1.69827454 1.6334499  3.12371546 0.57899688 0.12792523 3.93193321], Cost: 3.3518
Current parameters: [ 2.31746513  3.12331135  0.33194856  1.11792594  1.12453133  0.28634893
  1.29739465  2.5141448   2.7061123   1.6934705   1.51904647  2.76407284
  1.55183173  1.3859129   2.88705055  0.54352387 -0.13054433  2.59976001], Cost: 1.2014
```



```
Current parameters: [2.19802846 3.07145788 1.17683775 1.37325172 1.34078401 0.53043555
 1.5343261  2.49807544 2.74021777 1.9061371  1.72944708 2.87691018
 1.66952741 1.58485769 3.07725748 0.57203343 0.07718693 2.86672668], Cost: 1.8177
Current parameters: [2.06929653 3.2881132  0.71100305 1.43561497 1.39360366 0.59005363
 1.59219654 2.4941505  2.74854803 1.95808086 1.78083736 2.90447067
 1.69827454 1.6334499  3.12371546 0.57899688 0.12792523 2.93193321], Cost: 1.2033
Current parameters: [1.72187156 2.84888328 0.69279975 1.40903287 1.37108948 0.56464165
 1.56752948 2.49582349 2.74499729 1.93594003 1.75893245 2.89272312
 1.68602118 1.61273765 3.10391292 0.57602874 0.10629822 2.90413917], Cost: 0.3604
Current parameters: [1.72187156 2.84888328 0.69279975 1.58178751 1.37108948 0.38393239
 1.56752948 2.49582349 2.74499729 1.93594003 1.75893245 2.89272312
 1.68602118 1.61273765 3.10391292 0.57602874 0.10629822 2.90413917], Cost: 0.3112
Current parameters: [1.70482253 2.79130423 0.63517611 1.90854679 1.29981949 0.68661687
 1.48944442 2.50111944 2.73375721 1.86585189 1.68959111 2.85553552
 1.64723244 1.54717179 3.04122678 0.56663289 0.03783661 2.81615546], Cost: 2.1186
Current parameters: [1.72187156 2.84888328 0.69279975 1.58178751 1.1864386  0.38393239
 1.7360645  2.49582349 2.74499729 1.93594003 1.75893245 2.89272312
 1.68602118 1.61273765 3.10391292 0.57602874 0.10629822 2.90413917], Cost: 0.3329
Current parameters: [1.70666034 2.79751099 0.64138768 1.51246249 1.68012218 0.30901244
 1.90203416 2.50054856 2.73496884 1.87340708 1.69706579 2.85954418
 1.65141369 1.55423949 3.04798406 0.56764572 0.04521647 2.82563971], Cost: 2.3890
Current parameters: [1.72187156 2.84888328 0.69279975 1.58178751 1.37108948 0.38393239
 1.56752948 2.49582349 2.74499729 1.74039928 1.75893245 2.89272312
 1.68602118 1.61273765 3.10391292 0.57602874 0.10629822 3.05990764], Cost: 0.8355
Current parameters: [1.70649693 2.7969591  0.64083536 1.51171773 1.29177801 0.30820758
 1.47651146 2.50059932 2.7348611  2.26560176 1.69640116 2.85918774
 1.6510419  1.55361105 3.04738322 0.56755567 0.04456026 3.209938  ], Cost: 2.1062
Current parameters: [ 1.72187156  2.84888328  0.69279975  1.58178751  1.37108948  0.38393239
  1.56752948  2.49582349  2.74499729  1.93594003  1.93457702  2.89272312
  1.68602118  1.61273765  3.10391292  0.57602874 -0.07160339  2.90413917], Cost: 0.6674
Current parameters: [1.70935493 2.80661132 0.65049505 1.52474305 1.30652127 0.32228411
 1.49343086 2.49971154 2.73674533 1.91854988 2.05863389 2.86542165
 1.65754422 1.56460213 3.05789156 0.56913073 0.45451473 2.79435628], Cost: 1.9137
Current parameters: [1.72187156 2.84888328 0.69279975 1.58178751 1.37108948 0.38393239
 1.56752948 2.49582349 2.74499729 1.93594003 1.75893245 2.89272312
 1.68602118 1.78550153 2.92321249 0.57602874 0.10629822 2.90413917], Cost: 0.2639
Current parameters: [1.71082324 2.8115702  0.65545778 1.53143488 1.31409571 0.32951601
 1.50212331 2.49925544 2.73771336 1.9205899  1.67720492 2.86862436
 1.66088483 2.12385431 3.24066313 0.56993993 0.07178763 2.80723481], Cost: 1.3841
Current parameters: [1.72187156 2.84888328 0.69279975 1.58178751 1.37108948 0.38393239
 1.56752948 2.49582349 2.74499729 1.93594003 1.75893245 2.71226103
```



```
 1.85903401 1.78550153 2.92321249 0.57602874 0.10629822 2.90413917], Cost: 0.3970
Current parameters: [1.71557813 2.8276287  0.67152871 1.55310523 1.33862422 0.35293528
 1.53027228 2.49777843 2.74084815 1.92719616 1.71237814 3.23520963
 2.03314955 1.76096933 2.89631647 0.57256038 0.08664001 2.84893971], Cost: 2.2104
Current parameters: [1.68802821 2.83419018 0.82796013 1.65413614 1.43236643 0.45309631
 1.63466593 2.49582349 2.74499729 1.99620084 1.81855117 2.92469649
 1.71937121 1.84187415 2.97710917 0.57602874 0.16516056 2.97978635], Cost: 0.5666
Current parameters: [1.74246661 2.83809952 1.10045594 1.49288809 1.28709155 0.29513653
 1.47370231 2.49790538 2.74057871 1.87802353 1.66126803 2.83141259
 1.61131042 1.71390754 2.8510982  0.57233515 0.03788659 2.78434007], Cost: 1.1043
Current parameters: [1.69825241 2.90328644 0.69279975 1.58178751 1.37108948 0.38393239
 1.56752948 2.49582349 2.5021343  1.93594003 1.75893245 2.89272312
 1.68602118 1.78550153 2.92321249 0.57602874 0.10629822 2.90413917], Cost: 0.2210
Current parameters: [1.48416035 3.33808654 0.68301327 1.58481498 1.36751402 0.384139
 1.56234435 2.49729218 2.61860847 1.94984065 1.744209   2.87852485
 1.66362052 1.78622005 2.92131409 0.57342306 0.11152415 2.88836547], Cost: 0.2993
Current parameters: [1.75676946 2.93076677 0.69518284 1.58526751 1.37403693 0.3872592
 1.57075877 2.52778745 2.50259914 1.9388386  1.76180013 2.89426105
 1.68762533 1.78821307 2.92580495 0.33687647 0.10912952 2.90777784], Cost: 0.8166
Current parameters: [2.07285853 3.0855713  0.70177953 1.60725346 1.38827909 0.40636037
 1.58545854 2.71094268 2.50529095 1.9652675  1.76763632 2.89289586
 1.68078686 1.80417402 2.9392208  0.73743516 0.12898777 2.91725737], Cost: 0.6688
Current parameters: [1.79697762 2.95144337 0.69888095 1.60094751 1.3894646  0.37684085
 1.56041817 2.27577582 2.5033205  1.95368095 1.7473026  2.89664764
 1.69011468 1.77972901 2.9431029  0.57702031 0.12062505 2.89693353], Cost: 0.4957
Current parameters: [1.68452574 2.90095065 0.63985678 1.42667188 1.1942075  0.54822842
 1.71373554 2.43348085 2.49771301 1.81836574 1.925501   2.83492389
 1.60297485 1.92096692 2.72432014 0.63678137 0.00430137 3.02891897], Cost: 1.1985
Current parameters: [1.71362968 2.87750122 0.59549822 1.57325869 1.36802148 0.32660515
 1.51144216 2.49679461 2.58744221 2.03743147 1.70204206 2.91480301
 1.57909532 1.772337   2.83537477 0.57430582 0.1000795  2.97449709], Cost: 1.1290
Current parameters: [1.59603904 2.91320864 0.83743157 1.53287416 1.30390193 0.38664086
 1.55242956 2.51471685 2.28190695 2.1340361  1.72970532 2.751721
 1.83112827 1.72078761 3.09838146 0.6434554  0.08492135 3.10816668], Cost: 0.4933
Current parameters: [1.66387202 2.89130771 0.68843105 1.54938458 1.51651045 0.35161732
 1.72793324 2.50495905 2.46630554 1.92177945 1.71587352 2.84667254
 1.67357802 1.75585912 2.93339214 0.60618308 0.09165865 2.83794675], Cost: 0.2970
Current parameters: [1.69753134 2.90159008 0.69178182 1.6393919  1.36836934 0.44452767
 1.57047502 2.49599283 2.50094424 1.93222124 1.69589236 2.89125926
 1.68572985 1.78594297 2.91929069 0.5757283  0.03844413 2.90588233], Cost: 0.2020
Current parameters: [1.64959109 2.87914735 0.6774695  1.74594431 1.34816279 0.52639956
```

```
 1.53032539 2.50896023 2.45375204 1.91282289 1.74853317 2.82576775
 1.66185282 1.73078188 2.93331906 0.61589595 0.14685038 2.79448332], Cost: 0.1544
Current parameters: [1.6498486  2.88551309 0.68889624 1.7525261  1.35504582 0.53772242
 1.5432608  2.50847377 2.44860595 1.91877733 1.76064648 2.86646808
 1.71349577 1.65553287 2.86514327 0.616759   0.15369982 2.80779441], Cost: 0.2378
Current parameters: [1.60294828 2.86253621 0.64202198 1.72642605 1.31609921 0.52026041
 1.51284992 2.52339388 2.43411868 1.88082472 1.71380167 2.94432419
 1.78790216 1.80760375 3.00176154 0.66834464 0.1444805  2.68993958], Cost: 1.2959
Current parameters: [1.65314277 2.87500393 0.75958575 1.74266076 1.34910172 0.48361468
 1.4888444  2.51820737 2.46619742 1.91330841 1.70386925 2.82766458
 1.64457363 1.70499128 2.92987062 0.61547677 0.14302467 2.74909906], Cost: 0.2407
Current parameters: [1.63917725 2.8783673  0.76265584 1.67352166 1.26150552 0.59559974
 1.58649045 2.48434982 2.43433504 1.83084538 1.81377277 2.75187111
 1.59657091 1.76674058 2.8438567  0.6489776  0.09886188 2.80229639], Cost: 0.2972
Current parameters: [1.74036792 2.91864203 0.66840518 1.75563224 1.35752306 0.53473575
 1.53958859 2.55749829 2.4716806  1.89441345 1.75557567 2.839232
 1.65075633 1.74279657 2.9216605  0.64918402 0.15474343 2.77029996], Cost: 0.2935
Current parameters: [1.67597029 2.9185701  0.741312   1.74307592 1.33123953 0.54031687
 1.53182049 2.55797804 2.35144203 2.01308077 1.74561793 2.73197615
 1.70290941 1.69413397 3.01728533 0.66992325 0.14999014 2.89911899], Cost: 0.0876
Current parameters: [1.72906268 2.81162962 0.74232477 1.74388751 1.33375554 0.53713383
 1.5303322  2.55761573 2.32195627 1.99690217 1.74418393 2.73582542
 1.70743944 1.6914669  3.01918761 0.67056605 0.15259573 2.91188435], Cost: 0.0626
Current parameters: [1.69922641 2.8415551  0.74181926 1.78384466 1.3726549  0.50510066
 1.49899209 2.5602427  2.32327162 1.81542003 1.70511575 2.71947673
 1.69365243 1.65266964 3.05711911 0.68728169 0.19956265 3.03532881], Cost: 1.2704
Current parameters: [1.72579522 2.81388763 0.75001031 1.81698765 1.29524112 0.4689039
 1.57248297 2.55761573 2.30814649 2.00032875 1.74780124 2.727382
 1.71917372 1.7241437  2.99142712 0.67056605 0.15571261 2.91618583], Cost: 0.0887
Current parameters: [ 1.72468653  2.80301331  0.73759135  1.79029912  1.38356762  0.48949307
  1.48360181  2.55926943  2.32371219  2.00225227  1.8993445   2.73105182
  1.69232088  1.6452197   3.06168807  0.68183675 -0.0034574   2.88929564], Cost: 0.6637
Current parameters: [1.72460958 2.81912234 0.74635759 1.7460462  1.39493178 0.53919749
 1.47816721 2.55761573 2.28655065 1.99870019 1.74596279 2.71047088
 1.73365707 1.74867625 2.96271755 0.67056605 0.15435202 2.91414146], Cost: 0.1358
Current parameters: [1.70188879 2.77512767 0.86176976 1.8025708  1.40326558 0.60844445
 1.57803402 2.55891355 2.39632907 2.05601128 1.79723783 2.81545323
 1.67396277 1.76011263 3.0456191  0.67941124 0.20838378 2.96308746], Cost: 0.7593
Current parameters: [1.73629195 2.79432399 0.7489309  1.74446656 1.33396384 0.54102597
 1.53414016 2.5575498  2.40123418 1.99001157 1.74804641 2.6711143
 1.77530172 1.69236069 3.02515122 0.67068303 0.15301794 2.90710751], Cost: 0.2181
```



```
Current parameters: [1.71856239 2.76688724 0.63895114 1.71271029 1.29377703 0.52169173
 1.51596974 2.56450296 2.35011249 2.13583406 1.68492435 2.68113465
 1.60598019 1.6627632  2.93382557 0.70836498 0.16411033 2.92058827], Cost: 0.3386
Current parameters: [1.70591956 2.79878276 0.69897218 1.76305334 1.34248317 0.55226652
 1.53646034 2.54630587 2.31559733 1.99778966 1.7584291  2.70871955
 1.68730067 1.71130687 3.04911643 0.57447514 0.16975038 2.90956271], Cost: 0.1166
Current parameters: [1.8350572  2.86437658 0.82668912 1.68815503 1.28491396 0.55041409
 1.56165956 2.63306519 2.26001966 2.03678072 1.71622885 2.73653976
 1.73853095 1.63997339 2.96306819 0.55411204 0.13871166 2.86399721], Cost: 0.3432
Current parameters: [1.7761526  2.83453603 0.74566045 1.74922568 1.33671922 0.53981549
 1.53328697 2.4484809  2.30983288 2.01229464 1.73733642 2.72904483
 1.7081257  1.68533823 3.02842012 0.67656668 0.16218424 2.89846386], Cost: 0.1859
Current parameters: [1.72185911 2.80920618 0.74266439 1.73668567 1.37035966 0.52956044
 1.57027067 2.5601637  2.31315617 1.99382019 1.73421353 2.72191503
 1.70194291 1.68193896 3.02010373 0.67770101 0.14949251 2.89653852], Cost: 0.1648
Current parameters: [1.69427408 2.79619708 0.71221823 1.7156793  1.29040684 0.54865719
 1.53171305 2.55811314 2.28617045 2.00735234 1.72973048 2.76897299
 1.7573756  1.73253473 3.05104609 0.69144015 0.14907978 2.86584417], Cost: 0.3423
Current parameters: [1.72880169 2.81069761 0.74148523 1.77318479 1.33344914 0.56619883
 1.53110135 2.55767108 2.3218118  1.99509594 1.71469939 2.73560726
 1.70720579 1.69114723 3.01782875 0.67029465 0.11618676 2.9125596 ], Cost: 0.1814
Current parameters: [1.73336849 2.80843401 0.73522538 1.75040655 1.34301676 0.5038391
 1.50131008 2.56135772 2.3164346  2.01453477 1.82724372 2.73569279
 1.69752783 1.66461178 3.03402434 0.67327725 0.08271038 2.89510543], Cost: 0.2167
Current parameters: [1.74207781 2.81718724 0.72994881 1.75975533 1.3553318  0.51860645
 1.51722708 2.5698754  2.32698233 2.01727408 1.72592622 2.75655093
 1.72581114 1.67745034 3.03561139 0.66382384 0.16321524 2.90963648], Cost: 0.0690
Current parameters: [1.78110185 2.83775034 0.78811161 1.71051791 1.30122655 0.5417215
 1.53945793 2.59629504 2.29681928 2.01198216 1.73986101 2.73392997
 1.71760031 1.66912783 2.98571646 0.61115156 0.15757973 2.89749526], Cost: 0.1455
Current parameters: [1.73281004 2.81263236 0.73725773 1.71727409 1.33757066 0.51867445
 1.54116365 2.54936777 2.33429082 2.00155744 1.73270655 2.74870817
 1.70943325 1.71177972 3.01922179 0.65784232 0.12607801 2.94126839], Cost: 0.0416
Current parameters: [1.72937296 2.79196917 0.69107438 1.69992834 1.31261341 0.50724968
 1.52497627 2.5566382  2.35413541 2.06647916 1.71110243 2.72278651
 1.65776699 1.69723495 2.97338457 0.67755299 0.14657606 2.95335847], Cost: 0.1422
Current parameters: [1.69645287 2.79908079 0.76093498 1.71471994 1.33540794 0.50918525
 1.53135581 2.53301634 2.32422584 2.01007711 1.72271089 2.74696093
 1.72170569 1.68566086 3.01493937 0.64675602 0.12461583 2.95232705], Cost: 0.0465
Current parameters: [1.73906019 2.83694317 0.71983426 1.70984368 1.32567521 0.48724719
 1.51104405 2.56020026 2.32137454 1.91983512 1.72358693 2.74515053
```



```
 1.69263284 1.68782116 3.03192806 0.6649734  0.15615287 3.00100253], Cost: 0.2547
Current parameters: [1.73299572 2.81830359 0.71682083 1.72126046 1.33926036 0.54014076
 1.56133045 2.54933505 2.32203236 2.01136816 1.753596   2.75478343
 1.73368979 1.69738859 3.00656071 0.66228812 0.1307194  2.97047872], Cost: 0.0639
Current parameters: [1.72668168 2.79781111 0.80177578 1.73106182 1.35521739 0.53872257
 1.54824797 2.55581637 2.36780256 2.02940665 1.76322905 2.79606786
 1.67690678 1.745925   3.03407008 0.66059005 0.16653963 2.9736377 ], Cost: 0.2835
Current parameters: [1.73558518 2.82055007 0.74717848 1.71645391 1.35421015 0.51979108
 1.52178727 2.55745923 2.30652734 2.01757473 1.73084712 2.71821656
 1.7165015  1.7372884  3.02513813 0.66653201 0.12606107 2.95695285], Cost: 0.0528
Current parameters: [1.72373763 2.80924592 0.72547803 1.71747226 1.38592544 0.51605073
 1.50155873 2.53611973 2.33583108 1.97450743 1.74081977 2.7731737
 1.7284615  1.74255985 2.93102761 0.6414936  0.13263609 2.91875529], Cost: 0.0771
Current parameters: [1.71588559 2.79748463 0.7311059  1.69827592 1.31058385 0.53102798
 1.53048385 2.55175279 2.31707398 1.99492264 1.74123793 2.76030434
 1.71939342 1.72664473 3.02629341 0.67917419 0.14475203 2.92485818], Cost: 0.0408
Current parameters: [1.70925031 2.81770095 0.6963234  1.67745683 1.30527356 0.49111408
 1.55637689 2.55146938 2.30571122 2.02790913 1.70069169 2.79034044
 1.77026012 1.7571777  3.05318539 0.64695375 0.10793117 2.89592745], Cost: 0.2489
Current parameters: [1.72690856 2.79565583 0.72830974 1.69675505 1.31039721 0.51333678
 1.52151654 2.55392557 2.32559552 1.99427305 1.77761358 2.76356491
 1.7049093  1.71722212 3.03664862 0.67890922 0.10724387 2.91246767], Cost: 0.0791
Current parameters: [1.66827151 2.78528715 0.72842063 1.70186574 1.30974216 0.53868787
 1.48037582 2.55731245 2.25389832 2.0261074  1.78959222 2.75089814
 1.75292619 1.70892126 3.00661043 0.70314388 0.16976065 2.93796098], Cost: 0.0990
Current parameters: [1.73848526 2.80684444 0.73324237 1.69684696 1.30655182 0.53232794
 1.52565744 2.4975348  2.30883692 2.00098762 1.74401638 2.75854163
 1.7183609  1.72404028 3.02969511 0.68519458 0.15509998 2.91835782], Cost: 0.0943
Current parameters: [1.70696356 2.79482801 0.72040414 1.69162585 1.32679375 0.54093974
 1.52355394 2.5460175  2.32472923 1.99448233 1.7372262  2.76257377
 1.7180783  1.72475144 3.03788611 0.68599731 0.14107275 2.92792284], Cost: 0.0472
Current parameters: [1.73426608 2.80531075 0.74470191 1.66119758 1.31173326 0.55685758
 1.51900413 2.56638907 2.31159818 1.99896035 1.7379985  2.76140335
 1.72018729 1.70732119 3.0228983  0.65832084 0.1456049  2.91751817], Cost: 0.0163
Current parameters: [1.72967559 2.80027792 0.73538368 1.66500105 1.31004208 0.561001
 1.51855202 2.56319492 2.31114351 1.99733866 1.74552595 2.7528783
 1.71523411 1.71247098 3.02956996 0.63552245 0.15259649 2.91608999], Cost: 0.0151
Current parameters: [1.73458896 2.79071399 0.74209791 1.65915389 1.30243065 0.56113965
 1.51350783 2.56513597 2.34758942 1.99342668 1.75304876 2.72296777
 1.75074361 1.71234778 3.028456   0.64037074 0.15885379 2.91396283], Cost: 0.0040
Current parameters: [1.74712235 2.76574511 0.73855313 1.65933364 1.3060259  0.55885401
```



```
 1.51412865 2.56530895 2.33855473 1.99014858 1.74755347 2.72298843
 1.75470634 1.7099097  3.02880359 0.64023263 0.15571163 2.91722434], Cost: 0.0109
Current parameters: [1.73711601 2.77540891 0.77543487 1.66047759 1.31072858 0.57012429
 1.51753239 2.57302143 2.36613885 2.00881845 1.76665183 2.74709917
 1.74356822 1.72995452 3.03563654 0.63564524 0.17736175 2.92804408], Cost: 0.0348
Current parameters: [1.73455966 2.79716159 0.7507469  1.66237081 1.30710446 0.56144322
 1.51329795 2.56453047 2.34008185 1.97206487 1.75735316 2.72809862
 1.76222405 1.71298703 3.03924989 0.63731455 0.1581797  2.9173442 ], Cost: 0.0233
Current parameters: [1.73945535 2.79622233 0.72933301 1.6451001  1.28784223 0.54328596
 1.49644869 2.57950345 2.3597529  1.97622576 1.7428579  2.71683211
 1.7293979  1.70118604 3.02258622 0.64164444 0.18010203 2.94241727], Cost: 0.0588
Current parameters: [1.73403165 2.79117336 0.74455389 1.65895351 1.290612   0.56262537
 1.52226107 2.56851914 2.34308736 2.00225507 1.75092395 2.71678537
 1.75021518 1.70457728 3.05040446 0.64521821 0.15846317 2.91970956], Cost: 0.0052
Current parameters: [1.72775718 2.79869437 0.72746963 1.642083   1.29894934 0.54498564
 1.52079009 2.57516303 2.3480053  2.01639244 1.73208063 2.73606521
 1.7795263  1.72906084 3.03006231 0.62075405 0.1501036  2.90158105], Cost: 0.0272
Current parameters: [1.72536457 2.78860741 0.74066382 1.65228417 1.30885928 0.5531988
 1.50839495 2.56705955 2.33438917 2.00122751 1.7679681  2.72077276
 1.75787954 1.707061   3.02606351 0.64543358 0.17018611 2.91551392], Cost: 0.0037
Current parameters: [1.7083276  2.783702   0.74288507 1.67165258 1.28766923 0.58589554
 1.48089482 2.5762914  2.32219687 2.0126471  1.76644073 2.71560122
 1.76831546 1.70661717 3.01085724 0.64801861 0.15630863 2.91764492], Cost: 0.0033
Current parameters: [1.70051807 2.78070812 0.74637184 1.65599792 1.28229784 0.58848027
 1.48971975 2.56904169 2.31850762 2.00862876 1.77031665 2.71012192
 1.76603935 1.7173163  3.00467822 0.64877142 0.14587783 2.92953652], Cost: 0.0019
Current parameters: [1.68557219 2.7759792  0.75459722 1.65451782 1.29455665 0.60398297
 1.5181072  2.58438441 2.31493993 2.00523262 1.76357764 2.68463629
 1.75604757 1.70901887 2.99097555 0.65490504 0.15095925 2.89760346], Cost: 0.0452
Current parameters: [1.69645795 2.77670343 0.75897984 1.65104447 1.27934119 0.580208
 1.4751138  2.56107078 2.31489877 2.00783841 1.76161629 2.70448123
 1.75982437 1.72492618 3.01164092 0.65033999 0.14758992 2.91792052], Cost: 0.0067
Current parameters: [1.72322659 2.79295702 0.74164928 1.65467312 1.27379545 0.60403886
 1.49225719 2.52167144 2.313792   2.02496338 1.77420627 2.70828205
 1.77435021 1.71057411 2.99594907 0.65125791 0.15754823 2.92621054], Cost: 0.0086
Current parameters: [1.68979219 2.77161124 0.74563919 1.65186303 1.2697743  0.58690665
 1.49251689 2.563211   2.32498453 2.00252678 1.77426379 2.72462425
 1.7662693  1.70393674 3.00386172 0.64731692 0.14572813 2.92099005], Cost: 0.0138
Current parameters: [1.70051807 2.78070812 0.74637184 1.65599792 1.28229784 0.58848027
 1.48971975 2.56904169 2.31850762 2.00862876 1.77031665 2.71012192
 1.76603935 1.7173163  3.00467822 0.64877142 0.14587783 2.92953652], Cost: 0.0019
```

```
   Return from subroutine COBYLA because the MAXFUN limit has been reached.

   NFVALS =  100   F = 1.895520E-03    MAXCV = 0.000000E+00
   X = 1.700518E+00   2.780708E+00   7.463718E-01   1.655998E+00   1.282298E+00
       5.884803E-01   1.489720E+00   2.569042E+00   2.318508E+00   2.008629E+00
       1.770317E+00   2.710122E+00   1.766039E+00   1.717316E+00   3.004678E+00
       6.487714E-01   1.458778E-01   2.929537E+00
Simulating for QAOA depth p = 10
Current parameters: [1.69458143 0.28767029 2.10295663 1.40745378 0.46915245 2.94482897
 2.97415879 1.57490216 1.77548563 1.67261832 1.49677104 1.47477423
 1.30254815 2.92964063 1.58539748 3.05169531 2.40359045 0.62695692
 2.36227917 2.99080026], Cost: 3.0513
Current parameters: [0.69458143 1.28767029 2.10295663 1.40745378 0.46915245 2.94482897
 2.97415879 1.57490216 1.77548563 1.67261832 1.49677104 1.47477423
 1.30254815 2.92964063 1.58539748 3.05169531 2.40359045 0.62695692
 2.36227917 2.99080026], Cost: 3.0016
Current parameters: [0.69458143 0.28767029 3.10295663 1.40745378 0.46915245 2.94482897
 2.97415879 1.57490216 1.77548563 1.67261832 1.49677104 1.47477423
 1.30254815 2.92964063 1.58539748 3.05169531 2.40359045 0.62695692
 2.36227917 2.99080026], Cost: 2.7243
Current parameters: [0.69458143 0.28767029 2.10295663 2.40745378 0.46915245 2.94482897
 2.97415879 1.57490216 1.77548563 1.67261832 1.49677104 1.47477423
 1.30254815 2.92964063 1.58539748 3.05169531 2.40359045 0.62695692
 2.36227917 2.99080026], Cost: 2.0621
Current parameters: [0.69458143 0.28767029 2.10295663 1.40745378 1.46915245 2.94482897
 2.97415879 1.57490216 1.77548563 1.67261832 1.49677104 1.47477423
 1.30254815 2.92964063 1.58539748 3.05169531 2.40359045 0.62695692
 2.36227917 2.99080026], Cost: 2.9177
Current parameters: [0.69458143 0.28767029 2.10295663 1.40745378 0.46915245 3.94482897
 2.97415879 1.57490216 1.77548563 1.67261832 1.49677104 1.47477423
 1.30254815 2.92964063 1.58539748 3.05169531 2.40359045 0.62695692
 2.36227917 2.99080026], Cost: 2.8075
Current parameters: [0.69458143 0.28767029 2.10295663 1.40745378 0.46915245 2.94482897
 3.97415879 1.57490216 1.77548563 1.67261832 1.49677104 1.47477423
 1.30254815 2.92964063 1.58539748 3.05169531 2.40359045 0.62695692
 2.36227917 2.99080026], Cost: 3.3741
Current parameters: [0.69458143 0.28767029 2.10295663 1.40745378 0.46915245 2.94482897
 2.97415879 2.57490216 1.77548563 1.67261832 1.49677104 1.47477423
 1.30254815 2.92964063 1.58539748 3.05169531 2.40359045 0.62695692
 2.36227917 2.99080026], Cost: 1.6731
```



```
Current parameters: [0.69458143 0.28767029 2.10295663 1.40745378 0.46915245 2.94482897
 2.97415879 1.57490216 2.77548563 1.67261832 1.49677104 1.47477423
 1.30254815 2.92964063 1.58539748 3.05169531 2.40359045 0.62695692
 2.36227917 2.99080026], Cost: 0.9450
Current parameters: [0.69458143 0.28767029 2.10295663 1.40745378 0.46915245 2.94482897
 2.97415879 1.57490216 2.77548563 2.67261832 1.49677104 1.47477423
 1.30254815 2.92964063 1.58539748 3.05169531 2.40359045 0.62695692
 2.36227917 2.99080026], Cost: 1.1712
Current parameters: [0.69458143 0.28767029 2.10295663 1.40745378 0.46915245 2.94482897
 2.97415879 1.57490216 2.77548563 1.67261832 2.49677104 1.47477423
 1.30254815 2.92964063 1.58539748 3.05169531 2.40359045 0.62695692
 2.36227917 2.99080026], Cost: 3.3285
Current parameters: [0.69458143 0.28767029 2.10295663 1.40745378 0.46915245 2.94482897
 2.97415879 1.57490216 2.77548563 1.67261832 1.49677104 2.47477423
 1.30254815 2.92964063 1.58539748 3.05169531 2.40359045 0.62695692
 2.36227917 2.99080026], Cost: 3.2909
Current parameters: [0.69458143 0.28767029 2.10295663 1.40745378 0.46915245 2.94482897
 2.97415879 1.57490216 2.77548563 1.67261832 1.49677104 1.47477423
 2.30254815 2.92964063 1.58539748 3.05169531 2.40359045 0.62695692
 2.36227917 2.99080026], Cost: 1.3945
Current parameters: [0.69458143 0.28767029 2.10295663 1.40745378 0.46915245 2.94482897
 2.97415879 1.57490216 2.77548563 1.67261832 1.49677104 1.47477423
 1.30254815 3.92964063 1.58539748 3.05169531 2.40359045 0.62695692
 2.36227917 2.99080026], Cost: 2.3102
Current parameters: [0.69458143 0.28767029 2.10295663 1.40745378 0.46915245 2.94482897
 2.97415879 1.57490216 2.77548563 1.67261832 1.49677104 1.47477423
 1.30254815 2.92964063 2.58539748 3.05169531 2.40359045 0.62695692
 2.36227917 2.99080026], Cost: 1.8026
Current parameters: [0.69458143 0.28767029 2.10295663 1.40745378 0.46915245 2.94482897
 2.97415879 1.57490216 2.77548563 1.67261832 1.49677104 1.47477423
 1.30254815 2.92964063 1.58539748 4.05169531 2.40359045 0.62695692
 2.36227917 2.99080026], Cost: 1.9964
Current parameters: [0.69458143 0.28767029 2.10295663 1.40745378 0.46915245 2.94482897
 2.97415879 1.57490216 2.77548563 1.67261832 1.49677104 1.47477423
 1.30254815 2.92964063 1.58539748 3.05169531 3.40359045 0.62695692
 2.36227917 2.99080026], Cost: 2.2785
Current parameters: [0.69458143 0.28767029 2.10295663 1.40745378 0.46915245 2.94482897
 2.97415879 1.57490216 2.77548563 1.67261832 1.49677104 1.47477423
 1.30254815 2.92964063 1.58539748 3.05169531 2.40359045 1.62695692
 2.36227917 2.99080026], Cost: 2.6218
Current parameters: [0.69458143 0.28767029 2.10295663 1.40745378 0.46915245 2.94482897
```



```
 2.97415879 1.57490216 2.77548563 1.67261832 1.49677104 1.47477423
 1.30254815 2.92964063 1.58539748 3.05169531 2.40359045 0.62695692
 3.36227917 2.99080026], Cost: 2.3170
Current parameters: [0.69458143 0.28767029 2.10295663 1.40745378 0.46915245 2.94482897
 2.97415879 1.57490216 2.77548563 1.67261832 1.49677104 1.47477423
 1.30254815 2.92964063 1.58539748 3.05169531 2.40359045 0.62695692
 2.36227917 3.99080026], Cost: 1.3671
Current parameters: [ 0.39409211 -0.00565282  1.84968322  1.2498125   0.18795443  2.67954724
  2.62705689  1.47343022  2.77915379  1.63995524  1.15258658  1.13601513
  1.237644    2.73250405  1.46155512  2.89986607  2.21101795  0.38481993
  2.16415881  2.92985003], Cost: 2.5013
Current parameters: [1.17935054 0.25000636 2.07043525 1.38721197 0.43304543 2.91076567
 2.92958944 1.56187273 2.77595663 1.66842424 1.4525763  1.43127612
 1.29421418 2.90432746 1.5694956  3.03219979 2.37886332 0.59586551
 2.33683968 2.98297399], Cost: 0.9730
Current parameters: [0.69458143 0.45105637 1.91373456 1.40745378 0.46915245 2.94482897
 2.97415879 1.57490216 2.77548563 1.67261832 1.49677104 1.47477423
 1.30254815 2.92964063 1.58539748 3.05169531 2.40359045 0.62695692
 2.36227917 2.99080026], Cost: 0.9451
Current parameters: [0.70352215 0.64500249 2.41152093 1.37771948 0.41611289 2.89479154
 2.90868844 1.55576252 2.77617751 1.66645741 1.43185098 1.41087749
 1.29030593 2.89245674 1.56203834 3.02305728 2.36726743 0.58128505
 2.32490971 2.97930383], Cost: 1.3337
Current parameters: [0.69458143 0.28767029 2.10295663 1.62552393 0.34690103 2.94482897
 2.97415879 1.57490216 2.77548563 1.67261832 1.49677104 1.47477423
 1.30254815 2.92964063 1.58539748 3.05169531 2.40359045 0.62695692
 2.36227917 2.99080026], Cost: 0.9891
Current parameters: [0.70291998 0.28354635 2.0994158  1.63785455 0.88930477 2.89816163
 2.91309796 1.5570516  2.77613092 1.66687235 1.43622343 1.41518103
 1.29113046 2.89496112 1.56361161 3.02498609 2.36971384 0.58436111
 2.3274266  2.98007813], Cost: 0.6857
Current parameters: [0.70291998 0.28354635 2.0994158  1.63785455 0.88930477 2.699531
 3.06490663 1.5570516  2.77613092 1.66687235 1.43622343 1.41518103
 1.29113046 2.89496112 1.56361161 3.02498609 2.36971384 0.58436111
 2.3274266  2.98007813], Cost: 0.7209
Current parameters: [0.71749096 0.27634008 2.09322848 1.65231367 0.9311155  3.1772469
 3.26797235 1.52585911 2.77725851 1.65683172 1.33042106 1.31104641
 1.27117891 2.8343613  1.52554244 2.97831375 2.31051701 0.50992815
 2.26652436 2.96134201], Cost: 1.2603
Current parameters: [0.70291998 0.28354635 2.0994158  1.63785455 0.88930477 2.89816163
 2.91309796 1.79684744 2.77613092 1.66687235 1.36552719 1.41518103
```



```
 1.29113046 2.89496112 1.56361161 3.02498609 2.36971384 0.58436111
 2.3274266  2.98007813], Cost: 0.9502
Current parameters: [0.71747964 0.27634568 2.09323328 1.65230244 0.93108301 2.89162686
 2.89426725 1.64269262 2.77725763 1.65683952 1.89263627 1.31112732
 1.27119442 2.83440838 1.52557202 2.97835001 2.310563   0.50998598
 2.26657168 2.96135657], Cost: 0.3264
Current parameters: [0.71747964 0.27634568 2.09323328 1.65230244 0.93108301 2.89162686
 2.89426725 1.64269262 2.77725763 1.65683952 1.89263627 1.45650294
 1.27119442 2.83440838 1.52557202 2.97835001 2.310563   0.30659982
 2.26657168 2.96135657], Cost: 0.6530
Current parameters: [0.74374851 0.26335406 2.08207865 1.67836965 1.00646025 2.8798367
 2.86029245 1.58851227 2.77929048 1.63873805 2.00822756 1.57997511
 1.23522535 2.72515775 1.45694013 2.89420814 2.20384173 0.83068719
 2.15677584 2.92757871], Cost: 2.9334
Current parameters: [0.68267866 0.24237464 2.06390058 1.63404531 0.89851624 2.86090342
 2.85406785 1.63094072 2.77725763 1.65683952 1.85277476 1.27189414
 1.27119442 2.83440838 1.52557202 2.97835001 2.310563   0.48194304
 2.04014748 2.96135657], Cost: 0.9886
Current parameters: [0.64681934 0.18382676 2.01338517 1.62272022 0.8959137  2.80792795
 2.77344991 1.58040778 2.77849646 1.64580839 1.86380673 1.0197237
 1.24927469 2.7678305  1.4837474  2.92707354 2.24552652 0.38002754
 2.55944225 2.94077217], Cost: 2.1348
Current parameters: [0.71747964 0.27634568 2.09323328 1.65230244 0.93108301 2.89162686
 2.89426725 1.64269262 2.77725763 1.65683952 1.89263627 1.31112732
 1.27119442 3.00910278 1.52557202 2.97835001 2.13172825 0.50998598
 2.26657168 2.96135657], Cost: 0.7712
Current parameters: [0.7718211  0.31720265 2.12853165 1.68468692 0.99796087 2.92856517
 2.93674662 1.64115135 2.77789897 1.65112874 1.98185464 1.26277039
 1.25984665 3.13941796 1.50391956 2.95180436 2.67131293 0.51597275
 2.28858674 2.9507001 ], Cost: 2.2447
Current parameters: [0.71747964 0.27634568 2.09323328 1.65230244 0.93108301 2.89162686
 2.89426725 1.64269262 2.77725763 1.65683952 1.89263627 1.31112732
 1.27119442 2.83440838 1.33184431 3.13636764 2.310563   0.50998598
 2.26657168 2.96135657], Cost: 0.3662
Current parameters: [0.75144309 0.30188132 2.11529479 1.67254276 0.97288172 2.91471333
 2.92081688 1.64172933 2.77765847 1.65327028 1.94839782 1.28090421
 1.26410205 2.76768652 1.83383088 3.35228978 2.28463711 0.51372772
 2.28033111 2.95469627], Cost: 2.8024
Current parameters: [0.71747964 0.27634568 2.09323328 1.65230244 0.93108301 2.89162686
 2.89426725 1.64269262 2.77725763 1.65683952 1.89263627 1.31112732
 1.44233295 2.83440838 1.52557202 2.97835001 2.310563   0.50998598
```

```
 2.26657168 2.77911607], Cost: 0.8901
Current parameters: [0.7309905  0.28650391 2.10200948 1.66035416 0.94771078 2.9008108
 2.90482885 1.64230942 2.77741709 1.65541965 1.91481855 1.29910439
 1.62322607 2.80786603 1.50456002 2.95100554 2.30024953 0.51147447
 2.27204526 3.31136043], Cost: 0.8185
Current parameters: [0.71747964 0.27634568 2.09323328 1.65230244 0.93108301 2.89162686
 2.89426725 1.64269262 3.0256959  1.68473986 1.89263627 1.31112732
 1.27119442 2.83440838 1.52557202 2.97835001 2.310563   0.50998598
 2.26657168 2.96135657], Cost: 0.7628
Current parameters: [0.72240713 0.28005044 2.09643401 1.65523894 0.93714725 2.89497629
 2.89811912 1.64255287 2.71754434 2.15274272 1.90072627 1.30674249
 1.26503414 2.82472824 1.51790883 2.96837733 2.30680162 0.51052884
 2.26856792 2.96265333], Cost: 0.5242
Current parameters: [0.71970834 0.41782847 2.21539832 1.66335923 0.95080594 2.91703402
 2.78007264 1.53724234 2.77740589 1.65551932 1.85155675 1.29743512
 1.26857107 2.82644038 1.52056647 2.97221327 2.30277947 0.50019912
 2.25856391 2.95889304], Cost: 0.2979
Current parameters: [0.73467491 0.63499009 2.40291874 1.60508406 0.85124328 2.79312179
 2.99213422 1.77679328 2.75785029 1.63944783 1.90001032 1.21117259
 1.22378823 2.73838171 1.4568451  2.89102346 2.24700389 0.45806948
 2.23233583 2.95444964], Cost: 1.7523
Current parameters: [0.72957815 0.40257238 2.20222526 1.68132189 0.98284745 3.08388669
 2.91969071 1.47897938 2.75722881 1.63690519 1.82292162 1.24785144
 1.23557197 2.77712991 1.51378987 2.92889584 2.28554478 0.49195787
 2.24966723 2.95826027], Cost: 0.5312
Current parameters: [0.69682824 0.49356146 2.28082019 1.66865476 0.97365571 2.96774908
 2.69077476 1.36236415 2.92172068 1.77490832 1.71308765 1.34469726
 1.42661887 2.94312442 1.32586825 3.07470911 2.19939508 0.32127151
 2.15890834 2.91823942], Cost: 1.9104
Current parameters: [0.73656454 0.435904   2.23101322 1.67338706 0.97206167 2.93000083
 2.79332117 1.53316701 2.76752613 1.65780001 1.87598709 1.28227352
 1.2620271  2.79477708 1.66902141 3.16251204 2.28565621 0.49607898
 2.26217573 2.97238584], Cost: 0.5461
Current parameters: [0.59893729 0.4271356  2.22343467 1.66947434 0.96171398 2.92972102
 2.78769661 1.54784088 2.77711031 1.65647324 1.85754297 1.30852834
 1.27050036 2.83272282 1.52476423 2.97706337 2.30917826 0.50835849
 2.26522181 2.9609617 ], Cost: 0.3063
Current parameters: [0.74064197 0.41377354 2.21190009 1.69339218 1.00577655 3.01304903
 2.68569256 1.70444444 2.77149996 1.65430777 1.74365121 1.30259962
 1.26193216 2.81808352 1.52130732 2.97334711 2.3031914  0.50917929
 2.26861264 2.96360152], Cost: 0.4698
```



```
Current parameters: [0.71970834 0.3361359  2.31000881 1.66372818 0.95059911 2.91703402
 2.78007264 1.53724234 2.77740589 1.65551932 1.85155675 1.29743512
 1.26857107 2.82644038 1.52056647 2.97221327 2.30277947 0.50019912
 2.25856391 2.95889304], Cost: 0.2757
Current parameters: [0.71987859 0.33638165 2.30934793 1.8834375  0.83143859 2.91680136
 2.77710359 1.53608443 2.77693583 1.65581205 1.854644   1.29769855
 1.26780728 2.8246348  1.51946291 2.97277869 2.30167174 0.50036101
 2.25953438 2.9594969 ], Cost: 0.3466
Current parameters: [0.72094672 0.33835533 2.31192933 1.64428303 0.91780104 2.97598106
 2.73673561 1.55218763 2.78349504 1.65816138 1.93890444 1.28418252
 1.27267719 2.82459452 1.51089876 2.97040074 2.29210508 0.48292609
 2.24504843 2.95404353], Cost: 0.3181
Current parameters: [0.71362013 0.33428898 2.30910419 1.56604279 0.76993899 2.96797558
 2.78445565 1.52180351 2.76662307 1.65569549 1.76487044 1.35176414
 1.26674401 2.84367922 1.54414515 2.99171143 2.33471168 0.5505635
 2.30126998 2.97394059], Cost: 0.3229
Current parameters: [0.71503518 0.3378323  2.31147312 1.66160995 0.94660772 2.91417137
 2.77014192 1.53157533 2.79040563 1.63581583 1.84370277 1.3026101
 1.18902497 2.83936127 1.51672341 2.9844612  2.30041589 0.49072168
 2.25185255 2.86948755], Cost: 0.3888
Current parameters: [0.71709142 0.34106761 2.31475394 1.66300495 0.94980116 2.91558441
 2.76694782 1.52775263 2.81704307 1.41711968 1.84524151 1.30234233
 1.31578684 2.83801655 1.51017509 2.98488129 2.29631345 0.48687828
 2.25065947 2.98713755], Cost: 0.2532
Current parameters: [0.70868514 0.33140512 2.30641571 1.65967269 0.94612696 2.90567944
 2.75085796 1.51875379 2.82002206 1.41599839 1.84183834 1.34303867
 1.31487087 2.76011792 1.50252693 2.97999038 2.21625483 0.51248202
 2.24719224 2.98238796], Cost: 0.4571
Current parameters: [0.75112082 0.33346541 2.31467172 1.68657813 1.01192173 2.92010951
 2.74438113 1.48854479 2.76952113 1.39801704 1.91854158 1.40856861
 1.27767003 2.88345612 1.49799847 2.96055685 2.41098269 0.60870096
 2.27656318 3.03816683], Cost: 0.4584
Current parameters: [0.70649974 0.30811404 2.28630241 1.6571794  0.94060997 2.89170544
 2.75203201 1.53887388 2.78497492 1.39782523 1.85868934 1.24611633
 1.28061414 2.80331527 1.53458097 2.96097774 2.30997499 0.48850763
 2.31275675 3.00077818], Cost: 0.3993
Current parameters: [0.70111309 0.34021998 2.32306569 1.66312815 0.97428848 2.91674052
 2.67938287 1.40410861 2.84983507 1.45902846 1.78598199 1.26721122
 1.37837341 2.90804095 1.41690791 3.02541806 2.31441618 0.39533076
 2.29526825 3.0214859 ], Cost: 1.0756
Current parameters: [0.72111013 0.31242833 2.37221091 1.65509693 0.99856074 2.87354371
```



```
 2.80851851 1.54305044 2.80264428 1.45421207 1.8196722  1.29334355
 1.34497399 2.82312627 1.51316666 2.97246008 2.29598394 0.49294613
 2.25458706 3.028497  ], Cost: 0.4023
Current parameters: [0.73506541 0.50305373 2.38448156 1.64384489 0.86864631 2.88712463
 2.82944994 1.62287084 2.81467037 1.37338055 1.90182283 1.2921992
 1.27524007 2.84138491 1.45718361 2.97306999 2.3109312  0.47463037
 2.24258367 2.97697104], Cost: 0.5306
Current parameters: [0.70348564 0.32585411 2.29482376 1.65441946 0.93044011 2.90086262
 2.75226831 1.52760027 2.79620484 1.4106935  1.83423244 1.27911727
 1.30016216 2.83316942 1.51951371 2.97848533 2.32182966 0.49316073
 2.14417044 3.00632305], Cost: 0.7071
Current parameters: [0.71712812 0.34339747 2.31676569 1.66318702 0.95012595 2.9160028
 2.76506732 1.52601613 2.82113667 1.41755739 1.84456503 1.30451083
 1.33956238 2.80206458 1.50690245 2.98738239 2.33285501 0.48336785
 2.2505276  2.96173309], Cost: 0.1538
Current parameters: [0.72161701 0.33797526 2.30521006 1.66352016 0.94662623 2.91590304
 2.77788081 1.53801027 2.78604711 1.40955804 1.85679405 1.29431423
 1.40768418 2.84372335 1.5275483  2.97235365 2.31190284 0.51654611
 2.26282424 2.89206551], Cost: 0.0353
Current parameters: [0.72167336 0.34155264 2.308299   1.66379973 0.94712492 2.91654546
 2.77499341 1.53534397 2.82277932 1.41364937 1.85575536 1.26512444
 1.40761785 2.84352188 1.52252334 2.97619395 2.31170603 0.55665194
 2.26262176 2.89200322], Cost: 0.1059
Current parameters: [0.7267909  0.31628412 2.27819238 1.66273763 0.94029397 2.91306158
 2.80556153 1.56376651 2.85944178 1.41304977 1.87593922 1.33643561
 1.42228469 2.85645771 1.57372739 2.93796553 2.32673193 0.4835211
 2.27850887 2.88054552], Cost: 0.1948
Current parameters: [0.72279923 0.36333297 2.32710582 1.66593119 0.95108279 2.92117965
 2.75890665 1.51943301 2.78558335 1.40940448 1.85075087 1.29115756
 1.40687319 2.84079698 1.56714576 2.95260607 2.30973268 0.51464431
 2.26158767 2.89226117], Cost: 0.0155
Current parameters: [0.73474119 0.37172704 2.32894253 1.67168457 0.9597538  2.93095444
 2.77442728 1.52282161 2.77570451 1.40642621 1.8705394  1.2922274
 1.4124206  2.8321382  1.62431558 3.05726808 2.31005314 0.5136009
 2.2726401  2.89102213], Cost: 0.1791
Current parameters: [0.72011777 0.3618931  2.32586194 1.66257009 0.94480777 2.92655129
 2.79833099 1.477424   2.78379281 1.40690515 1.86810257 1.28281881
 1.40334008 2.83520346 1.565998   2.94599843 2.30710969 0.51163213
 2.25826598 2.89122587], Cost: 0.0333
Current parameters: [0.73689726 0.36619188 2.32360462 1.6837224  0.97960875 3.02133791
 2.78580541 1.54541874 2.77050956 1.3970804  1.81167259 1.2818905
```



```
 1.4014847  2.82644235 1.55941612 2.93247876 2.31241987 0.51455001
 2.27071845 2.88435947], Cost: 0.1283
Current parameters: [0.72216713 0.36752164 2.3230632  1.61095118 0.97552719 2.92428928
 2.75622684 1.51945766 2.78790797 1.40422835 1.85221144 1.29175385
 1.39822306 2.8430305  1.56662443 2.95423088 2.30948374 0.51304362
 2.26028544 2.8816287 ], Cost: 0.0089
Current parameters: [0.72053847 0.34415272 2.35805796 1.63857398 0.99304241 2.89779523
 2.76313378 1.51116567 2.79154555 1.40582245 1.82625552 1.29993029
 1.3317296  2.8557949  1.56296983 2.96078734 2.31357523 0.50807252
 2.26107309 2.80085529], Cost: 0.0468
Current parameters: [0.72352949 0.376858   2.31359002 1.63193048 1.01488386 2.91830195
 2.74978081 1.5238042  2.79232843 1.39839684 1.8813291  1.27728091
 1.40065006 2.83913872 1.5592897  2.94995214 2.30042837 0.49835858
 2.24887074 2.87843866], Cost: 0.0129
Current parameters: [0.71792126 0.38718571 2.32992211 1.60869705 0.97465398 2.92632731
 2.71257083 1.46900929 2.78847508 1.41453779 1.83571475 1.29008723
 1.44471405 2.87138134 1.51762086 2.96226477 2.31914763 0.46871388
 2.31054109 2.86386295], Cost: 0.0691
Current parameters: [0.78260869 0.36436674 2.31981826 1.60763206 0.96927807 2.91747609
 2.75308693 1.51521877 2.78794457 1.4034303  1.84974661 1.28600276
 1.39683474 2.83951692 1.56403127 2.95159055 2.3061368  0.50886381
 2.25701129 2.88057038], Cost: 0.0119
Current parameters: [0.72613865 0.44764465 2.36217521 1.60084625 0.9400978  2.90638637
 2.78456181 1.56578094 2.77677398 1.38423439 1.8828036  1.29480035
 1.39050564 2.83737805 1.53782244 2.94136326 2.31514482 0.50906885
 2.27503035 2.86705426], Cost: 0.0824
Current parameters: [0.72271145 0.39071106 2.29470875 1.61878799 0.98990388 2.90257649
 2.77010302 1.51165559 2.78676782 1.38521273 1.82244122 1.30160374
 1.39866808 2.84522342 1.56884918 2.95481659 2.31623267 0.52343831
 2.26561997 2.88734926], Cost: 0.0145
Current parameters: [0.73901937 0.35967754 2.33185856 1.62024113 1.00079963 2.93559405
 2.73520168 1.49905227 2.7622292  1.40156346 1.90027942 1.35335907
 1.38772948 2.85839202 1.55563073 2.93991532 2.35595846 0.57161897
 2.27788346 2.89371569], Cost: 0.2889
Current parameters: [0.72128302 0.34998441 2.34485688 1.61067421 0.97495095 2.92356682
 2.75300367 1.51735358 2.79627831 1.35090302 1.85055582 1.29236464
 1.40843535 2.84532196 1.564516   2.95687507 2.30844114 0.51025158
 2.25642213 2.88838504], Cost: 0.0030
Current parameters: [0.70527192 0.3510916  2.32488067 1.59955894 0.94599327 2.9119527
 2.73429442 1.51734633 2.76684674 1.3465608  1.82984721 1.26388962
 1.42940622 2.85334982 1.56850911 2.94696477 2.33870305 0.48792393
```

```
 2.16197646 2.86731541], Cost: 0.2395
Current parameters: [0.69695581 0.34127853 2.3401841  1.58063038 0.96483351 2.93727643
 2.74812029 1.52009124 2.79491377 1.37574419 1.84903933 1.30344033
 1.40896829 2.82279137 1.57190002 2.95753738 2.28425363 0.5187139
 2.25330517 2.88398421], Cost: 0.0352
Current parameters: [0.74659757 0.36136262 2.34079357 1.65181864 0.97816629 2.89292925
 2.73587807 1.5005953  2.79833248 1.30825804 1.84268702 1.32562998
 1.42830272 2.78951077 1.53933169 2.9485779  2.24648947 0.51281725
 2.2574817  2.86445837], Cost: 0.2055
Current parameters: [0.71808608 0.34614268 2.37529117 1.62318221 0.98208864 2.90048262
 2.74332543 1.53043282 2.79205353 1.37319009 1.83393255 1.29261582
 1.42578795 2.84268041 1.57945716 2.93904541 2.30816093 0.51359164
 2.25683517 2.90565999], Cost: 0.0141
Current parameters: [0.73360318 0.32348196 2.36544113 1.57019397 1.02773025 2.91251235
 2.83514377 1.51090168 2.78002881 1.35573312 1.84281679 1.27646521
 1.43434507 2.83610125 1.5321912  2.97639731 2.31121579 0.49702001
 2.26220709 2.89203844], Cost: 0.0795
Current parameters: [0.71513934 0.32875478 2.3277432  1.61171371 0.96655271 2.9093256
 2.74675476 1.53100664 2.75921434 1.34270019 1.85636523 1.27689805
 1.40243674 2.83634177 1.56471201 2.94995064 2.31125058 0.50370332
 2.28279292 2.88787689], Cost: 0.0187
Current parameters: [0.72095652 0.3650841  2.34954017 1.58953395 0.98374522 2.92488714
 2.74898694 1.49050896 2.85432412 1.3373719  1.84934327 1.22495735
 1.3858125  2.81341913 1.60706576 2.9314408  2.33116301 0.51527162
 2.29487383 2.89114072], Cost: 0.1294
Current parameters: [0.72131582 0.35192152 2.34445325 1.60704038 0.97469369 2.92366329
 2.75670581 1.51656942 2.7940925  1.34874024 1.84798207 1.28365951
 1.39121373 2.88630184 1.56836999 2.95237269 2.27091392 0.50678251
 2.25971906 2.90684087], Cost: 0.0330
Current parameters: [0.71950869 0.35287995 2.34996732 1.61104776 0.97598069 2.92346611
 2.74698776 1.51267808 2.79234868 1.35217224 1.84565998 1.29098997
 1.39260792 2.83525334 1.55361283 2.96446102 2.31099957 0.50539901
 2.25193026 2.90445238], Cost: 0.0240
Current parameters: [0.7202556  0.33626078 2.33161305 1.61181631 0.96828925 2.91791337
 2.76249259 1.53339725 2.82183307 1.35452325 1.85332387 1.31879567
 1.40281247 2.84679203 1.56819876 2.94822567 2.3178227  0.47282938
 2.26186624 2.89857014], Cost: 0.0333
Current parameters: [0.72235903 0.34954884 2.34432025 1.61526275 0.97888119 2.94297646
 2.75514456 1.52352566 2.79389316 1.34946507 1.83928381 1.29088744
 1.40700135 2.84350521 1.55646757 2.9392626  2.30896874 0.5099921
 2.25700606 2.88738663], Cost: 0.0220
```



```
Current parameters: [0.72690311 0.35744132 2.34914231 1.62670977 0.97830483 2.94874254
 2.754512   1.53339289 2.78696162 1.35143596 1.84203065 1.29145013
 1.41510191 2.84402976 1.59325762 2.99416331 2.30747446 0.4994595
 2.26627837 2.88728989], Cost: 0.0439
Current parameters: [0.72332452 0.35957024 2.34984751 1.60925002 0.96995279 2.92019144
 2.76569244 1.53370606 2.79397095 1.34498497 1.85865669 1.29348475
 1.39860105 2.83903939 1.5695147  2.95292319 2.30753937 0.51742771
 2.24927328 2.8895505 ], Cost: 0.0024
Current parameters: [0.71457902 0.39421246 2.36725551 1.59752449 0.95027765 2.91840177
 2.762068   1.54041592 2.79157708 1.35154403 1.86319489 1.29111862
 1.41078831 2.84757126 1.54644982 2.95337841 2.31091089 0.50078116
 2.27153617 2.87823197], Cost: 0.0198
Current parameters: [0.71957346 0.35378622 2.35903342 1.61322837 0.97199763 2.91510639
 2.76699715 1.53348518 2.79494086 1.3490986  1.850891   1.29449131
 1.38205091 2.84199398 1.56962867 2.95515282 2.30756969 0.51491357
 2.24962185 2.86896427], Cost: 0.0034
Current parameters: [0.7268164  0.3567368  2.35896642 1.60410177 0.9815854  2.92675436
 2.75761629 1.52435054 2.78208818 1.34932631 1.88416562 1.32395456
 1.39067914 2.85735389 1.5737622  2.94824859 2.33098071 0.5412925
 2.26144658 2.89043516], Cost: 0.0762
Current parameters: [0.72827982 0.35915481 2.34803425 1.61460087 0.97073365 2.91125772
 2.75947843 1.52069853 2.80044038 1.33386324 1.86215428 1.30014971
 1.41109584 2.83732671 1.56564643 2.9567866  2.2993529  0.52105388
 2.25064884 2.87778269], Cost: 0.0089
Current parameters: [0.73416372 0.3715539  2.35348206 1.62453    0.9744801  2.91677536
 2.77255841 1.55092464 2.78291862 1.33762663 1.85139775 1.30772675
 1.38287795 2.80845387 1.56673812 2.93881727 2.27571728 0.51106522
 2.25547855 2.90003112], Cost: 0.0513
Current parameters: [0.72670388 0.3530194  2.35484141 1.5990642  0.98301994 2.91631141
 2.78630109 1.53148263 2.79059592 1.34584196 1.85759444 1.29012512
 1.40624205 2.8377513  1.5620712  2.95824917 2.30729814 0.51464357
 2.2506545  2.88955625], Cost: 0.0076
Current parameters: [0.72374956 0.36324281 2.35162485 1.60496703 0.97229285 2.92058474
 2.76574604 1.52644831 2.79436906 1.34214322 1.85927575 1.29055642
 1.39687284 2.83615035 1.57781342 2.94801939 2.30885763 0.5127302
 2.25147587 2.88879981], Cost: 0.0036
Current parameters: [0.72343499 0.35759259 2.34814829 1.60953393 0.96934402 2.9174117
 2.76638766 1.53851692 2.81344816 1.34516265 1.85867443 1.27664981
 1.39546332 2.8345202  1.56947346 2.95399904 2.31019043 0.52866486
 2.25964852 2.89121274], Cost: 0.0184
Current parameters: [0.72332452 0.35957024 2.34984751 1.60925002 0.96995279 2.92019144
```



```
 2.76569244 1.53370606 2.79397095 1.34498497 1.85865669 1.29348475
 1.39860105 2.83903939 1.5695147  2.95292319 2.30753937 0.51742771
 2.24927328 2.8895505 ], Cost: 0.0024

   Return from subroutine COBYLA because the MAXFUN limit has been reached.

   NFVALS =  100   F = 2.425629E-03    MAXCV = 0.000000E+00
   X = 7.233245E-01   3.595702E-01   2.349848E+00   1.609250E+00   9.699528E-01
       2.920191E+00   2.765692E+00   1.533706E+00   2.793971E+00   1.344985E+00
       1.858657E+00   1.293485E+00   1.398601E+00   2.839039E+00   1.569515E+00
       2.952923E+00   2.307539E+00   5.174277E-01   2.249273E+00   2.889550E+00
Simulating for QAOA depth p = 11
Current parameters: [3.86690974 2.49952549 2.42117462 1.15631015 1.93797708 1.71042757
 2.65748297 2.1367816  2.42254641 1.06591924 1.41380511 0.74632511
 0.07402115 1.43786211 3.06543629 1.3347705  0.86465089 0.81242257
 2.29227382 2.3714326  1.28503933 0.22529708], Cost: 2.3497
Current parameters: [2.86690974 3.49952549 2.42117462 1.15631015 1.93797708 1.71042757
 2.65748297 2.1367816  2.42254641 1.06591924 1.41380511 0.74632511
 0.07402115 1.43786211 3.06543629 1.3347705  0.86465089 0.81242257
 2.29227382 2.3714326  1.28503933 0.22529708], Cost: 1.1241
Current parameters: [2.86690974 3.49952549 3.42117462 1.15631015 1.93797708 1.71042757
 2.65748297 2.1367816  2.42254641 1.06591924 1.41380511 0.74632511
 0.07402115 1.43786211 3.06543629 1.3347705  0.86465089 0.81242257
 2.29227382 2.3714326  1.28503933 0.22529708], Cost: 1.3385
Current parameters: [2.86690974 3.49952549 2.42117462 2.15631015 1.93797708 1.71042757
 2.65748297 2.1367816  2.42254641 1.06591924 1.41380511 0.74632511
 0.07402115 1.43786211 3.06543629 1.3347705  0.86465089 0.81242257
 2.29227382 2.3714326  1.28503933 0.22529708], Cost: 1.9105
Current parameters: [2.86690974 3.49952549 2.42117462 1.15631015 2.93797708 1.71042757
 2.65748297 2.1367816  2.42254641 1.06591924 1.41380511 0.74632511
 0.07402115 1.43786211 3.06543629 1.3347705  0.86465089 0.81242257
 2.29227382 2.3714326  1.28503933 0.22529708], Cost: 1.7958
Current parameters: [2.86690974 3.49952549 2.42117462 1.15631015 1.93797708 2.71042757
 2.65748297 2.1367816  2.42254641 1.06591924 1.41380511 0.74632511
 0.07402115 1.43786211 3.06543629 1.3347705  0.86465089 0.81242257
 2.29227382 2.3714326  1.28503933 0.22529708], Cost: 2.0741
Current parameters: [2.86690974 3.49952549 2.42117462 1.15631015 1.93797708 1.71042757
 3.65748297 2.1367816  2.42254641 1.06591924 1.41380511 0.74632511
 0.07402115 1.43786211 3.06543629 1.3347705  0.86465089 0.81242257
 2.29227382 2.3714326  1.28503933 0.22529708], Cost: 2.9890
Current parameters: [2.86690974 3.49952549 2.42117462 1.15631015 1.93797708 1.71042757
```



```
 2.65748297 3.1367816  2.42254641 1.06591924 1.41380511 0.74632511
 0.07402115 1.43786211 3.06543629 1.3347705  0.86465089 0.81242257
 2.29227382 2.3714326  1.28503933 0.22529708], Cost: 3.2292
Current parameters: [2.86690974 3.49952549 2.42117462 1.15631015 1.93797708 1.71042757
 2.65748297 2.1367816  3.42254641 1.06591924 1.41380511 0.74632511
 0.07402115 1.43786211 3.06543629 1.3347705  0.86465089 0.81242257
 2.29227382 2.3714326  1.28503933 0.22529708], Cost: 0.8815
Current parameters: [2.86690974 3.49952549 2.42117462 1.15631015 1.93797708 1.71042757
 2.65748297 2.1367816  3.42254641 2.06591924 1.41380511 0.74632511
 0.07402115 1.43786211 3.06543629 1.3347705  0.86465089 0.81242257
 2.29227382 2.3714326  1.28503933 0.22529708], Cost: 2.9164
Current parameters: [2.86690974 3.49952549 2.42117462 1.15631015 1.93797708 1.71042757
 2.65748297 2.1367816  3.42254641 1.06591924 2.41380511 0.74632511
 0.07402115 1.43786211 3.06543629 1.3347705  0.86465089 0.81242257
 2.29227382 2.3714326  1.28503933 0.22529708], Cost: 1.7690
Current parameters: [2.86690974 3.49952549 2.42117462 1.15631015 1.93797708 1.71042757
 2.65748297 2.1367816  3.42254641 1.06591924 1.41380511 1.74632511
 0.07402115 1.43786211 3.06543629 1.3347705  0.86465089 0.81242257
 2.29227382 2.3714326  1.28503933 0.22529708], Cost: 1.4842
Current parameters: [2.86690974 3.49952549 2.42117462 1.15631015 1.93797708 1.71042757
 2.65748297 2.1367816  3.42254641 1.06591924 1.41380511 0.74632511
 1.07402115 1.43786211 3.06543629 1.3347705  0.86465089 0.81242257
 2.29227382 2.3714326  1.28503933 0.22529708], Cost: 2.1006
Current parameters: [2.86690974 3.49952549 2.42117462 1.15631015 1.93797708 1.71042757
 2.65748297 2.1367816  3.42254641 1.06591924 1.41380511 0.74632511
 0.07402115 2.43786211 3.06543629 1.3347705  0.86465089 0.81242257
 2.29227382 2.3714326  1.28503933 0.22529708], Cost: 0.5344
Current parameters: [2.86690974 3.49952549 2.42117462 1.15631015 1.93797708 1.71042757
 2.65748297 2.1367816  3.42254641 1.06591924 1.41380511 0.74632511
 0.07402115 2.43786211 4.06543629 1.3347705  0.86465089 0.81242257
 2.29227382 2.3714326  1.28503933 0.22529708], Cost: 2.1688
Current parameters: [2.86690974 3.49952549 2.42117462 1.15631015 1.93797708 1.71042757
 2.65748297 2.1367816  3.42254641 1.06591924 1.41380511 0.74632511
 0.07402115 2.43786211 3.06543629 2.3347705  0.86465089 0.81242257
 2.29227382 2.3714326  1.28503933 0.22529708], Cost: 1.8802
Current parameters: [2.86690974 3.49952549 2.42117462 1.15631015 1.93797708 1.71042757
 2.65748297 2.1367816  3.42254641 1.06591924 1.41380511 0.74632511
 0.07402115 2.43786211 3.06543629 1.3347705  1.86465089 0.81242257
 2.29227382 2.3714326  1.28503933 0.22529708], Cost: 1.3716
Current parameters: [2.86690974 3.49952549 2.42117462 1.15631015 1.93797708 1.71042757
 2.65748297 2.1367816  3.42254641 1.06591924 1.41380511 0.74632511
```

```
 0.07402115 2.43786211 3.06543629 1.3347705  0.86465089 1.81242257
 2.29227382 2.3714326  1.28503933 0.22529708], Cost: 2.2364
Current parameters: [2.86690974 3.49952549 2.42117462 1.15631015 1.93797708 1.71042757
 2.65748297 2.1367816  3.42254641 1.06591924 1.41380511 0.74632511
 0.07402115 2.43786211 3.06543629 1.3347705  0.86465089 0.81242257
 3.29227382 2.3714326  1.28503933 0.22529708], Cost: 0.7638
Current parameters: [2.86690974 3.49952549 2.42117462 1.15631015 1.93797708 1.71042757
 2.65748297 2.1367816  3.42254641 1.06591924 1.41380511 0.74632511
 0.07402115 2.43786211 3.06543629 1.3347705  0.86465089 0.81242257
 2.29227382 3.3714326  1.28503933 0.22529708], Cost: 1.4532
Current parameters: [2.86690974 3.49952549 2.42117462 1.15631015 1.93797708 1.71042757
 2.65748297 2.1367816  3.42254641 1.06591924 1.41380511 0.74632511
 0.07402115 2.43786211 3.06543629 1.3347705  0.86465089 0.81242257
 2.29227382 2.3714326  2.28503933 0.22529708], Cost: 3.0003
Current parameters: [2.86690974 3.49952549 2.42117462 1.15631015 1.93797708 1.71042757
 2.65748297 2.1367816  3.42254641 1.06591924 1.41380511 0.74632511
 0.07402115 2.43786211 3.06543629 1.3347705  0.86465089 0.81242257
 2.29227382 2.3714326  1.28503933 1.22529708], Cost: 1.9772
Current parameters: [ 2.7696897   3.61012749  2.38482367  1.02296212  1.8240751   1.54934187
  2.3412576   1.77982171  3.46368669  0.72086852  1.26330229  0.64412845
 -0.1327107   2.49671541  2.78828928  1.10656443  0.72268249  0.52381493
  2.25336996  2.21562571  0.86689737 -0.01934928], Cost: 2.7030
Current parameters: [2.85511012 3.51294928 2.4167627  1.14012567 1.92415277 1.69087657
 3.14404471 2.0934573  3.42753961 1.02404036 1.39553855 0.73392148
 0.04893006 2.44500515 3.0317989  1.30707308 0.84742016 0.7773942
 2.28755205 2.35252229 1.23428935 0.1956043 ], Cost: 1.1939
Current parameters: [3.05468047 3.66457746 2.42117462 1.15631015 1.93797708 1.71042757
 2.65748297 2.1367816  3.42254641 1.06591924 1.41380511 0.74632511
 0.07402115 2.43786211 3.06543629 1.3347705  0.86465089 0.81242257
 2.29227382 2.3714326  1.28503933 0.22529708], Cost: 0.4698
Current parameters: [3.38473299 3.29286671 2.41910634 1.14872299 1.93149636 1.70126222
 2.65019812 2.11647152 3.42488718 1.04628676 1.4052419  0.74051039
 0.06225865 2.44121071 3.04966735 1.32178618 0.85657326 0.79600155
 2.29006029 2.3625676  1.26124815 0.21137734], Cost: 1.0237
Current parameters: [3.05468047 3.66457746 2.179976   1.22206139 1.93797708 1.71042757
 2.65748297 2.1367816  3.42254641 1.06591924 1.41380511 0.74632511
 0.07402115 2.43786211 3.06543629 1.3347705  0.86465089 0.81242257
 2.29227382 2.3714326  1.28503933 0.22529708], Cost: 0.6806
Current parameters: [3.05143592 3.67180631 2.55934679 1.63417261 1.93189924 1.701832
 2.650651   2.11773413 3.42474166 1.04750725 1.40577424 0.74087187
 0.06298988 2.44100254 3.05064765 1.32259337 0.85707542 0.79702239
```



```
 2.2901979  2.36311871 1.26272717 0.21224268], Cost: 0.6793
Current parameters: [3.05468047 3.66457746 2.42117462 1.15631015 2.14210291 1.56609238
 2.65748297 2.1367816  3.42254641 1.06591924 1.41380511 0.74632511
 0.07402115 2.43786211 3.06543629 1.3347705  0.86465089 0.81242257
 2.29227382 2.3714326  1.28503933 0.22529708], Cost: 0.4424
Current parameters: [3.04994759 3.67512231 2.43126992 1.15103827 2.42885791 1.96912968
 2.64751705 2.10899672 3.42574866 1.03906135 1.40209034 0.73837037
 0.05792965 2.4424431  3.04386385 1.31700751 0.85360042 0.78995807
 2.28924563 2.35930498 1.25249219 0.20625441], Cost: 1.3884
Current parameters: [3.05468047 3.66457746 2.42117462 1.15631015 2.14210291 1.56609238
 2.65748297 2.16540509 3.67090239 1.06591924 1.41380511 0.74632511
 0.07402115 2.43786211 3.06543629 1.3347705  0.86465089 0.81242257
 2.29227382 2.3714326  1.28503933 0.22529708], Cost: 0.5740
Current parameters: [3.04572555 3.68452899 2.44027557 1.14633541 2.12243727 1.53353963
 2.63862681 2.61386741 3.35433182 1.01510238 1.39164002 0.73127424
 0.043575   2.44652965 3.02461985 1.30116177 0.84374269 0.76991828
 2.2865443  2.34848636 1.22345805 0.18926712], Cost: 1.0947
Current parameters: [3.05468047 3.66457746 2.42117462 1.15631015 2.14210291 1.56609238
 2.65748297 2.1367816  3.42254641 1.16586922 1.18465451 0.74632511
 0.07402115 2.43786211 3.06543629 1.3347705  0.86465089 0.81242257
 2.29227382 2.3714326  1.28503933 0.22529708], Cost: 0.5111
Current parameters: [3.0444488  3.68737359 2.4429989  1.14491325 2.11963343 1.5288984
 2.63593838 2.11658394 3.40975844 1.50258832 1.6128211  0.72912835
 0.03923412 2.44776543 3.01880041 1.29636998 0.84076169 0.7638582
 2.28572741 2.34521478 1.21467806 0.18413013], Cost: 1.4650
Current parameters: [ 3.05468047  3.66457746  2.42117462  1.15631015  2.14210291  1.56609238
  2.65748297  2.1367816   3.42254641  1.06591924  1.41380511  0.97043663
 -0.03676705  2.43786211  3.06543629  1.3347705   0.86465089  0.81242257
  2.29227382  2.3714326   1.28503933  0.22529708], Cost: 0.6800
Current parameters: [3.04838571 3.67860216 2.43460141 1.14929852 2.12827918 1.5432098
 2.64422825 2.12435554 3.41467896 1.04472443 1.40982148 0.94953839
 0.52273931 2.44395486 3.03674484 1.31114563 0.84995371 0.78254467
 2.28824632 2.3553028  1.24175148 0.19997023], Cost: 0.9628
Current parameters: [3.05468047 3.66457746 2.42117462 1.15631015 2.14210291 1.56609238
 2.65748297 2.1367816  3.42254641 1.06591924 1.41380511 0.74632511
 0.07402115 2.19331514 3.01350574 1.3347705  0.86465089 0.81242257
 2.29227382 2.3714326  1.28503933 0.22529708], Cost: 0.3108
Current parameters: [3.0465759  3.68263441 2.43846176 1.1472826  2.1243047  1.53663081
 2.64041737 2.12078292 3.41241698 1.0386307  1.40867615 0.72152885
 0.07232513 2.0802372  3.48871857 1.30435322 0.84572812 0.77395445
 2.28708837 2.35066531 1.22930575 0.19268849], Cost: 3.0550
```



```
Current parameters: [3.05468047 3.66457746 2.42117462 1.15631015 2.14210291 1.56609238
 2.65748297 2.1367816  3.42254641 1.06591924 1.41380511 0.74632511
 0.07402115 2.19331514 3.01350574 1.46682822 0.6523757  0.81242257
 2.29227382 2.3714326  1.28503933 0.22529708], Cost: 0.1509
Current parameters: [3.04800792 3.67944387 2.43540724 1.14887771 2.12744952 1.54183647
 2.64343275 2.12360977 3.41420678 1.0434524  1.4095824  0.72591018
 0.0726248  2.20164619 2.92711129 1.88514839 0.89860072 0.7807515
 2.2880046  2.35433475 1.23915349 0.1984502 ], Cost: 1.6551
Current parameters: [3.05468047 3.66457746 2.42117462 1.15631015 2.14210291 1.56609238
 2.65748297 2.1367816  3.42254641 1.06591924 1.41380511 0.74632511
 0.07402115 2.19331514 3.01350574 1.46682822 0.6523757  0.84582012
 2.04451465 2.3714326  1.28503933 0.22529708], Cost: 0.3016
Current parameters: [3.0477865  3.6799372  2.43587954 1.14863107 2.12696327 1.54103156
 2.6429665  2.12317268 3.41393004 1.04270686 1.40944227 0.72523273
 0.07257847 2.20192265 2.92424438 1.42526117 0.61203698 1.28847801
 2.36813291 2.35376737 1.23763082 0.19755932], Cost: 0.9393
Current parameters: [3.05468047 3.66457746 2.42117462 1.15631015 2.14210291 1.56609238
 2.65748297 2.1367816  3.42254641 1.06591924 1.41380511 0.74632511
 0.07402115 2.19331514 3.01350574 1.46682822 0.6523757  0.81242257
 2.29227382 2.6056979  1.19774804 0.22529708], Cost: 1.0406
Current parameters: [3.04391118 3.68857141 2.44414566 1.14431441 2.11845278 1.52694405
 2.63480632 2.11552266 3.4090865  1.02965841 1.40698978 0.71337601
 0.07176749 2.20676121 2.87406763 1.40189496 0.58936126 0.75891372
 2.30331845 2.42698206 1.74021634 0.18196701], Cost: 1.2241
Current parameters: [3.06684119 3.65074287 2.42572156 1.17298992 2.15635028 1.58624171
 2.69703786 2.18143175 3.41740039 1.10907974 1.43263068 0.75910833
 0.0998801  2.18595351 3.04817253 1.49537326 0.67013374 0.84852292
 2.29714008 2.39092163 1.3373424  0.44390704], Cost: 0.9866
Current parameters: [3.01406953 3.7200335  2.42726932 1.1027954  2.08582752 1.48201139
 2.53512471 2.00208157 3.42553072 0.92332983 1.35870534 0.68658796
```
[truncated: 1,265,076 more chars]
